# Mapping geographical inequalities in oral rehydration therapy coverage in low-income and middle-income countries, 2000–17

**DOI:** 10.1016/S2214-109X(20)30230-8

**Published:** 2020-07-22

**Authors:** Local Burden of Disease Diarrhoea Collaborators, Kirsten E Wiens, Paulina A Lindstedt, Brigette F Blacker, Kimberly B Johnson, Mathew M Baumann, Lauren E Schaeffer, Hedayat Abbastabar, Foad Abd-Allah, Ahmed Abdelalim, Ibrahim Abdollahpour, Kedir Hussein Abegaz, Ayenew Negesse Abejie, Lucas Guimarães Abreu, Michael R M Abrigo, Ahmed Abualhasan, Manfred Mario Kokou Accrombessi, Dilaram Acharya, Maryam Adabi, Abdu A Adamu, Oladimeji M Adebayo, Rufus Adesoji Adedoyin, Victor Adekanmbi, Olatunji O Adetokunboh, Beyene Meressa Adhena, Mohsen Afarideh, Sohail Ahmad, Keivan Ahmadi, Anwar E Ahmed, Muktar Beshir Ahmed, Rushdia Ahmed, Temesgen Yihunie Akalu, Fares Alahdab, Ziyad Al-Aly, Noore Alam, Samiah Alam, Genet Melak Alamene, Turki M Alanzi, Jacqueline Elizabeth Alcalde-Rabanal, Beriwan Abdulqadir Ali, Mehran Alijanzadeh, Vahid Alipour, Syed Mohamed Aljunid, Ali Almasi, Amir Almasi-Hashiani, Hesham M Al-Mekhlafi, Khalid A Altirkawi, Nelson Alvis-Guzman, Nelson J Alvis-Zakzuk, Saeed Amini, Arianna Maever L Amit, Catalina Liliana Andrei, Mina Anjomshoa, Amir Anoushiravani, Fereshteh Ansari, Carl Abelardo T Antonio, Benny Antony, Ernoiz Antriyandarti, Jalal Arabloo, Hany Mohamed Amin Aref, Olatunde Aremu, Bahram Armoon, Amit Arora, Krishna K Aryal, Afsaneh Arzani, Mehran Asadi-Aliabadi, Hagos Tasew Atalay, Seyyed Shamsadin Athari, Seyyede Masoume Athari, Sachin R Atre, Marcel Ausloos, Nefsu Awoke, Beatriz Paulina Ayala Quintanilla, Getinet Ayano, Martin Amogre Ayanore, Yared Asmare Aynalem IV, Samad Azari, Peter S Azzopardi, Ebrahim Babaee, Tesleem Kayode Babalola, Alaa Badawi, Mohan Bairwa, Shankar M Bakkannavar, Senthilkumar Balakrishnan, Ayele Geleto Bali, Maciej Banach, Joseph Adel Mattar Banoub, Aleksandra Barac, Till Winfried Bärnighausen, Huda Basaleem, Sanjay Basu, Vo Dinh Bay, Mohsen Bayati, Estifanos Baye, Neeraj Bedi, Mahya Mahya Beheshti Beheshti, Masoud Behzadifar, Meysam Behzadifar, Bayu Begashaw Bekele, Yaschilal Muche Belayneh, Michellr L Bell, Derrick A Bennett, Dessalegn Ajema Berbada, Robert S Bernstein, Anusha Ganapati Bhat, Krittika Bhattacharyya, Suraj Bhattarai, Soumyadeep Bhaumik, Zulfiqar A Bhutta, Ali Bijani, Boris Bikbov, Binyam Minuye Birihane IV, Raaj Kishore Biswas, Somayeh Bohlouli, Hunduma Amensisa Amensisa Bojia I, Soufiane Boufous, Oliver J Brady, Nicola Luigi Bragazzi, Andrey Nikolaevich Briko, Nikolay Ivanovich Briko, Gabrielle B Britton, Sharath Burugina Nagaraja, Reinhard Busse, Zahid A Butt, Luis LA Alberto Cámera, Ismael R Campos-Nonato, Jorge Cano, Josip Car, Rosario Cárdenas, Felix Carvalho, Carlos A Castañeda-Orjuela, Franz Castro, Wagaye Fentahun Chanie, Pranab Chatterjee, Vijay Kumar Chattu, Tesfaye Yitna Yitna Chichiabellu, Ken Lee Chin, Devasahayam J Christopher, Dinh-Toi Chu, Natalie Maria Cormier, Vera Marisa Costa, Carlos Culquichicon, Matiwos Soboka Daba, Giovanni Damiani, Lalit Dandona, Rakhi Dandona, Anh Kim Dang, Aso Mohammad Darwesh, Amira Hamed Darwish, Ahmad Daryani, Jai K Das, Rajat Das Gupta, Aditya Prasad Dash, Gail Davey, Claudio Alberto Dávila-Cervantes, Adrian C Davis, Dragos Virgil Davitoiu, Fernando Pio De la Hoz, Asmamaw Bizuneh Demis, Dereje Bayissa Demissie, Getu Debalkie Demissie, Gebre Teklemariam Demoz, Edgar Denova-Gutiérrez, Kebede Deribe, Assefa Desalew, Aniruddha Deshpande, Samath Dhamminda Dharmaratne, Preeti Dhillon, Meghnath Dhimal, Govinda Prasad Dhungana, Daniel Diaz, Isaac Oluwafemi Dipeolu, Shirin Djalalinia, Kerrie E Doyle, Eleonora Dubljanin, Bereket Duko, Andre Rodrigues Duraes, Mohammad Ebrahimi Kalan, Hisham Atan Edinur, Andem Effiong, Aziz Eftekhari, Nevine El Nahas, Iman El Sayed, Maysaa El Sayed Zaki, Maha El Tantawi, Teshome Bekele Elema I, Hala Rashad Elhabashy, Shaimaa I El-Jaafary, Hajer Elkout, Aisha Elsharkawy, Iqbal RF Elyazar, Aklilu Endalamaw, Daniel Adane Endalew, Sharareh Eskandarieh, Alireza Esteghamati, Sadaf Esteghamati, Arash Etemadi, Oluchi Ezekannagha, Mohammad Fareed, Roghiyeh Faridnia, Farshad Farzadfar, Mehdi Fazlzadeh, Valery L Feigin, Seyed-Mohammad Fereshtehnejad, Eduarda Fernandes, Irina Filip, Florian Fischer, Nataliya A Foigt, Morenike Oluwatoyin Folayan, Masoud Foroutan, Richard Charles Franklin, Takeshi Fukumoto, Mohamed M Gad, Reta Tsegaye Gayesa, Teshome Gebre, Ketema Bizuwork Gebremedhin, Gebreamlak Gebremedhn Gebremeskel, Hailay Abrha Gesesew, Kebede Embaye Gezae, Keyghobad Ghadiri, Ahmad Ghashghaee, Pramesh Raj Ghimire, Paramjit Singh Gill, Tiffany K Gill, Themba G G Ginindza, Nelson G M Gomes, Sameer Vali Gopalani, Alessandra C Goulart, Bárbara Niegia Garcia Goulart, Ayman Grada, Mohammed Ibrahim Mohialdeen Gubari, Harish Chander Gugnani, Davide Guido, Rafael Alves Guimarães, Yuming Guo, Rajeev Gupta, Nima Hafezi-Nejad, Dessalegn H Haile, Gessessew Bugssa Hailu, Arvin Haj-Mirzaian, Arya Haj-Mirzaian, Randah R Hamadeh, Samer Hamidi, Demelash Woldeyohannes Handiso, Hamidreza Haririan, Ninuk Hariyani, Ahmed I Hasaballah, Md Mehedi Hasan, Edris Hasanpoor, Amir Hasanzadeh, Hadi Hassankhani, Hamid Yimam Hassen, Mohamed I Hegazy, Behzad Heibati, Behnam Heidari, Delia Hendrie, Nathaniel J Henry, Claudiu Herteliu, Fatemeh Heydarpour, Hagos Degefa de Hidru I, Thomas R Hird, Chi Linh Hoang, Enayatollah Homaie Rad, Praveen Hoogar, Mohammad Hoseini, Naznin Hossain, Mostafa Hosseini, Mehdi Hosseinzadeh, Mowafa Househ, Mohamed Hsairi, Guoqing Hu, Mohammedaman Mama Hussen, Segun Emmanuel Ibitoye, Ehimario U Igumbor, Olayinka Stephen Ilesanmi, Milena D Ilic, Mohammad Hasan Imani-Nasab, Usman Iqbal, Seyed Sina Naghibi Irvani, Sheikh Mohammed Shariful Islam, Chinwe Juliana Iwu, Neda Izadi, Anelisa Jaca, Nader Jahanmehr, Mihajlo Jakovljevic, Amir Jalali, Achala Upendra Jayatilleke, Ravi Prakash Jha, Vivekanand Jha, John S Ji, Jost B Jonas, Jacek Jerzy Jozwiak, Ali Kabir, Zubair Kabir, Amaha Kahsay, Hamed Kalani, Tanuj Kanchan, Behzad Karami Matin, André Karch, Mohd Anisul Karim, Hamidreza Karimi-Sari, Surendra Karki, Amir Kasaeian, Gebremicheal Gebreslassie Kasahun, Yawukal chane Kasahun, Habtamu Kebebe Kasaye, Gebrehiwot G Kassa, Getachew Mullu Kassa, Gbenga A Kayode, Ali Kazemi Karyani, Mihiretu M Kebede, Peter Njenga Keiyoro, Abraham Getachew Kelbore, Andre Pascal Kengne, Daniel Bekele Ketema, Yousef Saleh Khader, Morteza Abdullatif Khafaie, Nauman Khalid, Rovshan Khalilov, Ejaz Ahmad Khan, Junaid Khan, Md Nuruzzaman Khan I, Muhammad Shahzeb Khan, Khaled Khatab, Amir M Khater, Mona M Khater, Maryam Khayamzadeh, Mohammad Khazaei, Salman Khazaei, Mohammad Hossein Khosravi, Jagdish Khubchandani, Ali Kiadaliri, Yun Jin Kim, Ruth W Kimokoti, Adnan Kisa, Sezer Kisa, Niranjan Kissoon, Shivakumar KM Marulasiddaiah M KMShivakumar, Sonali Kochhar, Tufa Kolola, Hamidreza Komaki, Soewarta Kosen, Parvaiz A Koul, Ai Koyanagi, Moritz U G Kraemer, Kewal Krishan, Nuworza Kugbey, G Anil Kumar, Manasi Kumar, Pushpendra Kumar, Vivek Kumar, Dian Kusuma, Carlo La Vecchia, Ben Lacey, Sheetal D Lad, Dharmesh Kumar Lal, Felix Lam, Faris Hasan Lami, Prabhat Lamichhane, Van Charles Lansingh, Savita Lasrado, Avula Laxmaiah, Paul H Lee, Kate E LeGrand, Mostafa Leili, Tsegaye Lolaso Lenjebo, Cheru Tesema Leshargie, Aubrey J Levine, Shanshan Li, Shai Linn, Shiwei Liu, Simin Liu, Rakesh Lodha, Joshua Longbottom, Jaifred Christian F Lopez, Hassan Magdy Abd El Razek, Muhammed Magdy Abd El Razek, D R Mahadeshwara Prasad, Phetole Walter Mahasha, Narayan B Mahotra, Azeem Majeed, Reza Malekzadeh, Deborah Carvalho Malta, Abdullah A Mamun, Navid Manafi, Ana Laura Manda, Narendar Dawani Dawanu Manohar, Mohammad Ali Mansournia, Chabila Christopher Mapoma, Joemer C Maravilla, Gabriel Martinez, Santi Martini, Francisco Rogerlândio Martins-Melo, Anthony Masaka, Benjamin Ballard Massenburg, Manu Raj Mathur, Benjamin K Mayala, Mohsen Mazidi, Colm McAlinden, Birhanu Geta Meharie, Man Mohan Mehndiratta, Kala M Mehta, Tefera C Chane Mekonnen, Gebrekiros Gebremichael Meles, Peter T N Memiah, Ziad A Memish, Walter Mendoza, Ritesh G Menezes, Seid Tiku Mereta, Tuomo J Meretoja, Tomislav Mestrovic, Bartosz Miazgowski, Kebadnew Mulatu Mihretie, Ted R Miller, GK Mini, Erkin M Mirrakhimov, Babak Moazen, Bahram Mohajer, Amjad Mohamadi-Bolbanabad, Dara K Mohammad, Karzan Abdulmuhsin Mohammad, Yousef Mohammad, Naser Mohammad Gholi Mezerji, Roghayeh Mohammadibakhsh, Noushin Mohammadifard, Jemal Abdu Mohammed, Shafiu Mohammed, Farnam Mohebi, Ali H Mokdad, Mariam Molokhia, Lorenzo Monasta, Yoshan Moodley, Catrin E Moore, Ghobad Moradi, Masoud Moradi, Mohammad Moradi-Joo, Maziar Moradi-Lakeh, Paula Moraga, Linda Morales, Ilais Moreno Velásquez, Abbas Mosapour, Simin Mouodi, Seyyed Meysam Mousavi, Miliva Mozaffor I, Kindie Fentahun Muchie, Getahun Fentaw Mulaw, Sandra B Munro, Moses K Muriithi, Christopher J L Murray, GVS Murthy, Kamarul Imran Musa, Ghulam Mustafa, Saravanan Muthupandian, Ashraf F Nabhan, Mehdi Naderi, Ahamarshan Jayaraman Nagarajan, Kovin S Naidoo, Gurudatta Naik, Farid Najafi, Vinay Nangia, Jobert Richie Nansseu, Bruno Ramos Nascimento, Javad Nazari, Duduzile Edith Ndwandwe, Ionut Negoi, Henok Biresaw Netsere Netsere, Josephine W Ngunjiri, Cuong Tat Nguyen, Huong Lan Thi Nguyen, Trang Huyen Nguyen, Dabere Nigatu, Solomon Gedlu Nigatu, Dina Nur Anggraini Ningrum, Chukwudi A Nnaji, Marzieh Nojomi, Vuong Minh Nong, Ole F Norheim, Jean Jacques Noubiap, Soraya Nouraei Motlagh, Bogdan Oancea, Okechukwu Samuel Ogah, Felix Akpojene Ogbo, In-Hwan Oh, Andrew T Olagunju, Tinuke O Olagunju, Bolajoko Olubukunola Olusanya, Jacob Olusegun Olusanya, Obinna E Onwujekwe, Eyal Oren, Doris V V Ortega-Altamirano, Osayomwanbo Osarenotor, Frank B Osei, Mayowa O Owolabi, Mahesh P A, Jagadish Rao Padubidri, Smita Pakhale, Sangram Kishor Patel, Angel J Paternina-Caicedo, Ashish Pathak, George C Patton, Deepak Paudel, Kebreab Paulos, Veincent Christian Filipino Pepito, Alexandre Pereira, Norberto Perico, Aslam Pervaiz, Julia Moreira Pescarini, Bakhtiar Piroozi, Meghdad Pirsaheb, Maarten J Postma, Hadi Pourjafar, Farshad Pourmalek, Akram Pourshams, Hossein Poustchi, Sergio I Prada, Narayan Prasad, Liliana Preotescu, Hedley Quintana, Navid Rabiee, Amir Radfar, Alireza Rafiei, Fakher Rahim, Afarin Rahimi-Movaghar, Vafa Rahimi-Movaghar, Mohammad Hifz Ur Rahman, Muhammad Aziz Rahman, SHAFIUR Rahman, Fatemeh Rajati, Saleem Muhammad Rana, Chhabi Lal Ranabhat, Davide Rasella, David Laith Rawaf, Salman Rawaf, Lal Rawal, Wasiq Faraz Rawasia, Vishnu Renjith, Andre M N Renzaho, Serge Resnikoff, Melese Abate Reta, Negar Rezaei, Mohammad sadegh Rezai, Seyed Mohammad Riahi, Ana Isabel Ribeiro, Jennifer Rickard, Maria Rios-Blancas, Leonardo Roever, Luca Ronfani, Elias Merdassa Roro, Jennifer M Ross, Enrico Rubagotti, Salvatore Rubino, Anas M Saad, Yogesh Damodar Sabde, Siamak Sabour, Ehsan Sadeghi, Yahya Safari, Roya Safari-Faramani, Rajesh Sagar, Amirhossein Sahebkar, Mohammad Ali Sahraian, S Mohammad Sajadi, Mohammad Reza Salahshoor, Nasir Salam, Payman Salamati, Hosni Salem, Marwa R Rashad Salem I, Yahya Salimi, Hamideh Salimzadeh, Abdallah M Samy, Juan Sanabria, Milena M Santric-Milicevic, Bruno Piassi Sao Jose, Sivan Yegnanarayana Iyer Saraswathy, Kaushik Sarkar, Abdur Razzaque Sarker, Nizal Sarrafzadegan I, Benn Sartorius, Brijesh Sathian, Thirunavukkarasu Sathish, Monika Sawhney, Sonia Saxena, David C Schwebel, Anbissa Muleta Senbeta IV, Subramanian Senthilkumaran, Sadaf G Sepanlou, Edson Serván-Mori, Hosein Shabaninejad, Azadeh Shafieesabet, Masood Ali Shaikh, Ali S Shalash, Seifadin Ahmed Shallo, Mehran Shams-Beyranvand, MohammadBagher Shamsi, Morteza Shamsizadeh, Mohammed Shannawaz, Kiomars Sharafi, Hamid Sharifi, Hatem Samir Shehata, Aziz Sheikh, B Suresh Kumar Shetty, Kenji Shibuya, Wondimeneh Shibabaw Shiferaw, Desalegn Markos Shifti, Mika Shigematsu, Jae Il Shin, Rahman Shiri, Reza Shirkoohi, Soraya Siabani, Tariq Jamal Siddiqi, Diego Augusto Santos Silva, Ambrish Singh, Jasvinder A Singh, Narinder Pal Singh, Virendra Singh, Malede Mequanent Sisay, Eirini Skiadaresi, Mohammad Reza Sobhiyeh, Anton Sokhan, Shahin Soltani, Ranjani Somayaji, Moslem Soofi, Muluken Bekele Sorrie, Ireneous N Soyiri, Chandrashekhar T Sreeramareddy, Agus Sudaryanto, Mu'awiyyah Babale Sufiyan, Hafiz Ansar Rasul Suleria, Marufa Sultana, Bruno Fokas Sunguya, Bryan L Sykes, Rafael Tabarés-Seisdedos, Takahiro Tabuchi, Degena Bahrey Tadesse, Ingan Ukur Tarigan, Aberash Abay Tasew, Yonatal Mesfin Tefera, Merhawi Gebremedhin Tekle, Mohamad-Hani Temsah, Berhe Etsay Tesfay I, Fisaha Haile Haile Tesfay, Belay Tessema, Zemenu Tadesse Tessema, Kavumpurathu Raman Thankappan, Nihal Thomas, Alemayehu Toma Toma, Roman Topor-Madry, Marcos Roberto Roberto Tovani-Palone, Eugenio Traini, Bach Xuan Tran, Khanh Bao Tran, Irfan Ullah, Bhaskaran Unnikrishnan, Muhammad Shariq Usman, Benjamin S Chudi Uzochukwu, Pascual R Valdez, Santosh Varughese, Francesco S Violante, Sebastian Vollmer, Feleke Gebremeskel W/hawariat, Yasir Waheed, Mitchell Taylor Wallin, Yafeng Wang, Yuan-Pang Wang, Marcia Weaver, Bedilu Girma Weji, Girmay Teklay Weldesamuel, Catherine A Welgan, Andrea Werdecker, Ronny Westerman, Taweewat Wiangkham, Charles Shey Wiysonge, Haileab Fekadu Wolde, Dawit Zewdu Wondafrash, Tewodros Eshete Wonde, Getasew Taddesse Worku, Ai-Min Wu, Gelin Xu, Ali Yadollahpour, Seyed Hossein Yahyazadeh Jabbari, Tomohide Yamada, Hiroshi Yatsuya, Alex Yeshaneh, Christopher Sabo Yilgwan, Mekdes Tigistu Yilma, Paul Yip, Engida Yisma, Naohiro Yonemoto, Seok-Jun Yoon, Mustafa Z Younis, Mahmoud Yousefifard, Hebat-Allah Salah A Yousof, Chuanhua Yu, Hasan Yusefzadeh, Siddhesh Zadey, Zoubida Zaidi, Sojib Bin Zaman, Mohammad Zamani, Hamed Zandian, Nejimu Biza Zepro, Taddese Alemu Zerfu, Yunquan Zhang, Xiu-Ju George Zhao, Arash Ziapour, Sanjay Zodpey, Yves Miel H Zuniga, Simon I Hay, Robert C Reiner

## Abstract

**Background:**

Oral rehydration solution (ORS) is a form of oral rehydration therapy (ORT) for diarrhoea that has the potential to drastically reduce child mortality; yet, according to UNICEF estimates, less than half of children younger than 5 years with diarrhoea in low-income and middle-income countries (LMICs) received ORS in 2016. A variety of recommended home fluids (RHF) exist as alternative forms of ORT; however, it is unclear whether RHF prevent child mortality. Previous studies have shown considerable variation between countries in ORS and RHF use, but subnational variation is unknown. This study aims to produce high-resolution geospatial estimates of relative and absolute coverage of ORS, RHF, and ORT (use of either ORS or RHF) in LMICs.

**Methods:**

We used a Bayesian geostatistical model including 15 spatial covariates and data from 385 household surveys across 94 LMICs to estimate annual proportions of children younger than 5 years of age with diarrhoea who received ORS or RHF (or both) on continuous continent-wide surfaces in 2000–17, and aggregated results to policy-relevant administrative units. Additionally, we analysed geographical inequality in coverage across administrative units and estimated the number of diarrhoeal deaths averted by increased coverage over the study period. Uncertainty in the mean coverage estimates was calculated by taking 250 draws from the posterior joint distribution of the model and creating uncertainty intervals (UIs) with the 2·5th and 97·5th percentiles of those 250 draws.

**Findings:**

While ORS use among children with diarrhoea increased in some countries from 2000 to 2017, coverage remained below 50% in the majority (62·6%; 12 417 of 19 823) of second administrative-level units and an estimated 6 519 000 children (95% UI 5 254 000–7 733 000) with diarrhoea were not treated with any form of ORT in 2017. Increases in ORS use corresponded with declines in RHF in many locations, resulting in relatively constant overall ORT coverage from 2000 to 2017. Although ORS was uniformly distributed subnationally in some countries, within-country geographical inequalities persisted in others; 11 countries had at least a 50% difference in one of their units compared with the country mean. Increases in ORS use over time were correlated with declines in RHF use and in diarrhoeal mortality in many locations, and an estimated 52 230 diarrhoeal deaths (36 910–68 860) were averted by scaling up of ORS coverage between 2000 and 2017. Finally, we identified key subnational areas in Colombia, Nigeria, and Sudan as examples of where diarrhoeal mortality remains higher than average, while ORS coverage remains lower than average.

**Interpretation:**

To our knowledge, this study is the first to produce and map subnational estimates of ORS, RHF, and ORT coverage and attributable child diarrhoeal deaths across LMICs from 2000 to 2017, allowing for tracking progress over time. Our novel results, combined with detailed subnational estimates of diarrhoeal morbidity and mortality, can support subnational needs assessments aimed at furthering policy makers' understanding of within-country disparities. Over 50 years after the discovery that led to this simple, cheap, and life-saving therapy, large gains in reducing mortality could still be made by reducing geographical inequalities in ORS coverage.

**Funding:**

Bill & Melinda Gates Foundation.

## Introduction

Oral rehydration solution (ORS) is a simple treatment that can be prepared and used at home to prevent mortality due to dehydration and undernutrition in children with diarrhoea. This intervention is especially suitable in locations where intravenous fluids are scarce or unavailable,^1^ and replaces indiscriminate and unnecessary use of antibiotics to treat diarrhoea.^2^ ORS was discovered more than 50 years ago when a physician in Dhaka, Bangladesh, found that treating patients with cholera with glucose-electrolyte solutions in equivalent amounts to fluid losses could prevent the need for intravenous liquids in 80% of patients.^3^ Shortly thereafter, its ability to prevent dehydration was shown in a trial in Kolkata, India,^4^ and during a cholera outbreak among Bangladeshi refugees in India.^5^ Since then, WHO, UNICEF, and the US Centers for Disease Control and Prevention have promoted ORS as an essential medicine to treat diarrhoea, the third leading cause of death in children younger than 5 years of age worldwide.^6^ In the 1980s, in response to low ORS coverage (ie, the proportion of children with diarrhoea who received ORS), WHO promoted the use of so-called recommended home fluids (RHF) in addition to ORS, and oral rehydration therapy (ORT) became the phrase used to refer to treatment with ORS or RHF.^7^ Despite its inclusion in the WHO Essential Medicines List and Global Action Plan for the Prevention and Control of Pneumonia and Diarrhoea,^7^, ^8^, ^9^ coverage of ORS remains low. According to UNICEF surveys, only 34% of children younger than 5 years in low-income and middle-income countries (LMICs) in 2000 received ORS to treat diarrhoea; in 2016, the proportion increased to 44%, yet the majority remained untreated.^10^

**Research in context**

**Evidence before this study**

WHO's integrated Global Action Plan for the Prevention and Control of Pneumonia and Diarrhoea emphasises the need to make resources available to properly prevent and treat these childhood infections, including use of oral rehydration solution (ORS) to treat diarrhoea. In 2016, UNICEF published national-level estimates of the proportion of children with diarrhoea who received ORS or any alternative recommended home fluids (RHF) for countries and years with available household survey data. To understand the full landscape of currently published estimates, we did a literature review on Feb 11, 2019, with no date or language restrictions. We searched the PubMed database for the following terms in titles or abstracts: “ORS”, “ORT”, “RHF”, “oral rehydration solution”, “oral rehydration therapy”, “oral rehydration salts”, and “recommended home fluids”, with the necessary inclusion of “coverage”. This returned 229 total studies, seven of which presented or reviewed national-level estimates of ORS coverage globally or across multiple countries, and 26 of which estimated ORS or RHF subnational coverage in select countries. None of these studies, however, estimated ORS or RHF coverage subnationally across multiple regions or used geospatial modelling techniques to estimate ORS or RHF coverage in locations with sparse data.

**Added value of this study**

To our knowledge, this study presents the first high-resolution subnational estimates of the proportion and absolute number of children younger than 5 years with diarrhoea that received ORS or RHF in low-income and middle-income countries (LMICs) from 2000 to 2017. This work supports the examination of how patterns of coverage have changed over time since the establishment of the Millennium Development Goals in 2000, the identification of subnational areas in need of targeted interventions, and the stratification of oral rehydration therapy coverage into ORS and RHF estimates. We used Bayesian geostatistical modelling techniques and an extensive geolocated dataset to produce these estimates. Wherever possible, we tailored these methods to take into account national or subnational factors that might contribute to variation in ORS coverage, using spatially resolved covariates to estimate for areas with sparse data. These techniques produced estimates on continuous continent-wide surfaces, which we aggregated to policy-relevant administrative units. We show that ORS use has increased over time, and that increases in ORS use often corresponded to declines in RHF use to treat diarrhoea and in diarrhoeal mortality rate. We estimate that scaling up of ORS treatment over the study period prevented an estimated 52 230 deaths (36 910–68 860) across LMICs in 2017. Despite progress, coverage of ORS (ie, the proportion of children with diarrhoea who received ORS) remained below 50% in many locations where diarrhoea prevalence and mortality rates remain high. Importantly, we also show that while within-country geographical inequalities declined over time, large disparities remained in multiple countries with high diarrhoeal burden, including subnational areas of Colombia, Peru, Nigeria, and Sudan.

**Implications of all the available evidence**

Our mapped estimates identify areas with low ORS usage, which could indicate gaps in access to ORS or knowledge of its efficacy to treat diarrhoea, and illuminate areas where improvements in ORS coverage are needed. Together with maps of other key risk factors, including sanitation and childhood stunting, these results can be used to develop integrated strategies that prevent diarrhoeal morbidity and mortality on a local level. These estimates and corresponding visualisation tools can aid policy makers and public health practitioners in determining where increased efforts to reduce geographical inequalities in ORS coverage are needed to make further strides in reducing mortality with this simple therapy.

The efficacy of ORS and RHF in preventing child mortality relies on proper preparation of the solutions, which can vary depending on the resources available to a household. ORS is most commonly sold as premade packets with standardised sodium and glucose content, which need to be dissolved in 1 L of clean water and can then be stored for about 48 h.^11^ The cost of these packets varies by country; in Uganda, a single packet costs approximately 500 Ugandan shillings (about US$0·15),^12^ and in Nigeria in 2012, the cost of three packets ranged from $0·63 to $4·38 depending on location.^13^ By contrast, RHF can be made with household items and therefore can be less costly and more widely accessible. The composition of RHF varies by country and can include carefully measured sugar and salt added to clean water, or it can simply include plain juice, rice water, tea, or coconut water.^14^ A meta-analysis study in 2010 estimated that 100% coverage of ORS could prevent 93% of diarrhoeal deaths, yet found insufficient evidence on the effectiveness of RHF in preventing mortality, probably due to the broad range in RHF composition.^14^

To understand trends in diarrhoeal deaths and ORT coverage across space and time, it is crucial to analyse ORS and RHF treatment separately. A study in Ethiopia found subnational geographical variation in ORT coverage, which was driven primarily by differences in wealth.^15^ A recent study including data from 88 LMICs showed an 8 percentage-point difference in ORT coverage on average between the wealthiest and poorest household quintiles, which was low compared with other interventions such as improvements to water and sanitation.^16^ These studies, however, did not analyse ORS and RHF separately and might have underestimated variation. Other studies have shown that ORS use can vary broadly between countries, even between those sharing borders.^11^, ^17^ Additionally, studies have shown differences in ORS use between urban and rural populations in Kenya^18^ and Mexico.^19^ These findings suggest that there are subnational drivers of variation in ORS coverage, and that these drivers can differ between geographical regions. Moreover, previous studies showed subnational variation in diarrhoeal deaths and overall deaths in children younger than 5 years,^20^, ^21^, ^22^ some of which might be driven by subnational variation in ORS given its efficacy in reducing child mortality.

Furthermore, policies related to diarrhoea treatment set at the national level do not affect all subnational areas equally, and interventions are often implemented at the subnational level, such as those currently done in Nigeria and India.^23^, ^24^ Local-level estimates of ORS and RHF coverage are thus needed to identify vulnerable subpopulations most in need of increased efforts to prevent child mortality. Yet, to our knowledge, no study has estimated ORS coverage subnationally across multiple regions or has used geospatial modelling techniques to estimate ORS coverage in locations with sparse data, and no study has compared ORS coverage to patterns in RHF coverage.

Our aim in this study was to estimate the proportions of children with diarrhoea who were treated with ORS and RHF (ie, ORS and RHF coverage, respectively) over space and time in LMICs and examine geographical inequalities within countries. Here we present, to our knowledge, the first maps of ORS or RHF coverage for second administrative-level units (eg, districts, counties; henceforth referred to as units) in LMICs. We present both relative quantities (proportion of children) and absolute quantities (number of children), as these measures have distinct policy implications. We conclude by highlighting countries with some of the broadest differences in coverage across subnational units, which also have high diarrhoeal burdens and high subnational variation in mortality.

## Methods

### Definitions

For this study, ORS was defined as a pre-packaged electrolyte solution containing glucose or another form of sugar or starch, as well as sodium, chloride, potassium, and bicarbonate.^14^ Survey questions did not allow us to separate RHF into their different formulations; therefore, RHF were defined as all possible home fluid alternatives, including sugar-salt solution, cereal-salt solution, rice-water solution, and additional fluids, such as plain water, juice, tea, or rice water.^14^ To account for this variation, we adjusted all non-standard RHF definitions to the most common or standard definition across all surveys, using logistic regression to determine adjustments (appendix 1 p 3). ORT was defined as treatment with either ORS, RHF, or both. Coverage was defined as the proportion of children younger than 5 years of age with diarrhoea who received ORS, RHF, or ORT. Diarrhoea was defined as three or more abnormally loose or watery stools within a 24-h period.

### Data

We compiled 385 household surveys (including Demographic and Health Surveys, Multiple Indicator Cluster Surveys, and other country-specific surveys) representing 3 609 000 children with diarrhoea in 94 LMICs from 2000 to 2017, with geocoded information from 120 742 coordinates corresponding to survey clusters and 14 055 subnational polygon boundaries where point-level referencing was not available (appendix 1 p 4). We included surveys that asked if children younger than 5 years with diarrhoea received any kind of ORT, allowed for geolocation below the country level, and were representative of the populations in which they were conducted. We included surveys for countries classified as low income or middle income on the basis of their Socio-demographic Index (SDI) quintile: low SDI, low-middle SDI, or middle SDI.^25^ SDI, developed as part of the Global Burden of Diseases, Injuries, and Risk Factors Study (GBD), indicates the level of development based on a country's average education, fertility, and income, and is on a scale of 0 to 1.^25^ Only LMICs with relevant and available underlying data were included in subsequent analyses, and island nations with fewer than 1 million inhabitants were excluded (appendix 1 p 4). This study complied with the Guidelines for Accurate and Transparent Health Estimates Reporting recommendations (appendix 1 pp 85–86).^26^ Further details on data inclusion, coverage, and validation can be found in appendix 1 (pp 4, 8).

We compiled 15 spatial covariates that were indexed at the subnational level for all 94 countries included in the study and that had conceivable relationships with ORT, which were used as predictors in our model. Covariates related to urbanicity or access to cities were night-time lights, population, urban or rural location, urban proportion of the location, and access to cities. Covariates related to child health, support, and nutrition were prevalence of under-5 stunting, prevalence of under-5 wasting, ratio of child dependents (ages 0–14 years) to working adults (ages 15–64 years), number of children younger than 5 years per woman of childbearing age, number of people whose daily vitamin A needs could be met, and maternal education. Covariates related to environmental factors that might affect diarrhoeal burden, which might in turn affect ORS supply, were aridity, distance from rivers or lakes, elevation, and irrigation. We also included the Healthcare Access and Quality Index^27^ and the proportion of pregnant women who received four or more antenatal care visits as national-level covariates. We filtered these covariates for multicollinearity within each modelling region (appendix 1 p 5) using variance inflation factor (VIF) analysis with a VIF threshold of 3.^28^ Detailed covariate information can be found in appendix 1 (p 5).

### Statistical analysis

Analyses were done using R version 3.5.0. ORS, RHF, and ORT coverage were modelled separately using a Bayesian model-based geostatistical framework. Briefly, this framework uses a spatially and temporally explicit hierarchical logistic regression model to predict coverage in all locations, assuming that points that are closer together in space and time and that have similar covariate patterns have similar coverage. Potential non-linear relationships between covariates and coverage were incorporated through the use of a stacked generalisation technique.^29^ Posterior distributions of all model parameters and hyperparameters were estimated using the statistical package R-INLA (version 19.05.30.9000).^30^, ^31^ Uncertainty in the mean coverage estimates was calculated by taking 250 draws from the posterior joint distribution of the model, and each point value is reported with an uncertainty interval (UI), which represents the 2·5th and 97·5th percentiles of those 250 draws. Maps were produced using ArcGIS Desktop 10.6. Models were run independently in 14 geographically distinct modelling regions based on GBD,^32^ and an additional nine country-specific models due to distinct temporal patterns of ORS coverage in these countries compared with their surrounding regions. Additional methodological details can be found in appendix 1 (pp 5–7).

Models were validated using five-fold cross-validation. Holdout sets were created by combining randomised sets of datapoints at the second administrative-unit cluster level. Model performance was summarised by the bias (mean error), total variance (root-mean-square error), and 95% data coverage within prediction intervals, and correlation between observed data and predictions. Where possible, estimates from these models were compared against other existing estimates. All validation procedures and corresponding results are provided in appendix 1 (p 8).

We calculated population-weighted aggregations of the 250 draws of ORS, RHF, and ORT coverage estimates at the country level, first administrative-level unit, and second administrative-level unit. To quantify geographical inequalities within countries over time, we used three different measures of inequality, each with their own strengths. We calculated Gini coefficients as a summary measure of inequality at the country level;^33^ in brief, the Gini coefficient summarises the distribution of each indicator across the population, with a value of 0 representing perfect equality and a value of 1 representing maximum inequality (appendix 1 p 9). We quantified absolute percentage-point deviation from the country mean to illustrate the total percentage-point difference in coverage between each unit and its country mean. Finally, we used relative deviation from the country mean to illustrate the difference in ORS coverage between each unit and its country mean.

To investigate the relationship between ORT and diarrhoeal mortality, we used mortality estimates from Reiner and colleagues^34^ and compared them with ORS coverage at the country and second administrative-unit levels. In addition, we did a counterfactual analysis to determine the estimated number of deaths averted due to changes in ORS coverage between 2000 and 2017, which is described in detail in appendix 1 (pp 9–10). In the counterfactual analysis, we treated ORS coverage as an independent risk factor and did not take into account how changes in demography or other risk factors affect deaths. We additionally did a sensitivity analysis of these results by halving and doubling the estimated lives that could be saved with ORS treatment^14^ (appendix 1 pp 82–83).

### Role of the funding source

This research was supported by the Bill & Melinda Gates Foundation. The funder had no role in study design, data collection, data analysis, data interpretation, or writing of the report. The corresponding author had full access to all the data in the study and had final responsibility for the decision to submit for publication.

## Results

**Figure 1:**
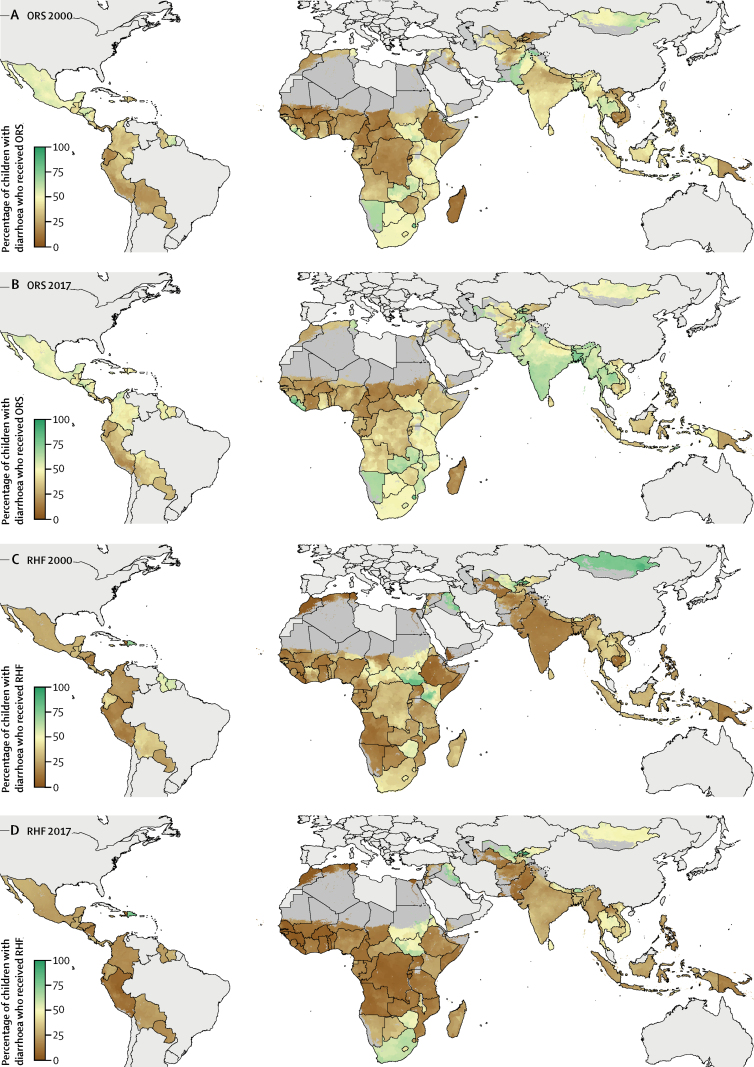
Proportion of children younger than 5 years with diarrhoea who received ORT at the second administrative-unit level, 2000 and 2017 Mean proportion of children with diarrhoea who received ORS in 2000 (A) and 2017 (B) or who received RHF in 2000 (C) and 2017 (D). All countries are aggregated to second administrative units. Maps reflect administrative boundaries, land cover, lakes, and population. Dark grey grid cells were classified as barren or sparsely vegetated and had fewer than ten people per 1 km × 1 km grid cell; light grey countries were not included in these analyses.^35^, ^36^, ^37^, ^38^, ^39^, ^40^ ORS=oral rehydration solution. ORT=oral rehydration therapy. RHF=recommended home fluids.

In all years from 2000 to 2017, we found both between-country and within-country variation in the proportion of children younger than 5 years with diarrhoea who received ORT. In general, ORS coverage was highest in south Asia, east Asia, central America, and southern sub-Saharan Africa, and lowest in central sub-Saharan Africa, parts of western and eastern sub-Saharan Africa, the Middle East, and South America (figure 1). Within these regions, some countries had fairly uniform subnational distribution of ORS across units, such as Zimbabwe in 2017, where coverage ranged from 35·1% (95% UI 11·8–66·6) in Chivi district, Masvingo province, to 44·6% (16·2–76·7) in Mazowe district, Mashonaland Central province. Other countries had notable subnational variation, such as Peru in 2017, where coverage ranged from 16·1% (12·1–20·6) in Azángaro province, Puno region, to 45·2% (38·2–51·5) in Trujillo province, La Libertad region (figure 1B). In terms of absolute coverage, RHF coverage was lower and more evenly distributed in Peru in 2017, with coverage ranging from 5·0% (2·9–8·5) in Coronel Portillo province, Ucayali, to 19·7% (12·3–28·9) in Daniel Alcides Carrión province, Pasco (figure 1D). Across all LMICs, ORS coverage remained below 50% in 62·6% (12 417 of 19 823) of units in 2017.

Although most changes were small, we found that ORS coverage increased while RHF coverage decreased between 2000 and 2017 in many locations (figure 1). We found significant increases in ORS coverage nationally and subnationally in Rwanda, Vietnam, Bolivia, Cambodia, and India (figure 1; appendix 1 p 75; appendix 2 pp 1–8, 25–1615), and significant declines in RHF coverage in Rwanda, Burundi, Bolivia, Niger, Chad, and India (figure 1; appendix 1 p 76; appendix 2 pp 9–16, 1616–3206). In Rwanda, ORS coverage increased from 12·0% (95% UI 9·8 to 14·6) to 33·9% (22·9 to 45·4), with an annualised rate of change (AROC) of 10·7% (3·2 to 17·6). At the same time, RHF coverage decreased from 28·1% (16·1 to 41·6) to 10·7% (3·3 to 25·8), with an AROC of −2·8% (−28·3 to 19·7). Increases in ORS, as measured by AROC, were significant (ie, 95% UIs did not include 0) in 27 of Rwanda's 30 units, while overall ORT coverage remained constant (appendix 1 pp 75–77; appendix 2 pp 1–8, 3207–4797). Kyrgyzstan, Yemen, and Liberia saw the largest increases in RHF coverage; however, uncertainty around these estimates was high, and only Yemen saw significant increases in RHF use (appendix 1 p 76; appendix 2 pp 9–16). Sudan and South Sudan were the only countries where AROC in ORS coverage declined substantially, with coverage decreasing from 32·3% (26·5 to 38·3) to 19·7% (14·6 to 26·2) in Sudan and from 52·0% (41·6 to 62·2) to 48·4% (37·6 to 59·5) in South Sudan. Declines were significant in eight of Sudan's 80 units and four of South Sudan's 45 units (figure 1; appendix 1 p 75; appendix 2 pp 1–8, 25–1615).

**Figure 2:**
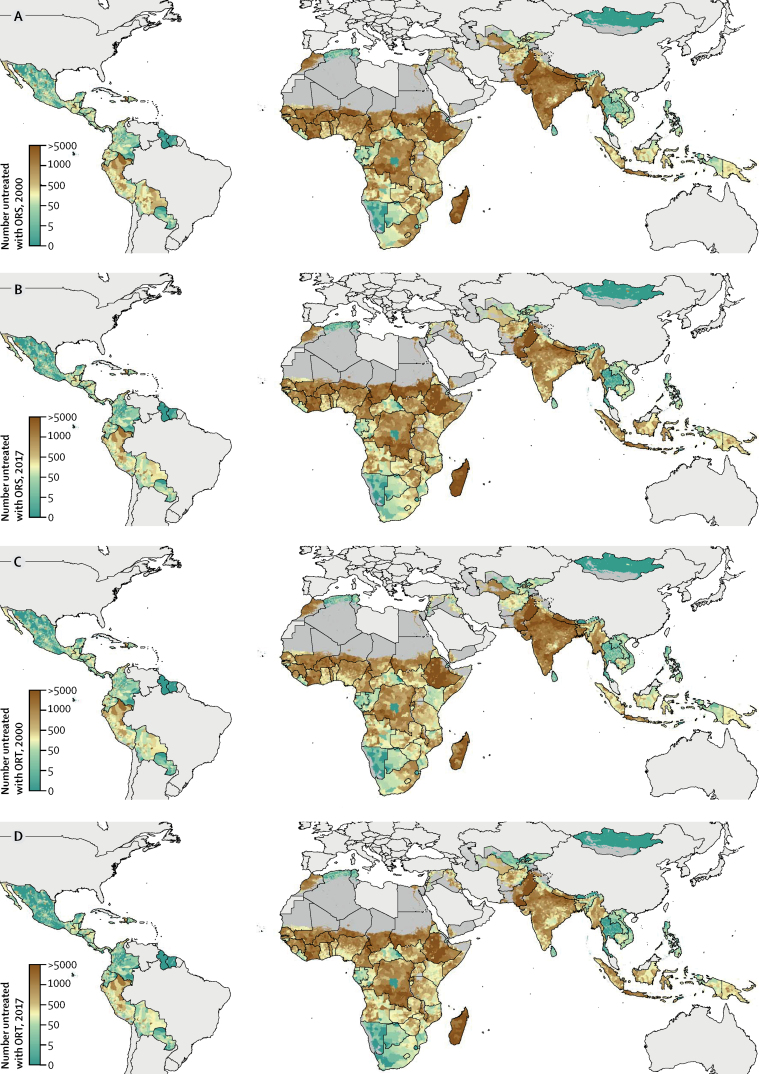
Number of children younger than 5 years with diarrhoea who did not receive ORT at the second administrative-unit level, 2000 and 2017 Number of children younger than 5 years with diarrhoea who did not receive ORS in 2000 (A) and 2017 (B) or did not receive ORT (either ORS or RHF) in 2000 (C) and 2017 (D). Countries are aggregated to second administrative units. Maps reflect administrative boundaries, land cover, lakes, and population. Dark grey grid cells were classified as barren or sparsely vegetated and had fewer than ten people per 1 km × 1 km grid cell; light grey countries were not included in these analyses.^35^, ^36^, ^37^, ^38^, ^39^, ^40^ ORS=oral rehydration solution. ORT=oral rehydration therapy. RHF=recommended home fluids.

In 2017, the highest number of children with diarrhoea who remained untreated by ORS were in parts of eastern sub-Saharan Africa, north Africa, south Asia, and southeast Asia (figure 2). In 2000, we estimated that approximately 6 668 000 children (95% UI 5 330 000–7 673 000) across the 94 LMICs included in this study were untreated with either ORS or RHF, out of a total of 12 873 000 children (12 344 000–13 471 000) with diarrhoea. Although prevalence of untreated children has declined, a substantial number remain in need of treatment; in 2017, we estimated 6 519 000 children (95% UI 5 254 000–7 733 000) with diarrhoea did not receive either ORS or RHF treatment, out of a total of 13 343 000 children (12 709 000–13 944 000) with diarrhoea, and this burden varied substantially within many countries (figure 2).

In addition to the results presented here, the full array of our model outputs for ORS, RHF, or ORT (either ORS or RHF) is provided in appendix 1 (pp 28–36) and is publicly available online, and can be further explored at various spatial levels via a user-friendly visualisation tool.

We found that inequality in ORS coverage, as measured by the Gini coefficient, decreased in the majority (63 [67%]) of countries from 2000 to 2017. In particular, although there were nine countries (Afghanistan, Cambodia, Cameroon, Côte d'Ivoire, Equatorial Guinea, Guinea, Iraq, Mali, and Mauritania) in 2000 whose Gini coefficient was greater than 0·15, only Afghanistan and Cameroon had coefficients above 0·15 in 2017.

**Figure 3:**
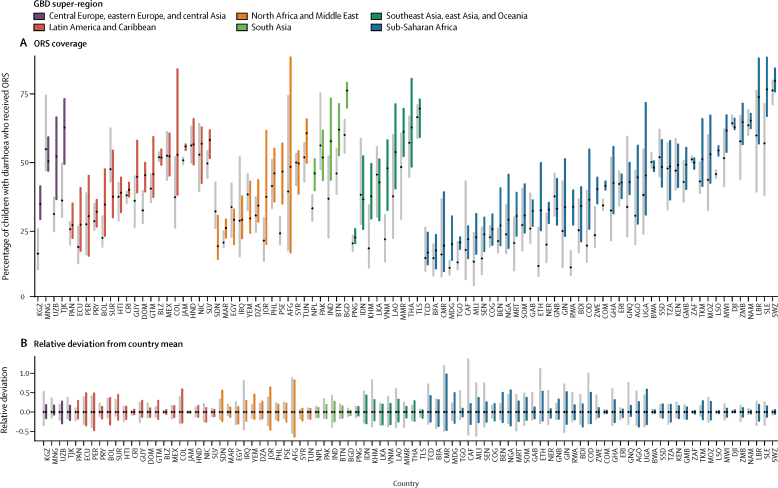
Geographical inequalities within countries in the proportion of children with diarrhoea who received ORS, 2000 and 2017 (A) Bars show range of ORS coverage at the second administrative-unit level for each country in 2000 (shown in grey) and 2017 (coloured by region), with the mean proportion (national-level aggregations) marked with a dot in each bar. (B) Bars show range of relative deviation from the country mean in the proportion of children younger than 5 years with diarrhoea who received ORS in 2000 (shown in grey) and 2017 (coloured by region). Countries are labelled by their ISO 3 codes. Geographical inequality in ORS coverage for each country is shown in detail in appendix 1 (p 78); inequalities in RHF and ORT coverage are shown in appendix 1 (pp 79–80). ORS=oral rehydration solution. ORT=oral rehydration therapy. RHF=recommended home fluids.

Absolute percentage-point differences between units with the highest and lowest ORS coverage declined in 40 countries, with notable decreases in Equatorial Guinea, Central African Republic, Iraq, Mongolia, Myanmar, and Sierra Leone (figure 3). Absolute inequalities increased in more than half (54 [57%]) of LMICs, with notable increases in Jordan, Colombia, Uzbekistan, Afghanistan, Bolivia, Turkmenistan, Palestine, Benin, and Madagascar (figure 3). By contrast, within-country absolute geographical inequalities in RHF coverage declined in most (55 [59%]) countries, with notable exceptions in Yemen and Tajikistan (appendix 1 p 79).

Analysis of relative deviation from the country mean revealed that 11 LMICs (Afghanistan, Benin, Cameroon, Democratic Republic of the Congo, Colombia, Ethiopia, Guinea, Jordan, Nigeria, Sudan, and Uganda) had at least 50% relative deviation in one of their units in ORS use in 2017 (figure 3). Additionally, as mean national-level ORS coverage increased over time in most (76 [81%]) countries (appendix 1 p 78), within-country relative differences in ORS coverage also declined in 64 (68%) LMICs, with greater than 50% declines in relative deviation in Central African Republic, Equatorial Guinea, Iraq, Mali, Cambodia, Ethiopia, Niger, Senegal, Kyrgyzstan, Togo, Democratic Republic of the Congo, and Côte d'Ivoire (figure 3). Exceptions to this pattern, where relative differences increased more than 20%, included Jordan, Benin, Madagascar, Yemen, Sudan, Suriname, Guatemala, Turkmenistan, and Bolivia. Furthermore, as mean national-level RHF coverage declined over time in most (69 [73%]) countries, within-country relative inequalities in RHF coverage declined in 45 (48%) countries (appendix 1 p 78). In 2017, relative inequalities in RHF coverage remained highest in North Africa and the Middle East (appendix 1 p 78).

We found that mean ORS coverage was less than 50% in 12 of 14 countries where diarrhoeal mortality in 2017 was greater than two children per 1000 (appendix 2 pp 1–8). Furthermore, we found that ORS coverage was negatively correlated with RHF coverage over time in 56·6% (10 786 of 19 064) of units and was negatively correlated with diarrhoeal mortality over time in 74·7% (14 241 of 19 064) of units (appendix 1 p 81).

**Figure 4:**
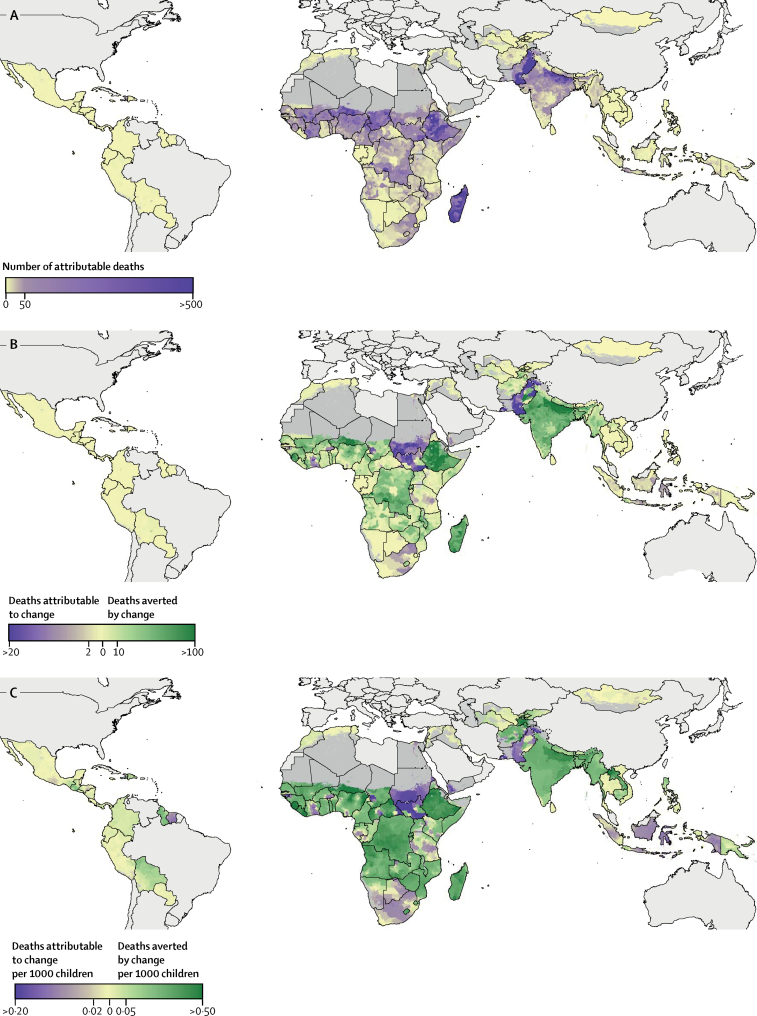
Averted child diarrhoeal deaths attributable to increased ORS coverage from 2000 to 2017 (A) Number of deaths in children younger than 5 years attributable to lack of ORS treatment in 2017. (B) Number of deaths in children younger than 5 years in 2017 averted by and attributable to changes in ORS coverage between 2000 and 2017. (C) Number of deaths per 1000 children younger than 5 years in 2017 averted by and attributable to changes in ORS between 2000 and 2017. Maps reflect administrative boundaries, land cover, lakes, and population. Dark grey grid cells were classified as barren or sparsely vegetated and had fewer than ten people per 1 km × 1 km grid cell; light grey countries were not included in these analyses.^35^, ^36^, ^37^, ^38^, ^39^, ^40^ ORS=oral rehydration solution.

To illustrate how our maps can be used to estimate the number of diarrhoeal deaths that were averted by changes in ORS coverage, we did a counterfactual analysis using a previous estimate that 75% ORS coverage could reduce diarrhoeal deaths by 69%.^14^ This estimate is based on a systematic review of three quasi-experimental studies with small sample sizes and that did not adjust for confounding variables (eg, stunting) to examine the risk of death in the absence of ORS treatment; thus, the results of this analysis should be interpreted with some caution. We found that of the 526 800 diarrhoeal deaths (95% UI 485 300–568 900) estimated to have occurred in 2017 in children younger than 5 years across the 94 LMICs included in our analysis, an estimated 299 900 deaths (274 000–324 300) could be attributable to lack of treatment with ORS. We also estimated that increase in ORS coverage during the study period prevented an additional 52 230 deaths (36 910–68 860). Nigeria, India, Ethiopia, Pakistan, Chad, and Madagascar contained units with high numbers of deaths attributable to lack of ORS treatment in 2017; however, these countries also contained units with the highest numbers of deaths averted by improved ORS coverage in 2017 (figure 4). By contrast, an estimated 4850 deaths (2200–10 080) globally were due to declines in ORS coverage, with some of the highest numbers of deaths attributable to worsening coverage in units of Sudan, South Sudan, and Pakistan (figure 4). Some of the highest rates of deaths averted were in units of Sierra Leone, where 0·9 deaths (0·2–1·9) were averted per 1000 children in Kambia district, Northern Province (figure 4), corresponding to 67 lives (18–141) saved in 2017 in this district alone.

In a sensitivity analysis, we found that, while the geographical patterns in deaths averted remained largely unchanged, the absolute number of averted deaths changed substantially in some places (appendix 1 pp 82–83). Reducing the percentage of diarrhoeal deaths that could be averted with ORS from 69% to 35% reduced the total number of deaths attributable to lack of ORS coverage in 2017 from 299 900 (95% UI 274 000–324 300) to 143 360 (130 400–156 000), the estimated total deaths averted by increase in ORS coverage from 52 230 (36 910–68 860) to 22 760 (15 600–30 650), and the averted deaths in Kambia district, Sierra Leone, from 67 (18–141) to 26 (8–53; appendix 1 p 82).

**Figure 5:**
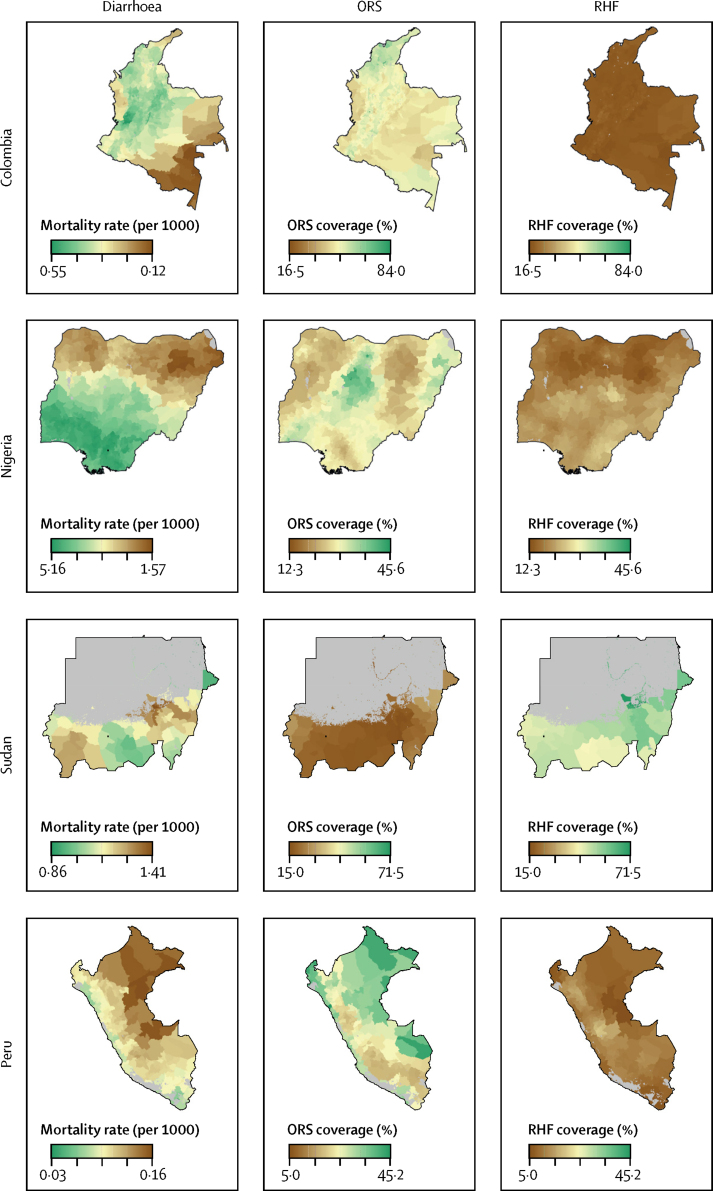
Subnational variation in the 2017 proportions of children who received ORT and diarrhoeal mortality in countries with high diarrhoeal burden at the second administrative-unit level Subnational variation in ORS, RHF, and diarrhoeal mortality per 1000 children is shown in four countries that had both high diarrhoeal burden and high geographical inequality in ORT in 2017. Results are shown for 2017 at second administrative units. Maps reflect administrative boundaries, land cover, lakes, and population. Dark grey grid cells were classified as barren or sparsely vegetated and had fewer than ten people per 1 km × 1 km grid cell.^35^, ^36^, ^37^, ^38^, ^39^, ^40^ ORS=oral rehydration solution. ORT=oral rehydration therapy. RHF=recommended home fluids.

Finally, to illustrate how these maps can be used to identify children in need, we present side-by-side maps of diarrhoeal mortality, ORS coverage, and RHF coverage at the unit level for three countries—Colombia, Nigeria, and Sudan—that had subnational locations with higher-than-average mortality rates and lower-than-average ORS coverage (figure 5). In Colombia, ORS and RHF coverage were lowest in the southern Amazonas region, where diarrhoeal burden was highest. In Nigeria, ORS coverage was lowest in the northern region, where diarrhoeal burden was highest. In Sudan, RHF remains widely used to treat diarrhoea, and there was not a clear trend between ORS, RHF, and diarrhoea distributions, but distinct areas in Darfur, in the southeast of the country, had high diarrhoeal mortality and particularly low ORS coverage. To illustrate that this pattern was not present everywhere, we also present results for Peru, where ORS coverage was relatively high in the Amazon Basin rainforests, which is where diarrhoeal mortality was also highest. There were gaps in coverage in the mountainous and arid regions of central and south Peru, where diarrhoeal mortality was lower (figure 5).

## Discussion

The discovery that led to the development of ORS as treatment for diarrhoea was hailed as “potentially the most important medical advancement of the century”.^41^ More than 50 years later, ORS is recognised as an important treatment for childhood diarrhoea, as well as a crucial component in treating other forms of dehydration, including dehydration-induced kidney injury and Ebola virus disease.^42^ By providing high-resolution estimates of the use of different forms of ORT—ORS, RHF, and either ORS or RHF—in children younger than 5 years with diarrhoea in LMICs, this study examines where uptake has occurred and which places stand to gain the most. While we show increases in ORS coverage in many locations, it is striking that these increases have been so incremental, given the importance and simplicity of this intervention. These slow changes are reflected in the relatively low number of total deaths estimated to have been averted by increases in ORS coverage between 2000 and 2017, and the substantial number of children with diarrhoea that remained untreated in 2017. ORS coverage remains below 50% in the majority (62·6%) of second administrative units, and there are various locations with high diarrhoeal mortality rates where geographical inequalities in ORS coverage are high. These areas need to be targeted with improved efforts to increase access to and awareness of this life-saving treatment.

We also show that increases in ORS coverage over time were correlated with declines in RHF coverage in many locations. It is possible that these results represent shifts over time in diarrhoea treatment, which might have contributed to declines in diarrhoeal mortality in these locations; ORS has shown effectiveness in preventing child mortality, whereas the effect of RHF on child mortality is unclear.^14^ However, if the rates of decline in RHF exceeded the rates of increase in ORS in some locations, this could have left a proportion of children completely untreated and in need of targeted interventions to prevent diarrhoeal mortality. These results further highlight the importance of reaching these vulnerable populations with targeted interventions to improve ORS coverage. It is important to note that there were also locations where there was apparently no relationship between ORS coverage and diarrhoeal mortality over time. This could, in part, be attributed to other risk factors that affect diarrhoeal mortality, which we did not take into account in this analysis.

Our estimates are comparable with previously published estimates at the national level.^10^, ^11^ We show notable differences in ORS coverage between countries in the same region (eg, Senegal *vs* Sierra Leone), consistent with a previous review.^8^ We show that ORS use has increased over time, with greater uptake in some regions compared with others (eg, south Asia *vs* the Horn of Africa), which is consistent with the conclusions of UNICEF's 2016 report.^8^, ^10^ However, we also show that the rates of increase in ORS coverage and decrease in RHF coverage were modest and that uncertainty in these estimates was high, which is consistent with previous studies that showed no substantial increases in ORT coverage between 1990 and 2001^43^ or between 1996 and 2003.^44^ We also show that relative and absolute geographical inequalities in ORS coverage declined over time in many countries, which is in contrast with a previous study that showed that absolute inequalities in ORT have remained the same over time in all but three LMICs.^16^ There are numerous methodological differences between that study and ours; most importantly, the previous study did not separate the effect of ORS from that of RHF. As we show, analysis of ORT (a combined variable) masks spatial and temporal variation in ORS and RHF.

We are surprised to see low use of ORS after so many years of programmes in many countries, especially those with high diarrhoeal burden. Ensuring access to ORS treatment is not only important for treating existing diarrhoea cases, but also in preparing for outbreaks and having supplies ready for emergencies. Moreover, educating caregivers on the causes of diarrhoea mortality—and how ORS can prevent those child deaths—is essential to ensure sustainable uptake. To address shortfalls in coverage, it will be essential to examine the root causes specific to each location. Previous studies have shown that challenges in using ORS include doctor and patient knowledge about ORS; ORS supply, cost, and taste; and access to clean water.^11^, ^45^ Studies have also shown that improvements in ORS coverage can be driven by changes in government policies, media campaigns, and community culture and beliefs.^2^, ^23^, ^24^ According to our results, Sierra Leone had some of the highest ORS coverage in western sub-Saharan Africa in 2017. Sierra Leone has previously been described as an example of how community mobilisation can promote access to and awareness of ORS, even after a devastating civil war.^11^ Our results also suggest that promotion of RHF over ORS might negatively affect ORS use and that locations with high RHF use, such as Sudan, can have very low ORS coverage. A previous study has similarly shown that inconsistent and unclear diarrhoea treatment recommendations present challenges in Sudan and Somalia and might have had implications for the recent cholera epidemic in Yemen.^46^ By determining key country-specific drivers of low uptake and subnational inequalities, including various social, cultural, political, and economic factors that might inhibit proper coverage, successful interventions such as those in Sierra Leone could be adapted and applied to similar contexts.

Our study has several limitations. Although we constructed a large database of geolocated ORT coverage data, spatial and temporal gaps remain, and data quality is likely to be variable by source, contributing to uncertainty in our estimates. Thus, results from zones of conflict and political instability, such as Yemen, Syria, Iraq, Afghanistan, and Pakistan, should be interpreted with caution. For RHF modelling, we included a broad range of RHF definitions in the survey data, and the RHF definitions in survey questionnaires do not always correspond to the actual solutions that governments have recommended. In addition, since the denominator of our input data was the proportion of children with diarrhoea (ie, diarrhoea prevalence), sample sizes were very small. Finally, heterogeneity within the data as well as amount of relevant available data varied between countries. Each of these factors probably contributed to uncertainty in our estimates, which varied by indicator and country (appendix 2).

As a further limitation, the modelling framework was optimised for prediction rather than causal inference, and there were overlaps between covariates used to estimate ORS, RHF, and diarrhoeal mortality, so we cannot make any conclusions about causal relationships between them. Additionally, we were unable to incorporate uncertainty into our estimates of the number of children with diarrhoea who were untreated because uncertainty from WorldPop datasets^35^ was not available. Furthermore, we fit our models using survey data, which depend on recall and are susceptible to biases that could be in the direction of increased or decreased coverage, depending on the context. Lastly, we mapped the reported use of ORS, yet use is not equivalent to proper preparation of the solution.^47^, ^48^

Future studies should examine the factors that have affected ORS coverage, particularly those that have contributed to shortfalls in efforts to increase coverage, to inform future interventions and implementation studies. Future work should also further investigate coverage of zinc treatment, which has shown effectiveness in reducing undernourishment and diarrhoeal mortality in many countries.^49^ In addition, promoting zinc use has shown a secondary effect of increasing ORS use in some places;^50^, ^51^ thus comprehensive approaches to overcome challenges to uptake and scaling up of coverage are warranted.^52^ Future work could investigate how missing data affect estimates of ORS coverage and how to account for this, as well as how to incorporate differences between urban and rural populations into the analysis. In addition, we did not map ORS availability, but rather the prevalence of its use, and future studies could map availability distribution patterns. Future work should examine the co-distribution of different interventions to prevent childhood mortality from diarrhoea, such as the co-distributions of ORS, zinc, and access to clean water. Finally, as with any study that involves estimation, the availability and quality of input data influences the certainty of our estimates; as LMICs work to improve their cause-specific vital registration systems, analyses that incorporate diarrhoea-specific cause of death data in estimates of diarrhoea mortality would improve future updates to this work.

In conclusion, our results show that advancement in ORS coverage was slow from 2000 to 2017, and that within-country inequalities in ORS coverage persist in many LMICs. Depending on the local context, low levels of coverage might reflect challenges in access to ORS or the need for education on the efficacy of ORS in preventing diarrhoea mortality. Increased efforts are needed, particularly where childhood deaths from diarrhoea are high yet ORS coverage remains low; in 2017, 12 of 14 LMICs where diarrhoeal mortality exceeded two children per 1000 had less than 50% ORS coverage. The subnational scale of these mapped estimates can aid in identifying where gaps in coverage of this life-saving intervention remain, contributing to the UN Sustainable Development Goals' commitment to address inequalities and leave no one behind.^53^ Our results illustrate that scaling up of ORS coverage has been insufficient, and that new efforts to improve access are desperately needed.

## Data sharing

The source code used to generate estimates is available online. The study data, including full sets of estimates at the first and second administrative levels, are available online.
