# Supplementary material for: Mapping geographical inequalities in oral rehydration therapy coverage in low-income and middle-income countries, 2000–17
Source: Lancet Glob Health. 2020 Jul 22;8(8):e1038–60. doi: 10.1016/S2214-109X(20)30230-8 (PMC7388204; doi:10.1016/S2214-109X(20)30230-8)
Supplement: Supplementary appendix 2 [file mmc2.pdf]

THE LANCET
Global Health

Supplementary appendix 2

This appendix formed part of the original submission and has been peer reviewed. We post it as supplied by the authors.

Supplement to: Local Burden of Disease Diarrhoea Collaborators. Mapping geographical inequalities in oral rehydration therapy coverage in low-income and middle-income countries, 2000–17. *Lancet Glob Health* 2020; **8:** e1038–60.

Table 1: ORS by Country

Country	Metric	Year	Mean	Upper	Lower
Afghanistan	Proportion	2000	39.5	52.9	26.1
Afghanistan	Proportion	2017	48.4	60.0	35.6
Afghanistan	Count	2000	136000	166000	106000
Afghanistan	Count	2017	196000	244000	152000
Algeria	Proportion	2000	30.8	38.2	24.5
Algeria	Proportion	2017	34.4	41.0	27.6
Algeria	Count	2000	79900	87200	71400
Algeria	Count	2017	85500	94200	76900
Angola	Proportion	2000	30.6	44.2	18.8
Angola	Proportion	2017	44.7	57.0	33.5
Angola	Count	2000	141000	165000	113000
Angola	Count	2017	125000	151000	97300
Bangladesh	Proportion	2000	60.0	68.1	50.8
Bangladesh	Proportion	2017	76.2	82.4	69.9
Bangladesh	Count	2000	136000	167000	109000
Bangladesh	Count	2017	44200	56000	32600
Belize	Proportion	2000	52.0	69.4	34.4
Belize	Proportion	2017	51.8	66.1	37.1
Belize	Count	2000	172	235	109
Belize	Count	2017	244	318	171
Benin	Proportion	2000	21.5	34.5	13.2
Benin	Proportion	2017	27.1	37.9	17.6
Benin	Count	2000	39100	43200	32600
Benin	Count	2017	30000	34000	25600
Bhutan	Proportion	2000	46.1	59.8	29.0
Bhutan	Proportion	2017	62.0	76.4	44.6
Bhutan	Count	2000	725	954	541
Bhutan	Count	2017	674	981	417
Bolivia	Proportion	2000	22.6	26.4	18.7
Bolivia	Proportion	2017	34.8	40.6	29.2
Bolivia	Count	2000	50500	53100	48000
Bolivia	Count	2017	31500	34200	28700
Botswana	Proportion	2000	50.2	56.8	44.2
Botswana	Proportion	2017	48.2	59.1	37.3
Botswana	Count	2000	2880	3230	2500
Botswana	Count	2017	2720	3280	2140
Burkina Faso	Proportion	2000	15.3	26.9	8.1
Burkina Faso	Proportion	2017	18.1	32.6	8.7
Burkina Faso	Count	2000	117000	127000	101000
Burkina Faso	Count	2017	112000	124000	91800
Burundi	Proportion	2000	25.4	31.7	19.6
Burundi	Proportion	2017	34.2	43.6	26.1
Burundi	Count	2000	39000	42000	35700
Burundi	Count	2017	40200	45200	34500
Cambodia	Proportion	2000	18.9	23.6	14.4
Cambodia	Proportion	2017	37.8	48.6	29.3
Cambodia	Count	2000	58800	62000	55400
Cambodia	Count	2017	34500	39100	28500
Cameroon	Proportion	2000	16.7	22.6	11.3

Table 1: ORS by Country *(continued)*

Country	Metric	Year	Mean	Upper	Lower
Cameroon	Proportion	2017	19.9	31.0	11.9
Cameroon	Count	2000	115000	122000	106000
Cameroon	Count	2017	160000	176000	138000
Central African Republic	Proportion	2000	18.3	20.0	16.9
Central African Republic	Proportion	2017	22.2	35.3	12.0
Central African Republic	Count	2000	31800	32400	31200
Central African Republic	Count	2017	25200	28500	21000
Chad	Proportion	2000	15.4	22.4	10.0
Chad	Proportion	2017	17.3	26.0	10.9
Chad	Count	2000	98800	105000	90500
Chad	Count	2017	171000	184000	153000
Colombia	Proportion	2000	37.4	40.5	34.2
Colombia	Proportion	2017	52.8	60.9	45.9
Colombia	Count	2000	117000	123000	112000
Colombia	Count	2017	65900	75600	54700
Comoros	Proportion	2000	34.4	53.4	16.4
Comoros	Proportion	2017	41.5	59.9	25.1
Comoros	Count	2000	2690	3440	1920
Comoros	Count	2017	1740	2240	1200
Costa Rica	Proportion	2000	38.1	57.8	17.5
Costa Rica	Proportion	2017	40.1	61.5	19.1
Costa Rica	Count	2000	6950	9260	4730
Costa Rica	Count	2017	7410	10000	4770
Côte d'Ivoire	Proportion	2000	19.5	28.0	12.6
Côte d'Ivoire	Proportion	2017	18.0	28.6	9.6
Côte d'Ivoire	Count	2000	118000	128000	106000
Côte d'Ivoire	Count	2017	141000	156000	123000
Democratic Republic of the Congo	Proportion	2000	19.9	25.4	15.1
Democratic Republic of the Congo	Proportion	2017	36.5	46.5	27.5
Democratic Republic of the Congo	Count	2000	388000	411000	361000
Democratic Republic of the Congo	Count	2017	391000	446000	329000
Djibouti	Proportion	2000	64.3	77.3	48.0
Djibouti	Proportion	2017	62.9	77.1	45.3
Djibouti	Count	2000	1070	1560	683
Djibouti	Count	2017	1210	1780	746
Dominican Republic	Proportion	2000	32.6	41.6	24.7
Dominican Republic	Proportion	2017	45.4	57.2	34.2
Dominican Republic	Count	2000	24800	27700	21500
Dominican Republic	Count	2017	19300	23200	15100
Ecuador	Proportion	2000	19.4	27.8	13.1
Ecuador	Proportion	2017	27.4	36.0	20.3
Ecuador	Count	2000	62100	67000	55600
Ecuador	Count	2017	26700	29300	23500
Egypt	Proportion	2000	33.8	37.0	30.7
Egypt	Proportion	2017	29.2	37.7	22.2
Egypt	Count	2000	234000	245000	223000
Egypt	Count	2017	347000	381000	305000
El Salvador	Proportion	2000	49.7	65.8	33.1
El Salvador	Proportion	2017	58.3	75.2	43.1

Table 1: ORS by Country *(continued)*

Country	Metric	Year	Mean	Upper	Lower
El Salvador	Count	2000	18200	24200	12400
El Salvador	Count	2017	8980	12200	5330
Equatorial Guinea	Proportion	2000	33.8	38.1	29.7
Equatorial Guinea	Proportion	2017	42.9	67.5	22.3
Equatorial Guinea	Count	2000	4940	5260	4630
Equatorial Guinea	Count	2017	4220	5750	2400
Eritrea	Proportion	2000	42.3	53.7	32.4
Eritrea	Proportion	2017	42.8	56.1	29.5
Eritrea	Count	2000	16300	19000	13000
Eritrea	Count	2017	15200	18700	11700
Ethiopia	Proportion	2000	12.5	14.5	10.6
Ethiopia	Proportion	2017	32.6	37.1	28.4
Ethiopia	Count	2000	562000	574000	549000
Ethiopia	Count	2017	347000	368000	324000
Gabon	Proportion	2000	26.0	29.2	22.7
Gabon	Proportion	2017	32.4	50.5	18.9
Gabon	Count	2000	5010	5230	4790
Gabon	Count	2017	6120	7340	4480
Gambia	Proportion	2000	43.0	55.3	30.4
Gambia	Proportion	2017	49.3	64.0	34.7
Gambia	Count	2000	6850	8360	5360
Gambia	Count	2017	6430	8270	4560
Ghana	Proportion	2000	32.5	45.8	21.9
Ghana	Proportion	2017	42.6	60.2	28.5
Ghana	Count	2000	75100	86900	60400
Ghana	Count	2017	77000	96000	53400
Guatemala	Proportion	2000	40.5	57.0	23.8
Guatemala	Proportion	2017	45.8	60.8	32.3
Guatemala	Count	2000	62600	80300	45300
Guatemala	Count	2017	62400	78000	45200
Guinea	Proportion	2000	25.1	38.5	15.0
Guinea	Proportion	2017	33.8	48.5	23.0
Guinea	Count	2000	52700	59800	43300
Guinea	Count	2017	50000	58200	38900
Guinea-Bissau	Proportion	2000	37.7	47.6	27.1
Guinea-Bissau	Proportion	2017	33.2	51.0	19.5
Guinea-Bissau	Count	2000	6880	8050	5780
Guinea-Bissau	Count	2017	6270	7550	4600
Guyana	Proportion	2000	36.1	42.3	30.0
Guyana	Proportion	2017	44.8	54.2	34.5
Guyana	Count	2000	1250	1370	1130
Guyana	Count	2017	703	835	584
Haiti	Proportion	2000	37.6	46.2	29.6
Haiti	Proportion	2017	39.0	49.4	29.2
Haiti	Count	2000	45700	51500	39400
Haiti	Count	2017	36400	42300	30200
Honduras	Proportion	2000	56.2	70.9	42.1
Honduras	Proportion	2017	56.7	72.2	41.2
Honduras	Count	2000	23900	31600	15900

Table 1: ORS by Country *(continued)*

Country	Metric	Year	Mean	Upper	Lower
Honduras	Count	2017	26600	36100	17100
India	Proportion	2000	36.8	42.8	32.2
India	Proportion	2017	57.8	61.8	53.9
India	Count	2000	1410000	1510000	1280000
India	Count	2017	734000	802000	664000
Indonesia	Proportion	2000	38.3	41.8	35.0
Indonesia	Proportion	2017	36.7	38.6	34.6
Indonesia	Count	2000	349000	367000	329000
Indonesia	Count	2017	484000	499000	469000
Iraq	Proportion	2000	28.9	37.0	21.2
Iraq	Proportion	2017	29.2	44.7	15.8
Iraq	Count	2000	81100	89900	71800
Iraq	Count	2017	94400	112000	73700
Jamaica	Proportion	2000	50.8	68.9	31.4
Jamaica	Proportion	2017	55.9	76.4	33.3
Jamaica	Count	2000	1110	1550	701
Jamaica	Count	2017	996	1500	532
Jordan	Proportion	2000	21.7	42.7	8.9
Jordan	Proportion	2017	37.5	50.5	26.1
Jordan	Count	2000	12300	14400	9030
Jordan	Count	2017	20100	23800	15900
Kenya	Proportion	2000	47.1	53.3	40.8
Kenya	Proportion	2017	49.1	56.8	39.4
Kenya	Count	2000	108000	121000	95800
Kenya	Count	2017	114000	136000	96700
Kyrgyzstan	Proportion	2000	17.0	27.8	9.3
Kyrgyzstan	Proportion	2017	35.0	51.2	22.3
Kyrgyzstan	Count	2000	8710	9500	7570
Kyrgyzstan	Count	2017	5850	6990	4390
Laos	Proportion	2000	37.7	46.0	30.3
Laos	Proportion	2017	53.8	62.3	46.2
Laos	Count	2000	11000	12400	9570
Laos	Count	2017	10400	12100	8490
Lesotho	Proportion	2000	45.8	59.5	31.0
Lesotho	Proportion	2017	54.4	69.3	40.6
Lesotho	Count	2000	5480	6970	4090
Lesotho	Count	2017	3020	3930	2040
Liberia	Proportion	2000	59.9	75.6	40.7
Liberia	Proportion	2017	73.9	83.5	61.5
Liberia	Count	2000	9450	14000	5740
Liberia	Count	2017	10500	15400	6600
Madagascar	Proportion	2000	11.8	14.8	9.0
Madagascar	Proportion	2017	20.4	26.6	14.6
Madagascar	Count	2000	99300	102000	95900
Madagascar	Count	2017	169000	181000	156000
Malawi	Proportion	2000	51.6	57.5	45.3
Malawi	Proportion	2017	61.6	69.8	51.6
Malawi	Count	2000	47500	53700	41700
Malawi	Count	2017	59300	74700	46600

Table 1: ORS by Country *(continued)*

Country	Metric	Year	Mean	Upper	Lower
Mali	Proportion	2000	14.0	20.7	8.7
Mali	Proportion	2017	22.9	34.8	14.2
Mali	Count	2000	103000	110000	95400
Mali	Count	2017	87600	97500	74100
Mauritania	Proportion	2000	20.8	28.2	15.0
Mauritania	Proportion	2017	30.6	42.3	21.0
Mauritania	Count	2000	17300	18500	15700
Mauritania	Count	2017	13000	14800	10800
Mexico	Proportion	2000	52.5	63.7	40.8
Mexico	Proportion	2017	52.3	63.4	41.1
Mexico	Count	2000	219000	273000	168000
Mexico	Count	2017	143000	177000	110000
Mongolia	Proportion	2000	54.9	59.3	50.4
Mongolia	Proportion	2017	50.5	73.0	27.7
Mongolia	Count	2000	2120	2330	1920
Mongolia	Count	2017	4100	5980	2240
Morocco	Proportion	2000	21.0	27.9	15.6
Morocco	Proportion	2017	26.2	33.5	20.2
Morocco	Count	2000	95000	101000	86600
Morocco	Count	2017	68800	74400	62000
Mozambique	Proportion	2000	43.7	49.5	38.0
Mozambique	Proportion	2017	53.1	58.7	47.2
Mozambique	Count	2000	71700	79000	64400
Mozambique	Count	2017	54300	61100	47800
Myanmar	Proportion	2000	48.4	56.1	40.8
Myanmar	Proportion	2017	61.2	69.6	52.3
Myanmar	Count	2000	98800	113000	84000
Myanmar	Count	2017	48200	59300	37800
Namibia	Proportion	2000	63.6	67.9	59.6
Namibia	Proportion	2017	65.3	73.3	56.0
Namibia	Count	2000	2810	3120	2480
Namibia	Count	2017	3630	4600	2800
Nepal	Proportion	2000	33.4	40.6	26.7
Nepal	Proportion	2017	46.1	53.8	38.1
Nepal	Count	2000	64100	70500	57100
Nepal	Count	2017	26000	29800	22300
Nicaragua	Proportion	2000	52.9	65.3	40.9
Nicaragua	Proportion	2017	56.9	70.3	40.7
Nicaragua	Count	2000	15900	19900	11700
Nicaragua	Count	2017	12600	17300	8690
Niger	Proportion	2000	20.2	27.5	14.2
Niger	Proportion	2017	32.9	45.4	20.9
Niger	Count	2000	161000	173000	146000
Niger	Count	2017	113000	133000	91800
Nigeria	Proportion	2000	23.9	32.1	17.3
Nigeria	Proportion	2017	29.2	37.2	21.2
Nigeria	Count	2000	730000	793000	651000
Nigeria	Count	2017	696000	775000	618000
Pakistan	Proportion	2000	56.2	63.1	49.2

Table 1: ORS by Country *(continued)*

Country	Metric	Year	Mean	Upper	Lower
Pakistan	Proportion	2017	51.9	59.4	43.1
Pakistan	Count	2000	228000	265000	192000
Pakistan	Count	2017	401000	474000	338000
Panama	Proportion	2000	25.8	41.7	14.6
Panama	Proportion	2017	27.3	40.9	15.9
Panama	Count	2000	12300	14100	9670
Panama	Count	2017	12700	14600	10300
Papua New Guinea	Proportion	2000	20.7	26.7	14.9
Papua New Guinea	Proportion	2017	22.9	30.4	15.4
Papua New Guinea	Count	2000	32500	34900	30000
Papua New Guinea	Count	2017	47700	52400	43100
Paraguay	Proportion	2000	28.7	37.1	21.0
Paraguay	Proportion	2017	32.1	40.0	24.3
Paraguay	Count	2000	14000	15500	12400
Paraguay	Count	2017	22500	25000	19900
Peru	Proportion	2000	27.6	31.4	24.0
Peru	Proportion	2017	30.5	33.8	27.3
Peru	Count	2000	97000	102000	91800
Peru	Count	2017	84400	88300	80300
Philippines	Proportion	2000	41.5	47.2	35.5
Philippines	Proportion	2017	46.1	49.9	42.4
Philippines	Count	2000	154000	170000	139000
Philippines	Count	2017	133000	142000	124000
Republic of Congo	Proportion	2000	22.9	35.0	13.4
Republic of Congo	Proportion	2017	25.8	37.0	15.4
Republic of Congo	Count	2000	14600	16400	12300
Republic of Congo	Count	2017	21900	25000	18600
Rwanda	Proportion	2000	12.0	14.6	9.8
Rwanda	Proportion	2017	33.9	45.4	22.9
Rwanda	Count	2000	62000	63500	60200
Rwanda	Count	2017	39100	45600	32300
São Tomé and Príncipe	Proportion	2000	38.6	43.8	33.1
São Tomé and Príncipe	Proportion	2017	50.6	75.8	28.3
São Tomé and Príncipe	Count	2000	587	639	537
São Tomé and Príncipe	Count	2017	429	623	210
Senegal	Proportion	2000	15.2	20.7	10.7
Senegal	Proportion	2017	23.9	31.5	16.9
Senegal	Count	2000	72300	76100	67500
Senegal	Count	2017	81100	88600	73100
Sierra Leone	Proportion	2000	57.0	68.5	44.8
Sierra Leone	Proportion	2017	76.7	83.9	68.3
Sierra Leone	Count	2000	15200	19500	11200
Sierra Leone	Count	2017	8080	11000	5580
Somalia	Proportion	2000	27.3	35.7	19.2
Somalia	Proportion	2017	30.8	40.9	22.9
Somalia	Count	2000	56300	62600	49800
Somalia	Count	2017	65900	73400	56300
South Africa	Proportion	2000	51.2	60.3	43.3
South Africa	Proportion	2017	50.0	57.2	42.2

Table 1: ORS by Country *(continued)*

Country	Metric	Year	Mean	Upper	Lower
South Africa	Count	2000	52200	60600	42400
South Africa	Count	2017	18100	20900	15500
South Sudan	Proportion	2000	52.0	62.2	41.6
South Sudan	Proportion	2017	48.4	59.5	37.6
South Sudan	Count	2000	38300	46600	30100
South Sudan	Count	2017	44700	54100	35100
Sri Lanka	Proportion	2000	45.6	64.7	30.2
Sri Lanka	Proportion	2017	42.8	61.2	23.7
Sri Lanka	Count	2000	11900	15300	7720
Sri Lanka	Count	2017	17400	23200	11800
Sudan	Proportion	2000	32.3	38.3	26.5
Sudan	Proportion	2017	19.7	26.2	14.6
Sudan	Count	2000	193000	209000	176000
Sudan	Count	2017	334000	355000	307000
Suriname	Proportion	2000	47.6	59.9	36.8
Suriname	Proportion	2017	37.6	52.1	26.7
Suriname	Count	2000	452	545	346
Suriname	Count	2017	398	467	305
Swaziland	Proportion	2000	76.3	82.2	69.5
Swaziland	Proportion	2017	79.7	89.1	68.6
Swaziland	Count	2000	1620	2080	1220
Swaziland	Count	2017	1080	1670	582
Syria	Proportion	2000	50.0	65.7	35.2
Syria	Proportion	2017	49.7	71.2	27.5
Syria	Count	2000	22000	28600	15100
Syria	Count	2017	16100	23200	9230
Tajikistan	Proportion	2000	36.2	39.4	33.2
Tajikistan	Proportion	2017	62.7	66.0	59.3
Tajikistan	Count	2000	19300	20200	18300
Tajikistan	Count	2017	12000	13100	10900
Tanzania	Proportion	2000	47.9	54.6	40.5
Tanzania	Proportion	2017	48.6	55.3	41.8
Tanzania	Count	2000	99200	113000	86400
Tanzania	Count	2017	116000	131000	101000
Thailand	Proportion	2000	57.2	66.1	47.2
Thailand	Proportion	2017	62.8	70.3	54.1
Thailand	Count	2000	34400	42500	27200
Thailand	Count	2017	20900	25800	16700
Timor-Leste	Proportion	2000	66.5	75.4	57.6
Timor-Leste	Proportion	2017	69.6	73.7	65.3
Timor-Leste	Count	2000	1660	2110	1220
Timor-Leste	Count	2017	1700	1930	1470
Togo	Proportion	2000	13.8	22.1	8.7
Togo	Proportion	2017	21.1	35.1	10.2
Togo	Count	2000	40700	43100	36800
Togo	Count	2017	37000	42200	30500
Tunisia	Proportion	2000	51.9	65.2	38.6
Tunisia	Proportion	2017	60.7	72.3	46.9
Tunisia	Count	2000	10900	13900	7880

Table 1: ORS by Country *(continued)*

Country	Metric	Year	Mean	Upper	Lower
Tunisia	Count	2017	9670	13100	6810
Turkmenistan	Proportion	2000	43.1	62.5	26.0
Turkmenistan	Proportion	2017	51.2	67.9	33.5
Turkmenistan	Count	2000	5750	7490	3790
Turkmenistan	Count	2017	2460	3360	1620
Uganda	Proportion	2000	38.2	45.4	30.4
Uganda	Proportion	2017	45.3	52.4	37.1
Uganda	Count	2000	149000	168000	132000
Uganda	Count	2017	129000	149000	112000
Uzbekistan	Proportion	2000	31.3	37.9	25.0
Uzbekistan	Proportion	2017	52.2	65.1	40.6
Uzbekistan	Count	2000	22200	24200	20000
Uzbekistan	Count	2017	16600	20600	12100
Vietnam	Proportion	2000	22.2	27.9	17.6
Vietnam	Proportion	2017	47.9	55.8	40.2
Vietnam	Count	2000	134000	142000	124000
Vietnam	Count	2017	92600	106000	78600
Yemen	Proportion	2000	38.4	45.9	31.2
Yemen	Proportion	2017	29.7	36.7	23.7
Yemen	Count	2000	177000	198000	155000
Yemen	Count	2017	272000	295000	245000
Zambia	Proportion	2000	57.7	65.2	51.0
Zambia	Proportion	2017	64.7	71.4	57.0
Zambia	Count	2000	40300	46800	33200
Zambia	Count	2017	38600	47000	31300
Zimbabwe	Proportion	2000	23.6	56.3	6.0
Zimbabwe	Proportion	2017	40.4	72.7	16.0
Zimbabwe	Count	2000	44300	54500	25300
Zimbabwe	Count	2017	56100	79000	25700

Table 2: RHF by Country

Country	Metric	Year	Mean	Upper	Lower
Afghanistan	Proportion	2000	21.6	46.7	4.8
Afghanistan	Proportion	2017	18.0	43.2	3.3
Afghanistan	Count	2000	176000	214000	120000
Afghanistan	Count	2017	312000	367000	216000
Algeria	Proportion	2000	13.1	55.2	0.5
Algeria	Proportion	2017	11.8	44.4	0.5
Algeria	Count	2000	1e+05	115000	51800
Algeria	Count	2017	115000	130000	72400
Angola	Proportion	2000	12.5	25.8	5.3
Angola	Proportion	2017	8.4	17.3	3.5
Angola	Count	2000	178000	192000	151000
Angola	Count	2017	207000	219000	187000
Bangladesh	Proportion	2000	21.5	41.7	7.8
Bangladesh	Proportion	2017	31.3	53.9	13.6
Bangladesh	Count	2000	267000	313000	198000
Bangladesh	Count	2017	128000	161000	85600
Belize	Proportion	2000	40.0	82.3	7.9
Belize	Proportion	2017	32.7	78.5	5.0
Belize	Count	2000	215	330	64
Belize	Count	2017	341	481	109
Benin	Proportion	2000	26.9	51.9	11.2
Benin	Proportion	2017	9.6	19.4	3.4
Benin	Count	2000	36400	44200	23900
Benin	Count	2017	37200	39800	33200
Bhutan	Proportion	2000	45.7	78.0	17.7
Bhutan	Proportion	2017	60.5	85.5	30.4
Bhutan	Count	2000	730	1110	296
Bhutan	Count	2017	700	1230	257
Bolivia	Proportion	2000	40.3	51.6	30.4
Bolivia	Proportion	2017	21.7	30.7	14.4
Bolivia	Count	2000	38900	45400	31600
Bolivia	Count	2017	37800	41300	33500
Botswana	Proportion	2000	17.6	22.4	13.1
Botswana	Proportion	2017	34.5	73.6	6.9
Botswana	Count	2000	4760	5030	4490
Botswana	Count	2017	3430	4880	1380
Burkina Faso	Proportion	2000	13.0	27.0	4.8
Burkina Faso	Proportion	2017	5.3	13.6	1.8
Burkina Faso	Count	2000	120000	132000	101000
Burkina Faso	Count	2017	129000	134000	118000
Burundi	Proportion	2000	21.8	37.9	9.9
Burundi	Proportion	2017	7.1	18.2	2.0
Burundi	Count	2000	40900	47100	32500
Burundi	Count	2017	56800	60000	50000
Cambodia	Proportion	2000	10.3	21.9	3.9
Cambodia	Proportion	2017	35.9	61.9	16.9
Cambodia	Count	2000	65000	69600	56500
Cambodia	Count	2017	35500	46000	21100
Cameroon	Proportion	2000	29.9	44.3	17.9

Table 2: RHF by Country *(continued)*

Country	Metric	Year	Mean	Upper	Lower
Cameroon	Proportion	2017	11.1	21.5	4.0
Cameroon	Count	2000	96400	113000	76600
Cameroon	Count	2017	178000	192000	157000
Central African Republic	Proportion	2000	45.3	47.1	43.4
Central African Republic	Proportion	2017	24.1	44.5	10.1
Central African Republic	Count	2000	21300	22100	20600
Central African Republic	Count	2017	24600	29100	18000
Chad	Proportion	2000	37.8	53.2	24.7
Chad	Proportion	2017	13.9	25.4	6.2
Chad	Count	2000	72500	87900	54600
Chad	Count	2017	178000	194000	154000
Colombia	Proportion	2000	21.3	29.8	14.4
Colombia	Proportion	2017	18.7	27.5	12.2
Colombia	Count	2000	148000	161000	132000
Colombia	Count	2017	114000	123000	101000
Comoros	Proportion	2000	40.8	77.6	10.8
Comoros	Proportion	2017	31.6	73.3	5.4
Comoros	Count	2000	2430	3660	918
Comoros	Count	2017	2040	2820	797
Costa Rica	Proportion	2000	32.1	75.3	2.2
Costa Rica	Proportion	2017	31.7	80.2	3.3
Costa Rica	Count	2000	7620	11000	2780
Costa Rica	Count	2017	8450	12000	2450
Côte d'Ivoire	Proportion	2000	26.5	43.5	13.0
Côte d'Ivoire	Proportion	2017	15.1	32.5	4.4
Côte d'Ivoire	Count	2000	108000	128000	82900
Côte d'Ivoire	Count	2017	146000	165000	116000
Democratic Republic of the Congo	Proportion	2000	33.6	50.0	20.0
Democratic Republic of the Congo	Proportion	2017	8.0	18.4	2.6
Democratic Republic of the Congo	Count	2000	321000	387000	242000
Democratic Republic of the Congo	Count	2017	566000	599000	502000
Djibouti	Proportion	2000	10.7	23.0	3.5
Djibouti	Proportion	2017	14.4	29.7	4.8
Djibouti	Count	2000	2680	2900	2320
Djibouti	Count	2017	2790	3110	2300
Dominican Republic	Proportion	2000	70.3	75.5	64.8
Dominican Republic	Proportion	2017	78.2	90.3	60.2
Dominican Republic	Count	2000	11000	13000	9040
Dominican Republic	Count	2017	7680	14000	3430
Ecuador	Proportion	2000	36.8	54.3	20.8
Ecuador	Proportion	2017	22.0	34.4	10.6
Ecuador	Count	2000	48700	61100	35200
Ecuador	Count	2017	28700	32900	24100
Egypt	Proportion	2000	5.5	7.6	4.0
Egypt	Proportion	2017	4.2	11.0	1.0
Egypt	Count	2000	334000	340000	327000
Egypt	Count	2017	469000	484000	436000
El Salvador	Proportion	2000	27.6	64.3	4.4
El Salvador	Proportion	2017	23.3	66.1	2.8

Table 2: RHF by Country *(continued)*

Country	Metric	Year	Mean	Upper	Lower
El Salvador	Count	2000	26100	34500	12900
El Salvador	Count	2017	16500	20900	7300
Equatorial Guinea	Proportion	2000	39.5	43.9	35.9
Equatorial Guinea	Proportion	2017	27.5	53.6	10.9
Equatorial Guinea	Count	2000	4530	4790	4200
Equatorial Guinea	Count	2017	5360	6590	3440
Eritrea	Proportion	2000	33.2	47.6	20.7
Eritrea	Proportion	2017	46.7	68.7	24.1
Eritrea	Count	2000	18800	22300	14700
Eritrea	Count	2017	14200	20200	8330
Ethiopia	Proportion	2000	9.8	10.9	8.8
Ethiopia	Proportion	2017	18.6	22.8	14.6
Ethiopia	Count	2000	579000	585000	572000
Ethiopia	Count	2017	419000	440000	397000
Gabon	Proportion	2000	18.9	22.7	15.9
Gabon	Proportion	2017	16.9	37.4	6.3
Gabon	Count	2000	5490	5690	5230
Gabon	Count	2017	7510	8470	5660
Gambia	Proportion	2000	21.3	39.0	10.3
Gambia	Proportion	2017	16.1	34.8	5.8
Gambia	Count	2000	9450	10800	7330
Gambia	Count	2017	10600	11900	8270
Ghana	Proportion	2000	15.7	31.8	6.1
Ghana	Proportion	2017	9.3	20.4	3.2
Ghana	Count	2000	93800	104000	75900
Ghana	Count	2017	122000	130000	107000
Guatemala	Proportion	2000	32.5	79.7	5.5
Guatemala	Proportion	2017	26.8	72.1	3.0
Guatemala	Count	2000	71000	99500	21400
Guatemala	Count	2017	84400	112000	32200
Guinea	Proportion	2000	12.7	27.9	4.6
Guinea	Proportion	2017	5.3	11.9	1.6
Guinea	Count	2000	61500	67200	50800
Guinea	Count	2017	71500	74400	66500
Guinea-Bissau	Proportion	2000	39.5	55.6	22.4
Guinea-Bissau	Proportion	2017	10.8	24.2	3.5
Guinea-Bissau	Count	2000	6680	8570	4910
Guinea-Bissau	Count	2017	8370	9060	7120
Guyana	Proportion	2000	58.0	72.7	41.0
Guyana	Proportion	2017	22.3	55.4	6.2
Guyana	Count	2000	823	1160	535
Guyana	Count	2017	990	1200	569
Haiti	Proportion	2000	8.3	9.7	7.0
Haiti	Proportion	2017	7.9	9.9	6.4
Haiti	Count	2000	67100	68100	66100
Haiti	Count	2017	55000	55900	53800
Honduras	Proportion	2000	23.5	67.3	4.1
Honduras	Proportion	2017	19.0	58.4	2.6
Honduras	Count	2000	41700	52300	17800

Table 2: RHF by Country *(continued)*

Country	Metric	Year	Mean	Upper	Lower
Honduras	Count	2017	49700	59800	25600
India	Proportion	2000	15.4	24.1	8.1
India	Proportion	2017	26.5	32.6	20.9
India	Count	2000	1890000	2050000	1690000
India	Count	2017	1280000	1370000	1170000
Indonesia	Proportion	2000	31.6	37.2	26.8
Indonesia	Proportion	2017	23.1	26.9	19.4
Indonesia	Count	2000	386000	414000	355000
Indonesia	Count	2017	587000	615000	558000
Iraq	Proportion	2000	69.8	72.1	67.4
Iraq	Proportion	2017	65.6	90.1	27.5
Iraq	Count	2000	34400	37200	31800
Iraq	Count	2017	45900	96600	13200
Jamaica	Proportion	2000	36.6	71.1	10.2
Jamaica	Proportion	2017	26.1	67.2	7.2
Jamaica	Count	2000	1430	2020	651
Jamaica	Count	2017	1670	2090	740
Jordan	Proportion	2000	52.7	87.4	18.5
Jordan	Proportion	2017	19.4	73.3	0.5
Jordan	Count	2000	7460	12900	1990
Jordan	Count	2017	26000	32000	8600
Kenya	Proportion	2000	67.4	71.2	63.3
Kenya	Proportion	2017	26.3	57.1	6.5
Kenya	Count	2000	66900	75300	59000
Kenya	Count	2017	165000	210000	96200
Kyrgyzstan	Proportion	2000	43.5	67.0	22.5
Kyrgyzstan	Proportion	2017	51.5	76.6	24.9
Kyrgyzstan	Count	2000	5920	8120	3460
Kyrgyzstan	Count	2017	4360	6750	2110
Laos	Proportion	2000	33.0	52.7	18.5
Laos	Proportion	2017	28.8	48.0	14.3
Laos	Count	2000	11900	14400	8390
Laos	Count	2017	16000	19300	11700
Lesotho	Proportion	2000	45.5	75.2	16.8
Lesotho	Proportion	2017	42.7	72.8	16.0
Lesotho	Count	2000	5500	8410	2510
Lesotho	Count	2017	3790	5560	1800
Liberia	Proportion	2000	10.6	22.3	3.5
Liberia	Proportion	2017	8.3	21.8	2.2
Liberia	Count	2000	21000	22700	18300
Liberia	Count	2017	36700	39100	31300
Madagascar	Proportion	2000	33.1	48.3	19.3
Madagascar	Proportion	2017	24.5	42.3	12.0
Madagascar	Count	2000	75300	90900	58200
Madagascar	Count	2017	160000	187000	122000
Malawi	Proportion	2000	55.4	80.0	30.8
Malawi	Proportion	2017	42.3	67.9	20.1
Malawi	Count	2000	43800	68000	19700
Malawi	Count	2017	89200	123000	49600

Table 2: RHF by Country *(continued)*

Country	Metric	Year	Mean	Upper	Lower
Mali	Proportion	2000	22.3	36.5	10.0
Mali	Proportion	2017	11.0	22.8	4.5
Mali	Count	2000	93400	108000	76400
Mali	Count	2017	101000	109000	87700
Mauritania	Proportion	2000	23.1	37.6	11.2
Mauritania	Proportion	2017	19.0	34.0	8.3
Mauritania	Count	2000	16800	19400	13600
Mauritania	Count	2017	15100	17100	12300
Mexico	Proportion	2000	28.1	69.0	5.3
Mexico	Proportion	2017	24.0	59.5	3.3
Mexico	Count	2000	332000	437000	143000
Mexico	Count	2017	228000	290000	122000
Mongolia	Proportion	2000	77.1	80.5	73.9
Mongolia	Proportion	2017	49.8	84.2	20.3
Mongolia	Count	2000	1080	1230	919
Mongolia	Count	2017	4160	6600	1300
Morocco	Proportion	2000	4.0	8.4	1.3
Morocco	Proportion	2017	3.9	10.0	1.2
Morocco	Count	2000	115000	119000	110000
Morocco	Count	2017	89600	92100	83900
Mozambique	Proportion	2000	20.7	34.9	11.7
Mozambique	Proportion	2017	16.0	26.6	8.3
Mozambique	Count	2000	101000	113000	83000
Mozambique	Count	2017	97200	106000	84900
Myanmar	Proportion	2000	33.6	52.8	18.2
Myanmar	Proportion	2017	23.9	40.5	12.4
Myanmar	Count	2000	127000	157000	90300
Myanmar	Count	2017	94600	109000	73900
Namibia	Proportion	2000	15.2	36.1	4.0
Namibia	Proportion	2017	28.7	65.0	6.6
Namibia	Count	2000	6550	7410	4930
Namibia	Count	2017	7460	9770	3660
Nepal	Proportion	2000	29.4	55.5	11.4
Nepal	Proportion	2017	41.3	60.3	22.4
Nepal	Count	2000	67800	85200	42800
Nepal	Count	2017	28300	37400	19100
Nicaragua	Proportion	2000	14.7	49.5	2.2
Nicaragua	Proportion	2017	13.0	42.8	1.4
Nicaragua	Count	2000	28800	33000	17100
Nicaragua	Count	2017	25400	28800	16700
Niger	Proportion	2000	34.4	49.6	21.6
Niger	Proportion	2017	14.0	27.7	6.1
Niger	Count	2000	132000	158000	102000
Niger	Count	2017	145000	158000	122000
Nigeria	Proportion	2000	18.2	25.1	12.1
Nigeria	Proportion	2017	18.6	27.4	12.5
Nigeria	Count	2000	785000	843000	719000
Nigeria	Count	2017	801000	860000	713000
Pakistan	Proportion	2000	17.8	22.3	13.7

Table 2: RHF by Country *(continued)*

Country	Metric	Year	Mean	Upper	Lower
Pakistan	Proportion	2017	10.6	13.1	8.6
Pakistan	Count	2000	429000	449000	405000
Pakistan	Count	2017	745000	762000	724000
Panama	Proportion	2000	26.2	73.0	2.5
Panama	Proportion	2017	28.4	73.1	2.3
Panama	Count	2000	12200	16200	4480
Panama	Count	2017	12500	17000	4680
Papua New Guinea	Proportion	2000	16.6	27.6	8.6
Papua New Guinea	Proportion	2017	16.8	26.8	8.9
Papua New Guinea	Count	2000	34100	37400	29600
Papua New Guinea	Count	2017	51500	56400	45300
Paraguay	Proportion	2000	23.7	40.2	12.0
Paraguay	Proportion	2017	19.2	32.3	9.2
Paraguay	Count	2000	15000	17300	11800
Paraguay	Count	2017	26700	30100	22400
Peru	Proportion	2000	15.4	21.1	10.8
Peru	Proportion	2017	10.4	14.2	7.5
Peru	Count	2000	113000	119000	106000
Peru	Count	2017	109000	112000	104000
Philippines	Proportion	2000	20.7	27.1	15.0
Philippines	Proportion	2017	19.0	24.9	14.5
Philippines	Count	2000	209000	224000	192000
Philippines	Count	2017	2e+05	211000	185000
Republic of Congo	Proportion	2000	18.1	32.1	9.3
Republic of Congo	Proportion	2017	13.7	28.4	5.4
Republic of Congo	Count	2000	15500	17200	12900
Republic of Congo	Count	2017	25500	28000	21200
Rwanda	Proportion	2000	28.1	41.6	16.1
Rwanda	Proportion	2017	10.7	25.8	3.4
Rwanda	Count	2000	50600	59100	41100
Rwanda	Count	2017	52800	57100	43900
São Tomé and Príncipe	Proportion	2000	31.9	37.3	27.4
São Tomé and Príncipe	Proportion	2017	20.2	41.5	6.6
São Tomé and Príncipe	Count	2000	650	693	599
São Tomé and Príncipe	Count	2017	694	811	509
Senegal	Proportion	2000	16.7	26.2	9.4
Senegal	Proportion	2017	11.4	20.6	5.3
Senegal	Count	2000	71000	77200	62900
Senegal	Count	2017	94500	101000	84700
Sierra Leone	Proportion	2000	23.9	42.3	10.5
Sierra Leone	Proportion	2017	13.3	28.3	5.3
Sierra Leone	Count	2000	27000	31700	20500
Sierra Leone	Count	2017	30000	32800	24800
Somalia	Proportion	2000	12.2	25.2	5.0
Somalia	Proportion	2017	13.2	27.8	5.8
Somalia	Count	2000	67900	73500	57900
Somalia	Count	2017	82700	89700	68700
South Africa	Proportion	2000	39.7	66.2	17.2
South Africa	Proportion	2017	63.0	86.8	33.6

Table 2: RHF by Country *(continued)*

Country	Metric	Year	Mean	Upper	Lower
South Africa	Count	2000	64500	88500	36100
South Africa	Count	2017	13400	24000	4760
South Sudan	Proportion	2000	61.0	66.9	55.6
South Sudan	Proportion	2017	56.0	75.3	35.8
South Sudan	Count	2000	31100	35400	26400
South Sudan	Count	2017	38100	55600	21400
Sri Lanka	Proportion	2000	35.4	79.7	4.8
Sri Lanka	Proportion	2017	48.3	90.9	9.8
Sri Lanka	Count	2000	14100	20800	4440
Sri Lanka	Count	2017	15700	27400	2750
Sudan	Proportion	2000	47.3	48.6	45.8
Sudan	Proportion	2017	55.3	71.3	37.5
Sudan	Count	2000	150000	154000	146000
Sudan	Count	2017	186000	260000	119000
Suriname	Proportion	2000	58.0	81.2	31.6
Suriname	Proportion	2017	28.5	46.0	15.6
Suriname	Count	2000	362	590	162
Suriname	Count	2017	455	538	344
Swaziland	Proportion	2000	41.7	64.4	19.9
Swaziland	Proportion	2017	23.2	52.2	7.5
Swaziland	Count	2000	3980	5470	2430
Swaziland	Count	2017	4090	4920	2540
Syria	Proportion	2000	25.7	60.1	5.0
Syria	Proportion	2017	25.6	63.8	4.2
Syria	Count	2000	32800	41900	17600
Syria	Count	2017	23800	30700	11600
Tajikistan	Proportion	2000	36.8	40.8	33.4
Tajikistan	Proportion	2017	37.0	40.5	33.2
Tajikistan	Count	2000	19100	20100	17900
Tajikistan	Count	2017	20200	21400	19100
Tanzania	Proportion	2000	25.1	41.4	12.8
Tanzania	Proportion	2017	14.3	25.6	6.4
Tanzania	Count	2000	143000	166000	111000
Tanzania	Count	2017	193000	211000	168000
Thailand	Proportion	2000	36.3	56.7	20.3
Thailand	Proportion	2017	47.2	68.0	28.0
Thailand	Count	2000	51200	64100	34800
Thailand	Count	2017	29700	40500	18000
Timor-Leste	Proportion	2000	47.6	61.5	32.9
Timor-Leste	Proportion	2017	46.3	59.3	33.2
Timor-Leste	Count	2000	2610	3340	1920
Timor-Leste	Count	2017	3000	3730	2270
Togo	Proportion	2000	22.8	42.8	9.1
Togo	Proportion	2017	8.4	21.6	2.2
Togo	Count	2000	36500	42900	27000
Togo	Count	2017	43000	45900	36800
Tunisia	Proportion	2000	8.4	29.5	0.4
Tunisia	Proportion	2017	7.2	28.2	0.5
Tunisia	Count	2000	20700	22500	16000

Table 2: RHF by Country *(continued)*

Country	Metric	Year	Mean	Upper	Lower
Tunisia	Count	2017	22800	24500	17700
Turkmenistan	Proportion	2000	11.6	30.5	3.2
Turkmenistan	Proportion	2017	15.6	42.6	2.8
Turkmenistan	Count	2000	8940	9790	7030
Turkmenistan	Count	2017	4260	4900	2900
Uganda	Proportion	2000	17.6	32.6	7.9
Uganda	Proportion	2017	12.0	24.9	4.3
Uganda	Count	2000	199000	223000	163000
Uganda	Count	2017	208000	226000	177000
Uzbekistan	Proportion	2000	69.9	76.4	62.2
Uzbekistan	Proportion	2017	73.0	86.6	57.1
Uzbekistan	Count	2000	9700	12200	7610
Uzbekistan	Count	2017	9370	14900	4630
Vietnam	Proportion	2000	36.3	54.1	20.0
Vietnam	Proportion	2017	44.5	64.1	24.4
Vietnam	Count	2000	110000	138000	79000
Vietnam	Count	2017	98800	134000	63900
Yemen	Proportion	2000	0.9	2.1	0.4
Yemen	Proportion	2017	26.8	48.8	11.4
Yemen	Count	2000	284000	286000	281000
Yemen	Count	2017	283000	342000	198000
Zambia	Proportion	2000	25.8	47.1	12.1
Zambia	Proportion	2017	13.3	26.9	5.2
Zambia	Count	2000	70800	83900	50500
Zambia	Count	2017	94800	104000	80000
Zimbabwe	Proportion	2000	54.8	67.3	42.1
Zimbabwe	Proportion	2017	53.6	67.1	40.2
Zimbabwe	Count	2000	26200	33600	19000
Zimbabwe	Count	2017	43600	56300	30900

Table 3: ORT by Country

Country	Metric	Year	Mean	Upper	Lower
Afghanistan	Proportion	2000	63.7	80.8	39.7
Afghanistan	Proportion	2017	58.3	72.7	44.9
Afghanistan	Count	2000	81500	135000	43000
Afghanistan	Count	2017	158000	209000	104000
Algeria	Proportion	2000	32.0	41.9	23.7
Algeria	Proportion	2017	33.6	42.2	25.7
Algeria	Count	2000	78500	88000	67100
Algeria	Count	2017	86500	96800	75300
Angola	Proportion	2000	42.7	61.2	25.1
Angola	Proportion	2017	43.4	62.6	28.5
Angola	Count	2000	116000	152000	78800
Angola	Count	2017	128000	162000	84600
Bangladesh	Proportion	2000	63.6	73.1	53.4
Bangladesh	Proportion	2017	82.1	87.6	75.4
Bangladesh	Count	2000	124000	158000	91600
Bangladesh	Count	2017	33200	45700	23000
Belize	Proportion	2000	58.9	75.6	41.0
Belize	Proportion	2017	57.5	73.1	44.7
Belize	Count	2000	147	211	87
Belize	Count	2017	215	280	136
Benin	Proportion	2000	38.9	54.8	24.9
Benin	Proportion	2017	30.7	44.5	19.7
Benin	Count	2000	30400	37400	22500
Benin	Count	2017	28500	33100	22900
Bhutan	Proportion	2000	62.9	80.3	42.6
Bhutan	Proportion	2017	78.1	88.6	63.1
Bhutan	Count	2000	499	772	265
Bhutan	Count	2017	388	654	203
Bolivia	Proportion	2000	62.6	69.9	56.0
Bolivia	Proportion	2017	52.1	60.7	43.5
Bolivia	Count	2000	24400	28700	19600
Bolivia	Count	2017	23100	27300	19000
Botswana	Proportion	2000	61.2	67.0	54.3
Botswana	Proportion	2017	70.7	81.3	58.7
Botswana	Count	2000	2250	2640	1910
Botswana	Count	2017	1530	2160	979
Burkina Faso	Proportion	2000	25.5	40.1	14.3
Burkina Faso	Proportion	2017	20.7	34.7	11.0
Burkina Faso	Count	2000	103000	118000	82700
Burkina Faso	Count	2017	108000	121000	89000
Burundi	Proportion	2000	43.7	59.0	31.5
Burundi	Proportion	2017	35.2	48.8	21.8
Burundi	Count	2000	29400	35800	21400
Burundi	Count	2017	39700	47800	31300
Cambodia	Proportion	2000	25.5	33.3	19.1
Cambodia	Proportion	2017	43.2	55.2	32.1
Cambodia	Count	2000	54000	58600	48300
Cambodia	Count	2017	31500	37600	24800
Cameroon	Proportion	2000	40.7	52.4	30.0

Table 3: ORT by Country *(continued)*

Country	Metric	Year	Mean	Upper	Lower
Cameroon	Proportion	2017	28.9	43.7	18.2
Cameroon	Count	2000	81600	96300	65500
Cameroon	Count	2017	142000	163000	113000
Central African Republic	Proportion	2000	38.8	49.3	29.3
Central African Republic	Proportion	2017	33.7	50.6	20.0
Central African Republic	Count	2000	23900	27600	19800
Central African Republic	Count	2017	21500	25900	16000
Chad	Proportion	2000	52.0	63.8	40.2
Chad	Proportion	2017	29.5	42.9	19.6
Chad	Count	2000	56000	69800	42200
Chad	Count	2017	146000	166000	118000
Colombia	Proportion	2000	45.9	49.3	42.6
Colombia	Proportion	2017	57.8	66.0	48.3
Colombia	Count	2000	101000	108000	95100
Colombia	Count	2017	58900	72300	47500
Comoros	Proportion	2000	53.2	75.1	24.2
Comoros	Proportion	2017	59.3	81.7	33.7
Comoros	Count	2000	1920	3110	1020
Comoros	Count	2017	1210	1980	548
Costa Rica	Proportion	2000	57.2	79.7	31.7
Costa Rica	Proportion	2017	57.2	79.5	34.8
Costa Rica	Count	2000	4800	7660	2280
Costa Rica	Count	2017	5300	8060	2540
Côte d'Ivoire	Proportion	2000	41.2	53.9	30.4
Côte d'Ivoire	Proportion	2017	34.5	48.8	21.2
Côte d'Ivoire	Count	2000	86200	102000	67600
Côte d'Ivoire	Count	2017	113000	136000	88300
Democratic Republic of the Congo	Proportion	2000	42.0	46.9	36.7
Democratic Republic of the Congo	Proportion	2017	40.6	47.8	35.6
Democratic Republic of the Congo	Count	2000	280000	306000	257000
Democratic Republic of the Congo	Count	2017	365000	396000	321000
Djibouti	Proportion	2000	67.8	80.7	52.6
Djibouti	Proportion	2017	72.1	87.9	51.2
Djibouti	Count	2000	968	1420	579
Djibouti	Count	2017	911	1590	396
Dominican Republic	Proportion	2000	58.2	69.2	48.1
Dominican Republic	Proportion	2017	60.8	72.8	49.1
Dominican Republic	Count	2000	15400	19100	11400
Dominican Republic	Count	2017	13800	17900	9580
Ecuador	Proportion	2000	45.9	59.3	33.2
Ecuador	Proportion	2017	46.4	59.0	36.2
Ecuador	Count	2000	41700	51400	31400
Ecuador	Count	2017	19700	23500	15100
Egypt	Proportion	2000	37.1	40.4	33.1
Egypt	Proportion	2017	30.1	38.5	22.0
Egypt	Count	2000	223000	237000	211000
Egypt	Count	2017	342000	382000	301000
El Salvador	Proportion	2000	57.2	73.2	39.7
El Salvador	Proportion	2017	64.4	78.2	47.3

Table 3: ORT by Country *(continued)*

Country	Metric	Year	Mean	Upper	Lower
El Salvador	Count	2000	15500	21800	9670
El Salvador	Count	2017	7660	11300	4700
Equatorial Guinea	Proportion	2000	50.7	65.0	37.1
Equatorial Guinea	Proportion	2017	49.1	74.3	27.1
Equatorial Guinea	Count	2000	3690	4700	2610
Equatorial Guinea	Count	2017	3770	5390	1900
Eritrea	Proportion	2000	59.4	71.8	46.9
Eritrea	Proportion	2017	64.2	78.6	47.9
Eritrea	Count	2000	11400	14900	7950
Eritrea	Count	2017	9510	13900	5680
Ethiopia	Proportion	2000	19.1	21.0	17.0
Ethiopia	Proportion	2017	40.1	44.1	36.1
Ethiopia	Count	2000	519000	533000	507000
Ethiopia	Count	2017	308000	329000	288000
Gabon	Proportion	2000	34.4	47.3	23.2
Gabon	Proportion	2017	36.5	55.8	21.0
Gabon	Count	2000	4440	5190	3570
Gabon	Count	2017	5740	7150	3990
Gambia	Proportion	2000	61.3	74.4	47.1
Gambia	Proportion	2017	48.8	64.9	32.6
Gambia	Count	2000	4640	6350	3070
Gambia	Count	2017	6500	8550	4450
Ghana	Proportion	2000	43.0	60.3	28.4
Ghana	Proportion	2017	46.8	63.7	29.1
Ghana	Count	2000	63400	79600	44200
Ghana	Count	2017	71400	95100	48700
Guatemala	Proportion	2000	58.8	73.5	41.9
Guatemala	Proportion	2017	59.3	73.2	42.1
Guatemala	Count	2000	43400	61200	27900
Guatemala	Count	2017	46800	66800	30900
Guinea	Proportion	2000	35.0	49.1	21.7
Guinea	Proportion	2017	30.1	44.8	18.5
Guinea	Count	2000	45700	55200	35900
Guinea	Count	2017	52800	61500	41700
Guinea-Bissau	Proportion	2000	58.7	72.0	43.8
Guinea-Bissau	Proportion	2017	35.9	54.8	18.7
Guinea-Bissau	Count	2000	4560	6200	3090
Guinea-Bissau	Count	2017	6020	7630	4240
Guyana	Proportion	2000	81.6	86.1	76.6
Guyana	Proportion	2017	51.1	64.9	39.1
Guyana	Count	2000	360	459	272
Guyana	Count	2017	623	776	447
Haiti	Proportion	2000	48.5	60.0	36.3
Haiti	Proportion	2017	42.5	54.3	32.0
Haiti	Count	2000	37700	46600	29300
Haiti	Count	2017	34300	40600	27300
Honduras	Proportion	2000	57.4	74.2	40.8
Honduras	Proportion	2017	58.3	76.0	42.2
Honduras	Count	2000	23200	32300	14100

Table 3: ORT by Country *(continued)*

Country	Metric	Year	Mean	Upper	Lower
Honduras	Count	2017	25600	35500	14700
India	Proportion	2000	40.8	47.8	34.1
India	Proportion	2017	62.7	65.8	59.0
India	Count	2000	1320000	1470000	1160000
India	Count	2017	648000	712000	594000
Indonesia	Proportion	2000	53.4	56.6	49.9
Indonesia	Proportion	2017	44.6	46.7	42.7
Indonesia	Count	2000	263000	283000	245000
Indonesia	Count	2017	423000	438000	407000
Iraq	Proportion	2000	77.3	79.2	75.1
Iraq	Proportion	2017	49.1	72.9	25.2
Iraq	Count	2000	25900	28400	23700
Iraq	Count	2017	67900	99700	36100
Jamaica	Proportion	2000	62.6	78.9	42.1
Jamaica	Proportion	2017	62.0	77.4	41.2
Jamaica	Count	2000	843	1310	477
Jamaica	Count	2017	857	1330	510
Jordan	Proportion	2000	55.2	75.5	34.2
Jordan	Proportion	2017	30.9	66.0	5.1
Jordan	Count	2000	7070	10400	3870
Jordan	Count	2017	22300	30600	10900
Kenya	Proportion	2000	82.6	84.9	79.7
Kenya	Proportion	2017	60.2	81.7	38.0
Kenya	Count	2000	35700	41700	30900
Kenya	Count	2017	89300	139000	41100
Kyrgyzstan	Proportion	2000	34.6	54.8	16.6
Kyrgyzstan	Proportion	2017	59.4	81.0	31.3
Kyrgyzstan	Count	2000	6850	8740	4740
Kyrgyzstan	Count	2017	3650	6180	1710
Laos	Proportion	2000	58.3	65.2	49.9
Laos	Proportion	2017	62.0	70.5	54.5
Laos	Count	2000	7400	8880	6170
Laos	Count	2017	8560	10200	6640
Lesotho	Proportion	2000	69.0	86.0	53.0
Lesotho	Proportion	2017	74.5	87.7	59.4
Lesotho	Count	2000	3130	4750	1410
Lesotho	Count	2017	1690	2690	812
Liberia	Proportion	2000	66.9	82.1	48.8
Liberia	Proportion	2017	77.5	88.6	63.7
Liberia	Count	2000	7800	12100	4220
Liberia	Count	2017	8990	14500	4560
Madagascar	Proportion	2000	43.0	54.8	31.6
Madagascar	Proportion	2017	43.8	57.7	30.9
Madagascar	Count	2000	64100	77000	50900
Madagascar	Count	2017	119000	147000	89700
Malawi	Proportion	2000	63.2	73.2	52.3
Malawi	Proportion	2017	69.8	79.8	57.7
Malawi	Count	2000	36200	46800	26400
Malawi	Count	2017	46700	65300	31300

Table 3: ORT by Country *(continued)*

Country	Metric	Year	Mean	Upper	Lower
Mali	Proportion	2000	35.6	46.7	25.7
Mali	Proportion	2017	30.1	42.9	17.9
Mali	Count	2000	77500	89400	64100
Mali	Count	2017	79400	93300	64900
Mauritania	Proportion	2000	38.9	50.0	28.2
Mauritania	Proportion	2017	40.6	54.5	27.2
Mauritania	Count	2000	13300	15700	10900
Mauritania	Count	2017	11100	13600	8490
Mexico	Proportion	2000	71.9	83.1	59.7
Mexico	Proportion	2017	71.3	83.0	58.3
Mexico	Count	2000	129000	186000	77800
Mexico	Count	2017	86100	125000	51100
Mongolia	Proportion	2000	86.6	89.3	83.7
Mongolia	Proportion	2017	73.5	89.2	50.9
Mongolia	Count	2000	633	769	504
Mongolia	Count	2017	2200	4060	891
Morocco	Proportion	2000	24.3	33.1	17.3
Morocco	Proportion	2017	27.4	36.0	19.9
Morocco	Count	2000	91000	99400	80400
Morocco	Count	2017	67700	74600	59700
Mozambique	Proportion	2000	51.9	62.6	39.9
Mozambique	Proportion	2017	59.2	69.9	47.4
Mozambique	Count	2000	61300	76600	47700
Mozambique	Count	2017	47200	60900	34800
Myanmar	Proportion	2000	67.8	74.8	60.9
Myanmar	Proportion	2017	68.8	75.8	61.2
Myanmar	Count	2000	61600	74900	48200
Myanmar	Count	2017	38800	48300	30100
Namibia	Proportion	2000	63.0	67.4	59.0
Namibia	Proportion	2017	71.7	81.8	59.9
Namibia	Count	2000	2860	3170	2520
Namibia	Count	2017	2960	4190	1910
Nepal	Proportion	2000	35.1	44.3	27.3
Nepal	Proportion	2017	53.9	63.5	44.0
Nepal	Count	2000	62400	69900	53500
Nepal	Count	2017	22200	27000	17600
Nicaragua	Proportion	2000	56.8	70.0	43.1
Nicaragua	Proportion	2017	60.1	74.3	43.2
Nicaragua	Count	2000	14600	19200	10100
Nicaragua	Count	2017	11700	16600	7500
Niger	Proportion	2000	54.3	65.5	40.8
Niger	Proportion	2017	41.2	56.2	27.6
Niger	Count	2000	92100	119000	69500
Niger	Count	2017	98800	122000	73600
Nigeria	Proportion	2000	38.3	51.3	26.4
Nigeria	Proportion	2017	48.3	58.8	38.7
Nigeria	Count	2000	592000	706000	467000
Nigeria	Count	2017	508000	603000	405000
Pakistan	Proportion	2000	56.3	62.3	51.5

Table 3: ORT by Country *(continued)*

Country	Metric	Year	Mean	Upper	Lower
Pakistan	Proportion	2017	63.7	69.8	57.5
Pakistan	Count	2000	228000	253000	196000
Pakistan	Count	2017	302000	354000	251000
Panama	Proportion	2000	48.8	65.0	33.6
Panama	Proportion	2017	46.1	61.5	31.7
Panama	Count	2000	8490	11000	5810
Panama	Count	2017	9380	11900	6710
Papua New Guinea	Proportion	2000	30.9	38.9	24.2
Papua New Guinea	Proportion	2017	34.3	43.9	26.2
Papua New Guinea	Count	2000	28300	31000	25000
Papua New Guinea	Count	2017	40700	45700	34700
Paraguay	Proportion	2000	45.9	61.3	34.1
Paraguay	Proportion	2017	48.6	62.2	36.3
Paraguay	Count	2000	10600	13000	7610
Paraguay	Count	2017	17000	21100	12500
Peru	Proportion	2000	38.5	44.8	32.5
Peru	Proportion	2017	36.3	42.4	30.7
Peru	Count	2000	82400	90400	73900
Peru	Count	2017	77300	84200	69900
Philippines	Proportion	2000	55.7	61.5	50.2
Philippines	Proportion	2017	53.6	57.0	49.8
Philippines	Count	2000	117000	131000	101000
Philippines	Count	2017	114000	124000	106000
Republic of Congo	Proportion	2000	30.9	48.6	16.7
Republic of Congo	Proportion	2017	30.6	44.7	17.6
Republic of Congo	Count	2000	13100	15800	9740
Republic of Congo	Count	2017	20500	24400	16400
Rwanda	Proportion	2000	35.3	45.6	25.6
Rwanda	Proportion	2017	40.6	56.2	26.6
Rwanda	Count	2000	45600	52400	38300
Rwanda	Count	2017	35100	43400	25900
São Tomé and Príncipe	Proportion	2000	53.9	66.1	39.0
São Tomé and Príncipe	Proportion	2017	50.2	73.7	27.5
São Tomé and Príncipe	Count	2000	440	582	324
São Tomé and Príncipe	Count	2017	433	630	228
Senegal	Proportion	2000	44.5	55.2	33.2
Senegal	Proportion	2017	28.7	39.1	20.3
Senegal	Count	2000	47300	56900	38200
Senegal	Count	2017	76000	85000	64900
Sierra Leone	Proportion	2000	67.5	80.5	54.0
Sierra Leone	Proportion	2017	74.5	83.6	62.6
Sierra Leone	Count	2000	11500	16300	6920
Sierra Leone	Count	2017	8820	13000	5690
Somalia	Proportion	2000	29.1	40.9	17.7
Somalia	Proportion	2017	32.9	48.4	19.1
Somalia	Count	2000	54900	63700	45800
Somalia	Count	2017	63900	77000	49200
South Africa	Proportion	2000	66.1	75.9	54.9
South Africa	Proportion	2017	74.7	81.3	66.4

Table 3: ORT by Country *(continued)*

Country	Metric	Year	Mean	Upper	Lower
South Africa	Count	2000	36300	48100	25800
South Africa	Count	2017	9120	12100	6760
South Sudan	Proportion	2000	69.5	78.5	60.4
South Sudan	Proportion	2017	59.3	76.3	43.2
South Sudan	Count	2000	24300	31600	17200
South Sudan	Count	2017	35200	49200	20600
Sri Lanka	Proportion	2000	38.6	60.3	18.9
Sri Lanka	Proportion	2017	47.7	67.1	28.2
Sri Lanka	Count	2000	13400	17700	8700
Sri Lanka	Count	2017	15900	21800	10000
Sudan	Proportion	2000	61.1	69.4	52.6
Sudan	Proportion	2017	58.7	70.8	46.2
Sudan	Count	2000	111000	135000	87000
Sudan	Count	2017	172000	224000	122000
Suriname	Proportion	2000	80.5	88.3	68.9
Suriname	Proportion	2017	54.3	67.4	38.2
Suriname	Count	2000	168	269	101
Suriname	Count	2017	291	394	207
Swaziland	Proportion	2000	86.2	93.0	76.5
Swaziland	Proportion	2017	86.0	93.3	73.9
Swaziland	Count	2000	940	1600	480
Swaziland	Count	2017	745	1390	356
Syria	Proportion	2000	60.9	77.3	41.6
Syria	Proportion	2017	49.6	77.6	20.8
Syria	Count	2000	17300	25700	10000
Syria	Count	2017	16200	25400	7170
Tajikistan	Proportion	2000	61.7	65.1	57.9
Tajikistan	Proportion	2017	70.1	73.2	66.6
Tajikistan	Count	2000	11600	12700	10600
Tajikistan	Count	2017	9610	10700	8610
Tanzania	Proportion	2000	57.3	70.8	44.5
Tanzania	Proportion	2017	58.0	70.9	45.2
Tanzania	Count	2000	81200	106000	55600
Tanzania	Count	2017	94700	124000	65600
Thailand	Proportion	2000	61.8	70.9	52.8
Thailand	Proportion	2017	70.3	78.7	60.7
Thailand	Count	2000	30700	38000	23400
Thailand	Count	2017	16700	22100	12000
Timor-Leste	Proportion	2000	75.1	81.3	68.1
Timor-Leste	Proportion	2017	77.2	81.1	73.1
Timor-Leste	Count	2000	1240	1590	928
Timor-Leste	Count	2017	1270	1500	1050
Togo	Proportion	2000	38.4	54.3	24.8
Togo	Proportion	2017	34.3	54.0	19.7
Togo	Count	2000	29100	35500	21600
Togo	Count	2017	30800	37700	21600
Tunisia	Proportion	2000	46.5	62.7	31.8
Tunisia	Proportion	2017	60.9	75.9	45.1
Tunisia	Count	2000	12100	15400	8450

Table 3: ORT by Country *(continued)*

Country	Metric	Year	Mean	Upper	Lower
Tunisia	Count	2017	9620	13500	5920
Turkmenistan	Proportion	2000	36.5	61.6	17.0
Turkmenistan	Proportion	2017	48.9	71.0	27.1
Turkmenistan	Count	2000	6420	8390	3880
Turkmenistan	Count	2017	2580	3680	1460
Uganda	Proportion	2000	47.3	61.0	35.3
Uganda	Proportion	2017	50.5	63.6	37.8
Uganda	Count	2000	127000	156000	94100
Uganda	Count	2017	117000	147000	85900
Uzbekistan	Proportion	2000	72.4	78.2	66.0
Uzbekistan	Proportion	2017	85.9	93.0	76.0
Uzbekistan	Count	2000	8910	11000	7030
Uzbekistan	Count	2017	4890	8310	2430
Vietnam	Proportion	2000	56.7	64.4	48.2
Vietnam	Proportion	2017	65.7	74.3	56.5
Vietnam	Count	2000	74500	89100	61200
Vietnam	Count	2017	61000	77300	45700
Yemen	Proportion	2000	38.6	47.7	31.2
Yemen	Proportion	2017	44.8	52.8	38.0
Yemen	Count	2000	176000	197000	150000
Yemen	Count	2017	213000	240000	183000
Zambia	Proportion	2000	61.4	73.6	47.6
Zambia	Proportion	2017	66.3	77.5	53.5
Zambia	Count	2000	36900	50000	25200
Zambia	Count	2017	36900	50900	24600
Zimbabwe	Proportion	2000	66.7	78.4	52.5
Zimbabwe	Proportion	2017	67.9	79.8	54.9
Zimbabwe	Count	2000	19300	27500	12500
Zimbabwe	Count	2017	30200	42400	19000

Table 4: ORS by Unit

Country	Unit	Metric	Year	Mean	Upper	Lower
Afghanistan	Baharak	Proportion	2000	43.8	76.9	15.8
Afghanistan	Baharak	Proportion	2017	70.9	88.9	42.3
Afghanistan	Baharak	Count	2000	0	1	0
Afghanistan	Baharak	Count	2017	1	1	0
Afghanistan	Ab Kamari	Proportion	2000	43.4	77.2	15.8
Afghanistan	Ab Kamari	Proportion	2017	61.5	84.3	33.2
Afghanistan	Ab Kamari	Count	2000	0	1	0
Afghanistan	Ab Kamari	Count	2017	1	1	0
Afghanistan	Andarab	Proportion	2000	25.2	60.6	5.9
Afghanistan	Andarab	Proportion	2017	38.1	67.9	17.1
Afghanistan	Andarab	Count	2000	0	1	0
Afghanistan	Andarab	Count	2017	0	1	0
Afghanistan	Balkh	Proportion	2000	19.2	46.8	4.9
Afghanistan	Balkh	Proportion	2017	30.8	57.8	10.0
Afghanistan	Balkh	Count	2000	0	0	0
Afghanistan	Balkh	Count	2017	0	1	0
Afghanistan	Bamyan City	Proportion	2000	34.0	68.8	7.4
Afghanistan	Bamyan City	Proportion	2017	35.3	62.1	13.4
Afghanistan	Bamyan City	Count	2000	0	1	0
Afghanistan	Bamyan City	Count	2017	0	1	0
Afghanistan	Day Kundi	Proportion	2000	23.6	51.7	6.8
Afghanistan	Day Kundi	Proportion	2017	27.3	51.7	10.2
Afghanistan	Day Kundi	Count	2000	0	1	0
Afghanistan	Day Kundi	Count	2017	0	1	0
Afghanistan	Anar Dara	Proportion	2000	44.1	71.8	18.3
Afghanistan	Anar Dara	Proportion	2017	43.2	71.6	20.7
Afghanistan	Anar Dara	Count	2000	0	1	0
Afghanistan	Anar Dara	Count	2017	0	1	0
Afghanistan	Almar	Proportion	2000	28.7	66.4	7.6
Afghanistan	Almar	Proportion	2017	38.5	70.5	12.9
Afghanistan	Almar	Count	2000	0	1	0
Afghanistan	Almar	Count	2017	0	1	0
Afghanistan	Ab Band	Proportion	2000	35.2	69.3	12.3
Afghanistan	Ab Band	Proportion	2017	41.2	69.3	17.4
Afghanistan	Ab Band	Count	2000	0	1	0
Afghanistan	Ab Band	Count	2017	0	1	0
Afghanistan	Chaghcharan	Proportion	2000	27.1	51.6	8.4
Afghanistan	Chaghcharan	Proportion	2017	40.1	63.6	20.7
Afghanistan	Chaghcharan	Count	2000	0	1	0
Afghanistan	Chaghcharan	Count	2017	0	1	0
Afghanistan	Bughran	Proportion	2000	26.6	59.4	7.3
Afghanistan	Bughran	Proportion	2017	30.8	55.2	10.0
Afghanistan	Bughran	Count	2000	0	1	0
Afghanistan	Bughran	Count	2017	0	1	0
Afghanistan	Adraskan	Proportion	2000	50.4	79.4	19.8
Afghanistan	Adraskan	Proportion	2017	56.4	79.5	33.1
Afghanistan	Adraskan	Count	2000	1	1	0
Afghanistan	Adraskan	Count	2017	1	1	0
Afghanistan	Aqcha	Proportion	2000	19.6	45.3	5.9

Table 4: ORS by Unit *(continued)*

Country	Unit	Metric	Year	Mean	Upper	Lower
Afghanistan	Aqcha	Proportion	2017	45.8	72.0	20.7
Afghanistan	Aqcha	Count	2000	0	0	0
Afghanistan	Aqcha	Count	2017	0	1	0
Afghanistan	Bagrami	Proportion	2000	45.3	79.1	11.6
Afghanistan	Bagrami	Proportion	2017	51.5	81.5	18.7
Afghanistan	Bagrami	Count	2000	0	1	0
Afghanistan	Bagrami	Count	2017	1	1	0
Afghanistan	Arghandab	Proportion	2000	29.2	63.6	7.9
Afghanistan	Arghandab	Proportion	2017	35.4	63.5	12.8
Afghanistan	Arghandab	Count	2000	0	1	0
Afghanistan	Arghandab	Count	2017	0	1	0
Afghanistan	Alasay	Proportion	2000	38.4	72.6	11.2
Afghanistan	Alasay	Proportion	2017	47.0	75.2	19.4
Afghanistan	Alasay	Count	2000	0	1	0
Afghanistan	Alasay	Count	2017	0	1	0
Afghanistan	Bak	Proportion	2000	48.5	79.8	17.8
Afghanistan	Bak	Proportion	2017	70.0	87.6	39.5
Afghanistan	Bak	Count	2000	0	1	0
Afghanistan	Bak	Count	2017	1	1	0
Afghanistan	Asad abad	Proportion	2000	68.3	91.3	36.3
Afghanistan	Asad abad	Proportion	2017	35.8	63.4	15.3
Afghanistan	Asad abad	Count	2000	1	1	0
Afghanistan	Asad abad	Count	2017	0	1	0
Afghanistan	Ali abad	Proportion	2000	36.2	68.8	11.2
Afghanistan	Ali abad	Proportion	2017	50.4	75.4	22.8
Afghanistan	Ali abad	Count	2000	0	1	0
Afghanistan	Ali abad	Count	2017	1	1	0
Afghanistan	Alingar	Proportion	2000	61.7	88.9	27.7
Afghanistan	Alingar	Proportion	2017	59.0	85.1	30.7
Afghanistan	Alingar	Count	2000	1	1	0
Afghanistan	Alingar	Count	2017	1	1	0
Afghanistan	Baraki Barak	Proportion	2000	39.9	72.6	11.7
Afghanistan	Baraki Barak	Proportion	2017	65.4	88.6	35.9
Afghanistan	Baraki Barak	Count	2000	0	1	0
Afghanistan	Baraki Barak	Count	2017	1	1	0
Afghanistan	Achin	Proportion	2000	69.0	92.4	35.7
Afghanistan	Achin	Proportion	2017	57.2	82.6	28.0
Afghanistan	Achin	Count	2000	1	1	0
Afghanistan	Achin	Count	2017	1	1	0
Afghanistan	Chakhansur	Proportion	2000	33.2	65.0	10.5
Afghanistan	Chakhansur	Proportion	2017	24.7	45.8	8.4
Afghanistan	Chakhansur	Count	2000	0	1	0
Afghanistan	Chakhansur	Count	2017	0	0	0
Afghanistan	Bargi Matal	Proportion	2000	45.9	82.1	16.9
Afghanistan	Bargi Matal	Proportion	2017	38.1	65.1	15.3
Afghanistan	Bargi Matal	Count	2000	0	1	0
Afghanistan	Bargi Matal	Count	2017	0	1	0
Afghanistan	Burmul	Proportion	2000	42.7	75.9	16.1
Afghanistan	Burmul	Proportion	2017	86.3	96.7	67.0

Table 4: ORS by Unit *(continued)*

Country	Unit	Metric	Year	Mean	Upper	Lower
Afghanistan	Burmul	Count	2000	0	1	0
Afghanistan	Burmul	Count	2017	1	1	1
Afghanistan	Azro	Proportion	2000	51.3	80.1	17.2
Afghanistan	Azro	Proportion	2017	56.8	84.5	23.3
Afghanistan	Azro	Count	2000	1	1	0
Afghanistan	Azro	Count	2017	1	1	0
Afghanistan	Hisa-I-Awali Panjsher	Proportion	2000	25.6	57.6	6.8
Afghanistan	Hisa-I-Awali Panjsher	Proportion	2017	33.5	59.1	13.4
Afghanistan	Hisa-I-Awali Panjsher	Count	2000	0	1	0
Afghanistan	Hisa-I-Awali Panjsher	Count	2017	0	1	0
Afghanistan	Bagram	Proportion	2000	39.7	74.6	10.1
Afghanistan	Bagram	Proportion	2017	48.9	76.7	20.7
Afghanistan	Bagram	Count	2000	0	1	0
Afghanistan	Bagram	Count	2017	0	1	0
Afghanistan	Aybak	Proportion	2000	19.7	45.5	4.6
Afghanistan	Aybak	Proportion	2017	34.0	63.4	12.9
Afghanistan	Aybak	Count	2000	0	0	0
Afghanistan	Aybak	Count	2017	0	1	0
Afghanistan	Balkhab	Proportion	2000	27.3	54.4	6.9
Afghanistan	Balkhab	Proportion	2017	33.6	60.8	13.1
Afghanistan	Balkhab	Count	2000	0	1	0
Afghanistan	Balkhab	Count	2017	0	1	0
Afghanistan	Bangi	Proportion	2000	44.5	75.2	15.8
Afghanistan	Bangi	Proportion	2017	46.1	69.1	21.6
Afghanistan	Bangi	Count	2000	0	1	0
Afghanistan	Bangi	Count	2017	0	1	0
Afghanistan	Chora	Proportion	2000	23.3	55.4	6.1
Afghanistan	Chora	Proportion	2017	57.5	80.3	29.9
Afghanistan	Chora	Count	2000	0	1	0
Afghanistan	Chora	Count	2017	1	1	0
Afghanistan	Chak	Proportion	2000	39.7	72.6	13.4
Afghanistan	Chak	Proportion	2017	57.4	81.4	29.8
Afghanistan	Chak	Count	2000	0	1	0
Afghanistan	Chak	Count	2017	1	1	0
Afghanistan	Arghandab	Proportion	2000	38.2	71.2	11.7
Afghanistan	Arghandab	Proportion	2017	50.4	75.1	24.6
Afghanistan	Arghandab	Count	2000	0	1	0
Afghanistan	Arghandab	Count	2017	1	1	0
Afghanistan	Darwaz	Proportion	2000	44.2	72.7	14.5
Afghanistan	Darwaz	Proportion	2017	58.4	87.5	27.2
Afghanistan	Darwaz	Count	2000	0	1	0
Afghanistan	Darwaz	Count	2017	1	1	0
Afghanistan	Ghormach	Proportion	2000	38.9	72.9	11.5
Afghanistan	Ghormach	Proportion	2017	51.9	79.2	23.3
Afghanistan	Ghormach	Count	2000	0	1	0
Afghanistan	Ghormach	Count	2017	1	1	0
Afghanistan	Baghlan City	Proportion	2000	22.3	46.8	5.1
Afghanistan	Baghlan City	Proportion	2017	37.8	68.0	14.6
Afghanistan	Baghlan City	Count	2000	0	0	0

Table 4: ORS by Unit *(continued)*

Country	Unit	Metric	Year	Mean	Upper	Lower
Afghanistan	Baghlan City	Count	2017	0	1	0
Afghanistan	Char Bolak	Proportion	2000	18.1	44.0	4.8
Afghanistan	Char Bolak	Proportion	2017	33.8	61.5	12.2
Afghanistan	Char Bolak	Count	2000	0	0	0
Afghanistan	Char Bolak	Count	2017	0	1	0
Afghanistan	Panjab	Proportion	2000	34.0	69.8	9.7
Afghanistan	Panjab	Proportion	2017	30.6	56.4	12.3
Afghanistan	Panjab	Count	2000	0	1	0
Afghanistan	Panjab	Count	2017	0	1	0
Afghanistan	Gizab	Proportion	2000	23.9	54.7	6.9
Afghanistan	Gizab	Proportion	2017	36.7	63.2	15.6
Afghanistan	Gizab	Count	2000	0	1	0
Afghanistan	Gizab	Count	2017	0	1	0
Afghanistan	Bakwa	Proportion	2000	40.6	74.2	14.5
Afghanistan	Bakwa	Proportion	2017	25.2	48.3	8.4
Afghanistan	Bakwa	Count	2000	0	1	0
Afghanistan	Bakwa	Count	2017	0	0	0
Afghanistan	Andkhoy	Proportion	2000	31.0	66.5	8.2
Afghanistan	Andkhoy	Proportion	2017	32.6	64.3	12.6
Afghanistan	Andkhoy	Count	2000	0	1	0
Afghanistan	Andkhoy	Count	2017	0	1	0
Afghanistan	Ajristan	Proportion	2000	33.7	70.2	9.2
Afghanistan	Ajristan	Proportion	2017	37.1	66.3	14.8
Afghanistan	Ajristan	Count	2000	0	1	0
Afghanistan	Ajristan	Count	2017	0	1	0
Afghanistan	Lal Wa Sarjangal	Proportion	2000	30.7	62.2	9.5
Afghanistan	Lal Wa Sarjangal	Proportion	2017	43.6	69.6	19.2
Afghanistan	Lal Wa Sarjangal	Count	2000	0	1	0
Afghanistan	Lal Wa Sarjangal	Count	2017	0	1	0
Afghanistan	Disho	Proportion	2000	29.5	58.8	8.6
Afghanistan	Disho	Proportion	2017	38.5	66.0	13.5
Afghanistan	Disho	Count	2000	0	1	0
Afghanistan	Disho	Count	2017	0	1	0
Afghanistan	Chishti Sharif	Proportion	2000	43.9	73.7	16.9
Afghanistan	Chishti Sharif	Proportion	2017	52.8	76.0	24.6
Afghanistan	Chishti Sharif	Count	2000	0	1	0
Afghanistan	Chishti Sharif	Count	2017	1	1	0
Afghanistan	Darzab	Proportion	2000	25.5	55.9	6.3
Afghanistan	Darzab	Proportion	2017	39.0	67.0	14.8
Afghanistan	Darzab	Count	2000	0	1	0
Afghanistan	Darzab	Count	2017	0	1	0
Afghanistan	Chahar Asyab	Proportion	2000	44.5	79.6	10.4
Afghanistan	Chahar Asyab	Proportion	2017	55.3	83.2	24.9
Afghanistan	Chahar Asyab	Count	2000	0	1	0
Afghanistan	Chahar Asyab	Count	2017	1	1	0
Afghanistan	Arghistan	Proportion	2000	30.2	65.0	6.9
Afghanistan	Arghistan	Proportion	2017	23.4	48.4	8.6
Afghanistan	Arghistan	Count	2000	0	1	0
Afghanistan	Arghistan	Count	2017	0	0	0

Table 4: ORS by Unit *(continued)*

Country	Unit	Metric	Year	Mean	Upper	Lower
Afghanistan	Kohband	Proportion	2000	39.5	77.6	9.8
Afghanistan	Kohband	Proportion	2017	45.4	75.5	17.6
Afghanistan	Kohband	Count	2000	0	1	0
Afghanistan	Kohband	Count	2017	0	1	0
Afghanistan	Gurbuz	Proportion	2000	38.8	74.8	12.4
Afghanistan	Gurbuz	Proportion	2017	75.0	91.3	48.4
Afghanistan	Gurbuz	Count	2000	0	1	0
Afghanistan	Gurbuz	Count	2017	1	1	0
Afghanistan	Bar Kunar	Proportion	2000	66.7	91.6	33.1
Afghanistan	Bar Kunar	Proportion	2017	32.1	61.1	12.7
Afghanistan	Bar Kunar	Count	2000	1	1	0
Afghanistan	Bar Kunar	Count	2017	0	1	0
Afghanistan	Char Dara	Proportion	2000	36.1	70.2	9.6
Afghanistan	Char Dara	Proportion	2017	51.7	77.6	25.2
Afghanistan	Char Dara	Count	2000	0	1	0
Afghanistan	Char Dara	Count	2017	1	1	0
Afghanistan	Alishing	Proportion	2000	60.5	88.1	27.0
Afghanistan	Alishing	Proportion	2017	65.0	88.6	35.7
Afghanistan	Alishing	Count	2000	1	1	0
Afghanistan	Alishing	Count	2017	1	1	0
Afghanistan	Charkh	Proportion	2000	38.7	71.8	13.3
Afghanistan	Charkh	Proportion	2017	63.6	87.4	38.4
Afghanistan	Charkh	Count	2000	0	1	0
Afghanistan	Charkh	Count	2017	1	1	0
Afghanistan	Bati Kot	Proportion	2000	72.3	93.7	40.3
Afghanistan	Bati Kot	Proportion	2017	59.6	83.5	31.5
Afghanistan	Bati Kot	Count	2000	1	1	0
Afghanistan	Bati Kot	Count	2017	1	1	0
Afghanistan	Char Burjak	Proportion	2000	31.3	59.0	11.0
Afghanistan	Char Burjak	Proportion	2017	33.1	55.3	13.0
Afghanistan	Char Burjak	Count	2000	0	1	0
Afghanistan	Char Burjak	Count	2017	0	1	0
Afghanistan	Kamdesh	Proportion	2000	51.0	84.4	17.8
Afghanistan	Kamdesh	Proportion	2017	35.4	65.1	13.5
Afghanistan	Kamdesh	Count	2000	1	1	0
Afghanistan	Kamdesh	Count	2017	0	1	0
Afghanistan	Dila	Proportion	2000	48.3	80.4	22.1
Afghanistan	Dila	Proportion	2017	72.3	90.0	47.7
Afghanistan	Dila	Count	2000	0	1	0
Afghanistan	Dila	Count	2017	1	1	0
Afghanistan	Chamkani	Proportion	2000	64.3	88.1	28.9
Afghanistan	Chamkani	Proportion	2017	65.3	87.2	32.3
Afghanistan	Chamkani	Count	2000	1	1	0
Afghanistan	Chamkani	Count	2017	1	1	0
Afghanistan	Hisa-I-Duwum Panjsher	Proportion	2000	28.4	60.5	7.5
Afghanistan	Hisa-I-Duwum Panjsher	Proportion	2017	39.0	65.6	15.7
Afghanistan	Hisa-I-Duwum Panjsher	Count	2000	0	1	0
Afghanistan	Hisa-I-Duwum Panjsher	Count	2017	0	1	0
Afghanistan	Chaharikar	Proportion	2000	38.4	74.3	9.6

Table 4: ORS by Unit *(continued)*

Country	Unit	Metric	Year	Mean	Upper	Lower
Afghanistan	Chaharikar	Proportion	2017	49.4	78.1	21.7
Afghanistan	Chaharikar	Count	2000	0	1	0
Afghanistan	Chaharikar	Count	2017	0	1	0
Afghanistan	Dara-i-Suf	Proportion	2000	19.8	45.7	5.1
Afghanistan	Dara-i-Suf	Proportion	2017	30.8	54.6	11.4
Afghanistan	Dara-i-Suf	Count	2000	0	0	0
Afghanistan	Dara-i-Suf	Count	2017	0	1	0
Afghanistan	Kohistanat	Proportion	2000	26.3	48.2	8.8
Afghanistan	Kohistanat	Proportion	2017	36.0	61.0	14.7
Afghanistan	Kohistanat	Count	2000	0	0	0
Afghanistan	Kohistanat	Count	2017	0	1	0
Afghanistan	Chah Ab	Proportion	2000	43.1	77.3	12.6
Afghanistan	Chah Ab	Proportion	2017	50.8	75.6	27.1
Afghanistan	Chah Ab	Count	2000	0	1	0
Afghanistan	Chah Ab	Count	2017	1	1	0
Afghanistan	Dihrawud	Proportion	2000	21.2	53.5	5.3
Afghanistan	Dihrawud	Proportion	2017	55.0	82.3	27.3
Afghanistan	Dihrawud	Count	2000	0	1	0
Afghanistan	Dihrawud	Count	2017	1	1	0
Afghanistan	Daymirdad	Proportion	2000	40.5	74.1	12.6
Afghanistan	Daymirdad	Proportion	2017	51.5	76.9	22.7
Afghanistan	Daymirdad	Count	2000	0	1	0
Afghanistan	Daymirdad	Count	2017	1	1	0
Afghanistan	Atghar	Proportion	2000	33.4	67.2	10.4
Afghanistan	Atghar	Proportion	2017	39.2	71.9	13.6
Afghanistan	Atghar	Count	2000	0	1	0
Afghanistan	Atghar	Count	2017	0	1	0
Afghanistan	Fayzabad	Proportion	2000	42.6	78.9	11.4
Afghanistan	Fayzabad	Proportion	2017	66.4	85.5	40.6
Afghanistan	Fayzabad	Count	2000	0	1	0
Afghanistan	Fayzabad	Count	2017	1	1	0
Afghanistan	Jawand	Proportion	2000	41.6	72.6	16.2
Afghanistan	Jawand	Proportion	2017	55.6	79.1	31.0
Afghanistan	Jawand	Count	2000	0	1	0
Afghanistan	Jawand	Count	2017	1	1	0
Afghanistan	Baghlani Jadid	Proportion	2000	27.6	54.8	7.5
Afghanistan	Baghlani Jadid	Proportion	2017	44.2	75.1	19.7
Afghanistan	Baghlani Jadid	Count	2000	0	1	0
Afghanistan	Baghlani Jadid	Count	2017	0	1	0
Afghanistan	Charkint	Proportion	2000	22.6	48.3	5.7
Afghanistan	Charkint	Proportion	2017	30.1	57.2	11.0
Afghanistan	Charkint	Count	2000	0	0	0
Afghanistan	Charkint	Count	2017	0	1	0
Afghanistan	Shibar	Proportion	2000	33.8	68.0	9.8
Afghanistan	Shibar	Proportion	2017	39.8	68.9	14.3
Afghanistan	Shibar	Count	2000	0	1	0
Afghanistan	Shibar	Count	2017	0	1	0
Afghanistan	Kijran	Proportion	2000	20.9	48.7	4.3
Afghanistan	Kijran	Proportion	2017	23.0	45.7	8.4

Table 4: ORS by Unit *(continued)*

Country	Unit	Metric	Year	Mean	Upper	Lower
Afghanistan	Kijran	Count	2000	0	0	0
Afghanistan	Kijran	Count	2017	0	0	0
Afghanistan	Bala Buluk	Proportion	2000	42.3	72.3	16.6
Afghanistan	Bala Buluk	Proportion	2017	31.1	57.1	11.3
Afghanistan	Bala Buluk	Count	2000	0	1	0
Afghanistan	Bala Buluk	Count	2017	0	1	0
Afghanistan	Bilchiragh	Proportion	2000	26.5	59.2	7.3
Afghanistan	Bilchiragh	Proportion	2017	39.2	66.0	15.2
Afghanistan	Bilchiragh	Count	2000	0	1	0
Afghanistan	Bilchiragh	Count	2017	0	1	0
Afghanistan	Andar	Proportion	2000	33.1	64.3	10.4
Afghanistan	Andar	Proportion	2017	45.7	71.5	19.4
Afghanistan	Andar	Count	2000	0	1	0
Afghanistan	Andar	Count	2017	0	1	0
Afghanistan	Pasaband	Proportion	2000	27.0	58.6	7.0
Afghanistan	Pasaband	Proportion	2017	30.5	54.2	13.2
Afghanistan	Pasaband	Count	2000	0	1	0
Afghanistan	Pasaband	Count	2017	0	1	0
Afghanistan	Garmser	Proportion	2000	28.2	57.1	9.2
Afghanistan	Garmser	Proportion	2017	45.2	69.1	20.2
Afghanistan	Garmser	Count	2000	0	1	0
Afghanistan	Garmser	Count	2017	0	1	0
Afghanistan	Farsi	Proportion	2000	42.9	75.2	13.4
Afghanistan	Farsi	Proportion	2017	48.7	76.7	24.3
Afghanistan	Farsi	Count	2000	0	1	0
Afghanistan	Farsi	Count	2017	0	1	0
Afghanistan	Fayz abad	Proportion	2000	20.9	48.3	6.6
Afghanistan	Fayz abad	Proportion	2017	43.5	68.1	20.3
Afghanistan	Fayz abad	Count	2000	0	0	0
Afghanistan	Fayz abad	Count	2017	0	1	0
Afghanistan	Dih Sabz	Proportion	2000	42.0	75.4	10.2
Afghanistan	Dih Sabz	Proportion	2017	49.2	80.2	19.4
Afghanistan	Dih Sabz	Count	2000	0	1	0
Afghanistan	Dih Sabz	Count	2017	0	1	0
Afghanistan	Daman	Proportion	2000	28.9	65.0	7.3
Afghanistan	Daman	Proportion	2017	26.9	55.8	8.8
Afghanistan	Daman	Count	2000	0	1	0
Afghanistan	Daman	Count	2017	0	1	0
Afghanistan	Kohistan	Proportion	2000	37.5	73.7	10.0
Afghanistan	Kohistan	Proportion	2017	45.7	77.1	19.2
Afghanistan	Kohistan	Count	2000	0	1	0
Afghanistan	Kohistan	Count	2017	0	1	0
Afghanistan	Jaji Maydan	Proportion	2000	44.8	75.2	14.6
Afghanistan	Jaji Maydan	Proportion	2017	64.4	84.5	35.1
Afghanistan	Jaji Maydan	Count	2000	0	1	0
Afghanistan	Jaji Maydan	Count	2017	1	1	0
Afghanistan	Chapa Dara	Proportion	2000	57.5	86.9	23.6
Afghanistan	Chapa Dara	Proportion	2017	39.0	64.6	17.0
Afghanistan	Chapa Dara	Count	2000	1	1	0

Table 4: ORS by Unit *(continued)*

Country	Unit	Metric	Year	Mean	Upper	Lower
Afghanistan	Chapa Dara	Count	2017	0	1	0
Afghanistan	Dashti Archi	Proportion	2000	40.1	75.6	11.5
Afghanistan	Dashti Archi	Proportion	2017	50.5	76.6	23.1
Afghanistan	Dashti Archi	Count	2000	0	1	0
Afghanistan	Dashti Archi	Count	2017	1	1	0
Afghanistan	Dawlat Shah	Proportion	2000	53.0	82.4	21.4
Afghanistan	Dawlat Shah	Proportion	2017	56.0	80.2	27.8
Afghanistan	Dawlat Shah	Count	2000	1	1	0
Afghanistan	Dawlat Shah	Count	2017	1	1	0
Afghanistan	Khushi	Proportion	2000	42.8	74.8	12.8
Afghanistan	Khushi	Proportion	2017	59.4	86.3	27.6
Afghanistan	Khushi	Count	2000	0	1	0
Afghanistan	Khushi	Count	2017	1	1	0
Afghanistan	Chaparhar	Proportion	2000	66.6	89.8	33.6
Afghanistan	Chaparhar	Proportion	2017	58.8	85.4	34.1
Afghanistan	Chaparhar	Count	2000	1	1	0
Afghanistan	Chaparhar	Count	2017	1	1	0
Afghanistan	Kang	Proportion	2000	32.3	67.2	8.8
Afghanistan	Kang	Proportion	2017	22.3	46.7	6.6
Afghanistan	Kang	Count	2000	0	1	0
Afghanistan	Kang	Count	2017	0	0	0
Afghanistan	Mandol	Proportion	2000	44.6	77.1	15.8
Afghanistan	Mandol	Proportion	2017	36.4	61.9	15.1
Afghanistan	Mandol	Count	2000	0	1	0
Afghanistan	Mandol	Count	2017	0	1	0
Afghanistan	Gayan	Proportion	2000	39.0	73.4	10.6
Afghanistan	Gayan	Proportion	2017	83.5	96.7	61.4
Afghanistan	Gayan	Count	2000	0	1	0
Afghanistan	Gayan	Count	2017	1	1	1
Afghanistan	Dand Wa Patan	Proportion	2000	56.5	83.6	23.3
Afghanistan	Dand Wa Patan	Proportion	2017	63.3	85.0	34.6
Afghanistan	Dand Wa Patan	Count	2000	1	1	0
Afghanistan	Dand Wa Patan	Count	2017	1	1	0
Afghanistan	Panjsher	Proportion	2000	31.2	65.9	7.7
Afghanistan	Panjsher	Proportion	2017	40.3	67.1	15.7
Afghanistan	Panjsher	Count	2000	0	1	0
Afghanistan	Panjsher	Count	2017	0	1	0
Afghanistan	Ghorband	Proportion	2000	36.3	71.7	9.1
Afghanistan	Ghorband	Proportion	2017	49.2	78.5	19.3
Afghanistan	Ghorband	Count	2000	0	1	0
Afghanistan	Ghorband	Count	2017	0	1	0
Afghanistan	Hazrati Sultan	Proportion	2000	21.7	51.0	5.0
Afghanistan	Hazrati Sultan	Proportion	2017	33.2	62.6	12.5
Afghanistan	Hazrati Sultan	Count	2000	0	1	0
Afghanistan	Hazrati Sultan	Count	2017	0	1	0
Afghanistan	Sangcharak	Proportion	2000	24.2	53.6	5.8
Afghanistan	Sangcharak	Proportion	2017	33.3	61.4	15.1
Afghanistan	Sangcharak	Count	2000	0	1	0
Afghanistan	Sangcharak	Count	2017	0	1	0

Table 4: ORS by Unit *(continued)*

Country	Unit	Metric	Year	Mean	Upper	Lower
Afghanistan	Chal	Proportion	2000	43.2	79.0	16.1
Afghanistan	Chal	Proportion	2017	37.4	63.1	17.2
Afghanistan	Chal	Count	2000	0	1	0
Afghanistan	Chal	Count	2017	0	1	0
Afghanistan	Khas Uruzgan	Proportion	2000	27.8	61.9	6.8
Afghanistan	Khas Uruzgan	Proportion	2017	60.7	84.1	34.6
Afghanistan	Khas Uruzgan	Count	2000	0	1	0
Afghanistan	Khas Uruzgan	Count	2017	1	1	0
Afghanistan	Hisa-i-Awali Bihsud	Proportion	2000	41.0	77.7	13.5
Afghanistan	Hisa-i-Awali Bihsud	Proportion	2017	47.8	74.4	19.4
Afghanistan	Hisa-i-Awali Bihsud	Count	2000	0	1	0
Afghanistan	Hisa-i-Awali Bihsud	Count	2017	0	1	0
Afghanistan	Daychopan	Proportion	2000	34.0	67.8	11.1
Afghanistan	Daychopan	Proportion	2017	56.4	79.5	29.9
Afghanistan	Daychopan	Count	2000	0	1	0
Afghanistan	Daychopan	Count	2017	1	1	0
Afghanistan	Ishkashim	Proportion	2000	42.6	72.9	13.3
Afghanistan	Ishkashim	Proportion	2017	62.6	87.8	30.7
Afghanistan	Ishkashim	Count	2000	0	1	0
Afghanistan	Ishkashim	Count	2017	1	1	0
Afghanistan	Muqur	Proportion	2000	41.0	74.5	12.9
Afghanistan	Muqur	Proportion	2017	62.9	86.4	33.7
Afghanistan	Muqur	Count	2000	0	1	0
Afghanistan	Muqur	Count	2017	1	1	0
Afghanistan	Burka	Proportion	2000	30.3	64.6	8.3
Afghanistan	Burka	Proportion	2017	40.3	68.2	15.3
Afghanistan	Burka	Count	2000	0	1	0
Afghanistan	Burka	Count	2017	0	1	0
Afghanistan	Chimtal	Proportion	2000	21.7	48.4	6.0
Afghanistan	Chimtal	Proportion	2017	34.6	61.5	13.9
Afghanistan	Chimtal	Count	2000	0	0	0
Afghanistan	Chimtal	Count	2017	0	1	0
Afghanistan	Waras	Proportion	2000	33.0	67.9	9.3
Afghanistan	Waras	Proportion	2017	29.0	56.7	12.0
Afghanistan	Waras	Count	2000	0	1	0
Afghanistan	Waras	Count	2017	0	1	0
Afghanistan	Shahristan	Proportion	2000	29.9	69.1	9.0
Afghanistan	Shahristan	Proportion	2017	32.3	60.3	12.4
Afghanistan	Shahristan	Count	2000	0	1	0
Afghanistan	Shahristan	Count	2017	0	1	0
Afghanistan	Farah City	Proportion	2000	42.3	74.7	11.5
Afghanistan	Farah City	Proportion	2017	29.4	58.7	12.3
Afghanistan	Farah City	Count	2000	0	1	0
Afghanistan	Farah City	Count	2017	0	1	0
Afghanistan	Dawlat abad	Proportion	2000	30.1	61.0	7.1
Afghanistan	Dawlat abad	Proportion	2017	35.5	61.0	14.9
Afghanistan	Dawlat abad	Count	2000	0	1	0
Afghanistan	Dawlat abad	Count	2017	0	1	0
Afghanistan	Dihyak	Proportion	2000	34.0	68.2	10.2

Table 4: ORS by Unit *(continued)*

Country	Unit	Metric	Year	Mean	Upper	Lower
Afghanistan	Dihyak	Proportion	2017	53.2	79.7	26.0
Afghanistan	Dihyak	Count	2000	0	1	0
Afghanistan	Dihyak	Count	2017	1	1	0
Afghanistan	Saghar	Proportion	2000	33.2	61.0	11.8
Afghanistan	Saghar	Proportion	2017	38.5	64.3	16.6
Afghanistan	Saghar	Count	2000	0	1	0
Afghanistan	Saghar	Count	2017	0	1	0
Afghanistan	Kajaki	Proportion	2000	27.3	58.0	7.7
Afghanistan	Kajaki	Proportion	2017	48.8	76.3	18.7
Afghanistan	Kajaki	Count	2000	0	1	0
Afghanistan	Kajaki	Count	2017	0	1	0
Afghanistan	Ghoryan	Proportion	2000	50.1	83.6	18.6
Afghanistan	Ghoryan	Proportion	2017	55.1	76.5	31.1
Afghanistan	Ghoryan	Count	2000	1	1	0
Afghanistan	Ghoryan	Count	2017	1	1	0
Afghanistan	Khamyab	Proportion	2000	23.7	54.0	6.3
Afghanistan	Khamyab	Proportion	2017	48.6	77.7	23.9
Afghanistan	Khamyab	Count	2000	0	1	0
Afghanistan	Khamyab	Count	2017	0	1	0
Afghanistan	Gul dara	Proportion	2000	41.1	76.3	11.3
Afghanistan	Gul dara	Proportion	2017	50.8	83.0	20.4
Afghanistan	Gul dara	Count	2000	0	1	0
Afghanistan	Gul dara	Count	2017	1	1	0
Afghanistan	Ghorak	Proportion	2000	29.5	65.9	8.1
Afghanistan	Ghorak	Proportion	2017	50.5	76.3	23.5
Afghanistan	Ghorak	Count	2000	0	1	0
Afghanistan	Ghorak	Count	2017	1	1	0
Afghanistan	Mahmud Raqi	Proportion	2000	39.1	74.6	10.1
Afghanistan	Mahmud Raqi	Proportion	2017	47.0	75.4	20.0
Afghanistan	Mahmud Raqi	Count	2000	0	1	0
Afghanistan	Mahmud Raqi	Count	2017	0	1	0
Afghanistan	Khost (Matun)	Proportion	2000	46.5	80.8	17.7
Afghanistan	Khost (Matun)	Proportion	2017	74.7	90.8	46.7
Afghanistan	Khost (Matun)	Count	2000	0	1	0
Afghanistan	Khost (Matun)	Count	2017	1	1	0
Afghanistan	Chawkay	Proportion	2000	72.1	93.2	39.2
Afghanistan	Chawkay	Proportion	2017	47.6	74.8	23.7
Afghanistan	Chawkay	Count	2000	1	1	0
Afghanistan	Chawkay	Count	2017	0	1	0
Afghanistan	Hazrati Imam	Proportion	2000	40.8	73.0	11.0
Afghanistan	Hazrati Imam	Proportion	2017	52.5	77.2	26.5
Afghanistan	Hazrati Imam	Count	2000	0	1	0
Afghanistan	Hazrati Imam	Count	2017	1	1	0
Afghanistan	Mihtarlam	Proportion	2000	66.3	90.4	31.1
Afghanistan	Mihtarlam	Proportion	2017	66.5	88.8	41.9
Afghanistan	Mihtarlam	Count	2000	1	1	0
Afghanistan	Mihtarlam	Count	2017	1	1	0
Afghanistan	Muhammad Agha	Proportion	2000	39.4	72.4	10.6
Afghanistan	Muhammad Agha	Proportion	2017	60.6	86.6	27.8

Table 4: ORS by Unit *(continued)*

Country	Unit	Metric	Year	Mean	Upper	Lower
Afghanistan	Muhammad Agha	Count	2000	0	1	0
Afghanistan	Muhammad Agha	Count	2017	1	1	0
Afghanistan	Dara-I-Nur	Proportion	2000	69.1	91.8	34.0
Afghanistan	Dara-I-Nur	Proportion	2017	52.5	79.4	27.8
Afghanistan	Dara-I-Nur	Count	2000	1	1	0
Afghanistan	Dara-I-Nur	Count	2017	1	1	0
Afghanistan	Khash Rod	Proportion	2000	36.8	67.8	12.3
Afghanistan	Khash Rod	Proportion	2017	30.2	54.8	11.4
Afghanistan	Khash Rod	Count	2000	0	1	0
Afghanistan	Khash Rod	Count	2017	0	1	0
Afghanistan	Nuristan	Proportion	2000	53.0	84.1	20.3
Afghanistan	Nuristan	Proportion	2017	43.6	69.5	19.2
Afghanistan	Nuristan	Count	2000	1	1	0
Afghanistan	Nuristan	Count	2017	0	1	0
Afghanistan	Gomal	Proportion	2000	43.9	77.6	17.5
Afghanistan	Gomal	Proportion	2017	85.0	95.3	66.4
Afghanistan	Gomal	Count	2000	0	1	0
Afghanistan	Gomal	Count	2017	1	1	1
Afghanistan	Gardez	Proportion	2000	57.4	86.7	22.5
Afghanistan	Gardez	Proportion	2017	70.1	89.5	45.2
Afghanistan	Gardez	Count	2000	1	1	0
Afghanistan	Gardez	Count	2017	1	1	0
Afghanistan	Jabalussaraj	Proportion	2000	38.0	75.3	10.9
Afghanistan	Jabalussaraj	Proportion	2017	48.3	78.1	22.2
Afghanistan	Jabalussaraj	Count	2000	0	1	0
Afghanistan	Jabalussaraj	Count	2017	0	1	0
Afghanistan	Khuram Wa Sarbagh	Proportion	2000	18.2	41.8	4.7
Afghanistan	Khuram Wa Sarbagh	Proportion	2017	34.6	65.5	14.3
Afghanistan	Khuram Wa Sarbagh	Count	2000	0	0	0
Afghanistan	Khuram Wa Sarbagh	Count	2017	0	1	0
Afghanistan	Sar-i-Pul City	Proportion	2000	26.0	58.6	6.7
Afghanistan	Sar-i-Pul City	Proportion	2017	41.1	69.9	16.8
Afghanistan	Sar-i-Pul City	Count	2000	0	1	0
Afghanistan	Sar-i-Pul City	Count	2017	0	1	0
Afghanistan	Darqad	Proportion	2000	42.5	77.9	12.5
Afghanistan	Darqad	Proportion	2017	43.1	67.6	19.2
Afghanistan	Darqad	Count	2000	0	1	0
Afghanistan	Darqad	Count	2017	0	1	0
Afghanistan	Nesh	Proportion	2000	24.0	59.7	5.8
Afghanistan	Nesh	Proportion	2017	59.2	84.3	32.0
Afghanistan	Nesh	Count	2000	0	1	0
Afghanistan	Nesh	Count	2017	1	1	0
Afghanistan	Jalrez	Proportion	2000	41.4	77.6	13.3
Afghanistan	Jalrez	Proportion	2017	60.1	84.7	29.0
Afghanistan	Jalrez	Count	2000	0	1	0
Afghanistan	Jalrez	Count	2017	1	1	0
Afghanistan	Mizan	Proportion	2000	39.3	70.3	10.1
Afghanistan	Mizan	Proportion	2017	48.9	77.1	20.8
Afghanistan	Mizan	Count	2000	0	1	0

Table 4: ORS by Unit *(continued)*

Country	Unit	Metric	Year	Mean	Upper	Lower
Afghanistan	Mizan	Count	2017	0	1	0
Afghanistan	Jurm	Proportion	2000	42.3	78.2	14.9
Afghanistan	Jurm	Proportion	2017	67.5	87.2	42.4
Afghanistan	Jurm	Count	2000	0	1	0
Afghanistan	Jurm	Count	2017	1	1	0
Afghanistan	Murghab	Proportion	2000	39.5	75.6	12.6
Afghanistan	Murghab	Proportion	2017	58.6	83.0	30.4
Afghanistan	Murghab	Count	2000	0	1	0
Afghanistan	Murghab	Count	2017	1	1	0
Afghanistan	Dahana-I-Ghori	Proportion	2000	22.6	49.4	5.4
Afghanistan	Dahana-I-Ghori	Proportion	2017	37.6	68.6	15.2
Afghanistan	Dahana-I-Ghori	Count	2000	0	0	0
Afghanistan	Dahana-I-Ghori	Count	2017	0	1	0
Afghanistan	Dawlat abad	Proportion	2000	19.3	44.5	4.8
Afghanistan	Dawlat abad	Proportion	2017	31.0	55.1	11.7
Afghanistan	Dawlat abad	Count	2000	0	0	0
Afghanistan	Dawlat abad	Count	2017	0	1	0
Afghanistan	Yakawlang	Proportion	2000	31.3	61.3	9.0
Afghanistan	Yakawlang	Proportion	2017	31.8	56.1	13.7
Afghanistan	Yakawlang	Count	2000	0	1	0
Afghanistan	Yakawlang	Count	2017	0	1	0
Afghanistan	Gulistan	Proportion	2000	42.2	75.4	16.7
Afghanistan	Gulistan	Proportion	2017	31.2	54.1	11.8
Afghanistan	Gulistan	Count	2000	0	1	0
Afghanistan	Gulistan	Count	2017	0	1	0
Afghanistan	Khan Char Bagh	Proportion	2000	28.8	60.7	6.9
Afghanistan	Khan Char Bagh	Proportion	2017	36.9	65.6	16.4
Afghanistan	Khan Char Bagh	Count	2000	0	1	0
Afghanistan	Khan Char Bagh	Count	2017	0	1	0
Afghanistan	Gelan	Proportion	2000	35.9	69.6	10.5
Afghanistan	Gelan	Proportion	2017	44.1	75.3	18.2
Afghanistan	Gelan	Count	2000	0	1	0
Afghanistan	Gelan	Count	2017	0	1	0
Afghanistan	Shahrak	Proportion	2000	29.3	58.7	9.2
Afghanistan	Shahrak	Proportion	2017	40.2	67.1	16.4
Afghanistan	Shahrak	Count	2000	0	1	0
Afghanistan	Shahrak	Count	2017	0	1	0
Afghanistan	Lashkargah	Proportion	2000	27.2	61.1	6.9
Afghanistan	Lashkargah	Proportion	2017	49.7	76.4	22.3
Afghanistan	Lashkargah	Count	2000	0	1	0
Afghanistan	Lashkargah	Count	2017	0	1	0
Afghanistan	Gulran	Proportion	2000	50.4	79.5	22.4
Afghanistan	Gulran	Proportion	2017	51.2	73.9	29.2
Afghanistan	Gulran	Count	2000	1	1	0
Afghanistan	Gulran	Count	2017	1	1	0
Afghanistan	Khwaja Du Koh	Proportion	2000	24.8	56.1	6.3
Afghanistan	Khwaja Du Koh	Proportion	2017	45.0	73.8	21.3
Afghanistan	Khwaja Du Koh	Count	2000	0	1	0
Afghanistan	Khwaja Du Koh	Count	2017	0	1	0

Table 4: ORS by Unit *(continued)*

Country	Unit	Metric	Year	Mean	Upper	Lower
Afghanistan	Istalif	Proportion	2000	40.2	75.8	10.9
Afghanistan	Istalif	Proportion	2017	49.4	80.9	20.5
Afghanistan	Istalif	Count	2000	0	1	0
Afghanistan	Istalif	Count	2017	0	1	0
Afghanistan	Kandahar City	Proportion	2000	30.7	67.5	8.5
Afghanistan	Kandahar City	Proportion	2017	30.8	60.6	10.4
Afghanistan	Kandahar City	Count	2000	0	1	0
Afghanistan	Kandahar City	Count	2017	0	1	0
Afghanistan	Nijrab	Proportion	2000	41.3	75.9	12.7
Afghanistan	Nijrab	Proportion	2017	46.5	76.3	18.7
Afghanistan	Nijrab	Count	2000	0	1	0
Afghanistan	Nijrab	Count	2017	0	1	0
Afghanistan	Mando Zayi	Proportion	2000	46.5	81.4	16.1
Afghanistan	Mando Zayi	Proportion	2017	77.6	92.6	51.3
Afghanistan	Mando Zayi	Count	2000	0	1	0
Afghanistan	Mando Zayi	Count	2017	1	1	1
Afghanistan	Dangam	Proportion	2000	66.9	90.4	34.6
Afghanistan	Dangam	Proportion	2017	31.7	61.1	12.6
Afghanistan	Dangam	Count	2000	1	1	0
Afghanistan	Dangam	Count	2017	0	1	0
Afghanistan	Khan Abad	Proportion	2000	41.6	73.9	13.6
Afghanistan	Khan Abad	Proportion	2017	54.0	77.9	22.5
Afghanistan	Khan Abad	Count	2000	0	1	0
Afghanistan	Khan Abad	Count	2017	1	1	0
Afghanistan	Qarghayi	Proportion	2000	62.4	87.8	28.7
Afghanistan	Qarghayi	Proportion	2017	63.6	88.4	37.5
Afghanistan	Qarghayi	Count	2000	1	1	0
Afghanistan	Qarghayi	Count	2017	1	1	0
Afghanistan	Puli Alam	Proportion	2000	38.9	70.5	10.9
Afghanistan	Puli Alam	Proportion	2017	63.8	87.3	36.5
Afghanistan	Puli Alam	Count	2000	0	1	0
Afghanistan	Puli Alam	Count	2017	1	1	0
Afghanistan	Dih Bala	Proportion	2000	62.7	88.1	28.7
Afghanistan	Dih Bala	Proportion	2017	58.2	83.0	29.2
Afghanistan	Dih Bala	Count	2000	1	1	0
Afghanistan	Dih Bala	Count	2017	1	1	0
Afghanistan	Zaranj	Proportion	2000	34.4	66.8	8.3
Afghanistan	Zaranj	Proportion	2017	24.0	48.0	7.4
Afghanistan	Zaranj	Count	2000	0	1	0
Afghanistan	Zaranj	Count	2017	0	0	0
Afghanistan	Wama	Proportion	2000	50.3	81.5	17.4
Afghanistan	Wama	Proportion	2017	31.6	55.3	11.8
Afghanistan	Wama	Count	2000	1	1	0
Afghanistan	Wama	Count	2017	0	1	0
Afghanistan	Mata Khan	Proportion	2000	49.0	81.7	18.2
Afghanistan	Mata Khan	Proportion	2017	78.0	92.1	51.6
Afghanistan	Mata Khan	Count	2000	0	1	0
Afghanistan	Mata Khan	Count	2017	1	1	1
Afghanistan	Jadran	Proportion	2000	50.7	79.1	17.8

Table 4: ORS by Unit *(continued)*

Country	Unit	Metric	Year	Mean	Upper	Lower
Afghanistan	Jadran	Proportion	2017	70.3	90.6	44.9
Afghanistan	Jadran	Count	2000	1	1	0
Afghanistan	Jadran	Count	2017	1	1	0
Afghanistan	Kohi Safi	Proportion	2000	39.5	72.3	11.6
Afghanistan	Kohi Safi	Proportion	2017	51.5	80.6	23.0
Afghanistan	Kohi Safi	Count	2000	0	1	0
Afghanistan	Kohi Safi	Count	2017	1	1	0
Afghanistan	Royi Du Ab	Proportion	2000	20.2	47.5	5.7
Afghanistan	Royi Du Ab	Proportion	2017	33.1	61.2	13.4
Afghanistan	Royi Du Ab	Count	2000	0	0	0
Afghanistan	Royi Du Ab	Count	2017	0	1	0
Afghanistan	Sayyad	Proportion	2000	25.9	59.6	6.5
Afghanistan	Sayyad	Proportion	2017	40.0	69.5	15.5
Afghanistan	Sayyad	Count	2000	0	1	0
Afghanistan	Sayyad	Count	2017	0	1	0
Afghanistan	Farkhar	Proportion	2000	42.3	75.0	14.1
Afghanistan	Farkhar	Proportion	2017	35.7	64.4	14.9
Afghanistan	Farkhar	Count	2000	0	1	0
Afghanistan	Farkhar	Count	2017	0	1	0
Afghanistan	Shahidi Hasas	Proportion	2000	20.9	53.8	5.3
Afghanistan	Shahidi Hasas	Proportion	2017	33.6	59.3	12.6
Afghanistan	Shahidi Hasas	Count	2000	0	1	0
Afghanistan	Shahidi Hasas	Count	2017	0	1	0
Afghanistan	Markazi Bihsud	Proportion	2000	40.3	72.3	11.8
Afghanistan	Markazi Bihsud	Proportion	2017	34.2	62.3	13.8
Afghanistan	Markazi Bihsud	Count	2000	0	1	0
Afghanistan	Markazi Bihsud	Count	2017	0	1	0
Afghanistan	Qalat	Proportion	2000	39.0	72.1	11.5
Afghanistan	Qalat	Proportion	2017	47.5	73.7	20.8
Afghanistan	Qalat	Count	2000	0	1	0
Afghanistan	Qalat	Count	2017	0	1	0
Afghanistan	Khwahan	Proportion	2000	44.3	73.2	15.6
Afghanistan	Khwahan	Proportion	2017	61.3	86.8	33.4
Afghanistan	Khwahan	Count	2000	0	1	0
Afghanistan	Khwahan	Count	2017	1	1	0
Afghanistan	Qadis	Proportion	2000	43.5	74.5	15.6
Afghanistan	Qadis	Proportion	2017	66.9	87.0	40.5
Afghanistan	Qadis	Count	2000	0	1	0
Afghanistan	Qadis	Count	2017	1	1	0
Afghanistan	Doshi	Proportion	2000	24.4	58.2	5.3
Afghanistan	Doshi	Proportion	2017	39.5	70.9	16.2
Afghanistan	Doshi	Count	2000	0	1	0
Afghanistan	Doshi	Count	2017	0	1	0
Afghanistan	Dihdadi	Proportion	2000	19.8	48.7	5.0
Afghanistan	Dihdadi	Proportion	2017	33.0	62.2	12.1
Afghanistan	Dihdadi	Count	2000	0	0	0
Afghanistan	Dihdadi	Count	2017	0	1	0
Afghanistan	Khaki Safed	Proportion	2000	43.7	78.2	14.4
Afghanistan	Khaki Safed	Proportion	2017	33.6	66.8	12.6

Table 4: ORS by Unit *(continued)*

Country	Unit	Metric	Year	Mean	Upper	Lower
Afghanistan	Khaki Safed	Count	2000	0	1	0
Afghanistan	Khaki Safed	Count	2017	0	1	0
Afghanistan	Khwaja Sabz Posh	Proportion	2000	28.9	64.9	7.6
Afghanistan	Khwaja Sabz Posh	Proportion	2017	36.4	68.2	13.4
Afghanistan	Khwaja Sabz Posh	Count	2000	0	1	0
Afghanistan	Khwaja Sabz Posh	Count	2017	0	1	0
Afghanistan	Ghazni	Proportion	2000	38.2	71.9	11.0
Afghanistan	Ghazni	Proportion	2017	42.4	70.7	15.2
Afghanistan	Ghazni	Count	2000	0	1	0
Afghanistan	Ghazni	Count	2017	0	1	0
Afghanistan	Taywara	Proportion	2000	30.6	61.3	9.1
Afghanistan	Taywara	Proportion	2017	35.9	61.5	15.2
Afghanistan	Taywara	Count	2000	0	1	0
Afghanistan	Taywara	Count	2017	0	1	0
Afghanistan	Musa Qala	Proportion	2000	28.5	61.0	8.0
Afghanistan	Musa Qala	Proportion	2017	42.8	71.7	16.3
Afghanistan	Musa Qala	Count	2000	0	1	0
Afghanistan	Musa Qala	Count	2017	0	1	0
Afghanistan	Guzara	Proportion	2000	50.6	80.1	19.4
Afghanistan	Guzara	Proportion	2017	54.4	79.6	25.3
Afghanistan	Guzara	Count	2000	1	1	0
Afghanistan	Guzara	Count	2017	1	1	0
Afghanistan	Mardyan	Proportion	2000	18.8	46.2	4.8
Afghanistan	Mardyan	Proportion	2017	43.8	70.2	20.8
Afghanistan	Mardyan	Count	2000	0	0	0
Afghanistan	Mardyan	Count	2017	0	1	0
Afghanistan	Kabul City	Proportion	2000	47.3	82.3	12.5
Afghanistan	Kabul City	Proportion	2017	54.0	83.3	23.3
Afghanistan	Kabul City	Count	2000	0	1	0
Afghanistan	Kabul City	Count	2017	1	1	0
Afghanistan	Khakrez	Proportion	2000	30.5	69.6	7.9
Afghanistan	Khakrez	Proportion	2017	48.7	75.9	23.2
Afghanistan	Khakrez	Count	2000	0	1	0
Afghanistan	Khakrez	Count	2017	0	1	0
Afghanistan	Tagab	Proportion	2000	40.7	72.5	12.6
Afghanistan	Tagab	Proportion	2017	51.2	80.2	22.2
Afghanistan	Tagab	Count	2000	0	1	0
Afghanistan	Tagab	Count	2017	1	1	0
Afghanistan	Musa Khel	Proportion	2000	51.6	81.1	17.2
Afghanistan	Musa Khel	Proportion	2017	70.2	89.1	41.6
Afghanistan	Musa Khel	Count	2000	1	1	0
Afghanistan	Musa Khel	Count	2017	1	1	0
Afghanistan	Dara-i-Pech	Proportion	2000	65.3	89.6	32.2
Afghanistan	Dara-i-Pech	Proportion	2017	38.7	66.9	18.0
Afghanistan	Dara-i-Pech	Count	2000	1	1	0
Afghanistan	Dara-i-Pech	Count	2017	0	1	0
Afghanistan	Kunduz	Proportion	2000	39.3	75.2	10.9
Afghanistan	Kunduz	Proportion	2017	53.3	78.0	25.3
Afghanistan	Kunduz	Count	2000	0	1	0

Table 4: ORS by Unit *(continued)*

Country	Unit	Metric	Year	Mean	Upper	Lower
Afghanistan	Kunduz	Count	2017	1	1	0
Afghanistan	Dur Baba	Proportion	2000	60.0	88.6	29.2
Afghanistan	Dur Baba	Proportion	2017	51.9	78.0	24.3
Afghanistan	Dur Baba	Count	2000	1	1	0
Afghanistan	Dur Baba	Count	2017	1	1	0
Afghanistan	Waygal	Proportion	2000	52.4	84.1	19.0
Afghanistan	Waygal	Proportion	2017	32.5	57.9	13.6
Afghanistan	Waygal	Count	2000	1	1	0
Afghanistan	Waygal	Count	2017	0	1	0
Afghanistan	Nika	Proportion	2000	47.0	78.2	15.2
Afghanistan	Nika	Proportion	2017	80.2	94.9	58.2
Afghanistan	Nika	Count	2000	0	1	0
Afghanistan	Nika	Count	2017	1	1	1
Afghanistan	Jaji	Proportion	2000	61.0	86.0	25.6
Afghanistan	Jaji	Proportion	2017	62.7	86.4	30.0
Afghanistan	Jaji	Count	2000	1	1	0
Afghanistan	Jaji	Count	2017	1	1	0
Afghanistan	Salang	Proportion	2000	30.9	68.2	7.6
Afghanistan	Salang	Proportion	2017	43.3	74.4	18.6
Afghanistan	Salang	Count	2000	0	1	0
Afghanistan	Salang	Count	2017	0	1	0
Afghanistan	Sozma Qala	Proportion	2000	24.7	53.7	6.5
Afghanistan	Sozma Qala	Proportion	2017	35.3	61.9	15.0
Afghanistan	Sozma Qala	Count	2000	0	1	0
Afghanistan	Sozma Qala	Count	2017	0	1	0
Afghanistan	Ishkamish	Proportion	2000	43.8	76.7	16.0
Afghanistan	Ishkamish	Proportion	2017	41.1	70.7	18.1
Afghanistan	Ishkamish	Count	2000	0	1	0
Afghanistan	Ishkamish	Count	2017	0	1	0
Afghanistan	Tirin Kot	Proportion	2000	20.6	51.8	4.8
Afghanistan	Tirin Kot	Proportion	2017	57.1	82.6	29.9
Afghanistan	Tirin Kot	Count	2000	0	1	0
Afghanistan	Tirin Kot	Count	2017	1	1	0
Afghanistan	Maydan Shahr	Proportion	2000	41.1	76.5	9.1
Afghanistan	Maydan Shahr	Proportion	2017	65.3	87.9	32.5
Afghanistan	Maydan Shahr	Count	2000	0	1	0
Afghanistan	Maydan Shahr	Count	2017	1	1	0
Afghanistan	Shahjoy	Proportion	2000	37.8	73.7	11.2
Afghanistan	Shahjoy	Proportion	2017	47.2	75.7	19.0
Afghanistan	Shahjoy	Count	2000	0	1	0
Afghanistan	Shahjoy	Count	2017	0	1	0
Afghanistan	Kishim	Proportion	2000	44.6	76.1	13.7
Afghanistan	Kishim	Proportion	2017	53.0	73.7	27.1
Afghanistan	Kishim	Count	2000	0	1	0
Afghanistan	Kishim	Count	2017	1	1	0
Afghanistan	Qala-I-Naw	Proportion	2000	43.8	77.3	16.7
Afghanistan	Qala-I-Naw	Proportion	2017	65.6	87.2	37.9
Afghanistan	Qala-I-Naw	Count	2000	0	1	0
Afghanistan	Qala-I-Naw	Count	2017	1	1	0

Table 4: ORS by Unit *(continued)*

Country	Unit	Metric	Year	Mean	Upper	Lower
Afghanistan	Kahmard	Proportion	2000	24.7	55.2	6.6
Afghanistan	Kahmard	Proportion	2017	30.4	56.7	10.9
Afghanistan	Kahmard	Count	2000	0	1	0
Afghanistan	Kahmard	Count	2017	0	1	0
Afghanistan	Kaldar	Proportion	2000	23.2	50.0	5.5
Afghanistan	Kaldar	Proportion	2017	22.1	45.8	6.5
Afghanistan	Kaldar	Count	2000	0	0	0
Afghanistan	Kaldar	Count	2017	0	0	0
Afghanistan	Lash Wa Juwayn	Proportion	2000	41.7	73.7	14.8
Afghanistan	Lash Wa Juwayn	Proportion	2017	25.3	49.2	8.7
Afghanistan	Lash Wa Juwayn	Count	2000	0	1	0
Afghanistan	Lash Wa Juwayn	Count	2017	0	0	0
Afghanistan	Kohistan	Proportion	2000	29.7	56.7	9.6
Afghanistan	Kohistan	Proportion	2017	41.8	68.4	18.7
Afghanistan	Kohistan	Count	2000	0	1	0
Afghanistan	Kohistan	Count	2017	0	1	0
Afghanistan	Giro	Proportion	2000	34.3	70.7	12.0
Afghanistan	Giro	Proportion	2017	48.4	75.8	23.2
Afghanistan	Giro	Count	2000	0	1	0
Afghanistan	Giro	Count	2017	0	1	0
Afghanistan	Tulak	Proportion	2000	33.7	61.4	9.7
Afghanistan	Tulak	Proportion	2017	43.8	69.0	21.1
Afghanistan	Tulak	Count	2000	0	1	0
Afghanistan	Tulak	Count	2017	0	1	0
Afghanistan	Nad Ali	Proportion	2000	26.9	58.7	6.2
Afghanistan	Nad Ali	Proportion	2017	50.2	76.2	23.8
Afghanistan	Nad Ali	Count	2000	0	1	0
Afghanistan	Nad Ali	Count	2017	1	1	0
Afghanistan	Hirat City	Proportion	2000	55.6	87.3	22.2
Afghanistan	Hirat City	Proportion	2017	55.2	81.3	28.7
Afghanistan	Hirat City	Count	2000	1	1	0
Afghanistan	Hirat City	Count	2017	1	1	0
Afghanistan	Mingajik	Proportion	2000	20.7	51.1	5.0
Afghanistan	Mingajik	Proportion	2017	49.0	76.7	26.0
Afghanistan	Mingajik	Count	2000	0	1	0
Afghanistan	Mingajik	Count	2017	0	1	0
Afghanistan	Kalakan	Proportion	2000	44.1	77.2	11.3
Afghanistan	Kalakan	Proportion	2017	50.6	78.9	18.8
Afghanistan	Kalakan	Count	2000	0	1	0
Afghanistan	Kalakan	Count	2017	1	1	0
Afghanistan	Maruf	Proportion	2000	30.9	62.7	8.4
Afghanistan	Maruf	Proportion	2017	31.0	60.3	10.6
Afghanistan	Maruf	Count	2000	0	1	0
Afghanistan	Maruf	Count	2017	0	1	0
Afghanistan	Nadir Shah Kot	Proportion	2000	44.5	76.5	16.0
Afghanistan	Nadir Shah Kot	Proportion	2017	72.8	91.5	45.8
Afghanistan	Nadir Shah Kot	Count	2000	0	1	0
Afghanistan	Nadir Shah Kot	Count	2017	1	1	0
Afghanistan	Khas Kunar	Proportion	2000	69.9	91.6	38.5

Table 4: ORS by Unit *(continued)*

Country	Unit	Metric	Year	Mean	Upper	Lower
Afghanistan	Khas Kunar	Proportion	2017	46.8	74.1	22.0
Afghanistan	Khas Kunar	Count	2000	1	1	0
Afghanistan	Khas Kunar	Count	2017	0	1	0
Afghanistan	Qala-I-Zal	Proportion	2000	37.5	71.2	9.4
Afghanistan	Qala-I-Zal	Proportion	2017	47.1	71.0	21.2
Afghanistan	Qala-I-Zal	Count	2000	0	1	0
Afghanistan	Qala-I-Zal	Count	2017	0	1	0
Afghanistan	Goshta	Proportion	2000	67.8	90.6	36.4
Afghanistan	Goshta	Proportion	2017	52.3	78.0	27.0
Afghanistan	Goshta	Count	2000	1	1	0
Afghanistan	Goshta	Count	2017	1	1	0
Afghanistan	Omna	Proportion	2000	45.4	77.8	15.9
Afghanistan	Omna	Proportion	2017	84.6	95.3	67.4
Afghanistan	Omna	Count	2000	0	1	0
Afghanistan	Omna	Count	2017	1	1	1
Afghanistan	Jani Khel	Proportion	2000	58.1	84.6	26.7
Afghanistan	Jani Khel	Proportion	2017	68.5	87.9	40.6
Afghanistan	Jani Khel	Count	2000	1	1	0
Afghanistan	Jani Khel	Count	2017	1	1	0
Afghanistan	Shekh Ali	Proportion	2000	34.8	67.4	9.9
Afghanistan	Shekh Ali	Proportion	2017	49.8	78.9	19.3
Afghanistan	Shekh Ali	Count	2000	0	1	0
Afghanistan	Shekh Ali	Count	2017	0	1	0
Afghanistan	Kalafgan	Proportion	2000	43.1	79.0	12.7
Afghanistan	Kalafgan	Proportion	2017	40.3	64.8	19.3
Afghanistan	Kalafgan	Count	2000	0	1	0
Afghanistan	Kalafgan	Count	2017	0	1	0
Afghanistan	Nirkh	Proportion	2000	41.0	75.9	10.3
Afghanistan	Nirkh	Proportion	2017	65.5	87.2	34.7
Afghanistan	Nirkh	Count	2000	0	1	0
Afghanistan	Nirkh	Count	2017	1	1	0
Afghanistan	Shamul zayi	Proportion	2000	34.5	64.9	11.6
Afghanistan	Shamul zayi	Proportion	2017	47.2	78.0	19.0
Afghanistan	Shamul zayi	Count	2000	0	1	0
Afghanistan	Shamul zayi	Count	2017	0	1	0
Afghanistan	Kuran Wa Munjan	Proportion	2000	41.0	74.7	15.4
Afghanistan	Kuran Wa Munjan	Proportion	2017	40.0	65.7	15.8
Afghanistan	Kuran Wa Munjan	Count	2000	0	1	0
Afghanistan	Kuran Wa Munjan	Count	2017	0	1	0
Afghanistan	Khinjan	Proportion	2000	28.0	67.5	6.6
Afghanistan	Khinjan	Proportion	2017	43.9	74.7	18.6
Afghanistan	Khinjan	Count	2000	0	1	0
Afghanistan	Khinjan	Count	2017	0	1	0
Afghanistan	Khulm	Proportion	2000	22.0	50.5	5.1
Afghanistan	Khulm	Proportion	2017	28.5	55.8	10.8
Afghanistan	Khulm	Count	2000	0	1	0
Afghanistan	Khulm	Count	2017	0	1	0
Afghanistan	Purchaman	Proportion	2000	39.4	69.2	17.3
Afghanistan	Purchaman	Proportion	2017	34.4	58.2	12.7

Table 4: ORS by Unit *(continued)*

Country	Unit	Metric	Year	Mean	Upper	Lower
Afghanistan	Purchaman	Count	2000	0	1	0
Afghanistan	Purchaman	Count	2017	0	1	0
Afghanistan	Maymana	Proportion	2000	28.2	62.7	6.6
Afghanistan	Maymana	Proportion	2017	38.0	66.2	14.7
Afghanistan	Maymana	Count	2000	0	1	0
Afghanistan	Maymana	Count	2017	0	1	0
Afghanistan	Jaghatu	Proportion	2000	39.2	70.5	13.6
Afghanistan	Jaghatu	Proportion	2017	39.9	67.2	15.2
Afghanistan	Jaghatu	Count	2000	0	1	0
Afghanistan	Jaghatu	Count	2017	0	1	0
Afghanistan	Nahri Sarraj	Proportion	2000	26.6	61.7	6.8
Afghanistan	Nahri Sarraj	Proportion	2017	46.4	71.7	18.3
Afghanistan	Nahri Sarraj	Count	2000	0	1	0
Afghanistan	Nahri Sarraj	Count	2017	0	1	0
Afghanistan	Injil	Proportion	2000	52.9	84.9	21.4
Afghanistan	Injil	Proportion	2017	53.2	80.4	26.3
Afghanistan	Injil	Count	2000	1	1	0
Afghanistan	Injil	Count	2017	1	1	0
Afghanistan	Qarqin	Proportion	2000	22.5	50.3	6.4
Afghanistan	Qarqin	Proportion	2017	47.8	75.7	24.3
Afghanistan	Qarqin	Count	2000	0	1	0
Afghanistan	Qarqin	Count	2017	0	1	0
Afghanistan	Khaki Jabar	Proportion	2000	42.8	75.9	12.8
Afghanistan	Khaki Jabar	Proportion	2017	55.2	84.9	20.4
Afghanistan	Khaki Jabar	Count	2000	0	1	0
Afghanistan	Khaki Jabar	Count	2017	1	1	0
Afghanistan	Maywand	Proportion	2000	28.1	57.8	6.7
Afghanistan	Maywand	Proportion	2017	34.7	61.6	12.2
Afghanistan	Maywand	Count	2000	0	1	0
Afghanistan	Maywand	Count	2017	0	1	0
Afghanistan	Qalandar	Proportion	2000	47.8	77.9	16.4
Afghanistan	Qalandar	Proportion	2017	70.1	89.2	42.4
Afghanistan	Qalandar	Count	2000	0	1	0
Afghanistan	Qalandar	Count	2017	1	1	0
Afghanistan	Marwara	Proportion	2000	73.0	92.7	43.1
Afghanistan	Marwara	Proportion	2017	37.3	65.5	14.2
Afghanistan	Marwara	Count	2000	1	1	0
Afghanistan	Marwara	Count	2017	0	1	0
Afghanistan	Hisarak	Proportion	2000	63.5	88.9	28.1
Afghanistan	Hisarak	Proportion	2017	65.0	88.4	33.4
Afghanistan	Hisarak	Count	2000	1	1	0
Afghanistan	Hisarak	Count	2017	1	1	0
Afghanistan	Sar Hawza	Proportion	2000	45.7	77.6	15.6
Afghanistan	Sar Hawza	Proportion	2017	84.7	95.6	66.1
Afghanistan	Sar Hawza	Count	2000	0	1	0
Afghanistan	Sar Hawza	Count	2017	1	1	1
Afghanistan	Lija Mangal	Proportion	2000	62.7	88.4	27.1
Afghanistan	Lija Mangal	Proportion	2017	64.4	87.6	29.3
Afghanistan	Lija Mangal	Count	2000	1	1	0

Table 4: ORS by Unit *(continued)*

Country	Unit	Metric	Year	Mean	Upper	Lower
Afghanistan	Lija Mangal	Count	2017	1	1	0
Afghanistan	Shinwari	Proportion	2000	36.5	74.1	9.4
Afghanistan	Shinwari	Proportion	2017	48.8	80.4	20.4
Afghanistan	Shinwari	Count	2000	0	1	0
Afghanistan	Shinwari	Count	2017	0	1	0
Afghanistan	Khwaja Ghar	Proportion	2000	45.3	76.3	14.5
Afghanistan	Khwaja Ghar	Proportion	2017	46.9	71.1	21.6
Afghanistan	Khwaja Ghar	Count	2000	0	1	0
Afghanistan	Khwaja Ghar	Count	2017	0	1	0
Afghanistan	Sayid Abad	Proportion	2000	38.3	71.5	13.1
Afghanistan	Sayid Abad	Proportion	2017	58.3	83.0	31.9
Afghanistan	Sayid Abad	Count	2000	0	1	0
Afghanistan	Sayid Abad	Count	2017	1	1	0
Afghanistan	Shinkay	Proportion	2000	37.0	72.0	10.8
Afghanistan	Shinkay	Proportion	2017	39.3	68.8	16.0
Afghanistan	Shinkay	Count	2000	0	1	0
Afghanistan	Shinkay	Count	2017	0	1	0
Afghanistan	Ragh	Proportion	2000	44.7	76.8	16.5
Afghanistan	Ragh	Proportion	2017	66.7	88.3	41.5
Afghanistan	Ragh	Count	2000	0	1	0
Afghanistan	Ragh	Count	2017	1	1	0
Afghanistan	Khost Wa Firing	Proportion	2000	26.6	58.8	7.8
Afghanistan	Khost Wa Firing	Proportion	2017	33.4	61.1	14.7
Afghanistan	Khost Wa Firing	Count	2000	0	1	0
Afghanistan	Khost Wa Firing	Count	2017	0	1	0
Afghanistan	Kishindih	Proportion	2000	22.0	47.1	5.8
Afghanistan	Kishindih	Proportion	2017	33.2	57.4	13.8
Afghanistan	Kishindih	Count	2000	0	0	0
Afghanistan	Kishindih	Count	2017	0	1	0
Afghanistan	Pusht Rod	Proportion	2000	40.5	73.7	12.8
Afghanistan	Pusht Rod	Proportion	2017	30.0	60.7	11.9
Afghanistan	Pusht Rod	Count	2000	0	1	0
Afghanistan	Pusht Rod	Count	2017	0	1	0
Afghanistan	Pashtun Kot	Proportion	2000	28.7	62.2	7.9
Afghanistan	Pashtun Kot	Proportion	2017	38.2	68.4	15.0
Afghanistan	Pashtun Kot	Count	2000	0	1	0
Afghanistan	Pashtun Kot	Count	2017	0	1	0
Afghanistan	Jaghuri	Proportion	2000	37.6	73.9	11.3
Afghanistan	Jaghuri	Proportion	2017	34.7	61.4	12.3
Afghanistan	Jaghuri	Count	2000	0	1	0
Afghanistan	Jaghuri	Count	2017	0	1	0
Afghanistan	Naw Zad	Proportion	2000	28.5	63.9	8.4
Afghanistan	Naw Zad	Proportion	2017	41.0	69.7	17.8
Afghanistan	Naw Zad	Count	2000	0	1	0
Afghanistan	Naw Zad	Count	2017	0	1	0
Afghanistan	Karukh	Proportion	2000	49.3	81.2	18.3
Afghanistan	Karukh	Proportion	2017	56.8	80.0	29.3
Afghanistan	Karukh	Count	2000	0	1	0
Afghanistan	Karukh	Count	2017	1	1	0

Table 4: ORS by Unit *(continued)*

Country	Unit	Metric	Year	Mean	Upper	Lower
Afghanistan	Shibirghan	Proportion	2000	21.7	53.0	5.2
Afghanistan	Shibirghan	Proportion	2017	43.9	69.0	19.0
Afghanistan	Shibirghan	Count	2000	0	1	0
Afghanistan	Shibirghan	Count	2017	0	1	0
Afghanistan	Mirbacha Kot	Proportion	2000	43.4	78.9	11.6
Afghanistan	Mirbacha Kot	Proportion	2017	50.1	79.3	17.9
Afghanistan	Mirbacha Kot	Count	2000	0	1	0
Afghanistan	Mirbacha Kot	Count	2017	1	1	0
Afghanistan	Panjwayi	Proportion	2000	28.4	64.1	8.5
Afghanistan	Panjwayi	Proportion	2017	29.7	57.7	11.3
Afghanistan	Panjwayi	Count	2000	0	1	0
Afghanistan	Panjwayi	Count	2017	0	1	0
Afghanistan	Sabari	Proportion	2000	50.0	81.2	20.6
Afghanistan	Sabari	Proportion	2017	71.4	89.5	41.3
Afghanistan	Sabari	Count	2000	0	1	0
Afghanistan	Sabari	Count	2017	1	1	0
Afghanistan	Nali	Proportion	2000	65.9	91.7	31.1
Afghanistan	Nali	Proportion	2017	34.3	65.4	13.7
Afghanistan	Nali	Count	2000	1	1	0
Afghanistan	Nali	Count	2017	0	1	0
Afghanistan	Jalal abad	Proportion	2000	67.9	89.8	36.0
Afghanistan	Jalal abad	Proportion	2017	60.5	86.9	32.6
Afghanistan	Jalal abad	Count	2000	1	1	0
Afghanistan	Jalal abad	Count	2017	1	1	0
Afghanistan	Sarobi	Proportion	2000	44.7	77.9	16.7
Afghanistan	Sarobi	Proportion	2017	88.3	97.3	73.3
Afghanistan	Sarobi	Count	2000	0	1	0
Afghanistan	Sarobi	Count	2017	1	1	1
Afghanistan	Sayid Karam	Proportion	2000	63.1	88.9	33.0
Afghanistan	Sayid Karam	Proportion	2017	65.8	87.4	35.6
Afghanistan	Sayid Karam	Count	2000	1	1	0
Afghanistan	Sayid Karam	Count	2017	1	1	0
Afghanistan	Surkhi Parsa	Proportion	2000	38.5	73.6	10.9
Afghanistan	Surkhi Parsa	Proportion	2017	53.6	81.8	21.9
Afghanistan	Surkhi Parsa	Count	2000	0	1	0
Afghanistan	Surkhi Parsa	Count	2017	1	1	0
Afghanistan	Rustaq	Proportion	2000	43.4	78.9	15.5
Afghanistan	Rustaq	Proportion	2017	46.5	69.2	21.6
Afghanistan	Rustaq	Count	2000	0	1	0
Afghanistan	Rustaq	Count	2017	0	1	0
Afghanistan	Tarnak Wa Jaldak	Proportion	2000	36.3	70.6	9.5
Afghanistan	Tarnak Wa Jaldak	Proportion	2017	35.6	63.5	12.7
Afghanistan	Tarnak Wa Jaldak	Count	2000	0	1	0
Afghanistan	Tarnak Wa Jaldak	Count	2017	0	1	0
Afghanistan	Shahri Buzurg	Proportion	2000	42.8	80.8	13.6
Afghanistan	Shahri Buzurg	Proportion	2017	59.6	82.6	32.3
Afghanistan	Shahri Buzurg	Count	2000	0	1	0
Afghanistan	Shahri Buzurg	Count	2017	1	1	0
Afghanistan	Nahrin	Proportion	2000	24.4	56.3	5.1

Table 4: ORS by Unit *(continued)*

Country	Unit	Metric	Year	Mean	Upper	Lower
Afghanistan	Nahrin	Proportion	2017	36.9	66.7	13.2
Afghanistan	Nahrin	Count	2000	0	1	0
Afghanistan	Nahrin	Count	2017	0	1	0
Afghanistan	Marmul	Proportion	2000	22.5	50.2	5.3
Afghanistan	Marmul	Proportion	2017	29.3	57.7	9.7
Afghanistan	Marmul	Count	2000	0	1	0
Afghanistan	Marmul	Count	2017	0	1	0
Afghanistan	Qala-I-Kah	Proportion	2000	43.4	72.4	15.2
Afghanistan	Qala-I-Kah	Proportion	2017	31.3	60.3	11.7
Afghanistan	Qala-I-Kah	Count	2000	0	1	0
Afghanistan	Qala-I-Kah	Count	2017	0	1	0
Afghanistan	Qaram Qol	Proportion	2000	31.1	63.9	8.0
Afghanistan	Qaram Qol	Proportion	2017	35.0	63.2	14.4
Afghanistan	Qaram Qol	Count	2000	0	1	0
Afghanistan	Qaram Qol	Count	2017	0	1	0
Afghanistan	Malistan	Proportion	2000	35.2	71.3	10.8
Afghanistan	Malistan	Proportion	2017	36.1	62.6	14.3
Afghanistan	Malistan	Count	2000	0	1	0
Afghanistan	Malistan	Count	2017	0	1	0
Afghanistan	Nawa-i-Barak Zayi	Proportion	2000	27.5	62.6	6.2
Afghanistan	Nawa-i-Barak Zayi	Proportion	2017	52.5	78.2	23.0
Afghanistan	Nawa-i-Barak Zayi	Count	2000	0	1	0
Afghanistan	Nawa-i-Barak Zayi	Count	2017	1	1	0
Afghanistan	Kuhsan	Proportion	2000	51.5	81.9	19.2
Afghanistan	Kuhsan	Proportion	2017	55.6	81.5	30.6
Afghanistan	Kuhsan	Count	2000	1	1	0
Afghanistan	Kuhsan	Count	2017	1	1	0
Afghanistan	Musayi	Proportion	2000	41.8	75.6	11.3
Afghanistan	Musayi	Proportion	2017	54.6	82.6	21.3
Afghanistan	Musayi	Count	2000	0	1	0
Afghanistan	Musayi	Count	2017	1	1	0
Afghanistan	Reg	Proportion	2000	28.6	55.1	11.5
Afghanistan	Reg	Proportion	2017	32.1	57.9	12.7
Afghanistan	Reg	Count	2000	0	1	0
Afghanistan	Reg	Count	2017	0	1	0
Afghanistan	Spera	Proportion	2000	38.2	70.8	10.8
Afghanistan	Spera	Proportion	2017	76.4	93.5	52.7
Afghanistan	Spera	Count	2000	0	1	0
Afghanistan	Spera	Count	2017	1	1	1
Afghanistan	Narang Wa Badil	Proportion	2000	72.8	92.8	41.7
Afghanistan	Narang Wa Badil	Proportion	2017	42.0	70.3	18.7
Afghanistan	Narang Wa Badil	Count	2000	1	1	0
Afghanistan	Narang Wa Badil	Count	2017	0	1	0
Afghanistan	Kama	Proportion	2000	74.3	92.9	42.4
Afghanistan	Kama	Proportion	2017	62.0	85.1	36.1
Afghanistan	Kama	Count	2000	1	1	0
Afghanistan	Kama	Count	2017	1	1	0
Afghanistan	Sharan	Proportion	2000	44.7	80.0	16.1
Afghanistan	Sharan	Proportion	2017	77.5	91.7	50.8

Table 4: ORS by Unit *(continued)*

Country	Unit	Metric	Year	Mean	Upper	Lower
Afghanistan	Sharan	Count	2000	0	1	0
Afghanistan	Sharan	Count	2017	1	1	1
Afghanistan	Shamul	Proportion	2000	44.7	75.1	14.5
Afghanistan	Shamul	Proportion	2017	72.2	91.5	44.1
Afghanistan	Shamul	Count	2000	0	1	0
Afghanistan	Shamul	Count	2017	1	1	0
Afghanistan	Taluqan	Proportion	2000	49.3	83.3	16.8
Afghanistan	Taluqan	Proportion	2017	48.0	72.2	23.5
Afghanistan	Taluqan	Count	2000	0	1	0
Afghanistan	Taluqan	Count	2017	0	1	0
Afghanistan	Shighnan	Proportion	2000	41.5	71.2	14.8
Afghanistan	Shighnan	Proportion	2017	64.1	84.7	36.9
Afghanistan	Shighnan	Count	2000	0	1	0
Afghanistan	Shighnan	Count	2017	1	1	0
Afghanistan	Puli Khumri	Proportion	2000	23.3	52.0	5.0
Afghanistan	Puli Khumri	Proportion	2017	34.1	62.5	11.9
Afghanistan	Puli Khumri	Count	2000	0	1	0
Afghanistan	Puli Khumri	Count	2017	0	1	0
Afghanistan	Mazar-i-Sharif	Proportion	2000	25.1	54.4	5.9
Afghanistan	Mazar-i-Sharif	Proportion	2017	35.2	63.3	13.0
Afghanistan	Mazar-i-Sharif	Count	2000	0	1	0
Afghanistan	Mazar-i-Sharif	Count	2017	0	1	0
Afghanistan	Shib Koh	Proportion	2000	43.2	74.0	14.0
Afghanistan	Shib Koh	Proportion	2017	28.1	53.5	9.0
Afghanistan	Shib Koh	Count	2000	0	1	0
Afghanistan	Shib Koh	Count	2017	0	1	0
Afghanistan	Qaysar	Proportion	2000	29.6	70.3	7.4
Afghanistan	Qaysar	Proportion	2017	42.8	72.7	17.8
Afghanistan	Qaysar	Count	2000	0	1	0
Afghanistan	Qaysar	Count	2017	0	1	0
Afghanistan	Muqur	Proportion	2000	36.5	69.2	12.1
Afghanistan	Muqur	Proportion	2017	36.1	65.3	13.8
Afghanistan	Muqur	Count	2000	0	1	0
Afghanistan	Muqur	Count	2017	0	1	0
Afghanistan	Reg	Proportion	2000	29.1	59.5	8.3
Afghanistan	Reg	Proportion	2017	45.0	70.8	19.6
Afghanistan	Reg	Count	2000	0	1	0
Afghanistan	Reg	Count	2017	0	1	0
Afghanistan	Kushk	Proportion	2000	48.9	81.1	20.1
Afghanistan	Kushk	Proportion	2017	56.2	80.6	29.8
Afghanistan	Kushk	Count	2000	0	1	0
Afghanistan	Kushk	Count	2017	1	1	0
Afghanistan	Paghman	Proportion	2000	45.5	82.0	11.6
Afghanistan	Paghman	Proportion	2017	55.9	83.7	24.1
Afghanistan	Paghman	Count	2000	0	1	0
Afghanistan	Paghman	Count	2017	1	1	0
Afghanistan	Shah Wali Kot	Proportion	2000	30.7	61.7	7.6
Afghanistan	Shah Wali Kot	Proportion	2017	47.1	73.2	22.3
Afghanistan	Shah Wali Kot	Count	2000	0	1	0

Table 4: ORS by Unit *(continued)*

Country	Unit	Metric	Year	Mean	Upper	Lower
Afghanistan	Shah Wali Kot	Count	2017	0	1	0
Afghanistan	Tani	Proportion	2000	36.5	70.6	11.2
Afghanistan	Tani	Proportion	2017	75.4	91.9	49.8
Afghanistan	Tani	Count	2000	0	1	0
Afghanistan	Tani	Count	2017	1	1	0
Afghanistan	Nurgal	Proportion	2000	69.0	91.3	38.0
Afghanistan	Nurgal	Proportion	2017	49.9	76.2	26.7
Afghanistan	Nurgal	Count	2000	1	1	0
Afghanistan	Nurgal	Count	2017	0	1	0
Afghanistan	Khogyani	Proportion	2000	67.5	90.5	31.9
Afghanistan	Khogyani	Proportion	2017	63.1	88.7	33.9
Afghanistan	Khogyani	Count	2000	1	1	0
Afghanistan	Khogyani	Count	2017	1	1	0
Afghanistan	Urgun	Proportion	2000	44.9	76.9	15.9
Afghanistan	Urgun	Proportion	2017	86.0	96.8	68.9
Afghanistan	Urgun	Count	2000	0	1	0
Afghanistan	Urgun	Count	2017	1	1	1
Afghanistan	Shwak	Proportion	2000	54.7	83.6	17.5
Afghanistan	Shwak	Proportion	2017	67.1	90.0	38.4
Afghanistan	Shwak	Count	2000	1	1	0
Afghanistan	Shwak	Count	2017	1	1	0
Afghanistan	Warsaj	Proportion	2000	37.2	69.8	11.1
Afghanistan	Warsaj	Proportion	2017	38.1	62.1	17.8
Afghanistan	Warsaj	Count	2000	0	1	0
Afghanistan	Warsaj	Count	2017	0	1	0
Afghanistan	Wakhan	Proportion	2000	39.9	65.4	18.1
Afghanistan	Wakhan	Proportion	2017	46.5	71.1	22.5
Afghanistan	Wakhan	Count	2000	0	1	0
Afghanistan	Wakhan	Count	2017	0	1	0
Afghanistan	Tala Wa Barfak	Proportion	2000	29.6	63.0	9.5
Afghanistan	Tala Wa Barfak	Proportion	2017	43.9	70.8	16.8
Afghanistan	Tala Wa Barfak	Count	2000	0	1	0
Afghanistan	Tala Wa Barfak	Count	2017	0	1	0
Afghanistan	Nahri Shahi	Proportion	2000	21.6	48.6	5.7
Afghanistan	Nahri Shahi	Proportion	2017	31.9	58.7	12.1
Afghanistan	Nahri Shahi	Count	2000	0	0	0
Afghanistan	Nahri Shahi	Count	2017	0	1	0
Afghanistan	Shirin Tagab	Proportion	2000	30.8	59.9	8.9
Afghanistan	Shirin Tagab	Proportion	2017	36.4	65.3	14.5
Afghanistan	Shirin Tagab	Count	2000	0	1	0
Afghanistan	Shirin Tagab	Count	2017	0	1	0
Afghanistan	Nawa	Proportion	2000	35.8	68.3	12.4
Afghanistan	Nawa	Proportion	2017	51.9	79.5	24.8
Afghanistan	Nawa	Count	2000	0	1	0
Afghanistan	Nawa	Count	2017	1	1	0
Afghanistan	Sangin	Proportion	2000	27.1	61.6	6.9
Afghanistan	Sangin	Proportion	2017	46.2	72.9	18.6
Afghanistan	Sangin	Count	2000	0	1	0
Afghanistan	Sangin	Count	2017	0	1	0

Table 4: ORS by Unit *(continued)*

Country	Unit	Metric	Year	Mean	Upper	Lower
Afghanistan	Kushki Kuhna	Proportion	2000	46.7	79.7	18.5
Afghanistan	Kushki Kuhna	Proportion	2017	60.3	82.7	31.4
Afghanistan	Kushki Kuhna	Count	2000	0	1	0
Afghanistan	Kushki Kuhna	Count	2017	1	1	0
Afghanistan	Qarabagh	Proportion	2000	41.8	75.8	11.1
Afghanistan	Qarabagh	Proportion	2017	50.5	80.1	20.3
Afghanistan	Qarabagh	Count	2000	0	1	0
Afghanistan	Qarabagh	Count	2017	1	1	0
Afghanistan	Shorabak	Proportion	2000	28.6	61.7	7.7
Afghanistan	Shorabak	Proportion	2017	23.2	50.2	5.9
Afghanistan	Shorabak	Count	2000	0	1	0
Afghanistan	Shorabak	Count	2017	0	1	0
Afghanistan	Tere Zayi	Proportion	2000	42.4	77.8	14.1
Afghanistan	Tere Zayi	Proportion	2017	71.2	88.8	39.6
Afghanistan	Tere Zayi	Count	2000	0	1	0
Afghanistan	Tere Zayi	Count	2017	1	1	0
Afghanistan	Sirkanay	Proportion	2000	70.6	90.9	40.6
Afghanistan	Sirkanay	Proportion	2017	40.3	67.5	17.3
Afghanistan	Sirkanay	Count	2000	1	1	0
Afghanistan	Sirkanay	Count	2017	0	1	0
Afghanistan	Kuz Kunar	Proportion	2000	68.9	91.2	35.2
Afghanistan	Kuz Kunar	Proportion	2017	55.5	82.3	31.3
Afghanistan	Kuz Kunar	Count	2000	1	1	0
Afghanistan	Kuz Kunar	Count	2017	1	1	0
Afghanistan	Wazakhwa	Proportion	2000	45.2	78.4	17.9
Afghanistan	Wazakhwa	Proportion	2017	76.7	92.9	50.6
Afghanistan	Wazakhwa	Count	2000	0	1	0
Afghanistan	Wazakhwa	Count	2017	1	1	1
Afghanistan	Zurmat	Proportion	2000	52.9	81.6	20.5
Afghanistan	Zurmat	Proportion	2017	75.4	91.3	49.2
Afghanistan	Zurmat	Count	2000	1	1	0
Afghanistan	Zurmat	Count	2017	1	1	0
Afghanistan	Yangi Qala	Proportion	2000	44.3	78.6	13.5
Afghanistan	Yangi Qala	Proportion	2017	46.1	68.5	23.5
Afghanistan	Yangi Qala	Count	2000	0	1	0
Afghanistan	Yangi Qala	Count	2017	0	1	0
Afghanistan	Zebak	Proportion	2000	43.7	74.8	13.3
Afghanistan	Zebak	Proportion	2017	55.7	81.4	25.8
Afghanistan	Zebak	Count	2000	0	1	0
Afghanistan	Zebak	Count	2017	1	1	0
Afghanistan	Sholgara	Proportion	2000	20.1	43.9	5.3
Afghanistan	Sholgara	Proportion	2017	32.3	59.6	14.0
Afghanistan	Sholgara	Count	2000	0	0	0
Afghanistan	Sholgara	Count	2017	0	1	0
Afghanistan	Nawur	Proportion	2000	37.5	68.2	10.9
Afghanistan	Nawur	Proportion	2017	30.0	54.0	10.5
Afghanistan	Nawur	Count	2000	0	1	0
Afghanistan	Nawur	Count	2017	0	1	0
Afghanistan	Washer	Proportion	2000	28.4	58.9	9.7

Table 4: ORS by Unit *(continued)*

Country	Unit	Metric	Year	Mean	Upper	Lower
Afghanistan	Washer	Proportion	2017	40.5	66.1	17.1
Afghanistan	Washer	Count	2000	0	1	0
Afghanistan	Washer	Count	2017	0	1	0
Afghanistan	Obe	Proportion	2000	51.9	83.7	21.8
Afghanistan	Obe	Proportion	2017	62.7	84.4	35.8
Afghanistan	Obe	Count	2000	1	1	0
Afghanistan	Obe	Count	2017	1	1	0
Afghanistan	Shakar Dara	Proportion	2000	42.6	77.9	10.1
Afghanistan	Shakar Dara	Proportion	2017	49.0	79.4	19.3
Afghanistan	Shakar Dara	Count	2000	0	1	0
Afghanistan	Shakar Dara	Count	2017	0	1	0
Afghanistan	Spin Boldak	Proportion	2000	27.9	64.4	6.7
Afghanistan	Spin Boldak	Proportion	2017	17.3	40.8	5.8
Afghanistan	Spin Boldak	Count	2000	0	1	0
Afghanistan	Spin Boldak	Count	2017	0	0	0
Afghanistan	Lal Pur	Proportion	2000	66.3	90.4	35.8
Afghanistan	Lal Pur	Proportion	2017	50.9	75.1	26.6
Afghanistan	Lal Pur	Count	2000	1	1	0
Afghanistan	Lal Pur	Count	2017	1	1	0
Afghanistan	Wolmamay	Proportion	2000	43.4	76.4	15.0
Afghanistan	Wolmamay	Proportion	2017	78.5	93.4	51.9
Afghanistan	Wolmamay	Count	2000	0	1	0
Afghanistan	Wolmamay	Count	2017	1	1	1
Afghanistan	Shortepa	Proportion	2000	22.2	48.6	6.5
Afghanistan	Shortepa	Proportion	2017	31.3	56.5	13.1
Afghanistan	Shortepa	Count	2000	0	0	0
Afghanistan	Shortepa	Count	2017	0	1	0
Afghanistan	Qarabagh	Proportion	2000	37.7	71.3	12.6
Afghanistan	Qarabagh	Proportion	2017	31.6	59.7	12.8
Afghanistan	Qarabagh	Count	2000	0	1	0
Afghanistan	Qarabagh	Count	2017	0	1	0
Afghanistan	Pashtun Zarghun	Proportion	2000	49.8	82.4	16.5
Afghanistan	Pashtun Zarghun	Proportion	2017	55.5	81.8	28.9
Afghanistan	Pashtun Zarghun	Count	2000	0	1	0
Afghanistan	Pashtun Zarghun	Count	2017	1	1	0
Afghanistan	Surobi	Proportion	2000	42.2	74.6	12.7
Afghanistan	Surobi	Proportion	2017	55.1	84.9	23.5
Afghanistan	Surobi	Count	2000	0	1	0
Afghanistan	Surobi	Count	2017	1	1	0
Afghanistan	Muhmand Dara	Proportion	2000	66.9	90.5	37.1
Afghanistan	Muhmand Dara	Proportion	2017	54.0	77.7	28.6
Afghanistan	Muhmand Dara	Count	2000	1	1	0
Afghanistan	Muhmand Dara	Count	2017	1	1	0
Afghanistan	Zarghun Shahr	Proportion	2000	47.9	80.3	20.6
Afghanistan	Zarghun Shahr	Proportion	2017	78.6	92.0	56.5
Afghanistan	Zarghun Shahr	Count	2000	0	1	0
Afghanistan	Zarghun Shahr	Count	2017	1	1	1
Afghanistan	Zana Khan	Proportion	2000	35.6	68.9	11.5
Afghanistan	Zana Khan	Proportion	2017	51.5	78.9	25.1

Table 4: ORS by Unit *(continued)*

Country	Unit	Metric	Year	Mean	Upper	Lower
Afghanistan	Zana Khan	Count	2000	0	1	0
Afghanistan	Zana Khan	Count	2017	1	1	0
Afghanistan	Shindand	Proportion	2000	46.7	77.1	16.0
Afghanistan	Shindand	Proportion	2017	56.8	82.3	25.6
Afghanistan	Shindand	Count	2000	0	1	0
Afghanistan	Shindand	Count	2017	1	1	0
Afghanistan	Nazyan	Proportion	2000	68.1	92.9	35.4
Afghanistan	Nazyan	Proportion	2017	56.5	81.4	28.8
Afghanistan	Nazyan	Count	2000	1	1	0
Afghanistan	Nazyan	Count	2017	1	1	0
Afghanistan	Ziluk	Proportion	2000	43.9	76.6	13.8
Afghanistan	Ziluk	Proportion	2017	79.1	94.8	56.6
Afghanistan	Ziluk	Count	2000	0	1	0
Afghanistan	Ziluk	Count	2017	1	1	1
Afghanistan	Zinda Jan	Proportion	2000	51.2	82.6	20.9
Afghanistan	Zinda Jan	Proportion	2017	53.1	77.9	28.2
Afghanistan	Zinda Jan	Count	2000	1	1	0
Afghanistan	Zinda Jan	Count	2017	1	1	0
Afghanistan	Pachir Wa Agam	Proportion	2000	63.4	89.6	28.1
Afghanistan	Pachir Wa Agam	Proportion	2017	61.2	87.0	34.1
Afghanistan	Pachir Wa Agam	Count	2000	1	1	0
Afghanistan	Pachir Wa Agam	Count	2017	1	1	0
Afghanistan	Rodat	Proportion	2000	66.1	89.4	33.7
Afghanistan	Rodat	Proportion	2017	58.9	82.7	33.7
Afghanistan	Rodat	Count	2000	1	1	0
Afghanistan	Rodat	Count	2017	1	1	0
Afghanistan	Sherzad	Proportion	2000	67.1	89.3	30.7
Afghanistan	Sherzad	Proportion	2017	65.6	89.6	37.8
Afghanistan	Sherzad	Count	2000	1	1	0
Afghanistan	Sherzad	Count	2017	1	1	0
Afghanistan	Shinwar	Proportion	2000	72.1	93.7	41.4
Afghanistan	Shinwar	Proportion	2017	60.9	84.7	33.1
Afghanistan	Shinwar	Count	2000	1	1	0
Afghanistan	Shinwar	Count	2017	1	1	0
Afghanistan	Surkh Rod	Proportion	2000	65.0	89.0	29.9
Afghanistan	Surkh Rod	Proportion	2017	60.4	85.4	34.4
Afghanistan	Surkh Rod	Count	2000	1	1	0
Afghanistan	Surkh Rod	Count	2017	1	1	0
Algeria	Adrar	Proportion	2000	28.5	43.2	16.8
Algeria	Adrar	Proportion	2017	28.5	43.6	17.2
Algeria	Adrar	Count	2000	0	0	0
Algeria	Adrar	Count	2017	0	0	0
Algeria	Ain Benian	Proportion	2000	31.7	45.7	19.5
Algeria	Ain Benian	Proportion	2017	32.9	45.8	19.4
Algeria	Ain Benian	Count	2000	0	0	0
Algeria	Ain Benian	Count	2017	0	0	0
Algeria	Aghlal	Proportion	2000	28.4	42.8	16.5
Algeria	Aghlal	Proportion	2017	34.3	49.4	21.5
Algeria	Aghlal	Count	2000	0	0	0

Table 4: ORS by Unit *(continued)*

Country	Unit	Metric	Year	Mean	Upper	Lower
Algeria	Aghlal	Count	2017	0	0	0
Algeria	Ain Benian	Proportion	2000	31.6	47.3	20.1
Algeria	Ain Benian	Proportion	2017	31.9	47.2	18.5
Algeria	Ain Benian	Count	2000	0	0	0
Algeria	Ain Benian	Count	2017	0	0	0
Algeria	Ain El Berda	Proportion	2000	34.6	50.9	21.5
Algeria	Ain El Berda	Proportion	2017	38.0	54.7	24.4
Algeria	Ain El Berda	Count	2000	0	1	0
Algeria	Ain El Berda	Count	2017	0	1	0
Algeria	Ain Djasser	Proportion	2000	33.7	49.0	18.1
Algeria	Ain Djasser	Proportion	2017	36.7	51.2	22.1
Algeria	Ain Djasser	Count	2000	0	0	0
Algeria	Ain Djasser	Count	2017	0	1	0
Algeria	Abadla	Proportion	2000	33.6	50.9	20.6
Algeria	Abadla	Proportion	2017	34.7	52.2	19.8
Algeria	Abadla	Count	2000	0	1	0
Algeria	Abadla	Count	2017	0	1	0
Algeria	Adekar	Proportion	2000	29.4	45.1	18.0
Algeria	Adekar	Proportion	2017	34.9	49.2	22.3
Algeria	Adekar	Count	2000	0	0	0
Algeria	Adekar	Count	2017	0	0	0
Algeria	Ain Naga	Proportion	2000	29.9	44.1	18.4
Algeria	Ain Naga	Proportion	2017	31.8	46.2	20.1
Algeria	Ain Naga	Count	2000	0	0	0
Algeria	Ain Naga	Count	2017	0	0	0
Algeria	Ain Romana	Proportion	2000	31.0	44.8	19.2
Algeria	Ain Romana	Proportion	2017	33.8	51.9	20.2
Algeria	Ain Romana	Count	2000	0	0	0
Algeria	Ain Romana	Count	2017	0	1	0
Algeria	Ain Taghrout	Proportion	2000	33.3	47.2	20.8
Algeria	Ain Taghrout	Proportion	2017	36.8	53.6	21.6
Algeria	Ain Taghrout	Count	2000	0	0	0
Algeria	Ain Taghrout	Count	2017	0	1	0
Algeria	Aghbalou	Proportion	2000	31.9	47.8	18.7
Algeria	Aghbalou	Proportion	2017	36.9	51.9	23.2
Algeria	Aghbalou	Count	2000	0	0	0
Algeria	Aghbalou	Count	2017	0	1	0
Algeria	Afir	Proportion	2000	28.7	45.7	16.2
Algeria	Afir	Proportion	2017	34.6	48.6	19.8
Algeria	Afir	Count	2000	0	0	0
Algeria	Afir	Count	2017	0	0	0
Algeria	Abou El Hassen	Proportion	2000	28.6	43.4	17.3
Algeria	Abou El Hassen	Proportion	2017	33.5	50.5	19.4
Algeria	Abou El Hassen	Count	2000	0	0	0
Algeria	Abou El Hassen	Count	2017	0	1	0
Algeria	Ain Abid	Proportion	2000	34.5	50.2	20.4
Algeria	Ain Abid	Proportion	2017	36.2	52.5	22.5
Algeria	Ain Abid	Count	2000	0	1	0
Algeria	Ain Abid	Count	2017	0	1	0

Table 4: ORS by Unit *(continued)*

Country	Unit	Metric	Year	Mean	Upper	Lower
Algeria	Ain Chouhada	Proportion	2000	30.4	45.9	17.7
Algeria	Ain Chouhada	Proportion	2017	31.4	46.1	20.4
Algeria	Ain Chouhada	Count	2000	0	0	0
Algeria	Ain Chouhada	Count	2017	0	0	0
Algeria	Ain El Orak	Proportion	2000	30.4	45.6	16.7
Algeria	Ain El Orak	Proportion	2017	33.4	47.4	21.0
Algeria	Ain El Orak	Count	2000	0	0	0
Algeria	Ain El Orak	Count	2017	0	0	0
Algeria	Bayadha	Proportion	2000	30.0	47.1	17.2
Algeria	Bayadha	Proportion	2017	29.7	45.7	17.2
Algeria	Bayadha	Count	2000	0	0	0
Algeria	Bayadha	Count	2017	0	0	0
Algeria	Ain El Assel	Proportion	2000	32.9	48.3	20.2
Algeria	Ain El Assel	Proportion	2017	37.9	52.7	24.6
Algeria	Ain El Assel	Count	2000	0	0	0
Algeria	Ain El Assel	Count	2017	0	1	0
Algeria	Berriane	Proportion	2000	29.8	45.1	18.6
Algeria	Berriane	Proportion	2017	32.2	45.4	20.3
Algeria	Berriane	Count	2000	0	0	0
Algeria	Berriane	Count	2017	0	0	0
Algeria	Ain Ben Beida	Proportion	2000	33.9	50.9	20.8
Algeria	Ain Ben Beida	Proportion	2017	37.4	55.0	22.6
Algeria	Ain Ben Beida	Count	2000	0	1	0
Algeria	Ain Ben Beida	Count	2017	0	1	0
Algeria	Bordj El Haouasse	Proportion	2000	28.7	40.3	18.7
Algeria	Bordj El Haouasse	Proportion	2017	32.0	46.2	20.9
Algeria	Bordj El Haouasse	Count	2000	0	0	0
Algeria	Bordj El Haouasse	Count	2017	0	0	0
Algeria	Bordj Tahar	Proportion	2000	32.6	47.0	20.7
Algeria	Bordj Tahar	Proportion	2017	38.0	54.0	24.3
Algeria	Bordj Tahar	Count	2000	0	0	0
Algeria	Bordj Tahar	Count	2017	0	1	0
Algeria	Ain Touila	Proportion	2000	30.9	48.2	19.1
Algeria	Ain Touila	Proportion	2017	35.1	51.1	21.6
Algeria	Ain Touila	Count	2000	0	0	0
Algeria	Ain Touila	Count	2017	0	1	0
Algeria	Aflou	Proportion	2000	29.3	44.7	16.3
Algeria	Aflou	Proportion	2017	33.0	46.8	21.1
Algeria	Aflou	Count	2000	0	0	0
Algeria	Aflou	Count	2017	0	0	0
Algeria	Ain El Hadjel	Proportion	2000	31.9	47.3	18.5
Algeria	Ain El Hadjel	Proportion	2017	33.5	47.6	20.7
Algeria	Ain El Hadjel	Count	2000	0	0	0
Algeria	Ain El Hadjel	Count	2017	0	0	0
Algeria	Ain Fares	Proportion	2000	27.2	41.5	16.2
Algeria	Ain Fares	Proportion	2017	34.7	50.9	20.6
Algeria	Ain Fares	Count	2000	0	0	0
Algeria	Ain Fares	Count	2017	0	1	0
Algeria	Ain Boucif	Proportion	2000	33.8	51.6	20.0

Table 4: ORS by Unit *(continued)*

Country	Unit	Metric	Year	Mean	Upper	Lower
Algeria	Ain Boucif	Proportion	2017	35.1	49.5	22.4
Algeria	Ain Boucif	Count	2000	0	1	0
Algeria	Ain Boucif	Count	2017	0	0	0
Algeria	Ahmed Rachedi	Proportion	2000	34.1	46.8	21.0
Algeria	Ahmed Rachedi	Proportion	2017	37.8	53.8	23.2
Algeria	Ahmed Rachedi	Count	2000	0	0	0
Algeria	Ahmed Rachedi	Count	2017	0	1	0
Algeria	Achaacha	Proportion	2000	27.0	43.9	15.2
Algeria	Achaacha	Proportion	2017	36.1	51.3	22.1
Algeria	Achaacha	Count	2000	0	0	0
Algeria	Achaacha	Count	2017	0	1	0
Algeria	Ain Ben Khelil	Proportion	2000	31.7	44.3	19.3
Algeria	Ain Ben Khelil	Proportion	2017	33.2	47.8	18.5
Algeria	Ain Ben Khelil	Count	2000	0	0	0
Algeria	Ain Ben Khelil	Count	2017	0	0	0
Algeria	Ain Biya	Proportion	2000	25.4	37.8	15.5
Algeria	Ain Biya	Proportion	2017	32.5	47.3	18.1
Algeria	Ain Biya	Count	2000	0	0	0
Algeria	Ain Biya	Count	2017	0	0	0
Algeria	Ain Beida	Proportion	2000	29.5	42.4	17.0
Algeria	Ain Beida	Proportion	2017	28.9	42.5	18.1
Algeria	Ain Beida	Count	2000	0	0	0
Algeria	Ain Beida	Count	2017	0	0	0
Algeria	Ain Bebouche	Proportion	2000	32.5	47.3	19.8
Algeria	Ain Bebouche	Proportion	2017	36.9	52.2	21.8
Algeria	Ain Bebouche	Count	2000	0	0	0
Algeria	Ain Bebouche	Count	2017	0	1	0
Algeria	Ain Rahma	Proportion	2000	26.4	40.8	15.8
Algeria	Ain Rahma	Proportion	2017	34.5	49.7	19.3
Algeria	Ain Rahma	Count	2000	0	0	0
Algeria	Ain Rahma	Count	2017	0	0	0
Algeria	Ain El Hadjar	Proportion	2000	31.2	46.5	19.5
Algeria	Ain El Hadjar	Proportion	2017	36.5	53.7	21.3
Algeria	Ain El Hadjar	Count	2000	0	0	0
Algeria	Ain El Hadjar	Count	2017	0	1	0
Algeria	Ain-Legradj	Proportion	2000	32.8	48.1	20.2
Algeria	Ain-Legradj	Proportion	2017	37.3	53.0	22.8
Algeria	Ain-Legradj	Count	2000	0	0	0
Algeria	Ain-Legradj	Count	2017	0	1	0
Algeria	Ain Adden	Proportion	2000	27.2	42.0	15.1
Algeria	Ain Adden	Proportion	2017	34.8	48.7	20.4
Algeria	Ain Adden	Count	2000	0	0	0
Algeria	Ain Adden	Count	2017	0	0	0
Algeria	Ain Bouziane	Proportion	2000	34.2	48.3	21.2
Algeria	Ain Bouziane	Proportion	2017	38.6	53.8	24.5
Algeria	Ain Bouziane	Count	2000	0	0	0
Algeria	Ain Bouziane	Count	2017	0	1	0
Algeria	Ain Soltane	Proportion	2000	33.9	48.7	21.8
Algeria	Ain Soltane	Proportion	2017	36.0	50.9	23.9

Table 4: ORS by Unit *(continued)*

Country	Unit	Metric	Year	Mean	Upper	Lower
Algeria	Ain Soltane	Count	2000	0	0	0
Algeria	Ain Soltane	Count	2017	0	1	0
Algeria	Abalissa	Proportion	2000	30.0	41.0	19.4
Algeria	Abalissa	Proportion	2017	30.8	39.6	21.7
Algeria	Abalissa	Count	2000	0	0	0
Algeria	Abalissa	Count	2017	0	0	0
Algeria	Ain Zerga	Proportion	2000	31.6	46.7	19.0
Algeria	Ain Zerga	Proportion	2017	36.3	50.7	23.1
Algeria	Ain Zerga	Count	2000	0	0	0
Algeria	Ain Zerga	Count	2017	0	1	0
Algeria	Ain Bouchekif	Proportion	2000	32.0	46.6	19.7
Algeria	Ain Bouchekif	Proportion	2017	35.2	50.1	22.9
Algeria	Ain Bouchekif	Count	2000	0	0	0
Algeria	Ain Bouchekif	Count	2017	0	1	0
Algeria	Oum El Assel	Proportion	2000	32.1	41.3	24.0
Algeria	Oum El Assel	Proportion	2017	31.4	40.3	22.9
Algeria	Oum El Assel	Count	2000	0	0	0
Algeria	Oum El Assel	Count	2017	0	0	0
Algeria	Aghbal	Proportion	2000	30.2	44.1	18.6
Algeria	Aghbal	Proportion	2017	33.0	47.2	20.9
Algeria	Aghbal	Count	2000	0	0	0
Algeria	Aghbal	Count	2017	0	0	0
Algeria	Ammari	Proportion	2000	32.7	48.4	20.2
Algeria	Ammari	Proportion	2017	35.2	50.5	20.3
Algeria	Ammari	Count	2000	0	0	0
Algeria	Ammari	Count	2017	0	1	0
Algeria	Abi Youcef	Proportion	2000	29.1	45.9	17.2
Algeria	Abi Youcef	Proportion	2017	35.0	49.6	22.3
Algeria	Abi Youcef	Count	2000	0	0	0
Algeria	Abi Youcef	Count	2017	0	0	0
Algeria	Ain Fettah	Proportion	2000	29.9	46.2	19.0
Algeria	Ain Fettah	Proportion	2017	35.4	51.7	20.8
Algeria	Ain Fettah	Count	2000	0	0	0
Algeria	Ain Fettah	Count	2017	0	1	0
Algeria	Akabli	Proportion	2000	28.7	42.3	19.2
Algeria	Akabli	Proportion	2017	30.4	45.0	18.4
Algeria	Akabli	Count	2000	0	0	0
Algeria	Akabli	Count	2017	0	0	0
Algeria	Ain Bouihi	Proportion	2000	30.4	45.7	18.3
Algeria	Ain Bouihi	Proportion	2017	33.9	47.9	19.4
Algeria	Ain Bouihi	Count	2000	0	0	0
Algeria	Ain Bouihi	Count	2017	0	0	0
Algeria	Ain El Arbaa	Proportion	2000	28.3	40.7	17.1
Algeria	Ain El Arbaa	Proportion	2017	34.7	48.8	21.3
Algeria	Ain El Arbaa	Count	2000	0	0	0
Algeria	Ain El Arbaa	Count	2017	0	0	0
Algeria	Baraki	Proportion	2000	30.3	45.1	19.4
Algeria	Baraki	Proportion	2017	32.7	48.0	19.7
Algeria	Baraki	Count	2000	0	0	0

Table 4: ORS by Unit *(continued)*

Country	Unit	Metric	Year	Mean	Upper	Lower
Algeria	Baraki	Count	2017	0	0	0
Algeria	Annaba	Proportion	2000	32.8	47.9	19.5
Algeria	Annaba	Proportion	2017	34.3	52.2	20.5
Algeria	Annaba	Count	2000	0	0	0
Algeria	Annaba	Count	2017	0	1	0
Algeria	Ain Touta	Proportion	2000	31.9	46.9	19.3
Algeria	Ain Touta	Proportion	2017	37.6	52.1	23.1
Algeria	Ain Touta	Count	2000	0	0	0
Algeria	Ain Touta	Count	2017	0	1	0
Algeria	Bechar	Proportion	2000	32.4	48.4	19.7
Algeria	Bechar	Proportion	2017	33.7	49.9	21.4
Algeria	Bechar	Count	2000	0	0	0
Algeria	Bechar	Count	2017	0	0	0
Algeria	Ait-Smail	Proportion	2000	30.7	44.5	18.3
Algeria	Ait-Smail	Proportion	2017	37.0	53.6	24.0
Algeria	Ait-Smail	Count	2000	0	0	0
Algeria	Ait-Smail	Count	2017	0	1	0
Algeria	Ain Zaatout	Proportion	2000	31.0	45.2	18.4
Algeria	Ain Zaatout	Proportion	2017	34.6	49.4	20.9
Algeria	Ain Zaatout	Count	2000	0	0	0
Algeria	Ain Zaatout	Count	2017	0	0	0
Algeria	Beni-Tamou	Proportion	2000	30.6	45.3	19.1
Algeria	Beni-Tamou	Proportion	2017	34.0	51.0	19.8
Algeria	Beni-Tamou	Count	2000	0	0	0
Algeria	Beni-Tamou	Count	2017	0	1	0
Algeria	Ain Tesra	Proportion	2000	33.6	48.2	20.7
Algeria	Ain Tesra	Proportion	2017	37.0	53.6	23.0
Algeria	Ain Tesra	Count	2000	0	0	0
Algeria	Ain Tesra	Count	2017	0	1	0
Algeria	Ahl El Ksar	Proportion	2000	32.4	48.2	19.8
Algeria	Ahl El Ksar	Proportion	2017	37.0	52.9	23.6
Algeria	Ahl El Ksar	Count	2000	0	0	0
Algeria	Ahl El Ksar	Count	2017	0	1	0
Algeria	Ammal	Proportion	2000	30.5	48.9	17.9
Algeria	Ammal	Proportion	2017	35.5	49.7	22.0
Algeria	Ammal	Count	2000	0	0	0
Algeria	Ammal	Count	2017	0	0	0
Algeria	Ain Merrane	Proportion	2000	28.2	43.2	15.7
Algeria	Ain Merrane	Proportion	2017	34.3	52.3	18.9
Algeria	Ain Merrane	Count	2000	0	0	0
Algeria	Ain Merrane	Count	2017	0	1	0
Algeria	Ain Semara	Proportion	2000	33.9	48.2	20.7
Algeria	Ain Semara	Proportion	2017	36.6	54.9	22.6
Algeria	Ain Semara	Count	2000	0	0	0
Algeria	Ain Semara	Count	2017	0	1	0
Algeria	Ain El Ibel	Proportion	2000	31.0	47.8	17.2
Algeria	Ain El Ibel	Proportion	2017	32.0	46.8	20.6
Algeria	Ain El Ibel	Count	2000	0	0	0
Algeria	Ain El Ibel	Count	2017	0	0	0

Table 4: ORS by Unit *(continued)*

Country	Unit	Metric	Year	Mean	Upper	Lower
Algeria	Arbaouat	Proportion	2000	31.3	46.1	17.5
Algeria	Arbaouat	Proportion	2017	34.2	48.6	19.9
Algeria	Arbaouat	Count	2000	0	0	0
Algeria	Arbaouat	Count	2017	0	0	0
Algeria	Ben Guecha	Proportion	2000	29.2	42.5	17.3
Algeria	Ben Guecha	Proportion	2017	32.3	47.8	18.5
Algeria	Ben Guecha	Count	2000	0	0	0
Algeria	Ben Guecha	Count	2017	0	0	0
Algeria	Ain Kerma	Proportion	2000	34.3	49.0	22.0
Algeria	Ain Kerma	Proportion	2017	37.8	53.7	23.7
Algeria	Ain Kerma	Count	2000	0	0	0
Algeria	Ain Kerma	Count	2017	0	1	0
Algeria	Bounoura	Proportion	2000	29.9	46.8	17.2
Algeria	Bounoura	Proportion	2017	30.4	45.2	17.5
Algeria	Bounoura	Count	2000	0	0	0
Algeria	Bounoura	Count	2017	0	0	0
Algeria	Ain Larbi	Proportion	2000	33.6	48.8	21.5
Algeria	Ain Larbi	Proportion	2017	36.1	50.5	23.2
Algeria	Ain Larbi	Count	2000	0	0	0
Algeria	Ain Larbi	Count	2017	0	1	0
Algeria	Bordj Omar Driss	Proportion	2000	31.6	42.3	22.7
Algeria	Bordj Omar Driss	Proportion	2017	33.3	42.4	23.8
Algeria	Bordj Omar Driss	Count	2000	0	0	0
Algeria	Bordj Omar Driss	Count	2017	0	0	0
Algeria	Boudria Beniyadjis	Proportion	2000	32.8	46.2	20.1
Algeria	Boudria Beniyadjis	Proportion	2017	37.6	54.9	23.7
Algeria	Boudria Beniyadjis	Count	2000	0	0	0
Algeria	Boudria Beniyadjis	Count	2017	0	1	0
Algeria	Babar	Proportion	2000	30.2	43.5	19.1
Algeria	Babar	Proportion	2017	34.9	48.2	21.7
Algeria	Babar	Count	2000	0	0	0
Algeria	Babar	Count	2017	0	0	0
Algeria	Ain Madhi	Proportion	2000	31.1	47.6	18.8
Algeria	Ain Madhi	Proportion	2017	33.2	47.2	21.6
Algeria	Ain Madhi	Count	2000	0	0	0
Algeria	Ain Madhi	Count	2017	0	0	0
Algeria	Ain El Melh	Proportion	2000	31.6	46.3	19.1
Algeria	Ain El Melh	Proportion	2017	34.0	50.3	20.8
Algeria	Ain El Melh	Count	2000	0	0	0
Algeria	Ain El Melh	Count	2017	0	1	0
Algeria	Ain Fekan	Proportion	2000	28.4	40.8	16.7
Algeria	Ain Fekan	Proportion	2017	34.7	50.9	21.1
Algeria	Ain Fekan	Count	2000	0	0	0
Algeria	Ain Fekan	Count	2017	0	1	0
Algeria	Ain Ouksir	Proportion	2000	34.4	50.8	19.7
Algeria	Ain Ouksir	Proportion	2017	36.1	51.5	22.2
Algeria	Ain Ouksir	Count	2000	0	1	0
Algeria	Ain Ouksir	Count	2017	0	1	0
Algeria	Ain Beida Harriche	Proportion	2000	33.9	48.7	20.5

Table 4: ORS by Unit *(continued)*

Country	Unit	Metric	Year	Mean	Upper	Lower
Algeria	Ain Beida Harriche	Proportion	2017	38.3	54.5	22.5
Algeria	Ain Beida Harriche	Count	2000	0	0	0
Algeria	Ain Beida Harriche	Count	2017	0	1	0
Algeria	Ain Boudinar	Proportion	2000	25.7	39.5	15.1
Algeria	Ain Boudinar	Proportion	2017	35.2	50.3	21.1
Algeria	Ain Boudinar	Count	2000	0	0	0
Algeria	Ain Boudinar	Count	2017	0	1	0
Algeria	Ain Safra	Proportion	2000	33.1	47.1	21.9
Algeria	Ain Safra	Proportion	2017	35.3	50.6	20.0
Algeria	Ain Safra	Count	2000	0	0	0
Algeria	Ain Safra	Count	2017	0	1	0
Algeria	Ain Kerma	Proportion	2000	27.3	39.5	16.2
Algeria	Ain Kerma	Proportion	2017	33.8	48.0	20.8
Algeria	Ain Kerma	Count	2000	0	0	0
Algeria	Ain Kerma	Count	2017	0	0	0
Algeria	Baladiet Amor	Proportion	2000	29.6	46.0	17.7
Algeria	Baladiet Amor	Proportion	2017	30.5	44.4	17.8
Algeria	Baladiet Amor	Count	2000	0	0	0
Algeria	Baladiet Amor	Count	2017	0	0	0
Algeria	Ain Beida	Proportion	2000	31.4	46.5	19.4
Algeria	Ain Beida	Proportion	2017	36.9	51.8	22.5
Algeria	Ain Beida	Count	2000	0	0	0
Algeria	Ain Beida	Count	2017	0	1	0
Algeria	Ain Tarek	Proportion	2000	30.1	46.8	17.1
Algeria	Ain Tarek	Proportion	2017	35.6	51.7	21.6
Algeria	Ain Tarek	Count	2000	0	0	0
Algeria	Ain Tarek	Count	2017	0	1	0
Algeria	Ain Sekhouna	Proportion	2000	33.1	48.9	20.4
Algeria	Ain Sekhouna	Proportion	2017	37.7	54.8	23.3
Algeria	Ain Sekhouna	Count	2000	0	0	0
Algeria	Ain Sekhouna	Count	2017	0	1	0
Algeria	Ain-Sebt	Proportion	2000	32.9	47.1	20.0
Algeria	Ain-Sebt	Proportion	2017	37.0	53.7	22.1
Algeria	Ain-Sebt	Count	2000	0	0	0
Algeria	Ain-Sebt	Count	2017	0	1	0
Algeria	Ain El Berd	Proportion	2000	27.2	39.8	15.8
Algeria	Ain El Berd	Proportion	2017	35.2	50.7	20.2
Algeria	Ain El Berd	Count	2000	0	0	0
Algeria	Ain El Berd	Count	2017	0	1	0
Algeria	Ain Charchar	Proportion	2000	34.3	50.7	22.0
Algeria	Ain Charchar	Proportion	2017	37.5	55.3	24.3
Algeria	Ain Charchar	Count	2000	0	1	0
Algeria	Ain Charchar	Count	2017	0	1	0
Algeria	Ain Zana	Proportion	2000	33.9	49.5	21.7
Algeria	Ain Zana	Proportion	2017	36.5	54.3	23.7
Algeria	Ain Zana	Count	2000	0	0	0
Algeria	Ain Zana	Count	2017	0	1	0
Algeria	Foggaret Azzouia	Proportion	2000	31.6	41.5	20.7
Algeria	Foggaret Azzouia	Proportion	2017	31.6	42.2	22.8

Table 4: ORS by Unit *(continued)*

Country	Unit	Metric	Year	Mean	Upper	Lower
Algeria	Foggaret Azzouia	Count	2000	0	0	0
Algeria	Foggaret Azzouia	Count	2017	0	0	0
Algeria	Bedjene	Proportion	2000	30.8	48.4	18.0
Algeria	Bedjene	Proportion	2017	35.4	52.6	21.5
Algeria	Bedjene	Count	2000	0	0	0
Algeria	Bedjene	Count	2017	0	1	0
Algeria	Ain Deheb	Proportion	2000	31.8	47.3	19.2
Algeria	Ain Deheb	Proportion	2017	36.9	53.8	22.5
Algeria	Ain Deheb	Count	2000	0	0	0
Algeria	Ain Deheb	Count	2017	0	1	0
Algeria	Tindouf	Proportion	2000	30.9	43.5	20.4
Algeria	Tindouf	Proportion	2017	30.5	42.3	20.6
Algeria	Tindouf	Count	2000	0	0	0
Algeria	Tindouf	Count	2017	0	0	0
Algeria	Ahmer El Ain	Proportion	2000	31.1	45.6	18.9
Algeria	Ahmer El Ain	Proportion	2017	34.1	51.8	19.7
Algeria	Ahmer El Ain	Count	2000	0	0	0
Algeria	Ahmer El Ain	Count	2017	0	1	0
Algeria	Beni Chaib	Proportion	2000	33.2	47.9	20.1
Algeria	Beni Chaib	Proportion	2017	35.0	50.2	20.1
Algeria	Beni Chaib	Count	2000	0	0	0
Algeria	Beni Chaib	Count	2017	0	1	0
Algeria	Aghni-Goughrane	Proportion	2000	30.0	47.2	17.0
Algeria	Aghni-Goughrane	Proportion	2017	35.4	50.7	21.6
Algeria	Aghni-Goughrane	Count	2000	0	0	0
Algeria	Aghni-Goughrane	Count	2017	0	1	0
Algeria	Ain Fezza	Proportion	2000	30.1	45.4	17.8
Algeria	Ain Fezza	Proportion	2017	35.2	50.8	22.0
Algeria	Ain Fezza	Count	2000	0	0	0
Algeria	Ain Fezza	Count	2017	0	1	0
Algeria	Aougrout	Proportion	2000	29.6	42.6	18.9
Algeria	Aougrout	Proportion	2017	30.8	44.6	19.6
Algeria	Aougrout	Count	2000	0	0	0
Algeria	Aougrout	Count	2017	0	0	0
Algeria	Ain Defla	Proportion	2000	31.2	46.0	19.2
Algeria	Ain Defla	Proportion	2017	33.2	48.6	18.9
Algeria	Ain Defla	Count	2000	0	0	0
Algeria	Ain Defla	Count	2017	0	0	0
Algeria	Ain Kihel	Proportion	2000	29.0	43.3	17.7
Algeria	Ain Kihel	Proportion	2017	34.5	49.5	21.6
Algeria	Ain Kihel	Count	2000	0	0	0
Algeria	Ain Kihel	Count	2017	0	0	0
Algeria	Birtouta	Proportion	2000	30.9	46.0	19.9
Algeria	Birtouta	Proportion	2017	35.1	49.5	21.1
Algeria	Birtouta	Count	2000	0	0	0
Algeria	Birtouta	Count	2017	0	0	0
Algeria	Berrahal	Proportion	2000	33.3	48.4	19.9
Algeria	Berrahal	Proportion	2017	36.5	53.3	23.3
Algeria	Berrahal	Count	2000	0	0	0

Table 4: ORS by Unit *(continued)*

Country	Unit	Metric	Year	Mean	Upper	Lower
Algeria	Berrahal	Count	2017	0	1	0
Algeria	Ain Yagout	Proportion	2000	32.8	46.8	19.0
Algeria	Ain Yagout	Proportion	2017	37.5	54.2	23.1
Algeria	Ain Yagout	Count	2000	0	0	0
Algeria	Ain Yagout	Count	2017	0	1	0
Algeria	Beni Abbes	Proportion	2000	32.1	46.8	20.2
Algeria	Beni Abbes	Proportion	2017	33.1	46.7	19.1
Algeria	Beni Abbes	Count	2000	0	0	0
Algeria	Beni Abbes	Count	2017	0	0	0
Algeria	Ait R'Zine	Proportion	2000	31.6	48.3	19.2
Algeria	Ait R'Zine	Proportion	2017	38.0	53.9	23.3
Algeria	Ait R'Zine	Count	2000	0	0	0
Algeria	Ait R'Zine	Count	2017	0	1	0
Algeria	Besbes	Proportion	2000	30.0	42.9	18.9
Algeria	Besbes	Proportion	2017	30.8	45.5	18.6
Algeria	Besbes	Count	2000	0	0	0
Algeria	Besbes	Count	2017	0	0	0
Algeria	Beni Merad	Proportion	2000	30.7	45.3	19.2
Algeria	Beni Merad	Proportion	2017	31.9	49.5	18.1
Algeria	Beni Merad	Count	2000	0	0	0
Algeria	Beni Merad	Count	2017	0	0	0
Algeria	Belimour	Proportion	2000	33.2	48.7	21.6
Algeria	Belimour	Proportion	2017	37.0	53.4	22.6
Algeria	Belimour	Count	2000	0	0	0
Algeria	Belimour	Count	2017	0	1	0
Algeria	Ahnif	Proportion	2000	32.5	48.6	18.6
Algeria	Ahnif	Proportion	2017	37.4	53.0	23.8
Algeria	Ahnif	Count	2000	0	0	0
Algeria	Ahnif	Count	2017	0	1	0
Algeria	Baghlia	Proportion	2000	29.1	46.9	17.0
Algeria	Baghlia	Proportion	2017	35.5	50.0	21.3
Algeria	Baghlia	Count	2000	0	0	0
Algeria	Baghlia	Count	2017	0	0	0
Algeria	Benairia	Proportion	2000	29.1	44.6	17.2
Algeria	Benairia	Proportion	2017	33.3	50.8	19.0
Algeria	Benairia	Count	2000	0	0	0
Algeria	Benairia	Count	2017	0	1	0
Algeria	Ben Badis	Proportion	2000	35.0	49.6	20.9
Algeria	Ben Badis	Proportion	2017	37.0	53.3	22.8
Algeria	Ben Badis	Count	2000	0	0	0
Algeria	Ben Badis	Count	2017	0	1	0
Algeria	Ain Fekka	Proportion	2000	31.4	47.6	18.1
Algeria	Ain Fekka	Proportion	2017	32.8	45.4	20.6
Algeria	Ain Fekka	Count	2000	0	0	0
Algeria	Ain Fekka	Count	2017	0	0	0
Algeria	Boualem	Proportion	2000	30.4	44.1	17.3
Algeria	Boualem	Proportion	2017	33.0	46.0	20.9
Algeria	Boualem	Count	2000	0	0	0
Algeria	Boualem	Count	2017	0	0	0

Table 4: ORS by Unit *(continued)*

Country	Unit	Metric	Year	Mean	Upper	Lower
Algeria	Debila	Proportion	2000	30.2	46.1	17.2
Algeria	Debila	Proportion	2017	29.1	44.2	16.7
Algeria	Debila	Count	2000	0	0	0
Algeria	Debila	Count	2017	0	0	0
Algeria	Asfour	Proportion	2000	33.3	49.1	20.8
Algeria	Asfour	Proportion	2017	36.9	52.9	23.8
Algeria	Asfour	Count	2000	0	0	0
Algeria	Asfour	Count	2017	0	1	0
Algeria	Dhayet Bendhahoua	Proportion	2000	30.4	47.0	18.9
Algeria	Dhayet Bendhahoua	Proportion	2017	32.2	47.1	19.5
Algeria	Dhayet Bendhahoua	Count	2000	0	0	0
Algeria	Dhayet Bendhahoua	Count	2017	0	0	0
Algeria	Ain Makhlouf	Proportion	2000	34.4	49.9	21.8
Algeria	Ain Makhlouf	Proportion	2017	36.7	51.3	23.3
Algeria	Ain Makhlouf	Count	2000	0	0	0
Algeria	Ain Makhlouf	Count	2017	0	1	0
Algeria	Debdeb	Proportion	2000	28.6	41.9	16.7
Algeria	Debdeb	Proportion	2017	30.3	43.2	19.9
Algeria	Debdeb	Count	2000	0	0	0
Algeria	Debdeb	Count	2017	0	0	0
Algeria	Bouraoui Belhadef	Proportion	2000	32.1	45.5	19.4
Algeria	Bouraoui Belhadef	Proportion	2017	37.2	53.4	23.5
Algeria	Bouraoui Belhadef	Count	2000	0	0	0
Algeria	Bouraoui Belhadef	Count	2017	0	1	0
Algeria	Baghai	Proportion	2000	30.6	46.5	18.3
Algeria	Baghai	Proportion	2017	34.7	48.1	19.6
Algeria	Baghai	Count	2000	0	0	0
Algeria	Baghai	Count	2017	0	0	0
Algeria	Ain Sidi Ali	Proportion	2000	30.1	45.5	17.7
Algeria	Ain Sidi Ali	Proportion	2017	32.6	46.4	21.0
Algeria	Ain Sidi Ali	Count	2000	0	0	0
Algeria	Ain Sidi Ali	Count	2017	0	0	0
Algeria	Ain Errich	Proportion	2000	31.6	45.6	19.0
Algeria	Ain Errich	Proportion	2017	33.6	48.5	20.4
Algeria	Ain Errich	Count	2000	0	0	0
Algeria	Ain Errich	Count	2017	0	0	0
Algeria	Ain Ferah	Proportion	2000	31.3	48.9	19.2
Algeria	Ain Ferah	Proportion	2017	35.9	53.2	21.4
Algeria	Ain Ferah	Count	2000	0	0	0
Algeria	Ain Ferah	Count	2017	0	1	0
Algeria	Aissaouia	Proportion	2000	32.8	48.5	20.4
Algeria	Aissaouia	Proportion	2017	34.7	49.1	22.2
Algeria	Aissaouia	Count	2000	0	0	0
Algeria	Aissaouia	Count	2017	0	0	0
Algeria	Ain Mellouk	Proportion	2000	33.8	47.7	20.7
Algeria	Ain Mellouk	Proportion	2017	36.4	51.4	21.3
Algeria	Ain Mellouk	Count	2000	0	0	0
Algeria	Ain Mellouk	Count	2017	0	1	0
Algeria	Ain Nouissy	Proportion	2000	25.9	39.9	15.9

Table 4: ORS by Unit *(continued)*

Country	Unit	Metric	Year	Mean	Upper	Lower
Algeria	Ain Nouissy	Proportion	2017	35.5	52.1	20.8
Algeria	Ain Nouissy	Count	2000	0	0	0
Algeria	Ain Nouissy	Count	2017	0	1	0
Algeria	Assela	Proportion	2000	32.5	44.5	21.5
Algeria	Assela	Proportion	2017	34.6	53.1	22.3
Algeria	Assela	Count	2000	0	0	0
Algeria	Assela	Count	2017	0	1	0
Algeria	Ain Turk	Proportion	2000	27.7	41.9	15.4
Algeria	Ain Turk	Proportion	2017	35.1	50.8	21.5
Algeria	Ain Turk	Count	2000	0	0	0
Algeria	Ain Turk	Count	2017	0	1	0
Algeria	Benaceur	Proportion	2000	30.0	45.0	17.9
Algeria	Benaceur	Proportion	2017	30.3	43.3	19.6
Algeria	Benaceur	Count	2000	0	0	0
Algeria	Benaceur	Count	2017	0	0	0
Algeria	Ain El Diss	Proportion	2000	33.0	48.5	19.9
Algeria	Ain El Diss	Proportion	2017	36.2	51.9	23.0
Algeria	Ain El Diss	Count	2000	0	0	0
Algeria	Ain El Diss	Count	2017	0	1	0
Algeria	Ammi Moussa	Proportion	2000	29.2	45.3	16.4
Algeria	Ammi Moussa	Proportion	2017	35.7	51.9	21.5
Algeria	Ammi Moussa	Count	2000	0	0	0
Algeria	Ammi Moussa	Count	2017	0	1	0
Algeria	Ain Soltane	Proportion	2000	30.6	45.3	18.6
Algeria	Ain Soltane	Proportion	2017	35.7	53.7	22.2
Algeria	Ain Soltane	Count	2000	0	0	0
Algeria	Ain Soltane	Count	2017	0	1	0
Algeria	Ain Abessa	Proportion	2000	33.4	46.1	20.4
Algeria	Ain Abessa	Proportion	2017	37.1	53.6	21.9
Algeria	Ain Abessa	Count	2000	0	0	0
Algeria	Ain Abessa	Count	2017	0	1	0
Algeria	Ain Kada	Proportion	2000	28.5	43.3	16.3
Algeria	Ain Kada	Proportion	2017	35.0	50.3	21.4
Algeria	Ain Kada	Count	2000	0	0	0
Algeria	Ain Kada	Count	2017	0	1	0
Algeria	Ain Kechra	Proportion	2000	32.9	47.1	20.5
Algeria	Ain Kechra	Proportion	2017	38.5	53.6	23.6
Algeria	Ain Kechra	Count	2000	0	0	0
Algeria	Ain Kechra	Count	2017	0	1	0
Algeria	Bir Bouhouche	Proportion	2000	33.1	48.3	21.0
Algeria	Bir Bouhouche	Proportion	2017	36.5	52.2	23.8
Algeria	Bir Bouhouche	Count	2000	0	0	0
Algeria	Bir Bouhouche	Count	2017	0	1	0
Algeria	Idles	Proportion	2000	29.4	40.2	20.5
Algeria	Idles	Proportion	2017	30.0	39.9	21.9
Algeria	Idles	Count	2000	0	0	0
Algeria	Idles	Count	2017	0	0	0
Algeria	Bekkaria	Proportion	2000	30.0	45.4	16.9
Algeria	Bekkaria	Proportion	2017	35.0	50.1	21.1

Table 4: ORS by Unit *(continued)*

Country	Unit	Metric	Year	Mean	Upper	Lower
Algeria	Bekkaria	Count	2000	0	0	0
Algeria	Bekkaria	Count	2017	0	1	0
Algeria	Ain El Hadid	Proportion	2000	30.2	46.8	17.4
Algeria	Ain El Hadid	Proportion	2017	35.7	54.3	23.2
Algeria	Ain El Hadid	Count	2000	0	0	0
Algeria	Ain El Hadid	Count	2017	0	1	0
Algeria	Ain Tagourait	Proportion	2000	32.0	48.3	19.7
Algeria	Ain Tagourait	Proportion	2017	33.3	50.9	19.2
Algeria	Ain Tagourait	Count	2000	0	0	0
Algeria	Ain Tagourait	Count	2017	0	1	0
Algeria	Beni Lahcene	Proportion	2000	32.8	48.0	19.6
Algeria	Beni Lahcene	Proportion	2017	34.6	49.7	19.6
Algeria	Beni Lahcene	Count	2000	0	0	0
Algeria	Beni Lahcene	Count	2017	0	0	0
Algeria	Aghrib	Proportion	2000	28.2	44.8	17.0
Algeria	Aghrib	Proportion	2017	34.4	48.1	21.3
Algeria	Aghrib	Count	2000	0	0	0
Algeria	Aghrib	Count	2017	0	0	0
Algeria	Ain Ghoraba	Proportion	2000	30.0	46.8	17.6
Algeria	Ain Ghoraba	Proportion	2017	34.5	50.1	20.6
Algeria	Ain Ghoraba	Count	2000	0	0	0
Algeria	Ain Ghoraba	Count	2017	0	1	0
Algeria	Aoulef	Proportion	2000	28.3	42.8	18.3
Algeria	Aoulef	Proportion	2017	30.7	45.5	18.5
Algeria	Aoulef	Count	2000	0	0	0
Algeria	Aoulef	Count	2017	0	0	0
Algeria	Ain Lechiakh	Proportion	2000	32.8	48.3	21.3
Algeria	Ain Lechiakh	Proportion	2017	33.8	47.8	20.6
Algeria	Ain Lechiakh	Count	2000	0	0	0
Algeria	Ain Lechiakh	Count	2017	0	0	0
Algeria	Ain Temouchent	Proportion	2000	29.1	43.6	17.4
Algeria	Ain Temouchent	Proportion	2017	35.0	49.9	21.3
Algeria	Ain Temouchent	Count	2000	0	0	0
Algeria	Ain Temouchent	Count	2017	0	0	0
Algeria	Bordj El Kiffan	Proportion	2000	30.2	46.1	18.5
Algeria	Bordj El Kiffan	Proportion	2017	32.4	48.1	19.6
Algeria	Bordj El Kiffan	Count	2000	0	0	0
Algeria	Bordj El Kiffan	Count	2017	0	0	0
Algeria	Chetaibi	Proportion	2000	32.5	49.1	20.4
Algeria	Chetaibi	Proportion	2017	36.1	50.2	23.1
Algeria	Chetaibi	Count	2000	0	0	0
Algeria	Chetaibi	Count	2017	0	1	0
Algeria	Arris	Proportion	2000	30.4	45.0	17.3
Algeria	Arris	Proportion	2017	35.8	52.3	21.2
Algeria	Arris	Count	2000	0	0	0
Algeria	Arris	Count	2017	0	1	0
Algeria	Beni Ikhlef	Proportion	2000	32.4	46.9	20.3
Algeria	Beni Ikhlef	Proportion	2017	33.1	46.3	20.3
Algeria	Beni Ikhlef	Count	2000	0	0	0

Table 4: ORS by Unit *(continued)*

Country	Unit	Metric	Year	Mean	Upper	Lower
Algeria	Beni Ikhlef	Count	2017	0	0	0
Algeria	Akbou	Proportion	2000	30.8	47.6	18.7
Algeria	Akbou	Proportion	2017	38.2	54.0	23.6
Algeria	Akbou	Count	2000	0	0	0
Algeria	Akbou	Count	2017	0	1	0
Algeria	Biskra	Proportion	2000	29.9	42.8	17.7
Algeria	Biskra	Proportion	2017	30.9	44.9	18.9
Algeria	Biskra	Count	2000	0	0	0
Algeria	Biskra	Count	2017	0	0	0
Algeria	Benkhelil	Proportion	2000	31.0	45.7	19.1
Algeria	Benkhelil	Proportion	2017	34.0	49.8	19.9
Algeria	Benkhelil	Count	2000	0	0	0
Algeria	Benkhelil	Count	2017	0	0	0
Algeria	Bendaoud	Proportion	2000	32.9	48.4	19.0
Algeria	Bendaoud	Proportion	2017	35.0	50.7	20.5
Algeria	Bendaoud	Count	2000	0	0	0
Algeria	Bendaoud	Count	2017	0	1	0
Algeria	Ain-Bessem	Proportion	2000	32.2	48.6	19.6
Algeria	Ain-Bessem	Proportion	2017	36.0	51.6	23.0
Algeria	Ain-Bessem	Count	2000	0	0	0
Algeria	Ain-Bessem	Count	2017	0	1	0
Algeria	Ben Choud	Proportion	2000	29.2	47.0	17.1
Algeria	Ben Choud	Proportion	2017	35.1	49.2	20.5
Algeria	Ben Choud	Count	2000	0	0	0
Algeria	Ben Choud	Count	2017	0	0	0
Algeria	Beni Bouattab	Proportion	2000	30.8	45.5	18.3
Algeria	Beni Bouattab	Proportion	2017	34.8	50.1	20.2
Algeria	Beni Bouattab	Count	2000	0	0	0
Algeria	Beni Bouattab	Count	2017	0	1	0
Algeria	Beni Hamidane	Proportion	2000	34.4	49.2	21.6
Algeria	Beni Hamidane	Proportion	2017	38.6	54.9	23.7
Algeria	Beni Hamidane	Count	2000	0	0	0
Algeria	Beni Hamidane	Count	2017	0	1	0
Algeria	Ain Maabed	Proportion	2000	31.1	44.9	17.0
Algeria	Ain Maabed	Proportion	2017	32.8	47.5	20.0
Algeria	Ain Maabed	Count	2000	0	0	0
Algeria	Ain Maabed	Count	2017	0	0	0
Algeria	Bougtoub	Proportion	2000	30.9	43.1	18.1
Algeria	Bougtoub	Proportion	2017	34.5	50.3	20.4
Algeria	Bougtoub	Count	2000	0	0	0
Algeria	Bougtoub	Count	2017	0	1	0
Algeria	Djamaa	Proportion	2000	30.2	46.3	18.0
Algeria	Djamaa	Proportion	2017	30.8	45.1	18.8
Algeria	Djamaa	Count	2000	0	0	0
Algeria	Djamaa	Count	2017	0	0	0
Algeria	Ben M'Hidi	Proportion	2000	33.1	48.0	19.9
Algeria	Ben M'Hidi	Proportion	2017	36.4	52.2	23.0
Algeria	Ben M'Hidi	Count	2000	0	0	0
Algeria	Ben M'Hidi	Count	2017	0	1	0

Table 4: ORS by Unit *(continued)*

Country	Unit	Metric	Year	Mean	Upper	Lower
Algeria	El Atteuf	Proportion	2000	30.2	46.0	17.8
Algeria	El Atteuf	Proportion	2017	30.6	45.3	17.6
Algeria	El Atteuf	Count	2000	0	0	0
Algeria	El Atteuf	Count	2017	0	0	0
Algeria	Ain Rekada	Proportion	2000	34.7	51.3	20.7
Algeria	Ain Rekada	Proportion	2017	36.9	52.8	22.8
Algeria	Ain Rekada	Count	2000	0	1	0
Algeria	Ain Rekada	Count	2017	0	1	0
Algeria	Djanet	Proportion	2000	28.6	41.5	18.3
Algeria	Djanet	Proportion	2017	30.4	41.6	21.3
Algeria	Djanet	Count	2000	0	0	0
Algeria	Djanet	Count	2017	0	0	0
Algeria	Boussif Ouled Askeur	Proportion	2000	32.5	45.5	20.3
Algeria	Boussif Ouled Askeur	Proportion	2017	36.9	53.6	23.3
Algeria	Boussif Ouled Askeur	Count	2000	0	0	0
Algeria	Boussif Ouled Askeur	Count	2017	0	1	0
Algeria	Bouhmama	Proportion	2000	30.7	46.1	19.3
Algeria	Bouhmama	Proportion	2017	35.9	51.6	21.1
Algeria	Bouhmama	Count	2000	0	0	0
Algeria	Bouhmama	Count	2017	0	1	0
Algeria	Brida	Proportion	2000	29.8	45.5	16.3
Algeria	Brida	Proportion	2017	32.2	46.0	20.5
Algeria	Brida	Count	2000	0	0	0
Algeria	Brida	Count	2017	0	0	0
Algeria	Ain Fares	Proportion	2000	30.7	45.4	17.7
Algeria	Ain Fares	Proportion	2017	33.3	49.2	19.6
Algeria	Ain Fares	Count	2000	0	0	0
Algeria	Ain Fares	Count	2017	0	0	0
Algeria	Ain Frass	Proportion	2000	27.7	42.8	15.4
Algeria	Ain Frass	Proportion	2017	34.7	49.7	20.5
Algeria	Ain Frass	Count	2000	0	0	0
Algeria	Ain Frass	Count	2017	0	0	0
Algeria	Azziz	Proportion	2000	33.3	50.6	21.8
Algeria	Azziz	Proportion	2017	35.9	49.9	23.0
Algeria	Azziz	Count	2000	0	1	0
Algeria	Azziz	Count	2017	0	0	0
Algeria	Ain Tine	Proportion	2000	33.9	47.1	21.1
Algeria	Ain Tine	Proportion	2017	37.4	54.7	23.1
Algeria	Ain Tine	Count	2000	0	0	0
Algeria	Ain Tine	Count	2017	0	1	0
Algeria	Ain Sidi Cherif	Proportion	2000	25.6	38.4	15.4
Algeria	Ain Sidi Cherif	Proportion	2017	34.9	50.7	20.4
Algeria	Ain Sidi Cherif	Count	2000	0	0	0
Algeria	Ain Sidi Cherif	Count	2017	0	1	0
Algeria	Djeniane Bourzeg	Proportion	2000	32.3	47.6	20.7
Algeria	Djeniane Bourzeg	Proportion	2017	34.5	51.1	21.4
Algeria	Djeniane Bourzeg	Count	2000	0	0	0
Algeria	Djeniane Bourzeg	Count	2017	0	1	0
Algeria	Arzew	Proportion	2000	25.6	37.6	15.3

Table 4: ORS by Unit *(continued)*

Country	Unit	Metric	Year	Mean	Upper	Lower
Algeria	Arzew	Proportion	2017	33.1	48.0	18.6
Algeria	Arzew	Count	2000	0	0	0
Algeria	Arzew	Count	2017	0	0	0
Algeria	El Allia	Proportion	2000	29.9	44.5	18.8
Algeria	El Allia	Proportion	2017	30.5	44.9	19.3
Algeria	El Allia	Count	2000	0	0	0
Algeria	El Allia	Count	2017	0	0	0
Algeria	Ain El Fakroun	Proportion	2000	33.1	48.8	19.7
Algeria	Ain El Fakroun	Proportion	2017	36.7	51.7	22.7
Algeria	Ain El Fakroun	Count	2000	0	0	0
Algeria	Ain El Fakroun	Count	2017	0	1	0
Algeria	Belaassel Bouzagza	Proportion	2000	26.2	41.0	15.5
Algeria	Belaassel Bouzagza	Proportion	2017	35.4	52.0	20.4
Algeria	Belaassel Bouzagza	Count	2000	0	0	0
Algeria	Belaassel Bouzagza	Count	2017	0	1	0
Algeria	Doui Thabet	Proportion	2000	30.9	47.1	19.6
Algeria	Doui Thabet	Proportion	2017	35.4	51.3	20.9
Algeria	Doui Thabet	Count	2000	0	0	0
Algeria	Doui Thabet	Count	2017	0	1	0
Algeria	Ain Arnat	Proportion	2000	33.4	46.9	20.7
Algeria	Ain Arnat	Proportion	2017	35.8	53.4	20.8
Algeria	Ain Arnat	Count	2000	0	0	0
Algeria	Ain Arnat	Count	2017	0	1	0
Algeria	Ain Thrid	Proportion	2000	28.5	43.1	15.9
Algeria	Ain Thrid	Proportion	2017	35.8	53.1	19.1
Algeria	Ain Thrid	Count	2000	0	0	0
Algeria	Ain Thrid	Count	2017	0	1	0
Algeria	Ain Zouit	Proportion	2000	32.7	49.4	19.8
Algeria	Ain Zouit	Proportion	2017	37.7	53.4	23.1
Algeria	Ain Zouit	Count	2000	0	0	0
Algeria	Ain Zouit	Count	2017	0	1	0
Algeria	Drea	Proportion	2000	33.2	48.3	20.1
Algeria	Drea	Proportion	2017	36.2	52.8	23.6
Algeria	Drea	Count	2000	0	0	0
Algeria	Drea	Count	2017	0	1	0
Algeria	In Ghar	Proportion	2000	29.6	41.2	20.5
Algeria	In Ghar	Proportion	2017	30.5	43.0	19.8
Algeria	In Ghar	Count	2000	0	0	0
Algeria	In Ghar	Count	2017	0	0	0
Algeria	Bir Dheb	Proportion	2000	31.1	45.5	17.6
Algeria	Bir Dheb	Proportion	2017	36.3	51.1	24.1
Algeria	Bir Dheb	Count	2000	0	0	0
Algeria	Bir Dheb	Count	2017	0	1	0
Algeria	Ain Kermes	Proportion	2000	30.5	47.2	18.7
Algeria	Ain Kermes	Proportion	2017	35.5	51.9	22.1
Algeria	Ain Kermes	Count	2000	0	0	0
Algeria	Ain Kermes	Count	2017	0	1	0
Algeria	Beni Mileuk	Proportion	2000	30.8	45.9	19.3
Algeria	Beni Mileuk	Proportion	2017	33.5	47.4	19.9

Table 4: ORS by Unit *(continued)*

Country	Unit	Metric	Year	Mean	Upper	Lower
Algeria	Beni Mileuk	Count	2000	0	0	0
Algeria	Beni Mileuk	Count	2017	0	0	0
Algeria	Bordj Bounaama	Proportion	2000	31.0	45.4	18.6
Algeria	Bordj Bounaama	Proportion	2017	34.4	50.1	19.6
Algeria	Bordj Bounaama	Count	2000	0	0	0
Algeria	Bordj Bounaama	Count	2017	0	1	0
Algeria	Ain-El-Hammam	Proportion	2000	28.4	45.4	17.1
Algeria	Ain-El-Hammam	Proportion	2017	35.7	50.1	23.0
Algeria	Ain-El-Hammam	Count	2000	0	0	0
Algeria	Ain-El-Hammam	Count	2017	0	1	0
Algeria	Ain Kebira	Proportion	2000	30.1	47.3	18.6
Algeria	Ain Kebira	Proportion	2017	35.7	51.3	20.6
Algeria	Ain Kebira	Count	2000	0	0	0
Algeria	Ain Kebira	Count	2017	0	1	0
Algeria	Bordj Badji Mokhtar	Proportion	2000	30.0	38.1	22.9
Algeria	Bordj Badji Mokhtar	Proportion	2017	30.1	38.6	23.5
Algeria	Bordj Badji Mokhtar	Count	2000	0	0	0
Algeria	Bordj Badji Mokhtar	Count	2017	0	0	0
Algeria	Ain Soltane	Proportion	2000	32.3	46.2	20.7
Algeria	Ain Soltane	Proportion	2017	33.7	47.5	19.6
Algeria	Ain Soltane	Count	2000	0	0	0
Algeria	Ain Soltane	Count	2017	0	0	0
Algeria	Ain Tolba	Proportion	2000	28.8	42.1	17.6
Algeria	Ain Tolba	Proportion	2017	34.3	48.6	20.5
Algeria	Ain Tolba	Count	2000	0	0	0
Algeria	Ain Tolba	Count	2017	0	0	0
Algeria	Bouzareah	Proportion	2000	29.8	44.7	19.1
Algeria	Bouzareah	Proportion	2017	30.6	47.3	17.5
Algeria	Bouzareah	Count	2000	0	0	0
Algeria	Bouzareah	Count	2017	0	0	0
Algeria	Chorfa	Proportion	2000	33.9	49.3	20.8
Algeria	Chorfa	Proportion	2017	37.8	54.9	24.1
Algeria	Chorfa	Count	2000	0	0	0
Algeria	Chorfa	Count	2017	0	1	0
Algeria	Azil Abdelkader (Metkouak)	Proportion	2000	33.0	49.0	19.8
Algeria	Azil Abdelkader (Metkouak)	Proportion	2017	35.3	50.6	21.7
Algeria	Azil Abdelkader (Metkouak)	Count	2000	0	0	0
Algeria	Azil Abdelkader (Metkouak)	Count	2017	0	1	0
Algeria	Beni Ounif	Proportion	2000	32.3	45.6	21.4
Algeria	Beni Ounif	Proportion	2017	34.7	48.5	23.9
Algeria	Beni Ounif	Count	2000	0	0	0
Algeria	Beni Ounif	Count	2017	0	0	0
Algeria	Akfadou	Proportion	2000	29.9	44.8	17.8
Algeria	Akfadou	Proportion	2017	35.5	50.3	22.3
Algeria	Akfadou	Count	2000	0	0	0
Algeria	Akfadou	Count	2017	0	1	0

Table 4: ORS by Unit *(continued)*

Country	Unit	Metric	Year	Mean	Upper	Lower
Algeria	Bordj Ben Azzouz	Proportion	2000	29.2	43.7	17.3
Algeria	Bordj Ben Azzouz	Proportion	2017	32.5	45.4	19.8
Algeria	Bordj Ben Azzouz	Count	2000	0	0	0
Algeria	Bordj Ben Azzouz	Count	2017	0	0	0
Algeria	Blida	Proportion	2000	30.1	44.7	19.2
Algeria	Blida	Proportion	2017	32.2	50.4	18.5
Algeria	Blida	Count	2000	0	0	0
Algeria	Blida	Count	2017	0	1	0
Algeria	Bir Kasdali	Proportion	2000	33.7	48.1	21.0
Algeria	Bir Kasdali	Proportion	2017	37.4	54.1	21.9
Algeria	Bir Kasdali	Count	2000	0	0	0
Algeria	Bir Kasdali	Count	2017	0	1	0
Algeria	Ain El Hadjar	Proportion	2000	32.0	48.7	19.5
Algeria	Ain El Hadjar	Proportion	2017	36.3	53.0	22.3
Algeria	Ain El Hadjar	Count	2000	0	0	0
Algeria	Ain El Hadjar	Count	2017	0	1	0
Algeria	Beni Amrane	Proportion	2000	30.5	49.3	18.4
Algeria	Beni Amrane	Proportion	2017	35.6	50.3	21.6
Algeria	Beni Amrane	Count	2000	0	0	0
Algeria	Beni Amrane	Count	2017	0	1	0
Algeria	Beni Haoua	Proportion	2000	29.8	43.2	18.3
Algeria	Beni Haoua	Proportion	2017	33.7	49.7	19.8
Algeria	Beni Haoua	Count	2000	0	0	0
Algeria	Beni Haoua	Count	2017	0	0	0
Algeria	Boudjeriou Messaoud	Proportion	2000	34.0	47.8	21.7
Algeria	Boudjeriou Messaoud	Proportion	2017	38.4	55.2	24.3
Algeria	Boudjeriou Messaoud	Count	2000	0	0	0
Algeria	Boudjeriou Messaoud	Count	2017	0	1	0
Algeria	Ain Oussera	Proportion	2000	31.5	47.0	17.0
Algeria	Ain Oussera	Proportion	2017	32.5	47.2	19.5
Algeria	Ain Oussera	Count	2000	0	0	0
Algeria	Ain Oussera	Count	2017	0	0	0
Algeria	Boussemghoun	Proportion	2000	32.1	45.9	19.0
Algeria	Boussemghoun	Proportion	2017	34.5	53.8	20.8
Algeria	Boussemghoun	Count	2000	0	0	0
Algeria	Boussemghoun	Count	2017	0	1	0
Algeria	Douar El Ma	Proportion	2000	30.0	42.7	19.0
Algeria	Douar El Ma	Proportion	2017	30.7	44.6	19.2
Algeria	Douar El Ma	Count	2000	0	0	0
Algeria	Douar El Ma	Count	2017	0	0	0
Algeria	Berrihane	Proportion	2000	33.4	48.4	19.9
Algeria	Berrihane	Proportion	2017	36.4	52.3	23.6
Algeria	Berrihane	Count	2000	0	0	0
Algeria	Berrihane	Count	2017	0	1	0
Algeria	El Guerrarra	Proportion	2000	30.4	46.3	16.9
Algeria	El Guerrarra	Proportion	2017	30.6	44.4	19.0
Algeria	El Guerrarra	Count	2000	0	0	0
Algeria	El Guerrarra	Count	2017	0	0	0
Algeria	Ain Sandel	Proportion	2000	33.4	48.9	21.0

Table 4: ORS by Unit *(continued)*

Country	Unit	Metric	Year	Mean	Upper	Lower
Algeria	Ain Sandel	Proportion	2017	35.6	51.1	22.6
Algeria	Ain Sandel	Count	2000	0	0	0
Algeria	Ain Sandel	Count	2017	0	1	0
Algeria	Illizi	Proportion	2000	29.1	40.0	21.0
Algeria	Illizi	Proportion	2017	33.7	45.3	23.5
Algeria	Illizi	Count	2000	0	0	0
Algeria	Illizi	Count	2017	0	0	0
Algeria	Chahna	Proportion	2000	31.6	45.1	20.1
Algeria	Chahna	Proportion	2017	37.6	53.6	24.3
Algeria	Chahna	Count	2000	0	0	0
Algeria	Chahna	Count	2017	0	1	0
Algeria	Chechar	Proportion	2000	30.6	45.7	19.0
Algeria	Chechar	Proportion	2017	36.1	50.5	21.0
Algeria	Chechar	Count	2000	0	0	0
Algeria	Chechar	Count	2017	0	1	0
Algeria	El Assafia	Proportion	2000	30.4	47.0	18.0
Algeria	El Assafia	Proportion	2017	33.2	50.6	20.8
Algeria	El Assafia	Count	2000	0	0	0
Algeria	El Assafia	Count	2017	0	1	0
Algeria	Ain Khadra	Proportion	2000	31.9	47.1	18.9
Algeria	Ain Khadra	Proportion	2017	34.7	50.5	20.8
Algeria	Ain Khadra	Count	2000	0	0	0
Algeria	Ain Khadra	Count	2017	0	1	0
Algeria	Alaimia	Proportion	2000	25.0	38.9	15.3
Algeria	Alaimia	Proportion	2017	33.9	48.4	19.5
Algeria	Alaimia	Count	2000	0	0	0
Algeria	Alaimia	Count	2017	0	0	0
Algeria	Azzizia	Proportion	2000	33.1	50.0	19.5
Algeria	Azzizia	Proportion	2017	35.6	50.6	22.4
Algeria	Azzizia	Count	2000	0	0	0
Algeria	Azzizia	Count	2017	0	1	0
Algeria	Amirat Arres	Proportion	2000	32.7	45.0	20.3
Algeria	Amirat Arres	Proportion	2017	37.0	53.9	22.5
Algeria	Amirat Arres	Count	2000	0	0	0
Algeria	Amirat Arres	Count	2017	0	1	0
Algeria	Ain Tedles	Proportion	2000	26.2	41.1	15.2
Algeria	Ain Tedles	Proportion	2017	35.6	50.9	20.4
Algeria	Ain Tedles	Count	2000	0	0	0
Algeria	Ain Tedles	Count	2017	0	1	0
Algeria	El Biod	Proportion	2000	31.6	43.0	20.1
Algeria	El Biod	Proportion	2017	34.6	49.1	20.6
Algeria	El Biod	Count	2000	0	0	0
Algeria	El Biod	Count	2017	0	0	0
Algeria	Ben Freha	Proportion	2000	26.1	39.5	16.0
Algeria	Ben Freha	Proportion	2017	34.9	49.5	21.1
Algeria	Ben Freha	Count	2000	0	0	0
Algeria	Ben Freha	Count	2017	0	0	0
Algeria	El Borma	Proportion	2000	30.5	42.0	21.5
Algeria	El Borma	Proportion	2017	30.2	39.8	21.1

Table 4: ORS by Unit *(continued)*

Country	Unit	Metric	Year	Mean	Upper	Lower
Algeria	El Borma	Count	2000	0	0	0
Algeria	El Borma	Count	2017	0	0	0
Algeria	Ain El Kercha	Proportion	2000	33.2	48.5	20.3
Algeria	Ain El Kercha	Proportion	2017	36.6	51.8	21.7
Algeria	Ain El Kercha	Count	2000	0	0	0
Algeria	Ain El Kercha	Count	2017	0	1	0
Algeria	Ben Daoud	Proportion	2000	26.9	43.0	16.1
Algeria	Ben Daoud	Proportion	2017	35.2	51.4	20.0
Algeria	Ben Daoud	Count	2000	0	0	0
Algeria	Ben Daoud	Count	2017	0	1	0
Algeria	El Hassasna	Proportion	2000	30.5	45.5	18.8
Algeria	El Hassasna	Proportion	2017	35.7	53.3	22.3
Algeria	El Hassasna	Count	2000	0	0	0
Algeria	El Hassasna	Count	2017	0	1	0
Algeria	Ain Azel	Proportion	2000	32.3	45.9	20.8
Algeria	Ain Azel	Proportion	2017	37.6	53.6	23.1
Algeria	Ain Azel	Count	2000	0	0	0
Algeria	Ain Azel	Count	2017	0	1	0
Algeria	Ain Tindamine	Proportion	2000	30.5	46.0	16.7
Algeria	Ain Tindamine	Proportion	2017	35.0	50.9	21.4
Algeria	Ain Tindamine	Count	2000	0	0	0
Algeria	Ain Tindamine	Count	2017	0	1	0
Algeria	Azzaba	Proportion	2000	33.4	48.6	20.7
Algeria	Azzaba	Proportion	2017	37.0	54.4	24.3
Algeria	Azzaba	Count	2000	0	0	0
Algeria	Azzaba	Count	2017	0	1	0
Algeria	Haddada	Proportion	2000	37.5	53.1	23.9
Algeria	Haddada	Proportion	2017	42.1	58.1	27.1
Algeria	Haddada	Count	2000	0	1	0
Algeria	Haddada	Count	2017	0	1	0
Algeria	In Guezzam	Proportion	2000	32.1	43.5	22.2
Algeria	In Guezzam	Proportion	2017	33.1	43.9	23.3
Algeria	In Guezzam	Count	2000	0	0	0
Algeria	In Guezzam	Count	2017	0	0	0
Algeria	Bir El Ater	Proportion	2000	30.8	45.6	16.9
Algeria	Bir El Ater	Proportion	2017	37.2	54.6	22.5
Algeria	Bir El Ater	Count	2000	0	0	0
Algeria	Bir El Ater	Count	2017	0	1	0
Algeria	Ain Zarit	Proportion	2000	31.6	46.2	19.3
Algeria	Ain Zarit	Proportion	2017	34.9	49.8	21.8
Algeria	Ain Zarit	Count	2000	0	0	0
Algeria	Ain Zarit	Count	2017	0	0	0
Algeria	Bou Ismail	Proportion	2000	31.0	46.0	19.1
Algeria	Bou Ismail	Proportion	2017	33.4	51.2	19.2
Algeria	Bou Ismail	Count	2000	0	0	0
Algeria	Bou Ismail	Count	2017	0	1	0
Algeria	Bordj El Emir Abdelkader	Proportion	2000	32.7	49.3	19.9
Algeria	Bordj El Emir Abdelkader	Proportion	2017	34.6	48.6	21.0
Algeria	Bordj El Emir Abdelkader	Count	2000	0	0	0

Table 4: ORS by Unit *(continued)*

Country	Unit	Metric	Year	Mean	Upper	Lower
Algeria	Bordj El Emir Abdelkader	Count	2017	0	0	0
Algeria	Ain-Zaouia	Proportion	2000	29.4	46.1	16.1
Algeria	Ain-Zaouia	Proportion	2017	35.4	49.4	21.2
Algeria	Ain-Zaouia	Count	2000	0	0	0
Algeria	Ain-Zaouia	Count	2017	0	0	0
Algeria	Ain Nehala	Proportion	2000	29.1	44.4	16.0
Algeria	Ain Nehala	Proportion	2017	34.7	50.7	22.0
Algeria	Ain Nehala	Count	2000	0	0	0
Algeria	Ain Nehala	Count	2017	0	1	0
Algeria	Bouda	Proportion	2000	28.9	43.6	16.9
Algeria	Bouda	Proportion	2017	30.8	44.1	19.3
Algeria	Bouda	Count	2000	0	0	0
Algeria	Bouda	Count	2017	0	0	0
Algeria	Ain Tork	Proportion	2000	31.4	44.7	20.0
Algeria	Ain Tork	Proportion	2017	32.7	46.2	18.9
Algeria	Ain Tork	Count	2000	0	0	0
Algeria	Ain Tork	Count	2017	0	0	0
Algeria	Aoubellil	Proportion	2000	28.7	43.3	16.2
Algeria	Aoubellil	Proportion	2017	34.7	49.4	21.8
Algeria	Aoubellil	Count	2000	0	0	0
Algeria	Aoubellil	Count	2017	0	0	0
Algeria	Cheraga	Proportion	2000	30.5	46.3	19.0
Algeria	Cheraga	Proportion	2017	30.8	46.4	17.4
Algeria	Cheraga	Count	2000	0	0	0
Algeria	Cheraga	Count	2017	0	0	0
Algeria	El Bouni	Proportion	2000	33.5	48.4	20.2
Algeria	El Bouni	Proportion	2017	34.0	51.3	20.3
Algeria	El Bouni	Count	2000	0	0	0
Algeria	El Bouni	Count	2017	0	1	0
Algeria	Barika	Proportion	2000	32.6	48.9	18.9
Algeria	Barika	Proportion	2017	37.1	53.9	21.4
Algeria	Barika	Count	2000	0	0	0
Algeria	Barika	Count	2017	0	1	0
Algeria	Boukais	Proportion	2000	33.2	48.7	19.6
Algeria	Boukais	Proportion	2017	34.0	49.7	20.7
Algeria	Boukais	Count	2000	0	0	0
Algeria	Boukais	Count	2017	0	0	0
Algeria	Amalou	Proportion	2000	31.3	47.6	18.9
Algeria	Amalou	Proportion	2017	37.5	53.8	23.3
Algeria	Amalou	Count	2000	0	0	0
Algeria	Amalou	Count	2017	0	1	0
Algeria	Bouchakroune	Proportion	2000	31.3	44.4	18.3
Algeria	Bouchakroune	Proportion	2017	31.5	45.1	19.4
Algeria	Bouchakroune	Count	2000	0	0	0
Algeria	Bouchakroune	Count	2017	0	0	0
Algeria	Bouaarfa	Proportion	2000	29.8	44.4	18.9
Algeria	Bouaarfa	Proportion	2017	32.5	50.4	19.1
Algeria	Bouaarfa	Count	2000	0	0	0
Algeria	Bouaarfa	Count	2017	0	1	0

Table 4: ORS by Unit *(continued)*

Country	Unit	Metric	Year	Mean	Upper	Lower
Algeria	Bordj Bou Arreridj	Proportion	2000	32.3	47.5	19.9
Algeria	Bordj Bou Arreridj	Proportion	2017	36.3	52.8	21.0
Algeria	Bordj Bou Arreridj	Count	2000	0	0	0
Algeria	Bordj Bou Arreridj	Count	2017	0	1	0
Algeria	Ain Laloui	Proportion	2000	32.5	48.4	20.0
Algeria	Ain Laloui	Proportion	2017	36.3	52.5	23.1
Algeria	Ain Laloui	Count	2000	0	0	0
Algeria	Ain Laloui	Count	2017	0	1	0
Algeria	Bordj Menaiel	Proportion	2000	29.1	48.8	16.9
Algeria	Bordj Menaiel	Proportion	2017	35.3	49.3	21.5
Algeria	Bordj Menaiel	Count	2000	0	0	0
Algeria	Bordj Menaiel	Count	2017	0	0	0
Algeria	Beni Rached	Proportion	2000	29.8	44.2	17.8
Algeria	Beni Rached	Proportion	2017	33.7	49.2	19.0
Algeria	Beni Rached	Count	2000	0	0	0
Algeria	Beni Rached	Count	2017	0	0	0
Algeria	Constantine	Proportion	2000	33.3	48.6	19.3
Algeria	Constantine	Proportion	2017	36.3	54.9	21.3
Algeria	Constantine	Count	2000	0	0	0
Algeria	Constantine	Count	2017	0	1	0
Algeria	Amourah	Proportion	2000	30.1	46.0	18.4
Algeria	Amourah	Proportion	2017	31.7	46.7	19.8
Algeria	Amourah	Count	2000	0	0	0
Algeria	Amourah	Count	2017	0	0	0
Algeria	Brezina	Proportion	2000	31.2	44.9	19.9
Algeria	Brezina	Proportion	2017	32.4	43.7	21.8
Algeria	Brezina	Count	2000	0	0	0
Algeria	Brezina	Count	2017	0	0	0
Algeria	El M'Ghair	Proportion	2000	29.4	45.5	17.0
Algeria	El M'Ghair	Proportion	2017	32.6	46.3	19.1
Algeria	El M'Ghair	Count	2000	0	0	0
Algeria	El M'Ghair	Count	2017	0	0	0
Algeria	Besbes	Proportion	2000	33.2	47.6	20.0
Algeria	Besbes	Proportion	2017	37.1	53.9	23.1
Algeria	Besbes	Count	2000	0	0	0
Algeria	Besbes	Count	2017	0	1	0
Algeria	El Meniaa	Proportion	2000	31.4	44.6	19.2
Algeria	El Meniaa	Proportion	2017	30.7	44.2	20.3
Algeria	El Meniaa	Count	2000	0	0	0
Algeria	El Meniaa	Count	2017	0	0	0
Algeria	Belkheir	Proportion	2000	34.1	50.5	21.1
Algeria	Belkheir	Proportion	2017	37.8	53.2	24.0
Algeria	Belkheir	Count	2000	0	1	0
Algeria	Belkheir	Count	2017	0	1	0
Algeria	In Amenas	Proportion	2000	27.5	42.0	16.7
Algeria	In Amenas	Proportion	2017	30.1	41.4	19.2
Algeria	In Amenas	Count	2000	0	0	0
Algeria	In Amenas	Count	2017	0	0	0
Algeria	Chekfa	Proportion	2000	31.4	45.5	19.4

Table 4: ORS by Unit *(continued)*

Country	Unit	Metric	Year	Mean	Upper	Lower
Algeria	Chekfa	Proportion	2017	37.9	55.0	24.3
Algeria	Chekfa	Count	2000	0	0	0
Algeria	Chekfa	Count	2017	0	1	0
Algeria	Chelia	Proportion	2000	30.9	45.5	19.2
Algeria	Chelia	Proportion	2017	35.9	51.9	21.0
Algeria	Chelia	Count	2000	0	0	0
Algeria	Chelia	Count	2017	0	1	0
Algeria	El Beidha	Proportion	2000	30.6	45.5	17.6
Algeria	El Beidha	Proportion	2017	33.4	48.5	21.0
Algeria	El Beidha	Count	2000	0	0	0
Algeria	El Beidha	Count	2017	0	0	0
Algeria	Belaiba	Proportion	2000	31.4	45.7	18.8
Algeria	Belaiba	Proportion	2017	35.2	51.8	21.8
Algeria	Belaiba	Count	2000	0	0	0
Algeria	Belaiba	Count	2017	0	1	0
Algeria	Aouf	Proportion	2000	30.1	45.6	18.1
Algeria	Aouf	Proportion	2017	35.2	51.2	21.5
Algeria	Aouf	Count	2000	0	0	0
Algeria	Aouf	Count	2017	0	1	0
Algeria	Baata	Proportion	2000	33.1	49.8	21.1
Algeria	Baata	Proportion	2017	34.5	49.6	21.3
Algeria	Baata	Count	2000	0	0	0
Algeria	Baata	Count	2017	0	0	0
Algeria	Benyahia Abderrahmane	Proportion	2000	33.6	46.1	20.5
Algeria	Benyahia Abderrahmane	Proportion	2017	36.7	51.7	21.9
Algeria	Benyahia Abderrahmane	Count	2000	0	0	0
Algeria	Benyahia Abderrahmane	Count	2017	0	1	0
Algeria	Benabdelmalek Ramdane	Proportion	2000	26.6	41.2	15.9
Algeria	Benabdelmalek Ramdane	Proportion	2017	36.2	51.5	21.7
Algeria	Benabdelmalek Ramdane	Count	2000	0	0	0
Algeria	Benabdelmalek Ramdane	Count	2017	0	1	0
Algeria	Kasdir	Proportion	2000	31.2	45.9	20.0
Algeria	Kasdir	Proportion	2017	32.1	44.8	19.3
Algeria	Kasdir	Count	2000	0	0	0
Algeria	Kasdir	Count	2017	0	0	0
Algeria	Bethioua	Proportion	2000	25.6	38.6	16.2
Algeria	Bethioua	Proportion	2017	33.6	47.7	19.4
Algeria	Bethioua	Count	2000	0	0	0
Algeria	Bethioua	Count	2017	0	0	0
Algeria	El Hadjira	Proportion	2000	29.7	45.0	17.8
Algeria	El Hadjira	Proportion	2017	30.4	44.4	18.5
Algeria	El Hadjira	Count	2000	0	0	0
Algeria	El Hadjira	Count	2017	0	0	0
Algeria	Ain M'Lila	Proportion	2000	32.6	47.9	19.5
Algeria	Ain M'Lila	Proportion	2017	35.7	50.0	21.9
Algeria	Ain M'Lila	Count	2000	0	0	0
Algeria	Ain M'Lila	Count	2017	0	0	0
Algeria	Beni Dergoun	Proportion	2000	28.8	45.5	17.4
Algeria	Beni Dergoun	Proportion	2017	35.4	50.9	21.1

Table 4: ORS by Unit *(continued)*

Country	Unit	Metric	Year	Mean	Upper	Lower
Algeria	Beni Dergoun	Count	2000	0	0	0
Algeria	Beni Dergoun	Count	2017	0	1	0
Algeria	Hounet	Proportion	2000	28.6	42.8	16.6
Algeria	Hounet	Proportion	2017	35.1	50.4	21.3
Algeria	Hounet	Count	2000	0	0	0
Algeria	Hounet	Count	2017	0	1	0
Algeria	Ain El Kebira	Proportion	2000	33.8	47.4	19.8
Algeria	Ain El Kebira	Proportion	2017	37.5	54.4	22.7
Algeria	Ain El Kebira	Count	2000	0	0	0
Algeria	Ain El Kebira	Count	2017	0	1	0
Algeria	Amernas	Proportion	2000	28.5	43.6	16.4
Algeria	Amernas	Proportion	2017	34.9	52.8	19.3
Algeria	Amernas	Count	2000	0	0	0
Algeria	Amernas	Count	2017	0	1	0
Algeria	Bekkouche Lakhdar	Proportion	2000	34.3	50.6	21.7
Algeria	Bekkouche Lakhdar	Proportion	2017	38.6	57.0	25.9
Algeria	Bekkouche Lakhdar	Count	2000	0	1	0
Algeria	Bekkouche Lakhdar	Count	2017	0	1	0
Algeria	Hanencha	Proportion	2000	33.0	50.1	20.3
Algeria	Hanencha	Proportion	2017	35.6	53.6	23.3
Algeria	Hanencha	Count	2000	0	1	0
Algeria	Hanencha	Count	2017	0	1	0
Algeria	In M'Guel	Proportion	2000	29.7	39.6	20.6
Algeria	In M'Guel	Proportion	2017	30.2	39.7	21.3
Algeria	In M'Guel	Count	2000	0	0	0
Algeria	In M'Guel	Count	2017	0	0	0
Algeria	Bir Mokkadem	Proportion	2000	30.3	44.7	16.2
Algeria	Bir Mokkadem	Proportion	2017	35.3	50.4	22.7
Algeria	Bir Mokkadem	Count	2000	0	0	0
Algeria	Bir Mokkadem	Count	2017	0	1	0
Algeria	Bougara	Proportion	2000	32.5	47.8	19.4
Algeria	Bougara	Proportion	2017	35.2	49.9	20.2
Algeria	Bougara	Count	2000	0	0	0
Algeria	Bougara	Count	2017	0	0	0
Algeria	Bourkika	Proportion	2000	31.4	45.2	19.3
Algeria	Bourkika	Proportion	2017	33.9	50.7	19.7
Algeria	Bourkika	Count	2000	0	0	0
Algeria	Bourkika	Count	2017	0	1	0
Algeria	Bou Caid	Proportion	2000	31.6	45.6	19.1
Algeria	Bou Caid	Proportion	2017	34.4	49.9	19.3
Algeria	Bou Caid	Count	2000	0	0	0
Algeria	Bou Caid	Count	2017	0	0	0
Algeria	Ait-Chaffaa	Proportion	2000	28.7	45.4	17.4
Algeria	Ait-Chaffaa	Proportion	2017	34.6	48.4	21.3
Algeria	Ait-Chaffaa	Count	2000	0	0	0
Algeria	Ait-Chaffaa	Count	2017	0	0	0
Algeria	Ain Tallout	Proportion	2000	30.0	45.6	17.2
Algeria	Ain Tallout	Proportion	2017	34.9	49.8	21.8
Algeria	Ain Tallout	Count	2000	0	0	0

Table 4: ORS by Unit *(continued)*

Country	Unit	Metric	Year	Mean	Upper	Lower
Algeria	Ain Tallout	Count	2017	0	0	0
Algeria	Charouine	Proportion	2000	29.7	45.8	18.7
Algeria	Charouine	Proportion	2017	30.6	47.0	18.6
Algeria	Charouine	Count	2000	0	0	0
Algeria	Charouine	Count	2017	0	0	0
Algeria	Arib	Proportion	2000	30.7	45.1	19.4
Algeria	Arib	Proportion	2017	33.4	49.8	19.2
Algeria	Arib	Count	2000	0	0	0
Algeria	Arib	Count	2017	0	0	0
Algeria	Beni Saf	Proportion	2000	28.5	43.3	16.6
Algeria	Beni Saf	Proportion	2017	34.5	48.6	19.8
Algeria	Beni Saf	Count	2000	0	0	0
Algeria	Beni Saf	Count	2017	0	0	0
Algeria	Dar El Beida	Proportion	2000	30.6	46.4	18.7
Algeria	Dar El Beida	Proportion	2017	33.2	49.0	19.7
Algeria	Dar El Beida	Count	2000	0	0	0
Algeria	Dar El Beida	Count	2017	0	0	0
Algeria	El Eulma	Proportion	2000	34.1	50.0	20.3
Algeria	El Eulma	Proportion	2017	37.8	56.1	24.2
Algeria	El Eulma	Count	2000	0	0	0
Algeria	El Eulma	Count	2017	0	1	0
Algeria	Batna	Proportion	2000	31.2	47.9	17.5
Algeria	Batna	Proportion	2017	37.2	52.3	21.3
Algeria	Batna	Count	2000	0	0	0
Algeria	Batna	Count	2017	0	1	0
Algeria	El Ouata	Proportion	2000	32.2	46.6	19.7
Algeria	El Ouata	Proportion	2017	33.3	48.1	20.6
Algeria	El Ouata	Count	2000	0	0	0
Algeria	El Ouata	Count	2017	0	0	0
Algeria	Amizour	Proportion	2000	30.0	44.5	18.8
Algeria	Amizour	Proportion	2017	36.5	51.9	23.0
Algeria	Amizour	Count	2000	0	0	0
Algeria	Amizour	Count	2017	0	1	0
Algeria	Branis	Proportion	2000	30.7	44.5	18.9
Algeria	Branis	Proportion	2017	33.3	47.7	20.4
Algeria	Branis	Count	2000	0	0	0
Algeria	Branis	Count	2017	0	0	0
Algeria	Boufarik	Proportion	2000	30.6	45.8	19.3
Algeria	Boufarik	Proportion	2017	34.1	49.4	20.4
Algeria	Boufarik	Count	2000	0	0	0
Algeria	Boufarik	Count	2017	0	0	0
Algeria	Bordj Ghdir	Proportion	2000	32.8	48.5	21.1
Algeria	Bordj Ghdir	Proportion	2017	36.4	52.9	21.8
Algeria	Bordj Ghdir	Count	2000	0	0	0
Algeria	Bordj Ghdir	Count	2017	0	1	0
Algeria	Ain Turk	Proportion	2000	31.7	48.6	19.1
Algeria	Ain Turk	Proportion	2017	36.4	52.4	22.4
Algeria	Ain Turk	Count	2000	0	0	0
Algeria	Ain Turk	Count	2017	0	1	0

Table 4: ORS by Unit *(continued)*

Country	Unit	Metric	Year	Mean	Upper	Lower
Algeria	Boudouaou	Proportion	2000	30.1	46.9	17.8
Algeria	Boudouaou	Proportion	2017	32.9	48.5	19.8
Algeria	Boudouaou	Count	2000	0	0	0
Algeria	Boudouaou	Count	2017	0	0	0
Algeria	Boukadir	Proportion	2000	28.5	43.6	16.5
Algeria	Boukadir	Proportion	2017	35.3	52.7	20.7
Algeria	Boukadir	Count	2000	0	0	0
Algeria	Boukadir	Count	2017	0	1	0
Algeria	Didouche Mourad	Proportion	2000	34.5	49.1	21.2
Algeria	Didouche Mourad	Proportion	2017	36.8	54.8	22.3
Algeria	Didouche Mourad	Count	2000	0	0	0
Algeria	Didouche Mourad	Count	2017	0	1	0
Algeria	Benhar	Proportion	2000	31.0	46.1	16.7
Algeria	Benhar	Proportion	2017	32.7	46.8	20.6
Algeria	Benhar	Count	2000	0	0	0
Algeria	Benhar	Count	2017	0	0	0
Algeria	Cheguig	Proportion	2000	30.6	45.3	18.9
Algeria	Cheguig	Proportion	2017	33.5	48.1	20.5
Algeria	Cheguig	Count	2000	0	0	0
Algeria	Cheguig	Count	2017	0	0	0
Algeria	El Ogla	Proportion	2000	31.2	48.2	18.2
Algeria	El Ogla	Proportion	2017	30.2	46.0	18.2
Algeria	El Ogla	Count	2000	0	0	0
Algeria	El Ogla	Count	2017	0	0	0
Algeria	Bougous	Proportion	2000	32.6	47.2	19.9
Algeria	Bougous	Proportion	2017	36.4	51.4	22.8
Algeria	Bougous	Count	2000	0	0	0
Algeria	Bougous	Count	2017	0	1	0
Algeria	Ghardaia	Proportion	2000	30.1	46.8	17.1
Algeria	Ghardaia	Proportion	2017	30.3	45.5	17.5
Algeria	Ghardaia	Count	2000	0	0	0
Algeria	Ghardaia	Count	2017	0	0	0
Algeria	Ben Djerrah	Proportion	2000	33.4	50.4	20.1
Algeria	Ben Djerrah	Proportion	2017	37.6	52.8	23.5
Algeria	Ben Djerrah	Count	2000	0	1	0
Algeria	Ben Djerrah	Count	2017	0	1	0
Algeria	Djemaa Beni Habibi	Proportion	2000	31.4	45.4	19.6
Algeria	Djemaa Beni Habibi	Proportion	2017	38.1	54.1	24.3
Algeria	Djemaa Beni Habibi	Count	2000	0	0	0
Algeria	Djemaa Beni Habibi	Count	2017	0	1	0
Algeria	Djellal	Proportion	2000	30.5	46.1	18.7
Algeria	Djellal	Proportion	2017	35.6	50.8	21.2
Algeria	Djellal	Count	2000	0	0	0
Algeria	Djellal	Count	2017	0	1	0
Algeria	El Ghicha	Proportion	2000	29.7	45.8	16.7
Algeria	El Ghicha	Proportion	2017	32.5	45.4	21.0
Algeria	El Ghicha	Count	2000	0	0	0
Algeria	El Ghicha	Count	2017	0	0	0
Algeria	Ben Srour	Proportion	2000	31.8	47.2	20.1

Table 4: ORS by Unit *(continued)*

Country	Unit	Metric	Year	Mean	Upper	Lower
Algeria	Ben Srour	Proportion	2017	33.8	49.7	19.7
Algeria	Ben Srour	Count	2000	0	0	0
Algeria	Ben Srour	Count	2017	0	0	0
Algeria	Benian	Proportion	2000	30.0	45.5	17.9
Algeria	Benian	Proportion	2017	35.2	52.6	21.3
Algeria	Benian	Count	2000	0	0	0
Algeria	Benian	Count	2017	0	1	0
Algeria	Ben Chicao	Proportion	2000	32.8	49.2	19.8
Algeria	Ben Chicao	Proportion	2017	34.2	49.3	21.2
Algeria	Ben Chicao	Count	2000	0	0	0
Algeria	Ben Chicao	Count	2017	0	0	0
Algeria	Bouhatem	Proportion	2000	33.3	46.0	20.3
Algeria	Bouhatem	Proportion	2017	37.2	53.0	22.1
Algeria	Bouhatem	Count	2000	0	0	0
Algeria	Bouhatem	Count	2017	0	1	0
Algeria	Bouguirat	Proportion	2000	25.8	39.4	15.5
Algeria	Bouguirat	Proportion	2017	35.1	50.6	19.7
Algeria	Bouguirat	Count	2000	0	0	0
Algeria	Bouguirat	Count	2017	0	1	0
Algeria	Makman Ben Amer	Proportion	2000	31.6	45.2	19.1
Algeria	Makman Ben Amer	Proportion	2017	32.9	47.8	19.9
Algeria	Makman Ben Amer	Count	2000	0	0	0
Algeria	Makman Ben Amer	Count	2017	0	0	0
Algeria	Bir El Djir	Proportion	2000	26.4	38.8	15.8
Algeria	Bir El Djir	Proportion	2017	33.0	47.7	19.5
Algeria	Bir El Djir	Count	2000	0	0	0
Algeria	Bir El Djir	Count	2017	0	0	0
Algeria	Hassi Ben Abdellah	Proportion	2000	29.4	41.6	17.1
Algeria	Hassi Ben Abdellah	Proportion	2017	30.5	43.7	19.9
Algeria	Hassi Ben Abdellah	Count	2000	0	0	0
Algeria	Hassi Ben Abdellah	Count	2017	0	0	0
Algeria	Ain Zitoun	Proportion	2000	32.2	47.6	20.1
Algeria	Ain Zitoun	Proportion	2017	36.3	51.8	21.1
Algeria	Ain Zitoun	Count	2000	0	0	0
Algeria	Ain Zitoun	Count	2017	0	1	0
Algeria	Beni Zentis	Proportion	2000	26.8	42.7	15.8
Algeria	Beni Zentis	Proportion	2017	35.3	50.6	21.3
Algeria	Beni Zentis	Count	2000	0	0	0
Algeria	Beni Zentis	Count	2017	0	1	0
Algeria	Maamora	Proportion	2000	30.8	44.8	18.8
Algeria	Maamora	Proportion	2017	36.1	52.8	21.5
Algeria	Maamora	Count	2000	0	0	0
Algeria	Maamora	Count	2017	0	1	0
Algeria	Ain Lahdjar	Proportion	2000	33.2	46.4	21.9
Algeria	Ain Lahdjar	Proportion	2017	36.9	51.5	22.1
Algeria	Ain Lahdjar	Count	2000	0	0	0
Algeria	Ain Lahdjar	Count	2017	0	1	0
Algeria	Badredine El Mokrani	Proportion	2000	29.2	45.5	16.2
Algeria	Badredine El Mokrani	Proportion	2017	34.7	50.6	21.4

Table 4: ORS by Unit *(continued)*

Country	Unit	Metric	Year	Mean	Upper	Lower
Algeria	Badredine El Mokrani	Count	2000	0	0	0
Algeria	Badredine El Mokrani	Count	2017	0	1	0
Algeria	Ben Azzouz	Proportion	2000	33.2	49.7	19.9
Algeria	Ben Azzouz	Proportion	2017	37.4	55.3	24.2
Algeria	Ben Azzouz	Count	2000	0	0	0
Algeria	Ben Azzouz	Count	2017	0	1	0
Algeria	Khedara	Proportion	2000	33.4	49.7	20.7
Algeria	Khedara	Proportion	2017	35.6	53.0	21.3
Algeria	Khedara	Count	2000	0	0	0
Algeria	Khedara	Count	2017	0	1	0
Algeria	In Salah	Proportion	2000	30.1	42.8	19.3
Algeria	In Salah	Proportion	2017	30.0	43.3	18.8
Algeria	In Salah	Count	2000	0	0	0
Algeria	In Salah	Count	2017	0	0	0
Algeria	Boukhadra	Proportion	2000	32.3	47.6	19.6
Algeria	Boukhadra	Proportion	2017	36.6	52.0	23.5
Algeria	Boukhadra	Count	2000	0	0	0
Algeria	Boukhadra	Count	2017	0	1	0
Algeria	Chehaima	Proportion	2000	31.6	45.7	20.3
Algeria	Chehaima	Proportion	2017	35.7	50.6	23.6
Algeria	Chehaima	Count	2000	0	0	0
Algeria	Chehaima	Count	2017	0	1	0
Algeria	Chaiba	Proportion	2000	30.8	45.9	18.6
Algeria	Chaiba	Proportion	2017	32.7	50.8	18.9
Algeria	Chaiba	Count	2000	0	0	0
Algeria	Chaiba	Count	2017	0	1	0
Algeria	El Youssoufia	Proportion	2000	32.4	47.1	19.2
Algeria	El Youssoufia	Proportion	2017	33.8	47.9	20.0
Algeria	El Youssoufia	Count	2000	0	0	0
Algeria	El Youssoufia	Count	2017	0	0	0
Algeria	Ait-Mahmoud	Proportion	2000	28.7	46.5	16.4
Algeria	Ait-Mahmoud	Proportion	2017	35.3	49.0	21.5
Algeria	Ait-Mahmoud	Count	2000	0	0	0
Algeria	Ait-Mahmoud	Count	2017	0	0	0
Algeria	Ain Youcef	Proportion	2000	29.5	45.8	18.4
Algeria	Ain Youcef	Proportion	2017	34.9	52.1	21.6
Algeria	Ain Youcef	Count	2000	0	0	0
Algeria	Ain Youcef	Count	2017	0	1	0
Algeria	Deldoul	Proportion	2000	29.6	43.7	18.2
Algeria	Deldoul	Proportion	2017	31.0	47.5	18.2
Algeria	Deldoul	Count	2000	0	0	0
Algeria	Deldoul	Count	2017	0	0	0
Algeria	Barbouche	Proportion	2000	33.0	49.3	21.6
Algeria	Barbouche	Proportion	2017	33.9	48.4	20.9
Algeria	Barbouche	Count	2000	0	0	0
Algeria	Barbouche	Count	2017	0	0	0
Algeria	Bou Zedjar	Proportion	2000	27.9	40.6	17.4
Algeria	Bou Zedjar	Proportion	2017	33.5	47.2	20.0
Algeria	Bou Zedjar	Count	2000	0	0	0

Table 4: ORS by Unit *(continued)*

Country	Unit	Metric	Year	Mean	Upper	Lower
Algeria	Bou Zedjar	Count	2017	0	0	0
Algeria	Djasr Kasentina	Proportion	2000	30.1	44.6	19.4
Algeria	Djasr Kasentina	Proportion	2017	30.9	46.7	17.1
Algeria	Djasr Kasentina	Count	2000	0	0	0
Algeria	Djasr Kasentina	Count	2017	0	0	0
Algeria	El Hadjar	Proportion	2000	33.5	48.6	20.4
Algeria	El Hadjar	Proportion	2017	36.3	53.8	21.7
Algeria	El Hadjar	Count	2000	0	0	0
Algeria	El Hadjar	Count	2017	0	1	0
Algeria	Beni Foudala El Hakania	Proportion	2000	31.4	46.9	19.5
Algeria	Beni Foudala El Hakania	Proportion	2017	36.9	51.4	22.5
Algeria	Beni Foudala El Hakania	Count	2000	0	0	0
Algeria	Beni Foudala El Hakania	Count	2017	0	1	0
Algeria	Erg Ferradj	Proportion	2000	33.7	50.1	20.8
Algeria	Erg Ferradj	Proportion	2017	34.4	50.0	21.5
Algeria	Erg Ferradj	Count	2000	0	1	0
Algeria	Erg Ferradj	Count	2017	0	0	0
Algeria	Aokas	Proportion	2000	30.6	45.4	18.5
Algeria	Aokas	Proportion	2017	37.5	54.4	24.8
Algeria	Aokas	Count	2000	0	0	0
Algeria	Aokas	Count	2017	0	1	0
Algeria	Chetma	Proportion	2000	29.9	43.4	17.6
Algeria	Chetma	Proportion	2017	31.7	46.2	19.6
Algeria	Chetma	Count	2000	0	0	0
Algeria	Chetma	Count	2017	0	0	0
Algeria	Bougara	Proportion	2000	31.0	46.7	19.5
Algeria	Bougara	Proportion	2017	35.9	50.6	22.3
Algeria	Bougara	Count	2000	0	0	0
Algeria	Bougara	Count	2017	0	1	0
Algeria	Bordj Zemoura	Proportion	2000	33.4	48.0	21.0
Algeria	Bordj Zemoura	Proportion	2017	37.3	53.6	22.4
Algeria	Bordj Zemoura	Count	2000	0	0	0
Algeria	Bordj Zemoura	Count	2017	0	1	0
Algeria	Ait Laaziz	Proportion	2000	30.9	47.5	17.9
Algeria	Ait Laaziz	Proportion	2017	36.1	51.0	22.6
Algeria	Ait Laaziz	Count	2000	0	0	0
Algeria	Ait Laaziz	Count	2017	0	1	0
Algeria	Boudouaou El Bahri	Proportion	2000	30.6	48.4	18.2
Algeria	Boudouaou El Bahri	Proportion	2017	33.9	49.8	20.7
Algeria	Boudouaou El Bahri	Count	2000	0	0	0
Algeria	Boudouaou El Bahri	Count	2017	0	0	0
Algeria	Bouzeghaia	Proportion	2000	29.0	44.0	17.8
Algeria	Bouzeghaia	Proportion	2017	33.6	50.0	19.5
Algeria	Bouzeghaia	Count	2000	0	0	0
Algeria	Bouzeghaia	Count	2017	0	0	0
Algeria	El Khroub	Proportion	2000	34.6	50.7	20.3
Algeria	El Khroub	Proportion	2017	36.2	53.9	21.3
Algeria	El Khroub	Count	2000	0	1	0
Algeria	El Khroub	Count	2017	0	1	0

Table 4: ORS by Unit *(continued)*

Country	Unit	Metric	Year	Mean	Upper	Lower
Algeria	Benyacoub	Proportion	2000	29.9	48.1	16.8
Algeria	Benyacoub	Proportion	2017	31.3	46.3	19.8
Algeria	Benyacoub	Count	2000	0	0	0
Algeria	Benyacoub	Count	2017	0	0	0
Algeria	Chellala	Proportion	2000	32.3	44.9	20.4
Algeria	Chellala	Proportion	2017	34.5	51.5	21.1
Algeria	Chellala	Count	2000	0	0	0
Algeria	Chellala	Count	2017	0	1	0
Algeria	El Oued	Proportion	2000	29.6	45.6	17.1
Algeria	El Oued	Proportion	2017	29.5	45.0	17.2
Algeria	El Oued	Count	2000	0	0	0
Algeria	El Oued	Count	2017	0	0	0
Algeria	Bouhadjar	Proportion	2000	33.8	49.0	21.0
Algeria	Bouhadjar	Proportion	2017	37.3	53.5	23.9
Algeria	Bouhadjar	Count	2000	0	0	0
Algeria	Bouhadjar	Count	2017	0	1	0
Algeria	Hassi Fehal	Proportion	2000	31.9	44.8	19.4
Algeria	Hassi Fehal	Proportion	2017	32.3	44.9	19.6
Algeria	Hassi Fehal	Count	2000	0	0	0
Algeria	Hassi Fehal	Count	2017	0	0	0
Algeria	Beni Mezline	Proportion	2000	34.0	49.5	21.3
Algeria	Beni Mezline	Proportion	2017	37.7	55.1	24.0
Algeria	Beni Mezline	Count	2000	0	0	0
Algeria	Beni Mezline	Count	2017	0	1	0
Algeria	Djemila	Proportion	2000	32.7	45.4	20.7
Algeria	Djemila	Proportion	2017	37.4	53.5	23.5
Algeria	Djemila	Count	2000	0	0	0
Algeria	Djemila	Count	2017	0	1	0
Algeria	El Hamma	Proportion	2000	31.0	46.5	19.2
Algeria	El Hamma	Proportion	2017	35.7	50.1	20.6
Algeria	El Hamma	Count	2000	0	0	0
Algeria	El Hamma	Count	2017	0	1	0
Algeria	El Haouaita	Proportion	2000	30.6	48.0	18.2
Algeria	El Haouaita	Proportion	2017	33.1	45.8	21.2
Algeria	El Haouaita	Count	2000	0	0	0
Algeria	El Haouaita	Count	2017	0	0	0
Algeria	Beni Ilmane	Proportion	2000	32.7	47.5	19.6
Algeria	Beni Ilmane	Proportion	2017	35.1	50.1	20.8
Algeria	Beni Ilmane	Count	2000	0	0	0
Algeria	Beni Ilmane	Count	2017	0	1	0
Algeria	Bou Henni	Proportion	2000	25.6	37.9	14.7
Algeria	Bou Henni	Proportion	2017	34.6	51.2	19.8
Algeria	Bou Henni	Count	2000	0	0	0
Algeria	Bou Henni	Count	2017	0	1	0
Algeria	Beni Slimane	Proportion	2000	33.8	51.1	19.4
Algeria	Beni Slimane	Proportion	2017	37.0	51.2	23.4
Algeria	Beni Slimane	Count	2000	0	1	0
Algeria	Beni Slimane	Count	2017	0	1	0
Algeria	Chelghoum Laid	Proportion	2000	33.7	47.3	19.6

Table 4: ORS by Unit *(continued)*

Country	Unit	Metric	Year	Mean	Upper	Lower
Algeria	Chelghoum Laid	Proportion	2017	37.3	52.3	21.9
Algeria	Chelghoum Laid	Count	2000	0	0	0
Algeria	Chelghoum Laid	Count	2017	0	1	0
Algeria	Fornaka	Proportion	2000	25.2	39.0	14.3
Algeria	Fornaka	Proportion	2017	35.0	51.1	20.5
Algeria	Fornaka	Count	2000	0	0	0
Algeria	Fornaka	Count	2017	0	1	0
Algeria	Mecheria	Proportion	2000	32.6	45.3	20.7
Algeria	Mecheria	Proportion	2017	35.8	52.5	20.5
Algeria	Mecheria	Count	2000	0	0	0
Algeria	Mecheria	Count	2017	0	1	0
Algeria	Boufatis	Proportion	2000	26.2	39.7	15.7
Algeria	Boufatis	Proportion	2017	34.9	49.8	20.9
Algeria	Boufatis	Count	2000	0	0	0
Algeria	Boufatis	Count	2017	0	0	0
Algeria	Hassi Messaoud	Proportion	2000	29.5	40.7	18.6
Algeria	Hassi Messaoud	Proportion	2017	30.9	44.3	19.8
Algeria	Hassi Messaoud	Count	2000	0	0	0
Algeria	Hassi Messaoud	Count	2017	0	0	0
Algeria	Belala	Proportion	2000	32.3	46.9	19.0
Algeria	Belala	Proportion	2017	36.5	51.6	23.2
Algeria	Belala	Count	2000	0	0	0
Algeria	Belala	Count	2017	0	1	0
Algeria	Dar Ben Abdelah	Proportion	2000	29.2	46.0	17.6
Algeria	Dar Ben Abdelah	Proportion	2017	35.4	50.9	20.3
Algeria	Dar Ben Abdelah	Count	2000	0	0	0
Algeria	Dar Ben Abdelah	Count	2017	0	1	0
Algeria	Moulay Larbi	Proportion	2000	30.5	45.7	18.1
Algeria	Moulay Larbi	Proportion	2017	34.8	50.8	20.5
Algeria	Moulay Larbi	Count	2000	0	0	0
Algeria	Moulay Larbi	Count	2017	0	1	0
Algeria	Ain Oulmane	Proportion	2000	32.6	45.1	19.9
Algeria	Ain Oulmane	Proportion	2017	36.9	53.5	21.8
Algeria	Ain Oulmane	Count	2000	0	0	0
Algeria	Ain Oulmane	Count	2017	0	1	0
Algeria	Belarbi	Proportion	2000	28.1	41.9	16.2
Algeria	Belarbi	Proportion	2017	35.0	50.4	20.2
Algeria	Belarbi	Count	2000	0	0	0
Algeria	Belarbi	Count	2017	0	1	0
Algeria	Beni Bechir	Proportion	2000	33.3	50.2	20.7
Algeria	Beni Bechir	Proportion	2017	37.9	55.3	23.3
Algeria	Beni Bechir	Count	2000	0	1	0
Algeria	Beni Bechir	Count	2017	0	1	0
Algeria	Khemissa	Proportion	2000	33.4	50.8	20.5
Algeria	Khemissa	Proportion	2017	35.7	50.6	23.4
Algeria	Khemissa	Count	2000	0	1	0
Algeria	Khemissa	Count	2017	0	1	0
Algeria	Tamenghasset	Proportion	2000	28.2	41.5	16.8
Algeria	Tamenghasset	Proportion	2017	28.9	42.2	16.9

Table 4: ORS by Unit *(continued)*

Country	Unit	Metric	Year	Mean	Upper	Lower
Algeria	Tamenghasset	Count	2000	0	0	0
Algeria	Tamenghasset	Count	2017	0	0	0
Algeria	Boulhaf Dyr	Proportion	2000	30.6	46.5	17.3
Algeria	Boulhaf Dyr	Proportion	2017	36.3	51.4	22.0
Algeria	Boulhaf Dyr	Count	2000	0	0	0
Algeria	Boulhaf Dyr	Count	2017	0	1	0
Algeria	Dahmouni	Proportion	2000	32.1	47.4	19.4
Algeria	Dahmouni	Proportion	2017	34.9	50.0	22.1
Algeria	Dahmouni	Count	2000	0	0	0
Algeria	Dahmouni	Count	2017	0	0	0
Algeria	Cherchel	Proportion	2000	31.3	44.1	20.2
Algeria	Cherchel	Proportion	2017	32.6	48.2	20.3
Algeria	Cherchel	Count	2000	0	0	0
Algeria	Cherchel	Count	2017	0	0	0
Algeria	Khemisti	Proportion	2000	33.6	49.5	20.3
Algeria	Khemisti	Proportion	2017	35.5	50.7	20.1
Algeria	Khemisti	Count	2000	0	0	0
Algeria	Khemisti	Count	2017	0	1	0
Algeria	Ait-Yahia	Proportion	2000	28.8	45.9	17.4
Algeria	Ait-Yahia	Proportion	2017	35.2	49.4	22.6
Algeria	Ait-Yahia	Count	2000	0	0	0
Algeria	Ait-Yahia	Count	2017	0	0	0
Algeria	Amieur	Proportion	2000	29.7	44.7	17.6
Algeria	Amieur	Proportion	2017	35.2	50.9	22.0
Algeria	Amieur	Count	2000	0	0	0
Algeria	Amieur	Count	2017	0	1	0
Algeria	Fenoughil	Proportion	2000	28.5	41.7	17.3
Algeria	Fenoughil	Proportion	2017	30.5	43.4	19.8
Algeria	Fenoughil	Count	2000	0	0	0
Algeria	Fenoughil	Count	2017	0	0	0
Algeria	Bathia	Proportion	2000	32.6	47.8	20.0
Algeria	Bathia	Proportion	2017	34.5	49.6	19.6
Algeria	Bathia	Count	2000	0	0	0
Algeria	Bathia	Count	2017	0	0	0
Algeria	Chaabet El Ham	Proportion	2000	29.0	43.8	17.1
Algeria	Chaabet El Ham	Proportion	2017	35.0	50.2	21.2
Algeria	Chaabet El Ham	Count	2000	0	0	0
Algeria	Chaabet El Ham	Count	2017	0	1	0
Algeria	Douera	Proportion	2000	31.1	45.9	19.6
Algeria	Douera	Proportion	2017	33.3	48.5	20.0
Algeria	Douera	Count	2000	0	0	0
Algeria	Douera	Count	2017	0	0	0
Algeria	Oued El Aneb	Proportion	2000	33.3	47.8	20.4
Algeria	Oued El Aneb	Proportion	2017	36.4	52.9	23.0
Algeria	Oued El Aneb	Count	2000	0	0	0
Algeria	Oued El Aneb	Count	2017	0	1	0
Algeria	Bitam	Proportion	2000	32.4	48.4	18.9
Algeria	Bitam	Proportion	2017	36.2	51.8	21.9
Algeria	Bitam	Count	2000	0	0	0

Table 4: ORS by Unit *(continued)*

Country	Unit	Metric	Year	Mean	Upper	Lower
Algeria	Bitam	Count	2017	0	1	0
Algeria	Igli	Proportion	2000	32.0	47.7	19.3
Algeria	Igli	Proportion	2017	32.6	45.1	18.1
Algeria	Igli	Count	2000	0	0	0
Algeria	Igli	Count	2017	0	0	0
Algeria	Bejaia	Proportion	2000	30.2	46.6	17.8
Algeria	Bejaia	Proportion	2017	34.2	49.4	21.7
Algeria	Bejaia	Count	2000	0	0	0
Algeria	Bejaia	Count	2017	0	0	0
Algeria	Chouaiba|Ouled Rahma	Proportion	2000	30.8	47.1	19.1
Algeria	Chouaiba|Ouled Rahma	Proportion	2017	31.4	44.5	18.7
Algeria	Chouaiba|Ouled Rahma	Count	2000	0	0	0
Algeria	Chouaiba|Ouled Rahma	Count	2017	0	0	0
Algeria	Bouinan	Proportion	2000	31.4	47.0	19.5
Algeria	Bouinan	Proportion	2017	34.7	50.8	20.9
Algeria	Bouinan	Count	2000	0	0	0
Algeria	Bouinan	Count	2017	0	1	0
Algeria	Colla	Proportion	2000	32.6	46.7	20.6
Algeria	Colla	Proportion	2017	36.3	51.5	22.5
Algeria	Colla	Count	2000	0	0	0
Algeria	Colla	Count	2017	0	1	0
Algeria	Aomar	Proportion	2000	30.5	47.4	18.1
Algeria	Aomar	Proportion	2017	36.1	50.0	22.6
Algeria	Aomar	Count	2000	0	0	0
Algeria	Aomar	Count	2017	0	0	0
Algeria	Boumerdes	Proportion	2000	29.5	46.4	17.3
Algeria	Boumerdes	Proportion	2017	35.3	50.7	21.7
Algeria	Boumerdes	Count	2000	0	0	0
Algeria	Boumerdes	Count	2017	0	1	0
Algeria	Breira	Proportion	2000	30.0	43.3	18.1
Algeria	Breira	Proportion	2017	33.3	48.7	19.9
Algeria	Breira	Count	2000	0	0	0
Algeria	Breira	Count	2017	0	0	0
Algeria	Hamma Bouziane	Proportion	2000	33.9	49.4	20.4
Algeria	Hamma Bouziane	Proportion	2017	37.1	55.2	22.2
Algeria	Hamma Bouziane	Count	2000	0	0	0
Algeria	Hamma Bouziane	Count	2017	0	1	0
Algeria	Birine	Proportion	2000	31.3	46.9	17.7
Algeria	Birine	Proportion	2017	32.9	47.1	20.7
Algeria	Birine	Count	2000	0	0	0
Algeria	Birine	Count	2017	0	0	0
Algeria	El Bayadh	Proportion	2000	30.6	46.8	17.4
Algeria	El Bayadh	Proportion	2017	33.2	48.3	19.1
Algeria	El Bayadh	Count	2000	0	0	0
Algeria	El Bayadh	Count	2017	0	0	0
Algeria	Guemar	Proportion	2000	30.9	47.7	18.0
Algeria	Guemar	Proportion	2017	29.9	45.3	17.3
Algeria	Guemar	Count	2000	0	0	0
Algeria	Guemar	Count	2017	0	0	0

Table 4: ORS by Unit *(continued)*

Country	Unit	Metric	Year	Mean	Upper	Lower
Algeria	Bouteldja	Proportion	2000	32.5	47.7	19.5
Algeria	Bouteldja	Proportion	2017	36.3	52.0	23.4
Algeria	Bouteldja	Count	2000	0	0	0
Algeria	Bouteldja	Count	2017	0	1	0
Algeria	Hassi Gara	Proportion	2000	31.1	45.4	19.1
Algeria	Hassi Gara	Proportion	2017	30.7	42.3	20.1
Algeria	Hassi Gara	Count	2000	0	0	0
Algeria	Hassi Gara	Count	2017	0	0	0
Algeria	Bordj Sebbat	Proportion	2000	34.3	49.0	21.6
Algeria	Bordj Sebbat	Proportion	2017	37.1	52.6	24.1
Algeria	Bordj Sebbat	Count	2000	0	0	0
Algeria	Bordj Sebbat	Count	2017	0	1	0
Algeria	El Ancer	Proportion	2000	31.8	45.1	19.5
Algeria	El Ancer	Proportion	2017	37.3	53.8	23.7
Algeria	El Ancer	Count	2000	0	0	0
Algeria	El Ancer	Count	2017	0	1	0
Algeria	El Mahmal	Proportion	2000	30.3	46.8	18.7
Algeria	El Mahmal	Proportion	2017	35.3	51.1	21.0
Algeria	El Mahmal	Count	2000	0	0	0
Algeria	El Mahmal	Count	2017	0	1	0
Algeria	Gueltat Sidi Saad	Proportion	2000	30.3	45.7	16.6
Algeria	Gueltat Sidi Saad	Proportion	2017	32.9	46.7	21.4
Algeria	Gueltat Sidi Saad	Count	2000	0	0	0
Algeria	Gueltat Sidi Saad	Count	2017	0	0	0
Algeria	Benzouh	Proportion	2000	32.1	47.9	18.7
Algeria	Benzouh	Proportion	2017	34.1	49.1	21.2
Algeria	Benzouh	Count	2000	0	0	0
Algeria	Benzouh	Count	2017	0	0	0
Algeria	Bouhanifia	Proportion	2000	26.9	40.7	15.6
Algeria	Bouhanifia	Proportion	2017	34.7	50.3	20.8
Algeria	Bouhanifia	Count	2000	0	0	0
Algeria	Bouhanifia	Count	2017	0	1	0
Algeria	Berrouaghia	Proportion	2000	33.5	49.9	20.6
Algeria	Berrouaghia	Proportion	2017	35.0	49.5	21.7
Algeria	Berrouaghia	Count	2000	0	0	0
Algeria	Berrouaghia	Count	2017	0	0	0
Algeria	Cheraga	Proportion	2000	31.5	44.1	19.4
Algeria	Cheraga	Proportion	2017	36.9	53.4	23.5
Algeria	Cheraga	Count	2000	0	0	0
Algeria	Cheraga	Count	2017	0	1	0
Algeria	Hadjadj	Proportion	2000	26.6	43.2	15.8
Algeria	Hadjadj	Proportion	2017	36.3	52.0	22.3
Algeria	Hadjadj	Count	2000	0	0	0
Algeria	Hadjadj	Count	2017	0	1	0
Algeria	Moghrar	Proportion	2000	32.4	47.2	22.5
Algeria	Moghrar	Proportion	2017	34.3	50.6	21.4
Algeria	Moghrar	Count	2000	0	0	0
Algeria	Moghrar	Count	2017	0	1	0
Algeria	Bousfer	Proportion	2000	27.6	41.7	15.4

Table 4: ORS by Unit *(continued)*

Country	Unit	Metric	Year	Mean	Upper	Lower
Algeria	Bousfer	Proportion	2017	34.9	50.6	21.3
Algeria	Bousfer	Count	2000	0	0	0
Algeria	Bousfer	Count	2017	0	1	0
Algeria	M'Naguer	Proportion	2000	30.2	43.9	19.3
Algeria	M'Naguer	Proportion	2017	31.1	44.7	19.3
Algeria	M'Naguer	Count	2000	0	0	0
Algeria	M'Naguer	Count	2017	0	0	0
Algeria	Berriche	Proportion	2000	32.2	47.1	20.0
Algeria	Berriche	Proportion	2017	36.5	51.3	21.7
Algeria	Berriche	Count	2000	0	0	0
Algeria	Berriche	Count	2017	0	1	0
Algeria	Djidiouia	Proportion	2000	27.4	41.9	16.1
Algeria	Djidiouia	Proportion	2017	35.4	51.0	21.1
Algeria	Djidiouia	Count	2000	0	0	0
Algeria	Djidiouia	Count	2017	0	1	0
Algeria	Ouled Brahim	Proportion	2000	30.7	45.4	19.1
Algeria	Ouled Brahim	Proportion	2017	36.1	53.2	22.6
Algeria	Ouled Brahim	Count	2000	0	0	0
Algeria	Ouled Brahim	Count	2017	0	1	0
Algeria	Ain Roua	Proportion	2000	33.0	46.4	20.2
Algeria	Ain Roua	Proportion	2017	37.0	53.4	22.5
Algeria	Ain Roua	Count	2000	0	0	0
Algeria	Ain Roua	Count	2017	0	1	0
Algeria	Ben Badis	Proportion	2000	29.6	45.1	16.1
Algeria	Ben Badis	Proportion	2017	35.0	50.8	22.0
Algeria	Ben Badis	Count	2000	0	0	0
Algeria	Ben Badis	Count	2017	0	1	0
Algeria	Beni Oulbane	Proportion	2000	33.9	49.3	20.5
Algeria	Beni Oulbane	Proportion	2017	39.1	55.0	24.4
Algeria	Beni Oulbane	Count	2000	0	0	0
Algeria	Beni Oulbane	Count	2017	0	1	0
Algeria	M'Daourouche	Proportion	2000	32.4	48.8	19.5
Algeria	M'Daourouche	Proportion	2017	37.4	53.9	24.6
Algeria	M'Daourouche	Count	2000	0	0	0
Algeria	M'Daourouche	Count	2017	0	1	0
Algeria	Tazrouk	Proportion	2000	30.0	40.2	21.6
Algeria	Tazrouk	Proportion	2017	31.3	41.3	22.2
Algeria	Tazrouk	Count	2000	0	0	0
Algeria	Tazrouk	Count	2017	0	0	0
Algeria	Chrea	Proportion	2000	30.6	46.8	16.9
Algeria	Chrea	Proportion	2017	36.0	52.7	23.9
Algeria	Chrea	Count	2000	0	0	0
Algeria	Chrea	Count	2017	0	1	0
Algeria	Djebilet Rosfa	Proportion	2000	30.5	44.3	17.9
Algeria	Djebilet Rosfa	Proportion	2017	35.7	52.7	22.3
Algeria	Djebilet Rosfa	Count	2000	0	0	0
Algeria	Djebilet Rosfa	Count	2017	0	1	0
Algeria	Damous	Proportion	2000	30.1	45.0	18.6
Algeria	Damous	Proportion	2017	33.5	47.4	20.5

Table 4: ORS by Unit *(continued)*

Country	Unit	Metric	Year	Mean	Upper	Lower
Algeria	Damous	Count	2000	0	0	0
Algeria	Damous	Count	2017	0	0	0
Algeria	Larbaa	Proportion	2000	30.0	44.0	17.5
Algeria	Larbaa	Proportion	2017	34.4	49.6	20.9
Algeria	Larbaa	Count	2000	0	0	0
Algeria	Larbaa	Count	2017	0	0	0
Algeria	Ait Aggouacha	Proportion	2000	28.0	45.7	17.0
Algeria	Ait Aggouacha	Proportion	2017	35.0	48.6	21.4
Algeria	Ait Aggouacha	Count	2000	0	0	0
Algeria	Ait Aggouacha	Count	2017	0	0	0
Algeria	Azails	Proportion	2000	30.5	47.7	18.0
Algeria	Azails	Proportion	2017	34.9	50.3	20.6
Algeria	Azails	Count	2000	0	0	0
Algeria	Azails	Count	2017	0	1	0
Algeria	In Zghmir	Proportion	2000	28.4	41.0	16.8
Algeria	In Zghmir	Proportion	2017	31.0	44.7	21.3
Algeria	In Zghmir	Count	2000	0	0	0
Algeria	In Zghmir	Count	2017	0	0	0
Algeria	Belaas	Proportion	2000	31.7	47.9	18.7
Algeria	Belaas	Proportion	2017	34.4	49.3	19.6
Algeria	Belaas	Count	2000	0	0	0
Algeria	Belaas	Count	2017	0	0	0
Algeria	Chentouf	Proportion	2000	28.4	42.4	16.6
Algeria	Chentouf	Proportion	2017	34.5	49.2	21.0
Algeria	Chentouf	Count	2000	0	0	0
Algeria	Chentouf	Count	2017	0	0	0
Algeria	Draria	Proportion	2000	30.5	45.6	18.8
Algeria	Draria	Proportion	2017	30.9	47.4	17.5
Algeria	Draria	Count	2000	0	0	0
Algeria	Draria	Count	2017	0	0	0
Algeria	Seraidi	Proportion	2000	33.3	48.4	20.2
Algeria	Seraidi	Proportion	2017	35.7	53.3	21.7
Algeria	Seraidi	Count	2000	0	0	0
Algeria	Seraidi	Count	2017	0	1	0
Algeria	Boulhilat	Proportion	2000	32.5	46.2	20.2
Algeria	Boulhilat	Proportion	2017	35.7	51.0	21.0
Algeria	Boulhilat	Count	2000	0	0	0
Algeria	Boulhilat	Count	2017	0	1	0
Algeria	Kenadsa	Proportion	2000	33.3	49.3	20.1
Algeria	Kenadsa	Proportion	2017	33.4	49.6	20.0
Algeria	Kenadsa	Count	2000	0	0	0
Algeria	Kenadsa	Count	2017	0	0	0
Algeria	Beni-Mellikeche	Proportion	2000	30.7	46.7	18.2
Algeria	Beni-Mellikeche	Proportion	2017	36.2	51.2	22.5
Algeria	Beni-Mellikeche	Count	2000	0	0	0
Algeria	Beni-Mellikeche	Count	2017	0	1	0
Algeria	Daoussen	Proportion	2000	30.7	46.1	18.6
Algeria	Daoussen	Proportion	2017	31.9	46.5	18.5
Algeria	Daoussen	Count	2000	0	0	0

Table 4: ORS by Unit *(continued)*

Country	Unit	Metric	Year	Mean	Upper	Lower
Algeria	Daoussen	Count	2017	0	0	0
Algeria	Chebli	Proportion	2000	30.7	46.6	19.2
Algeria	Chebli	Proportion	2017	34.9	49.8	20.7
Algeria	Chebli	Count	2000	0	0	0
Algeria	Chebli	Count	2017	0	0	0
Algeria	Djaafra	Proportion	2000	32.9	48.1	20.2
Algeria	Djaafra	Proportion	2017	36.6	51.8	22.6
Algeria	Djaafra	Count	2000	0	0	0
Algeria	Djaafra	Count	2017	0	1	0
Algeria	Ath Mansour Taourirt	Proportion	2000	32.1	48.2	18.4
Algeria	Ath Mansour Taourirt	Proportion	2017	37.8	53.8	23.2
Algeria	Ath Mansour Taourirt	Count	2000	0	0	0
Algeria	Ath Mansour Taourirt	Count	2017	0	1	0
Algeria	Bouzegza Keddara	Proportion	2000	30.1	47.9	17.7
Algeria	Bouzegza Keddara	Proportion	2017	34.8	49.1	21.0
Algeria	Bouzegza Keddara	Count	2000	0	0	0
Algeria	Bouzegza Keddara	Count	2017	0	0	0
Algeria	Chettia	Proportion	2000	29.0	44.8	16.6
Algeria	Chettia	Proportion	2017	35.8	54.2	21.2
Algeria	Chettia	Count	2000	0	0	0
Algeria	Chettia	Count	2017	0	1	0
Algeria	Ibn Ziad	Proportion	2000	34.3	48.6	21.9
Algeria	Ibn Ziad	Proportion	2017	37.8	54.9	24.1
Algeria	Ibn Ziad	Count	2000	0	0	0
Algeria	Ibn Ziad	Count	2017	0	1	0
Algeria	Bouira Lahdab	Proportion	2000	30.9	45.8	16.0
Algeria	Bouira Lahdab	Proportion	2017	32.6	47.4	20.6
Algeria	Bouira Lahdab	Count	2000	0	0	0
Algeria	Bouira Lahdab	Count	2017	0	0	0
Algeria	El Biodh Sidi Cheikh	Proportion	2000	31.5	44.8	18.3
Algeria	El Biodh Sidi Cheikh	Proportion	2017	34.1	49.4	20.4
Algeria	El Biodh Sidi Cheikh	Count	2000	0	0	0
Algeria	El Biodh Sidi Cheikh	Count	2017	0	0	0
Algeria	Hamraia	Proportion	2000	29.7	44.0	18.0
Algeria	Hamraia	Proportion	2017	30.0	43.2	18.3
Algeria	Hamraia	Count	2000	0	0	0
Algeria	Hamraia	Count	2017	0	0	0
Algeria	Chebaita Mokhtar	Proportion	2000	33.6	48.4	20.5
Algeria	Chebaita Mokhtar	Proportion	2017	37.7	55.8	22.6
Algeria	Chebaita Mokhtar	Count	2000	0	0	0
Algeria	Chebaita Mokhtar	Count	2017	0	1	0
Algeria	Mansoura	Proportion	2000	31.3	45.3	18.7
Algeria	Mansoura	Proportion	2017	31.8	44.4	19.5
Algeria	Mansoura	Count	2000	0	0	0
Algeria	Mansoura	Count	2017	0	0	0
Algeria	Bou Hachana	Proportion	2000	33.7	48.7	21.0
Algeria	Bou Hachana	Proportion	2017	36.3	51.1	23.3
Algeria	Bou Hachana	Count	2000	0	0	0
Algeria	Bou Hachana	Count	2017	0	1	0

Table 4: ORS by Unit *(continued)*

Country	Unit	Metric	Year	Mean	Upper	Lower
Algeria	El Aouana	Proportion	2000	31.2	45.2	18.9
Algeria	El Aouana	Proportion	2017	38.1	54.6	25.3
Algeria	El Aouana	Count	2000	0	0	0
Algeria	El Aouana	Count	2017	0	1	0
Algeria	El Oueldja	Proportion	2000	31.1	45.9	19.0
Algeria	El Oueldja	Proportion	2017	36.9	51.8	22.6
Algeria	El Oueldja	Count	2000	0	0	0
Algeria	El Oueldja	Count	2017	0	1	0
Algeria	Hadj Mechri	Proportion	2000	29.9	44.6	17.0
Algeria	Hadj Mechri	Proportion	2017	32.4	45.8	20.8
Algeria	Hadj Mechri	Count	2000	0	0	0
Algeria	Hadj Mechri	Count	2017	0	0	0
Algeria	Berhoum	Proportion	2000	31.8	46.0	18.9
Algeria	Berhoum	Proportion	2017	35.2	50.9	21.3
Algeria	Berhoum	Count	2000	0	0	0
Algeria	Berhoum	Count	2017	0	1	0
Algeria	Chorfa	Proportion	2000	26.0	41.0	15.0
Algeria	Chorfa	Proportion	2017	34.5	49.7	20.1
Algeria	Chorfa	Count	2000	0	0	0
Algeria	Chorfa	Count	2017	0	0	0
Algeria	Bir Ben Laabed	Proportion	2000	34.4	51.9	20.4
Algeria	Bir Ben Laabed	Proportion	2017	36.7	50.7	23.6
Algeria	Bir Ben Laabed	Count	2000	0	1	0
Algeria	Bir Ben Laabed	Count	2017	0	1	0
Algeria	Derradji Bousselah	Proportion	2000	33.5	47.0	20.1
Algeria	Derradji Bousselah	Proportion	2017	37.5	53.7	22.1
Algeria	Derradji Bousselah	Count	2000	0	0	0
Algeria	Derradji Bousselah	Count	2017	0	1	0
Algeria	Hassaine|Beni Yahi	Proportion	2000	25.5	38.8	15.7
Algeria	Hassaine|Beni Yahi	Proportion	2017	34.9	51.2	20.2
Algeria	Hassaine|Beni Yahi	Count	2000	0	0	0
Algeria	Hassaine|Beni Yahi	Count	2017	0	1	0
Algeria	Naama	Proportion	2000	32.8	46.7	20.4
Algeria	Naama	Proportion	2017	34.9	51.4	20.2
Algeria	Naama	Count	2000	0	0	0
Algeria	Naama	Count	2017	0	1	0
Algeria	Boutlelis	Proportion	2000	27.4	40.2	16.1
Algeria	Boutlelis	Proportion	2017	34.2	49.2	20.8
Algeria	Boutlelis	Count	2000	0	0	0
Algeria	Boutlelis	Count	2017	0	0	0
Algeria	Megarine	Proportion	2000	30.2	47.2	18.6
Algeria	Megarine	Proportion	2017	31.3	45.1	18.5
Algeria	Megarine	Count	2000	0	0	0
Algeria	Megarine	Count	2017	0	0	0
Algeria	Bhir El Chergui	Proportion	2000	32.4	47.9	19.6
Algeria	Bhir El Chergui	Proportion	2017	36.7	52.0	23.0
Algeria	Bhir El Chergui	Count	2000	0	0	0
Algeria	Bhir El Chergui	Count	2017	0	1	0
Algeria	El Guettar	Proportion	2000	27.8	42.6	16.0

Table 4: ORS by Unit *(continued)*

Country	Unit	Metric	Year	Mean	Upper	Lower
Algeria	El Guettar	Proportion	2017	35.1	51.8	20.2
Algeria	El Guettar	Count	2000	0	0	0
Algeria	El Guettar	Count	2017	0	1	0
Algeria	Ouled Khaled	Proportion	2000	31.1	47.4	20.0
Algeria	Ouled Khaled	Proportion	2017	35.7	53.2	21.0
Algeria	Ouled Khaled	Count	2000	0	0	0
Algeria	Ouled Khaled	Count	2017	0	1	0
Algeria	Ait-Tizi	Proportion	2000	32.3	46.1	20.3
Algeria	Ait-Tizi	Proportion	2017	36.8	53.2	23.6
Algeria	Ait-Tizi	Count	2000	0	0	0
Algeria	Ait-Tizi	Count	2017	0	1	0
Algeria	Benaicha Chelia	Proportion	2000	28.9	43.9	16.1
Algeria	Benaicha Chelia	Proportion	2017	34.7	51.8	20.6
Algeria	Benaicha Chelia	Count	2000	0	0	0
Algeria	Benaicha Chelia	Count	2017	0	1	0
Algeria	Beni Zid	Proportion	2000	32.5	47.3	20.0
Algeria	Beni Zid	Proportion	2017	37.9	53.3	22.7
Algeria	Beni Zid	Count	2000	0	0	0
Algeria	Beni Zid	Count	2017	0	1	0
Algeria	Machroha	Proportion	2000	33.1	49.5	20.2
Algeria	Machroha	Proportion	2017	35.5	52.7	22.9
Algeria	Machroha	Count	2000	0	0	0
Algeria	Machroha	Count	2017	0	1	0
Algeria	Tin Zaouatine	Proportion	2000	33.6	45.8	23.7
Algeria	Tin Zaouatine	Proportion	2017	32.7	44.2	23.3
Algeria	Tin Zaouatine	Count	2000	0	0	0
Algeria	Tin Zaouatine	Count	2017	0	0	0
Algeria	El Houidjbet	Proportion	2000	33.0	49.5	19.5
Algeria	El Houidjbet	Proportion	2017	38.3	52.8	24.5
Algeria	El Houidjbet	Count	2000	0	0	0
Algeria	El Houidjbet	Count	2017	0	1	0
Algeria	Djillali Ben Ammar	Proportion	2000	30.5	46.7	18.0
Algeria	Djillali Ben Ammar	Proportion	2017	35.4	53.3	21.6
Algeria	Djillali Ben Ammar	Count	2000	0	0	0
Algeria	Djillali Ben Ammar	Count	2017	0	1	0
Algeria	Douaouda	Proportion	2000	30.6	44.8	18.8
Algeria	Douaouda	Proportion	2017	34.6	50.7	20.4
Algeria	Douaouda	Count	2000	0	0	0
Algeria	Douaouda	Count	2017	0	1	0
Algeria	Lardjem	Proportion	2000	31.1	45.4	18.5
Algeria	Lardjem	Proportion	2017	35.5	49.8	20.1
Algeria	Lardjem	Count	2000	0	0	0
Algeria	Lardjem	Count	2017	0	0	0
Algeria	Ait Aissa Mimoun	Proportion	2000	28.5	46.3	16.1
Algeria	Ait Aissa Mimoun	Proportion	2017	34.6	48.5	20.9
Algeria	Ait Aissa Mimoun	Count	2000	0	0	0
Algeria	Ait Aissa Mimoun	Count	2017	0	0	0
Algeria	Bab El Assa	Proportion	2000	29.0	45.5	17.8
Algeria	Bab El Assa	Proportion	2017	35.0	50.8	22.0

Table 4: ORS by Unit *(continued)*

Country	Unit	Metric	Year	Mean	Upper	Lower
Algeria	Bab El Assa	Count	2000	0	0	0
Algeria	Bab El Assa	Count	2017	0	1	0
Algeria	Ksar Kaddour	Proportion	2000	30.6	44.6	20.3
Algeria	Ksar Kaddour	Proportion	2017	30.8	42.1	18.5
Algeria	Ksar Kaddour	Count	2000	0	0	0
Algeria	Ksar Kaddour	Count	2017	0	0	0
Algeria	Ben Allal	Proportion	2000	31.2	45.0	19.6
Algeria	Ben Allal	Proportion	2017	33.5	49.3	18.6
Algeria	Ben Allal	Count	2000	0	0	0
Algeria	Ben Allal	Count	2017	0	0	0
Algeria	El Amria	Proportion	2000	28.1	40.7	17.6
Algeria	El Amria	Proportion	2017	34.4	49.2	21.2
Algeria	El Amria	Count	2000	0	0	0
Algeria	El Amria	Count	2017	0	0	0
Algeria	El Achour	Proportion	2000	30.3	45.5	18.8
Algeria	El Achour	Proportion	2017	30.7	47.1	17.5
Algeria	El Achour	Count	2000	0	0	0
Algeria	El Achour	Count	2017	0	0	0
Algeria	Sidi Amar	Proportion	2000	33.8	48.6	20.6
Algeria	Sidi Amar	Proportion	2017	35.1	52.6	21.2
Algeria	Sidi Amar	Count	2000	0	0	0
Algeria	Sidi Amar	Count	2017	0	1	0
Algeria	Boumegueur	Proportion	2000	32.3	48.6	20.5
Algeria	Boumegueur	Proportion	2017	37.1	53.2	22.6
Algeria	Boumegueur	Count	2000	0	0	0
Algeria	Boumegueur	Count	2017	0	1	0
Algeria	Kerzaz	Proportion	2000	33.2	45.8	21.7
Algeria	Kerzaz	Proportion	2017	33.9	47.0	22.3
Algeria	Kerzaz	Count	2000	0	0	0
Algeria	Kerzaz	Count	2017	0	0	0
Algeria	Beni Dejllil	Proportion	2000	31.4	48.4	19.3
Algeria	Beni Dejllil	Proportion	2017	36.1	50.7	23.0
Algeria	Beni Dejllil	Count	2000	0	0	0
Algeria	Beni Dejllil	Count	2017	0	1	0
Algeria	Djamora	Proportion	2000	30.3	44.7	17.9
Algeria	Djamora	Proportion	2017	34.2	49.3	20.1
Algeria	Djamora	Count	2000	0	0	0
Algeria	Djamora	Count	2017	0	0	0
Algeria	Chiffa	Proportion	2000	30.4	45.3	18.5
Algeria	Chiffa	Proportion	2017	34.2	52.2	20.1
Algeria	Chiffa	Count	2000	0	0	0
Algeria	Chiffa	Count	2017	0	1	0
Algeria	El Ach	Proportion	2000	33.5	48.2	20.4
Algeria	El Ach	Proportion	2017	37.0	53.5	22.9
Algeria	El Ach	Count	2000	0	0	0
Algeria	El Ach	Count	2017	0	1	0
Algeria	Bechloul	Proportion	2000	32.2	49.1	19.1
Algeria	Bechloul	Proportion	2017	37.4	52.9	23.6
Algeria	Bechloul	Count	2000	0	0	0

Table 4: ORS by Unit *(continued)*

Country	Unit	Metric	Year	Mean	Upper	Lower
Algeria	Bechloul	Count	2017	0	1	0
Algeria	Chabet El Ameur	Proportion	2000	30.1	49.5	17.7
Algeria	Chabet El Ameur	Proportion	2017	35.5	50.1	21.9
Algeria	Chabet El Ameur	Count	2000	0	0	0
Algeria	Chabet El Ameur	Count	2017	0	1	0
Algeria	Chlef	Proportion	2000	29.5	46.5	16.5
Algeria	Chlef	Proportion	2017	33.1	51.2	18.8
Algeria	Chlef	Count	2000	0	0	0
Algeria	Chlef	Count	2017	0	1	0
Algeria	Ouled Rahmoune	Proportion	2000	34.3	48.6	20.6
Algeria	Ouled Rahmoune	Proportion	2017	38.3	54.0	23.2
Algeria	Ouled Rahmoune	Count	2000	0	0	0
Algeria	Ouled Rahmoune	Count	2017	0	1	0
Algeria	Charef	Proportion	2000	30.4	48.1	17.2
Algeria	Charef	Proportion	2017	31.9	47.2	20.3
Algeria	Charef	Count	2000	0	0	0
Algeria	Charef	Count	2017	0	0	0
Algeria	El Bnoud	Proportion	2000	31.9	43.3	21.2
Algeria	El Bnoud	Proportion	2017	32.4	46.2	21.0
Algeria	El Bnoud	Count	2000	0	0	0
Algeria	El Bnoud	Count	2017	0	0	0
Algeria	Hassani Abdelkrim	Proportion	2000	30.3	47.1	17.1
Algeria	Hassani Abdelkrim	Proportion	2017	29.7	45.4	17.0
Algeria	Hassani Abdelkrim	Count	2000	0	0	0
Algeria	Hassani Abdelkrim	Count	2017	0	0	0
Algeria	Chefia	Proportion	2000	33.4	48.9	20.7
Algeria	Chefia	Proportion	2017	36.6	51.9	23.1
Algeria	Chefia	Count	2000	0	0	0
Algeria	Chefia	Count	2017	0	1	0
Algeria	Metlili	Proportion	2000	31.1	46.5	18.7
Algeria	Metlili	Proportion	2017	31.2	43.0	18.8
Algeria	Metlili	Count	2000	0	0	0
Algeria	Metlili	Count	2017	0	0	0
Algeria	Bou Hamdane	Proportion	2000	34.3	48.9	21.4
Algeria	Bou Hamdane	Proportion	2017	37.7	53.2	24.0
Algeria	Bou Hamdane	Count	2000	0	0	0
Algeria	Bou Hamdane	Count	2017	0	1	0
Algeria	El Kennar Nouchfi	Proportion	2000	31.4	46.1	19.3
Algeria	El Kennar Nouchfi	Proportion	2017	37.7	54.3	24.2
Algeria	El Kennar Nouchfi	Count	2000	0	0	0
Algeria	El Kennar Nouchfi	Count	2017	0	1	0
Algeria	Ensigha	Proportion	2000	29.9	45.3	17.5
Algeria	Ensigha	Proportion	2017	35.8	51.1	20.6
Algeria	Ensigha	Count	2000	0	0	0
Algeria	Ensigha	Count	2017	0	1	0
Algeria	Hassi Delaa	Proportion	2000	29.8	43.3	19.7
Algeria	Hassi Delaa	Proportion	2017	31.6	46.2	20.5
Algeria	Hassi Delaa	Count	2000	0	0	0
Algeria	Hassi Delaa	Count	2017	0	0	0

Table 4: ORS by Unit *(continued)*

Country	Unit	Metric	Year	Mean	Upper	Lower
Algeria	Bir Foda	Proportion	2000	31.6	45.2	19.3
Algeria	Bir Foda	Proportion	2017	33.9	48.8	20.3
Algeria	Bir Foda	Count	2000	0	0	0
Algeria	Bir Foda	Count	2017	0	0	0
Algeria	El Bordj	Proportion	2000	27.0	42.4	16.1
Algeria	El Bordj	Proportion	2017	34.6	49.9	20.0
Algeria	El Bordj	Count	2000	0	0	0
Algeria	El Bordj	Count	2017	0	0	0
Algeria	Bouaiche	Proportion	2000	33.5	49.7	20.8
Algeria	Bouaiche	Proportion	2017	36.4	51.2	23.4
Algeria	Bouaiche	Count	2000	0	0	0
Algeria	Bouaiche	Count	2017	0	1	0
Algeria	El Mechira	Proportion	2000	33.7	48.0	20.5
Algeria	El Mechira	Proportion	2017	36.8	51.9	21.4
Algeria	El Mechira	Count	2000	0	0	0
Algeria	El Mechira	Count	2017	0	1	0
Algeria	Hassi Mameche	Proportion	2000	25.6	38.8	15.6
Algeria	Hassi Mameche	Proportion	2017	35.6	51.6	21.1
Algeria	Hassi Mameche	Count	2000	0	0	0
Algeria	Hassi Mameche	Count	2017	0	1	0
Algeria	Sfissifa	Proportion	2000	31.2	44.6	20.1
Algeria	Sfissifa	Proportion	2017	33.3	48.9	19.8
Algeria	Sfissifa	Count	2000	0	0	0
Algeria	Sfissifa	Count	2017	0	0	0
Algeria	El Ançar	Proportion	2000	27.2	40.5	15.6
Algeria	El Ançar	Proportion	2017	34.3	48.8	21.3
Algeria	El Ançar	Count	2000	0	0	0
Algeria	El Ançar	Count	2017	0	0	0
Algeria	N'Goussa	Proportion	2000	29.9	44.4	17.5
Algeria	N'Goussa	Proportion	2017	30.3	44.0	19.1
Algeria	N'Goussa	Count	2000	0	0	0
Algeria	N'Goussa	Count	2017	0	0	0
Algeria	Bir Chouhada	Proportion	2000	33.5	46.9	19.0
Algeria	Bir Chouhada	Proportion	2017	37.0	52.3	22.3
Algeria	Bir Chouhada	Count	2000	0	0	0
Algeria	Bir Chouhada	Count	2017	0	1	0
Algeria	El H'Madna	Proportion	2000	27.5	43.1	16.7
Algeria	El H'Madna	Proportion	2017	35.6	51.6	21.3
Algeria	El H'Madna	Count	2000	0	0	0
Algeria	El H'Madna	Count	2017	0	1	0
Algeria	Saida	Proportion	2000	31.3	48.4	20.0
Algeria	Saida	Proportion	2017	34.5	51.4	19.5
Algeria	Saida	Count	2000	0	0	0
Algeria	Saida	Count	2017	0	1	0
Algeria	Ait Naoual Mezada	Proportion	2000	32.0	46.0	19.9
Algeria	Ait Naoual Mezada	Proportion	2017	36.5	52.2	23.1
Algeria	Ait Naoual Mezada	Count	2000	0	0	0
Algeria	Ait Naoual Mezada	Count	2017	0	1	0
Algeria	Bir El Hammam	Proportion	2000	31.9	46.1	19.3

Table 4: ORS by Unit *(continued)*

Country	Unit	Metric	Year	Mean	Upper	Lower
Algeria	Bir El Hammam	Proportion	2017	34.1	47.5	21.1
Algeria	Bir El Hammam	Count	2000	0	0	0
Algeria	Bir El Hammam	Count	2017	0	0	0
Algeria	Bin El Ouiden	Proportion	2000	32.7	47.3	19.8
Algeria	Bin El Ouiden	Proportion	2017	37.1	52.6	22.2
Algeria	Bin El Ouiden	Count	2000	0	0	0
Algeria	Bin El Ouiden	Count	2017	0	1	0
Algeria	Merahna	Proportion	2000	32.6	48.0	20.4
Algeria	Merahna	Proportion	2017	35.2	52.1	22.2
Algeria	Merahna	Count	2000	0	0	0
Algeria	Merahna	Count	2017	0	1	0
Algeria	El Kouif	Proportion	2000	30.8	45.2	17.8
Algeria	El Kouif	Proportion	2017	35.9	49.4	22.1
Algeria	El Kouif	Count	2000	0	0	0
Algeria	El Kouif	Count	2017	0	0	0
Algeria	Faidja	Proportion	2000	31.3	44.2	19.8
Algeria	Faidja	Proportion	2017	35.0	49.4	23.5
Algeria	Faidja	Count	2000	0	0	0
Algeria	Faidja	Count	2017	0	0	0
Algeria	Fouka	Proportion	2000	30.6	44.8	18.6
Algeria	Fouka	Proportion	2017	32.7	50.2	19.3
Algeria	Fouka	Count	2000	0	0	0
Algeria	Fouka	Count	2017	0	1	0
Algeria	Layoune	Proportion	2000	33.4	50.2	20.1
Algeria	Layoune	Proportion	2017	35.7	50.1	20.2
Algeria	Layoune	Count	2000	0	1	0
Algeria	Layoune	Count	2017	0	1	0
Algeria	Ait Bouadou	Proportion	2000	28.7	45.7	16.0
Algeria	Ait Bouadou	Proportion	2017	34.5	49.6	20.9
Algeria	Ait Bouadou	Count	2000	0	0	0
Algeria	Ait Bouadou	Count	2017	0	0	0
Algeria	Beni Bahdel	Proportion	2000	29.9	46.5	17.7
Algeria	Beni Bahdel	Proportion	2017	34.8	50.3	19.9
Algeria	Beni Bahdel	Count	2000	0	0	0
Algeria	Beni Bahdel	Count	2017	0	1	0
Algeria	Metarfa	Proportion	2000	29.3	42.8	17.5
Algeria	Metarfa	Proportion	2017	30.4	45.4	17.9
Algeria	Metarfa	Count	2000	0	0	0
Algeria	Metarfa	Count	2017	0	0	0
Algeria	Bir Ould Khelifa	Proportion	2000	31.4	44.7	20.0
Algeria	Bir Ould Khelifa	Proportion	2017	33.9	48.8	19.7
Algeria	Bir Ould Khelifa	Count	2000	0	0	0
Algeria	Bir Ould Khelifa	Count	2017	0	0	0
Algeria	El Malah	Proportion	2000	28.7	43.1	17.0
Algeria	El Malah	Proportion	2017	34.9	49.9	21.1
Algeria	El Malah	Count	2000	0	0	0
Algeria	El Malah	Count	2017	0	0	0
Algeria	Haraoua	Proportion	2000	30.5	47.6	17.9
Algeria	Haraoua	Proportion	2017	31.9	48.1	19.4

Table 4: ORS by Unit *(continued)*

Country	Unit	Metric	Year	Mean	Upper	Lower
Algeria	Haraoua	Count	2000	0	0	0
Algeria	Haraoua	Count	2017	0	0	0
Algeria	Treat	Proportion	2000	32.6	48.2	20.1
Algeria	Treat	Proportion	2017	35.9	52.3	23.0
Algeria	Treat	Count	2000	0	0	0
Algeria	Treat	Count	2017	0	1	0
Algeria	Boumia	Proportion	2000	32.2	46.9	19.3
Algeria	Boumia	Proportion	2017	36.6	52.5	21.8
Algeria	Boumia	Count	2000	0	0	0
Algeria	Boumia	Count	2017	0	1	0
Algeria	Ksabi	Proportion	2000	32.1	49.4	19.5
Algeria	Ksabi	Proportion	2017	33.4	47.7	20.6
Algeria	Ksabi	Count	2000	0	0	0
Algeria	Ksabi	Count	2017	0	0	0
Algeria	Beni K'Sila	Proportion	2000	29.8	46.5	18.7
Algeria	Beni K'Sila	Proportion	2017	35.3	50.1	21.6
Algeria	Beni K'Sila	Count	2000	0	0	0
Algeria	Beni K'Sila	Count	2017	0	1	0
Algeria	El Feidh	Proportion	2000	28.6	42.8	16.1
Algeria	El Feidh	Proportion	2017	31.5	44.9	19.2
Algeria	El Feidh	Count	2000	0	0	0
Algeria	El Feidh	Count	2017	0	0	0
Algeria	Chrea	Proportion	2000	31.0	45.8	19.0
Algeria	Chrea	Proportion	2017	32.4	49.9	19.2
Algeria	Chrea	Count	2000	0	0	0
Algeria	Chrea	Count	2017	0	0	0
Algeria	El Achir	Proportion	2000	33.8	49.0	21.4
Algeria	El Achir	Proportion	2017	37.2	52.4	22.2
Algeria	El Achir	Count	2000	0	0	0
Algeria	El Achir	Count	2017	0	1	0
Algeria	Bir Ghbalou	Proportion	2000	33.6	50.6	19.6
Algeria	Bir Ghbalou	Proportion	2017	36.4	51.5	23.5
Algeria	Bir Ghbalou	Count	2000	0	1	0
Algeria	Bir Ghbalou	Count	2017	0	1	0
Algeria	Corso	Proportion	2000	30.1	47.5	17.7
Algeria	Corso	Proportion	2017	32.9	48.3	19.7
Algeria	Corso	Count	2000	0	0	0
Algeria	Corso	Count	2017	0	0	0
Algeria	Dahra	Proportion	2000	27.3	43.0	15.5
Algeria	Dahra	Proportion	2017	35.0	51.3	20.2
Algeria	Dahra	Count	2000	0	0	0
Algeria	Dahra	Count	2017	0	1	0
Algeria	Zighoud Youcef	Proportion	2000	34.4	48.2	21.5
Algeria	Zighoud Youcef	Proportion	2017	38.4	54.4	23.9
Algeria	Zighoud Youcef	Count	2000	0	0	0
Algeria	Zighoud Youcef	Count	2017	0	1	0
Algeria	Dar Chioukh	Proportion	2000	31.8	47.2	18.0
Algeria	Dar Chioukh	Proportion	2017	33.2	47.3	20.7
Algeria	Dar Chioukh	Count	2000	0	0	0

Table 4: ORS by Unit *(continued)*

Country	Unit	Metric	Year	Mean	Upper	Lower
Algeria	Dar Chioukh	Count	2017	0	0	0
Algeria	El Kheither	Proportion	2000	30.6	44.1	18.3
Algeria	El Kheither	Proportion	2017	33.8	49.5	20.3
Algeria	El Kheither	Count	2000	0	0	0
Algeria	El Kheither	Count	2017	0	0	0
Algeria	Hassi Khalifa	Proportion	2000	29.5	44.5	17.3
Algeria	Hassi Khalifa	Proportion	2017	31.3	46.7	17.8
Algeria	Hassi Khalifa	Count	2000	0	0	0
Algeria	Hassi Khalifa	Count	2017	0	0	0
Algeria	Chihani	Proportion	2000	33.6	48.3	20.1
Algeria	Chihani	Proportion	2017	37.4	53.6	23.2
Algeria	Chihani	Count	2000	0	0	0
Algeria	Chihani	Count	2017	0	1	0
Algeria	Sebseb	Proportion	2000	31.3	46.0	19.1
Algeria	Sebseb	Proportion	2017	32.1	43.8	20.6
Algeria	Sebseb	Count	2000	0	0	0
Algeria	Sebseb	Count	2017	0	0	0
Algeria	Bouati Mahmoud	Proportion	2000	34.9	50.4	22.0
Algeria	Bouati Mahmoud	Proportion	2017	38.0	54.9	24.5
Algeria	Bouati Mahmoud	Count	2000	0	1	0
Algeria	Bouati Mahmoud	Count	2017	0	1	0
Algeria	El Milia	Proportion	2000	32.0	45.2	19.9
Algeria	El Milia	Proportion	2017	38.1	54.6	24.0
Algeria	El Milia	Count	2000	0	0	0
Algeria	El Milia	Count	2017	0	1	0
Algeria	Kais	Proportion	2000	30.2	44.0	18.0
Algeria	Kais	Proportion	2017	36.3	51.8	20.9
Algeria	Kais	Count	2000	0	0	0
Algeria	Kais	Count	2017	0	1	0
Algeria	Hassi R'Mel	Proportion	2000	30.0	43.4	18.2
Algeria	Hassi R'Mel	Proportion	2017	32.3	47.0	19.8
Algeria	Hassi R'Mel	Count	2000	0	0	0
Algeria	Hassi R'Mel	Count	2017	0	0	0
Algeria	Bou Saada	Proportion	2000	32.2	47.3	19.6
Algeria	Bou Saada	Proportion	2017	33.4	48.3	19.8
Algeria	Bou Saada	Count	2000	0	0	0
Algeria	Bou Saada	Count	2017	0	0	0
Algeria	El Gaada	Proportion	2000	26.4	41.3	15.1
Algeria	El Gaada	Proportion	2017	34.4	49.6	20.6
Algeria	El Gaada	Count	2000	0	0	0
Algeria	El Gaada	Count	2017	0	0	0
Algeria	Bouaichoune	Proportion	2000	33.4	49.5	21.7
Algeria	Bouaichoune	Proportion	2017	34.5	49.2	21.5
Algeria	Bouaichoune	Count	2000	0	0	0
Algeria	Bouaichoune	Count	2017	0	0	0
Algeria	Elayadi Barbes	Proportion	2000	33.0	46.8	20.2
Algeria	Elayadi Barbes	Proportion	2017	37.3	52.9	21.9
Algeria	Elayadi Barbes	Count	2000	0	0	0
Algeria	Elayadi Barbes	Count	2017	0	1	0

Table 4: ORS by Unit *(continued)*

Country	Unit	Metric	Year	Mean	Upper	Lower
Algeria	Khadra	Proportion	2000	26.8	44.2	15.1
Algeria	Khadra	Proportion	2017	36.0	51.0	22.4
Algeria	Khadra	Count	2000	0	0	0
Algeria	Khadra	Count	2017	0	1	0
Algeria	Tiout	Proportion	2000	32.8	46.6	21.1
Algeria	Tiout	Proportion	2017	35.1	51.8	20.8
Algeria	Tiout	Count	2000	0	0	0
Algeria	Tiout	Count	2017	0	1	0
Algeria	El Braya	Proportion	2000	26.5	39.2	16.1
Algeria	El Braya	Proportion	2017	34.7	49.6	21.0
Algeria	El Braya	Count	2000	0	0	0
Algeria	El Braya	Count	2017	0	0	0
Algeria	Nezla	Proportion	2000	29.7	46.7	18.0
Algeria	Nezla	Proportion	2017	29.7	43.0	17.7
Algeria	Nezla	Count	2000	0	0	0
Algeria	Nezla	Count	2017	0	0	0
Algeria	El Amiria	Proportion	2000	34.3	50.5	20.9
Algeria	El Amiria	Proportion	2017	36.8	50.9	23.6
Algeria	El Amiria	Count	2000	0	1	0
Algeria	El Amiria	Count	2017	0	1	0
Algeria	El Hassi	Proportion	2000	30.4	47.8	17.8
Algeria	El Hassi	Proportion	2017	35.6	52.0	21.8
Algeria	El Hassi	Count	2000	0	0	0
Algeria	El Hassi	Count	2017	0	1	0
Algeria	Sidi Ahmed	Proportion	2000	30.6	45.7	18.1
Algeria	Sidi Ahmed	Proportion	2017	35.5	53.7	21.0
Algeria	Sidi Ahmed	Count	2000	0	0	0
Algeria	Sidi Ahmed	Count	2017	0	1	0
Algeria	Amoucha	Proportion	2000	33.3	46.8	20.0
Algeria	Amoucha	Proportion	2017	37.5	53.8	22.3
Algeria	Amoucha	Count	2000	0	0	0
Algeria	Amoucha	Count	2017	0	1	0
Algeria	Boudjebaa El Bordj	Proportion	2000	26.8	41.9	15.2
Algeria	Boudjebaa El Bordj	Proportion	2017	34.7	49.2	20.8
Algeria	Boudjebaa El Bordj	Count	2000	0	0	0
Algeria	Boudjebaa El Bordj	Count	2017	0	0	0
Algeria	Bouchetata	Proportion	2000	32.6	48.1	20.1
Algeria	Bouchetata	Proportion	2017	38.1	53.6	23.5
Algeria	Bouchetata	Count	2000	0	0	0
Algeria	Bouchetata	Count	2017	0	1	0
Algeria	Oued Kebrit	Proportion	2000	32.6	48.7	19.7
Algeria	Oued Kebrit	Proportion	2017	36.6	53.3	23.9
Algeria	Oued Kebrit	Count	2000	0	0	0
Algeria	Oued Kebrit	Count	2017	0	1	0
Algeria	El Malabiodh	Proportion	2000	30.8	46.0	16.0
Algeria	El Malabiodh	Proportion	2017	35.8	49.6	22.6
Algeria	El Malabiodh	Count	2000	0	0	0
Algeria	El Malabiodh	Count	2017	0	0	0
Algeria	Frenda	Proportion	2000	31.4	48.5	19.0

Table 4: ORS by Unit *(continued)*

Country	Unit	Metric	Year	Mean	Upper	Lower
Algeria	Frenda	Proportion	2017	37.0	55.3	23.3
Algeria	Frenda	Count	2000	0	0	0
Algeria	Frenda	Count	2017	0	1	0
Algeria	Gouraya	Proportion	2000	30.6	44.4	18.2
Algeria	Gouraya	Proportion	2017	33.6	49.0	21.6
Algeria	Gouraya	Count	2000	0	0	0
Algeria	Gouraya	Count	2017	0	0	0
Algeria	Lazharia	Proportion	2000	31.2	46.0	18.7
Algeria	Lazharia	Proportion	2017	34.5	50.3	19.7
Algeria	Lazharia	Count	2000	0	0	0
Algeria	Lazharia	Count	2017	0	1	0
Algeria	Ait Boumehdi	Proportion	2000	28.9	45.7	16.7
Algeria	Ait Boumehdi	Proportion	2017	34.4	49.6	21.4
Algeria	Ait Boumehdi	Count	2000	0	0	0
Algeria	Ait Boumehdi	Count	2017	0	0	0
Algeria	Beni Boussaid	Proportion	2000	30.4	48.5	18.3
Algeria	Beni Boussaid	Proportion	2017	34.6	49.8	19.4
Algeria	Beni Boussaid	Count	2000	0	0	0
Algeria	Beni Boussaid	Count	2017	0	0	0
Algeria	Ouled Ahmed Temmi	Proportion	2000	28.7	42.7	17.1
Algeria	Ouled Ahmed Temmi	Proportion	2017	28.5	43.2	17.2
Algeria	Ouled Ahmed Temmi	Count	2000	0	0	0
Algeria	Ouled Ahmed Temmi	Count	2017	0	0	0
Algeria	Bordj Emir Khaled	Proportion	2000	32.1	46.2	20.8
Algeria	Bordj Emir Khaled	Proportion	2017	33.8	47.6	20.2
Algeria	Bordj Emir Khaled	Count	2000	0	0	0
Algeria	Bordj Emir Khaled	Count	2017	0	0	0
Algeria	El Messaid	Proportion	2000	27.9	40.4	17.5
Algeria	El Messaid	Proportion	2017	33.7	47.6	20.3
Algeria	El Messaid	Count	2000	0	0	0
Algeria	El Messaid	Count	2017	0	0	0
Algeria	Khraicia	Proportion	2000	30.8	45.7	19.8
Algeria	Khraicia	Proportion	2017	32.2	47.5	18.6
Algeria	Khraicia	Count	2000	0	0	0
Algeria	Khraicia	Count	2017	0	0	0
Algeria	Bouzina	Proportion	2000	31.2	45.8	19.3
Algeria	Bouzina	Proportion	2017	35.8	50.9	21.4
Algeria	Bouzina	Count	2000	0	0	0
Algeria	Bouzina	Count	2017	0	1	0
Algeria	Lahmar	Proportion	2000	33.1	48.6	18.9
Algeria	Lahmar	Proportion	2017	34.0	49.6	20.3
Algeria	Lahmar	Count	2000	0	0	0
Algeria	Lahmar	Count	2017	0	0	0
Algeria	Benimaouche	Proportion	2000	31.8	47.7	19.7
Algeria	Benimaouche	Proportion	2017	36.6	51.8	23.1
Algeria	Benimaouche	Count	2000	0	0	0
Algeria	Benimaouche	Count	2017	0	1	0
Algeria	El Ghrous	Proportion	2000	30.0	44.2	18.1
Algeria	El Ghrous	Proportion	2017	31.9	45.3	18.7

Table 4: ORS by Unit *(continued)*

Country	Unit	Metric	Year	Mean	Upper	Lower
Algeria	El Ghrous	Count	2000	0	0	0
Algeria	El Ghrous	Count	2017	0	0	0
Algeria	Djebabra	Proportion	2000	31.6	47.1	19.5
Algeria	Djebabra	Proportion	2017	37.7	52.8	23.7
Algeria	Djebabra	Count	2000	0	0	0
Algeria	Djebabra	Count	2017	0	1	0
Algeria	El Anseur	Proportion	2000	32.0	47.6	19.6
Algeria	El Anseur	Proportion	2017	35.7	52.5	20.4
Algeria	El Anseur	Count	2000	0	0	0
Algeria	El Anseur	Count	2017	0	1	0
Algeria	Bordj Okhriss	Proportion	2000	33.4	49.1	20.8
Algeria	Bordj Okhriss	Proportion	2017	36.2	51.7	21.7
Algeria	Bordj Okhriss	Count	2000	0	0	0
Algeria	Bordj Okhriss	Count	2017	0	1	0
Algeria	Dellys	Proportion	2000	28.9	46.7	17.0
Algeria	Dellys	Proportion	2017	34.6	48.0	19.7
Algeria	Dellys	Count	2000	0	0	0
Algeria	Dellys	Count	2017	0	0	0
Algeria	El Hadjadj	Proportion	2000	29.7	44.3	17.1
Algeria	El Hadjadj	Proportion	2017	34.5	50.7	20.6
Algeria	El Hadjadj	Count	2000	0	0	0
Algeria	El Hadjadj	Count	2017	0	1	0
Algeria	Deldoul	Proportion	2000	30.7	45.8	17.8
Algeria	Deldoul	Proportion	2017	31.9	47.7	20.9
Algeria	Deldoul	Count	2000	0	0	0
Algeria	Deldoul	Count	2017	0	0	0
Algeria	El Mehara	Proportion	2000	30.1	42.8	18.3
Algeria	El Mehara	Proportion	2017	32.5	45.7	19.2
Algeria	El Mehara	Count	2000	0	0	0
Algeria	El Mehara	Count	2017	0	0	0
Algeria	Kouinine	Proportion	2000	30.8	47.6	18.2
Algeria	Kouinine	Proportion	2017	29.6	45.1	17.5
Algeria	Kouinine	Count	2000	0	0	0
Algeria	Kouinine	Count	2017	0	0	0
Algeria	Drean	Proportion	2000	34.5	50.4	21.0
Algeria	Drean	Proportion	2017	36.3	54.4	21.2
Algeria	Drean	Count	2000	0	1	0
Algeria	Drean	Count	2017	0	1	0
Algeria	Zelfana	Proportion	2000	30.4	45.6	16.9
Algeria	Zelfana	Proportion	2017	30.5	44.6	18.3
Algeria	Zelfana	Count	2000	0	0	0
Algeria	Zelfana	Count	2017	0	0	0
Algeria	Bouchekouf	Proportion	2000	32.7	48.4	20.4
Algeria	Bouchekouf	Proportion	2017	35.5	54.2	21.8
Algeria	Bouchekouf	Count	2000	0	0	0
Algeria	Bouchekouf	Count	2017	0	1	0
Algeria	Emir Abdelkader	Proportion	2000	30.6	45.2	18.1
Algeria	Emir Abdelkader	Proportion	2017	36.2	52.5	23.0
Algeria	Emir Abdelkader	Count	2000	0	0	0

Table 4: ORS by Unit *(continued)*

Country	Unit	Metric	Year	Mean	Upper	Lower
Algeria	Emir Abdelkader	Count	2017	0	1	0
Algeria	Khenchela	Proportion	2000	29.4	44.8	17.2
Algeria	Khenchela	Proportion	2017	33.3	46.7	18.9
Algeria	Khenchela	Count	2000	0	0	0
Algeria	Khenchela	Count	2017	0	0	0
Algeria	Kheneg	Proportion	2000	30.3	45.3	17.7
Algeria	Kheneg	Proportion	2017	33.4	49.2	21.5
Algeria	Kheneg	Count	2000	0	0	0
Algeria	Kheneg	Count	2017	0	0	0
Algeria	Bouti Sayeh	Proportion	2000	31.7	47.0	18.2
Algeria	Bouti Sayeh	Proportion	2017	33.5	48.1	21.2
Algeria	Bouti Sayeh	Count	2000	0	0	0
Algeria	Bouti Sayeh	Count	2017	0	0	0
Algeria	El Ghomri	Proportion	2000	25.6	39.8	15.5
Algeria	El Ghomri	Proportion	2017	34.7	50.4	20.4
Algeria	El Ghomri	Count	2000	0	0	0
Algeria	El Ghomri	Count	2017	0	1	0
Algeria	Boucherahil	Proportion	2000	33.6	50.4	20.5
Algeria	Boucherahil	Proportion	2017	36.1	50.6	22.5
Algeria	Boucherahil	Count	2000	0	1	0
Algeria	Boucherahil	Count	2017	0	1	0
Algeria	Ferdjioua	Proportion	2000	32.9	47.2	19.9
Algeria	Ferdjioua	Proportion	2017	36.8	53.0	21.9
Algeria	Ferdjioua	Count	2000	0	0	0
Algeria	Ferdjioua	Count	2017	0	1	0
Algeria	Kheiredine	Proportion	2000	26.0	39.6	15.5
Algeria	Kheiredine	Proportion	2017	35.3	51.1	21.0
Algeria	Kheiredine	Count	2000	0	0	0
Algeria	Kheiredine	Count	2017	0	1	0
Algeria	El Kerma	Proportion	2000	26.8	39.6	16.2
Algeria	El Kerma	Proportion	2017	34.2	49.8	21.0
Algeria	El Kerma	Count	2000	0	0	0
Algeria	El Kerma	Count	2017	0	0	0
Algeria	Ouargla	Proportion	2000	29.7	43.6	17.2
Algeria	Ouargla	Proportion	2017	29.2	43.5	17.7
Algeria	Ouargla	Count	2000	0	0	0
Algeria	Ouargla	Count	2017	0	0	0
Algeria	El Dhaala	Proportion	2000	31.8	48.6	19.0
Algeria	El Dhaala	Proportion	2017	35.9	53.0	22.7
Algeria	El Dhaala	Count	2000	0	0	0
Algeria	El Dhaala	Count	2017	0	1	0
Algeria	El Matmar	Proportion	2000	26.3	41.4	15.6
Algeria	El Matmar	Proportion	2017	35.4	51.4	20.2
Algeria	El Matmar	Count	2000	0	0	0
Algeria	El Matmar	Count	2017	0	1	0
Algeria	Sidi Amar	Proportion	2000	30.6	46.0	19.1
Algeria	Sidi Amar	Proportion	2017	35.7	52.6	21.9
Algeria	Sidi Amar	Count	2000	0	0	0
Algeria	Sidi Amar	Count	2017	0	1	0

Table 4: ORS by Unit *(continued)*

Country	Unit	Metric	Year	Mean	Upper	Lower
Algeria	Babor	Proportion	2000	33.1	46.6	19.3
Algeria	Babor	Proportion	2017	37.0	53.4	22.6
Algeria	Babor	Count	2000	0	0	0
Algeria	Babor	Count	2017	0	1	0
Algeria	Boukhenifis	Proportion	2000	28.6	43.5	16.6
Algeria	Boukhenifis	Proportion	2017	34.8	50.5	20.1
Algeria	Boukhenifis	Count	2000	0	0	0
Algeria	Boukhenifis	Count	2017	0	1	0
Algeria	Cheraia	Proportion	2000	31.3	46.0	19.0
Algeria	Cheraia	Proportion	2017	36.4	52.7	22.1
Algeria	Cheraia	Count	2000	0	0	0
Algeria	Cheraia	Count	2017	0	1	0
Algeria	Ouillen	Proportion	2000	34.0	50.4	21.1
Algeria	Ouillen	Proportion	2017	36.3	53.9	23.8
Algeria	Ouillen	Count	2000	0	1	0
Algeria	Ouillen	Count	2017	0	1	0
Algeria	El Meridj	Proportion	2000	32.6	48.1	19.8
Algeria	El Meridj	Proportion	2017	37.5	52.7	23.8
Algeria	El Meridj	Count	2000	0	0	0
Algeria	El Meridj	Count	2017	0	1	0
Algeria	Guertoufa	Proportion	2000	32.0	48.2	19.2
Algeria	Guertoufa	Proportion	2017	34.8	50.7	22.1
Algeria	Guertoufa	Count	2000	0	0	0
Algeria	Guertoufa	Count	2017	0	1	0
Algeria	Hadjout	Proportion	2000	30.8	45.1	19.2
Algeria	Hadjout	Proportion	2017	33.7	49.8	20.0
Algeria	Hadjout	Count	2000	0	0	0
Algeria	Hadjout	Count	2017	0	0	0
Algeria	Maacem	Proportion	2000	31.7	46.3	18.7
Algeria	Maacem	Proportion	2017	34.8	50.8	19.2
Algeria	Maacem	Count	2000	0	0	0
Algeria	Maacem	Count	2017	0	1	0
Algeria	Ait Khelili	Proportion	2000	28.3	44.8	17.2
Algeria	Ait Khelili	Proportion	2017	35.0	48.8	21.8
Algeria	Ait Khelili	Count	2000	0	0	0
Algeria	Ait Khelili	Count	2017	0	0	0
Algeria	Beni Khellad	Proportion	2000	29.3	44.7	17.3
Algeria	Beni Khellad	Proportion	2017	35.0	50.3	20.2
Algeria	Beni Khellad	Count	2000	0	0	0
Algeria	Beni Khellad	Count	2017	0	1	0
Algeria	Ouled Aissa	Proportion	2000	29.9	44.8	18.5
Algeria	Ouled Aissa	Proportion	2017	30.8	44.6	19.0
Algeria	Ouled Aissa	Count	2000	0	0	0
Algeria	Ouled Aissa	Count	2017	0	0	0
Algeria	Boumedfaa	Proportion	2000	31.7	46.7	19.8
Algeria	Boumedfaa	Proportion	2017	33.3	46.5	19.9
Algeria	Boumedfaa	Count	2000	0	0	0
Algeria	Boumedfaa	Count	2017	0	0	0
Algeria	Emir Abdelkader	Proportion	2000	28.5	43.1	16.7

Table 4: ORS by Unit *(continued)*

Country	Unit	Metric	Year	Mean	Upper	Lower
Algeria	Emir Abdelkader	Proportion	2017	34.2	48.7	19.8
Algeria	Emir Abdelkader	Count	2000	0	0	0
Algeria	Emir Abdelkader	Count	2017	0	0	0
Algeria	Kouba	Proportion	2000	30.0	44.6	19.3
Algeria	Kouba	Proportion	2017	30.5	46.8	16.7
Algeria	Kouba	Count	2000	0	0	0
Algeria	Kouba	Count	2017	0	0	0
Algeria	Chemora	Proportion	2000	31.8	45.9	19.3
Algeria	Chemora	Proportion	2017	35.6	50.6	20.7
Algeria	Chemora	Count	2000	0	0	0
Algeria	Chemora	Count	2017	0	1	0
Algeria	Mechraa Houari Boumediene	Proportion	2000	33.5	49.7	20.2
Algeria	Mechraa Houari Boumediene	Proportion	2017	34.0	51.0	20.7
Algeria	Mechraa Houari Boumediene	Count	2000	0	0	0
Algeria	Mechraa Houari Boumediene	Count	2017	0	1	0
Algeria	Berbacha	Proportion	2000	31.0	45.7	19.4
Algeria	Berbacha	Proportion	2017	36.5	51.5	23.8
Algeria	Berbacha	Count	2000	0	0	0
Algeria	Berbacha	Count	2017	0	1	0
Algeria	El Hadjab	Proportion	2000	30.7	43.9	18.1
Algeria	El Hadjab	Proportion	2017	32.4	46.8	19.6
Algeria	El Hadjab	Count	2000	0	0	0
Algeria	El Hadjab	Count	2017	0	0	0
Algeria	El-Affroun	Proportion	2000	31.2	45.4	19.5
Algeria	El-Affroun	Proportion	2017	34.2	50.8	20.2
Algeria	El-Affroun	Count	2000	0	0	0
Algeria	El-Affroun	Count	2017	0	1	0
Algeria	El Hamadia	Proportion	2000	33.8	49.0	21.2
Algeria	El Hamadia	Proportion	2017	37.5	53.2	22.3
Algeria	El Hamadia	Count	2000	0	0	0
Algeria	El Hamadia	Count	2017	0	1	0
Algeria	Bouderbala	Proportion	2000	30.7	47.5	17.9
Algeria	Bouderbala	Proportion	2017	34.8	48.9	21.1
Algeria	Bouderbala	Count	2000	0	0	0
Algeria	Bouderbala	Count	2017	0	0	0
Algeria	Djinet	Proportion	2000	29.9	48.5	17.8
Algeria	Djinet	Proportion	2017	35.1	49.0	21.6
Algeria	Djinet	Count	2000	0	0	0
Algeria	Djinet	Count	2017	0	0	0
Algeria	El Karimia	Proportion	2000	30.3	44.6	18.1
Algeria	El Karimia	Proportion	2017	35.1	49.4	20.9
Algeria	El Karimia	Count	2000	0	0	0
Algeria	El Karimia	Count	2017	0	0	0
Algeria	Djelfa	Proportion	2000	32.2	46.5	17.6
Algeria	Djelfa	Proportion	2017	32.9	48.9	19.5
Algeria	Djelfa	Count	2000	0	0	0

Table 4: ORS by Unit *(continued)*

Country	Unit	Metric	Year	Mean	Upper	Lower
Algeria	Djelfa	Count	2017	0	0	0
Algeria	Ghassoul	Proportion	2000	31.0	46.9	18.4
Algeria	Ghassoul	Proportion	2017	33.9	49.2	21.2
Algeria	Ghassoul	Count	2000	0	0	0
Algeria	Ghassoul	Count	2017	0	0	0
Algeria	M'Rara	Proportion	2000	30.7	47.9	18.0
Algeria	M'Rara	Proportion	2017	30.7	46.4	18.4
Algeria	M'Rara	Count	2000	0	0	0
Algeria	M'Rara	Count	2017	0	0	0
Algeria	Echatt	Proportion	2000	33.5	48.4	20.2
Algeria	Echatt	Proportion	2017	36.8	54.3	22.3
Algeria	Echatt	Count	2000	0	0	0
Algeria	Echatt	Count	2017	0	1	0
Algeria	Boumahra Ahmed	Proportion	2000	34.1	49.7	21.2
Algeria	Boumahra Ahmed	Proportion	2017	37.4	52.8	23.9
Algeria	Boumahra Ahmed	Count	2000	0	0	0
Algeria	Boumahra Ahmed	Count	2017	0	1	0
Algeria	Erraguene	Proportion	2000	33.3	46.7	19.2
Algeria	Erraguene	Proportion	2017	37.2	53.5	22.8
Algeria	Erraguene	Count	2000	0	0	0
Algeria	Erraguene	Count	2017	0	1	0
Algeria	Khirane	Proportion	2000	30.1	44.1	18.4
Algeria	Khirane	Proportion	2017	35.5	50.2	21.6
Algeria	Khirane	Count	2000	0	0	0
Algeria	Khirane	Count	2017	0	1	0
Algeria	Ksar Hirane	Proportion	2000	29.9	46.2	17.6
Algeria	Ksar Hirane	Proportion	2017	32.7	48.8	20.2
Algeria	Ksar Hirane	Count	2000	0	0	0
Algeria	Ksar Hirane	Count	2017	0	0	0
Algeria	Chellal	Proportion	2000	32.1	47.7	19.5
Algeria	Chellal	Proportion	2017	34.2	50.2	22.1
Algeria	Chellal	Count	2000	0	0	0
Algeria	Chellal	Count	2017	0	1	0
Algeria	El Guettana	Proportion	2000	26.5	39.7	15.9
Algeria	El Guettana	Proportion	2017	34.6	49.6	20.4
Algeria	El Guettana	Count	2000	0	0	0
Algeria	El Guettana	Count	2017	0	0	0
Algeria	Boughar	Proportion	2000	33.9	51.5	20.9
Algeria	Boughar	Proportion	2017	37.1	52.7	23.8
Algeria	Boughar	Count	2000	0	1	0
Algeria	Boughar	Count	2017	0	1	0
Algeria	Grarem Gouga	Proportion	2000	33.0	46.2	20.9
Algeria	Grarem Gouga	Proportion	2017	35.9	52.5	22.4
Algeria	Grarem Gouga	Count	2000	0	0	0
Algeria	Grarem Gouga	Count	2017	0	1	0
Algeria	Mansourah	Proportion	2000	26.0	39.6	15.6
Algeria	Mansourah	Proportion	2017	35.2	50.9	20.4
Algeria	Mansourah	Count	2000	0	0	0
Algeria	Mansourah	Count	2017	0	1	0

Table 4: ORS by Unit *(continued)*

Country	Unit	Metric	Year	Mean	Upper	Lower
Algeria	Es Senia	Proportion	2000	26.9	40.3	15.6
Algeria	Es Senia	Proportion	2017	34.0	49.1	20.2
Algeria	Es Senia	Count	2000	0	0	0
Algeria	Es Senia	Count	2017	0	0	0
Algeria	Rouissat	Proportion	2000	29.7	43.2	17.8
Algeria	Rouissat	Proportion	2017	29.7	43.7	17.8
Algeria	Rouissat	Count	2000	0	0	0
Algeria	Rouissat	Count	2017	0	0	0
Algeria	El Djazia	Proportion	2000	31.8	47.6	19.0
Algeria	El Djazia	Proportion	2017	36.1	52.1	22.3
Algeria	El Djazia	Count	2000	0	0	0
Algeria	El Djazia	Count	2017	0	1	0
Algeria	El Oueldja	Proportion	2000	29.5	44.9	16.9
Algeria	El Oueldja	Proportion	2017	35.7	52.2	21.3
Algeria	El Oueldja	Count	2000	0	0	0
Algeria	El Oueldja	Count	2017	0	1	0
Algeria	Sidi Boubekeur	Proportion	2000	30.1	45.0	18.3
Algeria	Sidi Boubekeur	Proportion	2017	35.4	51.9	22.0
Algeria	Sidi Boubekeur	Count	2000	0	0	0
Algeria	Sidi Boubekeur	Count	2017	0	1	0
Algeria	Bazer-Sakra	Proportion	2000	33.4	47.4	20.9
Algeria	Bazer-Sakra	Proportion	2017	36.6	51.7	21.0
Algeria	Bazer-Sakra	Count	2000	0	0	0
Algeria	Bazer-Sakra	Count	2017	0	1	0
Algeria	Chetouane Belaila	Proportion	2000	29.2	45.0	16.2
Algeria	Chetouane Belaila	Proportion	2017	34.8	50.4	21.8
Algeria	Chetouane Belaila	Count	2000	0	0	0
Algeria	Chetouane Belaila	Count	2017	0	1	0
Algeria	Collo	Proportion	2000	32.0	47.2	19.5
Algeria	Collo	Proportion	2017	34.9	51.7	20.9
Algeria	Collo	Count	2000	0	0	0
Algeria	Collo	Count	2017	0	1	0
Algeria	Ouled Driss	Proportion	2000	33.2	48.7	20.1
Algeria	Ouled Driss	Proportion	2017	35.7	52.3	23.2
Algeria	Ouled Driss	Count	2000	0	0	0
Algeria	Ouled Driss	Count	2017	0	1	0
Algeria	El Mezeraa	Proportion	2000	30.6	46.9	16.7
Algeria	El Mezeraa	Proportion	2017	35.6	51.8	22.3
Algeria	El Mezeraa	Count	2000	0	0	0
Algeria	El Mezeraa	Count	2017	0	1	0
Algeria	Hamadia	Proportion	2000	31.8	46.4	18.4
Algeria	Hamadia	Proportion	2017	34.5	48.5	19.9
Algeria	Hamadia	Count	2000	0	0	0
Algeria	Hamadia	Count	2017	0	0	0
Algeria	Hadjret Ennous	Proportion	2000	31.1	43.1	19.1
Algeria	Hadjret Ennous	Proportion	2017	33.4	49.3	21.2
Algeria	Hadjret Ennous	Count	2000	0	0	0
Algeria	Hadjret Ennous	Count	2017	0	0	0
Algeria	Melaab	Proportion	2000	31.2	45.8	18.3

Table 4: ORS by Unit *(continued)*

Country	Unit	Metric	Year	Mean	Upper	Lower
Algeria	Melaab	Proportion	2017	34.7	50.9	21.0
Algeria	Melaab	Count	2000	0	0	0
Algeria	Melaab	Count	2017	0	1	0
Algeria	Ait Oumalou	Proportion	2000	28.0	45.4	16.9
Algeria	Ait Oumalou	Proportion	2017	35.0	48.9	21.0
Algeria	Ait Oumalou	Count	2000	0	0	0
Algeria	Ait Oumalou	Count	2017	0	0	0
Algeria	Beni Mester	Proportion	2000	29.8	46.7	17.4
Algeria	Beni Mester	Proportion	2017	34.8	51.2	21.7
Algeria	Beni Mester	Count	2000	0	0	0
Algeria	Beni Mester	Count	2017	0	1	0
Algeria	Ouled Said	Proportion	2000	30.2	46.4	19.7
Algeria	Ouled Said	Proportion	2017	31.5	44.9	18.3
Algeria	Ouled Said	Count	2000	0	0	0
Algeria	Ouled Said	Count	2017	0	0	0
Algeria	Bourached	Proportion	2000	30.7	45.8	18.6
Algeria	Bourached	Proportion	2017	34.3	48.4	19.9
Algeria	Bourached	Count	2000	0	0	0
Algeria	Bourached	Count	2017	0	0	0
Algeria	Hammam Bouhadjar	Proportion	2000	28.1	41.2	16.7
Algeria	Hammam Bouhadjar	Proportion	2017	34.4	49.0	21.0
Algeria	Hammam Bouhadjar	Count	2000	0	0	0
Algeria	Hammam Bouhadjar	Count	2017	0	0	0
Algeria	Les Eucalyptus	Proportion	2000	30.8	46.2	19.0
Algeria	Les Eucalyptus	Proportion	2017	33.4	49.2	19.8
Algeria	Les Eucalyptus	Count	2000	0	0	0
Algeria	Les Eucalyptus	Count	2017	0	0	0
Algeria	Chir	Proportion	2000	31.1	45.0	19.0
Algeria	Chir	Proportion	2017	36.3	51.8	22.0
Algeria	Chir	Count	2000	0	0	0
Algeria	Chir	Count	2017	0	1	0
Algeria	Meridja	Proportion	2000	33.2	46.3	20.6
Algeria	Meridja	Proportion	2017	33.9	49.2	21.1
Algeria	Meridja	Count	2000	0	0	0
Algeria	Meridja	Count	2017	0	0	0
Algeria	Boudjellil	Proportion	2000	32.2	47.9	19.2
Algeria	Boudjellil	Proportion	2017	37.8	54.2	23.3
Algeria	Boudjellil	Count	2000	0	0	0
Algeria	Boudjellil	Count	2017	0	1	0
Algeria	El Haouch	Proportion	2000	30.0	44.1	19.0
Algeria	El Haouch	Proportion	2017	31.4	45.5	19.8
Algeria	El Haouch	Count	2000	0	0	0
Algeria	El Haouch	Count	2017	0	0	0
Algeria	Gherouaou	Proportion	2000	30.9	45.9	19.0
Algeria	Gherouaou	Proportion	2017	32.1	49.6	18.3
Algeria	Gherouaou	Count	2000	0	0	0
Algeria	Gherouaou	Count	2017	0	0	0
Algeria	El M'Hir	Proportion	2000	34.1	50.3	19.7
Algeria	El M'Hir	Proportion	2017	36.7	52.5	22.3

Table 4: ORS by Unit *(continued)*

Country	Unit	Metric	Year	Mean	Upper	Lower
Algeria	El M'Hir	Count	2000	0	1	0
Algeria	El M'Hir	Count	2017	0	1	0
Algeria	Bouira	Proportion	2000	31.7	47.9	19.0
Algeria	Bouira	Proportion	2017	37.8	54.0	23.8
Algeria	Bouira	Count	2000	0	0	0
Algeria	Bouira	Count	2017	0	1	0
Algeria	El Kharrouba	Proportion	2000	31.1	48.5	18.6
Algeria	El Kharrouba	Proportion	2017	35.4	50.6	21.4
Algeria	El Kharrouba	Count	2000	0	0	0
Algeria	El Kharrouba	Count	2017	0	1	0
Algeria	El Marsa	Proportion	2000	28.1	41.8	15.8
Algeria	El Marsa	Proportion	2017	35.0	50.8	21.2
Algeria	El Marsa	Count	2000	0	0	0
Algeria	El Marsa	Count	2017	0	1	0
Algeria	Douis	Proportion	2000	30.9	47.4	18.3
Algeria	Douis	Proportion	2017	31.9	46.4	21.0
Algeria	Douis	Count	2000	0	0	0
Algeria	Douis	Count	2017	0	0	0
Algeria	Kef El Ahmar	Proportion	2000	30.9	45.3	17.4
Algeria	Kef El Ahmar	Proportion	2017	34.5	49.3	19.5
Algeria	Kef El Ahmar	Count	2000	0	0	0
Algeria	Kef El Ahmar	Count	2017	0	0	0
Algeria	Magrane	Proportion	2000	30.0	45.9	18.0
Algeria	Magrane	Proportion	2017	31.3	46.5	18.0
Algeria	Magrane	Count	2000	0	0	0
Algeria	Magrane	Count	2017	0	0	0
Algeria	El Aioun	Proportion	2000	31.3	46.0	19.3
Algeria	El Aioun	Proportion	2017	36.1	51.0	22.1
Algeria	El Aioun	Count	2000	0	0	0
Algeria	El Aioun	Count	2017	0	1	0
Algeria	Dahouara	Proportion	2000	33.8	50.4	20.7
Algeria	Dahouara	Proportion	2017	36.9	54.1	23.6
Algeria	Dahouara	Count	2000	0	1	0
Algeria	Dahouara	Count	2017	0	1	0
Algeria	Ghebala	Proportion	2000	33.7	47.1	21.3
Algeria	Ghebala	Proportion	2017	38.0	53.8	24.2
Algeria	Ghebala	Count	2000	0	0	0
Algeria	Ghebala	Count	2017	0	1	0
Algeria	M'Sara	Proportion	2000	30.4	44.6	18.3
Algeria	M'Sara	Proportion	2017	34.5	49.6	20.4
Algeria	M'Sara	Count	2000	0	0	0
Algeria	M'Sara	Count	2017	0	0	0
Algeria	Laghouat	Proportion	2000	30.1	45.9	17.1
Algeria	Laghouat	Proportion	2017	33.0	50.5	20.7
Algeria	Laghouat	Count	2000	0	0	0
Algeria	Laghouat	Count	2017	0	1	0
Algeria	Dehahna	Proportion	2000	31.7	44.7	19.4
Algeria	Dehahna	Proportion	2017	35.2	50.9	21.4
Algeria	Dehahna	Count	2000	0	0	0

Table 4: ORS by Unit *(continued)*

Country	Unit	Metric	Year	Mean	Upper	Lower
Algeria	Dehahna	Count	2017	0	1	0
Algeria	El Hachem	Proportion	2000	29.2	46.4	17.9
Algeria	El Hachem	Proportion	2017	35.0	50.1	21.4
Algeria	El Hachem	Count	2000	0	0	0
Algeria	El Hachem	Count	2017	0	1	0
Algeria	Boughezoul	Proportion	2000	33.5	49.7	19.4
Algeria	Boughezoul	Proportion	2017	36.5	51.0	23.6
Algeria	Boughezoul	Count	2000	0	0	0
Algeria	Boughezoul	Count	2017	0	1	0
Algeria	Hamala	Proportion	2000	32.2	45.0	20.5
Algeria	Hamala	Proportion	2017	36.8	53.4	23.1
Algeria	Hamala	Count	2000	0	0	0
Algeria	Hamala	Count	2017	0	1	0
Algeria	Mesra	Proportion	2000	25.7	38.6	15.4
Algeria	Mesra	Proportion	2017	35.5	51.5	20.7
Algeria	Mesra	Count	2000	0	0	0
Algeria	Mesra	Count	2017	0	1	0
Algeria	Gdyel	Proportion	2000	26.1	38.6	15.9
Algeria	Gdyel	Proportion	2017	35.2	49.8	21.3
Algeria	Gdyel	Count	2000	0	0	0
Algeria	Gdyel	Count	2017	0	0	0
Algeria	Sidi Khouiled	Proportion	2000	29.7	43.9	17.3
Algeria	Sidi Khouiled	Proportion	2017	29.9	45.2	18.5
Algeria	Sidi Khouiled	Count	2000	0	0	0
Algeria	Sidi Khouiled	Count	2017	0	0	0
Algeria	El Fedjoudj Boughrara Saoudi	Proportion	2000	32.8	48.5	20.6
Algeria	El Fedjoudj Boughrara Saoudi	Proportion	2017	36.7	52.4	22.0
Algeria	El Fedjoudj Boughrara Saoudi	Count	2000	0	0	0
Algeria	El Fedjoudj Boughrara Saoudi	Count	2017	0	1	0
Algeria	Had Echkalla	Proportion	2000	31.1	48.6	18.3
Algeria	Had Echkalla	Proportion	2017	36.0	53.0	21.5
Algeria	Had Echkalla	Count	2000	0	0	0
Algeria	Had Echkalla	Count	2017	0	1	0
Algeria	Tircine	Proportion	2000	30.7	45.3	19.0
Algeria	Tircine	Proportion	2017	36.0	53.7	22.8
Algeria	Tircine	Count	2000	0	0	0
Algeria	Tircine	Count	2017	0	1	0
Algeria	Beidha Bordj	Proportion	2000	33.2	47.8	21.4
Algeria	Beidha Bordj	Proportion	2017	36.8	52.0	22.1
Algeria	Beidha Bordj	Count	2000	0	0	0
Algeria	Beidha Bordj	Count	2017	0	1	0
Algeria	Dhaya	Proportion	2000	30.8	46.0	17.0
Algeria	Dhaya	Proportion	2017	34.4	50.2	20.6
Algeria	Dhaya	Count	2000	0	0	0
Algeria	Dhaya	Count	2017	0	1	0

Table 4: ORS by Unit *(continued)*

Country	Unit	Metric	Year	Mean	Upper	Lower
Algeria	Djendel Saadi Mohamed	Proportion	2000	33.6	49.0	20.5
Algeria	Djendel Saadi Mohamed	Proportion	2017	37.7	54.2	25.6
Algeria	Djendel Saadi Mohamed	Count	2000	0	0	0
Algeria	Djendel Saadi Mohamed	Count	2017	0	1	0
Algeria	Ouled Moumen	Proportion	2000	35.9	51.9	23.4
Algeria	Ouled Moumen	Proportion	2017	40.3	56.8	25.7
Algeria	Ouled Moumen	Count	2000	0	1	0
Algeria	Ouled Moumen	Count	2017	0	1	0
Algeria	El Ogla	Proportion	2000	31.0	48.1	17.0
Algeria	El Ogla	Proportion	2017	35.8	51.6	21.2
Algeria	El Ogla	Count	2000	0	0	0
Algeria	El Ogla	Count	2017	0	1	0
Algeria	Ksar Chellala	Proportion	2000	32.4	46.1	19.1
Algeria	Ksar Chellala	Proportion	2017	36.9	52.2	23.9
Algeria	Ksar Chellala	Count	2000	0	0	0
Algeria	Ksar Chellala	Count	2017	0	1	0
Algeria	Hattatba	Proportion	2000	31.1	47.4	19.1
Algeria	Hattatba	Proportion	2017	33.9	52.6	19.4
Algeria	Hattatba	Count	2000	0	0	0
Algeria	Hattatba	Count	2017	0	1	0
Algeria	Ouled Bessem	Proportion	2000	33.0	48.9	19.4
Algeria	Ouled Bessem	Proportion	2017	35.7	51.7	20.1
Algeria	Ouled Bessem	Count	2000	0	0	0
Algeria	Ouled Bessem	Count	2017	0	1	0
Algeria	Ait Toudert	Proportion	2000	28.8	45.7	16.9
Algeria	Ait Toudert	Proportion	2017	35.5	50.1	21.8
Algeria	Ait Toudert	Count	2000	0	0	0
Algeria	Ait Toudert	Count	2017	0	1	0
Algeria	Beni Ouarsous	Proportion	2000	29.7	45.7	18.7
Algeria	Beni Ouarsous	Proportion	2017	35.3	51.2	20.2
Algeria	Beni Ouarsous	Count	2000	0	0	0
Algeria	Beni Ouarsous	Count	2017	0	1	0
Algeria	Reggane	Proportion	2000	29.8	40.7	20.5
Algeria	Reggane	Proportion	2017	30.7	40.3	22.9
Algeria	Reggane	Count	2000	0	0	0
Algeria	Reggane	Count	2017	0	0	0
Algeria	Djelida	Proportion	2000	31.1	45.0	19.7
Algeria	Djelida	Proportion	2017	33.7	49.3	19.1
Algeria	Djelida	Count	2000	0	0	0
Algeria	Djelida	Count	2017	0	0	0
Algeria	Hassasna	Proportion	2000	28.1	41.3	16.2
Algeria	Hassasna	Proportion	2017	34.2	48.1	20.6
Algeria	Hassasna	Count	2000	0	0	0
Algeria	Hassasna	Count	2017	0	0	0
Algeria	Mahelma	Proportion	2000	30.8	45.1	19.0
Algeria	Mahelma	Proportion	2017	34.4	50.7	20.9
Algeria	Mahelma	Count	2000	0	0	0
Algeria	Mahelma	Count	2017	0	1	0
Algeria	Djerma	Proportion	2000	32.0	46.9	18.6

Table 4: ORS by Unit *(continued)*

Country	Unit	Metric	Year	Mean	Upper	Lower
Algeria	Djerma	Proportion	2017	36.4	52.9	21.5
Algeria	Djerma	Count	2000	0	0	0
Algeria	Djerma	Count	2017	0	1	0
Algeria	Mogheul	Proportion	2000	32.4	49.1	19.3
Algeria	Mogheul	Proportion	2017	33.8	49.1	19.9
Algeria	Mogheul	Count	2000	0	0	0
Algeria	Mogheul	Count	2017	0	0	0
Algeria	Bouhamza	Proportion	2000	32.3	48.1	19.7
Algeria	Bouhamza	Proportion	2017	37.6	53.6	23.9
Algeria	Bouhamza	Count	2000	0	0	0
Algeria	Bouhamza	Count	2017	0	1	0
Algeria	El Kentara	Proportion	2000	30.5	44.4	18.0
Algeria	El Kentara	Proportion	2017	35.2	50.3	22.3
Algeria	El Kentara	Count	2000	0	0	0
Algeria	El Kentara	Count	2017	0	1	0
Algeria	Hammam Melouane	Proportion	2000	31.9	47.8	19.4
Algeria	Hammam Melouane	Proportion	2017	34.5	49.7	20.8
Algeria	Hammam Melouane	Count	2000	0	0	0
Algeria	Hammam Melouane	Count	2017	0	0	0
Algeria	El Main	Proportion	2000	33.1	48.8	20.6
Algeria	El Main	Proportion	2017	37.1	52.4	23.0
Algeria	El Main	Count	2000	0	0	0
Algeria	El Main	Count	2017	0	1	0
Algeria	Boukram	Proportion	2000	32.0	48.4	19.3
Algeria	Boukram	Proportion	2017	35.0	49.3	21.7
Algeria	Boukram	Count	2000	0	0	0
Algeria	Boukram	Count	2017	0	0	0
Algeria	Hammadi	Proportion	2000	32.0	48.8	19.5
Algeria	Hammadi	Proportion	2017	38.1	54.6	23.3
Algeria	Hammadi	Count	2000	0	0	0
Algeria	Hammadi	Count	2017	0	1	0
Algeria	Harchoune	Proportion	2000	30.2	43.9	18.1
Algeria	Harchoune	Proportion	2017	35.2	50.2	20.8
Algeria	Harchoune	Count	2000	0	0	0
Algeria	Harchoune	Count	2017	0	1	0
Algeria	El Guedid	Proportion	2000	30.7	45.1	17.5
Algeria	El Guedid	Proportion	2017	32.7	46.9	21.1
Algeria	El Guedid	Count	2000	0	0	0
Algeria	El Guedid	Count	2017	0	0	0
Algeria	Krakda	Proportion	2000	30.9	46.2	18.7
Algeria	Krakda	Proportion	2017	33.7	47.3	20.6
Algeria	Krakda	Count	2000	0	0	0
Algeria	Krakda	Count	2017	0	0	0
Algeria	Mih Ouansa	Proportion	2000	30.7	47.5	17.4
Algeria	Mih Ouansa	Proportion	2017	31.4	45.1	18.6
Algeria	Mih Ouansa	Count	2000	0	0	0
Algeria	Mih Ouansa	Count	2017	0	0	0
Algeria	El Kala	Proportion	2000	32.3	47.2	20.0
Algeria	El Kala	Proportion	2017	35.0	50.2	21.8

Table 4: ORS by Unit *(continued)*

Country	Unit	Metric	Year	Mean	Upper	Lower
Algeria	El Kala	Count	2000	0	0	0
Algeria	El Kala	Count	2017	0	1	0
Algeria	Djebala El Khemissi	Proportion	2000	34.3	50.4	21.8
Algeria	Djebala El Khemissi	Proportion	2017	37.7	52.7	24.1
Algeria	Djebala El Khemissi	Count	2000	0	1	0
Algeria	Djebala El Khemissi	Count	2017	0	1	0
Algeria	Jijel	Proportion	2000	31.0	44.6	19.2
Algeria	Jijel	Proportion	2017	36.2	51.3	24.7
Algeria	Jijel	Count	2000	0	0	0
Algeria	Jijel	Count	2017	0	1	0
Algeria	M'Toussa	Proportion	2000	31.1	47.0	19.0
Algeria	M'Toussa	Proportion	2017	35.2	49.2	20.2
Algeria	M'Toussa	Count	2000	0	0	0
Algeria	M'Toussa	Count	2017	0	0	0
Algeria	Mekhareg	Proportion	2000	30.0	45.1	18.1
Algeria	Mekhareg	Proportion	2017	32.7	48.6	20.8
Algeria	Mekhareg	Count	2000	0	0	0
Algeria	Mekhareg	Count	2017	0	0	0
Algeria	Djebel Messaad	Proportion	2000	31.5	46.3	19.6
Algeria	Djebel Messaad	Proportion	2017	33.6	50.0	20.1
Algeria	Djebel Messaad	Count	2000	0	0	0
Algeria	Djebel Messaad	Count	2017	0	0	0
Algeria	El Keurt	Proportion	2000	27.2	40.7	16.3
Algeria	El Keurt	Proportion	2017	35.9	52.3	22.0
Algeria	El Keurt	Count	2000	0	0	0
Algeria	El Keurt	Count	2017	0	1	0
Algeria	Bouskene	Proportion	2000	33.5	51.1	20.1
Algeria	Bouskene	Proportion	2017	36.1	50.9	22.7
Algeria	Bouskene	Count	2000	0	1	0
Algeria	Bouskene	Count	2017	0	1	0
Algeria	Mila	Proportion	2000	33.0	45.8	20.0
Algeria	Mila	Proportion	2017	36.3	53.8	22.3
Algeria	Mila	Count	2000	0	0	0
Algeria	Mila	Count	2017	0	1	0
Algeria	Mezghrane	Proportion	2000	25.0	38.4	15.3
Algeria	Mezghrane	Proportion	2017	34.8	50.2	20.9
Algeria	Mezghrane	Count	2000	0	0	0
Algeria	Mezghrane	Count	2017	0	1	0
Algeria	Hassi Ben Okba	Proportion	2000	26.6	38.7	16.4
Algeria	Hassi Ben Okba	Proportion	2017	35.1	49.9	21.9
Algeria	Hassi Ben Okba	Count	2000	0	0	0
Algeria	Hassi Ben Okba	Count	2017	0	0	0
Algeria	Sidi Slimane	Proportion	2000	30.3	46.2	19.1
Algeria	Sidi Slimane	Proportion	2017	31.2	45.3	18.6
Algeria	Sidi Slimane	Count	2000	0	0	0
Algeria	Sidi Slimane	Count	2017	0	0	0
Algeria	El Harmilia	Proportion	2000	33.5	48.4	20.0
Algeria	El Harmilia	Proportion	2017	36.5	51.0	22.1
Algeria	El Harmilia	Count	2000	0	0	0

Table 4: ORS by Unit *(continued)*

Country	Unit	Metric	Year	Mean	Upper	Lower
Algeria	El Harmilia	Count	2017	0	1	0
Algeria	Hamri	Proportion	2000	27.1	42.2	15.7
Algeria	Hamri	Proportion	2017	35.5	51.4	21.1
Algeria	Hamri	Count	2000	0	0	0
Algeria	Hamri	Count	2017	0	1	0
Algeria	Youb	Proportion	2000	29.5	44.6	16.9
Algeria	Youb	Proportion	2017	35.0	51.6	20.8
Algeria	Youb	Count	2000	0	0	0
Algeria	Youb	Count	2017	0	1	0
Algeria	Bellaa	Proportion	2000	33.8	47.3	21.0
Algeria	Bellaa	Proportion	2017	36.5	50.8	21.2
Algeria	Bellaa	Count	2000	0	0	0
Algeria	Bellaa	Count	2017	0	1	0
Algeria	El Hacaiba	Proportion	2000	30.9	47.1	17.5
Algeria	El Hacaiba	Proportion	2017	35.5	51.2	21.9
Algeria	El Hacaiba	Count	2000	0	0	0
Algeria	El Hacaiba	Count	2017	0	1	0
Algeria	El Ghedir	Proportion	2000	34.3	49.6	21.8
Algeria	El Ghedir	Proportion	2017	39.3	56.8	25.1
Algeria	El Ghedir	Count	2000	0	0	0
Algeria	El Ghedir	Count	2017	0	1	0
Algeria	Oum El Adhaim	Proportion	2000	32.5	48.6	20.0
Algeria	Oum El Adhaim	Proportion	2017	37.0	53.5	25.3
Algeria	Oum El Adhaim	Count	2000	0	0	0
Algeria	Oum El Adhaim	Count	2017	0	1	0
Algeria	El Ouinet	Proportion	2000	31.2	46.8	18.3
Algeria	El Ouinet	Proportion	2017	36.7	53.4	23.9
Algeria	El Ouinet	Count	2000	0	0	0
Algeria	El Ouinet	Count	2017	0	1	0
Algeria	Madna	Proportion	2000	31.1	45.3	18.6
Algeria	Madna	Proportion	2017	35.9	52.7	23.0
Algeria	Madna	Count	2000	0	0	0
Algeria	Madna	Count	2017	0	1	0
Algeria	Kolea	Proportion	2000	30.9	45.4	18.8
Algeria	Kolea	Proportion	2017	33.9	51.3	19.9
Algeria	Kolea	Count	2000	0	0	0
Algeria	Kolea	Count	2017	0	1	0
Algeria	Sidi Abed	Proportion	2000	32.5	47.2	20.0
Algeria	Sidi Abed	Proportion	2017	35.1	50.3	19.6
Algeria	Sidi Abed	Count	2000	0	0	0
Algeria	Sidi Abed	Count	2017	0	1	0
Algeria	Ait Yahia Moussa	Proportion	2000	28.4	45.4	16.1
Algeria	Ait Yahia Moussa	Proportion	2017	34.7	49.4	20.8
Algeria	Ait Yahia Moussa	Count	2000	0	0	0
Algeria	Ait Yahia Moussa	Count	2017	0	0	0
Algeria	Beni Smiel	Proportion	2000	29.9	44.4	16.9
Algeria	Beni Smiel	Proportion	2017	34.6	50.1	21.1
Algeria	Beni Smiel	Count	2000	0	0	0
Algeria	Beni Smiel	Count	2017	0	1	0

Table 4: ORS by Unit *(continued)*

Country	Unit	Metric	Year	Mean	Upper	Lower
Algeria	Sali	Proportion	2000	29.2	41.0	19.0
Algeria	Sali	Proportion	2017	31.1	43.3	21.6
Algeria	Sali	Count	2000	0	0	0
Algeria	Sali	Count	2017	0	0	0
Algeria	Djemaa Ouled Cheikh	Proportion	2000	31.8	46.1	20.1
Algeria	Djemaa Ouled Cheikh	Proportion	2017	34.2	48.2	19.6
Algeria	Djemaa Ouled Cheikh	Count	2000	0	0	0
Algeria	Djemaa Ouled Cheikh	Count	2017	0	0	0
Algeria	Hassi El Ghella	Proportion	2000	28.3	41.1	17.3
Algeria	Hassi El Ghella	Proportion	2017	34.7	49.3	21.5
Algeria	Hassi El Ghella	Count	2000	0	0	0
Algeria	Hassi El Ghella	Count	2017	0	0	0
Algeria	Ouled Chebel	Proportion	2000	31.2	46.4	19.7
Algeria	Ouled Chebel	Proportion	2017	35.0	49.6	21.1
Algeria	Ouled Chebel	Count	2000	0	0	0
Algeria	Ouled Chebel	Count	2017	0	0	0
Algeria	Djezzar	Proportion	2000	32.8	47.3	20.3
Algeria	Djezzar	Proportion	2017	36.9	53.5	22.3
Algeria	Djezzar	Count	2000	0	0	0
Algeria	Djezzar	Count	2017	0	1	0
Algeria	Ouled Khoudir	Proportion	2000	32.7	47.1	20.4
Algeria	Ouled Khoudir	Proportion	2017	33.7	48.1	21.2
Algeria	Ouled Khoudir	Count	2000	0	0	0
Algeria	Ouled Khoudir	Count	2017	0	0	0
Algeria	Boukhlifa	Proportion	2000	30.4	45.7	18.2
Algeria	Boukhlifa	Proportion	2017	36.1	51.4	23.1
Algeria	Boukhlifa	Count	2000	0	0	0
Algeria	Boukhlifa	Count	2017	0	1	0
Algeria	El Ouitaya	Proportion	2000	30.8	45.2	18.4
Algeria	El Ouitaya	Proportion	2017	33.5	48.3	20.3
Algeria	El Ouitaya	Count	2000	0	0	0
Algeria	El Ouitaya	Count	2017	0	0	0
Algeria	Larbaa	Proportion	2000	31.7	46.6	20.2
Algeria	Larbaa	Proportion	2017	36.8	52.3	23.1
Algeria	Larbaa	Count	2000	0	0	0
Algeria	Larbaa	Count	2017	0	1	0
Algeria	Ghilassa	Proportion	2000	32.1	47.9	20.1
Algeria	Ghilassa	Proportion	2017	35.5	52.5	21.3
Algeria	Ghilassa	Count	2000	0	0	0
Algeria	Ghilassa	Count	2017	0	1	0
Algeria	Chorfa	Proportion	2000	32.2	48.1	19.0
Algeria	Chorfa	Proportion	2017	37.6	53.1	23.4
Algeria	Chorfa	Count	2000	0	0	0
Algeria	Chorfa	Count	2017	0	1	0
Algeria	Isser	Proportion	2000	30.1	49.6	17.7
Algeria	Isser	Proportion	2017	35.9	49.9	21.8
Algeria	Isser	Count	2000	0	0	0
Algeria	Isser	Count	2017	0	0	0
Algeria	Herenfa	Proportion	2000	28.2	42.9	16.1

Table 4: ORS by Unit *(continued)*

Country	Unit	Metric	Year	Mean	Upper	Lower
Algeria	Herenfa	Proportion	2017	34.1	52.0	19.0
Algeria	Herenfa	Count	2000	0	0	0
Algeria	Herenfa	Count	2017	0	1	0
Algeria	El Idrissia	Proportion	2000	31.2	46.6	19.1
Algeria	El Idrissia	Proportion	2017	33.8	47.9	22.7
Algeria	El Idrissia	Count	2000	0	0	0
Algeria	El Idrissia	Count	2017	0	0	0
Algeria	Rogassa	Proportion	2000	31.2	45.6	18.7
Algeria	Rogassa	Proportion	2017	34.7	48.9	21.1
Algeria	Rogassa	Count	2000	0	0	0
Algeria	Rogassa	Count	2017	0	0	0
Algeria	Nakhla	Proportion	2000	31.2	48.1	17.5
Algeria	Nakhla	Proportion	2017	30.5	47.1	18.0
Algeria	Nakhla	Count	2000	0	0	0
Algeria	Nakhla	Count	2017	0	0	0
Algeria	El Tarf	Proportion	2000	32.9	48.3	20.0
Algeria	El Tarf	Proportion	2017	35.2	50.7	22.1
Algeria	El Tarf	Count	2000	0	0	0
Algeria	El Tarf	Count	2017	0	1	0
Algeria	El Fedjoudj	Proportion	2000	34.7	51.5	21.1
Algeria	El Fedjoudj	Proportion	2017	36.7	52.6	23.6
Algeria	El Fedjoudj	Count	2000	0	1	0
Algeria	El Fedjoudj	Count	2017	0	1	0
Algeria	Kheir Oued Adjoul	Proportion	2000	32.2	46.1	20.5
Algeria	Kheir Oued Adjoul	Proportion	2017	37.5	54.1	24.0
Algeria	Kheir Oued Adjoul	Count	2000	0	0	0
Algeria	Kheir Oued Adjoul	Count	2017	0	1	0
Algeria	Ouled Rechache	Proportion	2000	30.3	47.7	17.7
Algeria	Ouled Rechache	Proportion	2017	35.2	50.1	20.9
Algeria	Ouled Rechache	Count	2000	0	0	0
Algeria	Ouled Rechache	Count	2017	0	1	0
Algeria	Oued M'Zi	Proportion	2000	30.1	46.4	18.3
Algeria	Oued M'Zi	Proportion	2017	32.6	45.3	21.9
Algeria	Oued M'Zi	Count	2000	0	0	0
Algeria	Oued M'Zi	Count	2017	0	0	0
Algeria	El Houamed	Proportion	2000	31.6	45.7	19.6
Algeria	El Houamed	Proportion	2017	33.5	47.7	20.8
Algeria	El Houamed	Count	2000	0	0	0
Algeria	El Houamed	Count	2017	0	0	0
Algeria	El Mamounia	Proportion	2000	27.2	40.3	16.3
Algeria	El Mamounia	Proportion	2017	34.9	52.5	21.3
Algeria	El Mamounia	Count	2000	0	0	0
Algeria	El Mamounia	Count	2017	0	1	0
Algeria	Chahbounia	Proportion	2000	33.0	49.9	18.8
Algeria	Chahbounia	Proportion	2017	36.0	50.6	23.6
Algeria	Chahbounia	Count	2000	0	0	0
Algeria	Chahbounia	Count	2017	0	1	0
Algeria	Minar Zarza	Proportion	2000	32.8	45.3	20.7
Algeria	Minar Zarza	Proportion	2017	37.5	53.4	23.6

Table 4: ORS by Unit *(continued)*

Country	Unit	Metric	Year	Mean	Upper	Lower
Algeria	Minar Zarza	Count	2000	0	0	0
Algeria	Minar Zarza	Count	2017	0	1	0
Algeria	Mostaganem	Proportion	2000	24.8	38.0	14.7
Algeria	Mostaganem	Proportion	2017	33.7	48.1	20.2
Algeria	Mostaganem	Count	2000	0	0	0
Algeria	Mostaganem	Count	2017	0	0	0
Algeria	Hassi Bounif	Proportion	2000	26.0	38.2	16.3
Algeria	Hassi Bounif	Proportion	2017	34.8	49.7	21.8
Algeria	Hassi Bounif	Count	2000	0	0	0
Algeria	Hassi Bounif	Count	2017	0	0	0
Algeria	Taibet	Proportion	2000	30.3	45.2	18.5
Algeria	Taibet	Proportion	2017	30.6	42.9	19.9
Algeria	Taibet	Count	2000	0	0	0
Algeria	Taibet	Count	2017	0	0	0
Algeria	El Rahia	Proportion	2000	32.0	47.9	19.5
Algeria	El Rahia	Proportion	2017	36.2	51.3	22.5
Algeria	El Rahia	Count	2000	0	0	0
Algeria	El Rahia	Count	2017	0	1	0
Algeria	Kalaa	Proportion	2000	26.6	41.0	15.9
Algeria	Kalaa	Proportion	2017	34.4	49.3	19.9
Algeria	Kalaa	Count	2000	0	0	0
Algeria	Kalaa	Count	2017	0	0	0
Algeria	Beni-Aziz	Proportion	2000	32.8	47.3	19.2
Algeria	Beni-Aziz	Proportion	2017	37.1	53.9	22.3
Algeria	Beni-Aziz	Count	2000	0	0	0
Algeria	Beni-Aziz	Count	2017	0	1	0
Algeria	Hassi Dahou	Proportion	2000	28.1	41.9	16.5
Algeria	Hassi Dahou	Proportion	2017	34.5	51.6	19.6
Algeria	Hassi Dahou	Count	2000	0	0	0
Algeria	Hassi Dahou	Count	2017	0	1	0
Algeria	El Hadaiek	Proportion	2000	32.2	49.4	19.4
Algeria	El Hadaiek	Proportion	2017	35.6	52.5	21.0
Algeria	El Hadaiek	Count	2000	0	0	0
Algeria	El Hadaiek	Count	2017	0	1	0
Algeria	Ragouba	Proportion	2000	33.5	50.4	20.2
Algeria	Ragouba	Proportion	2017	35.6	51.6	23.6
Algeria	Ragouba	Count	2000	0	1	0
Algeria	Ragouba	Count	2017	0	1	0
Algeria	Ferkane	Proportion	2000	30.5	46.1	18.3
Algeria	Ferkane	Proportion	2017	35.6	50.6	20.9
Algeria	Ferkane	Count	2000	0	0	0
Algeria	Ferkane	Count	2017	0	1	0
Algeria	Mahdia	Proportion	2000	32.1	46.4	18.5
Algeria	Mahdia	Proportion	2017	34.7	49.0	20.9
Algeria	Mahdia	Count	2000	0	0	0
Algeria	Mahdia	Count	2017	0	0	0
Algeria	Larhat	Proportion	2000	30.6	44.4	18.9
Algeria	Larhat	Proportion	2017	33.1	46.9	20.8
Algeria	Larhat	Count	2000	0	0	0

Table 4: ORS by Unit *(continued)*

Country	Unit	Metric	Year	Mean	Upper	Lower
Algeria	Larhat	Count	2017	0	0	0
Algeria	Sidi Boutouchent	Proportion	2000	32.4	47.7	19.5
Algeria	Sidi Boutouchent	Proportion	2017	34.4	49.0	19.8
Algeria	Sidi Boutouchent	Count	2000	0	0	0
Algeria	Sidi Boutouchent	Count	2017	0	0	0
Algeria	Akbil	Proportion	2000	28.3	45.3	16.6
Algeria	Akbil	Proportion	2017	34.5	49.5	22.0
Algeria	Akbil	Count	2000	0	0	0
Algeria	Akbil	Count	2017	0	0	0
Algeria	Beni Snous	Proportion	2000	30.3	48.1	18.6
Algeria	Beni Snous	Proportion	2017	34.7	50.2	19.6
Algeria	Beni Snous	Count	2000	0	0	0
Algeria	Beni Snous	Count	2017	0	1	0
Algeria	Sebaa	Proportion	2000	29.3	43.4	18.0
Algeria	Sebaa	Proportion	2017	30.5	43.9	20.1
Algeria	Sebaa	Count	2000	0	0	0
Algeria	Sebaa	Count	2017	0	0	0
Algeria	Djendel	Proportion	2000	32.7	47.2	20.9
Algeria	Djendel	Proportion	2017	33.7	46.9	20.1
Algeria	Djendel	Count	2000	0	0	0
Algeria	Djendel	Count	2017	0	0	0
Algeria	Oued Berkeche	Proportion	2000	28.4	42.1	16.1
Algeria	Oued Berkeche	Proportion	2017	34.4	48.6	21.1
Algeria	Oued Berkeche	Count	2000	0	0	0
Algeria	Oued Berkeche	Count	2017	0	0	0
Algeria	Ouled Fayet	Proportion	2000	30.6	46.2	18.9
Algeria	Ouled Fayet	Proportion	2017	30.9	47.3	17.3
Algeria	Ouled Fayet	Count	2000	0	0	0
Algeria	Ouled Fayet	Count	2017	0	0	0
Algeria	El Hassi	Proportion	2000	33.7	49.7	19.6
Algeria	El Hassi	Proportion	2017	37.2	50.9	22.2
Algeria	El Hassi	Count	2000	0	0	0
Algeria	El Hassi	Count	2017	0	1	0
Algeria	Tabelbala	Proportion	2000	33.9	44.8	23.8
Algeria	Tabelbala	Proportion	2017	33.1	42.7	22.8
Algeria	Tabelbala	Count	2000	0	0	0
Algeria	Tabelbala	Count	2017	0	0	0
Algeria	Chellata	Proportion	2000	31.0	47.3	18.5
Algeria	Chellata	Proportion	2017	37.2	52.7	22.9
Algeria	Chellata	Count	2000	0	0	0
Algeria	Chellata	Count	2017	0	1	0
Algeria	Foughala	Proportion	2000	29.9	44.5	17.9
Algeria	Foughala	Proportion	2017	32.3	45.1	19.2
Algeria	Foughala	Count	2000	0	0	0
Algeria	Foughala	Count	2017	0	0	0
Algeria	Meftah	Proportion	2000	31.8	47.7	20.0
Algeria	Meftah	Proportion	2017	38.0	53.4	23.8
Algeria	Meftah	Count	2000	0	0	0
Algeria	Meftah	Count	2017	0	1	0

Table 4: ORS by Unit *(continued)*

Country	Unit	Metric	Year	Mean	Upper	Lower
Algeria	Haraza	Proportion	2000	34.0	50.0	19.8
Algeria	Haraza	Proportion	2017	35.9	51.7	21.3
Algeria	Haraza	Count	2000	0	0	0
Algeria	Haraza	Count	2017	0	1	0
Algeria	Dechmia	Proportion	2000	34.1	51.4	19.7
Algeria	Dechmia	Proportion	2017	35.5	50.1	22.4
Algeria	Dechmia	Count	2000	0	1	0
Algeria	Dechmia	Count	2017	0	1	0
Algeria	Khemis El Khechna	Proportion	2000	31.8	48.6	19.6
Algeria	Khemis El Khechna	Proportion	2017	36.9	53.1	22.4
Algeria	Khemis El Khechna	Count	2000	0	0	0
Algeria	Khemis El Khechna	Count	2017	0	1	0
Algeria	Labiod Medjadja	Proportion	2000	29.5	44.9	17.3
Algeria	Labiod Medjadja	Proportion	2017	33.9	51.8	19.2
Algeria	Labiod Medjadja	Count	2000	0	0	0
Algeria	Labiod Medjadja	Count	2017	0	1	0
Algeria	El Khemis	Proportion	2000	30.9	45.1	16.6
Algeria	El Khemis	Proportion	2017	32.4	46.3	19.9
Algeria	El Khemis	Count	2000	0	0	0
Algeria	El Khemis	Count	2017	0	0	0
Algeria	Sidi Ameur	Proportion	2000	30.5	43.5	18.0
Algeria	Sidi Ameur	Proportion	2017	32.8	46.3	21.6
Algeria	Sidi Ameur	Count	2000	0	0	0
Algeria	Sidi Ameur	Count	2017	0	0	0
Algeria	Oued El Alenda	Proportion	2000	31.1	48.2	18.5
Algeria	Oued El Alenda	Proportion	2017	31.6	47.3	19.1
Algeria	Oued El Alenda	Count	2000	0	0	0
Algeria	Oued El Alenda	Count	2017	0	0	0
Algeria	Hammam Ben Salah	Proportion	2000	33.6	48.6	20.1
Algeria	Hammam Ben Salah	Proportion	2017	36.8	53.4	23.5
Algeria	Hammam Ben Salah	Count	2000	0	0	0
Algeria	Hammam Ben Salah	Count	2017	0	1	0
Algeria	Guelma	Proportion	2000	34.6	51.5	21.2
Algeria	Guelma	Proportion	2017	36.3	51.8	23.1
Algeria	Guelma	Count	2000	0	1	0
Algeria	Guelma	Count	2017	0	1	0
Algeria	Kouas	Proportion	2000	31.0	44.4	18.7
Algeria	Kouas	Proportion	2017	37.2	53.9	24.4
Algeria	Kouas	Count	2000	0	0	0
Algeria	Kouas	Count	2017	0	1	0
Algeria	Remila	Proportion	2000	31.7	45.0	19.6
Algeria	Remila	Proportion	2017	35.3	50.7	20.0
Algeria	Remila	Count	2000	0	0	0
Algeria	Remila	Count	2017	0	1	0
Algeria	Oued Mora	Proportion	2000	29.4	44.1	18.5
Algeria	Oued Mora	Proportion	2017	32.0	45.5	21.5
Algeria	Oued Mora	Count	2000	0	0	0
Algeria	Oued Mora	Count	2017	0	0	0
Algeria	Hammam Dalaa	Proportion	2000	32.8	49.2	18.0

Table 4: ORS by Unit *(continued)*

Country	Unit	Metric	Year	Mean	Upper	Lower
Algeria	Hammam Dalaa	Proportion	2017	35.2	51.5	20.8
Algeria	Hammam Dalaa	Count	2000	0	0	0
Algeria	Hammam Dalaa	Count	2017	0	1	0
Algeria	El Matmor	Proportion	2000	28.1	43.3	16.9
Algeria	El Matmor	Proportion	2017	34.9	50.3	21.6
Algeria	El Matmor	Count	2000	0	0	0
Algeria	El Matmor	Count	2017	0	1	0
Algeria	Chellalet Ladhaoura	Proportion	2000	35.2	53.3	20.3
Algeria	Chellalet Ladhaoura	Proportion	2017	36.2	51.4	22.0
Algeria	Chellalet Ladhaoura	Count	2000	0	1	0
Algeria	Chellalet Ladhaoura	Count	2017	0	1	0
Algeria	Oued Athmenia	Proportion	2000	33.8	48.0	20.7
Algeria	Oued Athmenia	Proportion	2017	36.9	52.0	22.0
Algeria	Oued Athmenia	Count	2000	0	0	0
Algeria	Oued Athmenia	Count	2017	0	1	0
Algeria	Nekmaria	Proportion	2000	26.7	43.6	15.3
Algeria	Nekmaria	Proportion	2017	35.8	50.9	22.6
Algeria	Nekmaria	Count	2000	0	0	0
Algeria	Nekmaria	Count	2017	0	1	0
Algeria	Hassi Mefsoukh	Proportion	2000	26.4	39.3	16.0
Algeria	Hassi Mefsoukh	Proportion	2017	34.7	50.4	20.1
Algeria	Hassi Mefsoukh	Count	2000	0	0	0
Algeria	Hassi Mefsoukh	Count	2017	0	1	0
Algeria	Tebesbest	Proportion	2000	30.1	47.0	18.3
Algeria	Tebesbest	Proportion	2017	29.9	44.2	17.9
Algeria	Tebesbest	Count	2000	0	0	0
Algeria	Tebesbest	Count	2017	0	0	0
Algeria	Fkirina	Proportion	2000	31.3	46.3	19.3
Algeria	Fkirina	Proportion	2017	36.9	51.8	22.7
Algeria	Fkirina	Count	2000	0	0	0
Algeria	Fkirina	Count	2017	0	1	0
Algeria	Lahlef	Proportion	2000	28.9	44.7	17.0
Algeria	Lahlef	Proportion	2017	35.8	52.8	21.4
Algeria	Lahlef	Count	2000	0	0	0
Algeria	Lahlef	Count	2017	0	1	0
Algeria	Beni-Mouhli	Proportion	2000	32.2	46.4	19.7
Algeria	Beni-Mouhli	Proportion	2017	36.7	51.9	23.3
Algeria	Beni-Mouhli	Count	2000	0	0	0
Algeria	Beni-Mouhli	Count	2017	0	1	0
Algeria	Hassi Zehana	Proportion	2000	29.1	44.0	15.9
Algeria	Hassi Zehana	Proportion	2017	34.8	50.5	21.8
Algeria	Hassi Zehana	Count	2000	0	0	0
Algeria	Hassi Zehana	Count	2017	0	1	0
Algeria	El Harrouch	Proportion	2000	33.3	47.4	20.9
Algeria	El Harrouch	Proportion	2017	38.1	54.4	23.7
Algeria	El Harrouch	Count	2000	0	0	0
Algeria	El Harrouch	Count	2017	0	1	0
Algeria	Safel El Ouiden	Proportion	2000	33.2	48.9	20.7
Algeria	Safel El Ouiden	Proportion	2017	36.6	52.0	24.6

Table 4: ORS by Unit *(continued)*

Country	Unit	Metric	Year	Mean	Upper	Lower
Algeria	Safel El Ouiden	Count	2000	0	0	0
Algeria	Safel El Ouiden	Count	2017	0	1	0
Algeria	Guorriguer	Proportion	2000	31.1	47.6	17.7
Algeria	Guorriguer	Proportion	2017	35.8	53.4	23.2
Algeria	Guorriguer	Count	2000	0	0	0
Algeria	Guorriguer	Count	2017	0	1	0
Algeria	Mechraa Safa	Proportion	2000	31.8	48.9	19.1
Algeria	Mechraa Safa	Proportion	2017	35.1	52.2	21.1
Algeria	Mechraa Safa	Count	2000	0	0	0
Algeria	Mechraa Safa	Count	2017	0	1	0
Algeria	Menaceur	Proportion	2000	31.7	45.4	20.0
Algeria	Menaceur	Proportion	2017	33.3	48.2	20.4
Algeria	Menaceur	Count	2000	0	0	0
Algeria	Menaceur	Count	2017	0	0	0
Algeria	Sidi Lantri	Proportion	2000	31.7	46.2	18.7
Algeria	Sidi Lantri	Proportion	2017	35.0	49.7	20.0
Algeria	Sidi Lantri	Count	2000	0	0	0
Algeria	Sidi Lantri	Count	2017	0	0	0
Algeria	Akerrou	Proportion	2000	28.3	45.2	16.6
Algeria	Akerrou	Proportion	2017	34.2	48.3	21.0
Algeria	Akerrou	Count	2000	0	0	0
Algeria	Akerrou	Count	2017	0	0	0
Algeria	Bensekrane	Proportion	2000	29.5	44.3	17.3
Algeria	Bensekrane	Proportion	2017	35.2	50.6	22.0
Algeria	Bensekrane	Count	2000	0	0	0
Algeria	Bensekrane	Count	2017	0	1	0
Algeria	Talmine	Proportion	2000	30.4	45.1	20.0
Algeria	Talmine	Proportion	2017	31.1	46.8	18.7
Algeria	Talmine	Count	2000	0	0	0
Algeria	Talmine	Count	2017	0	0	0
Algeria	El Abadia	Proportion	2000	30.3	45.4	18.4
Algeria	El Abadia	Proportion	2017	34.6	48.8	19.8
Algeria	El Abadia	Count	2000	0	0	0
Algeria	El Abadia	Count	2017	0	0	0
Algeria	Oued Sebbah	Proportion	2000	27.8	40.6	17.1
Algeria	Oued Sebbah	Proportion	2017	34.4	48.2	20.8
Algeria	Oued Sebbah	Count	2000	0	0	0
Algeria	Oued Sebbah	Count	2017	0	0	0
Algeria	Reghaia	Proportion	2000	30.5	48.0	17.8
Algeria	Reghaia	Proportion	2017	32.6	48.6	20.0
Algeria	Reghaia	Count	2000	0	0	0
Algeria	Reghaia	Count	2017	0	0	0
Algeria	El Madher	Proportion	2000	31.5	46.2	18.4
Algeria	El Madher	Proportion	2017	36.0	52.2	21.3
Algeria	El Madher	Count	2000	0	0	0
Algeria	El Madher	Count	2017	0	1	0
Algeria	Taghit	Proportion	2000	32.7	48.4	21.8
Algeria	Taghit	Proportion	2017	33.5	49.9	20.7
Algeria	Taghit	Count	2000	0	0	0

Table 4: ORS by Unit *(continued)*

Country	Unit	Metric	Year	Mean	Upper	Lower
Algeria	Taghit	Count	2017	0	0	0
Algeria	Chemini	Proportion	2000	30.0	45.4	17.7
Algeria	Chemini	Proportion	2017	36.0	51.3	22.2
Algeria	Chemini	Count	2000	0	0	0
Algeria	Chemini	Count	2017	0	1	0
Algeria	Khenguet Sidi Nadji	Proportion	2000	29.1	44.2	16.4
Algeria	Khenguet Sidi Nadji	Proportion	2017	34.0	47.4	20.5
Algeria	Khenguet Sidi Nadji	Count	2000	0	0	0
Algeria	Khenguet Sidi Nadji	Count	2017	0	0	0
Algeria	Mouzaia	Proportion	2000	30.6	45.8	18.8
Algeria	Mouzaia	Proportion	2017	34.3	53.2	19.6
Algeria	Mouzaia	Count	2000	0	0	0
Algeria	Mouzaia	Count	2017	0	1	0
Algeria	Hasnaoua	Proportion	2000	32.9	47.6	20.5
Algeria	Hasnaoua	Proportion	2017	36.9	52.8	21.9
Algeria	Hasnaoua	Count	2000	0	0	0
Algeria	Hasnaoua	Count	2017	0	1	0
Algeria	Dirrah	Proportion	2000	33.8	50.4	19.9
Algeria	Dirrah	Proportion	2017	35.6	51.3	21.6
Algeria	Dirrah	Count	2000	0	1	0
Algeria	Dirrah	Count	2017	0	1	0
Algeria	Larbatache	Proportion	2000	31.7	48.4	19.3
Algeria	Larbatache	Proportion	2017	36.2	52.0	22.0
Algeria	Larbatache	Count	2000	0	0	0
Algeria	Larbatache	Count	2017	0	1	0
Algeria	Moussadek	Proportion	2000	27.9	41.9	15.9
Algeria	Moussadek	Proportion	2017	33.8	50.1	19.7
Algeria	Moussadek	Count	2000	0	0	0
Algeria	Moussadek	Count	2017	0	1	0
Algeria	Faidh El Botma	Proportion	2000	30.8	44.9	18.3
Algeria	Faidh El Botma	Proportion	2017	32.3	48.1	19.6
Algeria	Faidh El Botma	Count	2000	0	0	0
Algeria	Faidh El Botma	Count	2017	0	0	0
Algeria	Sidi Slimane	Proportion	2000	30.3	45.5	16.2
Algeria	Sidi Slimane	Proportion	2017	32.5	45.7	21.0
Algeria	Sidi Slimane	Count	2000	0	0	0
Algeria	Sidi Slimane	Count	2017	0	0	0
Algeria	Oum Touyour	Proportion	2000	31.0	46.1	19.0
Algeria	Oum Touyour	Proportion	2017	30.9	44.6	18.9
Algeria	Oum Touyour	Count	2000	0	0	0
Algeria	Oum Touyour	Count	2017	0	0	0
Algeria	Lac Des Oiseaux	Proportion	2000	32.6	48.5	19.3
Algeria	Lac Des Oiseaux	Proportion	2017	36.7	52.5	23.4
Algeria	Lac Des Oiseaux	Count	2000	0	0	0
Algeria	Lac Des Oiseaux	Count	2017	0	1	0
Algeria	Hammam Debagh	Proportion	2000	34.5	50.1	21.3
Algeria	Hammam Debagh	Proportion	2017	38.1	53.9	24.5
Algeria	Hammam Debagh	Count	2000	0	1	0
Algeria	Hammam Debagh	Count	2017	0	1	0

Table 4: ORS by Unit *(continued)*

Country	Unit	Metric	Year	Mean	Upper	Lower
Algeria	Oudjana	Proportion	2000	30.7	44.9	18.8
Algeria	Oudjana	Proportion	2017	38.6	56.1	24.4
Algeria	Oudjana	Count	2000	0	0	0
Algeria	Oudjana	Count	2017	0	1	0
Algeria	Tamza	Proportion	2000	30.2	44.5	18.5
Algeria	Tamza	Proportion	2017	35.7	50.5	20.7
Algeria	Tamza	Count	2000	0	0	0
Algeria	Tamza	Count	2017	0	1	0
Algeria	Sebgag	Proportion	2000	29.6	44.4	16.9
Algeria	Sebgag	Proportion	2017	32.0	44.7	20.3
Algeria	Sebgag	Count	2000	0	0	0
Algeria	Sebgag	Count	2017	0	0	0
Algeria	Hamri	Proportion	2000	32.3	47.2	19.1
Algeria	Hamri	Proportion	2017	34.2	50.7	19.3
Algeria	Hamri	Count	2000	0	0	0
Algeria	Hamri	Count	2017	0	1	0
Algeria	El Menaouer	Proportion	2000	26.8	41.9	16.1
Algeria	El Menaouer	Proportion	2017	34.3	48.5	20.1
Algeria	El Menaouer	Count	2000	0	0	0
Algeria	El Menaouer	Count	2017	0	0	0
Algeria	Cheniguel	Proportion	2000	34.9	53.1	20.4
Algeria	Cheniguel	Proportion	2017	36.3	52.4	21.9
Algeria	Cheniguel	Count	2000	0	1	0
Algeria	Cheniguel	Count	2017	0	1	0
Algeria	Oued Endja	Proportion	2000	33.6	45.3	20.6
Algeria	Oued Endja	Proportion	2017	38.4	55.0	23.7
Algeria	Oued Endja	Count	2000	0	0	0
Algeria	Oued Endja	Count	2017	0	1	0
Algeria	Oued El Kheir	Proportion	2000	26.0	40.0	15.0
Algeria	Oued El Kheir	Proportion	2017	35.5	51.3	20.6
Algeria	Oued El Kheir	Count	2000	0	0	0
Algeria	Oued El Kheir	Count	2017	0	1	0
Algeria	Marsat El Hadjadj	Proportion	2000	25.2	38.2	15.4
Algeria	Marsat El Hadjadj	Proportion	2017	34.5	49.1	20.6
Algeria	Marsat El Hadjadj	Count	2000	0	0	0
Algeria	Marsat El Hadjadj	Count	2017	0	0	0
Algeria	Temacine	Proportion	2000	30.1	46.2	18.5
Algeria	Temacine	Proportion	2017	29.3	42.7	17.4
Algeria	Temacine	Count	2000	0	0	0
Algeria	Temacine	Count	2017	0	0	0
Algeria	Hanchir Toumghani	Proportion	2000	33.0	48.9	19.7
Algeria	Hanchir Toumghani	Proportion	2017	36.4	51.1	21.9
Algeria	Hanchir Toumghani	Count	2000	0	0	0
Algeria	Hanchir Toumghani	Count	2017	0	1	0
Algeria	Mazouna	Proportion	2000	28.0	43.1	15.8
Algeria	Mazouna	Proportion	2017	35.1	51.7	20.0
Algeria	Mazouna	Count	2000	0	0	0
Algeria	Mazouna	Count	2017	0	1	0
Algeria	Beni-Ouartilane	Proportion	2000	32.5	48.7	20.2

Table 4: ORS by Unit *(continued)*

Country	Unit	Metric	Year	Mean	Upper	Lower
Algeria	Beni-Ouartilane	Proportion	2017	37.2	53.1	23.2
Algeria	Beni-Ouartilane	Count	2000	0	0	0
Algeria	Beni-Ouartilane	Count	2017	0	1	0
Algeria	Lemtar	Proportion	2000	29.0	44.2	16.4
Algeria	Lemtar	Proportion	2017	35.4	51.2	22.2
Algeria	Lemtar	Count	2000	0	0	0
Algeria	Lemtar	Count	2017	0	1	0
Algeria	El Marsa	Proportion	2000	32.8	49.4	20.4
Algeria	El Marsa	Proportion	2017	36.7	51.6	24.2
Algeria	El Marsa	Count	2000	0	0	0
Algeria	El Marsa	Count	2017	0	1	0
Algeria	Sedrata	Proportion	2000	33.6	50.9	20.8
Algeria	Sedrata	Proportion	2017	36.0	52.3	23.3
Algeria	Sedrata	Count	2000	0	1	0
Algeria	Sedrata	Count	2017	0	1	0
Algeria	Hammamet	Proportion	2000	30.4	44.3	17.0
Algeria	Hammamet	Proportion	2017	36.2	51.2	23.8
Algeria	Hammamet	Count	2000	0	0	0
Algeria	Hammamet	Count	2017	0	1	0
Algeria	Medrissa	Proportion	2000	31.4	47.0	19.0
Algeria	Medrissa	Proportion	2017	36.7	53.9	22.7
Algeria	Medrissa	Count	2000	0	0	0
Algeria	Medrissa	Count	2017	0	1	0
Algeria	Merad	Proportion	2000	32.0	46.8	19.9
Algeria	Merad	Proportion	2017	33.9	50.0	20.0
Algeria	Merad	Count	2000	0	0	0
Algeria	Merad	Count	2017	0	0	0
Algeria	Sidi Slimane	Proportion	2000	31.3	45.0	19.1
Algeria	Sidi Slimane	Proportion	2017	34.6	49.7	19.4
Algeria	Sidi Slimane	Count	2000	0	0	0
Algeria	Sidi Slimane	Count	2017	0	0	0
Algeria	Assi Youcef	Proportion	2000	29.3	46.6	16.2
Algeria	Assi Youcef	Proportion	2017	35.0	50.1	21.3
Algeria	Assi Youcef	Count	2000	0	0	0
Algeria	Assi Youcef	Count	2017	0	1	0
Algeria	Bouhlou	Proportion	2000	29.7	46.2	17.7
Algeria	Bouhlou	Proportion	2017	35.2	50.4	20.5
Algeria	Bouhlou	Count	2000	0	0	0
Algeria	Bouhlou	Count	2017	0	1	0
Algeria	Tamantit	Proportion	2000	28.6	40.5	17.8
Algeria	Tamantit	Proportion	2017	29.9	43.5	19.1
Algeria	Tamantit	Count	2000	0	0	0
Algeria	Tamantit	Count	2017	0	0	0
Algeria	El Amra	Proportion	2000	31.1	47.1	19.3
Algeria	El Amra	Proportion	2017	34.0	48.4	20.0
Algeria	El Amra	Count	2000	0	0	0
Algeria	El Amra	Count	2017	0	0	0
Algeria	Ouled Boudjemaa	Proportion	2000	28.2	40.9	17.3
Algeria	Ouled Boudjemaa	Proportion	2017	34.3	48.4	20.8

Table 4: ORS by Unit *(continued)*

Country	Unit	Metric	Year	Mean	Upper	Lower
Algeria	Ouled Boudjemaa	Count	2000	0	0	0
Algeria	Ouled Boudjemaa	Count	2017	0	0	0
Algeria	Rouiba	Proportion	2000	30.7	47.7	18.0
Algeria	Rouiba	Proportion	2017	33.1	49.1	20.5
Algeria	Rouiba	Count	2000	0	0	0
Algeria	Rouiba	Count	2017	0	0	0
Algeria	Fesdis	Proportion	2000	31.9	47.8	18.4
Algeria	Fesdis	Proportion	2017	37.1	52.8	21.8
Algeria	Fesdis	Count	2000	0	0	0
Algeria	Fesdis	Count	2017	0	1	0
Algeria	Tamtert	Proportion	2000	31.8	45.5	19.4
Algeria	Tamtert	Proportion	2017	32.9	47.0	19.7
Algeria	Tamtert	Count	2000	0	0	0
Algeria	Tamtert	Count	2017	0	0	0
Algeria	Darguina	Proportion	2000	31.1	45.5	17.8
Algeria	Darguina	Proportion	2017	37.8	55.3	23.8
Algeria	Darguina	Count	2000	0	0	0
Algeria	Darguina	Count	2017	0	1	0
Algeria	Lichana	Proportion	2000	31.1	44.2	18.4
Algeria	Lichana	Proportion	2017	31.7	44.6	19.3
Algeria	Lichana	Count	2000	0	0	0
Algeria	Lichana	Count	2017	0	0	0
Algeria	Oued Djer	Proportion	2000	31.4	45.3	19.5
Algeria	Oued Djer	Proportion	2017	34.3	49.1	20.1
Algeria	Oued Djer	Count	2000	0	0	0
Algeria	Oued Djer	Count	2017	0	0	0
Algeria	Khelil	Proportion	2000	33.1	46.5	20.6
Algeria	Khelil	Proportion	2017	37.2	52.9	21.8
Algeria	Khelil	Count	2000	0	0	0
Algeria	Khelil	Count	2017	0	1	0
Algeria	Djebahia	Proportion	2000	30.9	48.1	18.7
Algeria	Djebahia	Proportion	2017	36.2	50.1	22.5
Algeria	Djebahia	Count	2000	0	0	0
Algeria	Djebahia	Count	2017	0	1	0
Algeria	Leghata	Proportion	2000	29.8	49.3	17.5
Algeria	Leghata	Proportion	2017	35.6	49.8	21.9
Algeria	Leghata	Count	2000	0	0	0
Algeria	Leghata	Count	2017	0	0	0
Algeria	Oued Fodda	Proportion	2000	30.1	44.4	18.2
Algeria	Oued Fodda	Proportion	2017	35.4	49.8	21.2
Algeria	Oued Fodda	Count	2000	0	0	0
Algeria	Oued Fodda	Count	2017	0	0	0
Algeria	Guernini	Proportion	2000	30.7	44.9	16.3
Algeria	Guernini	Proportion	2017	32.3	45.9	19.5
Algeria	Guernini	Count	2000	0	0	0
Algeria	Guernini	Count	2017	0	0	0
Algeria	Sidi Tifour	Proportion	2000	30.3	44.0	17.9
Algeria	Sidi Tifour	Proportion	2017	32.1	45.2	20.6
Algeria	Sidi Tifour	Count	2000	0	0	0

Table 4: ORS by Unit *(continued)*

Country	Unit	Metric	Year	Mean	Upper	Lower
Algeria	Sidi Tifour	Count	2017	0	0	0
Algeria	Ourmes	Proportion	2000	30.5	47.9	18.3
Algeria	Ourmes	Proportion	2017	30.7	45.7	18.9
Algeria	Ourmes	Count	2000	0	0	0
Algeria	Ourmes	Count	2017	0	0	0
Algeria	Oued Zitoun	Proportion	2000	33.1	48.6	20.0
Algeria	Oued Zitoun	Proportion	2017	35.9	52.1	23.0
Algeria	Oued Zitoun	Count	2000	0	0	0
Algeria	Oued Zitoun	Count	2017	0	1	0
Algeria	Hammam N'Bail	Proportion	2000	33.3	49.1	20.5
Algeria	Hammam N'Bail	Proportion	2017	36.8	54.5	23.4
Algeria	Hammam N'Bail	Count	2000	0	0	0
Algeria	Hammam N'Bail	Count	2017	0	1	0
Algeria	Ouled Rabah	Proportion	2000	32.8	45.7	20.1
Algeria	Ouled Rabah	Proportion	2017	37.0	53.3	23.3
Algeria	Ouled Rabah	Count	2000	0	0	0
Algeria	Ouled Rabah	Count	2017	0	1	0
Algeria	Taouzianat	Proportion	2000	31.4	45.7	20.4
Algeria	Taouzianat	Proportion	2017	35.5	50.4	21.1
Algeria	Taouzianat	Count	2000	0	0	0
Algeria	Taouzianat	Count	2017	0	1	0
Algeria	Sidi Bouzid	Proportion	2000	29.8	44.0	18.0
Algeria	Sidi Bouzid	Proportion	2017	32.4	45.4	21.2
Algeria	Sidi Bouzid	Count	2000	0	0	0
Algeria	Sidi Bouzid	Count	2017	0	0	0
Algeria	Khatouti Sed Eldjir	Proportion	2000	32.5	48.2	18.0
Algeria	Khatouti Sed Eldjir	Proportion	2017	34.4	50.3	22.6
Algeria	Khatouti Sed Eldjir	Count	2000	0	0	0
Algeria	Khatouti Sed Eldjir	Count	2017	0	1	0
Algeria	Ferraguig	Proportion	2000	26.2	40.2	15.1
Algeria	Ferraguig	Proportion	2017	34.4	51.2	19.7
Algeria	Ferraguig	Count	2000	0	0	0
Algeria	Ferraguig	Count	2017	0	1	0
Algeria	Derrag	Proportion	2000	32.3	50.7	20.0
Algeria	Derrag	Proportion	2017	34.4	48.9	20.5
Algeria	Derrag	Count	2000	0	1	0
Algeria	Derrag	Count	2017	0	0	0
Algeria	Oued Seguen	Proportion	2000	34.3	47.8	21.5
Algeria	Oued Seguen	Proportion	2017	37.2	52.8	22.3
Algeria	Oued Seguen	Count	2000	0	0	0
Algeria	Oued Seguen	Count	2017	0	1	0
Algeria	Ouled Boughalem	Proportion	2000	27.4	43.6	15.3
Algeria	Ouled Boughalem	Proportion	2017	36.4	52.1	22.2
Algeria	Ouled Boughalem	Count	2000	0	0	0
Algeria	Ouled Boughalem	Count	2017	0	1	0
Algeria	Mers El Kebir	Proportion	2000	27.6	41.5	15.7
Algeria	Mers El Kebir	Proportion	2017	35.0	51.0	21.8
Algeria	Mers El Kebir	Count	2000	0	0	0
Algeria	Mers El Kebir	Count	2017	0	1	0

Table 4: ORS by Unit *(continued)*

Country	Unit	Metric	Year	Mean	Upper	Lower
Algeria	Touggourt	Proportion	2000	29.5	46.5	17.6
Algeria	Touggourt	Proportion	2017	28.8	42.6	16.8
Algeria	Touggourt	Count	2000	0	0	0
Algeria	Touggourt	Count	2017	0	0	0
Algeria	Ksar El Sbihi	Proportion	2000	33.7	48.0	21.5
Algeria	Ksar El Sbihi	Proportion	2017	36.7	51.1	23.5
Algeria	Ksar El Sbihi	Count	2000	0	0	0
Algeria	Ksar El Sbihi	Count	2017	0	1	0
Algeria	Mediouna	Proportion	2000	27.4	43.4	15.6
Algeria	Mediouna	Proportion	2017	35.2	51.4	21.2
Algeria	Mediouna	Count	2000	0	0	0
Algeria	Mediouna	Count	2017	0	1	0
Algeria	Beni Chebana	Proportion	2000	32.8	47.8	20.4
Algeria	Beni Chebana	Proportion	2017	37.0	52.4	23.0
Algeria	Beni Chebana	Count	2000	0	0	0
Algeria	Beni Chebana	Count	2017	0	1	0
Algeria	M'Cid	Proportion	2000	27.9	44.3	15.2
Algeria	M'Cid	Proportion	2017	35.0	49.5	20.7
Algeria	M'Cid	Count	2000	0	0	0
Algeria	M'Cid	Count	2017	0	0	0
Algeria	Emjez Edchich	Proportion	2000	33.1	48.1	20.3
Algeria	Emjez Edchich	Proportion	2017	38.9	54.0	23.9
Algeria	Emjez Edchich	Count	2000	0	0	0
Algeria	Emjez Edchich	Count	2017	0	1	0
Algeria	Sidi Fredj	Proportion	2000	33.7	49.0	21.9
Algeria	Sidi Fredj	Proportion	2017	36.3	51.6	23.0
Algeria	Sidi Fredj	Count	2000	0	0	0
Algeria	Sidi Fredj	Count	2017	0	1	0
Algeria	Morsot	Proportion	2000	31.9	47.0	18.9
Algeria	Morsot	Proportion	2017	36.8	51.3	23.2
Algeria	Morsot	Count	2000	0	0	0
Algeria	Morsot	Count	2017	0	1	0
Algeria	Medroussa	Proportion	2000	30.6	45.9	17.9
Algeria	Medroussa	Proportion	2017	34.5	52.2	20.3
Algeria	Medroussa	Count	2000	0	0	0
Algeria	Medroussa	Count	2017	0	1	0
Algeria	Messelmoun	Proportion	2000	30.5	44.1	18.9
Algeria	Messelmoun	Proportion	2017	33.1	49.0	21.5
Algeria	Messelmoun	Count	2000	0	0	0
Algeria	Messelmoun	Count	2017	0	0	0
Algeria	Tamelaht	Proportion	2000	31.6	46.6	19.1
Algeria	Tamelaht	Proportion	2017	35.0	49.8	19.3
Algeria	Tamelaht	Count	2000	0	0	0
Algeria	Tamelaht	Count	2017	0	0	0
Algeria	Azazga	Proportion	2000	28.6	44.4	17.0
Algeria	Azazga	Proportion	2017	35.9	49.6	22.0
Algeria	Azazga	Count	2000	0	0	0
Algeria	Azazga	Count	2017	0	0	0
Algeria	Bouihi	Proportion	2000	31.8	50.9	20.1

Table 4: ORS by Unit *(continued)*

Country	Unit	Metric	Year	Mean	Upper	Lower
Algeria	Bouihi	Proportion	2017	34.7	48.9	20.1
Algeria	Bouihi	Count	2000	0	1	0
Algeria	Bouihi	Count	2017	0	0	0
Algeria	Tamekten	Proportion	2000	28.7	42.5	18.8
Algeria	Tamekten	Proportion	2017	30.5	44.0	19.6
Algeria	Tamekten	Count	2000	0	0	0
Algeria	Tamekten	Count	2017	0	0	0
Algeria	El Attaf	Proportion	2000	30.0	45.8	18.1
Algeria	El Attaf	Proportion	2017	34.8	49.1	20.0
Algeria	El Attaf	Count	2000	0	0	0
Algeria	El Attaf	Count	2017	0	0	0
Algeria	Ouled Kihel	Proportion	2000	28.4	41.5	16.6
Algeria	Ouled Kihel	Proportion	2017	34.1	48.3	20.4
Algeria	Ouled Kihel	Count	2000	0	0	0
Algeria	Ouled Kihel	Count	2017	0	0	0
Algeria	Saoula	Proportion	2000	30.5	45.0	19.4
Algeria	Saoula	Proportion	2017	31.2	47.0	17.6
Algeria	Saoula	Count	2000	0	0	0
Algeria	Saoula	Count	2017	0	0	0
Algeria	Foum Toub	Proportion	2000	30.7	44.8	18.1
Algeria	Foum Toub	Proportion	2017	35.5	51.3	22.0
Algeria	Foum Toub	Count	2000	0	0	0
Algeria	Foum Toub	Count	2017	0	1	0
Algeria	Timoudi	Proportion	2000	33.0	47.0	20.6
Algeria	Timoudi	Proportion	2017	33.6	46.1	21.8
Algeria	Timoudi	Count	2000	0	0	0
Algeria	Timoudi	Count	2017	0	0	0
Algeria	Draa El Caid	Proportion	2000	33.3	47.2	19.9
Algeria	Draa El Caid	Proportion	2017	37.8	53.9	23.2
Algeria	Draa El Caid	Count	2000	0	0	0
Algeria	Draa El Caid	Count	2017	0	1	0
Algeria	Lioua	Proportion	2000	30.0	44.7	18.4
Algeria	Lioua	Proportion	2017	31.7	45.2	19.0
Algeria	Lioua	Count	2000	0	0	0
Algeria	Lioua	Count	2017	0	0	0
Algeria	Oued El Alleug	Proportion	2000	30.8	46.0	18.7
Algeria	Oued El Alleug	Proportion	2017	34.3	51.9	19.4
Algeria	Oued El Alleug	Count	2000	0	0	0
Algeria	Oued El Alleug	Count	2017	0	1	0
Algeria	Ksour	Proportion	2000	33.1	47.9	20.2
Algeria	Ksour	Proportion	2017	36.7	52.0	21.7
Algeria	Ksour	Count	2000	0	0	0
Algeria	Ksour	Count	2017	0	1	0
Algeria	El Adjiba	Proportion	2000	32.0	48.9	18.3
Algeria	El Adjiba	Proportion	2017	37.3	52.5	23.7
Algeria	El Adjiba	Count	2000	0	0	0
Algeria	El Adjiba	Count	2017	0	1	0
Algeria	Naciria	Proportion	2000	29.1	47.6	16.8
Algeria	Naciria	Proportion	2017	34.7	49.1	21.1

Table 4: ORS by Unit *(continued)*

Country	Unit	Metric	Year	Mean	Upper	Lower
Algeria	Naciria	Count	2000	0	0	0
Algeria	Naciria	Count	2017	0	0	0
Algeria	Oued Goussine	Proportion	2000	29.6	43.5	18.0
Algeria	Oued Goussine	Proportion	2017	33.6	49.9	19.0
Algeria	Oued Goussine	Count	2000	0	0	0
Algeria	Oued Goussine	Count	2017	0	0	0
Algeria	Guettara	Proportion	2000	30.4	44.8	18.0
Algeria	Guettara	Proportion	2017	30.5	43.1	20.0
Algeria	Guettara	Count	2000	0	0	0
Algeria	Guettara	Count	2017	0	0	0
Algeria	Stitten	Proportion	2000	30.1	44.2	17.2
Algeria	Stitten	Proportion	2017	33.2	47.5	20.5
Algeria	Stitten	Count	2000	0	0	0
Algeria	Stitten	Count	2017	0	0	0
Algeria	Reguiba	Proportion	2000	31.1	48.2	18.2
Algeria	Reguiba	Proportion	2017	31.3	45.0	18.6
Algeria	Reguiba	Count	2000	0	0	0
Algeria	Reguiba	Count	2017	0	0	0
Algeria	Raml Souk	Proportion	2000	35.0	49.5	22.7
Algeria	Raml Souk	Proportion	2017	43.7	58.2	28.5
Algeria	Raml Souk	Count	2000	0	0	0
Algeria	Raml Souk	Count	2017	0	1	0
Algeria	Helliopolis	Proportion	2000	35.4	52.2	22.1
Algeria	Helliopolis	Proportion	2017	36.3	51.9	23.2
Algeria	Helliopolis	Count	2000	0	1	0
Algeria	Helliopolis	Count	2017	0	1	0
Algeria	Ouled Yahia Khadrouche	Proportion	2000	32.1	45.3	19.3
Algeria	Ouled Yahia Khadrouche	Proportion	2017	37.4	53.7	23.4
Algeria	Ouled Yahia Khadrouche	Count	2000	0	0	0
Algeria	Ouled Yahia Khadrouche	Count	2017	0	1	0
Algeria	Yabous	Proportion	2000	30.4	45.5	18.8
Algeria	Yabous	Proportion	2017	35.5	51.3	21.4
Algeria	Yabous	Count	2000	0	0	0
Algeria	Yabous	Count	2017	0	1	0
Algeria	Sidi Makhlouf	Proportion	2000	31.2	48.4	18.9
Algeria	Sidi Makhlouf	Proportion	2017	33.3	49.0	21.8
Algeria	Sidi Makhlouf	Count	2000	0	0	0
Algeria	Sidi Makhlouf	Count	2017	0	0	0
Algeria	Khoubana	Proportion	2000	31.7	46.4	19.2
Algeria	Khoubana	Proportion	2017	33.7	49.0	21.4
Algeria	Khoubana	Count	2000	0	0	0
Algeria	Khoubana	Count	2017	0	0	0
Algeria	Froha	Proportion	2000	28.0	41.7	16.4
Algeria	Froha	Proportion	2017	35.2	51.3	21.8
Algeria	Froha	Count	2000	0	0	0
Algeria	Froha	Count	2017	0	1	0
Algeria	Deux Bassins	Proportion	2000	32.3	47.7	19.7
Algeria	Deux Bassins	Proportion	2017	34.2	48.8	21.8
Algeria	Deux Bassins	Count	2000	0	0	0

Table 4: ORS by Unit *(continued)*

Country	Unit	Metric	Year	Mean	Upper	Lower
Algeria	Deux Bassins	Count	2017	0	0	0
Algeria	Ouled Khelouf	Proportion	2000	34.2	48.3	19.4
Algeria	Ouled Khelouf	Proportion	2017	36.8	50.6	21.7
Algeria	Ouled Khelouf	Count	2000	0	0	0
Algeria	Ouled Khelouf	Count	2017	0	1	0
Algeria	Ouled Maalah	Proportion	2000	26.6	42.1	15.8
Algeria	Ouled Maalah	Proportion	2017	35.7	51.3	21.0
Algeria	Ouled Maalah	Count	2000	0	0	0
Algeria	Ouled Maalah	Count	2017	0	1	0
Algeria	Misserghin	Proportion	2000	27.6	41.6	15.9
Algeria	Misserghin	Proportion	2017	34.9	50.6	21.3
Algeria	Misserghin	Count	2000	0	0	0
Algeria	Misserghin	Count	2017	0	1	0
Algeria	Zaouia El Abidia	Proportion	2000	30.3	46.8	18.3
Algeria	Zaouia El Abidia	Proportion	2017	30.2	44.4	18.1
Algeria	Zaouia El Abidia	Count	2000	0	0	0
Algeria	Zaouia El Abidia	Count	2017	0	0	0
Algeria	Meskiana	Proportion	2000	32.0	47.9	18.9
Algeria	Meskiana	Proportion	2017	36.0	51.1	23.1
Algeria	Meskiana	Count	2000	0	0	0
Algeria	Meskiana	Count	2017	0	1	0
Algeria	Mendes	Proportion	2000	30.8	48.8	18.0
Algeria	Mendes	Proportion	2017	35.5	51.1	21.3
Algeria	Mendes	Count	2000	0	0	0
Algeria	Mendes	Count	2017	0	1	0
Algeria	Beni Fouda	Proportion	2000	33.5	46.7	20.2
Algeria	Beni Fouda	Proportion	2017	36.9	53.7	21.4
Algeria	Beni Fouda	Count	2000	0	0	0
Algeria	Beni Fouda	Count	2017	0	1	0
Algeria	Mekkedra	Proportion	2000	26.9	40.6	15.6
Algeria	Mekkedra	Proportion	2017	34.9	50.3	20.6
Algeria	Mekkedra	Count	2000	0	0	0
Algeria	Mekkedra	Count	2017	0	1	0
Algeria	Es Sebt	Proportion	2000	34.7	50.0	21.3
Algeria	Es Sebt	Proportion	2017	38.9	55.6	26.0
Algeria	Es Sebt	Count	2000	0	0	0
Algeria	Es Sebt	Count	2017	0	1	0
Algeria	Souk Ahras	Proportion	2000	32.0	48.9	19.0
Algeria	Souk Ahras	Proportion	2017	34.9	52.5	22.3
Algeria	Souk Ahras	Count	2000	0	0	0
Algeria	Souk Ahras	Count	2017	0	1	0
Algeria	Negrine	Proportion	2000	30.8	46.3	18.1
Algeria	Negrine	Proportion	2017	36.0	51.2	21.4
Algeria	Negrine	Count	2000	0	0	0
Algeria	Negrine	Count	2017	0	1	0
Algeria	Meghila	Proportion	2000	31.7	46.6	18.0
Algeria	Meghila	Proportion	2017	34.9	50.0	20.0
Algeria	Meghila	Count	2000	0	0	0
Algeria	Meghila	Count	2017	0	0	0

Table 4: ORS by Unit *(continued)*

Country	Unit	Metric	Year	Mean	Upper	Lower
Algeria	Nador	Proportion	2000	31.5	46.3	19.6
Algeria	Nador	Proportion	2017	33.9	48.8	21.0
Algeria	Nador	Count	2000	0	0	0
Algeria	Nador	Count	2017	0	0	0
Algeria	Theniet El Had	Proportion	2000	31.8	47.4	18.8
Algeria	Theniet El Had	Proportion	2017	34.6	49.3	19.8
Algeria	Theniet El Had	Count	2000	0	0	0
Algeria	Theniet El Had	Count	2017	0	0	0
Algeria	Azzefoun	Proportion	2000	28.5	45.4	17.3
Algeria	Azzefoun	Proportion	2017	33.9	47.3	20.6
Algeria	Azzefoun	Count	2000	0	0	0
Algeria	Azzefoun	Count	2017	0	0	0
Algeria	Chetouane	Proportion	2000	30.2	47.3	17.7
Algeria	Chetouane	Proportion	2017	35.1	51.8	21.5
Algeria	Chetouane	Count	2000	0	0	0
Algeria	Chetouane	Count	2017	0	1	0
Algeria	Tamest	Proportion	2000	28.5	41.6	16.1
Algeria	Tamest	Proportion	2017	30.9	43.8	20.2
Algeria	Tamest	Count	2000	0	0	0
Algeria	Tamest	Count	2017	0	0	0
Algeria	El Hassania	Proportion	2000	32.6	48.1	19.5
Algeria	El Hassania	Proportion	2017	34.4	49.5	19.8
Algeria	El Hassania	Count	2000	0	0	0
Algeria	El Hassania	Count	2017	0	0	0
Algeria	Oulhaca El Gheraba	Proportion	2000	28.9	45.1	16.8
Algeria	Oulhaca El Gheraba	Proportion	2017	34.5	49.4	19.8
Algeria	Oulhaca El Gheraba	Count	2000	0	0	0
Algeria	Oulhaca El Gheraba	Count	2017	0	0	0
Algeria	Sidi Moussa	Proportion	2000	30.8	45.5	19.9
Algeria	Sidi Moussa	Proportion	2017	35.7	50.6	21.7
Algeria	Sidi Moussa	Count	2000	0	0	0
Algeria	Sidi Moussa	Count	2017	0	1	0
Algeria	Ghessira	Proportion	2000	31.5	45.8	18.8
Algeria	Ghessira	Proportion	2017	37.0	53.3	22.5
Algeria	Ghessira	Count	2000	0	0	0
Algeria	Ghessira	Count	2017	0	1	0
Algeria	El Kseur	Proportion	2000	29.9	45.2	18.6
Algeria	El Kseur	Proportion	2017	34.0	48.2	20.6
Algeria	El Kseur	Count	2000	0	0	0
Algeria	El Kseur	Count	2017	0	0	0
Algeria	M'Lili	Proportion	2000	30.8	44.1	18.4
Algeria	M'Lili	Proportion	2017	31.4	45.3	19.2
Algeria	M'Lili	Count	2000	0	0	0
Algeria	M'Lili	Count	2017	0	0	0
Algeria	Ouled Slama	Proportion	2000	31.3	47.4	20.3
Algeria	Ouled Slama	Proportion	2017	36.5	51.5	22.9
Algeria	Ouled Slama	Count	2000	0	0	0
Algeria	Ouled Slama	Count	2017	0	1	0
Algeria	Mansoura	Proportion	2000	33.6	49.3	19.9

Table 4: ORS by Unit *(continued)*

Country	Unit	Metric	Year	Mean	Upper	Lower
Algeria	Mansoura	Proportion	2017	36.6	52.3	22.5
Algeria	Mansoura	Count	2000	0	0	0
Algeria	Mansoura	Count	2017	0	1	0
Algeria	El Asnam	Proportion	2000	31.8	47.9	18.9
Algeria	El Asnam	Proportion	2017	36.7	53.0	22.9
Algeria	El Asnam	Count	2000	0	0	0
Algeria	El Asnam	Count	2017	0	1	0
Algeria	Ouled Aissa	Proportion	2000	29.4	48.4	17.1
Algeria	Ouled Aissa	Proportion	2017	35.2	49.3	21.5
Algeria	Ouled Aissa	Count	2000	0	0	0
Algeria	Ouled Aissa	Count	2017	0	0	0
Algeria	Oued Sly	Proportion	2000	29.2	44.8	16.8
Algeria	Oued Sly	Proportion	2017	35.7	54.2	21.0
Algeria	Oued Sly	Count	2000	0	0	0
Algeria	Oued Sly	Count	2017	0	1	0
Algeria	Had Sahary	Proportion	2000	31.3	47.2	17.2
Algeria	Had Sahary	Proportion	2017	32.7	45.6	20.1
Algeria	Had Sahary	Count	2000	0	0	0
Algeria	Had Sahary	Count	2017	0	0	0
Algeria	Tousmouline	Proportion	2000	30.7	44.7	17.9
Algeria	Tousmouline	Proportion	2017	33.6	46.5	18.7
Algeria	Tousmouline	Count	2000	0	0	0
Algeria	Tousmouline	Count	2017	0	0	0
Algeria	Robbah	Proportion	2000	30.3	47.1	17.6
Algeria	Robbah	Proportion	2017	30.1	46.0	17.8
Algeria	Robbah	Count	2000	0	0	0
Algeria	Robbah	Count	2017	0	0	0
Algeria	Souarekh	Proportion	2000	31.7	46.3	19.7
Algeria	Souarekh	Proportion	2017	36.0	50.9	22.6
Algeria	Souarekh	Count	2000	0	0	0
Algeria	Souarekh	Count	2017	0	1	0
Algeria	Houari Boumediene	Proportion	2000	33.9	50.4	21.3
Algeria	Houari Boumediene	Proportion	2017	38.0	53.8	24.4
Algeria	Houari Boumediene	Count	2000	0	1	0
Algeria	Houari Boumediene	Count	2017	0	1	0
Algeria	Selma Benziada	Proportion	2000	32.3	46.4	18.9
Algeria	Selma Benziada	Proportion	2017	37.6	54.2	24.0
Algeria	Selma Benziada	Count	2000	0	0	0
Algeria	Selma Benziada	Count	2017	0	1	0
Algeria	Tadjemout	Proportion	2000	30.7	46.9	19.1
Algeria	Tadjemout	Proportion	2017	33.3	48.4	22.8
Algeria	Tadjemout	Count	2000	0	0	0
Algeria	Tadjemout	Count	2017	0	0	0
Algeria	M'Cif	Proportion	2000	31.8	47.2	20.0
Algeria	M'Cif	Proportion	2017	33.5	48.7	20.4
Algeria	M'Cif	Count	2000	0	0	0
Algeria	M'Cif	Count	2017	0	0	0
Algeria	Gharrous	Proportion	2000	30.0	45.7	18.5
Algeria	Gharrous	Proportion	2017	35.3	50.6	21.5

Table 4: ORS by Unit *(continued)*

Country	Unit	Metric	Year	Mean	Upper	Lower
Algeria	Gharrous	Count	2000	0	0	0
Algeria	Gharrous	Count	2017	0	1	0
Algeria	Djouab	Proportion	2000	34.3	52.2	20.2
Algeria	Djouab	Proportion	2017	35.7	50.5	23.2
Algeria	Djouab	Count	2000	0	1	0
Algeria	Djouab	Count	2017	0	1	0
Algeria	Rouached	Proportion	2000	33.2	45.8	20.3
Algeria	Rouached	Proportion	2017	38.1	54.1	23.2
Algeria	Rouached	Count	2000	0	0	0
Algeria	Rouached	Count	2017	0	1	0
Algeria	Safsaf	Proportion	2000	25.9	40.1	15.3
Algeria	Safsaf	Proportion	2017	35.3	51.4	21.0
Algeria	Safsaf	Count	2000	0	0	0
Algeria	Safsaf	Count	2017	0	1	0
Algeria	Oran	Proportion	2000	26.7	40.5	15.6
Algeria	Oran	Proportion	2017	33.0	47.8	19.7
Algeria	Oran	Count	2000	0	0	0
Algeria	Oran	Count	2017	0	0	0
Algeria	Oued Nini	Proportion	2000	31.5	47.8	18.9
Algeria	Oued Nini	Proportion	2017	35.8	49.6	21.1
Algeria	Oued Nini	Count	2000	0	0	0
Algeria	Oued Nini	Count	2017	0	0	0
Algeria	Merdja Sidi Abed	Proportion	2000	28.7	44.1	16.7
Algeria	Merdja Sidi Abed	Proportion	2017	35.6	52.4	20.8
Algeria	Merdja Sidi Abed	Count	2000	0	0	0
Algeria	Merdja Sidi Abed	Count	2017	0	1	0
Algeria	Beni Oussine	Proportion	2000	33.6	46.9	20.8
Algeria	Beni Oussine	Proportion	2017	37.5	53.0	22.4
Algeria	Beni Oussine	Count	2000	0	0	0
Algeria	Beni Oussine	Count	2017	0	1	0
Algeria	Merhoum	Proportion	2000	31.3	46.2	19.7
Algeria	Merhoum	Proportion	2017	34.9	50.6	20.8
Algeria	Merhoum	Count	2000	0	0	0
Algeria	Merhoum	Count	2017	0	1	0
Algeria	Filfila	Proportion	2000	33.7	50.6	20.5
Algeria	Filfila	Proportion	2017	34.9	51.0	22.2
Algeria	Filfila	Count	2000	0	1	0
Algeria	Filfila	Count	2017	0	1	0
Algeria	Taoura	Proportion	2000	33.3	49.0	21.0
Algeria	Taoura	Proportion	2017	35.9	52.5	23.6
Algeria	Taoura	Count	2000	0	0	0
Algeria	Taoura	Count	2017	0	1	0
Algeria	Ogla Melha	Proportion	2000	30.3	45.8	16.0
Algeria	Ogla Melha	Proportion	2017	35.3	48.8	22.7
Algeria	Ogla Melha	Count	2000	0	0	0
Algeria	Ogla Melha	Count	2017	0	0	0
Algeria	Mellakou	Proportion	2000	30.7	45.9	17.8
Algeria	Mellakou	Proportion	2017	34.5	50.8	21.2
Algeria	Mellakou	Count	2000	0	0	0

Table 4: ORS by Unit *(continued)*

Country	Unit	Metric	Year	Mean	Upper	Lower
Algeria	Mellakou	Count	2017	0	1	0
Algeria	Sidi Amar	Proportion	2000	31.6	46.2	19.9
Algeria	Sidi Amar	Proportion	2017	33.9	48.9	21.1
Algeria	Sidi Amar	Count	2000	0	0	0
Algeria	Sidi Amar	Count	2017	0	0	0
Algeria	Tissemsilt	Proportion	2000	32.9	49.1	20.0
Algeria	Tissemsilt	Proportion	2017	34.8	50.5	19.5
Algeria	Tissemsilt	Count	2000	0	0	0
Algeria	Tissemsilt	Count	2017	0	1	0
Algeria	Beni-Douala	Proportion	2000	28.6	46.6	16.4
Algeria	Beni-Douala	Proportion	2017	34.5	47.9	20.9
Algeria	Beni-Douala	Count	2000	0	0	0
Algeria	Beni-Douala	Count	2017	0	0	0
Algeria	Dar Yaghmouracene	Proportion	2000	30.0	47.5	18.2
Algeria	Dar Yaghmouracene	Proportion	2017	35.6	51.7	20.8
Algeria	Dar Yaghmouracene	Count	2000	0	0	0
Algeria	Dar Yaghmouracene	Count	2017	0	1	0
Algeria	Timiaouine	Proportion	2000	30.8	44.9	20.0
Algeria	Timiaouine	Proportion	2017	29.4	42.7	18.9
Algeria	Timiaouine	Count	2000	0	0	0
Algeria	Timiaouine	Count	2017	0	0	0
Algeria	El Maine	Proportion	2000	31.2	46.9	18.9
Algeria	El Maine	Proportion	2017	34.8	49.2	20.2
Algeria	El Maine	Count	2000	0	0	0
Algeria	El Maine	Count	2017	0	0	0
Algeria	Sidi Ben Adda	Proportion	2000	29.0	43.3	17.6
Algeria	Sidi Ben Adda	Proportion	2017	34.7	49.3	20.8
Algeria	Sidi Ben Adda	Count	2000	0	0	0
Algeria	Sidi Ben Adda	Count	2017	0	0	0
Algeria	Souidania	Proportion	2000	30.7	45.9	19.1
Algeria	Souidania	Proportion	2017	32.2	47.9	18.5
Algeria	Souidania	Count	2000	0	0	0
Algeria	Souidania	Count	2017	0	0	0
Algeria	Gosbat	Proportion	2000	33.1	47.4	21.9
Algeria	Gosbat	Proportion	2017	36.7	51.6	22.5
Algeria	Gosbat	Count	2000	0	0	0
Algeria	Gosbat	Count	2017	0	1	0
Algeria	Feraoun	Proportion	2000	30.8	45.3	18.7
Algeria	Feraoun	Proportion	2017	36.5	51.2	23.6
Algeria	Feraoun	Count	2000	0	0	0
Algeria	Feraoun	Count	2017	0	1	0
Algeria	M__Ziraa	Proportion	2000	29.5	43.2	17.6
Algeria	M__Ziraa	Proportion	2017	33.2	47.4	20.6
Algeria	M__Ziraa	Count	2000	0	0	0
Algeria	M__Ziraa	Count	2017	0	0	0
Algeria	Ouled Yaich	Proportion	2000	30.6	45.2	18.9
Algeria	Ouled Yaich	Proportion	2017	31.6	49.5	18.0
Algeria	Ouled Yaich	Count	2000	0	0	0
Algeria	Ouled Yaich	Count	2017	0	0	0

Table 4: ORS by Unit *(continued)*

Country	Unit	Metric	Year	Mean	Upper	Lower
Algeria	Medjana	Proportion	2000	33.2	48.3	20.6
Algeria	Medjana	Proportion	2017	37.1	52.6	21.9
Algeria	Medjana	Count	2000	0	0	0
Algeria	Medjana	Count	2017	0	1	0
Algeria	El Hachimia	Proportion	2000	33.2	49.4	21.1
Algeria	El Hachimia	Proportion	2017	36.4	52.5	22.8
Algeria	El Hachimia	Count	2000	0	0	0
Algeria	El Hachimia	Count	2017	0	1	0
Algeria	Ouled Hedadj	Proportion	2000	30.7	48.0	18.0
Algeria	Ouled Hedadj	Proportion	2017	32.9	48.5	20.3
Algeria	Ouled Hedadj	Count	2000	0	0	0
Algeria	Ouled Hedadj	Count	2017	0	0	0
Algeria	Ouled Abbes	Proportion	2000	29.8	44.5	18.1
Algeria	Ouled Abbes	Proportion	2017	34.8	50.0	19.9
Algeria	Ouled Abbes	Count	2000	0	0	0
Algeria	Ouled Abbes	Count	2017	0	0	0
Algeria	Hassi Bahbah	Proportion	2000	31.2	48.1	16.5
Algeria	Hassi Bahbah	Proportion	2017	34.3	49.4	21.6
Algeria	Hassi Bahbah	Count	2000	0	0	0
Algeria	Hassi Bahbah	Count	2017	0	0	0
Algeria	Sidi Amrane	Proportion	2000	30.5	46.7	18.9
Algeria	Sidi Amrane	Proportion	2017	31.6	46.0	19.3
Algeria	Sidi Amrane	Count	2000	0	0	0
Algeria	Sidi Amrane	Count	2017	0	0	0
Algeria	Zerizer	Proportion	2000	32.8	48.6	19.4
Algeria	Zerizer	Proportion	2017	36.9	52.6	23.4
Algeria	Zerizer	Count	2000	0	0	0
Algeria	Zerizer	Count	2017	0	1	0
Algeria	Kalaat Bousbaa	Proportion	2000	34.5	50.8	21.9
Algeria	Kalaat Bousbaa	Proportion	2017	37.4	53.6	24.1
Algeria	Kalaat Bousbaa	Count	2000	0	1	0
Algeria	Kalaat Bousbaa	Count	2017	0	1	0
Algeria	Settara	Proportion	2000	33.7	46.8	21.0
Algeria	Settara	Proportion	2017	39.1	54.3	25.4
Algeria	Settara	Count	2000	0	0	0
Algeria	Settara	Count	2017	0	1	0
Algeria	Tadjrouna	Proportion	2000	31.0	48.1	18.6
Algeria	Tadjrouna	Proportion	2017	32.5	47.7	21.1
Algeria	Tadjrouna	Count	2000	0	0	0
Algeria	Tadjrouna	Count	2017	0	0	0
Algeria	M'Sila	Proportion	2000	32.3	48.0	19.4
Algeria	M'Sila	Proportion	2017	35.5	51.7	22.3
Algeria	M'Sila	Count	2000	0	0	0
Algeria	M'Sila	Count	2017	0	1	0
Algeria	Ghriss	Proportion	2000	28.8	42.7	17.4
Algeria	Ghriss	Proportion	2017	35.2	51.6	21.9
Algeria	Ghriss	Count	2000	0	0	0
Algeria	Ghriss	Count	2017	0	1	0
Algeria	Draa Smar	Proportion	2000	33.8	49.6	20.8

Table 4: ORS by Unit *(continued)*

Country	Unit	Metric	Year	Mean	Upper	Lower
Algeria	Draa Smar	Proportion	2017	34.8	49.7	20.8
Algeria	Draa Smar	Count	2000	0	0	0
Algeria	Draa Smar	Count	2017	0	0	0
Algeria	Sidi Khelifa	Proportion	2000	34.2	47.6	22.1
Algeria	Sidi Khelifa	Proportion	2017	37.3	53.7	23.2
Algeria	Sidi Khelifa	Count	2000	0	0	0
Algeria	Sidi Khelifa	Count	2017	0	1	0
Algeria	Sayada	Proportion	2000	25.0	38.4	14.8
Algeria	Sayada	Proportion	2017	34.1	49.0	20.4
Algeria	Sayada	Count	2000	0	0	0
Algeria	Sayada	Count	2017	0	0	0
Algeria	Oued Tlelat	Proportion	2000	26.6	40.1	15.3
Algeria	Oued Tlelat	Proportion	2017	35.3	51.5	21.4
Algeria	Oued Tlelat	Count	2000	0	0	0
Algeria	Oued Tlelat	Count	2017	0	1	0
Algeria	Ouled Gacem	Proportion	2000	33.7	49.6	19.8
Algeria	Ouled Gacem	Proportion	2017	36.4	51.6	22.0
Algeria	Ouled Gacem	Count	2000	0	0	0
Algeria	Ouled Gacem	Count	2017	0	1	0
Algeria	Ouarizane	Proportion	2000	27.7	42.1	16.2
Algeria	Ouarizane	Proportion	2017	35.2	51.8	20.9
Algeria	Ouarizane	Count	2000	0	0	0
Algeria	Ouarizane	Count	2017	0	1	0
Algeria	Bir-El-Arch	Proportion	2000	34.0	47.5	20.8
Algeria	Bir-El-Arch	Proportion	2017	37.3	51.9	21.9
Algeria	Bir-El-Arch	Count	2000	0	0	0
Algeria	Bir-El-Arch	Count	2017	0	1	0
Algeria	Merine	Proportion	2000	30.3	46.8	18.2
Algeria	Merine	Proportion	2017	34.8	49.2	21.3
Algeria	Merine	Count	2000	0	0	0
Algeria	Merine	Count	2017	0	0	0
Algeria	Hamadi Krouma	Proportion	2000	32.4	49.4	20.3
Algeria	Hamadi Krouma	Proportion	2017	35.2	51.8	21.0
Algeria	Hamadi Krouma	Count	2000	0	0	0
Algeria	Hamadi Krouma	Count	2017	0	1	0
Algeria	Terraguelt	Proportion	2000	32.8	48.1	20.3
Algeria	Terraguelt	Proportion	2017	36.8	54.0	24.4
Algeria	Terraguelt	Count	2000	0	0	0
Algeria	Terraguelt	Count	2017	0	1	0
Algeria	Ouenza	Proportion	2000	30.7	46.4	18.3
Algeria	Ouenza	Proportion	2017	36.2	52.4	23.3
Algeria	Ouenza	Count	2000	0	0	0
Algeria	Ouenza	Count	2017	0	1	0
Algeria	Nadorah	Proportion	2000	32.0	46.3	19.1
Algeria	Nadorah	Proportion	2017	35.5	50.2	21.9
Algeria	Nadorah	Count	2000	0	0	0
Algeria	Nadorah	Count	2017	0	1	0
Algeria	Sidi Ghiles	Proportion	2000	31.8	44.0	20.1
Algeria	Sidi Ghiles	Proportion	2017	33.9	49.3	20.8

Table 4: ORS by Unit *(continued)*

Country	Unit	Metric	Year	Mean	Upper	Lower
Algeria	Sidi Ghiles	Count	2000	0	0	0
Algeria	Sidi Ghiles	Count	2017	0	0	0
Algeria	Beni-Zikki	Proportion	2000	28.8	43.6	16.5
Algeria	Beni-Zikki	Proportion	2017	33.7	48.7	19.9
Algeria	Beni-Zikki	Count	2000	0	0	0
Algeria	Beni-Zikki	Count	2017	0	0	0
Algeria	Djebala	Proportion	2000	30.0	46.2	18.1
Algeria	Djebala	Proportion	2017	35.6	51.9	20.9
Algeria	Djebala	Count	2000	0	0	0
Algeria	Djebala	Count	2017	0	1	0
Algeria	Timmimoun	Proportion	2000	30.0	44.1	19.7
Algeria	Timmimoun	Proportion	2017	30.7	43.1	19.5
Algeria	Timmimoun	Count	2000	0	0	0
Algeria	Timmimoun	Count	2017	0	0	0
Algeria	Hammam Righa	Proportion	2000	32.0	46.9	19.8
Algeria	Hammam Righa	Proportion	2017	33.1	46.1	19.9
Algeria	Hammam Righa	Count	2000	0	0	0
Algeria	Hammam Righa	Count	2017	0	0	0
Algeria	Sidi Boumediene	Proportion	2000	28.0	40.0	16.3
Algeria	Sidi Boumediene	Proportion	2017	34.3	48.7	20.9
Algeria	Sidi Boumediene	Count	2000	0	0	0
Algeria	Sidi Boumediene	Count	2017	0	0	0
Algeria	Staoueli	Proportion	2000	30.1	44.8	18.7
Algeria	Staoueli	Proportion	2017	31.1	47.3	17.6
Algeria	Staoueli	Count	2000	0	0	0
Algeria	Staoueli	Count	2017	0	0	0
Algeria	Guiga	Proportion	2000	33.4	47.9	21.9
Algeria	Guiga	Proportion	2017	37.0	51.8	22.9
Algeria	Guiga	Count	2000	0	0	0
Algeria	Guiga	Count	2017	0	1	0
Algeria	Ifelain Ilmathen	Proportion	2000	30.1	46.1	18.5
Algeria	Ifelain Ilmathen	Proportion	2017	36.0	50.3	22.9
Algeria	Ifelain Ilmathen	Count	2000	0	0	0
Algeria	Ifelain Ilmathen	Count	2017	0	1	0
Algeria	Mechouneche	Proportion	2000	29.7	45.0	18.4
Algeria	Mechouneche	Proportion	2017	33.7	50.1	20.6
Algeria	Mechouneche	Count	2000	0	0	0
Algeria	Mechouneche	Count	2017	0	1	0
Algeria	Souhan	Proportion	2000	32.1	47.4	20.2
Algeria	Souhan	Proportion	2017	35.5	50.3	22.6
Algeria	Souhan	Count	2000	0	0	0
Algeria	Souhan	Count	2017	0	1	0
Algeria	Ouled Brahem	Proportion	2000	32.7	46.9	20.1
Algeria	Ouled Brahem	Proportion	2017	36.1	52.5	22.3
Algeria	Ouled Brahem	Count	2000	0	0	0
Algeria	Ouled Brahem	Count	2017	0	1	0
Algeria	El Hakimia	Proportion	2000	33.9	50.2	20.8
Algeria	El Hakimia	Proportion	2017	35.8	50.7	22.3
Algeria	El Hakimia	Count	2000	0	1	0

Table 4: ORS by Unit *(continued)*

Country	Unit	Metric	Year	Mean	Upper	Lower
Algeria	El Hakimia	Count	2017	0	1	0
Algeria	Ouled Moussa	Proportion	2000	30.4	47.0	18.0
Algeria	Ouled Moussa	Proportion	2017	34.2	50.1	20.7
Algeria	Ouled Moussa	Count	2000	0	0	0
Algeria	Ouled Moussa	Count	2017	0	1	0
Algeria	Ouled Ben Abdelkader	Proportion	2000	29.2	44.8	16.8
Algeria	Ouled Ben Abdelkader	Proportion	2017	34.5	51.6	20.5
Algeria	Ouled Ben Abdelkader	Count	2000	0	0	0
Algeria	Ouled Ben Abdelkader	Count	2017	0	1	0
Algeria	Hassi El Euch	Proportion	2000	31.3	46.3	16.3
Algeria	Hassi El Euch	Proportion	2017	32.3	46.4	19.6
Algeria	Hassi El Euch	Count	2000	0	0	0
Algeria	Hassi El Euch	Count	2017	0	0	0
Algeria	Sidi Aoun	Proportion	2000	30.5	46.8	17.9
Algeria	Sidi Aoun	Proportion	2017	30.0	45.4	17.4
Algeria	Sidi Aoun	Count	2000	0	0	0
Algeria	Sidi Aoun	Count	2017	0	0	0
Algeria	Zitouna	Proportion	2000	32.9	47.6	20.2
Algeria	Zitouna	Proportion	2017	36.6	52.7	22.5
Algeria	Zitouna	Count	2000	0	0	0
Algeria	Zitouna	Count	2017	0	1	0
Algeria	Khezzara	Proportion	2000	33.3	48.2	20.4
Algeria	Khezzara	Proportion	2017	37.2	52.9	23.8
Algeria	Khezzara	Count	2000	0	0	0
Algeria	Khezzara	Count	2017	0	1	0
Algeria	Sidi Abdelaziz	Proportion	2000	32.6	46.9	21.0
Algeria	Sidi Abdelaziz	Proportion	2017	38.7	54.8	24.5
Algeria	Sidi Abdelaziz	Count	2000	0	0	0
Algeria	Sidi Abdelaziz	Count	2017	0	1	0
Algeria	Taouala	Proportion	2000	30.2	46.5	16.1
Algeria	Taouala	Proportion	2017	32.5	45.9	21.0
Algeria	Taouala	Count	2000	0	0	0
Algeria	Taouala	Count	2017	0	0	0
Algeria	M'Tarfa	Proportion	2000	32.1	47.5	19.2
Algeria	M'Tarfa	Proportion	2017	35.3	50.9	21.8
Algeria	M'Tarfa	Count	2000	0	0	0
Algeria	M'Tarfa	Count	2017	0	1	0
Algeria	Guerdjoum	Proportion	2000	29.3	42.1	17.5
Algeria	Guerdjoum	Proportion	2017	34.9	51.3	21.2
Algeria	Guerdjoum	Count	2000	0	0	0
Algeria	Guerdjoum	Count	2017	0	1	0
Algeria	El Guelb El Kebir	Proportion	2000	33.9	50.6	20.3
Algeria	El Guelb El Kebir	Proportion	2017	36.4	50.6	23.2
Algeria	El Guelb El Kebir	Count	2000	0	1	0
Algeria	El Guelb El Kebir	Count	2017	0	1	0
Algeria	Sidi Merouane	Proportion	2000	32.7	45.7	20.1
Algeria	Sidi Merouane	Proportion	2017	37.2	54.4	23.2
Algeria	Sidi Merouane	Count	2000	0	0	0
Algeria	Sidi Merouane	Count	2017	0	1	0

Table 4: ORS by Unit *(continued)*

Country	Unit	Metric	Year	Mean	Upper	Lower
Algeria	Sidi Ali	Proportion	2000	26.4	42.4	15.9
Algeria	Sidi Ali	Proportion	2017	35.9	51.2	22.1
Algeria	Sidi Ali	Count	2000	0	0	0
Algeria	Sidi Ali	Count	2017	0	1	0
Algeria	Sidi Ben Yebka	Proportion	2000	26.5	38.7	16.2
Algeria	Sidi Ben Yebka	Proportion	2017	35.3	51.0	21.0
Algeria	Sidi Ben Yebka	Count	2000	0	0	0
Algeria	Sidi Ben Yebka	Count	2017	0	1	0
Algeria	Ouled Hamla	Proportion	2000	33.3	47.2	20.5
Algeria	Ouled Hamla	Proportion	2017	37.0	51.9	22.1
Algeria	Ouled Hamla	Count	2000	0	0	0
Algeria	Ouled Hamla	Count	2017	0	1	0
Algeria	Oued El Djemaa	Proportion	2000	27.5	42.7	16.7
Algeria	Oued El Djemaa	Proportion	2017	35.7	52.0	21.2
Algeria	Oued El Djemaa	Count	2000	0	0	0
Algeria	Oued El Djemaa	Count	2017	0	1	0
Algeria	Bir Haddada	Proportion	2000	32.7	46.4	20.5
Algeria	Bir Haddada	Proportion	2017	36.6	52.6	21.4
Algeria	Bir Haddada	Count	2000	0	0	0
Algeria	Bir Haddada	Count	2017	0	1	0
Algeria	Mezaourou	Proportion	2000	30.1	46.1	17.5
Algeria	Mezaourou	Proportion	2017	34.9	50.7	21.5
Algeria	Mezaourou	Count	2000	0	0	0
Algeria	Mezaourou	Count	2017	0	1	0
Algeria	Kanoua	Proportion	2000	32.3	47.1	20.0
Algeria	Kanoua	Proportion	2017	36.1	50.0	21.9
Algeria	Kanoua	Count	2000	0	0	0
Algeria	Kanoua	Count	2017	0	0	0
Algeria	Tiffech	Proportion	2000	33.3	50.1	19.7
Algeria	Tiffech	Proportion	2017	35.3	52.1	23.3
Algeria	Tiffech	Count	2000	0	1	0
Algeria	Tiffech	Count	2017	0	1	0
Algeria	Oum Ali	Proportion	2000	31.4	47.1	17.4
Algeria	Oum Ali	Proportion	2017	37.0	52.2	23.7
Algeria	Oum Ali	Count	2000	0	0	0
Algeria	Oum Ali	Count	2017	0	1	0
Algeria	Naima	Proportion	2000	30.8	44.4	19.6
Algeria	Naima	Proportion	2017	35.4	50.6	22.3
Algeria	Naima	Count	2000	0	0	0
Algeria	Naima	Count	2017	0	1	0
Algeria	Sidi Rached	Proportion	2000	32.2	47.2	19.6
Algeria	Sidi Rached	Proportion	2017	33.5	50.1	19.6
Algeria	Sidi Rached	Count	2000	0	0	0
Algeria	Sidi Rached	Count	2017	0	1	0
Algeria	Beni Aissi	Proportion	2000	28.6	46.9	16.1
Algeria	Beni Aissi	Proportion	2017	34.5	48.3	21.0
Algeria	Beni Aissi	Count	2000	0	0	0
Algeria	Beni Aissi	Count	2017	0	0	0
Algeria	El Aricha	Proportion	2000	32.0	48.3	19.3

Table 4: ORS by Unit *(continued)*

Country	Unit	Metric	Year	Mean	Upper	Lower
Algeria	El Aricha	Proportion	2017	34.1	49.8	20.7
Algeria	El Aricha	Count	2000	0	0	0
Algeria	El Aricha	Count	2017	0	0	0
Algeria	Tinerkouk	Proportion	2000	30.4	43.4	19.8
Algeria	Tinerkouk	Proportion	2017	30.6	41.5	20.3
Algeria	Tinerkouk	Count	2000	0	0	0
Algeria	Tinerkouk	Count	2017	0	0	0
Algeria	Hoceinia	Proportion	2000	32.2	46.2	20.0
Algeria	Hoceinia	Proportion	2017	33.0	46.0	19.3
Algeria	Hoceinia	Count	2000	0	0	0
Algeria	Hoceinia	Count	2017	0	0	0
Algeria	Sidi Ouriache|Tadmaya	Proportion	2000	29.3	45.3	17.2
Algeria	Sidi Ouriache|Tadmaya	Proportion	2017	34.7	49.5	20.0
Algeria	Sidi Ouriache|Tadmaya	Count	2000	0	0	0
Algeria	Sidi Ouriache|Tadmaya	Count	2017	0	0	0
Algeria	Tessala-El-Merdja	Proportion	2000	30.9	46.3	19.6
Algeria	Tessala-El-Merdja	Proportion	2017	34.8	48.9	21.3
Algeria	Tessala-El-Merdja	Count	2000	0	0	0
Algeria	Tessala-El-Merdja	Count	2017	0	0	0
Algeria	Hidoussa	Proportion	2000	32.0	48.1	19.8
Algeria	Hidoussa	Proportion	2017	36.4	50.1	21.9
Algeria	Hidoussa	Count	2000	0	0	0
Algeria	Hidoussa	Count	2017	0	1	0
Algeria	Ighil-Ali	Proportion	2000	32.8	49.8	19.7
Algeria	Ighil-Ali	Proportion	2017	37.7	54.3	23.3
Algeria	Ighil-Ali	Count	2000	0	0	0
Algeria	Ighil-Ali	Count	2017	0	1	0
Algeria	Mekhadma	Proportion	2000	31.1	44.9	18.7
Algeria	Mekhadma	Proportion	2017	31.5	45.3	18.9
Algeria	Mekhadma	Count	2000	0	0	0
Algeria	Mekhadma	Count	2017	0	0	0
Algeria	Soumaa	Proportion	2000	31.1	46.3	19.1
Algeria	Soumaa	Proportion	2017	32.5	49.7	18.8
Algeria	Soumaa	Count	2000	0	0	0
Algeria	Soumaa	Count	2017	0	0	0
Algeria	Ouled Dahmane	Proportion	2000	33.1	47.6	20.4
Algeria	Ouled Dahmane	Proportion	2017	36.8	52.8	22.0
Algeria	Ouled Dahmane	Count	2000	0	0	0
Algeria	Ouled Dahmane	Count	2017	0	1	0
Algeria	El Khabouzia	Proportion	2000	33.2	50.1	19.8
Algeria	El Khabouzia	Proportion	2017	35.7	50.7	22.9
Algeria	El Khabouzia	Count	2000	0	1	0
Algeria	El Khabouzia	Count	2017	0	1	0
Algeria	Si Mustapha	Proportion	2000	30.5	50.6	17.9
Algeria	Si Mustapha	Proportion	2017	35.9	50.6	21.8
Algeria	Si Mustapha	Count	2000	0	1	0
Algeria	Si Mustapha	Count	2017	0	1	0
Algeria	Ouled Fares	Proportion	2000	29.2	44.2	17.1
Algeria	Ouled Fares	Proportion	2017	34.8	52.8	20.3

Table 4: ORS by Unit *(continued)*

Country	Unit	Metric	Year	Mean	Upper	Lower
Algeria	Ouled Fares	Count	2000	0	0	0
Algeria	Ouled Fares	Count	2017	0	1	0
Algeria	Hassi Fedoul	Proportion	2000	30.8	47.1	18.3
Algeria	Hassi Fedoul	Proportion	2017	32.8	46.6	20.2
Algeria	Hassi Fedoul	Count	2000	0	0	0
Algeria	Hassi Fedoul	Count	2017	0	0	0
Algeria	Sidi Khelil	Proportion	2000	31.3	46.8	18.8
Algeria	Sidi Khelil	Proportion	2017	30.9	43.8	18.0
Algeria	Sidi Khelil	Count	2000	0	0	0
Algeria	Sidi Khelil	Count	2017	0	0	0
Algeria	Medjaz Ammar	Proportion	2000	34.9	51.2	21.4
Algeria	Medjaz Ammar	Proportion	2017	38.1	53.7	24.5
Algeria	Medjaz Ammar	Count	2000	0	1	0
Algeria	Medjaz Ammar	Count	2017	0	1	0
Algeria	Sidi Marouf	Proportion	2000	33.2	46.0	20.7
Algeria	Sidi Marouf	Proportion	2017	38.5	54.2	24.6
Algeria	Sidi Marouf	Count	2000	0	0	0
Algeria	Sidi Marouf	Count	2017	0	1	0
Algeria	Maadid	Proportion	2000	32.2	47.3	19.1
Algeria	Maadid	Proportion	2017	35.6	52.5	21.3
Algeria	Maadid	Count	2000	0	0	0
Algeria	Maadid	Count	2017	0	1	0
Algeria	Hacine	Proportion	2000	26.2	39.2	15.6
Algeria	Hacine	Proportion	2017	34.6	50.7	20.3
Algeria	Hacine	Count	2000	0	0	0
Algeria	Hacine	Count	2017	0	1	0
Algeria	El Hamdania	Proportion	2000	33.1	48.9	20.5
Algeria	El Hamdania	Proportion	2017	34.0	49.6	20.0
Algeria	El Hamdania	Count	2000	0	0	0
Algeria	El Hamdania	Count	2017	0	0	0
Algeria	Tadjenanet	Proportion	2000	33.6	46.5	19.5
Algeria	Tadjenanet	Proportion	2017	37.5	52.0	23.0
Algeria	Tadjenanet	Count	2000	0	0	0
Algeria	Tadjenanet	Count	2017	0	1	0
Algeria	Sidi Belattar	Proportion	2000	26.0	41.3	15.0
Algeria	Sidi Belattar	Proportion	2017	35.8	51.1	20.8
Algeria	Sidi Belattar	Count	2000	0	0	0
Algeria	Sidi Belattar	Count	2017	0	1	0
Algeria	Sidi Chahmi	Proportion	2000	26.6	39.2	16.2
Algeria	Sidi Chahmi	Proportion	2017	33.4	48.3	20.1
Algeria	Sidi Chahmi	Count	2000	0	0	0
Algeria	Sidi Chahmi	Count	2017	0	0	0
Algeria	Ouled Zaoui	Proportion	2000	32.8	46.1	19.4
Algeria	Ouled Zaoui	Proportion	2017	37.2	51.9	23.3
Algeria	Ouled Zaoui	Count	2000	0	0	0
Algeria	Ouled Zaoui	Count	2017	0	1	0
Algeria	Oued Essalem	Proportion	2000	31.4	48.9	19.6
Algeria	Oued Essalem	Proportion	2017	35.6	51.8	21.1
Algeria	Oued Essalem	Count	2000	0	0	0

Table 4: ORS by Unit *(continued)*

Country	Unit	Metric	Year	Mean	Upper	Lower
Algeria	Oued Essalem	Count	2017	0	1	0
Algeria	Bouandas	Proportion	2000	31.6	45.8	20.1
Algeria	Bouandas	Proportion	2017	36.8	52.7	23.2
Algeria	Bouandas	Count	2000	0	0	0
Algeria	Bouandas	Count	2017	0	1	0
Algeria	Moulay Slissen	Proportion	2000	30.2	45.3	17.4
Algeria	Moulay Slissen	Proportion	2017	35.2	50.3	22.2
Algeria	Moulay Slissen	Count	2000	0	0	0
Algeria	Moulay Slissen	Count	2017	0	1	0
Algeria	Kerkera	Proportion	2000	31.3	45.7	19.5
Algeria	Kerkera	Proportion	2017	38.1	53.2	23.9
Algeria	Kerkera	Count	2000	0	0	0
Algeria	Kerkera	Count	2017	0	1	0
Algeria	Zaarouria	Proportion	2000	33.2	50.4	20.3
Algeria	Zaarouria	Proportion	2017	35.6	53.3	23.0
Algeria	Zaarouria	Count	2000	0	1	0
Algeria	Zaarouria	Count	2017	0	1	0
Algeria	Safsaf El Ouesra	Proportion	2000	31.3	46.4	17.8
Algeria	Safsaf El Ouesra	Proportion	2017	37.3	53.6	23.4
Algeria	Safsaf El Ouesra	Count	2000	0	0	0
Algeria	Safsaf El Ouesra	Count	2017	0	1	0
Algeria	Oued Lilli	Proportion	2000	32.0	47.3	18.9
Algeria	Oued Lilli	Proportion	2017	35.1	51.4	22.5
Algeria	Oued Lilli	Count	2000	0	0	0
Algeria	Oued Lilli	Count	2017	0	1	0
Algeria	Sidi Semiane	Proportion	2000	31.0	43.2	19.4
Algeria	Sidi Semiane	Proportion	2017	33.1	48.7	19.6
Algeria	Sidi Semiane	Count	2000	0	0	0
Algeria	Sidi Semiane	Count	2017	0	0	0
Algeria	Beni Yenni	Proportion	2000	28.9	46.6	17.3
Algeria	Beni Yenni	Proportion	2017	35.5	49.1	21.5
Algeria	Beni Yenni	Count	2000	0	0	0
Algeria	Beni Yenni	Count	2017	0	0	0
Algeria	El Fehoul	Proportion	2000	29.0	43.8	17.5
Algeria	El Fehoul	Proportion	2017	34.3	49.6	20.9
Algeria	El Fehoul	Count	2000	0	0	0
Algeria	El Fehoul	Count	2017	0	0	0
Algeria	Tit	Proportion	2000	28.6	43.5	19.0
Algeria	Tit	Proportion	2017	30.8	46.1	17.7
Algeria	Tit	Count	2000	0	0	0
Algeria	Tit	Count	2017	0	0	0
Algeria	Khemis Miliana	Proportion	2000	30.8	44.1	19.1
Algeria	Khemis Miliana	Proportion	2017	32.1	47.3	17.8
Algeria	Khemis Miliana	Count	2000	0	0	0
Algeria	Khemis Miliana	Count	2017	0	0	0
Algeria	Sidi Safi	Proportion	2000	28.3	41.9	17.0
Algeria	Sidi Safi	Proportion	2017	34.2	48.6	20.4
Algeria	Sidi Safi	Count	2000	0	0	0
Algeria	Sidi Safi	Count	2017	0	0	0

Table 4: ORS by Unit *(continued)*

Country	Unit	Metric	Year	Mean	Upper	Lower
Algeria	Zeralda	Proportion	2000	30.2	44.8	18.7
Algeria	Zeralda	Proportion	2017	33.4	48.9	20.1
Algeria	Zeralda	Count	2000	0	0	0
Algeria	Zeralda	Count	2017	0	0	0
Algeria	Ichmoul	Proportion	2000	30.6	45.7	18.1
Algeria	Ichmoul	Proportion	2017	35.2	51.1	21.9
Algeria	Ichmoul	Count	2000	0	0	0
Algeria	Ichmoul	Count	2017	0	1	0
Algeria	Ighrem	Proportion	2000	30.5	46.9	18.0
Algeria	Ighrem	Proportion	2017	37.1	52.5	23.0
Algeria	Ighrem	Count	2000	0	0	0
Algeria	Ighrem	Count	2017	0	1	0
Algeria	Ouled Djellal	Proportion	2000	30.4	45.0	17.7
Algeria	Ouled Djellal	Proportion	2017	30.2	45.6	17.9
Algeria	Ouled Djellal	Count	2000	0	0	0
Algeria	Ouled Djellal	Count	2017	0	0	0
Algeria	Ouled Sidi Brahim	Proportion	2000	33.2	48.9	19.4
Algeria	Ouled Sidi Brahim	Proportion	2017	36.7	52.5	23.0
Algeria	Ouled Sidi Brahim	Count	2000	0	0	0
Algeria	Ouled Sidi Brahim	Count	2017	0	1	0
Algeria	El Mokrani|El Madjen	Proportion	2000	32.1	49.6	19.0
Algeria	El Mokrani|El Madjen	Proportion	2017	35.5	50.0	21.8
Algeria	El Mokrani|El Madjen	Count	2000	0	0	0
Algeria	El Mokrani|El Madjen	Count	2017	0	0	0
Algeria	Sidi Daoud	Proportion	2000	29.1	47.3	17.1
Algeria	Sidi Daoud	Proportion	2017	35.3	49.2	21.0
Algeria	Sidi Daoud	Count	2000	0	0	0
Algeria	Sidi Daoud	Count	2017	0	0	0
Algeria	Oum Drou	Proportion	2000	29.8	46.2	17.4
Algeria	Oum Drou	Proportion	2017	34.7	52.0	19.7
Algeria	Oum Drou	Count	2000	0	0	0
Algeria	Oum Drou	Count	2017	0	1	0
Algeria	M'Liliha	Proportion	2000	31.0	45.5	18.4
Algeria	M'Liliha	Proportion	2017	32.0	45.2	19.3
Algeria	M'Liliha	Count	2000	0	0	0
Algeria	M'Liliha	Count	2017	0	0	0
Algeria	Still	Proportion	2000	31.0	46.0	19.4
Algeria	Still	Proportion	2017	31.3	45.1	19.0
Algeria	Still	Count	2000	0	0	0
Algeria	Still	Count	2017	0	0	0
Algeria	Medjaz Sfa	Proportion	2000	33.7	49.0	20.7
Algeria	Medjaz Sfa	Proportion	2017	37.5	54.9	24.3
Algeria	Medjaz Sfa	Count	2000	0	0	0
Algeria	Medjaz Sfa	Count	2017	0	1	0
Algeria	Taher	Proportion	2000	31.2	46.4	18.9
Algeria	Taher	Proportion	2017	36.6	53.1	23.1
Algeria	Taher	Count	2000	0	0	0
Algeria	Taher	Count	2017	0	1	0
Algeria	Maarif	Proportion	2000	31.6	47.5	17.7

Table 4: ORS by Unit *(continued)*

Country	Unit	Metric	Year	Mean	Upper	Lower
Algeria	Maarif	Proportion	2017	33.8	48.8	21.4
Algeria	Maarif	Count	2000	0	0	0
Algeria	Maarif	Count	2017	0	0	0
Algeria	Khalouia	Proportion	2000	27.5	42.7	16.7
Algeria	Khalouia	Proportion	2017	35.0	50.5	21.1
Algeria	Khalouia	Count	2000	0	0	0
Algeria	Khalouia	Count	2017	0	1	0
Algeria	El Kaf Lakhdar	Proportion	2000	34.6	53.4	20.2
Algeria	El Kaf Lakhdar	Proportion	2017	35.8	50.5	23.2
Algeria	El Kaf Lakhdar	Count	2000	0	1	0
Algeria	El Kaf Lakhdar	Count	2017	0	1	0
Algeria	Tassadane Haddada	Proportion	2000	32.7	45.5	20.7
Algeria	Tassadane Haddada	Proportion	2017	37.8	53.8	23.1
Algeria	Tassadane Haddada	Count	2000	0	0	0
Algeria	Tassadane Haddada	Count	2017	0	1	0
Algeria	Sidi Lakhdar	Proportion	2000	26.0	42.5	14.9
Algeria	Sidi Lakhdar	Proportion	2017	35.7	50.3	22.4
Algeria	Sidi Lakhdar	Count	2000	0	0	0
Algeria	Sidi Lakhdar	Count	2017	0	1	0
Algeria	Tafraoui	Proportion	2000	26.9	39.3	16.3
Algeria	Tafraoui	Proportion	2017	34.6	49.4	20.7
Algeria	Tafraoui	Count	2000	0	0	0
Algeria	Tafraoui	Count	2017	0	0	0
Algeria	Oum El Bouaghi	Proportion	2000	31.2	46.7	18.6
Algeria	Oum El Bouaghi	Proportion	2017	36.2	51.7	21.1
Algeria	Oum El Bouaghi	Count	2000	0	0	0
Algeria	Oum El Bouaghi	Count	2017	0	1	0
Algeria	Oued Rhiou	Proportion	2000	28.3	43.5	16.3
Algeria	Oued Rhiou	Proportion	2017	36.0	53.3	21.6
Algeria	Oued Rhiou	Count	2000	0	0	0
Algeria	Oued Rhiou	Count	2017	0	1	0
Algeria	Bougaa	Proportion	2000	32.6	46.5	20.1
Algeria	Bougaa	Proportion	2017	37.8	53.8	23.0
Algeria	Bougaa	Count	2000	0	0	0
Algeria	Bougaa	Count	2017	0	1	0
Algeria	Mustafa Ben Brahim	Proportion	2000	27.7	42.0	15.3
Algeria	Mustafa Ben Brahim	Proportion	2017	34.8	49.6	20.5
Algeria	Mustafa Ben Brahim	Count	2000	0	0	0
Algeria	Mustafa Ben Brahim	Count	2017	0	0	0
Algeria	Kheng Maoun	Proportion	2000	32.4	47.3	20.3
Algeria	Kheng Maoun	Proportion	2017	38.5	52.9	24.1
Algeria	Kheng Maoun	Count	2000	0	0	0
Algeria	Kheng Maoun	Count	2017	0	1	0
Algeria	Zouabi	Proportion	2000	33.2	49.3	21.3
Algeria	Zouabi	Proportion	2017	36.3	52.1	23.8
Algeria	Zouabi	Count	2000	0	0	0
Algeria	Zouabi	Count	2017	0	1	0
Algeria	Stah Guentis	Proportion	2000	30.7	45.8	17.7
Algeria	Stah Guentis	Proportion	2017	35.9	51.1	21.2

Table 4: ORS by Unit *(continued)*

Country	Unit	Metric	Year	Mean	Upper	Lower
Algeria	Stah Guentis	Count	2000	0	0	0
Algeria	Stah Guentis	Count	2017	0	1	0
Algeria	Rahouia	Proportion	2000	30.6	48.1	18.0
Algeria	Rahouia	Proportion	2017	34.2	51.6	19.1
Algeria	Rahouia	Count	2000	0	0	0
Algeria	Rahouia	Count	2017	0	1	0
Algeria	Tipaza	Proportion	2000	32.0	46.8	20.2
Algeria	Tipaza	Proportion	2017	33.6	48.9	20.6
Algeria	Tipaza	Count	2000	0	0	0
Algeria	Tipaza	Count	2017	0	0	0
Algeria	Beni Zmenzer	Proportion	2000	28.5	46.2	16.3
Algeria	Beni Zmenzer	Proportion	2017	34.4	47.7	20.6
Algeria	Beni Zmenzer	Count	2000	0	0	0
Algeria	Beni Zmenzer	Count	2017	0	0	0
Algeria	El Gor	Proportion	2000	30.9	46.4	18.0
Algeria	El Gor	Proportion	2017	34.2	49.1	20.3
Algeria	El Gor	Count	2000	0	0	0
Algeria	El Gor	Count	2017	0	0	0
Algeria	Tsabit	Proportion	2000	29.2	41.9	18.8
Algeria	Tsabit	Proportion	2017	30.6	42.3	20.2
Algeria	Tsabit	Count	2000	0	0	0
Algeria	Tsabit	Count	2017	0	0	0
Algeria	Mekhatria	Proportion	2000	31.0	45.8	19.5
Algeria	Mekhatria	Proportion	2017	33.5	48.9	19.3
Algeria	Mekhatria	Count	2000	0	0	0
Algeria	Mekhatria	Count	2017	0	0	0
Algeria	Tamzoura	Proportion	2000	27.3	40.2	16.2
Algeria	Tamzoura	Proportion	2017	34.3	49.0	19.9
Algeria	Tamzoura	Count	2000	0	0	0
Algeria	Tamzoura	Count	2017	0	0	0
Algeria	Inoughissen	Proportion	2000	30.3	45.5	18.0
Algeria	Inoughissen	Proportion	2017	34.3	50.0	21.1
Algeria	Inoughissen	Count	2000	0	0	0
Algeria	Inoughissen	Count	2017	0	0	0
Algeria	Kendira	Proportion	2000	31.7	45.5	20.1
Algeria	Kendira	Proportion	2017	36.4	51.7	23.3
Algeria	Kendira	Count	2000	0	0	0
Algeria	Kendira	Count	2017	0	1	0
Algeria	Oumache	Proportion	2000	30.6	44.5	18.9
Algeria	Oumache	Proportion	2017	31.5	46.3	19.3
Algeria	Oumache	Count	2000	0	0	0
Algeria	Oumache	Count	2017	0	0	0
Algeria	Rabta	Proportion	2000	33.0	48.0	20.1
Algeria	Rabta	Proportion	2017	36.8	52.9	21.7
Algeria	Rabta	Count	2000	0	0	0
Algeria	Rabta	Count	2017	0	1	0
Algeria	Guerrouma	Proportion	2000	32.1	48.8	19.1
Algeria	Guerrouma	Proportion	2017	34.7	48.8	21.2
Algeria	Guerrouma	Count	2000	0	0	0

Table 4: ORS by Unit *(continued)*

Country	Unit	Metric	Year	Mean	Upper	Lower
Algeria	Guerrouma	Count	2017	0	0	0
Algeria	Souk El Had	Proportion	2000	30.5	49.6	18.2
Algeria	Souk El Had	Proportion	2017	35.8	50.7	22.0
Algeria	Souk El Had	Count	2000	0	0	0
Algeria	Souk El Had	Count	2017	0	1	0
Algeria	Sendjas	Proportion	2000	29.8	44.8	17.3
Algeria	Sendjas	Proportion	2017	34.4	51.2	20.2
Algeria	Sendjas	Count	2000	0	0	0
Algeria	Sendjas	Count	2017	0	1	0
Algeria	Messaad	Proportion	2000	30.6	46.9	17.5
Algeria	Messaad	Proportion	2017	33.7	51.5	21.0
Algeria	Messaad	Count	2000	0	0	0
Algeria	Messaad	Count	2017	0	1	0
Algeria	Taghzout	Proportion	2000	30.2	47.0	17.8
Algeria	Taghzout	Proportion	2017	30.2	45.3	18.3
Algeria	Taghzout	Count	2000	0	0	0
Algeria	Taghzout	Count	2017	0	0	0
Algeria	Nechemaya	Proportion	2000	34.4	50.3	21.7
Algeria	Nechemaya	Proportion	2017	37.5	53.9	24.2
Algeria	Nechemaya	Count	2000	0	1	0
Algeria	Nechemaya	Count	2017	0	1	0
Algeria	Texenna	Proportion	2000	31.8	45.0	19.2
Algeria	Texenna	Proportion	2017	38.3	55.4	24.7
Algeria	Texenna	Count	2000	0	0	0
Algeria	Texenna	Count	2017	0	1	0
Algeria	Magra	Proportion	2000	31.0	45.7	18.6
Algeria	Magra	Proportion	2017	35.3	51.4	21.5
Algeria	Magra	Count	2000	0	0	0
Algeria	Magra	Count	2017	0	1	0
Algeria	Magtaa Douz	Proportion	2000	25.0	38.1	14.0
Algeria	Magtaa Douz	Proportion	2017	34.5	50.0	19.8
Algeria	Magtaa Douz	Count	2000	0	0	0
Algeria	Magtaa Douz	Count	2017	0	0	0
Algeria	El Ouinet	Proportion	2000	33.9	51.2	20.4
Algeria	El Ouinet	Proportion	2017	35.5	49.6	23.4
Algeria	El Ouinet	Count	2000	0	1	0
Algeria	El Ouinet	Count	2017	0	0	0
Algeria	Teleghma	Proportion	2000	33.3	46.3	20.9
Algeria	Teleghma	Proportion	2017	37.6	52.6	23.1
Algeria	Teleghma	Count	2000	0	0	0
Algeria	Teleghma	Count	2017	0	1	0
Algeria	Sirat	Proportion	2000	25.0	38.3	14.8
Algeria	Sirat	Proportion	2017	34.5	50.4	19.7
Algeria	Sirat	Count	2000	0	0	0
Algeria	Sirat	Count	2017	0	1	0
Algeria	Sigous	Proportion	2000	33.6	49.5	20.0
Algeria	Sigous	Proportion	2017	36.6	51.5	22.7
Algeria	Sigous	Count	2000	0	0	0
Algeria	Sigous	Count	2017	0	1	0

Table 4: ORS by Unit *(continued)*

Country	Unit	Metric	Year	Mean	Upper	Lower
Algeria	Ouled Sidi Mihoub	Proportion	2000	26.9	41.8	16.1
Algeria	Ouled Sidi Mihoub	Proportion	2017	35.6	51.0	20.7
Algeria	Ouled Sidi Mihoub	Count	2000	0	0	0
Algeria	Ouled Sidi Mihoub	Count	2017	0	1	0
Algeria	Bousselam	Proportion	2000	32.2	46.7	20.3
Algeria	Bousselam	Proportion	2017	36.8	51.7	23.2
Algeria	Bousselam	Count	2000	0	0	0
Algeria	Bousselam	Count	2017	0	1	0
Algeria	Oued Sebaa	Proportion	2000	31.5	48.0	17.9
Algeria	Oued Sebaa	Proportion	2017	34.7	49.7	20.7
Algeria	Oued Sebaa	Count	2000	0	0	0
Algeria	Oued Sebaa	Count	2017	0	0	0
Algeria	Oued Zhour	Proportion	2000	32.6	46.8	20.4
Algeria	Oued Zhour	Proportion	2017	38.3	53.5	23.5
Algeria	Oued Zhour	Count	2000	0	0	0
Algeria	Oued Zhour	Count	2017	0	1	0
Algeria	Tebessa	Proportion	2000	30.1	45.5	16.7
Algeria	Tebessa	Proportion	2017	35.1	49.6	21.3
Algeria	Tebessa	Count	2000	0	0	0
Algeria	Tebessa	Count	2017	0	0	0
Algeria	Rechaiga	Proportion	2000	32.0	46.8	19.2
Algeria	Rechaiga	Proportion	2017	35.2	49.4	21.5
Algeria	Rechaiga	Count	2000	0	0	0
Algeria	Rechaiga	Count	2017	0	0	0
Algeria	Boghni	Proportion	2000	29.4	46.5	16.4
Algeria	Boghni	Proportion	2017	35.5	49.8	21.4
Algeria	Boghni	Count	2000	0	0	0
Algeria	Boghni	Count	2017	0	0	0
Algeria	Fellaoucene	Proportion	2000	29.4	45.7	19.2
Algeria	Fellaoucene	Proportion	2017	35.1	51.3	20.8
Algeria	Fellaoucene	Count	2000	0	0	0
Algeria	Fellaoucene	Count	2017	0	1	0
Algeria	Zaouiet Kounta	Proportion	2000	28.3	40.9	16.6
Algeria	Zaouiet Kounta	Proportion	2017	30.9	44.4	20.6
Algeria	Zaouiet Kounta	Count	2000	0	0	0
Algeria	Zaouiet Kounta	Count	2017	0	0	0
Algeria	Miliana	Proportion	2000	30.9	44.4	19.2
Algeria	Miliana	Proportion	2017	32.9	47.8	18.7
Algeria	Miliana	Count	2000	0	0	0
Algeria	Miliana	Count	2017	0	0	0
Algeria	Terga	Proportion	2000	28.3	42.4	16.9
Algeria	Terga	Proportion	2017	34.3	48.7	20.4
Algeria	Terga	Count	2000	0	0	0
Algeria	Terga	Count	2017	0	0	0
Algeria	Kimmel	Proportion	2000	30.8	45.1	19.1
Algeria	Kimmel	Proportion	2017	35.4	50.7	21.8
Algeria	Kimmel	Count	2000	0	0	0
Algeria	Kimmel	Count	2017	0	1	0
Algeria	Kherrata	Proportion	2000	31.7	45.0	18.9

Table 4: ORS by Unit *(continued)*

Country	Unit	Metric	Year	Mean	Upper	Lower
Algeria	Kherrata	Proportion	2017	37.9	54.5	23.7
Algeria	Kherrata	Count	2000	0	0	0
Algeria	Kherrata	Count	2017	0	1	0
Algeria	Ourlal	Proportion	2000	30.7	44.1	18.2
Algeria	Ourlal	Proportion	2017	31.3	45.1	18.3
Algeria	Ourlal	Count	2000	0	0	0
Algeria	Ourlal	Count	2017	0	0	0
Algeria	Ras El Oued	Proportion	2000	33.3	48.0	20.6
Algeria	Ras El Oued	Proportion	2017	36.5	52.9	22.4
Algeria	Ras El Oued	Count	2000	0	0	0
Algeria	Ras El Oued	Count	2017	0	1	0
Algeria	Hadjera Zerga	Proportion	2000	33.7	50.4	19.3
Algeria	Hadjera Zerga	Proportion	2017	35.5	51.7	21.8
Algeria	Hadjera Zerga	Count	2000	0	1	0
Algeria	Hadjera Zerga	Count	2017	0	1	0
Algeria	Taourga	Proportion	2000	28.7	45.7	16.6
Algeria	Taourga	Proportion	2017	34.8	49.3	20.7
Algeria	Taourga	Count	2000	0	0	0
Algeria	Taourga	Count	2017	0	0	0
Algeria	Sidi Abderrahmane	Proportion	2000	28.8	43.2	17.4
Algeria	Sidi Abderrahmane	Proportion	2017	34.2	50.8	19.9
Algeria	Sidi Abderrahmane	Count	2000	0	0	0
Algeria	Sidi Abderrahmane	Count	2017	0	1	0
Algeria	Moudjebara	Proportion	2000	30.9	45.3	17.8
Algeria	Moudjebara	Proportion	2017	31.9	46.1	19.6
Algeria	Moudjebara	Count	2000	0	0	0
Algeria	Moudjebara	Count	2017	0	0	0
Algeria	Taleb Larbi	Proportion	2000	30.1	44.9	17.9
Algeria	Taleb Larbi	Proportion	2017	32.0	47.2	19.2
Algeria	Taleb Larbi	Count	2000	0	0	0
Algeria	Taleb Larbi	Count	2017	0	0	0
Algeria	Oued Cheham	Proportion	2000	33.8	50.0	21.1
Algeria	Oued Cheham	Proportion	2017	37.0	53.6	23.9
Algeria	Oued Cheham	Count	2000	0	0	0
Algeria	Oued Cheham	Count	2017	0	1	0
Algeria	Ziama Mansouria	Proportion	2000	31.6	46.3	17.1
Algeria	Ziama Mansouria	Proportion	2017	37.9	55.0	24.6
Algeria	Ziama Mansouria	Count	2000	0	0	0
Algeria	Ziama Mansouria	Count	2017	0	1	0
Algeria	Medjedel	Proportion	2000	32.0	46.7	18.4
Algeria	Medjedel	Proportion	2017	34.5	49.0	21.5
Algeria	Medjedel	Count	2000	0	0	0
Algeria	Medjedel	Count	2017	0	0	0
Algeria	Makhda	Proportion	2000	29.7	45.5	18.1
Algeria	Makhda	Proportion	2017	35.1	51.7	21.4
Algeria	Makhda	Count	2000	0	0	0
Algeria	Makhda	Count	2017	0	1	0
Algeria	El Oumaria	Proportion	2000	32.9	49.6	20.9
Algeria	El Oumaria	Proportion	2017	34.5	49.1	21.1

Table 4: ORS by Unit *(continued)*

Country	Unit	Metric	Year	Mean	Upper	Lower
Algeria	El Oumaria	Count	2000	0	0	0
Algeria	El Oumaria	Count	2017	0	0	0
Algeria	Terrai Bainnane	Proportion	2000	32.9	45.2	20.2
Algeria	Terrai Bainnane	Proportion	2017	36.9	53.1	22.8
Algeria	Terrai Bainnane	Count	2000	0	0	0
Algeria	Terrai Bainnane	Count	2017	0	1	0
Algeria	Souaflia	Proportion	2000	25.7	39.2	15.4
Algeria	Souaflia	Proportion	2017	34.7	50.1	19.5
Algeria	Souaflia	Count	2000	0	0	0
Algeria	Souaflia	Count	2017	0	1	0
Algeria	Souk Naamane	Proportion	2000	33.5	45.9	19.3
Algeria	Souk Naamane	Proportion	2017	36.5	50.9	23.1
Algeria	Souk Naamane	Count	2000	0	0	0
Algeria	Souk Naamane	Count	2017	0	1	0
Algeria	Ouled Yaich	Proportion	2000	29.1	45.8	17.0
Algeria	Ouled Yaich	Proportion	2017	35.2	51.8	21.1
Algeria	Ouled Yaich	Count	2000	0	0	0
Algeria	Ouled Yaich	Count	2017	0	1	0
Algeria	Boutaleb	Proportion	2000	32.9	46.5	21.1
Algeria	Boutaleb	Proportion	2017	36.4	52.6	23.1
Algeria	Boutaleb	Count	2000	0	0	0
Algeria	Boutaleb	Count	2017	0	1	0
Algeria	Oued Sefioune	Proportion	2000	28.4	44.3	16.3
Algeria	Oued Sefioune	Proportion	2017	34.8	51.5	21.4
Algeria	Oued Sefioune	Count	2000	0	0	0
Algeria	Oued Sefioune	Count	2017	0	1	0
Algeria	Ouldja Boulbalout	Proportion	2000	33.5	47.4	21.0
Algeria	Ouldja Boulbalout	Proportion	2017	38.6	54.0	23.5
Algeria	Ouldja Boulbalout	Count	2000	0	0	0
Algeria	Ouldja Boulbalout	Count	2017	0	1	0
Algeria	Thelidjene	Proportion	2000	30.3	43.7	17.8
Algeria	Thelidjene	Proportion	2017	35.8	51.3	22.1
Algeria	Thelidjene	Count	2000	0	0	0
Algeria	Thelidjene	Count	2017	0	1	0
Algeria	Sebaine	Proportion	2000	31.9	46.3	19.1
Algeria	Sebaine	Proportion	2017	34.4	50.2	21.0
Algeria	Sebaine	Count	2000	0	0	0
Algeria	Sebaine	Count	2017	0	1	0
Algeria	Boudjima	Proportion	2000	28.7	44.6	16.7
Algeria	Boudjima	Proportion	2017	34.8	48.7	20.2
Algeria	Boudjima	Count	2000	0	0	0
Algeria	Boudjima	Count	2017	0	0	0
Algeria	Ghazaouet	Proportion	2000	30.0	47.3	18.0
Algeria	Ghazaouet	Proportion	2017	35.7	52.5	21.6
Algeria	Ghazaouet	Count	2000	0	0	0
Algeria	Ghazaouet	Count	2017	0	1	0
Algeria	Oued Chorfa	Proportion	2000	33.0	48.9	21.8
Algeria	Oued Chorfa	Proportion	2017	34.4	48.4	21.1
Algeria	Oued Chorfa	Count	2000	0	0	0

Table 4: ORS by Unit *(continued)*

Country	Unit	Metric	Year	Mean	Upper	Lower
Algeria	Oued Chorfa	Count	2017	0	0	0
Algeria	Ksar Bellezma	Proportion	2000	33.5	50.3	20.7
Algeria	Ksar Bellezma	Proportion	2017	36.4	50.3	22.1
Algeria	Ksar Bellezma	Count	2000	0	1	0
Algeria	Ksar Bellezma	Count	2017	0	1	0
Algeria	Melbou	Proportion	2000	30.0	45.1	16.3
Algeria	Melbou	Proportion	2017	37.5	55.2	24.1
Algeria	Melbou	Count	2000	0	0	0
Algeria	Melbou	Count	2017	0	1	0
Algeria	Ras Miaad|Ouled Sassi	Proportion	2000	30.0	43.1	19.0
Algeria	Ras Miaad|Ouled Sassi	Proportion	2017	31.0	42.8	18.8
Algeria	Ras Miaad|Ouled Sassi	Count	2000	0	0	0
Algeria	Ras Miaad|Ouled Sassi	Count	2017	0	0	0
Algeria	Sidi Embarek	Proportion	2000	33.4	48.4	20.7
Algeria	Sidi Embarek	Proportion	2017	37.4	54.3	22.0
Algeria	Sidi Embarek	Count	2000	0	0	0
Algeria	Sidi Embarek	Count	2017	0	1	0
Algeria	Haizer	Proportion	2000	31.5	47.5	18.1
Algeria	Haizer	Proportion	2017	36.3	51.2	22.5
Algeria	Haizer	Count	2000	0	0	0
Algeria	Haizer	Count	2017	0	1	0
Algeria	Thenia	Proportion	2000	30.4	48.8	18.1
Algeria	Thenia	Proportion	2017	35.5	51.2	22.0
Algeria	Thenia	Count	2000	0	0	0
Algeria	Thenia	Count	2017	0	1	0
Algeria	Sidi Akkacha	Proportion	2000	29.3	43.7	17.8
Algeria	Sidi Akkacha	Proportion	2017	33.6	49.8	19.5
Algeria	Sidi Akkacha	Count	2000	0	0	0
Algeria	Sidi Akkacha	Count	2017	0	0	0
Algeria	Oum Laadham	Proportion	2000	30.0	44.1	17.9
Algeria	Oum Laadham	Proportion	2017	30.2	42.9	19.8
Algeria	Oum Laadham	Count	2000	0	0	0
Algeria	Oum Laadham	Count	2017	0	0	0
Algeria	Tenedla	Proportion	2000	30.4	46.5	17.1
Algeria	Tenedla	Proportion	2017	32.0	46.1	19.1
Algeria	Tenedla	Count	2000	0	0	0
Algeria	Tenedla	Count	2017	0	0	0
Algeria	Oued Fragha	Proportion	2000	33.9	50.2	21.7
Algeria	Oued Fragha	Proportion	2017	36.8	55.7	22.5
Algeria	Oued Fragha	Count	2000	0	1	0
Algeria	Oued Fragha	Count	2017	0	1	0
Algeria	Menaa	Proportion	2000	31.7	46.7	18.5
Algeria	Menaa	Proportion	2017	34.7	49.3	21.7
Algeria	Menaa	Count	2000	0	0	0
Algeria	Menaa	Count	2017	0	0	0
Algeria	Maoussa	Proportion	2000	27.8	42.3	16.9
Algeria	Maoussa	Proportion	2017	34.9	51.0	21.6
Algeria	Maoussa	Count	2000	0	0	0
Algeria	Maoussa	Count	2017	0	1	0

Table 4: ORS by Unit *(continued)*

Country	Unit	Metric	Year	Mean	Upper	Lower
Algeria	Hannacha	Proportion	2000	33.3	49.2	21.7
Algeria	Hannacha	Proportion	2017	34.5	48.5	21.3
Algeria	Hannacha	Count	2000	0	0	0
Algeria	Hannacha	Count	2017	0	0	0
Algeria	Tessala Lamtai	Proportion	2000	32.6	45.0	20.5
Algeria	Tessala Lamtai	Proportion	2017	36.7	52.7	22.7
Algeria	Tessala Lamtai	Count	2000	0	0	0
Algeria	Tessala Lamtai	Count	2017	0	1	0
Algeria	Sour	Proportion	2000	26.0	41.1	14.8
Algeria	Sour	Proportion	2017	35.7	51.6	20.5
Algeria	Sour	Count	2000	0	0	0
Algeria	Sour	Count	2017	0	1	0
Algeria	Zorg	Proportion	2000	32.2	47.2	20.0
Algeria	Zorg	Proportion	2017	36.0	50.9	22.7
Algeria	Zorg	Count	2000	0	0	0
Algeria	Zorg	Count	2017	0	1	0
Algeria	Ramka	Proportion	2000	30.3	45.5	17.6
Algeria	Ramka	Proportion	2017	35.1	52.2	22.1
Algeria	Ramka	Count	2000	0	0	0
Algeria	Ramka	Count	2017	0	1	0
Algeria	Dehamcha	Proportion	2000	33.2	46.9	19.4
Algeria	Dehamcha	Proportion	2017	37.1	54.2	22.3
Algeria	Dehamcha	Count	2000	0	0	0
Algeria	Dehamcha	Count	2017	0	1	0
Algeria	Oued Taourira	Proportion	2000	30.8	46.8	19.2
Algeria	Oued Taourira	Proportion	2017	34.6	48.9	21.5
Algeria	Oued Taourira	Count	2000	0	0	0
Algeria	Oued Taourira	Count	2017	0	0	0
Algeria	Ouled Attia	Proportion	2000	31.8	46.3	19.8
Algeria	Ouled Attia	Proportion	2017	36.7	51.0	22.3
Algeria	Ouled Attia	Count	2000	0	0	0
Algeria	Ouled Attia	Count	2017	0	1	0
Algeria	Sebt	Proportion	2000	31.0	45.5	18.0
Algeria	Sebt	Proportion	2017	34.4	50.2	20.2
Algeria	Sebt	Count	2000	0	0	0
Algeria	Sebt	Count	2017	0	1	0
Algeria	Bounouh	Proportion	2000	29.2	45.7	16.3
Algeria	Bounouh	Proportion	2017	35.1	49.1	21.7
Algeria	Bounouh	Count	2000	0	0	0
Algeria	Bounouh	Count	2017	0	0	0
Algeria	Hammam Boughrara	Proportion	2000	30.3	46.1	18.4
Algeria	Hammam Boughrara	Proportion	2017	36.1	52.5	20.7
Algeria	Hammam Boughrara	Count	2000	0	0	0
Algeria	Hammam Boughrara	Count	2017	0	1	0
Algeria	Oued Djemaa	Proportion	2000	32.6	49.0	20.9
Algeria	Oued Djemaa	Proportion	2017	34.0	47.5	21.1
Algeria	Oued Djemaa	Count	2000	0	0	0
Algeria	Oued Djemaa	Count	2017	0	0	0
Algeria	Larbaa	Proportion	2000	31.3	46.0	19.7

Table 4: ORS by Unit *(continued)*

Country	Unit	Metric	Year	Mean	Upper	Lower
Algeria	Larbaa	Proportion	2017	34.5	49.7	20.2
Algeria	Larbaa	Count	2000	0	0	0
Algeria	Larbaa	Count	2017	0	0	0
Algeria	Oued Ghir	Proportion	2000	29.4	45.5	17.5
Algeria	Oued Ghir	Proportion	2017	34.8	50.1	21.8
Algeria	Oued Ghir	Count	2000	0	0	0
Algeria	Oued Ghir	Count	2017	0	1	0
Algeria	Sidi Khaled	Proportion	2000	30.3	45.3	17.8
Algeria	Sidi Khaled	Proportion	2017	32.0	47.6	19.5
Algeria	Sidi Khaled	Count	2000	0	0	0
Algeria	Sidi Khaled	Count	2017	0	0	0
Algeria	Tafreg	Proportion	2000	33.2	48.8	20.5
Algeria	Tafreg	Proportion	2017	36.9	52.2	22.8
Algeria	Tafreg	Count	2000	0	0	0
Algeria	Tafreg	Count	2017	0	1	0
Algeria	Kadiria	Proportion	2000	30.7	48.7	18.1
Algeria	Kadiria	Proportion	2017	35.5	49.5	21.9
Algeria	Kadiria	Count	2000	0	0	0
Algeria	Kadiria	Count	2017	0	0	0
Algeria	Tidjelabine	Proportion	2000	29.9	47.2	17.7
Algeria	Tidjelabine	Proportion	2017	35.6	51.0	21.8
Algeria	Tidjelabine	Count	2000	0	0	0
Algeria	Tidjelabine	Count	2017	0	1	0
Algeria	Sobha	Proportion	2000	28.5	42.8	16.3
Algeria	Sobha	Proportion	2017	34.8	52.1	19.9
Algeria	Sobha	Count	2000	0	0	0
Algeria	Sobha	Count	2017	0	1	0
Algeria	Sed Rahal	Proportion	2000	30.2	44.7	18.5
Algeria	Sed Rahal	Proportion	2017	31.4	46.2	19.9
Algeria	Sed Rahal	Count	2000	0	0	0
Algeria	Sed Rahal	Count	2017	0	0	0
Algeria	Trifaoui	Proportion	2000	30.4	46.2	16.8
Algeria	Trifaoui	Proportion	2017	31.1	47.8	18.2
Algeria	Trifaoui	Count	2000	0	0	0
Algeria	Trifaoui	Count	2017	0	0	0
Algeria	Oued Zenati	Proportion	2000	34.8	51.0	21.9
Algeria	Oued Zenati	Proportion	2017	35.6	50.8	22.5
Algeria	Oued Zenati	Count	2000	0	1	0
Algeria	Oued Zenati	Count	2017	0	1	0
Algeria	Mohamed Boudiaf	Proportion	2000	30.7	44.9	19.0
Algeria	Mohamed Boudiaf	Proportion	2017	33.4	49.9	19.9
Algeria	Mohamed Boudiaf	Count	2000	0	0	0
Algeria	Mohamed Boudiaf	Count	2017	0	0	0
Algeria	Mascara	Proportion	2000	26.9	40.6	15.8
Algeria	Mascara	Proportion	2017	33.9	50.9	20.2
Algeria	Mascara	Count	2000	0	0	0
Algeria	Mascara	Count	2017	0	1	0
Algeria	Khams Djouamaa	Proportion	2000	33.5	51.1	21.0
Algeria	Khams Djouamaa	Proportion	2017	36.0	50.3	22.5

Table 4: ORS by Unit *(continued)*

Country	Unit	Metric	Year	Mean	Upper	Lower
Algeria	Khams Djouamaa	Count	2000	0	1	0
Algeria	Khams Djouamaa	Count	2017	0	1	0
Algeria	Tiberguent	Proportion	2000	33.9	46.8	20.7
Algeria	Tiberguent	Proportion	2017	38.4	55.4	23.3
Algeria	Tiberguent	Count	2000	0	0	0
Algeria	Tiberguent	Count	2017	0	1	0
Algeria	Stidia	Proportion	2000	25.6	40.1	15.1
Algeria	Stidia	Proportion	2017	35.4	51.9	21.0
Algeria	Stidia	Count	2000	0	0	0
Algeria	Stidia	Count	2017	0	1	0
Algeria	Relizane	Proportion	2000	26.6	42.7	15.6
Algeria	Relizane	Proportion	2017	34.3	50.7	19.7
Algeria	Relizane	Count	2000	0	0	0
Algeria	Relizane	Count	2017	0	1	0
Algeria	Djemila	Proportion	2000	33.5	47.9	20.3
Algeria	Djemila	Proportion	2017	37.2	54.3	21.6
Algeria	Djemila	Count	2000	0	0	0
Algeria	Djemila	Count	2017	0	1	0
Algeria	Ras El Ma	Proportion	2000	31.4	49.6	17.7
Algeria	Ras El Ma	Proportion	2017	35.3	50.1	21.0
Algeria	Ras El Ma	Count	2000	0	0	0
Algeria	Ras El Ma	Count	2017	0	1	0
Algeria	Ouled Hebbaba	Proportion	2000	34.4	47.7	21.6
Algeria	Ouled Hebbaba	Proportion	2017	37.8	54.6	23.9
Algeria	Ouled Hebbaba	Count	2000	0	0	0
Algeria	Ouled Hebbaba	Count	2017	0	1	0
Algeria	Serghine	Proportion	2000	31.6	46.4	17.1
Algeria	Serghine	Proportion	2017	34.9	49.8	21.0
Algeria	Serghine	Count	2000	0	0	0
Algeria	Serghine	Count	2017	0	0	0
Algeria	Bouzeguene	Proportion	2000	28.9	44.1	17.1
Algeria	Bouzeguene	Proportion	2017	35.3	49.9	21.5
Algeria	Bouzeguene	Count	2000	0	0	0
Algeria	Bouzeguene	Count	2017	0	0	0
Algeria	Hennaya	Proportion	2000	29.8	46.3	17.5
Algeria	Hennaya	Proportion	2017	35.3	52.3	21.6
Algeria	Hennaya	Count	2000	0	0	0
Algeria	Hennaya	Count	2017	0	1	0
Algeria	Rouina	Proportion	2000	30.5	46.2	18.1
Algeria	Rouina	Proportion	2017	35.2	48.7	20.7
Algeria	Rouina	Count	2000	0	0	0
Algeria	Rouina	Count	2017	0	0	0
Algeria	Lazrou	Proportion	2000	32.7	47.0	18.6
Algeria	Lazrou	Proportion	2017	36.9	53.1	23.0
Algeria	Lazrou	Count	2000	0	0	0
Algeria	Lazrou	Count	2017	0	1	0
Algeria	Ouzzelaguen	Proportion	2000	30.3	46.2	17.9
Algeria	Ouzzelaguen	Proportion	2017	37.4	53.0	23.1
Algeria	Ouzzelaguen	Count	2000	0	0	0

Table 4: ORS by Unit *(continued)*

Country	Unit	Metric	Year	Mean	Upper	Lower
Algeria	Ouzzelaguen	Count	2017	0	1	0
Algeria	Sidi Okba	Proportion	2000	28.7	40.9	16.6
Algeria	Sidi Okba	Proportion	2017	32.3	46.7	19.7
Algeria	Sidi Okba	Count	2000	0	0	0
Algeria	Sidi Okba	Count	2017	0	0	0
Algeria	Taglait	Proportion	2000	31.8	46.0	19.9
Algeria	Taglait	Proportion	2017	35.2	51.8	21.3
Algeria	Taglait	Count	2000	0	0	0
Algeria	Taglait	Count	2017	0	1	0
Algeria	Lakhdaria	Proportion	2000	30.1	47.6	17.2
Algeria	Lakhdaria	Proportion	2017	36.0	50.3	22.3
Algeria	Lakhdaria	Count	2000	0	0	0
Algeria	Lakhdaria	Count	2017	0	1	0
Algeria	Timezrit	Proportion	2000	29.6	48.1	16.8
Algeria	Timezrit	Proportion	2017	35.5	50.1	21.8
Algeria	Timezrit	Count	2000	0	0	0
Algeria	Timezrit	Count	2017	0	1	0
Algeria	Tadjena	Proportion	2000	28.1	42.0	16.1
Algeria	Tadjena	Proportion	2017	33.4	49.9	19.1
Algeria	Tadjena	Count	2000	0	0	0
Algeria	Tadjena	Count	2017	0	0	0
Algeria	Selmana	Proportion	2000	30.1	45.9	18.1
Algeria	Selmana	Proportion	2017	31.7	48.4	20.3
Algeria	Selmana	Count	2000	0	0	0
Algeria	Selmana	Count	2017	0	0	0
Algeria	Ras El Agba	Proportion	2000	34.4	50.6	21.4
Algeria	Ras El Agba	Proportion	2017	36.8	52.2	23.2
Algeria	Ras El Agba	Count	2000	0	1	0
Algeria	Ras El Agba	Count	2017	0	1	0
Algeria	Ouanougha	Proportion	2000	32.5	48.3	18.0
Algeria	Ouanougha	Proportion	2017	34.6	50.2	20.1
Algeria	Ouanougha	Count	2000	0	0	0
Algeria	Ouanougha	Count	2017	0	1	0
Algeria	Mohammadia	Proportion	2000	25.8	39.1	15.5
Algeria	Mohammadia	Proportion	2017	35.4	52.0	20.2
Algeria	Mohammadia	Count	2000	0	0	0
Algeria	Mohammadia	Count	2017	0	1	0
Algeria	Ksar El Boukhari	Proportion	2000	34.1	51.3	20.5
Algeria	Ksar El Boukhari	Proportion	2017	36.5	52.0	23.4
Algeria	Ksar El Boukhari	Count	2000	0	1	0
Algeria	Ksar El Boukhari	Count	2017	0	1	0
Algeria	Yahia Beniguecha	Proportion	2000	33.9	47.5	20.2
Algeria	Yahia Beniguecha	Proportion	2017	38.2	54.4	23.0
Algeria	Yahia Beniguecha	Count	2000	0	0	0
Algeria	Yahia Beniguecha	Count	2017	0	1	0
Algeria	Tazgait	Proportion	2000	26.2	43.0	15.2
Algeria	Tazgait	Proportion	2017	35.3	50.1	21.6
Algeria	Tazgait	Count	2000	0	0	0
Algeria	Tazgait	Count	2017	0	1	0

Table 4: ORS by Unit *(continued)*

Country	Unit	Metric	Year	Mean	Upper	Lower
Algeria	Sidi Khettab	Proportion	2000	26.2	41.3	15.9
Algeria	Sidi Khettab	Proportion	2017	35.8	52.2	20.8
Algeria	Sidi Khettab	Count	2000	0	0	0
Algeria	Sidi Khettab	Count	2017	0	1	0
Algeria	Draa-Kebila	Proportion	2000	33.2	47.5	20.4
Algeria	Draa-Kebila	Proportion	2017	37.3	52.4	23.5
Algeria	Draa-Kebila	Count	2000	0	0	0
Algeria	Draa-Kebila	Count	2017	0	1	0
Algeria	Redjem Demouche	Proportion	2000	32.3	50.5	18.9
Algeria	Redjem Demouche	Proportion	2017	34.5	48.5	20.9
Algeria	Redjem Demouche	Count	2000	0	1	0
Algeria	Redjem Demouche	Count	2017	0	0	0
Algeria	Oum Toub	Proportion	2000	33.5	47.9	20.4
Algeria	Oum Toub	Proportion	2017	38.8	53.9	24.2
Algeria	Oum Toub	Count	2000	0	0	0
Algeria	Oum Toub	Count	2017	0	1	0
Algeria	Si Abdelghani	Proportion	2000	31.3	45.9	19.2
Algeria	Si Abdelghani	Proportion	2017	35.5	50.2	22.4
Algeria	Si Abdelghani	Count	2000	0	0	0
Algeria	Si Abdelghani	Count	2017	0	1	0
Algeria	Draa-Ben-Khedda	Proportion	2000	28.3	45.3	16.2
Algeria	Draa-Ben-Khedda	Proportion	2017	33.5	48.0	19.6
Algeria	Draa-Ben-Khedda	Count	2000	0	0	0
Algeria	Draa-Ben-Khedda	Count	2017	0	0	0
Algeria	Honaine	Proportion	2000	29.4	45.5	17.5
Algeria	Honaine	Proportion	2017	35.3	51.0	20.6
Algeria	Honaine	Count	2000	0	0	0
Algeria	Honaine	Count	2017	0	1	0
Algeria	Sidi Lakhdar	Proportion	2000	31.5	45.1	19.8
Algeria	Sidi Lakhdar	Proportion	2017	34.3	51.2	19.1
Algeria	Sidi Lakhdar	Count	2000	0	0	0
Algeria	Sidi Lakhdar	Count	2017	0	1	0
Algeria	Lemsane	Proportion	2000	33.1	48.9	21.1
Algeria	Lemsane	Proportion	2017	37.2	51.1	22.7
Algeria	Lemsane	Count	2000	0	0	0
Algeria	Lemsane	Count	2017	0	1	0
Algeria	Seddouk	Proportion	2000	30.6	46.7	18.3
Algeria	Seddouk	Proportion	2017	36.9	52.2	23.4
Algeria	Seddouk	Count	2000	0	0	0
Algeria	Seddouk	Count	2017	0	1	0
Algeria	Tolga	Proportion	2000	30.2	44.1	17.8
Algeria	Tolga	Proportion	2017	32.2	45.2	19.7
Algeria	Tolga	Count	2000	0	0	0
Algeria	Tolga	Count	2017	0	0	0
Algeria	Teniet En Nasr	Proportion	2000	33.3	49.3	20.8
Algeria	Teniet En Nasr	Proportion	2017	36.3	52.1	23.1
Algeria	Teniet En Nasr	Count	2000	0	0	0
Algeria	Teniet En Nasr	Count	2017	0	1	0
Algeria	M'Chedallah	Proportion	2000	31.6	48.3	18.0

Table 4: ORS by Unit *(continued)*

Country	Unit	Metric	Year	Mean	Upper	Lower
Algeria	M'Chedallah	Proportion	2017	37.7	53.6	23.5
Algeria	M'Chedallah	Count	2000	0	0	0
Algeria	M'Chedallah	Count	2017	0	1	0
Algeria	Zemmouri	Proportion	2000	29.1	48.1	17.3
Algeria	Zemmouri	Proportion	2017	34.8	49.9	21.7
Algeria	Zemmouri	Count	2000	0	0	0
Algeria	Zemmouri	Count	2017	0	0	0
Algeria	Taougrit	Proportion	2000	28.0	43.2	15.9
Algeria	Taougrit	Proportion	2017	34.4	52.1	19.1
Algeria	Taougrit	Count	2000	0	0	0
Algeria	Taougrit	Count	2017	0	1	0
Algeria	Sidi Baizid	Proportion	2000	31.4	45.4	17.1
Algeria	Sidi Baizid	Proportion	2017	32.2	45.6	19.7
Algeria	Sidi Baizid	Count	2000	0	0	0
Algeria	Sidi Baizid	Count	2017	0	0	0
Algeria	Roknia	Proportion	2000	34.4	49.1	21.6
Algeria	Roknia	Proportion	2017	37.9	53.9	25.2
Algeria	Roknia	Count	2000	0	0	0
Algeria	Roknia	Count	2017	0	1	0
Algeria	Ouled Addi Guebala	Proportion	2000	31.9	47.1	19.1
Algeria	Ouled Addi Guebala	Proportion	2017	35.1	52.0	20.7
Algeria	Ouled Addi Guebala	Count	2000	0	0	0
Algeria	Ouled Addi Guebala	Count	2017	0	1	0
Algeria	Nesmoth	Proportion	2000	29.2	45.6	17.8
Algeria	Nesmoth	Proportion	2017	34.6	49.9	21.7
Algeria	Nesmoth	Count	2000	0	0	0
Algeria	Nesmoth	Count	2017	0	0	0
Algeria	Medea	Proportion	2000	32.1	48.4	19.4
Algeria	Medea	Proportion	2017	32.1	47.6	18.7
Algeria	Medea	Count	2000	0	0	0
Algeria	Medea	Count	2017	0	0	0
Algeria	Zeghaia	Proportion	2000	33.9	46.6	20.3
Algeria	Zeghaia	Proportion	2017	37.6	55.0	23.1
Algeria	Zeghaia	Count	2000	0	0	0
Algeria	Zeghaia	Count	2017	0	1	0
Algeria	Touahria	Proportion	2000	25.7	39.5	15.5
Algeria	Touahria	Proportion	2017	35.2	51.2	20.3
Algeria	Touahria	Count	2000	0	0	0
Algeria	Touahria	Count	2017	0	1	0
Algeria	Sidi Lazreg	Proportion	2000	30.4	48.9	18.4
Algeria	Sidi Lazreg	Proportion	2017	35.5	52.2	20.7
Algeria	Sidi Lazreg	Count	2000	0	0	0
Algeria	Sidi Lazreg	Count	2017	0	1	0
Algeria	El Eulma	Proportion	2000	32.5	47.1	19.9
Algeria	El Eulma	Proportion	2017	35.4	51.5	20.3
Algeria	El Eulma	Count	2000	0	0	0
Algeria	El Eulma	Count	2017	0	1	0
Algeria	Sehala Thaoura	Proportion	2000	28.3	42.1	16.1
Algeria	Sehala Thaoura	Proportion	2017	34.6	49.4	20.7

Table 4: ORS by Unit *(continued)*

Country	Unit	Metric	Year	Mean	Upper	Lower
Algeria	Sehala Thaoura	Count	2000	0	0	0
Algeria	Sehala Thaoura	Count	2017	0	0	0
Algeria	Ramdane Djamel	Proportion	2000	33.4	50.5	20.7
Algeria	Ramdane Djamel	Proportion	2017	37.1	55.0	22.0
Algeria	Ramdane Djamel	Count	2000	0	1	0
Algeria	Ramdane Djamel	Count	2017	0	1	0
Algeria	Sidi Abderrahmane	Proportion	2000	31.5	46.7	19.6
Algeria	Sidi Abderrahmane	Proportion	2017	36.0	52.4	22.8
Algeria	Sidi Abderrahmane	Count	2000	0	0	0
Algeria	Sidi Abderrahmane	Count	2017	0	1	0
Algeria	Draa El Mizan	Proportion	2000	29.4	47.0	16.6
Algeria	Draa El Mizan	Proportion	2017	35.1	48.7	21.7
Algeria	Draa El Mizan	Count	2000	0	0	0
Algeria	Draa El Mizan	Count	2017	0	0	0
Algeria	Maghnia	Proportion	2000	31.0	46.5	18.9
Algeria	Maghnia	Proportion	2017	36.8	52.7	21.3
Algeria	Maghnia	Count	2000	0	0	0
Algeria	Maghnia	Count	2017	0	1	0
Algeria	Tacheta Zegagha	Proportion	2000	30.1	43.7	18.5
Algeria	Tacheta Zegagha	Proportion	2017	33.3	48.0	18.8
Algeria	Tacheta Zegagha	Count	2000	0	0	0
Algeria	Tacheta Zegagha	Count	2017	0	0	0
Algeria	M'Doukal	Proportion	2000	33.4	51.2	18.4
Algeria	M'Doukal	Proportion	2017	35.3	51.2	22.6
Algeria	M'Doukal	Count	2000	0	1	0
Algeria	M'Doukal	Count	2017	0	1	0
Algeria	Sidi Said	Proportion	2000	31.0	47.9	18.6
Algeria	Sidi Said	Proportion	2017	36.0	50.5	22.6
Algeria	Sidi Said	Count	2000	0	0	0
Algeria	Sidi Said	Count	2017	0	1	0
Algeria	Zeribet El Oued	Proportion	2000	29.2	45.8	16.4
Algeria	Zeribet El Oued	Proportion	2017	32.5	46.3	19.9
Algeria	Zeribet El Oued	Count	2000	0	0	0
Algeria	Zeribet El Oued	Count	2017	0	0	0
Algeria	Tesmart	Proportion	2000	33.6	48.7	20.8
Algeria	Tesmart	Proportion	2017	37.5	53.5	22.6
Algeria	Tesmart	Count	2000	0	0	0
Algeria	Tesmart	Count	2017	0	1	0
Algeria	Maala	Proportion	2000	31.3	48.8	18.5
Algeria	Maala	Proportion	2017	35.6	50.5	22.0
Algeria	Maala	Count	2000	0	0	0
Algeria	Maala	Count	2017	0	1	0
Algeria	Telassa	Proportion	2000	28.6	42.2	16.9
Algeria	Telassa	Proportion	2017	34.2	50.5	19.8
Algeria	Telassa	Count	2000	0	0	0
Algeria	Telassa	Count	2017	0	1	0
Algeria	Sidi Ladjel	Proportion	2000	30.2	44.8	16.5
Algeria	Sidi Ladjel	Proportion	2017	32.9	47.0	20.0
Algeria	Sidi Ladjel	Count	2000	0	0	0

Table 4: ORS by Unit *(continued)*

Country	Unit	Metric	Year	Mean	Upper	Lower
Algeria	Sidi Ladjel	Count	2017	0	0	0
Algeria	Selaoua Announa	Proportion	2000	34.7	51.6	21.7
Algeria	Selaoua Announa	Proportion	2017	37.5	53.1	23.8
Algeria	Selaoua Announa	Count	2000	0	1	0
Algeria	Selaoua Announa	Count	2017	0	1	0
Algeria	Ouled Derradj	Proportion	2000	32.2	47.9	18.8
Algeria	Ouled Derradj	Proportion	2017	35.2	53.3	20.6
Algeria	Ouled Derradj	Count	2000	0	0	0
Algeria	Ouled Derradj	Count	2017	0	1	0
Algeria	Oggaz	Proportion	2000	26.0	40.6	15.1
Algeria	Oggaz	Proportion	2017	34.8	50.4	19.3
Algeria	Oggaz	Count	2000	0	0	0
Algeria	Oggaz	Count	2017	0	1	0
Algeria	Medjebar	Proportion	2000	34.3	52.0	20.6
Algeria	Medjebar	Proportion	2017	35.9	51.7	22.7
Algeria	Medjebar	Count	2000	0	1	0
Algeria	Medjebar	Count	2017	0	1	0
Algeria	Sidi M'Hamed Benali	Proportion	2000	28.1	43.6	15.6
Algeria	Sidi M'Hamed Benali	Proportion	2017	34.7	51.6	19.3
Algeria	Sidi M'Hamed Benali	Count	2000	0	0	0
Algeria	Sidi M'Hamed Benali	Count	2017	0	1	0
Algeria	El Oueldja	Proportion	2000	33.5	46.9	19.5
Algeria	El Oueldja	Proportion	2017	37.4	51.7	22.5
Algeria	El Oueldja	Count	2000	0	0	0
Algeria	El Oueldja	Count	2017	0	1	0
Algeria	Sfisef	Proportion	2000	27.7	43.7	14.9
Algeria	Sfisef	Proportion	2017	34.9	49.2	20.2
Algeria	Sfisef	Count	2000	0	0	0
Algeria	Sfisef	Count	2017	0	0	0
Algeria	Salah Bouchaour	Proportion	2000	33.2	47.9	20.7
Algeria	Salah Bouchaour	Proportion	2017	37.5	54.9	22.7
Algeria	Salah Bouchaour	Count	2000	0	0	0
Algeria	Salah Bouchaour	Count	2017	0	1	0
Algeria	Sidi Ali Mellal	Proportion	2000	30.7	46.5	18.3
Algeria	Sidi Ali Mellal	Proportion	2017	35.0	52.1	20.6
Algeria	Sidi Ali Mellal	Count	2000	0	0	0
Algeria	Sidi Ali Mellal	Count	2017	0	1	0
Algeria	Freha	Proportion	2000	28.9	44.0	17.1
Algeria	Freha	Proportion	2017	35.2	48.7	21.6
Algeria	Freha	Count	2000	0	0	0
Algeria	Freha	Count	2017	0	0	0
Algeria	Mansourah	Proportion	2000	29.7	47.6	17.2
Algeria	Mansourah	Proportion	2017	33.9	50.4	20.8
Algeria	Mansourah	Count	2000	0	0	0
Algeria	Mansourah	Count	2017	0	1	0
Algeria	Tarik Ibn-Ziad	Proportion	2000	32.2	47.6	19.6
Algeria	Tarik Ibn-Ziad	Proportion	2017	34.1	47.5	20.4
Algeria	Tarik Ibn-Ziad	Count	2000	0	0	0
Algeria	Tarik Ibn-Ziad	Count	2017	0	0	0

Table 4: ORS by Unit *(continued)*

Country	Unit	Metric	Year	Mean	Upper	Lower
Algeria	Maafa	Proportion	2000	32.0	46.5	19.7
Algeria	Maafa	Proportion	2017	36.9	52.4	22.8
Algeria	Maafa	Count	2000	0	0	0
Algeria	Maafa	Count	2017	0	1	0
Algeria	Smaoun	Proportion	2000	30.8	46.9	19.0
Algeria	Smaoun	Proportion	2017	36.2	50.5	23.0
Algeria	Smaoun	Count	2000	0	0	0
Algeria	Smaoun	Count	2017	0	1	0
Algeria	Tixter	Proportion	2000	33.7	46.9	21.4
Algeria	Tixter	Proportion	2017	36.8	53.5	22.5
Algeria	Tixter	Count	2000	0	0	0
Algeria	Tixter	Count	2017	0	1	0
Algeria	Maamora	Proportion	2000	34.1	51.9	19.7
Algeria	Maamora	Proportion	2017	35.7	51.5	21.6
Algeria	Maamora	Count	2000	0	1	0
Algeria	Maamora	Count	2017	0	1	0
Algeria	Tenes	Proportion	2000	29.5	44.2	17.8
Algeria	Tenes	Proportion	2017	33.8	49.8	20.0
Algeria	Tenes	Count	2000	0	0	0
Algeria	Tenes	Count	2017	0	0	0
Algeria	Tadmit	Proportion	2000	30.8	48.2	17.9
Algeria	Tadmit	Proportion	2017	31.8	48.5	20.2
Algeria	Tadmit	Count	2000	0	0	0
Algeria	Tadmit	Count	2017	0	0	0
Algeria	Tamlouka	Proportion	2000	34.4	50.3	20.8
Algeria	Tamlouka	Proportion	2017	38.0	53.2	24.1
Algeria	Tamlouka	Count	2000	0	1	0
Algeria	Tamlouka	Count	2017	0	1	0
Algeria	Ouled Madhi	Proportion	2000	32.4	49.0	20.7
Algeria	Ouled Madhi	Proportion	2017	34.2	50.1	21.7
Algeria	Ouled Madhi	Count	2000	0	0	0
Algeria	Ouled Madhi	Count	2017	0	1	0
Algeria	Oued El Abtal	Proportion	2000	29.2	45.7	18.0
Algeria	Oued El Abtal	Proportion	2017	34.8	50.6	21.0
Algeria	Oued El Abtal	Count	2000	0	0	0
Algeria	Oued El Abtal	Count	2017	0	1	0
Algeria	Meftaha	Proportion	2000	34.3	51.1	21.1
Algeria	Meftaha	Proportion	2017	36.2	51.5	23.4
Algeria	Meftaha	Count	2000	0	1	0
Algeria	Meftaha	Count	2017	0	1	0
Algeria	Sidi M'Hamed Benaouda	Proportion	2000	28.3	44.3	17.1
Algeria	Sidi M'Hamed Benaouda	Proportion	2017	35.3	50.3	20.4
Algeria	Sidi M'Hamed Benaouda	Count	2000	0	0	0
Algeria	Sidi M'Hamed Benaouda	Count	2017	0	1	0
Algeria	El Ouricia	Proportion	2000	33.8	47.1	20.1
Algeria	El Ouricia	Proportion	2017	37.4	55.5	22.0
Algeria	El Ouricia	Count	2000	0	0	0
Algeria	El Ouricia	Count	2017	0	1	0
Algeria	Sidi Ali Benyoub	Proportion	2000	28.6	43.1	16.5

Table 4: ORS by Unit *(continued)*

Country	Unit	Metric	Year	Mean	Upper	Lower
Algeria	Sidi Ali Benyoub	Proportion	2017	34.5	50.8	21.0
Algeria	Sidi Ali Benyoub	Count	2000	0	0	0
Algeria	Sidi Ali Benyoub	Count	2017	0	1	0
Algeria	Sidi Mezghiche	Proportion	2000	33.7	48.9	20.6
Algeria	Sidi Mezghiche	Proportion	2017	40.3	55.7	25.1
Algeria	Sidi Mezghiche	Count	2000	0	0	0
Algeria	Sidi Mezghiche	Count	2017	0	1	0
Algeria	Sidi Bakhti	Proportion	2000	30.8	46.6	18.3
Algeria	Sidi Bakhti	Proportion	2017	35.0	53.4	21.8
Algeria	Sidi Bakhti	Count	2000	0	0	0
Algeria	Sidi Bakhti	Count	2017	0	1	0
Algeria	Frikat	Proportion	2000	29.7	46.4	16.4
Algeria	Frikat	Proportion	2017	35.1	49.0	21.8
Algeria	Frikat	Count	2000	0	0	0
Algeria	Frikat	Count	2017	0	0	0
Algeria	Marsa Ben M'Hidi	Proportion	2000	28.0	42.7	16.4
Algeria	Marsa Ben M'Hidi	Proportion	2017	34.0	48.8	21.9
Algeria	Marsa Ben M'Hidi	Count	2000	0	0	0
Algeria	Marsa Ben M'Hidi	Count	2017	0	0	0
Algeria	Tiberkanine	Proportion	2000	30.5	45.8	18.6
Algeria	Tiberkanine	Proportion	2017	34.9	49.3	20.5
Algeria	Tiberkanine	Count	2000	0	0	0
Algeria	Tiberkanine	Count	2017	0	0	0
Algeria	Menaa	Proportion	2000	31.6	45.5	19.6
Algeria	Menaa	Proportion	2017	36.7	51.8	22.5
Algeria	Menaa	Count	2000	0	0	0
Algeria	Menaa	Count	2017	0	1	0
Algeria	Souk El Tenine	Proportion	2000	30.7	45.1	17.5
Algeria	Souk El Tenine	Proportion	2017	38.0	55.4	24.5
Algeria	Souk El Tenine	Count	2000	0	0	0
Algeria	Souk El Tenine	Count	2017	0	1	0
Algeria	Mezdour	Proportion	2000	33.3	49.0	20.5
Algeria	Mezdour	Proportion	2017	36.1	51.5	22.4
Algeria	Mezdour	Count	2000	0	0	0
Algeria	Mezdour	Count	2017	0	1	0
Algeria	Zeboudja	Proportion	2000	29.4	44.2	17.2
Algeria	Zeboudja	Proportion	2017	33.3	50.4	19.5
Algeria	Zeboudja	Count	2000	0	0	0
Algeria	Zeboudja	Count	2017	0	1	0
Algeria	Zaafrane	Proportion	2000	30.7	45.1	17.5
Algeria	Zaafrane	Proportion	2017	32.0	46.3	20.0
Algeria	Zaafrane	Count	2000	0	0	0
Algeria	Zaafrane	Count	2017	0	0	0
Algeria	Ouled Mansour	Proportion	2000	32.5	49.3	18.7
Algeria	Ouled Mansour	Proportion	2017	34.8	51.8	22.5
Algeria	Ouled Mansour	Count	2000	0	0	0
Algeria	Ouled Mansour	Count	2017	0	1	0
Algeria	Oued Taria	Proportion	2000	29.7	43.7	18.0
Algeria	Oued Taria	Proportion	2017	35.0	51.2	21.7

Table 4: ORS by Unit *(continued)*

Country	Unit	Metric	Year	Mean	Upper	Lower
Algeria	Oued Taria	Count	2000	0	0	0
Algeria	Oued Taria	Count	2017	0	1	0
Algeria	Megheraoua	Proportion	2000	32.9	49.9	19.0
Algeria	Megheraoua	Proportion	2017	35.0	49.9	21.4
Algeria	Megheraoua	Count	2000	0	0	0
Algeria	Megheraoua	Count	2017	0	0	0
Algeria	Sidi Saada	Proportion	2000	25.8	39.2	15.1
Algeria	Sidi Saada	Proportion	2017	35.1	50.8	19.4
Algeria	Sidi Saada	Count	2000	0	0	0
Algeria	Sidi Saada	Count	2017	0	1	0
Algeria	Guellal	Proportion	2000	33.5	46.5	20.9
Algeria	Guellal	Proportion	2017	36.8	53.5	21.2
Algeria	Guellal	Count	2000	0	0	0
Algeria	Guellal	Count	2017	0	1	0
Algeria	Sidi Ali Boussidi	Proportion	2000	28.9	44.1	16.5
Algeria	Sidi Ali Boussidi	Proportion	2017	35.5	51.1	22.3
Algeria	Sidi Ali Boussidi	Count	2000	0	0	0
Algeria	Sidi Ali Boussidi	Count	2017	0	1	0
Algeria	Skikda	Proportion	2000	32.2	49.5	19.6
Algeria	Skikda	Proportion	2017	35.0	51.7	20.9
Algeria	Skikda	Count	2000	0	0	0
Algeria	Skikda	Count	2017	0	1	0
Algeria	Sidi Hosni	Proportion	2000	32.3	47.2	19.1
Algeria	Sidi Hosni	Proportion	2017	34.6	49.5	20.7
Algeria	Sidi Hosni	Count	2000	0	0	0
Algeria	Sidi Hosni	Count	2017	0	0	0
Algeria	Iboudraren	Proportion	2000	29.4	46.5	17.5
Algeria	Iboudraren	Proportion	2017	35.1	49.7	22.2
Algeria	Iboudraren	Count	2000	0	0	0
Algeria	Iboudraren	Count	2017	0	0	0
Algeria	Msirda Fouaga	Proportion	2000	30.0	46.2	18.2
Algeria	Msirda Fouaga	Proportion	2017	35.3	50.6	22.2
Algeria	Msirda Fouaga	Count	2000	0	0	0
Algeria	Msirda Fouaga	Count	2017	0	1	0
Algeria	Zeddine	Proportion	2000	31.1	47.0	18.6
Algeria	Zeddine	Proportion	2017	34.7	48.6	20.2
Algeria	Zeddine	Count	2000	0	0	0
Algeria	Zeddine	Count	2017	0	0	0
Algeria	Merouana	Proportion	2000	32.4	48.7	19.9
Algeria	Merouana	Proportion	2017	37.2	51.4	22.5
Algeria	Merouana	Count	2000	0	0	0
Algeria	Merouana	Count	2017	0	1	0
Algeria	Souk Oufella	Proportion	2000	30.0	45.0	17.4
Algeria	Souk Oufella	Proportion	2017	35.9	51.1	22.8
Algeria	Souk Oufella	Count	2000	0	0	0
Algeria	Souk Oufella	Count	2017	0	1	0
Algeria	Oued El Berdi	Proportion	2000	32.9	49.2	20.5
Algeria	Oued El Berdi	Proportion	2017	36.7	53.7	22.6
Algeria	Oued El Berdi	Count	2000	0	0	0

Table 4: ORS by Unit *(continued)*

Country	Unit	Metric	Year	Mean	Upper	Lower
Algeria	Oued El Berdi	Count	2017	0	1	0
Algeria	Zaccar	Proportion	2000	30.9	46.4	17.3
Algeria	Zaccar	Proportion	2017	31.6	45.8	19.6
Algeria	Zaccar	Count	2000	0	0	0
Algeria	Zaccar	Count	2017	0	0	0
Algeria	Ouled Sidi Brahim	Proportion	2000	32.2	47.7	17.8
Algeria	Ouled Sidi Brahim	Proportion	2017	34.4	49.3	21.1
Algeria	Ouled Sidi Brahim	Count	2000	0	0	0
Algeria	Ouled Sidi Brahim	Count	2017	0	0	0
Algeria	Ras Ain Amirouche	Proportion	2000	25.5	39.8	15.3
Algeria	Ras Ain Amirouche	Proportion	2017	34.3	48.6	19.4
Algeria	Ras Ain Amirouche	Count	2000	0	0	0
Algeria	Ras Ain Amirouche	Count	2017	0	0	0
Algeria	Mezrenna	Proportion	2000	32.5	48.9	20.0
Algeria	Mezrenna	Proportion	2017	35.2	49.8	21.6
Algeria	Mezrenna	Count	2000	0	0	0
Algeria	Mezrenna	Count	2017	0	0	0
Algeria	Souk El Had	Proportion	2000	29.7	45.2	17.2
Algeria	Souk El Had	Proportion	2017	34.7	51.4	20.9
Algeria	Souk El Had	Count	2000	0	0	0
Algeria	Souk El Had	Count	2017	0	1	0
Algeria	Guelta Zerka	Proportion	2000	33.1	47.5	20.5
Algeria	Guelta Zerka	Proportion	2017	37.4	53.5	21.7
Algeria	Guelta Zerka	Count	2000	0	0	0
Algeria	Guelta Zerka	Count	2017	0	1	0
Algeria	Sidi Bel Abbes	Proportion	2000	28.4	43.4	16.0
Algeria	Sidi Bel Abbes	Proportion	2017	34.2	51.6	18.6
Algeria	Sidi Bel Abbes	Count	2000	0	0	0
Algeria	Sidi Bel Abbes	Count	2017	0	1	0
Algeria	Tamalous	Proportion	2000	32.3	48.0	19.9
Algeria	Tamalous	Proportion	2017	36.5	52.4	22.0
Algeria	Tamalous	Count	2000	0	0	0
Algeria	Tamalous	Count	2017	0	1	0
Algeria	Sougueur	Proportion	2000	31.4	47.0	17.7
Algeria	Sougueur	Proportion	2017	35.8	51.3	22.5
Algeria	Sougueur	Count	2000	0	0	0
Algeria	Sougueur	Count	2017	0	1	0
Algeria	Idjeur	Proportion	2000	29.0	44.7	17.7
Algeria	Idjeur	Proportion	2017	34.6	48.8	21.4
Algeria	Idjeur	Count	2000	0	0	0
Algeria	Idjeur	Count	2017	0	0	0
Algeria	Nedroma	Proportion	2000	29.8	47.0	17.5
Algeria	Nedroma	Proportion	2017	36.1	52.9	21.1
Algeria	Nedroma	Count	2000	0	0	0
Algeria	Nedroma	Count	2017	0	1	0
Algeria	N'Gaous	Proportion	2000	31.9	47.8	19.9
Algeria	N'Gaous	Proportion	2017	37.7	55.1	22.2
Algeria	N'Gaous	Count	2000	0	0	0
Algeria	N'Gaous	Count	2017	0	1	0

Table 4: ORS by Unit *(continued)*

Country	Unit	Metric	Year	Mean	Upper	Lower
Algeria	Tala Hamza	Proportion	2000	29.0	45.1	17.0
Algeria	Tala Hamza	Proportion	2017	35.5	50.6	22.4
Algeria	Tala Hamza	Count	2000	0	0	0
Algeria	Tala Hamza	Count	2017	0	1	0
Algeria	Ouled Rached	Proportion	2000	33.6	49.4	20.4
Algeria	Ouled Rached	Proportion	2017	36.3	52.0	23.0
Algeria	Ouled Rached	Count	2000	0	0	0
Algeria	Ouled Rached	Count	2017	0	1	0
Algeria	Ouled Slimane	Proportion	2000	31.7	47.6	20.2
Algeria	Ouled Slimane	Proportion	2017	32.7	46.7	19.1
Algeria	Ouled Slimane	Count	2000	0	0	0
Algeria	Ouled Slimane	Count	2017	0	0	0
Algeria	Sedjerara	Proportion	2000	26.3	41.1	15.8
Algeria	Sedjerara	Proportion	2017	34.4	50.0	19.8
Algeria	Sedjerara	Count	2000	0	0	0
Algeria	Sedjerara	Count	2017	0	0	0
Algeria	Mihoub	Proportion	2000	33.3	50.3	19.7
Algeria	Mihoub	Proportion	2017	35.4	50.6	21.6
Algeria	Mihoub	Count	2000	0	1	0
Algeria	Mihoub	Count	2017	0	1	0
Algeria	Yellel	Proportion	2000	26.0	39.5	15.5
Algeria	Yellel	Proportion	2017	35.1	50.4	19.6
Algeria	Yellel	Count	2000	0	0	0
Algeria	Yellel	Count	2017	0	1	0
Algeria	Guenzet Tassameurt	Proportion	2000	33.7	49.9	21.4
Algeria	Guenzet Tassameurt	Proportion	2017	37.5	53.3	22.5
Algeria	Guenzet Tassameurt	Count	2000	0	0	0
Algeria	Guenzet Tassameurt	Count	2017	0	1	0
Algeria	Sidi Brahim	Proportion	2000	28.3	42.6	16.3
Algeria	Sidi Brahim	Proportion	2017	35.6	52.5	19.5
Algeria	Sidi Brahim	Count	2000	0	0	0
Algeria	Sidi Brahim	Count	2017	0	1	0
Algeria	Zerdeza	Proportion	2000	34.3	49.1	21.9
Algeria	Zerdeza	Proportion	2017	39.0	55.8	24.4
Algeria	Zerdeza	Count	2000	0	0	0
Algeria	Zerdeza	Count	2017	0	1	0
Algeria	Tagdemt	Proportion	2000	31.4	46.9	18.4
Algeria	Tagdemt	Proportion	2017	34.5	50.4	21.6
Algeria	Tagdemt	Count	2000	0	0	0
Algeria	Tagdemt	Count	2017	0	1	0
Algeria	Iferhounene	Proportion	2000	29.4	45.6	17.6
Algeria	Iferhounene	Proportion	2017	34.6	49.0	21.9
Algeria	Iferhounene	Count	2000	0	0	0
Algeria	Iferhounene	Count	2017	0	0	0
Algeria	Oued Chouly	Proportion	2000	29.7	43.9	17.1
Algeria	Oued Chouly	Proportion	2017	34.8	49.7	21.8
Algeria	Oued Chouly	Count	2000	0	0	0
Algeria	Oued Chouly	Count	2017	0	0	0
Algeria	Oued Chaaba	Proportion	2000	30.5	47.5	17.0

Table 4: ORS by Unit *(continued)*

Country	Unit	Metric	Year	Mean	Upper	Lower
Algeria	Oued Chaaba	Proportion	2017	37.7	52.1	21.9
Algeria	Oued Chaaba	Count	2000	0	0	0
Algeria	Oued Chaaba	Count	2017	0	1	0
Algeria	Tamokra	Proportion	2000	32.5	48.2	20.0
Algeria	Tamokra	Proportion	2017	37.5	53.1	24.0
Algeria	Tamokra	Count	2000	0	0	0
Algeria	Tamokra	Count	2017	0	1	0
Algeria	Raouraoua	Proportion	2000	33.6	50.4	19.9
Algeria	Raouraoua	Proportion	2017	36.1	50.9	23.2
Algeria	Raouraoua	Count	2000	0	1	0
Algeria	Raouraoua	Count	2017	0	1	0
Algeria	Oultene	Proportion	2000	31.1	45.9	19.1
Algeria	Oultene	Proportion	2017	33.3	48.4	19.5
Algeria	Oultene	Count	2000	0	0	0
Algeria	Oultene	Count	2017	0	0	0
Algeria	Sehailia	Proportion	2000	27.4	43.5	16.4
Algeria	Sehailia	Proportion	2017	34.4	48.4	20.9
Algeria	Sehailia	Count	2000	0	0	0
Algeria	Sehailia	Count	2017	0	0	0
Algeria	Ouamri	Proportion	2000	32.8	48.0	21.2
Algeria	Ouamri	Proportion	2017	34.1	48.3	21.0
Algeria	Ouamri	Count	2000	0	0	0
Algeria	Ouamri	Count	2017	0	0	0
Algeria	Zemmoura	Proportion	2000	28.8	44.5	17.2
Algeria	Zemmoura	Proportion	2017	35.8	51.4	21.0
Algeria	Zemmoura	Count	2000	0	0	0
Algeria	Zemmoura	Count	2017	0	1	0
Algeria	Guidjel	Proportion	2000	32.9	45.7	20.3
Algeria	Guidjel	Proportion	2017	37.2	53.1	21.8
Algeria	Guidjel	Count	2000	0	0	0
Algeria	Guidjel	Count	2017	0	1	0
Algeria	Sidi Chouab	Proportion	2000	31.9	46.8	18.7
Algeria	Sidi Chouab	Proportion	2017	34.6	50.6	21.4
Algeria	Sidi Chouab	Count	2000	0	0	0
Algeria	Sidi Chouab	Count	2017	0	1	0
Algeria	Zitouna	Proportion	2000	31.9	46.5	19.4
Algeria	Zitouna	Proportion	2017	36.5	51.9	22.4
Algeria	Zitouna	Count	2000	0	0	0
Algeria	Zitouna	Count	2017	0	1	0
Algeria	Takhemaret	Proportion	2000	30.3	45.7	17.7
Algeria	Takhemaret	Proportion	2017	36.0	54.0	22.2
Algeria	Takhemaret	Count	2000	0	0	0
Algeria	Takhemaret	Count	2017	0	1	0
Algeria	Ifigha	Proportion	2000	28.8	45.0	17.4
Algeria	Ifigha	Proportion	2017	35.4	49.5	22.4
Algeria	Ifigha	Count	2000	0	0	0
Algeria	Ifigha	Count	2017	0	0	0
Algeria	Ouled Mimoun	Proportion	2000	29.5	43.9	17.1
Algeria	Ouled Mimoun	Proportion	2017	35.3	50.6	22.3

Table 4: ORS by Unit *(continued)*

Country	Unit	Metric	Year	Mean	Upper	Lower
Algeria	Ouled Mimoun	Count	2000	0	0	0
Algeria	Ouled Mimoun	Count	2017	0	1	0
Algeria	Oued El Ma	Proportion	2000	32.7	49.6	19.8
Algeria	Oued El Ma	Proportion	2017	36.8	49.9	22.6
Algeria	Oued El Ma	Count	2000	0	0	0
Algeria	Oued El Ma	Count	2017	0	0	0
Algeria	Tamridjet	Proportion	2000	31.4	45.5	17.3
Algeria	Tamridjet	Proportion	2017	37.1	54.0	23.3
Algeria	Tamridjet	Count	2000	0	0	0
Algeria	Tamridjet	Count	2017	0	1	0
Algeria	Ridane	Proportion	2000	34.4	52.8	20.1
Algeria	Ridane	Proportion	2017	35.2	51.0	22.7
Algeria	Ridane	Count	2000	0	1	0
Algeria	Ridane	Count	2017	0	1	0
Algeria	Sidi Aissa	Proportion	2000	32.8	49.3	18.4
Algeria	Sidi Aissa	Proportion	2017	34.5	50.4	21.0
Algeria	Sidi Aissa	Count	2000	0	0	0
Algeria	Sidi Aissa	Count	2017	0	1	0
Algeria	Sidi Abdeldjebar	Proportion	2000	28.1	44.1	16.9
Algeria	Sidi Abdeldjebar	Proportion	2017	34.7	49.3	20.7
Algeria	Sidi Abdeldjebar	Count	2000	0	0	0
Algeria	Sidi Abdeldjebar	Count	2017	0	0	0
Algeria	Oued Harbil	Proportion	2000	33.1	47.8	20.8
Algeria	Oued Harbil	Proportion	2017	34.2	49.2	21.0
Algeria	Oued Harbil	Count	2000	0	0	0
Algeria	Oued Harbil	Count	2017	0	0	0
Algeria	Hamma	Proportion	2000	33.4	48.0	21.7
Algeria	Hamma	Proportion	2017	36.7	52.4	23.1
Algeria	Hamma	Count	2000	0	0	0
Algeria	Hamma	Count	2017	0	1	0
Algeria	Sidi Dahou Zair	Proportion	2000	28.6	43.2	15.9
Algeria	Sidi Dahou Zair	Proportion	2017	34.7	49.8	21.4
Algeria	Sidi Dahou Zair	Count	2000	0	0	0
Algeria	Sidi Dahou Zair	Count	2017	0	0	0
Algeria	Tiaret	Proportion	2000	32.3	47.9	18.4
Algeria	Tiaret	Proportion	2017	33.8	49.0	21.1
Algeria	Tiaret	Count	2000	0	0	0
Algeria	Tiaret	Count	2017	0	0	0
Algeria	Iflissen	Proportion	2000	28.1	44.8	16.7
Algeria	Iflissen	Proportion	2017	34.4	47.7	19.8
Algeria	Iflissen	Count	2000	0	0	0
Algeria	Iflissen	Count	2017	0	0	0
Algeria	Ouled Riyah	Proportion	2000	29.4	45.5	18.6
Algeria	Ouled Riyah	Proportion	2017	34.7	50.6	21.2
Algeria	Ouled Riyah	Count	2000	0	0	0
Algeria	Ouled Riyah	Count	2017	0	1	0
Algeria	Oued Taga	Proportion	2000	30.9	44.4	18.6
Algeria	Oued Taga	Proportion	2017	35.7	50.9	21.1
Algeria	Oued Taga	Count	2000	0	0	0

Table 4: ORS by Unit *(continued)*

Country	Unit	Metric	Year	Mean	Upper	Lower
Algeria	Oued Taga	Count	2017	0	1	0
Algeria	Taourit Ighil	Proportion	2000	30.0	45.9	18.1
Algeria	Taourit Ighil	Proportion	2017	35.6	50.5	22.4
Algeria	Taourit Ighil	Count	2000	0	0	0
Algeria	Taourit Ighil	Count	2017	0	1	0
Algeria	Saharidj	Proportion	2000	31.1	48.1	18.1
Algeria	Saharidj	Proportion	2017	36.1	52.1	22.6
Algeria	Saharidj	Count	2000	0	0	0
Algeria	Saharidj	Count	2017	0	1	0
Algeria	Sidi Ameur	Proportion	2000	31.8	47.5	18.9
Algeria	Sidi Ameur	Proportion	2017	33.9	47.9	21.6
Algeria	Sidi Ameur	Count	2000	0	0	0
Algeria	Sidi Ameur	Count	2017	0	0	0
Algeria	Sidi Abdelmoumene	Proportion	2000	25.1	39.0	14.6
Algeria	Sidi Abdelmoumene	Proportion	2017	34.7	50.4	20.2
Algeria	Sidi Abdelmoumene	Count	2000	0	0	0
Algeria	Sidi Abdelmoumene	Count	2017	0	1	0
Algeria	Ouezra	Proportion	2000	33.1	49.6	20.3
Algeria	Ouezra	Proportion	2017	34.2	49.7	20.2
Algeria	Ouezra	Count	2000	0	0	0
Algeria	Ouezra	Count	2017	0	0	0
Algeria	Hammam Guergour	Proportion	2000	32.5	46.2	20.0
Algeria	Hammam Guergour	Proportion	2017	38.2	53.7	23.8
Algeria	Hammam Guergour	Count	2000	0	0	0
Algeria	Hammam Guergour	Count	2017	0	1	0
Algeria	Sidi Hamadouche	Proportion	2000	27.4	40.3	16.1
Algeria	Sidi Hamadouche	Proportion	2017	35.2	50.3	19.9
Algeria	Sidi Hamadouche	Count	2000	0	0	0
Algeria	Sidi Hamadouche	Count	2017	0	1	0
Algeria	Tidda	Proportion	2000	31.2	46.5	18.7
Algeria	Tidda	Proportion	2017	34.8	52.1	21.5
Algeria	Tidda	Count	2000	0	0	0
Algeria	Tidda	Count	2017	0	1	0
Algeria	Illilten	Proportion	2000	29.7	45.5	17.8
Algeria	Illilten	Proportion	2017	34.8	48.9	22.0
Algeria	Illilten	Count	2000	0	0	0
Algeria	Illilten	Count	2017	0	0	0
Algeria	Remchi	Proportion	2000	29.8	46.2	18.5
Algeria	Remchi	Proportion	2017	35.5	52.1	21.8
Algeria	Remchi	Count	2000	0	0	0
Algeria	Remchi	Count	2017	0	1	0
Algeria	Ouled Ammar	Proportion	2000	32.5	48.2	19.2
Algeria	Ouled Ammar	Proportion	2017	36.1	52.8	22.2
Algeria	Ouled Ammar	Count	2000	0	0	0
Algeria	Ouled Ammar	Count	2017	0	1	0
Algeria	Taskriout	Proportion	2000	30.3	44.0	17.5
Algeria	Taskriout	Proportion	2017	38.0	55.1	24.6
Algeria	Taskriout	Count	2000	0	0	0
Algeria	Taskriout	Count	2017	0	1	0

Table 4: ORS by Unit *(continued)*

Country	Unit	Metric	Year	Mean	Upper	Lower
Algeria	Souk El Khemis	Proportion	2000	32.3	49.6	19.7
Algeria	Souk El Khemis	Proportion	2017	35.4	49.4	22.0
Algeria	Souk El Khemis	Count	2000	0	0	0
Algeria	Souk El Khemis	Count	2017	0	0	0
Algeria	Sidi Hadjeres	Proportion	2000	32.4	47.6	18.7
Algeria	Sidi Hadjeres	Proportion	2017	34.0	49.1	21.0
Algeria	Sidi Hadjeres	Count	2000	0	0	0
Algeria	Sidi Hadjeres	Count	2017	0	0	0
Algeria	Sidi Boussaid	Proportion	2000	28.8	44.7	17.9
Algeria	Sidi Boussaid	Proportion	2017	35.0	50.5	21.9
Algeria	Sidi Boussaid	Count	2000	0	0	0
Algeria	Sidi Boussaid	Count	2017	0	1	0
Algeria	Ouled Antar	Proportion	2000	32.7	50.7	21.3
Algeria	Ouled Antar	Proportion	2017	34.4	48.8	22.3
Algeria	Ouled Antar	Count	2000	0	1	0
Algeria	Ouled Antar	Count	2017	0	0	0
Algeria	Hammam Soukhna	Proportion	2000	34.1	48.4	20.4
Algeria	Hammam Soukhna	Proportion	2017	37.0	51.9	22.4
Algeria	Hammam Soukhna	Count	2000	0	0	0
Algeria	Hammam Soukhna	Count	2017	0	1	0
Algeria	Sidi Khaled	Proportion	2000	28.6	43.1	16.8
Algeria	Sidi Khaled	Proportion	2017	35.1	51.8	20.1
Algeria	Sidi Khaled	Count	2000	0	0	0
Algeria	Sidi Khaled	Count	2017	0	1	0
Algeria	Tousnina	Proportion	2000	30.6	46.1	17.6
Algeria	Tousnina	Proportion	2017	34.9	52.1	20.8
Algeria	Tousnina	Count	2000	0	0	0
Algeria	Tousnina	Count	2017	0	1	0
Algeria	Illoula Oumalou	Proportion	2000	28.8	43.9	16.7
Algeria	Illoula Oumalou	Proportion	2017	34.9	49.2	21.6
Algeria	Illoula Oumalou	Count	2000	0	0	0
Algeria	Illoula Oumalou	Count	2017	0	0	0
Algeria	Sabra	Proportion	2000	29.5	45.9	17.8
Algeria	Sabra	Proportion	2017	35.1	50.8	21.0
Algeria	Sabra	Count	2000	0	0	0
Algeria	Sabra	Count	2017	0	1	0
Algeria	Ouled Aouf	Proportion	2000	33.2	48.4	20.4
Algeria	Ouled Aouf	Proportion	2017	36.0	50.4	21.7
Algeria	Ouled Aouf	Count	2000	0	0	0
Algeria	Ouled Aouf	Count	2017	0	1	0
Algeria	Tazmalt	Proportion	2000	31.8	47.7	18.8
Algeria	Tazmalt	Proportion	2017	37.1	52.3	23.1
Algeria	Tazmalt	Count	2000	0	0	0
Algeria	Tazmalt	Count	2017	0	1	0
Algeria	Sour El Ghouzlane	Proportion	2000	33.0	49.3	19.4
Algeria	Sour El Ghouzlane	Proportion	2017	33.9	48.6	21.6
Algeria	Sour El Ghouzlane	Count	2000	0	0	0
Algeria	Sour El Ghouzlane	Count	2017	0	0	0
Algeria	Sidi M'Hamed	Proportion	2000	30.8	45.6	18.9

Table 4: ORS by Unit *(continued)*

Country	Unit	Metric	Year	Mean	Upper	Lower
Algeria	Sidi M'Hamed	Proportion	2017	33.3	50.0	19.9
Algeria	Sidi M'Hamed	Count	2000	0	0	0
Algeria	Sidi M'Hamed	Count	2017	0	0	0
Algeria	Sidi Kada	Proportion	2000	28.1	43.6	17.3
Algeria	Sidi Kada	Proportion	2017	35.1	49.9	22.0
Algeria	Sidi Kada	Count	2000	0	0	0
Algeria	Sidi Kada	Count	2017	0	0	0
Algeria	Ouled Bouachra	Proportion	2000	33.5	50.1	21.5
Algeria	Ouled Bouachra	Proportion	2017	34.9	50.5	21.0
Algeria	Ouled Bouachra	Count	2000	0	1	0
Algeria	Ouled Bouachra	Count	2017	0	1	0
Algeria	Harbil	Proportion	2000	33.2	47.5	20.9
Algeria	Harbil	Proportion	2017	37.2	52.6	22.6
Algeria	Harbil	Count	2000	0	0	0
Algeria	Harbil	Count	2017	0	1	0
Algeria	Sidi Lahcene	Proportion	2000	28.5	42.9	16.5
Algeria	Sidi Lahcene	Proportion	2017	34.9	52.1	19.7
Algeria	Sidi Lahcene	Count	2000	0	0	0
Algeria	Sidi Lahcene	Count	2017	0	1	0
Algeria	Zmalet El Emir Abdelkader	Proportion	2000	31.5	44.9	18.5
Algeria	Zmalet El Emir Abdelkader	Proportion	2017	34.9	49.3	22.5
Algeria	Zmalet El Emir Abdelkader	Count	2000	0	0	0
Algeria	Zmalet El Emir Abdelkader	Count	2017	0	0	0
Algeria	Imsouhal	Proportion	2000	29.1	45.4	17.4
Algeria	Imsouhal	Proportion	2017	34.6	48.7	21.9
Algeria	Imsouhal	Count	2000	0	0	0
Algeria	Imsouhal	Count	2017	0	0	0
Algeria	Sebbaa Chioukh	Proportion	2000	28.9	43.7	17.6
Algeria	Sebbaa Chioukh	Proportion	2017	34.3	49.2	20.6
Algeria	Sebbaa Chioukh	Count	2000	0	0	0
Algeria	Sebbaa Chioukh	Count	2017	0	0	0
Algeria	Ouled Fadhel	Proportion	2000	31.9	46.0	20.5
Algeria	Ouled Fadhel	Proportion	2017	36.1	51.1	21.4
Algeria	Ouled Fadhel	Count	2000	0	0	0
Algeria	Ouled Fadhel	Count	2017	0	1	0
Algeria	Tichy	Proportion	2000	30.7	45.3	18.9
Algeria	Tichy	Proportion	2017	37.2	53.1	24.4
Algeria	Tichy	Count	2000	0	0	0
Algeria	Tichy	Count	2017	0	1	0
Algeria	Taghzout	Proportion	2000	31.2	48.0	17.9
Algeria	Taghzout	Proportion	2017	36.0	50.6	22.3
Algeria	Taghzout	Count	2000	0	0	0
Algeria	Taghzout	Count	2017	0	1	0
Algeria	Slim	Proportion	2000	32.1	45.8	19.2
Algeria	Slim	Proportion	2017	34.2	48.4	20.3
Algeria	Slim	Count	2000	0	0	0

Table 4: ORS by Unit *(continued)*

Country	Unit	Metric	Year	Mean	Upper	Lower
Algeria	Slim	Count	2017	0	0	0
Algeria	Sig	Proportion	2000	26.3	42.0	14.9
Algeria	Sig	Proportion	2017	35.7	52.0	20.5
Algeria	Sig	Count	2000	0	0	0
Algeria	Sig	Count	2017	0	1	0
Algeria	Ouled Brahim	Proportion	2000	32.7	49.0	20.1
Algeria	Ouled Brahim	Proportion	2017	34.1	48.8	20.9
Algeria	Ouled Brahim	Count	2000	0	0	0
Algeria	Ouled Brahim	Count	2017	0	0	0
Algeria	Ksar El Abtal	Proportion	2000	33.2	45.7	20.8
Algeria	Ksar El Abtal	Proportion	2017	37.0	53.6	21.8
Algeria	Ksar El Abtal	Count	2000	0	0	0
Algeria	Ksar El Abtal	Count	2017	0	1	0
Algeria	Sidi Yacoub	Proportion	2000	28.6	43.7	16.8
Algeria	Sidi Yacoub	Proportion	2017	35.0	50.3	20.9
Algeria	Sidi Yacoub	Count	2000	0	0	0
Algeria	Sidi Yacoub	Count	2017	0	1	0
Algeria	Irdjen	Proportion	2000	28.5	47.2	16.1
Algeria	Irdjen	Proportion	2017	35.6	50.0	21.7
Algeria	Irdjen	Count	2000	0	0	0
Algeria	Irdjen	Count	2017	0	0	0
Algeria	Sebdou	Proportion	2000	31.0	48.4	18.2
Algeria	Sebdou	Proportion	2017	35.2	51.1	20.6
Algeria	Sebdou	Count	2000	0	0	0
Algeria	Sebdou	Count	2017	0	1	0
Algeria	Ouled Sellem	Proportion	2000	33.6	49.5	20.6
Algeria	Ouled Sellem	Proportion	2017	36.7	51.8	22.3
Algeria	Ouled Sellem	Count	2000	0	0	0
Algeria	Ouled Sellem	Count	2017	0	1	0
Algeria	Tifra	Proportion	2000	29.8	45.2	17.7
Algeria	Tifra	Proportion	2017	35.7	50.4	22.4
Algeria	Tifra	Count	2000	0	0	0
Algeria	Tifra	Count	2017	0	1	0
Algeria	Taguedit	Proportion	2000	33.9	49.6	20.7
Algeria	Taguedit	Proportion	2017	36.4	51.6	22.5
Algeria	Taguedit	Count	2000	0	0	0
Algeria	Taguedit	Count	2017	0	1	0
Algeria	Soumaa	Proportion	2000	32.2	47.9	19.8
Algeria	Soumaa	Proportion	2017	34.4	51.0	20.3
Algeria	Soumaa	Count	2000	0	0	0
Algeria	Soumaa	Count	2017	0	1	0
Algeria	Tighenif	Proportion	2000	27.4	42.6	16.5
Algeria	Tighenif	Proportion	2017	35.1	50.1	21.6
Algeria	Tighenif	Count	2000	0	0	0
Algeria	Tighenif	Count	2017	0	1	0
Algeria	Ouled Daid	Proportion	2000	33.2	51.0	21.3
Algeria	Ouled Daid	Proportion	2017	34.5	49.0	21.5
Algeria	Ouled Daid	Count	2000	0	1	0
Algeria	Ouled Daid	Count	2017	0	0	0

Table 4: ORS by Unit *(continued)*

Country	Unit	Metric	Year	Mean	Upper	Lower
Algeria	Maaouia	Proportion	2000	33.4	47.9	19.4
Algeria	Maaouia	Proportion	2017	37.2	54.8	22.1
Algeria	Maaouia	Count	2000	0	0	0
Algeria	Maaouia	Count	2017	0	1	0
Algeria	Tabia	Proportion	2000	28.9	43.9	16.6
Algeria	Tabia	Proportion	2017	34.9	51.2	21.3
Algeria	Tabia	Count	2000	0	0	0
Algeria	Tabia	Count	2017	0	1	0
Algeria	Larbaa-Nath-Irathen	Proportion	2000	28.1	45.9	16.4
Algeria	Larbaa-Nath-Irathen	Proportion	2017	35.2	49.4	21.1
Algeria	Larbaa-Nath-Irathen	Count	2000	0	0	0
Algeria	Larbaa-Nath-Irathen	Count	2017	0	0	0
Algeria	Sidi Abdelli	Proportion	2000	29.5	43.6	17.3
Algeria	Sidi Abdelli	Proportion	2017	34.9	50.0	22.6
Algeria	Sidi Abdelli	Count	2000	0	0	0
Algeria	Sidi Abdelli	Count	2017	0	0	0
Algeria	Ouled Si Slimane	Proportion	2000	32.8	48.7	20.3
Algeria	Ouled Si Slimane	Proportion	2017	37.5	53.1	22.0
Algeria	Ouled Si Slimane	Count	2000	0	0	0
Algeria	Ouled Si Slimane	Count	2017	0	1	0
Algeria	Timezrit	Proportion	2000	30.5	47.2	18.2
Algeria	Timezrit	Proportion	2017	36.2	50.8	22.8
Algeria	Timezrit	Count	2000	0	0	0
Algeria	Timezrit	Count	2017	0	1	0
Algeria	Zbarbar|El Isseri	Proportion	2000	31.9	49.0	18.8
Algeria	Zbarbar|El Isseri	Proportion	2017	35.3	50.5	22.0
Algeria	Zbarbar|El Isseri	Count	2000	0	0	0
Algeria	Zbarbar|El Isseri	Count	2017	0	1	0
Algeria	Tamsa	Proportion	2000	31.9	47.0	19.3
Algeria	Tamsa	Proportion	2017	33.9	48.8	21.1
Algeria	Tamsa	Count	2000	0	0	0
Algeria	Tamsa	Count	2017	0	0	0
Algeria	Tizi	Proportion	2000	27.8	40.9	16.5
Algeria	Tizi	Proportion	2017	35.1	51.5	21.4
Algeria	Tizi	Count	2000	0	0	0
Algeria	Tizi	Count	2017	0	1	0
Algeria	Ouled Hellal	Proportion	2000	32.6	50.2	21.4
Algeria	Ouled Hellal	Proportion	2017	34.1	48.7	21.9
Algeria	Ouled Hellal	Count	2000	0	1	0
Algeria	Ouled Hellal	Count	2017	0	0	0
Algeria	Maouaklane	Proportion	2000	32.5	46.8	20.2
Algeria	Maouaklane	Proportion	2017	37.0	53.1	23.4
Algeria	Maouaklane	Count	2000	0	0	0
Algeria	Maouaklane	Count	2017	0	1	0
Algeria	Tafissour	Proportion	2000	31.6	47.3	18.8
Algeria	Tafissour	Proportion	2017	35.1	50.2	20.9
Algeria	Tafissour	Count	2000	0	0	0
Algeria	Tafissour	Count	2017	0	1	0
Algeria	M’Kira	Proportion	2000	28.9	47.3	16.5

Table 4: ORS by Unit *(continued)*

Country	Unit	Metric	Year	Mean	Upper	Lower
Algeria	M'Kira	Proportion	2017	34.9	49.8	22.0
Algeria	M'Kira	Count	2000	0	0	0
Algeria	M'Kira	Count	2017	0	0	0
Algeria	Sidi Djilali	Proportion	2000	31.5	50.1	19.4
Algeria	Sidi Djilali	Proportion	2017	34.4	49.4	20.0
Algeria	Sidi Djilali	Count	2000	0	1	0
Algeria	Sidi Djilali	Count	2017	0	0	0
Algeria	Ouyoun El Assafir	Proportion	2000	31.4	46.4	18.8
Algeria	Ouyoun El Assafir	Proportion	2017	36.1	52.4	21.8
Algeria	Ouyoun El Assafir	Count	2000	0	0	0
Algeria	Ouyoun El Assafir	Count	2017	0	1	0
Algeria	Tinedbar	Proportion	2000	30.2	46.3	17.8
Algeria	Tinedbar	Proportion	2017	34.8	49.5	21.7
Algeria	Tinedbar	Count	2000	0	0	0
Algeria	Tinedbar	Count	2017	0	0	0
Algeria	Tarmount	Proportion	2000	32.6	47.3	17.6
Algeria	Tarmount	Proportion	2017	34.6	50.2	20.5
Algeria	Tarmount	Count	2000	0	0	0
Algeria	Tarmount	Count	2017	0	1	0
Algeria	Zahana	Proportion	2000	26.4	41.1	15.2
Algeria	Zahana	Proportion	2017	34.7	50.3	20.5
Algeria	Zahana	Count	2000	0	0	0
Algeria	Zahana	Count	2017	0	1	0
Algeria	Ouled Maaraf	Proportion	2000	33.9	51.3	20.5
Algeria	Ouled Maaraf	Proportion	2017	36.2	51.0	23.4
Algeria	Ouled Maaraf	Count	2000	0	1	0
Algeria	Ouled Maaraf	Count	2017	0	1	0
Algeria	Mezloug	Proportion	2000	33.3	46.8	20.2
Algeria	Mezloug	Proportion	2017	37.2	54.2	21.7
Algeria	Mezloug	Count	2000	0	0	0
Algeria	Mezloug	Count	2017	0	1	0
Algeria	Taghlimet	Proportion	2000	29.6	46.0	16.1
Algeria	Taghlimet	Proportion	2017	34.7	52.0	20.3
Algeria	Taghlimet	Count	2000	0	0	0
Algeria	Taghlimet	Count	2017	0	1	0
Algeria	Maatkas	Proportion	2000	28.3	44.8	15.5
Algeria	Maatkas	Proportion	2017	34.7	48.2	20.0
Algeria	Maatkas	Count	2000	0	0	0
Algeria	Maatkas	Count	2017	0	0	0
Algeria	Sidi Medjahed	Proportion	2000	30.2	46.7	18.2
Algeria	Sidi Medjahed	Proportion	2017	35.7	51.6	20.4
Algeria	Sidi Medjahed	Count	2000	0	0	0
Algeria	Sidi Medjahed	Count	2017	0	1	0
Algeria	Ras El Aioun	Proportion	2000	33.0	48.4	21.1
Algeria	Ras El Aioun	Proportion	2017	37.2	52.8	22.3
Algeria	Ras El Aioun	Count	2000	0	0	0
Algeria	Ras El Aioun	Count	2017	0	1	0
Algeria	Tizi-N'Berber	Proportion	2000	30.5	44.8	18.6
Algeria	Tizi-N'Berber	Proportion	2017	37.0	53.8	24.3

Table 4: ORS by Unit *(continued)*

Country	Unit	Metric	Year	Mean	Upper	Lower
Algeria	Tizi-N'Berber	Count	2000	0	0	0
Algeria	Tizi-N'Berber	Count	2017	0	1	0
Algeria	Zarzour	Proportion	2000	31.6	49.3	20.2
Algeria	Zarzour	Proportion	2017	32.9	48.1	19.6
Algeria	Zarzour	Count	2000	0	0	0
Algeria	Zarzour	Count	2017	0	0	0
Algeria	Zelmata	Proportion	2000	29.7	45.5	18.3
Algeria	Zelmata	Proportion	2017	35.0	49.2	22.0
Algeria	Zelmata	Count	2000	0	0	0
Algeria	Zelmata	Count	2017	0	0	0
Algeria	Oum El Djellil	Proportion	2000	33.6	50.9	21.2
Algeria	Oum El Djellil	Proportion	2017	36.0	50.9	23.1
Algeria	Oum El Djellil	Count	2000	0	1	0
Algeria	Oum El Djellil	Count	2017	0	1	0
Algeria	Oued El Barad	Proportion	2000	33.2	46.8	19.4
Algeria	Oued El Barad	Proportion	2017	37.3	53.8	22.9
Algeria	Oued El Barad	Count	2000	0	0	0
Algeria	Oued El Barad	Count	2017	0	1	0
Algeria	Taoudmout	Proportion	2000	31.0	46.4	18.5
Algeria	Taoudmout	Proportion	2017	34.9	50.9	21.0
Algeria	Taoudmout	Count	2000	0	0	0
Algeria	Taoudmout	Count	2017	0	1	0
Algeria	Makouda	Proportion	2000	28.0	44.7	16.0
Algeria	Makouda	Proportion	2017	34.1	47.5	19.5
Algeria	Makouda	Count	2000	0	0	0
Algeria	Makouda	Count	2017	0	0	0
Algeria	Souahlia	Proportion	2000	29.6	45.8	17.1
Algeria	Souahlia	Proportion	2017	35.3	52.7	21.5
Algeria	Souahlia	Count	2000	0	0	0
Algeria	Souahlia	Count	2017	0	1	0
Algeria	Rehbat	Proportion	2000	32.7	47.9	21.1
Algeria	Rehbat	Proportion	2017	37.0	52.5	22.2
Algeria	Rehbat	Count	2000	0	0	0
Algeria	Rehbat	Count	2017	0	1	0
Algeria	Toudja	Proportion	2000	30.1	45.6	18.7
Algeria	Toudja	Proportion	2017	35.4	50.4	22.8
Algeria	Toudja	Count	2000	0	0	0
Algeria	Toudja	Count	2017	0	1	0
Algeria	Roubia	Proportion	2000	34.2	52.6	20.4
Algeria	Roubia	Proportion	2017	35.5	49.5	22.5
Algeria	Roubia	Count	2000	0	1	0
Algeria	Roubia	Count	2017	0	0	0
Algeria	Ouled Addouane	Proportion	2000	33.4	46.4	19.8
Algeria	Ouled Addouane	Proportion	2017	37.1	53.6	22.3
Algeria	Ouled Addouane	Count	2000	0	0	0
Algeria	Ouled Addouane	Count	2017	0	1	0
Algeria	Tassala	Proportion	2000	28.1	42.0	16.3
Algeria	Tassala	Proportion	2017	34.7	49.7	20.2
Algeria	Tassala	Count	2000	0	0	0

Table 4: ORS by Unit *(continued)*

Country	Unit	Metric	Year	Mean	Upper	Lower
Algeria	Tassala	Count	2017	0	0	0
Algeria	Mechtrass	Proportion	2000	29.0	46.2	16.2
Algeria	Mechtrass	Proportion	2017	35.6	50.3	21.3
Algeria	Mechtrass	Count	2000	0	0	0
Algeria	Mechtrass	Count	2017	0	1	0
Algeria	Souani	Proportion	2000	30.2	46.0	18.5
Algeria	Souani	Proportion	2017	35.7	51.4	22.1
Algeria	Souani	Count	2000	0	0	0
Algeria	Souani	Count	2017	0	1	0
Algeria	Sefiane	Proportion	2000	32.1	47.9	20.3
Algeria	Sefiane	Proportion	2017	36.7	51.8	22.6
Algeria	Sefiane	Count	2000	0	0	0
Algeria	Sefiane	Count	2017	0	1	0
Algeria	Saneg	Proportion	2000	34.2	51.0	20.8
Algeria	Saneg	Proportion	2017	36.5	51.9	23.8
Algeria	Saneg	Count	2000	0	1	0
Algeria	Saneg	Count	2017	0	1	0
Algeria	Ouled Saber	Proportion	2000	33.6	46.7	20.9
Algeria	Ouled Saber	Proportion	2017	37.6	53.9	21.5
Algeria	Ouled Saber	Count	2000	0	0	0
Algeria	Ouled Saber	Count	2017	0	1	0
Algeria	Telagh	Proportion	2000	29.7	45.9	17.1
Algeria	Telagh	Proportion	2017	35.9	52.9	22.2
Algeria	Telagh	Count	2000	0	0	0
Algeria	Telagh	Count	2017	0	1	0
Algeria	Mekla	Proportion	2000	28.8	45.0	17.5
Algeria	Mekla	Proportion	2017	35.4	49.3	21.7
Algeria	Mekla	Count	2000	0	0	0
Algeria	Mekla	Count	2017	0	0	0
Algeria	Souk Tleta	Proportion	2000	29.6	46.3	17.2
Algeria	Souk Tleta	Proportion	2017	35.3	51.5	22.2
Algeria	Souk Tleta	Count	2000	0	0	0
Algeria	Souk Tleta	Count	2017	0	1	0
Algeria	Seggana	Proportion	2000	32.5	47.7	20.4
Algeria	Seggana	Proportion	2017	37.1	53.2	22.9
Algeria	Seggana	Count	2000	0	0	0
Algeria	Seggana	Count	2017	0	1	0
Algeria	Sedraya	Proportion	2000	33.7	50.3	19.4
Algeria	Sedraya	Proportion	2017	36.3	51.0	23.4
Algeria	Sedraya	Count	2000	0	1	0
Algeria	Sedraya	Count	2017	0	1	0
Algeria	Ouled Si Ahmed	Proportion	2000	32.4	46.0	21.0
Algeria	Ouled Si Ahmed	Proportion	2017	36.2	53.0	21.3
Algeria	Ouled Si Ahmed	Count	2000	0	0	0
Algeria	Ouled Si Ahmed	Count	2017	0	1	0
Algeria	Tenira	Proportion	2000	28.4	43.5	15.9
Algeria	Tenira	Proportion	2017	34.8	52.5	20.4
Algeria	Tenira	Count	2000	0	0	0
Algeria	Tenira	Count	2017	0	1	0

Table 4: ORS by Unit *(continued)*

Country	Unit	Metric	Year	Mean	Upper	Lower
Algeria	Mizrana	Proportion	2000	27.9	43.5	16.3
Algeria	Mizrana	Proportion	2017	33.9	47.4	19.2
Algeria	Mizrana	Count	2000	0	0	0
Algeria	Mizrana	Count	2017	0	0	0
Algeria	Terny Beni Hediel	Proportion	2000	29.9	47.6	17.3
Algeria	Terny Beni Hediel	Proportion	2017	35.4	51.8	22.0
Algeria	Terny Beni Hediel	Count	2000	0	0	0
Algeria	Terny Beni Hediel	Count	2017	0	1	0
Algeria	Seriana	Proportion	2000	31.9	47.1	18.3
Algeria	Seriana	Proportion	2017	36.8	51.6	22.1
Algeria	Seriana	Count	2000	0	0	0
Algeria	Seriana	Count	2017	0	1	0
Algeria	Seghouane	Proportion	2000	34.1	50.8	21.5
Algeria	Seghouane	Proportion	2017	36.0	50.4	23.4
Algeria	Seghouane	Count	2000	0	1	0
Algeria	Seghouane	Count	2017	0	1	0
Algeria	Ouled Tebben	Proportion	2000	32.7	45.6	19.8
Algeria	Ouled Tebben	Proportion	2017	36.1	52.5	22.1
Algeria	Ouled Tebben	Count	2000	0	0	0
Algeria	Ouled Tebben	Count	2017	0	1	0
Algeria	Tilmouni	Proportion	2000	28.0	42.2	15.8
Algeria	Tilmouni	Proportion	2017	35.3	51.8	19.6
Algeria	Tilmouni	Count	2000	0	0	0
Algeria	Tilmouni	Count	2017	0	1	0
Algeria	Ouacif	Proportion	2000	29.1	46.2	17.4
Algeria	Ouacif	Proportion	2017	35.3	49.7	22.2
Algeria	Ouacif	Count	2000	0	0	0
Algeria	Ouacif	Count	2017	0	0	0
Algeria	Tianet	Proportion	2000	29.6	46.7	17.9
Algeria	Tianet	Proportion	2017	35.4	51.7	21.1
Algeria	Tianet	Count	2000	0	0	0
Algeria	Tianet	Count	2017	0	1	0
Algeria	T Kout	Proportion	2000	30.9	45.0	18.4
Algeria	T Kout	Proportion	2017	36.1	52.3	21.5
Algeria	T Kout	Count	2000	0	0	0
Algeria	T Kout	Count	2017	0	1	0
Algeria	Si El Mahdjoub	Proportion	2000	33.1	49.2	20.6
Algeria	Si El Mahdjoub	Proportion	2017	34.3	50.0	20.5
Algeria	Si El Mahdjoub	Count	2000	0	0	0
Algeria	Si El Mahdjoub	Count	2017	0	0	0
Algeria	Rosfa	Proportion	2000	33.1	45.8	21.0
Algeria	Rosfa	Proportion	2017	36.5	52.5	22.0
Algeria	Rosfa	Count	2000	0	0	0
Algeria	Rosfa	Count	2017	0	1	0
Algeria	Zerouala	Proportion	2000	27.6	41.2	15.9
Algeria	Zerouala	Proportion	2017	34.9	50.6	19.6
Algeria	Zerouala	Count	2000	0	0	0
Algeria	Zerouala	Count	2017	0	1	0
Algeria	Ouadhia	Proportion	2000	29.1	46.3	16.3

Table 4: ORS by Unit *(continued)*

Country	Unit	Metric	Year	Mean	Upper	Lower
Algeria	Ouadhia	Proportion	2017	35.5	49.3	21.4
Algeria	Ouadhia	Count	2000	0	0	0
Algeria	Ouadhia	Count	2017	0	0	0
Algeria	Tlemcen	Proportion	2000	29.8	47.6	17.2
Algeria	Tlemcen	Proportion	2017	33.8	50.5	20.6
Algeria	Tlemcen	Count	2000	0	0	0
Algeria	Tlemcen	Count	2017	0	1	0
Algeria	Talkhamt	Proportion	2000	33.1	47.9	21.3
Algeria	Talkhamt	Proportion	2017	36.9	51.3	22.6
Algeria	Talkhamt	Count	2000	0	0	0
Algeria	Talkhamt	Count	2017	0	1	0
Algeria	Sidi Damed	Proportion	2000	34.5	51.0	19.4
Algeria	Sidi Damed	Proportion	2017	35.9	51.2	23.2
Algeria	Sidi Damed	Count	2000	0	1	0
Algeria	Sidi Damed	Count	2017	0	1	0
Algeria	Salah Bey	Proportion	2000	32.8	46.3	20.5
Algeria	Salah Bey	Proportion	2017	36.9	52.9	22.0
Algeria	Salah Bey	Count	2000	0	0	0
Algeria	Salah Bey	Count	2017	0	1	0
Algeria	Ouaguenoun	Proportion	2000	28.8	44.5	16.5
Algeria	Ouaguenoun	Proportion	2017	34.7	48.7	20.8
Algeria	Ouaguenoun	Count	2000	0	0	0
Algeria	Ouaguenoun	Count	2017	0	0	0
Algeria	Zenata	Proportion	2000	29.6	45.9	18.4
Algeria	Zenata	Proportion	2017	35.2	51.4	21.5
Algeria	Zenata	Count	2000	0	0	0
Algeria	Zenata	Count	2017	0	1	0
Algeria	Taxlent	Proportion	2000	32.1	47.9	20.1
Algeria	Taxlent	Proportion	2017	37.0	50.7	22.4
Algeria	Taxlent	Count	2000	0	0	0
Algeria	Taxlent	Count	2017	0	1	0
Algeria	Sidi Errabia	Proportion	2000	33.8	51.0	20.2
Algeria	Sidi Errabia	Proportion	2017	36.2	50.5	22.6
Algeria	Sidi Errabia	Count	2000	0	1	0
Algeria	Sidi Errabia	Count	2017	0	1	0
Algeria	Serdj-El-Ghoul	Proportion	2000	33.1	46.9	19.6
Algeria	Serdj-El-Ghoul	Proportion	2017	37.0	53.7	22.9
Algeria	Serdj-El-Ghoul	Count	2000	0	0	0
Algeria	Serdj-El-Ghoul	Count	2017	0	1	0
Algeria	Sidi Naamane	Proportion	2000	28.5	45.4	16.2
Algeria	Sidi Naamane	Proportion	2017	34.7	49.0	20.1
Algeria	Sidi Naamane	Count	2000	0	0	0
Algeria	Sidi Naamane	Count	2017	0	0	0
Algeria	Tazoult	Proportion	2000	31.4	46.8	17.7
Algeria	Tazoult	Proportion	2017	37.5	52.7	21.8
Algeria	Tazoult	Count	2000	0	0	0
Algeria	Tazoult	Count	2017	0	1	0
Algeria	Sidi Naamane	Proportion	2000	33.6	51.1	21.4
Algeria	Sidi Naamane	Proportion	2017	35.9	50.1	22.0

Table 4: ORS by Unit *(continued)*

Country	Unit	Metric	Year	Mean	Upper	Lower
Algeria	Sidi Naamane	Count	2000	0	1	0
Algeria	Sidi Naamane	Count	2017	0	1	0
Algeria	Setif	Proportion	2000	32.2	46.3	19.2
Algeria	Setif	Proportion	2017	36.1	54.1	20.9
Algeria	Setif	Count	2000	0	0	0
Algeria	Setif	Count	2017	0	1	0
Algeria	Souamaa	Proportion	2000	28.6	44.6	17.3
Algeria	Souamaa	Proportion	2017	35.3	49.4	22.2
Algeria	Souamaa	Count	2000	0	0	0
Algeria	Souamaa	Count	2017	0	0	0
Algeria	Teniet El Abed	Proportion	2000	30.7	45.3	18.5
Algeria	Teniet El Abed	Proportion	2017	35.0	50.5	21.2
Algeria	Teniet El Abed	Count	2000	0	0	0
Algeria	Teniet El Abed	Count	2017	0	1	0
Algeria	Sidi Zahar	Proportion	2000	34.9	53.1	20.2
Algeria	Sidi Zahar	Proportion	2017	35.8	49.6	23.7
Algeria	Sidi Zahar	Count	2000	0	1	0
Algeria	Sidi Zahar	Count	2017	0	0	0
Algeria	Tachouda	Proportion	2000	33.5	47.9	20.5
Algeria	Tachouda	Proportion	2017	36.6	52.8	21.0
Algeria	Tachouda	Count	2000	0	0	0
Algeria	Tachouda	Count	2017	0	1	0
Algeria	Souk El Tenine	Proportion	2000	28.2	45.4	15.8
Algeria	Souk El Tenine	Proportion	2017	34.5	48.2	20.2
Algeria	Souk El Tenine	Count	2000	0	0	0
Algeria	Souk El Tenine	Count	2017	0	0	0
Algeria	Tighanimine	Proportion	2000	30.4	44.6	17.8
Algeria	Tighanimine	Proportion	2017	36.0	52.0	21.4
Algeria	Tighanimine	Count	2000	0	0	0
Algeria	Tighanimine	Count	2017	0	1	0
Algeria	Sidi Ziane	Proportion	2000	34.8	53.1	20.5
Algeria	Sidi Ziane	Proportion	2017	36.3	50.3	23.5
Algeria	Sidi Ziane	Count	2000	0	1	0
Algeria	Sidi Ziane	Count	2017	0	1	0
Algeria	Tala-Ifacene	Proportion	2000	32.2	46.1	20.2
Algeria	Tala-Ifacene	Proportion	2017	36.8	52.8	23.3
Algeria	Tala-Ifacene	Count	2000	0	0	0
Algeria	Tala-Ifacene	Count	2017	0	1	0
Algeria	Tadmait	Proportion	2000	28.4	45.2	16.1
Algeria	Tadmait	Proportion	2017	34.8	49.4	21.0
Algeria	Tadmait	Count	2000	0	0	0
Algeria	Tadmait	Count	2017	0	0	0
Algeria	Tigharghar	Proportion	2000	32.2	46.6	19.9
Algeria	Tigharghar	Proportion	2017	36.9	51.8	22.8
Algeria	Tigharghar	Count	2000	0	0	0
Algeria	Tigharghar	Count	2017	0	1	0
Algeria	Souagui	Proportion	2000	34.0	51.7	20.1
Algeria	Souagui	Proportion	2017	35.6	49.2	22.8
Algeria	Souagui	Count	2000	0	1	0

Table 4: ORS by Unit *(continued)*

Country	Unit	Metric	Year	Mean	Upper	Lower
Algeria	Souagui	Count	2017	0	0	0
Algeria	Taya	Proportion	2000	34.1	49.3	19.0
Algeria	Taya	Proportion	2017	36.6	51.4	22.0
Algeria	Taya	Count	2000	0	0	0
Algeria	Taya	Count	2017	0	1	0
Algeria	Tigzirt	Proportion	2000	28.2	44.8	16.8
Algeria	Tigzirt	Proportion	2017	34.5	48.0	19.7
Algeria	Tigzirt	Count	2000	0	0	0
Algeria	Tigzirt	Count	2017	0	0	0
Algeria	Tilatou	Proportion	2000	32.8	48.3	20.4
Algeria	Tilatou	Proportion	2017	37.1	52.5	23.0
Algeria	Tilatou	Count	2000	0	0	0
Algeria	Tilatou	Count	2017	0	1	0
Algeria	Tablat	Proportion	2000	32.0	48.5	19.3
Algeria	Tablat	Proportion	2017	34.6	49.4	21.7
Algeria	Tablat	Count	2000	0	0	0
Algeria	Tablat	Count	2017	0	0	0
Algeria	Telaa	Proportion	2000	33.6	47.8	21.4
Algeria	Telaa	Proportion	2017	36.9	51.9	21.6
Algeria	Telaa	Count	2000	0	0	0
Algeria	Telaa	Count	2017	0	1	0
Algeria	Timizart	Proportion	2000	28.2	43.7	16.7
Algeria	Timizart	Proportion	2017	34.8	47.9	21.6
Algeria	Timizart	Count	2000	0	0	0
Algeria	Timizart	Count	2017	0	0	0
Algeria	Timgad	Proportion	2000	32.1	45.5	19.1
Algeria	Timgad	Proportion	2017	37.0	53.1	23.2
Algeria	Timgad	Count	2000	0	0	0
Algeria	Timgad	Count	2017	0	1	0
Algeria	Tafraout	Proportion	2000	35.1	53.8	20.6
Algeria	Tafraout	Proportion	2017	36.1	51.2	22.9
Algeria	Tafraout	Count	2000	0	1	0
Algeria	Tafraout	Count	2017	0	1	0
Algeria	Tizi N'Bechar	Proportion	2000	33.1	46.5	20.2
Algeria	Tizi N'Bechar	Proportion	2017	37.7	53.5	22.8
Algeria	Tizi N'Bechar	Count	2000	0	0	0
Algeria	Tizi N'Bechar	Count	2017	0	1	0
Algeria	Tirmitine	Proportion	2000	28.5	45.3	15.8
Algeria	Tirmitine	Proportion	2017	34.2	48.8	20.0
Algeria	Tirmitine	Count	2000	0	0	0
Algeria	Tirmitine	Count	2017	0	0	0
Algeria	Zanet El Beida	Proportion	2000	33.5	47.8	18.4
Algeria	Zanet El Beida	Proportion	2017	36.9	51.1	22.5
Algeria	Zanet El Beida	Count	2000	0	0	0
Algeria	Zanet El Beida	Count	2017	0	1	0
Algeria	Tamezguida	Proportion	2000	33.0	47.7	21.1
Algeria	Tamezguida	Proportion	2017	33.8	48.9	21.1
Algeria	Tamezguida	Count	2000	0	0	0
Algeria	Tamezguida	Count	2017	0	0	0

Table 4: ORS by Unit *(continued)*

Country	Unit	Metric	Year	Mean	Upper	Lower
Algeria	Tizi-Ghenif	Proportion	2000	29.0	46.6	16.7
Algeria	Tizi-Ghenif	Proportion	2017	34.8	49.0	21.9
Algeria	Tizi-Ghenif	Count	2000	0	0	0
Algeria	Tizi-Ghenif	Count	2017	0	0	0
Algeria	Thleth Douair	Proportion	2000	34.2	52.2	21.1
Algeria	Thleth Douair	Proportion	2017	35.4	49.6	23.2
Algeria	Thleth Douair	Count	2000	0	1	0
Algeria	Thleth Douair	Count	2017	0	0	0
Algeria	Tizi-Rached	Proportion	2000	28.4	46.0	16.2
Algeria	Tizi-Rached	Proportion	2017	35.4	49.3	21.2
Algeria	Tizi-Rached	Count	2000	0	0	0
Algeria	Tizi-Rached	Count	2017	0	0	0
Algeria	Tizi Mahdi	Proportion	2000	33.6	50.1	20.3
Algeria	Tizi Mahdi	Proportion	2017	34.5	50.3	20.2
Algeria	Tizi Mahdi	Count	2000	0	1	0
Algeria	Tizi Mahdi	Count	2017	0	1	0
Algeria	Tizi N'Tleta	Proportion	2000	28.7	46.1	16.0
Algeria	Tizi N'Tleta	Proportion	2017	35.5	49.8	21.2
Algeria	Tizi N'Tleta	Count	2000	0	0	0
Algeria	Tizi N'Tleta	Count	2017	0	0	0
Algeria	Zoubiria	Proportion	2000	33.5	50.4	21.2
Algeria	Zoubiria	Proportion	2017	34.8	49.4	21.9
Algeria	Zoubiria	Count	2000	0	1	0
Algeria	Zoubiria	Count	2017	0	0	0
Algeria	Tizi Ouzou	Proportion	2000	28.4	46.2	16.3
Algeria	Tizi Ouzou	Proportion	2017	33.3	46.9	19.8
Algeria	Tizi Ouzou	Count	2000	0	0	0
Algeria	Tizi Ouzou	Count	2017	0	0	0
Algeria	Yakourene	Proportion	2000	28.4	44.6	17.2
Algeria	Yakourene	Proportion	2017	34.8	48.5	21.0
Algeria	Yakourene	Count	2000	0	0	0
Algeria	Yakourene	Count	2017	0	0	0
Algeria	Yatafene	Proportion	2000	29.3	46.3	17.4
Algeria	Yatafene	Proportion	2017	35.1	49.9	22.4
Algeria	Yatafene	Count	2000	0	0	0
Algeria	Yatafene	Count	2017	0	0	0
Algeria	Zekri	Proportion	2000	29.1	45.8	18.0
Algeria	Zekri	Proportion	2017	34.4	48.3	21.5
Algeria	Zekri	Count	2000	0	0	0
Algeria	Zekri	Count	2017	0	0	0
Angola	Ambriz	Proportion	2000	28.4	51.7	11.8
Angola	Ambriz	Proportion	2017	39.3	60.8	20.4
Angola	Ambriz	Count	2000	0	1	0
Angola	Ambriz	Count	2017	0	1	0
Angola	Baía Farta	Proportion	2000	28.2	49.7	12.4
Angola	Baía Farta	Proportion	2017	39.4	60.1	18.8
Angola	Baía Farta	Count	2000	0	0	0
Angola	Baía Farta	Count	2017	0	1	0
Angola	Andulo	Proportion	2000	30.5	50.9	13.0

Table 4: ORS by Unit *(continued)*

Country	Unit	Metric	Year	Mean	Upper	Lower
Angola	Andulo	Proportion	2017	38.1	62.4	20.6
Angola	Andulo	Count	2000	0	1	0
Angola	Andulo	Count	2017	0	1	0
Angola	Belize	Proportion	2000	23.8	43.0	10.0
Angola	Belize	Proportion	2017	33.4	54.7	16.0
Angola	Belize	Count	2000	0	0	0
Angola	Belize	Count	2017	0	1	0
Angola	Calai	Proportion	2000	36.7	58.8	17.3
Angola	Calai	Proportion	2017	43.5	66.1	22.1
Angola	Calai	Count	2000	0	1	0
Angola	Calai	Count	2017	0	1	0
Angola	Ambaca	Proportion	2000	28.8	49.9	12.2
Angola	Ambaca	Proportion	2017	44.0	65.3	25.6
Angola	Ambaca	Count	2000	0	0	0
Angola	Ambaca	Count	2017	0	1	0
Angola	Amboim	Proportion	2000	27.3	48.9	10.2
Angola	Amboim	Proportion	2017	39.2	62.8	18.9
Angola	Amboim	Count	2000	0	0	0
Angola	Amboim	Count	2017	0	1	0
Angola	Cahama	Proportion	2000	39.3	64.6	18.3
Angola	Cahama	Proportion	2017	40.7	63.3	21.7
Angola	Cahama	Count	2000	0	1	0
Angola	Cahama	Count	2017	0	1	0
Angola	Bailundo	Proportion	2000	30.1	50.2	14.3
Angola	Bailundo	Proportion	2017	41.1	64.9	22.4
Angola	Bailundo	Count	2000	0	1	0
Angola	Bailundo	Count	2017	0	1	0
Angola	Caconda	Proportion	2000	37.2	62.0	17.9
Angola	Caconda	Proportion	2017	41.0	62.1	22.7
Angola	Caconda	Count	2000	0	1	0
Angola	Caconda	Count	2017	0	1	0
Angola	Cacuaco	Proportion	2000	37.0	63.3	18.3
Angola	Cacuaco	Proportion	2017	50.3	72.9	29.3
Angola	Cacuaco	Count	2000	0	1	0
Angola	Cacuaco	Count	2017	1	1	0
Angola	Cambulo	Proportion	2000	20.3	39.2	9.7
Angola	Cambulo	Proportion	2017	35.8	55.1	18.0
Angola	Cambulo	Count	2000	0	0	0
Angola	Cambulo	Count	2017	0	1	0
Angola	Cacolo	Proportion	2000	30.0	48.8	14.6
Angola	Cacolo	Proportion	2017	33.2	58.0	16.5
Angola	Cacolo	Count	2000	0	0	0
Angola	Cacolo	Count	2017	0	1	0
Angola	Cacuzo	Proportion	2000	27.3	48.8	12.4
Angola	Cacuzo	Proportion	2017	42.6	65.5	24.8
Angola	Cacuzo	Count	2000	0	0	0
Angola	Cacuzo	Count	2017	0	1	0
Angola	Alto Zambeze	Proportion	2000	27.4	47.3	12.7
Angola	Alto Zambeze	Proportion	2017	38.1	58.8	21.7

Table 4: ORS by Unit *(continued)*

Country	Unit	Metric	Year	Mean	Upper	Lower
Angola	Alto Zambeze	Count	2000	0	0	0
Angola	Alto Zambeze	Count	2017	0	1	0
Angola	Bibala	Proportion	2000	36.7	60.3	16.5
Angola	Bibala	Proportion	2017	41.2	62.7	21.5
Angola	Bibala	Count	2000	0	1	0
Angola	Bibala	Count	2017	0	1	0
Angola	Alto Cauale	Proportion	2000	27.2	49.7	10.6
Angola	Alto Cauale	Proportion	2017	40.6	62.5	22.0
Angola	Alto Cauale	Count	2000	0	0	0
Angola	Alto Cauale	Count	2017	0	1	0
Angola	Cuimba	Proportion	2000	27.3	53.0	10.2
Angola	Cuimba	Proportion	2017	44.1	63.0	22.4
Angola	Cuimba	Count	2000	0	1	0
Angola	Cuimba	Count	2017	0	1	0
Angola	Dande	Proportion	2000	30.0	55.8	12.8
Angola	Dande	Proportion	2017	44.1	64.9	23.8
Angola	Dande	Count	2000	0	1	0
Angola	Dande	Count	2017	0	1	0
Angola	Balombo	Proportion	2000	30.5	55.8	13.6
Angola	Balombo	Proportion	2017	37.3	59.2	18.3
Angola	Balombo	Count	2000	0	1	0
Angola	Balombo	Count	2017	0	1	0
Angola	Camacupa	Proportion	2000	33.1	56.0	15.0
Angola	Camacupa	Proportion	2017	36.3	61.4	19.5
Angola	Camacupa	Count	2000	0	1	0
Angola	Camacupa	Count	2017	0	1	0
Angola	Buco Zau	Proportion	2000	23.2	41.9	9.4
Angola	Buco Zau	Proportion	2017	32.4	53.9	16.2
Angola	Buco Zau	Count	2000	0	0	0
Angola	Buco Zau	Count	2017	0	1	0
Angola	Cuangar	Proportion	2000	35.0	56.0	18.2
Angola	Cuangar	Proportion	2017	41.7	61.6	19.8
Angola	Cuangar	Count	2000	0	1	0
Angola	Cuangar	Count	2017	0	1	0
Angola	Banga	Proportion	2000	26.2	47.6	11.1
Angola	Banga	Proportion	2017	43.2	65.6	24.1
Angola	Banga	Count	2000	0	0	0
Angola	Banga	Count	2017	0	1	0
Angola	Cassongue	Proportion	2000	29.3	52.0	11.7
Angola	Cassongue	Proportion	2017	38.6	61.5	18.8
Angola	Cassongue	Count	2000	0	1	0
Angola	Cassongue	Count	2017	0	1	0
Angola	Cuanhama	Proportion	2000	34.6	56.8	16.0
Angola	Cuanhama	Proportion	2017	47.1	67.5	26.8
Angola	Cuanhama	Count	2000	0	1	0
Angola	Cuanhama	Count	2017	0	1	0
Angola	Caála	Proportion	2000	34.1	58.1	16.4
Angola	Caála	Proportion	2017	47.2	70.1	28.6
Angola	Caála	Count	2000	0	1	0

Table 4: ORS by Unit *(continued)*

Country	Unit	Metric	Year	Mean	Upper	Lower
Angola	Caála	Count	2017	0	1	0
Angola	Caluquembe	Proportion	2000	37.3	62.1	19.0
Angola	Caluquembe	Proportion	2017	41.2	61.6	21.9
Angola	Caluquembe	Count	2000	0	1	0
Angola	Caluquembe	Count	2017	0	1	0
Angola	Cazenga	Proportion	2000	39.8	67.1	19.6
Angola	Cazenga	Proportion	2017	55.4	76.4	33.3
Angola	Cazenga	Count	2000	0	1	0
Angola	Cazenga	Count	2017	1	1	0
Angola	Capenda	Proportion	2000	24.5	43.2	10.8
Angola	Capenda	Proportion	2017	29.0	52.7	13.8
Angola	Capenda	Count	2000	0	0	0
Angola	Capenda	Count	2017	0	1	0
Angola	Dala	Proportion	2000	31.1	51.3	15.1
Angola	Dala	Proportion	2017	37.1	56.9	21.1
Angola	Dala	Count	2000	0	1	0
Angola	Dala	Count	2017	0	1	0
Angola	Calandula	Proportion	2000	28.1	49.6	11.6
Angola	Calandula	Proportion	2017	42.1	64.8	23.4
Angola	Calandula	Count	2000	0	0	0
Angola	Calandula	Count	2017	0	1	0
Angola	Camanongue	Proportion	2000	32.7	55.2	15.3
Angola	Camanongue	Proportion	2017	41.2	63.6	23.7
Angola	Camanongue	Count	2000	0	1	0
Angola	Camanongue	Count	2017	0	1	0
Angola	Camacuio	Proportion	2000	28.8	50.9	13.3
Angola	Camacuio	Proportion	2017	35.2	54.1	16.4
Angola	Camacuio	Count	2000	0	1	0
Angola	Camacuio	Count	2017	0	1	0
Angola	Ambuila	Proportion	2000	29.9	54.6	13.5
Angola	Ambuila	Proportion	2017	44.7	63.4	24.4
Angola	Ambuila	Count	2000	0	1	0
Angola	Ambuila	Count	2017	0	1	0
Angola	M'Banza Congo	Proportion	2000	28.5	54.2	10.7
Angola	M'Banza Congo	Proportion	2017	46.5	67.9	23.3
Angola	M'Banza Congo	Count	2000	0	1	0
Angola	M'Banza Congo	Count	2017	0	1	0
Angola	Icolo e Bengo	Proportion	2000	30.0	53.1	13.5
Angola	Icolo e Bengo	Proportion	2017	42.8	66.3	21.7
Angola	Icolo e Bengo	Count	2000	0	1	0
Angola	Icolo e Bengo	Count	2017	0	1	0
Angola	Benguela	Proportion	2000	27.8	50.9	12.1
Angola	Benguela	Proportion	2017	43.8	65.9	22.7
Angola	Benguela	Count	2000	0	1	0
Angola	Benguela	Count	2017	0	1	0
Angola	Catabola	Proportion	2000	33.9	54.8	15.6
Angola	Catabola	Proportion	2017	37.9	61.5	19.1
Angola	Catabola	Count	2000	0	1	0
Angola	Catabola	Count	2017	0	1	0

Table 4: ORS by Unit *(continued)*

Country	Unit	Metric	Year	Mean	Upper	Lower
Angola	Cabinda	Proportion	2000	27.4	47.7	12.2
Angola	Cabinda	Proportion	2017	39.1	60.9	19.8
Angola	Cabinda	Count	2000	0	0	0
Angola	Cabinda	Count	2017	0	1	0
Angola	Cuchi	Proportion	2000	37.8	60.6	18.1
Angola	Cuchi	Proportion	2017	40.9	63.7	23.2
Angola	Cuchi	Count	2000	0	1	0
Angola	Cuchi	Count	2017	0	1	0
Angola	Bolongongo	Proportion	2000	27.6	50.8	10.9
Angola	Bolongongo	Proportion	2017	44.2	65.4	24.5
Angola	Bolongongo	Count	2000	0	1	0
Angola	Bolongongo	Count	2017	0	1	0
Angola	Conda	Proportion	2000	27.1	47.4	10.4
Angola	Conda	Proportion	2017	36.7	58.7	17.5
Angola	Conda	Count	2000	0	0	0
Angola	Conda	Count	2017	0	1	0
Angola	Curoca	Proportion	2000	35.2	61.0	16.3
Angola	Curoca	Proportion	2017	38.5	61.1	21.3
Angola	Curoca	Count	2000	0	1	0
Angola	Curoca	Count	2017	0	1	0
Angola	Catchiungo	Proportion	2000	35.2	56.6	16.1
Angola	Catchiungo	Proportion	2017	45.4	69.3	25.9
Angola	Catchiungo	Count	2000	0	1	0
Angola	Catchiungo	Count	2017	0	1	0
Angola	Chibia	Proportion	2000	41.8	65.1	20.2
Angola	Chibia	Proportion	2017	41.9	64.0	22.5
Angola	Chibia	Count	2000	0	1	0
Angola	Chibia	Count	2017	0	1	0
Angola	Ingombota	Proportion	2000	39.8	67.1	18.9
Angola	Ingombota	Proportion	2017	54.7	76.2	31.7
Angola	Ingombota	Count	2000	0	1	0
Angola	Ingombota	Count	2017	1	1	0
Angola	Caungula	Proportion	2000	23.3	42.9	9.8
Angola	Caungula	Proportion	2017	32.8	54.8	15.1
Angola	Caungula	Count	2000	0	0	0
Angola	Caungula	Count	2017	0	1	0
Angola	Muconda	Proportion	2000	26.6	44.3	13.9
Angola	Muconda	Proportion	2017	33.2	54.2	17.5
Angola	Muconda	Count	2000	0	0	0
Angola	Muconda	Count	2017	0	1	0
Angola	Cambundi-Catembo	Proportion	2000	26.9	47.9	10.3
Angola	Cambundi-Catembo	Proportion	2017	36.5	60.2	17.9
Angola	Cambundi-Catembo	Count	2000	0	0	0
Angola	Cambundi-Catembo	Count	2017	0	1	0
Angola	Cameia	Proportion	2000	29.0	51.8	12.3
Angola	Cameia	Proportion	2017	36.5	60.6	19.4
Angola	Cameia	Count	2000	0	1	0
Angola	Cameia	Count	2017	0	1	0
Angola	Namibe	Proportion	2000	34.9	58.4	15.9

Table 4: ORS by Unit *(continued)*

Country	Unit	Metric	Year	Mean	Upper	Lower
Angola	Namibe	Proportion	2017	47.9	69.3	27.7
Angola	Namibe	Count	2000	0	1	0
Angola	Namibe	Count	2017	0	1	0
Angola	Bembe	Proportion	2000	27.7	53.1	11.7
Angola	Bembe	Proportion	2017	44.3	62.4	26.4
Angola	Bembe	Count	2000	0	1	0
Angola	Bembe	Count	2017	0	1	0
Angola	N'Zeto	Proportion	2000	30.4	53.9	14.3
Angola	N'Zeto	Proportion	2017	43.7	66.7	25.2
Angola	N'Zeto	Count	2000	0	1	0
Angola	N'Zeto	Count	2017	0	1	0
Angola	Muxima	Proportion	2000	27.8	47.5	13.1
Angola	Muxima	Proportion	2017	36.8	58.9	20.5
Angola	Muxima	Count	2000	0	0	0
Angola	Muxima	Count	2017	0	1	0
Angola	Bocoio	Proportion	2000	26.6	47.4	10.8
Angola	Bocoio	Proportion	2017	31.6	53.8	14.5
Angola	Bocoio	Count	2000	0	0	0
Angola	Bocoio	Count	2017	0	1	0
Angola	Chinguar	Proportion	2000	35.6	56.1	16.2
Angola	Chinguar	Proportion	2017	42.7	66.9	23.4
Angola	Chinguar	Count	2000	0	1	0
Angola	Chinguar	Count	2017	0	1	0
Angola	Landana	Proportion	2000	26.1	47.4	11.5
Angola	Landana	Proportion	2017	34.1	56.6	17.8
Angola	Landana	Count	2000	0	0	0
Angola	Landana	Count	2017	0	1	0
Angola	Cuito Cuanavale	Proportion	2000	35.1	58.3	16.8
Angola	Cuito Cuanavale	Proportion	2017	37.3	57.1	20.5
Angola	Cuito Cuanavale	Count	2000	0	1	0
Angola	Cuito Cuanavale	Count	2017	0	1	0
Angola	Bula Atumba	Proportion	2000	25.5	46.5	9.4
Angola	Bula Atumba	Proportion	2017	41.7	63.5	22.5
Angola	Bula Atumba	Count	2000	0	0	0
Angola	Bula Atumba	Count	2017	0	1	0
Angola	Ebo	Proportion	2000	27.5	46.6	11.2
Angola	Ebo	Proportion	2017	35.9	60.1	15.3
Angola	Ebo	Count	2000	0	0	0
Angola	Ebo	Count	2017	0	1	0
Angola	Cuvelai	Proportion	2000	36.6	59.2	15.3
Angola	Cuvelai	Proportion	2017	41.7	63.4	22.9
Angola	Cuvelai	Count	2000	0	1	0
Angola	Cuvelai	Count	2017	0	1	0
Angola	Ekunha	Proportion	2000	31.1	53.8	13.5
Angola	Ekunha	Proportion	2017	45.3	67.5	25.5
Angola	Ekunha	Count	2000	0	1	0
Angola	Ekunha	Count	2017	0	1	0
Angola	Chicomba	Proportion	2000	39.5	63.3	20.2
Angola	Chicomba	Proportion	2017	40.0	61.8	19.9

Table 4: ORS by Unit *(continued)*

Country	Unit	Metric	Year	Mean	Upper	Lower
Angola	Chicomba	Count	2000	0	1	0
Angola	Chicomba	Count	2017	0	1	0
Angola	Kilamba Kiaxi	Proportion	2000	38.9	66.2	18.5
Angola	Kilamba Kiaxi	Proportion	2017	52.3	74.5	29.9
Angola	Kilamba Kiaxi	Count	2000	0	1	0
Angola	Kilamba Kiaxi	Count	2017	1	1	0
Angola	Chitato	Proportion	2000	20.9	40.5	8.4
Angola	Chitato	Proportion	2017	42.3	62.3	23.5
Angola	Chitato	Count	2000	0	0	0
Angola	Chitato	Count	2017	0	1	0
Angola	Saurimo	Proportion	2000	26.2	44.7	13.4
Angola	Saurimo	Proportion	2017	36.6	57.6	19.2
Angola	Saurimo	Count	2000	0	0	0
Angola	Saurimo	Count	2017	0	1	0
Angola	Cangandala	Proportion	2000	27.3	47.4	11.1
Angola	Cangandala	Proportion	2017	41.3	64.8	21.6
Angola	Cangandala	Count	2000	0	0	0
Angola	Cangandala	Count	2017	0	1	0
Angola	Léua	Proportion	2000	32.3	55.6	14.5
Angola	Léua	Proportion	2017	40.4	61.9	22.3
Angola	Léua	Count	2000	0	1	0
Angola	Léua	Count	2017	0	1	0
Angola	Tombwa	Proportion	2000	34.0	58.1	16.1
Angola	Tombwa	Proportion	2017	40.2	60.1	23.5
Angola	Tombwa	Count	2000	0	1	0
Angola	Tombwa	Count	2017	0	1	0
Angola	Buengas	Proportion	2000	27.2	50.4	12.6
Angola	Buengas	Proportion	2017	41.5	63.8	22.9
Angola	Buengas	Count	2000	0	1	0
Angola	Buengas	Count	2017	0	1	0
Angola	Noqui	Proportion	2000	27.3	50.9	10.0
Angola	Noqui	Proportion	2017	41.5	63.3	21.7
Angola	Noqui	Count	2000	0	1	0
Angola	Noqui	Count	2017	0	1	0
Angola	Nambuangongo	Proportion	2000	30.5	56.2	13.0
Angola	Nambuangongo	Proportion	2017	43.8	65.4	24.8
Angola	Nambuangongo	Count	2000	0	1	0
Angola	Nambuangongo	Count	2017	0	1	0
Angola	Caiambambo	Proportion	2000	27.2	46.8	11.8
Angola	Caiambambo	Proportion	2017	34.4	54.0	16.1
Angola	Caiambambo	Count	2000	0	0	0
Angola	Caiambambo	Count	2017	0	1	0
Angola	Chitembo	Proportion	2000	34.1	55.9	17.6
Angola	Chitembo	Proportion	2017	36.9	61.3	19.2
Angola	Chitembo	Count	2000	0	1	0
Angola	Chitembo	Count	2017	0	1	0
Angola	Dirico	Proportion	2000	36.3	58.2	16.2
Angola	Dirico	Proportion	2017	43.7	68.3	22.1
Angola	Dirico	Count	2000	0	1	0

Table 4: ORS by Unit *(continued)*

Country	Unit	Metric	Year	Mean	Upper	Lower
Angola	Dirico	Count	2017	0	1	0
Angola	Cambambe	Proportion	2000	25.6	44.1	11.0
Angola	Cambambe	Proportion	2017	39.2	64.4	22.2
Angola	Cambambe	Count	2000	0	0	0
Angola	Cambambe	Count	2017	0	1	0
Angola	Libolo	Proportion	2000	26.8	46.2	12.1
Angola	Libolo	Proportion	2017	37.8	61.0	21.1
Angola	Libolo	Count	2000	0	0	0
Angola	Libolo	Count	2017	0	1	0
Angola	Namakunde	Proportion	2000	32.1	53.7	14.6
Angola	Namakunde	Proportion	2017	42.7	61.7	23.6
Angola	Namakunde	Count	2000	0	1	0
Angola	Namakunde	Count	2017	0	1	0
Angola	Huambo	Proportion	2000	32.4	53.8	14.8
Angola	Huambo	Proportion	2017	51.7	73.7	31.1
Angola	Huambo	Count	2000	0	1	0
Angola	Huambo	Count	2017	1	1	0
Angola	Chipindo	Proportion	2000	36.7	59.3	17.6
Angola	Chipindo	Proportion	2017	40.8	65.5	23.9
Angola	Chipindo	Count	2000	0	1	0
Angola	Chipindo	Count	2017	0	1	0
Angola	Maianga	Proportion	2000	39.7	67.1	18.6
Angola	Maianga	Proportion	2017	55.6	76.8	32.4
Angola	Maianga	Count	2000	0	1	0
Angola	Maianga	Count	2017	1	1	0
Angola	Cuango	Proportion	2000	23.7	43.4	9.2
Angola	Cuango	Proportion	2017	33.1	55.6	16.1
Angola	Cuango	Count	2000	0	0	0
Angola	Cuango	Count	2017	0	1	0
Angola	Caombo	Proportion	2000	24.3	46.7	8.3
Angola	Caombo	Proportion	2017	38.7	62.1	21.6
Angola	Caombo	Count	2000	0	0	0
Angola	Caombo	Count	2017	0	1	0
Angola	Luau	Proportion	2000	29.0	48.9	13.4
Angola	Luau	Proportion	2017	37.0	59.3	18.2
Angola	Luau	Count	2000	0	0	0
Angola	Luau	Count	2017	0	1	0
Angola	Virei	Proportion	2000	33.4	55.9	15.2
Angola	Virei	Proportion	2017	34.6	51.6	17.7
Angola	Virei	Count	2000	0	1	0
Angola	Virei	Count	2017	0	1	0
Angola	Bungo	Proportion	2000	29.2	53.5	12.0
Angola	Bungo	Proportion	2017	44.8	66.3	24.9
Angola	Bungo	Count	2000	0	1	0
Angola	Bungo	Count	2017	0	1	0
Angola	Soyo	Proportion	2000	30.9	53.5	13.6
Angola	Soyo	Proportion	2017	43.7	69.3	24.2
Angola	Soyo	Count	2000	0	1	0
Angola	Soyo	Count	2017	0	1	0

Table 4: ORS by Unit *(continued)*

Country	Unit	Metric	Year	Mean	Upper	Lower
Angola	Chongoroi	Proportion	2000	28.5	49.1	14.5
Angola	Chongoroi	Proportion	2017	33.9	52.3	16.4
Angola	Chongoroi	Count	2000	0	0	0
Angola	Chongoroi	Count	2017	0	1	0
Angola	Cuemba	Proportion	2000	31.8	52.7	14.2
Angola	Cuemba	Proportion	2017	35.2	56.7	18.2
Angola	Cuemba	Count	2000	0	1	0
Angola	Cuemba	Count	2017	0	1	0
Angola	Mavinga	Proportion	2000	31.6	51.7	14.1
Angola	Mavinga	Proportion	2017	37.5	57.6	19.3
Angola	Mavinga	Count	2000	0	1	0
Angola	Mavinga	Count	2017	0	1	0
Angola	Cazengo	Proportion	2000	25.8	45.8	10.4
Angola	Cazengo	Proportion	2017	44.6	68.4	25.6
Angola	Cazengo	Count	2000	0	0	0
Angola	Cazengo	Count	2017	0	1	0
Angola	Mussende	Proportion	2000	27.7	47.4	14.2
Angola	Mussende	Proportion	2017	38.0	62.7	19.8
Angola	Mussende	Count	2000	0	0	0
Angola	Mussende	Count	2017	0	1	0
Angola	Ombadja	Proportion	2000	37.6	62.3	16.9
Angola	Ombadja	Proportion	2017	44.4	67.2	24.9
Angola	Ombadja	Count	2000	0	1	0
Angola	Ombadja	Count	2017	0	1	0
Angola	Londuimbale	Proportion	2000	31.2	54.6	14.6
Angola	Londuimbale	Proportion	2017	43.0	67.8	22.9
Angola	Londuimbale	Count	2000	0	1	0
Angola	Londuimbale	Count	2017	0	1	0
Angola	Gambos	Proportion	2000	40.3	65.8	18.3
Angola	Gambos	Proportion	2017	39.3	61.1	19.1
Angola	Gambos	Count	2000	0	1	0
Angola	Gambos	Count	2017	0	1	0
Angola	Rangel	Proportion	2000	39.6	67.1	19.2
Angola	Rangel	Proportion	2017	56.2	77.0	33.6
Angola	Rangel	Count	2000	0	1	0
Angola	Rangel	Count	2017	1	1	0
Angola	Cuilo	Proportion	2000	22.0	41.9	9.0
Angola	Cuilo	Proportion	2017	34.1	54.3	16.6
Angola	Cuilo	Count	2000	0	0	0
Angola	Cuilo	Count	2017	0	1	0
Angola	Cuaba Nzogo	Proportion	2000	26.2	47.4	10.1
Angola	Cuaba Nzogo	Proportion	2017	42.9	67.0	24.8
Angola	Cuaba Nzogo	Count	2000	0	0	0
Angola	Cuaba Nzogo	Count	2017	0	1	0
Angola	Lucano	Proportion	2000	27.5	47.9	13.2
Angola	Lucano	Proportion	2017	33.8	54.1	17.9
Angola	Lucano	Count	2000	0	0	0
Angola	Lucano	Count	2017	0	1	0
Angola	Damba	Proportion	2000	28.5	52.3	12.9

Table 4: ORS by Unit *(continued)*

Country	Unit	Metric	Year	Mean	Upper	Lower
Angola	Damba	Proportion	2017	44.0	64.5	25.1
Angola	Damba	Count	2000	0	1	0
Angola	Damba	Count	2017	0	1	0
Angola	Tomboco	Proportion	2000	28.4	52.2	11.9
Angola	Tomboco	Proportion	2017	42.9	64.9	24.8
Angola	Tomboco	Count	2000	0	1	0
Angola	Tomboco	Count	2017	0	1	0
Angola	Cubal	Proportion	2000	27.9	48.6	13.0
Angola	Cubal	Proportion	2017	33.9	53.0	16.2
Angola	Cubal	Count	2000	0	0	0
Angola	Cubal	Count	2017	0	1	0
Angola	Cunhinga	Proportion	2000	33.7	55.1	15.1
Angola	Cunhinga	Proportion	2017	40.0	64.7	20.3
Angola	Cunhinga	Count	2000	0	1	0
Angola	Cunhinga	Count	2017	0	1	0
Angola	Menongue	Proportion	2000	37.5	61.3	18.0
Angola	Menongue	Proportion	2017	44.4	65.1	24.1
Angola	Menongue	Count	2000	0	1	0
Angola	Menongue	Count	2017	0	1	0
Angola	Dembos	Proportion	2000	28.1	51.4	10.6
Angola	Dembos	Proportion	2017	44.5	66.9	25.0
Angola	Dembos	Count	2000	0	1	0
Angola	Dembos	Count	2017	0	1	0
Angola	Porto Amboim	Proportion	2000	26.2	47.5	11.3
Angola	Porto Amboim	Proportion	2017	40.5	62.3	21.2
Angola	Porto Amboim	Count	2000	0	0	0
Angola	Porto Amboim	Count	2017	0	1	0
Angola	Longonjo	Proportion	2000	32.0	56.6	14.0
Angola	Longonjo	Proportion	2017	43.7	66.5	25.0
Angola	Longonjo	Count	2000	0	1	0
Angola	Longonjo	Count	2017	0	1	0
Angola	Humpata	Proportion	2000	47.1	69.4	22.9
Angola	Humpata	Proportion	2017	54.7	73.8	31.6
Angola	Humpata	Count	2000	0	1	0
Angola	Humpata	Count	2017	1	1	0
Angola	Samba	Proportion	2000	36.0	62.2	16.6
Angola	Samba	Proportion	2017	49.1	71.8	27.2
Angola	Samba	Count	2000	0	1	0
Angola	Samba	Count	2017	0	1	0
Angola	Lubalo	Proportion	2000	23.4	40.0	10.5
Angola	Lubalo	Proportion	2017	27.7	50.7	12.2
Angola	Lubalo	Count	2000	0	0	0
Angola	Lubalo	Count	2017	0	1	0
Angola	Cunda-dia-Baza	Proportion	2000	22.6	44.4	7.8
Angola	Cunda-dia-Baza	Proportion	2017	33.4	56.6	17.8
Angola	Cunda-dia-Baza	Count	2000	0	0	0
Angola	Cunda-dia-Baza	Count	2017	0	1	0
Angola	Luchazes	Proportion	2000	32.5	51.8	17.4
Angola	Luchazes	Proportion	2017	36.6	57.3	19.1

Table 4: ORS by Unit *(continued)*

Country	Unit	Metric	Year	Mean	Upper	Lower
Angola	Luchazes	Count	2000	0	1	0
Angola	Luchazes	Count	2017	0	1	0
Angola	Maquela do Zombo	Proportion	2000	25.6	46.4	11.0
Angola	Maquela do Zombo	Proportion	2017	42.3	64.2	24.1
Angola	Maquela do Zombo	Count	2000	0	0	0
Angola	Maquela do Zombo	Count	2017	0	1	0
Angola	Ganda	Proportion	2000	30.5	55.4	13.7
Angola	Ganda	Proportion	2017	37.1	57.7	18.8
Angola	Ganda	Count	2000	0	1	0
Angola	Ganda	Count	2017	0	1	0
Angola	Kuito	Proportion	2000	35.6	56.3	17.1
Angola	Kuito	Proportion	2017	42.7	66.9	23.3
Angola	Kuito	Count	2000	0	1	0
Angola	Kuito	Count	2017	0	1	0
Angola	Nancova	Proportion	2000	32.0	50.9	15.2
Angola	Nancova	Proportion	2017	36.3	57.6	16.0
Angola	Nancova	Count	2000	0	1	0
Angola	Nancova	Count	2017	0	1	0
Angola	Golungo Alto	Proportion	2000	26.2	46.8	10.8
Angola	Golungo Alto	Proportion	2017	42.6	65.6	25.1
Angola	Golungo Alto	Count	2000	0	0	0
Angola	Golungo Alto	Count	2017	0	1	0
Angola	Quibala	Proportion	2000	28.3	47.0	13.8
Angola	Quibala	Proportion	2017	36.8	60.6	18.9
Angola	Quibala	Count	2000	0	0	0
Angola	Quibala	Count	2017	0	1	0
Angola	Mungo	Proportion	2000	30.5	52.2	11.7
Angola	Mungo	Proportion	2017	41.6	65.7	21.1
Angola	Mungo	Count	2000	0	1	0
Angola	Mungo	Count	2017	0	1	0
Angola	Jamba	Proportion	2000	39.8	63.1	20.0
Angola	Jamba	Proportion	2017	41.9	64.8	23.2
Angola	Jamba	Count	2000	0	1	0
Angola	Jamba	Count	2017	0	1	0
Angola	Sambizanga	Proportion	2000	39.5	67.6	18.9
Angola	Sambizanga	Proportion	2017	55.9	76.9	33.5
Angola	Sambizanga	Count	2000	0	1	0
Angola	Sambizanga	Count	2017	1	1	0
Angola	Lucapa	Proportion	2000	22.4	38.7	9.9
Angola	Lucapa	Proportion	2017	36.0	56.9	20.5
Angola	Lucapa	Count	2000	0	0	0
Angola	Lucapa	Count	2017	0	1	0
Angola	Luquembo	Proportion	2000	27.8	47.9	11.1
Angola	Luquembo	Proportion	2017	32.5	56.5	15.3
Angola	Luquembo	Count	2000	0	0	0
Angola	Luquembo	Count	2017	0	1	0
Angola	Lumbala-Nguimbo	Proportion	2000	30.5	49.0	14.2
Angola	Lumbala-Nguimbo	Proportion	2017	39.7	60.9	20.3
Angola	Lumbala-Nguimbo	Count	2000	0	0	0

Table 4: ORS by Unit *(continued)*

Country	Unit	Metric	Year	Mean	Upper	Lower
Angola	Lumbala-Nguimbo	Count	2017	0	1	0
Angola	Milunga	Proportion	2000	24.8	46.8	9.6
Angola	Milunga	Proportion	2017	37.6	60.1	20.4
Angola	Milunga	Count	2000	0	0	0
Angola	Milunga	Count	2017	0	1	0
Angola	Lobito	Proportion	2000	27.5	53.5	12.1
Angola	Lobito	Proportion	2017	39.7	62.1	20.8
Angola	Lobito	Count	2000	0	1	0
Angola	Lobito	Count	2017	0	1	0
Angola	Nharea	Proportion	2000	30.8	52.4	14.1
Angola	Nharea	Proportion	2017	35.2	58.6	18.0
Angola	Nharea	Count	2000	0	1	0
Angola	Nharea	Count	2017	0	1	0
Angola	Rivungo	Proportion	2000	32.3	54.1	14.4
Angola	Rivungo	Proportion	2017	42.1	64.6	22.4
Angola	Rivungo	Count	2000	0	1	0
Angola	Rivungo	Count	2017	0	1	0
Angola	Lucala	Proportion	2000	24.7	45.1	10.8
Angola	Lucala	Proportion	2017	42.5	64.2	24.9
Angola	Lucala	Count	2000	0	0	0
Angola	Lucala	Count	2017	0	1	0
Angola	Quilenda	Proportion	2000	26.3	47.5	10.4
Angola	Quilenda	Proportion	2017	35.5	58.4	16.8
Angola	Quilenda	Count	2000	0	0	0
Angola	Quilenda	Count	2017	0	1	0
Angola	Tchicala-Tcholoanga	Proportion	2000	33.7	56.0	16.0
Angola	Tchicala-Tcholoanga	Proportion	2017	44.5	68.4	26.3
Angola	Tchicala-Tcholoanga	Count	2000	0	1	0
Angola	Tchicala-Tcholoanga	Count	2017	0	1	0
Angola	Kuvango	Proportion	2000	37.6	58.7	19.1
Angola	Kuvango	Proportion	2017	40.0	61.6	23.4
Angola	Kuvango	Count	2000	0	1	0
Angola	Kuvango	Count	2017	0	1	0
Angola	Viana	Proportion	2000	35.1	60.9	16.3
Angola	Viana	Proportion	2017	49.0	71.6	28.2
Angola	Viana	Count	2000	0	1	0
Angola	Viana	Count	2017	0	1	0
Angola	Xá Muteba	Proportion	2000	23.6	41.7	9.6
Angola	Xá Muteba	Proportion	2017	32.6	52.8	16.0
Angola	Xá Muteba	Count	2000	0	0	0
Angola	Xá Muteba	Count	2017	0	1	0
Angola	Malanje	Proportion	2000	28.2	51.2	11.7
Angola	Malanje	Proportion	2017	49.5	71.9	29.1
Angola	Malanje	Count	2000	0	1	0
Angola	Malanje	Count	2017	0	1	0
Angola	Moxico	Proportion	2000	34.4	53.3	18.0
Angola	Moxico	Proportion	2017	42.5	61.9	25.1
Angola	Moxico	Count	2000	0	1	0
Angola	Moxico	Count	2017	0	1	0

Table 4: ORS by Unit *(continued)*

Country	Unit	Metric	Year	Mean	Upper	Lower
Angola	Mucaba	Proportion	2000	29.3	54.2	11.6
Angola	Mucaba	Proportion	2017	45.1	64.9	26.2
Angola	Mucaba	Count	2000	0	1	0
Angola	Mucaba	Count	2017	0	1	0
Angola	Ngonguembo	Proportion	2000	24.6	44.7	10.0
Angola	Ngonguembo	Proportion	2017	40.6	63.2	22.4
Angola	Ngonguembo	Count	2000	0	0	0
Angola	Ngonguembo	Count	2017	0	1	0
Angola	Seles	Proportion	2000	27.5	49.0	10.1
Angola	Seles	Proportion	2017	37.6	59.7	18.5
Angola	Seles	Count	2000	0	0	0
Angola	Seles	Count	2017	0	1	0
Angola	Tchindjenje	Proportion	2000	31.5	58.1	14.1
Angola	Tchindjenje	Proportion	2017	41.2	63.8	21.1
Angola	Tchindjenje	Count	2000	0	1	0
Angola	Tchindjenje	Count	2017	0	1	0
Angola	Lubango	Proportion	2000	43.9	67.6	21.6
Angola	Lubango	Proportion	2017	50.7	71.1	29.8
Angola	Lubango	Count	2000	0	1	0
Angola	Lubango	Count	2017	1	1	0
Angola	Marimba	Proportion	2000	23.4	45.1	8.3
Angola	Marimba	Proportion	2017	34.5	57.0	17.4
Angola	Marimba	Count	2000	0	0	0
Angola	Marimba	Count	2017	0	1	0
Angola	Negage	Proportion	2000	29.1	52.6	12.3
Angola	Negage	Proportion	2017	46.4	66.7	27.4
Angola	Negage	Count	2000	0	1	0
Angola	Negage	Count	2017	0	1	0
Angola	Pango Aluquém	Proportion	2000	26.7	49.5	11.0
Angola	Pango Aluquém	Proportion	2017	40.4	65.3	22.4
Angola	Pango Aluquém	Count	2000	0	0	0
Angola	Pango Aluquém	Count	2017	0	1	0
Angola	Sumbe	Proportion	2000	27.5	51.8	10.8
Angola	Sumbe	Proportion	2017	43.4	65.9	21.7
Angola	Sumbe	Count	2000	0	1	0
Angola	Sumbe	Count	2017	0	1	0
Angola	Ukuma	Proportion	2000	32.2	58.7	14.0
Angola	Ukuma	Proportion	2017	42.8	65.0	23.0
Angola	Ukuma	Count	2000	0	1	0
Angola	Ukuma	Count	2017	0	1	0
Angola	Matala	Proportion	2000	41.0	65.3	20.6
Angola	Matala	Proportion	2017	43.1	65.8	23.3
Angola	Matala	Count	2000	0	1	0
Angola	Matala	Count	2017	0	1	0
Angola	Massango	Proportion	2000	25.0	47.0	8.8
Angola	Massango	Proportion	2017	35.9	58.4	19.0
Angola	Massango	Count	2000	0	0	0
Angola	Massango	Count	2017	0	1	0
Angola	Puri	Proportion	2000	29.2	53.3	11.8

Table 4: ORS by Unit *(continued)*

Country	Unit	Metric	Year	Mean	Upper	Lower
Angola	Puri	Proportion	2017	44.7	66.6	24.9
Angola	Puri	Count	2000	0	1	0
Angola	Puri	Count	2017	0	1	0
Angola	Quiculungo	Proportion	2000	28.1	52.1	11.4
Angola	Quiculungo	Proportion	2017	45.2	66.0	25.2
Angola	Quiculungo	Count	2000	0	1	0
Angola	Quiculungo	Count	2017	0	1	0
Angola	Waku Kungo	Proportion	2000	28.5	47.6	11.9
Angola	Waku Kungo	Proportion	2017	38.7	60.6	17.1
Angola	Waku Kungo	Count	2000	0	0	0
Angola	Waku Kungo	Count	2017	0	1	0
Angola	Quilengues	Proportion	2000	34.0	57.6	17.2
Angola	Quilengues	Proportion	2017	38.3	56.9	20.4
Angola	Quilengues	Count	2000	0	1	0
Angola	Quilengues	Count	2017	0	1	0
Angola	Mucari	Proportion	2000	26.7	48.6	10.5
Angola	Mucari	Proportion	2017	43.1	65.0	24.7
Angola	Mucari	Count	2000	0	0	0
Angola	Mucari	Count	2017	0	1	0
Angola	Quimbele	Proportion	2000	25.1	46.1	10.3
Angola	Quimbele	Proportion	2017	38.9	61.8	19.8
Angola	Quimbele	Count	2000	0	0	0
Angola	Quimbele	Count	2017	0	1	0
Angola	Samba Cajú	Proportion	2000	27.3	49.9	11.7
Angola	Samba Cajú	Proportion	2017	42.8	63.9	24.3
Angola	Samba Cajú	Count	2000	0	0	0
Angola	Samba Cajú	Count	2017	0	1	0
Angola	Tchipungo	Proportion	2000	39.6	63.9	19.5
Angola	Tchipungo	Proportion	2017	39.8	62.1	20.5
Angola	Tchipungo	Count	2000	0	1	0
Angola	Tchipungo	Count	2017	0	1	0
Angola	Quela	Proportion	2000	24.9	46.0	9.2
Angola	Quela	Proportion	2017	38.4	59.3	21.4
Angola	Quela	Count	2000	0	0	0
Angola	Quela	Count	2017	0	1	0
Angola	Quitexe	Proportion	2000	30.6	53.5	12.8
Angola	Quitexe	Proportion	2017	46.2	65.7	27.2
Angola	Quitexe	Count	2000	0	1	0
Angola	Quitexe	Count	2017	0	1	0
Angola	Quirima	Proportion	2000	29.2	48.5	13.2
Angola	Quirima	Proportion	2017	31.8	53.8	15.0
Angola	Quirima	Count	2000	0	0	0
Angola	Quirima	Count	2017	0	1	0
Angola	Sanza Pombo	Proportion	2000	27.8	50.6	11.6
Angola	Sanza Pombo	Proportion	2017	42.0	65.8	23.5
Angola	Sanza Pombo	Count	2000	0	1	0
Angola	Sanza Pombo	Count	2017	0	1	0
Angola	Songo	Proportion	2000	29.5	55.6	12.9
Angola	Songo	Proportion	2017	46.2	64.8	27.0

Table 4: ORS by Unit *(continued)*

Country	Unit	Metric	Year	Mean	Upper	Lower
Angola	Songo	Count	2000	0	1	0
Angola	Songo	Count	2017	0	1	0
Angola	Uíge	Proportion	2000	30.3	56.8	12.9
Angola	Uíge	Proportion	2017	50.8	68.9	31.5
Angola	Uíge	Count	2000	0	1	0
Angola	Uíge	Count	2017	1	1	0
Bangladesh	Barguna	Proportion	2000	55.9	73.5	36.8
Bangladesh	Barguna	Proportion	2017	72.6	86.7	55.8
Bangladesh	Barguna	Count	2000	1	1	0
Bangladesh	Barguna	Count	2017	1	1	1
Bangladesh	Bandarban	Proportion	2000	61.1	76.5	44.7
Bangladesh	Bandarban	Proportion	2017	75.1	87.1	60.3
Bangladesh	Bandarban	Count	2000	1	1	0
Bangladesh	Bandarban	Count	2017	1	1	1
Bangladesh	Dhaka	Proportion	2000	64.7	82.1	45.2
Bangladesh	Dhaka	Proportion	2017	79.1	90.5	63.9
Bangladesh	Dhaka	Count	2000	1	1	0
Bangladesh	Dhaka	Count	2017	1	1	1
Bangladesh	Bagerhat	Proportion	2000	59.7	76.8	38.2
Bangladesh	Bagerhat	Proportion	2017	74.0	85.7	60.4
Bangladesh	Bagerhat	Count	2000	1	1	0
Bangladesh	Bagerhat	Count	2017	1	1	1
Bangladesh	Bogra	Proportion	2000	59.0	77.5	39.9
Bangladesh	Bogra	Proportion	2017	76.1	88.0	59.0
Bangladesh	Bogra	Count	2000	1	1	0
Bangladesh	Bogra	Count	2017	1	1	1
Bangladesh	Dinajpur	Proportion	2000	55.8	74.2	37.2
Bangladesh	Dinajpur	Proportion	2017	74.6	86.8	56.6
Bangladesh	Dinajpur	Count	2000	1	1	0
Bangladesh	Dinajpur	Count	2017	1	1	1
Bangladesh	Habiganj	Proportion	2000	59.6	75.3	41.2
Bangladesh	Habiganj	Proportion	2017	77.1	89.0	60.3
Bangladesh	Habiganj	Count	2000	1	1	0
Bangladesh	Habiganj	Count	2017	1	1	1
Bangladesh	Jamalpur	Proportion	2000	58.3	76.5	39.5
Bangladesh	Jamalpur	Proportion	2017	76.4	87.4	59.6
Bangladesh	Jamalpur	Count	2000	1	1	0
Bangladesh	Jamalpur	Count	2017	1	1	1
Bangladesh	Barisal	Proportion	2000	61.1	77.3	41.7
Bangladesh	Barisal	Proportion	2017	75.3	86.4	58.0
Bangladesh	Barisal	Count	2000	1	1	0
Bangladesh	Barisal	Count	2017	1	1	1
Bangladesh	Brahamanbaria	Proportion	2000	62.2	79.4	41.4
Bangladesh	Brahamanbaria	Proportion	2017	78.7	89.5	64.0
Bangladesh	Brahamanbaria	Count	2000	1	1	0
Bangladesh	Brahamanbaria	Count	2017	1	1	1
Bangladesh	Faridpur	Proportion	2000	59.0	76.0	40.6
Bangladesh	Faridpur	Proportion	2017	75.5	86.9	61.2
Bangladesh	Faridpur	Count	2000	1	1	0

Table 4: ORS by Unit *(continued)*

Country	Unit	Metric	Year	Mean	Upper	Lower
Bangladesh	Faridpur	Count	2017	1	1	1
Bangladesh	Chuadanga	Proportion	2000	58.9	75.3	40.4
Bangladesh	Chuadanga	Proportion	2017	74.0	87.8	56.4
Bangladesh	Chuadanga	Count	2000	1	1	0
Bangladesh	Chuadanga	Count	2017	1	1	1
Bangladesh	Joypurhat	Proportion	2000	57.0	75.3	33.9
Bangladesh	Joypurhat	Proportion	2017	74.4	88.5	53.4
Bangladesh	Joypurhat	Count	2000	1	1	0
Bangladesh	Joypurhat	Count	2017	1	1	1
Bangladesh	Gaibandha	Proportion	2000	58.7	76.8	39.1
Bangladesh	Gaibandha	Proportion	2017	76.4	88.0	61.8
Bangladesh	Gaibandha	Count	2000	1	1	0
Bangladesh	Gaibandha	Count	2017	1	1	1
Bangladesh	Maulvibazar	Proportion	2000	58.9	75.2	43.0
Bangladesh	Maulvibazar	Proportion	2017	75.0	87.8	60.7
Bangladesh	Maulvibazar	Count	2000	1	1	0
Bangladesh	Maulvibazar	Count	2017	1	1	1
Bangladesh	Mymensingh	Proportion	2000	58.2	73.0	39.5
Bangladesh	Mymensingh	Proportion	2017	75.4	87.1	61.1
Bangladesh	Mymensingh	Count	2000	1	1	0
Bangladesh	Mymensingh	Count	2017	1	1	1
Bangladesh	Bhola	Proportion	2000	59.9	78.3	42.7
Bangladesh	Bhola	Proportion	2017	76.1	87.6	61.1
Bangladesh	Bhola	Count	2000	1	1	0
Bangladesh	Bhola	Count	2017	1	1	1
Bangladesh	Chandpur	Proportion	2000	61.3	79.3	42.7
Bangladesh	Chandpur	Proportion	2017	77.5	89.0	61.3
Bangladesh	Chandpur	Count	2000	1	1	0
Bangladesh	Chandpur	Count	2017	1	1	1
Bangladesh	Gazipur	Proportion	2000	62.5	79.7	43.0
Bangladesh	Gazipur	Proportion	2017	76.7	89.3	61.4
Bangladesh	Gazipur	Count	2000	1	1	0
Bangladesh	Gazipur	Count	2017	1	1	1
Bangladesh	Jessore	Proportion	2000	59.7	75.5	39.7
Bangladesh	Jessore	Proportion	2017	74.8	87.1	58.4
Bangladesh	Jessore	Count	2000	1	1	0
Bangladesh	Jessore	Count	2017	1	1	1
Bangladesh	Naogaon	Proportion	2000	54.7	70.7	33.9
Bangladesh	Naogaon	Proportion	2017	71.4	84.6	54.9
Bangladesh	Naogaon	Count	2000	1	1	0
Bangladesh	Naogaon	Count	2017	1	1	1
Bangladesh	Kurigram	Proportion	2000	56.5	72.8	36.0
Bangladesh	Kurigram	Proportion	2017	75.4	87.7	59.3
Bangladesh	Kurigram	Count	2000	1	1	0
Bangladesh	Kurigram	Count	2017	1	1	1
Bangladesh	Sunamganj	Proportion	2000	60.6	77.5	39.8
Bangladesh	Sunamganj	Proportion	2017	77.4	88.2	64.8
Bangladesh	Sunamganj	Count	2000	1	1	0
Bangladesh	Sunamganj	Count	2017	1	1	1

Table 4: ORS by Unit *(continued)*

Country	Unit	Metric	Year	Mean	Upper	Lower
Bangladesh	Netrakona	Proportion	2000	58.3	73.6	39.3
Bangladesh	Netrakona	Proportion	2017	75.6	86.8	59.8
Bangladesh	Netrakona	Count	2000	1	1	0
Bangladesh	Netrakona	Count	2017	1	1	1
Bangladesh	Jhalokati	Proportion	2000	59.8	75.8	37.5
Bangladesh	Jhalokati	Proportion	2017	74.7	86.1	57.2
Bangladesh	Jhalokati	Count	2000	1	1	0
Bangladesh	Jhalokati	Count	2017	1	1	1
Bangladesh	Chittagong	Proportion	2000	65.6	81.3	47.3
Bangladesh	Chittagong	Proportion	2017	78.4	89.7	64.4
Bangladesh	Chittagong	Count	2000	1	1	0
Bangladesh	Chittagong	Count	2017	1	1	1
Bangladesh	Gopalganj	Proportion	2000	59.0	76.6	40.3
Bangladesh	Gopalganj	Proportion	2017	74.3	86.2	58.4
Bangladesh	Gopalganj	Count	2000	1	1	0
Bangladesh	Gopalganj	Count	2017	1	1	1
Bangladesh	Jhenaidah	Proportion	2000	59.0	75.5	41.9
Bangladesh	Jhenaidah	Proportion	2017	73.4	85.7	58.7
Bangladesh	Jhenaidah	Count	2000	1	1	0
Bangladesh	Jhenaidah	Count	2017	1	1	1
Bangladesh	Natore	Proportion	2000	57.4	77.8	38.0
Bangladesh	Natore	Proportion	2017	73.5	86.9	56.5
Bangladesh	Natore	Count	2000	1	1	0
Bangladesh	Natore	Count	2017	1	1	1
Bangladesh	Lalmonirhat	Proportion	2000	56.7	74.9	38.4
Bangladesh	Lalmonirhat	Proportion	2017	76.0	88.3	61.4
Bangladesh	Lalmonirhat	Count	2000	1	1	0
Bangladesh	Lalmonirhat	Count	2017	1	1	1
Bangladesh	Sylhet	Proportion	2000	63.2	79.2	44.9
Bangladesh	Sylhet	Proportion	2017	78.4	88.4	63.6
Bangladesh	Sylhet	Count	2000	1	1	0
Bangladesh	Sylhet	Count	2017	1	1	1
Bangladesh	Sherpur	Proportion	2000	57.1	77.7	38.9
Bangladesh	Sherpur	Proportion	2017	76.0	88.6	59.0
Bangladesh	Sherpur	Count	2000	1	1	0
Bangladesh	Sherpur	Count	2017	1	1	1
Bangladesh	Patuakhali	Proportion	2000	57.1	72.8	39.4
Bangladesh	Patuakhali	Proportion	2017	73.8	86.4	59.0
Bangladesh	Patuakhali	Count	2000	1	1	0
Bangladesh	Patuakhali	Count	2017	1	1	1
Bangladesh	Comilla	Proportion	2000	63.0	79.1	44.6
Bangladesh	Comilla	Proportion	2017	78.2	89.4	63.9
Bangladesh	Comilla	Count	2000	1	1	0
Bangladesh	Comilla	Count	2017	1	1	1
Bangladesh	Kishoreganj	Proportion	2000	61.3	77.0	43.7
Bangladesh	Kishoreganj	Proportion	2017	77.4	88.6	60.5
Bangladesh	Kishoreganj	Count	2000	1	1	0
Bangladesh	Kishoreganj	Count	2017	1	1	1
Bangladesh	Khulna	Proportion	2000	59.8	76.6	39.0

Table 4: ORS by Unit *(continued)*

Country	Unit	Metric	Year	Mean	Upper	Lower
Bangladesh	Khulna	Proportion	2017	74.5	87.4	54.4
Bangladesh	Khulna	Count	2000	1	1	0
Bangladesh	Khulna	Count	2017	1	1	1
Bangladesh	Nawabganj	Proportion	2000	53.6	71.5	32.8
Bangladesh	Nawabganj	Proportion	2017	69.9	82.5	50.8
Bangladesh	Nawabganj	Count	2000	1	1	0
Bangladesh	Nawabganj	Count	2017	1	1	1
Bangladesh	Nilphamari	Proportion	2000	56.7	74.9	35.7
Bangladesh	Nilphamari	Proportion	2017	76.2	88.9	58.9
Bangladesh	Nilphamari	Count	2000	1	1	0
Bangladesh	Nilphamari	Count	2017	1	1	1
Bangladesh	Pirojpur	Proportion	2000	58.4	74.7	37.5
Bangladesh	Pirojpur	Proportion	2017	73.2	84.4	58.1
Bangladesh	Pirojpur	Count	2000	1	1	0
Bangladesh	Pirojpur	Count	2017	1	1	1
Bangladesh	Cox'S Bazar	Proportion	2000	62.2	77.8	45.7
Bangladesh	Cox'S Bazar	Proportion	2017	76.4	87.2	62.2
Bangladesh	Cox'S Bazar	Count	2000	1	1	0
Bangladesh	Cox'S Bazar	Count	2017	1	1	1
Bangladesh	Madaripur	Proportion	2000	59.1	76.6	40.4
Bangladesh	Madaripur	Proportion	2017	74.8	87.4	58.3
Bangladesh	Madaripur	Count	2000	1	1	0
Bangladesh	Madaripur	Count	2017	1	1	1
Bangladesh	Kushtia	Proportion	2000	61.9	78.3	45.5
Bangladesh	Kushtia	Proportion	2017	76.8	88.2	59.7
Bangladesh	Kushtia	Count	2000	1	1	0
Bangladesh	Kushtia	Count	2017	1	1	1
Bangladesh	Pabna	Proportion	2000	59.1	76.8	39.9
Bangladesh	Pabna	Proportion	2017	75.2	87.6	59.8
Bangladesh	Pabna	Count	2000	1	1	0
Bangladesh	Pabna	Count	2017	1	1	1
Bangladesh	Panchagarh	Proportion	2000	57.0	77.0	33.4
Bangladesh	Panchagarh	Proportion	2017	75.2	89.5	59.6
Bangladesh	Panchagarh	Count	2000	1	1	0
Bangladesh	Panchagarh	Count	2017	1	1	1
Bangladesh	Feni	Proportion	2000	61.8	77.7	41.1
Bangladesh	Feni	Proportion	2017	76.1	88.0	56.4
Bangladesh	Feni	Count	2000	1	1	0
Bangladesh	Feni	Count	2017	1	1	1
Bangladesh	Manikganj	Proportion	2000	58.0	77.6	38.0
Bangladesh	Manikganj	Proportion	2017	74.5	86.6	60.4
Bangladesh	Manikganj	Count	2000	1	1	0
Bangladesh	Manikganj	Count	2017	1	1	1
Bangladesh	Magura	Proportion	2000	60.5	78.7	41.2
Bangladesh	Magura	Proportion	2017	74.8	86.8	60.0
Bangladesh	Magura	Count	2000	1	1	0
Bangladesh	Magura	Count	2017	1	1	1
Bangladesh	Rajshahi	Proportion	2000	57.8	75.8	37.1
Bangladesh	Rajshahi	Proportion	2017	73.3	85.3	57.0

Table 4: ORS by Unit *(continued)*

Country	Unit	Metric	Year	Mean	Upper	Lower
Bangladesh	Rajshahi	Count	2000	1	1	0
Bangladesh	Rajshahi	Count	2017	1	1	1
Bangladesh	Rangpur	Proportion	2000	58.6	77.1	38.7
Bangladesh	Rangpur	Proportion	2017	76.9	88.3	60.3
Bangladesh	Rangpur	Count	2000	1	1	0
Bangladesh	Rangpur	Count	2017	1	1	1
Bangladesh	Khagrachhari	Proportion	2000	60.6	76.2	41.3
Bangladesh	Khagrachhari	Proportion	2017	74.1	87.4	57.7
Bangladesh	Khagrachhari	Count	2000	1	1	0
Bangladesh	Khagrachhari	Count	2017	1	1	1
Bangladesh	Munshiganj	Proportion	2000	59.4	78.9	40.2
Bangladesh	Munshiganj	Proportion	2017	76.6	88.4	61.5
Bangladesh	Munshiganj	Count	2000	1	1	0
Bangladesh	Munshiganj	Count	2017	1	1	1
Bangladesh	Meherpur	Proportion	2000	56.0	76.4	36.0
Bangladesh	Meherpur	Proportion	2017	72.5	87.9	47.8
Bangladesh	Meherpur	Count	2000	1	1	0
Bangladesh	Meherpur	Count	2017	1	1	0
Bangladesh	Sirajganj	Proportion	2000	59.1	76.5	39.4
Bangladesh	Sirajganj	Proportion	2017	76.2	87.9	62.1
Bangladesh	Sirajganj	Count	2000	1	1	0
Bangladesh	Sirajganj	Count	2017	1	1	1
Bangladesh	Thakurgaon	Proportion	2000	55.9	75.6	34.1
Bangladesh	Thakurgaon	Proportion	2017	74.8	88.4	57.6
Bangladesh	Thakurgaon	Count	2000	1	1	0
Bangladesh	Thakurgaon	Count	2017	1	1	1
Bangladesh	Lakshmipur	Proportion	2000	62.3	78.8	44.0
Bangladesh	Lakshmipur	Proportion	2017	77.1	88.3	60.6
Bangladesh	Lakshmipur	Count	2000	1	1	0
Bangladesh	Lakshmipur	Count	2017	1	1	1
Bangladesh	Narayanganj	Proportion	2000	62.8	80.7	41.2
Bangladesh	Narayanganj	Proportion	2017	78.1	90.2	61.4
Bangladesh	Narayanganj	Count	2000	1	1	0
Bangladesh	Narayanganj	Count	2017	1	1	1
Bangladesh	Narail	Proportion	2000	59.6	75.4	38.8
Bangladesh	Narail	Proportion	2017	74.2	87.4	57.7
Bangladesh	Narail	Count	2000	1	1	0
Bangladesh	Narail	Count	2017	1	1	1
Bangladesh	Noakhali	Proportion	2000	60.0	78.7	40.3
Bangladesh	Noakhali	Proportion	2017	74.5	87.7	58.1
Bangladesh	Noakhali	Count	2000	1	1	0
Bangladesh	Noakhali	Count	2017	1	1	1
Bangladesh	Narsingdi	Proportion	2000	62.5	79.2	43.7
Bangladesh	Narsingdi	Proportion	2017	77.4	88.7	63.5
Bangladesh	Narsingdi	Count	2000	1	1	0
Bangladesh	Narsingdi	Count	2017	1	1	1
Bangladesh	Satkhira	Proportion	2000	57.6	73.3	37.2
Bangladesh	Satkhira	Proportion	2017	73.4	86.0	56.8
Bangladesh	Satkhira	Count	2000	1	1	0

Table 4: ORS by Unit *(continued)*

Country	Unit	Metric	Year	Mean	Upper	Lower
Bangladesh	Satkhira	Count	2017	1	1	1
Bangladesh	Rangamati	Proportion	2000	61.4	76.9	44.6
Bangladesh	Rangamati	Proportion	2017	75.4	86.6	59.9
Bangladesh	Rangamati	Count	2000	1	1	0
Bangladesh	Rangamati	Count	2017	1	1	1
Bangladesh	Rajbari	Proportion	2000	58.2	77.7	37.9
Bangladesh	Rajbari	Proportion	2017	74.3	87.2	56.5
Bangladesh	Rajbari	Count	2000	1	1	0
Bangladesh	Rajbari	Count	2017	1	1	1
Bangladesh	Shariatpur	Proportion	2000	58.9	75.8	39.7
Bangladesh	Shariatpur	Proportion	2017	74.6	86.6	56.6
Bangladesh	Shariatpur	Count	2000	1	1	0
Bangladesh	Shariatpur	Count	2017	1	1	1
Bangladesh	Tangail	Proportion	2000	59.1	78.3	37.6
Bangladesh	Tangail	Proportion	2017	75.5	87.1	60.2
Bangladesh	Tangail	Count	2000	1	1	0
Bangladesh	Tangail	Count	2017	1	1	1
Belize	Belize	Proportion	2000	50.5	70.9	31.1
Belize	Belize	Proportion	2017	51.2	67.5	35.8
Belize	Belize	Count	2000	1	1	0
Belize	Belize	Count	2017	1	1	0
Belize	Cayo	Proportion	2000	52.6	71.9	33.9
Belize	Cayo	Proportion	2017	52.2	67.9	35.0
Belize	Cayo	Count	2000	1	1	0
Belize	Cayo	Count	2017	1	1	0
Belize	Corozal	Proportion	2000	53.8	73.5	31.8
Belize	Corozal	Proportion	2017	54.8	70.4	39.8
Belize	Corozal	Count	2000	1	1	0
Belize	Corozal	Count	2017	1	1	0
Belize	Orange Walk	Proportion	2000	52.1	72.1	32.6
Belize	Orange Walk	Proportion	2017	52.5	67.7	37.4
Belize	Orange Walk	Count	2000	1	1	0
Belize	Orange Walk	Count	2017	1	1	0
Belize	Stann Creek	Proportion	2000	51.5	71.4	30.7
Belize	Stann Creek	Proportion	2017	50.6	67.7	32.1
Belize	Stann Creek	Count	2000	1	1	0
Belize	Stann Creek	Count	2017	1	1	0
Belize	Toledo	Proportion	2000	50.9	71.9	32.2
Belize	Toledo	Proportion	2017	49.1	65.8	31.6
Belize	Toledo	Count	2000	1	1	0
Belize	Toledo	Count	2017	0	1	0
Benin	Banikoara	Proportion	2000	20.4	34.1	10.4
Benin	Banikoara	Proportion	2017	19.9	30.1	11.6
Benin	Banikoara	Count	2000	0	0	0
Benin	Banikoara	Count	2017	0	0	0
Benin	Boukoumbé	Proportion	2000	18.8	32.6	9.7
Benin	Boukoumbé	Proportion	2017	24.4	36.0	14.1
Benin	Boukoumbé	Count	2000	0	0	0
Benin	Boukoumbé	Count	2017	0	0	0

Table 4: ORS by Unit *(continued)*

Country	Unit	Metric	Year	Mean	Upper	Lower
Benin	Abomey-Calavi	Proportion	2000	22.3	37.7	10.2
Benin	Abomey-Calavi	Proportion	2017	23.9	35.4	14.4
Benin	Abomey-Calavi	Count	2000	0	0	0
Benin	Abomey-Calavi	Count	2017	0	0	0
Benin	Bembéréké	Proportion	2000	18.0	32.4	8.8
Benin	Bembéréké	Proportion	2017	25.4	38.1	14.4
Benin	Bembéréké	Count	2000	0	0	0
Benin	Bembéréké	Count	2017	0	0	0
Benin	Bantè	Proportion	2000	21.1	38.8	10.7
Benin	Bantè	Proportion	2017	34.5	48.9	20.2
Benin	Bantè	Count	2000	0	0	0
Benin	Bantè	Count	2017	0	0	0
Benin	Bassila	Proportion	2000	19.5	32.4	9.7
Benin	Bassila	Proportion	2017	32.9	46.1	20.4
Benin	Bassila	Count	2000	0	0	0
Benin	Bassila	Count	2017	0	0	0
Benin	Aplahoué	Proportion	2000	17.8	34.1	9.5
Benin	Aplahoué	Proportion	2017	29.0	43.5	17.6
Benin	Aplahoué	Count	2000	0	0	0
Benin	Aplahoué	Count	2017	0	0	0
Benin	Cotonou	Proportion	2000	25.1	42.6	11.7
Benin	Cotonou	Proportion	2017	25.8	39.2	15.6
Benin	Cotonou	Count	2000	0	0	0
Benin	Cotonou	Count	2017	0	0	0
Benin	Athiémé	Proportion	2000	17.5	31.4	8.6
Benin	Athiémé	Proportion	2017	25.4	38.5	14.6
Benin	Athiémé	Count	2000	0	0	0
Benin	Athiémé	Count	2017	0	0	0
Benin	Adjarra	Proportion	2000	25.9	44.5	12.1
Benin	Adjarra	Proportion	2017	24.1	36.9	13.9
Benin	Adjarra	Count	2000	0	0	0
Benin	Adjarra	Count	2017	0	0	0
Benin	Adja-Ouèrè	Proportion	2000	24.1	42.4	11.4
Benin	Adja-Ouèrè	Proportion	2017	24.9	36.3	15.5
Benin	Adja-Ouèrè	Count	2000	0	0	0
Benin	Adja-Ouèrè	Count	2017	0	0	0
Benin	Abomey	Proportion	2000	19.5	37.6	9.5
Benin	Abomey	Proportion	2017	30.1	45.7	19.0
Benin	Abomey	Count	2000	0	0	0
Benin	Abomey	Count	2017	0	0	0
Benin	Gogounou	Proportion	2000	20.9	34.3	10.5
Benin	Gogounou	Proportion	2017	22.9	35.6	13.1
Benin	Gogounou	Count	2000	0	0	0
Benin	Gogounou	Count	2017	0	0	0
Benin	Cobly	Proportion	2000	20.7	35.1	10.7
Benin	Cobly	Proportion	2017	24.8	38.1	14.5
Benin	Cobly	Count	2000	0	0	0
Benin	Cobly	Count	2017	0	0	0
Benin	Allada	Proportion	2000	19.8	35.2	10.2

Table 4: ORS by Unit *(continued)*

Country	Unit	Metric	Year	Mean	Upper	Lower
Benin	Allada	Proportion	2017	23.9	36.1	14.8
Benin	Allada	Count	2000	0	0	0
Benin	Allada	Count	2017	0	0	0
Benin	Kalalé	Proportion	2000	21.6	36.7	10.0
Benin	Kalalé	Proportion	2017	25.9	40.6	14.2
Benin	Kalalé	Count	2000	0	0	0
Benin	Kalalé	Count	2017	0	0	0
Benin	Dassa-Zoumè	Proportion	2000	24.6	45.0	12.6
Benin	Dassa-Zoumè	Proportion	2017	38.8	54.8	25.2
Benin	Dassa-Zoumè	Count	2000	0	0	0
Benin	Dassa-Zoumè	Count	2017	0	1	0
Benin	Copargo	Proportion	2000	18.2	32.3	9.4
Benin	Copargo	Proportion	2017	27.5	39.2	16.9
Benin	Copargo	Count	2000	0	0	0
Benin	Copargo	Count	2017	0	0	0
Benin	Djakotomey	Proportion	2000	18.7	34.4	9.7
Benin	Djakotomey	Proportion	2017	27.9	42.3	16.9
Benin	Djakotomey	Count	2000	0	0	0
Benin	Djakotomey	Count	2017	0	0	0
Benin	Bopa	Proportion	2000	17.9	32.7	8.7
Benin	Bopa	Proportion	2017	24.4	37.0	14.8
Benin	Bopa	Count	2000	0	0	0
Benin	Bopa	Count	2017	0	0	0
Benin	Adjohoun	Proportion	2000	22.4	37.8	11.2
Benin	Adjohoun	Proportion	2017	22.2	32.5	13.7
Benin	Adjohoun	Count	2000	0	0	0
Benin	Adjohoun	Count	2017	0	0	0
Benin	Ifangni	Proportion	2000	25.4	42.8	12.7
Benin	Ifangni	Proportion	2017	23.4	34.1	14.5
Benin	Ifangni	Count	2000	0	0	0
Benin	Ifangni	Count	2017	0	0	0
Benin	Agbangnizoun	Proportion	2000	19.5	37.8	9.3
Benin	Agbangnizoun	Proportion	2017	29.6	44.6	18.6
Benin	Agbangnizoun	Count	2000	0	0	0
Benin	Agbangnizoun	Count	2017	0	0	0
Benin	Kandi	Proportion	2000	23.0	36.8	11.2
Benin	Kandi	Proportion	2017	23.5	36.3	13.4
Benin	Kandi	Count	2000	0	0	0
Benin	Kandi	Count	2017	0	0	0
Benin	Kérou	Proportion	2000	18.9	31.5	9.1
Benin	Kérou	Proportion	2017	20.3	31.1	11.7
Benin	Kérou	Count	2000	0	0	0
Benin	Kérou	Count	2017	0	0	0
Benin	Kpomassè	Proportion	2000	19.4	35.1	9.5
Benin	Kpomassè	Proportion	2017	24.7	36.5	14.6
Benin	Kpomassè	Count	2000	0	0	0
Benin	Kpomassè	Count	2017	0	0	0
Benin	N'Dali	Proportion	2000	18.7	32.6	9.2
Benin	N'Dali	Proportion	2017	30.6	45.0	19.2

Table 4: ORS by Unit *(continued)*

Country	Unit	Metric	Year	Mean	Upper	Lower
Benin	N'Dali	Count	2000	0	0	0
Benin	N'Dali	Count	2017	0	0	0
Benin	Glazoué	Proportion	2000	24.0	42.6	11.4
Benin	Glazoué	Proportion	2017	40.0	55.7	25.8
Benin	Glazoué	Count	2000	0	0	0
Benin	Glazoué	Count	2017	0	1	0
Benin	Djougou	Proportion	2000	19.0	34.6	10.0
Benin	Djougou	Proportion	2017	31.8	44.5	20.9
Benin	Djougou	Count	2000	0	0	0
Benin	Djougou	Count	2017	0	0	0
Benin	Dogbo	Proportion	2000	18.1	32.7	9.0
Benin	Dogbo	Proportion	2017	27.1	41.4	16.3
Benin	Dogbo	Count	2000	0	0	0
Benin	Dogbo	Count	2017	0	0	0
Benin	Comè	Proportion	2000	18.7	33.2	9.3
Benin	Comè	Proportion	2017	24.8	37.2	14.6
Benin	Comè	Count	2000	0	0	0
Benin	Comè	Count	2017	0	0	0
Benin	Aguégués	Proportion	2000	24.5	42.0	11.4
Benin	Aguégués	Proportion	2017	23.2	34.8	13.4
Benin	Aguégués	Count	2000	0	0	0
Benin	Aguégués	Count	2017	0	0	0
Benin	Kétou	Proportion	2000	25.4	46.1	11.6
Benin	Kétou	Proportion	2017	31.3	45.1	18.8
Benin	Kétou	Count	2000	0	0	0
Benin	Kétou	Count	2017	0	0	0
Benin	Bohicon	Proportion	2000	22.0	40.1	11.2
Benin	Bohicon	Proportion	2017	32.3	48.4	21.1
Benin	Bohicon	Count	2000	0	0	0
Benin	Bohicon	Count	2017	0	0	0
Benin	Karimama	Proportion	2000	21.5	34.1	11.5
Benin	Karimama	Proportion	2017	22.5	33.8	13.8
Benin	Karimama	Count	2000	0	0	0
Benin	Karimama	Count	2017	0	0	0
Benin	Kouandé	Proportion	2000	18.5	34.4	9.5
Benin	Kouandé	Proportion	2017	23.5	34.6	15.1
Benin	Kouandé	Count	2000	0	0	0
Benin	Kouandé	Count	2017	0	0	0
Benin	Ouidah	Proportion	2000	20.8	35.1	9.9
Benin	Ouidah	Proportion	2017	25.0	36.3	15.1
Benin	Ouidah	Count	2000	0	0	0
Benin	Ouidah	Count	2017	0	0	0
Benin	Nikki	Proportion	2000	20.0	37.8	8.6
Benin	Nikki	Proportion	2017	27.5	41.6	15.6
Benin	Nikki	Count	2000	0	0	0
Benin	Nikki	Count	2017	0	0	0
Benin	Ouèssè	Proportion	2000	22.7	41.2	11.6
Benin	Ouèssè	Proportion	2017	39.0	54.4	25.4
Benin	Ouèssè	Count	2000	0	0	0

Table 4: ORS by Unit *(continued)*

Country	Unit	Metric	Year	Mean	Upper	Lower
Benin	Ouèssè	Count	2017	0	1	0
Benin	Ouaké	Proportion	2000	18.0	33.5	9.8
Benin	Ouaké	Proportion	2017	29.7	43.6	17.0
Benin	Ouaké	Count	2000	0	0	0
Benin	Ouaké	Count	2017	0	0	0
Benin	Klouékanmè	Proportion	2000	18.2	34.9	9.0
Benin	Klouékanmè	Proportion	2017	28.8	42.6	18.1
Benin	Klouékanmè	Count	2000	0	0	0
Benin	Klouékanmè	Count	2017	0	0	0
Benin	Grand-Popo	Proportion	2000	17.6	29.6	9.3
Benin	Grand-Popo	Proportion	2017	24.0	36.5	13.7
Benin	Grand-Popo	Count	2000	0	0	0
Benin	Grand-Popo	Count	2017	0	0	0
Benin	Akpro-Missérété	Proportion	2000	25.5	42.0	12.1
Benin	Akpro-Missérété	Proportion	2017	23.2	34.1	13.1
Benin	Akpro-Missérété	Count	2000	0	0	0
Benin	Akpro-Missérété	Count	2017	0	0	0
Benin	Pobè	Proportion	2000	24.7	42.6	11.1
Benin	Pobè	Proportion	2017	25.7	38.0	15.2
Benin	Pobè	Count	2000	0	0	0
Benin	Pobè	Count	2017	0	0	0
Benin	Covè	Proportion	2000	23.1	41.4	11.5
Benin	Covè	Proportion	2017	31.5	44.9	19.9
Benin	Covè	Count	2000	0	0	0
Benin	Covè	Count	2017	0	0	0
Benin	Malanville	Proportion	2000	25.5	38.6	13.9
Benin	Malanville	Proportion	2017	30.2	43.9	19.1
Benin	Malanville	Count	2000	0	0	0
Benin	Malanville	Count	2017	0	0	0
Benin	Matéri	Proportion	2000	22.5	38.1	11.8
Benin	Matéri	Proportion	2017	22.0	34.2	13.1
Benin	Matéri	Count	2000	0	0	0
Benin	Matéri	Count	2017	0	0	0
Benin	Sô-Ava	Proportion	2000	24.2	42.9	11.2
Benin	Sô-Ava	Proportion	2017	24.4	36.2	14.9
Benin	Sô-Ava	Count	2000	0	0	0
Benin	Sô-Ava	Count	2017	0	0	0
Benin	Parakou	Proportion	2000	20.5	36.0	9.5
Benin	Parakou	Proportion	2017	34.4	49.8	21.7
Benin	Parakou	Count	2000	0	0	0
Benin	Parakou	Count	2017	0	0	0
Benin	Savalou	Proportion	2000	22.2	40.5	11.3
Benin	Savalou	Proportion	2017	35.6	49.8	22.5
Benin	Savalou	Count	2000	0	0	0
Benin	Savalou	Count	2017	0	0	0
Benin	Lalo	Proportion	2000	18.8	36.6	9.5
Benin	Lalo	Proportion	2017	26.9	41.0	16.5
Benin	Lalo	Count	2000	0	0	0
Benin	Lalo	Count	2017	0	0	0

Table 4: ORS by Unit *(continued)*

Country	Unit	Metric	Year	Mean	Upper	Lower
Benin	Houéyogbé	Proportion	2000	17.5	30.5	8.1
Benin	Houéyogbé	Proportion	2017	24.8	37.9	14.4
Benin	Houéyogbé	Count	2000	0	0	0
Benin	Houéyogbé	Count	2017	0	0	0
Benin	Avrankou	Proportion	2000	25.3	42.2	11.7
Benin	Avrankou	Proportion	2017	23.6	35.4	13.8
Benin	Avrankou	Count	2000	0	0	0
Benin	Avrankou	Count	2017	0	0	0
Benin	Sakété	Proportion	2000	24.1	40.1	11.5
Benin	Sakété	Proportion	2017	22.6	32.8	13.9
Benin	Sakété	Count	2000	0	0	0
Benin	Sakété	Count	2017	0	0	0
Benin	Djidja	Proportion	2000	20.3	38.1	10.4
Benin	Djidja	Proportion	2017	33.1	48.7	20.3
Benin	Djidja	Count	2000	0	0	0
Benin	Djidja	Count	2017	0	0	0
Benin	Segbana	Proportion	2000	24.4	40.4	11.8
Benin	Segbana	Proportion	2017	28.0	42.2	15.8
Benin	Segbana	Count	2000	0	0	0
Benin	Segbana	Count	2017	0	0	0
Benin	Natitingou	Proportion	2000	19.2	34.2	9.6
Benin	Natitingou	Proportion	2017	24.3	36.1	14.5
Benin	Natitingou	Count	2000	0	0	0
Benin	Natitingou	Count	2017	0	0	0
Benin	Toffo	Proportion	2000	19.8	35.1	9.6
Benin	Toffo	Proportion	2017	23.9	36.0	14.5
Benin	Toffo	Count	2000	0	0	0
Benin	Toffo	Count	2017	0	0	0
Benin	Pèrèrè	Proportion	2000	19.5	35.6	8.7
Benin	Pèrèrè	Proportion	2017	29.5	43.0	17.7
Benin	Pèrèrè	Count	2000	0	0	0
Benin	Pèrèrè	Count	2017	0	0	0
Benin	Savè	Proportion	2000	25.1	42.8	12.2
Benin	Savè	Proportion	2017	40.7	57.2	26.3
Benin	Savè	Count	2000	0	0	0
Benin	Savè	Count	2017	0	1	0
Benin	Toviklin	Proportion	2000	18.8	35.9	9.3
Benin	Toviklin	Proportion	2017	28.1	42.5	17.4
Benin	Toviklin	Count	2000	0	0	0
Benin	Toviklin	Count	2017	0	0	0
Benin	Bonou	Proportion	2000	22.6	39.1	11.1
Benin	Bonou	Proportion	2017	23.2	34.1	14.1
Benin	Bonou	Count	2000	0	0	0
Benin	Bonou	Count	2017	0	0	0
Benin	Ouinhi	Proportion	2000	23.7	43.5	12.1
Benin	Ouinhi	Proportion	2017	26.7	39.4	16.3
Benin	Ouinhi	Count	2000	0	0	0
Benin	Ouinhi	Count	2017	0	0	0
Benin	Péhunco	Proportion	2000	18.2	33.2	8.3

Table 4: ORS by Unit *(continued)*

Country	Unit	Metric	Year	Mean	Upper	Lower
Benin	Péhunco	Proportion	2017	26.0	38.4	15.6
Benin	Péhunco	Count	2000	0	0	0
Benin	Péhunco	Count	2017	0	0	0
Benin	Tori-Bossito	Proportion	2000	19.8	35.1	9.8
Benin	Tori-Bossito	Proportion	2017	23.6	35.1	14.3
Benin	Tori-Bossito	Count	2000	0	0	0
Benin	Tori-Bossito	Count	2017	0	0	0
Benin	Sinendé	Proportion	2000	17.4	31.3	8.0
Benin	Sinendé	Proportion	2017	25.9	38.4	14.8
Benin	Sinendé	Count	2000	0	0	0
Benin	Sinendé	Count	2017	0	0	0
Benin	Dangbo	Proportion	2000	23.5	38.7	11.1
Benin	Dangbo	Proportion	2017	22.6	33.8	13.6
Benin	Dangbo	Count	2000	0	0	0
Benin	Dangbo	Count	2017	0	0	0
Benin	Za-Kpota	Proportion	2000	22.0	38.8	11.2
Benin	Za-Kpota	Proportion	2017	32.5	47.7	20.9
Benin	Za-Kpota	Count	2000	0	0	0
Benin	Za-Kpota	Count	2017	0	0	0
Benin	Tanguiéta	Proportion	2000	20.3	34.7	10.3
Benin	Tanguiéta	Proportion	2017	20.3	29.8	12.1
Benin	Tanguiéta	Count	2000	0	0	0
Benin	Tanguiéta	Count	2017	0	0	0
Benin	Zè	Proportion	2000	21.1	35.9	10.3
Benin	Zè	Proportion	2017	22.8	33.4	13.9
Benin	Zè	Count	2000	0	0	0
Benin	Zè	Count	2017	0	0	0
Benin	Tchaourou	Proportion	2000	20.4	35.3	9.7
Benin	Tchaourou	Proportion	2017	34.7	48.9	23.2
Benin	Tchaourou	Count	2000	0	0	0
Benin	Tchaourou	Count	2017	0	0	0
Benin	Porto-Novo	Proportion	2000	26.1	45.1	12.3
Benin	Porto-Novo	Proportion	2017	23.8	35.2	13.5
Benin	Porto-Novo	Count	2000	0	0	0
Benin	Porto-Novo	Count	2017	0	0	0
Benin	Zagnanado	Proportion	2000	23.8	43.0	11.7
Benin	Zagnanado	Proportion	2017	29.7	42.3	18.8
Benin	Zagnanado	Count	2000	0	0	0
Benin	Zagnanado	Count	2017	0	0	0
Benin	Toucountouna	Proportion	2000	19.1	32.6	9.5
Benin	Toucountouna	Proportion	2017	21.4	31.7	13.0
Benin	Toucountouna	Count	2000	0	0	0
Benin	Toucountouna	Count	2017	0	0	0
Benin	Sèmè-Kpodji	Proportion	2000	25.2	43.6	12.1
Benin	Sèmè-Kpodji	Proportion	2017	23.3	34.9	13.1
Benin	Sèmè-Kpodji	Count	2000	0	0	0
Benin	Sèmè-Kpodji	Count	2017	0	0	0
Benin	Zogbodomey	Proportion	2000	20.2	37.5	10.3
Benin	Zogbodomey	Proportion	2017	27.6	41.9	17.6

Table 4: ORS by Unit *(continued)*

Country	Unit	Metric	Year	Mean	Upper	Lower
Benin	Zogbodomey	Count	2000	0	0	0
Benin	Zogbodomey	Count	2017	0	0	0
Bhutan	Chhoekhor	Proportion	2000	43.7	64.8	23.6
Bhutan	Chhoekhor	Proportion	2017	57.8	76.9	32.5
Bhutan	Chhoekhor	Count	2000	0	1	0
Bhutan	Chhoekhor	Count	2017	1	1	0
Bhutan	Bjachho	Proportion	2000	48.3	73.7	24.9
Bhutan	Bjachho	Proportion	2017	64.4	84.5	37.9
Bhutan	Bjachho	Count	2000	0	1	0
Bhutan	Bjachho	Count	2017	1	1	0
Bhutan	Deorali	Proportion	2000	42.4	67.3	20.1
Bhutan	Deorali	Proportion	2017	62.4	83.0	35.9
Bhutan	Deorali	Count	2000	0	1	0
Bhutan	Deorali	Count	2017	1	1	0
Bhutan	Goenkhame	Proportion	2000	46.8	71.8	21.3
Bhutan	Goenkhame	Proportion	2017	59.9	81.6	35.9
Bhutan	Goenkhame	Count	2000	0	1	0
Bhutan	Goenkhame	Count	2017	1	1	0
Bhutan	Bji	Proportion	2000	51.4	71.6	31.7
Bhutan	Bji	Proportion	2017	55.6	74.8	32.1
Bhutan	Bji	Count	2000	1	1	0
Bhutan	Bji	Count	2017	1	1	0
Bhutan	Gangzur	Proportion	2000	48.6	72.4	23.1
Bhutan	Gangzur	Proportion	2017	63.6	83.3	36.4
Bhutan	Gangzur	Count	2000	0	1	0
Bhutan	Gangzur	Count	2017	1	1	0
Bhutan	Balam	Proportion	2000	45.6	69.6	24.4
Bhutan	Balam	Proportion	2017	61.4	80.8	38.3
Bhutan	Balam	Count	2000	0	1	0
Bhutan	Balam	Count	2017	1	1	0
Bhutan	Doga	Proportion	2000	47.4	69.1	24.7
Bhutan	Doga	Proportion	2017	62.1	81.1	38.8
Bhutan	Doga	Count	2000	0	1	0
Bhutan	Doga	Count	2017	1	1	0
Bhutan	Chhimung	Proportion	2000	48.5	75.5	23.8
Bhutan	Chhimung	Proportion	2017	64.5	83.0	40.4
Bhutan	Chhimung	Count	2000	0	1	0
Bhutan	Chhimung	Count	2017	1	1	0
Bhutan	Bapisa	Proportion	2000	45.1	69.1	19.1
Bhutan	Bapisa	Proportion	2017	59.8	80.3	32.2
Bhutan	Bapisa	Count	2000	0	1	0
Bhutan	Bapisa	Count	2017	1	1	0
Bhutan	Dewathang	Proportion	2000	40.5	65.7	19.9
Bhutan	Dewathang	Proportion	2017	58.3	80.2	33.2
Bhutan	Dewathang	Count	2000	0	1	0
Bhutan	Dewathang	Count	2017	1	1	0
Bhutan	Bara	Proportion	2000	50.7	74.2	26.6
Bhutan	Bara	Proportion	2017	64.4	83.0	41.3
Bhutan	Bara	Count	2000	1	1	0

Table 4: ORS by Unit *(continued)*

Country	Unit	Metric	Year	Mean	Upper	Lower
Bhutan	Bara	Count	2017	1	1	0
Bhutan	Bhur	Proportion	2000	41.0	71.4	16.9
Bhutan	Bhur	Proportion	2017	62.1	86.1	32.7
Bhutan	Bhur	Count	2000	0	1	0
Bhutan	Bhur	Count	2017	1	1	0
Bhutan	Chang	Proportion	2000	49.9	72.0	23.7
Bhutan	Chang	Proportion	2017	64.0	86.3	37.9
Bhutan	Chang	Count	2000	0	1	0
Bhutan	Chang	Count	2017	1	1	0
Bhutan	Bartsham	Proportion	2000	44.2	68.3	22.8
Bhutan	Bartsham	Proportion	2017	60.1	81.9	35.4
Bhutan	Bartsham	Count	2000	0	1	0
Bhutan	Bartsham	Count	2017	1	1	0
Bhutan	Dragteng	Proportion	2000	43.3	68.2	19.6
Bhutan	Dragteng	Proportion	2017	58.9	81.5	28.0
Bhutan	Dragteng	Count	2000	0	1	0
Bhutan	Dragteng	Count	2017	1	1	0
Bhutan	Barzhong	Proportion	2000	38.5	65.4	13.7
Bhutan	Barzhong	Proportion	2017	60.6	84.2	31.7
Bhutan	Barzhong	Count	2000	0	1	0
Bhutan	Barzhong	Count	2017	1	1	0
Bhutan	Athang	Proportion	2000	46.0	67.0	24.0
Bhutan	Athang	Proportion	2017	62.4	83.0	38.6
Bhutan	Athang	Count	2000	0	1	0
Bhutan	Athang	Count	2017	1	1	0
Bhutan	Bumdeling	Proportion	2000	47.4	69.7	25.9
Bhutan	Bumdeling	Proportion	2017	61.1	83.6	38.4
Bhutan	Bumdeling	Count	2000	0	1	0
Bhutan	Bumdeling	Count	2017	1	1	0
Bhutan	Bardo	Proportion	2000	52.9	75.4	29.9
Bhutan	Bardo	Proportion	2017	68.7	85.7	40.6
Bhutan	Bardo	Count	2000	1	1	0
Bhutan	Bardo	Count	2017	1	1	0
Bhutan	Chhume	Proportion	2000	45.4	68.2	23.3
Bhutan	Chhume	Proportion	2017	58.6	78.2	30.4
Bhutan	Chhume	Count	2000	0	1	0
Bhutan	Chhume	Count	2017	1	1	0
Bhutan	Bongo	Proportion	2000	46.2	68.5	23.4
Bhutan	Bongo	Proportion	2017	64.9	84.8	38.9
Bhutan	Bongo	Count	2000	0	1	0
Bhutan	Bongo	Count	2017	1	1	0
Bhutan	Dorona	Proportion	2000	47.0	70.5	22.1
Bhutan	Dorona	Proportion	2017	67.7	87.3	43.4
Bhutan	Dorona	Count	2000	0	1	0
Bhutan	Dorona	Count	2017	1	1	0
Bhutan	Goenkhatoe	Proportion	2000	45.4	69.4	21.4
Bhutan	Goenkhatoe	Proportion	2017	57.7	78.6	32.9
Bhutan	Goenkhatoe	Count	2000	0	1	0
Bhutan	Goenkhatoe	Count	2017	1	1	0

Table 4: ORS by Unit *(continued)*

Country	Unit	Metric	Year	Mean	Upper	Lower
Bhutan	Gakiling	Proportion	2000	52.4	73.2	29.9
Bhutan	Gakiling	Proportion	2017	66.0	82.7	45.1
Bhutan	Gakiling	Count	2000	1	1	0
Bhutan	Gakiling	Count	2017	1	1	0
Bhutan	Jaray	Proportion	2000	47.5	68.6	27.3
Bhutan	Jaray	Proportion	2017	62.3	81.9	38.1
Bhutan	Jaray	Count	2000	0	1	0
Bhutan	Jaray	Count	2017	1	1	0
Bhutan	Chaskhar	Proportion	2000	44.2	70.7	19.0
Bhutan	Chaskhar	Proportion	2017	61.3	82.0	36.2
Bhutan	Chaskhar	Count	2000	0	1	0
Bhutan	Chaskhar	Count	2017	1	1	0
Bhutan	Dopshari	Proportion	2000	47.4	76.7	22.9
Bhutan	Dopshari	Proportion	2017	61.5	81.1	37.3
Bhutan	Dopshari	Count	2000	0	1	0
Bhutan	Dopshari	Count	2017	1	1	0
Bhutan	Chokhorling	Proportion	2000	43.9	68.8	23.3
Bhutan	Chokhorling	Proportion	2017	62.0	81.2	39.4
Bhutan	Chokhorling	Count	2000	0	1	0
Bhutan	Chokhorling	Count	2017	1	1	0
Bhutan	Chhubu	Proportion	2000	49.6	75.7	24.5
Bhutan	Chhubu	Proportion	2017	63.3	85.8	33.6
Bhutan	Chhubu	Count	2000	0	1	0
Bhutan	Chhubu	Count	2017	1	1	0
Bhutan	Gomdar	Proportion	2000	46.8	72.3	25.0
Bhutan	Gomdar	Proportion	2017	61.2	81.1	39.0
Bhutan	Gomdar	Count	2000	0	1	0
Bhutan	Gomdar	Count	2017	1	1	0
Bhutan	Biru	Proportion	2000	43.3	71.1	17.8
Bhutan	Biru	Proportion	2017	58.9	80.6	35.6
Bhutan	Biru	Count	2000	0	1	0
Bhutan	Biru	Count	2017	1	1	0
Bhutan	Chhuzagang	Proportion	2000	39.9	68.9	15.8
Bhutan	Chhuzagang	Proportion	2017	59.2	81.7	30.5
Bhutan	Chhuzagang	Count	2000	0	1	0
Bhutan	Chhuzagang	Count	2017	1	1	0
Bhutan	Dagala	Proportion	2000	46.3	66.8	22.7
Bhutan	Dagala	Proportion	2017	59.5	82.3	34.2
Bhutan	Dagala	Count	2000	0	1	0
Bhutan	Dagala	Count	2017	1	1	0
Bhutan	Bidung	Proportion	2000	45.2	68.6	20.7
Bhutan	Bidung	Proportion	2017	60.3	81.9	36.6
Bhutan	Bidung	Count	2000	0	1	0
Bhutan	Bidung	Count	2017	1	1	0
Bhutan	Korphu	Proportion	2000	50.6	73.2	28.7
Bhutan	Korphu	Proportion	2017	68.0	85.1	42.9
Bhutan	Korphu	Count	2000	1	1	0
Bhutan	Korphu	Count	2017	1	1	0
Bhutan	Beteni	Proportion	2000	41.3	63.8	18.7

Table 4: ORS by Unit *(continued)*

Country	Unit	Metric	Year	Mean	Upper	Lower
Bhutan	Beteni	Proportion	2017	62.6	82.1	35.2
Bhutan	Beteni	Count	2000	0	1	0
Bhutan	Beteni	Count	2017	1	1	0
Bhutan	Bjena	Proportion	2000	45.8	68.8	21.7
Bhutan	Bjena	Proportion	2017	60.6	82.1	34.8
Bhutan	Bjena	Count	2000	0	1	0
Bhutan	Bjena	Count	2017	1	1	0
Bhutan	Jamkhar	Proportion	2000	45.0	68.0	23.3
Bhutan	Jamkhar	Proportion	2017	60.8	80.3	36.8
Bhutan	Jamkhar	Count	2000	0	1	0
Bhutan	Jamkhar	Count	2017	1	1	0
Bhutan	Bjoka	Proportion	2000	49.9	72.8	24.2
Bhutan	Bjoka	Proportion	2017	68.3	85.4	41.8
Bhutan	Bjoka	Count	2000	0	1	0
Bhutan	Bjoka	Count	2017	1	1	0
Bhutan	Tang	Proportion	2000	44.8	67.3	23.3
Bhutan	Tang	Proportion	2017	57.8	77.8	31.5
Bhutan	Tang	Count	2000	0	1	0
Bhutan	Tang	Count	2017	1	1	0
Bhutan	Chapchha	Proportion	2000	48.3	72.3	25.8
Bhutan	Chapchha	Proportion	2017	63.1	83.9	39.8
Bhutan	Chapchha	Count	2000	0	1	0
Bhutan	Chapchha	Count	2017	1	1	0
Bhutan	Drugyelgang	Proportion	2000	40.7	65.2	14.9
Bhutan	Drugyelgang	Proportion	2017	59.9	82.2	32.2
Bhutan	Drugyelgang	Count	2000	0	1	0
Bhutan	Drugyelgang	Count	2017	1	1	0
Bhutan	Laya	Proportion	2000	43.5	68.7	22.3
Bhutan	Laya	Proportion	2017	56.1	75.9	30.9
Bhutan	Laya	Count	2000	0	1	0
Bhutan	Laya	Count	2017	1	1	0
Bhutan	Katsho	Proportion	2000	45.1	68.6	21.2
Bhutan	Katsho	Proportion	2017	57.9	79.9	32.3
Bhutan	Katsho	Count	2000	0	1	0
Bhutan	Katsho	Count	2017	1	1	0
Bhutan	Khoma	Proportion	2000	47.6	68.6	25.1
Bhutan	Khoma	Proportion	2017	62.0	83.3	36.0
Bhutan	Khoma	Count	2000	0	1	0
Bhutan	Khoma	Count	2017	1	1	0
Bhutan	Chhali	Proportion	2000	46.6	71.5	22.1
Bhutan	Chhali	Proportion	2017	62.1	83.5	32.7
Bhutan	Chhali	Count	2000	0	1	0
Bhutan	Chhali	Count	2017	1	1	0
Bhutan	Doteng	Proportion	2000	46.2	70.2	21.8
Bhutan	Doteng	Proportion	2017	59.4	79.7	36.7
Bhutan	Doteng	Count	2000	0	1	0
Bhutan	Doteng	Count	2017	1	1	0
Bhutan	Chongshing	Proportion	2000	49.2	77.6	21.5
Bhutan	Chongshing	Proportion	2017	66.5	85.9	40.2

Table 4: ORS by Unit *(continued)*

Country	Unit	Metric	Year	Mean	Upper	Lower
Bhutan	Chongshing	Count	2000	0	1	0
Bhutan	Chongshing	Count	2017	1	1	0
Bhutan	Dzoma	Proportion	2000	45.1	69.2	19.7
Bhutan	Dzoma	Proportion	2017	59.1	83.4	31.6
Bhutan	Dzoma	Count	2000	0	1	0
Bhutan	Dzoma	Count	2017	1	1	0
Bhutan	Jangchhubling	Proportion	2000	45.4	68.6	23.9
Bhutan	Jangchhubling	Proportion	2017	62.3	81.7	37.9
Bhutan	Jangchhubling	Count	2000	0	1	0
Bhutan	Jangchhubling	Count	2017	1	1	0
Bhutan	Chargharay	Proportion	2000	47.6	72.7	22.0
Bhutan	Chargharay	Proportion	2017	62.0	80.7	39.3
Bhutan	Chargharay	Count	2000	0	1	0
Bhutan	Chargharay	Count	2017	1	1	0
Bhutan	Dekiling	Proportion	2000	43.4	67.3	19.1
Bhutan	Dekiling	Proportion	2017	64.4	84.1	36.0
Bhutan	Dekiling	Count	2000	0	1	0
Bhutan	Dekiling	Count	2017	1	1	0
Bhutan	Genye	Proportion	2000	45.9	66.7	21.0
Bhutan	Genye	Proportion	2017	60.0	79.7	36.2
Bhutan	Genye	Count	2000	0	1	0
Bhutan	Genye	Count	2017	1	1	0
Bhutan	Kanglung	Proportion	2000	43.7	68.7	20.7
Bhutan	Kanglung	Proportion	2017	59.3	80.0	34.5
Bhutan	Kanglung	Count	2000	0	1	0
Bhutan	Kanglung	Count	2017	1	1	0
Bhutan	Langthil	Proportion	2000	47.3	70.1	25.5
Bhutan	Langthil	Proportion	2017	63.2	81.5	34.0
Bhutan	Langthil	Count	2000	0	1	0
Bhutan	Langthil	Count	2017	1	1	0
Bhutan	Dunglegang	Proportion	2000	39.8	63.3	16.6
Bhutan	Dunglegang	Proportion	2017	60.6	82.7	32.2
Bhutan	Dunglegang	Count	2000	0	1	0
Bhutan	Dunglegang	Count	2017	1	1	0
Bhutan	Daga	Proportion	2000	47.6	71.5	22.6
Bhutan	Daga	Proportion	2017	64.6	84.0	39.6
Bhutan	Daga	Count	2000	0	1	0
Bhutan	Daga	Count	2017	1	1	0
Bhutan	Khamdang	Proportion	2000	45.3	68.1	24.5
Bhutan	Khamdang	Proportion	2017	60.9	82.3	37.2
Bhutan	Khamdang	Count	2000	0	1	0
Bhutan	Khamdang	Count	2017	1	1	0
Bhutan	Gozhing	Proportion	2000	53.4	78.2	28.8
Bhutan	Gozhing	Proportion	2017	71.3	88.9	44.7
Bhutan	Gozhing	Count	2000	1	1	0
Bhutan	Gozhing	Count	2017	1	1	0
Bhutan	Ura	Proportion	2000	45.9	67.7	23.3
Bhutan	Ura	Proportion	2017	58.4	79.2	30.3
Bhutan	Ura	Count	2000	0	1	0

Table 4: ORS by Unit *(continued)*

Country	Unit	Metric	Year	Mean	Upper	Lower
Bhutan	Ura	Count	2017	1	1	0
Bhutan	Dala	Proportion	2000	43.2	67.1	21.1
Bhutan	Dala	Proportion	2017	62.2	84.4	32.5
Bhutan	Dala	Count	2000	0	1	0
Bhutan	Dala	Count	2017	1	1	0
Bhutan	Gesarling	Proportion	2000	42.5	68.7	18.4
Bhutan	Gesarling	Proportion	2017	63.7	83.3	34.0
Bhutan	Gesarling	Count	2000	0	1	0
Bhutan	Gesarling	Count	2017	1	1	0
Bhutan	Lunana	Proportion	2000	40.8	63.5	19.6
Bhutan	Lunana	Proportion	2017	55.1	74.8	31.3
Bhutan	Lunana	Count	2000	0	1	0
Bhutan	Lunana	Count	2017	1	1	0
Bhutan	Sama	Proportion	2000	46.7	69.1	24.7
Bhutan	Sama	Proportion	2017	59.5	78.5	34.7
Bhutan	Sama	Count	2000	0	1	0
Bhutan	Sama	Count	2017	1	1	0
Bhutan	Kurtoe	Proportion	2000	50.8	71.9	28.1
Bhutan	Kurtoe	Proportion	2017	56.2	78.2	30.4
Bhutan	Kurtoe	Count	2000	1	1	0
Bhutan	Kurtoe	Count	2017	1	1	0
Bhutan	Drametse	Proportion	2000	44.4	67.1	23.3
Bhutan	Drametse	Proportion	2017	59.0	82.0	32.9
Bhutan	Drametse	Count	2000	0	1	0
Bhutan	Drametse	Count	2017	1	1	0
Bhutan	Hungrel	Proportion	2000	47.1	72.6	22.9
Bhutan	Hungrel	Proportion	2017	61.3	80.5	38.0
Bhutan	Hungrel	Count	2000	0	1	0
Bhutan	Hungrel	Count	2017	1	1	0
Bhutan	Dechhenling	Proportion	2000	47.3	70.5	25.7
Bhutan	Dechhenling	Proportion	2017	66.3	84.4	42.5
Bhutan	Dechhenling	Count	2000	0	1	0
Bhutan	Dechhenling	Count	2017	1	1	0
Bhutan	Goenshari	Proportion	2000	48.1	72.0	22.0
Bhutan	Goenshari	Proportion	2017	62.2	84.6	39.0
Bhutan	Goenshari	Count	2000	0	1	0
Bhutan	Goenshari	Count	2017	1	1	0
Bhutan	Langchhenphu	Proportion	2000	41.4	66.4	20.7
Bhutan	Langchhenphu	Proportion	2017	55.0	77.2	28.3
Bhutan	Langchhenphu	Count	2000	0	1	0
Bhutan	Langchhenphu	Count	2017	1	1	0
Bhutan	Chengmari	Proportion	2000	46.5	70.7	22.0
Bhutan	Chengmari	Proportion	2017	62.3	80.9	35.7
Bhutan	Chengmari	Count	2000	0	1	0
Bhutan	Chengmari	Count	2017	1	1	0
Bhutan	Doban	Proportion	2000	47.0	69.8	22.8
Bhutan	Doban	Proportion	2017	66.5	86.5	41.2
Bhutan	Doban	Count	2000	0	1	0
Bhutan	Doban	Count	2017	1	1	0

Table 4: ORS by Unit *(continued)*

Country	Unit	Metric	Year	Mean	Upper	Lower
Bhutan	Kawang	Proportion	2000	46.8	69.4	22.6
Bhutan	Kawang	Proportion	2017	60.6	83.4	34.1
Bhutan	Kawang	Count	2000	0	1	0
Bhutan	Kawang	Count	2017	1	1	0
Bhutan	Kangpara	Proportion	2000	49.6	72.2	26.8
Bhutan	Kangpara	Proportion	2017	63.6	82.1	40.9
Bhutan	Kangpara	Count	2000	0	1	0
Bhutan	Kangpara	Count	2017	1	1	0
Bhutan	Nubi	Proportion	2000	44.8	67.7	22.2
Bhutan	Nubi	Proportion	2017	60.0	80.8	33.8
Bhutan	Nubi	Count	2000	0	1	0
Bhutan	Nubi	Count	2017	1	1	0
Bhutan	Gosarling	Proportion	2000	39.4	66.1	12.6
Bhutan	Gosarling	Proportion	2017	60.1	83.5	31.4
Bhutan	Gosarling	Count	2000	0	1	0
Bhutan	Gosarling	Count	2017	1	1	0
Bhutan	Dangchhu	Proportion	2000	45.5	70.0	19.2
Bhutan	Dangchhu	Proportion	2017	58.7	82.8	31.9
Bhutan	Dangchhu	Count	2000	0	1	0
Bhutan	Dangchhu	Count	2017	1	1	0
Bhutan	Ramjar	Proportion	2000	44.8	70.2	22.3
Bhutan	Ramjar	Proportion	2017	60.3	80.7	35.2
Bhutan	Ramjar	Count	2000	0	1	0
Bhutan	Ramjar	Count	2017	1	1	0
Bhutan	Nangkor	Proportion	2000	50.7	74.4	25.3
Bhutan	Nangkor	Proportion	2017	67.2	84.9	39.9
Bhutan	Nangkor	Count	2000	1	1	0
Bhutan	Nangkor	Count	2017	1	1	0
Bhutan	Dungna	Proportion	2000	47.9	68.9	26.5
Bhutan	Dungna	Proportion	2017	63.6	80.3	41.5
Bhutan	Dungna	Count	2000	0	1	0
Bhutan	Dungna	Count	2017	1	1	0
Bhutan	Gozhi	Proportion	2000	39.1	67.8	15.8
Bhutan	Gozhi	Proportion	2017	61.9	83.3	30.0
Bhutan	Gozhi	Count	2000	0	1	0
Bhutan	Gozhi	Count	2017	1	1	0
Bhutan	Sombey	Proportion	2000	55.1	74.9	32.6
Bhutan	Sombey	Proportion	2017	63.3	80.7	43.2
Bhutan	Sombey	Count	2000	1	1	0
Bhutan	Sombey	Count	2017	1	1	0
Bhutan	Menbi	Proportion	2000	46.9	70.1	22.6
Bhutan	Menbi	Proportion	2017	62.0	83.3	36.0
Bhutan	Menbi	Count	2000	0	1	0
Bhutan	Menbi	Count	2017	1	1	0
Bhutan	Drepung	Proportion	2000	45.7	70.1	21.2
Bhutan	Drepung	Proportion	2017	62.0	82.7	36.1
Bhutan	Drepung	Count	2000	0	1	0
Bhutan	Drepung	Count	2017	1	1	0
Bhutan	Lamgong	Proportion	2000	46.6	71.2	22.5

Table 4: ORS by Unit *(continued)*

Country	Unit	Metric	Year	Mean	Upper	Lower
Bhutan	Lamgong	Proportion	2017	60.6	81.9	36.4
Bhutan	Lamgong	Count	2000	0	1	0
Bhutan	Lamgong	Count	2017	1	1	0
Bhutan	Dungmin	Proportion	2000	48.7	74.1	23.9
Bhutan	Dungmin	Proportion	2017	66.5	84.2	43.7
Bhutan	Dungmin	Count	2000	0	1	0
Bhutan	Dungmin	Count	2017	1	1	0
Bhutan	Guma	Proportion	2000	46.0	68.9	21.7
Bhutan	Guma	Proportion	2017	60.6	83.3	32.7
Bhutan	Guma	Count	2000	0	1	0
Bhutan	Guma	Count	2017	1	1	0
Bhutan	Lauri	Proportion	2000	52.8	73.4	30.9
Bhutan	Lauri	Proportion	2017	67.0	85.1	44.6
Bhutan	Lauri	Count	2000	1	1	0
Bhutan	Lauri	Count	2017	1	1	0
Bhutan	Denchhukha	Proportion	2000	48.3	71.7	26.3
Bhutan	Denchhukha	Proportion	2017	63.4	82.4	40.5
Bhutan	Denchhukha	Count	2000	0	1	0
Bhutan	Denchhukha	Count	2017	1	1	0
Bhutan	Gelephu	Proportion	2000	41.8	72.5	16.2
Bhutan	Gelephu	Proportion	2017	62.2	86.1	31.2
Bhutan	Gelephu	Count	2000	0	1	0
Bhutan	Gelephu	Count	2017	1	1	0
Bhutan	Lingzhi	Proportion	2000	44.3	69.5	24.0
Bhutan	Lingzhi	Proportion	2017	57.1	77.7	36.2
Bhutan	Lingzhi	Count	2000	0	1	0
Bhutan	Lingzhi	Count	2017	1	1	0
Bhutan	Khaling	Proportion	2000	47.1	69.4	25.0
Bhutan	Khaling	Proportion	2017	61.8	81.3	36.9
Bhutan	Khaling	Count	2000	0	1	0
Bhutan	Khaling	Count	2017	1	1	0
Bhutan	Tangsibji	Proportion	2000	44.4	66.1	21.8
Bhutan	Tangsibji	Proportion	2017	59.6	80.1	32.1
Bhutan	Tangsibji	Count	2000	0	1	0
Bhutan	Tangsibji	Count	2017	1	1	0
Bhutan	Kikorthang	Proportion	2000	38.9	65.6	15.3
Bhutan	Kikorthang	Proportion	2017	60.0	81.8	33.6
Bhutan	Kikorthang	Count	2000	0	1	0
Bhutan	Kikorthang	Count	2017	1	1	0
Bhutan	Gangte	Proportion	2000	40.5	65.1	17.7
Bhutan	Gangte	Proportion	2017	52.6	76.6	27.4
Bhutan	Gangte	Count	2000	0	1	0
Bhutan	Gangte	Count	2017	1	1	0
Bhutan	Toetsho	Proportion	2000	45.7	69.1	23.8
Bhutan	Toetsho	Proportion	2017	61.5	82.6	35.7
Bhutan	Toetsho	Count	2000	0	1	0
Bhutan	Toetsho	Count	2017	1	1	0
Bhutan	Ngangla	Proportion	2000	48.9	73.9	25.1
Bhutan	Ngangla	Proportion	2017	67.6	86.2	41.1

Table 4: ORS by Unit *(continued)*

Country	Unit	Metric	Year	Mean	Upper	Lower
Bhutan	Ngangla	Count	2000	0	1	0
Bhutan	Ngangla	Count	2017	1	1	0
Bhutan	Geling	Proportion	2000	46.8	69.8	24.7
Bhutan	Geling	Proportion	2017	63.6	83.3	35.1
Bhutan	Geling	Count	2000	0	1	0
Bhutan	Geling	Count	2017	1	1	0
Bhutan	Kalidzingkha	Proportion	2000	45.4	69.3	18.7
Bhutan	Kalidzingkha	Proportion	2017	64.2	84.7	37.0
Bhutan	Kalidzingkha	Count	2000	0	1	0
Bhutan	Kalidzingkha	Count	2017	1	1	0
Bhutan	Uesu	Proportion	2000	46.7	71.6	24.7
Bhutan	Uesu	Proportion	2017	59.4	79.3	35.5
Bhutan	Uesu	Count	2000	0	1	0
Bhutan	Uesu	Count	2017	1	1	0
Bhutan	Metsho	Proportion	2000	48.5	70.2	24.4
Bhutan	Metsho	Proportion	2017	63.3	82.7	39.0
Bhutan	Metsho	Count	2000	0	1	0
Bhutan	Metsho	Count	2017	1	1	0
Bhutan	Gongdue	Proportion	2000	49.6	71.2	26.7
Bhutan	Gongdue	Proportion	2017	66.6	83.7	40.5
Bhutan	Gongdue	Count	2000	0	1	0
Bhutan	Gongdue	Count	2017	1	1	0
Bhutan	Lungnyi	Proportion	2000	48.4	73.1	25.3
Bhutan	Lungnyi	Proportion	2017	61.8	82.5	39.7
Bhutan	Lungnyi	Count	2000	0	1	0
Bhutan	Lungnyi	Count	2017	1	1	0
Bhutan	Khar	Proportion	2000	49.3	73.2	25.3
Bhutan	Khar	Proportion	2017	66.0	85.7	40.1
Bhutan	Khar	Count	2000	0	1	0
Bhutan	Khar	Count	2017	1	1	0
Bhutan	Kabjisa	Proportion	2000	47.6	71.9	25.3
Bhutan	Kabjisa	Proportion	2017	61.8	85.1	37.8
Bhutan	Kabjisa	Count	2000	0	1	0
Bhutan	Kabjisa	Count	2017	1	1	0
Bhutan	Martshala	Proportion	2000	48.7	72.1	27.3
Bhutan	Martshala	Proportion	2017	63.6	83.1	41.7
Bhutan	Martshala	Count	2000	0	1	0
Bhutan	Martshala	Count	2017	1	1	0
Bhutan	Dorokha	Proportion	2000	45.4	68.0	20.2
Bhutan	Dorokha	Proportion	2017	60.8	81.1	38.7
Bhutan	Dorokha	Count	2000	0	1	0
Bhutan	Dorokha	Count	2017	1	1	0
Bhutan	Hiley	Proportion	2000	42.5	65.9	18.9
Bhutan	Hiley	Proportion	2017	63.3	84.9	36.1
Bhutan	Hiley	Count	2000	0	1	0
Bhutan	Hiley	Count	2017	1	1	0
Bhutan	Mewang	Proportion	2000	46.5	68.5	24.2
Bhutan	Mewang	Proportion	2017	61.5	82.5	36.3
Bhutan	Mewang	Count	2000	0	1	0

Table 4: ORS by Unit *(continued)*

Country	Unit	Metric	Year	Mean	Upper	Lower
Bhutan	Mewang	Count	2017	1	1	0
Bhutan	Lumang	Proportion	2000	46.3	70.9	23.1
Bhutan	Lumang	Proportion	2017	60.3	81.2	35.8
Bhutan	Lumang	Count	2000	0	1	0
Bhutan	Lumang	Count	2017	1	1	0
Bhutan	Mendrelgang	Proportion	2000	39.5	62.6	16.8
Bhutan	Mendrelgang	Proportion	2017	61.1	81.5	33.0
Bhutan	Mendrelgang	Count	2000	0	1	0
Bhutan	Mendrelgang	Count	2017	1	1	0
Bhutan	Gasetsho Gom	Proportion	2000	46.1	73.9	20.8
Bhutan	Gasetsho Gom	Proportion	2017	60.0	83.6	34.0
Bhutan	Gasetsho Gom	Count	2000	0	1	0
Bhutan	Gasetsho Gom	Count	2017	1	1	0
Bhutan	Tomzhangtshen	Proportion	2000	45.4	69.8	23.5
Bhutan	Tomzhangtshen	Proportion	2017	60.9	82.7	34.8
Bhutan	Tomzhangtshen	Count	2000	0	1	0
Bhutan	Tomzhangtshen	Count	2017	1	1	0
Bhutan	Pangkhar	Proportion	2000	51.1	71.6	28.4
Bhutan	Pangkhar	Proportion	2017	69.9	86.8	46.2
Bhutan	Pangkhar	Count	2000	1	1	0
Bhutan	Pangkhar	Count	2017	1	1	0
Bhutan	Getana	Proportion	2000	47.8	70.0	23.5
Bhutan	Getana	Proportion	2017	67.3	88.1	44.8
Bhutan	Getana	Count	2000	0	1	0
Bhutan	Getana	Count	2017	1	1	0
Bhutan	Khipisa	Proportion	2000	42.0	69.0	17.0
Bhutan	Khipisa	Proportion	2017	61.8	83.4	36.1
Bhutan	Khipisa	Count	2000	0	1	0
Bhutan	Khipisa	Count	2017	1	1	0
Bhutan	Minjay	Proportion	2000	50.1	73.7	26.1
Bhutan	Minjay	Proportion	2017	64.5	85.8	39.9
Bhutan	Minjay	Count	2000	1	1	0
Bhutan	Minjay	Count	2017	1	1	0
Bhutan	Jurmey	Proportion	2000	45.7	70.5	23.8
Bhutan	Jurmey	Proportion	2017	62.3	81.0	38.1
Bhutan	Jurmey	Count	2000	0	1	0
Bhutan	Jurmey	Count	2017	1	1	0
Bhutan	Naja	Proportion	2000	48.1	70.5	25.8
Bhutan	Naja	Proportion	2017	61.2	80.2	40.0
Bhutan	Naja	Count	2000	0	1	0
Bhutan	Naja	Count	2017	1	1	0
Bhutan	Nanong	Proportion	2000	46.7	69.4	25.5
Bhutan	Nanong	Proportion	2017	61.0	81.4	36.6
Bhutan	Nanong	Count	2000	0	1	0
Bhutan	Nanong	Count	2017	1	1	0
Bhutan	Lingmukha	Proportion	2000	44.1	69.8	18.5
Bhutan	Lingmukha	Proportion	2017	59.5	84.2	33.6
Bhutan	Lingmukha	Count	2000	0	1	0
Bhutan	Lingmukha	Count	2017	1	1	0

Table 4: ORS by Unit *(continued)*

Country	Unit	Metric	Year	Mean	Upper	Lower
Bhutan	Pemathang	Proportion	2000	42.6	67.3	21.5
Bhutan	Pemathang	Proportion	2017	58.9	79.7	34.0
Bhutan	Pemathang	Count	2000	0	1	0
Bhutan	Pemathang	Count	2017	1	1	0
Bhutan	Dungtoe	Proportion	2000	46.7	72.3	21.4
Bhutan	Dungtoe	Proportion	2017	62.1	82.4	37.5
Bhutan	Dungtoe	Count	2000	0	1	0
Bhutan	Dungtoe	Count	2017	1	1	0
Bhutan	Jigmichhoeling	Proportion	2000	48.1	72.7	25.5
Bhutan	Jigmichhoeling	Proportion	2017	67.6	86.1	41.4
Bhutan	Jigmichhoeling	Count	2000	0	1	0
Bhutan	Jigmichhoeling	Count	2017	1	1	0
Bhutan	Naro	Proportion	2000	44.9	68.7	20.1
Bhutan	Naro	Proportion	2017	57.4	79.7	35.7
Bhutan	Naro	Count	2000	0	1	0
Bhutan	Naro	Count	2017	1	1	0
Bhutan	Merak	Proportion	2000	48.7	70.5	23.6
Bhutan	Merak	Proportion	2017	62.4	80.7	40.2
Bhutan	Merak	Count	2000	0	1	0
Bhutan	Merak	Count	2017	1	1	0
Bhutan	Patakla	Proportion	2000	43.7	68.8	17.6
Bhutan	Patakla	Proportion	2017	63.2	82.3	39.0
Bhutan	Patakla	Count	2000	0	1	0
Bhutan	Patakla	Count	2017	1	1	0
Bhutan	Gasetsho Om	Proportion	2000	44.6	67.4	19.8
Bhutan	Gasetsho Om	Proportion	2017	59.2	81.2	33.6
Bhutan	Gasetsho Om	Count	2000	0	1	0
Bhutan	Gasetsho Om	Count	2017	1	1	0
Bhutan	Trashiyangtse	Proportion	2000	46.4	70.2	25.3
Bhutan	Trashiyangtse	Proportion	2017	61.7	82.3	38.1
Bhutan	Trashiyangtse	Count	2000	0	1	0
Bhutan	Trashiyangtse	Count	2017	1	1	0
Bhutan	Shingkhar	Proportion	2000	53.8	74.5	30.0
Bhutan	Shingkhar	Proportion	2017	68.4	85.3	38.3
Bhutan	Shingkhar	Count	2000	1	1	0
Bhutan	Shingkhar	Count	2017	1	1	0
Bhutan	Logchina	Proportion	2000	44.6	69.7	22.0
Bhutan	Logchina	Proportion	2017	61.1	81.6	35.7
Bhutan	Logchina	Count	2000	0	1	0
Bhutan	Logchina	Count	2017	1	1	0
Bhutan	Lajab	Proportion	2000	44.2	67.2	19.0
Bhutan	Lajab	Proportion	2017	64.4	85.0	37.9
Bhutan	Lajab	Count	2000	0	1	0
Bhutan	Lajab	Count	2017	1	1	0
Bhutan	Tsenkhar	Proportion	2000	47.8	71.0	24.5
Bhutan	Tsenkhar	Proportion	2017	62.1	82.9	36.7
Bhutan	Tsenkhar	Count	2000	0	1	0
Bhutan	Tsenkhar	Count	2017	1	1	0
Bhutan	Kengkhar	Proportion	2000	45.3	71.8	23.9

Table 4: ORS by Unit *(continued)*

Country	Unit	Metric	Year	Mean	Upper	Lower
Bhutan	Kengkhar	Proportion	2017	61.6	80.3	36.2
Bhutan	Kengkhar	Count	2000	0	1	0
Bhutan	Kengkhar	Count	2017	1	1	0
Bhutan	Shapa	Proportion	2000	47.1	70.6	25.6
Bhutan	Shapa	Proportion	2017	61.6	79.6	38.8
Bhutan	Shapa	Count	2000	0	1	0
Bhutan	Shapa	Count	2017	1	1	0
Bhutan	Norbugang	Proportion	2000	44.5	70.4	21.9
Bhutan	Norbugang	Proportion	2017	64.6	82.4	37.2
Bhutan	Norbugang	Count	2000	0	1	0
Bhutan	Norbugang	Count	2017	1	1	0
Bhutan	Shengabjimi	Proportion	2000	46.0	70.4	20.1
Bhutan	Shengabjimi	Proportion	2017	59.7	82.2	32.0
Bhutan	Shengabjimi	Count	2000	0	1	0
Bhutan	Shengabjimi	Count	2017	1	1	0
Bhutan	Phuntsthothang	Proportion	2000	41.3	68.1	20.6
Bhutan	Phuntsthothang	Proportion	2017	59.9	80.9	35.5
Bhutan	Phuntsthothang	Count	2000	0	1	0
Bhutan	Phuntsthothang	Count	2017	1	1	0
Bhutan	Namgyel Chhoeling	Proportion	2000	45.8	68.7	22.0
Bhutan	Namgyel Chhoeling	Proportion	2017	60.6	81.2	41.4
Bhutan	Namgyel Chhoeling	Count	2000	0	1	0
Bhutan	Namgyel Chhoeling	Count	2017	1	1	0
Bhutan	Senge	Proportion	2000	41.7	64.5	19.0
Bhutan	Senge	Proportion	2017	63.9	84.0	37.0
Bhutan	Senge	Count	2000	0	1	0
Bhutan	Senge	Count	2017	1	1	0
Bhutan	Soe	Proportion	2000	43.8	67.5	20.6
Bhutan	Soe	Proportion	2017	57.8	78.7	36.0
Bhutan	Soe	Count	2000	0	1	0
Bhutan	Soe	Count	2017	1	1	0
Bhutan	Phongme	Proportion	2000	47.6	72.5	21.9
Bhutan	Phongme	Proportion	2017	63.2	82.6	38.4
Bhutan	Phongme	Count	2000	0	1	0
Bhutan	Phongme	Count	2017	1	1	0
Bhutan	Phuentenchhu	Proportion	2000	41.3	66.5	14.9
Bhutan	Phuentenchhu	Proportion	2017	62.2	83.2	34.5
Bhutan	Phuentenchhu	Count	2000	0	1	0
Bhutan	Phuentenchhu	Count	2017	1	1	0
Bhutan	Kazhi	Proportion	2000	44.8	67.2	22.7
Bhutan	Kazhi	Proportion	2017	58.9	80.2	33.9
Bhutan	Kazhi	Count	2000	0	1	0
Bhutan	Kazhi	Count	2017	1	1	0
Bhutan	Yalang	Proportion	2000	47.5	71.2	25.0
Bhutan	Yalang	Proportion	2017	62.5	82.8	37.6
Bhutan	Yalang	Count	2000	0	1	0
Bhutan	Yalang	Count	2017	1	1	0
Bhutan	Trong	Proportion	2000	50.6	72.8	28.1
Bhutan	Trong	Proportion	2017	67.9	84.3	41.5

Table 4: ORS by Unit *(continued)*

Country	Unit	Metric	Year	Mean	Upper	Lower
Bhutan	Trong	Count	2000	1	1	0
Bhutan	Trong	Count	2017	1	1	0
Bhutan	Metap	Proportion	2000	48.4	71.3	26.3
Bhutan	Metap	Proportion	2017	64.1	82.1	42.2
Bhutan	Metap	Count	2000	0	1	0
Bhutan	Metap	Count	2017	1	1	0
Bhutan	Lhamoizingkha	Proportion	2000	41.1	66.7	17.8
Bhutan	Lhamoizingkha	Proportion	2017	59.6	81.6	34.4
Bhutan	Lhamoizingkha	Count	2000	0	1	0
Bhutan	Lhamoizingkha	Count	2017	1	1	0
Bhutan	Mongar	Proportion	2000	46.1	72.0	22.5
Bhutan	Mongar	Proportion	2017	62.6	82.3	36.2
Bhutan	Mongar	Count	2000	0	1	0
Bhutan	Mongar	Count	2017	1	1	0
Bhutan	Tsento	Proportion	2000	46.3	69.1	24.7
Bhutan	Tsento	Proportion	2017	59.1	78.2	35.8
Bhutan	Tsento	Count	2000	0	1	0
Bhutan	Tsento	Count	2017	1	1	0
Bhutan	Shumer	Proportion	2000	45.8	71.1	23.4
Bhutan	Shumer	Proportion	2017	63.2	83.1	38.7
Bhutan	Shumer	Count	2000	0	1	0
Bhutan	Shumer	Count	2017	1	1	0
Bhutan	Talo	Proportion	2000	45.4	68.8	20.6
Bhutan	Talo	Proportion	2017	59.7	83.1	32.4
Bhutan	Talo	Count	2000	0	1	0
Bhutan	Talo	Count	2017	1	1	0
Bhutan	Samrang	Proportion	2000	46.0	71.7	22.7
Bhutan	Samrang	Proportion	2017	61.1	84.7	32.9
Bhutan	Samrang	Count	2000	0	1	0
Bhutan	Samrang	Count	2017	1	1	0
Bhutan	Pagli	Proportion	2000	41.9	67.2	17.5
Bhutan	Pagli	Proportion	2017	59.9	80.9	32.6
Bhutan	Pagli	Count	2000	0	1	0
Bhutan	Pagli	Count	2017	1	1	0
Bhutan	Sherzhong	Proportion	2000	42.5	68.9	17.0
Bhutan	Sherzhong	Proportion	2017	62.5	85.2	34.4
Bhutan	Sherzhong	Count	2000	0	1	0
Bhutan	Sherzhong	Count	2017	1	1	0
Bhutan	Radi	Proportion	2000	46.3	72.9	19.0
Bhutan	Radi	Proportion	2017	61.7	83.5	32.5
Bhutan	Radi	Count	2000	0	1	0
Bhutan	Radi	Count	2017	1	1	0
Bhutan	Rangthangling	Proportion	2000	38.9	64.6	16.1
Bhutan	Rangthangling	Proportion	2017	61.9	83.2	36.7
Bhutan	Rangthangling	Count	2000	0	1	0
Bhutan	Rangthangling	Count	2017	1	1	0
Bhutan	Nahi	Proportion	2000	47.2	68.8	21.7
Bhutan	Nahi	Proportion	2017	61.0	81.2	36.2
Bhutan	Nahi	Count	2000	0	1	0

Table 4: ORS by Unit *(continued)*

Country	Unit	Metric	Year	Mean	Upper	Lower
Bhutan	Nahi	Count	2017	1	1	0
Bhutan	Phuentsholing	Proportion	2000	47.4	72.7	21.4
Bhutan	Phuentsholing	Proportion	2017	63.3	83.6	34.2
Bhutan	Phuentsholing	Count	2000	0	1	0
Bhutan	Phuentsholing	Count	2017	1	1	0
Bhutan	Nichula	Proportion	2000	43.9	69.4	19.5
Bhutan	Nichula	Proportion	2017	64.8	83.8	37.5
Bhutan	Nichula	Count	2000	0	1	0
Bhutan	Nichula	Count	2017	1	1	0
Bhutan	Narang	Proportion	2000	43.2	66.1	21.8
Bhutan	Narang	Proportion	2017	59.5	81.4	36.2
Bhutan	Narang	Count	2000	0	1	0
Bhutan	Narang	Count	2017	1	1	0
Bhutan	Wangchang	Proportion	2000	48.3	71.8	23.5
Bhutan	Wangchang	Proportion	2017	62.5	82.1	37.6
Bhutan	Wangchang	Count	2000	0	1	0
Bhutan	Wangchang	Count	2017	1	1	0
Bhutan	Yurung	Proportion	2000	47.9	75.6	21.9
Bhutan	Yurung	Proportion	2017	64.9	84.1	37.1
Bhutan	Yurung	Count	2000	0	1	0
Bhutan	Yurung	Count	2017	1	1	0
Bhutan	Toepisa	Proportion	2000	47.8	71.0	21.5
Bhutan	Toepisa	Proportion	2017	62.1	83.9	36.3
Bhutan	Toepisa	Count	2000	0	1	0
Bhutan	Toepisa	Count	2017	1	1	0
Bhutan	Serthig	Proportion	2000	52.0	72.9	30.4
Bhutan	Serthig	Proportion	2017	66.1	84.7	43.3
Bhutan	Serthig	Count	2000	1	1	0
Bhutan	Serthig	Count	2017	1	1	0
Bhutan	Samtse	Proportion	2000	44.9	70.2	20.1
Bhutan	Samtse	Proportion	2017	61.2	81.8	32.6
Bhutan	Samtse	Count	2000	0	1	0
Bhutan	Samtse	Count	2017	1	1	0
Bhutan	Shompangkha	Proportion	2000	39.7	67.5	16.7
Bhutan	Shompangkha	Proportion	2017	62.8	85.2	31.7
Bhutan	Shompangkha	Count	2000	0	1	0
Bhutan	Shompangkha	Count	2017	1	1	0
Bhutan	Sakteng	Proportion	2000	49.9	71.0	26.0
Bhutan	Sakteng	Proportion	2017	62.5	81.3	40.1
Bhutan	Sakteng	Count	2000	0	1	0
Bhutan	Sakteng	Count	2017	1	1	0
Bhutan	Shemjong	Proportion	2000	39.2	66.2	16.2
Bhutan	Shemjong	Proportion	2017	60.6	83.9	31.1
Bhutan	Shemjong	Count	2000	0	1	0
Bhutan	Shemjong	Count	2017	1	1	0
Bhutan	Nyisho	Proportion	2000	46.4	68.3	19.9
Bhutan	Nyisho	Proportion	2017	61.4	84.1	36.2
Bhutan	Nyisho	Count	2000	0	1	0
Bhutan	Nyisho	Count	2017	1	1	0

Table 4: ORS by Unit *(continued)*

Country	Unit	Metric	Year	Mean	Upper	Lower
Bhutan	Samphelling	Proportion	2000	43.8	69.8	20.2
Bhutan	Samphelling	Proportion	2017	61.8	83.1	31.4
Bhutan	Samphelling	Count	2000	0	1	0
Bhutan	Samphelling	Count	2017	1	1	0
Bhutan	Trashiding	Proportion	2000	40.3	67.3	16.6
Bhutan	Trashiding	Proportion	2017	61.9	84.1	33.6
Bhutan	Trashiding	Count	2000	0	1	0
Bhutan	Trashiding	Count	2017	1	1	0
Bhutan	Ngatshang	Proportion	2000	46.0	69.8	23.2
Bhutan	Ngatshang	Proportion	2017	62.4	81.1	39.3
Bhutan	Ngatshang	Count	2000	0	1	0
Bhutan	Ngatshang	Count	2017	1	1	0
Bhutan	Zobel	Proportion	2000	48.5	72.4	22.9
Bhutan	Zobel	Proportion	2017	63.7	83.0	39.3
Bhutan	Zobel	Count	2000	0	1	0
Bhutan	Zobel	Count	2017	1	1	0
Bhutan	Toewang	Proportion	2000	47.6	72.2	23.1
Bhutan	Toewang	Proportion	2017	61.6	84.1	36.5
Bhutan	Toewang	Count	2000	0	1	0
Bhutan	Toewang	Count	2017	1	1	0
Bhutan	Wangphu	Proportion	2000	46.0	73.5	23.9
Bhutan	Wangphu	Proportion	2017	61.6	82.5	36.2
Bhutan	Wangphu	Count	2000	0	1	0
Bhutan	Wangphu	Count	2017	1	1	0
Bhutan	Sipsu	Proportion	2000	42.6	68.1	17.9
Bhutan	Sipsu	Proportion	2017	58.5	82.9	33.4
Bhutan	Sipsu	Count	2000	0	1	0
Bhutan	Sipsu	Count	2017	1	1	0
Bhutan	Taklai	Proportion	2000	40.6	69.3	16.7
Bhutan	Taklai	Proportion	2017	60.2	85.3	30.8
Bhutan	Taklai	Count	2000	0	1	0
Bhutan	Taklai	Count	2017	1	1	0
Bhutan	Samkhar	Proportion	2000	45.7	67.9	23.2
Bhutan	Samkhar	Proportion	2017	61.3	81.3	38.0
Bhutan	Samkhar	Count	2000	0	1	0
Bhutan	Samkhar	Count	2017	1	1	0
Bhutan	Tsholingkhor	Proportion	2000	39.4	69.2	12.9
Bhutan	Tsholingkhor	Proportion	2017	60.1	83.7	28.5
Bhutan	Tsholingkhor	Count	2000	0	1	0
Bhutan	Tsholingkhor	Count	2017	1	1	0
Bhutan	Phangyuel	Proportion	2000	44.0	66.5	18.2
Bhutan	Phangyuel	Proportion	2017	59.4	80.5	31.5
Bhutan	Phangyuel	Count	2000	0	1	0
Bhutan	Phangyuel	Count	2017	1	1	0
Bhutan	Tsangkha	Proportion	2000	39.2	65.6	16.7
Bhutan	Tsangkha	Proportion	2017	60.6	81.9	34.2
Bhutan	Tsangkha	Count	2000	0	1	0
Bhutan	Tsangkha	Count	2017	1	1	0
Bhutan	Saleng	Proportion	2000	48.4	70.6	27.1

Table 4: ORS by Unit *(continued)*

Country	Unit	Metric	Year	Mean	Upper	Lower
Bhutan	Saleng	Proportion	2017	64.5	81.4	37.9
Bhutan	Saleng	Count	2000	0	1	0
Bhutan	Saleng	Count	2017	1	1	0
Bhutan	Tading	Proportion	2000	44.2	69.2	20.5
Bhutan	Tading	Proportion	2017	60.0	83.1	32.4
Bhutan	Tading	Count	2000	0	1	0
Bhutan	Tading	Count	2017	1	1	0
Bhutan	Umling	Proportion	2000	43.3	69.8	19.0
Bhutan	Umling	Proportion	2017	62.9	82.8	36.3
Bhutan	Umling	Count	2000	0	1	0
Bhutan	Umling	Count	2017	1	1	0
Bhutan	Shongphu	Proportion	2000	47.2	70.8	22.8
Bhutan	Shongphu	Proportion	2017	62.8	82.4	36.5
Bhutan	Shongphu	Count	2000	0	1	0
Bhutan	Shongphu	Count	2017	1	1	0
Bhutan	Tsirangtoe	Proportion	2000	38.9	68.3	15.0
Bhutan	Tsirangtoe	Proportion	2017	60.5	83.4	31.0
Bhutan	Tsirangtoe	Count	2000	0	1	0
Bhutan	Tsirangtoe	Count	2017	1	1	0
Bhutan	Phobji	Proportion	2000	40.0	63.3	17.4
Bhutan	Phobji	Proportion	2017	53.6	78.9	28.7
Bhutan	Phobji	Count	2000	0	1	0
Bhutan	Phobji	Count	2017	1	1	0
Bhutan	Tsendagang	Proportion	2000	42.3	68.8	18.8
Bhutan	Tsendagang	Proportion	2017	63.1	82.8	37.9
Bhutan	Tsendagang	Count	2000	0	1	0
Bhutan	Tsendagang	Count	2017	1	1	0
Bhutan	Shermung	Proportion	2000	48.9	69.5	27.8
Bhutan	Shermung	Proportion	2017	63.1	82.6	39.8
Bhutan	Shermung	Count	2000	0	1	0
Bhutan	Shermung	Count	2017	1	1	0
Bhutan	Tendu	Proportion	2000	47.3	73.3	24.3
Bhutan	Tendu	Proportion	2017	61.7	80.3	39.8
Bhutan	Tendu	Count	2000	0	1	0
Bhutan	Tendu	Count	2017	1	1	0
Bhutan	Thrimshing	Proportion	2000	46.5	71.8	24.7
Bhutan	Thrimshing	Proportion	2017	60.3	81.0	34.7
Bhutan	Thrimshing	Count	2000	0	1	0
Bhutan	Thrimshing	Count	2017	1	1	0
Bhutan	Ruepisa	Proportion	2000	46.2	67.8	18.1
Bhutan	Ruepisa	Proportion	2017	61.1	81.6	37.0
Bhutan	Ruepisa	Count	2000	0	1	0
Bhutan	Ruepisa	Count	2017	1	1	0
Bhutan	Tseza	Proportion	2000	48.1	71.9	21.9
Bhutan	Tseza	Proportion	2017	64.9	84.6	39.8
Bhutan	Tseza	Count	2000	0	1	0
Bhutan	Tseza	Count	2017	1	1	0
Bhutan	Silambi	Proportion	2000	49.6	71.3	27.9
Bhutan	Silambi	Proportion	2017	66.0	83.6	40.7

Table 4: ORS by Unit *(continued)*

Country	Unit	Metric	Year	Mean	Upper	Lower
Bhutan	Silambi	Count	2000	0	1	0
Bhutan	Silambi	Count	2017	1	1	0
Bhutan	Ugentse	Proportion	2000	47.1	72.4	21.8
Bhutan	Ugentse	Proportion	2017	62.1	82.5	33.9
Bhutan	Ugentse	Count	2000	0	1	0
Bhutan	Ugentse	Count	2017	1	1	0
Bhutan	Udzorong	Proportion	2000	43.9	66.0	22.4
Bhutan	Udzorong	Proportion	2017	59.9	81.2	37.6
Bhutan	Udzorong	Count	2000	0	1	0
Bhutan	Udzorong	Count	2017	1	1	0
Bhutan	Sephu	Proportion	2000	41.7	63.7	21.0
Bhutan	Sephu	Proportion	2017	54.8	79.1	33.0
Bhutan	Sephu	Count	2000	0	1	0
Bhutan	Sephu	Count	2017	1	1	0
Bhutan	Thangrong	Proportion	2000	43.4	68.2	20.7
Bhutan	Thangrong	Proportion	2017	60.0	80.7	33.4
Bhutan	Thangrong	Count	2000	0	1	0
Bhutan	Thangrong	Count	2017	1	1	0
Bhutan	Yoeseltse	Proportion	2000	42.9	69.9	16.6
Bhutan	Yoeseltse	Proportion	2017	58.7	80.7	30.2
Bhutan	Yoeseltse	Count	2000	0	1	0
Bhutan	Yoeseltse	Count	2017	1	1	0
Bhutan	Yangnyer	Proportion	2000	44.4	66.7	21.3
Bhutan	Yangnyer	Proportion	2017	59.5	80.3	34.4
Bhutan	Yangnyer	Count	2000	0	1	0
Bhutan	Yangnyer	Count	2017	1	1	0
Bhutan	Thedtsho	Proportion	2000	46.2	68.4	17.8
Bhutan	Thedtsho	Proportion	2017	60.8	85.1	34.9
Bhutan	Thedtsho	Count	2000	0	1	0
Bhutan	Thedtsho	Count	2017	1	1	0
Bhutan	Tsakaling	Proportion	2000	47.0	70.1	24.0
Bhutan	Tsakaling	Proportion	2017	61.8	81.3	35.4
Bhutan	Tsakaling	Count	2000	0	1	0
Bhutan	Tsakaling	Count	2017	1	1	0
Bhutan	Tsamang	Proportion	2000	47.9	69.0	25.9
Bhutan	Tsamang	Proportion	2017	63.2	82.5	40.0
Bhutan	Tsamang	Count	2000	0	1	0
Bhutan	Tsamang	Count	2017	1	1	0
Bolivia	Belisario Boeto	Proportion	2000	25.5	33.5	19.1
Bolivia	Belisario Boeto	Proportion	2017	35.6	43.9	26.9
Bolivia	Belisario Boeto	Count	2000	0	0	0
Bolivia	Belisario Boeto	Count	2017	0	0	0
Bolivia	Arani	Proportion	2000	20.4	24.7	15.9
Bolivia	Arani	Proportion	2017	33.0	41.4	25.2
Bolivia	Arani	Count	2000	0	0	0
Bolivia	Arani	Count	2017	0	0	0
Bolivia	Cercado	Proportion	2000	21.8	29.2	15.3
Bolivia	Cercado	Proportion	2017	39.5	51.7	30.8
Bolivia	Cercado	Count	2000	0	0	0

Table 4: ORS by Unit *(continued)*

Country	Unit	Metric	Year	Mean	Upper	Lower
Bolivia	Cercado	Count	2017	0	1	0
Bolivia	Abel Iturralde	Proportion	2000	21.2	27.4	16.1
Bolivia	Abel Iturralde	Proportion	2017	43.3	52.5	35.0
Bolivia	Abel Iturralde	Count	2000	0	0	0
Bolivia	Abel Iturralde	Count	2017	0	1	0
Bolivia	Atahuallpa	Proportion	2000	22.8	30.2	16.1
Bolivia	Atahuallpa	Proportion	2017	27.1	36.3	19.9
Bolivia	Atahuallpa	Count	2000	0	0	0
Bolivia	Atahuallpa	Count	2017	0	0	0
Bolivia	Federico Román	Proportion	2000	24.9	32.4	19.5
Bolivia	Federico Román	Proportion	2017	46.3	55.6	37.3
Bolivia	Federico Román	Count	2000	0	0	0
Bolivia	Federico Román	Count	2017	0	1	0
Bolivia	Alonso de Ibáñez	Proportion	2000	26.5	33.4	20.9
Bolivia	Alonso de Ibáñez	Proportion	2017	32.2	40.1	24.6
Bolivia	Alonso de Ibáñez	Count	2000	0	0	0
Bolivia	Alonso de Ibáñez	Count	2017	0	0	0
Bolivia	Andrés Ibáñez	Proportion	2000	22.2	28.1	16.9
Bolivia	Andrés Ibáñez	Proportion	2017	38.8	46.4	30.8
Bolivia	Andrés Ibáñez	Count	2000	0	0	0
Bolivia	Andrés Ibáñez	Count	2017	0	0	0
Bolivia	Aniceto Arce	Proportion	2000	23.6	30.0	17.7
Bolivia	Aniceto Arce	Proportion	2017	34.9	44.5	27.0
Bolivia	Aniceto Arce	Count	2000	0	0	0
Bolivia	Aniceto Arce	Count	2017	0	0	0
Bolivia	Hernando Siles	Proportion	2000	26.5	34.2	19.9
Bolivia	Hernando Siles	Proportion	2017	37.4	46.8	28.8
Bolivia	Hernando Siles	Count	2000	0	0	0
Bolivia	Hernando Siles	Count	2017	0	0	0
Bolivia	Arque	Proportion	2000	24.6	30.7	19.2
Bolivia	Arque	Proportion	2017	31.8	40.3	24.8
Bolivia	Arque	Count	2000	0	0	0
Bolivia	Arque	Count	2017	0	0	0
Bolivia	José Ballivián	Proportion	2000	19.4	24.8	14.1
Bolivia	José Ballivián	Proportion	2017	37.1	47.2	28.5
Bolivia	José Ballivián	Count	2000	0	0	0
Bolivia	José Ballivián	Count	2017	0	0	0
Bolivia	Aroma	Proportion	2000	21.7	26.8	16.9
Bolivia	Aroma	Proportion	2017	27.7	36.3	20.6
Bolivia	Aroma	Count	2000	0	0	0
Bolivia	Aroma	Count	2017	0	0	0
Bolivia	Carangas	Proportion	2000	21.9	28.0	16.1
Bolivia	Carangas	Proportion	2017	27.2	35.9	20.3
Bolivia	Carangas	Count	2000	0	0	0
Bolivia	Carangas	Count	2017	0	0	0
Bolivia	Madre de Dios	Proportion	2000	23.6	30.1	17.6
Bolivia	Madre de Dios	Proportion	2017	46.2	55.2	38.0
Bolivia	Madre de Dios	Count	2000	0	0	0
Bolivia	Madre de Dios	Count	2017	0	1	0

Table 4: ORS by Unit *(continued)*

Country	Unit	Metric	Year	Mean	Upper	Lower
Bolivia	Antonio Quijarro	Proportion	2000	28.2	34.8	21.9
Bolivia	Antonio Quijarro	Proportion	2017	32.2	40.8	24.7
Bolivia	Antonio Quijarro	Count	2000	0	0	0
Bolivia	Antonio Quijarro	Count	2017	0	0	0
Bolivia	Chiquitos	Proportion	2000	21.8	26.9	16.6
Bolivia	Chiquitos	Proportion	2017	39.0	46.4	31.2
Bolivia	Chiquitos	Count	2000	0	0	0
Bolivia	Chiquitos	Count	2017	0	0	0
Bolivia	Burnet O'Connor	Proportion	2000	23.7	30.5	18.1
Bolivia	Burnet O'Connor	Proportion	2017	38.9	48.5	30.7
Bolivia	Burnet O'Connor	Count	2000	0	0	0
Bolivia	Burnet O'Connor	Count	2017	0	0	0
Bolivia	Jaime Zudáñez	Proportion	2000	26.6	32.3	20.6
Bolivia	Jaime Zudáñez	Proportion	2017	35.8	45.1	27.4
Bolivia	Jaime Zudáñez	Count	2000	0	0	0
Bolivia	Jaime Zudáñez	Count	2017	0	0	0
Bolivia	Ayopaya	Proportion	2000	19.5	23.9	15.6
Bolivia	Ayopaya	Proportion	2017	30.2	37.6	23.3
Bolivia	Ayopaya	Count	2000	0	0	0
Bolivia	Ayopaya	Count	2017	0	0	0
Bolivia	Mamoré	Proportion	2000	22.2	29.7	16.1
Bolivia	Mamoré	Proportion	2017	42.4	53.6	32.7
Bolivia	Mamoré	Count	2000	0	0	0
Bolivia	Mamoré	Count	2017	0	1	0
Bolivia	Bautista Saavedra	Proportion	2000	20.1	26.3	14.4
Bolivia	Bautista Saavedra	Proportion	2017	28.3	36.6	21.4
Bolivia	Bautista Saavedra	Count	2000	0	0	0
Bolivia	Bautista Saavedra	Count	2017	0	0	0
Bolivia	Cercado	Proportion	2000	22.5	28.3	17.5
Bolivia	Cercado	Proportion	2017	33.0	42.9	25.2
Bolivia	Cercado	Count	2000	0	0	0
Bolivia	Cercado	Count	2017	0	0	0
Bolivia	Manuripi	Proportion	2000	23.4	28.9	18.6
Bolivia	Manuripi	Proportion	2017	47.6	54.9	38.7
Bolivia	Manuripi	Count	2000	0	0	0
Bolivia	Manuripi	Count	2017	0	1	0
Bolivia	Bernardino Bilbao	Proportion	2000	28.3	34.3	22.3
Bolivia	Bernardino Bilbao	Proportion	2017	34.6	43.3	26.2
Bolivia	Bernardino Bilbao	Count	2000	0	0	0
Bolivia	Bernardino Bilbao	Count	2017	0	0	0
Bolivia	Cordillera	Proportion	2000	24.6	31.1	18.9
Bolivia	Cordillera	Proportion	2017	42.4	50.7	33.4
Bolivia	Cordillera	Count	2000	0	0	0
Bolivia	Cordillera	Count	2017	0	1	0
Bolivia	Cercado	Proportion	2000	22.1	28.4	16.0
Bolivia	Cercado	Proportion	2017	35.3	43.9	26.8
Bolivia	Cercado	Count	2000	0	0	0
Bolivia	Cercado	Count	2017	0	0	0
Bolivia	Juana Azurduay de Padilla	Proportion	2000	29.0	36.4	22.5

Table 4: ORS by Unit *(continued)*

Country	Unit	Metric	Year	Mean	Upper	Lower
Bolivia	Juana Azurduay de Padilla	Proportion	2017	38.4	47.0	30.3
Bolivia	Juana Azurduay de Padilla	Count	2000	0	0	0
Bolivia	Juana Azurduay de Padilla	Count	2017	0	0	0
Bolivia	Capinota	Proportion	2000	21.4	26.3	16.7
Bolivia	Capinota	Proportion	2017	32.9	40.6	25.5
Bolivia	Capinota	Count	2000	0	0	0
Bolivia	Capinota	Count	2017	0	0	0
Bolivia	Marbán	Proportion	2000	21.4	28.1	16.2
Bolivia	Marbán	Proportion	2017	41.8	50.9	32.9
Bolivia	Marbán	Count	2000	0	0	0
Bolivia	Marbán	Count	2017	0	1	0
Bolivia	Eliodoro Camacho	Proportion	2000	19.9	26.3	14.1
Bolivia	Eliodoro Camacho	Proportion	2017	24.4	31.4	18.4
Bolivia	Eliodoro Camacho	Count	2000	0	0	0
Bolivia	Eliodoro Camacho	Count	2017	0	0	0
Bolivia	Eduardo Avaroa	Proportion	2000	25.0	31.7	19.0
Bolivia	Eduardo Avaroa	Proportion	2017	32.7	42.0	25.2
Bolivia	Eduardo Avaroa	Count	2000	0	0	0
Bolivia	Eduardo Avaroa	Count	2017	0	0	0
Bolivia	Charcas	Proportion	2000	27.5	33.5	21.3
Bolivia	Charcas	Proportion	2017	35.5	43.9	27.5
Bolivia	Charcas	Count	2000	0	0	0
Bolivia	Charcas	Count	2017	0	0	0
Bolivia	Florida	Proportion	2000	22.1	28.6	15.7
Bolivia	Florida	Proportion	2017	36.2	44.7	26.9
Bolivia	Florida	Count	2000	0	0	0
Bolivia	Florida	Count	2017	0	0	0
Bolivia	Eustaquio Méndez	Proportion	2000	22.5	28.7	16.8
Bolivia	Eustaquio Méndez	Proportion	2017	33.9	42.6	26.2
Bolivia	Eustaquio Méndez	Count	2000	0	0	0
Bolivia	Eustaquio Méndez	Count	2017	0	0	0
Bolivia	Luis Calvo	Proportion	2000	26.3	32.8	20.0
Bolivia	Luis Calvo	Proportion	2017	41.9	51.4	33.5
Bolivia	Luis Calvo	Count	2000	0	0	0
Bolivia	Luis Calvo	Count	2017	0	1	0
Bolivia	Carrasco	Proportion	2000	21.0	26.1	16.4
Bolivia	Carrasco	Proportion	2017	39.2	48.0	30.6
Bolivia	Carrasco	Count	2000	0	0	0
Bolivia	Carrasco	Count	2017	0	0	0
Bolivia	Moxos	Proportion	2000	20.2	26.0	14.7
Bolivia	Moxos	Proportion	2017	38.0	48.2	28.7
Bolivia	Moxos	Count	2000	0	0	0
Bolivia	Moxos	Count	2017	0	0	0
Bolivia	Franz Tamayo	Proportion	2000	20.4	26.6	15.4
Bolivia	Franz Tamayo	Proportion	2017	34.0	43.0	26.1
Bolivia	Franz Tamayo	Count	2000	0	0	0
Bolivia	Franz Tamayo	Count	2017	0	0	0
Bolivia	Ladislao Cabrera	Proportion	2000	23.9	31.4	17.3
Bolivia	Ladislao Cabrera	Proportion	2017	29.4	38.4	21.9

Table 4: ORS by Unit *(continued)*

Country	Unit	Metric	Year	Mean	Upper	Lower
Bolivia	Ladislao Cabrera	Count	2000	0	0	0
Bolivia	Ladislao Cabrera	Count	2017	0	0	0
Bolivia	Chayanta	Proportion	2000	28.1	34.1	21.9
Bolivia	Chayanta	Proportion	2017	35.7	43.8	27.8
Bolivia	Chayanta	Count	2000	0	0	0
Bolivia	Chayanta	Count	2017	0	0	0
Bolivia	Ichilo	Proportion	2000	20.8	25.8	15.5
Bolivia	Ichilo	Proportion	2017	38.7	46.4	29.0
Bolivia	Ichilo	Count	2000	0	0	0
Bolivia	Ichilo	Count	2017	0	0	0
Bolivia	Gran Chaco	Proportion	2000	22.7	29.9	17.0
Bolivia	Gran Chaco	Proportion	2017	36.7	45.9	27.9
Bolivia	Gran Chaco	Count	2000	0	0	0
Bolivia	Gran Chaco	Count	2017	0	0	0
Bolivia	Nor Cinti	Proportion	2000	26.8	33.8	20.7
Bolivia	Nor Cinti	Proportion	2017	33.8	43.0	26.8
Bolivia	Nor Cinti	Count	2000	0	0	0
Bolivia	Nor Cinti	Count	2017	0	0	0
Bolivia	Cercado	Proportion	2000	20.6	25.4	16.0
Bolivia	Cercado	Proportion	2017	34.4	42.3	26.7
Bolivia	Cercado	Count	2000	0	0	0
Bolivia	Cercado	Count	2017	0	0	0
Bolivia	Vaca Díez	Proportion	2000	23.2	29.2	17.6
Bolivia	Vaca Díez	Proportion	2017	45.3	55.1	36.8
Bolivia	Vaca Díez	Count	2000	0	0	0
Bolivia	Vaca Díez	Count	2017	0	1	0
Bolivia	Gualberto Villarroel	Proportion	2000	22.1	27.4	16.6
Bolivia	Gualberto Villarroel	Proportion	2017	28.9	37.5	21.1
Bolivia	Gualberto Villarroel	Count	2000	0	0	0
Bolivia	Gualberto Villarroel	Count	2017	0	0	0
Bolivia	Litoral	Proportion	2000	22.9	30.9	16.3
Bolivia	Litoral	Proportion	2017	26.2	35.4	19.2
Bolivia	Litoral	Count	2000	0	0	0
Bolivia	Litoral	Count	2017	0	0	0
Bolivia	Cornelio Saavedra	Proportion	2000	28.4	34.5	22.7
Bolivia	Cornelio Saavedra	Proportion	2017	37.4	46.4	29.8
Bolivia	Cornelio Saavedra	Count	2000	0	0	0
Bolivia	Cornelio Saavedra	Count	2017	0	0	0
Bolivia	Ignacio Warnes	Proportion	2000	19.8	24.9	15.1
Bolivia	Ignacio Warnes	Proportion	2017	36.5	45.0	28.5
Bolivia	Ignacio Warnes	Count	2000	0	0	0
Bolivia	Ignacio Warnes	Count	2017	0	0	0
Bolivia	José María Avilés	Proportion	2000	22.3	28.3	16.5
Bolivia	José María Avilés	Proportion	2017	34.4	42.6	26.0
Bolivia	José María Avilés	Count	2000	0	0	0
Bolivia	José María Avilés	Count	2017	0	0	0
Bolivia	Oropeza	Proportion	2000	27.6	33.1	21.1
Bolivia	Oropeza	Proportion	2017	37.0	45.8	28.9
Bolivia	Oropeza	Count	2000	0	0	0

Table 4: ORS by Unit *(continued)*

Country	Unit	Metric	Year	Mean	Upper	Lower
Bolivia	Oropeza	Count	2017	0	0	0
Bolivia	Chapare	Proportion	2000	20.3	24.5	15.8
Bolivia	Chapare	Proportion	2017	32.1	39.3	24.9
Bolivia	Chapare	Count	2000	0	0	0
Bolivia	Chapare	Count	2017	0	0	0
Bolivia	Yacuma	Proportion	2000	21.4	27.1	16.2
Bolivia	Yacuma	Proportion	2017	41.0	49.9	31.2
Bolivia	Yacuma	Count	2000	0	0	0
Bolivia	Yacuma	Count	2017	0	0	0
Bolivia	Ingavi	Proportion	2000	21.9	26.4	16.7
Bolivia	Ingavi	Proportion	2017	26.9	34.4	20.2
Bolivia	Ingavi	Count	2000	0	0	0
Bolivia	Ingavi	Count	2017	0	0	0
Bolivia	Pantaleón Dalence	Proportion	2000	24.5	31.0	18.7
Bolivia	Pantaleón Dalence	Proportion	2017	33.7	43.4	26.1
Bolivia	Pantaleón Dalence	Count	2000	0	0	0
Bolivia	Pantaleón Dalence	Count	2017	0	0	0
Bolivia	Daniel Campos	Proportion	2000	26.2	34.3	19.3
Bolivia	Daniel Campos	Proportion	2017	31.8	41.5	23.1
Bolivia	Daniel Campos	Count	2000	0	0	0
Bolivia	Daniel Campos	Count	2017	0	0	0
Bolivia	José Miguel de Velasco	Proportion	2000	22.3	27.9	16.8
Bolivia	José Miguel de Velasco	Proportion	2017	39.8	48.3	31.6
Bolivia	José Miguel de Velasco	Count	2000	0	0	0
Bolivia	José Miguel de Velasco	Count	2017	0	0	0
Bolivia	Sud Cinti	Proportion	2000	25.8	31.8	20.1
Bolivia	Sud Cinti	Proportion	2017	36.2	45.2	28.9
Bolivia	Sud Cinti	Count	2000	0	0	0
Bolivia	Sud Cinti	Count	2017	0	0	0
Bolivia	Esteban Arce	Proportion	2000	21.9	26.5	17.1
Bolivia	Esteban Arce	Proportion	2017	32.5	40.4	25.1
Bolivia	Esteban Arce	Count	2000	0	0	0
Bolivia	Esteban Arce	Count	2017	0	0	0
Bolivia	Inquisivi	Proportion	2000	20.4	25.4	16.0
Bolivia	Inquisivi	Proportion	2017	29.5	37.3	21.8
Bolivia	Inquisivi	Count	2000	0	0	0
Bolivia	Inquisivi	Count	2017	0	0	0
Bolivia	Poopó	Proportion	2000	24.0	30.9	18.6
Bolivia	Poopó	Proportion	2017	31.3	40.9	24.0
Bolivia	Poopó	Count	2000	0	0	0
Bolivia	Poopó	Count	2017	0	0	0
Bolivia	José María Linares	Proportion	2000	28.9	35.2	22.8
Bolivia	José María Linares	Proportion	2017	37.1	46.2	28.9
Bolivia	José María Linares	Count	2000	0	0	0
Bolivia	José María Linares	Count	2017	0	0	0
Bolivia	Manuel María Caballero	Proportion	2000	22.2	27.7	16.9
Bolivia	Manuel María Caballero	Proportion	2017	35.4	43.4	26.7
Bolivia	Manuel María Caballero	Count	2000	0	0	0
Bolivia	Manuel María Caballero	Count	2017	0	0	0

Table 4: ORS by Unit *(continued)*

Country	Unit	Metric	Year	Mean	Upper	Lower
Bolivia	Tomina	Proportion	2000	27.1	34.3	20.6
Bolivia	Tomina	Proportion	2017	35.0	43.9	26.3
Bolivia	Tomina	Count	2000	0	0	0
Bolivia	Tomina	Count	2017	0	0	0
Bolivia	Germán Jordán	Proportion	2000	20.7	25.2	16.1
Bolivia	Germán Jordán	Proportion	2017	33.3	41.7	25.7
Bolivia	Germán Jordán	Count	2000	0	0	0
Bolivia	Germán Jordán	Count	2017	0	0	0
Bolivia	Larecaja	Proportion	2000	20.0	25.7	14.7
Bolivia	Larecaja	Proportion	2017	28.8	37.0	21.6
Bolivia	Larecaja	Count	2000	0	0	0
Bolivia	Larecaja	Count	2017	0	0	0
Bolivia	Sajama	Proportion	2000	20.9	26.9	15.2
Bolivia	Sajama	Proportion	2017	26.1	34.2	19.1
Bolivia	Sajama	Count	2000	0	0	0
Bolivia	Sajama	Count	2017	0	0	0
Bolivia	Modesto Omiste	Proportion	2000	27.9	34.6	21.7
Bolivia	Modesto Omiste	Proportion	2017	31.6	39.9	23.3
Bolivia	Modesto Omiste	Count	2000	0	0	0
Bolivia	Modesto Omiste	Count	2017	0	0	0
Bolivia	Ñuflo de Chávez	Proportion	2000	20.3	26.4	16.0
Bolivia	Ñuflo de Chávez	Proportion	2017	39.8	48.5	32.1
Bolivia	Ñuflo de Chávez	Count	2000	0	0	0
Bolivia	Ñuflo de Chávez	Count	2017	0	0	0
Bolivia	Yamparáez	Proportion	2000	28.1	34.6	22.0
Bolivia	Yamparáez	Proportion	2017	37.0	46.0	28.7
Bolivia	Yamparáez	Count	2000	0	0	0
Bolivia	Yamparáez	Count	2017	0	0	0
Bolivia	Mizque	Proportion	2000	21.9	26.9	17.0
Bolivia	Mizque	Proportion	2017	32.2	39.6	24.4
Bolivia	Mizque	Count	2000	0	0	0
Bolivia	Mizque	Count	2017	0	0	0
Bolivia	Loayza	Proportion	2000	22.0	26.8	17.4
Bolivia	Loayza	Proportion	2017	29.3	38.5	21.0
Bolivia	Loayza	Count	2000	0	0	0
Bolivia	Loayza	Count	2017	0	0	0
Bolivia	Saucarí	Proportion	2000	23.0	28.8	17.2
Bolivia	Saucarí	Proportion	2017	29.1	38.3	21.3
Bolivia	Saucarí	Count	2000	0	0	0
Bolivia	Saucarí	Count	2017	0	0	0
Bolivia	Nor Chichas	Proportion	2000	28.0	35.3	21.9
Bolivia	Nor Chichas	Proportion	2017	33.2	42.1	24.8
Bolivia	Nor Chichas	Count	2000	0	0	0
Bolivia	Nor Chichas	Count	2017	0	0	0
Bolivia	Obispo Santistevan	Proportion	2000	20.2	25.5	14.9
Bolivia	Obispo Santistevan	Proportion	2017	36.4	45.0	28.2
Bolivia	Obispo Santistevan	Count	2000	0	0	0
Bolivia	Obispo Santistevan	Count	2017	0	0	0
Bolivia	Narciso Campero	Proportion	2000	23.4	28.5	17.8

Table 4: ORS by Unit *(continued)*

Country	Unit	Metric	Year	Mean	Upper	Lower
Bolivia	Narciso Campero	Proportion	2017	35.6	43.9	28.2
Bolivia	Narciso Campero	Count	2000	0	0	0
Bolivia	Narciso Campero	Count	2017	0	0	0
Bolivia	Los Andes	Proportion	2000	20.5	25.3	15.7
Bolivia	Los Andes	Proportion	2017	26.6	33.5	19.8
Bolivia	Los Andes	Count	2000	0	0	0
Bolivia	Los Andes	Count	2017	0	0	0
Bolivia	Nor Lípez	Proportion	2000	28.0	35.6	20.6
Bolivia	Nor Lípez	Proportion	2017	34.1	43.2	25.3
Bolivia	Nor Lípez	Count	2000	0	0	0
Bolivia	Nor Lípez	Count	2017	0	0	0
Bolivia	Sara	Proportion	2000	20.8	26.3	15.3
Bolivia	Sara	Proportion	2017	40.9	49.4	31.8
Bolivia	Sara	Count	2000	0	0	0
Bolivia	Sara	Count	2017	0	0	0
Bolivia	Punata	Proportion	2000	20.5	25.0	16.0
Bolivia	Punata	Proportion	2017	31.3	39.2	24.0
Bolivia	Punata	Count	2000	0	0	0
Bolivia	Punata	Count	2017	0	0	0
Bolivia	Manco Kapac	Proportion	2000	23.0	28.6	17.0
Bolivia	Manco Kapac	Proportion	2017	23.4	31.0	17.6
Bolivia	Manco Kapac	Count	2000	0	0	0
Bolivia	Manco Kapac	Count	2017	0	0	0
Bolivia	Rafael Bustillo	Proportion	2000	27.0	33.8	20.9
Bolivia	Rafael Bustillo	Proportion	2017	34.4	44.1	26.7
Bolivia	Rafael Bustillo	Count	2000	0	0	0
Bolivia	Rafael Bustillo	Count	2017	0	0	0
Bolivia	Vallegrande	Proportion	2000	22.6	29.0	16.4
Bolivia	Vallegrande	Proportion	2017	35.2	43.4	26.6
Bolivia	Vallegrande	Count	2000	0	0	0
Bolivia	Vallegrande	Count	2017	0	0	0
Bolivia	Quillacollo	Proportion	2000	20.1	24.5	15.9
Bolivia	Quillacollo	Proportion	2017	31.8	39.3	24.4
Bolivia	Quillacollo	Count	2000	0	0	0
Bolivia	Quillacollo	Count	2017	0	0	0
Bolivia	Muñecas	Proportion	2000	20.2	26.2	13.9
Bolivia	Muñecas	Proportion	2017	25.6	33.3	19.4
Bolivia	Muñecas	Count	2000	0	0	0
Bolivia	Muñecas	Count	2017	0	0	0
Bolivia	Sud Chichas	Proportion	2000	27.8	34.5	20.7
Bolivia	Sud Chichas	Proportion	2017	31.3	41.0	22.9
Bolivia	Sud Chichas	Count	2000	0	0	0
Bolivia	Sud Chichas	Count	2017	0	0	0
Bolivia	Tapacarí	Proportion	2000	21.3	26.8	16.6
Bolivia	Tapacarí	Proportion	2017	31.5	40.1	24.0
Bolivia	Tapacarí	Count	2000	0	0	0
Bolivia	Tapacarí	Count	2017	0	0	0
Bolivia	Nor Yungas	Proportion	2000	20.2	25.9	15.2
Bolivia	Nor Yungas	Proportion	2017	31.2	38.9	23.2

Table 4: ORS by Unit *(continued)*

Country	Unit	Metric	Year	Mean	Upper	Lower
Bolivia	Nor Yungas	Count	2000	0	0	0
Bolivia	Nor Yungas	Count	2017	0	0	0
Bolivia	Sud Lípez	Proportion	2000	29.9	37.1	22.3
Bolivia	Sud Lípez	Proportion	2017	39.1	49.1	29.3
Bolivia	Sud Lípez	Count	2000	0	0	0
Bolivia	Sud Lípez	Count	2017	0	0	0
Bolivia	Omasuyos	Proportion	2000	19.9	25.0	14.6
Bolivia	Omasuyos	Proportion	2017	23.7	31.1	17.3
Bolivia	Omasuyos	Count	2000	0	0	0
Bolivia	Omasuyos	Count	2017	0	0	0
Bolivia	Tomás Frías	Proportion	2000	30.5	37.2	24.5
Bolivia	Tomás Frías	Proportion	2017	35.6	44.3	27.6
Bolivia	Tomás Frías	Count	2000	0	0	0
Bolivia	Tomás Frías	Count	2017	0	0	0
Bolivia	Pacajes	Proportion	2000	21.8	27.4	16.2
Bolivia	Pacajes	Proportion	2017	26.2	33.2	19.9
Bolivia	Pacajes	Count	2000	0	0	0
Bolivia	Pacajes	Count	2017	0	0	0
Bolivia	Pedro Domingo Murillo	Proportion	2000	21.7	26.7	16.4
Bolivia	Pedro Domingo Murillo	Proportion	2017	28.7	36.1	21.5
Bolivia	Pedro Domingo Murillo	Count	2000	0	0	0
Bolivia	Pedro Domingo Murillo	Count	2017	0	0	0
Bolivia	Sud Yungas	Proportion	2000	19.8	24.7	15.4
Bolivia	Sud Yungas	Proportion	2017	30.7	38.4	22.6
Bolivia	Sud Yungas	Count	2000	0	0	0
Bolivia	Sud Yungas	Count	2017	0	0	0
Botswana	Bobonong	Proportion	2000	51.5	58.9	43.5
Botswana	Bobonong	Proportion	2017	48.1	61.8	34.9
Botswana	Bobonong	Count	2000	1	1	0
Botswana	Bobonong	Count	2017	0	1	0
Botswana	Chobe	Proportion	2000	53.6	62.7	44.4
Botswana	Chobe	Proportion	2017	50.5	63.8	37.2
Botswana	Chobe	Count	2000	1	1	0
Botswana	Chobe	Count	2017	1	1	0
Botswana	Francistown	Proportion	2000	52.1	60.6	43.6
Botswana	Francistown	Proportion	2017	48.1	61.5	35.4
Botswana	Francistown	Count	2000	1	1	0
Botswana	Francistown	Count	2017	0	1	0
Botswana	Gaborone	Proportion	2000	50.4	59.0	42.6
Botswana	Gaborone	Proportion	2017	48.1	60.3	35.5
Botswana	Gaborone	Count	2000	1	1	0
Botswana	Gaborone	Count	2017	0	1	0
Botswana	Ghanzi	Proportion	2000	50.9	60.4	40.6
Botswana	Ghanzi	Proportion	2017	50.4	62.7	38.5
Botswana	Ghanzi	Count	2000	1	1	0
Botswana	Ghanzi	Count	2017	1	1	0
Botswana	Jwaneng	Proportion	2000	48.8	57.9	40.2
Botswana	Jwaneng	Proportion	2017	46.8	59.1	35.0
Botswana	Jwaneng	Count	2000	0	1	0

Table 4: ORS by Unit *(continued)*

Country	Unit	Metric	Year	Mean	Upper	Lower
Botswana	Jwaneng	Count	2017	0	1	0
Botswana	Gemsbok	Proportion	2000	50.1	60.9	38.7
Botswana	Gemsbok	Proportion	2017	48.2	60.3	37.4
Botswana	Gemsbok	Count	2000	1	1	0
Botswana	Gemsbok	Count	2017	0	1	0
Botswana	Kgatleng	Proportion	2000	50.1	58.8	42.6
Botswana	Kgatleng	Proportion	2017	48.1	60.4	35.7
Botswana	Kgatleng	Count	2000	1	1	0
Botswana	Kgatleng	Count	2017	0	1	0
Botswana	Kweneng North	Proportion	2000	49.4	58.0	41.7
Botswana	Kweneng North	Proportion	2017	47.7	60.1	34.8
Botswana	Kweneng North	Count	2000	0	1	0
Botswana	Kweneng North	Count	2017	0	1	0
Botswana	Lobatse	Proportion	2000	48.9	59.3	40.0
Botswana	Lobatse	Proportion	2017	47.4	59.6	34.6
Botswana	Lobatse	Count	2000	0	1	0
Botswana	Lobatse	Count	2017	0	1	0
Botswana	Masungu	Proportion	2000	51.2	59.7	42.8
Botswana	Masungu	Proportion	2017	47.7	61.2	35.0
Botswana	Masungu	Count	2000	1	1	0
Botswana	Masungu	Count	2017	0	1	0
Botswana	Ngamiland East	Proportion	2000	51.3	60.0	43.3
Botswana	Ngamiland East	Proportion	2017	49.9	64.5	35.7
Botswana	Ngamiland East	Count	2000	1	1	0
Botswana	Ngamiland East	Count	2017	0	1	0
Botswana	Selibe Phikwe	Proportion	2000	51.8	59.5	43.5
Botswana	Selibe Phikwe	Proportion	2017	47.9	61.1	34.7
Botswana	Selibe Phikwe	Count	2000	1	1	0
Botswana	Selibe Phikwe	Count	2017	0	1	0
Botswana	South East	Proportion	2000	49.2	58.4	41.1
Botswana	South East	Proportion	2017	47.7	60.0	35.2
Botswana	South East	Count	2000	0	1	0
Botswana	South East	Count	2017	0	1	0
Botswana	Barolong	Proportion	2000	48.3	59.0	38.9
Botswana	Barolong	Proportion	2017	47.3	59.6	34.4
Botswana	Barolong	Count	2000	0	1	0
Botswana	Barolong	Count	2017	0	1	0
Botswana	Sowa	Proportion	2000	50.9	59.1	43.1
Botswana	Sowa	Proportion	2017	47.9	61.1	34.0
Botswana	Sowa	Count	2000	1	1	0
Botswana	Sowa	Count	2017	0	1	0
Botswana	Lethlakane	Proportion	2000	51.2	58.8	43.7
Botswana	Lethlakane	Proportion	2017	48.9	61.4	35.7
Botswana	Lethlakane	Count	2000	1	1	0
Botswana	Lethlakane	Count	2017	0	1	0
Botswana	Hukunsti	Proportion	2000	51.2	61.7	41.4
Botswana	Hukunsti	Proportion	2017	49.5	60.7	37.3
Botswana	Hukunsti	Count	2000	1	1	0
Botswana	Hukunsti	Count	2017	0	1	0

Table 4: ORS by Unit *(continued)*

Country	Unit	Metric	Year	Mean	Upper	Lower
Botswana	Kweneng South	Proportion	2000	49.3	57.4	41.3
Botswana	Kweneng South	Proportion	2017	47.9	59.7	35.8
Botswana	Kweneng South	Count	2000	0	1	0
Botswana	Kweneng South	Count	2017	0	1	0
Botswana	Ngamiland West	Proportion	2000	48.8	57.7	40.4
Botswana	Ngamiland West	Proportion	2017	50.0	63.2	37.1
Botswana	Ngamiland West	Count	2000	0	1	0
Botswana	Ngamiland West	Count	2017	0	1	0
Botswana	Ngwaketse Central	Proportion	2000	48.1	57.9	39.6
Botswana	Ngwaketse Central	Proportion	2017	47.2	59.0	34.6
Botswana	Ngwaketse Central	Count	2000	0	1	0
Botswana	Ngwaketse Central	Count	2017	0	1	0
Botswana	Machaneng	Proportion	2000	50.6	58.1	42.0
Botswana	Machaneng	Proportion	2017	48.0	61.5	35.1
Botswana	Machaneng	Count	2000	1	1	0
Botswana	Machaneng	Count	2017	0	1	0
Botswana	Tshabong	Proportion	2000	50.2	60.6	39.9
Botswana	Tshabong	Proportion	2017	48.2	60.1	36.9
Botswana	Tshabong	Count	2000	1	1	0
Botswana	Tshabong	Count	2017	0	1	0
Botswana	Ngwaketse North	Proportion	2000	48.4	57.1	40.3
Botswana	Ngwaketse North	Proportion	2017	47.4	58.9	35.1
Botswana	Ngwaketse North	Count	2000	0	1	0
Botswana	Ngwaketse North	Count	2017	0	1	0
Botswana	Mahalapye	Proportion	2000	50.5	58.2	42.5
Botswana	Mahalapye	Proportion	2017	48.0	61.0	35.9
Botswana	Mahalapye	Count	2000	1	1	0
Botswana	Mahalapye	Count	2017	0	1	0
Botswana	Ngwaketse South	Proportion	2000	48.4	58.5	39.1
Botswana	Ngwaketse South	Proportion	2017	47.4	59.6	34.7
Botswana	Ngwaketse South	Count	2000	0	1	0
Botswana	Ngwaketse South	Count	2017	0	1	0
Botswana	Palapye	Proportion	2000	50.4	57.6	42.0
Botswana	Palapye	Proportion	2017	47.5	60.6	34.7
Botswana	Palapye	Count	2000	1	1	0
Botswana	Palapye	Count	2017	0	1	0
Botswana	Serowe	Proportion	2000	50.7	57.2	44.0
Botswana	Serowe	Proportion	2017	48.0	60.2	35.3
Botswana	Serowe	Count	2000	1	1	0
Botswana	Serowe	Count	2017	0	1	0
Botswana	Tuli	Proportion	2000	51.4	58.6	43.3
Botswana	Tuli	Proportion	2017	48.5	61.6	35.6
Botswana	Tuli	Count	2000	1	1	0
Botswana	Tuli	Count	2017	0	1	0
Botswana	Tutume	Proportion	2000	50.7	58.3	43.0
Botswana	Tutume	Proportion	2017	48.2	60.5	35.0
Botswana	Tutume	Count	2000	1	1	0
Botswana	Tutume	Count	2017	0	1	0
Burkina Faso	Balé	Proportion	2000	15.7	31.2	6.5

Table 4: ORS by Unit *(continued)*

Country	Unit	Metric	Year	Mean	Upper	Lower
Burkina Faso	Balé	Proportion	2017	19.3	37.4	6.7
Burkina Faso	Balé	Count	2000	0	0	0
Burkina Faso	Balé	Count	2017	0	0	0
Burkina Faso	Comoé	Proportion	2000	20.2	37.7	10.0
Burkina Faso	Comoé	Proportion	2017	22.4	43.2	10.0
Burkina Faso	Comoé	Count	2000	0	0	0
Burkina Faso	Comoé	Count	2017	0	0	0
Burkina Faso	Boulgou	Proportion	2000	20.4	38.8	9.9
Burkina Faso	Boulgou	Proportion	2017	17.8	34.2	6.4
Burkina Faso	Boulgou	Count	2000	0	0	0
Burkina Faso	Boulgou	Count	2017	0	0	0
Burkina Faso	Bam	Proportion	2000	12.4	27.5	4.5
Burkina Faso	Bam	Proportion	2017	17.0	38.9	6.0
Burkina Faso	Bam	Count	2000	0	0	0
Burkina Faso	Bam	Count	2017	0	0	0
Burkina Faso	Boulkiemdé	Proportion	2000	15.8	32.0	6.3
Burkina Faso	Boulkiemdé	Proportion	2017	18.5	37.4	6.8
Burkina Faso	Boulkiemdé	Count	2000	0	0	0
Burkina Faso	Boulkiemdé	Count	2017	0	0	0
Burkina Faso	Bazèga	Proportion	2000	16.6	34.3	6.4
Burkina Faso	Bazèga	Proportion	2017	18.5	37.0	6.5
Burkina Faso	Bazèga	Count	2000	0	0	0
Burkina Faso	Bazèga	Count	2017	0	0	0
Burkina Faso	Kadiogo	Proportion	2000	16.9	36.4	5.8
Burkina Faso	Kadiogo	Proportion	2017	19.4	40.2	7.3
Burkina Faso	Kadiogo	Count	2000	0	0	0
Burkina Faso	Kadiogo	Count	2017	0	0	0
Burkina Faso	Gnagna	Proportion	2000	14.3	28.0	6.3
Burkina Faso	Gnagna	Proportion	2017	16.6	36.9	5.2
Burkina Faso	Gnagna	Count	2000	0	0	0
Burkina Faso	Gnagna	Count	2017	0	0	0
Burkina Faso	Houet	Proportion	2000	17.2	33.1	6.4
Burkina Faso	Houet	Proportion	2017	20.6	40.4	7.8
Burkina Faso	Houet	Count	2000	0	0	0
Burkina Faso	Houet	Count	2017	0	0	0
Burkina Faso	Loroum	Proportion	2000	10.4	21.6	3.7
Burkina Faso	Loroum	Proportion	2017	16.9	34.8	6.2
Burkina Faso	Loroum	Count	2000	0	0	0
Burkina Faso	Loroum	Count	2017	0	0	0
Burkina Faso	Ganzourgou	Proportion	2000	17.4	36.2	6.8
Burkina Faso	Ganzourgou	Proportion	2017	17.0	34.1	5.1
Burkina Faso	Ganzourgou	Count	2000	0	0	0
Burkina Faso	Ganzourgou	Count	2017	0	0	0
Burkina Faso	Oudalan	Proportion	2000	8.9	18.0	3.6
Burkina Faso	Oudalan	Proportion	2017	16.9	33.4	5.6
Burkina Faso	Oudalan	Count	2000	0	0	0
Burkina Faso	Oudalan	Count	2017	0	0	0
Burkina Faso	Bougouriba	Proportion	2000	16.8	34.3	6.7
Burkina Faso	Bougouriba	Proportion	2017	20.8	40.6	8.3

Table 4: ORS by Unit *(continued)*

Country	Unit	Metric	Year	Mean	Upper	Lower
Burkina Faso	Bougouriba	Count	2000	0	0	0
Burkina Faso	Bougouriba	Count	2017	0	0	0
Burkina Faso	Banwa	Proportion	2000	14.0	27.8	6.2
Burkina Faso	Banwa	Proportion	2017	17.8	33.6	6.4
Burkina Faso	Banwa	Count	2000	0	0	0
Burkina Faso	Banwa	Count	2017	0	0	0
Burkina Faso	Léraba	Proportion	2000	20.0	34.1	9.8
Burkina Faso	Léraba	Proportion	2017	19.8	40.7	7.6
Burkina Faso	Léraba	Count	2000	0	0	0
Burkina Faso	Léraba	Count	2017	0	0	0
Burkina Faso	Koulpélogo	Proportion	2000	20.0	36.3	9.4
Burkina Faso	Koulpélogo	Proportion	2017	15.7	29.1	6.4
Burkina Faso	Koulpélogo	Count	2000	0	0	0
Burkina Faso	Koulpélogo	Count	2017	0	0	0
Burkina Faso	Namentenga	Proportion	2000	13.4	28.9	5.1
Burkina Faso	Namentenga	Proportion	2017	17.0	35.4	5.3
Burkina Faso	Namentenga	Count	2000	0	0	0
Burkina Faso	Namentenga	Count	2017	0	0	0
Burkina Faso	Sanguié	Proportion	2000	15.1	30.0	6.1
Burkina Faso	Sanguié	Proportion	2017	18.0	34.6	7.4
Burkina Faso	Sanguié	Count	2000	0	0	0
Burkina Faso	Sanguié	Count	2017	0	0	0
Burkina Faso	Nahouri	Proportion	2000	20.2	38.8	7.7
Burkina Faso	Nahouri	Proportion	2017	20.3	39.5	7.5
Burkina Faso	Nahouri	Count	2000	0	0	0
Burkina Faso	Nahouri	Count	2017	0	0	0
Burkina Faso	Gourma	Proportion	2000	18.0	33.3	8.4
Burkina Faso	Gourma	Proportion	2017	15.1	29.5	6.1
Burkina Faso	Gourma	Count	2000	0	0	0
Burkina Faso	Gourma	Count	2017	0	0	0
Burkina Faso	Kénédougou	Proportion	2000	17.2	32.4	7.6
Burkina Faso	Kénédougou	Proportion	2017	18.7	37.5	6.5
Burkina Faso	Kénédougou	Count	2000	0	0	0
Burkina Faso	Kénédougou	Count	2017	0	0	0
Burkina Faso	Passoré	Proportion	2000	13.9	30.1	5.2
Burkina Faso	Passoré	Proportion	2017	17.3	36.0	6.1
Burkina Faso	Passoré	Count	2000	0	0	0
Burkina Faso	Passoré	Count	2017	0	0	0
Burkina Faso	Kourwéogo	Proportion	2000	15.0	33.2	5.3
Burkina Faso	Kourwéogo	Proportion	2017	18.0	40.5	6.6
Burkina Faso	Kourwéogo	Count	2000	0	0	0
Burkina Faso	Kourwéogo	Count	2017	0	0	0
Burkina Faso	Séno	Proportion	2000	10.1	21.2	4.0
Burkina Faso	Séno	Proportion	2017	17.4	35.8	6.6
Burkina Faso	Séno	Count	2000	0	0	0
Burkina Faso	Séno	Count	2017	0	0	0
Burkina Faso	Ioba	Proportion	2000	16.8	34.1	6.5
Burkina Faso	Ioba	Proportion	2017	20.9	41.0	8.0
Burkina Faso	Ioba	Count	2000	0	0	0

Table 4: ORS by Unit *(continued)*

Country	Unit	Metric	Year	Mean	Upper	Lower
Burkina Faso	Ioba	Count	2017	0	0	0
Burkina Faso	Kossi	Proportion	2000	11.0	23.5	4.6
Burkina Faso	Kossi	Proportion	2017	16.6	32.6	6.3
Burkina Faso	Kossi	Count	2000	0	0	0
Burkina Faso	Kossi	Count	2017	0	0	0
Burkina Faso	Kouritenga	Proportion	2000	18.7	38.5	7.8
Burkina Faso	Kouritenga	Proportion	2017	17.1	33.4	5.6
Burkina Faso	Kouritenga	Count	2000	0	0	0
Burkina Faso	Kouritenga	Count	2017	0	0	0
Burkina Faso	Sanmatenga	Proportion	2000	13.3	27.7	4.7
Burkina Faso	Sanmatenga	Proportion	2017	17.1	36.4	5.3
Burkina Faso	Sanmatenga	Count	2000	0	0	0
Burkina Faso	Sanmatenga	Count	2017	0	0	0
Burkina Faso	Sissili	Proportion	2000	16.6	33.2	6.5
Burkina Faso	Sissili	Proportion	2017	20.5	40.3	8.1
Burkina Faso	Sissili	Count	2000	0	0	0
Burkina Faso	Sissili	Count	2017	0	0	0
Burkina Faso	Zoundwéogo	Proportion	2000	18.7	38.8	7.4
Burkina Faso	Zoundwéogo	Proportion	2017	18.3	35.4	6.0
Burkina Faso	Zoundwéogo	Count	2000	0	0	0
Burkina Faso	Zoundwéogo	Count	2017	0	0	0
Burkina Faso	Komandjoari	Proportion	2000	14.0	25.2	6.3
Burkina Faso	Komandjoari	Proportion	2017	15.2	30.7	4.9
Burkina Faso	Komandjoari	Count	2000	0	0	0
Burkina Faso	Komandjoari	Count	2017	0	0	0
Burkina Faso	Tuy	Proportion	2000	16.5	34.1	6.9
Burkina Faso	Tuy	Proportion	2017	20.2	39.9	8.0
Burkina Faso	Tuy	Count	2000	0	0	0
Burkina Faso	Tuy	Count	2017	0	0	0
Burkina Faso	Yatenga	Proportion	2000	11.7	25.2	4.5
Burkina Faso	Yatenga	Proportion	2017	17.2	32.9	6.6
Burkina Faso	Yatenga	Count	2000	0	0	0
Burkina Faso	Yatenga	Count	2017	0	0	0
Burkina Faso	Oubritenga	Proportion	2000	15.2	32.4	5.4
Burkina Faso	Oubritenga	Proportion	2017	17.6	37.9	5.9
Burkina Faso	Oubritenga	Count	2000	0	0	0
Burkina Faso	Oubritenga	Count	2017	0	0	0
Burkina Faso	Soum	Proportion	2000	9.5	19.3	3.7
Burkina Faso	Soum	Proportion	2017	16.4	36.2	6.3
Burkina Faso	Soum	Count	2000	0	0	0
Burkina Faso	Soum	Count	2017	0	0	0
Burkina Faso	Noumbiel	Proportion	2000	16.0	32.4	6.7
Burkina Faso	Noumbiel	Proportion	2017	23.8	45.0	9.8
Burkina Faso	Noumbiel	Count	2000	0	0	0
Burkina Faso	Noumbiel	Count	2017	0	0	0
Burkina Faso	Mouhoun	Proportion	2000	14.0	28.4	5.7
Burkina Faso	Mouhoun	Proportion	2017	18.1	35.0	6.7
Burkina Faso	Mouhoun	Count	2000	0	0	0
Burkina Faso	Mouhoun	Count	2017	0	0	0

Table 4: ORS by Unit *(continued)*

Country	Unit	Metric	Year	Mean	Upper	Lower
Burkina Faso	Ziro	Proportion	2000	16.0	33.3	6.0
Burkina Faso	Ziro	Proportion	2017	19.6	37.8	6.6
Burkina Faso	Ziro	Count	2000	0	0	0
Burkina Faso	Ziro	Count	2017	0	0	0
Burkina Faso	Kompienga	Proportion	2000	16.8	33.2	7.8
Burkina Faso	Kompienga	Proportion	2017	12.8	23.9	5.8
Burkina Faso	Kompienga	Count	2000	0	0	0
Burkina Faso	Kompienga	Count	2017	0	0	0
Burkina Faso	Zondoma	Proportion	2000	12.6	28.0	4.8
Burkina Faso	Zondoma	Proportion	2017	17.0	34.9	6.5
Burkina Faso	Zondoma	Count	2000	0	0	0
Burkina Faso	Zondoma	Count	2017	0	0	0
Burkina Faso	Yagha	Proportion	2000	11.6	22.6	5.2
Burkina Faso	Yagha	Proportion	2017	16.3	34.5	5.8
Burkina Faso	Yagha	Count	2000	0	0	0
Burkina Faso	Yagha	Count	2017	0	0	0
Burkina Faso	Poni	Proportion	2000	16.6	31.6	6.9
Burkina Faso	Poni	Proportion	2017	22.5	43.6	9.1
Burkina Faso	Poni	Count	2000	0	0	0
Burkina Faso	Poni	Count	2017	0	0	0
Burkina Faso	Nayala	Proportion	2000	12.8	27.1	5.0
Burkina Faso	Nayala	Proportion	2017	17.0	34.6	6.6
Burkina Faso	Nayala	Count	2000	0	0	0
Burkina Faso	Nayala	Count	2017	0	0	0
Burkina Faso	Tapoa	Proportion	2000	13.2	23.4	5.8
Burkina Faso	Tapoa	Proportion	2017	11.6	21.8	5.0
Burkina Faso	Tapoa	Count	2000	0	0	0
Burkina Faso	Tapoa	Count	2017	0	0	0
Burkina Faso	Sourou	Proportion	2000	11.4	22.9	4.7
Burkina Faso	Sourou	Proportion	2017	16.9	31.5	6.7
Burkina Faso	Sourou	Count	2000	0	0	0
Burkina Faso	Sourou	Count	2017	0	0	0
Burundi	Bubanza	Proportion	2000	28.8	39.5	19.5
Burundi	Bubanza	Proportion	2017	39.7	53.5	27.3
Burundi	Bubanza	Count	2000	0	0	0
Burundi	Bubanza	Count	2017	0	1	0
Burundi	Buterere	Proportion	2000	36.0	50.1	23.7
Burundi	Buterere	Proportion	2017	38.3	56.4	23.2
Burundi	Buterere	Count	2000	0	1	0
Burundi	Buterere	Count	2017	0	1	0
Burundi	Bugarama	Proportion	2000	30.6	41.6	21.0
Burundi	Bugarama	Proportion	2017	27.9	40.0	18.7
Burundi	Bugarama	Count	2000	0	0	0
Burundi	Bugarama	Count	2017	0	0	0
Burundi	Burambi	Proportion	2000	32.3	44.2	22.0
Burundi	Burambi	Proportion	2017	29.6	40.7	19.5
Burundi	Burambi	Count	2000	0	0	0
Burundi	Burambi	Count	2017	0	0	0
Burundi	Cankuzo	Proportion	2000	25.8	36.4	18.0

Table 4: ORS by Unit *(continued)*

Country	Unit	Metric	Year	Mean	Upper	Lower
Burundi	Cankuzo	Proportion	2017	37.0	48.8	25.0
Burundi	Cankuzo	Count	2000	0	0	0
Burundi	Cankuzo	Count	2017	0	0	0
Burundi	Buganda	Proportion	2000	27.4	36.9	18.8
Burundi	Buganda	Proportion	2017	41.1	54.2	28.1
Burundi	Buganda	Count	2000	0	0	0
Burundi	Buganda	Count	2017	0	1	0
Burundi	Bugendana	Proportion	2000	20.7	29.8	14.6
Burundi	Bugendana	Proportion	2017	30.1	42.0	19.9
Burundi	Bugendana	Count	2000	0	0	0
Burundi	Bugendana	Count	2017	0	0	0
Burundi	Bugenyuzi	Proportion	2000	18.9	25.6	12.6
Burundi	Bugenyuzi	Proportion	2017	29.5	41.4	19.4
Burundi	Bugenyuzi	Count	2000	0	0	0
Burundi	Bugenyuzi	Count	2017	0	0	0
Burundi	Butaganzwa1	Proportion	2000	18.8	26.7	12.2
Burundi	Butaganzwa1	Proportion	2017	26.2	38.5	16.7
Burundi	Butaganzwa1	Count	2000	0	0	0
Burundi	Butaganzwa1	Count	2017	0	0	0
Burundi	Bugabira	Proportion	2000	17.9	24.9	12.2
Burundi	Bugabira	Proportion	2017	35.6	48.0	23.7
Burundi	Bugabira	Count	2000	0	0	0
Burundi	Bugabira	Count	2017	0	0	0
Burundi	Kayogoro	Proportion	2000	34.2	46.1	23.0
Burundi	Kayogoro	Proportion	2017	36.1	49.4	24.6
Burundi	Kayogoro	Count	2000	0	0	0
Burundi	Kayogoro	Count	2017	0	0	0
Burundi	Bukeye	Proportion	2000	24.3	34.1	17.1
Burundi	Bukeye	Proportion	2017	28.3	39.7	18.6
Burundi	Bukeye	Count	2000	0	0	0
Burundi	Bukeye	Count	2017	0	0	0
Burundi	Buhinyuza	Proportion	2000	21.0	30.6	13.7
Burundi	Buhinyuza	Proportion	2017	35.7	48.1	23.6
Burundi	Buhinyuza	Count	2000	0	0	0
Burundi	Buhinyuza	Count	2017	0	0	0
Burundi	Bisoro	Proportion	2000	28.2	39.7	17.9
Burundi	Bisoro	Proportion	2017	27.4	39.0	16.8
Burundi	Bisoro	Count	2000	0	0	0
Burundi	Bisoro	Count	2017	0	0	0
Burundi	Busiga	Proportion	2000	19.2	26.8	12.9
Burundi	Busiga	Proportion	2017	34.6	47.4	23.6
Burundi	Busiga	Count	2000	0	0	0
Burundi	Busiga	Count	2017	0	0	0
Burundi	Bukemba	Proportion	2000	35.5	48.1	23.6
Burundi	Bukemba	Proportion	2017	38.6	52.6	24.9
Burundi	Bukemba	Count	2000	0	0	0
Burundi	Bukemba	Count	2017	0	1	0
Burundi	Butaganzwa2	Proportion	2000	28.6	39.0	19.3
Burundi	Butaganzwa2	Proportion	2017	36.6	48.0	25.5

Table 4: ORS by Unit *(continued)*

Country	Unit	Metric	Year	Mean	Upper	Lower
Burundi	Butaganzwa2	Count	2000	0	0	0
Burundi	Butaganzwa2	Count	2017	0	0	0
Burundi	Gihanga	Proportion	2000	31.2	42.2	22.3
Burundi	Gihanga	Proportion	2017	38.7	52.6	26.7
Burundi	Gihanga	Count	2000	0	0	0
Burundi	Gihanga	Count	2017	0	1	0
Burundi	Buyenze	Proportion	2000	36.7	52.5	22.7
Burundi	Buyenze	Proportion	2017	38.7	59.6	22.7
Burundi	Buyenze	Count	2000	0	1	0
Burundi	Buyenze	Count	2017	0	1	0
Burundi	Isale	Proportion	2000	29.9	41.1	20.1
Burundi	Isale	Proportion	2017	31.6	44.7	20.8
Burundi	Isale	Count	2000	0	0	0
Burundi	Isale	Count	2017	0	0	0
Burundi	Bururi	Proportion	2000	33.2	44.7	22.9
Burundi	Bururi	Proportion	2017	30.3	44.8	20.6
Burundi	Bururi	Count	2000	0	0	0
Burundi	Bururi	Count	2017	0	0	0
Burundi	Cendajuru	Proportion	2000	27.2	38.0	17.8
Burundi	Cendajuru	Proportion	2017	36.3	49.4	24.1
Burundi	Cendajuru	Count	2000	0	0	0
Burundi	Cendajuru	Count	2017	0	0	0
Burundi	Bukinanyana	Proportion	2000	23.7	31.3	15.9
Burundi	Bukinanyana	Proportion	2017	37.3	49.2	25.8
Burundi	Bukinanyana	Count	2000	0	0	0
Burundi	Bukinanyana	Count	2017	0	0	0
Burundi	Bukirasazi	Proportion	2000	30.1	44.3	19.0
Burundi	Bukirasazi	Proportion	2017	35.2	49.5	22.5
Burundi	Bukirasazi	Count	2000	0	0	0
Burundi	Bukirasazi	Count	2017	0	0	0
Burundi	Buhiga	Proportion	2000	19.3	27.4	12.4
Burundi	Buhiga	Proportion	2017	29.6	40.4	20.2
Burundi	Buhiga	Count	2000	0	0	0
Burundi	Buhiga	Count	2017	0	0	0
Burundi	Gahombo	Proportion	2000	17.4	25.6	11.5
Burundi	Gahombo	Proportion	2017	28.6	41.8	17.9
Burundi	Gahombo	Count	2000	0	0	0
Burundi	Gahombo	Count	2017	0	0	0
Burundi	Busoni	Proportion	2000	19.2	25.9	13.8
Burundi	Busoni	Proportion	2017	38.6	50.9	27.3
Burundi	Busoni	Count	2000	0	0	0
Burundi	Busoni	Count	2017	0	1	0
Burundi	Kibago	Proportion	2000	34.5	46.9	22.9
Burundi	Kibago	Proportion	2017	35.9	51.4	23.5
Burundi	Kibago	Count	2000	0	0	0
Burundi	Kibago	Count	2017	0	1	0
Burundi	Kiganda	Proportion	2000	22.6	34.7	15.3
Burundi	Kiganda	Proportion	2017	23.2	33.3	14.1
Burundi	Kiganda	Count	2000	0	0	0

Table 4: ORS by Unit *(continued)*

Country	Unit	Metric	Year	Mean	Upper	Lower
Burundi	Kiganda	Count	2017	0	0	0
Burundi	Butihinda	Proportion	2000	19.6	26.1	13.5
Burundi	Butihinda	Proportion	2017	36.6	47.7	25.5
Burundi	Butihinda	Count	2000	0	0	0
Burundi	Butihinda	Count	2017	0	0	0
Burundi	Gisozi	Proportion	2000	24.6	37.4	15.8
Burundi	Gisozi	Proportion	2017	23.3	34.1	13.7
Burundi	Gisozi	Count	2000	0	0	0
Burundi	Gisozi	Count	2017	0	0	0
Burundi	Gashikanwa	Proportion	2000	18.8	26.2	12.7
Burundi	Gashikanwa	Proportion	2017	33.8	46.3	21.7
Burundi	Gashikanwa	Count	2000	0	0	0
Burundi	Gashikanwa	Count	2017	0	0	0
Burundi	Giharo	Proportion	2000	32.9	44.7	22.6
Burundi	Giharo	Proportion	2017	40.3	54.0	27.5
Burundi	Giharo	Count	2000	0	0	0
Burundi	Giharo	Count	2017	0	1	0
Burundi	Butezi	Proportion	2000	25.7	34.9	17.6
Burundi	Butezi	Proportion	2017	36.2	47.6	25.3
Burundi	Butezi	Count	2000	0	0	0
Burundi	Butezi	Count	2017	0	0	0
Burundi	Mpanda	Proportion	2000	31.0	42.9	20.4
Burundi	Mpanda	Proportion	2017	38.2	52.7	25.4
Burundi	Mpanda	Count	2000	0	0	0
Burundi	Mpanda	Count	2017	0	1	0
Burundi	Bwiza	Proportion	2000	36.7	51.2	24.2
Burundi	Bwiza	Proportion	2017	38.6	58.2	23.1
Burundi	Bwiza	Count	2000	0	1	0
Burundi	Bwiza	Count	2017	0	1	0
Burundi	Kabezi	Proportion	2000	30.1	41.6	20.3
Burundi	Kabezi	Proportion	2017	30.4	43.8	19.9
Burundi	Kabezi	Count	2000	0	0	0
Burundi	Kabezi	Count	2017	0	0	0
Burundi	Buyengero	Proportion	2000	32.2	44.0	21.1
Burundi	Buyengero	Proportion	2017	29.6	40.9	19.4
Burundi	Buyengero	Count	2000	0	0	0
Burundi	Buyengero	Count	2017	0	0	0
Burundi	Gisagara	Proportion	2000	26.8	38.2	17.8
Burundi	Gisagara	Proportion	2017	35.2	47.0	24.1
Burundi	Gisagara	Count	2000	0	0	0
Burundi	Gisagara	Count	2017	0	0	0
Burundi	Mabayi	Proportion	2000	23.0	30.7	15.3
Burundi	Mabayi	Proportion	2017	38.5	52.8	26.6
Burundi	Mabayi	Count	2000	0	0	0
Burundi	Mabayi	Count	2017	0	1	0
Burundi	Buraza	Proportion	2000	31.4	43.9	20.7
Burundi	Buraza	Proportion	2017	33.5	45.7	22.2
Burundi	Buraza	Count	2000	0	0	0
Burundi	Buraza	Count	2017	0	0	0

Table 4: ORS by Unit *(continued)*

Country	Unit	Metric	Year	Mean	Upper	Lower
Burundi	Gihogazi	Proportion	2000	20.3	29.5	13.7
Burundi	Gihogazi	Proportion	2017	31.6	44.6	20.8
Burundi	Gihogazi	Count	2000	0	0	0
Burundi	Gihogazi	Count	2017	0	0	0
Burundi	Gatara	Proportion	2000	20.1	28.9	13.7
Burundi	Gatara	Proportion	2017	32.2	44.2	20.9
Burundi	Gatara	Count	2000	0	0	0
Burundi	Gatara	Count	2017	0	0	0
Burundi	Bwambarangwe	Proportion	2000	19.3	27.4	12.6
Burundi	Bwambarangwe	Proportion	2017	39.4	53.5	26.5
Burundi	Bwambarangwe	Count	2000	0	0	0
Burundi	Bwambarangwe	Count	2017	0	1	0
Burundi	Lake Tanganyika	Proportion	2000	33.7	48.3	21.8
Burundi	Lake Tanganyika	Proportion	2017	39.2	56.2	24.8
Burundi	Lake Tanganyika	Count	2000	0	0	0
Burundi	Lake Tanganyika	Count	2017	0	1	0
Burundi	Mbuye	Proportion	2000	20.6	29.8	13.3
Burundi	Mbuye	Proportion	2017	24.1	34.6	15.0
Burundi	Mbuye	Count	2000	0	0	0
Burundi	Mbuye	Count	2017	0	0	0
Burundi	Gashoho	Proportion	2000	19.7	27.3	13.1
Burundi	Gashoho	Proportion	2017	35.6	49.4	24.6
Burundi	Gashoho	Count	2000	0	0	0
Burundi	Gashoho	Count	2017	0	0	0
Burundi	Kayokwe	Proportion	2000	23.9	34.4	14.7
Burundi	Kayokwe	Proportion	2017	25.5	37.0	15.6
Burundi	Kayokwe	Count	2000	0	0	0
Burundi	Kayokwe	Count	2017	0	0	0
Burundi	Kiremba	Proportion	2000	18.7	26.1	12.7
Burundi	Kiremba	Proportion	2017	32.9	44.7	21.9
Burundi	Kiremba	Count	2000	0	0	0
Burundi	Kiremba	Count	2017	0	0	0
Burundi	Gitanga	Proportion	2000	35.4	47.6	24.0
Burundi	Gitanga	Proportion	2017	33.6	47.0	22.0
Burundi	Gitanga	Count	2000	0	0	0
Burundi	Gitanga	Count	2017	0	0	0
Burundi	Bweru	Proportion	2000	26.7	36.2	18.0
Burundi	Bweru	Proportion	2017	37.8	50.3	26.6
Burundi	Bweru	Count	2000	0	0	0
Burundi	Bweru	Count	2017	0	1	0
Burundi	Musigati	Proportion	2000	25.8	34.9	18.5
Burundi	Musigati	Proportion	2017	37.1	49.7	26.3
Burundi	Musigati	Count	2000	0	0	0
Burundi	Musigati	Count	2017	0	0	0
Burundi	Cibitoke	Proportion	2000	35.7	50.8	22.8
Burundi	Cibitoke	Proportion	2017	37.7	55.6	22.9
Burundi	Cibitoke	Count	2000	0	1	0
Burundi	Cibitoke	Count	2017	0	1	0
Burundi	Kanyosha1	Proportion	2000	30.9	42.6	20.7

Table 4: ORS by Unit *(continued)*

Country	Unit	Metric	Year	Mean	Upper	Lower
Burundi	Kanyosha1	Proportion	2017	31.0	44.4	18.4
Burundi	Kanyosha1	Count	2000	0	0	0
Burundi	Kanyosha1	Count	2017	0	0	0
Burundi	Lake Tanganyika	Proportion	2000	34.1	48.0	22.1
Burundi	Lake Tanganyika	Proportion	2017	36.7	50.7	25.1
Burundi	Lake Tanganyika	Count	2000	0	0	0
Burundi	Lake Tanganyika	Count	2017	0	1	0
Burundi	Kigamba	Proportion	2000	23.1	32.5	15.7
Burundi	Kigamba	Proportion	2017	35.4	47.5	24.8
Burundi	Kigamba	Count	2000	0	0	0
Burundi	Kigamba	Count	2017	0	0	0
Burundi	Mugina	Proportion	2000	22.4	29.5	15.2
Burundi	Mugina	Proportion	2017	36.1	49.5	24.4
Burundi	Mugina	Count	2000	0	0	0
Burundi	Mugina	Count	2017	0	0	0
Burundi	Giheta	Proportion	2000	22.3	31.0	14.8
Burundi	Giheta	Proportion	2017	30.2	41.4	19.2
Burundi	Giheta	Count	2000	0	0	0
Burundi	Giheta	Count	2017	0	0	0
Burundi	Gitaramuka	Proportion	2000	19.7	27.0	13.6
Burundi	Gitaramuka	Proportion	2017	31.8	43.8	22.1
Burundi	Gitaramuka	Count	2000	0	0	0
Burundi	Gitaramuka	Count	2017	0	0	0
Burundi	Kabarore	Proportion	2000	20.2	27.2	14.0
Burundi	Kabarore	Proportion	2017	34.4	48.1	22.5
Burundi	Kabarore	Count	2000	0	0	0
Burundi	Kabarore	Count	2017	0	0	0
Burundi	Gitobe	Proportion	2000	18.3	25.2	12.4
Burundi	Gitobe	Proportion	2017	36.1	48.0	25.1
Burundi	Gitobe	Count	2000	0	0	0
Burundi	Gitobe	Count	2017	0	0	0
Burundi	Mabanda	Proportion	2000	33.3	46.4	22.4
Burundi	Mabanda	Proportion	2017	33.9	50.1	22.1
Burundi	Mabanda	Count	2000	0	0	0
Burundi	Mabanda	Count	2017	0	1	0
Burundi	Muramvya	Proportion	2000	26.0	36.1	17.9
Burundi	Muramvya	Proportion	2017	27.6	38.3	17.9
Burundi	Muramvya	Count	2000	0	0	0
Burundi	Muramvya	Count	2017	0	0	0
Burundi	Gasorwe	Proportion	2000	19.6	27.4	12.9
Burundi	Gasorwe	Proportion	2017	34.2	45.5	23.6
Burundi	Gasorwe	Count	2000	0	0	0
Burundi	Gasorwe	Count	2017	0	0	0
Burundi	Ndava	Proportion	2000	22.0	32.6	14.9
Burundi	Ndava	Proportion	2017	25.2	35.3	15.5
Burundi	Ndava	Count	2000	0	0	0
Burundi	Ndava	Count	2017	0	0	0
Burundi	Marangara	Proportion	2000	18.7	25.5	13.6
Burundi	Marangara	Proportion	2017	34.5	47.0	22.6

Table 4: ORS by Unit *(continued)*

Country	Unit	Metric	Year	Mean	Upper	Lower
Burundi	Marangara	Count	2000	0	0	0
Burundi	Marangara	Count	2017	0	0	0
Burundi	Mpinga-Kayove	Proportion	2000	32.0	42.8	21.4
Burundi	Mpinga-Kayove	Proportion	2017	37.9	50.2	25.5
Burundi	Mpinga-Kayove	Count	2000	0	0	0
Burundi	Mpinga-Kayove	Count	2017	0	1	0
Burundi	Gisuru	Proportion	2000	28.9	39.7	19.3
Burundi	Gisuru	Proportion	2017	38.0	50.3	25.5
Burundi	Gisuru	Count	2000	0	0	0
Burundi	Gisuru	Count	2017	0	1	0
Burundi	Rugazi	Proportion	2000	28.3	40.0	19.6
Burundi	Rugazi	Proportion	2017	34.0	46.8	23.0
Burundi	Rugazi	Count	2000	0	0	0
Burundi	Rugazi	Count	2017	0	0	0
Burundi	Gihosha	Proportion	2000	34.1	51.8	20.4
Burundi	Gihosha	Proportion	2017	35.9	54.0	19.7
Burundi	Gihosha	Count	2000	0	1	0
Burundi	Gihosha	Count	2017	0	1	0
Burundi	Lake Tanganyika	Proportion	2000	33.2	45.6	21.7
Burundi	Lake Tanganyika	Proportion	2017	35.2	49.2	23.6
Burundi	Lake Tanganyika	Count	2000	0	0	0
Burundi	Lake Tanganyika	Count	2017	0	0	0
Burundi	Matana	Proportion	2000	32.5	43.7	21.5
Burundi	Matana	Proportion	2017	29.7	42.1	19.2
Burundi	Matana	Count	2000	0	0	0
Burundi	Matana	Count	2017	0	0	0
Burundi	Mishiha	Proportion	2000	25.1	36.4	16.9
Burundi	Mishiha	Proportion	2017	36.5	49.4	25.4
Burundi	Mishiha	Count	2000	0	0	0
Burundi	Mishiha	Count	2017	0	0	0
Burundi	Murwi	Proportion	2000	25.3	33.9	17.1
Burundi	Murwi	Proportion	2017	38.6	52.1	26.6
Burundi	Murwi	Count	2000	0	0	0
Burundi	Murwi	Count	2017	0	1	0
Burundi	Gishubi	Proportion	2000	28.8	40.8	18.7
Burundi	Gishubi	Proportion	2017	32.8	45.5	21.7
Burundi	Gishubi	Count	2000	0	0	0
Burundi	Gishubi	Count	2017	0	0	0
Burundi	Mutumba	Proportion	2000	21.5	29.9	14.9
Burundi	Mutumba	Proportion	2017	32.9	45.2	22.1
Burundi	Mutumba	Count	2000	0	0	0
Burundi	Mutumba	Count	2017	0	0	0
Burundi	Kayanza	Proportion	2000	23.3	32.0	16.2
Burundi	Kayanza	Proportion	2017	37.0	51.0	23.7
Burundi	Kayanza	Count	2000	0	0	0
Burundi	Kayanza	Count	2017	0	1	0
Burundi	Kirundo	Proportion	2000	18.4	26.2	12.4
Burundi	Kirundo	Proportion	2017	35.7	48.8	24.8
Burundi	Kirundo	Count	2000	0	0	0

Table 4: ORS by Unit *(continued)*

Country	Unit	Metric	Year	Mean	Upper	Lower
Burundi	Kirundo	Count	2017	0	0	0
Burundi	Makamba	Proportion	2000	33.8	45.0	22.6
Burundi	Makamba	Proportion	2017	33.3	47.9	21.2
Burundi	Makamba	Count	2000	0	0	0
Burundi	Makamba	Count	2017	0	0	0
Burundi	Rutegama	Proportion	2000	21.8	30.1	14.3
Burundi	Rutegama	Proportion	2017	24.5	35.1	15.0
Burundi	Rutegama	Count	2000	0	0	0
Burundi	Rutegama	Count	2017	0	0	0
Burundi	Giteranyi	Proportion	2000	20.6	27.0	13.8
Burundi	Giteranyi	Proportion	2017	40.0	52.3	26.5
Burundi	Giteranyi	Count	2000	0	0	0
Burundi	Giteranyi	Count	2017	0	1	0
Burundi	Nyabihanga	Proportion	2000	24.3	35.7	16.6
Burundi	Nyabihanga	Proportion	2017	29.0	42.2	17.2
Burundi	Nyabihanga	Count	2000	0	0	0
Burundi	Nyabihanga	Count	2017	0	0	0
Burundi	Mwumba	Proportion	2000	18.7	27.1	12.2
Burundi	Mwumba	Proportion	2017	33.9	47.6	21.2
Burundi	Mwumba	Count	2000	0	0	0
Burundi	Mwumba	Count	2017	0	0	0
Burundi	Musongati	Proportion	2000	31.9	44.4	21.1
Burundi	Musongati	Proportion	2017	36.8	49.0	24.9
Burundi	Musongati	Count	2000	0	0	0
Burundi	Musongati	Count	2017	0	0	0
Burundi	Kinyinya	Proportion	2000	31.1	43.7	21.1
Burundi	Kinyinya	Proportion	2017	39.6	53.3	27.4
Burundi	Kinyinya	Count	2000	0	0	0
Burundi	Kinyinya	Count	2017	0	1	0
Burundi	Kamenge	Proportion	2000	34.1	51.9	19.7
Burundi	Kamenge	Proportion	2017	35.8	54.6	19.4
Burundi	Kamenge	Count	2000	0	1	0
Burundi	Kamenge	Count	2017	0	1	0
Burundi	Mubimbi	Proportion	2000	28.5	40.8	19.7
Burundi	Mubimbi	Proportion	2017	31.5	43.8	20.3
Burundi	Mubimbi	Count	2000	0	0	0
Burundi	Mubimbi	Count	2017	0	0	0
Burundi	Mugamba	Proportion	2000	28.8	40.1	19.2
Burundi	Mugamba	Proportion	2017	25.0	35.7	16.0
Burundi	Mugamba	Count	2000	0	0	0
Burundi	Mugamba	Count	2017	0	0	0
Burundi	Rugombo	Proportion	2000	25.9	34.9	17.8
Burundi	Rugombo	Proportion	2017	40.3	54.4	28.1
Burundi	Rugombo	Count	2000	0	0	0
Burundi	Rugombo	Count	2017	0	1	0
Burundi	Gitega	Proportion	2000	24.1	35.4	15.9
Burundi	Gitega	Proportion	2017	32.5	46.5	21.5
Burundi	Gitega	Count	2000	0	0	0
Burundi	Gitega	Count	2017	0	0	0

Table 4: ORS by Unit *(continued)*

Country	Unit	Metric	Year	Mean	Upper	Lower
Burundi	Nyabikere	Proportion	2000	22.7	32.2	15.4
Burundi	Nyabikere	Proportion	2017	33.8	46.4	22.7
Burundi	Nyabikere	Count	2000	0	0	0
Burundi	Nyabikere	Count	2017	0	0	0
Burundi	Matongo	Proportion	2000	22.9	32.3	16.1
Burundi	Matongo	Proportion	2017	31.9	43.1	21.3
Burundi	Matongo	Count	2000	0	0	0
Burundi	Matongo	Count	2017	0	0	0
Burundi	Ntega	Proportion	2000	18.5	25.3	13.2
Burundi	Ntega	Proportion	2017	35.1	47.9	23.5
Burundi	Ntega	Count	2000	0	0	0
Burundi	Ntega	Count	2017	0	0	0
Burundi	Nyanza-Lac	Proportion	2000	34.5	47.7	22.6
Burundi	Nyanza-Lac	Proportion	2017	37.6	52.8	26.2
Burundi	Nyanza-Lac	Count	2000	0	0	0
Burundi	Nyanza-Lac	Count	2017	0	1	0
Burundi	Muyinga	Proportion	2000	20.4	28.6	13.7
Burundi	Muyinga	Proportion	2017	35.9	48.7	23.9
Burundi	Muyinga	Count	2000	0	0	0
Burundi	Muyinga	Count	2017	0	0	0
Burundi	Rusaka	Proportion	2000	21.6	32.3	14.1
Burundi	Rusaka	Proportion	2017	21.4	31.0	13.1
Burundi	Rusaka	Count	2000	0	0	0
Burundi	Rusaka	Count	2017	0	0	0
Burundi	Ngozi	Proportion	2000	20.5	28.6	13.9
Burundi	Ngozi	Proportion	2017	32.8	45.9	21.5
Burundi	Ngozi	Count	2000	0	0	0
Burundi	Ngozi	Count	2017	0	0	0
Burundi	Rutana	Proportion	2000	34.5	46.2	23.1
Burundi	Rutana	Proportion	2017	35.6	48.7	24.4
Burundi	Rutana	Count	2000	0	0	0
Burundi	Rutana	Count	2017	0	0	0
Burundi	Nyabitsinda	Proportion	2000	28.7	40.4	18.6
Burundi	Nyabitsinda	Proportion	2017	37.9	50.5	27.3
Burundi	Nyabitsinda	Count	2000	0	0	0
Burundi	Nyabitsinda	Count	2017	0	1	0
Burundi	Kanyosha2	Proportion	2000	34.9	54.0	21.1
Burundi	Kanyosha2	Proportion	2017	35.1	51.6	21.5
Burundi	Kanyosha2	Count	2000	0	1	0
Burundi	Kanyosha2	Count	2017	0	1	0
Burundi	Mugongomanga	Proportion	2000	26.2	38.2	17.6
Burundi	Mugongomanga	Proportion	2017	24.8	35.5	15.5
Burundi	Mugongomanga	Count	2000	0	0	0
Burundi	Mugongomanga	Count	2017	0	0	0
Burundi	Rumonge	Proportion	2000	35.0	48.9	23.1
Burundi	Rumonge	Proportion	2017	35.4	48.8	23.5
Burundi	Rumonge	Count	2000	0	0	0
Burundi	Rumonge	Count	2017	0	0	0
Burundi	Itaba	Proportion	2000	28.6	40.1	18.2

Table 4: ORS by Unit *(continued)*

Country	Unit	Metric	Year	Mean	Upper	Lower
Burundi	Itaba	Proportion	2017	35.8	49.4	24.4
Burundi	Itaba	Count	2000	0	0	0
Burundi	Itaba	Count	2017	0	0	0
Burundi	Shombo	Proportion	2000	21.5	29.6	14.2
Burundi	Shombo	Proportion	2017	33.0	46.5	22.0
Burundi	Shombo	Count	2000	0	0	0
Burundi	Shombo	Count	2017	0	0	0
Burundi	Muhanga	Proportion	2000	18.5	26.0	12.6
Burundi	Muhanga	Proportion	2017	29.1	41.8	19.1
Burundi	Muhanga	Count	2000	0	0	0
Burundi	Muhanga	Count	2017	0	0	0
Burundi	Vumbi	Proportion	2000	18.6	25.4	12.8
Burundi	Vumbi	Proportion	2017	34.3	47.2	24.4
Burundi	Vumbi	Count	2000	0	0	0
Burundi	Vumbi	Count	2017	0	0	0
Burundi	Vugizo	Proportion	2000	32.8	44.1	21.2
Burundi	Vugizo	Proportion	2017	31.1	46.2	21.0
Burundi	Vugizo	Count	2000	0	0	0
Burundi	Vugizo	Count	2017	0	0	0
Burundi	Mwakiro	Proportion	2000	20.7	30.0	13.4
Burundi	Mwakiro	Proportion	2017	33.1	45.8	21.9
Burundi	Mwakiro	Count	2000	0	0	0
Burundi	Mwakiro	Count	2017	0	0	0
Burundi	Nyamurenza	Proportion	2000	18.8	26.3	12.2
Burundi	Nyamurenza	Proportion	2017	34.6	48.9	22.0
Burundi	Nyamurenza	Count	2000	0	0	0
Burundi	Nyamurenza	Count	2017	0	0	0
Burundi	Ruyigi	Proportion	2000	28.0	38.4	18.8
Burundi	Ruyigi	Proportion	2017	37.2	48.9	25.2
Burundi	Ruyigi	Count	2000	0	0	0
Burundi	Ruyigi	Count	2017	0	0	0
Burundi	Kinama	Proportion	2000	35.0	49.0	23.3
Burundi	Kinama	Proportion	2017	36.3	52.5	23.2
Burundi	Kinama	Count	2000	0	0	0
Burundi	Kinama	Count	2017	0	1	0
Burundi	Muhuta	Proportion	2000	29.6	40.1	19.4
Burundi	Muhuta	Proportion	2017	27.2	39.6	17.4
Burundi	Muhuta	Count	2000	0	0	0
Burundi	Muhuta	Count	2017	0	0	0
Burundi	Rutovu	Proportion	2000	34.1	45.1	23.0
Burundi	Rutovu	Proportion	2017	31.6	43.4	21.1
Burundi	Rutovu	Count	2000	0	0	0
Burundi	Rutovu	Count	2017	0	0	0
Burundi	Makebuko	Proportion	2000	27.1	38.6	17.6
Burundi	Makebuko	Proportion	2017	33.6	45.8	21.7
Burundi	Makebuko	Count	2000	0	0	0
Burundi	Makebuko	Count	2017	0	0	0
Burundi	Muruta	Proportion	2000	22.4	30.1	15.0
Burundi	Muruta	Proportion	2017	36.5	50.7	24.6

Table 4: ORS by Unit *(continued)*

Country	Unit	Metric	Year	Mean	Upper	Lower
Burundi	Muruta	Count	2000	0	0	0
Burundi	Muruta	Count	2017	0	1	0
Burundi	Ruhororo	Proportion	2000	19.9	27.3	13.8
Burundi	Ruhororo	Proportion	2017	33.7	47.4	23.9
Burundi	Ruhororo	Count	2000	0	0	0
Burundi	Ruhororo	Count	2017	0	0	0
Burundi	Kinindo	Proportion	2000	36.2	53.4	21.1
Burundi	Kinindo	Proportion	2017	37.1	55.2	21.2
Burundi	Kinindo	Count	2000	0	1	0
Burundi	Kinindo	Count	2017	0	1	0
Burundi	Mukike	Proportion	2000	27.5	39.0	18.0
Burundi	Mukike	Proportion	2017	24.7	35.7	15.3
Burundi	Mukike	Count	2000	0	0	0
Burundi	Mukike	Count	2017	0	0	0
Burundi	Songa	Proportion	2000	32.1	45.3	20.7
Burundi	Songa	Proportion	2017	28.8	42.2	18.6
Burundi	Songa	Count	2000	0	0	0
Burundi	Songa	Count	2017	0	0	0
Burundi	Mutaho	Proportion	2000	19.9	28.2	13.3
Burundi	Mutaho	Proportion	2017	30.4	42.7	20.1
Burundi	Mutaho	Count	2000	0	0	0
Burundi	Mutaho	Count	2017	0	0	0
Burundi	Rango	Proportion	2000	18.6	26.0	12.5
Burundi	Rango	Proportion	2017	24.4	34.4	16.0
Burundi	Rango	Count	2000	0	0	0
Burundi	Rango	Count	2017	0	0	0
Burundi	Tangara	Proportion	2000	18.3	24.5	12.7
Burundi	Tangara	Proportion	2017	30.8	42.7	20.6
Burundi	Tangara	Count	2000	0	0	0
Burundi	Tangara	Count	2017	0	0	0
Burundi	Lake Tanganyika	Proportion	2000	36.1	51.4	23.8
Burundi	Lake Tanganyika	Proportion	2017	37.5	54.2	23.7
Burundi	Lake Tanganyika	Count	2000	0	1	0
Burundi	Lake Tanganyika	Count	2017	0	1	0
Burundi	Mutambu	Proportion	2000	29.3	40.8	20.0
Burundi	Mutambu	Proportion	2017	27.2	39.4	17.0
Burundi	Mutambu	Count	2000	0	0	0
Burundi	Mutambu	Count	2017	0	0	0
Burundi	Vyanda	Proportion	2000	33.6	45.5	23.3
Burundi	Vyanda	Proportion	2017	32.0	45.4	21.0
Burundi	Vyanda	Count	2000	0	0	0
Burundi	Vyanda	Count	2017	0	0	0
Burundi	Nyanrusange	Proportion	2000	27.1	39.1	18.2
Burundi	Nyanrusange	Proportion	2017	30.2	42.9	20.3
Burundi	Nyanrusange	Count	2000	0	0	0
Burundi	Nyanrusange	Count	2017	0	0	0
Burundi	Musaga	Proportion	2000	34.4	48.7	22.7
Burundi	Musaga	Proportion	2017	35.4	50.1	22.7
Burundi	Musaga	Count	2000	0	0	0

Table 4: ORS by Unit *(continued)*

Country	Unit	Metric	Year	Mean	Upper	Lower
Burundi	Musaga	Count	2017	0	1	0
Burundi	Mutimbuzi	Proportion	2000	33.4	45.0	23.3
Burundi	Mutimbuzi	Proportion	2017	37.1	51.1	25.4
Burundi	Mutimbuzi	Count	2000	0	0	0
Burundi	Mutimbuzi	Count	2017	0	1	0
Burundi	Ryansoro	Proportion	2000	32.0	44.2	21.4
Burundi	Ryansoro	Proportion	2017	31.8	44.2	20.4
Burundi	Ryansoro	Count	2000	0	0	0
Burundi	Ryansoro	Count	2017	0	0	0
Burundi	Ngagara	Proportion	2000	36.4	51.5	22.6
Burundi	Ngagara	Proportion	2017	38.5	59.6	22.8
Burundi	Ngagara	Count	2000	0	1	0
Burundi	Ngagara	Count	2017	0	1	0
Burundi	Nyabiraba	Proportion	2000	28.3	39.0	19.5
Burundi	Nyabiraba	Proportion	2017	27.2	39.2	16.0
Burundi	Nyabiraba	Count	2000	0	0	0
Burundi	Nyabiraba	Count	2017	0	0	0
Burundi	Nyakabiga	Proportion	2000	33.6	48.6	21.9
Burundi	Nyakabiga	Proportion	2017	35.5	51.0	21.4
Burundi	Nyakabiga	Count	2000	0	0	0
Burundi	Nyakabiga	Count	2017	0	1	0
Burundi	Roherero	Proportion	2000	34.9	50.3	22.0
Burundi	Roherero	Proportion	2017	36.4	53.7	22.3
Burundi	Roherero	Count	2000	0	1	0
Burundi	Roherero	Count	2017	0	1	0
Cambodia	Malai	Proportion	2000	17.8	25.8	11.7
Cambodia	Malai	Proportion	2017	34.1	47.6	21.1
Cambodia	Malai	Count	2000	0	0	0
Cambodia	Malai	Count	2017	0	0	0
Cambodia	Aek Phnum	Proportion	2000	20.1	27.3	12.6
Cambodia	Aek Phnum	Proportion	2017	36.5	50.7	23.3
Cambodia	Aek Phnum	Count	2000	0	0	0
Cambodia	Aek Phnum	Count	2017	0	1	0
Cambodia	Batheay	Proportion	2000	16.7	23.0	11.7
Cambodia	Batheay	Proportion	2017	38.3	54.7	23.6
Cambodia	Batheay	Count	2000	0	0	0
Cambodia	Batheay	Count	2017	0	1	0
Cambodia	Baribour	Proportion	2000	13.4	19.9	8.5
Cambodia	Baribour	Proportion	2017	44.2	63.3	30.7
Cambodia	Baribour	Count	2000	0	0	0
Cambodia	Baribour	Count	2017	0	1	0
Cambodia	Aoral	Proportion	2000	12.5	18.6	7.5
Cambodia	Aoral	Proportion	2017	39.7	56.0	27.7
Cambodia	Aoral	Count	2000	0	0	0
Cambodia	Aoral	Count	2017	0	1	0
Cambodia	Baray	Proportion	2000	12.9	18.9	8.5
Cambodia	Baray	Proportion	2017	36.0	51.1	22.2
Cambodia	Baray	Count	2000	0	0	0
Cambodia	Baray	Count	2017	0	1	0

Table 4: ORS by Unit *(continued)*

Country	Unit	Metric	Year	Mean	Upper	Lower
Cambodia	Angkor Chey	Proportion	2000	16.7	23.6	11.1
Cambodia	Angkor Chey	Proportion	2017	39.0	55.6	24.9
Cambodia	Angkor Chey	Count	2000	0	0	0
Cambodia	Angkor Chey	Count	2017	0	1	0
Cambodia	Angk Snuol	Proportion	2000	26.1	33.9	19.5
Cambodia	Angk Snuol	Proportion	2017	39.4	55.1	25.4
Cambodia	Angk Snuol	Count	2000	0	0	0
Cambodia	Angk Snuol	Count	2017	0	1	0
Cambodia	Botum Sakor	Proportion	2000	13.7	20.1	8.4
Cambodia	Botum Sakor	Proportion	2017	41.1	56.4	25.8
Cambodia	Botum Sakor	Count	2000	0	0	0
Cambodia	Botum Sakor	Count	2017	0	1	0
Cambodia	Kaeb	Proportion	2000	14.0	21.8	8.6
Cambodia	Kaeb	Proportion	2017	37.4	56.6	24.1
Cambodia	Kaeb	Count	2000	0	0	0
Cambodia	Kaeb	Count	2017	0	1	0
Cambodia	Chhloung	Proportion	2000	13.0	20.0	7.6
Cambodia	Chhloung	Proportion	2017	32.2	47.0	19.9
Cambodia	Chhloung	Count	2000	0	0	0
Cambodia	Chhloung	Count	2017	0	0	0
Cambodia	Pailin	Proportion	2000	17.4	26.1	9.8
Cambodia	Pailin	Proportion	2017	32.0	45.5	19.8
Cambodia	Pailin	Count	2000	0	0	0
Cambodia	Pailin	Count	2017	0	0	0
Cambodia	Mittakpheap	Proportion	2000	19.3	28.5	11.3
Cambodia	Mittakpheap	Proportion	2017	43.4	61.3	27.7
Cambodia	Mittakpheap	Count	2000	0	0	0
Cambodia	Mittakpheap	Count	2017	0	1	0
Cambodia	Kaev Seima	Proportion	2000	14.4	21.9	9.4
Cambodia	Kaev Seima	Proportion	2017	34.7	51.3	22.4
Cambodia	Kaev Seima	Count	2000	0	0	0
Cambodia	Kaev Seima	Count	2017	0	1	0
Cambodia	Anlong Veaeng	Proportion	2000	16.0	23.8	9.2
Cambodia	Anlong Veaeng	Proportion	2017	36.0	53.2	23.0
Cambodia	Anlong Veaeng	Count	2000	0	0	0
Cambodia	Anlong Veaeng	Count	2017	0	1	0
Cambodia	Dangkao	Proportion	2000	33.3	42.1	24.8
Cambodia	Dangkao	Proportion	2017	42.5	58.9	27.4
Cambodia	Dangkao	Count	2000	0	0	0
Cambodia	Dangkao	Count	2017	0	1	0
Cambodia	Bakan	Proportion	2000	13.8	19.0	9.4
Cambodia	Bakan	Proportion	2017	40.1	55.2	25.1
Cambodia	Bakan	Count	2000	0	0	0
Cambodia	Bakan	Count	2017	0	1	0
Cambodia	Chey Saen	Proportion	2000	20.8	29.6	14.3
Cambodia	Chey Saen	Proportion	2017	30.6	48.1	18.6
Cambodia	Chey Saen	Count	2000	0	0	0
Cambodia	Chey Saen	Count	2017	0	0	0
Cambodia	Ba Phnum	Proportion	2000	20.7	28.7	13.7

Table 4: ORS by Unit *(continued)*

Country	Unit	Metric	Year	Mean	Upper	Lower
Cambodia	Ba Phnum	Proportion	2017	37.8	53.1	23.7
Cambodia	Ba Phnum	Count	2000	0	0	0
Cambodia	Ba Phnum	Count	2017	0	1	0
Cambodia	Andoung Meas	Proportion	2000	14.8	22.6	8.5
Cambodia	Andoung Meas	Proportion	2017	41.4	58.8	26.5
Cambodia	Andoung Meas	Count	2000	0	0	0
Cambodia	Andoung Meas	Count	2017	0	1	0
Cambodia	Angkor Chum	Proportion	2000	12.0	18.3	6.5
Cambodia	Angkor Chum	Proportion	2017	38.4	54.4	22.8
Cambodia	Angkor Chum	Count	2000	0	0	0
Cambodia	Angkor Chum	Count	2017	0	1	0
Cambodia	Sesan	Proportion	2000	18.1	26.1	11.2
Cambodia	Sesan	Proportion	2017	34.1	50.0	21.2
Cambodia	Sesan	Count	2000	0	0	0
Cambodia	Sesan	Count	2017	0	0	0
Cambodia	Chantrea	Proportion	2000	15.4	22.4	9.9
Cambodia	Chantrea	Proportion	2017	39.6	57.5	23.5
Cambodia	Chantrea	Count	2000	0	0	0
Cambodia	Chantrea	Count	2017	0	1	0
Cambodia	Angkor Borei	Proportion	2000	20.5	28.3	13.9
Cambodia	Angkor Borei	Proportion	2017	40.0	56.3	25.9
Cambodia	Angkor Borei	Count	2000	0	0	0
Cambodia	Angkor Borei	Count	2017	0	1	0
Cambodia	Dambae	Proportion	2000	13.0	18.5	7.9
Cambodia	Dambae	Proportion	2017	32.7	47.8	19.0
Cambodia	Dambae	Count	2000	0	0	0
Cambodia	Dambae	Count	2017	0	0	0
Cambodia	Mongkol Borei	Proportion	2000	17.2	24.5	10.8
Cambodia	Mongkol Borei	Proportion	2017	34.8	49.3	21.8
Cambodia	Mongkol Borei	Count	2000	0	0	0
Cambodia	Mongkol Borei	Count	2017	0	0	0
Cambodia	Banan	Proportion	2000	20.0	28.3	12.2
Cambodia	Banan	Proportion	2017	33.5	47.7	21.0
Cambodia	Banan	Count	2000	0	0	0
Cambodia	Banan	Count	2017	0	0	0
Cambodia	Chamkar Leu	Proportion	2000	13.4	19.1	8.7
Cambodia	Chamkar Leu	Proportion	2017	35.7	53.0	22.1
Cambodia	Chamkar Leu	Count	2000	0	0	0
Cambodia	Chamkar Leu	Count	2017	0	1	0
Cambodia	Chol Kiri	Proportion	2000	15.0	20.4	10.4
Cambodia	Chol Kiri	Proportion	2017	42.3	60.0	27.2
Cambodia	Chol Kiri	Count	2000	0	0	0
Cambodia	Chol Kiri	Count	2017	0	1	0
Cambodia	Basedth	Proportion	2000	18.3	25.4	11.8
Cambodia	Basedth	Proportion	2017	38.1	53.3	25.0
Cambodia	Basedth	Count	2000	0	0	0
Cambodia	Basedth	Count	2017	0	1	0
Cambodia	Kampong Svay	Proportion	2000	14.1	21.2	9.2
Cambodia	Kampong Svay	Proportion	2017	38.4	54.6	24.6

Table 4: ORS by Unit *(continued)*

Country	Unit	Metric	Year	Mean	Upper	Lower
Cambodia	Kampong Svay	Count	2000	0	0	0
Cambodia	Kampong Svay	Count	2017	0	1	0
Cambodia	Banteay Meas	Proportion	2000	15.0	21.4	9.9
Cambodia	Banteay Meas	Proportion	2017	38.5	57.0	25.0
Cambodia	Banteay Meas	Count	2000	0	0	0
Cambodia	Banteay Meas	Count	2017	0	1	0
Cambodia	Kandal Stueng	Proportion	2000	27.6	35.2	20.3
Cambodia	Kandal Stueng	Proportion	2017	38.6	55.3	25.2
Cambodia	Kandal Stueng	Count	2000	0	0	0
Cambodia	Kandal Stueng	Count	2017	0	1	0
Cambodia	Kampong Seila	Proportion	2000	14.4	20.9	9.1
Cambodia	Kampong Seila	Proportion	2017	40.1	55.4	26.4
Cambodia	Kampong Seila	Count	2000	0	0	0
Cambodia	Kampong Seila	Count	2017	0	1	0
Cambodia	Kracheh	Proportion	2000	13.9	20.7	8.4
Cambodia	Kracheh	Proportion	2017	33.1	48.7	20.9
Cambodia	Kracheh	Count	2000	0	0	0
Cambodia	Kracheh	Count	2017	0	0	0
Cambodia	Sala Krau	Proportion	2000	16.6	25.3	9.6
Cambodia	Sala Krau	Proportion	2017	31.5	45.3	19.0
Cambodia	Sala Krau	Count	2000	0	0	0
Cambodia	Sala Krau	Count	2017	0	0	0
Cambodia	Prey Nob	Proportion	2000	16.8	26.4	9.9
Cambodia	Prey Nob	Proportion	2017	43.0	59.9	28.5
Cambodia	Prey Nob	Count	2000	0	0	0
Cambodia	Prey Nob	Count	2017	0	1	0
Cambodia	Kaoh Nheaek	Proportion	2000	15.9	23.8	9.7
Cambodia	Kaoh Nheaek	Proportion	2017	37.2	53.2	24.0
Cambodia	Kaoh Nheaek	Count	2000	0	0	0
Cambodia	Kaoh Nheaek	Count	2017	0	1	0
Cambodia	Banteay Ampil	Proportion	2000	13.4	20.4	8.0
Cambodia	Banteay Ampil	Proportion	2017	34.6	49.8	21.7
Cambodia	Banteay Ampil	Count	2000	0	0	0
Cambodia	Banteay Ampil	Count	2017	0	0	0
Cambodia	Mean Chey	Proportion	2000	34.5	43.4	25.6
Cambodia	Mean Chey	Proportion	2017	43.2	60.0	28.1
Cambodia	Mean Chey	Count	2000	0	0	0
Cambodia	Mean Chey	Count	2017	0	1	0
Cambodia	Kandieng	Proportion	2000	13.8	19.2	9.5
Cambodia	Kandieng	Proportion	2017	42.8	57.3	27.6
Cambodia	Kandieng	Count	2000	0	0	0
Cambodia	Kandieng	Count	2017	0	1	0
Cambodia	Chhaeb	Proportion	2000	23.1	31.4	16.2
Cambodia	Chhaeb	Proportion	2017	33.0	48.5	21.1
Cambodia	Chhaeb	Count	2000	0	0	0
Cambodia	Chhaeb	Count	2017	0	0	0
Cambodia	Kamchay Mear	Proportion	2000	16.4	23.3	10.5
Cambodia	Kamchay Mear	Proportion	2017	35.4	50.5	21.3
Cambodia	Kamchay Mear	Count	2000	0	0	0

Table 4: ORS by Unit *(continued)*

Country	Unit	Metric	Year	Mean	Upper	Lower
Cambodia	Kamchay Mear	Count	2017	0	1	0
Cambodia	Ban Lung	Proportion	2000	22.6	32.3	14.0
Cambodia	Ban Lung	Proportion	2017	42.4	59.4	27.8
Cambodia	Ban Lung	Count	2000	0	0	0
Cambodia	Ban Lung	Count	2017	0	1	0
Cambodia	Angkor Thum	Proportion	2000	12.3	17.9	7.3
Cambodia	Angkor Thum	Proportion	2017	38.2	55.8	23.8
Cambodia	Angkor Thum	Count	2000	0	0	0
Cambodia	Angkor Thum	Count	2017	0	1	0
Cambodia	Siem Bouk	Proportion	2000	18.2	27.5	10.7
Cambodia	Siem Bouk	Proportion	2017	32.5	48.4	19.5
Cambodia	Siem Bouk	Count	2000	0	0	0
Cambodia	Siem Bouk	Count	2017	0	0	0
Cambodia	Kampong Rou	Proportion	2000	15.9	22.9	10.8
Cambodia	Kampong Rou	Proportion	2017	39.3	57.9	24.6
Cambodia	Kampong Rou	Count	2000	0	0	0
Cambodia	Kampong Rou	Count	2017	0	1	0
Cambodia	Bati	Proportion	2000	23.4	30.7	16.1
Cambodia	Bati	Proportion	2017	37.6	54.2	25.1
Cambodia	Bati	Count	2000	0	0	0
Cambodia	Bati	Count	2017	0	1	0
Cambodia	Kampong Siem	Proportion	2000	14.7	21.4	9.2
Cambodia	Kampong Siem	Proportion	2017	34.0	49.9	19.3
Cambodia	Kampong Siem	Count	2000	0	0	0
Cambodia	Kampong Siem	Count	2017	0	0	0
Cambodia	Ou Chrov	Proportion	2000	18.6	25.8	12.8
Cambodia	Ou Chrov	Proportion	2017	36.5	50.1	22.7
Cambodia	Ou Chrov	Count	2000	0	0	0
Cambodia	Ou Chrov	Count	2017	0	1	0
Cambodia	Bat Dambang	Proportion	2000	20.1	27.0	12.7
Cambodia	Bat Dambang	Proportion	2017	34.7	47.8	22.1
Cambodia	Bat Dambang	Count	2000	0	0	0
Cambodia	Bat Dambang	Count	2017	0	0	0
Cambodia	Cheung Prey	Proportion	2000	14.8	21.2	10.2
Cambodia	Cheung Prey	Proportion	2017	36.7	53.9	22.8
Cambodia	Cheung Prey	Count	2000	0	0	0
Cambodia	Cheung Prey	Count	2017	0	1	0
Cambodia	Kampong Chhnang	Proportion	2000	17.0	23.6	11.5
Cambodia	Kampong Chhnang	Proportion	2017	44.8	64.0	29.7
Cambodia	Kampong Chhnang	Count	2000	0	0	0
Cambodia	Kampong Chhnang	Count	2017	0	1	0
Cambodia	Chbar Mon	Proportion	2000	19.9	27.7	12.7
Cambodia	Chbar Mon	Proportion	2017	36.8	54.6	25.1
Cambodia	Chbar Mon	Count	2000	0	0	0
Cambodia	Chbar Mon	Count	2017	0	1	0
Cambodia	Prasat Balangk	Proportion	2000	14.5	21.4	9.5
Cambodia	Prasat Balangk	Proportion	2017	34.5	49.9	22.0
Cambodia	Prasat Balangk	Count	2000	0	0	0
Cambodia	Prasat Balangk	Count	2017	0	0	0

Table 4: ORS by Unit *(continued)*

Country	Unit	Metric	Year	Mean	Upper	Lower
Cambodia	Chhuk	Proportion	2000	15.5	23.0	10.3
Cambodia	Chhuk	Proportion	2017	37.4	54.3	24.7
Cambodia	Chhuk	Count	2000	0	0	0
Cambodia	Chhuk	Count	2017	0	1	0
Cambodia	Kaoh Thum	Proportion	2000	25.5	34.0	17.8
Cambodia	Kaoh Thum	Proportion	2017	42.5	58.7	28.2
Cambodia	Kaoh Thum	Count	2000	0	0	0
Cambodia	Kaoh Thum	Count	2017	0	1	0
Cambodia	Kaoh Kong	Proportion	2000	15.6	23.2	9.6
Cambodia	Kaoh Kong	Proportion	2017	44.2	60.9	27.9
Cambodia	Kaoh Kong	Count	2000	0	0	0
Cambodia	Kaoh Kong	Count	2017	0	1	0
Cambodia	Preaek Prasab	Proportion	2000	13.2	19.4	8.1
Cambodia	Preaek Prasab	Proportion	2017	33.3	48.5	21.5
Cambodia	Preaek Prasab	Count	2000	0	0	0
Cambodia	Preaek Prasab	Count	2017	0	0	0
Cambodia	Stueng hav	Proportion	2000	16.2	24.2	9.3
Cambodia	Stueng hav	Proportion	2017	42.8	59.4	29.3
Cambodia	Stueng hav	Count	2000	0	0	0
Cambodia	Stueng hav	Count	2017	0	1	0
Cambodia	Ou Reang	Proportion	2000	20.6	29.1	13.2
Cambodia	Ou Reang	Proportion	2017	42.2	58.9	28.9
Cambodia	Ou Reang	Count	2000	0	0	0
Cambodia	Ou Reang	Count	2017	0	1	0
Cambodia	Chong Kal	Proportion	2000	12.1	18.3	6.5
Cambodia	Chong Kal	Proportion	2017	36.7	53.4	22.7
Cambodia	Chong Kal	Count	2000	0	0	0
Cambodia	Chong Kal	Count	2017	0	1	0
Cambodia	Phnom Penh	Proportion	2000	34.4	43.6	25.6
Cambodia	Phnom Penh	Proportion	2017	44.7	61.6	29.3
Cambodia	Phnom Penh	Count	2000	0	0	0
Cambodia	Phnom Penh	Count	2017	0	1	0
Cambodia	Krakor	Proportion	2000	12.8	18.4	8.8
Cambodia	Krakor	Proportion	2017	43.7	59.7	31.2
Cambodia	Krakor	Count	2000	0	0	0
Cambodia	Krakor	Count	2017	0	1	0
Cambodia	Choam Khsant	Proportion	2000	19.4	29.3	13.1
Cambodia	Choam Khsant	Proportion	2017	33.9	50.0	21.9
Cambodia	Choam Khsant	Count	2000	0	0	0
Cambodia	Choam Khsant	Count	2017	0	0	0
Cambodia	Kampong Leav	Proportion	2000	24.0	33.2	15.4
Cambodia	Kampong Leav	Proportion	2017	36.8	54.1	22.5
Cambodia	Kampong Leav	Count	2000	0	0	0
Cambodia	Kampong Leav	Count	2017	0	1	0
Cambodia	Bar Kaev	Proportion	2000	17.3	26.4	10.3
Cambodia	Bar Kaev	Proportion	2017	41.2	57.7	25.9
Cambodia	Bar Kaev	Count	2000	0	0	0
Cambodia	Bar Kaev	Count	2017	0	1	0
Cambodia	Banteay Srei	Proportion	2000	13.2	20.0	8.2

Table 4: ORS by Unit *(continued)*

Country	Unit	Metric	Year	Mean	Upper	Lower
Cambodia	Banteay Srei	Proportion	2017	38.3	56.1	25.1
Cambodia	Banteay Srei	Count	2000	0	0	0
Cambodia	Banteay Srei	Count	2017	0	1	0
Cambodia	Siem Pang	Proportion	2000	17.0	24.5	10.9
Cambodia	Siem Pang	Proportion	2017	36.9	52.0	24.3
Cambodia	Siem Pang	Count	2000	0	0	0
Cambodia	Siem Pang	Count	2017	0	1	0
Cambodia	Romeas Haek	Proportion	2000	17.3	25.0	11.7
Cambodia	Romeas Haek	Proportion	2017	37.9	54.6	23.5
Cambodia	Romeas Haek	Count	2000	0	0	0
Cambodia	Romeas Haek	Count	2017	0	1	0
Cambodia	Bourei Cholsar	Proportion	2000	20.2	28.1	13.4
Cambodia	Bourei Cholsar	Proportion	2017	45.2	59.8	29.4
Cambodia	Bourei Cholsar	Count	2000	0	0	0
Cambodia	Bourei Cholsar	Count	2017	0	1	0
Cambodia	Krouch Chhmar	Proportion	2000	14.0	20.4	8.7
Cambodia	Krouch Chhmar	Proportion	2017	34.8	51.3	19.4
Cambodia	Krouch Chhmar	Count	2000	0	0	0
Cambodia	Krouch Chhmar	Count	2017	0	1	0
Cambodia	Phnum Srok	Proportion	2000	13.8	20.2	8.2
Cambodia	Phnum Srok	Proportion	2017	39.1	54.5	25.3
Cambodia	Phnum Srok	Count	2000	0	0	0
Cambodia	Phnum Srok	Count	2017	0	1	0
Cambodia	Bavel	Proportion	2000	17.5	24.8	11.6
Cambodia	Bavel	Proportion	2017	33.0	47.2	20.8
Cambodia	Bavel	Count	2000	0	0	0
Cambodia	Bavel	Count	2017	0	0	0
Cambodia	Kampong Cham	Proportion	2000	17.7	25.0	11.9
Cambodia	Kampong Cham	Proportion	2017	35.8	51.3	21.7
Cambodia	Kampong Cham	Count	2000	0	0	0
Cambodia	Kampong Cham	Count	2017	0	1	0
Cambodia	Kampong Leaeng	Proportion	2000	14.0	19.9	9.3
Cambodia	Kampong Leaeng	Proportion	2017	43.3	61.8	28.9
Cambodia	Kampong Leaeng	Count	2000	0	0	0
Cambodia	Kampong Leaeng	Count	2017	0	1	0
Cambodia	Kong Pisei	Proportion	2000	20.9	27.7	14.0
Cambodia	Kong Pisei	Proportion	2017	37.1	52.9	25.8
Cambodia	Kong Pisei	Count	2000	0	0	0
Cambodia	Kong Pisei	Count	2017	0	1	0
Cambodia	Prasat Sambour	Proportion	2000	14.6	21.9	8.8
Cambodia	Prasat Sambour	Proportion	2017	34.0	51.1	21.0
Cambodia	Prasat Sambour	Count	2000	0	0	0
Cambodia	Prasat Sambour	Count	2017	0	1	0
Cambodia	Chum Kiri	Proportion	2000	15.8	22.3	10.1
Cambodia	Chum Kiri	Proportion	2017	37.9	53.8	25.3
Cambodia	Chum Kiri	Count	2000	0	0	0
Cambodia	Chum Kiri	Count	2017	0	1	0
Cambodia	Khsach Kandal	Proportion	2000	27.3	34.5	19.8
Cambodia	Khsach Kandal	Proportion	2017	39.3	55.2	24.7

Table 4: ORS by Unit *(continued)*

Country	Unit	Metric	Year	Mean	Upper	Lower
Cambodia	Khsach Kandal	Count	2000	0	0	0
Cambodia	Khsach Kandal	Count	2017	0	1	0
Cambodia	Kiri Sakor	Proportion	2000	15.1	22.4	9.4
Cambodia	Kiri Sakor	Proportion	2017	42.0	57.7	26.6
Cambodia	Kiri Sakor	Count	2000	0	0	0
Cambodia	Kiri Sakor	Count	2017	0	1	0
Cambodia	Sambour	Proportion	2000	14.2	20.5	8.7
Cambodia	Sambour	Proportion	2017	32.9	48.1	21.5
Cambodia	Sambour	Count	2000	0	0	0
Cambodia	Sambour	Count	2017	0	0	0
Cambodia	Pechr Chenda	Proportion	2000	18.2	26.2	11.4
Cambodia	Pechr Chenda	Proportion	2017	38.1	54.0	25.8
Cambodia	Pechr Chenda	Count	2000	0	0	0
Cambodia	Pechr Chenda	Count	2017	0	1	0
Cambodia	Samraong	Proportion	2000	14.9	22.2	8.2
Cambodia	Samraong	Proportion	2017	37.9	53.2	24.4
Cambodia	Samraong	Count	2000	0	0	0
Cambodia	Samraong	Count	2017	0	1	0
Cambodia	Ruessei Kaev	Proportion	2000	33.5	42.0	24.7
Cambodia	Ruessei Kaev	Proportion	2017	43.4	59.6	28.1
Cambodia	Ruessei Kaev	Count	2000	0	0	0
Cambodia	Ruessei Kaev	Count	2017	0	1	0
Cambodia	Phnum Kravanh	Proportion	2000	13.1	18.6	8.3
Cambodia	Phnum Kravanh	Proportion	2017	41.2	55.5	27.8
Cambodia	Phnum Kravanh	Count	2000	0	0	0
Cambodia	Phnum Kravanh	Count	2017	0	1	0
Cambodia	Kuleaen	Proportion	2000	18.9	28.9	11.3
Cambodia	Kuleaen	Proportion	2017	33.9	51.7	20.9
Cambodia	Kuleaen	Count	2000	0	0	0
Cambodia	Kuleaen	Count	2017	0	1	0
Cambodia	Kampong Trabaek	Proportion	2000	18.5	26.9	12.2
Cambodia	Kampong Trabaek	Proportion	2017	38.8	55.8	24.3
Cambodia	Kampong Trabaek	Count	2000	0	0	0
Cambodia	Kampong Trabaek	Count	2017	0	1	0
Cambodia	Koun Mom	Proportion	2000	18.3	26.4	11.5
Cambodia	Koun Mom	Proportion	2017	37.9	54.4	23.7
Cambodia	Koun Mom	Count	2000	0	0	0
Cambodia	Koun Mom	Count	2017	0	1	0
Cambodia	Chi Kraeng	Proportion	2000	14.1	21.5	8.4
Cambodia	Chi Kraeng	Proportion	2017	39.0	55.9	25.0
Cambodia	Chi Kraeng	Count	2000	0	0	0
Cambodia	Chi Kraeng	Count	2017	0	1	0
Cambodia	Stueng Traeng	Proportion	2000	21.5	31.2	13.3
Cambodia	Stueng Traeng	Proportion	2017	33.2	48.5	20.1
Cambodia	Stueng Traeng	Count	2000	0	0	0
Cambodia	Stueng Traeng	Count	2017	0	0	0
Cambodia	Rumduol	Proportion	2000	17.3	25.4	11.6
Cambodia	Rumduol	Proportion	2017	38.7	57.1	23.3
Cambodia	Rumduol	Count	2000	0	0	0

Table 4: ORS by Unit *(continued)*

Country	Unit	Metric	Year	Mean	Upper	Lower
Cambodia	Rumduol	Count	2017	0	1	0
Cambodia	Doun Kaev	Proportion	2000	21.8	30.3	14.9
Cambodia	Doun Kaev	Proportion	2017	40.8	56.8	25.9
Cambodia	Doun Kaev	Count	2000	0	0	0
Cambodia	Doun Kaev	Count	2017	0	1	0
Cambodia	Memot	Proportion	2000	13.2	18.7	7.9
Cambodia	Memot	Proportion	2017	32.3	47.4	19.0
Cambodia	Memot	Count	2000	0	0	0
Cambodia	Memot	Count	2017	0	0	0
Cambodia	Preah Netr Preah	Proportion	2000	15.2	22.0	9.4
Cambodia	Preah Netr Preah	Proportion	2017	38.3	52.0	24.6
Cambodia	Preah Netr Preah	Count	2000	0	0	0
Cambodia	Preah Netr Preah	Count	2017	0	1	0
Cambodia	Moung Ruessei	Proportion	2000	15.8	22.8	10.9
Cambodia	Moung Ruessei	Proportion	2017	35.5	51.0	21.8
Cambodia	Moung Ruessei	Count	2000	0	0	0
Cambodia	Moung Ruessei	Count	2017	0	1	0
Cambodia	Kampong Siem	Proportion	2000	15.5	22.5	10.5
Cambodia	Kampong Siem	Proportion	2017	34.7	50.3	20.4
Cambodia	Kampong Siem	Count	2000	0	0	0
Cambodia	Kampong Siem	Count	2017	0	1	0
Cambodia	Kampong Tralach	Proportion	2000	17.5	23.9	12.4
Cambodia	Kampong Tralach	Proportion	2017	41.9	58.6	26.4
Cambodia	Kampong Tralach	Count	2000	0	0	0
Cambodia	Kampong Tralach	Count	2017	0	1	0
Cambodia	Odongk	Proportion	2000	18.3	25.8	13.1
Cambodia	Odongk	Proportion	2017	38.1	54.4	24.8
Cambodia	Odongk	Count	2000	0	0	0
Cambodia	Odongk	Count	2017	0	1	0
Cambodia	Sandan	Proportion	2000	16.0	25.0	9.6
Cambodia	Sandan	Proportion	2017	31.6	48.6	19.9
Cambodia	Sandan	Count	2000	0	0	0
Cambodia	Sandan	Count	2017	0	0	0
Cambodia	Dang Tong	Proportion	2000	14.4	21.4	9.5
Cambodia	Dang Tong	Proportion	2017	37.2	54.9	23.9
Cambodia	Dang Tong	Count	2000	0	0	0
Cambodia	Dang Tong	Count	2017	0	1	0
Cambodia	Kien Svay	Proportion	2000	29.9	37.9	21.0
Cambodia	Kien Svay	Proportion	2017	38.6	54.8	25.2
Cambodia	Kien Svay	Count	2000	0	0	0
Cambodia	Kien Svay	Count	2017	0	1	0
Cambodia	Mondol Seima	Proportion	2000	25.1	33.8	16.5
Cambodia	Mondol Seima	Proportion	2017	49.6	65.0	33.6
Cambodia	Mondol Seima	Count	2000	0	0	0
Cambodia	Mondol Seima	Count	2017	0	1	0
Cambodia	Snuol	Proportion	2000	12.2	18.3	7.5
Cambodia	Snuol	Proportion	2017	30.8	46.7	19.1
Cambodia	Snuol	Count	2000	0	0	0
Cambodia	Snuol	Count	2017	0	0	0

Table 4: ORS by Unit *(continued)*

Country	Unit	Metric	Year	Mean	Upper	Lower
Cambodia	Saen Monourom	Proportion	2000	21.3	31.0	12.9
Cambodia	Saen Monourom	Proportion	2017	41.6	59.4	27.7
Cambodia	Saen Monourom	Count	2000	0	0	0
Cambodia	Saen Monourom	Count	2017	0	1	0
Cambodia	Sampov Meas	Proportion	2000	14.2	19.8	9.7
Cambodia	Sampov Meas	Proportion	2017	42.3	56.9	26.8
Cambodia	Sampov Meas	Count	2000	0	0	0
Cambodia	Sampov Meas	Count	2017	0	1	0
Cambodia	Rovieng	Proportion	2000	19.5	27.9	12.8
Cambodia	Rovieng	Proportion	2017	30.3	46.2	18.2
Cambodia	Rovieng	Count	2000	0	0	0
Cambodia	Rovieng	Count	2017	0	0	0
Cambodia	Kanhchriech	Proportion	2000	16.2	23.5	9.8
Cambodia	Kanhchriech	Proportion	2017	34.3	49.5	19.8
Cambodia	Kanhchriech	Count	2000	0	0	0
Cambodia	Kanhchriech	Count	2017	0	0	0
Cambodia	Lumphat	Proportion	2000	17.3	25.5	10.8
Cambodia	Lumphat	Proportion	2017	39.4	55.9	24.9
Cambodia	Lumphat	Count	2000	0	0	0
Cambodia	Lumphat	Count	2017	0	1	0
Cambodia	Kralanh	Proportion	2000	13.8	20.7	8.4
Cambodia	Kralanh	Proportion	2017	40.2	54.8	26.1
Cambodia	Kralanh	Count	2000	0	0	0
Cambodia	Kralanh	Count	2017	0	1	0
Cambodia	Thala Barivat	Proportion	2000	19.9	27.8	13.6
Cambodia	Thala Barivat	Proportion	2017	33.0	48.3	20.3
Cambodia	Thala Barivat	Count	2000	0	0	0
Cambodia	Thala Barivat	Count	2017	0	0	0
Cambodia	Svay Chrum	Proportion	2000	18.1	25.5	12.5
Cambodia	Svay Chrum	Proportion	2017	38.9	56.5	24.2
Cambodia	Svay Chrum	Count	2000	0	0	0
Cambodia	Svay Chrum	Count	2017	0	1	0
Cambodia	Kaoh Andaet	Proportion	2000	17.9	26.5	11.5
Cambodia	Kaoh Andaet	Proportion	2017	42.2	59.0	26.2
Cambodia	Kaoh Andaet	Count	2000	0	0	0
Cambodia	Kaoh Andaet	Count	2017	0	1	0
Cambodia	Ou Reang Ov	Proportion	2000	16.1	23.8	10.0
Cambodia	Ou Reang Ov	Proportion	2017	33.7	49.1	19.4
Cambodia	Ou Reang Ov	Count	2000	0	0	0
Cambodia	Ou Reang Ov	Count	2017	0	0	0
Cambodia	Serei Saophoan	Proportion	2000	17.2	24.5	10.6
Cambodia	Serei Saophoan	Proportion	2017	35.0	49.0	22.1
Cambodia	Serei Saophoan	Count	2000	0	0	0
Cambodia	Serei Saophoan	Count	2017	0	0	0
Cambodia	Phnum Proek	Proportion	2000	16.9	25.8	10.5
Cambodia	Phnum Proek	Proportion	2017	33.6	46.5	21.0
Cambodia	Phnum Proek	Count	2000	0	0	0
Cambodia	Phnum Proek	Count	2017	0	0	0
Cambodia	Kang Meas	Proportion	2000	17.9	24.5	12.6

Table 4: ORS by Unit *(continued)*

Country	Unit	Metric	Year	Mean	Upper	Lower
Cambodia	Kang Meas	Proportion	2017	36.2	51.3	23.0
Cambodia	Kang Meas	Count	2000	0	0	0
Cambodia	Kang Meas	Count	2017	0	1	0
Cambodia	Rolea B'ier	Proportion	2000	15.0	20.7	10.5
Cambodia	Rolea B'ier	Proportion	2017	43.8	61.9	29.0
Cambodia	Rolea B'ier	Count	2000	0	0	0
Cambodia	Rolea B'ier	Count	2017	0	1	0
Cambodia	Phnum Sruoch	Proportion	2000	15.8	22.2	9.7
Cambodia	Phnum Sruoch	Proportion	2017	37.3	53.0	25.3
Cambodia	Phnum Sruoch	Count	2000	0	0	0
Cambodia	Phnum Sruoch	Count	2017	0	1	0
Cambodia	Santuk	Proportion	2000	12.8	19.0	8.3
Cambodia	Santuk	Proportion	2017	34.8	49.7	21.8
Cambodia	Santuk	Count	2000	0	0	0
Cambodia	Santuk	Count	2017	0	0	0
Cambodia	Kampong Bay	Proportion	2000	16.8	26.1	10.0
Cambodia	Kampong Bay	Proportion	2017	38.8	57.1	24.4
Cambodia	Kampong Bay	Count	2000	0	0	0
Cambodia	Kampong Bay	Count	2017	0	1	0
Cambodia	Leuk Daek	Proportion	2000	23.8	32.4	16.2
Cambodia	Leuk Daek	Proportion	2017	40.6	56.9	26.0
Cambodia	Leuk Daek	Count	2000	0	0	0
Cambodia	Leuk Daek	Count	2017	0	1	0
Cambodia	Smach Mean Chey	Proportion	2000	20.9	31.6	12.1
Cambodia	Smach Mean Chey	Proportion	2017	46.0	63.6	28.8
Cambodia	Smach Mean Chey	Count	2000	0	0	0
Cambodia	Smach Mean Chey	Count	2017	0	1	0
Cambodia	Veal Veaeng	Proportion	2000	15.3	23.2	9.6
Cambodia	Veal Veaeng	Proportion	2017	38.0	52.7	24.9
Cambodia	Veal Veaeng	Count	2000	0	0	0
Cambodia	Veal Veaeng	Count	2017	0	1	0
Cambodia	Sangkom Thmei	Proportion	2000	19.2	28.5	12.5
Cambodia	Sangkom Thmei	Proportion	2017	31.6	47.8	18.5
Cambodia	Sangkom Thmei	Count	2000	0	0	0
Cambodia	Sangkom Thmei	Count	2017	0	0	0
Cambodia	Me Sang	Proportion	2000	18.6	26.2	12.3
Cambodia	Me Sang	Proportion	2017	37.6	55.3	23.8
Cambodia	Me Sang	Count	2000	0	0	0
Cambodia	Me Sang	Count	2017	0	1	0
Cambodia	Ou Chum	Proportion	2000	18.4	27.2	11.5
Cambodia	Ou Chum	Proportion	2017	41.4	57.7	26.9
Cambodia	Ou Chum	Count	2000	0	0	0
Cambodia	Ou Chum	Count	2017	0	1	0
Cambodia	Prasat Bakong	Proportion	2000	15.2	22.8	9.4
Cambodia	Prasat Bakong	Proportion	2017	40.6	57.0	27.7
Cambodia	Prasat Bakong	Count	2000	0	0	0
Cambodia	Prasat Bakong	Count	2017	0	1	0
Cambodia	Svay Rieng	Proportion	2000	18.9	28.0	12.5
Cambodia	Svay Rieng	Proportion	2017	38.9	57.4	23.7

Table 4: ORS by Unit *(continued)*

Country	Unit	Metric	Year	Mean	Upper	Lower
Cambodia	Svay Rieng	Count	2000	0	0	0
Cambodia	Svay Rieng	Count	2017	0	1	0
Cambodia	Kiri Vong	Proportion	2000	16.9	24.7	10.8
Cambodia	Kiri Vong	Proportion	2017	41.7	59.9	26.3
Cambodia	Kiri Vong	Count	2000	0	0	0
Cambodia	Kiri Vong	Count	2017	0	1	0
Cambodia	Ponhea Kraek	Proportion	2000	14.2	20.1	8.5
Cambodia	Ponhea Kraek	Proportion	2017	33.3	50.5	18.4
Cambodia	Ponhea Kraek	Count	2000	0	0	0
Cambodia	Ponhea Kraek	Count	2017	0	1	0
Cambodia	Svay Chek	Proportion	2000	14.7	20.9	9.7
Cambodia	Svay Chek	Proportion	2017	35.7	49.8	22.3
Cambodia	Svay Chek	Count	2000	0	0	0
Cambodia	Svay Chek	Count	2017	0	0	0
Cambodia	Rotanak Mondol	Proportion	2000	16.7	25.5	10.0
Cambodia	Rotanak Mondol	Proportion	2017	32.3	45.3	19.9
Cambodia	Rotanak Mondol	Count	2000	0	0	0
Cambodia	Rotanak Mondol	Count	2017	0	0	0
Cambodia	Kaoh Soutin	Proportion	2000	17.3	24.0	11.6
Cambodia	Kaoh Soutin	Proportion	2017	35.1	50.6	20.6
Cambodia	Kaoh Soutin	Count	2000	0	0	0
Cambodia	Kaoh Soutin	Count	2017	0	1	0
Cambodia	Sameakki Mean Chey	Proportion	2000	15.9	22.8	11.2
Cambodia	Sameakki Mean Chey	Proportion	2017	40.3	57.4	26.1
Cambodia	Sameakki Mean Chey	Count	2000	0	0	0
Cambodia	Sameakki Mean Chey	Count	2017	0	1	0
Cambodia	Samraong Tong	Proportion	2000	19.6	26.9	13.0
Cambodia	Samraong Tong	Proportion	2017	37.2	54.3	25.4
Cambodia	Samraong Tong	Count	2000	0	0	0
Cambodia	Samraong Tong	Count	2017	0	1	0
Cambodia	Stoung	Proportion	2000	13.9	22.1	8.8
Cambodia	Stoung	Proportion	2017	37.7	52.2	24.1
Cambodia	Stoung	Count	2000	0	0	0
Cambodia	Stoung	Count	2017	0	1	0
Cambodia	Kampong Trach	Proportion	2000	14.7	21.7	9.2
Cambodia	Kampong Trach	Proportion	2017	38.2	57.0	24.5
Cambodia	Kampong Trach	Count	2000	0	0	0
Cambodia	Kampong Trach	Count	2017	0	1	0
Cambodia	Lvea Aem	Proportion	2000	30.2	37.8	21.8
Cambodia	Lvea Aem	Proportion	2017	40.2	56.3	26.2
Cambodia	Lvea Aem	Count	2000	0	0	0
Cambodia	Lvea Aem	Count	2017	0	1	0
Cambodia	Srae Ambel	Proportion	2000	13.7	20.9	8.5
Cambodia	Srae Ambel	Proportion	2017	39.5	55.8	24.9
Cambodia	Srae Ambel	Count	2000	0	0	0
Cambodia	Srae Ambel	Count	2017	0	1	0
Cambodia	Tbaeng Mean chey	Proportion	2000	20.5	30.7	13.1
Cambodia	Tbaeng Mean chey	Proportion	2017	29.6	45.0	17.4
Cambodia	Tbaeng Mean chey	Count	2000	0	0	0

Table 4: ORS by Unit *(continued)*

Country	Unit	Metric	Year	Mean	Upper	Lower
Cambodia	Tbaeng Mean chey	Count	2017	0	0	0
Cambodia	Pea Reang	Proportion	2000	21.4	28.7	14.6
Cambodia	Pea Reang	Proportion	2017	36.2	51.5	22.3
Cambodia	Pea Reang	Count	2000	0	0	0
Cambodia	Pea Reang	Count	2017	0	1	0
Cambodia	Ou Ya Dav	Proportion	2000	15.2	23.7	9.0
Cambodia	Ou Ya Dav	Proportion	2017	41.0	58.8	26.3
Cambodia	Ou Ya Dav	Count	2000	0	0	0
Cambodia	Ou Ya Dav	Count	2017	0	1	0
Cambodia	Puok	Proportion	2000	14.1	20.3	8.7
Cambodia	Puok	Proportion	2017	39.7	54.9	25.8
Cambodia	Puok	Count	2000	0	0	0
Cambodia	Puok	Count	2017	0	1	0
Cambodia	Svay Teab	Proportion	2000	16.8	24.7	11.2
Cambodia	Svay Teab	Proportion	2017	38.9	57.4	23.3
Cambodia	Svay Teab	Count	2000	0	0	0
Cambodia	Svay Teab	Count	2017	0	1	0
Cambodia	Prey Kabbas	Proportion	2000	24.1	32.1	17.5
Cambodia	Prey Kabbas	Proportion	2017	39.2	56.0	25.6
Cambodia	Prey Kabbas	Count	2000	0	0	0
Cambodia	Prey Kabbas	Count	2017	0	1	0
Cambodia	Tboung Khmum	Proportion	2000	14.7	21.5	9.3
Cambodia	Tboung Khmum	Proportion	2017	33.3	48.9	19.3
Cambodia	Tboung Khmum	Count	2000	0	0	0
Cambodia	Tboung Khmum	Count	2017	0	0	0
Cambodia	Thma Puok	Proportion	2000	15.4	22.6	9.4
Cambodia	Thma Puok	Proportion	2017	36.7	52.3	23.0
Cambodia	Thma Puok	Count	2000	0	0	0
Cambodia	Thma Puok	Count	2017	0	1	0
Cambodia	Samlout	Proportion	2000	15.9	24.1	9.3
Cambodia	Samlout	Proportion	2017	33.6	47.9	21.4
Cambodia	Samlout	Count	2000	0	0	0
Cambodia	Samlout	Count	2017	0	0	0
Cambodia	Prey Chhor	Proportion	2000	15.2	22.2	10.4
Cambodia	Prey Chhor	Proportion	2017	36.2	52.7	22.4
Cambodia	Prey Chhor	Count	2000	0	0	0
Cambodia	Prey Chhor	Count	2017	0	1	0
Cambodia	Tuek Phos	Proportion	2000	13.2	18.8	9.1
Cambodia	Tuek Phos	Proportion	2017	42.2	58.6	29.0
Cambodia	Tuek Phos	Count	2000	0	0	0
Cambodia	Tuek Phos	Count	2017	0	1	0
Cambodia	Thpong	Proportion	2000	15.2	22.6	10.3
Cambodia	Thpong	Proportion	2017	38.9	55.2	25.8
Cambodia	Thpong	Count	2000	0	0	0
Cambodia	Thpong	Count	2017	0	1	0
Cambodia	Stueng Saen	Proportion	2000	15.1	22.3	9.7
Cambodia	Stueng Saen	Proportion	2017	38.5	55.1	23.4
Cambodia	Stueng Saen	Count	2000	0	0	0
Cambodia	Stueng Saen	Count	2017	0	1	0

Table 4: ORS by Unit *(continued)*

Country	Unit	Metric	Year	Mean	Upper	Lower
Cambodia	Kampot	Proportion	2000	15.3	23.2	9.2
Cambodia	Kampot	Proportion	2017	38.8	56.7	25.4
Cambodia	Kampot	Count	2000	0	0	0
Cambodia	Kampot	Count	2017	0	1	0
Cambodia	Mukh Kampul	Proportion	2000	25.8	33.2	19.1
Cambodia	Mukh Kampul	Proportion	2017	39.8	56.3	24.9
Cambodia	Mukh Kampul	Count	2000	0	0	0
Cambodia	Mukh Kampul	Count	2017	0	1	0
Cambodia	Thma Bang	Proportion	2000	12.4	18.5	7.8
Cambodia	Thma Bang	Proportion	2017	41.9	58.6	26.0
Cambodia	Thma Bang	Count	2000	0	0	0
Cambodia	Thma Bang	Count	2017	0	1	0
Cambodia	Peam Chor	Proportion	2000	20.2	27.6	13.5
Cambodia	Peam Chor	Proportion	2017	40.4	56.3	26.2
Cambodia	Peam Chor	Count	2000	0	0	0
Cambodia	Peam Chor	Count	2017	0	1	0
Cambodia	Ta Veaeng	Proportion	2000	16.2	23.9	9.4
Cambodia	Ta Veaeng	Proportion	2017	42.2	56.6	29.8
Cambodia	Ta Veaeng	Count	2000	0	0	0
Cambodia	Ta Veaeng	Count	2017	0	1	0
Cambodia	Siem Reab	Proportion	2000	17.5	25.5	10.8
Cambodia	Siem Reab	Proportion	2017	40.7	57.3	26.2
Cambodia	Siem Reab	Count	2000	0	0	0
Cambodia	Siem Reab	Count	2017	0	1	0
Cambodia	Samraong	Proportion	2000	21.9	29.7	15.0
Cambodia	Samraong	Proportion	2017	38.5	54.7	25.4
Cambodia	Samraong	Count	2000	0	0	0
Cambodia	Samraong	Count	2017	0	1	0
Cambodia	Sangkae	Proportion	2000	21.5	29.3	13.2
Cambodia	Sangkae	Proportion	2017	35.0	49.8	21.6
Cambodia	Sangkae	Count	2000	0	0	0
Cambodia	Sangkae	Count	2017	0	0	0
Cambodia	Srei Santhor	Proportion	2000	19.6	26.2	13.9
Cambodia	Srei Santhor	Proportion	2017	35.9	51.2	22.0
Cambodia	Srei Santhor	Count	2000	0	0	0
Cambodia	Srei Santhor	Count	2017	0	1	0
Cambodia	Ponhea Lueu	Proportion	2000	24.9	32.2	18.1
Cambodia	Ponhea Lueu	Proportion	2017	40.8	57.8	26.4
Cambodia	Ponhea Lueu	Count	2000	0	0	0
Cambodia	Ponhea Lueu	Count	2017	0	1	0
Cambodia	Peam Ro	Proportion	2000	24.5	33.8	15.8
Cambodia	Peam Ro	Proportion	2017	37.9	54.1	23.8
Cambodia	Peam Ro	Count	2000	0	0	0
Cambodia	Peam Ro	Count	2017	0	1	0
Cambodia	Veun Sai	Proportion	2000	18.0	26.8	10.2
Cambodia	Veun Sai	Proportion	2017	41.2	57.2	27.0
Cambodia	Veun Sai	Count	2000	0	0	0
Cambodia	Veun Sai	Count	2017	0	1	0
Cambodia	Soutr Nikom	Proportion	2000	14.3	21.8	9.1

Table 4: ORS by Unit *(continued)*

Country	Unit	Metric	Year	Mean	Upper	Lower
Cambodia	Soutr Nikom	Proportion	2017	39.6	56.2	26.8
Cambodia	Soutr Nikom	Count	2000	0	0	0
Cambodia	Soutr Nikom	Count	2017	0	1	0
Cambodia	Tram Kak	Proportion	2000	19.0	26.6	12.8
Cambodia	Tram Kak	Proportion	2017	39.1	55.0	25.0
Cambodia	Tram Kak	Count	2000	0	0	0
Cambodia	Tram Kak	Count	2017	0	1	0
Cambodia	Svay Pao	Proportion	2000	24.5	34.3	14.6
Cambodia	Svay Pao	Proportion	2017	34.8	50.0	20.6
Cambodia	Svay Pao	Count	2000	0	0	0
Cambodia	Svay Pao	Count	2017	0	0	0
Cambodia	Stueng Trang	Proportion	2000	13.8	20.2	8.9
Cambodia	Stueng Trang	Proportion	2017	36.0	52.8	22.0
Cambodia	Stueng Trang	Count	2000	0	0	0
Cambodia	Stueng Trang	Count	2017	0	1	0
Cambodia	S'ang	Proportion	2000	28.7	37.3	20.4
Cambodia	S'ang	Proportion	2017	39.0	56.2	26.5
Cambodia	S'ang	Count	2000	0	0	0
Cambodia	S'ang	Count	2017	0	1	0
Cambodia	Preah Sdach	Proportion	2000	19.7	27.5	12.4
Cambodia	Preah Sdach	Proportion	2017	39.3	55.9	23.8
Cambodia	Preah Sdach	Count	2000	0	0	0
Cambodia	Preah Sdach	Count	2017	0	1	0
Cambodia	Srei Snam	Proportion	2000	12.6	19.3	7.2
Cambodia	Srei Snam	Proportion	2017	39.0	55.6	25.0
Cambodia	Srei Snam	Count	2000	0	0	0
Cambodia	Srei Snam	Count	2017	0	1	0
Cambodia	Treang	Proportion	2000	19.4	27.3	12.8
Cambodia	Treang	Proportion	2017	40.6	56.9	25.7
Cambodia	Treang	Count	2000	0	0	0
Cambodia	Treang	Count	2017	0	1	0
Cambodia	Ta Khmau	Proportion	2000	33.3	41.8	24.8
Cambodia	Ta Khmau	Proportion	2017	39.7	56.6	26.0
Cambodia	Ta Khmau	Count	2000	0	0	0
Cambodia	Ta Khmau	Count	2017	0	1	0
Cambodia	Prey Veaeng	Proportion	2000	19.2	27.0	12.0
Cambodia	Prey Veaeng	Proportion	2017	35.6	51.2	21.6
Cambodia	Prey Veaeng	Count	2000	0	0	0
Cambodia	Prey Veaeng	Count	2017	0	1	0
Cambodia	Svay Leu	Proportion	2000	13.6	20.1	8.4
Cambodia	Svay Leu	Proportion	2017	36.2	52.9	24.0
Cambodia	Svay Leu	Count	2000	0	0	0
Cambodia	Svay Leu	Count	2017	0	1	0
Cambodia	Sithor Kandal	Proportion	2000	17.4	24.4	11.2
Cambodia	Sithor Kandal	Proportion	2017	34.6	50.1	20.6
Cambodia	Sithor Kandal	Count	2000	0	0	0
Cambodia	Sithor Kandal	Count	2017	0	1	0
Cambodia	Varin	Proportion	2000	11.8	17.9	6.5
Cambodia	Varin	Proportion	2017	36.7	54.0	24.0

Table 4: ORS by Unit *(continued)*

Country	Unit	Metric	Year	Mean	Upper	Lower
Cambodia	Varin	Count	2000	0	0	0
Cambodia	Varin	Count	2017	0	1	0
Cameroon	Djerem	Proportion	2000	16.2	31.0	7.4
Cameroon	Djerem	Proportion	2017	18.4	37.4	7.9
Cameroon	Djerem	Count	2000	0	0	0
Cameroon	Djerem	Count	2017	0	0	0
Cameroon	Haute Sanaga	Proportion	2000	11.4	20.9	5.0
Cameroon	Haute Sanaga	Proportion	2017	17.1	33.8	5.7
Cameroon	Haute Sanaga	Count	2000	0	0	0
Cameroon	Haute Sanaga	Count	2017	0	0	0
Cameroon	Boumba et Ngoko	Proportion	2000	12.0	21.9	5.6
Cameroon	Boumba et Ngoko	Proportion	2017	14.5	27.0	6.4
Cameroon	Boumba et Ngoko	Count	2000	0	0	0
Cameroon	Boumba et Ngoko	Count	2017	0	0	0
Cameroon	Diamaré	Proportion	2000	10.9	17.9	6.3
Cameroon	Diamaré	Proportion	2017	10.9	21.6	3.7
Cameroon	Diamaré	Count	2000	0	0	0
Cameroon	Diamaré	Count	2017	0	0	0
Cameroon	Moungo	Proportion	2000	31.4	44.8	18.4
Cameroon	Moungo	Proportion	2017	33.5	58.9	14.2
Cameroon	Moungo	Count	2000	0	0	0
Cameroon	Moungo	Count	2017	0	1	0
Cameroon	Boyo	Proportion	2000	21.6	34.9	11.8
Cameroon	Boyo	Proportion	2017	21.8	43.5	8.0
Cameroon	Boyo	Count	2000	0	0	0
Cameroon	Boyo	Count	2017	0	0	0
Cameroon	Bénoué	Proportion	2000	8.5	14.2	4.5
Cameroon	Bénoué	Proportion	2017	13.5	24.8	5.6
Cameroon	Bénoué	Count	2000	0	0	0
Cameroon	Bénoué	Count	2017	0	0	0
Cameroon	Bamboutos	Proportion	2000	18.2	28.7	10.8
Cameroon	Bamboutos	Proportion	2017	19.1	38.9	6.8
Cameroon	Bamboutos	Count	2000	0	0	0
Cameroon	Bamboutos	Count	2017	0	0	0
Cameroon	Fako	Proportion	2000	30.2	45.1	16.6
Cameroon	Fako	Proportion	2017	32.8	58.2	13.4
Cameroon	Fako	Count	2000	0	0	0
Cameroon	Fako	Count	2017	0	1	0
Cameroon	Dja et Lobo	Proportion	2000	11.6	21.1	6.1
Cameroon	Dja et Lobo	Proportion	2017	16.8	32.9	7.6
Cameroon	Dja et Lobo	Count	2000	0	0	0
Cameroon	Dja et Lobo	Count	2017	0	0	0
Cameroon	Faro et Déo	Proportion	2000	13.9	26.4	5.9
Cameroon	Faro et Déo	Proportion	2017	16.8	33.2	6.2
Cameroon	Faro et Déo	Count	2000	0	0	0
Cameroon	Faro et Déo	Count	2017	0	0	0
Cameroon	Lekié	Proportion	2000	11.0	19.4	5.3
Cameroon	Lekié	Proportion	2017	18.3	37.9	7.4
Cameroon	Lekié	Count	2000	0	0	0

Table 4: ORS by Unit *(continued)*

Country	Unit	Metric	Year	Mean	Upper	Lower
Cameroon	Lekié	Count	2017	0	0	0
Cameroon	Haut Nyong	Proportion	2000	12.1	19.7	6.7
Cameroon	Haut Nyong	Proportion	2017	16.8	31.4	6.4
Cameroon	Haut Nyong	Count	2000	0	0	0
Cameroon	Haut Nyong	Count	2017	0	0	0
Cameroon	Logone et Chari	Proportion	2000	13.2	18.6	8.2
Cameroon	Logone et Chari	Proportion	2017	13.0	26.8	5.5
Cameroon	Logone et Chari	Count	2000	0	0	0
Cameroon	Logone et Chari	Count	2017	0	0	0
Cameroon	Nkam	Proportion	2000	29.9	44.3	17.8
Cameroon	Nkam	Proportion	2017	33.3	58.1	13.6
Cameroon	Nkam	Count	2000	0	0	0
Cameroon	Nkam	Count	2017	0	1	0
Cameroon	Bui	Proportion	2000	18.0	29.9	10.4
Cameroon	Bui	Proportion	2017	18.8	39.7	6.5
Cameroon	Bui	Count	2000	0	0	0
Cameroon	Bui	Count	2017	0	0	0
Cameroon	Faro	Proportion	2000	10.3	17.8	4.9
Cameroon	Faro	Proportion	2017	15.5	29.5	5.9
Cameroon	Faro	Count	2000	0	0	0
Cameroon	Faro	Count	2017	0	0	0
Cameroon	Haut Nkam	Proportion	2000	20.3	32.9	11.9
Cameroon	Haut Nkam	Proportion	2017	21.9	44.8	8.0
Cameroon	Haut Nkam	Count	2000	0	0	0
Cameroon	Haut Nkam	Count	2017	0	0	0
Cameroon	Koupé Manengouba	Proportion	2000	25.8	39.6	14.8
Cameroon	Koupé Manengouba	Proportion	2017	27.1	55.0	10.7
Cameroon	Koupé Manengouba	Count	2000	0	0	0
Cameroon	Koupé Manengouba	Count	2017	0	1	0
Cameroon	Mvila	Proportion	2000	14.4	25.9	7.1
Cameroon	Mvila	Proportion	2017	20.1	43.0	7.6
Cameroon	Mvila	Count	2000	0	0	0
Cameroon	Mvila	Count	2017	0	0	0
Cameroon	Mayo Banyo	Proportion	2000	17.2	30.2	8.6
Cameroon	Mayo Banyo	Proportion	2017	18.7	37.8	6.4
Cameroon	Mayo Banyo	Count	2000	0	0	0
Cameroon	Mayo Banyo	Count	2017	0	0	0
Cameroon	Mbam et Inoubou	Proportion	2000	13.1	24.0	6.9
Cameroon	Mbam et Inoubou	Proportion	2017	18.6	36.7	7.2
Cameroon	Mbam et Inoubou	Count	2000	0	0	0
Cameroon	Mbam et Inoubou	Count	2017	0	0	0
Cameroon	Kadey	Proportion	2000	12.0	20.4	5.8
Cameroon	Kadey	Proportion	2017	15.6	31.0	5.0
Cameroon	Kadey	Count	2000	0	0	0
Cameroon	Kadey	Count	2017	0	0	0
Cameroon	Mayo Danay	Proportion	2000	11.1	17.3	6.6
Cameroon	Mayo Danay	Proportion	2017	10.6	20.7	4.8
Cameroon	Mayo Danay	Count	2000	0	0	0
Cameroon	Mayo Danay	Count	2017	0	0	0

Table 4: ORS by Unit *(continued)*

Country	Unit	Metric	Year	Mean	Upper	Lower
Cameroon	Sanaga Maritime	Proportion	2000	28.6	41.7	16.0
Cameroon	Sanaga Maritime	Proportion	2017	34.0	59.1	14.0
Cameroon	Sanaga Maritime	Count	2000	0	0	0
Cameroon	Sanaga Maritime	Count	2017	0	1	0
Cameroon	Donga Mantung	Proportion	2000	19.6	34.1	10.2
Cameroon	Donga Mantung	Proportion	2017	20.1	44.3	7.4
Cameroon	Donga Mantung	Count	2000	0	0	0
Cameroon	Donga Mantung	Count	2017	0	0	0
Cameroon	Mayo Louti	Proportion	2000	9.1	14.7	5.0
Cameroon	Mayo Louti	Proportion	2017	12.7	25.6	4.7
Cameroon	Mayo Louti	Count	2000	0	0	0
Cameroon	Mayo Louti	Count	2017	0	0	0
Cameroon	Hauts Plateaux	Proportion	2000	15.2	25.3	8.9
Cameroon	Hauts Plateaux	Proportion	2017	16.6	36.2	5.9
Cameroon	Hauts Plateaux	Count	2000	0	0	0
Cameroon	Hauts Plateaux	Count	2017	0	0	0
Cameroon	Lebialem	Proportion	2000	16.4	26.6	9.1
Cameroon	Lebialem	Proportion	2017	17.8	39.4	6.0
Cameroon	Lebialem	Count	2000	0	0	0
Cameroon	Lebialem	Count	2017	0	0	0
Cameroon	Océan	Proportion	2000	24.2	41.1	12.1
Cameroon	Océan	Proportion	2017	30.4	52.9	13.4
Cameroon	Océan	Count	2000	0	0	0
Cameroon	Océan	Count	2017	0	1	0
Cameroon	Mbéré	Proportion	2000	13.9	26.7	6.1
Cameroon	Mbéré	Proportion	2017	16.9	31.6	7.3
Cameroon	Mbéré	Count	2000	0	0	0
Cameroon	Mbéré	Count	2017	0	0	0
Cameroon	Mbam et Kim	Proportion	2000	13.0	22.5	6.9
Cameroon	Mbam et Kim	Proportion	2017	17.8	34.5	7.2
Cameroon	Mbam et Kim	Count	2000	0	0	0
Cameroon	Mbam et Kim	Count	2017	0	0	0
Cameroon	Lom et Djerem	Proportion	2000	13.4	22.6	6.7
Cameroon	Lom et Djerem	Proportion	2017	16.7	31.4	6.9
Cameroon	Lom et Djerem	Count	2000	0	0	0
Cameroon	Lom et Djerem	Count	2017	0	0	0
Cameroon	Mayo Kani	Proportion	2000	10.3	16.0	6.0
Cameroon	Mayo Kani	Proportion	2017	11.1	21.7	4.5
Cameroon	Mayo Kani	Count	2000	0	0	0
Cameroon	Mayo Kani	Count	2017	0	0	0
Cameroon	Wouri	Proportion	2000	36.6	52.0	22.3
Cameroon	Wouri	Proportion	2017	39.3	67.1	17.4
Cameroon	Wouri	Count	2000	0	1	0
Cameroon	Wouri	Count	2017	0	1	0
Cameroon	Menchum	Proportion	2000	23.5	38.6	12.1
Cameroon	Menchum	Proportion	2017	23.8	48.2	8.8
Cameroon	Menchum	Count	2000	0	0	0
Cameroon	Menchum	Count	2017	0	0	0
Cameroon	Mayo Rey	Proportion	2000	9.8	16.8	5.4

Table 4: ORS by Unit *(continued)*

Country	Unit	Metric	Year	Mean	Upper	Lower
Cameroon	Mayo Rey	Proportion	2017	13.8	25.6	5.7
Cameroon	Mayo Rey	Count	2000	0	0	0
Cameroon	Mayo Rey	Count	2017	0	0	0
Cameroon	Koung Khi	Proportion	2000	15.6	26.6	8.8
Cameroon	Koung Khi	Proportion	2017	17.4	37.0	6.1
Cameroon	Koung Khi	Count	2000	0	0	0
Cameroon	Koung Khi	Count	2017	0	0	0
Cameroon	Manyu	Proportion	2000	25.8	40.4	13.6
Cameroon	Manyu	Proportion	2017	26.6	55.0	10.5
Cameroon	Manyu	Count	2000	0	0	0
Cameroon	Manyu	Count	2017	0	1	0
Cameroon	Vallée du Ntem	Proportion	2000	17.6	32.9	7.2
Cameroon	Vallée du Ntem	Proportion	2017	22.3	44.3	8.7
Cameroon	Vallée du Ntem	Count	2000	0	0	0
Cameroon	Vallée du Ntem	Count	2017	0	0	0
Cameroon	Vina	Proportion	2000	12.4	24.4	5.9
Cameroon	Vina	Proportion	2017	15.7	28.4	6.2
Cameroon	Vina	Count	2000	0	0	0
Cameroon	Vina	Count	2017	0	0	0
Cameroon	Mefou et Afamba	Proportion	2000	10.9	18.6	5.6
Cameroon	Mefou et Afamba	Proportion	2017	19.2	37.8	7.2
Cameroon	Mefou et Afamba	Count	2000	0	0	0
Cameroon	Mefou et Afamba	Count	2017	0	0	0
Cameroon	Mayo Sava	Proportion	2000	11.5	19.7	6.2
Cameroon	Mayo Sava	Proportion	2017	11.8	24.8	4.2
Cameroon	Mayo Sava	Count	2000	0	0	0
Cameroon	Mayo Sava	Count	2017	0	0	0
Cameroon	Mezam	Proportion	2000	20.9	32.7	12.5
Cameroon	Mezam	Proportion	2017	21.5	42.4	6.8
Cameroon	Mezam	Count	2000	0	0	0
Cameroon	Mezam	Count	2017	0	0	0
Cameroon	Menoua	Proportion	2000	18.2	30.0	10.5
Cameroon	Menoua	Proportion	2017	19.5	42.5	6.8
Cameroon	Menoua	Count	2000	0	0	0
Cameroon	Menoua	Count	2017	0	0	0
Cameroon	Meme	Proportion	2000	30.0	43.7	16.4
Cameroon	Meme	Proportion	2017	31.5	57.4	12.3
Cameroon	Meme	Count	2000	0	0	0
Cameroon	Meme	Count	2017	0	1	0
Cameroon	Mefou et Akono	Proportion	2000	11.1	18.5	6.1
Cameroon	Mefou et Akono	Proportion	2017	19.0	36.7	7.5
Cameroon	Mefou et Akono	Count	2000	0	0	0
Cameroon	Mefou et Akono	Count	2017	0	0	0
Cameroon	Mayo Tsanaga	Proportion	2000	9.8	16.4	5.2
Cameroon	Mayo Tsanaga	Proportion	2017	11.9	23.8	4.0
Cameroon	Mayo Tsanaga	Count	2000	0	0	0
Cameroon	Mayo Tsanaga	Count	2017	0	0	0
Cameroon	Momo	Proportion	2000	21.7	33.4	12.3
Cameroon	Momo	Proportion	2017	22.5	45.5	8.0

Table 4: ORS by Unit *(continued)*

Country	Unit	Metric	Year	Mean	Upper	Lower
Cameroon	Momo	Count	2000	0	0	0
Cameroon	Momo	Count	2017	0	0	0
Cameroon	Mifi	Proportion	2000	16.9	26.9	9.5
Cameroon	Mifi	Proportion	2017	18.6	37.6	6.6
Cameroon	Mifi	Count	2000	0	0	0
Cameroon	Mifi	Count	2017	0	0	0
Cameroon	Ndian	Proportion	2000	32.6	48.6	16.2
Cameroon	Ndian	Proportion	2017	34.3	57.7	13.9
Cameroon	Ndian	Count	2000	0	0	0
Cameroon	Ndian	Count	2017	0	1	0
Cameroon	Mfoundi	Proportion	2000	11.1	18.7	6.0
Cameroon	Mfoundi	Proportion	2017	19.4	38.5	7.2
Cameroon	Mfoundi	Count	2000	0	0	0
Cameroon	Mfoundi	Count	2017	0	0	0
Cameroon	Ngo Ketunjia	Proportion	2000	20.1	31.5	11.7
Cameroon	Ngo Ketunjia	Proportion	2017	20.9	42.7	7.2
Cameroon	Ngo Ketunjia	Count	2000	0	0	0
Cameroon	Ngo Ketunjia	Count	2017	0	0	0
Cameroon	Ndé	Proportion	2000	16.2	28.7	9.1
Cameroon	Ndé	Proportion	2017	18.9	38.2	6.7
Cameroon	Ndé	Count	2000	0	0	0
Cameroon	Ndé	Count	2017	0	0	0
Cameroon	Nyong et Kéllé	Proportion	2000	16.1	25.4	8.5
Cameroon	Nyong et Kéllé	Proportion	2017	24.0	43.6	9.6
Cameroon	Nyong et Kéllé	Count	2000	0	0	0
Cameroon	Nyong et Kéllé	Count	2017	0	0	0
Cameroon	Noun	Proportion	2000	17.7	27.8	9.7
Cameroon	Noun	Proportion	2017	19.3	39.7	7.4
Cameroon	Noun	Count	2000	0	0	0
Cameroon	Noun	Count	2017	0	0	0
Cameroon	Nyong et Mfoumou	Proportion	2000	11.3	20.8	5.2
Cameroon	Nyong et Mfoumou	Proportion	2017	17.8	35.9	6.5
Cameroon	Nyong et Mfoumou	Count	2000	0	0	0
Cameroon	Nyong et Mfoumou	Count	2017	0	0	0
Cameroon	Nyong et So'o	Proportion	2000	12.6	20.3	7.0
Cameroon	Nyong et So'o	Proportion	2017	20.6	39.5	8.4
Cameroon	Nyong et So'o	Count	2000	0	0	0
Cameroon	Nyong et So'o	Count	2017	0	0	0
Central African Republic	Bamingui	Proportion	2000	16.7	21.4	12.4
Central African Republic	Bamingui	Proportion	2017	20.8	35.3	8.9
Central African Republic	Bamingui	Count	2000	0	0	0
Central African Republic	Bamingui	Count	2017	0	0	0
Central African Republic	Bangui	Proportion	2000	24.1	27.6	20.6
Central African Republic	Bangui	Proportion	2017	26.9	47.7	10.8
Central African Republic	Bangui	Count	2000	0	0	0
Central African Republic	Bangui	Count	2017	0	0	0
Central African Republic	Alindao	Proportion	2000	8.3	11.2	5.8
Central African Republic	Alindao	Proportion	2017	18.7	33.8	6.7
Central African Republic	Alindao	Count	2000	0	0	0

Table 4: ORS by Unit *(continued)*

Country	Unit	Metric	Year	Mean	Upper	Lower
Central African Republic	Alindao	Count	2017	0	0	0
Central African Republic	Djemah	Proportion	2000	24.4	34.2	16.7
Central African Republic	Djemah	Proportion	2017	15.7	29.8	7.0
Central African Republic	Djemah	Count	2000	0	0	0
Central African Republic	Djemah	Count	2017	0	0	0
Central African Republic	Bria	Proportion	2000	18.8	23.3	14.2
Central African Republic	Bria	Proportion	2017	22.0	40.2	8.9
Central African Republic	Bria	Count	2000	0	0	0
Central African Republic	Bria	Count	2017	0	0	0
Central African Republic	Dékoa	Proportion	2000	20.7	25.2	16.8
Central African Republic	Dékoa	Proportion	2017	19.7	41.0	8.6
Central African Republic	Dékoa	Count	2000	0	0	0
Central African Republic	Dékoa	Count	2017	0	0	0
Central African Republic	Boda	Proportion	2000	19.8	25.4	15.1
Central African Republic	Boda	Proportion	2017	22.5	45.2	8.8
Central African Republic	Boda	Count	2000	0	0	0
Central African Republic	Boda	Count	2017	0	0	0
Central African Republic	Berbérati	Proportion	2000	16.8	23.3	11.8
Central African Republic	Berbérati	Proportion	2017	23.4	43.2	8.0
Central African Republic	Berbérati	Count	2000	0	0	0
Central African Republic	Berbérati	Count	2017	0	0	0
Central African Republic	Bakouma	Proportion	2000	13.6	17.8	10.0
Central African Republic	Bakouma	Proportion	2017	20.4	37.8	8.2
Central African Republic	Bakouma	Count	2000	0	0	0
Central African Republic	Bakouma	Count	2017	0	0	0
Central African Republic	Kaga-Bandoro	Proportion	2000	19.5	23.7	15.0
Central African Republic	Kaga-Bandoro	Proportion	2017	20.7	40.2	8.6
Central African Republic	Kaga-Bandoro	Count	2000	0	0	0
Central African Republic	Kaga-Bandoro	Count	2017	0	0	0
Central African Republic	Baboua	Proportion	2000	15.3	22.6	9.7
Central African Republic	Baboua	Proportion	2017	22.8	40.9	8.4
Central African Republic	Baboua	Count	2000	0	0	0
Central African Republic	Baboua	Count	2017	0	0	0
Central African Republic	Bimbo	Proportion	2000	22.3	25.1	18.8
Central African Republic	Bimbo	Proportion	2017	25.3	45.0	9.6
Central African Republic	Bimbo	Count	2000	0	0	0
Central African Republic	Bimbo	Count	2017	0	0	0
Central African Republic	Bakala	Proportion	2000	16.0	21.5	12.1
Central African Republic	Bakala	Proportion	2017	18.1	36.7	7.5
Central African Republic	Bakala	Count	2000	0	0	0
Central African Republic	Bakala	Count	2017	0	0	0
Central African Republic	Bocaranga	Proportion	2000	15.8	21.6	10.6
Central African Republic	Bocaranga	Proportion	2017	22.9	39.2	9.9
Central African Republic	Bocaranga	Count	2000	0	0	0
Central African Republic	Bocaranga	Count	2017	0	0	0
Central African Republic	Batangafo	Proportion	2000	21.6	29.0	15.5
Central African Republic	Batangafo	Proportion	2017	21.4	41.4	8.6
Central African Republic	Batangafo	Count	2000	0	0	0
Central African Republic	Batangafo	Count	2017	0	0	0

Table 4: ORS by Unit *(continued)*

Country	Unit	Metric	Year	Mean	Upper	Lower
Central African Republic	Bambio	Proportion	2000	20.0	25.7	15.2
Central African Republic	Bambio	Proportion	2017	23.1	47.3	8.3
Central African Republic	Bambio	Count	2000	0	0	0
Central African Republic	Bambio	Count	2017	0	0	0
Central African Republic	Birao	Proportion	2000	11.2	15.3	7.7
Central African Republic	Birao	Proportion	2017	20.4	34.7	9.2
Central African Republic	Birao	Count	2000	0	0	0
Central African Republic	Birao	Count	2017	0	0	0
Central African Republic	Ndélé	Proportion	2000	15.6	21.1	11.5
Central African Republic	Ndélé	Proportion	2017	20.3	36.4	9.3
Central African Republic	Ndélé	Count	2000	0	0	0
Central African Republic	Ndélé	Count	2017	0	0	0
Central African Republic	Kembé	Proportion	2000	7.8	10.4	5.5
Central African Republic	Kembé	Proportion	2017	18.3	34.5	6.6
Central African Republic	Kembé	Count	2000	0	0	0
Central African Republic	Kembé	Count	2017	0	0	0
Central African Republic	Obo	Proportion	2000	43.3	52.0	33.2
Central African Republic	Obo	Proportion	2017	25.1	50.2	10.7
Central African Republic	Obo	Count	2000	0	1	0
Central African Republic	Obo	Count	2017	0	1	0
Central African Republic	Ouadda	Proportion	2000	18.6	25.1	12.6
Central African Republic	Ouadda	Proportion	2017	23.7	44.4	9.9
Central African Republic	Ouadda	Count	2000	0	0	0
Central African Republic	Ouadda	Count	2017	0	0	0
Central African Republic	Sibut	Proportion	2000	22.2	27.4	18.3
Central African Republic	Sibut	Proportion	2017	19.0	35.8	8.1
Central African Republic	Sibut	Count	2000	0	0	0
Central African Republic	Sibut	Count	2017	0	0	0
Central African Republic	M'Baïki	Proportion	2000	19.3	23.7	15.3
Central African Republic	M'Baïki	Proportion	2017	21.6	40.2	8.0
Central African Republic	M'Baïki	Count	2000	0	0	0
Central African Republic	M'Baïki	Count	2017	0	0	0
Central African Republic	Carnot	Proportion	2000	17.7	23.4	13.7
Central African Republic	Carnot	Proportion	2017	23.2	42.5	8.1
Central African Republic	Carnot	Count	2000	0	0	0
Central African Republic	Carnot	Count	2017	0	0	0
Central African Republic	Bangassou	Proportion	2000	12.4	17.3	9.0
Central African Republic	Bangassou	Proportion	2017	19.1	35.2	7.4
Central African Republic	Bangassou	Count	2000	0	0	0
Central African Republic	Bangassou	Count	2017	0	0	0
Central African Republic	Mbrès	Proportion	2000	18.7	23.4	13.8
Central African Republic	Mbrès	Proportion	2017	20.3	38.3	8.3
Central African Republic	Mbrès	Count	2000	0	0	0
Central African Republic	Mbrès	Count	2017	0	0	0
Central African Republic	Baoro	Proportion	2000	16.4	22.2	12.0
Central African Republic	Baoro	Proportion	2017	23.4	44.4	9.1
Central African Republic	Baoro	Count	2000	0	0	0
Central African Republic	Baoro	Count	2017	0	0	0
Central African Republic	Boali	Proportion	2000	23.8	29.7	18.8

Table 4: ORS by Unit *(continued)*

Country	Unit	Metric	Year	Mean	Upper	Lower
Central African Republic	Boali	Proportion	2017	24.4	45.2	8.8
Central African Republic	Boali	Count	2000	0	0	0
Central African Republic	Boali	Count	2017	0	0	0
Central African Republic	Bambari	Proportion	2000	13.4	17.4	10.2
Central African Republic	Bambari	Proportion	2017	18.4	32.6	7.6
Central African Republic	Bambari	Count	2000	0	0	0
Central African Republic	Bambari	Count	2017	0	0	0
Central African Republic	Bozoum	Proportion	2000	16.9	23.2	12.7
Central African Republic	Bozoum	Proportion	2017	23.4	44.5	9.2
Central African Republic	Bozoum	Count	2000	0	0	0
Central African Republic	Bozoum	Count	2017	0	0	0
Central African Republic	Bossangoa	Proportion	2000	20.9	29.2	15.3
Central African Republic	Bossangoa	Proportion	2017	20.4	42.2	7.7
Central African Republic	Bossangoa	Count	2000	0	0	0
Central African Republic	Bossangoa	Count	2017	0	0	0
Central African Republic	Nola	Proportion	2000	19.2	25.0	14.9
Central African Republic	Nola	Proportion	2017	23.7	43.1	8.0
Central African Republic	Nola	Count	2000	0	0	0
Central African Republic	Nola	Count	2017	0	0	0
Central African Republic	Ouanda Djallé	Proportion	2000	13.9	20.4	9.0
Central African Republic	Ouanda Djallé	Proportion	2017	22.1	40.5	10.1
Central African Republic	Ouanda Djallé	Count	2000	0	0	0
Central African Republic	Ouanda Djallé	Count	2017	0	0	0
Central African Republic	Mingala	Proportion	2000	10.2	14.2	6.8
Central African Republic	Mingala	Proportion	2017	20.3	36.7	7.6
Central African Republic	Mingala	Count	2000	0	0	0
Central African Republic	Mingala	Count	2017	0	0	0
Central African Republic	Zémio	Proportion	2000	33.5	45.7	22.8
Central African Republic	Zémio	Proportion	2017	22.7	41.6	9.8
Central African Republic	Zémio	Count	2000	0	0	0
Central African Republic	Zémio	Count	2017	0	0	0
Central African Republic	Yalinga	Proportion	2000	18.6	25.1	13.5
Central African Republic	Yalinga	Proportion	2017	21.6	39.7	9.3
Central African Republic	Yalinga	Count	2000	0	0	0
Central African Republic	Yalinga	Count	2017	0	0	0
Central African Republic	Mongoumba	Proportion	2000	17.7	23.8	13.0
Central African Republic	Mongoumba	Proportion	2017	20.2	42.1	8.5
Central African Republic	Mongoumba	Count	2000	0	0	0
Central African Republic	Mongoumba	Count	2017	0	0	0
Central African Republic	Gamboula	Proportion	2000	17.2	24.0	11.7
Central African Republic	Gamboula	Proportion	2017	23.6	42.4	8.6
Central African Republic	Gamboula	Count	2000	0	0	0
Central African Republic	Gamboula	Count	2017	0	0	0
Central African Republic	Gambo-Ouango	Proportion	2000	11.1	15.1	7.6
Central African Republic	Gambo-Ouango	Proportion	2017	19.2	35.8	7.2
Central African Republic	Gambo-Ouango	Count	2000	0	0	0
Central African Republic	Gambo-Ouango	Count	2017	0	0	0
Central African Republic	Bouar	Proportion	2000	15.5	21.5	10.3
Central African Republic	Bouar	Proportion	2017	22.6	45.1	8.6

Table 4: ORS by Unit *(continued)*

Country	Unit	Metric	Year	Mean	Upper	Lower
Central African Republic	Bouar	Count	2000	0	0	0
Central African Republic	Bouar	Count	2017	0	0	0
Central African Republic	Damara	Proportion	2000	23.3	28.4	18.7
Central African Republic	Damara	Proportion	2017	21.7	38.5	8.6
Central African Republic	Damara	Count	2000	0	0	0
Central African Republic	Damara	Count	2017	0	0	0
Central African Republic	Grimari	Proportion	2000	17.0	21.8	12.8
Central African Republic	Grimari	Proportion	2017	17.8	33.4	7.7
Central African Republic	Grimari	Count	2000	0	0	0
Central African Republic	Grimari	Count	2017	0	0	0
Central African Republic	Paoua	Proportion	2000	15.9	21.4	10.9
Central African Republic	Paoua	Proportion	2017	21.7	40.1	8.0
Central African Republic	Paoua	Count	2000	0	0	0
Central African Republic	Paoua	Count	2017	0	0	0
Central African Republic	Bouca	Proportion	2000	22.1	28.8	16.4
Central African Republic	Bouca	Proportion	2017	20.5	41.8	7.3
Central African Republic	Bouca	Count	2000	0	0	0
Central African Republic	Bouca	Count	2017	0	0	0
Central African Republic	Mobaye	Proportion	2000	7.6	11.0	5.1
Central African Republic	Mobaye	Proportion	2017	16.8	30.3	6.7
Central African Republic	Mobaye	Count	2000	0	0	0
Central African Republic	Mobaye	Count	2017	0	0	0
Central African Republic	Rafaï	Proportion	2000	19.3	27.3	13.2
Central African Republic	Rafaï	Proportion	2017	19.1	35.6	8.2
Central African Republic	Rafaï	Count	2000	0	0	0
Central African Republic	Rafaï	Count	2017	0	0	0
Central African Republic	Yaloké	Proportion	2000	23.1	29.7	17.4
Central African Republic	Yaloké	Proportion	2017	24.6	45.4	9.8
Central African Republic	Yaloké	Count	2000	0	0	0
Central African Republic	Yaloké	Count	2017	0	0	0
Central African Republic	Ippy	Proportion	2000	14.8	19.3	11.0
Central African Republic	Ippy	Proportion	2017	19.4	35.8	7.8
Central African Republic	Ippy	Count	2000	0	0	0
Central African Republic	Ippy	Count	2017	0	0	0
Central African Republic	Kabo	Proportion	2000	20.1	28.3	14.2
Central African Republic	Kabo	Proportion	2017	22.8	42.0	10.0
Central African Republic	Kabo	Count	2000	0	0	0
Central African Republic	Kabo	Count	2017	0	0	0
Central African Republic	Kouango	Proportion	2000	12.4	16.8	9.2
Central African Republic	Kouango	Proportion	2017	15.2	26.8	6.9
Central African Republic	Kouango	Count	2000	0	0	0
Central African Republic	Kouango	Count	2017	0	0	0
Central African Republic	Markounda	Proportion	2000	19.0	26.0	12.9
Central African Republic	Markounda	Proportion	2017	20.4	37.8	7.7
Central African Republic	Markounda	Count	2000	0	0	0
Central African Republic	Markounda	Count	2017	0	0	0
Chad	Barh El Gazel	Proportion	2000	12.2	21.3	6.2
Chad	Barh El Gazel	Proportion	2017	17.9	34.3	8.1
Chad	Barh El Gazel	Count	2000	0	0	0

Table 4: ORS by Unit *(continued)*

Country	Unit	Metric	Year	Mean	Upper	Lower
Chad	Barh El Gazel	Count	2017	0	0	0
Chad	Batha Est	Proportion	2000	13.1	23.4	5.6
Chad	Batha Est	Proportion	2017	14.5	28.1	6.1
Chad	Batha Est	Count	2000	0	0	0
Chad	Batha Est	Count	2017	0	0	0
Chad	Borkou	Proportion	2000	15.4	25.0	7.8
Chad	Borkou	Proportion	2017	22.8	36.9	11.2
Chad	Borkou	Count	2000	0	0	0
Chad	Borkou	Count	2017	0	0	0
Chad	Baguirmi	Proportion	2000	17.0	26.5	9.6
Chad	Baguirmi	Proportion	2017	13.0	24.9	5.1
Chad	Baguirmi	Count	2000	0	0	0
Chad	Baguirmi	Count	2017	0	0	0
Chad	Ennedi Est	Proportion	2000	13.5	25.3	5.3
Chad	Ennedi Est	Proportion	2017	24.6	44.6	9.6
Chad	Ennedi Est	Count	2000	0	0	0
Chad	Ennedi Est	Count	2017	0	0	0
Chad	Ennedi Ouest	Proportion	2000	14.6	27.2	6.5
Chad	Ennedi Ouest	Proportion	2017	24.0	39.9	10.4
Chad	Ennedi Ouest	Count	2000	0	0	0
Chad	Ennedi Ouest	Count	2017	0	0	0
Chad	Barh Signaka	Proportion	2000	14.5	28.5	5.1
Chad	Barh Signaka	Proportion	2017	17.4	32.9	6.5
Chad	Barh Signaka	Count	2000	0	0	0
Chad	Barh Signaka	Count	2017	0	0	0
Chad	Dababa	Proportion	2000	13.6	22.5	6.6
Chad	Dababa	Proportion	2017	14.9	27.3	6.7
Chad	Dababa	Count	2000	0	0	0
Chad	Dababa	Count	2017	0	0	0
Chad	Kanem	Proportion	2000	12.6	23.2	6.1
Chad	Kanem	Proportion	2017	17.0	32.3	6.8
Chad	Kanem	Count	2000	0	0	0
Chad	Kanem	Count	2017	0	0	0
Chad	Mamdi	Proportion	2000	12.8	22.5	7.1
Chad	Mamdi	Proportion	2017	15.5	28.5	5.4
Chad	Mamdi	Count	2000	0	0	0
Chad	Mamdi	Count	2017	0	0	0
Chad	Dodjé	Proportion	2000	17.6	29.1	10.4
Chad	Dodjé	Proportion	2017	17.7	35.0	6.9
Chad	Dodjé	Count	2000	0	0	0
Chad	Dodjé	Count	2017	0	0	0
Chad	Lanya	Proportion	2000	14.8	23.9	7.5
Chad	Lanya	Proportion	2017	18.6	39.1	7.6
Chad	Lanya	Count	2000	0	0	0
Chad	Lanya	Count	2017	0	0	0
Chad	Barh Sara	Proportion	2000	11.2	20.0	5.6
Chad	Barh Sara	Proportion	2017	17.0	33.9	7.1
Chad	Barh Sara	Count	2000	0	0	0
Chad	Barh Sara	Count	2017	0	0	0

Table 4: ORS by Unit *(continued)*

Country	Unit	Metric	Year	Mean	Upper	Lower
Chad	Kabbia	Proportion	2000	17.6	30.0	9.8
Chad	Kabbia	Proportion	2017	17.7	34.7	7.2
Chad	Kabbia	Count	2000	0	0	0
Chad	Kabbia	Count	2017	0	0	0
Chad	Lac Léré	Proportion	2000	16.3	25.7	9.3
Chad	Lac Léré	Proportion	2017	18.0	33.2	8.1
Chad	Lac Léré	Count	2000	0	0	0
Chad	Lac Léré	Count	2017	0	0	0
Chad	Barh Köh	Proportion	2000	10.6	18.2	5.8
Chad	Barh Köh	Proportion	2017	16.7	31.5	7.1
Chad	Barh Köh	Count	2000	0	0	0
Chad	Barh Köh	Count	2017	0	0	0
Chad	Assoungha	Proportion	2000	13.0	23.1	6.0
Chad	Assoungha	Proportion	2017	15.1	29.7	5.8
Chad	Assoungha	Count	2000	0	0	0
Chad	Assoungha	Count	2017	0	0	0
Chad	Aboudeïa	Proportion	2000	12.9	24.8	5.3
Chad	Aboudeïa	Proportion	2017	16.8	31.8	6.5
Chad	Aboudeïa	Count	2000	0	0	0
Chad	Aboudeïa	Count	2017	0	0	0
Chad	Djourf Al Ahmar	Proportion	2000	12.7	21.7	5.7
Chad	Djourf Al Ahmar	Proportion	2017	14.3	26.1	5.8
Chad	Djourf Al Ahmar	Count	2000	0	0	0
Chad	Djourf Al Ahmar	Count	2017	0	0	0
Chad	Béré	Proportion	2000	18.0	27.5	10.3
Chad	Béré	Proportion	2017	17.8	33.4	7.6
Chad	Béré	Count	2000	0	0	0
Chad	Béré	Count	2017	0	0	0
Chad	Tibesti	Proportion	2000	17.2	30.6	7.4
Chad	Tibesti	Proportion	2017	22.5	39.1	10.8
Chad	Tibesti	Count	2000	0	0	0
Chad	Tibesti	Count	2017	0	0	0
Chad	N'Djamena	Proportion	2000	26.4	36.8	17.4
Chad	N'Djamena	Proportion	2017	20.8	38.6	9.2
Chad	N'Djamena	Count	2000	0	0	0
Chad	N'Djamena	Count	2017	0	0	0
Chad	Biltine	Proportion	2000	13.1	26.0	6.0
Chad	Biltine	Proportion	2017	20.2	34.9	7.8
Chad	Biltine	Count	2000	0	0	0
Chad	Biltine	Count	2017	0	0	0
Chad	Batha Oues	Proportion	2000	12.5	21.3	5.7
Chad	Batha Oues	Proportion	2017	14.5	26.2	6.0
Chad	Batha Oues	Count	2000	0	0	0
Chad	Batha Oues	Count	2017	0	0	0
Chad	Loug Chari	Proportion	2000	15.5	27.4	8.0
Chad	Loug Chari	Proportion	2017	14.7	28.2	5.9
Chad	Loug Chari	Count	2000	0	0	0
Chad	Loug Chari	Count	2017	0	0	0
Chad	Bitkine	Proportion	2000	14.1	27.1	6.2

Table 4: ORS by Unit *(continued)*

Country	Unit	Metric	Year	Mean	Upper	Lower
Chad	Bitkine	Proportion	2017	15.5	29.2	6.3
Chad	Bitkine	Count	2000	0	0	0
Chad	Bitkine	Count	2017	0	0	0
Chad	Dagana	Proportion	2000	14.7	26.3	8.3
Chad	Dagana	Proportion	2017	15.6	30.1	6.9
Chad	Dagana	Count	2000	0	0	0
Chad	Dagana	Count	2017	0	0	0
Chad	Nokou	Proportion	2000	12.2	22.2	5.8
Chad	Nokou	Proportion	2017	18.7	33.4	8.0
Chad	Nokou	Count	2000	0	0	0
Chad	Nokou	Count	2017	0	0	0
Chad	Wayi	Proportion	2000	13.3	24.4	6.9
Chad	Wayi	Proportion	2017	15.3	29.8	5.9
Chad	Wayi	Count	2000	0	0	0
Chad	Wayi	Count	2017	0	0	0
Chad	Lac Wey	Proportion	2000	17.4	27.0	10.1
Chad	Lac Wey	Proportion	2017	17.5	34.6	7.3
Chad	Lac Wey	Count	2000	0	0	0
Chad	Lac Wey	Count	2017	0	0	0
Chad	Monts de Lam	Proportion	2000	15.4	23.9	8.3
Chad	Monts de Lam	Proportion	2017	19.9	38.9	8.8
Chad	Monts de Lam	Count	2000	0	0	0
Chad	Monts de Lam	Count	2017	0	0	0
Chad	Mandoul Occidental	Proportion	2000	12.2	20.9	6.6
Chad	Mandoul Occidental	Proportion	2017	18.8	39.2	7.6
Chad	Mandoul Occidental	Count	2000	0	0	0
Chad	Mandoul Occidental	Count	2017	0	0	0
Chad	Mayo-Boneye	Proportion	2000	17.6	27.1	9.3
Chad	Mayo-Boneye	Proportion	2017	15.1	27.4	6.8
Chad	Mayo-Boneye	Count	2000	0	0	0
Chad	Mayo-Boneye	Count	2017	0	0	0
Chad	Mayo-Dallah	Proportion	2000	17.1	28.4	9.8
Chad	Mayo-Dallah	Proportion	2017	17.5	35.1	6.7
Chad	Mayo-Dallah	Count	2000	0	0	0
Chad	Mayo-Dallah	Count	2017	0	0	0
Chad	Grande Sido	Proportion	2000	10.8	19.8	4.9
Chad	Grande Sido	Proportion	2017	16.4	30.9	6.3
Chad	Grande Sido	Count	2000	0	0	0
Chad	Grande Sido	Count	2017	0	0	0
Chad	Djourf Al Ahmar	Proportion	2000	13.5	22.9	6.2
Chad	Djourf Al Ahmar	Proportion	2017	14.5	29.2	5.2
Chad	Djourf Al Ahmar	Count	2000	0	0	0
Chad	Djourf Al Ahmar	Count	2017	0	0	0
Chad	Barh Azoum	Proportion	2000	11.8	22.8	5.0
Chad	Barh Azoum	Proportion	2017	14.3	27.1	6.6
Chad	Barh Azoum	Count	2000	0	0	0
Chad	Barh Azoum	Count	2017	0	0	0
Chad	Sila	Proportion	2000	12.4	21.9	5.3
Chad	Sila	Proportion	2017	13.8	26.7	5.5

Table 4: ORS by Unit *(continued)*

Country	Unit	Metric	Year	Mean	Upper	Lower
Chad	Sila	Count	2000	0	0	0
Chad	Sila	Count	2017	0	0	0
Chad	Tandjilé Est	Proportion	2000	15.5	24.4	9.1
Chad	Tandjilé Est	Proportion	2017	18.2	33.4	8.0
Chad	Tandjilé Est	Count	2000	0	0	0
Chad	Tandjilé Est	Count	2017	0	0	0
Chad	Dar Tama	Proportion	2000	11.7	20.6	4.7
Chad	Dar Tama	Proportion	2017	18.5	32.0	7.1
Chad	Dar Tama	Count	2000	0	0	0
Chad	Dar Tama	Count	2017	0	0	0
Chad	Fitri	Proportion	2000	12.1	21.0	5.4
Chad	Fitri	Proportion	2017	14.8	30.2	6.9
Chad	Fitri	Count	2000	0	0	0
Chad	Fitri	Count	2017	0	0	0
Chad	N'Djamena	Proportion	2000	23.8	34.1	15.1
Chad	N'Djamena	Proportion	2017	18.0	32.9	7.4
Chad	N'Djamena	Count	2000	0	0	0
Chad	N'Djamena	Count	2017	0	0	0
Chad	Guéra	Proportion	2000	12.5	22.3	6.2
Chad	Guéra	Proportion	2017	14.8	27.2	6.1
Chad	Guéra	Count	2000	0	0	0
Chad	Guéra	Count	2017	0	0	0
Chad	Haraze Al Biar	Proportion	2000	18.5	28.1	11.5
Chad	Haraze Al Biar	Proportion	2017	18.4	36.2	8.1
Chad	Haraze Al Biar	Count	2000	0	0	0
Chad	Haraze Al Biar	Count	2017	0	0	0
Chad	Ngourkosso	Proportion	2000	16.9	25.4	9.9
Chad	Ngourkosso	Proportion	2017	17.1	33.2	6.8
Chad	Ngourkosso	Count	2000	0	0	0
Chad	Ngourkosso	Count	2017	0	0	0
Chad	Nya Pendé	Proportion	2000	15.2	24.2	8.6
Chad	Nya Pendé	Proportion	2017	19.4	37.3	8.4
Chad	Nya Pendé	Count	2000	0	0	0
Chad	Nya Pendé	Count	2017	0	0	0
Chad	Mandoul Oriental	Proportion	2000	11.5	20.8	5.8
Chad	Mandoul Oriental	Proportion	2017	18.3	36.6	7.7
Chad	Mandoul Oriental	Count	2000	0	0	0
Chad	Mandoul Oriental	Count	2017	0	0	0
Chad	Mont Illi	Proportion	2000	17.2	27.9	9.8
Chad	Mont Illi	Proportion	2017	17.2	31.8	7.9
Chad	Mont Illi	Count	2000	0	0	0
Chad	Mont Illi	Count	2017	0	0	0
Chad	Lac Iro	Proportion	2000	11.1	20.1	5.1
Chad	Lac Iro	Proportion	2017	16.3	29.7	7.0
Chad	Lac Iro	Count	2000	0	0	0
Chad	Lac Iro	Count	2017	0	0	0
Chad	Ouara	Proportion	2000	13.1	22.3	5.8
Chad	Ouara	Proportion	2017	16.0	29.4	6.1
Chad	Ouara	Count	2000	0	0	0

Table 4: ORS by Unit *(continued)*

Country	Unit	Metric	Year	Mean	Upper	Lower
Chad	Ouara	Count	2017	0	0	0
Chad	Haraze Mangueigne	Proportion	2000	11.3	21.0	4.7
Chad	Haraze Mangueigne	Proportion	2017	13.6	25.6	5.4
Chad	Haraze Mangueigne	Count	2000	0	0	0
Chad	Haraze Mangueigne	Count	2017	0	0	0
Chad	Tandjilé Ouest	Proportion	2000	18.5	31.3	10.6
Chad	Tandjilé Ouest	Proportion	2017	18.2	36.2	7.8
Chad	Tandjilé Ouest	Count	2000	0	0	0
Chad	Tandjilé Ouest	Count	2017	0	0	0
Chad	Kobé	Proportion	2000	12.8	23.6	5.8
Chad	Kobé	Proportion	2017	23.1	39.9	8.8
Chad	Kobé	Count	2000	0	0	0
Chad	Kobé	Count	2017	0	0	0
Chad	Mangalmé	Proportion	2000	13.7	25.0	5.4
Chad	Mangalmé	Proportion	2017	16.7	32.1	6.2
Chad	Mangalmé	Count	2000	0	0	0
Chad	Mangalmé	Count	2017	0	0	0
Chad	Pendé	Proportion	2000	13.7	23.0	7.2
Chad	Pendé	Proportion	2017	19.4	37.9	8.2
Chad	Pendé	Count	2000	0	0	0
Chad	Pendé	Count	2017	0	0	0
Colombia	El Encanto	Proportion	2000	39.0	50.9	28.9
Colombia	El Encanto	Proportion	2017	53.9	65.9	41.8
Colombia	El Encanto	Count	2000	0	1	0
Colombia	El Encanto	Count	2017	1	1	0
Colombia	Abejorral	Proportion	2000	35.1	41.9	28.0
Colombia	Abejorral	Proportion	2017	49.2	62.0	38.6
Colombia	Abejorral	Count	2000	0	0	0
Colombia	Abejorral	Count	2017	0	1	0
Colombia	Arauca	Proportion	2000	38.9	48.9	30.2
Colombia	Arauca	Proportion	2017	52.5	62.7	40.1
Colombia	Arauca	Count	2000	0	0	0
Colombia	Arauca	Count	2017	1	1	0
Colombia	Baranoa	Proportion	2000	45.3	54.6	36.9
Colombia	Baranoa	Proportion	2017	54.4	64.5	43.2
Colombia	Baranoa	Count	2000	0	1	0
Colombia	Baranoa	Count	2017	1	1	0
Colombia	Achí	Proportion	2000	40.1	48.4	31.2
Colombia	Achí	Proportion	2017	52.4	62.2	41.9
Colombia	Achí	Count	2000	0	0	0
Colombia	Achí	Count	2017	1	1	0
Colombia	Almeida	Proportion	2000	27.8	34.4	21.7
Colombia	Almeida	Proportion	2017	44.5	58.6	32.0
Colombia	Almeida	Count	2000	0	0	0
Colombia	Almeida	Count	2017	0	1	0
Colombia	Aguadas	Proportion	2000	35.8	42.4	28.2
Colombia	Aguadas	Proportion	2017	53.9	65.8	43.8
Colombia	Aguadas	Count	2000	0	0	0
Colombia	Aguadas	Count	2017	1	1	0

Table 4: ORS by Unit *(continued)*

Country	Unit	Metric	Year	Mean	Upper	Lower
Colombia	Albania	Proportion	2000	36.8	46.2	26.9
Colombia	Albania	Proportion	2017	45.1	54.9	34.6
Colombia	Albania	Count	2000	0	0	0
Colombia	Albania	Count	2017	0	1	0
Colombia	Aguazul	Proportion	2000	33.4	42.4	25.5
Colombia	Aguazul	Proportion	2017	46.7	59.2	33.7
Colombia	Aguazul	Count	2000	0	0	0
Colombia	Aguazul	Count	2017	0	1	0
Colombia	Almaguer	Proportion	2000	32.5	40.7	25.1
Colombia	Almaguer	Proportion	2017	52.9	63.5	41.2
Colombia	Almaguer	Count	2000	0	0	0
Colombia	Almaguer	Count	2017	1	1	0
Colombia	Aguachica	Proportion	2000	38.6	46.7	30.0
Colombia	Aguachica	Proportion	2017	52.9	64.2	41.7
Colombia	Aguachica	Count	2000	0	0	0
Colombia	Aguachica	Count	2017	1	1	0
Colombia	Acandí	Proportion	2000	36.0	47.2	26.7
Colombia	Acandí	Proportion	2017	46.9	58.5	36.6
Colombia	Acandí	Count	2000	0	0	0
Colombia	Acandí	Count	2017	0	1	0
Colombia	Ayapel	Proportion	2000	36.7	45.7	29.2
Colombia	Ayapel	Proportion	2017	54.9	66.1	44.2
Colombia	Ayapel	Count	2000	0	0	0
Colombia	Ayapel	Count	2017	1	1	0
Colombia	Agua de Dios	Proportion	2000	37.1	43.0	30.8
Colombia	Agua de Dios	Proportion	2017	53.3	65.3	41.6
Colombia	Agua de Dios	Count	2000	0	0	0
Colombia	Agua de Dios	Count	2017	1	1	0
Colombia	Barranco Minas	Proportion	2000	41.0	50.4	31.1
Colombia	Barranco Minas	Proportion	2017	51.8	62.7	40.1
Colombia	Barranco Minas	Count	2000	0	1	0
Colombia	Barranco Minas	Count	2017	1	1	0
Colombia	Calamar	Proportion	2000	35.8	45.5	25.7
Colombia	Calamar	Proportion	2017	45.6	58.3	34.2
Colombia	Calamar	Count	2000	0	0	0
Colombia	Calamar	Count	2017	0	1	0
Colombia	Acevedo	Proportion	2000	35.7	44.2	27.3
Colombia	Acevedo	Proportion	2017	52.9	63.7	40.8
Colombia	Acevedo	Count	2000	0	0	0
Colombia	Acevedo	Count	2017	1	1	0
Colombia	Barrancas	Proportion	2000	37.0	47.5	27.9
Colombia	Barrancas	Proportion	2017	53.5	64.7	43.4
Colombia	Barrancas	Count	2000	0	0	0
Colombia	Barrancas	Count	2017	1	1	0
Colombia	Aracataca	Proportion	2000	43.2	52.1	35.1
Colombia	Aracataca	Proportion	2017	55.7	65.7	45.3
Colombia	Aracataca	Count	2000	0	1	0
Colombia	Aracataca	Count	2017	1	1	0
Colombia	Acacías	Proportion	2000	30.6	38.1	24.0

Table 4: ORS by Unit *(continued)*

Country	Unit	Metric	Year	Mean	Upper	Lower
Colombia	Acacías	Proportion	2017	45.4	57.5	33.4
Colombia	Acacías	Count	2000	0	0	0
Colombia	Acacías	Count	2017	0	1	0
Colombia	Albán	Proportion	2000	36.0	45.2	27.1
Colombia	Albán	Proportion	2017	55.7	67.4	44.4
Colombia	Albán	Count	2000	0	0	0
Colombia	Albán	Count	2017	1	1	0
Colombia	Abrego	Proportion	2000	39.8	47.4	31.7
Colombia	Abrego	Proportion	2017	51.1	63.5	39.9
Colombia	Abrego	Count	2000	0	0	0
Colombia	Abrego	Count	2017	1	1	0
Colombia	Colón	Proportion	2000	32.2	41.8	23.5
Colombia	Colón	Proportion	2017	49.6	61.6	38.8
Colombia	Colón	Count	2000	0	0	0
Colombia	Colón	Count	2017	0	1	0
Colombia	Armenia	Proportion	2000	35.4	42.8	28.4
Colombia	Armenia	Proportion	2017	49.7	63.0	38.3
Colombia	Armenia	Count	2000	0	0	0
Colombia	Armenia	Count	2017	0	1	0
Colombia	Apía	Proportion	2000	33.3	40.6	26.2
Colombia	Apía	Proportion	2017	49.9	62.8	39.5
Colombia	Apía	Count	2000	0	0	0
Colombia	Apía	Count	2017	0	1	0
Colombia	n.a. (1)	Proportion	2000	NA	NA	NA
Colombia	n.a. (1)	Proportion	2017	NA	NA	NA
Colombia	n.a. (1)	Count	2000	NA	NA	NA
Colombia	n.a. (1)	Count	2017	NA	NA	NA
Colombia	Aguada	Proportion	2000	34.3	42.7	26.1
Colombia	Aguada	Proportion	2017	48.6	62.1	37.2
Colombia	Aguada	Count	2000	0	0	0
Colombia	Aguada	Count	2017	0	1	0
Colombia	Buenavista	Proportion	2000	43.7	52.8	35.7
Colombia	Buenavista	Proportion	2017	55.6	66.5	44.2
Colombia	Buenavista	Count	2000	0	1	0
Colombia	Buenavista	Count	2017	1	1	0
Colombia	Alpujarra	Proportion	2000	34.5	43.4	26.8
Colombia	Alpujarra	Proportion	2017	50.8	61.6	38.9
Colombia	Alpujarra	Count	2000	0	0	0
Colombia	Alpujarra	Count	2017	1	1	0
Colombia	Alcalá	Proportion	2000	34.3	41.3	28.1
Colombia	Alcalá	Proportion	2017	52.8	66.4	41.7
Colombia	Alcalá	Count	2000	0	0	0
Colombia	Alcalá	Count	2017	1	1	0
Colombia	Carurú	Proportion	2000	27.9	37.8	18.5
Colombia	Carurú	Proportion	2017	38.3	50.9	27.2
Colombia	Carurú	Count	2000	0	0	0
Colombia	Carurú	Count	2017	0	1	0
Colombia	Cumaribo	Proportion	2000	34.4	45.8	25.4
Colombia	Cumaribo	Proportion	2017	47.9	59.0	36.1

Table 4: ORS by Unit *(continued)*

Country	Unit	Metric	Year	Mean	Upper	Lower
Colombia	Cumaribo	Count	2000	0	0	0
Colombia	Cumaribo	Count	2017	0	1	0
Colombia	La Chorrera	Proportion	2000	39.2	49.6	28.7
Colombia	La Chorrera	Proportion	2017	52.1	63.6	40.3
Colombia	La Chorrera	Count	2000	0	0	0
Colombia	La Chorrera	Count	2017	1	1	0
Colombia	Abriaquí	Proportion	2000	35.2	43.4	27.0
Colombia	Abriaquí	Proportion	2017	45.4	58.3	33.0
Colombia	Abriaquí	Count	2000	0	0	0
Colombia	Abriaquí	Count	2017	0	1	0
Colombia	Arauquita	Proportion	2000	39.4	48.4	30.6
Colombia	Arauquita	Proportion	2017	50.1	61.9	37.3
Colombia	Arauquita	Count	2000	0	0	0
Colombia	Arauquita	Count	2017	1	1	0
Colombia	Barranquilla	Proportion	2000	49.9	57.9	42.4
Colombia	Barranquilla	Proportion	2017	66.5	74.1	57.6
Colombia	Barranquilla	Count	2000	0	1	0
Colombia	Barranquilla	Count	2017	1	1	1
Colombia	Altos del Rosario	Proportion	2000	41.0	48.8	32.7
Colombia	Altos del Rosario	Proportion	2017	51.3	62.4	39.8
Colombia	Altos del Rosario	Count	2000	0	0	0
Colombia	Altos del Rosario	Count	2017	1	1	0
Colombia	Aquitania	Proportion	2000	30.2	38.2	23.5
Colombia	Aquitania	Proportion	2017	48.5	62.3	36.3
Colombia	Aquitania	Count	2000	0	0	0
Colombia	Aquitania	Count	2017	0	1	0
Colombia	Anserma	Proportion	2000	33.7	40.5	27.0
Colombia	Anserma	Proportion	2017	52.4	65.0	42.0
Colombia	Anserma	Count	2000	0	0	0
Colombia	Anserma	Count	2017	1	1	0
Colombia	Belén de los Andaquies	Proportion	2000	35.1	44.4	25.8
Colombia	Belén de los Andaquies	Proportion	2017	45.2	55.5	34.4
Colombia	Belén de los Andaquies	Count	2000	0	0	0
Colombia	Belén de los Andaquies	Count	2017	0	1	0
Colombia	Chámeza	Proportion	2000	30.0	37.3	22.9
Colombia	Chámeza	Proportion	2017	43.0	55.8	30.6
Colombia	Chámeza	Count	2000	0	0	0
Colombia	Chámeza	Count	2017	0	1	0
Colombia	Argelia	Proportion	2000	30.7	38.4	23.7
Colombia	Argelia	Proportion	2017	47.6	59.1	36.2
Colombia	Argelia	Count	2000	0	0	0
Colombia	Argelia	Count	2017	0	1	0
Colombia	Agustín Codazzi	Proportion	2000	40.9	50.2	31.6
Colombia	Agustín Codazzi	Proportion	2017	56.9	68.0	45.2
Colombia	Agustín Codazzi	Count	2000	0	1	0
Colombia	Agustín Codazzi	Count	2017	1	1	0
Colombia	Alto Baudó	Proportion	2000	31.8	41.8	24.2
Colombia	Alto Baudó	Proportion	2017	42.5	53.9	31.5
Colombia	Alto Baudó	Count	2000	0	0	0

Table 4: ORS by Unit *(continued)*

Country	Unit	Metric	Year	Mean	Upper	Lower
Colombia	Alto Baudó	Count	2017	0	1	0
Colombia	Buenavista	Proportion	2000	36.4	45.2	28.3
Colombia	Buenavista	Proportion	2017	56.5	67.7	44.4
Colombia	Buenavista	Count	2000	0	0	0
Colombia	Buenavista	Count	2017	1	1	0
Colombia	Albán	Proportion	2000	32.9	38.6	27.1
Colombia	Albán	Proportion	2017	48.7	61.8	37.2
Colombia	Albán	Count	2000	0	0	0
Colombia	Albán	Count	2017	0	1	0
Colombia	Cacahual	Proportion	2000	40.1	51.6	29.9
Colombia	Cacahual	Proportion	2017	52.6	65.0	38.5
Colombia	Cacahual	Count	2000	0	1	0
Colombia	Cacahual	Count	2017	1	1	0
Colombia	El Retorno	Proportion	2000	35.0	43.9	25.8
Colombia	El Retorno	Proportion	2017	45.0	57.4	33.6
Colombia	El Retorno	Count	2000	0	0	0
Colombia	El Retorno	Count	2017	0	1	0
Colombia	Agrado	Proportion	2000	35.0	42.2	27.3
Colombia	Agrado	Proportion	2017	53.4	64.7	40.0
Colombia	Agrado	Count	2000	0	0	0
Colombia	Agrado	Count	2017	1	1	0
Colombia	El Molino	Proportion	2000	37.2	47.8	28.3
Colombia	El Molino	Proportion	2017	52.0	62.9	41.2
Colombia	El Molino	Count	2000	0	0	0
Colombia	El Molino	Count	2017	1	1	0
Colombia	Ariguaní	Proportion	2000	42.9	51.8	35.4
Colombia	Ariguaní	Proportion	2017	57.8	69.2	46.2
Colombia	Ariguaní	Count	2000	0	1	0
Colombia	Ariguaní	Count	2017	1	1	0
Colombia	Barranca de Upía	Proportion	2000	29.7	37.6	22.8
Colombia	Barranca de Upía	Proportion	2017	44.2	57.3	32.5
Colombia	Barranca de Upía	Count	2000	0	0	0
Colombia	Barranca de Upía	Count	2017	0	1	0
Colombia	Aldana	Proportion	2000	34.9	45.1	25.4
Colombia	Aldana	Proportion	2017	54.3	65.8	43.9
Colombia	Aldana	Count	2000	0	0	0
Colombia	Aldana	Count	2017	1	1	0
Colombia	Arboledas	Proportion	2000	36.7	44.5	28.5
Colombia	Arboledas	Proportion	2017	48.1	61.5	37.3
Colombia	Arboledas	Count	2000	0	0	0
Colombia	Arboledas	Count	2017	0	1	0
Colombia	Orito	Proportion	2000	36.2	45.4	27.4
Colombia	Orito	Proportion	2017	44.1	56.6	33.6
Colombia	Orito	Count	2000	0	0	0
Colombia	Orito	Count	2017	0	1	0
Colombia	Buenavista	Proportion	2000	35.7	42.3	29.7
Colombia	Buenavista	Proportion	2017	55.2	67.4	43.6
Colombia	Buenavista	Count	2000	0	0	0
Colombia	Buenavista	Count	2017	1	1	0

Table 4: ORS by Unit *(continued)*

Country	Unit	Metric	Year	Mean	Upper	Lower
Colombia	Balboa	Proportion	2000	33.9	41.3	26.5
Colombia	Balboa	Proportion	2017	53.4	66.1	43.4
Colombia	Balboa	Count	2000	0	0	0
Colombia	Balboa	Count	2017	1	1	0
Colombia	Providencia	Proportion	2000	NA	NA	NA
Colombia	Providencia	Proportion	2017	NA	NA	NA
Colombia	Providencia	Count	2000	NA	NA	NA
Colombia	Providencia	Count	2017	NA	NA	NA
Colombia	Albania	Proportion	2000	31.8	38.3	24.6
Colombia	Albania	Proportion	2017	46.8	60.6	35.4
Colombia	Albania	Count	2000	0	0	0
Colombia	Albania	Count	2017	0	1	0
Colombia	Caimito	Proportion	2000	41.5	51.0	33.0
Colombia	Caimito	Proportion	2017	57.8	68.1	45.8
Colombia	Caimito	Count	2000	0	1	0
Colombia	Caimito	Count	2017	1	1	0
Colombia	Alvarado	Proportion	2000	37.4	43.5	31.3
Colombia	Alvarado	Proportion	2017	52.5	65.7	40.9
Colombia	Alvarado	Count	2000	0	0	0
Colombia	Alvarado	Count	2017	1	1	0
Colombia	Andalucía	Proportion	2000	36.8	43.3	29.2
Colombia	Andalucía	Proportion	2017	54.2	66.5	42.7
Colombia	Andalucía	Count	2000	0	0	0
Colombia	Andalucía	Count	2017	1	1	0
Colombia	Mitú	Proportion	2000	28.2	38.1	19.6
Colombia	Mitú	Proportion	2017	40.1	51.6	29.2
Colombia	Mitú	Count	2000	0	0	0
Colombia	Mitú	Count	2017	0	1	0
Colombia	La Primavera	Proportion	2000	33.1	43.4	23.7
Colombia	La Primavera	Proportion	2017	44.6	55.1	33.1
Colombia	La Primavera	Count	2000	0	0	0
Colombia	La Primavera	Count	2017	0	1	0
Colombia	La Pedrera	Proportion	2000	44.1	54.3	33.6
Colombia	La Pedrera	Proportion	2017	53.3	62.8	40.7
Colombia	La Pedrera	Count	2000	0	1	0
Colombia	La Pedrera	Count	2017	1	1	0
Colombia	Alejandría	Proportion	2000	34.6	41.5	27.2
Colombia	Alejandría	Proportion	2017	46.9	58.4	36.0
Colombia	Alejandría	Count	2000	0	0	0
Colombia	Alejandría	Count	2017	0	1	0
Colombia	Cravo Norte	Proportion	2000	36.4	46.7	26.2
Colombia	Cravo Norte	Proportion	2017	45.3	56.8	32.7
Colombia	Cravo Norte	Count	2000	0	0	0
Colombia	Cravo Norte	Count	2017	0	1	0
Colombia	Campo de la Cruz	Proportion	2000	46.6	55.1	39.1
Colombia	Campo de la Cruz	Proportion	2017	59.6	69.8	48.9
Colombia	Campo de la Cruz	Count	2000	0	1	0
Colombia	Campo de la Cruz	Count	2017	1	1	0
Colombia	Arjona	Proportion	2000	46.0	54.7	37.8

Table 4: ORS by Unit *(continued)*

Country	Unit	Metric	Year	Mean	Upper	Lower
Colombia	Arjona	Proportion	2017	56.7	67.2	45.4
Colombia	Arjona	Count	2000	0	1	0
Colombia	Arjona	Count	2017	1	1	0
Colombia	Arcabuco	Proportion	2000	28.4	35.7	22.0
Colombia	Arcabuco	Proportion	2017	48.7	63.5	37.4
Colombia	Arcabuco	Count	2000	0	0	0
Colombia	Arcabuco	Count	2017	0	1	0
Colombia	Aranzazú	Proportion	2000	34.5	41.3	27.8
Colombia	Aranzazú	Proportion	2017	53.6	64.5	43.5
Colombia	Aranzazú	Count	2000	0	0	0
Colombia	Aranzazú	Count	2017	1	1	0
Colombia	Cartagena del Chairá	Proportion	2000	35.9	45.5	26.7
Colombia	Cartagena del Chairá	Proportion	2017	43.6	54.6	33.4
Colombia	Cartagena del Chairá	Count	2000	0	0	0
Colombia	Cartagena del Chairá	Count	2017	0	1	0
Colombia	Hato Corozal	Proportion	2000	35.8	44.8	27.7
Colombia	Hato Corozal	Proportion	2017	45.7	57.4	34.0
Colombia	Hato Corozal	Count	2000	0	0	0
Colombia	Hato Corozal	Count	2017	0	1	0
Colombia	Balboa	Proportion	2000	31.9	40.2	24.8
Colombia	Balboa	Proportion	2017	50.1	61.4	38.3
Colombia	Balboa	Count	2000	0	0	0
Colombia	Balboa	Count	2017	1	1	0
Colombia	Astrea	Proportion	2000	44.9	53.2	36.9
Colombia	Astrea	Proportion	2017	58.3	70.4	46.2
Colombia	Astrea	Count	2000	0	1	0
Colombia	Astrea	Count	2017	1	1	0
Colombia	Bagadó	Proportion	2000	29.0	37.2	21.7
Colombia	Bagadó	Proportion	2017	42.0	55.5	30.5
Colombia	Bagadó	Count	2000	0	0	0
Colombia	Bagadó	Count	2017	0	1	0
Colombia	Canalete	Proportion	2000	37.4	46.2	30.0
Colombia	Canalete	Proportion	2017	58.0	69.0	46.5
Colombia	Canalete	Count	2000	0	0	0
Colombia	Canalete	Count	2017	1	1	0
Colombia	Anapoima	Proportion	2000	37.6	43.5	31.8
Colombia	Anapoima	Proportion	2017	52.6	64.8	41.1
Colombia	Anapoima	Count	2000	0	0	0
Colombia	Anapoima	Count	2017	1	1	0
Colombia	La Guadalupe	Proportion	2000	45.6	56.6	34.9
Colombia	La Guadalupe	Proportion	2017	54.4	66.6	41.0
Colombia	La Guadalupe	Count	2000	0	1	0
Colombia	La Guadalupe	Count	2017	1	1	0
Colombia	Miraflores	Proportion	2000	35.9	46.2	25.7
Colombia	Miraflores	Proportion	2017	46.0	57.9	33.1
Colombia	Miraflores	Count	2000	0	0	0
Colombia	Miraflores	Count	2017	0	1	0
Colombia	Aipe	Proportion	2000	35.8	43.2	28.1
Colombia	Aipe	Proportion	2017	53.4	64.1	41.9

Table 4: ORS by Unit *(continued)*

Country	Unit	Metric	Year	Mean	Upper	Lower
Colombia	Aipe	Count	2000	0	0	0
Colombia	Aipe	Count	2017	1	1	0
Colombia	Fonseca	Proportion	2000	36.2	46.2	27.4
Colombia	Fonseca	Proportion	2017	51.0	62.6	40.6
Colombia	Fonseca	Count	2000	0	0	0
Colombia	Fonseca	Count	2017	1	1	0
Colombia	Cerro de San Antonio	Proportion	2000	47.1	55.6	39.6
Colombia	Cerro de San Antonio	Proportion	2017	59.7	69.6	49.0
Colombia	Cerro de San Antonio	Count	2000	0	1	0
Colombia	Cerro de San Antonio	Count	2017	1	1	0
Colombia	Cabuyaro	Proportion	2000	31.1	39.5	23.8
Colombia	Cabuyaro	Proportion	2017	45.6	58.7	34.3
Colombia	Cabuyaro	Count	2000	0	0	0
Colombia	Cabuyaro	Count	2017	0	1	0
Colombia	Ancuyá	Proportion	2000	35.9	45.2	28.0
Colombia	Ancuyá	Proportion	2017	55.6	67.1	44.3
Colombia	Ancuyá	Count	2000	0	0	0
Colombia	Ancuyá	Count	2017	1	1	0
Colombia	Bochalema	Proportion	2000	37.2	45.1	28.7
Colombia	Bochalema	Proportion	2017	49.4	62.3	37.0
Colombia	Bochalema	Count	2000	0	0	0
Colombia	Bochalema	Count	2017	0	1	0
Colombia	Puerto Asís	Proportion	2000	36.2	46.0	27.2
Colombia	Puerto Asís	Proportion	2017	44.4	56.2	33.6
Colombia	Puerto Asís	Count	2000	0	0	0
Colombia	Puerto Asís	Count	2017	0	1	0
Colombia	Calarcá	Proportion	2000	34.3	41.6	27.6
Colombia	Calarcá	Proportion	2017	48.2	61.5	36.7
Colombia	Calarcá	Count	2000	0	0	0
Colombia	Calarcá	Count	2017	0	1	0
Colombia	Belén de Umbría	Proportion	2000	32.9	40.2	26.3
Colombia	Belén de Umbría	Proportion	2017	50.9	63.6	40.4
Colombia	Belén de Umbría	Count	2000	0	0	0
Colombia	Belén de Umbría	Count	2017	1	1	0
Colombia	San Andrés	Proportion	2000	NA	NA	NA
Colombia	San Andrés	Proportion	2017	NA	NA	NA
Colombia	San Andrés	Count	2000	NA	NA	NA
Colombia	San Andrés	Count	2017	NA	NA	NA
Colombia	Aratoca	Proportion	2000	35.5	42.1	28.4
Colombia	Aratoca	Proportion	2017	51.5	65.1	39.3
Colombia	Aratoca	Count	2000	0	0	0
Colombia	Aratoca	Count	2017	1	1	0
Colombia	Chalán	Proportion	2000	43.3	51.9	35.3
Colombia	Chalán	Proportion	2017	56.8	67.8	45.7
Colombia	Chalán	Count	2000	0	1	0
Colombia	Chalán	Count	2017	1	1	0
Colombia	Ambalema	Proportion	2000	38.1	44.7	31.4
Colombia	Ambalema	Proportion	2017	52.4	65.9	40.4
Colombia	Ambalema	Count	2000	0	0	0

Table 4: ORS by Unit *(continued)*

Country	Unit	Metric	Year	Mean	Upper	Lower
Colombia	Ambalema	Count	2017	1	1	0
Colombia	Ansermanuevo	Proportion	2000	33.7	40.8	26.8
Colombia	Ansermanuevo	Proportion	2017	49.9	63.2	39.1
Colombia	Ansermanuevo	Count	2000	0	0	0
Colombia	Ansermanuevo	Count	2017	0	1	0
Colombia	Pacoa	Proportion	2000	30.7	40.8	22.2
Colombia	Pacoa	Proportion	2017	43.0	54.6	31.1
Colombia	Pacoa	Count	2000	0	0	0
Colombia	Pacoa	Count	2017	0	1	0
Colombia	Puerto Carreño	Proportion	2000	39.2	50.5	28.7
Colombia	Puerto Carreño	Proportion	2017	53.4	63.3	42.8
Colombia	Puerto Carreño	Count	2000	0	1	0
Colombia	Puerto Carreño	Count	2017	1	1	0
Colombia	Leticia	Proportion	2000	38.7	49.0	28.7
Colombia	Leticia	Proportion	2017	50.9	60.6	38.1
Colombia	Leticia	Count	2000	0	0	0
Colombia	Leticia	Count	2017	1	1	0
Colombia	Amagá	Proportion	2000	37.3	43.9	29.7
Colombia	Amagá	Proportion	2017	55.9	67.8	44.4
Colombia	Amagá	Count	2000	0	0	0
Colombia	Amagá	Count	2017	1	1	0
Colombia	Fortul	Proportion	2000	36.9	46.1	28.8
Colombia	Fortul	Proportion	2017	45.3	57.6	33.8
Colombia	Fortul	Count	2000	0	0	0
Colombia	Fortul	Count	2017	0	1	0
Colombia	Candelaria	Proportion	2000	47.0	55.7	39.5
Colombia	Candelaria	Proportion	2017	59.0	69.2	48.2
Colombia	Candelaria	Count	2000	0	1	0
Colombia	Candelaria	Count	2017	1	1	0
Colombia	Barranco de Loba	Proportion	2000	42.2	50.2	33.6
Colombia	Barranco de Loba	Proportion	2017	52.7	63.3	40.9
Colombia	Barranco de Loba	Count	2000	0	1	0
Colombia	Barranco de Loba	Count	2017	1	1	0
Colombia	Belén	Proportion	2000	29.6	36.8	23.2
Colombia	Belén	Proportion	2017	54.2	66.7	42.1
Colombia	Belén	Count	2000	0	0	0
Colombia	Belén	Count	2017	1	1	0
Colombia	Belalcázar	Proportion	2000	34.0	40.4	27.2
Colombia	Belalcázar	Proportion	2017	56.3	68.7	45.8
Colombia	Belalcázar	Count	2000	0	0	0
Colombia	Belalcázar	Count	2017	1	1	0
Colombia	Curillo	Proportion	2000	37.9	47.5	28.1
Colombia	Curillo	Proportion	2017	46.7	57.3	36.3
Colombia	Curillo	Count	2000	0	0	0
Colombia	Curillo	Count	2017	0	1	0
Colombia	La Salina	Proportion	2000	31.5	39.7	24.8
Colombia	La Salina	Proportion	2017	46.4	59.9	33.8
Colombia	La Salina	Count	2000	0	0	0
Colombia	La Salina	Count	2017	0	1	0

Table 4: ORS by Unit *(continued)*

Country	Unit	Metric	Year	Mean	Upper	Lower
Colombia	Bolívar	Proportion	2000	33.6	41.9	26.4
Colombia	Bolívar	Proportion	2017	52.0	62.6	40.3
Colombia	Bolívar	Count	2000	0	0	0
Colombia	Bolívar	Count	2017	1	1	0
Colombia	Becerril	Proportion	2000	40.6	50.5	30.5
Colombia	Becerril	Proportion	2017	55.8	66.9	44.1
Colombia	Becerril	Count	2000	0	1	0
Colombia	Becerril	Count	2017	1	1	0
Colombia	Bahía Solano	Proportion	2000	30.6	39.4	22.6
Colombia	Bahía Solano	Proportion	2017	38.5	49.6	27.6
Colombia	Bahía Solano	Count	2000	0	0	0
Colombia	Bahía Solano	Count	2017	0	0	0
Colombia	Cereté	Proportion	2000	36.0	45.3	28.8
Colombia	Cereté	Proportion	2017	54.7	65.3	43.6
Colombia	Cereté	Count	2000	0	0	0
Colombia	Cereté	Count	2017	1	1	0
Colombia	Anolaima	Proportion	2000	33.0	39.0	27.5
Colombia	Anolaima	Proportion	2017	48.4	61.2	36.6
Colombia	Anolaima	Count	2000	0	0	0
Colombia	Anolaima	Count	2017	0	1	0
Colombia	Pana Pana	Proportion	2000	40.6	51.9	31.1
Colombia	Pana Pana	Proportion	2017	50.5	62.5	38.0
Colombia	Pana Pana	Count	2000	0	1	0
Colombia	Pana Pana	Count	2017	1	1	0
Colombia	San José del Guaviare	Proportion	2000	33.8	43.1	25.3
Colombia	San José del Guaviare	Proportion	2017	44.1	55.9	32.5
Colombia	San José del Guaviare	Count	2000	0	0	0
Colombia	San José del Guaviare	Count	2017	0	1	0
Colombia	Algeciras	Proportion	2000	33.3	40.8	24.6
Colombia	Algeciras	Proportion	2017	50.3	62.1	37.1
Colombia	Algeciras	Count	2000	0	0	0
Colombia	Algeciras	Count	2017	1	1	0
Colombia	Maicao	Proportion	2000	40.2	50.5	31.0
Colombia	Maicao	Proportion	2017	60.7	72.4	51.2
Colombia	Maicao	Count	2000	0	1	0
Colombia	Maicao	Count	2017	1	1	1
Colombia	Chivolo	Proportion	2000	46.7	55.3	39.6
Colombia	Chivolo	Proportion	2017	59.0	69.3	47.7
Colombia	Chivolo	Count	2000	0	1	0
Colombia	Chivolo	Count	2017	1	1	0
Colombia	Castilla la Nueva	Proportion	2000	31.1	39.1	23.5
Colombia	Castilla la Nueva	Proportion	2017	46.0	58.0	34.1
Colombia	Castilla la Nueva	Count	2000	0	0	0
Colombia	Castilla la Nueva	Count	2017	0	1	0
Colombia	Arboleda	Proportion	2000	36.1	45.3	27.8
Colombia	Arboleda	Proportion	2017	54.1	65.9	42.2
Colombia	Arboleda	Count	2000	0	0	0
Colombia	Arboleda	Count	2017	1	1	0
Colombia	Bucarasica	Proportion	2000	41.6	49.5	33.3

Table 4: ORS by Unit *(continued)*

Country	Unit	Metric	Year	Mean	Upper	Lower
Colombia	Bucarasica	Proportion	2017	49.8	62.3	38.4
Colombia	Bucarasica	Count	2000	0	0	0
Colombia	Bucarasica	Count	2017	0	1	0
Colombia	Puerto Caicedo	Proportion	2000	36.4	46.1	27.3
Colombia	Puerto Caicedo	Proportion	2017	43.6	55.9	33.0
Colombia	Puerto Caicedo	Count	2000	0	0	0
Colombia	Puerto Caicedo	Count	2017	0	1	0
Colombia	Circasia	Proportion	2000	33.3	40.4	26.7
Colombia	Circasia	Proportion	2017	52.7	65.1	41.4
Colombia	Circasia	Count	2000	0	0	0
Colombia	Circasia	Count	2017	1	1	0
Colombia	Dosquebradas	Proportion	2000	34.8	41.8	27.8
Colombia	Dosquebradas	Proportion	2017	84.0	88.1	80.1
Colombia	Dosquebradas	Count	2000	0	0	0
Colombia	Dosquebradas	Count	2017	1	1	1
Colombia	Barbosa	Proportion	2000	35.1	43.2	27.2
Colombia	Barbosa	Proportion	2017	56.4	68.6	44.6
Colombia	Barbosa	Count	2000	0	0	0
Colombia	Barbosa	Count	2017	1	1	0
Colombia	Colosó	Proportion	2000	45.7	54.2	37.4
Colombia	Colosó	Proportion	2017	58.2	69.3	46.4
Colombia	Colosó	Count	2000	0	1	0
Colombia	Colosó	Count	2017	1	1	0
Colombia	Anzoátegui	Proportion	2000	31.3	37.8	24.6
Colombia	Anzoátegui	Proportion	2017	46.3	59.1	35.5
Colombia	Anzoátegui	Count	2000	0	0	0
Colombia	Anzoátegui	Count	2017	0	1	0
Colombia	Argelia	Proportion	2000	32.0	39.4	24.8
Colombia	Argelia	Proportion	2017	47.0	60.2	36.1
Colombia	Argelia	Count	2000	0	0	0
Colombia	Argelia	Count	2017	0	1	0
Colombia	Papunahua	Proportion	2000	28.1	37.4	19.4
Colombia	Papunahua	Proportion	2017	39.5	51.2	27.0
Colombia	Papunahua	Count	2000	0	0	0
Colombia	Papunahua	Count	2017	0	1	0
Colombia	San Jose de Ocune	Proportion	2000	32.9	42.9	24.4
Colombia	San Jose de Ocune	Proportion	2017	46.1	56.6	35.6
Colombia	San Jose de Ocune	Count	2000	0	0	0
Colombia	San Jose de Ocune	Count	2017	0	1	0
Colombia	Mirití-Paraná	Proportion	2000	46.2	56.7	35.4
Colombia	Mirití-Paraná	Proportion	2017	55.0	66.3	42.3
Colombia	Mirití-Paraná	Count	2000	0	1	0
Colombia	Mirití-Paraná	Count	2017	1	1	0
Colombia	Amalfi	Proportion	2000	34.8	41.3	27.5
Colombia	Amalfi	Proportion	2017	48.1	58.8	37.3
Colombia	Amalfi	Count	2000	0	0	0
Colombia	Amalfi	Count	2017	0	1	0
Colombia	Puerto Rondón	Proportion	2000	38.3	47.6	29.7
Colombia	Puerto Rondón	Proportion	2017	48.4	59.2	35.6

Table 4: ORS by Unit *(continued)*

Country	Unit	Metric	Year	Mean	Upper	Lower
Colombia	Puerto Rondón	Count	2000	0	0	0
Colombia	Puerto Rondón	Count	2017	0	1	0
Colombia	Galapa	Proportion	2000	46.0	54.9	38.5
Colombia	Galapa	Proportion	2017	64.9	72.7	55.4
Colombia	Galapa	Count	2000	0	1	0
Colombia	Galapa	Count	2017	1	1	1
Colombia	Calamar	Proportion	2000	47.9	57.0	40.4
Colombia	Calamar	Proportion	2017	59.0	68.8	48.3
Colombia	Calamar	Count	2000	0	1	0
Colombia	Calamar	Count	2017	1	1	0
Colombia	Berbeo	Proportion	2000	30.4	38.1	23.7
Colombia	Berbeo	Proportion	2017	50.5	63.6	38.3
Colombia	Berbeo	Count	2000	0	0	0
Colombia	Berbeo	Count	2017	1	1	0
Colombia	Chinchiná	Proportion	2000	33.2	39.8	26.7
Colombia	Chinchiná	Proportion	2017	52.3	65.0	41.9
Colombia	Chinchiná	Count	2000	0	0	0
Colombia	Chinchiná	Count	2017	1	1	0
Colombia	El Doncello	Proportion	2000	35.7	44.8	28.0
Colombia	El Doncello	Proportion	2017	47.7	58.9	36.6
Colombia	El Doncello	Count	2000	0	0	0
Colombia	El Doncello	Count	2017	0	1	0
Colombia	Maní	Proportion	2000	32.1	41.8	24.1
Colombia	Maní	Proportion	2017	45.0	57.0	33.2
Colombia	Maní	Count	2000	0	0	0
Colombia	Maní	Count	2017	0	1	0
Colombia	Buenos Aires	Proportion	2000	31.8	38.9	25.1
Colombia	Buenos Aires	Proportion	2017	50.9	61.8	39.1
Colombia	Buenos Aires	Count	2000	0	0	0
Colombia	Buenos Aires	Count	2017	1	1	0
Colombia	Bosconia	Proportion	2000	44.1	52.8	35.1
Colombia	Bosconia	Proportion	2017	59.5	69.8	48.1
Colombia	Bosconia	Count	2000	0	1	0
Colombia	Bosconia	Count	2017	1	1	0
Colombia	Bajo Baudó	Proportion	2000	31.4	40.0	23.2
Colombia	Bajo Baudó	Proportion	2017	42.9	55.6	32.6
Colombia	Bajo Baudó	Count	2000	0	0	0
Colombia	Bajo Baudó	Count	2017	0	1	0
Colombia	Chimá	Proportion	2000	38.6	46.9	31.2
Colombia	Chimá	Proportion	2017	60.3	70.9	49.6
Colombia	Chimá	Count	2000	0	0	0
Colombia	Chimá	Count	2017	1	1	0
Colombia	Apulo	Proportion	2000	38.5	44.7	32.4
Colombia	Apulo	Proportion	2017	52.6	65.1	41.7
Colombia	Apulo	Count	2000	0	0	0
Colombia	Apulo	Count	2017	1	1	0
Colombia	Puerto Colombia	Proportion	2000	43.3	54.3	32.0
Colombia	Puerto Colombia	Proportion	2017	54.8	66.3	42.0
Colombia	Puerto Colombia	Count	2000	0	1	0

Table 4: ORS by Unit *(continued)*

Country	Unit	Metric	Year	Mean	Upper	Lower
Colombia	Puerto Colombia	Count	2017	1	1	0
Colombia	Altamira	Proportion	2000	35.6	43.5	27.9
Colombia	Altamira	Proportion	2017	53.5	64.0	40.9
Colombia	Altamira	Count	2000	0	0	0
Colombia	Altamira	Count	2017	1	1	0
Colombia	Manaure	Proportion	2000	38.5	48.0	29.5
Colombia	Manaure	Proportion	2017	55.8	68.8	44.0
Colombia	Manaure	Count	2000	0	0	0
Colombia	Manaure	Count	2017	1	1	0
Colombia	Ciénaga	Proportion	2000	47.4	55.3	39.6
Colombia	Ciénaga	Proportion	2017	67.8	75.7	58.1
Colombia	Ciénaga	Count	2000	0	1	0
Colombia	Ciénaga	Count	2017	1	1	1
Colombia	Cumaral	Proportion	2000	30.0	37.7	23.4
Colombia	Cumaral	Proportion	2017	44.7	57.4	33.2
Colombia	Cumaral	Count	2000	0	0	0
Colombia	Cumaral	Count	2017	0	1	0
Colombia	Barbacoas	Proportion	2000	36.7	45.6	28.2
Colombia	Barbacoas	Proportion	2017	43.7	54.8	32.9
Colombia	Barbacoas	Count	2000	0	0	0
Colombia	Barbacoas	Count	2017	0	1	0
Colombia	Cáchira	Proportion	2000	37.8	45.1	29.1
Colombia	Cáchira	Proportion	2017	48.8	61.0	37.9
Colombia	Cáchira	Count	2000	0	0	0
Colombia	Cáchira	Count	2017	0	1	0
Colombia	Puerto Guzmán	Proportion	2000	35.5	45.2	27.1
Colombia	Puerto Guzmán	Proportion	2017	42.9	54.7	32.5
Colombia	Puerto Guzmán	Count	2000	0	0	0
Colombia	Puerto Guzmán	Count	2017	0	1	0
Colombia	Córdoba	Proportion	2000	34.1	41.3	28.0
Colombia	Córdoba	Proportion	2017	51.8	64.4	39.9
Colombia	Córdoba	Count	2000	0	0	0
Colombia	Córdoba	Count	2017	1	1	0
Colombia	Guática	Proportion	2000	33.7	40.7	27.2
Colombia	Guática	Proportion	2017	49.2	62.1	38.7
Colombia	Guática	Count	2000	0	0	0
Colombia	Guática	Count	2017	0	1	0
Colombia	Barichara	Proportion	2000	34.3	41.9	27.7
Colombia	Barichara	Proportion	2017	52.3	65.0	40.1
Colombia	Barichara	Count	2000	0	0	0
Colombia	Barichara	Count	2017	1	1	0
Colombia	Corozal	Proportion	2000	43.2	52.0	34.6
Colombia	Corozal	Proportion	2017	59.2	69.3	47.3
Colombia	Corozal	Count	2000	0	1	0
Colombia	Corozal	Count	2017	1	1	0
Colombia	Armero	Proportion	2000	37.8	44.4	31.4
Colombia	Armero	Proportion	2017	52.1	64.9	40.6
Colombia	Armero	Count	2000	0	0	0
Colombia	Armero	Count	2017	1	1	0

Table 4: ORS by Unit *(continued)*

Country	Unit	Metric	Year	Mean	Upper	Lower
Colombia	Bolívar	Proportion	2000	34.5	41.6	27.4
Colombia	Bolívar	Proportion	2017	50.3	63.5	40.0
Colombia	Bolívar	Count	2000	0	0	0
Colombia	Bolívar	Count	2017	1	1	0
Colombia	Taraira	Proportion	2000	38.0	50.2	26.3
Colombia	Taraira	Proportion	2017	50.2	61.1	36.6
Colombia	Taraira	Count	2000	0	1	0
Colombia	Taraira	Count	2017	1	1	0
Colombia	Santa Rita	Proportion	2000	34.9	46.0	25.1
Colombia	Santa Rita	Proportion	2017	49.7	60.0	37.9
Colombia	Santa Rita	Count	2000	0	0	0
Colombia	Santa Rita	Count	2017	0	1	0
Colombia	Puerto Nariño	Proportion	2000	39.9	49.8	29.2
Colombia	Puerto Nariño	Proportion	2017	51.8	61.7	41.2
Colombia	Puerto Nariño	Count	2000	0	0	0
Colombia	Puerto Nariño	Count	2017	1	1	0
Colombia	Andes	Proportion	2000	35.6	43.8	27.9
Colombia	Andes	Proportion	2017	50.3	63.0	39.1
Colombia	Andes	Count	2000	0	0	0
Colombia	Andes	Count	2017	1	1	0
Colombia	Saravena	Proportion	2000	39.0	48.7	30.3
Colombia	Saravena	Proportion	2017	47.6	59.7	34.8
Colombia	Saravena	Count	2000	0	0	0
Colombia	Saravena	Count	2017	0	1	0
Colombia	Juan de Acosta	Proportion	2000	44.7	53.3	36.9
Colombia	Juan de Acosta	Proportion	2017	55.1	65.0	44.2
Colombia	Juan de Acosta	Count	2000	0	1	0
Colombia	Juan de Acosta	Count	2017	1	1	0
Colombia	Cantagallo	Proportion	2000	39.0	46.1	31.4
Colombia	Cantagallo	Proportion	2017	49.0	60.7	37.3
Colombia	Cantagallo	Count	2000	0	0	0
Colombia	Cantagallo	Count	2017	0	1	0
Colombia	Beteitiva	Proportion	2000	29.3	36.8	22.8
Colombia	Beteitiva	Proportion	2017	52.0	64.9	39.8
Colombia	Beteitiva	Count	2000	0	0	0
Colombia	Beteitiva	Count	2017	1	1	0
Colombia	Filadelfia	Proportion	2000	34.4	41.4	27.8
Colombia	Filadelfia	Proportion	2017	54.6	66.6	44.0
Colombia	Filadelfia	Count	2000	0	0	0
Colombia	Filadelfia	Count	2017	1	1	0
Colombia	El Paujíl	Proportion	2000	35.9	45.0	27.5
Colombia	El Paujíl	Proportion	2017	46.5	57.4	35.5
Colombia	El Paujíl	Count	2000	0	0	0
Colombia	El Paujíl	Count	2017	0	1	0
Colombia	Monterrey	Proportion	2000	31.3	40.1	23.9
Colombia	Monterrey	Proportion	2017	45.4	57.1	33.4
Colombia	Monterrey	Count	2000	0	0	0
Colombia	Monterrey	Count	2017	0	1	0
Colombia	Cajibío	Proportion	2000	29.7	36.8	23.0

Table 4: ORS by Unit *(continued)*

Country	Unit	Metric	Year	Mean	Upper	Lower
Colombia	Cajibío	Proportion	2017	53.0	64.3	42.0
Colombia	Cajibío	Count	2000	0	0	0
Colombia	Cajibío	Count	2017	1	1	0
Colombia	Chimichagua	Proportion	2000	43.1	50.7	33.8
Colombia	Chimichagua	Proportion	2017	56.4	67.6	45.1
Colombia	Chimichagua	Count	2000	0	1	0
Colombia	Chimichagua	Count	2017	1	1	0
Colombia	Bojayá	Proportion	2000	31.1	40.2	23.5
Colombia	Bojayá	Proportion	2017	40.2	51.2	30.1
Colombia	Bojayá	Count	2000	0	0	0
Colombia	Bojayá	Count	2017	0	1	0
Colombia	Chinú	Proportion	2000	41.2	50.1	33.3
Colombia	Chinú	Proportion	2017	61.7	71.8	49.8
Colombia	Chinú	Count	2000	0	1	0
Colombia	Chinú	Count	2017	1	1	0
Colombia	Arbeláez	Proportion	2000	34.4	41.1	28.2
Colombia	Arbeláez	Proportion	2017	50.5	61.8	39.0
Colombia	Arbeláez	Count	2000	0	0	0
Colombia	Arbeláez	Count	2017	1	1	0
Colombia	Puerto Inírida	Proportion	2000	40.1	50.9	29.8
Colombia	Puerto Inírida	Proportion	2017	53.3	63.9	40.5
Colombia	Puerto Inírida	Count	2000	0	1	0
Colombia	Puerto Inírida	Count	2017	1	1	0
Colombia	Baraya	Proportion	2000	37.4	46.7	29.0
Colombia	Baraya	Proportion	2017	54.3	65.1	41.7
Colombia	Baraya	Count	2000	0	0	0
Colombia	Baraya	Count	2017	1	1	0
Colombia	Riohacha	Proportion	2000	39.2	47.9	30.0
Colombia	Riohacha	Proportion	2017	56.7	68.4	45.5
Colombia	Riohacha	Count	2000	0	0	0
Colombia	Riohacha	Count	2017	1	1	0
Colombia	El Banco	Proportion	2000	42.6	51.1	33.8
Colombia	El Banco	Proportion	2017	55.7	66.9	44.4
Colombia	El Banco	Count	2000	0	1	0
Colombia	El Banco	Count	2017	1	1	0
Colombia	El Calvario	Proportion	2000	28.8	35.5	22.6
Colombia	El Calvario	Proportion	2017	43.2	57.0	31.5
Colombia	El Calvario	Count	2000	0	0	0
Colombia	El Calvario	Count	2017	0	1	0
Colombia	Belén	Proportion	2000	34.2	43.4	25.6
Colombia	Belén	Proportion	2017	53.8	65.0	42.9
Colombia	Belén	Count	2000	0	0	0
Colombia	Belén	Count	2017	1	1	0
Colombia	Cácota	Proportion	2000	34.3	41.6	27.5
Colombia	Cácota	Proportion	2017	46.6	60.1	35.6
Colombia	Cácota	Count	2000	0	0	0
Colombia	Cácota	Count	2017	0	1	0
Colombia	Puerto Leguízamo	Proportion	2000	36.1	45.6	26.9
Colombia	Puerto Leguízamo	Proportion	2017	45.2	57.1	34.6

Table 4: ORS by Unit *(continued)*

Country	Unit	Metric	Year	Mean	Upper	Lower
Colombia	Puerto Leguízamo	Count	2000	0	0	0
Colombia	Puerto Leguízamo	Count	2017	0	1	0
Colombia	Filandia	Proportion	2000	34.2	41.1	27.7
Colombia	Filandia	Proportion	2017	54.5	67.3	43.8
Colombia	Filandia	Count	2000	0	0	0
Colombia	Filandia	Count	2017	1	1	0
Colombia	La Celia	Proportion	2000	31.9	39.4	24.5
Colombia	La Celia	Proportion	2017	46.3	59.8	36.2
Colombia	La Celia	Count	2000	0	0	0
Colombia	La Celia	Count	2017	0	1	0
Colombia	Barrancabermeja	Proportion	2000	36.1	43.9	27.7
Colombia	Barrancabermeja	Proportion	2017	55.2	67.1	45.1
Colombia	Barrancabermeja	Count	2000	0	0	0
Colombia	Barrancabermeja	Count	2017	1	1	0
Colombia	Galeras	Proportion	2000	45.1	54.0	36.8
Colombia	Galeras	Proportion	2017	65.7	75.1	55.1
Colombia	Galeras	Count	2000	0	1	0
Colombia	Galeras	Count	2017	1	1	1
Colombia	Ataco	Proportion	2000	35.1	42.6	27.9
Colombia	Ataco	Proportion	2017	50.9	60.9	39.2
Colombia	Ataco	Count	2000	0	0	0
Colombia	Ataco	Count	2017	1	1	0
Colombia	Buenaventura	Proportion	2000	31.8	40.4	24.4
Colombia	Buenaventura	Proportion	2017	44.7	55.3	35.1
Colombia	Buenaventura	Count	2000	0	0	0
Colombia	Buenaventura	Count	2017	0	1	0
Colombia	Yavaraté	Proportion	2000	31.8	43.1	22.8
Colombia	Yavaraté	Proportion	2017	41.8	54.0	29.8
Colombia	Yavaraté	Count	2000	0	0	0
Colombia	Yavaraté	Count	2017	0	1	0
Colombia	Santa Rosalía	Proportion	2000	31.1	41.9	22.2
Colombia	Santa Rosalía	Proportion	2017	43.1	54.5	31.9
Colombia	Santa Rosalía	Count	2000	0	0	0
Colombia	Santa Rosalía	Count	2017	0	1	0
Colombia	Puerto Santander	Proportion	2000	43.5	54.1	32.3
Colombia	Puerto Santander	Proportion	2017	53.7	66.1	41.9
Colombia	Puerto Santander	Count	2000	0	1	0
Colombia	Puerto Santander	Count	2017	1	1	0
Colombia	Angelópolis	Proportion	2000	36.4	42.7	28.7
Colombia	Angelópolis	Proportion	2017	52.7	65.4	41.1
Colombia	Angelópolis	Count	2000	0	0	0
Colombia	Angelópolis	Count	2017	1	1	0
Colombia	Tame	Proportion	2000	36.4	45.4	28.6
Colombia	Tame	Proportion	2017	45.1	57.6	33.2
Colombia	Tame	Count	2000	0	0	0
Colombia	Tame	Count	2017	0	1	0
Colombia	Luruaco	Proportion	2000	45.2	53.9	37.1
Colombia	Luruaco	Proportion	2017	56.1	66.1	45.0
Colombia	Luruaco	Count	2000	0	1	0

Table 4: ORS by Unit *(continued)*

Country	Unit	Metric	Year	Mean	Upper	Lower
Colombia	Luruaco	Count	2017	1	1	0
Colombia	Cartagena de Indias	Proportion	2000	48.0	57.1	39.9
Colombia	Cartagena de Indias	Proportion	2017	56.2	67.3	44.5
Colombia	Cartagena de Indias	Count	2000	0	1	0
Colombia	Cartagena de Indias	Count	2017	1	1	0
Colombia	Boavita	Proportion	2000	29.8	37.4	23.7
Colombia	Boavita	Proportion	2017	47.9	61.5	35.5
Colombia	Boavita	Count	2000	0	0	0
Colombia	Boavita	Count	2017	0	1	0
Colombia	La Dorada	Proportion	2000	36.4	43.4	29.0
Colombia	La Dorada	Proportion	2017	55.1	66.7	43.7
Colombia	La Dorada	Count	2000	0	0	0
Colombia	La Dorada	Count	2017	1	1	0
Colombia	Florencia	Proportion	2000	33.3	41.5	24.7
Colombia	Florencia	Proportion	2017	45.5	56.6	34.5
Colombia	Florencia	Count	2000	0	0	0
Colombia	Florencia	Count	2017	0	1	0
Colombia	Nunchía	Proportion	2000	35.6	45.4	27.5
Colombia	Nunchía	Proportion	2017	46.7	58.8	34.7
Colombia	Nunchía	Count	2000	0	0	0
Colombia	Nunchía	Count	2017	0	1	0
Colombia	Caldonó	Proportion	2000	29.5	36.3	22.8
Colombia	Caldonó	Proportion	2017	52.1	63.6	40.7
Colombia	Caldonó	Count	2000	0	0	0
Colombia	Caldonó	Count	2017	1	1	0
Colombia	Chiriguaná	Proportion	2000	41.3	49.5	31.7
Colombia	Chiriguaná	Proportion	2017	55.7	66.8	43.6
Colombia	Chiriguaná	Count	2000	0	0	0
Colombia	Chiriguaná	Count	2017	1	1	0
Colombia	Condoto	Proportion	2000	30.1	39.4	22.0
Colombia	Condoto	Proportion	2017	42.2	56.3	31.3
Colombia	Condoto	Count	2000	0	0	0
Colombia	Condoto	Count	2017	0	1	0
Colombia	Ciénaga de Oro	Proportion	2000	38.6	47.7	30.9
Colombia	Ciénaga de Oro	Proportion	2017	59.0	69.8	47.8
Colombia	Ciénaga de Oro	Count	2000	0	0	0
Colombia	Ciénaga de Oro	Count	2017	1	1	0
Colombia	Beltrán	Proportion	2000	37.6	43.9	30.8
Colombia	Beltrán	Proportion	2017	52.4	65.7	40.2
Colombia	Beltrán	Count	2000	0	0	0
Colombia	Beltrán	Count	2017	1	1	0
Colombia	San Felipe	Proportion	2000	43.5	54.2	31.7
Colombia	San Felipe	Proportion	2017	51.8	63.8	37.8
Colombia	San Felipe	Count	2000	0	1	0
Colombia	San Felipe	Count	2017	1	1	0
Colombia	Campoalegre	Proportion	2000	37.2	46.1	28.5
Colombia	Campoalegre	Proportion	2017	54.1	66.3	40.3
Colombia	Campoalegre	Count	2000	0	0	0
Colombia	Campoalegre	Count	2017	1	1	0

Table 4: ORS by Unit *(continued)*

Country	Unit	Metric	Year	Mean	Upper	Lower
Colombia	San Juan del Cesar	Proportion	2000	37.3	46.7	27.8
Colombia	San Juan del Cesar	Proportion	2017	52.2	63.1	41.5
Colombia	San Juan del Cesar	Count	2000	0	0	0
Colombia	San Juan del Cesar	Count	2017	1	1	0
Colombia	El Piñón	Proportion	2000	45.8	54.7	39.3
Colombia	El Piñón	Proportion	2017	61.3	71.2	51.0
Colombia	El Piñón	Count	2000	0	1	0
Colombia	El Piñón	Count	2017	1	1	1
Colombia	El Castillo	Proportion	2000	30.9	39.5	23.9
Colombia	El Castillo	Proportion	2017	47.3	58.4	34.6
Colombia	El Castillo	Count	2000	0	0	0
Colombia	El Castillo	Count	2017	0	1	0
Colombia	Buesaco	Proportion	2000	36.3	45.2	28.5
Colombia	Buesaco	Proportion	2017	53.4	65.0	41.3
Colombia	Buesaco	Count	2000	0	0	0
Colombia	Buesaco	Count	2017	1	1	0
Colombia	Chinácota	Proportion	2000	37.7	45.8	29.3
Colombia	Chinácota	Proportion	2017	53.2	66.1	41.3
Colombia	Chinácota	Count	2000	0	0	0
Colombia	Chinácota	Count	2017	1	1	0
Colombia	San Francisco	Proportion	2000	31.8	40.9	22.9
Colombia	San Francisco	Proportion	2017	49.3	61.3	38.8
Colombia	San Francisco	Count	2000	0	0	0
Colombia	San Francisco	Count	2017	0	1	0
Colombia	Génova	Proportion	2000	33.1	40.0	26.6
Colombia	Génova	Proportion	2017	51.5	63.2	39.5
Colombia	Génova	Count	2000	0	0	0
Colombia	Génova	Count	2017	1	1	0
Colombia	La Virginia	Proportion	2000	40.2	47.3	32.9
Colombia	La Virginia	Proportion	2017	65.8	76.7	55.6
Colombia	La Virginia	Count	2000	0	0	0
Colombia	La Virginia	Count	2017	1	1	1
Colombia	Betulia	Proportion	2000	34.9	42.7	28.3
Colombia	Betulia	Proportion	2017	50.3	62.4	38.6
Colombia	Betulia	Count	2000	0	0	0
Colombia	Betulia	Count	2017	1	1	0
Colombia	Guarandá	Proportion	2000	37.3	45.8	28.5
Colombia	Guarandá	Proportion	2017	50.2	60.6	40.0
Colombia	Guarandá	Count	2000	0	0	0
Colombia	Guarandá	Count	2017	1	1	0
Colombia	Cajamarca	Proportion	2000	31.8	38.5	25.5
Colombia	Cajamarca	Proportion	2017	49.4	62.4	38.0
Colombia	Cajamarca	Count	2000	0	0	0
Colombia	Cajamarca	Count	2017	0	1	0
Colombia	Bugalagrande	Proportion	2000	35.8	42.5	28.5
Colombia	Bugalagrande	Proportion	2017	53.2	65.6	42.0
Colombia	Bugalagrande	Count	2000	0	0	0
Colombia	Bugalagrande	Count	2017	1	1	0
Colombia	Tarapacá	Proportion	2000	44.7	55.1	34.1

Table 4: ORS by Unit *(continued)*

Country	Unit	Metric	Year	Mean	Upper	Lower
Colombia	Tarapacá	Proportion	2017	53.3	62.7	42.0
Colombia	Tarapacá	Count	2000	0	1	0
Colombia	Tarapacá	Count	2017	1	1	0
Colombia	Angostura	Proportion	2000	33.2	39.5	25.7
Colombia	Angostura	Proportion	2017	44.4	56.6	34.1
Colombia	Angostura	Count	2000	0	0	0
Colombia	Angostura	Count	2017	0	1	0
Colombia	Malambo	Proportion	2000	50.3	58.7	42.8
Colombia	Malambo	Proportion	2017	76.7	82.3	70.4
Colombia	Malambo	Count	2000	1	1	0
Colombia	Malambo	Count	2017	1	1	1
Colombia	Cicuco	Proportion	2000	45.4	54.0	37.4
Colombia	Cicuco	Proportion	2017	56.9	68.2	45.7
Colombia	Cicuco	Count	2000	0	1	0
Colombia	Cicuco	Count	2017	1	1	0
Colombia	Boyacá	Proportion	2000	28.0	35.2	21.6
Colombia	Boyacá	Proportion	2017	47.6	61.7	35.5
Colombia	Boyacá	Count	2000	0	0	0
Colombia	Boyacá	Count	2017	0	1	0
Colombia	La Merced	Proportion	2000	34.7	41.7	27.3
Colombia	La Merced	Proportion	2017	56.2	67.4	45.9
Colombia	La Merced	Count	2000	0	0	0
Colombia	La Merced	Count	2017	1	1	0
Colombia	La Montañita	Proportion	2000	35.3	44.3	26.9
Colombia	La Montañita	Proportion	2017	45.1	55.8	34.9
Colombia	La Montañita	Count	2000	0	0	0
Colombia	La Montañita	Count	2017	0	1	0
Colombia	Orocué	Proportion	2000	33.8	42.6	25.9
Colombia	Orocué	Proportion	2017	45.7	56.9	34.1
Colombia	Orocué	Count	2000	0	0	0
Colombia	Orocué	Count	2017	0	1	0
Colombia	Caloto	Proportion	2000	33.5	41.1	27.3
Colombia	Caloto	Proportion	2017	53.1	64.1	41.2
Colombia	Caloto	Count	2000	0	0	0
Colombia	Caloto	Count	2017	1	1	0
Colombia	Curumaní	Proportion	2000	41.1	50.1	31.2
Colombia	Curumaní	Proportion	2017	52.2	63.2	40.8
Colombia	Curumaní	Count	2000	0	1	0
Colombia	Curumaní	Count	2017	1	1	0
Colombia	El Cantón del San Pablo	Proportion	2000	30.9	40.3	23.3
Colombia	El Cantón del San Pablo	Proportion	2017	43.5	55.7	32.4
Colombia	El Cantón del San Pablo	Count	2000	0	0	0
Colombia	El Cantón del San Pablo	Count	2017	0	1	0
Colombia	Los Córdobas	Proportion	2000	37.1	46.3	29.6
Colombia	Los Córdobas	Proportion	2017	56.8	68.0	45.2
Colombia	Los Córdobas	Count	2000	0	0	0
Colombia	Los Córdobas	Count	2017	1	1	0
Colombia	Bituima	Proportion	2000	31.1	37.1	25.4
Colombia	Bituima	Proportion	2017	47.8	61.4	35.8

Table 4: ORS by Unit *(continued)*

Country	Unit	Metric	Year	Mean	Upper	Lower
Colombia	Bituima	Count	2000	0	0	0
Colombia	Bituima	Count	2017	0	1	0
Colombia	Colombia	Proportion	2000	32.8	41.4	25.6
Colombia	Colombia	Proportion	2017	46.4	56.7	35.1
Colombia	Colombia	Count	2000	0	0	0
Colombia	Colombia	Count	2017	0	1	0
Colombia	Uribia	Proportion	2000	39.8	49.9	30.6
Colombia	Uribia	Proportion	2017	54.1	66.2	42.4
Colombia	Uribia	Count	2000	0	0	0
Colombia	Uribia	Count	2017	1	1	0
Colombia	Fundación	Proportion	2000	42.8	51.7	34.7
Colombia	Fundación	Proportion	2017	56.3	66.4	45.7
Colombia	Fundación	Count	2000	0	1	0
Colombia	Fundación	Count	2017	1	1	0
Colombia	El Dorado	Proportion	2000	31.9	40.0	24.4
Colombia	El Dorado	Proportion	2017	48.8	60.4	36.8
Colombia	El Dorado	Count	2000	0	0	0
Colombia	El Dorado	Count	2017	0	1	0
Colombia	Chachagüí	Proportion	2000	37.1	46.0	29.0
Colombia	Chachagüí	Proportion	2017	54.8	66.3	42.9
Colombia	Chachagüí	Count	2000	0	0	0
Colombia	Chachagüí	Count	2017	1	1	0
Colombia	Chitagá	Proportion	2000	32.9	39.8	25.3
Colombia	Chitagá	Proportion	2017	44.5	57.5	33.9
Colombia	Chitagá	Count	2000	0	0	0
Colombia	Chitagá	Count	2017	0	1	0
Colombia	San Miguel de Mocoa	Proportion	2000	34.8	43.4	26.0
Colombia	San Miguel de Mocoa	Proportion	2017	48.5	59.1	37.7
Colombia	San Miguel de Mocoa	Count	2000	0	0	0
Colombia	San Miguel de Mocoa	Count	2017	0	1	0
Colombia	La Tebaida	Proportion	2000	35.7	42.6	29.6
Colombia	La Tebaida	Proportion	2017	61.1	72.1	49.6
Colombia	La Tebaida	Count	2000	0	0	0
Colombia	La Tebaida	Count	2017	1	1	0
Colombia	Marsella	Proportion	2000	34.8	41.6	27.9
Colombia	Marsella	Proportion	2017	56.1	68.6	45.6
Colombia	Marsella	Count	2000	0	0	0
Colombia	Marsella	Count	2017	1	1	0
Colombia	Bolívar	Proportion	2000	33.8	40.9	26.1
Colombia	Bolívar	Proportion	2017	48.0	60.9	37.0
Colombia	Bolívar	Count	2000	0	0	0
Colombia	Bolívar	Count	2017	0	1	0
Colombia	La Unión de Sucre	Proportion	2000	43.0	52.0	34.7
Colombia	La Unión de Sucre	Proportion	2017	62.8	72.9	51.4
Colombia	La Unión de Sucre	Count	2000	0	1	0
Colombia	La Unión de Sucre	Count	2017	1	1	1
Colombia	Carmen de Apicalá	Proportion	2000	38.2	45.5	31.3
Colombia	Carmen de Apicalá	Proportion	2017	52.9	64.7	41.1
Colombia	Carmen de Apicalá	Count	2000	0	0	0

Table 4: ORS by Unit *(continued)*

Country	Unit	Metric	Year	Mean	Upper	Lower
Colombia	Carmen de Apicalá	Count	2017	1	1	0
Colombia	Caicedonia	Proportion	2000	34.0	40.7	27.7
Colombia	Caicedonia	Proportion	2017	53.8	65.4	41.7
Colombia	Caicedonia	Count	2000	0	0	0
Colombia	Caicedonia	Count	2017	1	1	0
Colombia	Anorí	Proportion	2000	34.7	41.0	27.6
Colombia	Anorí	Proportion	2017	46.7	57.6	36.3
Colombia	Anorí	Count	2000	0	0	0
Colombia	Anorí	Count	2017	0	1	0
Colombia	Manatí	Proportion	2000	45.8	55.0	38.3
Colombia	Manatí	Proportion	2017	59.8	69.7	49.2
Colombia	Manatí	Count	2000	0	1	0
Colombia	Manatí	Count	2017	1	1	0
Colombia	Córdoba	Proportion	2000	47.3	56.3	39.8
Colombia	Córdoba	Proportion	2017	65.0	75.3	54.3
Colombia	Córdoba	Count	2000	0	1	0
Colombia	Córdoba	Count	2017	1	1	1
Colombia	Briceño	Proportion	2000	31.0	36.9	24.1
Colombia	Briceño	Proportion	2017	48.2	61.8	36.8
Colombia	Briceño	Count	2000	0	0	0
Colombia	Briceño	Count	2017	0	1	0
Colombia	Manizales	Proportion	2000	35.9	43.8	29.0
Colombia	Manizales	Proportion	2017	53.9	65.6	43.6
Colombia	Manizales	Count	2000	0	0	0
Colombia	Manizales	Count	2017	1	1	0
Colombia	Milán	Proportion	2000	36.6	46.0	28.4
Colombia	Milán	Proportion	2017	43.1	54.5	32.4
Colombia	Milán	Count	2000	0	0	0
Colombia	Milán	Count	2017	0	1	0
Colombia	Paz de Ariporo	Proportion	2000	35.4	44.1	27.2
Colombia	Paz de Ariporo	Proportion	2017	46.3	57.3	35.3
Colombia	Paz de Ariporo	Count	2000	0	0	0
Colombia	Paz de Ariporo	Count	2017	0	1	0
Colombia	Corinto	Proportion	2000	33.9	41.4	27.3
Colombia	Corinto	Proportion	2017	52.1	63.8	39.4
Colombia	Corinto	Count	2000	0	0	0
Colombia	Corinto	Count	2017	1	1	0
Colombia	El Copey	Proportion	2000	43.2	51.9	34.2
Colombia	El Copey	Proportion	2017	57.0	67.3	46.6
Colombia	El Copey	Count	2000	0	1	0
Colombia	El Copey	Count	2017	1	1	0
Colombia	El Carmen de Atrato	Proportion	2000	28.6	35.7	20.7
Colombia	El Carmen de Atrato	Proportion	2017	42.0	54.6	31.2
Colombia	El Carmen de Atrato	Count	2000	0	0	0
Colombia	El Carmen de Atrato	Count	2017	0	1	0
Colombia	Momil	Proportion	2000	38.1	46.3	30.6
Colombia	Momil	Proportion	2017	58.1	68.9	47.5
Colombia	Momil	Count	2000	0	0	0
Colombia	Momil	Count	2017	1	1	0

Table 4: ORS by Unit *(continued)*

Country	Unit	Metric	Year	Mean	Upper	Lower
Colombia	Bojacá	Proportion	2000	32.9	38.5	27.3
Colombia	Bojacá	Proportion	2017	51.8	64.0	39.5
Colombia	Bojacá	Count	2000	0	0	0
Colombia	Bojacá	Count	2017	1	1	0
Colombia	Elías	Proportion	2000	35.5	43.3	27.6
Colombia	Elías	Proportion	2017	55.1	66.4	42.7
Colombia	Elías	Count	2000	0	0	0
Colombia	Elías	Count	2017	1	1	0
Colombia	Urumita	Proportion	2000	38.8	48.2	29.7
Colombia	Urumita	Proportion	2017	54.8	65.9	43.5
Colombia	Urumita	Count	2000	0	0	0
Colombia	Urumita	Count	2017	1	1	0
Colombia	Guamal	Proportion	2000	43.8	51.7	35.6
Colombia	Guamal	Proportion	2017	60.3	71.4	49.2
Colombia	Guamal	Count	2000	0	1	0
Colombia	Guamal	Count	2017	1	1	0
Colombia	Fuente de Oro	Proportion	2000	33.4	42.5	25.3
Colombia	Fuente de Oro	Proportion	2017	49.2	60.7	36.6
Colombia	Fuente de Oro	Count	2000	0	0	0
Colombia	Fuente de Oro	Count	2017	0	1	0
Colombia	Colón	Proportion	2000	35.9	44.8	27.9
Colombia	Colón	Proportion	2017	56.3	67.5	44.8
Colombia	Colón	Count	2000	0	0	0
Colombia	Colón	Count	2017	1	1	0
Colombia	Convención	Proportion	2000	39.9	49.1	31.6
Colombia	Convención	Proportion	2017	50.0	61.2	39.4
Colombia	Convención	Count	2000	0	0	0
Colombia	Convención	Count	2017	0	1	0
Colombia	Santiago	Proportion	2000	33.6	42.9	24.4
Colombia	Santiago	Proportion	2017	48.1	60.1	37.6
Colombia	Santiago	Count	2000	0	0	0
Colombia	Santiago	Count	2017	0	1	0
Colombia	Montenegro	Proportion	2000	34.0	41.2	28.0
Colombia	Montenegro	Proportion	2017	54.5	66.9	43.1
Colombia	Montenegro	Count	2000	0	0	0
Colombia	Montenegro	Count	2017	1	1	0
Colombia	Mistrato	Proportion	2000	35.3	43.7	28.3
Colombia	Mistrato	Proportion	2017	49.9	62.8	39.0
Colombia	Mistrato	Count	2000	0	0	0
Colombia	Mistrato	Count	2017	0	1	0
Colombia	Bucaramanga	Proportion	2000	35.1	41.9	28.3
Colombia	Bucaramanga	Proportion	2017	49.2	64.1	36.9
Colombia	Bucaramanga	Count	2000	0	0	0
Colombia	Bucaramanga	Count	2017	0	1	0
Colombia	Los Palmitos	Proportion	2000	42.7	51.5	34.5
Colombia	Los Palmitos	Proportion	2017	58.6	68.6	46.9
Colombia	Los Palmitos	Count	2000	0	1	0
Colombia	Los Palmitos	Count	2017	1	1	0
Colombia	Casabianca	Proportion	2000	29.7	36.7	23.8

Table 4: ORS by Unit *(continued)*

Country	Unit	Metric	Year	Mean	Upper	Lower
Colombia	Casabianca	Proportion	2017	43.5	55.4	33.7
Colombia	Casabianca	Count	2000	0	0	0
Colombia	Casabianca	Count	2017	0	1	0
Colombia	Calima	Proportion	2000	34.7	41.9	28.2
Colombia	Calima	Proportion	2017	51.0	63.5	40.4
Colombia	Calima	Count	2000	0	0	0
Colombia	Calima	Count	2017	1	1	0
Colombia	Anzá	Proportion	2000	37.4	43.3	29.2
Colombia	Anzá	Proportion	2017	51.4	65.1	39.5
Colombia	Anzá	Count	2000	0	0	0
Colombia	Anzá	Count	2017	1	1	0
Colombia	Palmar de Varela	Proportion	2000	47.4	56.8	39.3
Colombia	Palmar de Varela	Proportion	2017	58.9	68.7	48.0
Colombia	Palmar de Varela	Count	2000	0	1	0
Colombia	Palmar de Varela	Count	2017	1	1	0
Colombia	El Carmen de Bolívar	Proportion	2000	44.0	52.0	35.6
Colombia	El Carmen de Bolívar	Proportion	2017	56.7	67.1	45.3
Colombia	El Carmen de Bolívar	Count	2000	0	1	0
Colombia	El Carmen de Bolívar	Count	2017	1	1	0
Colombia	Buenavista	Proportion	2000	31.8	37.7	25.6
Colombia	Buenavista	Proportion	2017	49.8	63.9	38.5
Colombia	Buenavista	Count	2000	0	0	0
Colombia	Buenavista	Count	2017	0	1	0
Colombia	Manzanares	Proportion	2000	32.6	40.3	26.4
Colombia	Manzanares	Proportion	2017	49.2	61.1	39.2
Colombia	Manzanares	Count	2000	0	0	0
Colombia	Manzanares	Count	2017	0	1	0
Colombia	Morelia	Proportion	2000	34.5	43.3	25.3
Colombia	Morelia	Proportion	2017	44.2	55.0	33.8
Colombia	Morelia	Count	2000	0	0	0
Colombia	Morelia	Count	2017	0	1	0
Colombia	Pore	Proportion	2000	36.4	45.6	27.9
Colombia	Pore	Proportion	2017	47.6	59.8	35.8
Colombia	Pore	Count	2000	0	0	0
Colombia	Pore	Count	2017	0	1	0
Colombia	El Tambo	Proportion	2000	30.4	37.3	23.4
Colombia	El Tambo	Proportion	2017	46.2	57.3	34.7
Colombia	El Tambo	Count	2000	0	0	0
Colombia	El Tambo	Count	2017	0	1	0
Colombia	El Paso	Proportion	2000	43.2	52.3	33.3
Colombia	El Paso	Proportion	2017	61.5	72.1	49.9
Colombia	El Paso	Count	2000	0	1	0
Colombia	El Paso	Count	2017	1	1	0
Colombia	El Litoral del San Juan	Proportion	2000	29.5	37.1	21.5
Colombia	El Litoral del San Juan	Proportion	2017	42.0	54.3	32.2
Colombia	El Litoral del San Juan	Count	2000	0	0	0
Colombia	El Litoral del San Juan	Count	2017	0	1	0
Colombia	Moñitos	Proportion	2000	37.0	46.8	29.0
Colombia	Moñitos	Proportion	2017	56.8	67.3	44.6

Table 4: ORS by Unit *(continued)*

Country	Unit	Metric	Year	Mean	Upper	Lower
Colombia	Moñitos	Count	2000	0	0	0
Colombia	Moñitos	Count	2017	1	1	0
Colombia	Cabrera	Proportion	2000	31.3	38.5	24.9
Colombia	Cabrera	Proportion	2017	44.4	56.2	32.7
Colombia	Cabrera	Count	2000	0	0	0
Colombia	Cabrera	Count	2017	0	1	0
Colombia	Garzón	Proportion	2000	35.4	42.8	27.1
Colombia	Garzón	Proportion	2017	54.4	65.6	41.7
Colombia	Garzón	Count	2000	0	0	0
Colombia	Garzón	Count	2017	1	1	0
Colombia	Villanueva	Proportion	2000	36.0	45.4	27.0
Colombia	Villanueva	Proportion	2017	51.9	63.1	40.9
Colombia	Villanueva	Count	2000	0	0	0
Colombia	Villanueva	Count	2017	1	1	0
Colombia	Pedraza	Proportion	2000	46.6	54.9	39.5
Colombia	Pedraza	Proportion	2017	60.7	70.2	50.0
Colombia	Pedraza	Count	2000	0	1	0
Colombia	Pedraza	Count	2017	1	1	0
Colombia	Granada	Proportion	2000	33.6	42.7	25.7
Colombia	Granada	Proportion	2017	49.8	60.8	37.2
Colombia	Granada	Count	2000	0	0	0
Colombia	Granada	Count	2017	0	1	0
Colombia	Consacá	Proportion	2000	38.4	48.1	30.3
Colombia	Consacá	Proportion	2017	57.8	68.9	45.7
Colombia	Consacá	Count	2000	0	0	0
Colombia	Consacá	Count	2017	1	1	0
Colombia	Cucutilla	Proportion	2000	36.5	44.6	28.6
Colombia	Cucutilla	Proportion	2017	49.1	62.5	38.6
Colombia	Cucutilla	Count	2000	0	0	0
Colombia	Cucutilla	Count	2017	0	1	0
Colombia	Sibundoy	Proportion	2000	32.3	41.9	23.6
Colombia	Sibundoy	Proportion	2017	51.3	63.2	40.4
Colombia	Sibundoy	Count	2000	0	0	0
Colombia	Sibundoy	Count	2017	1	1	0
Colombia	Pijao	Proportion	2000	34.9	41.8	28.7
Colombia	Pijao	Proportion	2017	55.1	66.7	43.4
Colombia	Pijao	Count	2000	0	0	0
Colombia	Pijao	Count	2017	1	1	0
Colombia	Pereira	Proportion	2000	33.2	39.9	26.5
Colombia	Pereira	Proportion	2017	57.0	68.6	47.1
Colombia	Pereira	Count	2000	0	0	0
Colombia	Pereira	Count	2017	1	1	0
Colombia	Cabrera	Proportion	2000	35.5	44.2	28.6
Colombia	Cabrera	Proportion	2017	51.0	63.8	39.0
Colombia	Cabrera	Count	2000	0	0	0
Colombia	Cabrera	Count	2017	1	1	0
Colombia	Majagual	Proportion	2000	38.9	47.4	29.9
Colombia	Majagual	Proportion	2017	53.4	63.7	42.6
Colombia	Majagual	Count	2000	0	0	0

Table 4: ORS by Unit *(continued)*

Country	Unit	Metric	Year	Mean	Upper	Lower
Colombia	Majagual	Count	2017	1	1	0
Colombia	Chaparral	Proportion	2000	34.7	42.1	27.5
Colombia	Chaparral	Proportion	2017	50.8	61.8	39.8
Colombia	Chaparral	Count	2000	0	0	0
Colombia	Chaparral	Count	2017	1	1	0
Colombia	Candelaria	Proportion	2000	36.5	44.1	30.0
Colombia	Candelaria	Proportion	2017	50.9	61.8	39.0
Colombia	Candelaria	Count	2000	0	0	0
Colombia	Candelaria	Count	2017	1	1	0
Colombia	Apartadó	Proportion	2000	38.6	48.1	28.8
Colombia	Apartadó	Proportion	2017	52.7	65.0	41.6
Colombia	Apartadó	Count	2000	0	0	0
Colombia	Apartadó	Count	2017	1	1	0
Colombia	Piojó	Proportion	2000	42.8	51.1	34.8
Colombia	Piojó	Proportion	2017	52.5	63.2	41.7
Colombia	Piojó	Count	2000	0	1	0
Colombia	Piojó	Count	2017	1	1	0
Colombia	El Guamo	Proportion	2000	45.7	53.8	37.1
Colombia	El Guamo	Proportion	2017	58.5	68.5	47.4
Colombia	El Guamo	Count	2000	0	1	0
Colombia	El Guamo	Count	2017	1	1	0
Colombia	Busbanza	Proportion	2000	29.4	36.8	22.8
Colombia	Busbanza	Proportion	2017	52.0	64.6	39.8
Colombia	Busbanza	Count	2000	0	0	0
Colombia	Busbanza	Count	2017	1	1	0
Colombia	Marmato	Proportion	2000	35.2	41.9	27.6
Colombia	Marmato	Proportion	2017	55.8	67.0	45.6
Colombia	Marmato	Count	2000	0	0	0
Colombia	Marmato	Count	2017	1	1	0
Colombia	Puerto Rico	Proportion	2000	35.4	43.9	27.4
Colombia	Puerto Rico	Proportion	2017	47.4	58.5	37.2
Colombia	Puerto Rico	Count	2000	0	0	0
Colombia	Puerto Rico	Count	2017	0	1	0
Colombia	Recetor	Proportion	2000	29.3	37.3	21.8
Colombia	Recetor	Proportion	2017	42.7	55.7	30.0
Colombia	Recetor	Count	2000	0	0	0
Colombia	Recetor	Count	2017	0	1	0
Colombia	Florencia	Proportion	2000	34.9	43.7	27.2
Colombia	Florencia	Proportion	2017	52.9	64.1	41.4
Colombia	Florencia	Count	2000	0	0	0
Colombia	Florencia	Count	2017	1	1	0
Colombia	Gamarra	Proportion	2000	40.6	48.6	31.9
Colombia	Gamarra	Proportion	2017	55.0	66.8	44.0
Colombia	Gamarra	Count	2000	0	0	0
Colombia	Gamarra	Count	2017	1	1	0
Colombia	Istmina	Proportion	2000	32.0	41.0	24.1
Colombia	Istmina	Proportion	2017	45.5	58.8	34.2
Colombia	Istmina	Count	2000	0	0	0
Colombia	Istmina	Count	2017	0	1	0

Table 4: ORS by Unit *(continued)*

Country	Unit	Metric	Year	Mean	Upper	Lower
Colombia	Montelíbano	Proportion	2000	36.7	44.7	29.3
Colombia	Montelíbano	Proportion	2017	53.6	65.6	41.7
Colombia	Montelíbano	Count	2000	0	0	0
Colombia	Montelíbano	Count	2017	1	1	0
Colombia	Cachipay	Proportion	2000	36.0	41.9	30.0
Colombia	Cachipay	Proportion	2017	50.7	63.2	38.6
Colombia	Cachipay	Count	2000	0	0	0
Colombia	Cachipay	Count	2017	1	1	0
Colombia	Gigante	Proportion	2000	36.1	44.1	28.0
Colombia	Gigante	Proportion	2017	54.6	65.7	41.6
Colombia	Gigante	Count	2000	0	0	0
Colombia	Gigante	Count	2017	1	1	0
Colombia	Pivijay	Proportion	2000	44.9	53.2	37.3
Colombia	Pivijay	Proportion	2017	58.6	69.1	48.5
Colombia	Pivijay	Count	2000	0	1	0
Colombia	Pivijay	Count	2017	1	1	0
Colombia	Guamal	Proportion	2000	30.3	38.1	24.1
Colombia	Guamal	Proportion	2017	44.5	56.3	32.3
Colombia	Guamal	Count	2000	0	0	0
Colombia	Guamal	Count	2017	0	1	0
Colombia	Contadero	Proportion	2000	36.5	46.0	27.6
Colombia	Contadero	Proportion	2017	53.6	65.0	43.0
Colombia	Contadero	Count	2000	0	0	0
Colombia	Contadero	Count	2017	1	1	0
Colombia	Durania	Proportion	2000	39.4	47.4	30.8
Colombia	Durania	Proportion	2017	48.5	61.2	36.7
Colombia	Durania	Count	2000	0	0	0
Colombia	Durania	Count	2017	0	1	0
Colombia	Valle del Guamuez	Proportion	2000	36.3	45.9	27.3
Colombia	Valle del Guamuez	Proportion	2017	43.6	57.0	33.2
Colombia	Valle del Guamuez	Count	2000	0	0	0
Colombia	Valle del Guamuez	Count	2017	0	1	0
Colombia	Quimbaya	Proportion	2000	35.2	42.0	29.0
Colombia	Quimbaya	Proportion	2017	54.5	67.7	43.3
Colombia	Quimbaya	Count	2000	0	0	0
Colombia	Quimbaya	Count	2017	1	1	0
Colombia	Pueblo Rico	Proportion	2000	32.0	39.7	25.3
Colombia	Pueblo Rico	Proportion	2017	45.2	58.3	34.1
Colombia	Pueblo Rico	Count	2000	0	0	0
Colombia	Pueblo Rico	Count	2017	0	1	0
Colombia	California	Proportion	2000	34.8	43.0	27.9
Colombia	California	Proportion	2017	47.3	60.6	35.5
Colombia	California	Count	2000	0	0	0
Colombia	California	Count	2017	0	1	0
Colombia	Morroa	Proportion	2000	44.2	52.9	35.5
Colombia	Morroa	Proportion	2017	60.1	70.3	48.2
Colombia	Morroa	Count	2000	0	1	0
Colombia	Morroa	Count	2017	1	1	0
Colombia	Coello	Proportion	2000	36.7	43.3	30.5

Table 4: ORS by Unit *(continued)*

Country	Unit	Metric	Year	Mean	Upper	Lower
Colombia	Coello	Proportion	2017	52.4	65.2	41.0
Colombia	Coello	Count	2000	0	0	0
Colombia	Coello	Count	2017	1	1	0
Colombia	Cartago	Proportion	2000	33.7	40.2	27.0
Colombia	Cartago	Proportion	2017	54.1	67.3	43.1
Colombia	Cartago	Count	2000	0	0	0
Colombia	Cartago	Count	2017	1	1	0
Colombia	Arboletes	Proportion	2000	40.3	49.2	32.5
Colombia	Arboletes	Proportion	2017	56.2	67.2	45.0
Colombia	Arboletes	Count	2000	0	0	0
Colombia	Arboletes	Count	2017	1	1	0
Colombia	Polonuevo	Proportion	2000	47.2	56.5	39.0
Colombia	Polonuevo	Proportion	2017	58.2	68.1	47.2
Colombia	Polonuevo	Count	2000	0	1	0
Colombia	Polonuevo	Count	2017	1	1	0
Colombia	Hatillo de Loba	Proportion	2000	42.3	50.6	33.7
Colombia	Hatillo de Loba	Proportion	2017	53.2	64.7	41.1
Colombia	Hatillo de Loba	Count	2000	0	1	0
Colombia	Hatillo de Loba	Count	2017	1	1	0
Colombia	Caldas	Proportion	2000	28.3	34.3	22.3
Colombia	Caldas	Proportion	2017	48.0	61.5	36.7
Colombia	Caldas	Count	2000	0	0	0
Colombia	Caldas	Count	2017	0	1	0
Colombia	Marquetalia	Proportion	2000	34.9	43.0	27.5
Colombia	Marquetalia	Proportion	2017	52.9	65.1	42.4
Colombia	Marquetalia	Count	2000	0	0	0
Colombia	Marquetalia	Count	2017	1	1	0
Colombia	San José del Fragua	Proportion	2000	36.0	45.8	26.4
Colombia	San José del Fragua	Proportion	2017	44.7	55.0	33.9
Colombia	San José del Fragua	Count	2000	0	0	0
Colombia	San José del Fragua	Count	2017	0	1	0
Colombia	Sabanalarga	Proportion	2000	32.7	41.1	25.4
Colombia	Sabanalarga	Proportion	2017	47.3	59.8	34.7
Colombia	Sabanalarga	Count	2000	0	0	0
Colombia	Sabanalarga	Count	2017	0	1	0
Colombia	Guapí	Proportion	2000	30.8	37.1	23.7
Colombia	Guapí	Proportion	2017	41.9	52.4	30.5
Colombia	Guapí	Count	2000	0	0	0
Colombia	Guapí	Count	2017	0	1	0
Colombia	González	Proportion	2000	38.1	46.4	30.6
Colombia	González	Proportion	2017	52.2	63.3	40.7
Colombia	González	Count	2000	0	0	0
Colombia	González	Count	2017	1	1	0
Colombia	Juradó	Proportion	2000	34.1	44.4	25.0
Colombia	Juradó	Proportion	2017	40.8	52.9	30.4
Colombia	Juradó	Count	2000	0	0	0
Colombia	Juradó	Count	2017	0	1	0
Colombia	Montería	Proportion	2000	37.0	45.3	29.6
Colombia	Montería	Proportion	2017	55.0	65.4	44.1

Table 4: ORS by Unit *(continued)*

Country	Unit	Metric	Year	Mean	Upper	Lower
Colombia	Montería	Count	2000	0	0	0
Colombia	Montería	Count	2017	1	1	0
Colombia	Cajicá	Proportion	2000	31.5	37.8	25.6
Colombia	Cajicá	Proportion	2017	48.1	61.5	35.4
Colombia	Cajicá	Count	2000	0	0	0
Colombia	Cajicá	Count	2017	0	1	0
Colombia	Guadalupe	Proportion	2000	34.8	42.4	27.2
Colombia	Guadalupe	Proportion	2017	53.1	64.1	41.1
Colombia	Guadalupe	Count	2000	0	0	0
Colombia	Guadalupe	Count	2017	1	1	0
Colombia	Plato	Proportion	2000	45.3	53.7	37.0
Colombia	Plato	Proportion	2017	59.6	70.9	48.3
Colombia	Plato	Count	2000	0	1	0
Colombia	Plato	Count	2017	1	1	0
Colombia	La Macarena	Proportion	2000	32.9	41.8	23.9
Colombia	La Macarena	Proportion	2017	44.7	54.4	34.3
Colombia	La Macarena	Count	2000	0	0	0
Colombia	La Macarena	Count	2017	0	1	0
Colombia	Córdoba	Proportion	2000	35.6	45.2	26.6
Colombia	Córdoba	Proportion	2017	53.4	64.9	42.8
Colombia	Córdoba	Count	2000	0	0	0
Colombia	Córdoba	Count	2017	1	1	0
Colombia	El Carmen	Proportion	2000	39.3	48.4	31.4
Colombia	El Carmen	Proportion	2017	48.8	59.9	38.1
Colombia	El Carmen	Count	2000	0	0	0
Colombia	El Carmen	Count	2017	0	1	0
Colombia	Villagarzón	Proportion	2000	34.9	43.2	25.6
Colombia	Villagarzón	Proportion	2017	44.4	55.5	34.1
Colombia	Villagarzón	Count	2000	0	0	0
Colombia	Villagarzón	Count	2017	0	1	0
Colombia	Salento	Proportion	2000	33.6	40.6	26.4
Colombia	Salento	Proportion	2017	50.4	62.3	39.1
Colombia	Salento	Count	2000	0	0	0
Colombia	Salento	Count	2017	1	1	0
Colombia	Quinchía	Proportion	2000	32.7	39.6	26.3
Colombia	Quinchía	Proportion	2017	48.8	61.8	38.2
Colombia	Quinchía	Count	2000	0	0	0
Colombia	Quinchía	Count	2017	0	1	0
Colombia	Capitanejo	Proportion	2000	36.2	44.1	29.0
Colombia	Capitanejo	Proportion	2017	53.4	67.0	42.1
Colombia	Capitanejo	Count	2000	0	0	0
Colombia	Capitanejo	Count	2017	1	1	0
Colombia	Ovejas	Proportion	2000	44.2	52.8	35.6
Colombia	Ovejas	Proportion	2017	57.3	68.0	45.9
Colombia	Ovejas	Count	2000	0	1	0
Colombia	Ovejas	Count	2017	1	1	0
Colombia	Coyaima	Proportion	2000	37.3	44.5	29.6
Colombia	Coyaima	Proportion	2017	53.7	65.1	41.6
Colombia	Coyaima	Count	2000	0	0	0

Table 4: ORS by Unit *(continued)*

Country	Unit	Metric	Year	Mean	Upper	Lower
Colombia	Coyaima	Count	2017	1	1	0
Colombia	Dagua	Proportion	2000	35.9	42.6	30.0
Colombia	Dagua	Proportion	2017	52.3	64.4	41.6
Colombia	Dagua	Count	2000	0	0	0
Colombia	Dagua	Count	2017	1	1	0
Colombia	Argelia	Proportion	2000	33.2	40.5	25.8
Colombia	Argelia	Proportion	2017	47.3	58.9	36.8
Colombia	Argelia	Count	2000	0	0	0
Colombia	Argelia	Count	2017	0	1	0
Colombia	Ponedera	Proportion	2000	45.6	54.8	38.1
Colombia	Ponedera	Proportion	2017	57.5	67.4	46.5
Colombia	Ponedera	Count	2000	0	1	0
Colombia	Ponedera	Count	2017	1	1	0
Colombia	Mahates	Proportion	2000	47.1	55.8	38.2
Colombia	Mahates	Proportion	2017	57.4	67.6	46.2
Colombia	Mahates	Count	2000	0	1	0
Colombia	Mahates	Count	2017	1	1	0
Colombia	Campohermoso	Proportion	2000	30.3	38.2	23.8
Colombia	Campohermoso	Proportion	2017	43.7	56.2	31.6
Colombia	Campohermoso	Count	2000	0	0	0
Colombia	Campohermoso	Count	2017	0	1	0
Colombia	Marulanda	Proportion	2000	30.6	37.7	24.6
Colombia	Marulanda	Proportion	2017	43.4	54.8	33.3
Colombia	Marulanda	Count	2000	0	0	0
Colombia	Marulanda	Count	2017	0	1	0
Colombia	San Vicente del Caguán	Proportion	2000	34.6	42.8	26.3
Colombia	San Vicente del Caguán	Proportion	2017	46.6	55.8	35.5
Colombia	San Vicente del Caguán	Count	2000	0	0	0
Colombia	San Vicente del Caguán	Count	2017	0	1	0
Colombia	Sácama	Proportion	2000	33.6	42.1	26.1
Colombia	Sácama	Proportion	2017	45.8	59.5	33.3
Colombia	Sácama	Count	2000	0	0	0
Colombia	Sácama	Count	2017	0	1	0
Colombia	Inzá	Proportion	2000	30.2	37.6	23.1
Colombia	Inzá	Proportion	2017	50.6	61.9	38.7
Colombia	Inzá	Count	2000	0	0	0
Colombia	Inzá	Count	2017	1	1	0
Colombia	La Gloria	Proportion	2000	40.7	49.2	32.4
Colombia	La Gloria	Proportion	2017	53.7	64.5	42.5
Colombia	La Gloria	Count	2000	0	0	0
Colombia	La Gloria	Count	2017	1	1	0
Colombia	Lloró	Proportion	2000	30.0	38.7	22.4
Colombia	Lloró	Proportion	2017	44.3	57.2	33.3
Colombia	Lloró	Count	2000	0	0	0
Colombia	Lloró	Count	2017	0	1	0
Colombia	Planeta Rica	Proportion	2000	36.3	45.3	28.7
Colombia	Planeta Rica	Proportion	2017	55.2	66.5	43.3
Colombia	Planeta Rica	Count	2000	0	0	0
Colombia	Planeta Rica	Count	2017	1	1	0

Table 4: ORS by Unit *(continued)*

Country	Unit	Metric	Year	Mean	Upper	Lower
Colombia	Caparrapí	Proportion	2000	35.6	41.6	28.4
Colombia	Caparrapí	Proportion	2017	50.0	63.0	38.2
Colombia	Caparrapí	Count	2000	0	0	0
Colombia	Caparrapí	Count	2017	0	1	0
Colombia	Hobo	Proportion	2000	38.6	47.0	30.1
Colombia	Hobo	Proportion	2017	54.9	66.8	41.5
Colombia	Hobo	Count	2000	0	0	0
Colombia	Hobo	Count	2017	1	1	0
Colombia	Pueblo Viejo	Proportion	2000	45.2	52.9	37.5
Colombia	Pueblo Viejo	Proportion	2017	60.4	70.1	48.8
Colombia	Pueblo Viejo	Count	2000	0	1	0
Colombia	Pueblo Viejo	Count	2017	1	1	0
Colombia	La Uribe	Proportion	2000	31.7	39.9	24.3
Colombia	La Uribe	Proportion	2017	44.1	54.8	32.0
Colombia	La Uribe	Count	2000	0	0	0
Colombia	La Uribe	Count	2017	0	1	0
Colombia	Cuaspud	Proportion	2000	35.2	45.7	25.6
Colombia	Cuaspud	Proportion	2017	55.4	66.7	44.5
Colombia	Cuaspud	Count	2000	0	0	0
Colombia	Cuaspud	Count	2017	1	1	0
Colombia	El Tarra	Proportion	2000	42.5	51.8	34.2
Colombia	El Tarra	Proportion	2017	48.7	60.7	37.1
Colombia	El Tarra	Count	2000	0	1	0
Colombia	El Tarra	Count	2017	0	1	0
Colombia	Santa Rosa de Cabal	Proportion	2000	32.1	39.2	25.2
Colombia	Santa Rosa de Cabal	Proportion	2017	52.6	64.6	41.5
Colombia	Santa Rosa de Cabal	Count	2000	0	0	0
Colombia	Santa Rosa de Cabal	Count	2017	1	1	0
Colombia	Carcasí	Proportion	2000	32.5	40.1	25.6
Colombia	Carcasí	Proportion	2017	46.6	61.3	35.0
Colombia	Carcasí	Count	2000	0	0	0
Colombia	Carcasí	Count	2017	0	1	0
Colombia	Sampués	Proportion	2000	41.1	49.9	33.4
Colombia	Sampués	Proportion	2017	57.8	68.3	45.8
Colombia	Sampués	Count	2000	0	0	0
Colombia	Sampués	Count	2017	1	1	0
Colombia	Cunday	Proportion	2000	35.8	43.5	29.0
Colombia	Cunday	Proportion	2017	51.0	62.7	38.8
Colombia	Cunday	Count	2000	0	0	0
Colombia	Cunday	Count	2017	1	1	0
Colombia	El Águila	Proportion	2000	30.2	37.4	23.0
Colombia	El Águila	Proportion	2017	43.9	57.6	33.1
Colombia	El Águila	Count	2000	0	0	0
Colombia	El Águila	Count	2017	0	1	0
Colombia	Armenia	Proportion	2000	39.8	46.3	31.4
Colombia	Armenia	Proportion	2017	58.1	70.3	46.2
Colombia	Armenia	Count	2000	0	0	0
Colombia	Armenia	Count	2017	1	1	0

Table 4: ORS by Unit *(continued)*

Country	Unit	Metric	Year	Mean	Upper	Lower
Colombia	Puerto Colombia	Proportion	2000	47.5	56.0	40.3
Colombia	Puerto Colombia	Proportion	2017	77.0	82.2	70.6
Colombia	Puerto Colombia	Count	2000	0	1	0
Colombia	Puerto Colombia	Count	2017	1	1	1
Colombia	Margarita	Proportion	2000	43.7	51.5	35.5
Colombia	Margarita	Proportion	2017	54.5	66.0	42.5
Colombia	Margarita	Count	2000	0	1	0
Colombia	Margarita	Count	2017	1	1	0
Colombia	Cerinza	Proportion	2000	29.5	36.4	22.9
Colombia	Cerinza	Proportion	2017	52.1	64.7	40.3
Colombia	Cerinza	Count	2000	0	0	0
Colombia	Cerinza	Count	2017	1	1	0
Colombia	Neira	Proportion	2000	35.2	42.3	28.7
Colombia	Neira	Proportion	2017	54.3	66.0	44.6
Colombia	Neira	Count	2000	0	0	0
Colombia	Neira	Count	2017	1	1	0
Colombia	Solano	Proportion	2000	37.2	45.6	28.5
Colombia	Solano	Proportion	2017	46.7	57.1	36.6
Colombia	Solano	Count	2000	0	0	0
Colombia	Solano	Count	2017	0	1	0
Colombia	San Luis de Palenque	Proportion	2000	33.7	42.8	25.4
Colombia	San Luis de Palenque	Proportion	2017	45.7	56.6	33.6
Colombia	San Luis de Palenque	Count	2000	0	0	0
Colombia	San Luis de Palenque	Count	2017	0	1	0
Colombia	Jambaló	Proportion	2000	30.1	37.1	23.5
Colombia	Jambaló	Proportion	2017	55.0	65.9	42.9
Colombia	Jambaló	Count	2000	0	0	0
Colombia	Jambaló	Count	2017	1	1	0
Colombia	La Jagua de Ibirico	Proportion	2000	40.5	49.6	29.9
Colombia	La Jagua de Ibirico	Proportion	2017	55.7	66.8	44.2
Colombia	La Jagua de Ibirico	Count	2000	0	0	0
Colombia	La Jagua de Ibirico	Count	2017	1	1	0
Colombia	Novita	Proportion	2000	28.7	36.6	21.4
Colombia	Novita	Proportion	2017	40.4	54.0	30.1
Colombia	Novita	Count	2000	0	0	0
Colombia	Novita	Count	2017	0	1	0
Colombia	Pueblo Nuevo	Proportion	2000	36.6	46.4	28.4
Colombia	Pueblo Nuevo	Proportion	2017	56.4	67.4	45.1
Colombia	Pueblo Nuevo	Count	2000	0	0	0
Colombia	Pueblo Nuevo	Count	2017	1	1	0
Colombia	Cáqueza	Proportion	2000	30.2	36.6	24.4
Colombia	Cáqueza	Proportion	2017	47.8	62.4	35.8
Colombia	Cáqueza	Count	2000	0	0	0
Colombia	Cáqueza	Count	2017	0	1	0
Colombia	Iquira	Proportion	2000	34.2	41.6	26.7
Colombia	Iquira	Proportion	2017	51.9	63.4	39.1
Colombia	Iquira	Count	2000	0	0	0
Colombia	Iquira	Count	2017	1	1	0
Colombia	Remolino	Proportion	2000	43.9	52.7	36.6

Table 4: ORS by Unit *(continued)*

Country	Unit	Metric	Year	Mean	Upper	Lower
Colombia	Remolino	Proportion	2017	59.0	68.9	48.2
Colombia	Remolino	Count	2000	0	1	0
Colombia	Remolino	Count	2017	1	1	0
Colombia	Lejanías	Proportion	2000	32.1	40.9	24.6
Colombia	Lejanías	Proportion	2017	46.9	58.5	34.3
Colombia	Lejanías	Count	2000	0	0	0
Colombia	Lejanías	Count	2017	0	1	0
Colombia	Cumbal	Proportion	2000	36.2	47.3	26.7
Colombia	Cumbal	Proportion	2017	52.6	64.2	42.4
Colombia	Cumbal	Count	2000	0	0	0
Colombia	Cumbal	Count	2017	1	1	0
Colombia	El Zulia	Proportion	2000	40.3	48.5	32.1
Colombia	El Zulia	Proportion	2017	51.2	63.4	39.5
Colombia	El Zulia	Count	2000	0	0	0
Colombia	El Zulia	Count	2017	1	1	0
Colombia	Santuario	Proportion	2000	32.7	39.6	25.2
Colombia	Santuario	Proportion	2017	51.0	63.5	40.8
Colombia	Santuario	Count	2000	0	0	0
Colombia	Santuario	Count	2017	1	1	0
Colombia	Cepitá	Proportion	2000	37.8	44.5	30.5
Colombia	Cepitá	Proportion	2017	54.3	68.2	42.3
Colombia	Cepitá	Count	2000	0	0	0
Colombia	Cepitá	Count	2017	1	1	0
Colombia	San Antonio de Palmito	Proportion	2000	42.5	50.7	34.6
Colombia	San Antonio de Palmito	Proportion	2017	61.8	72.2	50.5
Colombia	San Antonio de Palmito	Count	2000	0	1	0
Colombia	San Antonio de Palmito	Count	2017	1	1	1
Colombia	Dolores	Proportion	2000	34.5	42.2	27.6
Colombia	Dolores	Proportion	2017	50.2	61.1	38.3
Colombia	Dolores	Count	2000	0	0	0
Colombia	Dolores	Count	2017	1	1	0
Colombia	El Cairo	Proportion	2000	28.1	35.8	21.3
Colombia	El Cairo	Proportion	2017	41.5	55.2	30.8
Colombia	El Cairo	Count	2000	0	0	0
Colombia	El Cairo	Count	2017	0	1	0
Colombia	Barbosa	Proportion	2000	37.4	44.2	30.3
Colombia	Barbosa	Proportion	2017	56.8	68.0	45.1
Colombia	Barbosa	Count	2000	0	0	0
Colombia	Barbosa	Count	2017	1	1	0
Colombia	Repelón	Proportion	2000	45.1	54.2	37.5
Colombia	Repelón	Proportion	2017	56.8	66.8	46.4
Colombia	Repelón	Count	2000	0	1	0
Colombia	Repelón	Count	2017	1	1	0
Colombia	María la Baja	Proportion	2000	46.1	54.7	37.6
Colombia	María la Baja	Proportion	2017	57.1	67.5	46.5
Colombia	María la Baja	Count	2000	0	1	0
Colombia	María la Baja	Count	2017	1	1	0
Colombia	Chinavita	Proportion	2000	30.4	37.9	24.1
Colombia	Chinavita	Proportion	2017	50.5	64.2	38.6

Table 4: ORS by Unit *(continued)*

Country	Unit	Metric	Year	Mean	Upper	Lower
Colombia	Chinavita	Count	2000	0	0	0
Colombia	Chinavita	Count	2017	1	1	0
Colombia	Pácora	Proportion	2000	35.3	41.9	27.7
Colombia	Pácora	Proportion	2017	55.8	66.6	45.5
Colombia	Pácora	Count	2000	0	0	0
Colombia	Pácora	Count	2017	1	1	0
Colombia	Valparaíso	Proportion	2000	37.3	46.7	28.5
Colombia	Valparaíso	Proportion	2017	44.8	55.4	34.5
Colombia	Valparaíso	Count	2000	0	0	0
Colombia	Valparaíso	Count	2017	0	1	0
Colombia	Támara	Proportion	2000	33.3	41.6	25.3
Colombia	Támara	Proportion	2017	43.6	56.5	31.3
Colombia	Támara	Count	2000	0	0	0
Colombia	Támara	Count	2017	0	1	0
Colombia	La Sierra	Proportion	2000	30.2	37.5	23.8
Colombia	La Sierra	Proportion	2017	51.0	62.7	38.6
Colombia	La Sierra	Count	2000	0	0	0
Colombia	La Sierra	Count	2017	1	1	0
Colombia	La Paz	Proportion	2000	40.7	49.8	31.4
Colombia	La Paz	Proportion	2017	56.5	67.5	45.2
Colombia	La Paz	Count	2000	0	0	0
Colombia	La Paz	Count	2017	1	1	0
Colombia	Nuquí	Proportion	2000	31.6	40.9	23.9
Colombia	Nuquí	Proportion	2017	39.9	51.2	29.5
Colombia	Nuquí	Count	2000	0	0	0
Colombia	Nuquí	Count	2017	0	1	0
Colombia	Puerto Escondido	Proportion	2000	36.8	46.1	29.1
Colombia	Puerto Escondido	Proportion	2017	56.0	67.3	43.7
Colombia	Puerto Escondido	Count	2000	0	0	0
Colombia	Puerto Escondido	Count	2017	1	1	0
Colombia	Carmen de Carupa	Proportion	2000	29.1	34.9	23.3
Colombia	Carmen de Carupa	Proportion	2017	46.2	61.3	34.6
Colombia	Carmen de Carupa	Count	2000	0	0	0
Colombia	Carmen de Carupa	Count	2017	0	1	0
Colombia	Isnos	Proportion	2000	33.1	40.6	25.4
Colombia	Isnos	Proportion	2017	54.0	65.4	41.4
Colombia	Isnos	Count	2000	0	0	0
Colombia	Isnos	Count	2017	1	1	0
Colombia	Salamina	Proportion	2000	44.3	53.3	37.5
Colombia	Salamina	Proportion	2017	58.8	68.7	48.1
Colombia	Salamina	Count	2000	0	1	0
Colombia	Salamina	Count	2017	1	1	0
Colombia	Mapiripán	Proportion	2000	31.1	40.3	22.1
Colombia	Mapiripán	Proportion	2017	42.8	54.5	31.3
Colombia	Mapiripán	Count	2000	0	0	0
Colombia	Mapiripán	Count	2017	0	1	0
Colombia	Cumbitara	Proportion	2000	37.9	47.2	29.1
Colombia	Cumbitara	Proportion	2017	55.8	67.4	44.8
Colombia	Cumbitara	Count	2000	0	0	0

Table 4: ORS by Unit *(continued)*

Country	Unit	Metric	Year	Mean	Upper	Lower
Colombia	Cumbitara	Count	2017	1	1	0
Colombia	Gramalote	Proportion	2000	39.8	47.4	32.1
Colombia	Gramalote	Proportion	2017	51.0	63.4	39.5
Colombia	Gramalote	Count	2000	0	0	0
Colombia	Gramalote	Count	2017	1	1	0
Colombia	Cerrito	Proportion	2000	31.0	37.7	23.5
Colombia	Cerrito	Proportion	2017	44.2	57.7	32.7
Colombia	Cerrito	Count	2000	0	0	0
Colombia	Cerrito	Count	2017	0	1	0
Colombia	San Benito Abad	Proportion	2000	42.4	51.3	33.9
Colombia	San Benito Abad	Proportion	2017	58.1	68.2	47.5
Colombia	San Benito Abad	Count	2000	0	1	0
Colombia	San Benito Abad	Count	2017	1	1	0
Colombia	Espinal	Proportion	2000	31.6	38.9	24.8
Colombia	Espinal	Proportion	2017	47.5	58.6	36.5
Colombia	Espinal	Count	2000	0	0	0
Colombia	Espinal	Count	2017	0	1	0
Colombia	El Cerrito	Proportion	2000	36.5	43.5	29.8
Colombia	El Cerrito	Proportion	2017	55.1	66.7	43.8
Colombia	El Cerrito	Count	2000	0	0	0
Colombia	El Cerrito	Count	2017	1	1	0
Colombia	Bello	Proportion	2000	37.0	44.1	29.1
Colombia	Bello	Proportion	2017	51.2	63.8	39.0
Colombia	Bello	Count	2000	0	0	0
Colombia	Bello	Count	2017	1	1	0
Colombia	Sabanagrande	Proportion	2000	47.7	56.7	40.1
Colombia	Sabanagrande	Proportion	2017	65.7	74.9	55.2
Colombia	Sabanagrande	Count	2000	0	1	0
Colombia	Sabanagrande	Count	2017	1	1	1
Colombia	Mompós	Proportion	2000	43.3	51.1	34.9
Colombia	Mompós	Proportion	2017	55.9	67.3	44.1
Colombia	Mompós	Count	2000	0	1	0
Colombia	Mompós	Count	2017	1	1	0
Colombia	Chiquinquirá	Proportion	2000	29.8	35.8	23.1
Colombia	Chiquinquirá	Proportion	2017	50.2	63.9	38.6
Colombia	Chiquinquirá	Count	2000	0	0	0
Colombia	Chiquinquirá	Count	2017	1	1	0
Colombia	Palestina	Proportion	2000	34.5	40.9	28.2
Colombia	Palestina	Proportion	2017	57.4	69.4	47.0
Colombia	Palestina	Count	2000	0	0	0
Colombia	Palestina	Count	2017	1	1	0
Colombia	Tauramena	Proportion	2000	31.8	40.9	24.5
Colombia	Tauramena	Proportion	2017	44.9	56.7	32.3
Colombia	Tauramena	Count	2000	0	0	0
Colombia	Tauramena	Count	2017	0	1	0
Colombia	La Vega	Proportion	2000	30.9	38.5	24.3
Colombia	La Vega	Proportion	2017	50.0	61.0	37.5
Colombia	La Vega	Count	2000	0	0	0
Colombia	La Vega	Count	2017	0	1	0

Table 4: ORS by Unit *(continued)*

Country	Unit	Metric	Year	Mean	Upper	Lower
Colombia	Manaure	Proportion	2000	40.7	50.6	31.6
Colombia	Manaure	Proportion	2017	59.0	69.9	48.2
Colombia	Manaure	Count	2000	0	1	0
Colombia	Manaure	Count	2017	1	1	0
Colombia	Quibdó	Proportion	2000	31.4	40.4	24.0
Colombia	Quibdó	Proportion	2017	43.8	55.9	32.7
Colombia	Quibdó	Count	2000	0	0	0
Colombia	Quibdó	Count	2017	0	1	0
Colombia	Puerto Libertador	Proportion	2000	35.5	42.8	28.4
Colombia	Puerto Libertador	Proportion	2017	51.0	63.1	39.6
Colombia	Puerto Libertador	Count	2000	0	0	0
Colombia	Puerto Libertador	Count	2017	1	1	0
Colombia	Chaguaní	Proportion	2000	37.1	43.4	29.9
Colombia	Chaguaní	Proportion	2017	51.7	64.5	39.4
Colombia	Chaguaní	Count	2000	0	0	0
Colombia	Chaguaní	Count	2017	1	1	0
Colombia	La Argentina	Proportion	2000	33.7	41.3	26.2
Colombia	La Argentina	Proportion	2017	52.8	64.0	41.1
Colombia	La Argentina	Count	2000	0	0	0
Colombia	La Argentina	Count	2017	1	1	0
Colombia	San Sebastián de Buenavista	Proportion	2000	46.0	53.9	38.0
Colombia	San Sebastián de Buenavista	Proportion	2017	61.7	72.6	50.7
Colombia	San Sebastián de Buenavista	Count	2000	0	1	0
Colombia	San Sebastián de Buenavista	Count	2017	1	1	1
Colombia	Mesetas	Proportion	2000	30.9	40.1	22.9
Colombia	Mesetas	Proportion	2017	42.9	54.3	30.1
Colombia	Mesetas	Count	2000	0	0	0
Colombia	Mesetas	Count	2017	0	1	0
Colombia	El Charco	Proportion	2000	36.6	44.1	28.5
Colombia	El Charco	Proportion	2017	46.5	56.6	35.3
Colombia	El Charco	Count	2000	0	0	0
Colombia	El Charco	Count	2017	0	1	0
Colombia	Hacarí	Proportion	2000	41.7	50.2	33.1
Colombia	Hacarí	Proportion	2017	50.7	61.8	39.3
Colombia	Hacarí	Count	2000	0	1	0
Colombia	Hacarí	Count	2017	1	1	0
Colombia	Charalá	Proportion	2000	33.8	41.8	26.7
Colombia	Charalá	Proportion	2017	48.2	61.7	36.9
Colombia	Charalá	Count	2000	0	0	0
Colombia	Charalá	Count	2017	0	1	0
Colombia	San Juan de Betulia	Proportion	2000	43.5	52.3	34.8
Colombia	San Juan de Betulia	Proportion	2017	58.6	68.7	46.7
Colombia	San Juan de Betulia	Count	2000	0	1	0
Colombia	San Juan de Betulia	Count	2017	1	1	0
Colombia	Falán	Proportion	2000	35.0	42.2	28.3

Table 4: ORS by Unit *(continued)*

Country	Unit	Metric	Year	Mean	Upper	Lower
Colombia	Falán	Proportion	2017	48.8	60.9	38.4
Colombia	Falán	Count	2000	0	0	0
Colombia	Falán	Count	2017	0	1	0
Colombia	El Dovio	Proportion	2000	33.6	40.8	26.4
Colombia	El Dovio	Proportion	2017	49.1	62.4	38.8
Colombia	El Dovio	Count	2000	0	0	0
Colombia	El Dovio	Count	2017	0	1	0
Colombia	Belmira	Proportion	2000	34.1	41.1	26.2
Colombia	Belmira	Proportion	2017	45.2	59.2	33.4
Colombia	Belmira	Count	2000	0	0	0
Colombia	Belmira	Count	2017	0	1	0
Colombia	Sabanalarga	Proportion	2000	43.9	53.0	35.7
Colombia	Sabanalarga	Proportion	2017	56.1	66.0	45.5
Colombia	Sabanalarga	Count	2000	0	1	0
Colombia	Sabanalarga	Count	2017	1	1	0
Colombia	Montecristo	Proportion	2000	35.1	42.4	27.3
Colombia	Montecristo	Proportion	2017	43.9	54.5	33.3
Colombia	Montecristo	Count	2000	0	0	0
Colombia	Montecristo	Count	2017	0	1	0
Colombia	Chíquiza	Proportion	2000	29.7	37.0	23.5
Colombia	Chíquiza	Proportion	2017	50.2	63.8	38.6
Colombia	Chíquiza	Count	2000	0	0	0
Colombia	Chíquiza	Count	2017	1	1	0
Colombia	Pensilvania	Proportion	2000	34.1	41.7	27.0
Colombia	Pensilvania	Proportion	2017	49.2	60.5	39.3
Colombia	Pensilvania	Count	2000	0	0	0
Colombia	Pensilvania	Count	2017	0	1	0
Colombia	Trinidad	Proportion	2000	36.8	46.4	28.0
Colombia	Trinidad	Proportion	2017	48.3	59.1	35.4
Colombia	Trinidad	Count	2000	0	0	0
Colombia	Trinidad	Count	2017	0	1	0
Colombia	López de Micay	Proportion	2000	31.1	37.6	24.5
Colombia	López de Micay	Proportion	2017	42.1	52.4	31.2
Colombia	López de Micay	Count	2000	0	0	0
Colombia	López de Micay	Count	2017	0	1	0
Colombia	Pailitas	Proportion	2000	40.2	49.2	31.0
Colombia	Pailitas	Proportion	2017	51.4	62.4	40.9
Colombia	Pailitas	Count	2000	0	0	0
Colombia	Pailitas	Count	2017	1	1	0
Colombia	Riosucio	Proportion	2000	33.1	42.8	23.9
Colombia	Riosucio	Proportion	2017	42.4	53.6	31.7
Colombia	Riosucio	Count	2000	0	0	0
Colombia	Riosucio	Count	2017	0	1	0
Colombia	Purísima	Proportion	2000	39.5	47.8	31.5
Colombia	Purísima	Proportion	2017	60.5	70.7	49.4
Colombia	Purísima	Count	2000	0	0	0
Colombia	Purísima	Count	2017	1	1	0
Colombia	Chía	Proportion	2000	32.6	38.9	26.6
Colombia	Chía	Proportion	2017	47.7	61.0	35.2

Table 4: ORS by Unit *(continued)*

Country	Unit	Metric	Year	Mean	Upper	Lower
Colombia	Chía	Count	2000	0	0	0
Colombia	Chía	Count	2017	0	1	0
Colombia	La Plata	Proportion	2000	34.5	42.2	27.1
Colombia	La Plata	Proportion	2017	55.3	66.1	43.3
Colombia	La Plata	Count	2000	0	0	0
Colombia	La Plata	Count	2017	1	1	0
Colombia	San Zenón	Proportion	2000	50.6	58.8	41.6
Colombia	San Zenón	Proportion	2017	79.3	85.7	72.8
Colombia	San Zenón	Count	2000	1	1	0
Colombia	San Zenón	Count	2017	1	1	1
Colombia	Puerto Concordia	Proportion	2000	33.4	43.8	24.4
Colombia	Puerto Concordia	Proportion	2017	47.5	59.0	34.2
Colombia	Puerto Concordia	Count	2000	0	0	0
Colombia	Puerto Concordia	Count	2017	0	1	0
Colombia	El Rosario	Proportion	2000	38.1	47.3	29.1
Colombia	El Rosario	Proportion	2017	55.9	67.5	44.0
Colombia	El Rosario	Count	2000	0	0	0
Colombia	El Rosario	Count	2017	1	1	0
Colombia	Herrán	Proportion	2000	38.4	46.8	30.0
Colombia	Herrán	Proportion	2017	50.8	63.7	39.4
Colombia	Herrán	Count	2000	0	0	0
Colombia	Herrán	Count	2017	1	1	0
Colombia	Charta	Proportion	2000	34.9	42.6	28.1
Colombia	Charta	Proportion	2017	49.5	64.1	37.8
Colombia	Charta	Count	2000	0	0	0
Colombia	Charta	Count	2017	0	1	0
Colombia	San Marcos	Proportion	2000	41.3	51.0	32.7
Colombia	San Marcos	Proportion	2017	58.0	68.5	46.5
Colombia	San Marcos	Count	2000	0	1	0
Colombia	San Marcos	Count	2017	1	1	0
Colombia	Flandes	Proportion	2000	37.9	44.6	31.1
Colombia	Flandes	Proportion	2017	53.4	65.9	41.6
Colombia	Flandes	Count	2000	0	0	0
Colombia	Flandes	Count	2017	1	1	0
Colombia	Florida	Proportion	2000	35.4	43.1	28.7
Colombia	Florida	Proportion	2017	57.6	68.8	46.0
Colombia	Florida	Count	2000	0	0	0
Colombia	Florida	Count	2017	1	1	0
Colombia	Betania	Proportion	2000	35.3	43.7	27.5
Colombia	Betania	Proportion	2017	48.7	61.3	37.6
Colombia	Betania	Count	2000	0	0	0
Colombia	Betania	Count	2017	0	1	0
Colombia	Santa Lucía	Proportion	2000	46.1	55.0	38.6
Colombia	Santa Lucía	Proportion	2017	57.3	67.2	46.7
Colombia	Santa Lucía	Count	2000	0	1	0
Colombia	Santa Lucía	Count	2017	1	1	0
Colombia	Morales	Proportion	2000	40.9	48.4	32.2
Colombia	Morales	Proportion	2017	53.2	63.9	42.3
Colombia	Morales	Count	2000	0	0	0

Table 4: ORS by Unit *(continued)*

Country	Unit	Metric	Year	Mean	Upper	Lower
Colombia	Morales	Count	2017	1	1	0
Colombia	Chiscas	Proportion	2000	30.4	37.9	23.6
Colombia	Chiscas	Proportion	2017	44.7	58.4	33.7
Colombia	Chiscas	Count	2000	0	0	0
Colombia	Chiscas	Count	2017	0	1	0
Colombia	Riosucio	Proportion	2000	32.6	39.6	25.7
Colombia	Riosucio	Proportion	2017	49.8	61.8	39.6
Colombia	Riosucio	Count	2000	0	0	0
Colombia	Riosucio	Count	2017	0	1	0
Colombia	Villanueva	Proportion	2000	33.6	42.4	26.4
Colombia	Villanueva	Proportion	2017	48.8	61.4	36.4
Colombia	Villanueva	Count	2000	0	0	0
Colombia	Villanueva	Count	2017	0	1	0
Colombia	Mercaderes	Proportion	2000	37.2	45.5	29.3
Colombia	Mercaderes	Proportion	2017	52.0	63.8	39.6
Colombia	Mercaderes	Count	2000	0	0	0
Colombia	Mercaderes	Count	2017	1	1	0
Colombia	Pelaya	Proportion	2000	40.6	49.0	32.0
Colombia	Pelaya	Proportion	2017	53.5	64.1	42.3
Colombia	Pelaya	Count	2000	0	0	0
Colombia	Pelaya	Count	2017	1	1	0
Colombia	San José del Palmar	Proportion	2000	26.9	35.0	20.1
Colombia	San José del Palmar	Proportion	2017	40.6	54.5	30.1
Colombia	San José del Palmar	Count	2000	0	0	0
Colombia	San José del Palmar	Count	2017	0	1	0
Colombia	San Andrés de Sotavento	Proportion	2000	39.8	48.3	32.1
Colombia	San Andrés de Sotavento	Proportion	2017	61.8	72.0	50.7
Colombia	San Andrés de Sotavento	Count	2000	0	0	0
Colombia	San Andrés de Sotavento	Count	2017	1	1	1
Colombia	Chipaque	Proportion	2000	31.9	38.1	26.6
Colombia	Chipaque	Proportion	2017	48.5	61.5	36.3
Colombia	Chipaque	Count	2000	0	0	0
Colombia	Chipaque	Count	2017	0	1	0
Colombia	Nátaga	Proportion	2000	34.5	41.9	27.1
Colombia	Nátaga	Proportion	2017	56.3	67.3	43.6
Colombia	Nátaga	Count	2000	0	0	0
Colombia	Nátaga	Count	2017	1	1	0
Colombia	Santa Ana	Proportion	2000	45.0	53.4	37.2
Colombia	Santa Ana	Proportion	2017	60.4	71.4	48.3
Colombia	Santa Ana	Count	2000	0	1	0
Colombia	Santa Ana	Count	2017	1	1	0
Colombia	Puerto Gaitán	Proportion	2000	31.2	40.3	23.2
Colombia	Puerto Gaitán	Proportion	2017	43.7	54.6	32.8
Colombia	Puerto Gaitán	Count	2000	0	0	0
Colombia	Puerto Gaitán	Count	2017	0	1	0
Colombia	El Tablón de Gomez	Proportion	2000	36.0	45.0	27.6
Colombia	El Tablón de Gomez	Proportion	2017	55.0	66.6	43.5
Colombia	El Tablón de Gomez	Count	2000	0	0	0
Colombia	El Tablón de Gomez	Count	2017	1	1	0

Table 4: ORS by Unit *(continued)*

Country	Unit	Metric	Year	Mean	Upper	Lower
Colombia	La Esperanza	Proportion	2000	38.3	45.8	30.0
Colombia	La Esperanza	Proportion	2017	50.8	62.7	39.4
Colombia	La Esperanza	Count	2000	0	0	0
Colombia	La Esperanza	Count	2017	1	1	0
Colombia	Chimá	Proportion	2000	32.4	40.1	25.1
Colombia	Chimá	Proportion	2017	45.9	59.0	35.3
Colombia	Chimá	Count	2000	0	0	0
Colombia	Chimá	Count	2017	0	1	0
Colombia	San Onofre	Proportion	2000	45.1	53.9	36.3
Colombia	San Onofre	Proportion	2017	59.5	70.0	47.4
Colombia	San Onofre	Count	2000	0	1	0
Colombia	San Onofre	Count	2017	1	1	0
Colombia	Fresno	Proportion	2000	31.8	39.3	25.3
Colombia	Fresno	Proportion	2017	46.9	59.1	37.0
Colombia	Fresno	Count	2000	0	0	0
Colombia	Fresno	Count	2017	0	1	0
Colombia	Ginebra	Proportion	2000	35.8	42.6	29.1
Colombia	Ginebra	Proportion	2017	54.3	66.5	42.9
Colombia	Ginebra	Count	2000	0	0	0
Colombia	Ginebra	Count	2017	1	1	0
Colombia	Betulia	Proportion	2000	36.8	43.4	28.5
Colombia	Betulia	Proportion	2017	50.3	63.5	39.1
Colombia	Betulia	Count	2000	0	0	0
Colombia	Betulia	Count	2017	1	1	0
Colombia	Santo Tomás	Proportion	2000	45.8	55.3	37.6
Colombia	Santo Tomás	Proportion	2017	59.1	69.0	48.3
Colombia	Santo Tomás	Count	2000	0	1	0
Colombia	Santo Tomás	Count	2017	1	1	0
Colombia	Pinillos	Proportion	2000	40.5	48.3	31.7
Colombia	Pinillos	Proportion	2017	51.6	61.5	40.5
Colombia	Pinillos	Count	2000	0	0	0
Colombia	Pinillos	Count	2017	1	1	0
Colombia	Chita	Proportion	2000	30.1	37.8	23.9
Colombia	Chita	Proportion	2017	48.4	61.4	35.4
Colombia	Chita	Count	2000	0	0	0
Colombia	Chita	Count	2017	0	1	0
Colombia	Risaralda	Proportion	2000	33.7	40.2	27.5
Colombia	Risaralda	Proportion	2017	55.0	67.4	44.7
Colombia	Risaralda	Count	2000	0	0	0
Colombia	Risaralda	Count	2017	1	1	0
Colombia	Yopal	Proportion	2000	33.9	43.1	26.0
Colombia	Yopal	Proportion	2017	45.7	58.2	32.9
Colombia	Yopal	Count	2000	0	0	0
Colombia	Yopal	Count	2017	0	1	0
Colombia	Miranda	Proportion	2000	34.5	42.3	27.7
Colombia	Miranda	Proportion	2017	53.1	64.7	40.6
Colombia	Miranda	Count	2000	0	0	0
Colombia	Miranda	Count	2017	1	1	0
Colombia	Río de Oro	Proportion	2000	38.5	46.5	30.6

Table 4: ORS by Unit *(continued)*

Country	Unit	Metric	Year	Mean	Upper	Lower
Colombia	Río de Oro	Proportion	2017	51.6	63.7	40.4
Colombia	Río de Oro	Count	2000	0	0	0
Colombia	Río de Oro	Count	2017	1	1	0
Colombia	Sipí	Proportion	2000	28.4	35.6	21.3
Colombia	Sipí	Proportion	2017	39.4	52.1	29.2
Colombia	Sipí	Count	2000	0	0	0
Colombia	Sipí	Count	2017	0	1	0
Colombia	San Antero	Proportion	2000	42.1	50.3	34.3
Colombia	San Antero	Proportion	2017	59.0	70.0	47.8
Colombia	San Antero	Count	2000	0	1	0
Colombia	San Antero	Count	2017	1	1	0
Colombia	Choachí	Proportion	2000	31.1	37.1	25.6
Colombia	Choachí	Proportion	2017	50.1	62.7	38.3
Colombia	Choachí	Count	2000	0	0	0
Colombia	Choachí	Count	2017	1	1	0
Colombia	Neiva	Proportion	2000	38.4	46.1	30.1
Colombia	Neiva	Proportion	2017	59.3	71.0	47.0
Colombia	Neiva	Count	2000	0	0	0
Colombia	Neiva	Count	2017	1	1	0
Colombia	Santa Marta (Dist. Esp.)	Proportion	2000	45.2	53.1	37.3
Colombia	Santa Marta (Dist. Esp.)	Proportion	2017	62.9	72.8	51.4
Colombia	Santa Marta (Dist. Esp.)	Count	2000	0	1	0
Colombia	Santa Marta (Dist. Esp.)	Count	2017	1	1	1
Colombia	Puerto Lleras	Proportion	2000	31.1	40.2	24.0
Colombia	Puerto Lleras	Proportion	2017	44.7	56.0	33.3
Colombia	Puerto Lleras	Count	2000	0	0	0
Colombia	Puerto Lleras	Count	2017	0	1	0
Colombia	El Tambo	Proportion	2000	38.6	47.9	30.2
Colombia	El Tambo	Proportion	2017	55.5	67.2	43.9
Colombia	El Tambo	Count	2000	0	0	0
Colombia	El Tambo	Count	2017	1	1	0
Colombia	La Playa de Belén	Proportion	2000	40.6	48.6	32.7
Colombia	La Playa de Belén	Proportion	2017	52.1	63.8	40.1
Colombia	La Playa de Belén	Count	2000	0	0	0
Colombia	La Playa de Belén	Count	2017	1	1	0
Colombia	Chipatá	Proportion	2000	32.9	40.7	25.3
Colombia	Chipatá	Proportion	2017	49.8	63.1	38.3
Colombia	Chipatá	Count	2000	0	0	0
Colombia	Chipatá	Count	2017	0	1	0
Colombia	San Pedro	Proportion	2000	44.2	53.3	36.3
Colombia	San Pedro	Proportion	2017	58.7	68.9	47.7
Colombia	San Pedro	Count	2000	0	1	0
Colombia	San Pedro	Count	2017	1	1	0
Colombia	Guamo	Proportion	2000	36.4	43.5	29.2
Colombia	Guamo	Proportion	2017	53.6	64.6	42.4
Colombia	Guamo	Count	2000	0	0	0
Colombia	Guamo	Count	2017	1	1	0
Colombia	Guacarí	Proportion	2000	40.3	47.3	33.1
Colombia	Guacarí	Proportion	2017	55.6	68.0	44.4

Table 4: ORS by Unit *(continued)*

Country	Unit	Metric	Year	Mean	Upper	Lower
Colombia	Guacarí	Count	2000	0	0	0
Colombia	Guacarí	Count	2017	1	1	0
Colombia	Bolívar	Proportion	2000	33.4	41.5	25.9
Colombia	Bolívar	Proportion	2017	46.3	59.0	34.5
Colombia	Bolívar	Count	2000	0	0	0
Colombia	Bolívar	Count	2017	0	1	0
Colombia	Soledad	Proportion	2000	52.1	59.7	44.5
Colombia	Soledad	Proportion	2017	64.3	72.5	54.4
Colombia	Soledad	Count	2000	1	1	0
Colombia	Soledad	Count	2017	1	1	1
Colombia	Río Viejo	Proportion	2000	42.2	50.3	33.6
Colombia	Río Viejo	Proportion	2017	54.1	65.3	42.8
Colombia	Río Viejo	Count	2000	0	1	0
Colombia	Río Viejo	Count	2017	1	1	0
Colombia	Chitaraque	Proportion	2000	32.2	39.9	24.7
Colombia	Chitaraque	Proportion	2017	50.6	64.6	39.2
Colombia	Chitaraque	Count	2000	0	0	0
Colombia	Chitaraque	Count	2017	1	1	0
Colombia	Salamina	Proportion	2000	34.6	41.1	27.3
Colombia	Salamina	Proportion	2017	50.5	61.5	40.1
Colombia	Salamina	Count	2000	0	0	0
Colombia	Salamina	Count	2017	1	1	0
Colombia	Morales	Proportion	2000	30.4	37.2	23.7
Colombia	Morales	Proportion	2017	51.1	62.2	39.9
Colombia	Morales	Count	2000	0	0	0
Colombia	Morales	Count	2017	1	1	0
Colombia	San Alberto	Proportion	2000	38.8	46.8	30.9
Colombia	San Alberto	Proportion	2017	51.9	64.0	40.8
Colombia	San Alberto	Count	2000	0	0	0
Colombia	San Alberto	Count	2017	1	1	0
Colombia	Tadó	Proportion	2000	30.7	39.7	23.3
Colombia	Tadó	Proportion	2017	43.7	56.3	32.2
Colombia	Tadó	Count	2000	0	0	0
Colombia	Tadó	Count	2017	0	1	0
Colombia	San Bernardino de Sahagún	Proportion	2000	38.6	47.4	30.6
Colombia	San Bernardino de Sahagún	Proportion	2017	59.8	70.2	48.9
Colombia	San Bernardino de Sahagún	Count	2000	0	0	0
Colombia	San Bernardino de Sahagún	Count	2017	1	1	0
Colombia	Chocontá	Proportion	2000	28.8	35.0	22.6
Colombia	Chocontá	Proportion	2017	45.6	60.8	33.9
Colombia	Chocontá	Count	2000	0	0	0
Colombia	Chocontá	Count	2017	0	1	0
Colombia	Oporapa	Proportion	2000	33.5	41.3	26.0
Colombia	Oporapa	Proportion	2017	54.5	65.7	42.2
Colombia	Oporapa	Count	2000	0	0	0
Colombia	Oporapa	Count	2017	1	1	0

Table 4: ORS by Unit *(continued)*

Country	Unit	Metric	Year	Mean	Upper	Lower
Colombia	Tenerife	Proportion	2000	46.6	54.8	38.3
Colombia	Tenerife	Proportion	2017	61.4	71.4	50.7
Colombia	Tenerife	Count	2000	0	1	0
Colombia	Tenerife	Count	2017	1	1	1
Colombia	Puerto López	Proportion	2000	31.1	39.7	23.7
Colombia	Puerto López	Proportion	2017	46.0	57.7	35.2
Colombia	Puerto López	Count	2000	0	0	0
Colombia	Puerto López	Count	2017	0	1	0
Colombia	Francisco Pizarro	Proportion	2000	35.8	45.0	27.1
Colombia	Francisco Pizarro	Proportion	2017	44.0	55.1	32.4
Colombia	Francisco Pizarro	Count	2000	0	0	0
Colombia	Francisco Pizarro	Count	2017	0	1	0
Colombia	Labateca	Proportion	2000	36.0	43.6	28.5
Colombia	Labateca	Proportion	2017	47.9	60.3	36.5
Colombia	Labateca	Count	2000	0	0	0
Colombia	Labateca	Count	2017	0	1	0
Colombia	Cimitarra	Proportion	2000	37.2	45.0	29.1
Colombia	Cimitarra	Proportion	2017	54.1	66.0	43.5
Colombia	Cimitarra	Count	2000	0	0	0
Colombia	Cimitarra	Count	2017	1	1	0
Colombia	Sincé	Proportion	2000	45.4	54.1	37.3
Colombia	Sincé	Proportion	2017	60.1	70.3	48.9
Colombia	Sincé	Count	2000	0	1	0
Colombia	Sincé	Count	2017	1	1	0
Colombia	Herveo	Proportion	2000	28.1	34.8	22.4
Colombia	Herveo	Proportion	2017	42.2	54.4	32.4
Colombia	Herveo	Count	2000	0	0	0
Colombia	Herveo	Count	2017	0	1	0
Colombia	Guadalajara de Buga	Proportion	2000	38.8	46.3	31.8
Colombia	Guadalajara de Buga	Proportion	2017	53.3	66.4	42.1
Colombia	Guadalajara de Buga	Count	2000	0	0	0
Colombia	Guadalajara de Buga	Count	2017	1	1	0
Colombia	Briceño	Proportion	2000	36.8	44.5	28.9
Colombia	Briceño	Proportion	2017	51.6	63.8	40.8
Colombia	Briceño	Count	2000	0	0	0
Colombia	Briceño	Count	2017	1	1	0
Colombia	Suan	Proportion	2000	51.2	59.7	43.5
Colombia	Suan	Proportion	2017	58.5	68.8	47.8
Colombia	Suan	Count	2000	1	1	0
Colombia	Suan	Count	2017	1	1	0
Colombia	San Estanislao de Kostka	Proportion	2000	46.5	56.1	39.0
Colombia	San Estanislao de Kostka	Proportion	2017	57.8	68.1	47.2
Colombia	San Estanislao de Kostka	Count	2000	0	1	0
Colombia	San Estanislao de Kostka	Count	2017	1	1	0
Colombia	Chivatá	Proportion	2000	27.1	34.8	21.0
Colombia	Chivatá	Proportion	2017	46.2	61.7	34.1
Colombia	Chivatá	Count	2000	0	0	0
Colombia	Chivatá	Count	2017	0	1	0
Colombia	Samaná	Proportion	2000	35.5	43.0	28.6

Table 4: ORS by Unit *(continued)*

Country	Unit	Metric	Year	Mean	Upper	Lower
Colombia	Samaná	Proportion	2017	52.4	64.0	41.8
Colombia	Samaná	Count	2000	0	0	0
Colombia	Samaná	Count	2017	1	1	0
Colombia	Padilla	Proportion	2000	33.6	41.0	27.3
Colombia	Padilla	Proportion	2017	52.4	63.7	40.3
Colombia	Padilla	Count	2000	0	0	0
Colombia	Padilla	Count	2017	1	1	0
Colombia	San Diego	Proportion	2000	43.4	52.6	34.3
Colombia	San Diego	Proportion	2017	59.0	69.8	47.8
Colombia	San Diego	Count	2000	0	1	0
Colombia	San Diego	Count	2017	1	1	0
Colombia	Unguía	Proportion	2000	34.6	44.7	25.6
Colombia	Unguía	Proportion	2017	46.6	58.0	36.1
Colombia	Unguía	Count	2000	0	0	0
Colombia	Unguía	Count	2017	0	1	0
Colombia	San Bernardo del Viento	Proportion	2000	39.9	48.6	31.6
Colombia	San Bernardo del Viento	Proportion	2017	58.6	69.0	46.8
Colombia	San Bernardo del Viento	Count	2000	0	0	0
Colombia	San Bernardo del Viento	Count	2017	1	1	0
Colombia	Cogua	Proportion	2000	30.0	35.6	24.2
Colombia	Cogua	Proportion	2017	45.1	60.3	33.1
Colombia	Cogua	Count	2000	0	0	0
Colombia	Cogua	Count	2017	0	1	0
Colombia	Paicol	Proportion	2000	36.0	44.1	27.9
Colombia	Paicol	Proportion	2017	53.7	64.9	40.9
Colombia	Paicol	Count	2000	0	0	0
Colombia	Paicol	Count	2017	1	1	0
Colombia	Puerto Rico	Proportion	2000	31.2	40.6	22.5
Colombia	Puerto Rico	Proportion	2017	44.6	56.2	33.3
Colombia	Puerto Rico	Count	2000	0	0	0
Colombia	Puerto Rico	Count	2017	0	1	0
Colombia	Funes	Proportion	2000	36.3	46.2	27.3
Colombia	Funes	Proportion	2017	53.1	64.6	42.5
Colombia	Funes	Count	2000	0	0	0
Colombia	Funes	Count	2017	1	1	0
Colombia	Los Patios	Proportion	2000	38.9	47.1	30.0
Colombia	Los Patios	Proportion	2017	56.7	69.3	45.1
Colombia	Los Patios	Count	2000	0	0	0
Colombia	Los Patios	Count	2017	1	1	0
Colombia	Concepción	Proportion	2000	33.5	40.8	26.2
Colombia	Concepción	Proportion	2017	47.9	62.4	36.1
Colombia	Concepción	Count	2000	0	0	0
Colombia	Concepción	Count	2017	0	1	0
Colombia	Sincelejo	Proportion	2000	45.0	53.7	36.7
Colombia	Sincelejo	Proportion	2017	58.6	69.4	46.4
Colombia	Sincelejo	Count	2000	0	1	0
Colombia	Sincelejo	Count	2017	1	1	0
Colombia	Honda	Proportion	2000	35.5	42.2	28.3
Colombia	Honda	Proportion	2017	52.8	65.0	41.7

Table 4: ORS by Unit *(continued)*

Country	Unit	Metric	Year	Mean	Upper	Lower
Colombia	Honda	Count	2000	0	0	0
Colombia	Honda	Count	2017	1	1	0
Colombia	Jamundí	Proportion	2000	34.7	42.0	28.2
Colombia	Jamundí	Proportion	2017	52.7	63.6	41.1
Colombia	Jamundí	Count	2000	0	0	0
Colombia	Jamundí	Count	2017	1	1	0
Colombia	Buriticá	Proportion	2000	36.4	43.8	28.3
Colombia	Buriticá	Proportion	2017	50.8	63.8	37.8
Colombia	Buriticá	Count	2000	0	0	0
Colombia	Buriticá	Count	2017	1	1	0
Colombia	Tubará	Proportion	2000	43.9	52.3	36.3
Colombia	Tubará	Proportion	2017	54.0	64.3	43.0
Colombia	Tubará	Count	2000	0	1	0
Colombia	Tubará	Count	2017	1	1	0
Colombia	San Fernando	Proportion	2000	48.3	56.3	39.2
Colombia	San Fernando	Proportion	2017	62.7	71.8	52.9
Colombia	San Fernando	Count	2000	0	1	0
Colombia	San Fernando	Count	2017	1	1	1
Colombia	Chivor	Proportion	2000	31.2	38.1	25.0
Colombia	Chivor	Proportion	2017	44.6	57.7	32.5
Colombia	Chivor	Count	2000	0	0	0
Colombia	Chivor	Count	2017	0	1	0
Colombia	Supía	Proportion	2000	36.3	43.7	28.9
Colombia	Supía	Proportion	2017	56.6	68.2	46.3
Colombia	Supía	Count	2000	0	0	0
Colombia	Supía	Count	2017	1	1	0
Colombia	Páez	Proportion	2000	31.1	38.0	24.1
Colombia	Páez	Proportion	2017	51.1	61.8	39.0
Colombia	Páez	Count	2000	0	0	0
Colombia	Páez	Count	2017	1	1	0
Colombia	San Martín	Proportion	2000	39.2	47.3	30.8
Colombia	San Martín	Proportion	2017	51.4	63.9	40.3
Colombia	San Martín	Count	2000	0	0	0
Colombia	San Martín	Count	2017	1	1	0
Colombia	San Carlos	Proportion	2000	39.7	48.8	32.1
Colombia	San Carlos	Proportion	2017	58.7	69.6	47.5
Colombia	San Carlos	Count	2000	0	0	0
Colombia	San Carlos	Count	2017	1	1	0
Colombia	Cota	Proportion	2000	33.4	39.4	27.7
Colombia	Cota	Proportion	2017	54.9	66.2	43.1
Colombia	Cota	Count	2000	0	0	0
Colombia	Cota	Count	2017	1	1	0
Colombia	Palermo	Proportion	2000	37.3	45.0	29.3
Colombia	Palermo	Proportion	2017	54.3	65.8	41.1
Colombia	Palermo	Count	2000	0	0	0
Colombia	Palermo	Count	2017	1	1	0
Colombia	Restrepo	Proportion	2000	28.9	36.1	22.7
Colombia	Restrepo	Proportion	2017	42.9	54.9	32.2
Colombia	Restrepo	Count	2000	0	0	0

Table 4: ORS by Unit *(continued)*

Country	Unit	Metric	Year	Mean	Upper	Lower
Colombia	Restrepo	Count	2017	0	1	0
Colombia	Guachucal	Proportion	2000	35.6	46.0	26.4
Colombia	Guachucal	Proportion	2017	54.6	66.0	43.9
Colombia	Guachucal	Count	2000	0	0	0
Colombia	Guachucal	Count	2017	1	1	0
Colombia	Lourdes	Proportion	2000	39.9	48.0	32.3
Colombia	Lourdes	Proportion	2017	52.3	64.7	41.0
Colombia	Lourdes	Count	2000	0	0	0
Colombia	Lourdes	Count	2017	1	1	0
Colombia	Confines	Proportion	2000	33.5	41.6	25.8
Colombia	Confines	Proportion	2017	48.7	61.6	37.9
Colombia	Confines	Count	2000	0	0	0
Colombia	Confines	Count	2017	0	1	0
Colombia	Sucre	Proportion	2000	41.2	49.8	32.1
Colombia	Sucre	Proportion	2017	55.4	65.5	44.2
Colombia	Sucre	Count	2000	0	0	0
Colombia	Sucre	Count	2017	1	1	0
Colombia	Ibagué	Proportion	2000	34.7	41.0	27.6
Colombia	Ibagué	Proportion	2017	52.8	64.6	41.6
Colombia	Ibagué	Count	2000	0	0	0
Colombia	Ibagué	Count	2017	1	1	0
Colombia	La Cumbre	Proportion	2000	34.8	41.8	28.9
Colombia	La Cumbre	Proportion	2017	50.4	62.8	39.5
Colombia	La Cumbre	Count	2000	0	0	0
Colombia	La Cumbre	Count	2017	1	1	0
Colombia	Cáceres	Proportion	2000	38.5	45.9	30.8
Colombia	Cáceres	Proportion	2017	52.4	63.3	42.1
Colombia	Cáceres	Count	2000	0	0	0
Colombia	Cáceres	Count	2017	1	1	0
Colombia	Usiacurí	Proportion	2000	46.4	55.5	38.0
Colombia	Usiacurí	Proportion	2017	55.5	65.7	44.4
Colombia	Usiacurí	Count	2000	0	1	0
Colombia	Usiacurí	Count	2017	1	1	0
Colombia	San Jacinto	Proportion	2000	43.9	51.9	35.0
Colombia	San Jacinto	Proportion	2017	56.5	67.3	45.0
Colombia	San Jacinto	Count	2000	0	1	0
Colombia	San Jacinto	Count	2017	1	1	0
Colombia	Ciénaga	Proportion	2000	27.1	34.5	20.8
Colombia	Ciénaga	Proportion	2017	45.8	60.7	33.7
Colombia	Ciénaga	Count	2000	0	0	0
Colombia	Ciénaga	Count	2017	0	1	0
Colombia	Victoria	Proportion	2000	36.6	43.4	29.2
Colombia	Victoria	Proportion	2017	51.9	63.8	40.9
Colombia	Victoria	Count	2000	0	0	0
Colombia	Victoria	Count	2017	1	1	0
Colombia	Patía	Proportion	2000	34.1	42.0	27.1
Colombia	Patía	Proportion	2017	51.4	62.9	38.9
Colombia	Patía	Count	2000	0	0	0
Colombia	Patía	Count	2017	1	1	0

Table 4: ORS by Unit *(continued)*

Country	Unit	Metric	Year	Mean	Upper	Lower
Colombia	Tamalameque	Proportion	2000	40.9	49.8	32.0
Colombia	Tamalameque	Proportion	2017	54.5	65.3	43.4
Colombia	Tamalameque	Count	2000	0	0	0
Colombia	Tamalameque	Count	2017	1	1	0
Colombia	San Pelayo	Proportion	2000	41.0	50.7	33.6
Colombia	San Pelayo	Proportion	2017	60.1	71.0	48.8
Colombia	San Pelayo	Count	2000	0	1	0
Colombia	San Pelayo	Count	2017	1	1	0
Colombia	Cucunubá	Proportion	2000	27.7	33.4	21.8
Colombia	Cucunubá	Proportion	2017	43.4	59.4	31.1
Colombia	Cucunubá	Count	2000	0	0	0
Colombia	Cucunubá	Count	2017	0	1	0
Colombia	Palestina	Proportion	2000	35.8	44.9	26.9
Colombia	Palestina	Proportion	2017	51.5	62.1	39.6
Colombia	Palestina	Count	2000	0	0	0
Colombia	Palestina	Count	2017	1	1	0
Colombia	San Carlos de Guaroa	Proportion	2000	31.3	40.3	24.2
Colombia	San Carlos de Guaroa	Proportion	2017	46.1	57.8	35.2
Colombia	San Carlos de Guaroa	Count	2000	0	0	0
Colombia	San Carlos de Guaroa	Count	2017	0	1	0
Colombia	Guaitarilla	Proportion	2000	35.7	45.3	27.3
Colombia	Guaitarilla	Proportion	2017	57.4	68.5	46.0
Colombia	Guaitarilla	Count	2000	0	0	0
Colombia	Guaitarilla	Count	2017	1	1	0
Colombia	Mutiscua	Proportion	2000	34.4	41.9	27.1
Colombia	Mutiscua	Proportion	2017	48.4	61.0	37.1
Colombia	Mutiscua	Count	2000	0	0	0
Colombia	Mutiscua	Count	2017	0	1	0
Colombia	Contratación	Proportion	2000	31.6	39.7	24.4
Colombia	Contratación	Proportion	2017	45.7	58.8	34.8
Colombia	Contratación	Count	2000	0	0	0
Colombia	Contratación	Count	2017	0	1	0
Colombia	Tolú	Proportion	2000	42.3	51.2	34.2
Colombia	Tolú	Proportion	2017	59.0	70.3	46.8
Colombia	Tolú	Count	2000	0	1	0
Colombia	Tolú	Count	2017	1	1	0
Colombia	Icononzo	Proportion	2000	34.6	41.4	27.9
Colombia	Icononzo	Proportion	2017	50.8	62.2	39.3
Colombia	Icononzo	Count	2000	0	0	0
Colombia	Icononzo	Count	2017	1	1	0
Colombia	La Unión de Sucre	Proportion	2000	38.9	46.1	31.3
Colombia	La Unión de Sucre	Proportion	2017	60.7	73.7	49.3
Colombia	La Unión de Sucre	Count	2000	0	0	0
Colombia	La Unión de Sucre	Count	2017	1	1	0
Colombia	Caicedo	Proportion	2000	35.9	42.6	28.2
Colombia	Caicedo	Proportion	2017	48.0	62.0	35.9
Colombia	Caicedo	Count	2000	0	0	0
Colombia	Caicedo	Count	2017	0	1	0
Colombia	San Juan Nepomuceno	Proportion	2000	43.0	51.3	33.7

Table 4: ORS by Unit *(continued)*

Country	Unit	Metric	Year	Mean	Upper	Lower
Colombia	San Juan Nepomuceno	Proportion	2017	53.5	64.2	41.6
Colombia	San Juan Nepomuceno	Count	2000	0	1	0
Colombia	San Juan Nepomuceno	Count	2017	1	1	0
Colombia	Cómbita	Proportion	2000	29.0	36.8	22.5
Colombia	Cómbita	Proportion	2017	55.3	69.2	43.6
Colombia	Cómbita	Count	2000	0	0	0
Colombia	Cómbita	Count	2017	1	1	0
Colombia	Villamaría	Proportion	2000	35.4	42.8	28.5
Colombia	Villamaría	Proportion	2017	56.1	67.6	45.2
Colombia	Villamaría	Count	2000	0	0	0
Colombia	Villamaría	Count	2017	1	1	0
Colombia	Piendamó	Proportion	2000	28.9	35.9	22.0
Colombia	Piendamó	Proportion	2017	52.3	63.6	41.2
Colombia	Piendamó	Count	2000	0	0	0
Colombia	Piendamó	Count	2017	1	1	0
Colombia	Valledupar	Proportion	2000	41.4	49.6	32.7
Colombia	Valledupar	Proportion	2017	56.9	67.6	46.2
Colombia	Valledupar	Count	2000	0	0	0
Colombia	Valledupar	Count	2017	1	1	0
Colombia	Santa Cruz de Lorica	Proportion	2000	40.6	49.7	32.2
Colombia	Santa Cruz de Lorica	Proportion	2017	59.0	69.8	47.1
Colombia	Santa Cruz de Lorica	Count	2000	0	0	0
Colombia	Santa Cruz de Lorica	Count	2017	1	1	0
Colombia	El Colegio	Proportion	2000	34.6	40.7	29.2
Colombia	El Colegio	Proportion	2017	51.6	63.0	39.6
Colombia	El Colegio	Count	2000	0	0	0
Colombia	El Colegio	Count	2017	1	1	0
Colombia	Pital	Proportion	2000	34.1	41.3	26.7
Colombia	Pital	Proportion	2017	53.2	64.7	40.3
Colombia	Pital	Count	2000	0	0	0
Colombia	Pital	Count	2017	1	1	0
Colombia	San Juan de Arama	Proportion	2000	32.5	41.4	24.2
Colombia	San Juan de Arama	Proportion	2017	44.8	55.6	32.8
Colombia	San Juan de Arama	Count	2000	0	0	0
Colombia	San Juan de Arama	Count	2017	0	1	0
Colombia	Gualmatán	Proportion	2000	36.9	46.7	27.9
Colombia	Gualmatán	Proportion	2017	56.2	67.0	44.9
Colombia	Gualmatán	Count	2000	0	0	0
Colombia	Gualmatán	Count	2017	1	1	0
Colombia	Ocaña	Proportion	2000	38.1	45.7	30.8
Colombia	Ocaña	Proportion	2017	53.8	65.6	42.6
Colombia	Ocaña	Count	2000	0	0	0
Colombia	Ocaña	Count	2017	1	1	0
Colombia	Coromoro	Proportion	2000	32.6	39.5	26.0
Colombia	Coromoro	Proportion	2017	47.8	60.9	36.3
Colombia	Coromoro	Count	2000	0	0	0
Colombia	Coromoro	Count	2017	0	1	0
Colombia	Toluviejo	Proportion	2000	44.3	52.9	36.5
Colombia	Toluviejo	Proportion	2017	58.2	69.6	45.9

Table 4: ORS by Unit *(continued)*

Country	Unit	Metric	Year	Mean	Upper	Lower
Colombia	Toluviejo	Count	2000	0	1	0
Colombia	Toluviejo	Count	2017	1	1	0
Colombia	Lérida	Proportion	2000	38.4	45.5	31.7
Colombia	Lérida	Proportion	2017	54.6	67.3	43.2
Colombia	Lérida	Count	2000	0	0	0
Colombia	Lérida	Count	2017	1	1	0
Colombia	La Victoria	Proportion	2000	36.9	44.0	29.9
Colombia	La Victoria	Proportion	2017	56.4	69.8	45.5
Colombia	La Victoria	Count	2000	0	0	0
Colombia	La Victoria	Count	2017	1	1	0
Colombia	Caldas	Proportion	2000	35.8	42.8	28.6
Colombia	Caldas	Proportion	2017	53.3	65.5	42.0
Colombia	Caldas	Count	2000	0	0	0
Colombia	Caldas	Count	2017	1	1	0
Colombia	San Martín de Loba	Proportion	2000	42.6	50.9	33.9
Colombia	San Martín de Loba	Proportion	2017	53.4	64.7	41.4
Colombia	San Martín de Loba	Count	2000	0	1	0
Colombia	San Martín de Loba	Count	2017	1	1	0
Colombia	Coper	Proportion	2000	32.3	38.0	26.0
Colombia	Coper	Proportion	2017	47.5	61.6	36.6
Colombia	Coper	Count	2000	0	0	0
Colombia	Coper	Count	2017	0	1	0
Colombia	Viterbo	Proportion	2000	33.5	40.4	26.6
Colombia	Viterbo	Proportion	2017	51.6	64.5	41.1
Colombia	Viterbo	Count	2000	0	0	0
Colombia	Viterbo	Count	2017	1	1	0
Colombia	Popayán	Proportion	2000	31.6	39.1	24.3
Colombia	Popayán	Proportion	2017	54.4	65.9	42.4
Colombia	Popayán	Count	2000	0	0	0
Colombia	Popayán	Count	2017	1	1	0
Colombia	Tierralta	Proportion	2000	36.4	43.4	29.4
Colombia	Tierralta	Proportion	2017	52.6	63.8	41.1
Colombia	Tierralta	Count	2000	0	0	0
Colombia	Tierralta	Count	2017	1	1	0
Colombia	El Peñon	Proportion	2000	34.6	39.8	27.8
Colombia	El Peñon	Proportion	2017	49.5	62.5	38.4
Colombia	El Peñon	Count	2000	0	0	0
Colombia	El Peñon	Count	2017	0	1	0
Colombia	Pitalito	Proportion	2000	35.4	44.1	26.9
Colombia	Pitalito	Proportion	2017	53.4	64.6	41.1
Colombia	Pitalito	Count	2000	0	0	0
Colombia	Pitalito	Count	2017	1	1	0
Colombia	San Juanito	Proportion	2000	29.1	35.9	23.2
Colombia	San Juanito	Proportion	2017	42.9	55.9	31.4
Colombia	San Juanito	Count	2000	0	0	0
Colombia	San Juanito	Count	2017	0	1	0
Colombia	Iles	Proportion	2000	36.9	46.9	27.9
Colombia	Iles	Proportion	2017	57.4	68.3	46.0
Colombia	Iles	Count	2000	0	0	0

Table 4: ORS by Unit *(continued)*

Country	Unit	Metric	Year	Mean	Upper	Lower
Colombia	Iles	Count	2017	1	1	0
Colombia	Pamplona	Proportion	2000	37.0	44.9	29.2
Colombia	Pamplona	Proportion	2017	53.2	66.1	41.6
Colombia	Pamplona	Count	2000	0	0	0
Colombia	Pamplona	Count	2017	1	1	0
Colombia	Curití	Proportion	2000	36.4	43.6	29.0
Colombia	Curití	Proportion	2017	55.4	67.6	43.7
Colombia	Curití	Count	2000	0	0	0
Colombia	Curití	Count	2017	1	1	0
Colombia	Líbano	Proportion	2000	32.3	39.1	26.2
Colombia	Líbano	Proportion	2017	47.2	60.0	36.3
Colombia	Líbano	Count	2000	0	0	0
Colombia	Líbano	Count	2017	0	1	0
Colombia	Obando	Proportion	2000	35.8	43.0	29.2
Colombia	Obando	Proportion	2017	55.4	69.5	44.1
Colombia	Obando	Count	2000	0	0	0
Colombia	Obando	Count	2017	1	1	0
Colombia	Campamento	Proportion	2000	34.7	41.8	27.0
Colombia	Campamento	Proportion	2017	49.5	60.9	38.4
Colombia	Campamento	Count	2000	0	0	0
Colombia	Campamento	Count	2017	0	1	0
Colombia	San Pablo	Proportion	2000	39.0	45.9	30.9
Colombia	San Pablo	Proportion	2017	50.3	62.0	38.8
Colombia	San Pablo	Count	2000	0	0	0
Colombia	San Pablo	Count	2017	1	1	0
Colombia	Corrales	Proportion	2000	29.2	36.9	22.7
Colombia	Corrales	Proportion	2017	48.9	61.7	36.6
Colombia	Corrales	Count	2000	0	0	0
Colombia	Corrales	Count	2017	0	1	0
Colombia	Puerto Tejada	Proportion	2000	35.3	42.8	29.2
Colombia	Puerto Tejada	Proportion	2017	55.2	66.2	43.8
Colombia	Puerto Tejada	Count	2000	0	0	0
Colombia	Puerto Tejada	Count	2017	1	1	0
Colombia	Valencia	Proportion	2000	36.0	43.7	28.7
Colombia	Valencia	Proportion	2017	55.5	66.9	43.3
Colombia	Valencia	Count	2000	0	0	0
Colombia	Valencia	Count	2017	1	1	0
Colombia	Facatativá	Proportion	2000	32.7	38.2	26.8
Colombia	Facatativá	Proportion	2017	50.3	63.1	38.2
Colombia	Facatativá	Count	2000	0	0	0
Colombia	Facatativá	Count	2017	1	1	0
Colombia	Rivera	Proportion	2000	36.5	44.5	27.8
Colombia	Rivera	Proportion	2017	54.4	66.8	41.0
Colombia	Rivera	Count	2000	0	0	0
Colombia	Rivera	Count	2017	1	1	0
Colombia	San Luis de Cubarral	Proportion	2000	29.2	37.0	23.2
Colombia	San Luis de Cubarral	Proportion	2017	43.1	54.5	31.4
Colombia	San Luis de Cubarral	Count	2000	0	0	0
Colombia	San Luis de Cubarral	Count	2017	0	1	0

Table 4: ORS by Unit *(continued)*

Country	Unit	Metric	Year	Mean	Upper	Lower
Colombia	Imués	Proportion	2000	36.2	46.0	27.5
Colombia	Imués	Proportion	2017	56.7	68.3	44.9
Colombia	Imués	Count	2000	0	0	0
Colombia	Imués	Count	2017	1	1	0
Colombia	Pamplonita	Proportion	2000	35.5	43.5	27.9
Colombia	Pamplonita	Proportion	2017	50.6	63.3	38.8
Colombia	Pamplonita	Count	2000	0	0	0
Colombia	Pamplonita	Count	2017	1	1	0
Colombia	El Carmen de Chucurí	Proportion	2000	36.0	44.0	28.2
Colombia	El Carmen de Chucurí	Proportion	2017	51.5	64.1	40.1
Colombia	El Carmen de Chucurí	Count	2000	0	0	0
Colombia	El Carmen de Chucurí	Count	2017	1	1	0
Colombia	Melgar	Proportion	2000	37.7	44.3	31.2
Colombia	Melgar	Proportion	2017	57.5	68.5	45.1
Colombia	Melgar	Count	2000	0	0	0
Colombia	Melgar	Count	2017	1	1	0
Colombia	Palmira	Proportion	2000	36.2	43.2	29.8
Colombia	Palmira	Proportion	2017	51.6	63.3	40.6
Colombia	Palmira	Count	2000	0	0	0
Colombia	Palmira	Count	2017	1	1	0
Colombia	Cañasgordas	Proportion	2000	37.4	45.3	28.8
Colombia	Cañasgordas	Proportion	2017	53.4	64.9	41.2
Colombia	Cañasgordas	Count	2000	0	0	0
Colombia	Cañasgordas	Count	2017	1	1	0
Colombia	Santa Catalina	Proportion	2000	44.9	53.6	36.5
Colombia	Santa Catalina	Proportion	2017	56.1	66.3	45.0
Colombia	Santa Catalina	Count	2000	0	1	0
Colombia	Santa Catalina	Count	2017	1	1	0
Colombia	Covarachía	Proportion	2000	32.3	39.5	25.6
Colombia	Covarachía	Proportion	2017	49.4	63.0	37.7
Colombia	Covarachía	Count	2000	0	0	0
Colombia	Covarachía	Count	2017	0	1	0
Colombia	Puracé	Proportion	2000	27.2	34.8	20.4
Colombia	Puracé	Proportion	2017	47.8	59.0	35.9
Colombia	Puracé	Count	2000	0	0	0
Colombia	Puracé	Count	2017	0	1	0
Colombia	Fómeque	Proportion	2000	30.0	36.3	24.1
Colombia	Fómeque	Proportion	2017	44.7	58.9	33.1
Colombia	Fómeque	Count	2000	0	0	0
Colombia	Fómeque	Count	2017	0	1	0
Colombia	Saladoblanco	Proportion	2000	33.3	41.1	25.6
Colombia	Saladoblanco	Proportion	2017	53.0	64.5	40.4
Colombia	Saladoblanco	Count	2000	0	0	0
Colombia	Saladoblanco	Count	2017	1	1	0
Colombia	San Martín	Proportion	2000	31.2	39.6	24.1
Colombia	San Martín	Proportion	2017	47.0	57.8	35.2
Colombia	San Martín	Count	2000	0	0	0
Colombia	San Martín	Count	2017	0	1	0
Colombia	Ipiales	Proportion	2000	38.6	48.7	30.1

Table 4: ORS by Unit *(continued)*

Country	Unit	Metric	Year	Mean	Upper	Lower
Colombia	Ipiales	Proportion	2017	56.5	67.4	46.4
Colombia	Ipiales	Count	2000	0	0	0
Colombia	Ipiales	Count	2017	1	1	0
Colombia	Puerto Santander	Proportion	2000	39.9	48.9	31.5
Colombia	Puerto Santander	Proportion	2017	51.5	62.4	39.8
Colombia	Puerto Santander	Count	2000	0	0	0
Colombia	Puerto Santander	Count	2017	1	1	0
Colombia	El Guacamayo	Proportion	2000	32.1	40.1	24.7
Colombia	El Guacamayo	Proportion	2017	46.3	59.4	35.2
Colombia	El Guacamayo	Count	2000	0	0	0
Colombia	El Guacamayo	Count	2017	0	1	0
Colombia	Murillo	Proportion	2000	26.8	34.0	20.1
Colombia	Murillo	Proportion	2017	39.6	52.3	29.7
Colombia	Murillo	Count	2000	0	0	0
Colombia	Murillo	Count	2017	0	1	0
Colombia	Pradera	Proportion	2000	34.7	42.0	28.4
Colombia	Pradera	Proportion	2017	54.4	65.5	43.1
Colombia	Pradera	Count	2000	0	0	0
Colombia	Pradera	Count	2017	1	1	0
Colombia	Caracolí	Proportion	2000	40.0	47.1	31.6
Colombia	Caracolí	Proportion	2017	56.2	67.1	45.4
Colombia	Caracolí	Count	2000	0	0	0
Colombia	Caracolí	Count	2017	1	1	0
Colombia	Santa Rosa	Proportion	2000	45.3	54.2	37.7
Colombia	Santa Rosa	Proportion	2017	53.5	64.7	42.5
Colombia	Santa Rosa	Count	2000	0	1	0
Colombia	Santa Rosa	Count	2017	1	1	0
Colombia	Cubará	Proportion	2000	33.2	40.7	25.5
Colombia	Cubará	Proportion	2017	42.5	55.4	30.8
Colombia	Cubará	Count	2000	0	0	0
Colombia	Cubará	Count	2017	0	1	0
Colombia	Rosas	Proportion	2000	30.8	38.2	24.4
Colombia	Rosas	Proportion	2017	51.7	63.0	39.6
Colombia	Rosas	Count	2000	0	0	0
Colombia	Rosas	Count	2017	1	1	0
Colombia	Fosca	Proportion	2000	28.6	35.0	22.9
Colombia	Fosca	Proportion	2017	46.5	60.0	34.6
Colombia	Fosca	Count	2000	0	0	0
Colombia	Fosca	Count	2017	0	1	0
Colombia	San Agustín	Proportion	2000	33.5	41.3	25.7
Colombia	San Agustín	Proportion	2017	50.3	61.4	38.8
Colombia	San Agustín	Count	2000	0	0	0
Colombia	San Agustín	Count	2017	1	1	0
Colombia	Villavicencio	Proportion	2000	29.9	37.5	23.2
Colombia	Villavicencio	Proportion	2017	44.3	56.6	33.3
Colombia	Villavicencio	Count	2000	0	0	0
Colombia	Villavicencio	Count	2017	0	1	0
Colombia	La Cruz	Proportion	2000	34.7	43.9	26.3
Colombia	La Cruz	Proportion	2017	55.0	66.0	44.8

Table 4: ORS by Unit *(continued)*

Country	Unit	Metric	Year	Mean	Upper	Lower
Colombia	La Cruz	Count	2000	0	0	0
Colombia	La Cruz	Count	2017	1	1	0
Colombia	Ragonvalia	Proportion	2000	39.4	47.8	30.8
Colombia	Ragonvalia	Proportion	2017	51.9	64.8	40.3
Colombia	Ragonvalia	Count	2000	0	0	0
Colombia	Ragonvalia	Count	2017	1	1	0
Colombia	El Peñon	Proportion	2000	33.4	40.8	25.3
Colombia	El Peñon	Proportion	2017	43.8	57.3	32.7
Colombia	El Peñon	Count	2000	0	0	0
Colombia	El Peñon	Count	2017	0	1	0
Colombia	Natagaima	Proportion	2000	38.0	45.3	30.5
Colombia	Natagaima	Proportion	2017	54.7	65.5	42.2
Colombia	Natagaima	Count	2000	0	0	0
Colombia	Natagaima	Count	2017	1	1	0
Colombia	Restrepo	Proportion	2000	36.3	44.1	29.9
Colombia	Restrepo	Proportion	2017	55.1	67.4	43.9
Colombia	Restrepo	Count	2000	0	0	0
Colombia	Restrepo	Count	2017	1	1	0
Colombia	Caramanta	Proportion	2000	31.7	38.5	24.5
Colombia	Caramanta	Proportion	2017	46.6	59.5	36.6
Colombia	Caramanta	Count	2000	0	0	0
Colombia	Caramanta	Count	2017	0	1	0
Colombia	Santa Rosa del Sur	Proportion	2000	36.8	43.6	29.1
Colombia	Santa Rosa del Sur	Proportion	2017	46.6	57.9	36.2
Colombia	Santa Rosa del Sur	Count	2000	0	0	0
Colombia	Santa Rosa del Sur	Count	2017	0	1	0
Colombia	Cucaita	Proportion	2000	26.8	34.2	20.9
Colombia	Cucaita	Proportion	2017	45.0	60.3	33.3
Colombia	Cucaita	Count	2000	0	0	0
Colombia	Cucaita	Count	2017	0	1	0
Colombia	San Sebastián	Proportion	2000	30.7	38.7	22.9
Colombia	San Sebastián	Proportion	2017	49.6	60.6	37.8
Colombia	San Sebastián	Count	2000	0	0	0
Colombia	San Sebastián	Count	2017	0	1	0
Colombia	Funza	Proportion	2000	34.6	40.6	29.2
Colombia	Funza	Proportion	2017	56.1	68.3	43.3
Colombia	Funza	Count	2000	0	0	0
Colombia	Funza	Count	2017	1	1	0
Colombia	Santa María	Proportion	2000	33.2	40.5	26.2
Colombia	Santa María	Proportion	2017	52.0	62.7	39.5
Colombia	Santa María	Count	2000	0	0	0
Colombia	Santa María	Count	2017	1	1	0
Colombia	Vista Hermosa	Proportion	2000	32.8	41.8	24.3
Colombia	Vista Hermosa	Proportion	2017	45.2	56.6	33.7
Colombia	Vista Hermosa	Count	2000	0	0	0
Colombia	Vista Hermosa	Count	2017	0	1	0
Colombia	La Florida	Proportion	2000	36.3	45.6	28.1
Colombia	La Florida	Proportion	2017	55.4	66.8	43.9
Colombia	La Florida	Count	2000	0	0	0

Table 4: ORS by Unit *(continued)*

Country	Unit	Metric	Year	Mean	Upper	Lower
Colombia	La Florida	Count	2017	1	1	0
Colombia	Salazar de las Palmas	Proportion	2000	38.6	46.5	30.5
Colombia	Salazar de las Palmas	Proportion	2017	47.9	61.0	37.2
Colombia	Salazar de las Palmas	Count	2000	0	0	0
Colombia	Salazar de las Palmas	Count	2017	0	1	0
Colombia	El Playón	Proportion	2000	36.4	43.4	28.3
Colombia	El Playón	Proportion	2017	50.7	62.7	39.1
Colombia	El Playón	Count	2000	0	0	0
Colombia	El Playón	Count	2017	1	1	0
Colombia	Ortega	Proportion	2000	38.6	45.6	31.3
Colombia	Ortega	Proportion	2017	54.8	66.5	43.0
Colombia	Ortega	Count	2000	0	0	0
Colombia	Ortega	Count	2017	1	1	0
Colombia	Riofrío	Proportion	2000	35.5	42.6	28.2
Colombia	Riofrío	Proportion	2017	52.1	64.9	41.4
Colombia	Riofrío	Count	2000	0	0	0
Colombia	Riofrío	Count	2017	1	1	0
Colombia	Carepa	Proportion	2000	37.6	47.1	28.2
Colombia	Carepa	Proportion	2017	50.5	62.7	39.5
Colombia	Carepa	Count	2000	0	0	0
Colombia	Carepa	Count	2017	1	1	0
Colombia	Simití	Proportion	2000	39.9	46.9	31.5
Colombia	Simití	Proportion	2017	52.2	63.9	41.4
Colombia	Simití	Count	2000	0	0	0
Colombia	Simití	Count	2017	1	1	0
Colombia	Cuítiva	Proportion	2000	28.5	35.6	22.3
Colombia	Cuítiva	Proportion	2017	49.2	64.0	36.8
Colombia	Cuítiva	Count	2000	0	0	0
Colombia	Cuítiva	Count	2017	0	1	0
Colombia	Santa Rosa	Proportion	2000	32.9	40.9	25.0
Colombia	Santa Rosa	Proportion	2017	45.1	55.5	35.2
Colombia	Santa Rosa	Count	2000	0	0	0
Colombia	Santa Rosa	Count	2017	0	1	0
Colombia	Fúquene	Proportion	2000	28.9	34.7	23.0
Colombia	Fúquene	Proportion	2017	45.3	60.5	33.4
Colombia	Fúquene	Count	2000	0	0	0
Colombia	Fúquene	Count	2017	0	1	0
Colombia	Suaza	Proportion	2000	35.8	43.6	27.7
Colombia	Suaza	Proportion	2017	53.9	64.2	41.9
Colombia	Suaza	Count	2000	0	0	0
Colombia	Suaza	Count	2017	1	1	0
Colombia	La Llanada	Proportion	2000	39.0	48.6	29.4
Colombia	La Llanada	Proportion	2017	53.9	65.6	42.9
Colombia	La Llanada	Count	2000	0	0	0
Colombia	La Llanada	Count	2017	1	1	0
Colombia	San Calixto	Proportion	2000	41.6	50.2	33.4
Colombia	San Calixto	Proportion	2017	51.0	62.4	39.2
Colombia	San Calixto	Count	2000	0	1	0
Colombia	San Calixto	Count	2017	1	1	0

Table 4: ORS by Unit *(continued)*

Country	Unit	Metric	Year	Mean	Upper	Lower
Colombia	Encino	Proportion	2000	30.2	37.3	23.3
Colombia	Encino	Proportion	2017	47.7	61.0	36.2
Colombia	Encino	Count	2000	0	0	0
Colombia	Encino	Count	2017	0	1	0
Colombia	Piedras	Proportion	2000	38.5	44.5	32.6
Colombia	Piedras	Proportion	2017	53.3	66.5	41.4
Colombia	Piedras	Count	2000	0	0	0
Colombia	Piedras	Count	2017	1	1	0
Colombia	Roldanillo	Proportion	2000	35.9	43.1	28.9
Colombia	Roldanillo	Proportion	2017	56.1	68.7	45.7
Colombia	Roldanillo	Count	2000	0	0	0
Colombia	Roldanillo	Count	2017	1	1	0
Colombia	Carolina del Principe	Proportion	2000	33.8	40.6	26.5
Colombia	Carolina del Principe	Proportion	2017	43.9	56.5	33.1
Colombia	Carolina del Principe	Count	2000	0	0	0
Colombia	Carolina del Principe	Count	2017	0	1	0
Colombia	Soplaviento	Proportion	2000	46.7	56.5	39.2
Colombia	Soplaviento	Proportion	2017	58.8	68.7	48.1
Colombia	Soplaviento	Count	2000	0	1	0
Colombia	Soplaviento	Count	2017	1	1	0
Colombia	Duitama	Proportion	2000	29.2	37.4	22.5
Colombia	Duitama	Proportion	2017	53.9	66.9	42.0
Colombia	Duitama	Count	2000	0	0	0
Colombia	Duitama	Count	2017	1	1	0
Colombia	Santander de Quilichao	Proportion	2000	32.9	40.1	26.5
Colombia	Santander de Quilichao	Proportion	2017	53.5	64.8	41.7
Colombia	Santander de Quilichao	Count	2000	0	0	0
Colombia	Santander de Quilichao	Count	2017	1	1	0
Colombia	Fusagasugá	Proportion	2000	33.7	39.9	27.9
Colombia	Fusagasugá	Proportion	2017	53.1	64.1	40.9
Colombia	Fusagasugá	Count	2000	0	0	0
Colombia	Fusagasugá	Count	2017	1	1	0
Colombia	Tarquí	Proportion	2000	33.4	41.1	26.3
Colombia	Tarquí	Proportion	2017	53.3	64.6	40.8
Colombia	Tarquí	Count	2000	0	0	0
Colombia	Tarquí	Count	2017	1	1	0
Colombia	La Tola	Proportion	2000	37.3	45.5	28.8
Colombia	La Tola	Proportion	2017	47.4	58.0	36.3
Colombia	La Tola	Count	2000	0	0	0
Colombia	La Tola	Count	2017	0	1	0
Colombia	San Cayetano	Proportion	2000	40.0	48.5	31.3
Colombia	San Cayetano	Proportion	2017	50.8	63.9	39.3
Colombia	San Cayetano	Count	2000	0	0	0
Colombia	San Cayetano	Count	2017	1	1	0
Colombia	Enciso	Proportion	2000	37.4	45.1	29.8
Colombia	Enciso	Proportion	2017	52.4	66.8	40.2
Colombia	Enciso	Count	2000	0	0	0
Colombia	Enciso	Count	2017	1	1	0
Colombia	Planadas	Proportion	2000	32.2	39.0	25.6

Table 4: ORS by Unit *(continued)*

Country	Unit	Metric	Year	Mean	Upper	Lower
Colombia	Planadas	Proportion	2017	47.3	57.6	34.9
Colombia	Planadas	Count	2000	0	0	0
Colombia	Planadas	Count	2017	0	1	0
Colombia	San Pedro	Proportion	2000	38.9	46.3	31.4
Colombia	San Pedro	Proportion	2017	55.9	68.6	44.8
Colombia	San Pedro	Count	2000	0	0	0
Colombia	San Pedro	Count	2017	1	1	0
Colombia	Caucasia	Proportion	2000	39.0	47.8	30.9
Colombia	Caucasia	Proportion	2017	56.4	66.9	45.4
Colombia	Caucasia	Count	2000	0	0	0
Colombia	Caucasia	Count	2017	1	1	0
Colombia	Talaigua Nuevo	Proportion	2000	45.3	53.5	37.1
Colombia	Talaigua Nuevo	Proportion	2017	58.1	69.3	46.4
Colombia	Talaigua Nuevo	Count	2000	0	1	0
Colombia	Talaigua Nuevo	Count	2017	1	1	0
Colombia	El Cocuy	Proportion	2000	27.0	34.9	20.2
Colombia	El Cocuy	Proportion	2017	43.6	57.0	31.6
Colombia	El Cocuy	Count	2000	0	0	0
Colombia	El Cocuy	Count	2017	0	1	0
Colombia	Silvia	Proportion	2000	27.6	34.9	21.0
Colombia	Silvia	Proportion	2017	51.3	63.2	39.5
Colombia	Silvia	Count	2000	0	0	0
Colombia	Silvia	Count	2017	1	1	0
Colombia	Gachalá	Proportion	2000	31.5	38.6	25.2
Colombia	Gachalá	Proportion	2017	44.2	56.5	32.3
Colombia	Gachalá	Count	2000	0	0	0
Colombia	Gachalá	Count	2017	0	1	0
Colombia	Tello	Proportion	2000	38.4	46.5	30.1
Colombia	Tello	Proportion	2017	56.1	67.4	43.6
Colombia	Tello	Count	2000	0	0	0
Colombia	Tello	Count	2017	1	1	0
Colombia	La Unión de Sucre	Proportion	2000	36.4	45.5	28.2
Colombia	La Unión de Sucre	Proportion	2017	55.7	66.9	43.7
Colombia	La Unión de Sucre	Count	2000	0	0	0
Colombia	La Unión de Sucre	Count	2017	1	1	0
Colombia	San José de Cúcuta	Proportion	2000	44.5	53.3	35.4
Colombia	San José de Cúcuta	Proportion	2017	59.2	71.5	47.4
Colombia	San José de Cúcuta	Count	2000	0	1	0
Colombia	San José de Cúcuta	Count	2017	1	1	0
Colombia	Florián	Proportion	2000	34.1	40.6	26.4
Colombia	Florián	Proportion	2017	47.0	60.1	35.7
Colombia	Florián	Count	2000	0	0	0
Colombia	Florián	Count	2017	0	1	0
Colombia	Prado	Proportion	2000	37.8	45.3	30.8
Colombia	Prado	Proportion	2017	52.7	63.9	41.3
Colombia	Prado	Count	2000	0	0	0
Colombia	Prado	Count	2017	1	1	0
Colombia	Santiago de Cali	Proportion	2000	36.1	43.7	29.9
Colombia	Santiago de Cali	Proportion	2017	51.7	62.7	40.2

Table 4: ORS by Unit *(continued)*

Country	Unit	Metric	Year	Mean	Upper	Lower
Colombia	Santiago de Cali	Count	2000	0	0	0
Colombia	Santiago de Cali	Count	2017	1	1	0
Colombia	Chigorodó	Proportion	2000	36.5	46.3	26.7
Colombia	Chigorodó	Proportion	2017	48.5	60.3	37.5
Colombia	Chigorodó	Count	2000	0	0	0
Colombia	Chigorodó	Count	2017	0	1	0
Colombia	Turbaco	Proportion	2000	45.9	54.8	38.1
Colombia	Turbaco	Proportion	2017	55.9	66.6	44.6
Colombia	Turbaco	Count	2000	0	1	0
Colombia	Turbaco	Count	2017	1	1	0
Colombia	El Espino	Proportion	2000	32.1	39.9	25.5
Colombia	El Espino	Proportion	2017	53.8	67.6	42.1
Colombia	El Espino	Count	2000	0	0	0
Colombia	El Espino	Count	2017	1	1	0
Colombia	Sotará	Proportion	2000	29.1	36.4	22.3
Colombia	Sotará	Proportion	2017	50.6	62.5	38.7
Colombia	Sotará	Count	2000	0	0	0
Colombia	Sotará	Count	2017	1	1	0
Colombia	Gachancipá	Proportion	2000	29.3	35.4	23.3
Colombia	Gachancipá	Proportion	2017	48.5	63.4	36.0
Colombia	Gachancipá	Count	2000	0	0	0
Colombia	Gachancipá	Count	2017	0	1	0
Colombia	Teruel	Proportion	2000	35.0	42.1	27.5
Colombia	Teruel	Proportion	2017	53.8	64.8	40.9
Colombia	Teruel	Count	2000	0	0	0
Colombia	Teruel	Count	2017	1	1	0
Colombia	Leiva	Proportion	2000	36.4	44.8	27.7
Colombia	Leiva	Proportion	2017	53.0	64.6	40.8
Colombia	Leiva	Count	2000	0	0	0
Colombia	Leiva	Count	2017	1	1	0
Colombia	Santiago	Proportion	2000	41.2	49.0	33.5
Colombia	Santiago	Proportion	2017	50.3	62.8	38.7
Colombia	Santiago	Count	2000	0	0	0
Colombia	Santiago	Count	2017	1	1	0
Colombia	Floridablanca	Proportion	2000	35.7	42.4	29.0
Colombia	Floridablanca	Proportion	2017	47.4	61.9	35.3
Colombia	Floridablanca	Count	2000	0	0	0
Colombia	Floridablanca	Count	2017	0	1	0
Colombia	Purificación	Proportion	2000	38.5	45.9	31.5
Colombia	Purificación	Proportion	2017	55.8	66.7	45.1
Colombia	Purificación	Count	2000	0	0	0
Colombia	Purificación	Count	2017	1	1	0
Colombia	Sevilla	Proportion	2000	33.6	40.2	26.7
Colombia	Sevilla	Proportion	2017	52.8	64.7	40.7
Colombia	Sevilla	Count	2000	0	0	0
Colombia	Sevilla	Count	2017	1	1	0
Colombia	Cisneros	Proportion	2000	34.0	41.0	27.1
Colombia	Cisneros	Proportion	2017	50.1	60.7	39.1
Colombia	Cisneros	Count	2000	0	0	0

Table 4: ORS by Unit *(continued)*

Country	Unit	Metric	Year	Mean	Upper	Lower
Colombia	Cisneros	Count	2017	1	1	0
Colombia	Turbaná	Proportion	2000	45.4	54.6	37.2
Colombia	Turbaná	Proportion	2017	56.5	66.9	45.2
Colombia	Turbaná	Count	2000	0	1	0
Colombia	Turbaná	Count	2017	1	1	0
Colombia	Firavitoba	Proportion	2000	27.4	34.8	21.5
Colombia	Firavitoba	Proportion	2017	46.1	61.1	33.8
Colombia	Firavitoba	Count	2000	0	0	0
Colombia	Firavitoba	Count	2017	0	1	0
Colombia	Suárez	Proportion	2000	30.0	37.1	23.3
Colombia	Suárez	Proportion	2017	50.3	61.1	38.7
Colombia	Suárez	Count	2000	0	0	0
Colombia	Suárez	Count	2017	1	1	0
Colombia	Gachetá	Proportion	2000	30.7	37.5	24.7
Colombia	Gachetá	Proportion	2017	49.7	63.1	38.1
Colombia	Gachetá	Count	2000	0	0	0
Colombia	Gachetá	Count	2017	0	1	0
Colombia	Tesalia	Proportion	2000	37.4	45.1	29.3
Colombia	Tesalia	Proportion	2017	54.4	65.5	41.7
Colombia	Tesalia	Count	2000	0	0	0
Colombia	Tesalia	Count	2017	1	1	0
Colombia	Linares	Proportion	2000	38.7	48.0	30.4
Colombia	Linares	Proportion	2017	56.0	67.6	45.0
Colombia	Linares	Count	2000	0	0	0
Colombia	Linares	Count	2017	1	1	0
Colombia	Santo Domingo de Silos	Proportion	2000	33.4	40.3	25.9
Colombia	Santo Domingo de Silos	Proportion	2017	47.0	60.1	36.1
Colombia	Santo Domingo de Silos	Count	2000	0	0	0
Colombia	Santo Domingo de Silos	Count	2017	0	1	0
Colombia	Galán	Proportion	2000	32.6	40.2	26.2
Colombia	Galán	Proportion	2017	46.0	59.0	33.5
Colombia	Galán	Count	2000	0	0	0
Colombia	Galán	Count	2017	0	1	0
Colombia	Rioblanco	Proportion	2000	31.3	38.8	24.6
Colombia	Rioblanco	Proportion	2017	44.8	54.7	33.4
Colombia	Rioblanco	Count	2000	0	0	0
Colombia	Rioblanco	Count	2017	0	1	0
Colombia	Toro	Proportion	2000	35.1	42.1	27.5
Colombia	Toro	Proportion	2017	54.1	67.3	43.2
Colombia	Toro	Count	2000	0	0	0
Colombia	Toro	Count	2017	1	1	0
Colombia	Cocorná	Proportion	2000	33.3	40.8	26.3
Colombia	Cocorná	Proportion	2017	47.6	59.9	37.6
Colombia	Cocorná	Count	2000	0	0	0
Colombia	Cocorná	Count	2017	0	1	0
Colombia	Villanueva	Proportion	2000	45.7	54.4	38.1
Colombia	Villanueva	Proportion	2017	55.4	66.1	44.1
Colombia	Villanueva	Count	2000	0	1	0
Colombia	Villanueva	Count	2017	1	1	0

Table 4: ORS by Unit *(continued)*

Country	Unit	Metric	Year	Mean	Upper	Lower
Colombia	Floresta	Proportion	2000	29.1	36.6	22.7
Colombia	Floresta	Proportion	2017	50.4	63.4	38.4
Colombia	Floresta	Count	2000	0	0	0
Colombia	Floresta	Count	2017	1	1	0
Colombia	Timbío	Proportion	2000	30.7	38.3	23.9
Colombia	Timbío	Proportion	2017	53.5	65.0	41.5
Colombia	Timbío	Count	2000	0	0	0
Colombia	Timbío	Count	2017	1	1	0
Colombia	Gama	Proportion	2000	31.3	38.3	25.0
Colombia	Gama	Proportion	2017	47.8	60.6	36.1
Colombia	Gama	Count	2000	0	0	0
Colombia	Gama	Count	2017	0	1	0
Colombia	Timaná	Proportion	2000	35.5	43.7	27.2
Colombia	Timaná	Proportion	2017	54.1	64.8	41.7
Colombia	Timaná	Count	2000	0	0	0
Colombia	Timaná	Count	2017	1	1	0
Colombia	Los Andes	Proportion	2000	38.4	47.9	29.1
Colombia	Los Andes	Proportion	2017	54.8	66.3	43.6
Colombia	Los Andes	Count	2000	0	0	0
Colombia	Los Andes	Count	2017	1	1	0
Colombia	Sardinata	Proportion	2000	41.7	50.1	33.5
Colombia	Sardinata	Proportion	2017	48.8	60.6	37.1
Colombia	Sardinata	Count	2000	0	1	0
Colombia	Sardinata	Count	2017	0	1	0
Colombia	Gámbita	Proportion	2000	29.8	36.9	22.6
Colombia	Gámbita	Proportion	2017	48.0	63.0	36.7
Colombia	Gámbita	Count	2000	0	0	0
Colombia	Gámbita	Count	2017	0	1	0
Colombia	Roncesvalles	Proportion	2000	30.3	37.4	24.0
Colombia	Roncesvalles	Proportion	2017	44.5	56.4	32.6
Colombia	Roncesvalles	Count	2000	0	0	0
Colombia	Roncesvalles	Count	2017	0	1	0
Colombia	Trujillo	Proportion	2000	33.9	41.1	26.7
Colombia	Trujillo	Proportion	2017	51.3	64.0	40.7
Colombia	Trujillo	Count	2000	0	0	0
Colombia	Trujillo	Count	2017	1	1	0
Colombia	Concepción	Proportion	2000	34.8	41.6	27.5
Colombia	Concepción	Proportion	2017	49.0	61.0	37.9
Colombia	Concepción	Count	2000	0	0	0
Colombia	Concepción	Count	2017	0	1	0
Colombia	Zambrano	Proportion	2000	48.1	56.7	40.6
Colombia	Zambrano	Proportion	2017	64.6	75.0	53.1
Colombia	Zambrano	Count	2000	0	1	0
Colombia	Zambrano	Count	2017	1	1	1
Colombia	Gachantivá	Proportion	2000	27.8	35.0	21.6
Colombia	Gachantivá	Proportion	2017	48.0	62.7	36.6
Colombia	Gachantivá	Count	2000	0	0	0
Colombia	Gachantivá	Count	2017	0	1	0
Colombia	Timbiquí	Proportion	2000	31.1	38.2	24.1

Table 4: ORS by Unit *(continued)*

Country	Unit	Metric	Year	Mean	Upper	Lower
Colombia	Timbiquí	Proportion	2017	42.7	53.2	30.4
Colombia	Timbiquí	Count	2000	0	0	0
Colombia	Timbiquí	Count	2017	0	1	0
Colombia	Girardot	Proportion	2000	35.8	41.9	29.4
Colombia	Girardot	Proportion	2017	51.1	63.4	39.2
Colombia	Girardot	Count	2000	0	0	0
Colombia	Girardot	Count	2017	1	1	0
Colombia	Villavieja	Proportion	2000	38.2	45.8	30.2
Colombia	Villavieja	Proportion	2017	55.2	66.0	42.5
Colombia	Villavieja	Count	2000	0	0	0
Colombia	Villavieja	Count	2017	1	1	0
Colombia	Magüí	Proportion	2000	37.5	46.0	29.1
Colombia	Magüí	Proportion	2017	45.7	57.5	34.5
Colombia	Magüí	Count	2000	0	0	0
Colombia	Magüí	Count	2017	0	1	0
Colombia	Teorama	Proportion	2000	40.8	50.1	32.6
Colombia	Teorama	Proportion	2017	49.3	60.8	38.7
Colombia	Teorama	Count	2000	0	1	0
Colombia	Teorama	Count	2017	0	1	0
Colombia	Girón	Proportion	2000	35.8	42.8	29.1
Colombia	Girón	Proportion	2017	53.3	67.0	42.0
Colombia	Girón	Count	2000	0	0	0
Colombia	Girón	Count	2017	1	1	0
Colombia	Rovira	Proportion	2000	33.7	40.7	26.8
Colombia	Rovira	Proportion	2017	49.6	62.2	38.7
Colombia	Rovira	Count	2000	0	0	0
Colombia	Rovira	Count	2017	0	1	0
Colombia	Tuluá	Proportion	2000	37.0	43.8	29.4
Colombia	Tuluá	Proportion	2017	52.8	65.7	41.6
Colombia	Tuluá	Count	2000	0	0	0
Colombia	Tuluá	Count	2017	1	1	0
Colombia	Concordia	Proportion	2000	36.6	43.4	28.6
Colombia	Concordia	Proportion	2017	52.0	65.0	40.6
Colombia	Concordia	Count	2000	0	0	0
Colombia	Concordia	Count	2017	1	1	0
Colombia	Gámeza	Proportion	2000	31.4	39.0	24.5
Colombia	Gámeza	Proportion	2017	51.0	63.8	38.7
Colombia	Gámeza	Count	2000	0	0	0
Colombia	Gámeza	Count	2017	1	1	0
Colombia	Toribío	Proportion	2000	31.9	39.0	25.7
Colombia	Toribío	Proportion	2017	53.8	65.0	41.4
Colombia	Toribío	Count	2000	0	0	0
Colombia	Toribío	Count	2017	1	1	0
Colombia	Guachetá	Proportion	2000	27.7	33.8	21.9
Colombia	Guachetá	Proportion	2017	45.6	60.3	34.0
Colombia	Guachetá	Count	2000	0	0	0
Colombia	Guachetá	Count	2017	0	1	0
Colombia	Yaguará	Proportion	2000	37.5	45.6	29.5
Colombia	Yaguará	Proportion	2017	54.2	65.7	41.0

Table 4: ORS by Unit *(continued)*

Country	Unit	Metric	Year	Mean	Upper	Lower
Colombia	Yaguará	Count	2000	0	0	0
Colombia	Yaguará	Count	2017	1	1	0
Colombia	Mallama	Proportion	2000	37.5	47.9	28.7
Colombia	Mallama	Proportion	2017	51.1	62.6	40.7
Colombia	Mallama	Count	2000	0	0	0
Colombia	Mallama	Count	2017	1	1	0
Colombia	Tibú	Proportion	2000	40.9	50.0	32.1
Colombia	Tibú	Proportion	2017	47.0	58.6	36.0
Colombia	Tibú	Count	2000	0	0	0
Colombia	Tibú	Count	2017	0	1	0
Colombia	Guaca	Proportion	2000	32.3	39.3	24.7
Colombia	Guaca	Proportion	2017	46.9	61.0	34.9
Colombia	Guaca	Count	2000	0	0	0
Colombia	Guaca	Count	2017	0	1	0
Colombia	Saldaña	Proportion	2000	35.5	43.0	28.2
Colombia	Saldaña	Proportion	2017	51.7	62.6	40.9
Colombia	Saldaña	Count	2000	0	0	0
Colombia	Saldaña	Count	2017	1	1	0
Colombia	Ulloa	Proportion	2000	35.1	42.0	28.4
Colombia	Ulloa	Proportion	2017	54.7	67.6	43.6
Colombia	Ulloa	Count	2000	0	0	0
Colombia	Ulloa	Count	2017	1	1	0
Colombia	Copacabana	Proportion	2000	38.8	45.8	31.2
Colombia	Copacabana	Proportion	2017	53.0	65.3	41.1
Colombia	Copacabana	Count	2000	0	0	0
Colombia	Copacabana	Count	2017	1	1	0
Colombia	Garagoa	Proportion	2000	29.6	37.2	23.1
Colombia	Garagoa	Proportion	2017	47.4	61.1	34.7
Colombia	Garagoa	Count	2000	0	0	0
Colombia	Garagoa	Count	2017	0	1	0
Colombia	Totoró	Proportion	2000	27.4	34.7	20.5
Colombia	Totoró	Proportion	2017	50.3	61.5	39.0
Colombia	Totoró	Count	2000	0	0	0
Colombia	Totoró	Count	2017	1	1	0
Colombia	Guaduas	Proportion	2000	36.3	42.8	28.9
Colombia	Guaduas	Proportion	2017	55.0	66.8	43.2
Colombia	Guaduas	Count	2000	0	0	0
Colombia	Guaduas	Count	2017	1	1	0
Colombia	Mosquera	Proportion	2000	35.5	44.2	26.7
Colombia	Mosquera	Proportion	2017	43.1	53.5	32.4
Colombia	Mosquera	Count	2000	0	0	0
Colombia	Mosquera	Count	2017	0	1	0
Colombia	Toledo	Proportion	2000	35.6	43.6	27.8
Colombia	Toledo	Proportion	2017	45.8	57.4	34.8
Colombia	Toledo	Count	2000	0	0	0
Colombia	Toledo	Count	2017	0	1	0
Colombia	Guadalupe	Proportion	2000	32.3	40.5	25.1
Colombia	Guadalupe	Proportion	2017	47.2	60.4	36.2
Colombia	Guadalupe	Count	2000	0	0	0

Table 4: ORS by Unit *(continued)*

Country	Unit	Metric	Year	Mean	Upper	Lower
Colombia	Guadalupe	Count	2017	0	1	0
Colombia	San Antonio	Proportion	2000	32.6	39.4	25.5
Colombia	San Antonio	Proportion	2017	48.5	60.2	36.4
Colombia	San Antonio	Count	2000	0	0	0
Colombia	San Antonio	Count	2017	0	1	0
Colombia	Versalles	Proportion	2000	32.6	39.6	25.4
Colombia	Versalles	Proportion	2017	46.7	59.9	36.3
Colombia	Versalles	Count	2000	0	0	0
Colombia	Versalles	Count	2017	0	1	0
Colombia	Dabeiba	Proportion	2000	37.1	45.0	29.2
Colombia	Dabeiba	Proportion	2017	47.4	58.8	36.0
Colombia	Dabeiba	Count	2000	0	0	0
Colombia	Dabeiba	Count	2017	0	1	0
Colombia	Guacamayas	Proportion	2000	31.8	39.6	25.5
Colombia	Guacamayas	Proportion	2017	50.7	64.5	38.5
Colombia	Guacamayas	Count	2000	0	0	0
Colombia	Guacamayas	Count	2017	1	1	0
Colombia	Guasca	Proportion	2000	30.5	36.8	24.5
Colombia	Guasca	Proportion	2017	47.0	61.2	34.6
Colombia	Guasca	Count	2000	0	0	0
Colombia	Guasca	Count	2017	0	1	0
Colombia	Olaya Herrera	Proportion	2000	36.4	44.6	27.6
Colombia	Olaya Herrera	Proportion	2017	45.1	55.4	35.0
Colombia	Olaya Herrera	Count	2000	0	0	0
Colombia	Olaya Herrera	Count	2017	0	1	0
Colombia	Villa Caro	Proportion	2000	39.9	47.5	32.0
Colombia	Villa Caro	Proportion	2017	50.6	63.3	39.6
Colombia	Villa Caro	Count	2000	0	0	0
Colombia	Villa Caro	Count	2017	1	1	0
Colombia	Guapotá	Proportion	2000	33.3	41.3	25.5
Colombia	Guapotá	Proportion	2017	51.0	63.7	40.2
Colombia	Guapotá	Count	2000	0	0	0
Colombia	Guapotá	Count	2017	1	1	0
Colombia	San Luís	Proportion	2000	39.2	46.2	32.6
Colombia	San Luís	Proportion	2017	54.7	67.8	43.3
Colombia	San Luís	Count	2000	0	0	0
Colombia	San Luís	Count	2017	1	1	0
Colombia	Vijes	Proportion	2000	36.7	44.3	30.4
Colombia	Vijes	Proportion	2017	55.7	67.9	44.8
Colombia	Vijes	Count	2000	0	0	0
Colombia	Vijes	Count	2017	1	1	0
Colombia	Don Matías	Proportion	2000	36.7	43.5	29.5
Colombia	Don Matías	Proportion	2017	51.5	63.6	39.2
Colombia	Don Matías	Count	2000	0	0	0
Colombia	Don Matías	Count	2017	1	1	0
Colombia	Guateque	Proportion	2000	30.3	37.1	23.9
Colombia	Guateque	Proportion	2017	47.5	62.0	35.3
Colombia	Guateque	Count	2000	0	0	0
Colombia	Guateque	Count	2017	0	1	0

Table 4: ORS by Unit *(continued)*

Country	Unit	Metric	Year	Mean	Upper	Lower
Colombia	Guataquí	Proportion	2000	38.1	44.2	32.0
Colombia	Guataquí	Proportion	2017	52.8	65.5	40.7
Colombia	Guataquí	Count	2000	0	0	0
Colombia	Guataquí	Count	2017	1	1	0
Colombia	Ospina	Proportion	2000	37.7	47.4	28.9
Colombia	Ospina	Proportion	2017	57.0	68.7	45.7
Colombia	Ospina	Count	2000	0	0	0
Colombia	Ospina	Count	2017	1	1	0
Colombia	Villa del Rosario	Proportion	2000	42.7	51.4	33.6
Colombia	Villa del Rosario	Proportion	2017	55.5	68.5	43.1
Colombia	Villa del Rosario	Count	2000	0	1	0
Colombia	Villa del Rosario	Count	2017	1	1	0
Colombia	Guavatá	Proportion	2000	32.6	39.8	25.2
Colombia	Guavatá	Proportion	2017	47.6	61.8	36.1
Colombia	Guavatá	Count	2000	0	0	0
Colombia	Guavatá	Count	2017	0	1	0
Colombia	San Sebastian de Mariquita	Proportion	2000	35.5	42.9	28.3
Colombia	San Sebastian de Mariquita	Proportion	2017	53.3	65.5	42.4
Colombia	San Sebastian de Mariquita	Count	2000	0	0	0
Colombia	San Sebastian de Mariquita	Count	2017	1	1	0
Colombia	Yotoco	Proportion	2000	36.8	44.3	30.0
Colombia	Yotoco	Proportion	2017	54.1	66.8	43.0
Colombia	Yotoco	Count	2000	0	0	0
Colombia	Yotoco	Count	2017	1	1	0
Colombia	Ebéjico	Proportion	2000	38.1	43.8	29.9
Colombia	Ebéjico	Proportion	2017	55.8	68.7	43.8
Colombia	Ebéjico	Count	2000	0	0	0
Colombia	Ebéjico	Count	2017	1	1	0
Colombia	Guayatá	Proportion	2000	28.7	35.6	22.7
Colombia	Guayatá	Proportion	2017	46.3	60.4	34.4
Colombia	Guayatá	Count	2000	0	0	0
Colombia	Guayatá	Count	2017	0	1	0
Colombia	Guatavita	Proportion	2000	28.6	35.1	22.7
Colombia	Guatavita	Proportion	2017	46.1	60.9	33.6
Colombia	Guatavita	Count	2000	0	0	0
Colombia	Guatavita	Count	2017	0	1	0
Colombia	Policarpa	Proportion	2000	39.8	48.9	30.8
Colombia	Policarpa	Proportion	2017	55.6	67.4	43.8
Colombia	Policarpa	Count	2000	0	0	0
Colombia	Policarpa	Count	2017	1	1	0
Colombia	Güepsa	Proportion	2000	34.5	42.8	26.5
Colombia	Güepsa	Proportion	2017	52.5	65.0	41.3
Colombia	Güepsa	Count	2000	0	0	0
Colombia	Güepsa	Count	2017	1	1	0
Colombia	Santa Isabel	Proportion	2000	29.9	36.5	23.7

Table 4: ORS by Unit *(continued)*

Country	Unit	Metric	Year	Mean	Upper	Lower
Colombia	Santa Isabel	Proportion	2017	44.4	57.1	33.6
Colombia	Santa Isabel	Count	2000	0	0	0
Colombia	Santa Isabel	Count	2017	0	1	0
Colombia	Yumbo	Proportion	2000	35.5	43.1	29.4
Colombia	Yumbo	Proportion	2017	50.8	62.2	39.3
Colombia	Yumbo	Count	2000	0	0	0
Colombia	Yumbo	Count	2017	1	1	0
Colombia	El Bagre	Proportion	2000	38.5	45.7	30.4
Colombia	El Bagre	Proportion	2017	51.7	61.8	40.6
Colombia	El Bagre	Count	2000	0	0	0
Colombia	El Bagre	Count	2017	1	1	0
Colombia	Guicán	Proportion	2000	29.1	36.8	21.9
Colombia	Guicán	Proportion	2017	42.8	56.0	31.7
Colombia	Guicán	Count	2000	0	0	0
Colombia	Guicán	Count	2017	0	1	0
Colombia	Guayabal de Síquima	Proportion	2000	33.4	39.5	27.3
Colombia	Guayabal de Síquima	Proportion	2017	48.1	61.8	36.4
Colombia	Guayabal de Síquima	Count	2000	0	0	0
Colombia	Guayabal de Síquima	Count	2017	0	1	0
Colombia	Potosí	Proportion	2000	36.0	45.9	28.1
Colombia	Potosí	Proportion	2017	53.7	65.8	43.0
Colombia	Potosí	Count	2000	0	0	0
Colombia	Potosí	Count	2017	1	1	0
Colombia	Hato	Proportion	2000	32.2	39.9	25.4
Colombia	Hato	Proportion	2017	46.6	59.3	35.2
Colombia	Hato	Count	2000	0	0	0
Colombia	Hato	Count	2017	0	1	0
Colombia	Suárez	Proportion	2000	38.6	45.5	31.8
Colombia	Suárez	Proportion	2017	54.8	66.9	43.0
Colombia	Suárez	Count	2000	0	0	0
Colombia	Suárez	Count	2017	1	1	0
Colombia	Zarzal	Proportion	2000	34.7	41.6	27.9
Colombia	Zarzal	Proportion	2017	53.9	66.8	42.8
Colombia	Zarzal	Count	2000	0	0	0
Colombia	Zarzal	Count	2017	1	1	0
Colombia	El Carmen de Viboral	Proportion	2000	36.0	43.4	28.9
Colombia	El Carmen de Viboral	Proportion	2017	51.7	63.8	41.8
Colombia	El Carmen de Viboral	Count	2000	0	0	0
Colombia	El Carmen de Viboral	Count	2017	1	1	0
Colombia	Izá	Proportion	2000	27.3	34.4	21.4
Colombia	Izá	Proportion	2017	47.5	62.6	35.2
Colombia	Izá	Count	2000	0	0	0
Colombia	Izá	Count	2017	0	1	0
Colombia	Guayabetal	Proportion	2000	29.3	36.4	23.4
Colombia	Guayabetal	Proportion	2017	43.1	55.7	31.1
Colombia	Guayabetal	Count	2000	0	0	0
Colombia	Guayabetal	Count	2017	0	1	0
Colombia	Providencia	Proportion	2000	36.5	45.5	28.4
Colombia	Providencia	Proportion	2017	56.3	67.7	45.4

Table 4: ORS by Unit *(continued)*

Country	Unit	Metric	Year	Mean	Upper	Lower
Colombia	Providencia	Count	2000	0	0	0
Colombia	Providencia	Count	2017	1	1	0
Colombia	Jesús María	Proportion	2000	32.6	39.5	25.1
Colombia	Jesús María	Proportion	2017	47.6	61.5	36.3
Colombia	Jesús María	Count	2000	0	0	0
Colombia	Jesús María	Count	2017	0	1	0
Colombia	Valle de San Juan	Proportion	2000	38.3	45.9	31.6
Colombia	Valle de San Juan	Proportion	2017	54.1	66.7	43.0
Colombia	Valle de San Juan	Count	2000	0	0	0
Colombia	Valle de San Juan	Count	2017	1	1	0
Colombia	El Santuario	Proportion	2000	35.0	42.2	27.7
Colombia	El Santuario	Proportion	2017	49.9	61.7	40.0
Colombia	El Santuario	Count	2000	0	0	0
Colombia	El Santuario	Count	2017	0	1	0
Colombia	Jenesano	Proportion	2000	29.3	36.4	22.9
Colombia	Jenesano	Proportion	2017	51.3	64.9	39.2
Colombia	Jenesano	Count	2000	0	0	0
Colombia	Jenesano	Count	2017	1	1	0
Colombia	Gutiérrez	Proportion	2000	33.8	41.5	27.0
Colombia	Gutiérrez	Proportion	2017	48.7	59.9	36.5
Colombia	Gutiérrez	Count	2000	0	0	0
Colombia	Gutiérrez	Count	2017	0	1	0
Colombia	Puerres	Proportion	2000	35.3	44.9	26.3
Colombia	Puerres	Proportion	2017	50.7	62.4	40.4
Colombia	Puerres	Count	2000	0	0	0
Colombia	Puerres	Count	2017	1	1	0
Colombia	Jordán	Proportion	2000	36.4	43.3	29.2
Colombia	Jordán	Proportion	2017	54.5	67.3	41.7
Colombia	Jordán	Count	2000	0	0	0
Colombia	Jordán	Count	2017	1	1	0
Colombia	Venadillo	Proportion	2000	37.2	44.0	31.3
Colombia	Venadillo	Proportion	2017	52.2	65.2	40.2
Colombia	Venadillo	Count	2000	0	0	0
Colombia	Venadillo	Count	2017	1	1	0
Colombia	Entrerríos	Proportion	2000	33.8	40.3	25.8
Colombia	Entrerríos	Proportion	2017	46.1	60.2	34.0
Colombia	Entrerríos	Count	2000	0	0	0
Colombia	Entrerríos	Count	2017	0	1	0
Colombia	Jericó	Proportion	2000	29.6	37.5	23.1
Colombia	Jericó	Proportion	2017	48.8	62.5	37.0
Colombia	Jericó	Count	2000	0	0	0
Colombia	Jericó	Count	2017	0	1	0
Colombia	Jerusalén	Proportion	2000	35.8	41.8	29.6
Colombia	Jerusalén	Proportion	2017	51.3	64.2	39.5
Colombia	Jerusalén	Count	2000	0	0	0
Colombia	Jerusalén	Count	2017	1	1	0
Colombia	Pupiales	Proportion	2000	35.7	45.7	26.7
Colombia	Pupiales	Proportion	2017	56.5	67.4	45.6
Colombia	Pupiales	Count	2000	0	0	0

Table 4: ORS by Unit *(continued)*

Country	Unit	Metric	Year	Mean	Upper	Lower
Colombia	Pupiales	Count	2017	1	1	0
Colombia	La Belleza	Proportion	2000	33.7	40.5	26.0
Colombia	La Belleza	Proportion	2017	48.3	61.8	37.1
Colombia	La Belleza	Count	2000	0	0	0
Colombia	La Belleza	Count	2017	0	1	0
Colombia	Villahermosa	Proportion	2000	30.4	37.4	24.6
Colombia	Villahermosa	Proportion	2017	44.0	55.9	33.9
Colombia	Villahermosa	Count	2000	0	0	0
Colombia	Villahermosa	Count	2017	0	1	0
Colombia	Envigado	Proportion	2000	40.5	46.9	33.1
Colombia	Envigado	Proportion	2017	52.4	65.1	40.5
Colombia	Envigado	Count	2000	0	0	0
Colombia	Envigado	Count	2017	1	1	0
Colombia	La Capilla	Proportion	2000	28.9	35.3	22.7
Colombia	La Capilla	Proportion	2017	47.2	62.1	35.4
Colombia	La Capilla	Count	2000	0	0	0
Colombia	La Capilla	Count	2017	0	1	0
Colombia	Junín	Proportion	2000	30.6	37.6	24.5
Colombia	Junín	Proportion	2017	46.2	59.2	34.6
Colombia	Junín	Count	2000	0	0	0
Colombia	Junín	Count	2017	0	1	0
Colombia	Ricaurte	Proportion	2000	37.5	47.6	29.0
Colombia	Ricaurte	Proportion	2017	45.8	57.2	36.3
Colombia	Ricaurte	Count	2000	0	0	0
Colombia	Ricaurte	Count	2017	0	1	0
Colombia	La Paz	Proportion	2000	33.4	41.3	25.5
Colombia	La Paz	Proportion	2017	48.8	61.9	37.6
Colombia	La Paz	Count	2000	0	0	0
Colombia	La Paz	Count	2017	0	1	0
Colombia	Villarrica	Proportion	2000	33.1	40.6	26.3
Colombia	Villarrica	Proportion	2017	47.7	59.7	36.1
Colombia	Villarrica	Count	2000	0	0	0
Colombia	Villarrica	Count	2017	0	1	0
Colombia	Fredonia	Proportion	2000	36.9	43.8	29.4
Colombia	Fredonia	Proportion	2017	53.3	65.4	41.8
Colombia	Fredonia	Count	2000	0	0	0
Colombia	Fredonia	Count	2017	1	1	0
Colombia	La Uvita	Proportion	2000	29.4	37.3	23.2
Colombia	La Uvita	Proportion	2017	50.0	63.8	37.2
Colombia	La Uvita	Count	2000	0	0	0
Colombia	La Uvita	Count	2017	0	1	0
Colombia	La Calera	Proportion	2000	32.0	37.8	26.3
Colombia	La Calera	Proportion	2017	45.4	58.3	33.9
Colombia	La Calera	Count	2000	0	0	0
Colombia	La Calera	Count	2017	0	1	0
Colombia	Roberto Payán	Proportion	2000	36.9	45.0	28.5
Colombia	Roberto Payán	Proportion	2017	44.3	55.2	32.6
Colombia	Roberto Payán	Count	2000	0	0	0
Colombia	Roberto Payán	Count	2017	0	1	0

Table 4: ORS by Unit *(continued)*

Country	Unit	Metric	Year	Mean	Upper	Lower
Colombia	Landázuri	Proportion	2000	34.2	41.4	25.6
Colombia	Landázuri	Proportion	2017	48.8	61.2	37.4
Colombia	Landázuri	Count	2000	0	0	0
Colombia	Landázuri	Count	2017	0	1	0
Colombia	Frontino	Proportion	2000	38.0	46.3	30.3
Colombia	Frontino	Proportion	2017	50.4	61.6	39.5
Colombia	Frontino	Count	2000	0	0	0
Colombia	Frontino	Count	2017	1	1	0
Colombia	La Victoria	Proportion	2000	33.9	39.9	27.2
Colombia	La Victoria	Proportion	2017	48.2	61.8	37.4
Colombia	La Victoria	Count	2000	0	0	0
Colombia	La Victoria	Count	2017	0	1	0
Colombia	La Mesa	Proportion	2000	37.2	43.3	31.2
Colombia	La Mesa	Proportion	2017	53.3	65.0	41.2
Colombia	La Mesa	Count	2000	0	0	0
Colombia	La Mesa	Count	2017	1	1	0
Colombia	Samaniego	Proportion	2000	38.3	47.5	30.1
Colombia	Samaniego	Proportion	2017	56.1	67.5	45.4
Colombia	Samaniego	Count	2000	0	0	0
Colombia	Samaniego	Count	2017	1	1	0
Colombia	Lebrija	Proportion	2000	37.1	44.2	30.3
Colombia	Lebrija	Proportion	2017	54.4	67.5	43.0
Colombia	Lebrija	Count	2000	0	0	0
Colombia	Lebrija	Count	2017	1	1	0
Colombia	Giraldo	Proportion	2000	34.8	42.8	26.8
Colombia	Giraldo	Proportion	2017	49.6	62.5	36.2
Colombia	Giraldo	Count	2000	0	0	0
Colombia	Giraldo	Count	2017	0	1	0
Colombia	Labranzagrande	Proportion	2000	30.7	39.4	23.5
Colombia	Labranzagrande	Proportion	2017	45.2	58.4	32.4
Colombia	Labranzagrande	Count	2000	0	0	0
Colombia	Labranzagrande	Count	2017	0	1	0
Colombia	La Palma	Proportion	2000	35.0	40.6	28.2
Colombia	La Palma	Proportion	2017	51.0	63.4	39.6
Colombia	La Palma	Count	2000	0	0	0
Colombia	La Palma	Count	2017	1	1	0
Colombia	San Bernardo	Proportion	2000	35.0	44.2	26.2
Colombia	San Bernardo	Proportion	2017	53.6	65.3	42.6
Colombia	San Bernardo	Count	2000	0	0	0
Colombia	San Bernardo	Count	2017	1	1	0
Colombia	Los Santos	Proportion	2000	36.8	44.1	29.7
Colombia	Los Santos	Proportion	2017	52.8	66.4	40.3
Colombia	Los Santos	Count	2000	0	0	0
Colombia	Los Santos	Count	2017	1	1	0
Colombia	Girardota	Proportion	2000	37.8	45.1	30.4
Colombia	Girardota	Proportion	2017	56.6	68.2	45.0
Colombia	Girardota	Count	2000	0	0	0
Colombia	Girardota	Count	2017	1	1	0
Colombia	Macanal	Proportion	2000	28.0	34.7	21.5

Table 4: ORS by Unit *(continued)*

Country	Unit	Metric	Year	Mean	Upper	Lower
Colombia	Macanal	Proportion	2017	44.0	57.6	31.2
Colombia	Macanal	Count	2000	0	0	0
Colombia	Macanal	Count	2017	0	1	0
Colombia	La Peña	Proportion	2000	36.7	42.5	29.7
Colombia	La Peña	Proportion	2017	52.7	65.2	41.1
Colombia	La Peña	Count	2000	0	0	0
Colombia	La Peña	Count	2017	1	1	0
Colombia	San Juan de Pasto	Proportion	2000	39.0	48.8	30.6
Colombia	San Juan de Pasto	Proportion	2017	58.7	69.4	47.4
Colombia	San Juan de Pasto	Count	2000	0	0	0
Colombia	San Juan de Pasto	Count	2017	1	1	0
Colombia	Macaravita	Proportion	2000	32.7	40.7	26.3
Colombia	Macaravita	Proportion	2017	48.2	62.0	36.8
Colombia	Macaravita	Count	2000	0	0	0
Colombia	Macaravita	Count	2017	0	1	0
Colombia	Gómez Plata	Proportion	2000	35.0	41.7	28.0
Colombia	Gómez Plata	Proportion	2017	47.1	58.6	36.9
Colombia	Gómez Plata	Count	2000	0	0	0
Colombia	Gómez Plata	Count	2017	0	1	0
Colombia	Maripí	Proportion	2000	32.8	39.0	26.4
Colombia	Maripí	Proportion	2017	48.1	61.6	37.0
Colombia	Maripí	Count	2000	0	0	0
Colombia	Maripí	Count	2017	0	1	0
Colombia	La Vega	Proportion	2000	31.8	37.3	25.8
Colombia	La Vega	Proportion	2017	47.9	61.0	36.7
Colombia	La Vega	Count	2000	0	0	0
Colombia	La Vega	Count	2017	0	1	0
Colombia	San Lorenzo	Proportion	2000	37.3	46.7	29.2
Colombia	San Lorenzo	Proportion	2017	54.7	66.5	43.3
Colombia	San Lorenzo	Count	2000	0	0	0
Colombia	San Lorenzo	Count	2017	1	1	0
Colombia	Málaga	Proportion	2000	34.4	42.1	27.3
Colombia	Málaga	Proportion	2017	51.3	65.6	39.1
Colombia	Málaga	Count	2000	0	0	0
Colombia	Málaga	Count	2017	1	1	0
Colombia	Granada	Proportion	2000	33.0	40.4	25.6
Colombia	Granada	Proportion	2017	46.4	58.9	36.2
Colombia	Granada	Count	2000	0	0	0
Colombia	Granada	Count	2017	0	1	0
Colombia	Miraflores	Proportion	2000	28.3	35.5	21.7
Colombia	Miraflores	Proportion	2017	45.6	58.5	32.9
Colombia	Miraflores	Count	2000	0	0	0
Colombia	Miraflores	Count	2017	0	1	0
Colombia	Lenguazaque	Proportion	2000	28.6	34.7	22.9
Colombia	Lenguazaque	Proportion	2017	45.8	60.9	34.1
Colombia	Lenguazaque	Count	2000	0	0	0
Colombia	Lenguazaque	Count	2017	0	1	0
Colombia	San Pablo	Proportion	2000	35.5	44.4	28.0
Colombia	San Pablo	Proportion	2017	56.3	67.1	45.3

Table 4: ORS by Unit *(continued)*

Country	Unit	Metric	Year	Mean	Upper	Lower
Colombia	San Pablo	Count	2000	0	0	0
Colombia	San Pablo	Count	2017	1	1	0
Colombia	Matanza	Proportion	2000	35.5	43.2	28.4
Colombia	Matanza	Proportion	2017	52.9	65.7	41.6
Colombia	Matanza	Count	2000	0	0	0
Colombia	Matanza	Count	2017	1	1	0
Colombia	Guadalupe	Proportion	2000	33.5	40.4	26.1
Colombia	Guadalupe	Proportion	2017	45.4	57.7	34.9
Colombia	Guadalupe	Count	2000	0	0	0
Colombia	Guadalupe	Count	2017	0	1	0
Colombia	Mongua	Proportion	2000	30.9	39.2	23.9
Colombia	Mongua	Proportion	2017	48.2	60.4	35.9
Colombia	Mongua	Count	2000	0	0	0
Colombia	Mongua	Count	2017	0	1	0
Colombia	Machetá	Proportion	2000	29.8	36.8	23.3
Colombia	Machetá	Proportion	2017	46.8	61.7	35.2
Colombia	Machetá	Count	2000	0	0	0
Colombia	Machetá	Count	2017	0	1	0
Colombia	San Pedro de Cartago	Proportion	2000	34.5	43.4	25.9
Colombia	San Pedro de Cartago	Proportion	2017	53.8	65.3	42.0
Colombia	San Pedro de Cartago	Count	2000	0	0	0
Colombia	San Pedro de Cartago	Count	2017	1	1	0
Colombia	Mogotes	Proportion	2000	35.1	43.0	27.8
Colombia	Mogotes	Proportion	2017	50.2	63.8	38.7
Colombia	Mogotes	Count	2000	0	0	0
Colombia	Mogotes	Count	2017	1	1	0
Colombia	Guarne	Proportion	2000	37.3	44.6	29.8
Colombia	Guarne	Proportion	2017	55.9	67.3	44.5
Colombia	Guarne	Count	2000	0	0	0
Colombia	Guarne	Count	2017	1	1	0
Colombia	Monguí	Proportion	2000	30.4	38.2	23.9
Colombia	Monguí	Proportion	2017	50.2	62.9	38.1
Colombia	Monguí	Count	2000	0	0	0
Colombia	Monguí	Count	2017	1	1	0
Colombia	Madrid	Proportion	2000	31.5	37.3	26.1
Colombia	Madrid	Proportion	2017	50.7	63.2	38.9
Colombia	Madrid	Count	2000	0	0	0
Colombia	Madrid	Count	2017	1	1	0
Colombia	Sandoná	Proportion	2000	38.1	47.5	30.0
Colombia	Sandoná	Proportion	2017	58.9	70.0	47.6
Colombia	Sandoná	Count	2000	0	0	0
Colombia	Sandoná	Count	2017	1	1	0
Colombia	Molagavita	Proportion	2000	35.1	42.3	27.9
Colombia	Molagavita	Proportion	2017	50.9	65.1	39.0
Colombia	Molagavita	Count	2000	0	0	0
Colombia	Molagavita	Count	2017	1	1	0
Colombia	Guatapé	Proportion	2000	31.5	38.6	24.7
Colombia	Guatapé	Proportion	2017	44.9	57.1	35.0
Colombia	Guatapé	Count	2000	0	0	0

Table 4: ORS by Unit *(continued)*

Country	Unit	Metric	Year	Mean	Upper	Lower
Colombia	Guatapé	Count	2017	0	1	0
Colombia	Moniquirá	Proportion	2000	34.6	42.4	26.8
Colombia	Moniquirá	Proportion	2017	55.0	68.1	43.6
Colombia	Moniquirá	Count	2000	0	0	0
Colombia	Moniquirá	Count	2017	1	1	0
Colombia	Manta	Proportion	2000	29.3	36.1	23.1
Colombia	Manta	Proportion	2017	45.7	60.4	33.9
Colombia	Manta	Count	2000	0	0	0
Colombia	Manta	Count	2017	0	1	0
Colombia	Santa Bárbara	Proportion	2000	35.4	42.5	26.8
Colombia	Santa Bárbara	Proportion	2017	43.4	53.7	31.8
Colombia	Santa Bárbara	Count	2000	0	0	0
Colombia	Santa Bárbara	Count	2017	0	1	0
Colombia	Ocamonte	Proportion	2000	33.0	41.3	26.1
Colombia	Ocamonte	Proportion	2017	46.6	60.1	35.4
Colombia	Ocamonte	Count	2000	0	0	0
Colombia	Ocamonte	Count	2017	0	1	0
Colombia	Heliconia	Proportion	2000	38.4	44.3	30.4
Colombia	Heliconia	Proportion	2017	54.9	67.6	43.2
Colombia	Heliconia	Count	2000	0	0	0
Colombia	Heliconia	Count	2017	1	1	0
Colombia	Motavita	Proportion	2000	29.7	37.2	23.1
Colombia	Motavita	Proportion	2017	48.6	62.9	37.2
Colombia	Motavita	Count	2000	0	0	0
Colombia	Motavita	Count	2017	0	1	0
Colombia	Medina	Proportion	2000	29.0	36.1	22.7
Colombia	Medina	Proportion	2017	42.4	54.9	31.1
Colombia	Medina	Count	2000	0	0	0
Colombia	Medina	Count	2017	0	1	0
Colombia	Santa Cruz	Proportion	2000	37.6	47.0	29.2
Colombia	Santa Cruz	Proportion	2017	52.9	64.3	42.1
Colombia	Santa Cruz	Count	2000	0	0	0
Colombia	Santa Cruz	Count	2017	1	1	0
Colombia	Oiba	Proportion	2000	34.4	42.8	26.5
Colombia	Oiba	Proportion	2017	50.4	63.2	39.6
Colombia	Oiba	Count	2000	0	0	0
Colombia	Oiba	Count	2017	1	1	0
Colombia	Hispania	Proportion	2000	35.6	43.9	28.2
Colombia	Hispania	Proportion	2017	50.8	63.8	39.4
Colombia	Hispania	Count	2000	0	0	0
Colombia	Hispania	Count	2017	1	1	0
Colombia	Muzo	Proportion	2000	33.4	39.3	26.7
Colombia	Muzo	Proportion	2017	47.1	60.6	36.4
Colombia	Muzo	Count	2000	0	0	0
Colombia	Muzo	Count	2017	0	1	0
Colombia	Mosquera	Proportion	2000	33.1	38.8	28.0
Colombia	Mosquera	Proportion	2017	53.2	65.6	40.6
Colombia	Mosquera	Count	2000	0	0	0
Colombia	Mosquera	Count	2017	1	1	0

Table 4: ORS by Unit *(continued)*

Country	Unit	Metric	Year	Mean	Upper	Lower
Colombia	Sapuyes	Proportion	2000	35.7	45.7	26.8
Colombia	Sapuyes	Proportion	2017	56.2	67.5	45.2
Colombia	Sapuyes	Count	2000	0	0	0
Colombia	Sapuyes	Count	2017	1	1	0
Colombia	Onzaga	Proportion	2000	30.7	37.7	24.3
Colombia	Onzaga	Proportion	2017	46.3	60.0	34.5
Colombia	Onzaga	Count	2000	0	0	0
Colombia	Onzaga	Count	2017	0	1	0
Colombia	Itagüí	Proportion	2000	39.5	46.1	31.7
Colombia	Itagüí	Proportion	2017	53.1	65.9	41.4
Colombia	Itagüí	Count	2000	0	0	0
Colombia	Itagüí	Count	2017	1	1	0
Colombia	Nobsa	Proportion	2000	30.6	38.8	23.5
Colombia	Nobsa	Proportion	2017	46.1	59.9	34.2
Colombia	Nobsa	Count	2000	0	0	0
Colombia	Nobsa	Count	2017	0	1	0
Colombia	Nariño	Proportion	2000	37.2	43.2	31.0
Colombia	Nariño	Proportion	2017	53.0	65.4	40.9
Colombia	Nariño	Count	2000	0	0	0
Colombia	Nariño	Count	2017	1	1	0
Colombia	Taminango	Proportion	2000	39.2	48.2	30.5
Colombia	Taminango	Proportion	2017	54.2	66.3	42.4
Colombia	Taminango	Count	2000	0	0	0
Colombia	Taminango	Count	2017	1	1	0
Colombia	Palmar	Proportion	2000	35.3	43.2	28.2
Colombia	Palmar	Proportion	2017	50.7	63.1	39.3
Colombia	Palmar	Count	2000	0	0	0
Colombia	Palmar	Count	2017	1	1	0
Colombia	Ituango	Proportion	2000	36.6	44.0	28.1
Colombia	Ituango	Proportion	2017	48.2	60.1	38.2
Colombia	Ituango	Count	2000	0	0	0
Colombia	Ituango	Count	2017	0	1	0
Colombia	Nuevo Colón	Proportion	2000	26.3	33.1	20.3
Colombia	Nuevo Colón	Proportion	2017	45.5	60.0	33.1
Colombia	Nuevo Colón	Count	2000	0	0	0
Colombia	Nuevo Colón	Count	2017	0	1	0
Colombia	Nemocón	Proportion	2000	30.1	35.9	24.3
Colombia	Nemocón	Proportion	2017	46.1	61.7	33.5
Colombia	Nemocón	Count	2000	0	0	0
Colombia	Nemocón	Count	2017	0	1	0
Colombia	Tangua	Proportion	2000	36.8	46.8	28.0
Colombia	Tangua	Proportion	2017	54.7	65.7	42.7
Colombia	Tangua	Count	2000	0	0	0
Colombia	Tangua	Count	2017	1	1	0
Colombia	Palmas del Socorro	Proportion	2000	34.7	42.8	26.7
Colombia	Palmas del Socorro	Proportion	2017	51.8	64.4	40.8
Colombia	Palmas del Socorro	Count	2000	0	0	0
Colombia	Palmas del Socorro	Count	2017	1	1	0
Colombia	Jardín	Proportion	2000	36.0	44.5	28.1

Table 4: ORS by Unit *(continued)*

Country	Unit	Metric	Year	Mean	Upper	Lower
Colombia	Jardín	Proportion	2017	50.6	62.7	40.1
Colombia	Jardín	Count	2000	0	0	0
Colombia	Jardín	Count	2017	1	1	0
Colombia	Oicatá	Proportion	2000	28.7	36.6	22.3
Colombia	Oicatá	Proportion	2017	52.9	66.7	41.3
Colombia	Oicatá	Count	2000	0	0	0
Colombia	Oicatá	Count	2017	1	1	0
Colombia	Nilo	Proportion	2000	36.5	42.9	30.1
Colombia	Nilo	Proportion	2017	52.8	64.9	41.1
Colombia	Nilo	Count	2000	0	0	0
Colombia	Nilo	Count	2017	1	1	0
Colombia	Tumaco	Proportion	2000	38.0	47.4	28.9
Colombia	Tumaco	Proportion	2017	48.1	60.2	37.8
Colombia	Tumaco	Count	2000	0	0	0
Colombia	Tumaco	Count	2017	0	1	0
Colombia	Páramo	Proportion	2000	34.5	42.3	27.1
Colombia	Páramo	Proportion	2017	49.9	62.9	39.0
Colombia	Páramo	Count	2000	0	0	0
Colombia	Páramo	Count	2017	0	1	0
Colombia	Jericó	Proportion	2000	36.3	44.2	28.6
Colombia	Jericó	Proportion	2017	50.0	62.5	38.7
Colombia	Jericó	Count	2000	0	0	0
Colombia	Jericó	Count	2017	0	1	0
Colombia	Otanche	Proportion	2000	33.2	39.7	26.2
Colombia	Otanche	Proportion	2017	47.5	60.5	36.1
Colombia	Otanche	Count	2000	0	0	0
Colombia	Otanche	Count	2017	0	1	0
Colombia	Nimaima	Proportion	2000	36.2	42.1	29.3
Colombia	Nimaima	Proportion	2017	52.0	64.4	40.6
Colombia	Nimaima	Count	2000	0	0	0
Colombia	Nimaima	Count	2017	1	1	0
Colombia	Túquerres	Proportion	2000	34.8	44.7	26.0
Colombia	Túquerres	Proportion	2017	56.1	67.1	45.2
Colombia	Túquerres	Count	2000	0	0	0
Colombia	Túquerres	Count	2017	1	1	0
Colombia	Piedecuesta	Proportion	2000	36.3	42.8	29.3
Colombia	Piedecuesta	Proportion	2017	55.2	67.6	43.1
Colombia	Piedecuesta	Count	2000	0	0	0
Colombia	Piedecuesta	Count	2017	1	1	0
Colombia	La Ceja	Proportion	2000	37.7	44.9	29.9
Colombia	La Ceja	Proportion	2017	55.5	67.6	44.5
Colombia	La Ceja	Count	2000	0	0	0
Colombia	La Ceja	Count	2017	1	1	0
Colombia	Pachavita	Proportion	2000	30.3	37.6	24.1
Colombia	Pachavita	Proportion	2017	49.5	63.3	37.6
Colombia	Pachavita	Count	2000	0	0	0
Colombia	Pachavita	Count	2017	0	1	0
Colombia	Nocaima	Proportion	2000	35.2	41.0	28.4
Colombia	Nocaima	Proportion	2017	51.2	63.7	39.8

Table 4: ORS by Unit *(continued)*

Country	Unit	Metric	Year	Mean	Upper	Lower
Colombia	Nocaima	Count	2000	0	0	0
Colombia	Nocaima	Count	2017	1	1	0
Colombia	Yacuanquer	Proportion	2000	36.7	46.5	28.2
Colombia	Yacuanquer	Proportion	2017	54.1	65.4	41.8
Colombia	Yacuanquer	Count	2000	0	0	0
Colombia	Yacuanquer	Count	2017	1	1	0
Colombia	Pinchote	Proportion	2000	35.5	43.2	28.2
Colombia	Pinchote	Proportion	2017	54.6	66.4	42.9
Colombia	Pinchote	Count	2000	0	0	0
Colombia	Pinchote	Count	2017	1	1	0
Colombia	La Estrella	Proportion	2000	37.3	43.9	29.7
Colombia	La Estrella	Proportion	2017	55.7	68.2	43.8
Colombia	La Estrella	Count	2000	0	0	0
Colombia	La Estrella	Count	2017	1	1	0
Colombia	Páez	Proportion	2000	29.6	36.8	22.9
Colombia	Páez	Proportion	2017	42.1	55.1	30.1
Colombia	Páez	Count	2000	0	0	0
Colombia	Páez	Count	2017	0	1	0
Colombia	Pacho	Proportion	2000	31.6	37.0	25.3
Colombia	Pacho	Proportion	2017	47.7	62.2	35.7
Colombia	Pacho	Count	2000	0	0	0
Colombia	Pacho	Count	2017	0	1	0
Colombia	Puente Nacional	Proportion	2000	33.9	41.3	26.6
Colombia	Puente Nacional	Proportion	2017	51.5	65.5	40.1
Colombia	Puente Nacional	Count	2000	0	0	0
Colombia	Puente Nacional	Count	2017	1	1	0
Colombia	La Unión de Sucre	Proportion	2000	34.2	42.3	27.0
Colombia	La Unión de Sucre	Proportion	2017	48.4	60.8	38.2
Colombia	La Unión de Sucre	Count	2000	0	0	0
Colombia	La Unión de Sucre	Count	2017	0	1	0
Colombia	Paipa	Proportion	2000	29.4	37.7	23.2
Colombia	Paipa	Proportion	2017	52.7	66.2	40.4
Colombia	Paipa	Count	2000	0	0	0
Colombia	Paipa	Count	2017	1	1	0
Colombia	Paime	Proportion	2000	34.1	39.4	27.6
Colombia	Paime	Proportion	2017	47.6	61.6	36.8
Colombia	Paime	Count	2000	0	0	0
Colombia	Paime	Count	2017	0	1	0
Colombia	Puerto Parra	Proportion	2000	35.1	43.3	26.9
Colombia	Puerto Parra	Proportion	2017	49.9	62.3	39.1
Colombia	Puerto Parra	Count	2000	0	0	0
Colombia	Puerto Parra	Count	2017	0	1	0
Colombia	Liborina	Proportion	2000	37.1	44.8	29.0
Colombia	Liborina	Proportion	2017	54.3	66.7	41.2
Colombia	Liborina	Count	2000	0	0	0
Colombia	Liborina	Count	2017	1	1	0
Colombia	Pajarito	Proportion	2000	30.6	39.0	23.3
Colombia	Pajarito	Proportion	2017	44.2	57.1	31.4
Colombia	Pajarito	Count	2000	0	0	0

Table 4: ORS by Unit *(continued)*

Country	Unit	Metric	Year	Mean	Upper	Lower
Colombia	Pajarito	Count	2017	0	1	0
Colombia	Pandi	Proportion	2000	35.3	42.3	28.7
Colombia	Pandi	Proportion	2017	50.5	61.9	39.1
Colombia	Pandi	Count	2000	0	0	0
Colombia	Pandi	Count	2017	1	1	0
Colombia	Puerto Wilches	Proportion	2000	38.6	45.9	30.4
Colombia	Puerto Wilches	Proportion	2017	54.4	66.0	43.4
Colombia	Puerto Wilches	Count	2000	0	0	0
Colombia	Puerto Wilches	Count	2017	1	1	0
Colombia	Maceo	Proportion	2000	38.4	45.1	29.9
Colombia	Maceo	Proportion	2017	53.4	64.9	42.7
Colombia	Maceo	Count	2000	0	0	0
Colombia	Maceo	Count	2017	1	1	0
Colombia	Panqueba	Proportion	2000	30.1	37.9	23.3
Colombia	Panqueba	Proportion	2017	49.5	63.1	37.3
Colombia	Panqueba	Count	2000	0	0	0
Colombia	Panqueba	Count	2017	0	1	0
Colombia	Paratebueno	Proportion	2000	30.9	39.3	23.8
Colombia	Paratebueno	Proportion	2017	44.2	57.7	32.5
Colombia	Paratebueno	Count	2000	0	0	0
Colombia	Paratebueno	Count	2017	0	1	0
Colombia	Rionegro	Proportion	2000	36.5	43.5	29.2
Colombia	Rionegro	Proportion	2017	52.4	65.0	40.8
Colombia	Rionegro	Count	2000	0	0	0
Colombia	Rionegro	Count	2017	1	1	0
Colombia	Marinilla	Proportion	2000	35.8	43.1	28.2
Colombia	Marinilla	Proportion	2017	52.6	64.3	42.5
Colombia	Marinilla	Count	2000	0	0	0
Colombia	Marinilla	Count	2017	1	1	0
Colombia	Pauna	Proportion	2000	32.2	38.1	25.4
Colombia	Pauna	Proportion	2017	47.9	61.1	36.7
Colombia	Pauna	Count	2000	0	0	0
Colombia	Pauna	Count	2017	0	1	0
Colombia	Pasca	Proportion	2000	31.2	37.6	25.6
Colombia	Pasca	Proportion	2017	47.9	60.0	35.7
Colombia	Pasca	Count	2000	0	0	0
Colombia	Pasca	Count	2017	0	1	0
Colombia	Sabana de Torres	Proportion	2000	36.4	43.7	28.6
Colombia	Sabana de Torres	Proportion	2017	50.6	62.0	39.7
Colombia	Sabana de Torres	Count	2000	0	0	0
Colombia	Sabana de Torres	Count	2017	1	1	0
Colombia	Medellín	Proportion	2000	38.8	45.5	30.9
Colombia	Medellín	Proportion	2017	51.5	64.5	39.1
Colombia	Medellín	Count	2000	0	0	0
Colombia	Medellín	Count	2017	1	1	0
Colombia	Paya	Proportion	2000	32.3	41.0	24.7
Colombia	Paya	Proportion	2017	43.5	56.8	31.1
Colombia	Paya	Count	2000	0	0	0
Colombia	Paya	Count	2017	0	1	0

Table 4: ORS by Unit *(continued)*

Country	Unit	Metric	Year	Mean	Upper	Lower
Colombia	Puerto Salgar	Proportion	2000	35.9	42.5	28.7
Colombia	Puerto Salgar	Proportion	2017	55.0	66.7	43.3
Colombia	Puerto Salgar	Count	2000	0	0	0
Colombia	Puerto Salgar	Count	2017	1	1	0
Colombia	San Andrés de Cuerquia	Proportion	2000	33.2	40.3	25.8
Colombia	San Andrés de Cuerquia	Proportion	2017	48.7	63.1	36.8
Colombia	San Andrés de Cuerquia	Count	2000	0	0	0
Colombia	San Andrés de Cuerquia	Count	2017	0	1	0
Colombia	Montebello	Proportion	2000	35.7	43.0	28.2
Colombia	Montebello	Proportion	2017	49.8	62.9	39.1
Colombia	Montebello	Count	2000	0	0	0
Colombia	Montebello	Count	2017	0	1	0
Colombia	Paz de Río	Proportion	2000	28.6	36.6	22.3
Colombia	Paz de Río	Proportion	2017	50.7	64.0	38.5
Colombia	Paz de Río	Count	2000	0	0	0
Colombia	Paz de Río	Count	2017	1	1	0
Colombia	Pulí	Proportion	2000	36.6	42.7	30.4
Colombia	Pulí	Proportion	2017	50.6	63.7	38.7
Colombia	Pulí	Count	2000	0	0	0
Colombia	Pulí	Count	2017	1	1	0
Colombia	San Benito	Proportion	2000	34.0	42.3	25.9
Colombia	San Benito	Proportion	2017	47.6	61.1	36.3
Colombia	San Benito	Count	2000	0	0	0
Colombia	San Benito	Count	2017	0	1	0
Colombia	Murindó	Proportion	2000	35.6	44.8	27.4
Colombia	Murindó	Proportion	2017	44.8	55.5	34.2
Colombia	Murindó	Count	2000	0	0	0
Colombia	Murindó	Count	2017	0	1	0
Colombia	Pesca	Proportion	2000	27.5	34.7	21.5
Colombia	Pesca	Proportion	2017	45.2	60.0	33.0
Colombia	Pesca	Count	2000	0	0	0
Colombia	Pesca	Count	2017	0	1	0
Colombia	Quebradanegra	Proportion	2000	33.3	39.5	26.6
Colombia	Quebradanegra	Proportion	2017	48.7	60.8	37.5
Colombia	Quebradanegra	Count	2000	0	0	0
Colombia	Quebradanegra	Count	2017	0	1	0
Colombia	San Gil	Proportion	2000	35.7	43.0	28.3
Colombia	San Gil	Proportion	2017	56.0	67.9	44.4
Colombia	San Gil	Count	2000	0	0	0
Colombia	San Gil	Count	2017	1	1	0
Colombia	Mutatá	Proportion	2000	34.3	42.5	25.8
Colombia	Mutatá	Proportion	2017	44.8	56.1	33.5
Colombia	Mutatá	Count	2000	0	0	0
Colombia	Mutatá	Count	2017	0	1	0
Colombia	Pisba	Proportion	2000	30.3	38.8	23.5
Colombia	Pisba	Proportion	2017	44.2	57.8	31.7
Colombia	Pisba	Count	2000	0	0	0
Colombia	Pisba	Count	2017	0	1	0
Colombia	Quetame	Proportion	2000	28.6	34.9	22.8

Table 4: ORS by Unit *(continued)*

Country	Unit	Metric	Year	Mean	Upper	Lower
Colombia	Quetame	Proportion	2017	44.9	58.8	32.7
Colombia	Quetame	Count	2000	0	0	0
Colombia	Quetame	Count	2017	0	1	0
Colombia	San Joaquín	Proportion	2000	31.9	38.9	25.6
Colombia	San Joaquín	Proportion	2017	47.0	60.7	35.5
Colombia	San Joaquín	Count	2000	0	0	0
Colombia	San Joaquín	Count	2017	0	1	0
Colombia	Nariño	Proportion	2000	34.8	41.8	27.5
Colombia	Nariño	Proportion	2017	49.0	60.2	39.2
Colombia	Nariño	Count	2000	0	0	0
Colombia	Nariño	Count	2017	0	1	0
Colombia	Puerto Boyacá	Proportion	2000	36.6	43.7	28.6
Colombia	Puerto Boyacá	Proportion	2017	53.7	65.4	41.9
Colombia	Puerto Boyacá	Count	2000	0	0	0
Colombia	Puerto Boyacá	Count	2017	1	1	0
Colombia	Quipile	Proportion	2000	33.3	38.9	27.6
Colombia	Quipile	Proportion	2017	48.0	61.6	36.5
Colombia	Quipile	Count	2000	0	0	0
Colombia	Quipile	Count	2017	0	1	0
Colombia	San José de Miranda	Proportion	2000	33.4	40.8	26.3
Colombia	San José de Miranda	Proportion	2017	48.4	62.5	36.4
Colombia	San José de Miranda	Count	2000	0	0	0
Colombia	San José de Miranda	Count	2017	0	1	0
Colombia	Nechí	Proportion	2000	39.1	47.5	30.9
Colombia	Nechí	Proportion	2017	52.4	62.5	41.1
Colombia	Nechí	Count	2000	0	0	0
Colombia	Nechí	Count	2017	1	1	0
Colombia	Quípama	Proportion	2000	34.1	40.4	27.3
Colombia	Quípama	Proportion	2017	48.1	61.7	37.4
Colombia	Quípama	Count	2000	0	0	0
Colombia	Quípama	Count	2017	0	1	0
Colombia	Ricaurte	Proportion	2000	35.8	41.9	29.4
Colombia	Ricaurte	Proportion	2017	52.0	64.4	40.3
Colombia	Ricaurte	Count	2000	0	0	0
Colombia	Ricaurte	Count	2017	1	1	0
Colombia	San Miguel	Proportion	2000	35.0	42.5	28.0
Colombia	San Miguel	Proportion	2017	51.3	65.2	39.9
Colombia	San Miguel	Count	2000	0	0	0
Colombia	San Miguel	Count	2017	1	1	0
Colombia	Necoclí	Proportion	2000	39.5	48.5	30.8
Colombia	Necoclí	Proportion	2017	52.9	64.5	42.0
Colombia	Necoclí	Count	2000	0	0	0
Colombia	Necoclí	Count	2017	1	1	0
Colombia	Ramiriquí	Proportion	2000	29.2	36.2	23.0
Colombia	Ramiriquí	Proportion	2017	51.3	64.7	39.6
Colombia	Ramiriquí	Count	2000	0	0	0
Colombia	Ramiriquí	Count	2017	1	1	0
Colombia	San Antonio del Tequendama	Proportion	2000	33.0	38.9	27.7

Table 4: ORS by Unit *(continued)*

Country	Unit	Metric	Year	Mean	Upper	Lower
Colombia	San Antonio del Tequendama	Proportion	2017	49.3	61.0	37.2
Colombia	San Antonio del Tequendama	Count	2000	0	0	0
Colombia	San Antonio del Tequendama	Count	2017	0	1	0
Colombia	San Vicente de Chucurí	Proportion	2000	35.9	43.9	28.1
Colombia	San Vicente de Chucurí	Proportion	2017	53.0	65.0	42.0
Colombia	San Vicente de Chucurí	Count	2000	0	0	0
Colombia	San Vicente de Chucurí	Count	2017	1	1	0
Colombia	Olaya	Proportion	2000	40.4	47.5	32.1
Colombia	Olaya	Proportion	2017	57.0	69.6	43.3
Colombia	Olaya	Count	2000	0	0	0
Colombia	Olaya	Count	2017	1	1	0
Colombia	Ráquira	Proportion	2000	27.8	34.5	22.0
Colombia	Ráquira	Proportion	2017	48.1	62.4	36.7
Colombia	Ráquira	Count	2000	0	0	0
Colombia	Ráquira	Count	2017	0	1	0
Colombia	San Bernardo	Proportion	2000	31.9	38.7	25.2
Colombia	San Bernardo	Proportion	2017	48.3	60.0	36.7
Colombia	San Bernardo	Count	2000	0	0	0
Colombia	San Bernardo	Count	2017	0	1	0
Colombia	Santa Bárbara	Proportion	2000	35.4	42.0	28.1
Colombia	Santa Bárbara	Proportion	2017	51.7	64.4	39.4
Colombia	Santa Bárbara	Count	2000	0	0	0
Colombia	Santa Bárbara	Count	2017	1	1	0
Colombia	Peñol	Proportion	2000	32.0	39.0	24.8
Colombia	Peñol	Proportion	2017	47.1	58.9	37.1
Colombia	Peñol	Count	2000	0	0	0
Colombia	Peñol	Count	2017	0	1	0
Colombia	Rondón	Proportion	2000	28.4	35.7	22.0
Colombia	Rondón	Proportion	2017	45.8	60.3	33.8
Colombia	Rondón	Count	2000	0	0	0
Colombia	Rondón	Count	2017	0	1	0
Colombia	San Cayetano	Proportion	2000	31.4	36.5	25.2
Colombia	San Cayetano	Proportion	2017	46.8	62.0	35.3
Colombia	San Cayetano	Count	2000	0	0	0
Colombia	San Cayetano	Count	2017	0	1	0
Colombia	Santa Helena del Opón	Proportion	2000	33.0	40.6	25.5
Colombia	Santa Helena del Opón	Proportion	2017	46.2	58.7	35.5
Colombia	Santa Helena del Opón	Count	2000	0	0	0
Colombia	Santa Helena del Opón	Count	2017	0	1	0
Colombia	Pequé	Proportion	2000	36.6	44.0	28.6
Colombia	Pequé	Proportion	2017	51.2	63.4	39.6
Colombia	Pequé	Count	2000	0	0	0
Colombia	Pequé	Count	2017	1	1	0
Colombia	Saboyá	Proportion	2000	29.3	35.7	22.6
Colombia	Saboyá	Proportion	2017	49.8	63.7	38.5
Colombia	Saboyá	Count	2000	0	0	0
Colombia	Saboyá	Count	2017	0	1	0

Table 4: ORS by Unit *(continued)*

Country	Unit	Metric	Year	Mean	Upper	Lower
Colombia	San Francisco	Proportion	2000	30.9	36.5	25.1
Colombia	San Francisco	Proportion	2017	47.7	61.1	36.5
Colombia	San Francisco	Count	2000	0	0	0
Colombia	San Francisco	Count	2017	0	1	0
Colombia	Simacota	Proportion	2000	34.6	42.1	27.0
Colombia	Simacota	Proportion	2017	48.8	61.4	38.2
Colombia	Simacota	Count	2000	0	0	0
Colombia	Simacota	Count	2017	0	1	0
Colombia	Pueblorrico	Proportion	2000	36.4	44.6	28.5
Colombia	Pueblorrico	Proportion	2017	51.2	63.8	39.3
Colombia	Pueblorrico	Count	2000	0	0	0
Colombia	Pueblorrico	Count	2017	1	1	0
Colombia	Sáchica	Proportion	2000	26.6	33.6	20.5
Colombia	Sáchica	Proportion	2017	45.2	59.5	34.1
Colombia	Sáchica	Count	2000	0	0	0
Colombia	Sáchica	Count	2017	0	1	0
Colombia	San Juan de Río Seco	Proportion	2000	35.9	41.9	29.8
Colombia	San Juan de Río Seco	Proportion	2017	51.6	64.8	39.6
Colombia	San Juan de Río Seco	Count	2000	0	0	0
Colombia	San Juan de Río Seco	Count	2017	1	1	0
Colombia	Socorro	Proportion	2000	36.3	44.3	28.7
Colombia	Socorro	Proportion	2017	57.4	69.8	45.7
Colombia	Socorro	Count	2000	0	0	0
Colombia	Socorro	Count	2017	1	1	0
Colombia	Puerto Berrío	Proportion	2000	38.7	46.1	30.1
Colombia	Puerto Berrío	Proportion	2017	55.3	67.8	43.9
Colombia	Puerto Berrío	Count	2000	0	0	0
Colombia	Puerto Berrío	Count	2017	1	1	0
Colombia	Samacá	Proportion	2000	28.0	34.8	22.2
Colombia	Samacá	Proportion	2017	45.5	59.6	34.3
Colombia	Samacá	Count	2000	0	0	0
Colombia	Samacá	Count	2017	0	1	0
Colombia	Santafé de Bogotá	Proportion	2000	35.6	41.5	30.1
Colombia	Santafé de Bogotá	Proportion	2017	48.8	61.5	37.2
Colombia	Santafé de Bogotá	Count	2000	0	0	0
Colombia	Santafé de Bogotá	Count	2017	0	1	0
Colombia	Suaita	Proportion	2000	34.5	42.9	26.1
Colombia	Suaita	Proportion	2017	49.0	62.5	38.0
Colombia	Suaita	Count	2000	0	0	0
Colombia	Suaita	Count	2017	0	1	0
Colombia	Puerto Nare	Proportion	2000	38.3	45.6	29.8
Colombia	Puerto Nare	Proportion	2017	54.0	65.6	42.5
Colombia	Puerto Nare	Count	2000	0	0	0
Colombia	Puerto Nare	Count	2017	1	1	0
Colombia	San Eduardo	Proportion	2000	30.3	38.0	23.5
Colombia	San Eduardo	Proportion	2017	47.0	59.8	35.0
Colombia	San Eduardo	Count	2000	0	0	0
Colombia	San Eduardo	Count	2017	0	1	0
Colombia	Sasaima	Proportion	2000	32.0	37.6	26.0

Table 4: ORS by Unit *(continued)*

Country	Unit	Metric	Year	Mean	Upper	Lower
Colombia	Sasaima	Proportion	2017	48.1	61.1	36.8
Colombia	Sasaima	Count	2000	0	0	0
Colombia	Sasaima	Count	2017	0	1	0
Colombia	Sucre	Proportion	2000	31.8	38.9	24.1
Colombia	Sucre	Proportion	2017	44.5	58.0	33.7
Colombia	Sucre	Count	2000	0	0	0
Colombia	Sucre	Count	2017	0	1	0
Colombia	Puerto Triunfo	Proportion	2000	36.2	43.1	28.3
Colombia	Puerto Triunfo	Proportion	2017	53.9	65.2	42.0
Colombia	Puerto Triunfo	Count	2000	0	0	0
Colombia	Puerto Triunfo	Count	2017	1	1	0
Colombia	San José de Pare	Proportion	2000	33.1	41.2	25.5
Colombia	San José de Pare	Proportion	2017	52.5	65.6	41.0
Colombia	San José de Pare	Count	2000	0	0	0
Colombia	San José de Pare	Count	2017	1	1	0
Colombia	Sesquilé	Proportion	2000	29.2	35.3	23.3
Colombia	Sesquilé	Proportion	2017	46.9	62.1	34.7
Colombia	Sesquilé	Count	2000	0	0	0
Colombia	Sesquilé	Count	2017	0	1	0
Colombia	Suratá	Proportion	2000	35.4	42.7	27.3
Colombia	Suratá	Proportion	2017	47.8	61.1	36.2
Colombia	Suratá	Count	2000	0	0	0
Colombia	Suratá	Count	2017	0	1	0
Colombia	Remedios	Proportion	2000	36.1	43.1	29.0
Colombia	Remedios	Proportion	2017	49.4	59.8	37.5
Colombia	Remedios	Count	2000	0	0	0
Colombia	Remedios	Count	2017	0	1	0
Colombia	San Luis de Gaceno	Proportion	2000	33.1	40.7	25.9
Colombia	San Luis de Gaceno	Proportion	2017	44.9	57.0	33.4
Colombia	San Luis de Gaceno	Count	2000	0	0	0
Colombia	San Luis de Gaceno	Count	2017	0	1	0
Colombia	Sibaté	Proportion	2000	32.9	38.6	27.5
Colombia	Sibaté	Proportion	2017	52.3	64.8	39.5
Colombia	Sibaté	Count	2000	0	0	0
Colombia	Sibaté	Count	2017	1	1	0
Colombia	Tona	Proportion	2000	36.8	43.7	29.6
Colombia	Tona	Proportion	2017	54.1	66.0	41.8
Colombia	Tona	Count	2000	0	0	0
Colombia	Tona	Count	2017	1	1	0
Colombia	Retiro	Proportion	2000	38.7	45.5	31.2
Colombia	Retiro	Proportion	2017	52.1	64.3	40.5
Colombia	Retiro	Count	2000	0	0	0
Colombia	Retiro	Count	2017	1	1	0
Colombia	San Mateo	Proportion	2000	30.4	38.2	24.1
Colombia	San Mateo	Proportion	2017	49.2	62.9	36.8
Colombia	San Mateo	Count	2000	0	0	0
Colombia	San Mateo	Count	2017	0	1	0
Colombia	Silvania	Proportion	2000	32.9	38.7	27.0
Colombia	Silvania	Proportion	2017	50.4	61.9	38.4

Table 4: ORS by Unit *(continued)*

Country	Unit	Metric	Year	Mean	Upper	Lower
Colombia	Silvania	Count	2000	0	0	0
Colombia	Silvania	Count	2017	1	1	0
Colombia	Valle de San José	Proportion	2000	34.8	42.9	27.4
Colombia	Valle de San José	Proportion	2017	49.6	63.0	38.1
Colombia	Valle de San José	Count	2000	0	0	0
Colombia	Valle de San José	Count	2017	0	1	0
Colombia	Rionegro	Proportion	2000	37.2	44.4	29.5
Colombia	Rionegro	Proportion	2017	54.8	66.6	44.0
Colombia	Rionegro	Count	2000	0	0	0
Colombia	Rionegro	Count	2017	1	1	0
Colombia	San Miguel de Sema	Proportion	2000	29.2	35.3	23.0
Colombia	San Miguel de Sema	Proportion	2017	49.6	63.1	38.1
Colombia	San Miguel de Sema	Count	2000	0	0	0
Colombia	San Miguel de Sema	Count	2017	0	1	0
Colombia	Simijaca	Proportion	2000	28.1	34.0	22.0
Colombia	Simijaca	Proportion	2017	46.0	59.9	34.8
Colombia	Simijaca	Count	2000	0	0	0
Colombia	Simijaca	Count	2017	0	1	0
Colombia	Vélez	Proportion	2000	32.8	40.1	25.0
Colombia	Vélez	Proportion	2017	49.3	62.5	38.1
Colombia	Vélez	Count	2000	0	0	0
Colombia	Vélez	Count	2017	0	1	0
Colombia	Sabanalarga	Proportion	2000	38.5	45.5	30.3
Colombia	Sabanalarga	Proportion	2017	57.6	68.8	45.5
Colombia	Sabanalarga	Count	2000	0	0	0
Colombia	Sabanalarga	Count	2017	1	1	0
Colombia	San Pablo de Borbur	Proportion	2000	31.8	38.1	25.1
Colombia	San Pablo de Borbur	Proportion	2017	46.9	59.5	35.9
Colombia	San Pablo de Borbur	Count	2000	0	0	0
Colombia	San Pablo de Borbur	Count	2017	0	1	0
Colombia	Soacha	Proportion	2000	33.5	39.4	28.3
Colombia	Soacha	Proportion	2017	51.2	63.4	38.6
Colombia	Soacha	Count	2000	0	0	0
Colombia	Soacha	Count	2017	1	1	0
Colombia	Vetas	Proportion	2000	35.2	43.3	27.9
Colombia	Vetas	Proportion	2017	49.0	62.9	37.8
Colombia	Vetas	Count	2000	0	0	0
Colombia	Vetas	Count	2017	0	1	0
Colombia	Sabaneta	Proportion	2000	38.8	45.5	31.2
Colombia	Sabaneta	Proportion	2017	54.9	67.5	42.8
Colombia	Sabaneta	Count	2000	0	0	0
Colombia	Sabaneta	Count	2017	1	1	0
Colombia	Santa María	Proportion	2000	30.2	37.1	23.9
Colombia	Santa María	Proportion	2017	41.7	54.7	30.2
Colombia	Santa María	Count	2000	0	0	0
Colombia	Santa María	Count	2017	0	1	0
Colombia	Sopó	Proportion	2000	32.3	38.6	26.2
Colombia	Sopó	Proportion	2017	48.8	62.0	36.2
Colombia	Sopó	Count	2000	0	0	0

Table 4: ORS by Unit *(continued)*

Country	Unit	Metric	Year	Mean	Upper	Lower
Colombia	Sopó	Count	2017	0	1	0
Colombia	Villanueva	Proportion	2000	35.6	42.9	28.4
Colombia	Villanueva	Proportion	2017	55.7	67.9	43.8
Colombia	Villanueva	Count	2000	0	0	0
Colombia	Villanueva	Count	2017	1	1	0
Colombia	Salgar	Proportion	2000	35.3	42.1	26.9
Colombia	Salgar	Proportion	2017	47.1	60.7	35.5
Colombia	Salgar	Count	2000	0	0	0
Colombia	Salgar	Count	2017	0	1	0
Colombia	Santa Rosa de Viterbo	Proportion	2000	28.8	36.7	22.2
Colombia	Santa Rosa de Viterbo	Proportion	2017	51.1	64.2	39.5
Colombia	Santa Rosa de Viterbo	Count	2000	0	0	0
Colombia	Santa Rosa de Viterbo	Count	2017	1	1	0
Colombia	Subachoque	Proportion	2000	29.6	35.2	24.1
Colombia	Subachoque	Proportion	2017	47.0	61.1	35.6
Colombia	Subachoque	Count	2000	0	0	0
Colombia	Subachoque	Count	2017	0	1	0
Colombia	Zapatoca	Proportion	2000	34.6	41.9	27.9
Colombia	Zapatoca	Proportion	2017	49.8	62.8	37.5
Colombia	Zapatoca	Count	2000	0	0	0
Colombia	Zapatoca	Count	2017	0	1	0
Colombia	San Andrés de Cuerquia	Proportion	2000	36.1	43.1	28.0
Colombia	San Andrés de Cuerquia	Proportion	2017	49.9	62.4	38.3
Colombia	San Andrés de Cuerquia	Count	2000	0	0	0
Colombia	San Andrés de Cuerquia	Count	2017	0	1	0
Colombia	Santa Sofía	Proportion	2000	27.5	34.3	21.5
Colombia	Santa Sofía	Proportion	2017	47.4	61.7	36.4
Colombia	Santa Sofía	Count	2000	0	0	0
Colombia	Santa Sofía	Count	2017	0	1	0
Colombia	Suesca	Proportion	2000	29.9	35.6	23.9
Colombia	Suesca	Proportion	2017	47.8	63.3	35.7
Colombia	Suesca	Count	2000	0	0	0
Colombia	Suesca	Count	2017	0	1	0
Colombia	San Carlos	Proportion	2000	37.1	44.8	29.0
Colombia	San Carlos	Proportion	2017	50.9	62.4	40.3
Colombia	San Carlos	Count	2000	0	0	0
Colombia	San Carlos	Count	2017	1	1	0
Colombia	Santana	Proportion	2000	35.2	43.7	27.0
Colombia	Santana	Proportion	2017	50.7	63.7	39.5
Colombia	Santana	Count	2000	0	0	0
Colombia	Santana	Count	2017	1	1	0
Colombia	Supatá	Proportion	2000	31.8	37.0	25.4
Colombia	Supatá	Proportion	2017	47.7	61.0	35.9
Colombia	Supatá	Count	2000	0	0	0
Colombia	Supatá	Count	2017	0	1	0
Colombia	San Francisco	Proportion	2000	36.2	43.7	28.2
Colombia	San Francisco	Proportion	2017	48.3	60.3	37.3
Colombia	San Francisco	Count	2000	0	0	0
Colombia	San Francisco	Count	2017	0	1	0

Table 4: ORS by Unit *(continued)*

Country	Unit	Metric	Year	Mean	Upper	Lower
Colombia	Sativanorte	Proportion	2000	29.7	37.9	23.5
Colombia	Sativanorte	Proportion	2017	47.2	60.6	35.2
Colombia	Sativanorte	Count	2000	0	0	0
Colombia	Sativanorte	Count	2017	0	1	0
Colombia	Susa	Proportion	2000	29.4	35.1	23.3
Colombia	Susa	Proportion	2017	46.1	60.8	34.2
Colombia	Susa	Count	2000	0	0	0
Colombia	Susa	Count	2017	0	1	0
Colombia	San Jerónimo	Proportion	2000	38.5	45.6	30.2
Colombia	San Jerónimo	Proportion	2017	56.3	69.5	43.4
Colombia	San Jerónimo	Count	2000	0	0	0
Colombia	San Jerónimo	Count	2017	1	1	0
Colombia	Sativasur	Proportion	2000	29.3	37.7	23.1
Colombia	Sativasur	Proportion	2017	47.4	61.1	35.6
Colombia	Sativasur	Count	2000	0	0	0
Colombia	Sativasur	Count	2017	0	1	0
Colombia	Sutatausa	Proportion	2000	28.6	34.4	22.6
Colombia	Sutatausa	Proportion	2017	45.6	61.8	32.9
Colombia	Sutatausa	Count	2000	0	0	0
Colombia	Sutatausa	Count	2017	0	1	0
Colombia	San José de la Montaña	Proportion	2000	32.9	39.9	24.9
Colombia	San José de la Montaña	Proportion	2017	43.7	56.4	32.3
Colombia	San José de la Montaña	Count	2000	0	0	0
Colombia	San José de la Montaña	Count	2017	0	1	0
Colombia	Siachoque	Proportion	2000	28.3	35.7	22.0
Colombia	Siachoque	Proportion	2017	44.5	59.6	32.5
Colombia	Siachoque	Count	2000	0	0	0
Colombia	Siachoque	Count	2017	0	1	0
Colombia	Tabio	Proportion	2000	30.4	36.2	24.7
Colombia	Tabio	Proportion	2017	48.9	62.8	36.2
Colombia	Tabio	Count	2000	0	0	0
Colombia	Tabio	Count	2017	0	1	0
Colombia	San Juan de Urabá	Proportion	2000	42.0	51.3	33.2
Colombia	San Juan de Urabá	Proportion	2017	58.1	68.9	46.8
Colombia	San Juan de Urabá	Count	2000	0	1	0
Colombia	San Juan de Urabá	Count	2017	1	1	0
Colombia	Soatá	Proportion	2000	30.0	37.4	23.7
Colombia	Soatá	Proportion	2017	46.8	60.3	34.9
Colombia	Soatá	Count	2000	0	0	0
Colombia	Soatá	Count	2017	0	1	0
Colombia	Tausa	Proportion	2000	28.8	34.2	23.1
Colombia	Tausa	Proportion	2017	44.7	60.7	32.4
Colombia	Tausa	Count	2000	0	0	0
Colombia	Tausa	Count	2017	0	1	0
Colombia	San Luis	Proportion	2000	36.3	44.0	28.2
Colombia	San Luis	Proportion	2017	49.3	60.1	38.2
Colombia	San Luis	Count	2000	0	0	0
Colombia	San Luis	Count	2017	0	1	0
Colombia	Socha	Proportion	2000	31.3	39.6	24.6

Table 4: ORS by Unit *(continued)*

Country	Unit	Metric	Year	Mean	Upper	Lower
Colombia	Socha	Proportion	2017	53.3	66.8	41.0
Colombia	Socha	Count	2000	0	0	0
Colombia	Socha	Count	2017	1	1	0
Colombia	Tena	Proportion	2000	35.0	41.1	29.7
Colombia	Tena	Proportion	2017	51.3	63.4	39.0
Colombia	Tena	Count	2000	0	0	0
Colombia	Tena	Count	2017	1	1	0
Colombia	San Pedro de los Milagros	Proportion	2000	36.2	43.0	28.7
Colombia	San Pedro de los Milagros	Proportion	2017	52.2	65.1	39.7
Colombia	San Pedro de los Milagros	Count	2000	0	0	0
Colombia	San Pedro de los Milagros	Count	2017	1	1	0
Colombia	Socotá	Proportion	2000	30.1	37.7	23.4
Colombia	Socotá	Proportion	2017	48.3	61.8	36.1
Colombia	Socotá	Count	2000	0	0	0
Colombia	Socotá	Count	2017	0	1	0
Colombia	Tenjo	Proportion	2000	30.7	36.7	25.2
Colombia	Tenjo	Proportion	2017	49.3	62.1	37.4
Colombia	Tenjo	Count	2000	0	0	0
Colombia	Tenjo	Count	2017	0	1	0
Colombia	San Pedro de Urabá	Proportion	2000	39.2	47.4	31.8
Colombia	San Pedro de Urabá	Proportion	2017	55.1	66.5	43.9
Colombia	San Pedro de Urabá	Count	2000	0	0	0
Colombia	San Pedro de Urabá	Count	2017	1	1	0
Colombia	Sogamoso	Proportion	2000	32.3	40.5	25.2
Colombia	Sogamoso	Proportion	2017	51.0	64.8	38.9
Colombia	Sogamoso	Count	2000	0	0	0
Colombia	Sogamoso	Count	2017	1	1	0
Colombia	Tibacuy	Proportion	2000	35.4	41.7	29.4
Colombia	Tibacuy	Proportion	2017	52.1	63.8	40.3
Colombia	Tibacuy	Count	2000	0	0	0
Colombia	Tibacuy	Count	2017	1	1	0
Colombia	San Rafael	Proportion	2000	35.0	42.3	27.2
Colombia	San Rafael	Proportion	2017	48.8	60.6	37.9
Colombia	San Rafael	Count	2000	0	0	0
Colombia	San Rafael	Count	2017	0	1	0
Colombia	Somondoco	Proportion	2000	28.6	35.4	22.5
Colombia	Somondoco	Proportion	2017	45.8	59.9	33.5
Colombia	Somondoco	Count	2000	0	0	0
Colombia	Somondoco	Count	2017	0	1	0
Colombia	Tibirita	Proportion	2000	30.7	37.1	24.1
Colombia	Tibirita	Proportion	2017	48.2	63.2	36.3
Colombia	Tibirita	Count	2000	0	0	0
Colombia	Tibirita	Count	2017	0	1	0
Colombia	San Roque	Proportion	2000	36.0	43.5	28.2
Colombia	San Roque	Proportion	2017	50.0	61.1	38.7
Colombia	San Roque	Count	2000	0	0	0
Colombia	San Roque	Count	2017	0	1	0
Colombia	Sora	Proportion	2000	26.6	33.8	20.7
Colombia	Sora	Proportion	2017	45.0	59.9	33.6

Table 4: ORS by Unit *(continued)*

Country	Unit	Metric	Year	Mean	Upper	Lower
Colombia	Sora	Count	2000	0	0	0
Colombia	Sora	Count	2017	0	1	0
Colombia	Tocaima	Proportion	2000	38.1	43.8	31.8
Colombia	Tocaima	Proportion	2017	53.3	65.6	41.9
Colombia	Tocaima	Count	2000	0	0	0
Colombia	Tocaima	Count	2017	1	1	0
Colombia	San Vicente	Proportion	2000	34.6	41.0	27.3
Colombia	San Vicente	Proportion	2017	49.9	62.6	39.1
Colombia	San Vicente	Count	2000	0	0	0
Colombia	San Vicente	Count	2017	0	1	0
Colombia	Soracá	Proportion	2000	28.1	35.7	21.9
Colombia	Soracá	Proportion	2017	45.3	60.5	33.5
Colombia	Soracá	Count	2000	0	0	0
Colombia	Soracá	Count	2017	0	1	0
Colombia	Tocancipá	Proportion	2000	30.6	36.9	24.7
Colombia	Tocancipá	Proportion	2017	49.2	63.5	36.4
Colombia	Tocancipá	Count	2000	0	0	0
Colombia	Tocancipá	Count	2017	0	1	0
Colombia	Santa Bárbara	Proportion	2000	36.7	43.4	28.8
Colombia	Santa Bárbara	Proportion	2017	52.7	66.2	41.6
Colombia	Santa Bárbara	Count	2000	0	0	0
Colombia	Santa Bárbara	Count	2017	1	1	0
Colombia	Sotaquirá	Proportion	2000	27.5	35.0	21.4
Colombia	Sotaquirá	Proportion	2017	47.2	62.5	35.0
Colombia	Sotaquirá	Count	2000	0	0	0
Colombia	Sotaquirá	Count	2017	0	1	0
Colombia	Topaipí	Proportion	2000	36.5	42.4	29.7
Colombia	Topaipí	Proportion	2017	50.4	63.4	39.5
Colombia	Topaipí	Count	2000	0	0	0
Colombia	Topaipí	Count	2017	1	1	0
Colombia	Santa Fe de Antioquia	Proportion	2000	38.5	45.8	30.4
Colombia	Santa Fe de Antioquia	Proportion	2017	52.6	65.8	38.8
Colombia	Santa Fe de Antioquia	Count	2000	0	0	0
Colombia	Santa Fe de Antioquia	Count	2017	1	1	0
Colombia	Susacón	Proportion	2000	28.5	36.0	22.5
Colombia	Susacón	Proportion	2017	45.3	58.3	33.1
Colombia	Susacón	Count	2000	0	0	0
Colombia	Susacón	Count	2017	0	1	0
Colombia	Ubalá	Proportion	2000	31.5	38.0	25.2
Colombia	Ubalá	Proportion	2017	47.5	60.1	35.8
Colombia	Ubalá	Count	2000	0	0	0
Colombia	Ubalá	Count	2017	0	1	0
Colombia	Santa Rosa de Osos	Proportion	2000	34.6	41.5	27.1
Colombia	Santa Rosa de Osos	Proportion	2017	47.3	60.2	35.3
Colombia	Santa Rosa de Osos	Count	2000	0	0	0
Colombia	Santa Rosa de Osos	Count	2017	0	1	0
Colombia	Sutamarchán	Proportion	2000	28.6	35.5	22.5
Colombia	Sutamarchán	Proportion	2017	48.9	63.1	37.5
Colombia	Sutamarchán	Count	2000	0	0	0

Table 4: ORS by Unit *(continued)*

Country	Unit	Metric	Year	Mean	Upper	Lower
Colombia	Sutamarchán	Count	2017	0	1	0
Colombia	Ubaque	Proportion	2000	30.7	36.9	25.5
Colombia	Ubaque	Proportion	2017	47.9	60.6	35.9
Colombia	Ubaque	Count	2000	0	0	0
Colombia	Ubaque	Count	2017	0	1	0
Colombia	Santo Domingo	Proportion	2000	35.2	42.2	28.2
Colombia	Santo Domingo	Proportion	2017	50.5	61.0	39.7
Colombia	Santo Domingo	Count	2000	0	0	0
Colombia	Santo Domingo	Count	2017	1	1	0
Colombia	Sutatenza	Proportion	2000	30.0	36.6	23.6
Colombia	Sutatenza	Proportion	2017	47.8	62.1	35.5
Colombia	Sutatenza	Count	2000	0	0	0
Colombia	Sutatenza	Count	2017	0	1	0
Colombia	Une	Proportion	2000	31.6	38.1	25.9
Colombia	Une	Proportion	2017	47.5	60.5	35.7
Colombia	Une	Count	2000	0	0	0
Colombia	Une	Count	2017	0	1	0
Colombia	Segovia	Proportion	2000	35.8	42.5	28.4
Colombia	Segovia	Proportion	2017	48.0	59.2	36.2
Colombia	Segovia	Count	2000	0	0	0
Colombia	Segovia	Count	2017	0	1	0
Colombia	Tasco	Proportion	2000	30.7	38.8	23.9
Colombia	Tasco	Proportion	2017	50.2	63.1	37.8
Colombia	Tasco	Count	2000	0	0	0
Colombia	Tasco	Count	2017	1	1	0
Colombia	Utica	Proportion	2000	37.4	43.4	30.2
Colombia	Utica	Proportion	2017	53.8	65.7	41.7
Colombia	Utica	Count	2000	0	0	0
Colombia	Utica	Count	2017	1	1	0
Colombia	Sonsón	Proportion	2000	35.9	43.1	28.8
Colombia	Sonsón	Proportion	2017	50.6	61.5	40.1
Colombia	Sonsón	Count	2000	0	0	0
Colombia	Sonsón	Count	2017	1	1	0
Colombia	Tenza	Proportion	2000	30.4	37.0	24.2
Colombia	Tenza	Proportion	2017	48.9	63.1	36.8
Colombia	Tenza	Count	2000	0	0	0
Colombia	Tenza	Count	2017	0	1	0
Colombia	Venecia	Proportion	2000	31.9	38.4	25.5
Colombia	Venecia	Proportion	2017	48.2	60.5	36.7
Colombia	Venecia	Count	2000	0	0	0
Colombia	Venecia	Count	2017	0	1	0
Colombia	Sopetrán	Proportion	2000	39.1	46.4	31.0
Colombia	Sopetrán	Proportion	2017	56.9	69.7	43.6
Colombia	Sopetrán	Count	2000	0	0	0
Colombia	Sopetrán	Count	2017	1	1	0
Colombia	Tibaná	Proportion	2000	28.1	35.1	21.8
Colombia	Tibaná	Proportion	2017	50.3	64.3	38.5
Colombia	Tibaná	Count	2000	0	0	0
Colombia	Tibaná	Count	2017	1	1	0

Table 4: ORS by Unit *(continued)*

Country	Unit	Metric	Year	Mean	Upper	Lower
Colombia	Vergara	Proportion	2000	34.8	40.4	28.1
Colombia	Vergara	Proportion	2017	49.1	61.6	38.0
Colombia	Vergara	Count	2000	0	0	0
Colombia	Vergara	Count	2017	0	1	0
Colombia	Támesis	Proportion	2000	32.7	39.4	25.7
Colombia	Támesis	Proportion	2017	47.4	61.3	36.2
Colombia	Támesis	Count	2000	0	0	0
Colombia	Támesis	Count	2017	0	1	0
Colombia	Tibasosa	Proportion	2000	28.9	36.7	22.2
Colombia	Tibasosa	Proportion	2017	51.8	65.0	39.6
Colombia	Tibasosa	Count	2000	0	0	0
Colombia	Tibasosa	Count	2017	1	1	0
Colombia	Vianí	Proportion	2000	32.8	38.7	26.6
Colombia	Vianí	Proportion	2017	49.5	62.7	37.6
Colombia	Vianí	Count	2000	0	0	0
Colombia	Vianí	Count	2017	0	1	0
Colombia	Tarazá	Proportion	2000	37.4	45.4	29.7
Colombia	Tarazá	Proportion	2017	50.0	61.4	39.2
Colombia	Tarazá	Count	2000	0	0	0
Colombia	Tarazá	Count	2017	0	1	0
Colombia	Tinjacá	Proportion	2000	28.3	34.8	22.2
Colombia	Tinjacá	Proportion	2017	49.9	63.8	38.9
Colombia	Tinjacá	Count	2000	0	0	0
Colombia	Tinjacá	Count	2017	0	1	0
Colombia	Villa de San Diego de Ubaté	Proportion	2000	28.1	33.9	22.2
Colombia	Villa de San Diego de Ubaté	Proportion	2017	44.3	60.1	32.4
Colombia	Villa de San Diego de Ubaté	Count	2000	0	0	0
Colombia	Villa de San Diego de Ubaté	Count	2017	0	1	0
Colombia	Tarso	Proportion	2000	36.7	44.4	29.1
Colombia	Tarso	Proportion	2017	53.5	65.9	41.3
Colombia	Tarso	Count	2000	0	0	0
Colombia	Tarso	Count	2017	1	1	0
Colombia	Tipacoque	Proportion	2000	28.5	35.8	22.5
Colombia	Tipacoque	Proportion	2017	46.1	59.4	34.3
Colombia	Tipacoque	Count	2000	0	0	0
Colombia	Tipacoque	Count	2017	0	1	0
Colombia	Villagómez	Proportion	2000	33.3	38.3	27.0
Colombia	Villagómez	Proportion	2017	48.4	62.4	37.2
Colombia	Villagómez	Count	2000	0	0	0
Colombia	Villagómez	Count	2017	0	1	0
Colombia	Titiribí	Proportion	2000	38.0	44.8	30.4
Colombia	Titiribí	Proportion	2017	55.6	67.4	44.3
Colombia	Titiribí	Count	2000	0	0	0
Colombia	Titiribí	Count	2017	1	1	0
Colombia	Toca	Proportion	2000	27.5	35.1	21.2

Table 4: ORS by Unit *(continued)*

Country	Unit	Metric	Year	Mean	Upper	Lower
Colombia	Toca	Proportion	2017	44.0	59.3	32.0
Colombia	Toca	Count	2000	0	0	0
Colombia	Toca	Count	2017	0	1	0
Colombia	Villapinzón	Proportion	2000	29.8	36.2	23.7
Colombia	Villapinzón	Proportion	2017	46.4	60.7	35.1
Colombia	Villapinzón	Count	2000	0	0	0
Colombia	Villapinzón	Count	2017	0	1	0
Colombia	Toledo	Proportion	2000	37.6	44.7	29.8
Colombia	Toledo	Proportion	2017	54.1	66.3	42.4
Colombia	Toledo	Count	2000	0	0	0
Colombia	Toledo	Count	2017	1	1	0
Colombia	Toguí	Proportion	2000	32.5	40.2	25.2
Colombia	Toguí	Proportion	2017	50.8	65.0	39.3
Colombia	Toguí	Count	2000	0	0	0
Colombia	Toguí	Count	2017	1	1	0
Colombia	Villeta	Proportion	2000	35.8	41.9	28.8
Colombia	Villeta	Proportion	2017	53.0	64.9	41.1
Colombia	Villeta	Count	2000	0	0	0
Colombia	Villeta	Count	2017	1	1	0
Colombia	Turbo	Proportion	2000	38.3	47.2	29.6
Colombia	Turbo	Proportion	2017	52.8	64.8	42.2
Colombia	Turbo	Count	2000	0	0	0
Colombia	Turbo	Count	2017	1	1	0
Colombia	Topagá	Proportion	2000	31.5	39.8	24.7
Colombia	Topagá	Proportion	2017	52.1	65.2	39.7
Colombia	Topagá	Count	2000	0	0	0
Colombia	Topagá	Count	2017	1	1	0
Colombia	Viotá	Proportion	2000	37.4	43.6	31.6
Colombia	Viotá	Proportion	2017	54.4	65.8	42.7
Colombia	Viotá	Count	2000	0	0	0
Colombia	Viotá	Count	2017	1	1	0
Colombia	Uramita	Proportion	2000	39.0	46.6	30.5
Colombia	Uramita	Proportion	2017	53.2	64.5	41.5
Colombia	Uramita	Count	2000	0	0	0
Colombia	Uramita	Count	2017	1	1	0
Colombia	Tota	Proportion	2000	29.3	36.9	23.2
Colombia	Tota	Proportion	2017	49.8	64.2	37.3
Colombia	Tota	Count	2000	0	0	0
Colombia	Tota	Count	2017	0	1	0
Colombia	Yacopí	Proportion	2000	36.8	42.8	29.5
Colombia	Yacopí	Proportion	2017	48.8	62.4	37.6
Colombia	Yacopí	Count	2000	0	0	0
Colombia	Yacopí	Count	2017	0	1	0
Colombia	Urrao	Proportion	2000	35.7	43.1	27.9
Colombia	Urrao	Proportion	2017	45.5	57.9	35.2
Colombia	Urrao	Count	2000	0	0	0
Colombia	Urrao	Count	2017	0	1	0
Colombia	Tunja	Proportion	2000	30.5	38.6	23.8
Colombia	Tunja	Proportion	2017	45.0	60.0	33.5

Table 4: ORS by Unit *(continued)*

Country	Unit	Metric	Year	Mean	Upper	Lower
Colombia	Tunja	Count	2000	0	0	0
Colombia	Tunja	Count	2017	0	1	0
Colombia	Zipacón	Proportion	2000	33.2	39.0	27.7
Colombia	Zipacón	Proportion	2017	48.7	61.2	37.0
Colombia	Zipacón	Count	2000	0	0	0
Colombia	Zipacón	Count	2017	0	1	0
Colombia	Valdivia	Proportion	2000	36.3	44.1	28.4
Colombia	Valdivia	Proportion	2017	49.9	61.2	38.6
Colombia	Valdivia	Count	2000	0	0	0
Colombia	Valdivia	Count	2017	0	1	0
Colombia	Tunungua	Proportion	2000	32.4	38.7	25.1
Colombia	Tunungua	Proportion	2017	47.6	61.1	36.2
Colombia	Tunungua	Count	2000	0	0	0
Colombia	Tunungua	Count	2017	0	1	0
Colombia	Zipaquirá	Proportion	2000	29.4	35.0	23.6
Colombia	Zipaquirá	Proportion	2017	45.5	60.5	33.3
Colombia	Zipaquirá	Count	2000	0	0	0
Colombia	Zipaquirá	Count	2017	0	1	0
Colombia	Valparaíso	Proportion	2000	35.8	42.3	28.5
Colombia	Valparaíso	Proportion	2017	53.5	66.4	42.6
Colombia	Valparaíso	Count	2000	0	0	0
Colombia	Valparaíso	Count	2017	1	1	0
Colombia	Turmequé	Proportion	2000	27.8	34.3	21.8
Colombia	Turmequé	Proportion	2017	46.3	60.3	34.6
Colombia	Turmequé	Count	2000	0	0	0
Colombia	Turmequé	Count	2017	0	1	0
Colombia	Vegachí	Proportion	2000	36.3	43.3	29.3
Colombia	Vegachí	Proportion	2017	49.5	60.3	37.7
Colombia	Vegachí	Count	2000	0	0	0
Colombia	Vegachí	Count	2017	0	1	0
Colombia	Tuta	Proportion	2000	28.2	36.0	22.3
Colombia	Tuta	Proportion	2017	47.3	62.9	35.1
Colombia	Tuta	Count	2000	0	0	0
Colombia	Tuta	Count	2017	0	1	0
Colombia	Venecia	Proportion	2000	37.3	44.4	29.8
Colombia	Venecia	Proportion	2017	54.0	66.6	42.4
Colombia	Venecia	Count	2000	0	0	0
Colombia	Venecia	Count	2017	1	1	0
Colombia	Tutazá	Proportion	2000	30.8	38.2	24.4
Colombia	Tutazá	Proportion	2017	52.2	64.9	40.2
Colombia	Tutazá	Count	2000	0	0	0
Colombia	Tutazá	Count	2017	1	1	0
Colombia	Vigía del Fuerte	Proportion	2000	31.4	39.9	23.9
Colombia	Vigía del Fuerte	Proportion	2017	42.7	54.4	32.4
Colombia	Vigía del Fuerte	Count	2000	0	0	0
Colombia	Vigía del Fuerte	Count	2017	0	1	0
Colombia	Umbita	Proportion	2000	27.5	34.0	21.5
Colombia	Umbita	Proportion	2017	46.4	60.5	34.8
Colombia	Umbita	Count	2000	0	0	0

Table 4: ORS by Unit *(continued)*

Country	Unit	Metric	Year	Mean	Upper	Lower
Colombia	Umbita	Count	2017	0	1	0
Colombia	Yalí	Proportion	2000	36.4	43.3	29.1
Colombia	Yalí	Proportion	2017	49.6	60.8	38.2
Colombia	Yalí	Count	2000	0	0	0
Colombia	Yalí	Count	2017	0	1	0
Colombia	Ventaquemada	Proportion	2000	28.1	34.7	22.0
Colombia	Ventaquemada	Proportion	2017	46.4	60.6	34.4
Colombia	Ventaquemada	Count	2000	0	0	0
Colombia	Ventaquemada	Count	2017	0	1	0
Colombia	Yarumal	Proportion	2000	33.9	40.9	25.7
Colombia	Yarumal	Proportion	2017	46.3	58.0	35.3
Colombia	Yarumal	Count	2000	0	0	0
Colombia	Yarumal	Count	2017	0	1	0
Colombia	Villa de Leyva	Proportion	2000	30.4	37.9	24.3
Colombia	Villa de Leyva	Proportion	2017	51.8	65.6	40.1
Colombia	Villa de Leyva	Count	2000	0	0	0
Colombia	Villa de Leyva	Count	2017	1	1	0
Colombia	Yolombó	Proportion	2000	35.7	42.2	28.2
Colombia	Yolombó	Proportion	2017	49.1	59.9	37.9
Colombia	Yolombó	Count	2000	0	0	0
Colombia	Yolombó	Count	2017	0	1	0
Colombia	Viracachá	Proportion	2000	27.7	35.2	21.4
Colombia	Viracachá	Proportion	2017	47.7	62.5	35.7
Colombia	Viracachá	Count	2000	0	0	0
Colombia	Viracachá	Count	2017	0	1	0
Colombia	Yondó	Proportion	2000	38.2	46.0	30.6
Colombia	Yondó	Proportion	2017	55.6	68.0	44.6
Colombia	Yondó	Count	2000	0	0	0
Colombia	Yondó	Count	2017	1	1	0
Colombia	Zetaquirá	Proportion	2000	29.7	37.3	23.2
Colombia	Zetaquirá	Proportion	2017	47.5	60.7	35.5
Colombia	Zetaquirá	Count	2000	0	0	0
Colombia	Zetaquirá	Count	2017	0	1	0
Colombia	Zaragoza	Proportion	2000	38.0	44.8	29.6
Colombia	Zaragoza	Proportion	2017	52.0	63.0	40.5
Colombia	Zaragoza	Count	2000	0	0	0
Colombia	Zaragoza	Count	2017	1	1	0
Comoros	Mwali	Proportion	2000	34.0	57.9	14.7
Comoros	Mwali	Proportion	2017	42.0	61.1	23.6
Comoros	Mwali	Count	2000	0	1	0
Comoros	Mwali	Count	2017	0	1	0
Comoros	Njazídja	Proportion	2000	32.7	52.3	14.0
Comoros	Njazídja	Proportion	2017	40.1	58.1	22.9
Comoros	Njazídja	Count	2000	0	1	0
Comoros	Njazídja	Count	2017	0	1	0
Comoros	Nzwani	Proportion	2000	36.6	58.8	17.0
Comoros	Nzwani	Proportion	2017	43.2	63.6	23.4
Comoros	Nzwani	Count	2000	0	1	0
Comoros	Nzwani	Count	2017	0	1	0

Table 4: ORS by Unit *(continued)*

Country	Unit	Metric	Year	Mean	Upper	Lower
Costa Rica	Alajuela	Proportion	2000	38.3	61.6	16.5
Costa Rica	Alajuela	Proportion	2017	40.3	62.1	19.2
Costa Rica	Alajuela	Count	2000	0	1	0
Costa Rica	Alajuela	Count	2017	0	1	0
Costa Rica	Alvarado	Proportion	2000	37.0	58.8	17.5
Costa Rica	Alvarado	Proportion	2017	38.6	62.4	17.6
Costa Rica	Alvarado	Count	2000	0	1	0
Costa Rica	Alvarado	Count	2017	0	1	0
Costa Rica	Abangares	Proportion	2000	37.6	58.6	17.0
Costa Rica	Abangares	Proportion	2017	41.2	64.4	19.7
Costa Rica	Abangares	Count	2000	0	1	0
Costa Rica	Abangares	Count	2017	0	1	0
Costa Rica	Barva	Proportion	2000	38.2	62.3	17.4
Costa Rica	Barva	Proportion	2017	39.3	61.3	18.0
Costa Rica	Barva	Count	2000	0	1	0
Costa Rica	Barva	Count	2017	0	1	0
Costa Rica	Guácimo	Proportion	2000	37.0	59.2	17.5
Costa Rica	Guácimo	Proportion	2017	38.0	62.3	17.5
Costa Rica	Guácimo	Count	2000	0	1	0
Costa Rica	Guácimo	Count	2017	0	1	0
Costa Rica	Aguirre	Proportion	2000	38.0	59.3	14.7
Costa Rica	Aguirre	Proportion	2017	39.1	61.9	17.8
Costa Rica	Aguirre	Count	2000	0	1	0
Costa Rica	Aguirre	Count	2017	0	1	0
Costa Rica	Acosta	Proportion	2000	38.4	61.2	16.3
Costa Rica	Acosta	Proportion	2017	40.5	62.2	19.7
Costa Rica	Acosta	Count	2000	0	1	0
Costa Rica	Acosta	Count	2017	0	1	0
Costa Rica	Alfaro Ruiz	Proportion	2000	37.2	59.5	15.9
Costa Rica	Alfaro Ruiz	Proportion	2017	39.7	64.6	19.4
Costa Rica	Alfaro Ruiz	Count	2000	0	1	0
Costa Rica	Alfaro Ruiz	Count	2017	0	1	0
Costa Rica	Cartago	Proportion	2000	38.6	60.6	17.6
Costa Rica	Cartago	Proportion	2017	39.9	63.6	18.5
Costa Rica	Cartago	Count	2000	0	1	0
Costa Rica	Cartago	Count	2017	0	1	0
Costa Rica	Bagaces	Proportion	2000	37.8	57.2	18.1
Costa Rica	Bagaces	Proportion	2017	40.8	63.5	19.8
Costa Rica	Bagaces	Count	2000	0	1	0
Costa Rica	Bagaces	Count	2017	0	1	0
Costa Rica	Belén	Proportion	2000	38.6	62.6	16.8
Costa Rica	Belén	Proportion	2017	40.6	64.4	18.1
Costa Rica	Belén	Count	2000	0	1	0
Costa Rica	Belén	Count	2017	0	1	0
Costa Rica	Limón	Proportion	2000	36.3	59.3	16.2
Costa Rica	Limón	Proportion	2017	37.7	60.7	17.0
Costa Rica	Limón	Count	2000	0	1	0
Costa Rica	Limón	Count	2017	0	1	0
Costa Rica	Buenos Aires	Proportion	2000	38.6	62.6	17.5

Table 4: ORS by Unit *(continued)*

Country	Unit	Metric	Year	Mean	Upper	Lower
Costa Rica	Buenos Aires	Proportion	2017	40.9	63.1	19.5
Costa Rica	Buenos Aires	Count	2000	0	1	0
Costa Rica	Buenos Aires	Count	2017	0	1	0
Costa Rica	Alajuelita	Proportion	2000	38.6	60.3	17.6
Costa Rica	Alajuelita	Proportion	2017	41.1	63.8	18.7
Costa Rica	Alajuelita	Count	2000	0	1	0
Costa Rica	Alajuelita	Count	2017	0	1	0
Costa Rica	Atenas	Proportion	2000	37.9	60.6	15.8
Costa Rica	Atenas	Proportion	2017	40.9	63.1	18.8
Costa Rica	Atenas	Count	2000	0	1	0
Costa Rica	Atenas	Count	2017	0	1	0
Costa Rica	El Guarco	Proportion	2000	38.8	62.3	17.6
Costa Rica	El Guarco	Proportion	2017	40.1	66.3	17.9
Costa Rica	El Guarco	Count	2000	0	1	0
Costa Rica	El Guarco	Count	2017	0	1	0
Costa Rica	Cañas	Proportion	2000	37.9	60.6	16.0
Costa Rica	Cañas	Proportion	2017	41.4	64.7	19.6
Costa Rica	Cañas	Count	2000	0	1	0
Costa Rica	Cañas	Count	2017	0	1	0
Costa Rica	Flores	Proportion	2000	38.3	66.1	14.8
Costa Rica	Flores	Proportion	2017	39.7	67.1	16.0
Costa Rica	Flores	Count	2000	0	1	0
Costa Rica	Flores	Count	2017	0	1	0
Costa Rica	Matina	Proportion	2000	37.0	59.6	17.1
Costa Rica	Matina	Proportion	2017	38.1	61.7	17.3
Costa Rica	Matina	Count	2000	0	1	0
Costa Rica	Matina	Count	2017	0	1	0
Costa Rica	Corredores	Proportion	2000	37.5	63.9	17.1
Costa Rica	Corredores	Proportion	2017	42.2	65.2	19.0
Costa Rica	Corredores	Count	2000	0	1	0
Costa Rica	Corredores	Count	2017	0	1	0
Costa Rica	Aserrí	Proportion	2000	38.4	60.8	16.9
Costa Rica	Aserrí	Proportion	2017	41.0	65.3	18.7
Costa Rica	Aserrí	Count	2000	0	1	0
Costa Rica	Aserrí	Count	2017	0	1	0
Costa Rica	Grecia	Proportion	2000	37.5	60.3	17.3
Costa Rica	Grecia	Proportion	2017	39.7	61.9	18.3
Costa Rica	Grecia	Count	2000	0	1	0
Costa Rica	Grecia	Count	2017	0	1	0
Costa Rica	Jiménez	Proportion	2000	37.4	58.3	17.8
Costa Rica	Jiménez	Proportion	2017	38.8	62.5	18.7
Costa Rica	Jiménez	Count	2000	0	1	0
Costa Rica	Jiménez	Count	2017	0	1	0
Costa Rica	Carrillo	Proportion	2000	37.9	60.4	18.5
Costa Rica	Carrillo	Proportion	2017	42.0	66.1	19.9
Costa Rica	Carrillo	Count	2000	0	1	0
Costa Rica	Carrillo	Count	2017	0	1	0
Costa Rica	Heredia	Proportion	2000	38.8	64.7	18.2
Costa Rica	Heredia	Proportion	2017	40.4	62.4	18.5

Table 4: ORS by Unit *(continued)*

Country	Unit	Metric	Year	Mean	Upper	Lower
Costa Rica	Heredia	Count	2000	0	1	0
Costa Rica	Heredia	Count	2017	0	1	0
Costa Rica	Pococí	Proportion	2000	37.1	57.9	17.3
Costa Rica	Pococí	Proportion	2017	38.5	63.0	18.3
Costa Rica	Pococí	Count	2000	0	1	0
Costa Rica	Pococí	Count	2017	0	1	0
Costa Rica	Coto Brus	Proportion	2000	38.4	65.9	17.0
Costa Rica	Coto Brus	Proportion	2017	42.3	64.9	19.1
Costa Rica	Coto Brus	Count	2000	0	1	0
Costa Rica	Coto Brus	Count	2017	0	1	0
Costa Rica	Curridabat	Proportion	2000	38.3	63.3	17.8
Costa Rica	Curridabat	Proportion	2017	39.6	63.8	17.7
Costa Rica	Curridabat	Count	2000	0	1	0
Costa Rica	Curridabat	Count	2017	0	1	0
Costa Rica	Guatuso	Proportion	2000	37.6	57.1	17.0
Costa Rica	Guatuso	Proportion	2017	40.0	64.0	19.3
Costa Rica	Guatuso	Count	2000	0	1	0
Costa Rica	Guatuso	Count	2017	0	1	0
Costa Rica	La Unión	Proportion	2000	38.3	61.2	16.1
Costa Rica	La Unión	Proportion	2017	39.3	63.2	18.4
Costa Rica	La Unión	Count	2000	0	1	0
Costa Rica	La Unión	Count	2017	0	1	0
Costa Rica	Hojancha	Proportion	2000	38.4	59.4	17.2
Costa Rica	Hojancha	Proportion	2017	42.1	66.7	20.9
Costa Rica	Hojancha	Count	2000	0	1	0
Costa Rica	Hojancha	Count	2017	0	1	0
Costa Rica	San Isidro	Proportion	2000	37.5	62.4	15.7
Costa Rica	San Isidro	Proportion	2017	39.1	63.7	18.2
Costa Rica	San Isidro	Count	2000	0	1	0
Costa Rica	San Isidro	Count	2017	0	1	0
Costa Rica	Siquirres	Proportion	2000	37.2	59.0	16.6
Costa Rica	Siquirres	Proportion	2017	38.3	60.9	17.9
Costa Rica	Siquirres	Count	2000	0	1	0
Costa Rica	Siquirres	Count	2017	0	1	0
Costa Rica	Esparza	Proportion	2000	38.3	62.3	17.1
Costa Rica	Esparza	Proportion	2017	41.0	66.8	20.0
Costa Rica	Esparza	Count	2000	0	1	0
Costa Rica	Esparza	Count	2017	0	1	0
Costa Rica	Desamparados	Proportion	2000	38.7	62.9	16.9
Costa Rica	Desamparados	Proportion	2017	40.1	63.5	18.0
Costa Rica	Desamparados	Count	2000	0	1	0
Costa Rica	Desamparados	Count	2017	0	1	0
Costa Rica	Los Chiles	Proportion	2000	38.0	56.9	18.4
Costa Rica	Los Chiles	Proportion	2017	40.3	64.1	19.4
Costa Rica	Los Chiles	Count	2000	0	1	0
Costa Rica	Los Chiles	Count	2017	0	1	0
Costa Rica	Oreamuno	Proportion	2000	37.3	58.9	16.3
Costa Rica	Oreamuno	Proportion	2017	38.9	61.9	17.4
Costa Rica	Oreamuno	Count	2000	0	1	0

Table 4: ORS by Unit *(continued)*

Country	Unit	Metric	Year	Mean	Upper	Lower
Costa Rica	Oreamuno	Count	2017	0	1	0
Costa Rica	La Cruz	Proportion	2000	38.9	59.7	18.6
Costa Rica	La Cruz	Proportion	2017	42.7	65.6	22.6
Costa Rica	La Cruz	Count	2000	0	1	0
Costa Rica	La Cruz	Count	2017	0	1	0
Costa Rica	San Pablo	Proportion	2000	38.7	65.2	18.5
Costa Rica	San Pablo	Proportion	2017	39.8	60.7	18.2
Costa Rica	San Pablo	Count	2000	0	1	0
Costa Rica	San Pablo	Count	2017	0	1	0
Costa Rica	Talamanca	Proportion	2000	35.2	58.6	16.3
Costa Rica	Talamanca	Proportion	2017	37.6	58.8	16.1
Costa Rica	Talamanca	Count	2000	0	1	0
Costa Rica	Talamanca	Count	2017	0	1	0
Costa Rica	Garabito	Proportion	2000	38.4	60.7	16.7
Costa Rica	Garabito	Proportion	2017	40.8	63.9	19.4
Costa Rica	Garabito	Count	2000	0	1	0
Costa Rica	Garabito	Count	2017	0	1	0
Costa Rica	Dota	Proportion	2000	37.7	61.6	15.5
Costa Rica	Dota	Proportion	2017	38.9	62.6	18.5
Costa Rica	Dota	Count	2000	0	1	0
Costa Rica	Dota	Count	2017	0	1	0
Costa Rica	Naranjo	Proportion	2000	37.5	60.2	16.3
Costa Rica	Naranjo	Proportion	2017	40.0	63.7	18.2
Costa Rica	Naranjo	Count	2000	0	1	0
Costa Rica	Naranjo	Count	2017	0	1	0
Costa Rica	Paraíso	Proportion	2000	38.3	60.8	16.4
Costa Rica	Paraíso	Proportion	2017	39.3	63.5	18.7
Costa Rica	Paraíso	Count	2000	0	1	0
Costa Rica	Paraíso	Count	2017	0	1	0
Costa Rica	Liberia	Proportion	2000	37.7	59.3	19.3
Costa Rica	Liberia	Proportion	2017	40.9	62.9	20.3
Costa Rica	Liberia	Count	2000	0	1	0
Costa Rica	Liberia	Count	2017	0	1	0
Costa Rica	San Rafael	Proportion	2000	38.2	62.7	18.3
Costa Rica	San Rafael	Proportion	2017	39.2	63.0	18.4
Costa Rica	San Rafael	Count	2000	0	1	0
Costa Rica	San Rafael	Count	2017	0	1	0
Costa Rica	Golfito	Proportion	2000	38.1	63.7	17.5
Costa Rica	Golfito	Proportion	2017	41.9	63.9	18.8
Costa Rica	Golfito	Count	2000	0	1	0
Costa Rica	Golfito	Count	2017	0	1	0
Costa Rica	Escazú	Proportion	2000	39.3	64.6	15.5
Costa Rica	Escazú	Proportion	2017	41.2	64.4	17.5
Costa Rica	Escazú	Count	2000	0	1	0
Costa Rica	Escazú	Count	2017	0	1	0
Costa Rica	Orotina	Proportion	2000	37.2	59.7	14.9
Costa Rica	Orotina	Proportion	2017	40.2	64.9	19.0
Costa Rica	Orotina	Count	2000	0	1	0
Costa Rica	Orotina	Count	2017	0	1	0

Table 4: ORS by Unit *(continued)*

Country	Unit	Metric	Year	Mean	Upper	Lower
Costa Rica	Turrialba	Proportion	2000	37.3	60.5	18.3
Costa Rica	Turrialba	Proportion	2017	39.0	62.0	19.0
Costa Rica	Turrialba	Count	2000	0	1	0
Costa Rica	Turrialba	Count	2017	0	1	0
Costa Rica	Nandayure	Proportion	2000	39.0	61.0	17.9
Costa Rica	Nandayure	Proportion	2017	42.6	67.3	20.8
Costa Rica	Nandayure	Count	2000	0	1	0
Costa Rica	Nandayure	Count	2017	0	1	0
Costa Rica	Santa Bárbara	Proportion	2000	37.7	61.9	16.2
Costa Rica	Santa Bárbara	Proportion	2017	39.5	60.9	16.3
Costa Rica	Santa Bárbara	Count	2000	0	1	0
Costa Rica	Santa Bárbara	Count	2017	0	1	0
Costa Rica	Montes de Oro	Proportion	2000	38.4	61.7	15.4
Costa Rica	Montes de Oro	Proportion	2017	41.1	64.9	18.8
Costa Rica	Montes de Oro	Count	2000	0	1	0
Costa Rica	Montes de Oro	Count	2017	0	1	0
Costa Rica	Goicoechea	Proportion	2000	37.9	61.9	17.4
Costa Rica	Goicoechea	Proportion	2017	39.8	62.7	19.7
Costa Rica	Goicoechea	Count	2000	0	1	0
Costa Rica	Goicoechea	Count	2017	0	1	0
Costa Rica	Palmares	Proportion	2000	38.6	61.7	17.4
Costa Rica	Palmares	Proportion	2017	41.6	65.4	18.4
Costa Rica	Palmares	Count	2000	0	1	0
Costa Rica	Palmares	Count	2017	0	1	0
Costa Rica	Nicoya	Proportion	2000	37.9	59.4	17.3
Costa Rica	Nicoya	Proportion	2017	41.7	66.2	20.9
Costa Rica	Nicoya	Count	2000	0	1	0
Costa Rica	Nicoya	Count	2017	0	1	0
Costa Rica	Santo Domingo	Proportion	2000	38.4	62.8	17.0
Costa Rica	Santo Domingo	Proportion	2017	40.3	60.8	18.0
Costa Rica	Santo Domingo	Count	2000	0	1	0
Costa Rica	Santo Domingo	Count	2017	0	1	0
Costa Rica	Osa	Proportion	2000	38.9	62.9	17.1
Costa Rica	Osa	Proportion	2017	41.0	63.2	18.6
Costa Rica	Osa	Count	2000	0	1	0
Costa Rica	Osa	Count	2017	0	1	0
Costa Rica	León Cortés	Proportion	2000	38.6	60.6	16.1
Costa Rica	León Cortés	Proportion	2017	39.5	63.0	19.8
Costa Rica	León Cortés	Count	2000	0	1	0
Costa Rica	León Cortés	Count	2017	0	1	0
Costa Rica	Poás	Proportion	2000	37.3	59.9	18.2
Costa Rica	Poás	Proportion	2017	39.8	60.6	17.7
Costa Rica	Poás	Count	2000	0	1	0
Costa Rica	Poás	Count	2017	0	1	0
Costa Rica	Santa Cruz	Proportion	2000	38.4	59.9	18.2
Costa Rica	Santa Cruz	Proportion	2017	42.3	65.7	20.2
Costa Rica	Santa Cruz	Count	2000	0	1	0
Costa Rica	Santa Cruz	Count	2017	0	1	0
Costa Rica	Sarapiquí	Proportion	2000	36.9	55.5	16.9

Table 4: ORS by Unit *(continued)*

Country	Unit	Metric	Year	Mean	Upper	Lower
Costa Rica	Sarapiquí	Proportion	2017	38.3	61.7	16.8
Costa Rica	Sarapiquí	Count	2000	0	1	0
Costa Rica	Sarapiquí	Count	2017	0	1	0
Costa Rica	Parrita	Proportion	2000	37.9	60.1	15.1
Costa Rica	Parrita	Proportion	2017	40.0	63.1	18.7
Costa Rica	Parrita	Count	2000	0	1	0
Costa Rica	Parrita	Count	2017	0	1	0
Costa Rica	Montes de Oca	Proportion	2000	38.3	62.8	18.4
Costa Rica	Montes de Oca	Proportion	2017	39.8	64.3	19.1
Costa Rica	Montes de Oca	Count	2000	0	1	0
Costa Rica	Montes de Oca	Count	2017	0	1	0
Costa Rica	San Carlos	Proportion	2000	37.1	57.4	17.0
Costa Rica	San Carlos	Proportion	2017	39.4	62.6	19.8
Costa Rica	San Carlos	Count	2000	0	1	0
Costa Rica	San Carlos	Count	2017	0	1	0
Costa Rica	Tilarán	Proportion	2000	38.5	58.8	17.0
Costa Rica	Tilarán	Proportion	2017	41.7	65.1	21.0
Costa Rica	Tilarán	Count	2000	0	1	0
Costa Rica	Tilarán	Count	2017	0	1	0
Costa Rica	Puntarenas	Proportion	2000	39.1	62.7	17.3
Costa Rica	Puntarenas	Proportion	2017	42.2	67.2	19.5
Costa Rica	Puntarenas	Count	2000	0	1	0
Costa Rica	Puntarenas	Count	2017	0	1	0
Costa Rica	Mora	Proportion	2000	38.6	61.8	19.1
Costa Rica	Mora	Proportion	2017	40.8	64.9	19.8
Costa Rica	Mora	Count	2000	0	1	0
Costa Rica	Mora	Count	2017	0	1	0
Costa Rica	San Mateo	Proportion	2000	37.4	59.3	15.3
Costa Rica	San Mateo	Proportion	2017	39.7	63.2	19.0
Costa Rica	San Mateo	Count	2000	0	1	0
Costa Rica	San Mateo	Count	2017	0	1	0
Costa Rica	Moravia	Proportion	2000	37.6	63.0	15.4
Costa Rica	Moravia	Proportion	2017	39.7	63.2	18.2
Costa Rica	Moravia	Count	2000	0	1	0
Costa Rica	Moravia	Count	2017	0	1	0
Costa Rica	San Ramón	Proportion	2000	37.8	61.5	17.1
Costa Rica	San Ramón	Proportion	2017	40.6	63.3	19.2
Costa Rica	San Ramón	Count	2000	0	1	0
Costa Rica	San Ramón	Count	2017	0	1	0
Costa Rica	Pérez Zeledón	Proportion	2000	38.5	61.0	16.9
Costa Rica	Pérez Zeledón	Proportion	2017	39.5	63.7	17.9
Costa Rica	Pérez Zeledón	Count	2000	0	1	0
Costa Rica	Pérez Zeledón	Count	2017	0	1	0
Costa Rica	Upala	Proportion	2000	38.8	58.0	18.9
Costa Rica	Upala	Proportion	2017	41.4	66.8	21.2
Costa Rica	Upala	Count	2000	0	1	0
Costa Rica	Upala	Count	2017	0	1	0
Costa Rica	Puriscal	Proportion	2000	38.2	61.2	17.2
Costa Rica	Puriscal	Proportion	2017	40.5	63.7	19.6

Table 4: ORS by Unit *(continued)*

Country	Unit	Metric	Year	Mean	Upper	Lower
Costa Rica	Puriscal	Count	2000	0	1	0
Costa Rica	Puriscal	Count	2017	0	1	0
Costa Rica	Valverde Vega	Proportion	2000	37.5	60.4	15.7
Costa Rica	Valverde Vega	Proportion	2017	40.0	64.9	17.9
Costa Rica	Valverde Vega	Count	2000	0	1	0
Costa Rica	Valverde Vega	Count	2017	0	1	0
Costa Rica	San José	Proportion	2000	38.7	61.1	17.5
Costa Rica	San José	Proportion	2017	40.4	63.8	18.7
Costa Rica	San José	Count	2000	0	1	0
Costa Rica	San José	Count	2017	0	1	0
Costa Rica	Santa Ana	Proportion	2000	39.5	63.4	17.4
Costa Rica	Santa Ana	Proportion	2017	41.3	64.6	18.5
Costa Rica	Santa Ana	Count	2000	0	1	0
Costa Rica	Santa Ana	Count	2017	0	1	0
Costa Rica	Tarrazú	Proportion	2000	38.4	59.9	16.3
Costa Rica	Tarrazú	Proportion	2017	39.4	64.1	17.6
Costa Rica	Tarrazú	Count	2000	0	1	0
Costa Rica	Tarrazú	Count	2017	0	1	0
Costa Rica	Tibás	Proportion	2000	38.7	63.2	17.7
Costa Rica	Tibás	Proportion	2017	40.7	61.0	18.3
Costa Rica	Tibás	Count	2000	0	1	0
Costa Rica	Tibás	Count	2017	0	1	0
Costa Rica	Turrubares	Proportion	2000	37.7	60.3	15.5
Costa Rica	Turrubares	Proportion	2017	40.1	63.2	19.1
Costa Rica	Turrubares	Count	2000	0	1	0
Costa Rica	Turrubares	Count	2017	0	1	0
Costa Rica	Vásquez de Coronado	Proportion	2000	37.4	60.8	16.9
Costa Rica	Vásquez de Coronado	Proportion	2017	39.2	63.7	17.3
Costa Rica	Vásquez de Coronado	Count	2000	0	1	0
Costa Rica	Vásquez de Coronado	Count	2017	0	1	0
Côte d'Ivoire	Abidjan	Proportion	2000	33.8	48.4	21.8
Côte d'Ivoire	Abidjan	Proportion	2017	24.1	42.0	9.9
Côte d'Ivoire	Abidjan	Count	2000	0	0	0
Côte d'Ivoire	Abidjan	Count	2017	0	0	0
Côte d'Ivoire	Gbôkle	Proportion	2000	16.0	28.2	8.6
Côte d'Ivoire	Gbôkle	Proportion	2017	16.9	32.6	7.6
Côte d'Ivoire	Gbôkle	Count	2000	0	0	0
Côte d'Ivoire	Gbôkle	Count	2017	0	0	0
Côte d'Ivoire	Indénié-Djuablin	Proportion	2000	17.1	28.3	9.6
Côte d'Ivoire	Indénié-Djuablin	Proportion	2017	18.8	35.4	7.5
Côte d'Ivoire	Indénié-Djuablin	Count	2000	0	0	0
Côte d'Ivoire	Indénié-Djuablin	Count	2017	0	0	0
Côte d'Ivoire	Folon	Proportion	2000	11.5	22.4	5.3
Côte d'Ivoire	Folon	Proportion	2017	14.2	26.1	5.7
Côte d'Ivoire	Folon	Count	2000	0	0	0
Côte d'Ivoire	Folon	Count	2017	0	0	0
Côte d'Ivoire	Gôh	Proportion	2000	14.9	24.1	8.5
Côte d'Ivoire	Gôh	Proportion	2017	13.9	26.1	5.3
Côte d'Ivoire	Gôh	Count	2000	0	0	0

Table 4: ORS by Unit *(continued)*

Country	Unit	Metric	Year	Mean	Upper	Lower
Côte d'Ivoire	Gôh	Count	2017	0	0	0
Côte d'Ivoire	Bélier	Proportion	2000	18.0	29.2	9.8
Côte d'Ivoire	Bélier	Proportion	2017	14.0	26.3	5.3
Côte d'Ivoire	Bélier	Count	2000	0	0	0
Côte d'Ivoire	Bélier	Count	2017	0	0	0
Côte d'Ivoire	Agnéby-Tiassa	Proportion	2000	22.3	34.9	12.8
Côte d'Ivoire	Agnéby-Tiassa	Proportion	2017	16.8	31.4	6.4
Côte d'Ivoire	Agnéby-Tiassa	Count	2000	0	0	0
Côte d'Ivoire	Agnéby-Tiassa	Count	2017	0	0	0
Côte d'Ivoire	Cavally	Proportion	2000	13.0	21.1	6.8
Côte d'Ivoire	Cavally	Proportion	2017	22.6	39.9	10.2
Côte d'Ivoire	Cavally	Count	2000	0	0	0
Côte d'Ivoire	Cavally	Count	2017	0	0	0
Côte d'Ivoire	Haut-Sassandra	Proportion	2000	13.2	20.0	8.2
Côte d'Ivoire	Haut-Sassandra	Proportion	2017	14.4	30.1	5.8
Côte d'Ivoire	Haut-Sassandra	Count	2000	0	0	0
Côte d'Ivoire	Haut-Sassandra	Count	2017	0	0	0
Côte d'Ivoire	Bagoué	Proportion	2000	13.3	22.8	6.8
Côte d'Ivoire	Bagoué	Proportion	2017	14.3	28.3	6.4
Côte d'Ivoire	Bagoué	Count	2000	0	0	0
Côte d'Ivoire	Bagoué	Count	2017	0	0	0
Côte d'Ivoire	Gbeke	Proportion	2000	23.3	38.4	12.8
Côte d'Ivoire	Gbeke	Proportion	2017	15.4	29.8	6.0
Côte d'Ivoire	Gbeke	Count	2000	0	0	0
Côte d'Ivoire	Gbeke	Count	2017	0	0	0
Côte d'Ivoire	Bafing	Proportion	2000	13.2	24.4	6.2
Côte d'Ivoire	Bafing	Proportion	2017	15.9	30.1	7.2
Côte d'Ivoire	Bafing	Count	2000	0	0	0
Côte d'Ivoire	Bafing	Count	2017	0	0	0
Côte d'Ivoire	Yamoussoukro	Proportion	2000	17.1	28.8	9.4
Côte d'Ivoire	Yamoussoukro	Proportion	2017	13.4	26.7	4.5
Côte d'Ivoire	Yamoussoukro	Count	2000	0	0	0
Côte d'Ivoire	Yamoussoukro	Count	2017	0	0	0
Côte d'Ivoire	Bounkani	Proportion	2000	12.2	20.8	6.2
Côte d'Ivoire	Bounkani	Proportion	2017	17.7	31.6	8.1
Côte d'Ivoire	Bounkani	Count	2000	0	0	0
Côte d'Ivoire	Bounkani	Count	2017	0	0	0
Côte d'Ivoire	Nawa	Proportion	2000	12.2	20.6	6.8
Côte d'Ivoire	Nawa	Proportion	2017	16.0	29.2	6.7
Côte d'Ivoire	Nawa	Count	2000	0	0	0
Côte d'Ivoire	Nawa	Count	2017	0	0	0
Côte d'Ivoire	Sud Comoé	Proportion	2000	27.5	40.9	16.0
Côte d'Ivoire	Sud Comoé	Proportion	2017	24.7	41.8	11.8
Côte d'Ivoire	Sud Comoé	Count	2000	0	0	0
Côte d'Ivoire	Sud Comoé	Count	2017	0	0	0
Côte d'Ivoire	Kabadougou	Proportion	2000	12.5	21.5	5.7
Côte d'Ivoire	Kabadougou	Proportion	2017	14.7	26.8	5.9
Côte d'Ivoire	Kabadougou	Count	2000	0	0	0
Côte d'Ivoire	Kabadougou	Count	2017	0	0	0

Table 4: ORS by Unit *(continued)*

Country	Unit	Metric	Year	Mean	Upper	Lower
Côte d'Ivoire	Lôh-Djiboua	Proportion	2000	19.2	30.8	11.2
Côte d'Ivoire	Lôh-Djiboua	Proportion	2017	15.7	29.0	5.8
Côte d'Ivoire	Lôh-Djiboua	Count	2000	0	0	0
Côte d'Ivoire	Lôh-Djiboua	Count	2017	0	0	0
Côte d'Ivoire	Iffou	Proportion	2000	16.7	28.1	8.4
Côte d'Ivoire	Iffou	Proportion	2017	16.3	31.2	5.8
Côte d'Ivoire	Iffou	Count	2000	0	0	0
Côte d'Ivoire	Iffou	Count	2017	0	0	0
Côte d'Ivoire	Grands Ponts	Proportion	2000	27.3	41.0	16.1
Côte d'Ivoire	Grands Ponts	Proportion	2017	20.3	36.4	7.6
Côte d'Ivoire	Grands Ponts	Count	2000	0	0	0
Côte d'Ivoire	Grands Ponts	Count	2017	0	0	0
Côte d'Ivoire	Guémon	Proportion	2000	12.6	20.9	7.0
Côte d'Ivoire	Guémon	Proportion	2017	19.4	36.2	8.4
Côte d'Ivoire	Guémon	Count	2000	0	0	0
Côte d'Ivoire	Guémon	Count	2017	0	0	0
Côte d'Ivoire	Marahoué	Proportion	2000	15.6	24.4	9.6
Côte d'Ivoire	Marahoué	Proportion	2017	13.4	29.5	5.5
Côte d'Ivoire	Marahoué	Count	2000	0	0	0
Côte d'Ivoire	Marahoué	Count	2017	0	0	0
Côte d'Ivoire	Poro	Proportion	2000	14.6	24.0	7.3
Côte d'Ivoire	Poro	Proportion	2017	15.1	31.6	6.9
Côte d'Ivoire	Poro	Count	2000	0	0	0
Côte d'Ivoire	Poro	Count	2017	0	0	0
Côte d'Ivoire	Hambol	Proportion	2000	19.1	30.8	10.1
Côte d'Ivoire	Hambol	Proportion	2017	15.5	29.2	7.3
Côte d'Ivoire	Hambol	Count	2000	0	0	0
Côte d'Ivoire	Hambol	Count	2017	0	0	0
Côte d'Ivoire	Béré	Proportion	2000	16.9	28.3	9.4
Côte d'Ivoire	Béré	Proportion	2017	14.3	27.5	6.0
Côte d'Ivoire	Béré	Count	2000	0	0	0
Côte d'Ivoire	Béré	Count	2017	0	0	0
Côte d'Ivoire	Gontougo	Proportion	2000	12.8	20.7	6.6
Côte d'Ivoire	Gontougo	Proportion	2017	17.9	35.0	7.8
Côte d'Ivoire	Gontougo	Count	2000	0	0	0
Côte d'Ivoire	Gontougo	Count	2017	0	0	0
Côte d'Ivoire	San-Pédro	Proportion	2000	14.4	24.6	7.0
Côte d'Ivoire	San-Pédro	Proportion	2017	20.0	37.1	9.5
Côte d'Ivoire	San-Pédro	Count	2000	0	0	0
Côte d'Ivoire	San-Pédro	Count	2017	0	0	0
Côte d'Ivoire	Moronou	Proportion	2000	17.3	30.2	8.9
Côte d'Ivoire	Moronou	Proportion	2017	15.5	28.6	5.2
Côte d'Ivoire	Moronou	Count	2000	0	0	0
Côte d'Ivoire	Moronou	Count	2017	0	0	0
Côte d'Ivoire	La Mé	Proportion	2000	22.1	34.2	13.1
Côte d'Ivoire	La Mé	Proportion	2017	18.8	33.8	7.7
Côte d'Ivoire	La Mé	Count	2000	0	0	0
Côte d'Ivoire	La Mé	Count	2017	0	0	0
Côte d'Ivoire	Tonkpi	Proportion	2000	14.1	22.8	7.7

Table 4: ORS by Unit *(continued)*

Country	Unit	Metric	Year	Mean	Upper	Lower
Côte d'Ivoire	Tonkpi	Proportion	2017	21.7	37.4	9.4
Côte d'Ivoire	Tonkpi	Count	2000	0	0	0
Côte d'Ivoire	Tonkpi	Count	2017	0	0	0
Côte d'Ivoire	Tchologo	Proportion	2000	15.2	24.9	7.2
Côte d'Ivoire	Tchologo	Proportion	2017	15.8	32.3	7.7
Côte d'Ivoire	Tchologo	Count	2000	0	0	0
Côte d'Ivoire	Tchologo	Count	2017	0	0	0
Côte d'Ivoire	Worodougou	Proportion	2000	14.3	24.2	7.0
Côte d'Ivoire	Worodougou	Proportion	2017	14.8	30.9	6.0
Côte d'Ivoire	Worodougou	Count	2000	0	0	0
Côte d'Ivoire	Worodougou	Count	2017	0	0	0
Côte d'Ivoire	N'zi	Proportion	2000	17.9	29.4	9.5
Côte d'Ivoire	N'zi	Proportion	2017	15.2	29.4	5.2
Côte d'Ivoire	N'zi	Count	2000	0	0	0
Côte d'Ivoire	N'zi	Count	2017	0	0	0
Democratic Republic of the Congo	Aketi	Proportion	2000	19.6	33.7	7.9
Democratic Republic of the Congo	Aketi	Proportion	2017	30.8	49.1	15.4
Democratic Republic of the Congo	Aketi	Count	2000	0	0	0
Democratic Republic of the Congo	Aketi	Count	2017	0	0	0
Democratic Republic of the Congo	Basankusu	Proportion	2000	17.8	31.4	8.4
Democratic Republic of the Congo	Basankusu	Proportion	2017	40.2	61.7	20.3
Democratic Republic of the Congo	Basankusu	Count	2000	0	0	0
Democratic Republic of the Congo	Basankusu	Count	2017	0	1	0
Democratic Republic of the Congo	Kambove	Proportion	2000	16.9	28.8	7.2
Democratic Republic of the Congo	Kambove	Proportion	2017	31.4	52.4	15.2
Democratic Republic of the Congo	Kambove	Count	2000	0	0	0
Democratic Republic of the Congo	Kambove	Count	2017	0	1	0
Democratic Republic of the Congo	Bukama	Proportion	2000	18.8	32.1	9.7
Democratic Republic of the Congo	Bukama	Proportion	2017	38.6	61.5	19.8
Democratic Republic of the Congo	Bukama	Count	2000	0	0	0
Democratic Republic of the Congo	Bukama	Count	2017	0	1	0
Democratic Republic of the Congo	Aba	Proportion	2000	20.8	38.5	9.3
Democratic Republic of the Congo	Aba	Proportion	2017	34.0	56.9	12.8

Table 4: ORS by Unit *(continued)*

Country	Unit	Metric	Year	Mean	Upper	Lower
Democratic Republic of the Congo	Aba	Count	2000	0	0	0
Democratic Republic of the Congo	Aba	Count	2017	0	1	0
Democratic Republic of the Congo	Ariwara	Proportion	2000	29.6	51.9	12.7
Democratic Republic of the Congo	Ariwara	Proportion	2017	54.8	78.9	27.5
Democratic Republic of the Congo	Ariwara	Count	2000	0	1	0
Democratic Republic of the Congo	Ariwara	Count	2017	1	1	0
Democratic Republic of the Congo	Demba	Proportion	2000	17.1	30.1	8.1
Democratic Republic of the Congo	Demba	Proportion	2017	40.6	61.1	21.0
Democratic Republic of the Congo	Demba	Count	2000	0	0	0
Democratic Republic of the Congo	Demba	Count	2017	0	1	0
Democratic Republic of the Congo	Kabeya-Kamwanga	Proportion	2000	15.8	31.2	7.0
Democratic Republic of the Congo	Kabeya-Kamwanga	Proportion	2017	39.9	62.4	20.5
Democratic Republic of the Congo	Kabeya-Kamwanga	Count	2000	0	0	0
Democratic Republic of the Congo	Kabeya-Kamwanga	Count	2017	0	1	0
Democratic Republic of the Congo	Dekese	Proportion	2000	16.0	26.2	7.5
Democratic Republic of the Congo	Dekese	Proportion	2017	36.4	58.0	17.9
Democratic Republic of the Congo	Dekese	Count	2000	0	0	0
Democratic Republic of the Congo	Dekese	Count	2017	0	1	0
Democratic Republic of the Congo	Kinshasa	Proportion	2000	28.6	46.9	14.5
Democratic Republic of the Congo	Kinshasa	Proportion	2017	38.9	63.1	20.3
Democratic Republic of the Congo	Kinshasa	Count	2000	0	0	0
Democratic Republic of the Congo	Kinshasa	Count	2017	0	1	0
Democratic Republic of the Congo	Bangu	Proportion	2000	30.5	49.9	13.3
Democratic Republic of the Congo	Bangu	Proportion	2017	52.3	77.9	29.0
Democratic Republic of the Congo	Bangu	Count	2000	0	0	0
Democratic Republic of the Congo	Bangu	Count	2017	1	1	0

Table 4: ORS by Unit *(continued)*

Country	Unit	Metric	Year	Mean	Upper	Lower
Democratic Republic of the Congo	Feshi	Proportion	2000	14.7	26.1	6.8
Democratic Republic of the Congo	Feshi	Proportion	2017	37.5	57.0	19.2
Democratic Republic of the Congo	Feshi	Count	2000	0	0	0
Democratic Republic of the Congo	Feshi	Count	2017	0	1	0
Democratic Republic of the Congo	Bagata	Proportion	2000	16.5	29.3	8.3
Democratic Republic of the Congo	Bagata	Proportion	2017	40.9	65.7	22.9
Democratic Republic of the Congo	Bagata	Count	2000	0	0	0
Democratic Republic of the Congo	Bagata	Count	2017	0	1	0
Democratic Republic of the Congo	Kabinda	Proportion	2000	15.1	26.2	6.9
Democratic Republic of the Congo	Kabinda	Proportion	2017	39.8	60.8	21.5
Democratic Republic of the Congo	Kabinda	Count	2000	0	0	0
Democratic Republic of the Congo	Kabinda	Count	2017	0	1	0
Democratic Republic of the Congo	Dilolo	Proportion	2000	20.6	37.1	9.5
Democratic Republic of the Congo	Dilolo	Proportion	2017	25.9	44.7	12.1
Democratic Republic of the Congo	Dilolo	Count	2000	0	0	0
Democratic Republic of the Congo	Dilolo	Count	2017	0	0	0
Democratic Republic of the Congo	Bolobo	Proportion	2000	18.7	33.2	7.8
Democratic Republic of the Congo	Bolobo	Proportion	2017	37.9	59.9	20.3
Democratic Republic of the Congo	Bolobo	Count	2000	0	0	0
Democratic Republic of the Congo	Bolobo	Count	2017	0	1	0
Democratic Republic of the Congo	Kabambare	Proportion	2000	16.2	30.0	7.5
Democratic Republic of the Congo	Kabambare	Proportion	2017	39.3	62.1	21.5
Democratic Republic of the Congo	Kabambare	Count	2000	0	0	0
Democratic Republic of the Congo	Kabambare	Count	2017	0	1	0
Democratic Republic of the Congo	Bongandanga	Proportion	2000	18.4	29.9	9.1
Democratic Republic of the Congo	Bongandanga	Proportion	2017	35.5	57.4	18.6

Table 4: ORS by Unit *(continued)*

Country	Unit	Metric	Year	Mean	Upper	Lower
Democratic Republic of the Congo	Bongandanga	Count	2000	0	0	0
Democratic Republic of the Congo	Bongandanga	Count	2017	0	1	0
Democratic Republic of the Congo	Beni	Proportion	2000	20.0	37.1	9.7
Democratic Republic of the Congo	Beni	Proportion	2017	34.5	55.3	16.3
Democratic Republic of the Congo	Beni	Count	2000	0	0	0
Democratic Republic of the Congo	Beni	Count	2017	0	1	0
Democratic Republic of the Congo	Bosobolo	Proportion	2000	19.3	33.3	8.8
Democratic Republic of the Congo	Bosobolo	Proportion	2017	29.3	48.8	14.5
Democratic Republic of the Congo	Bosobolo	Count	2000	0	0	0
Democratic Republic of the Congo	Bosobolo	Count	2017	0	0	0
Democratic Republic of the Congo	Bena-Dibele	Proportion	2000	NA	NA	NA
Democratic Republic of the Congo	Bena-Dibele	Proportion	2017	NA	NA	NA
Democratic Republic of the Congo	Bena-Dibele	Count	2000	NA	NA	NA
Democratic Republic of the Congo	Bena-Dibele	Count	2017	NA	NA	NA
Democratic Republic of the Congo	Baraka	Proportion	2000	22.1	47.2	7.4
Democratic Republic of the Congo	Baraka	Proportion	2017	41.5	67.3	17.9
Democratic Republic of the Congo	Baraka	Count	2000	0	0	0
Democratic Republic of the Congo	Baraka	Count	2017	0	1	0
Democratic Republic of the Congo	Budjala	Proportion	2000	19.3	33.8	8.9
Democratic Republic of the Congo	Budjala	Proportion	2017	33.8	54.0	15.5
Democratic Republic of the Congo	Budjala	Count	2000	0	0	0
Democratic Republic of the Congo	Budjala	Count	2017	0	1	0
Democratic Republic of the Congo	Kabalo	Proportion	2000	16.0	27.2	7.7
Democratic Republic of the Congo	Kabalo	Proportion	2017	32.4	54.6	17.0
Democratic Republic of the Congo	Kabalo	Count	2000	0	0	0
Democratic Republic of the Congo	Kabalo	Count	2017	0	1	0

Table 4: ORS by Unit *(continued)*

Country	Unit	Metric	Year	Mean	Upper	Lower
Democratic Republic of the Congo	Bafwasende	Proportion	2000	19.6	33.7	9.7
Democratic Republic of the Congo	Bafwasende	Proportion	2017	33.0	50.8	17.4
Democratic Republic of the Congo	Bafwasende	Count	2000	0	0	0
Democratic Republic of the Congo	Bafwasende	Count	2017	0	1	0
Democratic Republic of the Congo	Befale	Proportion	2000	16.1	27.4	7.5
Democratic Republic of the Congo	Befale	Proportion	2017	36.1	55.4	18.0
Democratic Republic of the Congo	Befale	Count	2000	0	0	0
Democratic Republic of the Congo	Befale	Count	2017	0	1	0
Democratic Republic of the Congo	Aketi (ville)	Proportion	2000	20.6	37.1	7.7
Democratic Republic of the Congo	Aketi (ville)	Proportion	2017	34.2	58.2	15.6
Democratic Republic of the Congo	Aketi (ville)	Count	2000	0	0	0
Democratic Republic of the Congo	Aketi (ville)	Count	2017	0	1	0
Democratic Republic of the Congo	Basankusu (ville)	Proportion	2000	18.8	34.9	7.3
Democratic Republic of the Congo	Basankusu (ville)	Proportion	2017	41.6	66.2	19.7
Democratic Republic of the Congo	Basankusu (ville)	Count	2000	0	0	0
Democratic Republic of the Congo	Basankusu (ville)	Count	2017	0	1	0
Democratic Republic of the Congo	Kasenga	Proportion	2000	18.5	31.6	8.8
Democratic Republic of the Congo	Kasenga	Proportion	2017	31.3	50.7	14.4
Democratic Republic of the Congo	Kasenga	Count	2000	0	0	0
Democratic Republic of the Congo	Kasenga	Count	2017	0	1	0
Democratic Republic of the Congo	Kabongo	Proportion	2000	16.0	26.3	8.0
Democratic Republic of the Congo	Kabongo	Proportion	2017	35.9	55.3	16.8
Democratic Republic of the Congo	Kabongo	Count	2000	0	0	0
Democratic Republic of the Congo	Kabongo	Count	2017	0	1	0
Democratic Republic of the Congo	Dungu	Proportion	2000	20.0	35.0	9.0
Democratic Republic of the Congo	Dungu	Proportion	2017	28.3	45.0	13.0

Table 4: ORS by Unit *(continued)*

Country	Unit	Metric	Year	Mean	Upper	Lower
Democratic Republic of the Congo	Dungu	Count	2000	0	0	0
Democratic Republic of the Congo	Dungu	Count	2017	0	0	0
Democratic Republic of the Congo	Aru	Proportion	2000	24.7	39.7	12.2
Democratic Republic of the Congo	Aru	Proportion	2017	40.5	63.3	21.8
Democratic Republic of the Congo	Aru	Count	2000	0	0	0
Democratic Republic of the Congo	Aru	Count	2017	0	1	0
Democratic Republic of the Congo	Dibaya	Proportion	2000	16.3	30.6	7.5
Democratic Republic of the Congo	Dibaya	Proportion	2017	38.4	60.2	19.6
Democratic Republic of the Congo	Dibaya	Count	2000	0	0	0
Democratic Republic of the Congo	Dibaya	Count	2017	0	1	0
Democratic Republic of the Congo	Katanda	Proportion	2000	19.0	34.1	8.3
Democratic Republic of the Congo	Katanda	Proportion	2017	40.1	62.7	19.3
Democratic Republic of the Congo	Katanda	Count	2000	0	0	0
Democratic Republic of the Congo	Katanda	Count	2017	0	1	0
Democratic Republic of the Congo	Ilebo	Proportion	2000	16.9	29.1	8.0
Democratic Republic of the Congo	Ilebo	Proportion	2017	38.9	60.9	21.4
Democratic Republic of the Congo	Ilebo	Count	2000	0	0	0
Democratic Republic of the Congo	Ilebo	Count	2017	0	1	0
Democratic Republic of the Congo	Boma	Proportion	2000	26.0	47.8	11.2
Democratic Republic of the Congo	Boma	Proportion	2017	42.3	68.0	19.8
Democratic Republic of the Congo	Boma	Count	2000	0	0	0
Democratic Republic of the Congo	Boma	Count	2017	0	1	0
Democratic Republic of the Congo	Kahemba	Proportion	2000	15.5	27.1	6.7
Democratic Republic of the Congo	Kahemba	Proportion	2017	32.4	52.4	16.6
Democratic Republic of the Congo	Kahemba	Count	2000	0	0	0
Democratic Republic of the Congo	Kahemba	Count	2017	0	1	0

Table 4: ORS by Unit *(continued)*

Country	Unit	Metric	Year	Mean	Upper	Lower
Democratic Republic of the Congo	Bandundu	Proportion	2000	18.0	33.2	7.8
Democratic Republic of the Congo	Bandundu	Proportion	2017	39.8	63.7	19.9
Democratic Republic of the Congo	Bandundu	Count	2000	0	0	0
Democratic Republic of the Congo	Bandundu	Count	2017	0	1	0
Democratic Republic of the Congo	Kabinda (ville)	Proportion	2000	18.3	35.4	6.9
Democratic Republic of the Congo	Kabinda (ville)	Proportion	2017	44.9	68.5	23.1
Democratic Republic of the Congo	Kabinda (ville)	Count	2000	0	0	0
Democratic Republic of the Congo	Kabinda (ville)	Count	2017	0	1	0
Democratic Republic of the Congo	Kapanga	Proportion	2000	16.6	31.4	8.0
Democratic Republic of the Congo	Kapanga	Proportion	2017	32.8	51.5	16.7
Democratic Republic of the Congo	Kapanga	Count	2000	0	0	0
Democratic Republic of the Congo	Kapanga	Count	2017	0	1	0
Democratic Republic of the Congo	Bolobo (ville)	Proportion	2000	20.9	37.4	8.1
Democratic Republic of the Congo	Bolobo (ville)	Proportion	2017	37.7	62.8	16.7
Democratic Republic of the Congo	Bolobo (ville)	Count	2000	0	0	0
Democratic Republic of the Congo	Bolobo (ville)	Count	2017	0	1	0
Democratic Republic of the Congo	Kailo	Proportion	2000	19.0	30.7	9.3
Democratic Republic of the Congo	Kailo	Proportion	2017	39.8	59.0	24.8
Democratic Republic of the Congo	Kailo	Count	2000	0	0	0
Democratic Republic of the Congo	Kailo	Count	2017	0	1	0
Democratic Republic of the Congo	Bumba	Proportion	2000	21.8	37.7	8.8
Democratic Republic of the Congo	Bumba	Proportion	2017	34.3	54.8	18.0
Democratic Republic of the Congo	Bumba	Count	2000	0	0	0
Democratic Republic of the Congo	Bumba	Count	2017	0	1	0
Democratic Republic of the Congo	Butembo	Proportion	2000	23.4	41.0	11.2
Democratic Republic of the Congo	Butembo	Proportion	2017	41.2	62.4	21.4

Table 4: ORS by Unit *(continued)*

Country	Unit	Metric	Year	Mean	Upper	Lower
Democratic Republic of the Congo	Butembo	Count	2000	0	0	0
Democratic Republic of the Congo	Butembo	Count	2017	0	1	0
Democratic Republic of the Congo	Businga	Proportion	2000	19.3	35.7	8.8
Democratic Republic of the Congo	Businga	Proportion	2017	30.5	51.3	15.3
Democratic Republic of the Congo	Businga	Count	2000	0	0	0
Democratic Republic of the Congo	Businga	Count	2017	0	1	0
Democratic Republic of the Congo	Katako-Kombe	Proportion	2000	16.5	30.1	7.3
Democratic Republic of the Congo	Katako-Kombe	Proportion	2017	35.0	55.3	18.6
Democratic Republic of the Congo	Katako-Kombe	Count	2000	0	0	0
Democratic Republic of the Congo	Katako-Kombe	Count	2017	0	1	0
Democratic Republic of the Congo	Bukavu	Proportion	2000	19.2	34.8	8.3
Democratic Republic of the Congo	Bukavu	Proportion	2017	37.6	61.7	18.0
Democratic Republic of the Congo	Bukavu	Count	2000	0	0	0
Democratic Republic of the Congo	Bukavu	Count	2017	0	1	0
Democratic Republic of the Congo	Gemena	Proportion	2000	20.1	36.3	9.3
Democratic Republic of the Congo	Gemena	Proportion	2017	30.8	51.3	14.3
Democratic Republic of the Congo	Gemena	Count	2000	0	0	0
Democratic Republic of the Congo	Gemena	Count	2017	0	1	0
Democratic Republic of the Congo	Kalemie	Proportion	2000	16.6	29.8	7.2
Democratic Republic of the Congo	Kalemie	Proportion	2017	30.5	52.8	13.4
Democratic Republic of the Congo	Kalemie	Count	2000	0	0	0
Democratic Republic of the Congo	Kalemie	Count	2017	0	1	0
Democratic Republic of the Congo	Banalia	Proportion	2000	21.6	39.5	10.9
Democratic Republic of the Congo	Banalia	Proportion	2017	35.2	52.5	20.2
Democratic Republic of the Congo	Banalia	Count	2000	0	0	0
Democratic Republic of the Congo	Banalia	Count	2017	0	1	0

Table 4: ORS by Unit *(continued)*

Country	Unit	Metric	Year	Mean	Upper	Lower
Democratic Republic of the Congo	Boende	Proportion	2000	17.1	30.6	8.1
Democratic Republic of the Congo	Boende	Proportion	2017	37.7	58.1	18.8
Democratic Republic of the Congo	Boende	Count	2000	0	0	0
Democratic Republic of the Congo	Boende	Count	2017	0	1	0
Democratic Republic of the Congo	Ango	Proportion	2000	20.2	33.8	9.3
Democratic Republic of the Congo	Ango	Proportion	2017	30.1	49.6	14.3
Democratic Republic of the Congo	Ango	Count	2000	0	0	0
Democratic Republic of the Congo	Ango	Count	2017	0	0	0
Democratic Republic of the Congo	Bikoro	Proportion	2000	18.7	31.0	8.9
Democratic Republic of the Congo	Bikoro	Proportion	2017	42.3	66.5	23.3
Democratic Republic of the Congo	Bikoro	Count	2000	0	0	0
Democratic Republic of the Congo	Bikoro	Count	2017	0	1	0
Democratic Republic of the Congo	Kipushi	Proportion	2000	19.1	35.3	8.1
Democratic Republic of the Congo	Kipushi	Proportion	2017	34.9	56.7	16.5
Democratic Republic of the Congo	Kipushi	Count	2000	0	0	0
Democratic Republic of the Congo	Kipushi	Count	2017	0	1	0
Democratic Republic of the Congo	Kamina	Proportion	2000	16.4	26.0	7.8
Democratic Republic of the Congo	Kamina	Proportion	2017	35.8	57.4	18.4
Democratic Republic of the Congo	Kamina	Count	2000	0	0	0
Democratic Republic of the Congo	Kamina	Count	2017	0	1	0
Democratic Republic of the Congo	Dungu (ville)	Proportion	2000	22.0	41.5	8.6
Democratic Republic of the Congo	Dungu (ville)	Proportion	2017	35.1	58.8	15.1
Democratic Republic of the Congo	Dungu (ville)	Count	2000	0	0	0
Democratic Republic of the Congo	Dungu (ville)	Count	2017	0	1	0
Democratic Republic of the Congo	Aru (ville)	Proportion	2000	27.9	47.9	12.8
Democratic Republic of the Congo	Aru (ville)	Proportion	2017	45.2	69.9	23.3

Table 4: ORS by Unit *(continued)*

Country	Unit	Metric	Year	Mean	Upper	Lower
Democratic Republic of the Congo	Aru (ville)	Count	2000	0	0	0
Democratic Republic of the Congo	Aru (ville)	Count	2017	0	1	0
Democratic Republic of the Congo	Dimbelenge	Proportion	2000	16.4	29.5	7.5
Democratic Republic of the Congo	Dimbelenge	Proportion	2017	37.7	57.9	18.9
Democratic Republic of the Congo	Dimbelenge	Count	2000	0	0	0
Democratic Republic of the Congo	Dimbelenge	Count	2017	0	1	0
Democratic Republic of the Congo	Lukalaba	Proportion	2000	14.8	31.2	4.7
Democratic Republic of the Congo	Lukalaba	Proportion	2017	38.2	63.1	17.0
Democratic Republic of the Congo	Lukalaba	Count	2000	0	0	0
Democratic Republic of the Congo	Lukalaba	Count	2017	0	1	0
Democratic Republic of the Congo	Ilebo (ville)	Proportion	2000	17.7	32.3	7.5
Democratic Republic of the Congo	Ilebo (ville)	Proportion	2017	37.9	64.3	15.6
Democratic Republic of the Congo	Ilebo (ville)	Count	2000	0	0	0
Democratic Republic of the Congo	Ilebo (ville)	Count	2017	0	1	0
Democratic Republic of the Congo	Inkisi	Proportion	2000	30.5	49.2	15.7
Democratic Republic of the Congo	Inkisi	Proportion	2017	48.9	72.8	22.8
Democratic Republic of the Congo	Inkisi	Count	2000	0	0	0
Democratic Republic of the Congo	Inkisi	Count	2017	0	1	0
Democratic Republic of the Congo	Kahemba (ville)	Proportion	2000	16.0	31.2	6.2
Democratic Republic of the Congo	Kahemba (ville)	Proportion	2017	36.7	58.7	15.5
Democratic Republic of the Congo	Kahemba (ville)	Count	2000	0	0	0
Democratic Republic of the Congo	Kahemba (ville)	Count	2017	0	1	0
Democratic Republic of the Congo	Bulungu	Proportion	2000	16.5	29.1	7.8
Democratic Republic of the Congo	Bulungu	Proportion	2017	43.8	67.9	24.4
Democratic Republic of the Congo	Bulungu	Count	2000	0	0	0
Democratic Republic of the Congo	Bulungu	Count	2017	0	1	0

Table 4: ORS by Unit *(continued)*

Country	Unit	Metric	Year	Mean	Upper	Lower
Democratic Republic of the Congo	Kamiji	Proportion	2000	15.5	27.4	7.2
Democratic Republic of the Congo	Kamiji	Proportion	2017	39.0	60.8	18.8
Democratic Republic of the Congo	Kamiji	Count	2000	0	0	0
Democratic Republic of the Congo	Kamiji	Count	2017	0	1	0
Democratic Republic of the Congo	Kasaji	Proportion	2000	20.5	39.2	8.8
Democratic Republic of the Congo	Kasaji	Proportion	2017	29.3	53.3	10.7
Democratic Republic of the Congo	Kasaji	Count	2000	0	0	0
Democratic Republic of the Congo	Kasaji	Count	2017	0	1	0
Democratic Republic of the Congo	Inongo	Proportion	2000	16.8	27.5	8.4
Democratic Republic of the Congo	Inongo	Proportion	2017	37.4	58.3	21.1
Democratic Republic of the Congo	Inongo	Count	2000	0	0	0
Democratic Republic of the Congo	Inongo	Count	2017	0	1	0
Democratic Republic of the Congo	Kalima	Proportion	2000	16.5	29.4	7.1
Democratic Republic of the Congo	Kalima	Proportion	2017	36.2	59.0	19.2
Democratic Republic of the Congo	Kalima	Count	2000	0	0	0
Democratic Republic of the Congo	Kalima	Count	2017	0	1	0
Democratic Republic of the Congo	Bumba (ville)	Proportion	2000	22.3	42.1	8.3
Democratic Republic of the Congo	Bumba (ville)	Proportion	2017	34.9	62.4	15.1
Democratic Republic of the Congo	Bumba (ville)	Count	2000	0	0	0
Democratic Republic of the Congo	Bumba (ville)	Count	2017	0	1	0
Democratic Republic of the Congo	Goma	Proportion	2000	19.3	37.3	8.3
Democratic Republic of the Congo	Goma	Proportion	2017	38.5	62.4	17.7
Democratic Republic of the Congo	Goma	Count	2000	0	0	0
Democratic Republic of the Congo	Goma	Count	2017	0	1	0
Democratic Republic of the Congo	Gbadolite	Proportion	2000	19.8	35.4	8.6
Democratic Republic of the Congo	Gbadolite	Proportion	2017	33.7	57.8	15.4

Table 4: ORS by Unit *(continued)*

Country	Unit	Metric	Year	Mean	Upper	Lower
Democratic Republic of the Congo	Gbadolite	Count	2000	0	0	0
Democratic Republic of the Congo	Gbadolite	Count	2017	0	1	0
Democratic Republic of the Congo	Kole	Proportion	2000	16.5	27.4	7.7
Democratic Republic of the Congo	Kole	Proportion	2017	36.1	56.3	18.2
Democratic Republic of the Congo	Kole	Count	2000	0	0	0
Democratic Republic of the Congo	Kole	Count	2017	0	1	0
Democratic Republic of the Congo	Fizi	Proportion	2000	19.8	34.8	8.8
Democratic Republic of the Congo	Fizi	Proportion	2017	37.7	57.8	21.7
Democratic Republic of the Congo	Fizi	Count	2000	0	0	0
Democratic Republic of the Congo	Fizi	Count	2017	0	1	0
Democratic Republic of the Congo	Gemena (ville)	Proportion	2000	19.5	37.1	7.7
Democratic Republic of the Congo	Gemena (ville)	Proportion	2017	40.5	66.4	20.1
Democratic Republic of the Congo	Gemena (ville)	Count	2000	0	0	0
Democratic Republic of the Congo	Gemena (ville)	Count	2017	0	1	0
Democratic Republic of the Congo	Kalemie (ville)	Proportion	2000	21.1	38.8	9.0
Democratic Republic of the Congo	Kalemie (ville)	Proportion	2017	36.7	65.1	16.5
Democratic Republic of the Congo	Kalemie (ville)	Count	2000	0	0	0
Democratic Republic of the Congo	Kalemie (ville)	Count	2017	0	1	0
Democratic Republic of the Congo	Basoko	Proportion	2000	19.5	34.3	8.7
Democratic Republic of the Congo	Basoko	Proportion	2017	28.0	47.1	14.7
Democratic Republic of the Congo	Basoko	Count	2000	0	0	0
Democratic Republic of the Congo	Basoko	Count	2017	0	0	0
Democratic Republic of the Congo	Boende (ville)	Proportion	2000	18.3	35.7	8.0
Democratic Republic of the Congo	Boende (ville)	Proportion	2017	38.6	63.7	16.6
Democratic Republic of the Congo	Boende (ville)	Count	2000	0	0	0
Democratic Republic of the Congo	Boende (ville)	Count	2017	0	1	0

Table 4: ORS by Unit *(continued)*

Country	Unit	Metric	Year	Mean	Upper	Lower
Democratic Republic of the Congo	Bambesa	Proportion	2000	20.4	34.0	9.1
Democratic Republic of the Congo	Bambesa	Proportion	2017	31.0	49.7	14.7
Democratic Republic of the Congo	Bambesa	Count	2000	0	0	0
Democratic Republic of the Congo	Bambesa	Count	2017	0	0	0
Democratic Republic of the Congo	Bolomba	Proportion	2000	18.4	30.4	9.5
Democratic Republic of the Congo	Bolomba	Proportion	2017	41.7	64.6	22.5
Democratic Republic of the Congo	Bolomba	Count	2000	0	0	0
Democratic Republic of the Congo	Bolomba	Count	2017	0	1	0
Democratic Republic of the Congo	Kipushi (ville)	Proportion	2000	19.6	39.0	7.4
Democratic Republic of the Congo	Kipushi (ville)	Proportion	2017	32.1	57.1	12.8
Democratic Republic of the Congo	Kipushi (ville)	Count	2000	0	0	0
Democratic Republic of the Congo	Kipushi (ville)	Count	2017	0	1	0
Democratic Republic of the Congo	Kamina (ville)	Proportion	2000	18.4	35.8	7.6
Democratic Republic of the Congo	Kamina (ville)	Proportion	2017	37.7	65.2	18.3
Democratic Republic of the Congo	Kamina (ville)	Count	2000	0	0	0
Democratic Republic of the Congo	Kamina (ville)	Count	2017	0	1	0
Democratic Republic of the Congo	Faradje	Proportion	2000	20.8	36.5	9.8
Democratic Republic of the Congo	Faradje	Proportion	2017	32.4	52.8	14.7
Democratic Republic of the Congo	Faradje	Count	2000	0	0	0
Democratic Republic of the Congo	Faradje	Count	2017	0	1	0
Democratic Republic of the Congo	Bunia	Proportion	2000	29.2	50.0	15.5
Democratic Republic of the Congo	Bunia	Proportion	2017	42.9	67.4	22.5
Democratic Republic of the Congo	Bunia	Count	2000	0	0	0
Democratic Republic of the Congo	Bunia	Count	2017	0	1	0
Democratic Republic of the Congo	Kananga	Proportion	2000	17.9	33.3	7.8
Democratic Republic of the Congo	Kananga	Proportion	2017	41.3	63.8	20.4

Table 4: ORS by Unit *(continued)*

Country	Unit	Metric	Year	Mean	Upper	Lower
Democratic Republic of the Congo	Kananga	Count	2000	0	0	0
Democratic Republic of the Congo	Kananga	Count	2017	0	1	0
Democratic Republic of the Congo	Lupatapata	Proportion	2000	16.4	29.8	7.3
Democratic Republic of the Congo	Lupatapata	Proportion	2017	37.0	57.5	19.1
Democratic Republic of the Congo	Lupatapata	Count	2000	0	0	0
Democratic Republic of the Congo	Lupatapata	Count	2017	0	1	0
Democratic Republic of the Congo	Kamonia	Proportion	2000	17.7	31.9	8.4
Democratic Republic of the Congo	Kamonia	Proportion	2017	35.9	56.5	18.9
Democratic Republic of the Congo	Kamonia	Count	2000	0	0	0
Democratic Republic of the Congo	Kamonia	Count	2017	0	1	0
Democratic Republic of the Congo	Kasangulu	Proportion	2000	22.9	37.2	11.7
Democratic Republic of the Congo	Kasangulu	Proportion	2017	43.4	68.2	21.4
Democratic Republic of the Congo	Kasangulu	Count	2000	0	0	0
Democratic Republic of the Congo	Kasangulu	Count	2017	0	1	0
Democratic Republic of the Congo	Kasongo-Lunda	Proportion	2000	17.1	29.8	8.1
Democratic Republic of the Congo	Kasongo-Lunda	Proportion	2017	33.7	53.7	15.8
Democratic Republic of the Congo	Kasongo-Lunda	Count	2000	0	0	0
Democratic Republic of the Congo	Kasongo-Lunda	Count	2017	0	1	0
Democratic Republic of the Congo	Bulungu (ville)	Proportion	2000	18.6	39.6	6.9
Democratic Republic of the Congo	Bulungu (ville)	Proportion	2017	42.8	68.9	19.9
Democratic Republic of the Congo	Bulungu (ville)	Count	2000	0	0	0
Democratic Republic of the Congo	Bulungu (ville)	Count	2017	0	1	0
Democratic Republic of the Congo	Lubao	Proportion	2000	15.9	28.1	7.1
Democratic Republic of the Congo	Lubao	Proportion	2017	36.7	57.1	20.4
Democratic Republic of the Congo	Lubao	Count	2000	0	0	0
Democratic Republic of the Congo	Lubao	Count	2017	0	1	0

Table 4: ORS by Unit *(continued)*

Country	Unit	Metric	Year	Mean	Upper	Lower
Democratic Republic of the Congo	Kolwezi	Proportion	2000	19.2	36.5	8.1
Democratic Republic of the Congo	Kolwezi	Proportion	2017	32.8	55.6	13.3
Democratic Republic of the Congo	Kolwezi	Count	2000	0	0	0
Democratic Republic of the Congo	Kolwezi	Count	2017	0	1	0
Democratic Republic of the Congo	Inongo (ville)	Proportion	2000	15.6	27.1	6.0
Democratic Republic of the Congo	Inongo (ville)	Proportion	2017	35.0	58.9	15.5
Democratic Republic of the Congo	Inongo (ville)	Count	2000	0	0	0
Democratic Republic of the Congo	Inongo (ville)	Count	2017	0	1	0
Democratic Republic of the Congo	Kasongo	Proportion	2000	18.5	33.7	9.1
Democratic Republic of the Congo	Kasongo	Proportion	2017	41.9	62.4	23.7
Democratic Republic of the Congo	Kasongo	Count	2000	0	0	0
Democratic Republic of the Congo	Kasongo	Count	2017	0	1	0
Democratic Republic of the Congo	Lisala	Proportion	2000	20.1	34.9	9.5
Democratic Republic of the Congo	Lisala	Proportion	2017	36.2	58.4	19.2
Democratic Republic of the Congo	Lisala	Count	2000	0	0	0
Democratic Republic of the Congo	Lisala	Count	2017	0	1	0
Democratic Republic of the Congo	Lubero	Proportion	2000	23.5	39.2	12.8
Democratic Republic of the Congo	Lubero	Proportion	2017	39.1	58.2	21.5
Democratic Republic of the Congo	Lubero	Count	2000	0	0	0
Democratic Republic of the Congo	Lubero	Count	2017	0	1	0
Democratic Republic of the Congo	Mobayi-Mbongo	Proportion	2000	18.8	33.0	8.8
Democratic Republic of the Congo	Mobayi-Mbongo	Proportion	2017	29.5	50.9	13.7
Democratic Republic of the Congo	Mobayi-Mbongo	Count	2000	0	0	0
Democratic Republic of the Congo	Mobayi-Mbongo	Count	2017	0	1	0
Democratic Republic of the Congo	Lodja	Proportion	2000	16.1	30.2	6.5
Democratic Republic of the Congo	Lodja	Proportion	2017	32.6	55.4	14.5

Table 4: ORS by Unit *(continued)*

Country	Unit	Metric	Year	Mean	Upper	Lower
Democratic Republic of the Congo	Lodja	Count	2000	0	0	0
Democratic Republic of the Congo	Lodja	Count	2017	0	1	0
Democratic Republic of the Congo	Idjwi	Proportion	2000	16.5	30.0	7.9
Democratic Republic of the Congo	Idjwi	Proportion	2017	31.0	50.1	15.9
Democratic Republic of the Congo	Idjwi	Count	2000	0	0	0
Democratic Republic of the Congo	Idjwi	Count	2017	0	1	0
Democratic Republic of the Congo	Kungu	Proportion	2000	21.3	36.5	9.2
Democratic Republic of the Congo	Kungu	Proportion	2017	37.4	60.3	18.6
Democratic Republic of the Congo	Kungu	Count	2000	0	0	0
Democratic Republic of the Congo	Kungu	Count	2017	0	1	0
Democratic Republic of the Congo	Kaoze	Proportion	2000	21.4	42.8	6.8
Democratic Republic of the Congo	Kaoze	Proportion	2017	34.8	63.6	14.7
Democratic Republic of the Congo	Kaoze	Count	2000	0	0	0
Democratic Republic of the Congo	Kaoze	Count	2017	0	1	0
Democratic Republic of the Congo	Basoko (ville)	Proportion	2000	20.9	41.5	7.7
Democratic Republic of the Congo	Basoko (ville)	Proportion	2017	31.5	52.7	14.4
Democratic Republic of the Congo	Basoko (ville)	Count	2000	0	0	0
Democratic Republic of the Congo	Basoko (ville)	Count	2017	0	1	0
Democratic Republic of the Congo	Bokungu	Proportion	2000	16.5	28.7	7.6
Democratic Republic of the Congo	Bokungu	Proportion	2017	35.0	57.0	17.8
Democratic Republic of the Congo	Bokungu	Count	2000	0	0	0
Democratic Republic of the Congo	Bokungu	Count	2017	0	1	0
Democratic Republic of the Congo	Bondo	Proportion	2000	19.7	33.8	8.3
Democratic Republic of the Congo	Bondo	Proportion	2017	28.8	48.1	14.9
Democratic Republic of the Congo	Bondo	Count	2000	0	0	0
Democratic Republic of the Congo	Bondo	Count	2017	0	0	0

Table 4: ORS by Unit *(continued)*

Country	Unit	Metric	Year	Mean	Upper	Lower
Democratic Republic of the Congo	Bomongo	Proportion	2000	24.4	38.9	11.2
Democratic Republic of the Congo	Bomongo	Proportion	2017	41.3	62.4	21.6
Democratic Republic of the Congo	Bomongo	Count	2000	0	0	0
Democratic Republic of the Congo	Bomongo	Count	2017	0	1	0
Democratic Republic of the Congo	Likasi	Proportion	2000	20.8	35.7	8.4
Democratic Republic of the Congo	Likasi	Proportion	2017	34.0	59.4	15.8
Democratic Republic of the Congo	Likasi	Count	2000	0	0	0
Democratic Republic of the Congo	Likasi	Count	2017	0	1	0
Democratic Republic of the Congo	Kaniama	Proportion	2000	16.7	28.7	7.7
Democratic Republic of the Congo	Kaniama	Proportion	2017	37.7	57.7	19.6
Democratic Republic of the Congo	Kaniama	Count	2000	0	0	0
Democratic Republic of the Congo	Kaniama	Count	2017	0	1	0
Democratic Republic of the Congo	Isiro	Proportion	2000	21.4	40.9	7.5
Democratic Republic of the Congo	Isiro	Proportion	2017	38.2	62.6	16.6
Democratic Republic of the Congo	Isiro	Count	2000	0	0	0
Democratic Republic of the Congo	Isiro	Count	2017	0	1	0
Democratic Republic of the Congo	Djugu	Proportion	2000	22.3	37.8	10.5
Democratic Republic of the Congo	Djugu	Proportion	2017	33.1	54.5	16.5
Democratic Republic of the Congo	Djugu	Count	2000	0	0	0
Democratic Republic of the Congo	Djugu	Count	2017	0	1	0
Democratic Republic of the Congo	Kazumba	Proportion	2000	16.4	30.5	7.9
Democratic Republic of the Congo	Kazumba	Proportion	2017	34.8	54.2	17.5
Democratic Republic of the Congo	Kazumba	Count	2000	0	0	0
Democratic Republic of the Congo	Kazumba	Count	2017	0	1	0
Democratic Republic of the Congo	Mbuji-Mayi	Proportion	2000	17.1	31.4	7.3
Democratic Republic of the Congo	Mbuji-Mayi	Proportion	2017	37.9	60.3	18.8

Table 4: ORS by Unit *(continued)*

Country	Unit	Metric	Year	Mean	Upper	Lower
Democratic Republic of the Congo	Mbuji-Mayi	Count	2000	0	0	0
Democratic Republic of the Congo	Mbuji-Mayi	Count	2017	0	1	0
Democratic Republic of the Congo	Luebo	Proportion	2000	17.4	32.2	7.8
Democratic Republic of the Congo	Luebo	Proportion	2017	37.4	58.7	19.4
Democratic Republic of the Congo	Luebo	Count	2000	0	0	0
Democratic Republic of the Congo	Luebo	Count	2017	0	1	0
Democratic Republic of the Congo	Kasangulu (ville)	Proportion	2000	25.8	46.4	11.9
Democratic Republic of the Congo	Kasangulu (ville)	Proportion	2017	42.8	70.3	18.1
Democratic Republic of the Congo	Kasangulu (ville)	Count	2000	0	0	0
Democratic Republic of the Congo	Kasangulu (ville)	Count	2017	0	1	0
Democratic Republic of the Congo	Kasongo-Lunda (ville)	Proportion	2000	23.8	43.8	9.7
Democratic Republic of the Congo	Kasongo-Lunda (ville)	Proportion	2017	39.3	68.7	13.8
Democratic Republic of the Congo	Kasongo-Lunda (ville)	Count	2000	0	0	0
Democratic Republic of the Congo	Kasongo-Lunda (ville)	Count	2017	0	1	0
Democratic Republic of the Congo	Dibaya-Lubwe	Proportion	2000	19.2	40.5	6.9
Democratic Republic of the Congo	Dibaya-Lubwe	Proportion	2017	42.5	70.7	19.2
Democratic Republic of the Congo	Dibaya-Lubwe	Count	2000	0	0	0
Democratic Republic of the Congo	Dibaya-Lubwe	Count	2017	0	1	0
Democratic Republic of the Congo	Lubao (ville)	Proportion	2000	18.8	36.8	7.2
Democratic Republic of the Congo	Lubao (ville)	Proportion	2017	39.6	63.1	21.3
Democratic Republic of the Congo	Lubao (ville)	Count	2000	0	0	0
Democratic Republic of the Congo	Lubao (ville)	Count	2017	0	1	0
Democratic Republic of the Congo	Lubudi	Proportion	2000	16.3	27.8	7.6
Democratic Republic of the Congo	Lubudi	Proportion	2017	35.1	58.0	18.1
Democratic Republic of the Congo	Lubudi	Count	2000	0	0	0
Democratic Republic of the Congo	Lubudi	Count	2017	0	1	0

Table 4: ORS by Unit *(continued)*

Country	Unit	Metric	Year	Mean	Upper	Lower
Democratic Republic of the Congo	Kiri	Proportion	2000	16.0	27.1	7.2
Democratic Republic of the Congo	Kiri	Proportion	2017	38.2	58.9	20.9
Democratic Republic of the Congo	Kiri	Count	2000	0	0	0
Democratic Republic of the Congo	Kiri	Count	2017	0	1	0
Democratic Republic of the Congo	Kasongo (ville)	Proportion	2000	23.0	39.8	10.7
Democratic Republic of the Congo	Kasongo (ville)	Proportion	2017	51.4	75.1	29.2
Democratic Republic of the Congo	Kasongo (ville)	Count	2000	0	0	0
Democratic Republic of the Congo	Kasongo (ville)	Count	2017	1	1	0
Democratic Republic of the Congo	Lisala (ville)	Proportion	2000	21.5	41.1	9.3
Democratic Republic of the Congo	Lisala (ville)	Proportion	2017	38.3	64.5	16.8
Democratic Republic of the Congo	Lisala (ville)	Count	2000	0	0	0
Democratic Republic of the Congo	Lisala (ville)	Count	2017	0	1	0
Democratic Republic of the Congo	Masisi	Proportion	2000	18.4	30.9	9.3
Democratic Republic of the Congo	Masisi	Proportion	2017	36.3	57.2	19.3
Democratic Republic of the Congo	Masisi	Count	2000	0	0	0
Democratic Republic of the Congo	Masisi	Count	2017	0	1	0
Democratic Republic of the Congo	Mobayi-Mbongo (ville)	Proportion	2000	19.4	42.0	6.0
Democratic Republic of the Congo	Mobayi-Mbongo (ville)	Proportion	2017	29.1	58.3	10.8
Democratic Republic of the Congo	Mobayi-Mbongo (ville)	Count	2000	0	0	0
Democratic Republic of the Congo	Mobayi-Mbongo (ville)	Count	2017	0	1	0
Democratic Republic of the Congo	Lodja (ville)	Proportion	2000	NA	NA	NA
Democratic Republic of the Congo	Lodja (ville)	Proportion	2017	NA	NA	NA
Democratic Republic of the Congo	Lodja (ville)	Count	2000	NA	NA	NA
Democratic Republic of the Congo	Lodja (ville)	Count	2017	NA	NA	NA
Democratic Republic of the Congo	Kabare	Proportion	2000	19.1	32.0	9.1
Democratic Republic of the Congo	Kabare	Proportion	2017	37.3	56.8	19.0

Table 4: ORS by Unit *(continued)*

Country	Unit	Metric	Year	Mean	Upper	Lower
Democratic Republic of the Congo	Kabare	Count	2000	0	0	0
Democratic Republic of the Congo	Kabare	Count	2017	0	1	0
Democratic Republic of the Congo	Libenge	Proportion	2000	23.3	38.8	10.1
Democratic Republic of the Congo	Libenge	Proportion	2017	34.8	54.6	16.6
Democratic Republic of the Congo	Libenge	Count	2000	0	0	0
Democratic Republic of the Congo	Libenge	Count	2017	0	1	0
Democratic Republic of the Congo	Kongolo	Proportion	2000	15.8	26.4	7.4
Democratic Republic of the Congo	Kongolo	Proportion	2017	33.5	55.7	16.6
Democratic Republic of the Congo	Kongolo	Count	2000	0	0	0
Democratic Republic of the Congo	Kongolo	Count	2017	0	1	0
Democratic Republic of the Congo	Isangi	Proportion	2000	20.8	37.7	10.1
Democratic Republic of the Congo	Isangi	Proportion	2017	33.6	52.9	18.3
Democratic Republic of the Congo	Isangi	Count	2000	0	0	0
Democratic Republic of the Congo	Isangi	Count	2017	0	1	0
Democratic Republic of the Congo	Djolu	Proportion	2000	15.9	27.5	7.4
Democratic Republic of the Congo	Djolu	Proportion	2017	28.1	48.7	13.9
Democratic Republic of the Congo	Djolu	Count	2000	0	0	0
Democratic Republic of the Congo	Djolu	Count	2017	0	0	0
Democratic Republic of the Congo	Bondo (ville)	Proportion	2000	20.9	39.3	7.3
Democratic Republic of the Congo	Bondo (ville)	Proportion	2017	35.3	66.1	14.8
Democratic Republic of the Congo	Bondo (ville)	Count	2000	0	0	0
Democratic Republic of the Congo	Bondo (ville)	Count	2017	0	1	0
Democratic Republic of the Congo	Ingende	Proportion	2000	16.6	28.8	8.2
Democratic Republic of the Congo	Ingende	Proportion	2017	39.9	61.5	21.6
Democratic Republic of the Congo	Ingende	Count	2000	0	0	0
Democratic Republic of the Congo	Ingende	Count	2017	0	1	0

Table 4: ORS by Unit *(continued)*

Country	Unit	Metric	Year	Mean	Upper	Lower
Democratic Republic of the Congo	Lubumbashi	Proportion	2000	22.6	41.6	9.4
Democratic Republic of the Congo	Lubumbashi	Proportion	2017	35.1	59.4	16.4
Democratic Republic of the Congo	Lubumbashi	Count	2000	0	0	0
Democratic Republic of the Congo	Lubumbashi	Count	2017	0	1	0
Democratic Republic of the Congo	Malemba-Nkulu	Proportion	2000	18.7	33.7	9.1
Democratic Republic of the Congo	Malemba-Nkulu	Proportion	2017	35.8	58.7	17.4
Democratic Republic of the Congo	Malemba-Nkulu	Count	2000	0	0	0
Democratic Republic of the Congo	Malemba-Nkulu	Count	2017	0	1	0
Democratic Republic of the Congo	Niangara	Proportion	2000	21.9	42.0	9.8
Democratic Republic of the Congo	Niangara	Proportion	2017	30.3	48.8	15.6
Democratic Republic of the Congo	Niangara	Count	2000	0	0	0
Democratic Republic of the Congo	Niangara	Count	2017	0	0	0
Democratic Republic of the Congo	Ingbokolo	Proportion	2000	18.7	38.2	6.8
Democratic Republic of the Congo	Ingbokolo	Proportion	2017	30.4	54.4	11.5
Democratic Republic of the Congo	Ingbokolo	Count	2000	0	0	0
Democratic Republic of the Congo	Ingbokolo	Count	2017	0	1	0
Democratic Republic of the Congo	Luiza	Proportion	2000	16.6	28.3	8.2
Democratic Republic of the Congo	Luiza	Proportion	2017	33.2	53.2	15.5
Democratic Republic of the Congo	Luiza	Count	2000	0	0	0
Democratic Republic of the Congo	Luiza	Count	2017	0	1	0
Democratic Republic of the Congo	Miabi	Proportion	2000	15.0	28.4	6.3
Democratic Republic of the Congo	Miabi	Proportion	2017	37.7	58.7	18.8
Democratic Republic of the Congo	Miabi	Count	2000	0	0	0
Democratic Republic of the Congo	Miabi	Count	2017	0	1	0
Democratic Republic of the Congo	Luebo (ville)	Proportion	2000	19.9	38.5	6.7
Democratic Republic of the Congo	Luebo (ville)	Proportion	2017	42.6	65.9	17.8

Table 4: ORS by Unit *(continued)*

Country	Unit	Metric	Year	Mean	Upper	Lower
Democratic Republic of the Congo	Luebo (ville)	Count	2000	0	0	0
Democratic Republic of the Congo	Luebo (ville)	Count	2017	0	1	0
Democratic Republic of the Congo	Kimvula	Proportion	2000	20.3	34.0	9.8
Democratic Republic of the Congo	Kimvula	Proportion	2017	33.5	57.3	15.9
Democratic Republic of the Congo	Kimvula	Count	2000	0	0	0
Democratic Republic of the Congo	Kimvula	Count	2017	0	1	0
Democratic Republic of the Congo	Kenge	Proportion	2000	16.6	29.0	8.1
Democratic Republic of the Congo	Kenge	Proportion	2017	41.7	66.6	22.0
Democratic Republic of the Congo	Kenge	Count	2000	0	0	0
Democratic Republic of the Congo	Kenge	Count	2017	0	1	0
Democratic Republic of the Congo	Gungu	Proportion	2000	15.7	29.6	7.3
Democratic Republic of the Congo	Gungu	Proportion	2017	43.4	63.0	23.6
Democratic Republic of the Congo	Gungu	Count	2000	0	0	0
Democratic Republic of the Congo	Gungu	Count	2017	0	1	0
Democratic Republic of the Congo	Luilu	Proportion	2000	16.8	29.2	7.7
Democratic Republic of the Congo	Luilu	Proportion	2017	42.9	65.0	22.5
Democratic Republic of the Congo	Luilu	Count	2000	0	0	0
Democratic Republic of the Congo	Luilu	Count	2017	0	1	0
Democratic Republic of the Congo	Mutshatsha	Proportion	2000	18.2	31.3	8.5
Democratic Republic of the Congo	Mutshatsha	Proportion	2017	31.5	53.5	13.9
Democratic Republic of the Congo	Mutshatsha	Count	2000	0	0	0
Democratic Republic of the Congo	Mutshatsha	Count	2017	0	1	0
Democratic Republic of the Congo	Kutu	Proportion	2000	16.0	28.7	7.6
Democratic Republic of the Congo	Kutu	Proportion	2017	37.3	60.0	19.8
Democratic Republic of the Congo	Kutu	Count	2000	0	0	0
Democratic Republic of the Congo	Kutu	Count	2017	0	1	0

Table 4: ORS by Unit *(continued)*

Country	Unit	Metric	Year	Mean	Upper	Lower
Democratic Republic of the Congo	Kibombo	Proportion	2000	16.7	30.1	7.7
Democratic Republic of the Congo	Kibombo	Proportion	2017	38.9	56.3	21.7
Democratic Republic of the Congo	Kibombo	Count	2000	0	0	0
Democratic Republic of the Congo	Kibombo	Count	2017	0	1	0
Democratic Republic of the Congo	Nyiragongo	Proportion	2000	20.8	37.4	9.9
Democratic Republic of the Congo	Nyiragongo	Proportion	2017	44.2	66.3	23.9
Democratic Republic of the Congo	Nyiragongo	Count	2000	0	0	0
Democratic Republic of the Congo	Nyiragongo	Count	2017	0	1	0
Democratic Republic of the Congo	Yakoma	Proportion	2000	20.5	37.2	9.5
Democratic Republic of the Congo	Yakoma	Proportion	2017	31.5	56.6	15.1
Democratic Republic of the Congo	Yakoma	Count	2000	0	0	0
Democratic Republic of the Congo	Yakoma	Count	2017	0	1	0
Democratic Republic of the Congo	Lomela	Proportion	2000	18.1	30.9	8.0
Democratic Republic of the Congo	Lomela	Proportion	2017	41.7	63.4	22.8
Democratic Republic of the Congo	Lomela	Count	2000	0	0	0
Democratic Republic of the Congo	Lomela	Count	2017	0	1	0
Democratic Republic of the Congo	Kalehe	Proportion	2000	17.0	30.8	8.2
Democratic Republic of the Congo	Kalehe	Proportion	2017	31.5	49.1	15.2
Democratic Republic of the Congo	Kalehe	Count	2000	0	0	0
Democratic Republic of the Congo	Kalehe	Count	2017	0	0	0
Democratic Republic of the Congo	Zongo	Proportion	2000	25.5	47.0	10.3
Democratic Republic of the Congo	Zongo	Proportion	2017	37.8	61.1	15.6
Democratic Republic of the Congo	Zongo	Count	2000	0	0	0
Democratic Republic of the Congo	Zongo	Count	2017	0	1	0
Democratic Republic of the Congo	Kongolo (ville)	Proportion	2000	17.3	32.0	7.6
Democratic Republic of the Congo	Kongolo (ville)	Proportion	2017	40.2	65.5	19.7

Table 4: ORS by Unit *(continued)*

Country	Unit	Metric	Year	Mean	Upper	Lower
Democratic Republic of the Congo	Kongolo (ville)	Count	2000	0	0	0
Democratic Republic of the Congo	Kongolo (ville)	Count	2017	0	1	0
Democratic Republic of the Congo	Isangi (ville)	Proportion	2000	22.5	44.4	8.7
Democratic Republic of the Congo	Isangi (ville)	Proportion	2017	35.9	61.6	14.9
Democratic Republic of the Congo	Isangi (ville)	Count	2000	0	0	0
Democratic Republic of the Congo	Isangi (ville)	Count	2017	0	1	0
Democratic Republic of the Congo	Ikela	Proportion	2000	18.2	30.8	9.6
Democratic Republic of the Congo	Ikela	Proportion	2017	33.6	53.8	18.1
Democratic Republic of the Congo	Ikela	Count	2000	0	0	0
Democratic Republic of the Congo	Ikela	Count	2017	0	1	0
Democratic Republic of the Congo	Buta	Proportion	2000	22.9	39.4	11.1
Democratic Republic of the Congo	Buta	Proportion	2017	34.8	55.1	16.8
Democratic Republic of the Congo	Buta	Count	2000	0	0	0
Democratic Republic of the Congo	Buta	Count	2017	0	1	0
Democratic Republic of the Congo	Lukolela	Proportion	2000	21.9	32.6	9.7
Democratic Republic of the Congo	Lukolela	Proportion	2017	41.7	65.1	22.1
Democratic Republic of the Congo	Lukolela	Count	2000	0	0	0
Democratic Republic of the Congo	Lukolela	Count	2017	0	1	0
Democratic Republic of the Congo	Mitwaba	Proportion	2000	15.9	28.0	8.1
Democratic Republic of the Congo	Mitwaba	Proportion	2017	31.6	50.9	14.6
Democratic Republic of the Congo	Mitwaba	Count	2000	0	0	0
Democratic Republic of the Congo	Mitwaba	Count	2017	0	1	0
Democratic Republic of the Congo	Rungu	Proportion	2000	18.9	36.4	8.0
Democratic Republic of the Congo	Rungu	Proportion	2017	29.7	46.2	15.2
Democratic Republic of the Congo	Rungu	Count	2000	0	0	0
Democratic Republic of the Congo	Rungu	Count	2017	0	0	0

Table 4: ORS by Unit *(continued)*

Country	Unit	Metric	Year	Mean	Upper	Lower
Democratic Republic of the Congo	Irumu	Proportion	2000	23.7	39.1	12.1
Democratic Republic of the Congo	Irumu	Proportion	2017	34.7	55.3	17.1
Democratic Republic of the Congo	Irumu	Count	2000	0	0	0
Democratic Republic of the Congo	Irumu	Count	2017	0	1	0
Democratic Republic of the Congo	Tshimbulu	Proportion	2000	15.9	31.2	6.0
Democratic Republic of the Congo	Tshimbulu	Proportion	2017	40.1	67.9	15.3
Democratic Republic of the Congo	Tshimbulu	Count	2000	0	0	0
Democratic Republic of the Congo	Tshimbulu	Count	2017	0	1	0
Democratic Republic of the Congo	Miabi (ville)	Proportion	2000	14.8	28.1	5.9
Democratic Republic of the Congo	Miabi (ville)	Proportion	2017	37.6	61.8	17.7
Democratic Republic of the Congo	Miabi (ville)	Count	2000	0	0	0
Democratic Republic of the Congo	Miabi (ville)	Count	2017	0	1	0
Democratic Republic of the Congo	Mweka	Proportion	2000	17.7	28.7	8.5
Democratic Republic of the Congo	Mweka	Proportion	2017	40.4	60.1	20.2
Democratic Republic of the Congo	Mweka	Count	2000	0	0	0
Democratic Republic of the Congo	Mweka	Count	2017	0	1	0
Democratic Republic of the Congo	Lukula	Proportion	2000	24.2	39.3	12.0
Democratic Republic of the Congo	Lukula	Proportion	2017	45.2	67.5	24.8
Democratic Republic of the Congo	Lukula	Count	2000	0	0	0
Democratic Republic of the Congo	Lukula	Count	2017	0	1	0
Democratic Republic of the Congo	Kenge (ville)	Proportion	2000	19.5	37.4	7.9
Democratic Republic of the Congo	Kenge (ville)	Proportion	2017	48.7	73.7	27.0
Democratic Republic of the Congo	Kenge (ville)	Count	2000	0	0	0
Democratic Republic of the Congo	Kenge (ville)	Count	2017	0	1	0
Democratic Republic of the Congo	Gungu (ville)	Proportion	2000	17.5	35.4	6.5
Democratic Republic of the Congo	Gungu (ville)	Proportion	2017	44.1	68.7	19.1

Table 4: ORS by Unit *(continued)*

Country	Unit	Metric	Year	Mean	Upper	Lower
Democratic Republic of the Congo	Gungu (ville)	Count	2000	0	0	0
Democratic Republic of the Congo	Gungu (ville)	Count	2017	0	1	0
Democratic Republic of the Congo	Mwene-Ditu	Proportion	2000	17.0	33.5	7.5
Democratic Republic of the Congo	Mwene-Ditu	Proportion	2017	43.8	67.0	21.1
Democratic Republic of the Congo	Mwene-Ditu	Count	2000	0	0	0
Democratic Republic of the Congo	Mwene-Ditu	Count	2017	0	1	0
Democratic Republic of the Congo	Sandoa	Proportion	2000	17.3	33.3	8.2
Democratic Republic of the Congo	Sandoa	Proportion	2017	27.7	45.2	11.1
Democratic Republic of the Congo	Sandoa	Count	2000	0	0	0
Democratic Republic of the Congo	Sandoa	Count	2017	0	0	0
Democratic Republic of the Congo	Kwamouth	Proportion	2000	17.7	32.2	9.2
Democratic Republic of the Congo	Kwamouth	Proportion	2017	39.0	60.2	21.5
Democratic Republic of the Congo	Kwamouth	Count	2000	0	0	0
Democratic Republic of the Congo	Kwamouth	Count	2017	0	1	0
Democratic Republic of the Congo	Kindu	Proportion	2000	22.7	37.4	10.5
Democratic Republic of the Congo	Kindu	Proportion	2017	43.8	64.6	25.6
Democratic Republic of the Congo	Kindu	Count	2000	0	0	0
Democratic Republic of the Congo	Kindu	Count	2017	0	1	0
Democratic Republic of the Congo	Oicha	Proportion	2000	21.1	36.2	11.0
Democratic Republic of the Congo	Oicha	Proportion	2017	33.1	51.3	16.7
Democratic Republic of the Congo	Oicha	Count	2000	0	0	0
Democratic Republic of the Congo	Oicha	Count	2017	0	1	0
Democratic Republic of the Congo	Lubefu	Proportion	2000	14.8	25.8	6.6
Democratic Republic of the Congo	Lubefu	Proportion	2017	36.4	58.2	19.4
Democratic Republic of the Congo	Lubefu	Count	2000	0	0	0
Democratic Republic of the Congo	Lubefu	Count	2017	0	1	0

Table 4: ORS by Unit *(continued)*

Country	Unit	Metric	Year	Mean	Upper	Lower
Democratic Republic of the Congo	Kamituga	Proportion	2000	15.4	30.4	5.0
Democratic Republic of the Congo	Kamituga	Proportion	2017	36.8	65.6	11.7
Democratic Republic of the Congo	Kamituga	Count	2000	0	0	0
Democratic Republic of the Congo	Kamituga	Count	2017	0	1	0
Democratic Republic of the Congo	Manono	Proportion	2000	17.6	30.9	8.9
Democratic Republic of the Congo	Manono	Proportion	2017	32.5	52.5	17.4
Democratic Republic of the Congo	Manono	Count	2000	0	0	0
Democratic Republic of the Congo	Manono	Count	2017	0	1	0
Democratic Republic of the Congo	Kisangani	Proportion	2000	23.1	42.4	11.0
Democratic Republic of the Congo	Kisangani	Proportion	2017	38.1	58.8	19.6
Democratic Republic of the Congo	Kisangani	Count	2000	0	0	0
Democratic Republic of the Congo	Kisangani	Count	2017	0	1	0
Democratic Republic of the Congo	Monkoto	Proportion	2000	13.9	25.7	6.6
Democratic Republic of the Congo	Monkoto	Proportion	2017	32.0	51.2	16.4
Democratic Republic of the Congo	Monkoto	Count	2000	0	0	0
Democratic Republic of the Congo	Monkoto	Count	2017	0	1	0
Democratic Republic of the Congo	Buta (ville)	Proportion	2000	28.9	51.6	12.0
Democratic Republic of the Congo	Buta (ville)	Proportion	2017	47.0	72.2	26.3
Democratic Republic of the Congo	Buta (ville)	Count	2000	0	1	0
Democratic Republic of the Congo	Buta (ville)	Count	2017	0	1	0
Democratic Republic of the Congo	Makanza	Proportion	2000	20.7	37.6	10.0
Democratic Republic of the Congo	Makanza	Proportion	2017	39.8	62.8	19.6
Democratic Republic of the Congo	Makanza	Count	2000	0	0	0
Democratic Republic of the Congo	Makanza	Count	2017	0	1	0
Democratic Republic of the Congo	Pweto	Proportion	2000	19.1	31.5	10.5
Democratic Republic of the Congo	Pweto	Proportion	2017	27.7	48.2	13.5

Table 4: ORS by Unit *(continued)*

Country	Unit	Metric	Year	Mean	Upper	Lower
Democratic Republic of the Congo	Pweto	Count	2000	0	0	0
Democratic Republic of the Congo	Pweto	Count	2017	0	0	0
Democratic Republic of the Congo	Wamba	Proportion	2000	19.9	37.8	8.7
Democratic Republic of the Congo	Wamba	Proportion	2017	30.9	50.1	15.6
Democratic Republic of the Congo	Wamba	Count	2000	0	0	0
Democratic Republic of the Congo	Wamba	Count	2017	0	1	0
Democratic Republic of the Congo	Mahagi	Proportion	2000	23.9	42.4	11.3
Democratic Republic of the Congo	Mahagi	Proportion	2017	35.9	54.8	19.2
Democratic Republic of the Congo	Mahagi	Count	2000	0	0	0
Democratic Republic of the Congo	Mahagi	Count	2017	0	1	0
Democratic Republic of the Congo	Tshilenge	Proportion	2000	15.5	28.5	6.7
Democratic Republic of the Congo	Tshilenge	Proportion	2017	37.5	59.5	18.9
Democratic Republic of the Congo	Tshilenge	Count	2000	0	0	0
Democratic Republic of the Congo	Tshilenge	Count	2017	0	1	0
Democratic Republic of the Congo	Tshikapa	Proportion	2000	18.5	40.2	6.8
Democratic Republic of the Congo	Tshikapa	Proportion	2017	38.7	58.8	18.7
Democratic Republic of the Congo	Tshikapa	Count	2000	0	0	0
Democratic Republic of the Congo	Tshikapa	Count	2017	0	1	0
Democratic Republic of the Congo	Lukula (ville)	Proportion	2000	23.4	48.1	9.1
Democratic Republic of the Congo	Lukula (ville)	Proportion	2017	48.1	76.9	22.8
Democratic Republic of the Congo	Lukula (ville)	Count	2000	0	0	0
Democratic Republic of the Congo	Lukula (ville)	Count	2017	0	1	0
Democratic Republic of the Congo	Popokabaka	Proportion	2000	18.9	32.8	8.7
Democratic Republic of the Congo	Popokabaka	Proportion	2017	31.0	54.1	13.7
Democratic Republic of the Congo	Popokabaka	Count	2000	0	0	0
Democratic Republic of the Congo	Popokabaka	Count	2017	0	1	0

Table 4: ORS by Unit *(continued)*

Country	Unit	Metric	Year	Mean	Upper	Lower
Democratic Republic of the Congo	Idiofa	Proportion	2000	17.2	28.4	7.6
Democratic Republic of the Congo	Idiofa	Proportion	2017	47.5	72.4	26.6
Democratic Republic of the Congo	Idiofa	Count	2000	0	0	0
Democratic Republic of the Congo	Idiofa	Count	2017	0	1	0
Democratic Republic of the Congo	Ngandajika	Proportion	2000	15.9	28.1	6.9
Democratic Republic of the Congo	Ngandajika	Proportion	2017	40.6	63.5	21.7
Democratic Republic of the Congo	Ngandajika	Count	2000	0	0	0
Democratic Republic of the Congo	Ngandajika	Count	2017	0	1	0
Democratic Republic of the Congo	Mushie	Proportion	2000	16.1	28.9	7.3
Democratic Republic of the Congo	Mushie	Proportion	2017	37.6	58.9	20.0
Democratic Republic of the Congo	Mushie	Count	2000	0	0	0
Democratic Republic of the Congo	Mushie	Count	2017	0	1	0
Democratic Republic of the Congo	Lubutu	Proportion	2000	18.7	33.6	9.2
Democratic Republic of the Congo	Lubutu	Proportion	2017	39.1	59.7	20.2
Democratic Republic of the Congo	Lubutu	Count	2000	0	0	0
Democratic Republic of the Congo	Lubutu	Count	2017	0	1	0
Democratic Republic of the Congo	Oïcha (ville)	Proportion	2000	19.1	35.5	8.5
Democratic Republic of the Congo	Oïcha (ville)	Proportion	2017	32.2	54.2	13.5
Democratic Republic of the Congo	Oïcha (ville)	Count	2000	0	0	0
Democratic Republic of the Congo	Oïcha (ville)	Count	2017	0	1	0
Democratic Republic of the Congo	Lusambo	Proportion	2000	15.5	27.9	6.8
Democratic Republic of the Congo	Lusambo	Proportion	2017	35.0	54.5	17.2
Democratic Republic of the Congo	Lusambo	Count	2000	0	0	0
Democratic Republic of the Congo	Lusambo	Count	2017	0	1	0
Democratic Republic of the Congo	Mwenga	Proportion	2000	15.7	28.8	6.6
Democratic Republic of the Congo	Mwenga	Proportion	2017	33.4	54.5	15.9

Table 4: ORS by Unit *(continued)*

Country	Unit	Metric	Year	Mean	Upper	Lower
Democratic Republic of the Congo	Mwenga	Count	2000	0	0	0
Democratic Republic of the Congo	Mwenga	Count	2017	0	1	0
Democratic Republic of the Congo	Manono (ville)	Proportion	2000	16.3	31.3	7.0
Democratic Republic of the Congo	Manono (ville)	Proportion	2017	34.5	58.5	17.2
Democratic Republic of the Congo	Manono (ville)	Count	2000	0	0	0
Democratic Republic of the Congo	Manono (ville)	Count	2017	0	1	0
Democratic Republic of the Congo	Opala	Proportion	2000	18.9	31.6	9.4
Democratic Republic of the Congo	Opala	Proportion	2017	32.1	50.5	17.4
Democratic Republic of the Congo	Opala	Count	2000	0	0	0
Democratic Republic of the Congo	Opala	Count	2017	0	1	0
Democratic Republic of the Congo	Dingila	Proportion	2000	21.9	44.0	6.7
Democratic Republic of the Congo	Dingila	Proportion	2017	34.3	63.2	11.7
Democratic Republic of the Congo	Dingila	Count	2000	0	0	0
Democratic Republic of the Congo	Dingila	Count	2017	0	1	0
Democratic Republic of the Congo	Mbandaka	Proportion	2000	25.7	45.0	12.4
Democratic Republic of the Congo	Mbandaka	Proportion	2017	43.3	67.6	21.6
Democratic Republic of the Congo	Mbandaka	Count	2000	0	0	0
Democratic Republic of the Congo	Mbandaka	Count	2017	0	1	0
Democratic Republic of the Congo	Sakania	Proportion	2000	21.2	38.6	10.3
Democratic Republic of the Congo	Sakania	Proportion	2017	32.7	52.9	15.9
Democratic Republic of the Congo	Sakania	Count	2000	0	0	0
Democratic Republic of the Congo	Sakania	Count	2017	0	1	0
Democratic Republic of the Congo	Wamba (ville)	Proportion	2000	19.9	39.7	7.3
Democratic Republic of the Congo	Wamba (ville)	Proportion	2017	33.2	55.7	14.6
Democratic Republic of the Congo	Wamba (ville)	Count	2000	0	0	0
Democratic Republic of the Congo	Wamba (ville)	Count	2017	0	1	0

Table 4: ORS by Unit *(continued)*

Country	Unit	Metric	Year	Mean	Upper	Lower
Democratic Republic of the Congo	Mahagi (ville)	Proportion	2000	26.5	45.6	11.9
Democratic Republic of the Congo	Mahagi (ville)	Proportion	2017	42.3	64.8	22.5
Democratic Republic of the Congo	Mahagi (ville)	Count	2000	0	0	0
Democratic Republic of the Congo	Mahagi (ville)	Count	2017	0	1	0
Democratic Republic of the Congo	Tshilenge (ville)	Proportion	2000	17.1	33.9	7.1
Democratic Republic of the Congo	Tshilenge (ville)	Proportion	2017	38.9	65.9	17.5
Democratic Republic of the Congo	Tshilenge (ville)	Count	2000	0	0	0
Democratic Republic of the Congo	Tshilenge (ville)	Count	2017	0	1	0
Democratic Republic of the Congo	Luozi	Proportion	2000	22.2	37.1	11.5
Democratic Republic of the Congo	Luozi	Proportion	2017	42.0	63.4	22.9
Democratic Republic of the Congo	Luozi	Count	2000	0	0	0
Democratic Republic of the Congo	Luozi	Count	2017	0	1	0
Democratic Republic of the Congo	Idiofa (ville)	Proportion	2000	18.5	37.0	6.8
Democratic Republic of the Congo	Idiofa (ville)	Proportion	2017	48.9	73.2	23.7
Democratic Republic of the Congo	Idiofa (ville)	Count	2000	0	0	0
Democratic Republic of the Congo	Idiofa (ville)	Count	2017	0	1	0
Democratic Republic of the Congo	Ngandajika (ville)	Proportion	2000	15.5	29.9	6.6
Democratic Republic of the Congo	Ngandajika (ville)	Proportion	2017	38.7	60.7	18.6
Democratic Republic of the Congo	Ngandajika (ville)	Count	2000	0	0	0
Democratic Republic of the Congo	Ngandajika (ville)	Count	2017	0	1	0
Democratic Republic of the Congo	Nioki	Proportion	2000	18.6	33.9	7.8
Democratic Republic of the Congo	Nioki	Proportion	2017	38.5	64.9	17.7
Democratic Republic of the Congo	Nioki	Count	2000	0	0	0
Democratic Republic of the Congo	Nioki	Count	2017	0	1	0
Democratic Republic of the Congo	Lubutu (ville)	Proportion	2000	20.7	39.3	8.3
Democratic Republic of the Congo	Lubutu (ville)	Proportion	2017	47.4	71.8	24.0

Table 4: ORS by Unit *(continued)*

Country	Unit	Metric	Year	Mean	Upper	Lower
Democratic Republic of the Congo	Lubutu (ville)	Count	2000	0	0	0
Democratic Republic of the Congo	Lubutu (ville)	Count	2017	0	1	0
Democratic Republic of the Congo	Rutshuru	Proportion	2000	20.8	35.8	10.9
Democratic Republic of the Congo	Rutshuru	Proportion	2017	36.7	54.9	19.6
Democratic Republic of the Congo	Rutshuru	Count	2000	0	0	0
Democratic Republic of the Congo	Rutshuru	Count	2017	0	1	0
Democratic Republic of the Congo	Lusambo (ville)	Proportion	2000	17.7	34.4	7.0
Democratic Republic of the Congo	Lusambo (ville)	Proportion	2017	38.1	63.0	15.7
Democratic Republic of the Congo	Lusambo (ville)	Count	2000	0	0	0
Democratic Republic of the Congo	Lusambo (ville)	Count	2017	0	1	0
Democratic Republic of the Congo	Shabunda	Proportion	2000	14.1	25.2	7.0
Democratic Republic of the Congo	Shabunda	Proportion	2017	31.0	50.5	16.1
Democratic Republic of the Congo	Shabunda	Count	2000	0	0	0
Democratic Republic of the Congo	Shabunda	Count	2017	0	1	0
Democratic Republic of the Congo	Moba	Proportion	2000	17.2	30.0	8.0
Democratic Republic of the Congo	Moba	Proportion	2017	27.4	45.4	12.8
Democratic Republic of the Congo	Moba	Count	2000	0	0	0
Democratic Republic of the Congo	Moba	Count	2017	0	0	0
Democratic Republic of the Congo	Ubundu	Proportion	2000	19.2	32.7	9.7
Democratic Republic of the Congo	Ubundu	Proportion	2017	33.3	52.0	17.9
Democratic Republic of the Congo	Ubundu	Count	2000	0	0	0
Democratic Republic of the Congo	Ubundu	Count	2017	0	1	0
Democratic Republic of the Congo	Poko	Proportion	2000	20.4	37.0	7.8
Democratic Republic of the Congo	Poko	Proportion	2017	32.7	51.1	16.9
Democratic Republic of the Congo	Poko	Count	2000	0	0	0
Democratic Republic of the Congo	Poko	Count	2017	0	1	0

Table 4: ORS by Unit *(continued)*

Country	Unit	Metric	Year	Mean	Upper	Lower
Democratic Republic of the Congo	Watsa	Proportion	2000	21.5	37.1	10.3
Democratic Republic of the Congo	Watsa	Proportion	2017	35.6	56.4	16.9
Democratic Republic of the Congo	Watsa	Count	2000	0	0	0
Democratic Republic of the Congo	Watsa	Count	2017	0	1	0
Democratic Republic of the Congo	Mambasa	Proportion	2000	20.9	37.2	11.2
Democratic Republic of the Congo	Mambasa	Proportion	2017	32.1	50.8	17.0
Democratic Republic of the Congo	Mambasa	Count	2000	0	0	0
Democratic Republic of the Congo	Mambasa	Count	2017	0	1	0
Democratic Republic of the Congo	Madimba	Proportion	2000	22.7	36.3	11.4
Democratic Republic of the Congo	Madimba	Proportion	2017	37.3	58.2	18.4
Democratic Republic of the Congo	Madimba	Count	2000	0	0	0
Democratic Republic of the Congo	Madimba	Count	2017	0	1	0
Democratic Republic of the Congo	Kikwit	Proportion	2000	16.4	32.3	6.4
Democratic Republic of the Congo	Kikwit	Proportion	2017	40.6	65.9	17.9
Democratic Republic of the Congo	Kikwit	Count	2000	0	0	0
Democratic Republic of the Congo	Kikwit	Count	2017	0	1	0
Democratic Republic of the Congo	Oshwe	Proportion	2000	15.5	27.4	7.4
Democratic Republic of the Congo	Oshwe	Proportion	2017	37.3	59.6	20.2
Democratic Republic of the Congo	Oshwe	Count	2000	0	0	0
Democratic Republic of the Congo	Oshwe	Count	2017	0	1	0
Democratic Republic of the Congo	Namoya	Proportion	2000	16.5	34.3	6.7
Democratic Republic of the Congo	Namoya	Proportion	2017	46.4	71.2	19.0
Democratic Republic of the Congo	Namoya	Count	2000	0	0	0
Democratic Republic of the Congo	Namoya	Count	2017	0	1	0
Democratic Republic of the Congo	Rutshuru (ville)	Proportion	2000	17.4	35.2	7.7
Democratic Republic of the Congo	Rutshuru (ville)	Proportion	2017	33.3	54.9	14.5

Table 4: ORS by Unit *(continued)*

Country	Unit	Metric	Year	Mean	Upper	Lower
Democratic Republic of the Congo	Rutshuru (ville)	Count	2000	0	0	0
Democratic Republic of the Congo	Rutshuru (ville)	Count	2017	0	1	0
Democratic Republic of the Congo	Shabunda (ville)	Proportion	2000	14.4	29.4	5.5
Democratic Republic of the Congo	Shabunda (ville)	Proportion	2017	32.8	57.3	12.8
Democratic Republic of the Congo	Shabunda (ville)	Count	2000	0	0	0
Democratic Republic of the Congo	Shabunda (ville)	Count	2017	0	1	0
Democratic Republic of the Congo	Nyunzu	Proportion	2000	15.4	26.3	7.5
Democratic Republic of the Congo	Nyunzu	Proportion	2017	31.8	54.6	14.5
Democratic Republic of the Congo	Nyunzu	Count	2000	0	0	0
Democratic Republic of the Congo	Nyunzu	Count	2017	0	1	0
Democratic Republic of the Congo	Yahuma	Proportion	2000	18.1	30.3	8.1
Democratic Republic of the Congo	Yahuma	Proportion	2017	26.2	44.5	14.3
Democratic Republic of the Congo	Yahuma	Count	2000	0	0	0
Democratic Republic of the Congo	Yahuma	Count	2017	0	0	0
Democratic Republic of the Congo	Watsa (ville)	Proportion	2000	22.0	40.4	10.0
Democratic Republic of the Congo	Watsa (ville)	Proportion	2017	36.9	61.0	16.6
Democratic Republic of the Congo	Watsa (ville)	Count	2000	0	0	0
Democratic Republic of the Congo	Watsa (ville)	Count	2017	0	1	0
Democratic Republic of the Congo	Mongwalu	Proportion	2000	20.6	44.4	6.9
Democratic Republic of the Congo	Mongwalu	Proportion	2017	30.2	56.7	12.0
Democratic Republic of the Congo	Mongwalu	Count	2000	0	0	0
Democratic Republic of the Congo	Mongwalu	Count	2017	0	1	0
Democratic Republic of the Congo	Matadi	Proportion	2000	30.0	47.7	12.1
Democratic Republic of the Congo	Matadi	Proportion	2017	42.2	68.2	19.9
Democratic Republic of the Congo	Matadi	Count	2000	0	0	0
Democratic Republic of the Congo	Matadi	Count	2017	0	1	0

Table 4: ORS by Unit *(continued)*

Country	Unit	Metric	Year	Mean	Upper	Lower
Democratic Republic of the Congo	Mangai	Proportion	2000	19.2	38.4	7.8
Democratic Republic of the Congo	Mangai	Proportion	2017	42.1	67.9	17.1
Democratic Republic of the Congo	Mangai	Count	2000	0	0	0
Democratic Republic of the Congo	Mangai	Count	2017	0	1	0
Democratic Republic of the Congo	Yumbi	Proportion	2000	19.3	33.7	7.6
Democratic Republic of the Congo	Yumbi	Proportion	2017	39.6	63.7	20.2
Democratic Republic of the Congo	Yumbi	Count	2000	0	0	0
Democratic Republic of the Congo	Yumbi	Count	2017	0	1	0
Democratic Republic of the Congo	Pangi	Proportion	2000	16.5	27.8	8.4
Democratic Republic of the Congo	Pangi	Proportion	2017	37.7	58.9	22.8
Democratic Republic of the Congo	Pangi	Count	2000	0	0	0
Democratic Republic of the Congo	Pangi	Count	2017	0	1	0
Democratic Republic of the Congo	Walikale	Proportion	2000	18.2	30.7	8.7
Democratic Republic of the Congo	Walikale	Proportion	2017	35.6	52.8	19.9
Democratic Republic of the Congo	Walikale	Count	2000	0	0	0
Democratic Republic of the Congo	Walikale	Count	2017	0	1	0
Democratic Republic of the Congo	Uvira	Proportion	2000	20.7	37.6	10.1
Democratic Republic of the Congo	Uvira	Proportion	2017	38.4	60.5	19.5
Democratic Republic of the Congo	Uvira	Count	2000	0	0	0
Democratic Republic of the Congo	Uvira	Count	2017	0	1	0
Democratic Republic of the Congo	Yangambi	Proportion	2000	23.2	47.7	9.7
Democratic Republic of the Congo	Yangambi	Proportion	2017	39.0	64.7	18.7
Democratic Republic of the Congo	Yangambi	Count	2000	0	0	0
Democratic Republic of the Congo	Yangambi	Count	2017	0	1	0
Democratic Republic of the Congo	Mbanza-Ngungu	Proportion	2000	25.1	39.7	13.7
Democratic Republic of the Congo	Mbanza-Ngungu	Proportion	2017	45.8	69.5	25.9

Table 4: ORS by Unit *(continued)*

Country	Unit	Metric	Year	Mean	Upper	Lower
Democratic Republic of the Congo	Mbanza-Ngungu	Count	2000	0	0	0
Democratic Republic of the Congo	Mbanza-Ngungu	Count	2017	0	1	0
Democratic Republic of the Congo	Masi-Manimba	Proportion	2000	16.0	29.6	7.4
Democratic Republic of the Congo	Masi-Manimba	Proportion	2017	48.2	71.9	27.4
Democratic Republic of the Congo	Masi-Manimba	Count	2000	0	0	0
Democratic Republic of the Congo	Masi-Manimba	Count	2017	0	1	0
Democratic Republic of the Congo	Punia	Proportion	2000	17.7	30.9	8.9
Democratic Republic of the Congo	Punia	Proportion	2017	36.4	58.1	18.9
Democratic Republic of the Congo	Punia	Count	2000	0	0	0
Democratic Republic of the Congo	Punia	Count	2017	0	1	0
Democratic Republic of the Congo	Uvira (ville)	Proportion	2000	21.6	45.0	9.6
Democratic Republic of the Congo	Uvira (ville)	Proportion	2017	39.2	65.6	16.1
Democratic Republic of the Congo	Uvira (ville)	Count	2000	0	0	0
Democratic Republic of the Congo	Uvira (ville)	Count	2017	0	1	0
Democratic Republic of the Congo	Mbanza-Ngungu (ville)	Proportion	2000	26.6	44.1	12.1
Democratic Republic of the Congo	Mbanza-Ngungu (ville)	Proportion	2017	46.2	68.4	24.8
Democratic Republic of the Congo	Mbanza-Ngungu (ville)	Count	2000	0	0	0
Democratic Republic of the Congo	Mbanza-Ngungu (ville)	Count	2017	0	1	0
Democratic Republic of the Congo	Masi-Manimba (ville)	Proportion	2000	16.9	38.6	4.2
Democratic Republic of the Congo	Masi-Manimba (ville)	Proportion	2017	48.9	77.8	19.1
Democratic Republic of the Congo	Masi-Manimba (ville)	Count	2000	0	0	0
Democratic Republic of the Congo	Masi-Manimba (ville)	Count	2017	0	1	0
Democratic Republic of the Congo	Punia (ville)	Proportion	2000	20.9	37.2	9.7
Democratic Republic of the Congo	Punia (ville)	Proportion	2017	43.0	69.5	20.4
Democratic Republic of the Congo	Punia (ville)	Count	2000	0	0	0
Democratic Republic of the Congo	Punia (ville)	Count	2017	0	1	0

Table 4: ORS by Unit *(continued)*

Country	Unit	Metric	Year	Mean	Upper	Lower
Democratic Republic of the Congo	Walungu	Proportion	2000	19.5	34.3	8.8
Democratic Republic of the Congo	Walungu	Proportion	2017	36.6	58.2	18.4
Democratic Republic of the Congo	Walungu	Count	2000	0	0	0
Democratic Republic of the Congo	Walungu	Count	2017	0	1	0
Democratic Republic of the Congo	Moanda	Proportion	2000	26.0	42.1	13.2
Democratic Republic of the Congo	Moanda	Proportion	2017	45.0	67.6	25.5
Democratic Republic of the Congo	Moanda	Count	2000	0	0	0
Democratic Republic of the Congo	Moanda	Count	2017	0	1	0
Democratic Republic of the Congo	Moanda (ville)	Proportion	2000	39.8	64.2	20.1
Democratic Republic of the Congo	Moanda (ville)	Proportion	2017	52.6	75.9	29.3
Democratic Republic of the Congo	Moanda (ville)	Count	2000	0	1	0
Democratic Republic of the Congo	Moanda (ville)	Count	2017	1	1	0
Democratic Republic of the Congo	Seke-Banza	Proportion	2000	23.7	36.9	12.2
Democratic Republic of the Congo	Seke-Banza	Proportion	2017	42.8	65.5	23.3
Democratic Republic of the Congo	Seke-Banza	Count	2000	0	0	0
Democratic Republic of the Congo	Seke-Banza	Count	2017	0	1	0
Democratic Republic of the Congo	Songololo	Proportion	2000	24.2	38.4	12.9
Democratic Republic of the Congo	Songololo	Proportion	2017	45.6	69.2	26.1
Democratic Republic of the Congo	Songololo	Count	2000	0	0	0
Democratic Republic of the Congo	Songololo	Count	2017	0	1	0
Democratic Republic of the Congo	Tshela	Proportion	2000	25.4	41.9	13.2
Democratic Republic of the Congo	Tshela	Proportion	2017	45.8	68.8	24.7
Democratic Republic of the Congo	Tshela	Count	2000	0	0	0
Democratic Republic of the Congo	Tshela	Count	2017	0	1	0
Democratic Republic of the Congo	Tshela (ville)	Proportion	2000	28.2	48.5	13.3
Democratic Republic of the Congo	Tshela (ville)	Proportion	2017	45.9	70.4	23.0

Table 4: ORS by Unit *(continued)*

Country	Unit	Metric	Year	Mean	Upper	Lower
Democratic Republic of the Congo	Tshela (ville)	Count	2000	0	0	0
Democratic Republic of the Congo	Tshela (ville)	Count	2017	0	1	0
Djibouti	Ali Sabieh	Proportion	2000	66.0	78.8	49.7
Djibouti	Ali Sabieh	Proportion	2017	64.5	78.9	46.9
Djibouti	Ali Sabieh	Count	2000	1	1	0
Djibouti	Ali Sabieh	Count	2017	1	1	0
Djibouti	As Eyla	Proportion	2000	63.4	77.2	48.6
Djibouti	As Eyla	Proportion	2017	62.1	76.4	41.3
Djibouti	As Eyla	Count	2000	1	1	0
Djibouti	As Eyla	Count	2017	1	1	0
Djibouti	Djibouti	Proportion	2000	64.0	77.3	47.0
Djibouti	Djibouti	Proportion	2017	62.2	76.9	44.4
Djibouti	Djibouti	Count	2000	1	1	0
Djibouti	Djibouti	Count	2017	1	1	0
Djibouti	Alaili Dadda	Proportion	2000	63.9	76.9	47.3
Djibouti	Alaili Dadda	Proportion	2017	62.9	78.2	42.9
Djibouti	Alaili Dadda	Count	2000	1	1	0
Djibouti	Alaili Dadda	Count	2017	1	1	0
Djibouti	Balha	Proportion	2000	64.2	78.5	48.6
Djibouti	Balha	Proportion	2017	62.7	77.9	43.4
Djibouti	Balha	Count	2000	1	1	0
Djibouti	Balha	Count	2017	1	1	0
Djibouti	Dikhil	Proportion	2000	65.5	78.3	48.9
Djibouti	Dikhil	Proportion	2017	64.4	78.8	44.7
Djibouti	Dikhil	Count	2000	1	1	0
Djibouti	Dikhil	Count	2017	1	1	0
Djibouti	Obock	Proportion	2000	64.1	77.4	47.5
Djibouti	Obock	Proportion	2017	63.3	78.5	43.9
Djibouti	Obock	Count	2000	1	1	0
Djibouti	Obock	Count	2017	1	1	0
Djibouti	Dorra	Proportion	2000	64.5	76.8	49.1
Djibouti	Dorra	Proportion	2017	63.4	77.7	43.8
Djibouti	Dorra	Count	2000	1	1	0
Djibouti	Dorra	Count	2017	1	1	0
Djibouti	Yoboki	Proportion	2000	63.4	77.4	47.9
Djibouti	Yoboki	Proportion	2017	62.4	77.0	42.2
Djibouti	Yoboki	Count	2000	1	1	0
Djibouti	Yoboki	Count	2017	1	1	0
Djibouti	Randa	Proportion	2000	67.2	80.2	51.4
Djibouti	Randa	Proportion	2017	66.0	79.7	45.8
Djibouti	Randa	Count	2000	1	1	1
Djibouti	Randa	Count	2017	1	1	0
Djibouti	Tadjourah	Proportion	2000	64.6	78.8	48.2
Djibouti	Tadjourah	Proportion	2017	63.4	78.1	43.7
Djibouti	Tadjourah	Count	2000	1	1	0
Djibouti	Tadjourah	Count	2017	1	1	0
Dominican Republic	Azua de Compostela	Proportion	2000	36.4	49.8	24.1

Table 4: ORS by Unit *(continued)*

Country	Unit	Metric	Year	Mean	Upper	Lower
Dominican Republic	Azua de Compostela	Proportion	2017	47.6	62.7	31.0
Dominican Republic	Azua de Compostela	Count	2000	0	0	0
Dominican Republic	Azua de Compostela	Count	2017	0	1	0
Dominican Republic	Galvan	Proportion	2000	38.2	48.9	27.0
Dominican Republic	Galvan	Proportion	2017	47.3	62.3	33.3
Dominican Republic	Galvan	Count	2000	0	0	0
Dominican Republic	Galvan	Count	2017	0	1	0
Dominican Republic	Cabral	Proportion	2000	38.2	51.8	26.4
Dominican Republic	Cabral	Proportion	2017	45.1	63.2	31.3
Dominican Republic	Cabral	Count	2000	0	1	0
Dominican Republic	Cabral	Count	2017	0	1	0
Dominican Republic	Dajabón	Proportion	2000	38.4	48.9	29.2
Dominican Republic	Dajabón	Proportion	2017	46.5	60.1	34.0
Dominican Republic	Dajabón	Count	2000	0	0	0
Dominican Republic	Dajabón	Count	2017	0	1	0
Dominican Republic	Distrito Nacional	Proportion	2000	32.6	46.2	21.5
Dominican Republic	Distrito Nacional	Proportion	2017	45.9	59.9	31.8
Dominican Republic	Distrito Nacional	Count	2000	0	0	0
Dominican Republic	Distrito Nacional	Count	2017	0	1	0
Dominican Republic	Arenoso	Proportion	2000	29.4	41.4	20.7
Dominican Republic	Arenoso	Proportion	2017	40.6	54.5	26.2
Dominican Republic	Arenoso	Count	2000	0	0	0
Dominican Republic	Arenoso	Count	2017	0	1	0
Dominican Republic	Miches	Proportion	2000	29.6	41.7	19.3
Dominican Republic	Miches	Proportion	2017	39.8	54.7	24.8
Dominican Republic	Miches	Count	2000	0	0	0
Dominican Republic	Miches	Count	2017	0	1	0
Dominican Republic	Cayetano Germosén	Proportion	2000	29.8	42.2	18.3
Dominican Republic	Cayetano Germosén	Proportion	2017	44.6	59.3	29.4
Dominican Republic	Cayetano Germosén	Count	2000	0	0	0
Dominican Republic	Cayetano Germosén	Count	2017	0	1	0
Dominican Republic	El Valle	Proportion	2000	29.1	40.9	19.5
Dominican Republic	El Valle	Proportion	2017	39.4	55.0	25.3
Dominican Republic	El Valle	Count	2000	0	0	0
Dominican Republic	El Valle	Count	2017	0	1	0
Dominican Republic	Cristobal	Proportion	2000	38.5	49.6	27.7
Dominican Republic	Cristobal	Proportion	2017	46.4	59.9	31.8
Dominican Republic	Cristobal	Count	2000	0	0	0
Dominican Republic	Cristobal	Count	2017	0	1	0
Dominican Republic	La Laguna de Nisibón	Proportion	2000	31.1	43.2	20.1
Dominican Republic	La Laguna de Nisibón	Proportion	2017	40.9	56.1	25.6
Dominican Republic	La Laguna de Nisibón	Count	2000	0	0	0
Dominican Republic	La Laguna de Nisibón	Count	2017	0	1	0
Dominican Republic	Banica	Proportion	2000	39.5	49.9	30.3
Dominican Republic	Banica	Proportion	2017	48.2	60.3	36.5
Dominican Republic	Banica	Count	2000	0	0	0
Dominican Republic	Banica	Count	2017	0	1	0
Dominican Republic	Guaymate	Proportion	2000	30.9	42.3	20.2
Dominican Republic	Guaymate	Proportion	2017	41.0	57.3	26.5

Table 4: ORS by Unit *(continued)*

Country	Unit	Metric	Year	Mean	Upper	Lower
Dominican Republic	Guaymate	Count	2000	0	0	0
Dominican Republic	Guaymate	Count	2017	0	1	0
Dominican Republic	Concepción de la Vega	Proportion	2000	30.5	42.6	20.7
Dominican Republic	Concepción de la Vega	Proportion	2017	45.5	58.3	31.2
Dominican Republic	Concepción de la Vega	Count	2000	0	0	0
Dominican Republic	Concepción de la Vega	Count	2017	0	1	0
Dominican Republic	Cabrera	Proportion	2000	28.9	40.8	18.7
Dominican Republic	Cabrera	Proportion	2017	40.8	55.1	27.4
Dominican Republic	Cabrera	Count	2000	0	0	0
Dominican Republic	Cabrera	Count	2017	0	1	0
Dominican Republic	Bonao	Proportion	2000	30.6	42.6	20.3
Dominican Republic	Bonao	Proportion	2017	44.8	59.4	30.3
Dominican Republic	Bonao	Count	2000	0	0	0
Dominican Republic	Bonao	Count	2017	0	1	0
Dominican Republic	Castañuela	Proportion	2000	37.9	53.1	27.1
Dominican Republic	Castañuela	Proportion	2017	48.8	63.0	35.8
Dominican Republic	Castañuela	Count	2000	0	1	0
Dominican Republic	Castañuela	Count	2017	0	1	0
Dominican Republic	Bayaguana	Proportion	2000	30.2	42.0	20.8
Dominican Republic	Bayaguana	Proportion	2017	41.9	55.2	28.1
Dominican Republic	Bayaguana	Count	2000	0	0	0
Dominican Republic	Bayaguana	Count	2017	0	1	0
Dominican Republic	Pedernales	Proportion	2000	38.7	49.3	28.8
Dominican Republic	Pedernales	Proportion	2017	43.1	56.2	30.5
Dominican Republic	Pedernales	Count	2000	0	0	0
Dominican Republic	Pedernales	Count	2017	0	1	0
Dominican Republic	Baní	Proportion	2000	36.3	49.8	23.7
Dominican Republic	Baní	Proportion	2017	48.0	63.5	32.3
Dominican Republic	Baní	Count	2000	0	0	0
Dominican Republic	Baní	Count	2017	0	1	0
Dominican Republic	Altamira	Proportion	2000	30.8	42.3	21.2
Dominican Republic	Altamira	Proportion	2017	46.2	58.4	33.2
Dominican Republic	Altamira	Count	2000	0	0	0
Dominican Republic	Altamira	Count	2017	0	1	0
Dominican Republic	Salcedo	Proportion	2000	29.7	40.1	20.2
Dominican Republic	Salcedo	Proportion	2017	44.6	57.6	31.3
Dominican Republic	Salcedo	Count	2000	0	0	0
Dominican Republic	Salcedo	Count	2017	0	1	0
Dominican Republic	Las Terrenas	Proportion	2000	29.6	44.0	18.7
Dominican Republic	Las Terrenas	Proportion	2017	40.1	56.7	24.4
Dominican Republic	Las Terrenas	Count	2000	0	0	0
Dominican Republic	Las Terrenas	Count	2017	0	1	0
Dominican Republic	Bajos de Haina	Proportion	2000	33.5	46.3	22.8
Dominican Republic	Bajos de Haina	Proportion	2017	46.6	62.1	32.5
Dominican Republic	Bajos de Haina	Count	2000	0	0	0
Dominican Republic	Bajos de Haina	Count	2017	0	1	0
Dominican Republic	Rancho Arriba	Proportion	2000	34.4	47.1	23.3
Dominican Republic	Rancho Arriba	Proportion	2017	47.7	61.2	33.8
Dominican Republic	Rancho Arriba	Count	2000	0	0	0

Table 4: ORS by Unit *(continued)*

Country	Unit	Metric	Year	Mean	Upper	Lower
Dominican Republic	Rancho Arriba	Count	2017	0	1	0
Dominican Republic	Bohechio	Proportion	2000	35.0	46.7	25.0
Dominican Republic	Bohechio	Proportion	2017	49.4	62.4	35.2
Dominican Republic	Bohechio	Count	2000	0	0	0
Dominican Republic	Bohechio	Count	2017	0	1	0
Dominican Republic	Consuelo	Proportion	2000	30.1	43.0	19.2
Dominican Republic	Consuelo	Proportion	2017	40.7	55.8	25.5
Dominican Republic	Consuelo	Count	2000	0	0	0
Dominican Republic	Consuelo	Count	2017	0	1	0
Dominican Republic	Cevicos	Proportion	2000	29.3	39.5	20.3
Dominican Republic	Cevicos	Proportion	2017	41.6	55.1	27.9
Dominican Republic	Cevicos	Count	2000	0	0	0
Dominican Republic	Cevicos	Count	2017	0	1	0
Dominican Republic	Los Almácigos	Proportion	2000	37.4	48.9	28.1
Dominican Republic	Los Almácigos	Proportion	2017	49.9	64.6	34.8
Dominican Republic	Los Almácigos	Count	2000	0	0	0
Dominican Republic	Los Almácigos	Count	2017	0	1	0
Dominican Republic	Janico	Proportion	2000	32.5	44.6	22.8
Dominican Republic	Janico	Proportion	2017	48.5	60.4	36.2
Dominican Republic	Janico	Count	2000	0	0	0
Dominican Republic	Janico	Count	2017	0	1	0
Dominican Republic	Boca Chica	Proportion	2000	33.0	44.8	22.2
Dominican Republic	Boca Chica	Proportion	2017	45.6	61.3	30.3
Dominican Republic	Boca Chica	Count	2000	0	0	0
Dominican Republic	Boca Chica	Count	2017	0	1	0
Dominican Republic	Esperanza	Proportion	2000	31.8	43.8	22.1
Dominican Republic	Esperanza	Proportion	2017	48.1	60.7	33.1
Dominican Republic	Esperanza	Count	2000	0	0	0
Dominican Republic	Esperanza	Count	2017	0	1	0
Dominican Republic	Estebania	Proportion	2000	36.6	49.4	25.1
Dominican Republic	Estebania	Proportion	2017	48.3	62.4	34.1
Dominican Republic	Estebania	Count	2000	0	0	0
Dominican Republic	Estebania	Count	2017	0	1	0
Dominican Republic	Los Rios	Proportion	2000	39.4	51.1	28.5
Dominican Republic	Los Rios	Proportion	2017	46.3	60.2	33.3
Dominican Republic	Los Rios	Count	2000	0	1	0
Dominican Republic	Los Rios	Count	2017	0	1	0
Dominican Republic	El Peñón	Proportion	2000	36.9	50.5	24.9
Dominican Republic	El Peñón	Proportion	2017	44.8	62.3	29.7
Dominican Republic	El Peñón	Count	2000	0	1	0
Dominican Republic	El Peñón	Count	2017	0	1	0
Dominican Republic	El Pino	Proportion	2000	37.5	50.9	25.0
Dominican Republic	El Pino	Proportion	2017	48.7	65.0	34.6
Dominican Republic	El Pino	Count	2000	0	1	0
Dominican Republic	El Pino	Count	2017	0	1	0
Dominican Republic	Castillo	Proportion	2000	29.4	40.2	19.7
Dominican Republic	Castillo	Proportion	2017	41.6	55.8	27.5
Dominican Republic	Castillo	Count	2000	0	0	0
Dominican Republic	Castillo	Count	2017	0	1	0

Table 4: ORS by Unit *(continued)*

Country	Unit	Metric	Year	Mean	Upper	Lower
Dominican Republic	Santa Cruz del Seybo	Proportion	2000	30.0	41.2	20.4
Dominican Republic	Santa Cruz del Seybo	Proportion	2017	40.4	56.7	26.8
Dominican Republic	Santa Cruz del Seybo	Count	2000	0	0	0
Dominican Republic	Santa Cruz del Seybo	Count	2017	0	1	0
Dominican Republic	Gaspar Hernández	Proportion	2000	30.2	41.5	20.1
Dominican Republic	Gaspar Hernández	Proportion	2017	43.0	57.5	29.4
Dominican Republic	Gaspar Hernández	Count	2000	0	0	0
Dominican Republic	Gaspar Hernández	Count	2017	0	1	0
Dominican Republic	Hato Mayor del Rey	Proportion	2000	29.9	41.9	21.4
Dominican Republic	Hato Mayor del Rey	Proportion	2017	40.6	53.8	26.9
Dominican Republic	Hato Mayor del Rey	Count	2000	0	0	0
Dominican Republic	Hato Mayor del Rey	Count	2017	0	1	0
Dominican Republic	Duvergé	Proportion	2000	39.4	50.6	29.6
Dominican Republic	Duvergé	Proportion	2017	45.8	58.7	33.6
Dominican Republic	Duvergé	Count	2000	0	1	0
Dominican Republic	Duvergé	Count	2017	0	1	0
Dominican Republic	Salvaleón de Higüey	Proportion	2000	32.0	46.6	21.2
Dominican Republic	Salvaleón de Higüey	Proportion	2017	42.2	57.5	27.4
Dominican Republic	Salvaleón de Higüey	Count	2000	0	0	0
Dominican Republic	Salvaleón de Higüey	Count	2017	0	1	0
Dominican Republic	Comendador	Proportion	2000	39.1	49.3	29.4
Dominican Republic	Comendador	Proportion	2017	47.1	59.5	34.6
Dominican Republic	Comendador	Count	2000	0	0	0
Dominican Republic	Comendador	Count	2017	0	1	0
Dominican Republic	La Romana	Proportion	2000	31.3	44.3	20.2
Dominican Republic	La Romana	Proportion	2017	41.9	58.0	25.1
Dominican Republic	La Romana	Count	2000	0	0	0
Dominican Republic	La Romana	Count	2017	0	1	0
Dominican Republic	Constanza	Proportion	2000	33.4	45.4	22.8
Dominican Republic	Constanza	Proportion	2017	48.4	62.4	33.6
Dominican Republic	Constanza	Count	2000	0	0	0
Dominican Republic	Constanza	Count	2017	0	1	0
Dominican Republic	El Factor	Proportion	2000	29.1	42.4	18.8
Dominican Republic	El Factor	Proportion	2017	40.6	55.0	27.7
Dominican Republic	El Factor	Count	2000	0	0	0
Dominican Republic	El Factor	Count	2017	0	1	0
Dominican Republic	Maimón	Proportion	2000	30.9	42.3	19.8
Dominican Republic	Maimón	Proportion	2017	44.4	60.9	31.2
Dominican Republic	Maimón	Count	2000	0	0	0
Dominican Republic	Maimón	Count	2017	0	1	0
Dominican Republic	Guayubín	Proportion	2000	35.1	47.5	26.4
Dominican Republic	Guayubín	Proportion	2017	49.1	62.4	35.5
Dominican Republic	Guayubín	Count	2000	0	0	0
Dominican Republic	Guayubín	Count	2017	0	1	0
Dominican Republic	Esperalvillo	Proportion	2000	30.3	42.7	19.7
Dominican Republic	Esperalvillo	Proportion	2017	42.8	57.2	26.2
Dominican Republic	Esperalvillo	Count	2000	0	0	0
Dominican Republic	Esperalvillo	Count	2017	0	1	0
Dominican Republic	Nizao	Proportion	2000	36.1	48.6	22.7

Table 4: ORS by Unit *(continued)*

Country	Unit	Metric	Year	Mean	Upper	Lower
Dominican Republic	Nizao	Proportion	2017	47.8	64.1	33.6
Dominican Republic	Nizao	Count	2000	0	0	0
Dominican Republic	Nizao	Count	2017	0	1	0
Dominican Republic	Guananico	Proportion	2000	32.1	45.2	20.6
Dominican Republic	Guananico	Proportion	2017	47.2	59.7	32.6
Dominican Republic	Guananico	Count	2000	0	0	0
Dominican Republic	Guananico	Count	2017	0	1	0
Dominican Republic	Tenares	Proportion	2000	30.0	41.5	20.9
Dominican Republic	Tenares	Proportion	2017	44.2	57.0	30.3
Dominican Republic	Tenares	Count	2000	0	0	0
Dominican Republic	Tenares	Count	2017	0	1	0
Dominican Republic	Sánchez	Proportion	2000	29.8	41.6	20.3
Dominican Republic	Sánchez	Proportion	2017	40.8	53.8	26.9
Dominican Republic	Sánchez	Count	2000	0	0	0
Dominican Republic	Sánchez	Count	2017	0	1	0
Dominican Republic	Cambita Garabito	Proportion	2000	34.8	48.0	23.9
Dominican Republic	Cambita Garabito	Proportion	2017	47.5	61.3	32.6
Dominican Republic	Cambita Garabito	Count	2000	0	0	0
Dominican Republic	Cambita Garabito	Count	2017	0	1	0
Dominican Republic	Sabana Larga	Proportion	2000	34.5	46.2	22.9
Dominican Republic	Sabana Larga	Proportion	2017	47.2	63.0	30.4
Dominican Republic	Sabana Larga	Count	2000	0	0	0
Dominican Republic	Sabana Larga	Count	2017	0	1	0
Dominican Republic	El Cercado	Proportion	2000	37.8	49.6	27.8
Dominican Republic	El Cercado	Proportion	2017	48.4	62.9	35.2
Dominican Republic	El Cercado	Count	2000	0	0	0
Dominican Republic	El Cercado	Count	2017	0	1	0
Dominican Republic	Guayacanes	Proportion	2000	32.3	47.6	22.5
Dominican Republic	Guayacanes	Proportion	2017	43.4	58.5	28.6
Dominican Republic	Guayacanes	Count	2000	0	0	0
Dominican Republic	Guayacanes	Count	2017	0	1	0
Dominican Republic	Cotuí	Proportion	2000	29.5	40.3	20.5
Dominican Republic	Cotuí	Proportion	2017	43.2	56.6	29.1
Dominican Republic	Cotuí	Count	2000	0	0	0
Dominican Republic	Cotuí	Count	2017	0	1	0
Dominican Republic	Monción	Proportion	2000	33.3	46.0	22.6
Dominican Republic	Monción	Proportion	2017	49.0	63.6	33.4
Dominican Republic	Monción	Count	2000	0	0	0
Dominican Republic	Monción	Count	2017	0	1	0
Dominican Republic	Licey al Medio	Proportion	2000	31.3	45.8	20.2
Dominican Republic	Licey al Medio	Proportion	2017	46.9	61.7	31.0
Dominican Republic	Licey al Medio	Count	2000	0	0	0
Dominican Republic	Licey al Medio	Count	2017	0	1	0
Dominican Republic	Guerra	Proportion	2000	32.2	44.5	22.2
Dominican Republic	Guerra	Proportion	2017	44.4	57.2	29.2
Dominican Republic	Guerra	Count	2000	0	0	0
Dominican Republic	Guerra	Count	2017	0	1	0
Dominican Republic	Laguna Salada	Proportion	2000	32.5	43.7	23.1
Dominican Republic	Laguna Salada	Proportion	2017	47.6	60.2	34.3

Table 4: ORS by Unit *(continued)*

Country	Unit	Metric	Year	Mean	Upper	Lower
Dominican Republic	Laguna Salada	Count	2000	0	0	0
Dominican Republic	Laguna Salada	Count	2017	0	1	0
Dominican Republic	Guayabal	Proportion	2000	35.1	46.0	25.3
Dominican Republic	Guayabal	Proportion	2017	49.5	62.8	36.3
Dominican Republic	Guayabal	Count	2000	0	0	0
Dominican Republic	Guayabal	Count	2017	0	1	0
Dominican Republic	Neyba	Proportion	2000	38.7	51.2	27.8
Dominican Republic	Neyba	Proportion	2017	46.9	61.0	32.5
Dominican Republic	Neyba	Count	2000	0	1	0
Dominican Republic	Neyba	Count	2017	0	1	0
Dominican Republic	Enriquillo	Proportion	2000	39.4	52.0	28.7
Dominican Republic	Enriquillo	Proportion	2017	44.7	60.5	30.6
Dominican Republic	Enriquillo	Count	2000	0	1	0
Dominican Republic	Enriquillo	Count	2017	0	1	0
Dominican Republic	Loma de Cabrera	Proportion	2000	38.9	49.5	29.4
Dominican Republic	Loma de Cabrera	Proportion	2017	48.0	61.3	34.1
Dominican Republic	Loma de Cabrera	Count	2000	0	0	0
Dominican Republic	Loma de Cabrera	Count	2017	0	1	0
Dominican Republic	Hostos	Proportion	2000	29.1	40.1	18.4
Dominican Republic	Hostos	Proportion	2017	41.4	55.7	27.2
Dominican Republic	Hostos	Count	2000	0	0	0
Dominican Republic	Hostos	Count	2017	0	1	0
Dominican Republic	Jamao al Norte	Proportion	2000	31.0	43.5	20.4
Dominican Republic	Jamao al Norte	Proportion	2017	44.5	58.7	29.7
Dominican Republic	Jamao al Norte	Count	2000	0	0	0
Dominican Republic	Jamao al Norte	Count	2017	0	1	0
Dominican Republic	Sabana de la Mar	Proportion	2000	29.4	42.0	20.7
Dominican Republic	Sabana de la Mar	Proportion	2017	40.1	54.7	27.0
Dominican Republic	Sabana de la Mar	Count	2000	0	0	0
Dominican Republic	Sabana de la Mar	Count	2017	0	1	0
Dominican Republic	Jimaní	Proportion	2000	38.9	49.5	30.7
Dominican Republic	Jimaní	Proportion	2017	44.0	57.3	33.6
Dominican Republic	Jimaní	Count	2000	0	0	0
Dominican Republic	Jimaní	Count	2017	0	1	0
Dominican Republic	San Rafael del Yuma	Proportion	2000	32.4	45.9	22.0
Dominican Republic	San Rafael del Yuma	Proportion	2017	42.5	58.9	27.9
Dominican Republic	San Rafael del Yuma	Count	2000	0	0	0
Dominican Republic	San Rafael del Yuma	Count	2017	0	1	0
Dominican Republic	El Llano	Proportion	2000	37.4	48.5	28.1
Dominican Republic	El Llano	Proportion	2017	46.3	59.8	33.4
Dominican Republic	El Llano	Count	2000	0	0	0
Dominican Republic	El Llano	Count	2017	0	1	0
Dominican Republic	Villa Hermosa	Proportion	2000	31.2	45.8	19.4
Dominican Republic	Villa Hermosa	Proportion	2017	41.8	57.9	26.0
Dominican Republic	Villa Hermosa	Count	2000	0	0	0
Dominican Republic	Villa Hermosa	Count	2017	0	1	0
Dominican Republic	Jarabacoa	Proportion	2000	33.5	44.3	22.2
Dominican Republic	Jarabacoa	Proportion	2017	48.6	62.8	33.8
Dominican Republic	Jarabacoa	Count	2000	0	0	0

Table 4: ORS by Unit *(continued)*

Country	Unit	Metric	Year	Mean	Upper	Lower
Dominican Republic	Jarabacoa	Count	2017	0	1	0
Dominican Republic	Nagua	Proportion	2000	29.3	42.5	20.7
Dominican Republic	Nagua	Proportion	2017	41.1	56.5	27.8
Dominican Republic	Nagua	Count	2000	0	0	0
Dominican Republic	Nagua	Count	2017	0	1	0
Dominican Republic	Piedra Blanca	Proportion	2000	31.9	43.0	21.9
Dominican Republic	Piedra Blanca	Proportion	2017	45.5	59.7	32.0
Dominican Republic	Piedra Blanca	Count	2000	0	0	0
Dominican Republic	Piedra Blanca	Count	2017	0	1	0
Dominican Republic	Las Matas de Santa Cruz	Proportion	2000	38.5	52.1	28.4
Dominican Republic	Las Matas de Santa Cruz	Proportion	2017	49.7	64.4	35.4
Dominican Republic	Las Matas de Santa Cruz	Count	2000	0	1	0
Dominican Republic	Las Matas de Santa Cruz	Count	2017	0	1	0
Dominican Republic	Monte Plata	Proportion	2000	30.0	41.7	20.0
Dominican Republic	Monte Plata	Proportion	2017	41.8	54.8	27.3
Dominican Republic	Monte Plata	Count	2000	0	0	0
Dominican Republic	Monte Plata	Count	2017	0	1	0
Dominican Republic	Imbert	Proportion	2000	30.9	43.0	20.0
Dominican Republic	Imbert	Proportion	2017	46.1	58.9	33.1
Dominican Republic	Imbert	Count	2000	0	0	0
Dominican Republic	Imbert	Count	2017	0	1	0
Dominican Republic	Villa Tapia	Proportion	2000	29.3	39.9	20.9
Dominican Republic	Villa Tapia	Proportion	2017	44.0	58.0	30.2
Dominican Republic	Villa Tapia	Count	2000	0	0	0
Dominican Republic	Villa Tapia	Count	2017	0	1	0
Dominican Republic	Santa Bárbara de Samaná	Proportion	2000	29.8	43.4	20.2
Dominican Republic	Santa Bárbara de Samaná	Proportion	2017	40.1	54.0	26.3
Dominican Republic	Santa Bárbara de Samaná	Count	2000	0	0	0
Dominican Republic	Santa Bárbara de Samaná	Count	2017	0	1	0
Dominican Republic	Los Cacaos	Proportion	2000	36.1	49.2	25.9
Dominican Republic	Los Cacaos	Proportion	2017	48.8	63.5	34.1
Dominican Republic	Los Cacaos	Count	2000	0	0	0
Dominican Republic	Los Cacaos	Count	2017	0	1	0
Dominican Republic	San José de Ocoa	Proportion	2000	35.9	48.2	24.2
Dominican Republic	San José de Ocoa	Proportion	2017	48.3	63.8	32.4
Dominican Republic	San José de Ocoa	Count	2000	0	0	0
Dominican Republic	San José de Ocoa	Count	2017	0	1	0
Dominican Republic	Juan de Herrera	Proportion	2000	34.2	48.5	24.5
Dominican Republic	Juan de Herrera	Proportion	2017	48.1	61.8	35.3
Dominican Republic	Juan de Herrera	Count	2000	0	0	0
Dominican Republic	Juan de Herrera	Count	2017	0	1	0
Dominican Republic	Los Llanos	Proportion	2000	31.2	44.4	21.9
Dominican Republic	Los Llanos	Proportion	2017	42.4	56.2	29.1
Dominican Republic	Los Llanos	Count	2000	0	0	0
Dominican Republic	Los Llanos	Count	2017	0	1	0
Dominican Republic	Fantino	Proportion	2000	29.6	41.4	19.6
Dominican Republic	Fantino	Proportion	2017	43.1	58.5	27.5
Dominican Republic	Fantino	Count	2000	0	0	0
Dominican Republic	Fantino	Count	2017	0	1	0

Table 4: ORS by Unit *(continued)*

Country	Unit	Metric	Year	Mean	Upper	Lower
Dominican Republic	San Ignacio de Sabaneta	Proportion	2000	35.5	47.0	26.2
Dominican Republic	San Ignacio de Sabaneta	Proportion	2017	48.8	63.1	35.5
Dominican Republic	San Ignacio de Sabaneta	Count	2000	0	0	0
Dominican Republic	San Ignacio de Sabaneta	Count	2017	0	1	0
Dominican Republic	Puñal	Proportion	2000	30.6	43.9	19.9
Dominican Republic	Puñal	Proportion	2017	47.1	61.6	33.6
Dominican Republic	Puñal	Count	2000	0	0	0
Dominican Republic	Puñal	Count	2017	0	1	0
Dominican Republic	Los Alcarrizos	Proportion	2000	32.3	45.8	20.9
Dominican Republic	Los Alcarrizos	Proportion	2017	46.1	61.3	31.0
Dominican Republic	Los Alcarrizos	Count	2000	0	0	0
Dominican Republic	Los Alcarrizos	Count	2017	0	1	0
Dominican Republic	Mao	Proportion	2000	32.3	43.8	22.9
Dominican Republic	Mao	Proportion	2017	48.6	61.2	35.1
Dominican Republic	Mao	Count	2000	0	0	0
Dominican Republic	Mao	Count	2017	0	1	0
Dominican Republic	Las Charcas	Proportion	2000	36.7	49.1	25.9
Dominican Republic	Las Charcas	Proportion	2017	48.1	63.7	32.0
Dominican Republic	Las Charcas	Count	2000	0	0	0
Dominican Republic	Las Charcas	Count	2017	0	1	0
Dominican Republic	Tamayo	Proportion	2000	37.6	50.1	27.3
Dominican Republic	Tamayo	Proportion	2017	47.8	62.2	33.9
Dominican Republic	Tamayo	Count	2000	0	1	0
Dominican Republic	Tamayo	Count	2017	0	1	0
Dominican Republic	Fundación	Proportion	2000	36.6	48.4	23.5
Dominican Republic	Fundación	Proportion	2017	45.1	62.1	29.1
Dominican Republic	Fundación	Count	2000	0	0	0
Dominican Republic	Fundación	Count	2017	0	1	0
Dominican Republic	Partido	Proportion	2000	37.8	49.0	27.4
Dominican Republic	Partido	Proportion	2017	48.7	63.5	34.7
Dominican Republic	Partido	Count	2000	0	0	0
Dominican Republic	Partido	Count	2017	0	1	0
Dominican Republic	Las Guaranas	Proportion	2000	29.0	38.7	20.2
Dominican Republic	Las Guaranas	Proportion	2017	42.0	57.5	27.8
Dominican Republic	Las Guaranas	Count	2000	0	0	0
Dominican Republic	Las Guaranas	Count	2017	0	1	0
Dominican Republic	Moca	Proportion	2000	30.4	41.6	20.1
Dominican Republic	Moca	Proportion	2017	45.8	57.7	32.1
Dominican Republic	Moca	Count	2000	0	0	0
Dominican Republic	Moca	Count	2017	0	1	0
Dominican Republic	La Descubierta	Proportion	2000	39.8	50.9	30.3
Dominican Republic	La Descubierta	Proportion	2017	45.4	58.8	33.2
Dominican Republic	La Descubierta	Count	2000	0	1	0
Dominican Republic	La Descubierta	Count	2017	0	1	0
Dominican Republic	Hondo Valle	Proportion	2000	39.9	51.5	29.7
Dominican Republic	Hondo Valle	Proportion	2017	47.9	59.5	35.8
Dominican Republic	Hondo Valle	Count	2000	0	1	0
Dominican Republic	Hondo Valle	Count	2017	0	1	0
Dominican Republic	Jima Abajo	Proportion	2000	30.1	40.5	20.9

Table 4: ORS by Unit *(continued)*

Country	Unit	Metric	Year	Mean	Upper	Lower
Dominican Republic	Jima Abajo	Proportion	2017	44.2	58.1	30.3
Dominican Republic	Jima Abajo	Count	2000	0	0	0
Dominican Republic	Jima Abajo	Count	2017	0	1	0
Dominican Republic	Rio San Juan	Proportion	2000	29.4	40.8	19.3
Dominican Republic	Rio San Juan	Proportion	2017	41.3	55.7	27.5
Dominican Republic	Rio San Juan	Count	2000	0	0	0
Dominican Republic	Rio San Juan	Count	2017	0	1	0
Dominican Republic	Pepillo Salcedo	Proportion	2000	39.6	51.1	29.4
Dominican Republic	Pepillo Salcedo	Proportion	2017	47.7	61.5	34.7
Dominican Republic	Pepillo Salcedo	Count	2000	0	1	0
Dominican Republic	Pepillo Salcedo	Count	2017	0	1	0
Dominican Republic	Sabana Grande de Boyá	Proportion	2000	30.5	42.2	20.9
Dominican Republic	Sabana Grande de Boyá	Proportion	2017	42.2	55.7	28.0
Dominican Republic	Sabana Grande de Boyá	Count	2000	0	0	0
Dominican Republic	Sabana Grande de Boyá	Count	2017	0	1	0
Dominican Republic	La Isabela	Proportion	2000	33.2	47.3	22.8
Dominican Republic	La Isabela	Proportion	2017	46.7	61.0	31.6
Dominican Republic	La Isabela	Count	2000	0	0	0
Dominican Republic	La Isabela	Count	2017	0	1	0
Dominican Republic	Nigua	Proportion	2000	34.4	47.8	22.6
Dominican Republic	Nigua	Proportion	2017	47.1	62.6	31.8
Dominican Republic	Nigua	Count	2000	0	0	0
Dominican Republic	Nigua	Count	2017	0	1	0
Dominican Republic	Las Matas de Farfan	Proportion	2000	37.3	48.6	27.3
Dominican Republic	Las Matas de Farfan	Proportion	2017	48.6	61.6	36.5
Dominican Republic	Las Matas de Farfan	Count	2000	0	0	0
Dominican Republic	Las Matas de Farfan	Count	2017	0	1	0
Dominican Republic	Quisquella	Proportion	2000	31.0	44.4	21.2
Dominican Republic	Quisquella	Proportion	2017	41.5	56.1	26.6
Dominican Republic	Quisquella	Count	2000	0	0	0
Dominican Republic	Quisquella	Count	2017	0	1	0
Dominican Republic	La Mata	Proportion	2000	29.3	39.8	20.5
Dominican Republic	La Mata	Proportion	2017	42.3	56.2	27.9
Dominican Republic	La Mata	Count	2000	0	0	0
Dominican Republic	La Mata	Count	2017	0	1	0
Dominican Republic	Sabana Iglesia	Proportion	2000	31.9	44.8	22.2
Dominican Republic	Sabana Iglesia	Proportion	2017	47.6	62.3	32.3
Dominican Republic	Sabana Iglesia	Count	2000	0	0	0
Dominican Republic	Sabana Iglesia	Count	2017	0	1	0
Dominican Republic	Pedro Brand	Proportion	2000	32.0	44.4	21.2
Dominican Republic	Pedro Brand	Proportion	2017	44.7	58.8	29.9
Dominican Republic	Pedro Brand	Count	2000	0	0	0
Dominican Republic	Pedro Brand	Count	2017	0	1	0
Dominican Republic	Las Yayas de Viajama	Proportion	2000	35.1	45.9	26.3
Dominican Republic	Las Yayas de Viajama	Proportion	2017	47.5	61.1	35.1
Dominican Republic	Las Yayas de Viajama	Count	2000	0	0	0
Dominican Republic	Las Yayas de Viajama	Count	2017	0	1	0
Dominican Republic	Villa Jaragua	Proportion	2000	38.6	50.4	28.2
Dominican Republic	Villa Jaragua	Proportion	2017	46.7	60.8	31.6

Table 4: ORS by Unit *(continued)*

Country	Unit	Metric	Year	Mean	Upper	Lower
Dominican Republic	Villa Jaragua	Count	2000	0	1	0
Dominican Republic	Villa Jaragua	Count	2017	0	1	0
Dominican Republic	Jaquimeyes	Proportion	2000	37.0	49.2	24.9
Dominican Republic	Jaquimeyes	Proportion	2017	45.2	60.2	30.9
Dominican Republic	Jaquimeyes	Count	2000	0	0	0
Dominican Republic	Jaquimeyes	Count	2017	0	1	0
Dominican Republic	Restauración	Proportion	2000	39.4	49.7	30.5
Dominican Republic	Restauración	Proportion	2017	48.6	60.7	36.1
Dominican Republic	Restauración	Count	2000	0	0	0
Dominican Republic	Restauración	Count	2017	0	1	0
Dominican Republic	Pimentel	Proportion	2000	28.0	38.8	18.8
Dominican Republic	Pimentel	Proportion	2017	41.0	56.8	27.4
Dominican Republic	Pimentel	Count	2000	0	0	0
Dominican Republic	Pimentel	Count	2017	0	1	0
Dominican Republic	Mella	Proportion	2000	39.1	50.3	28.0
Dominican Republic	Mella	Proportion	2017	46.5	60.7	33.7
Dominican Republic	Mella	Count	2000	0	1	0
Dominican Republic	Mella	Count	2017	0	1	0
Dominican Republic	Juan Santiago	Proportion	2000	38.8	50.1	28.6
Dominican Republic	Juan Santiago	Proportion	2017	48.2	61.2	35.1
Dominican Republic	Juan Santiago	Count	2000	0	1	0
Dominican Republic	Juan Santiago	Count	2017	0	1	0
Dominican Republic	San Fernando de Monte Cristi	Proportion	2000	39.6	52.9	29.3
Dominican Republic	San Fernando de Monte Cristi	Proportion	2017	48.4	64.4	34.3
Dominican Republic	San Fernando de Monte Cristi	Count	2000	0	1	0
Dominican Republic	San Fernando de Monte Cristi	Count	2017	0	1	0
Dominican Republic	Yamasá	Proportion	2000	30.7	42.3	20.6
Dominican Republic	Yamasá	Proportion	2017	42.9	57.2	28.6
Dominican Republic	Yamasá	Count	2000	0	0	0
Dominican Republic	Yamasá	Count	2017	0	1	0
Dominican Republic	Los Hidalgos	Proportion	2000	32.5	44.8	22.1
Dominican Republic	Los Hidalgos	Proportion	2017	47.1	60.5	33.6
Dominican Republic	Los Hidalgos	Count	2000	0	0	0
Dominican Republic	Los Hidalgos	Count	2017	0	1	0
Dominican Republic	Sabana Grande de Palenque	Proportion	2000	35.9	49.4	23.5
Dominican Republic	Sabana Grande de Palenque	Proportion	2017	47.9	66.0	32.8
Dominican Republic	Sabana Grande de Palenque	Count	2000	0	0	0
Dominican Republic	Sabana Grande de Palenque	Count	2017	0	1	0
Dominican Republic	San Juan de la Maguana	Proportion	2000	34.7	46.6	25.5
Dominican Republic	San Juan de la Maguana	Proportion	2017	48.1	61.3	36.0
Dominican Republic	San Juan de la Maguana	Count	2000	0	0	0
Dominican Republic	San Juan de la Maguana	Count	2017	0	1	0

Table 4: ORS by Unit *(continued)*

Country	Unit	Metric	Year	Mean	Upper	Lower
Dominican Republic	Ramón Santana	Proportion	2000	31.2	45.2	20.7
Dominican Republic	Ramón Santana	Proportion	2017	41.4	56.6	25.0
Dominican Republic	Ramón Santana	Count	2000	0	0	0
Dominican Republic	Ramón Santana	Count	2017	0	1	0
Dominican Republic	San José de Las Matas	Proportion	2000	32.7	43.5	23.6
Dominican Republic	San José de Las Matas	Proportion	2017	48.7	60.7	35.1
Dominican Republic	San José de Las Matas	Count	2000	0	0	0
Dominican Republic	San José de Las Matas	Count	2017	0	1	0
Dominican Republic	Santo Domingo Este	Proportion	2000	32.6	44.5	23.0
Dominican Republic	Santo Domingo Este	Proportion	2017	45.7	60.9	31.4
Dominican Republic	Santo Domingo Este	Count	2000	0	0	0
Dominican Republic	Santo Domingo Este	Count	2017	0	1	0
Dominican Republic	Padre Las Casas	Proportion	2000	34.6	46.0	25.1
Dominican Republic	Padre Las Casas	Proportion	2017	48.5	63.2	35.7
Dominican Republic	Padre Las Casas	Count	2000	0	0	0
Dominican Republic	Padre Las Casas	Count	2017	0	1	0
Dominican Republic	La Cienaga	Proportion	2000	38.7	51.2	26.3
Dominican Republic	La Cienaga	Proportion	2017	45.3	60.9	32.1
Dominican Republic	La Cienaga	Count	2000	0	1	0
Dominican Republic	La Cienaga	Count	2017	0	1	0
Dominican Republic	San Francisco de Macorís	Proportion	2000	29.1	39.9	20.7
Dominican Republic	San Francisco de Macorís	Proportion	2017	42.7	57.0	28.4
Dominican Republic	San Francisco de Macorís	Count	2000	0	0	0
Dominican Republic	San Francisco de Macorís	Count	2017	0	1	0
Dominican Republic	Postrer Rio	Proportion	2000	39.8	51.0	29.5
Dominican Republic	Postrer Rio	Proportion	2017	46.7	59.6	34.7
Dominican Republic	Postrer Rio	Count	2000	0	1	0
Dominican Republic	Postrer Rio	Count	2017	0	1	0
Dominican Republic	Pedro Santana	Proportion	2000	38.6	48.4	29.3
Dominican Republic	Pedro Santana	Proportion	2017	50.0	61.4	36.9
Dominican Republic	Pedro Santana	Count	2000	0	0	0
Dominican Republic	Pedro Santana	Count	2017	0	1	0
Dominican Republic	Villa Vázquez	Proportion	2000	38.0	50.5	27.5
Dominican Republic	Villa Vázquez	Proportion	2017	49.5	63.6	34.9
Dominican Republic	Villa Vázquez	Count	2000	0	1	0
Dominican Republic	Villa Vázquez	Count	2017	0	1	0
Dominican Republic	Luperon	Proportion	2000	31.9	45.0	22.1
Dominican Republic	Luperon	Proportion	2017	45.8	58.7	32.2
Dominican Republic	Luperon	Count	2000	0	0	0
Dominican Republic	Luperon	Count	2017	0	1	0
Dominican Republic	San Cristóbal	Proportion	2000	33.3	44.6	22.9
Dominican Republic	San Cristóbal	Proportion	2017	46.5	61.7	32.7
Dominican Republic	San Cristóbal	Count	2000	0	0	0
Dominican Republic	San Cristóbal	Count	2017	0	1	0
Dominican Republic	Vallejuelo	Proportion	2000	37.6	48.7	27.9
Dominican Republic	Vallejuelo	Proportion	2017	48.7	62.9	35.2
Dominican Republic	Vallejuelo	Count	2000	0	0	0
Dominican Republic	Vallejuelo	Count	2017	0	1	0
Dominican Republic	San Pedro de Macorís	Proportion	2000	30.7	45.3	20.3

Table 4: ORS by Unit *(continued)*

Country	Unit	Metric	Year	Mean	Upper	Lower
Dominican Republic	San Pedro de Macorís	Proportion	2017	41.4	56.4	28.3
Dominican Republic	San Pedro de Macorís	Count	2000	0	0	0
Dominican Republic	San Pedro de Macorís	Count	2017	0	1	0
Dominican Republic	Santiago de los Caballeros	Proportion	2000	30.8	43.4	21.6
Dominican Republic	Santiago de los Caballeros	Proportion	2017	47.1	58.5	34.3
Dominican Republic	Santiago de los Caballeros	Count	2000	0	0	0
Dominican Republic	Santiago de los Caballeros	Count	2017	0	1	0
Dominican Republic	Santo Domingo Norte	Proportion	2000	32.4	45.2	22.1
Dominican Republic	Santo Domingo Norte	Proportion	2017	45.5	60.0	32.5
Dominican Republic	Santo Domingo Norte	Count	2000	0	0	0
Dominican Republic	Santo Domingo Norte	Count	2017	0	1	0
Dominican Republic	Peralta	Proportion	2000	36.2	46.6	26.2
Dominican Republic	Peralta	Proportion	2017	48.7	62.9	33.3
Dominican Republic	Peralta	Count	2000	0	0	0
Dominican Republic	Peralta	Count	2017	0	1	0
Dominican Republic	Las Salinas	Proportion	2000	38.6	50.1	28.0
Dominican Republic	Las Salinas	Proportion	2017	45.9	60.5	32.8
Dominican Republic	Las Salinas	Count	2000	0	1	0
Dominican Republic	Las Salinas	Count	2017	0	1	0
Dominican Republic	Villa Rivas	Proportion	2000	29.3	40.4	21.0
Dominican Republic	Villa Rivas	Proportion	2017	40.9	54.0	25.9
Dominican Republic	Villa Rivas	Count	2000	0	0	0
Dominican Republic	Villa Rivas	Count	2017	0	1	0
Dominican Republic	Montellano	Proportion	2000	29.7	43.0	19.0
Dominican Republic	Montellano	Proportion	2017	44.2	58.3	27.9
Dominican Republic	Montellano	Count	2000	0	0	0
Dominican Republic	Montellano	Count	2017	0	1	0
Dominican Republic	San Gregorio de Yaguate	Proportion	2000	35.6	49.0	24.1
Dominican Republic	San Gregorio de Yaguate	Proportion	2017	48.1	62.5	32.8
Dominican Republic	San Gregorio de Yaguate	Count	2000	0	0	0
Dominican Republic	San Gregorio de Yaguate	Count	2017	0	1	0
Dominican Republic	Tamboril	Proportion	2000	31.8	45.5	21.3
Dominican Republic	Tamboril	Proportion	2017	47.3	60.5	33.1
Dominican Republic	Tamboril	Count	2000	0	0	0
Dominican Republic	Tamboril	Count	2017	0	1	0
Dominican Republic	Santo Domingo Oeste	Proportion	2000	33.5	45.8	20.8
Dominican Republic	Santo Domingo Oeste	Proportion	2017	46.9	62.6	31.9
Dominican Republic	Santo Domingo Oeste	Count	2000	0	0	0
Dominican Republic	Santo Domingo Oeste	Count	2017	0	1	0
Dominican Republic	Pueblo Viejo	Proportion	2000	36.8	48.9	26.3
Dominican Republic	Pueblo Viejo	Proportion	2017	47.7	63.6	33.4
Dominican Republic	Pueblo Viejo	Count	2000	0	0	0
Dominican Republic	Pueblo Viejo	Count	2017	0	1	0
Dominican Republic	Paraiso	Proportion	2000	38.8	51.2	28.0
Dominican Republic	Paraiso	Proportion	2017	44.3	60.4	29.4
Dominican Republic	Paraiso	Count	2000	0	1	0
Dominican Republic	Paraiso	Count	2017	0	1	0
Dominican Republic	San Felipe de Puerto Plata	Proportion	2000	29.7	40.7	18.8
Dominican Republic	San Felipe de Puerto Plata	Proportion	2017	44.7	57.0	31.3

Table 4: ORS by Unit *(continued)*

Country	Unit	Metric	Year	Mean	Upper	Lower
Dominican Republic	San Felipe de Puerto Plata	Count	2000	0	0	0
Dominican Republic	San Felipe de Puerto Plata	Count	2017	0	1	0
Dominican Republic	Villa Altagracia	Proportion	2000	31.9	44.1	22.6
Dominican Republic	Villa Altagracia	Proportion	2017	44.7	58.2	30.8
Dominican Republic	Villa Altagracia	Count	2000	0	0	0
Dominican Republic	Villa Altagracia	Count	2017	0	1	0
Dominican Republic	Villa Bisonó	Proportion	2000	31.4	46.6	21.8
Dominican Republic	Villa Bisonó	Proportion	2017	47.8	60.2	33.8
Dominican Republic	Villa Bisonó	Count	2000	0	0	0
Dominican Republic	Villa Bisonó	Count	2017	0	1	0
Dominican Republic	Sabana Yegua	Proportion	2000	35.8	48.4	24.7
Dominican Republic	Sabana Yegua	Proportion	2017	46.8	62.6	31.3
Dominican Republic	Sabana Yegua	Count	2000	0	0	0
Dominican Republic	Sabana Yegua	Count	2017	0	1	0
Dominican Republic	Polo	Proportion	2000	39.9	51.2	28.6
Dominican Republic	Polo	Proportion	2017	45.8	60.8	32.1
Dominican Republic	Polo	Count	2000	0	1	0
Dominican Republic	Polo	Count	2017	0	1	0
Dominican Republic	Sosua	Proportion	2000	30.5	41.6	20.4
Dominican Republic	Sosua	Proportion	2017	44.0	58.6	30.3
Dominican Republic	Sosua	Count	2000	0	0	0
Dominican Republic	Sosua	Count	2017	0	1	0
Dominican Republic	Villa Gonzalez	Proportion	2000	31.0	42.4	20.4
Dominican Republic	Villa Gonzalez	Proportion	2017	47.2	59.6	34.9
Dominican Republic	Villa Gonzalez	Count	2000	0	0	0
Dominican Republic	Villa Gonzalez	Count	2017	0	1	0
Dominican Republic	Villa Tabara Arriba	Proportion	2000	35.4	47.1	25.8
Dominican Republic	Villa Tabara Arriba	Proportion	2017	46.9	61.7	33.6
Dominican Republic	Villa Tabara Arriba	Count	2000	0	0	0
Dominican Republic	Villa Tabara Arriba	Count	2017	0	1	0
Dominican Republic	Santa Cruz de Barahona	Proportion	2000	36.8	48.4	25.0
Dominican Republic	Santa Cruz de Barahona	Proportion	2017	45.2	61.4	29.6
Dominican Republic	Santa Cruz de Barahona	Count	2000	0	0	0
Dominican Republic	Santa Cruz de Barahona	Count	2017	0	1	0
Dominican Republic	Vicente Noble	Proportion	2000	37.4	50.7	27.2
Dominican Republic	Vicente Noble	Proportion	2017	47.2	61.2	32.3
Dominican Republic	Vicente Noble	Count	2000	0	1	0
Dominican Republic	Vicente Noble	Count	2017	0	1	0
Ecuador	Camilo Ponce Enriquez	Proportion	2000	18.0	26.3	11.6
Ecuador	Camilo Ponce Enriquez	Proportion	2017	24.9	34.7	17.0
Ecuador	Camilo Ponce Enriquez	Count	2000	0	0	0
Ecuador	Camilo Ponce Enriquez	Count	2017	0	0	0
Ecuador	Caluma	Proportion	2000	17.8	26.3	11.3
Ecuador	Caluma	Proportion	2017	27.1	37.0	18.3
Ecuador	Caluma	Count	2000	0	0	0
Ecuador	Caluma	Count	2017	0	0	0
Ecuador	Azogues	Proportion	2000	17.1	24.4	10.8
Ecuador	Azogues	Proportion	2017	24.3	32.7	17.3
Ecuador	Azogues	Count	2000	0	0	0

Table 4: ORS by Unit *(continued)*

Country	Unit	Metric	Year	Mean	Upper	Lower
Ecuador	Azogues	Count	2017	0	0	0
Ecuador	Bolívar	Proportion	2000	16.4	24.8	10.5
Ecuador	Bolívar	Proportion	2017	21.4	29.6	14.3
Ecuador	Bolívar	Count	2000	0	0	0
Ecuador	Bolívar	Count	2017	0	0	0
Ecuador	Alausí	Proportion	2000	15.4	23.5	9.9
Ecuador	Alausí	Proportion	2017	20.3	28.4	13.8
Ecuador	Alausí	Count	2000	0	0	0
Ecuador	Alausí	Count	2017	0	0	0
Ecuador	La Maná	Proportion	2000	17.7	27.9	11.6
Ecuador	La Maná	Proportion	2017	26.5	37.7	18.1
Ecuador	La Maná	Count	2000	0	0	0
Ecuador	La Maná	Count	2017	0	0	0
Ecuador	Arenillas	Proportion	2000	21.3	29.8	14.7
Ecuador	Arenillas	Proportion	2017	31.0	41.4	22.3
Ecuador	Arenillas	Count	2000	0	0	0
Ecuador	Arenillas	Count	2017	0	0	0
Ecuador	Atacames	Proportion	2000	19.6	29.6	12.7
Ecuador	Atacames	Proportion	2017	27.9	38.2	18.9
Ecuador	Atacames	Count	2000	0	0	0
Ecuador	Atacames	Count	2017	0	0	0
Ecuador	Isabela	Proportion	2000	17.0	27.1	10.1
Ecuador	Isabela	Proportion	2017	19.0	27.7	12.0
Ecuador	Isabela	Count	2000	0	0	0
Ecuador	Isabela	Count	2017	0	0	0
Ecuador	Alfredo Baquerizo Moreno	Proportion	2000	19.0	28.0	11.8
Ecuador	Alfredo Baquerizo Moreno	Proportion	2017	28.9	38.0	20.9
Ecuador	Alfredo Baquerizo Moreno	Count	2000	0	0	0
Ecuador	Alfredo Baquerizo Moreno	Count	2017	0	0	0
Ecuador	Antonio Ante	Proportion	2000	15.9	24.6	10.1
Ecuador	Antonio Ante	Proportion	2017	20.8	28.8	14.6
Ecuador	Antonio Ante	Count	2000	0	0	0
Ecuador	Antonio Ante	Count	2017	0	0	0
Ecuador	Calvas	Proportion	2000	18.2	26.6	12.1
Ecuador	Calvas	Proportion	2017	22.2	31.3	14.9
Ecuador	Calvas	Count	2000	0	0	0
Ecuador	Calvas	Count	2017	0	0	0
Ecuador	Baba	Proportion	2000	20.4	30.4	12.8
Ecuador	Baba	Proportion	2017	29.6	40.5	21.7
Ecuador	Baba	Count	2000	0	0	0
Ecuador	Baba	Count	2017	0	0	0
Ecuador	24 De Mayo	Proportion	2000	23.4	35.0	15.3
Ecuador	24 De Mayo	Proportion	2017	29.6	38.6	20.7
Ecuador	24 De Mayo	Count	2000	0	0	0
Ecuador	24 De Mayo	Count	2017	0	0	0
Ecuador	Gualaquiza	Proportion	2000	16.6	24.5	10.9
Ecuador	Gualaquiza	Proportion	2017	21.8	29.8	14.8
Ecuador	Gualaquiza	Count	2000	0	0	0
Ecuador	Gualaquiza	Count	2017	0	0	0

Table 4: ORS by Unit *(continued)*

Country	Unit	Metric	Year	Mean	Upper	Lower
Ecuador	Archidona	Proportion	2000	17.8	27.6	11.0
Ecuador	Archidona	Proportion	2017	28.0	37.9	19.9
Ecuador	Archidona	Count	2000	0	0	0
Ecuador	Archidona	Count	2017	0	0	0
Ecuador	Aguarico	Proportion	2000	23.5	34.0	15.5
Ecuador	Aguarico	Proportion	2017	29.9	41.0	21.0
Ecuador	Aguarico	Count	2000	0	0	0
Ecuador	Aguarico	Count	2017	0	0	0
Ecuador	Arajuno	Proportion	2000	18.6	28.2	11.9
Ecuador	Arajuno	Proportion	2017	26.4	35.6	18.6
Ecuador	Arajuno	Count	2000	0	0	0
Ecuador	Arajuno	Count	2017	0	0	0
Ecuador	Cayambe	Proportion	2000	15.1	23.2	9.3
Ecuador	Cayambe	Proportion	2017	20.1	28.5	14.1
Ecuador	Cayambe	Count	2000	0	0	0
Ecuador	Cayambe	Count	2017	0	0	0
Ecuador	La Libertad	Proportion	2000	21.2	32.4	13.0
Ecuador	La Libertad	Proportion	2017	33.4	43.6	24.1
Ecuador	La Libertad	Count	2000	0	0	0
Ecuador	La Libertad	Count	2017	0	0	0
Ecuador	La Concordia	Proportion	2000	19.6	29.9	12.2
Ecuador	La Concordia	Proportion	2017	29.5	41.5	19.8
Ecuador	La Concordia	Count	2000	0	0	0
Ecuador	La Concordia	Count	2017	0	0	0
Ecuador	Cascales	Proportion	2000	21.2	30.5	13.6
Ecuador	Cascales	Proportion	2017	27.9	37.3	19.4
Ecuador	Cascales	Count	2000	0	0	0
Ecuador	Cascales	Count	2017	0	0	0
Ecuador	Ambato	Proportion	2000	15.3	23.9	9.9
Ecuador	Ambato	Proportion	2017	22.5	32.5	15.3
Ecuador	Ambato	Count	2000	0	0	0
Ecuador	Ambato	Count	2017	0	0	0
Ecuador	Centinela del Cóndor	Proportion	2000	17.0	25.1	10.6
Ecuador	Centinela del Cóndor	Proportion	2017	20.9	28.8	14.0
Ecuador	Centinela del Cóndor	Count	2000	0	0	0
Ecuador	Centinela del Cóndor	Count	2017	0	0	0
Ecuador	Chordeleg	Proportion	2000	15.1	22.1	9.3
Ecuador	Chordeleg	Proportion	2017	20.3	28.1	14.4
Ecuador	Chordeleg	Count	2000	0	0	0
Ecuador	Chordeleg	Count	2017	0	0	0
Ecuador	Chillanes	Proportion	2000	17.9	26.4	11.1
Ecuador	Chillanes	Proportion	2017	24.2	33.2	16.5
Ecuador	Chillanes	Count	2000	0	0	0
Ecuador	Chillanes	Count	2017	0	0	0
Ecuador	Biblián	Proportion	2000	17.5	24.8	11.3
Ecuador	Biblián	Proportion	2017	22.5	30.7	15.7
Ecuador	Biblián	Count	2000	0	0	0
Ecuador	Biblián	Count	2017	0	0	0
Ecuador	Espejo	Proportion	2000	16.2	24.1	10.5

Table 4: ORS by Unit *(continued)*

Country	Unit	Metric	Year	Mean	Upper	Lower
Ecuador	Espejo	Proportion	2017	20.7	28.9	14.1
Ecuador	Espejo	Count	2000	0	0	0
Ecuador	Espejo	Count	2017	0	0	0
Ecuador	Chambo	Proportion	2000	15.5	23.6	10.0
Ecuador	Chambo	Proportion	2017	22.0	31.6	14.9
Ecuador	Chambo	Count	2000	0	0	0
Ecuador	Chambo	Count	2017	0	0	0
Ecuador	Latacunga	Proportion	2000	14.8	24.0	9.2
Ecuador	Latacunga	Proportion	2017	21.0	29.9	14.2
Ecuador	Latacunga	Count	2000	0	0	0
Ecuador	Latacunga	Count	2017	0	0	0
Ecuador	Atahualpa	Proportion	2000	18.4	26.8	11.8
Ecuador	Atahualpa	Proportion	2017	24.7	33.7	17.1
Ecuador	Atahualpa	Count	2000	0	0	0
Ecuador	Atahualpa	Count	2017	0	0	0
Ecuador	Eloy Alfaro	Proportion	2000	19.0	27.7	12.2
Ecuador	Eloy Alfaro	Proportion	2017	24.8	35.6	17.0
Ecuador	Eloy Alfaro	Count	2000	0	0	0
Ecuador	Eloy Alfaro	Count	2017	0	0	0
Ecuador	San Cristóbal	Proportion	2000	22.1	32.5	14.3
Ecuador	San Cristóbal	Proportion	2017	26.0	37.6	16.5
Ecuador	San Cristóbal	Count	2000	0	0	0
Ecuador	San Cristóbal	Count	2017	0	0	0
Ecuador	Balao	Proportion	2000	20.7	30.2	13.9
Ecuador	Balao	Proportion	2017	29.1	39.9	20.5
Ecuador	Balao	Count	2000	0	0	0
Ecuador	Balao	Count	2017	0	0	0
Ecuador	Cotacachi	Proportion	2000	17.0	25.8	11.0
Ecuador	Cotacachi	Proportion	2017	21.6	29.6	15.3
Ecuador	Cotacachi	Count	2000	0	0	0
Ecuador	Cotacachi	Count	2017	0	0	0
Ecuador	Catamayo	Proportion	2000	21.7	30.8	14.1
Ecuador	Catamayo	Proportion	2017	26.7	36.6	18.3
Ecuador	Catamayo	Count	2000	0	0	0
Ecuador	Catamayo	Count	2017	0	0	0
Ecuador	Babahoyo	Proportion	2000	20.1	30.0	12.7
Ecuador	Babahoyo	Proportion	2017	29.5	39.0	21.3
Ecuador	Babahoyo	Count	2000	0	0	0
Ecuador	Babahoyo	Count	2017	0	0	0
Ecuador	Bolívar	Proportion	2000	21.0	31.0	13.5
Ecuador	Bolívar	Proportion	2017	30.4	40.5	20.9
Ecuador	Bolívar	Count	2000	0	0	0
Ecuador	Bolívar	Count	2017	0	0	0
Ecuador	Huamboya	Proportion	2000	14.8	23.1	9.7
Ecuador	Huamboya	Proportion	2017	20.3	28.3	13.4
Ecuador	Huamboya	Count	2000	0	0	0
Ecuador	Huamboya	Count	2017	0	0	0
Ecuador	Carlos Julio Arosemena Tola	Proportion	2000	18.5	29.1	12.0

Table 4: ORS by Unit *(continued)*

Country	Unit	Metric	Year	Mean	Upper	Lower
Ecuador	Carlos Julio Arosemena Tola	Proportion	2017	24.9	34.8	17.4
Ecuador	Carlos Julio Arosemena Tola	Count	2000	0	0	0
Ecuador	Carlos Julio Arosemena Tola	Count	2017	0	0	0
Ecuador	La Joya de los Sachas	Proportion	2000	19.3	29.7	12.5
Ecuador	La Joya de los Sachas	Proportion	2017	26.8	37.2	17.7
Ecuador	La Joya de los Sachas	Count	2000	0	0	0
Ecuador	La Joya de los Sachas	Count	2017	0	0	0
Ecuador	Mejía	Proportion	2000	14.3	22.8	9.4
Ecuador	Mejía	Proportion	2017	19.9	27.9	13.6
Ecuador	Mejía	Count	2000	0	0	0
Ecuador	Mejía	Count	2017	0	0	0
Ecuador	Mejía	Proportion	2000	15.0	24.4	9.3
Ecuador	Mejía	Proportion	2017	22.3	31.4	14.8
Ecuador	Mejía	Count	2000	0	0	0
Ecuador	Mejía	Count	2017	0	0	0
Ecuador	Salinas	Proportion	2000	24.4	35.9	15.4
Ecuador	Salinas	Proportion	2017	39.4	50.1	29.1
Ecuador	Salinas	Count	2000	0	0	0
Ecuador	Salinas	Count	2017	0	1	0
Ecuador	Santo Domingo	Proportion	2000	17.4	27.3	11.0
Ecuador	Santo Domingo	Proportion	2017	27.4	39.2	18.5
Ecuador	Santo Domingo	Count	2000	0	0	0
Ecuador	Santo Domingo	Count	2017	0	0	0
Ecuador	Cuyabeno	Proportion	2000	22.3	33.0	14.6
Ecuador	Cuyabeno	Proportion	2017	28.6	41.0	18.6
Ecuador	Cuyabeno	Count	2000	0	0	0
Ecuador	Cuyabeno	Count	2017	0	0	0
Ecuador	Baños de Agua Santa	Proportion	2000	15.5	24.4	10.0
Ecuador	Baños de Agua Santa	Proportion	2017	22.1	31.9	15.2
Ecuador	Baños de Agua Santa	Count	2000	0	0	0
Ecuador	Baños de Agua Santa	Count	2017	0	0	0
Ecuador	Chinchipe	Proportion	2000	19.7	28.9	12.8
Ecuador	Chinchipe	Proportion	2017	22.7	30.9	15.4
Ecuador	Chinchipe	Count	2000	0	0	0
Ecuador	Chinchipe	Count	2017	0	0	0
Ecuador	Cuenca	Proportion	2000	14.4	21.0	8.9
Ecuador	Cuenca	Proportion	2017	21.9	30.0	15.4
Ecuador	Cuenca	Count	2000	0	0	0
Ecuador	Cuenca	Count	2017	0	0	0
Ecuador	Chimbo	Proportion	2000	15.9	23.6	9.9
Ecuador	Chimbo	Proportion	2017	24.1	33.3	15.9
Ecuador	Chimbo	Count	2000	0	0	0
Ecuador	Chimbo	Count	2017	0	0	0
Ecuador	Cañar	Proportion	2000	16.8	24.5	10.8
Ecuador	Cañar	Proportion	2017	21.6	30.4	15.0
Ecuador	Cañar	Count	2000	0	0	0
Ecuador	Cañar	Count	2017	0	0	0

Table 4: ORS by Unit *(continued)*

Country	Unit	Metric	Year	Mean	Upper	Lower
Ecuador	Mira	Proportion	2000	17.2	25.6	11.0
Ecuador	Mira	Proportion	2017	22.8	30.7	15.8
Ecuador	Mira	Count	2000	0	0	0
Ecuador	Mira	Count	2017	0	0	0
Ecuador	Chunchi	Proportion	2000	15.5	23.6	9.9
Ecuador	Chunchi	Proportion	2017	19.4	27.4	13.3
Ecuador	Chunchi	Count	2000	0	0	0
Ecuador	Chunchi	Count	2017	0	0	0
Ecuador	Pangua	Proportion	2000	17.4	27.1	11.3
Ecuador	Pangua	Proportion	2017	24.6	34.4	16.5
Ecuador	Pangua	Count	2000	0	0	0
Ecuador	Pangua	Count	2017	0	0	0
Ecuador	Balsas	Proportion	2000	20.1	28.4	13.2
Ecuador	Balsas	Proportion	2017	25.5	34.5	17.5
Ecuador	Balsas	Count	2000	0	0	0
Ecuador	Balsas	Count	2017	0	0	0
Ecuador	Esmeraldas	Proportion	2000	22.6	32.7	14.8
Ecuador	Esmeraldas	Proportion	2017	31.9	42.4	22.5
Ecuador	Esmeraldas	Count	2000	0	0	0
Ecuador	Esmeraldas	Count	2017	0	0	0
Ecuador	Santa Cruz	Proportion	2000	19.9	30.4	12.2
Ecuador	Santa Cruz	Proportion	2017	27.1	37.6	17.9
Ecuador	Santa Cruz	Count	2000	0	0	0
Ecuador	Santa Cruz	Count	2017	0	0	0
Ecuador	Balzar	Proportion	2000	23.8	35.9	15.6
Ecuador	Balzar	Proportion	2017	30.6	41.2	21.9
Ecuador	Balzar	Count	2000	0	0	0
Ecuador	Balzar	Count	2017	0	0	0
Ecuador	Ibarra	Proportion	2000	16.9	25.9	11.0
Ecuador	Ibarra	Proportion	2017	22.5	30.9	15.8
Ecuador	Ibarra	Count	2000	0	0	0
Ecuador	Ibarra	Count	2017	0	0	0
Ecuador	Celica	Proportion	2000	20.8	29.7	13.7
Ecuador	Celica	Proportion	2017	25.2	35.9	18.1
Ecuador	Celica	Count	2000	0	0	0
Ecuador	Celica	Count	2017	0	0	0
Ecuador	Buena Fé	Proportion	2000	19.9	30.4	13.0
Ecuador	Buena Fé	Proportion	2017	28.4	38.9	19.9
Ecuador	Buena Fé	Count	2000	0	0	0
Ecuador	Buena Fé	Count	2017	0	0	0
Ecuador	Chone	Proportion	2000	20.4	31.5	13.2
Ecuador	Chone	Proportion	2017	28.5	39.5	19.9
Ecuador	Chone	Count	2000	0	0	0
Ecuador	Chone	Count	2017	0	0	0
Ecuador	Limón Indanza	Proportion	2000	17.2	24.6	11.1
Ecuador	Limón Indanza	Proportion	2017	22.4	31.2	15.9
Ecuador	Limón Indanza	Count	2000	0	0	0
Ecuador	Limón Indanza	Count	2017	0	0	0
Ecuador	El Chaco	Proportion	2000	17.0	26.1	10.3

Table 4: ORS by Unit *(continued)*

Country	Unit	Metric	Year	Mean	Upper	Lower
Ecuador	El Chaco	Proportion	2017	24.1	33.3	16.1
Ecuador	El Chaco	Count	2000	0	0	0
Ecuador	El Chaco	Count	2017	0	0	0
Ecuador	Loreto	Proportion	2000	17.4	26.5	10.7
Ecuador	Loreto	Proportion	2017	25.0	33.8	17.1
Ecuador	Loreto	Count	2000	0	0	0
Ecuador	Loreto	Count	2017	0	0	0
Ecuador	Mera	Proportion	2000	13.4	21.4	8.8
Ecuador	Mera	Proportion	2017	19.5	26.8	13.0
Ecuador	Mera	Count	2000	0	0	0
Ecuador	Mera	Count	2017	0	0	0
Ecuador	Pedro Moncayo	Proportion	2000	16.1	24.7	10.1
Ecuador	Pedro Moncayo	Proportion	2017	20.9	29.4	14.8
Ecuador	Pedro Moncayo	Count	2000	0	0	0
Ecuador	Pedro Moncayo	Count	2017	0	0	0
Ecuador	Santa Elena	Proportion	2000	23.0	33.4	14.6
Ecuador	Santa Elena	Proportion	2017	33.2	43.9	23.9
Ecuador	Santa Elena	Count	2000	0	0	0
Ecuador	Santa Elena	Count	2017	0	0	0
Ecuador	Gonzalo Pizarro	Proportion	2000	19.0	27.5	11.8
Ecuador	Gonzalo Pizarro	Proportion	2017	26.4	35.3	18.1
Ecuador	Gonzalo Pizarro	Count	2000	0	0	0
Ecuador	Gonzalo Pizarro	Count	2017	0	0	0
Ecuador	Cevallos	Proportion	2000	15.1	22.9	9.9
Ecuador	Cevallos	Proportion	2017	22.8	32.9	15.6
Ecuador	Cevallos	Count	2000	0	0	0
Ecuador	Cevallos	Count	2017	0	0	0
Ecuador	El Pangui	Proportion	2000	17.3	26.0	11.1
Ecuador	El Pangui	Proportion	2017	22.3	30.6	15.2
Ecuador	El Pangui	Count	2000	0	0	0
Ecuador	El Pangui	Count	2017	0	0	0
Ecuador	El Pan	Proportion	2000	16.9	24.0	10.6
Ecuador	El Pan	Proportion	2017	19.5	27.0	13.8
Ecuador	El Pan	Count	2000	0	0	0
Ecuador	El Pan	Count	2017	0	0	0
Ecuador	Echeandía	Proportion	2000	17.1	26.0	10.9
Ecuador	Echeandía	Proportion	2017	26.1	35.8	17.5
Ecuador	Echeandía	Count	2000	0	0	0
Ecuador	Echeandía	Count	2017	0	0	0
Ecuador	Déleg	Proportion	2000	16.0	22.8	10.2
Ecuador	Déleg	Proportion	2017	20.3	28.0	14.1
Ecuador	Déleg	Count	2000	0	0	0
Ecuador	Déleg	Count	2017	0	0	0
Ecuador	Montúfar	Proportion	2000	14.6	22.1	9.1
Ecuador	Montúfar	Proportion	2017	19.5	27.4	12.9
Ecuador	Montúfar	Count	2000	0	0	0
Ecuador	Montúfar	Count	2017	0	0	0
Ecuador	Colta	Proportion	2000	15.4	23.4	9.7
Ecuador	Colta	Proportion	2017	20.8	29.7	13.8

Table 4: ORS by Unit *(continued)*

Country	Unit	Metric	Year	Mean	Upper	Lower
Ecuador	Colta	Count	2000	0	0	0
Ecuador	Colta	Count	2017	0	0	0
Ecuador	Pujilí	Proportion	2000	14.6	23.6	9.2
Ecuador	Pujilí	Proportion	2017	20.8	29.9	13.9
Ecuador	Pujilí	Count	2000	0	0	0
Ecuador	Pujilí	Count	2017	0	0	0
Ecuador	Chilla	Proportion	2000	17.4	25.2	11.2
Ecuador	Chilla	Proportion	2017	22.1	31.1	15.2
Ecuador	Chilla	Count	2000	0	0	0
Ecuador	Chilla	Count	2017	0	0	0
Ecuador	Muisne	Proportion	2000	20.8	32.2	13.3
Ecuador	Muisne	Proportion	2017	25.0	35.5	17.1
Ecuador	Muisne	Count	2000	0	0	0
Ecuador	Muisne	Count	2017	0	0	0
Ecuador	Colimes	Proportion	2000	24.6	36.9	16.0
Ecuador	Colimes	Proportion	2017	31.8	43.0	22.8
Ecuador	Colimes	Count	2000	0	0	0
Ecuador	Colimes	Count	2017	0	0	0
Ecuador	Otavalo	Proportion	2000	15.6	23.9	9.8
Ecuador	Otavalo	Proportion	2017	19.6	27.2	13.3
Ecuador	Otavalo	Count	2000	0	0	0
Ecuador	Otavalo	Count	2017	0	0	0
Ecuador	Chaguarpamba	Proportion	2000	19.4	27.5	12.7
Ecuador	Chaguarpamba	Proportion	2017	24.0	32.9	16.2
Ecuador	Chaguarpamba	Count	2000	0	0	0
Ecuador	Chaguarpamba	Count	2017	0	0	0
Ecuador	Mocache	Proportion	2000	21.1	32.2	13.9
Ecuador	Mocache	Proportion	2017	28.6	38.9	19.7
Ecuador	Mocache	Count	2000	0	0	0
Ecuador	Mocache	Count	2017	0	0	0
Ecuador	El Carmen	Proportion	2000	18.4	27.4	11.7
Ecuador	El Carmen	Proportion	2017	27.8	39.4	19.2
Ecuador	El Carmen	Count	2000	0	0	0
Ecuador	El Carmen	Count	2017	0	0	0
Ecuador	Logroño	Proportion	2000	17.8	26.0	11.4
Ecuador	Logroño	Proportion	2017	22.9	31.8	16.0
Ecuador	Logroño	Count	2000	0	0	0
Ecuador	Logroño	Count	2017	0	0	0
Ecuador	Quijos	Proportion	2000	16.3	25.3	9.6
Ecuador	Quijos	Proportion	2017	23.3	31.9	16.0
Ecuador	Quijos	Count	2000	0	0	0
Ecuador	Quijos	Count	2017	0	0	0
Ecuador	Orellana	Proportion	2000	19.3	29.8	12.7
Ecuador	Orellana	Proportion	2017	27.7	38.0	18.6
Ecuador	Orellana	Count	2000	0	0	0
Ecuador	Orellana	Count	2017	0	0	0
Ecuador	Pastaza	Proportion	2000	16.3	25.1	11.1
Ecuador	Pastaza	Proportion	2017	23.9	32.1	16.6
Ecuador	Pastaza	Count	2000	0	0	0

Table 4: ORS by Unit *(continued)*

Country	Unit	Metric	Year	Mean	Upper	Lower
Ecuador	Pastaza	Count	2017	0	0	0
Ecuador	Pedro Vicente Maldonado	Proportion	2000	21.1	31.7	12.7
Ecuador	Pedro Vicente Maldonado	Proportion	2017	28.5	40.8	18.7
Ecuador	Pedro Vicente Maldonado	Count	2000	0	0	0
Ecuador	Pedro Vicente Maldonado	Count	2017	0	0	0
Ecuador	Lago Agrio	Proportion	2000	19.3	28.9	12.0
Ecuador	Lago Agrio	Proportion	2017	25.4	34.4	17.0
Ecuador	Lago Agrio	Count	2000	0	0	0
Ecuador	Lago Agrio	Count	2017	0	0	0
Ecuador	Mocha	Proportion	2000	14.6	22.7	9.6
Ecuador	Mocha	Proportion	2017	21.8	31.9	14.8
Ecuador	Mocha	Count	2000	0	0	0
Ecuador	Mocha	Count	2017	0	0	0
Ecuador	Nangaritza	Proportion	2000	17.5	26.0	11.3
Ecuador	Nangaritza	Proportion	2017	20.7	27.8	13.9
Ecuador	Nangaritza	Count	2000	0	0	0
Ecuador	Nangaritza	Count	2017	0	0	0
Ecuador	Girón	Proportion	2000	15.6	23.6	9.9
Ecuador	Girón	Proportion	2017	20.3	28.1	13.8
Ecuador	Girón	Count	2000	0	0	0
Ecuador	Girón	Count	2017	0	0	0
Ecuador	Guaranda	Proportion	2000	15.3	23.8	9.9
Ecuador	Guaranda	Proportion	2017	21.7	30.6	14.4
Ecuador	Guaranda	Count	2000	0	0	0
Ecuador	Guaranda	Count	2017	0	0	0
Ecuador	El Tambo	Proportion	2000	18.0	26.1	11.8
Ecuador	El Tambo	Proportion	2017	21.3	30.1	14.8
Ecuador	El Tambo	Count	2000	0	0	0
Ecuador	El Tambo	Count	2017	0	0	0
Ecuador	San Pedro de Huaca	Proportion	2000	15.7	23.2	9.7
Ecuador	San Pedro de Huaca	Proportion	2017	20.1	28.4	13.4
Ecuador	San Pedro de Huaca	Count	2000	0	0	0
Ecuador	San Pedro de Huaca	Count	2017	0	0	0
Ecuador	Cumanda	Proportion	2000	18.8	27.7	12.1
Ecuador	Cumanda	Proportion	2017	25.5	34.5	17.7
Ecuador	Cumanda	Count	2000	0	0	0
Ecuador	Cumanda	Count	2017	0	0	0
Ecuador	Salcedo	Proportion	2000	15.4	24.8	9.9
Ecuador	Salcedo	Proportion	2017	21.7	30.8	14.7
Ecuador	Salcedo	Count	2000	0	0	0
Ecuador	Salcedo	Count	2017	0	0	0
Ecuador	El Guabo	Proportion	2000	18.2	26.8	11.7
Ecuador	El Guabo	Proportion	2017	26.8	37.0	18.5
Ecuador	El Guabo	Count	2000	0	0	0
Ecuador	El Guabo	Count	2017	0	0	0
Ecuador	Quinindé	Proportion	2000	20.6	31.4	13.4
Ecuador	Quinindé	Proportion	2017	29.4	40.7	20.6
Ecuador	Quinindé	Count	2000	0	0	0
Ecuador	Quinindé	Count	2017	0	0	0

Table 4: ORS by Unit *(continued)*

Country	Unit	Metric	Year	Mean	Upper	Lower
Ecuador	Coronel Marcelino Maridueña	Proportion	2000	18.7	27.2	12.0
Ecuador	Coronel Marcelino Maridueña	Proportion	2017	27.8	36.8	19.5
Ecuador	Coronel Marcelino Maridueña	Count	2000	0	0	0
Ecuador	Coronel Marcelino Maridueña	Count	2017	0	0	0
Ecuador	Pimampiro	Proportion	2000	18.7	27.6	12.2
Ecuador	Pimampiro	Proportion	2017	25.0	33.7	17.1
Ecuador	Pimampiro	Count	2000	0	0	0
Ecuador	Pimampiro	Count	2017	0	0	0
Ecuador	Espíndola	Proportion	2000	18.5	27.0	12.1
Ecuador	Espíndola	Proportion	2017	21.3	29.9	14.5
Ecuador	Espíndola	Count	2000	0	0	0
Ecuador	Espíndola	Count	2017	0	0	0
Ecuador	Montalvo	Proportion	2000	21.0	30.6	13.4
Ecuador	Montalvo	Proportion	2017	31.6	41.8	22.8
Ecuador	Montalvo	Count	2000	0	0	0
Ecuador	Montalvo	Count	2017	0	0	0
Ecuador	Flavio Alfaro	Proportion	2000	21.3	31.3	13.7
Ecuador	Flavio Alfaro	Proportion	2017	27.2	38.0	18.7
Ecuador	Flavio Alfaro	Count	2000	0	0	0
Ecuador	Flavio Alfaro	Count	2017	0	0	0
Ecuador	Morona	Proportion	2000	16.3	25.0	10.5
Ecuador	Morona	Proportion	2017	22.4	30.7	15.2
Ecuador	Morona	Count	2000	0	0	0
Ecuador	Morona	Count	2017	0	0	0
Ecuador	Tena	Proportion	2000	18.8	28.7	11.6
Ecuador	Tena	Proportion	2017	29.0	38.9	20.9
Ecuador	Tena	Count	2000	0	0	0
Ecuador	Tena	Count	2017	0	0	0
Ecuador	Santa Clara	Proportion	2000	16.3	26.0	10.5
Ecuador	Santa Clara	Proportion	2017	23.4	32.4	16.4
Ecuador	Santa Clara	Count	2000	0	0	0
Ecuador	Santa Clara	Count	2017	0	0	0
Ecuador	Puerto Quito	Proportion	2000	21.3	32.5	13.6
Ecuador	Puerto Quito	Proportion	2017	29.9	42.3	20.4
Ecuador	Puerto Quito	Count	2000	0	0	0
Ecuador	Puerto Quito	Count	2017	0	0	0
Ecuador	Putumayo	Proportion	2000	21.6	31.6	13.9
Ecuador	Putumayo	Proportion	2017	28.5	40.6	19.3
Ecuador	Putumayo	Count	2000	0	0	0
Ecuador	Putumayo	Count	2017	0	0	0
Ecuador	Patate	Proportion	2000	14.1	21.9	9.1
Ecuador	Patate	Proportion	2017	21.4	31.3	14.6
Ecuador	Patate	Count	2000	0	0	0
Ecuador	Patate	Count	2017	0	0	0
Ecuador	Palanda	Proportion	2000	18.7	28.3	12.1

Table 4: ORS by Unit *(continued)*

Country	Unit	Metric	Year	Mean	Upper	Lower
Ecuador	Palanda	Proportion	2017	21.9	30.7	14.3
Ecuador	Palanda	Count	2000	0	0	0
Ecuador	Palanda	Count	2017	0	0	0
Ecuador	Guachapala	Proportion	2000	17.5	24.9	11.1
Ecuador	Guachapala	Proportion	2017	20.6	28.3	14.6
Ecuador	Guachapala	Count	2000	0	0	0
Ecuador	Guachapala	Count	2017	0	0	0
Ecuador	Las Naves	Proportion	2000	18.7	28.5	12.0
Ecuador	Las Naves	Proportion	2017	25.9	35.5	17.6
Ecuador	Las Naves	Count	2000	0	0	0
Ecuador	Las Naves	Count	2017	0	0	0
Ecuador	La Troncal	Proportion	2000	18.2	26.6	11.5
Ecuador	La Troncal	Proportion	2017	27.6	37.6	19.0
Ecuador	La Troncal	Count	2000	0	0	0
Ecuador	La Troncal	Count	2017	0	0	0
Ecuador	Tulcán	Proportion	2000	17.3	25.5	10.9
Ecuador	Tulcán	Proportion	2017	20.6	29.2	14.3
Ecuador	Tulcán	Count	2000	0	0	0
Ecuador	Tulcán	Count	2017	0	0	0
Ecuador	Guamote	Proportion	2000	14.8	22.9	9.4
Ecuador	Guamote	Proportion	2017	20.3	28.8	13.6
Ecuador	Guamote	Count	2000	0	0	0
Ecuador	Guamote	Count	2017	0	0	0
Ecuador	Saquisilí	Proportion	2000	14.5	23.7	9.0
Ecuador	Saquisilí	Proportion	2017	20.2	29.2	13.4
Ecuador	Saquisilí	Count	2000	0	0	0
Ecuador	Saquisilí	Count	2017	0	0	0
Ecuador	Huaquillas	Proportion	2000	26.8	36.4	19.3
Ecuador	Huaquillas	Proportion	2017	40.8	51.8	30.8
Ecuador	Huaquillas	Count	2000	0	0	0
Ecuador	Huaquillas	Count	2017	0	1	0
Ecuador	Río Verde	Proportion	2000	19.0	28.3	12.1
Ecuador	Río Verde	Proportion	2017	25.3	35.7	16.7
Ecuador	Río Verde	Count	2000	0	0	0
Ecuador	Río Verde	Count	2017	0	0	0
Ecuador	Daule	Proportion	2000	20.7	30.2	13.2
Ecuador	Daule	Proportion	2017	32.6	42.9	24.0
Ecuador	Daule	Count	2000	0	0	0
Ecuador	Daule	Count	2017	0	0	0
Ecuador	San Miguel de Urcuquí	Proportion	2000	16.8	26.1	10.9
Ecuador	San Miguel de Urcuquí	Proportion	2017	21.1	28.7	14.7
Ecuador	San Miguel de Urcuquí	Count	2000	0	0	0
Ecuador	San Miguel de Urcuquí	Count	2017	0	0	0
Ecuador	Gonzanamá	Proportion	2000	19.9	28.8	13.4
Ecuador	Gonzanamá	Proportion	2017	24.0	34.2	16.2
Ecuador	Gonzanamá	Count	2000	0	0	0
Ecuador	Gonzanamá	Count	2017	0	0	0
Ecuador	Palenque	Proportion	2000	24.8	36.1	16.4
Ecuador	Palenque	Proportion	2017	31.0	42.0	22.3

Table 4: ORS by Unit *(continued)*

Country	Unit	Metric	Year	Mean	Upper	Lower
Ecuador	Palenque	Count	2000	0	0	0
Ecuador	Palenque	Count	2017	0	0	0
Ecuador	Jama	Proportion	2000	20.1	30.8	12.4
Ecuador	Jama	Proportion	2017	29.5	40.7	19.7
Ecuador	Jama	Count	2000	0	0	0
Ecuador	Jama	Count	2017	0	0	0
Ecuador	Pablo Sexto	Proportion	2000	16.8	25.4	11.3
Ecuador	Pablo Sexto	Proportion	2017	21.3	30.0	14.2
Ecuador	Pablo Sexto	Count	2000	0	0	0
Ecuador	Pablo Sexto	Count	2017	0	0	0
Ecuador	Quito	Proportion	2000	17.3	26.5	10.9
Ecuador	Quito	Proportion	2017	23.6	32.5	16.2
Ecuador	Quito	Count	2000	0	0	0
Ecuador	Quito	Count	2017	0	0	0
Ecuador	Shushufindi	Proportion	2000	20.7	31.2	13.6
Ecuador	Shushufindi	Proportion	2017	27.8	39.1	18.1
Ecuador	Shushufindi	Count	2000	0	0	0
Ecuador	Shushufindi	Count	2017	0	0	0
Ecuador	Quero	Proportion	2000	14.7	22.6	9.7
Ecuador	Quero	Proportion	2017	21.0	30.5	14.2
Ecuador	Quero	Count	2000	0	0	0
Ecuador	Quero	Count	2017	0	0	0
Ecuador	Paquisha	Proportion	2000	17.4	26.3	11.1
Ecuador	Paquisha	Proportion	2017	21.2	29.1	14.3
Ecuador	Paquisha	Count	2000	0	0	0
Ecuador	Paquisha	Count	2017	0	0	0
Ecuador	Gualaceo	Proportion	2000	15.4	22.4	9.5
Ecuador	Gualaceo	Proportion	2017	20.9	28.8	14.8
Ecuador	Gualaceo	Count	2000	0	0	0
Ecuador	Gualaceo	Count	2017	0	0	0
Ecuador	San Miguel	Proportion	2000	15.8	23.7	9.9
Ecuador	San Miguel	Proportion	2017	23.1	32.2	15.4
Ecuador	San Miguel	Count	2000	0	0	0
Ecuador	San Miguel	Count	2017	0	0	0
Ecuador	Suscal	Proportion	2000	17.8	25.7	11.6
Ecuador	Suscal	Proportion	2017	21.8	30.5	15.0
Ecuador	Suscal	Count	2000	0	0	0
Ecuador	Suscal	Count	2017	0	0	0
Ecuador	Guano	Proportion	2000	15.7	24.1	10.1
Ecuador	Guano	Proportion	2017	23.7	33.7	16.2
Ecuador	Guano	Count	2000	0	0	0
Ecuador	Guano	Count	2017	0	0	0
Ecuador	Saquisili	Proportion	2000	14.0	22.8	8.5
Ecuador	Saquisili	Proportion	2017	20.7	29.4	13.9
Ecuador	Saquisili	Count	2000	0	0	0
Ecuador	Saquisili	Count	2017	0	0	0
Ecuador	Las Lajas	Proportion	2000	20.4	28.8	13.4
Ecuador	Las Lajas	Proportion	2017	26.6	35.3	19.0
Ecuador	Las Lajas	Count	2000	0	0	0

Table 4: ORS by Unit *(continued)*

Country	Unit	Metric	Year	Mean	Upper	Lower
Ecuador	Las Lajas	Count	2017	0	0	0
Ecuador	San Lorenzo	Proportion	2000	18.8	28.4	12.0
Ecuador	San Lorenzo	Proportion	2017	25.3	35.5	17.2
Ecuador	San Lorenzo	Count	2000	0	0	0
Ecuador	San Lorenzo	Count	2017	0	0	0
Ecuador	Durán	Proportion	2000	22.4	32.9	14.5
Ecuador	Durán	Proportion	2017	32.1	42.2	23.2
Ecuador	Durán	Count	2000	0	0	0
Ecuador	Durán	Count	2017	0	0	0
Ecuador	Loja	Proportion	2000	19.0	27.3	12.3
Ecuador	Loja	Proportion	2017	22.1	30.5	14.8
Ecuador	Loja	Count	2000	0	0	0
Ecuador	Loja	Count	2017	0	0	0
Ecuador	Pueblo Viejo	Proportion	2000	20.8	31.2	13.3
Ecuador	Pueblo Viejo	Proportion	2017	31.3	42.0	22.9
Ecuador	Pueblo Viejo	Count	2000	0	0	0
Ecuador	Pueblo Viejo	Count	2017	0	0	0
Ecuador	Jaramijó	Proportion	2000	21.1	30.4	13.4
Ecuador	Jaramijó	Proportion	2017	31.8	41.7	22.2
Ecuador	Jaramijó	Count	2000	0	0	0
Ecuador	Jaramijó	Count	2017	0	0	0
Ecuador	Palora	Proportion	2000	16.3	25.1	11.2
Ecuador	Palora	Proportion	2017	22.6	30.7	15.1
Ecuador	Palora	Count	2000	0	0	0
Ecuador	Palora	Count	2017	0	0	0
Ecuador	Rumiñahui	Proportion	2000	17.8	27.4	11.1
Ecuador	Rumiñahui	Proportion	2017	25.6	35.4	17.6
Ecuador	Rumiñahui	Count	2000	0	0	0
Ecuador	Rumiñahui	Count	2017	0	0	0
Ecuador	Sucumbíos	Proportion	2000	18.1	26.5	11.3
Ecuador	Sucumbíos	Proportion	2017	21.3	29.7	14.1
Ecuador	Sucumbíos	Count	2000	0	0	0
Ecuador	Sucumbíos	Count	2017	0	0	0
Ecuador	San Pedro de Pelileo	Proportion	2000	14.3	22.1	9.3
Ecuador	San Pedro de Pelileo	Proportion	2017	22.4	32.4	15.3
Ecuador	San Pedro de Pelileo	Count	2000	0	0	0
Ecuador	San Pedro de Pelileo	Count	2017	0	0	0
Ecuador	Yacuambi	Proportion	2000	16.9	24.5	10.4
Ecuador	Yacuambi	Proportion	2017	20.7	28.4	13.9
Ecuador	Yacuambi	Count	2000	0	0	0
Ecuador	Yacuambi	Count	2017	0	0	0
Ecuador	Nabón	Proportion	2000	17.0	24.9	10.9
Ecuador	Nabón	Proportion	2017	20.4	27.6	13.9
Ecuador	Nabón	Count	2000	0	0	0
Ecuador	Nabón	Count	2017	0	0	0
Ecuador	Pallatanga	Proportion	2000	17.6	26.4	11.1
Ecuador	Pallatanga	Proportion	2017	23.1	31.7	15.5
Ecuador	Pallatanga	Count	2000	0	0	0
Ecuador	Pallatanga	Count	2017	0	0	0

Table 4: ORS by Unit *(continued)*

Country	Unit	Metric	Year	Mean	Upper	Lower
Ecuador	Sigchos	Proportion	2000	14.1	22.8	8.7
Ecuador	Sigchos	Proportion	2017	20.0	29.2	13.0
Ecuador	Sigchos	Count	2000	0	0	0
Ecuador	Sigchos	Count	2017	0	0	0
Ecuador	Machala	Proportion	2000	21.2	30.0	14.4
Ecuador	Machala	Proportion	2017	30.9	41.6	22.2
Ecuador	Machala	Count	2000	0	0	0
Ecuador	Machala	Count	2017	0	0	0
Ecuador	El Empalme	Proportion	2000	19.9	29.7	13.0
Ecuador	El Empalme	Proportion	2017	29.6	39.6	20.3
Ecuador	El Empalme	Count	2000	0	0	0
Ecuador	El Empalme	Count	2017	0	0	0
Ecuador	Macará	Proportion	2000	23.4	31.8	16.8
Ecuador	Macará	Proportion	2017	27.1	35.1	20.8
Ecuador	Macará	Count	2000	0	0	0
Ecuador	Macará	Count	2017	0	0	0
Ecuador	Quevedo	Proportion	2000	17.2	26.1	10.8
Ecuador	Quevedo	Proportion	2017	29.7	40.6	20.3
Ecuador	Quevedo	Count	2000	0	0	0
Ecuador	Quevedo	Count	2017	0	0	0
Ecuador	Jipijapa	Proportion	2000	21.6	31.7	13.9
Ecuador	Jipijapa	Proportion	2017	30.0	38.9	21.2
Ecuador	Jipijapa	Count	2000	0	0	0
Ecuador	Jipijapa	Count	2017	0	0	0
Ecuador	San Juan Bosco	Proportion	2000	15.7	22.8	10.3
Ecuador	San Juan Bosco	Proportion	2017	21.0	29.5	14.9
Ecuador	San Juan Bosco	Count	2000	0	0	0
Ecuador	San Juan Bosco	Count	2017	0	0	0
Ecuador	San Miguel de los Bancos	Proportion	2000	19.6	30.2	12.0
Ecuador	San Miguel de los Bancos	Proportion	2017	27.7	39.0	18.7
Ecuador	San Miguel de los Bancos	Count	2000	0	0	0
Ecuador	San Miguel de los Bancos	Count	2017	0	0	0
Ecuador	Santiago de Pillaro	Proportion	2000	14.6	23.3	9.3
Ecuador	Santiago de Pillaro	Proportion	2017	21.0	30.3	14.3
Ecuador	Santiago de Pillaro	Count	2000	0	0	0
Ecuador	Santiago de Pillaro	Count	2017	0	0	0
Ecuador	Yantzaza	Proportion	2000	17.5	25.8	11.0
Ecuador	Yantzaza	Proportion	2017	22.2	30.3	15.1
Ecuador	Yantzaza	Count	2000	0	0	0
Ecuador	Yantzaza	Count	2017	0	0	0
Ecuador	Oña	Proportion	2000	17.8	25.5	11.5
Ecuador	Oña	Proportion	2017	20.6	28.2	13.9
Ecuador	Oña	Count	2000	0	0	0
Ecuador	Oña	Count	2017	0	0	0
Ecuador	Penipe	Proportion	2000	16.3	24.5	10.7
Ecuador	Penipe	Proportion	2017	22.3	32.0	15.2
Ecuador	Penipe	Count	2000	0	0	0
Ecuador	Penipe	Count	2017	0	0	0
Ecuador	Marcabelí	Proportion	2000	19.9	28.2	13.1

Table 4: ORS by Unit *(continued)*

Country	Unit	Metric	Year	Mean	Upper	Lower
Ecuador	Marcabelí	Proportion	2017	26.3	35.0	18.4
Ecuador	Marcabelí	Count	2000	0	0	0
Ecuador	Marcabelí	Count	2017	0	0	0
Ecuador	El Triunfo	Proportion	2000	18.7	27.0	11.9
Ecuador	El Triunfo	Proportion	2017	27.2	36.6	18.8
Ecuador	El Triunfo	Count	2000	0	0	0
Ecuador	El Triunfo	Count	2017	0	0	0
Ecuador	Olmedo	Proportion	2000	19.4	27.9	12.6
Ecuador	Olmedo	Proportion	2017	23.7	32.6	16.0
Ecuador	Olmedo	Count	2000	0	0	0
Ecuador	Olmedo	Count	2017	0	0	0
Ecuador	Quinsaloma	Proportion	2000	20.8	31.9	13.4
Ecuador	Quinsaloma	Proportion	2017	27.6	37.8	18.8
Ecuador	Quinsaloma	Count	2000	0	0	0
Ecuador	Quinsaloma	Count	2017	0	0	0
Ecuador	Junín	Proportion	2000	20.1	29.1	12.6
Ecuador	Junín	Proportion	2017	29.7	39.7	20.6
Ecuador	Junín	Count	2000	0	0	0
Ecuador	Junín	Count	2017	0	0	0
Ecuador	Santiago	Proportion	2000	17.7	26.1	11.7
Ecuador	Santiago	Proportion	2017	23.6	32.5	16.7
Ecuador	Santiago	Count	2000	0	0	0
Ecuador	Santiago	Count	2017	0	0	0
Ecuador	Tisaleo	Proportion	2000	14.0	21.7	9.1
Ecuador	Tisaleo	Proportion	2017	21.2	31.2	14.5
Ecuador	Tisaleo	Count	2000	0	0	0
Ecuador	Tisaleo	Count	2017	0	0	0
Ecuador	Zamora	Proportion	2000	19.5	28.8	12.4
Ecuador	Zamora	Proportion	2017	23.3	31.7	16.0
Ecuador	Zamora	Count	2000	0	0	0
Ecuador	Zamora	Count	2017	0	0	0
Ecuador	Paute	Proportion	2000	18.5	26.5	11.8
Ecuador	Paute	Proportion	2017	23.3	31.7	16.8
Ecuador	Paute	Count	2000	0	0	0
Ecuador	Paute	Count	2017	0	0	0
Ecuador	Riobamba	Proportion	2000	15.1	23.0	9.6
Ecuador	Riobamba	Proportion	2017	22.7	32.4	15.3
Ecuador	Riobamba	Count	2000	0	0	0
Ecuador	Riobamba	Count	2017	0	0	0
Ecuador	Pasaje	Proportion	2000	19.2	27.8	12.6
Ecuador	Pasaje	Proportion	2017	27.9	38.9	19.8
Ecuador	Pasaje	Count	2000	0	0	0
Ecuador	Pasaje	Count	2017	0	0	0
Ecuador	General Antonio Elizalde	Proportion	2000	20.4	29.2	13.2
Ecuador	General Antonio Elizalde	Proportion	2017	27.2	36.4	18.9
Ecuador	General Antonio Elizalde	Count	2000	0	0	0
Ecuador	General Antonio Elizalde	Count	2017	0	0	0
Ecuador	Paltas	Proportion	2000	19.2	27.8	12.7
Ecuador	Paltas	Proportion	2017	23.6	32.9	16.0

Table 4: ORS by Unit *(continued)*

Country	Unit	Metric	Year	Mean	Upper	Lower
Ecuador	Paltas	Count	2000	0	0	0
Ecuador	Paltas	Count	2017	0	0	0
Ecuador	Urdaneta	Proportion	2000	19.9	29.7	13.0
Ecuador	Urdaneta	Proportion	2017	29.5	40.0	21.1
Ecuador	Urdaneta	Count	2000	0	0	0
Ecuador	Urdaneta	Count	2017	0	0	0
Ecuador	Manta	Proportion	2000	23.1	32.9	14.9
Ecuador	Manta	Proportion	2017	34.1	43.9	23.9
Ecuador	Manta	Count	2000	0	0	0
Ecuador	Manta	Count	2017	0	0	0
Ecuador	Sucúa	Proportion	2000	16.9	24.8	10.8
Ecuador	Sucúa	Proportion	2017	23.0	31.6	16.2
Ecuador	Sucúa	Count	2000	0	0	0
Ecuador	Sucúa	Count	2017	0	0	0
Ecuador	Pucará	Proportion	2000	17.4	25.1	11.1
Ecuador	Pucará	Proportion	2017	23.1	31.7	15.9
Ecuador	Pucará	Count	2000	0	0	0
Ecuador	Pucará	Count	2017	0	0	0
Ecuador	Piñas	Proportion	2000	19.4	28.4	12.6
Ecuador	Piñas	Proportion	2017	25.9	35.0	17.8
Ecuador	Piñas	Count	2000	0	0	0
Ecuador	Piñas	Count	2017	0	0	0
Ecuador	Guayaquil	Proportion	2000	23.9	33.2	15.2
Ecuador	Guayaquil	Proportion	2017	33.7	44.3	24.3
Ecuador	Guayaquil	Count	2000	0	0	0
Ecuador	Guayaquil	Count	2017	0	0	0
Ecuador	Pindal	Proportion	2000	21.8	30.8	14.7
Ecuador	Pindal	Proportion	2017	26.1	36.6	18.6
Ecuador	Pindal	Count	2000	0	0	0
Ecuador	Pindal	Count	2017	0	0	0
Ecuador	Valencia	Proportion	2000	20.8	32.3	13.7
Ecuador	Valencia	Proportion	2017	29.8	40.9	20.8
Ecuador	Valencia	Count	2000	0	0	0
Ecuador	Valencia	Count	2017	0	0	0
Ecuador	Montecristi	Proportion	2000	22.0	31.9	14.1
Ecuador	Montecristi	Proportion	2017	32.5	42.9	23.0
Ecuador	Montecristi	Count	2000	0	0	0
Ecuador	Montecristi	Count	2017	0	0	0
Ecuador	Taisha	Proportion	2000	18.6	27.9	11.9
Ecuador	Taisha	Proportion	2017	24.4	33.1	16.7
Ecuador	Taisha	Count	2000	0	0	0
Ecuador	Taisha	Count	2017	0	0	0
Ecuador	San Fernando	Proportion	2000	15.6	24.0	9.8
Ecuador	San Fernando	Proportion	2017	19.2	26.9	12.9
Ecuador	San Fernando	Count	2000	0	0	0
Ecuador	San Fernando	Count	2017	0	0	0
Ecuador	Portovelo	Proportion	2000	19.7	28.6	12.5
Ecuador	Portovelo	Proportion	2017	26.3	36.1	18.0
Ecuador	Portovelo	Count	2000	0	0	0

Table 4: ORS by Unit *(continued)*

Country	Unit	Metric	Year	Mean	Upper	Lower
Ecuador	Portovelo	Count	2017	0	0	0
Ecuador	Isidro Ayora	Proportion	2000	24.9	36.5	16.0
Ecuador	Isidro Ayora	Proportion	2017	33.2	43.1	24.0
Ecuador	Isidro Ayora	Count	2000	0	0	0
Ecuador	Isidro Ayora	Count	2017	0	0	0
Ecuador	Puyango	Proportion	2000	20.7	29.0	13.6
Ecuador	Puyango	Proportion	2017	25.9	35.2	18.3
Ecuador	Puyango	Count	2000	0	0	0
Ecuador	Puyango	Count	2017	0	0	0
Ecuador	Ventanas	Proportion	2000	19.0	28.8	12.3
Ecuador	Ventanas	Proportion	2017	28.0	38.5	19.4
Ecuador	Ventanas	Count	2000	0	0	0
Ecuador	Ventanas	Count	2017	0	0	0
Ecuador	Olmedo	Proportion	2000	23.6	35.0	15.4
Ecuador	Olmedo	Proportion	2017	29.6	39.7	20.9
Ecuador	Olmedo	Count	2000	0	0	0
Ecuador	Olmedo	Count	2017	0	0	0
Ecuador	Tiwintza	Proportion	2000	18.6	27.1	11.9
Ecuador	Tiwintza	Proportion	2017	24.0	33.2	17.0
Ecuador	Tiwintza	Count	2000	0	0	0
Ecuador	Tiwintza	Count	2017	0	0	0
Ecuador	Santa Isabel	Proportion	2000	17.8	27.0	11.4
Ecuador	Santa Isabel	Proportion	2017	22.9	31.0	15.8
Ecuador	Santa Isabel	Count	2000	0	0	0
Ecuador	Santa Isabel	Count	2017	0	0	0
Ecuador	Santa Rosa	Proportion	2000	20.0	28.8	13.6
Ecuador	Santa Rosa	Proportion	2017	29.2	39.9	20.8
Ecuador	Santa Rosa	Count	2000	0	0	0
Ecuador	Santa Rosa	Count	2017	0	0	0
Ecuador	Lomas de Sargentillo	Proportion	2000	22.4	33.0	14.2
Ecuador	Lomas de Sargentillo	Proportion	2017	32.4	41.7	23.2
Ecuador	Lomas de Sargentillo	Count	2000	0	0	0
Ecuador	Lomas de Sargentillo	Count	2017	0	0	0
Ecuador	Quilanga	Proportion	2000	17.1	25.2	11.2
Ecuador	Quilanga	Proportion	2017	21.1	30.4	14.2
Ecuador	Quilanga	Count	2000	0	0	0
Ecuador	Quilanga	Count	2017	0	0	0
Ecuador	Vinces	Proportion	2000	21.5	32.1	13.9
Ecuador	Vinces	Proportion	2017	29.5	40.4	21.5
Ecuador	Vinces	Count	2000	0	0	0
Ecuador	Vinces	Count	2017	0	0	0
Ecuador	Paján	Proportion	2000	25.0	37.1	16.7
Ecuador	Paján	Proportion	2017	30.4	39.4	21.9
Ecuador	Paján	Count	2000	0	0	0
Ecuador	Paján	Count	2017	0	0	0
Ecuador	Sevilla de Oro	Proportion	2000	17.7	25.2	11.3
Ecuador	Sevilla de Oro	Proportion	2017	20.7	28.6	14.6
Ecuador	Sevilla de Oro	Count	2000	0	0	0
Ecuador	Sevilla de Oro	Count	2017	0	0	0

Table 4: ORS by Unit *(continued)*

Country	Unit	Metric	Year	Mean	Upper	Lower
Ecuador	Zaruma	Proportion	2000	18.1	26.8	11.5
Ecuador	Zaruma	Proportion	2017	24.5	33.6	16.9
Ecuador	Zaruma	Count	2000	0	0	0
Ecuador	Zaruma	Count	2017	0	0	0
Ecuador	Milagro	Proportion	2000	19.6	29.2	12.4
Ecuador	Milagro	Proportion	2017	28.6	37.7	20.7
Ecuador	Milagro	Count	2000	0	0	0
Ecuador	Milagro	Count	2017	0	0	0
Ecuador	Saraguro	Proportion	2000	15.8	22.7	9.9
Ecuador	Saraguro	Proportion	2017	19.9	27.1	13.4
Ecuador	Saraguro	Count	2000	0	0	0
Ecuador	Saraguro	Count	2017	0	0	0
Ecuador	Pedernales	Proportion	2000	20.2	30.9	12.7
Ecuador	Pedernales	Proportion	2017	26.4	37.1	18.0
Ecuador	Pedernales	Count	2000	0	0	0
Ecuador	Pedernales	Count	2017	0	0	0
Ecuador	Sigsig	Proportion	2000	14.8	21.7	9.4
Ecuador	Sigsig	Proportion	2017	17.9	24.8	12.4
Ecuador	Sigsig	Count	2000	0	0	0
Ecuador	Sigsig	Count	2017	0	0	0
Ecuador	Naranjal	Proportion	2000	21.6	31.9	14.2
Ecuador	Naranjal	Proportion	2017	30.4	41.1	21.5
Ecuador	Naranjal	Count	2000	0	0	0
Ecuador	Naranjal	Count	2017	0	0	0
Ecuador	Sozoranga	Proportion	2000	19.9	28.7	13.6
Ecuador	Sozoranga	Proportion	2017	23.5	32.3	16.1
Ecuador	Sozoranga	Count	2000	0	0	0
Ecuador	Sozoranga	Count	2017	0	0	0
Ecuador	Pichincha	Proportion	2000	22.5	33.5	14.8
Ecuador	Pichincha	Proportion	2017	29.8	40.1	20.4
Ecuador	Pichincha	Count	2000	0	0	0
Ecuador	Pichincha	Count	2017	0	0	0
Ecuador	Naranjito	Proportion	2000	18.6	27.4	11.9
Ecuador	Naranjito	Proportion	2017	28.7	37.3	20.5
Ecuador	Naranjito	Count	2000	0	0	0
Ecuador	Naranjito	Count	2017	0	0	0
Ecuador	Zapotillo	Proportion	2000	25.8	34.9	18.3
Ecuador	Zapotillo	Proportion	2017	30.3	41.3	22.1
Ecuador	Zapotillo	Count	2000	0	0	0
Ecuador	Zapotillo	Count	2017	0	0	0
Ecuador	Portoviejo	Proportion	2000	21.0	30.9	13.2
Ecuador	Portoviejo	Proportion	2017	32.6	42.8	23.0
Ecuador	Portoviejo	Count	2000	0	0	0
Ecuador	Portoviejo	Count	2017	0	0	0
Ecuador	Nobol	Proportion	2000	21.4	31.5	13.5
Ecuador	Nobol	Proportion	2017	31.6	41.5	23.3
Ecuador	Nobol	Count	2000	0	0	0
Ecuador	Nobol	Count	2017	0	0	0
Ecuador	Puerto López	Proportion	2000	24.3	34.8	16.0

Table 4: ORS by Unit *(continued)*

Country	Unit	Metric	Year	Mean	Upper	Lower
Ecuador	Puerto López	Proportion	2017	32.9	42.7	23.3
Ecuador	Puerto López	Count	2000	0	0	0
Ecuador	Puerto López	Count	2017	0	0	0
Ecuador	Palestina	Proportion	2000	21.0	31.5	13.2
Ecuador	Palestina	Proportion	2017	30.1	41.0	21.6
Ecuador	Palestina	Count	2000	0	0	0
Ecuador	Palestina	Count	2017	0	0	0
Ecuador	Rocafuerte	Proportion	2000	21.2	31.0	13.1
Ecuador	Rocafuerte	Proportion	2017	32.4	43.1	22.4
Ecuador	Rocafuerte	Count	2000	0	0	0
Ecuador	Rocafuerte	Count	2017	0	0	0
Ecuador	Pedro Carbo	Proportion	2000	25.9	39.6	16.7
Ecuador	Pedro Carbo	Proportion	2017	34.6	44.9	24.9
Ecuador	Pedro Carbo	Count	2000	0	0	0
Ecuador	Pedro Carbo	Count	2017	0	0	0
Ecuador	San Vicente	Proportion	2000	20.4	31.4	12.8
Ecuador	San Vicente	Proportion	2017	30.7	42.5	20.8
Ecuador	San Vicente	Count	2000	0	0	0
Ecuador	San Vicente	Count	2017	0	0	0
Ecuador	Playas	Proportion	2000	24.8	35.9	16.3
Ecuador	Playas	Proportion	2017	33.4	44.0	23.6
Ecuador	Playas	Count	2000	0	0	0
Ecuador	Playas	Count	2017	0	0	0
Ecuador	Santa Ana	Proportion	2000	22.2	33.6	14.3
Ecuador	Santa Ana	Proportion	2017	29.9	40.4	20.7
Ecuador	Santa Ana	Count	2000	0	0	0
Ecuador	Santa Ana	Count	2017	0	0	0
Ecuador	Samborondón	Proportion	2000	21.0	30.2	13.6
Ecuador	Samborondón	Proportion	2017	31.2	41.5	22.6
Ecuador	Samborondón	Count	2000	0	0	0
Ecuador	Samborondón	Count	2017	0	0	0
Ecuador	Sucre	Proportion	2000	20.6	30.6	13.0
Ecuador	Sucre	Proportion	2017	30.8	42.1	21.5
Ecuador	Sucre	Count	2000	0	0	0
Ecuador	Sucre	Count	2017	0	0	0
Ecuador	San Jacinto de Yaguachi	Proportion	2000	20.7	30.8	13.2
Ecuador	San Jacinto de Yaguachi	Proportion	2017	30.5	40.0	22.1
Ecuador	San Jacinto de Yaguachi	Count	2000	0	0	0
Ecuador	San Jacinto de Yaguachi	Count	2017	0	0	0
Ecuador	Tosagua	Proportion	2000	20.4	30.9	12.8
Ecuador	Tosagua	Proportion	2017	32.8	43.9	22.6
Ecuador	Tosagua	Count	2000	0	0	0
Ecuador	Tosagua	Count	2017	0	0	0
Ecuador	Santa Lucia	Proportion	2000	22.5	33.4	14.4
Ecuador	Santa Lucia	Proportion	2017	30.7	41.0	22.1
Ecuador	Santa Lucia	Count	2000	0	0	0
Ecuador	Santa Lucia	Count	2017	0	0	0
Ecuador	Simon Bolivar	Proportion	2000	19.9	29.5	12.8
Ecuador	Simon Bolivar	Proportion	2017	29.4	38.6	20.9

Table 4: ORS by Unit *(continued)*

Country	Unit	Metric	Year	Mean	Upper	Lower
Ecuador	Simon Bolivar	Count	2000	0	0	0
Ecuador	Simon Bolivar	Count	2017	0	0	0
Ecuador	Urbina Jado	Proportion	2000	20.3	30.0	12.7
Ecuador	Urbina Jado	Proportion	2017	30.2	41.4	21.9
Ecuador	Urbina Jado	Count	2000	0	0	0
Ecuador	Urbina Jado	Count	2017	0	0	0
Egypt	Aja	Proportion	2000	38.4	45.3	30.3
Egypt	Aja	Proportion	2017	29.9	42.4	18.6
Egypt	Aja	Count	2000	0	0	0
Egypt	Aja	Count	2017	0	0	0
Egypt	Al-Ghurdaqah	Proportion	2000	28.2	41.2	17.6
Egypt	Al-Ghurdaqah	Proportion	2017	26.7	39.5	16.9
Egypt	Al-Ghurdaqah	Count	2000	0	0	0
Egypt	Al-Ghurdaqah	Count	2017	0	0	0
Egypt	Abu al-Matamir	Proportion	2000	29.8	39.3	22.5
Egypt	Abu al-Matamir	Proportion	2017	31.5	45.4	19.9
Egypt	Abu al-Matamir	Count	2000	0	0	0
Egypt	Abu al-Matamir	Count	2017	0	0	0
Egypt	Al-Fayyum	Proportion	2000	37.7	46.8	29.7
Egypt	Al-Fayyum	Proportion	2017	28.9	43.7	19.1
Egypt	Al-Fayyum	Count	2000	0	0	0
Egypt	Al-Fayyum	Count	2017	0	0	0
Egypt	Al-Mahallah al-Kubra	Proportion	2000	38.7	46.4	31.1
Egypt	Al-Mahallah al-Kubra	Proportion	2017	31.6	43.8	20.3
Egypt	Al-Mahallah al-Kubra	Count	2000	0	0	0
Egypt	Al-Mahallah al-Kubra	Count	2017	0	0	0
Egypt	Ad-Dukhaylah	Proportion	2000	23.9	32.7	16.1
Egypt	Ad-Dukhaylah	Proportion	2017	29.5	46.2	18.4
Egypt	Ad-Dukhaylah	Count	2000	0	0	0
Egypt	Ad-Dukhaylah	Count	2017	0	0	0
Egypt	Al-Qantarah	Proportion	2000	32.9	42.9	23.5
Egypt	Al-Qantarah	Proportion	2017	28.9	43.3	17.5
Egypt	Al-Qantarah	Count	2000	0	0	0
Egypt	Al-Qantarah	Count	2017	0	0	0
Egypt	Ad-Duqi	Proportion	2000	30.2	36.8	23.9
Egypt	Ad-Duqi	Proportion	2017	26.2	38.4	16.8
Egypt	Ad-Duqi	Count	2000	0	0	0
Egypt	Ad-Duqi	Count	2017	0	0	0
Egypt	Al-Bajur	Proportion	2000	34.5	43.1	26.3
Egypt	Al-Bajur	Proportion	2017	29.7	42.1	18.9
Egypt	Al-Bajur	Count	2000	0	0	0
Egypt	Al-Bajur	Count	2017	0	0	0
Egypt	Abu Qurqas	Proportion	2000	34.2	44.0	24.9
Egypt	Abu Qurqas	Proportion	2017	30.3	44.5	17.8
Egypt	Abu Qurqas	Count	2000	0	0	0
Egypt	Abu Qurqas	Count	2017	0	0	0
Egypt	'Abdin	Proportion	2000	30.8	37.6	24.5
Egypt	'Abdin	Proportion	2017	27.0	39.0	17.5
Egypt	'Abdin	Count	2000	0	0	0

Table 4: ORS by Unit *(continued)*

Country	Unit	Metric	Year	Mean	Upper	Lower
Egypt	'Abdin	Count	2017	0	0	0
Egypt	Al-'Ubur	Proportion	2000	32.0	39.1	24.9
Egypt	Al-'Ubur	Proportion	2017	27.6	41.7	17.5
Egypt	Al-'Ubur	Count	2000	0	0	0
Egypt	Al-'Ubur	Count	2017	0	0	0
Egypt	Luxor	Proportion	2000	28.8	38.5	19.9
Egypt	Luxor	Proportion	2017	29.6	42.7	18.1
Egypt	Luxor	Count	2000	0	0	0
Egypt	Luxor	Count	2017	0	0	0
Egypt	Al-Wahat al-Kharijah	Proportion	2000	28.4	37.2	20.6
Egypt	Al-Wahat al-Kharijah	Proportion	2017	27.4	37.3	19.0
Egypt	Al-Wahat al-Kharijah	Count	2000	0	0	0
Egypt	Al-Wahat al-Kharijah	Count	2017	0	0	0
Egypt	'Ataqah	Proportion	2000	30.8	42.3	20.7
Egypt	'Ataqah	Proportion	2017	26.9	38.5	16.8
Egypt	'Ataqah	Count	2000	0	0	0
Egypt	'Ataqah	Count	2017	0	0	0
Egypt	10 Ramadan 1	Proportion	2000	35.3	45.4	26.1
Egypt	10 Ramadan 1	Proportion	2017	27.7	41.8	17.9
Egypt	10 Ramadan 1	Count	2000	0	0	0
Egypt	10 Ramadan 1	Count	2017	0	0	0
Egypt	Abu Simbel	Proportion	2000	NA	NA	NA
Egypt	Abu Simbel	Proportion	2017	NA	NA	NA
Egypt	Abu Simbel	Count	2000	NA	NA	NA
Egypt	Abu Simbel	Count	2017	NA	NA	NA
Egypt	Abnub	Proportion	2000	33.2	41.0	25.7
Egypt	Abnub	Proportion	2017	30.3	45.2	16.7
Egypt	Abnub	Count	2000	0	0	0
Egypt	Abnub	Count	2017	0	0	0
Egypt	Al-Fashn	Proportion	2000	41.6	52.1	31.6
Egypt	Al-Fashn	Proportion	2017	28.5	42.2	17.1
Egypt	Al-Fashn	Count	2000	0	1	0
Egypt	Al-Fashn	Count	2017	0	0	0
Egypt	Ad-Dawahy	Proportion	2000	36.6	46.9	27.2
Egypt	Ad-Dawahy	Proportion	2017	27.4	41.8	17.0
Egypt	Ad-Dawahy	Count	2000	0	0	0
Egypt	Ad-Dawahy	Count	2017	0	0	0
Egypt	Damietta	Proportion	2000	40.1	51.6	30.0
Egypt	Damietta	Proportion	2017	26.6	42.7	15.1
Egypt	Damietta	Count	2000	0	1	0
Egypt	Damietta	Count	2017	0	0	0
Egypt	Abu Radis	Proportion	2000	31.6	42.2	22.5
Egypt	Abu Radis	Proportion	2017	27.8	40.7	17.4
Egypt	Abu Radis	Count	2000	0	0	0
Egypt	Abu Radis	Count	2017	0	0	0
Egypt	Al-Burulus	Proportion	2000	40.1	50.8	29.8
Egypt	Al-Burulus	Proportion	2017	32.2	46.6	19.6
Egypt	Al-Burulus	Count	2000	0	1	0
Egypt	Al-Burulus	Count	2017	0	0	0

Table 4: ORS by Unit *(continued)*

Country	Unit	Metric	Year	Mean	Upper	Lower
Egypt	Ad-Dab'ah	Proportion	2000	28.3	40.1	17.7
Egypt	Ad-Dab'ah	Proportion	2017	26.6	39.4	15.3
Egypt	Ad-Dab'ah	Count	2000	0	0	0
Egypt	Ad-Dab'ah	Count	2017	0	0	0
Egypt	Abu Tisht	Proportion	2000	33.7	43.6	26.6
Egypt	Abu Tisht	Proportion	2017	26.8	38.5	15.6
Egypt	Abu Tisht	Count	2000	0	0	0
Egypt	Abu Tisht	Count	2017	0	0	0
Egypt	Al-'Arish 1	Proportion	2000	30.2	45.6	18.9
Egypt	Al-'Arish 1	Proportion	2017	30.3	44.7	18.2
Egypt	Al-'Arish 1	Count	2000	0	0	0
Egypt	Al-'Arish 1	Count	2017	0	0	0
Egypt	Akhmim	Proportion	2000	34.8	45.4	25.9
Egypt	Akhmim	Proportion	2017	25.5	38.0	14.7
Egypt	Akhmim	Count	2000	0	0	0
Egypt	Akhmim	Count	2017	0	0	0
Egypt	Al-Jamaliyah	Proportion	2000	41.5	50.6	33.5
Egypt	Al-Jamaliyah	Proportion	2017	30.8	45.9	19.3
Egypt	Al-Jamaliyah	Count	2000	0	1	0
Egypt	Al-Jamaliyah	Count	2017	0	0	0
Egypt	Al-Ghurdaqah 2	Proportion	2000	29.1	40.2	19.9
Egypt	Al-Ghurdaqah 2	Proportion	2017	28.7	42.3	18.3
Egypt	Al-Ghurdaqah 2	Count	2000	0	0	0
Egypt	Al-Ghurdaqah 2	Count	2017	0	0	0
Egypt	Abu Hummus	Proportion	2000	29.6	37.6	22.1
Egypt	Abu Hummus	Proportion	2017	32.0	46.7	20.6
Egypt	Abu Hummus	Count	2000	0	0	0
Egypt	Abu Hummus	Count	2017	0	0	0
Egypt	Al-Fayyum City	Proportion	2000	39.1	48.4	31.4
Egypt	Al-Fayyum City	Proportion	2017	29.0	43.7	18.9
Egypt	Al-Fayyum City	Count	2000	0	0	0
Egypt	Al-Fayyum City	Count	2017	0	0	0
Egypt	Al-Mahallah al-Kubra 1	Proportion	2000	38.1	45.3	30.4
Egypt	Al-Mahallah al-Kubra 1	Proportion	2017	29.1	41.5	18.5
Egypt	Al-Mahallah al-Kubra 1	Count	2000	0	0	0
Egypt	Al-Mahallah al-Kubra 1	Count	2017	0	0	0
Egypt	Al-'Amriyah	Proportion	2000	25.6	34.9	18.2
Egypt	Al-'Amriyah	Proportion	2017	30.7	46.7	19.3
Egypt	Al-'Amriyah	Count	2000	0	0	0
Egypt	Al-'Amriyah	Count	2017	0	0	0
Egypt	Al-Qantarah ash-Sharqiyah	Proportion	2000	31.1	40.0	21.8
Egypt	Al-Qantarah ash-Sharqiyah	Proportion	2017	28.0	41.1	17.4
Egypt	Al-Qantarah ash-Sharqiyah	Count	2000	0	0	0
Egypt	Al-Qantarah ash-Sharqiyah	Count	2017	0	0	0
Egypt	Al-'Ajuzah	Proportion	2000	30.1	36.7	24.1
Egypt	Al-'Ajuzah	Proportion	2017	26.3	38.3	17.1
Egypt	Al-'Ajuzah	Count	2000	0	0	0
Egypt	Al-'Ajuzah	Count	2017	0	0	0
Egypt	Ash-Shuhada	Proportion	2000	35.1	44.4	26.9

Table 4: ORS by Unit *(continued)*

Country	Unit	Metric	Year	Mean	Upper	Lower
Egypt	Ash-Shuhada	Proportion	2017	30.8	45.4	19.9
Egypt	Ash-Shuhada	Count	2000	0	0	0
Egypt	Ash-Shuhada	Count	2017	0	0	0
Egypt	Al-'Idwah	Proportion	2000	42.3	52.3	32.9
Egypt	Al-'Idwah	Proportion	2017	30.6	43.4	18.9
Egypt	Al-'Idwah	Count	2000	0	1	0
Egypt	Al-'Idwah	Count	2017	0	0	0
Egypt	'Ain Schams	Proportion	2000	30.6	37.9	23.6
Egypt	'Ain Schams	Proportion	2017	25.8	37.6	16.3
Egypt	'Ain Schams	Count	2000	0	0	0
Egypt	'Ain Schams	Count	2017	0	0	0
Egypt	Al-Khankah	Proportion	2000	32.3	39.2	25.4
Egypt	Al-Khankah	Proportion	2017	28.8	43.1	18.7
Egypt	Al-Khankah	Count	2000	0	0	0
Egypt	Al-Khankah	Count	2017	0	0	0
Egypt	Tibah Police Dept.	Proportion	2000	30.2	40.7	22.2
Egypt	Tibah Police Dept.	Proportion	2017	30.8	44.3	18.7
Egypt	Tibah Police Dept.	Count	2000	0	0	0
Egypt	Tibah Police Dept.	Count	2017	0	0	0
Egypt	Baris Shurtah	Proportion	2000	26.4	38.2	16.1
Egypt	Baris Shurtah	Proportion	2017	28.8	41.9	18.5
Egypt	Baris Shurtah	Count	2000	0	0	0
Egypt	Baris Shurtah	Count	2017	0	0	0
Egypt	Al-Arb'in	Proportion	2000	30.6	42.7	18.8
Egypt	Al-Arb'in	Proportion	2017	25.7	37.7	15.4
Egypt	Al-Arb'in	Count	2000	0	0	0
Egypt	Al-Arb'in	Count	2017	0	0	0
Egypt	10 Ramadan 2	Proportion	2000	34.9	44.4	26.1
Egypt	10 Ramadan 2	Proportion	2017	28.8	43.1	18.8
Egypt	10 Ramadan 2	Count	2000	0	0	0
Egypt	10 Ramadan 2	Count	2017	0	0	0
Egypt	Aswan	Proportion	2000	27.9	38.4	19.0
Egypt	Aswan	Proportion	2017	28.6	44.7	16.1
Egypt	Aswan	Count	2000	0	0	0
Egypt	Aswan	Count	2017	0	0	0
Egypt	Abu Tij	Proportion	2000	32.7	41.4	25.3
Egypt	Abu Tij	Proportion	2017	29.6	46.1	16.9
Egypt	Abu Tij	Count	2000	0	0	0
Egypt	Abu Tij	Count	2017	0	0	0
Egypt	Al-Wasta	Proportion	2000	38.5	46.8	31.0
Egypt	Al-Wasta	Proportion	2017	29.9	43.6	17.7
Egypt	Al-Wasta	Count	2000	0	0	0
Egypt	Al-Wasta	Count	2017	0	0	0
Egypt	Al-'Arab	Proportion	2000	36.3	46.7	26.7
Egypt	Al-'Arab	Proportion	2017	26.4	40.8	16.5
Egypt	Al-'Arab	Count	2000	0	0	0
Egypt	Al-'Arab	Count	2017	0	0	0
Egypt	Damietta 1	Proportion	2000	37.4	48.1	27.2
Egypt	Damietta 1	Proportion	2017	25.0	40.5	14.0

Table 4: ORS by Unit *(continued)*

Country	Unit	Metric	Year	Mean	Upper	Lower
Egypt	Damietta 1	Count	2000	0	0	0
Egypt	Damietta 1	Count	2017	0	0	0
Egypt	Abu Zenima	Proportion	2000	30.6	43.7	20.8
Egypt	Abu Zenima	Proportion	2017	26.9	40.5	16.1
Egypt	Abu Zenima	Count	2000	0	0	0
Egypt	Abu Zenima	Count	2017	0	0	0
Egypt	Al-Hamul	Proportion	2000	40.1	49.7	31.4
Egypt	Al-Hamul	Proportion	2017	32.4	46.7	20.5
Egypt	Al-Hamul	Count	2000	0	0	0
Egypt	Al-Hamul	Count	2017	0	0	0
Egypt	Al-Hammam	Proportion	2000	25.4	36.6	17.3
Egypt	Al-Hammam	Proportion	2017	26.7	40.5	14.8
Egypt	Al-Hammam	Count	2000	0	0	0
Egypt	Al-Hammam	Count	2017	0	0	0
Egypt	Al-Waqf	Proportion	2000	31.7	40.3	23.7
Egypt	Al-Waqf	Proportion	2017	28.2	40.6	17.0
Egypt	Al-Waqf	Count	2000	0	0	0
Egypt	Al-Waqf	Count	2017	0	0	0
Egypt	Al-'Arish 2	Proportion	2000	30.1	44.2	18.3
Egypt	Al-'Arish 2	Proportion	2017	29.8	43.9	18.2
Egypt	Al-'Arish 2	Count	2000	0	0	0
Egypt	Al-'Arish 2	Count	2017	0	0	0
Egypt	Al-'Usayrat	Proportion	2000	35.1	46.6	26.1
Egypt	Al-'Usayrat	Proportion	2017	25.9	38.1	15.0
Egypt	Al-'Usayrat	Count	2000	0	0	0
Egypt	Al-'Usayrat	Count	2017	0	0	0
Egypt	Al-Mansurah	Proportion	2000	39.2	45.4	32.0
Egypt	Al-Mansurah	Proportion	2017	29.2	42.2	18.9
Egypt	Al-Mansurah	Count	2000	0	0	0
Egypt	Al-Mansurah	Count	2017	0	0	0
Egypt	Al-Qusayr	Proportion	2000	29.4	40.3	20.5
Egypt	Al-Qusayr	Proportion	2017	25.4	38.3	15.1
Egypt	Al-Qusayr	Count	2000	0	0	0
Egypt	Al-Qusayr	Count	2017	0	0	0
Egypt	Ad-Dilinat	Proportion	2000	33.3	41.4	25.5
Egypt	Ad-Dilinat	Proportion	2017	32.0	45.3	21.5
Egypt	Ad-Dilinat	Count	2000	0	0	0
Egypt	Ad-Dilinat	Count	2017	0	0	0
Egypt	Ibshaway	Proportion	2000	38.5	48.4	28.1
Egypt	Ibshaway	Proportion	2017	28.6	42.8	18.8
Egypt	Ibshaway	Count	2000	0	0	0
Egypt	Ibshaway	Count	2017	0	0	0
Egypt	Al-Mahallah al-Kubra 2	Proportion	2000	38.5	46.3	30.5
Egypt	Al-Mahallah al-Kubra 2	Proportion	2017	31.8	44.4	20.4
Egypt	Al-Mahallah al-Kubra 2	Count	2000	0	0	0
Egypt	Al-Mahallah al-Kubra 2	Count	2017	0	0	0
Egypt	Al-'Atarin	Proportion	2000	22.9	31.4	15.8
Egypt	Al-'Atarin	Proportion	2017	26.3	41.7	16.2
Egypt	Al-'Atarin	Count	2000	0	0	0

Table 4: ORS by Unit *(continued)*

Country	Unit	Metric	Year	Mean	Upper	Lower
Egypt	Al-'Atarin	Count	2017	0	0	0
Egypt	At-Tall al-Kabir	Proportion	2000	33.8	42.7	26.0
Egypt	At-Tall al-Kabir	Proportion	2017	28.9	44.0	17.1
Egypt	At-Tall al-Kabir	Count	2000	0	0	0
Egypt	At-Tall al-Kabir	Count	2017	0	0	0
Egypt	Al-'Ayyat	Proportion	2000	35.3	43.8	27.7
Egypt	Al-'Ayyat	Proportion	2017	29.3	44.4	19.0
Egypt	Al-'Ayyat	Count	2000	0	0	0
Egypt	Al-'Ayyat	Count	2017	0	0	0
Egypt	Ashmun	Proportion	2000	34.1	42.5	26.8
Egypt	Ashmun	Proportion	2017	29.4	43.1	18.9
Egypt	Ashmun	Count	2000	0	0	0
Egypt	Ashmun	Count	2017	0	0	0
Egypt	Al-Minya	Proportion	2000	34.3	43.8	26.2
Egypt	Al-Minya	Proportion	2017	30.1	44.3	18.3
Egypt	Al-Minya	Count	2000	0	0	0
Egypt	Al-Minya	Count	2017	0	0	0
Egypt	15 Mayu	Proportion	2000	31.7	40.0	24.3
Egypt	15 Mayu	Proportion	2017	28.9	44.0	18.8
Egypt	15 Mayu	Count	2000	0	0	0
Egypt	15 Mayu	Count	2017	0	0	0
Egypt	Al-Khusus	Proportion	2000	30.5	38.0	23.6
Egypt	Al-Khusus	Proportion	2017	26.4	39.7	16.4
Egypt	Al-Khusus	Count	2000	0	0	0
Egypt	Al-Khusus	Count	2017	0	0	0
Egypt	Unorganized in Al Uqsur	Proportion	2000	29.5	38.6	20.9
Egypt	Unorganized in Al Uqsur	Proportion	2017	30.3	43.1	18.5
Egypt	Unorganized in Al Uqsur	Count	2000	0	0	0
Egypt	Unorganized in Al Uqsur	Count	2017	0	0	0
Egypt	Shurtah al-Dakhlah	Proportion	2000	31.8	45.6	19.6
Egypt	Shurtah al-Dakhlah	Proportion	2017	31.2	46.5	19.7
Egypt	Shurtah al-Dakhlah	Count	2000	0	0	0
Egypt	Shurtah al-Dakhlah	Count	2017	0	0	0
Egypt	Al-Janayin	Proportion	2000	30.4	41.8	19.8
Egypt	Al-Janayin	Proportion	2017	28.1	41.1	16.6
Egypt	Al-Janayin	Count	2000	0	0	0
Egypt	Al-Janayin	Count	2017	0	0	0
Egypt	Abu Hammad	Proportion	2000	37.2	44.3	29.1
Egypt	Abu Hammad	Proportion	2017	30.6	46.5	18.8
Egypt	Abu Hammad	Count	2000	0	0	0
Egypt	Abu Hammad	Count	2017	0	0	0
Egypt	Aswan City	Proportion	2000	28.8	39.2	20.0
Egypt	Aswan City	Proportion	2017	30.2	46.0	17.2
Egypt	Aswan City	Count	2000	0	0	0
Egypt	Aswan City	Count	2017	0	0	0
Egypt	Al-Badari	Proportion	2000	33.7	43.3	26.2
Egypt	Al-Badari	Proportion	2017	29.8	46.3	18.0
Egypt	Al-Badari	Count	2000	0	0	0
Egypt	Al-Badari	Count	2017	0	0	0

Table 4: ORS by Unit *(continued)*

Country	Unit	Metric	Year	Mean	Upper	Lower
Egypt	Bani Suwayf	Proportion	2000	39.5	47.9	31.3
Egypt	Bani Suwayf	Proportion	2017	29.3	44.5	17.8
Egypt	Bani Suwayf	Count	2000	0	0	0
Egypt	Bani Suwayf	Count	2017	0	0	0
Egypt	Al-Ganoub	Proportion	2000	37.8	48.2	28.1
Egypt	Al-Ganoub	Proportion	2017	30.3	44.7	18.7
Egypt	Al-Ganoub	Count	2000	0	0	0
Egypt	Al-Ganoub	Count	2017	0	0	0
Egypt	Damietta 2	Proportion	2000	40.6	52.4	30.2
Egypt	Damietta 2	Proportion	2017	26.2	43.0	15.3
Egypt	Damietta 2	Count	2000	0	1	0
Egypt	Damietta 2	Count	2017	0	0	0
Egypt	At-Tur	Proportion	2000	31.3	42.4	22.3
Egypt	At-Tur	Proportion	2017	29.4	45.7	18.2
Egypt	At-Tur	Count	2000	0	0	0
Egypt	At-Tur	Count	2017	0	0	0
Egypt	Ar-Riyad	Proportion	2000	36.7	45.8	28.2
Egypt	Ar-Riyad	Proportion	2017	32.4	46.5	20.3
Egypt	Ar-Riyad	Count	2000	0	0	0
Egypt	Ar-Riyad	Count	2017	0	0	0
Egypt	As-Salum	Proportion	2000	27.0	40.8	16.7
Egypt	As-Salum	Proportion	2017	28.6	42.4	18.0
Egypt	As-Salum	Count	2000	0	0	0
Egypt	As-Salum	Count	2017	0	0	0
Egypt	Armant	Proportion	2000	29.5	39.2	19.7
Egypt	Armant	Proportion	2017	29.8	42.0	18.4
Egypt	Armant	Count	2000	0	0	0
Egypt	Armant	Count	2017	0	0	0
Egypt	Al-'Arish 3	Proportion	2000	28.2	41.7	16.9
Egypt	Al-'Arish 3	Proportion	2017	28.9	43.3	17.4
Egypt	Al-'Arish 3	Count	2000	0	0	0
Egypt	Al-'Arish 3	Count	2017	0	0	0
Egypt	Al-Baliyana	Proportion	2000	34.4	45.2	26.9
Egypt	Al-Baliyana	Proportion	2017	25.7	37.4	15.0
Egypt	Al-Baliyana	Count	2000	0	0	0
Egypt	Al-Baliyana	Count	2017	0	0	0
Egypt	Al-Mansurah 1	Proportion	2000	37.7	44.2	30.0
Egypt	Al-Mansurah 1	Proportion	2017	26.5	38.5	16.7
Egypt	Al-Mansurah 1	Count	2000	0	0	0
Egypt	Al-Mansurah 1	Count	2017	0	0	0
Egypt	Ash-Shalatin	Proportion	2000	28.8	38.5	20.5
Egypt	Ash-Shalatin	Proportion	2017	30.5	38.6	22.3
Egypt	Ash-Shalatin	Count	2000	0	0	0
Egypt	Ash-Shalatin	Count	2017	0	0	0
Egypt	Al-Mahmudiyah	Proportion	2000	31.1	39.4	23.5
Egypt	Al-Mahmudiyah	Proportion	2017	32.3	46.8	20.5
Egypt	Al-Mahmudiyah	Count	2000	0	0	0
Egypt	Al-Mahmudiyah	Count	2017	0	0	0
Egypt	Itsa	Proportion	2000	41.0	50.6	32.5

Table 4: ORS by Unit *(continued)*

Country	Unit	Metric	Year	Mean	Upper	Lower
Egypt	Itsa	Proportion	2017	29.5	44.9	19.3
Egypt	Itsa	Count	2000	0	1	0
Egypt	Itsa	Count	2017	0	0	0
Egypt	As-Santah	Proportion	2000	37.5	44.1	29.5
Egypt	As-Santah	Proportion	2017	32.3	45.6	20.7
Egypt	As-Santah	Count	2000	0	0	0
Egypt	As-Santah	Count	2017	0	0	0
Egypt	Al-Jumruk	Proportion	2000	23.3	31.8	16.2
Egypt	Al-Jumruk	Proportion	2017	27.1	42.7	16.7
Egypt	Al-Jumruk	Count	2000	0	0	0
Egypt	Al-Jumruk	Count	2017	0	0	0
Egypt	Fa'id	Proportion	2000	32.5	42.5	22.7
Egypt	Fa'id	Proportion	2017	29.8	43.1	17.6
Egypt	Fa'id	Count	2000	0	0	0
Egypt	Fa'id	Count	2017	0	0	0
Egypt	Al-'Umraniyah	Proportion	2000	30.0	37.2	24.0
Egypt	Al-'Umraniyah	Proportion	2017	26.4	39.8	16.7
Egypt	Al-'Umraniyah	Count	2000	0	0	0
Egypt	Al-'Umraniyah	Count	2017	0	0	0
Egypt	Birkat as-Sab'	Proportion	2000	36.6	43.5	29.0
Egypt	Birkat as-Sab'	Proportion	2017	32.1	45.0	21.1
Egypt	Birkat as-Sab'	Count	2000	0	0	0
Egypt	Birkat as-Sab'	Count	2017	0	0	0
Egypt	Al-Minya City	Proportion	2000	33.4	42.9	25.5
Egypt	Al-Minya City	Proportion	2017	29.5	43.1	17.7
Egypt	Al-Minya City	Count	2000	0	0	0
Egypt	Al-Minya City	Count	2017	0	0	0
Egypt	Ad-Darb al-Ahmar	Proportion	2000	30.0	36.6	23.7
Egypt	Ad-Darb al-Ahmar	Proportion	2017	24.5	37.0	15.6
Egypt	Ad-Darb al-Ahmar	Count	2000	0	0	0
Egypt	Ad-Darb al-Ahmar	Count	2017	0	0	0
Egypt	Al-Qanatir al-Khayriyah	Proportion	2000	32.1	39.7	25.7
Egypt	Al-Qanatir al-Khayriyah	Proportion	2017	28.5	42.0	18.3
Egypt	Al-Qanatir al-Khayriyah	Count	2000	0	0	0
Egypt	Al-Qanatir al-Khayriyah	Count	2017	0	0	0
Egypt	Shurtah al-Farafirah	Proportion	2000	29.8	43.7	17.1
Egypt	Shurtah al-Farafirah	Proportion	2017	29.6	45.0	18.0
Egypt	Shurtah al-Farafirah	Count	2000	0	0	0
Egypt	Shurtah al-Farafirah	Count	2017	0	0	0
Egypt	Faisal	Proportion	2000	30.6	41.3	20.2
Egypt	Faisal	Proportion	2017	26.6	38.5	16.0
Egypt	Faisal	Count	2000	0	0	0
Egypt	Faisal	Count	2017	0	0	0
Egypt	Abu Kabir	Proportion	2000	39.2	47.1	30.8
Egypt	Abu Kabir	Proportion	2017	31.6	47.6	18.5
Egypt	Abu Kabir	Count	2000	0	0	0
Egypt	Abu Kabir	Count	2017	0	0	0
Egypt	Daraw	Proportion	2000	28.5	38.1	20.2
Egypt	Daraw	Proportion	2017	29.5	43.0	16.9

Table 4: ORS by Unit *(continued)*

Country	Unit	Metric	Year	Mean	Upper	Lower
Egypt	Daraw	Count	2000	0	0	0
Egypt	Daraw	Count	2017	0	0	0
Egypt	Al-Fath	Proportion	2000	31.5	39.7	24.1
Egypt	Al-Fath	Proportion	2017	29.9	45.1	16.2
Egypt	Al-Fath	Count	2000	0	0	0
Egypt	Al-Fath	Count	2017	0	0	0
Egypt	Bani Suwayf City	Proportion	2000	38.1	46.0	29.7
Egypt	Bani Suwayf City	Proportion	2017	29.3	44.6	18.0
Egypt	Bani Suwayf City	Count	2000	0	0	0
Egypt	Bani Suwayf City	Count	2017	0	0	0
Egypt	Al-Ganoub 2	Proportion	2000	33.3	43.4	24.3
Egypt	Al-Ganoub 2	Proportion	2017	28.4	42.8	17.9
Egypt	Al-Ganoub 2	Count	2000	0	0	0
Egypt	Al-Ganoub 2	Count	2017	0	0	0
Egypt	Faraskur	Proportion	2000	32.1	42.2	24.4
Egypt	Faraskur	Proportion	2017	21.7	37.2	11.9
Egypt	Faraskur	Count	2000	0	0	0
Egypt	Faraskur	Count	2017	0	0	0
Egypt	Dahab	Proportion	2000	31.8	43.5	19.2
Egypt	Dahab	Proportion	2017	30.7	44.3	20.0
Egypt	Dahab	Count	2000	0	0	0
Egypt	Dahab	Count	2017	0	0	0
Egypt	Biyala	Proportion	2000	40.8	50.1	32.8
Egypt	Biyala	Proportion	2017	31.8	46.1	20.1
Egypt	Biyala	Count	2000	0	1	0
Egypt	Biyala	Count	2017	0	0	0
Egypt	Marina al-'Alamayn as-Siyahiyah	Proportion	2000	28.7	42.7	17.7
Egypt	Marina al-'Alamayn as-Siyahiyah	Proportion	2017	26.3	39.2	14.8
Egypt	Marina al-'Alamayn as-Siyahiyah	Count	2000	0	0	0
Egypt	Marina al-'Alamayn as-Siyahiyah	Count	2017	0	0	0
Egypt	Dishna	Proportion	2000	32.1	40.6	24.2
Egypt	Dishna	Proportion	2017	28.7	40.7	17.3
Egypt	Dishna	Count	2000	0	0	0
Egypt	Dishna	Count	2017	0	0	0
Egypt	Al-'Arish 4	Proportion	2000	28.9	41.2	17.8
Egypt	Al-'Arish 4	Proportion	2017	28.6	42.9	17.8
Egypt	Al-'Arish 4	Count	2000	0	0	0
Egypt	Al-'Arish 4	Count	2017	0	0	0
Egypt	Al-Kawtar	Proportion	2000	33.9	44.9	25.0
Egypt	Al-Kawtar	Proportion	2017	24.6	37.1	14.1
Egypt	Al-Kawtar	Count	2000	0	0	0
Egypt	Al-Kawtar	Count	2017	0	0	0
Egypt	Al-Mansurah 2	Proportion	2000	37.9	44.3	30.2
Egypt	Al-Mansurah 2	Proportion	2017	26.7	38.9	17.1
Egypt	Al-Mansurah 2	Count	2000	0	0	0
Egypt	Al-Mansurah 2	Count	2017	0	0	0

Table 4: ORS by Unit *(continued)*

Country	Unit	Metric	Year	Mean	Upper	Lower
Egypt	Marsa ’Alam	Proportion	2000	28.2	38.5	20.6
Egypt	Marsa ’Alam	Proportion	2017	27.1	37.2	18.7
Egypt	Marsa ’Alam	Count	2000	0	0	0
Egypt	Marsa ’Alam	Count	2017	0	0	0
Egypt	Ar-Rahmaniyah	Proportion	2000	32.2	41.3	24.3
Egypt	Ar-Rahmaniyah	Proportion	2017	32.1	46.4	20.8
Egypt	Ar-Rahmaniyah	Count	2000	0	0	0
Egypt	Ar-Rahmaniyah	Count	2017	0	0	0
Egypt	Sinnuris	Proportion	2000	37.9	47.4	29.7
Egypt	Sinnuris	Proportion	2017	29.0	42.6	18.7
Egypt	Sinnuris	Count	2000	0	0	0
Egypt	Sinnuris	Count	2017	0	0	0
Egypt	Basyun	Proportion	2000	35.1	43.7	27.3
Egypt	Basyun	Proportion	2017	31.8	44.5	21.3
Egypt	Basyun	Count	2000	0	0	0
Egypt	Basyun	Count	2017	0	0	0
Egypt	Al-Laban	Proportion	2000	22.9	31.4	15.8
Egypt	Al-Laban	Proportion	2017	26.3	41.7	16.2
Egypt	Al-Laban	Count	2000	0	0	0
Egypt	Al-Laban	Count	2017	0	0	0
Egypt	Ismailia	Proportion	2000	32.2	41.6	22.9
Egypt	Ismailia	Proportion	2017	28.9	43.1	17.3
Egypt	Ismailia	Count	2000	0	0	0
Egypt	Ismailia	Count	2017	0	0	0
Egypt	Al-Ahram	Proportion	2000	30.4	37.4	24.4
Egypt	Al-Ahram	Proportion	2017	27.1	39.8	17.4
Egypt	Al-Ahram	Count	2000	0	0	0
Egypt	Al-Ahram	Count	2017	0	0	0
Egypt	Minuf	Proportion	2000	35.2	44.0	27.2
Egypt	Minuf	Proportion	2017	30.3	43.8	20.3
Egypt	Minuf	Count	2000	0	0	0
Egypt	Minuf	Count	2017	0	0	0
Egypt	Bani Mazar	Proportion	2000	38.9	47.9	29.9
Egypt	Bani Mazar	Proportion	2017	30.6	42.8	18.4
Egypt	Bani Mazar	Count	2000	0	0	0
Egypt	Bani Mazar	Count	2017	0	0	0
Egypt	Al-Azbakiyah	Proportion	2000	30.5	37.0	24.2
Egypt	Al-Azbakiyah	Proportion	2017	26.2	38.4	17.0
Egypt	Al-Azbakiyah	Count	2000	0	0	0
Egypt	Al-Azbakiyah	Count	2017	0	0	0
Egypt	Banha	Proportion	2000	33.8	41.6	26.2
Egypt	Banha	Proportion	2017	29.5	42.7	17.1
Egypt	Banha	Count	2000	0	0	0
Egypt	Banha	Count	2017	0	0	0
Egypt	Port Suez Police Department	Proportion	2000	31.5	43.8	19.3
Egypt	Port Suez Police Department	Proportion	2017	28.4	42.9	17.0

Table 4: ORS by Unit *(continued)*

Country	Unit	Metric	Year	Mean	Upper	Lower
Egypt	Port Suez Police Department	Count	2000	0	0	0
Egypt	Port Suez Police Department	Count	2017	0	0	0
Egypt	Al-Husayniyah	Proportion	2000	38.4	46.7	29.9
Egypt	Al-Husayniyah	Proportion	2017	31.0	45.3	19.1
Egypt	Al-Husayniyah	Count	2000	0	0	0
Egypt	Al-Husayniyah	Count	2017	0	0	0
Egypt	Idfu	Proportion	2000	28.0	37.9	19.0
Egypt	Idfu	Proportion	2017	30.4	44.4	18.2
Egypt	Idfu	Count	2000	0	0	0
Egypt	Idfu	Count	2017	0	0	0
Egypt	Al-Ghanayim	Proportion	2000	32.6	41.3	24.6
Egypt	Al-Ghanayim	Proportion	2017	29.6	46.0	17.4
Egypt	Al-Ghanayim	Count	2000	0	0	0
Egypt	Al-Ghanayim	Count	2017	0	0	0
Egypt	Biba	Proportion	2000	41.6	50.9	32.2
Egypt	Biba	Proportion	2017	28.6	43.0	17.4
Egypt	Biba	Count	2000	0	1	0
Egypt	Biba	Count	2017	0	0	0
Egypt	Al-Manakh	Proportion	2000	35.8	46.1	26.6
Egypt	Al-Manakh	Proportion	2017	25.9	40.3	16.1
Egypt	Al-Manakh	Count	2000	0	0	0
Egypt	Al-Manakh	Count	2017	0	0	0
Egypt	Kafr Sa'd	Proportion	2000	30.3	41.0	22.2
Egypt	Kafr Sa'd	Proportion	2017	20.3	34.7	10.7
Egypt	Kafr Sa'd	Count	2000	0	0	0
Egypt	Kafr Sa'd	Count	2017	0	0	0
Egypt	Nuweiba'	Proportion	2000	29.6	41.2	19.0
Egypt	Nuweiba'	Proportion	2017	29.1	41.9	18.0
Egypt	Nuweiba'	Count	2000	0	0	0
Egypt	Nuweiba'	Count	2017	0	0	0
Egypt	Disuq	Proportion	2000	32.9	41.0	25.3
Egypt	Disuq	Proportion	2017	31.8	46.0	21.4
Egypt	Disuq	Count	2000	0	0	0
Egypt	Disuq	Count	2017	0	0	0
Egypt	Marsa Matruh	Proportion	2000	26.3	36.3	17.3
Egypt	Marsa Matruh	Proportion	2017	28.1	40.7	17.9
Egypt	Marsa Matruh	Count	2000	0	0	0
Egypt	Marsa Matruh	Count	2017	0	0	0
Egypt	Farshut	Proportion	2000	32.3	41.8	25.1
Egypt	Farshut	Proportion	2017	28.0	40.3	16.6
Egypt	Farshut	Count	2000	0	0	0
Egypt	Farshut	Count	2017	0	0	0
Egypt	Al-Hasanah	Proportion	2000	28.6	39.2	18.0
Egypt	Al-Hasanah	Proportion	2017	27.5	38.9	17.4
Egypt	Al-Hasanah	Count	2000	0	0	0
Egypt	Al-Hasanah	Count	2017	0	0	0
Egypt	Al-Manshah	Proportion	2000	36.1	48.2	26.5

Table 4: ORS by Unit *(continued)*

Country	Unit	Metric	Year	Mean	Upper	Lower
Egypt	Al-Manshah	Proportion	2017	25.6	37.7	15.0
Egypt	Al-Manshah	Count	2000	0	0	0
Egypt	Al-Manshah	Count	2017	0	0	0
Egypt	Al-Manzilah	Proportion	2000	40.9	50.5	32.8
Egypt	Al-Manzilah	Proportion	2017	31.2	46.7	19.8
Egypt	Al-Manzilah	Count	2000	0	1	0
Egypt	Al-Manzilah	Count	2017	0	0	0
Egypt	Ras Gharib	Proportion	2000	31.2	42.6	21.4
Egypt	Ras Gharib	Proportion	2017	28.1	42.7	17.2
Egypt	Ras Gharib	Count	2000	0	0	0
Egypt	Ras Gharib	Count	2017	0	0	0
Egypt	Badr	Proportion	2000	34.0	42.2	25.5
Egypt	Badr	Proportion	2017	30.5	45.4	20.9
Egypt	Badr	Count	2000	0	0	0
Egypt	Badr	Count	2017	0	0	0
Egypt	Tamiyah	Proportion	2000	37.4	46.1	29.6
Egypt	Tamiyah	Proportion	2017	29.1	42.3	18.1
Egypt	Tamiyah	Count	2000	0	0	0
Egypt	Tamiyah	Count	2017	0	0	0
Egypt	Kafr az-Zayyat	Proportion	2000	34.6	42.8	26.3
Egypt	Kafr az-Zayyat	Proportion	2017	31.5	44.3	21.2
Egypt	Kafr az-Zayyat	Count	2000	0	0	0
Egypt	Kafr az-Zayyat	Count	2017	0	0	0
Egypt	Al-Manshiyah	Proportion	2000	22.9	31.4	15.8
Egypt	Al-Manshiyah	Proportion	2017	26.3	41.7	16.2
Egypt	Al-Manshiyah	Count	2000	0	0	0
Egypt	Al-Manshiyah	Count	2017	0	0	0
Egypt	Ismailia 1	Proportion	2000	31.0	40.5	21.7
Egypt	Ismailia 1	Proportion	2017	27.7	40.8	16.5
Egypt	Ismailia 1	Count	2000	0	0	0
Egypt	Ismailia 1	Count	2017	0	0	0
Egypt	Al-Badrashayn	Proportion	2000	31.5	39.4	24.5
Egypt	Al-Badrashayn	Proportion	2017	27.8	43.7	17.0
Egypt	Al-Badrashayn	Count	2000	0	0	0
Egypt	Al-Badrashayn	Count	2017	0	0	0
Egypt	Minuf City	Proportion	2000	35.4	44.0	27.6
Egypt	Minuf City	Proportion	2017	30.6	44.0	20.4
Egypt	Minuf City	Count	2000	0	0	0
Egypt	Minuf City	Count	2017	0	0	0
Egypt	Dayr Mawas	Proportion	2000	33.3	43.2	25.0
Egypt	Dayr Mawas	Proportion	2017	29.7	44.7	17.0
Egypt	Dayr Mawas	Count	2000	0	0	0
Egypt	Dayr Mawas	Count	2017	0	0	0
Egypt	Al-Basatin	Proportion	2000	31.3	38.8	24.9
Egypt	Al-Basatin	Proportion	2017	28.3	42.2	18.4
Egypt	Al-Basatin	Count	2000	0	0	0
Egypt	Al-Basatin	Count	2017	0	0	0
Egypt	Kafr Shukr	Proportion	2000	35.1	42.2	27.9
Egypt	Kafr Shukr	Proportion	2017	29.4	41.6	17.7

Table 4: ORS by Unit *(continued)*

Country	Unit	Metric	Year	Mean	Upper	Lower
Egypt	Kafr Shukr	Count	2000	0	0	0
Egypt	Kafr Shukr	Count	2017	0	0	0
Egypt	Suez	Proportion	2000	31.6	44.4	19.4
Egypt	Suez	Proportion	2017	28.4	43.5	17.0
Egypt	Suez	Count	2000	0	0	0
Egypt	Suez	Count	2017	0	0	0
Egypt	Al-Ibrahimiyah	Proportion	2000	38.9	46.7	30.8
Egypt	Al-Ibrahimiyah	Proportion	2017	31.5	47.2	19.1
Egypt	Al-Ibrahimiyah	Count	2000	0	0	0
Egypt	Al-Ibrahimiyah	Count	2017	0	0	0
Egypt	Kawm Umbu	Proportion	2000	29.8	39.6	21.0
Egypt	Kawm Umbu	Proportion	2017	30.7	45.4	18.0
Egypt	Kawm Umbu	Count	2000	0	0	0
Egypt	Kawm Umbu	Count	2017	0	0	0
Egypt	Al-Qusiyah	Proportion	2000	33.4	42.6	25.8
Egypt	Al-Qusiyah	Proportion	2017	29.6	44.2	16.6
Egypt	Al-Qusiyah	Count	2000	0	0	0
Egypt	Al-Qusiyah	Count	2017	0	0	0
Egypt	Ihnasiya	Proportion	2000	42.4	51.6	33.5
Egypt	Ihnasiya	Proportion	2017	29.0	44.2	18.2
Egypt	Ihnasiya	Count	2000	0	1	0
Egypt	Ihnasiya	Count	2017	0	0	0
Egypt	Al-Manasrah	Proportion	2000	36.5	46.2	27.4
Egypt	Al-Manasrah	Proportion	2017	26.8	41.0	16.8
Egypt	Al-Manasrah	Count	2000	0	0	0
Egypt	Al-Manasrah	Count	2017	0	0	0
Egypt	New Damietta City	Proportion	2000	38.6	48.8	29.3
Egypt	New Damietta City	Proportion	2017	25.3	41.0	14.0
Egypt	New Damietta City	Count	2000	0	0	0
Egypt	New Damietta City	Count	2017	0	0	0
Egypt	Ras Sidr	Proportion	2000	29.9	43.6	19.4
Egypt	Ras Sidr	Proportion	2017	27.2	37.4	16.9
Egypt	Ras Sidr	Count	2000	0	0	0
Egypt	Ras Sidr	Count	2017	0	0	0
Egypt	Fuwah	Proportion	2000	31.2	39.9	23.1
Egypt	Fuwah	Proportion	2017	32.0	46.1	20.6
Egypt	Fuwah	Count	2000	0	0	0
Egypt	Fuwah	Count	2017	0	0	0
Egypt	Sidi Barrani	Proportion	2000	24.5	35.4	14.5
Egypt	Sidi Barrani	Proportion	2017	27.6	40.4	17.4
Egypt	Sidi Barrani	Count	2000	0	0	0
Egypt	Sidi Barrani	Count	2017	0	0	0
Egypt	Isna	Proportion	2000	29.3	39.7	19.2
Egypt	Isna	Proportion	2017	30.2	43.4	17.9
Egypt	Isna	Count	2000	0	0	0
Egypt	Isna	Count	2017	0	0	0
Egypt	Bir al-'Abd	Proportion	2000	30.5	40.6	20.7
Egypt	Bir al-'Abd	Proportion	2017	28.3	41.0	17.4
Egypt	Bir al-'Abd	Count	2000	0	0	0

Table 4: ORS by Unit *(continued)*

Country	Unit	Metric	Year	Mean	Upper	Lower
Egypt	Bir al-'Abd	Count	2017	0	0	0
Egypt	Al-Maraghah	Proportion	2000	35.8	45.6	28.3
Egypt	Al-Maraghah	Proportion	2017	26.4	40.0	15.8
Egypt	Al-Maraghah	Count	2000	0	0	0
Egypt	Al-Maraghah	Count	2017	0	0	0
Egypt	Al-Matariyah	Proportion	2000	40.9	51.4	31.8
Egypt	Al-Matariyah	Proportion	2017	30.9	46.7	19.4
Egypt	Al-Matariyah	Count	2000	0	1	0
Egypt	Al-Matariyah	Count	2017	0	0	0
Egypt	Safaja	Proportion	2000	29.0	41.1	19.1
Egypt	Safaja	Proportion	2017	27.1	41.2	17.5
Egypt	Safaja	Count	2000	0	0	0
Egypt	Safaja	Count	2017	0	0	0
Egypt	Damanhur	Proportion	2000	30.5	39.5	22.6
Egypt	Damanhur	Proportion	2017	31.8	45.4	20.5
Egypt	Damanhur	Count	2000	0	0	0
Egypt	Damanhur	Count	2017	0	0	0
Egypt	Unorganized in Al Fayoum	Proportion	2000	38.1	46.5	30.1
Egypt	Unorganized in Al Fayoum	Proportion	2017	28.2	41.3	19.3
Egypt	Unorganized in Al Fayoum	Count	2000	0	0	0
Egypt	Unorganized in Al Fayoum	Count	2017	0	0	0
Egypt	Qutur	Proportion	2000	37.1	45.5	29.1
Egypt	Qutur	Proportion	2017	33.0	46.7	21.2
Egypt	Qutur	Count	2000	0	0	0
Egypt	Qutur	Count	2017	0	0	0
Egypt	Al-Muntazah	Proportion	2000	25.1	33.8	18.2
Egypt	Al-Muntazah	Proportion	2017	32.2	49.0	20.4
Egypt	Al-Muntazah	Count	2000	0	0	0
Egypt	Al-Muntazah	Count	2017	0	0	0
Egypt	Ismailia 2	Proportion	2000	31.1	40.8	21.7
Egypt	Ismailia 2	Proportion	2017	27.7	40.9	16.5
Egypt	Ismailia 2	Count	2000	0	0	0
Egypt	Ismailia 2	Count	2017	0	0	0
Egypt	Al-Hawamidiyah	Proportion	2000	28.6	36.4	21.8
Egypt	Al-Hawamidiyah	Proportion	2017	23.2	38.0	13.7
Egypt	Al-Hawamidiyah	Count	2000	0	0	0
Egypt	Al-Hawamidiyah	Count	2017	0	0	0
Egypt	Quwaysina	Proportion	2000	35.1	42.9	27.2
Egypt	Quwaysina	Proportion	2017	30.0	42.9	18.4
Egypt	Quwaysina	Count	2000	0	0	0
Egypt	Quwaysina	Count	2017	0	0	0
Egypt	Maghaghah	Proportion	2000	40.3	49.9	31.5
Egypt	Maghaghah	Proportion	2017	30.4	42.9	18.1
Egypt	Maghaghah	Count	2000	0	0	0
Egypt	Maghaghah	Count	2017	0	0	0
Egypt	Al-Jamaliyah	Proportion	2000	29.6	36.2	23.2
Egypt	Al-Jamaliyah	Proportion	2017	23.1	35.6	14.3
Egypt	Al-Jamaliyah	Count	2000	0	0	0
Egypt	Al-Jamaliyah	Count	2017	0	0	0

Table 4: ORS by Unit *(continued)*

Country	Unit	Metric	Year	Mean	Upper	Lower
Egypt	Qaha	Proportion	2000	32.5	39.9	26.2
Egypt	Qaha	Proportion	2017	29.0	41.7	18.0
Egypt	Qaha	Count	2000	0	0	0
Egypt	Qaha	Count	2017	0	0	0
Egypt	Al-Qanayat	Proportion	2000	37.3	44.6	29.9
Egypt	Al-Qanayat	Proportion	2017	31.4	45.9	19.1
Egypt	Al-Qanayat	Count	2000	0	0	0
Egypt	Al-Qanayat	Count	2017	0	0	0
Egypt	Nasr	Proportion	2000	30.2	39.9	21.5
Egypt	Nasr	Proportion	2017	30.9	45.7	18.1
Egypt	Nasr	Count	2000	0	0	0
Egypt	Nasr	Count	2017	0	0	0
Egypt	Asyut	Proportion	2000	31.7	39.7	23.8
Egypt	Asyut	Proportion	2017	29.4	44.1	16.2
Egypt	Asyut	Count	2000	0	0	0
Egypt	Asyut	Count	2017	0	0	0
Egypt	Nasir Bush	Proportion	2000	42.3	51.3	34.4
Egypt	Nasir Bush	Proportion	2017	30.6	44.2	18.8
Egypt	Nasir Bush	Count	2000	0	1	0
Egypt	Nasir Bush	Count	2017	0	0	0
Egypt	Ash-Sharq	Proportion	2000	37.2	47.9	27.4
Egypt	Ash-Sharq	Proportion	2017	28.0	42.5	17.5
Egypt	Ash-Sharq	Count	2000	0	0	0
Egypt	Ash-Sharq	Count	2017	0	0	0
Egypt	Port of Damietta Police Department	Proportion	2000	42.3	53.8	31.8
Egypt	Port of Damietta Police Department	Proportion	2017	27.3	44.4	15.9
Egypt	Port of Damietta Police Department	Count	2000	0	1	0
Egypt	Port of Damietta Police Department	Count	2017	0	0	0
Egypt	Sant Katrin	Proportion	2000	32.8	45.1	20.6
Egypt	Sant Katrin	Proportion	2017	30.9	43.8	20.3
Egypt	Sant Katrin	Count	2000	0	0	0
Egypt	Sant Katrin	Count	2017	0	0	0
Egypt	Kafr ash-Shaykh	Proportion	2000	36.5	45.2	29.2
Egypt	Kafr ash-Shaykh	Proportion	2017	31.9	46.4	20.1
Egypt	Kafr ash-Shaykh	Count	2000	0	0	0
Egypt	Kafr ash-Shaykh	Count	2017	0	0	0
Egypt	Siwa	Proportion	2000	27.8	41.9	16.8
Egypt	Siwa	Proportion	2017	26.6	40.2	16.1
Egypt	Siwa	Count	2000	0	0	0
Egypt	Siwa	Count	2017	0	0	0
Egypt	Naj' Hammadi	Proportion	2000	31.9	41.2	24.3
Egypt	Naj' Hammadi	Proportion	2017	27.8	40.3	16.4
Egypt	Naj' Hammadi	Count	2000	0	0	0
Egypt	Naj' Hammadi	Count	2017	0	0	0
Egypt	Nakhl	Proportion	2000	28.0	38.8	18.7

Table 4: ORS by Unit *(continued)*

Country	Unit	Metric	Year	Mean	Upper	Lower
Egypt	Nakhl	Proportion	2017	27.0	36.8	17.5
Egypt	Nakhl	Count	2000	0	0	0
Egypt	Nakhl	Count	2017	0	0	0
Egypt	Dar as-Salam	Proportion	2000	34.7	45.2	27.5
Egypt	Dar as-Salam	Proportion	2017	26.7	39.5	15.7
Egypt	Dar as-Salam	Count	2000	0	0	0
Egypt	Dar as-Salam	Count	2017	0	0	0
Egypt	As-Sinbillawayn	Proportion	2000	39.2	46.5	31.9
Egypt	As-Sinbillawayn	Proportion	2017	30.6	44.6	19.1
Egypt	As-Sinbillawayn	Count	2000	0	0	0
Egypt	As-Sinbillawayn	Count	2017	0	0	0
Egypt	Hawsh 'Isa	Proportion	2000	31.0	40.0	23.5
Egypt	Hawsh 'Isa	Proportion	2017	32.2	45.0	21.4
Egypt	Hawsh 'Isa	Count	2000	0	0	0
Egypt	Hawsh 'Isa	Count	2017	0	0	0
Egypt	Yusuf as-Sidiq	Proportion	2000	39.6	49.7	29.4
Egypt	Yusuf as-Sidiq	Proportion	2017	27.8	42.4	18.1
Egypt	Yusuf as-Sidiq	Count	2000	0	0	0
Egypt	Yusuf as-Sidiq	Count	2017	0	0	0
Egypt	Samannud	Proportion	2000	38.9	46.1	30.8
Egypt	Samannud	Proportion	2017	31.0	43.6	19.3
Egypt	Samannud	Count	2000	0	0	0
Egypt	Samannud	Count	2017	0	0	0
Egypt	Ar-Raml 1	Proportion	2000	23.4	31.8	16.4
Egypt	Ar-Raml 1	Proportion	2017	27.6	43.8	17.0
Egypt	Ar-Raml 1	Count	2000	0	0	0
Egypt	Ar-Raml 1	Count	2017	0	0	0
Egypt	Ismailia 3	Proportion	2000	31.0	40.6	21.6
Egypt	Ismailia 3	Proportion	2017	27.6	40.8	16.4
Egypt	Ismailia 3	Count	2000	0	0	0
Egypt	Ismailia 3	Count	2017	0	0	0
Egypt	Al-Wahat al-Bahariyah	Proportion	2000	31.9	46.8	20.0
Egypt	Al-Wahat al-Bahariyah	Proportion	2017	29.4	42.6	17.3
Egypt	Al-Wahat al-Bahariyah	Count	2000	0	0	0
Egypt	Al-Wahat al-Bahariyah	Count	2017	0	0	0
Egypt	Sadat City	Proportion	2000	33.2	41.8	24.9
Egypt	Sadat City	Proportion	2017	28.7	42.2	18.9
Egypt	Sadat City	Count	2000	0	0	0
Egypt	Sadat City	Count	2017	0	0	0
Egypt	Mallawi	Proportion	2000	33.7	43.2	25.1
Egypt	Mallawi	Proportion	2017	29.7	43.9	17.3
Egypt	Mallawi	Count	2000	0	0	0
Egypt	Mallawi	Count	2017	0	0	0
Egypt	Al-Khalifa	Proportion	2000	31.1	38.2	24.5
Egypt	Al-Khalifa	Proportion	2017	28.1	41.1	18.3
Egypt	Al-Khalifa	Count	2000	0	0	0
Egypt	Al-Khalifa	Count	2017	0	0	0
Egypt	Qalyub	Proportion	2000	31.5	39.3	25.1
Egypt	Qalyub	Proportion	2017	28.1	41.4	17.9

Table 4: ORS by Unit *(continued)*

Country	Unit	Metric	Year	Mean	Upper	Lower
Egypt	Qalyub	Count	2000	0	0	0
Egypt	Qalyub	Count	2017	0	0	0
Egypt	Al-Qurayn	Proportion	2000	37.3	44.2	29.1
Egypt	Al-Qurayn	Proportion	2017	30.2	46.0	17.9
Egypt	Al-Qurayn	Count	2000	0	0	0
Egypt	Al-Qurayn	Count	2017	0	0	0
Egypt	New Tushka City	Proportion	2000	26.1	39.7	14.7
Egypt	New Tushka City	Proportion	2017	28.9	41.7	17.4
Egypt	New Tushka City	Count	2000	0	0	0
Egypt	New Tushka City	Count	2017	0	0	0
Egypt	Asyut 1	Proportion	2000	30.6	39.5	22.6
Egypt	Asyut 1	Proportion	2017	29.1	44.6	15.6
Egypt	Asyut 1	Count	2000	0	0	0
Egypt	Asyut 1	Count	2017	0	0	0
Egypt	Sumusta al-Waqf	Proportion	2000	42.4	52.3	32.8
Egypt	Sumusta al-Waqf	Proportion	2017	28.8	43.3	17.6
Egypt	Sumusta al-Waqf	Count	2000	0	1	0
Egypt	Sumusta al-Waqf	Count	2017	0	0	0
Egypt	Az-Zohur	Proportion	2000	35.8	46.0	26.5
Egypt	Az-Zohur	Proportion	2017	25.8	40.3	16.1
Egypt	Az-Zohur	Count	2000	0	0	0
Egypt	Az-Zohur	Count	2017	0	0	0
Egypt	Zarqa	Proportion	2000	36.5	44.1	28.7
Egypt	Zarqa	Proportion	2017	25.1	40.9	13.7
Egypt	Zarqa	Count	2000	0	0	0
Egypt	Zarqa	Count	2017	0	0	0
Egypt	Sharm el-Sheikh	Proportion	2000	29.6	41.4	18.4
Egypt	Sharm el-Sheikh	Proportion	2017	25.7	40.9	15.4
Egypt	Sharm el-Sheikh	Count	2000	0	0	0
Egypt	Sharm el-Sheikh	Count	2017	0	0	0
Egypt	Mutubis	Proportion	2000	29.7	38.4	21.6
Egypt	Mutubis	Proportion	2017	31.7	46.1	20.0
Egypt	Mutubis	Count	2000	0	0	0
Egypt	Mutubis	Count	2017	0	0	0
Egypt	Naqadah	Proportion	2000	28.8	38.5	20.8
Egypt	Naqadah	Proportion	2017	28.7	43.6	17.5
Egypt	Naqadah	Count	2000	0	0	0
Egypt	Naqadah	Count	2017	0	0	0
Egypt	Rafah	Proportion	2000	26.3	40.4	15.6
Egypt	Rafah	Proportion	2017	28.8	43.3	16.7
Egypt	Rafah	Count	2000	0	0	0
Egypt	Rafah	Count	2017	0	0	0
Egypt	Jirja	Proportion	2000	35.2	46.0	27.6
Egypt	Jirja	Proportion	2017	26.1	38.0	15.0
Egypt	Jirja	Count	2000	0	0	0
Egypt	Jirja	Count	2017	0	0	0
Egypt	Bani Ubayd	Proportion	2000	40.8	48.1	33.4
Egypt	Bani Ubayd	Proportion	2017	31.4	45.4	19.7
Egypt	Bani Ubayd	Count	2000	0	0	0

Table 4: ORS by Unit *(continued)*

Country	Unit	Metric	Year	Mean	Upper	Lower
Egypt	Bani Ubayd	Count	2017	0	0	0
Egypt	Idku	Proportion	2000	27.5	36.2	19.0
Egypt	Idku	Proportion	2017	32.3	48.2	19.4
Egypt	Idku	Count	2000	0	0	0
Egypt	Idku	Count	2017	0	0	0
Egypt	Tanta	Proportion	2000	36.0	43.3	28.2
Egypt	Tanta	Proportion	2017	31.9	45.8	20.3
Egypt	Tanta	Count	2000	0	0	0
Egypt	Tanta	Count	2017	0	0	0
Egypt	Ar-Raml 2	Proportion	2000	23.7	32.1	16.8
Egypt	Ar-Raml 2	Proportion	2017	28.8	44.8	18.1
Egypt	Ar-Raml 2	Count	2000	0	0	0
Egypt	Ar-Raml 2	Count	2017	0	0	0
Egypt	Al-Warraq	Proportion	2000	31.0	37.8	24.7
Egypt	Al-Warraq	Proportion	2017	27.2	39.7	18.0
Egypt	Al-Warraq	Count	2000	0	0	0
Egypt	Al-Warraq	Count	2017	0	0	0
Egypt	Shibin al-Kawm	Proportion	2000	34.3	42.5	26.5
Egypt	Shibin al-Kawm	Proportion	2017	29.4	42.6	18.6
Egypt	Shibin al-Kawm	Count	2000	0	0	0
Egypt	Shibin al-Kawm	Count	2017	0	0	0
Egypt	Mallawi City	Proportion	2000	32.5	42.0	24.2
Egypt	Mallawi City	Proportion	2017	29.1	43.1	16.3
Egypt	Mallawi City	Count	2000	0	0	0
Egypt	Mallawi City	Count	2017	0	0	0
Egypt	Al-Ma'adi	Proportion	2000	31.5	38.9	25.0
Egypt	Al-Ma'adi	Proportion	2017	28.3	42.4	18.5
Egypt	Al-Ma'adi	Count	2000	0	0	0
Egypt	Al-Ma'adi	Count	2017	0	0	0
Egypt	Shibin al-Qanatir	Proportion	2000	33.0	40.0	26.3
Egypt	Shibin al-Qanatir	Proportion	2017	29.6	42.8	18.8
Egypt	Shibin al-Qanatir	Count	2000	0	0	0
Egypt	Shibin al-Qanatir	Count	2017	0	0	0
Egypt	Awlad Saqr	Proportion	2000	40.4	47.8	33.1
Egypt	Awlad Saqr	Proportion	2017	31.7	45.6	20.2
Egypt	Awlad Saqr	Count	2000	0	0	0
Egypt	Awlad Saqr	Count	2017	0	0	0
Egypt	Unorganized in Aswan	Proportion	2000	28.7	37.4	21.5
Egypt	Unorganized in Aswan	Proportion	2017	30.1	43.2	18.5
Egypt	Unorganized in Aswan	Count	2000	0	0	0
Egypt	Unorganized in Aswan	Count	2017	0	0	0
Egypt	Asyut 2	Proportion	2000	31.2	40.1	23.3
Egypt	Asyut 2	Proportion	2017	28.8	44.3	15.2
Egypt	Asyut 2	Count	2000	0	0	0
Egypt	Asyut 2	Count	2017	0	0	0
Egypt	Unorganized in Bani Suwayf	Proportion	2000	42.1	50.8	34.7
Egypt	Unorganized in Bani Suwayf	Proportion	2017	30.3	44.8	19.7

Table 4: ORS by Unit *(continued)*

Country	Unit	Metric	Year	Mean	Upper	Lower
Egypt	Unorganized in Bani Suwayf	Count	2000	0	1	0
Egypt	Unorganized in Bani Suwayf	Count	2017	0	0	0
Egypt	Mubarak - Sharq at-Tafri'tah	Proportion	2000	34.6	43.9	24.9
Egypt	Mubarak - Sharq at-Tafri'tah	Proportion	2017	29.4	43.4	18.8
Egypt	Mubarak - Sharq at-Tafri'tah	Count	2000	0	0	0
Egypt	Mubarak - Sharq at-Tafri'tah	Count	2017	0	0	0
Egypt	Taba	Proportion	2000	27.3	40.6	15.9
Egypt	Taba	Proportion	2017	28.5	43.8	17.0
Egypt	Taba	Count	2000	0	0	0
Egypt	Taba	Count	2017	0	0	0
Egypt	Qallin	Proportion	2000	34.8	43.4	26.6
Egypt	Qallin	Proportion	2017	31.8	46.1	20.7
Egypt	Qallin	Count	2000	0	0	0
Egypt	Qallin	Count	2017	0	0	0
Egypt	Qift	Proportion	2000	29.6	39.6	20.8
Egypt	Qift	Proportion	2017	29.6	44.7	18.0
Egypt	Qift	Count	2000	0	0	0
Egypt	Qift	Count	2017	0	0	0
Egypt	Sheikh Zawid	Proportion	2000	27.3	41.5	17.3
Egypt	Sheikh Zawid	Proportion	2017	27.9	40.7	16.6
Egypt	Sheikh Zawid	Count	2000	0	0	0
Egypt	Sheikh Zawid	Count	2017	0	0	0
Egypt	Juhaynah al-Gharbiyah	Proportion	2000	35.2	45.0	26.5
Egypt	Juhaynah al-Gharbiyah	Proportion	2017	27.1	41.3	15.5
Egypt	Juhaynah al-Gharbiyah	Count	2000	0	0	0
Egypt	Juhaynah al-Gharbiyah	Count	2017	0	0	0
Egypt	Bilqas	Proportion	2000	41.3	49.5	34.1
Egypt	Bilqas	Proportion	2017	30.4	45.0	19.1
Egypt	Bilqas	Count	2000	0	0	0
Egypt	Bilqas	Count	2017	0	0	0
Egypt	Ityay al-Barud	Proportion	2000	32.9	41.3	25.1
Egypt	Ityay al-Barud	Proportion	2017	31.0	42.9	21.0
Egypt	Ityay al-Barud	Count	2000	0	0	0
Egypt	Ityay al-Barud	Count	2017	0	0	0
Egypt	Tanta 1	Proportion	2000	35.8	43.0	27.6
Egypt	Tanta 1	Proportion	2017	31.5	45.1	19.9
Egypt	Tanta 1	Count	2000	0	0	0
Egypt	Tanta 1	Count	2017	0	0	0
Egypt	Bab Sharqi	Proportion	2000	24.2	33.1	17.1
Egypt	Bab Sharqi	Proportion	2017	30.9	47.6	19.4
Egypt	Bab Sharqi	Count	2000	0	0	0
Egypt	Bab Sharqi	Count	2017	0	0	0
Egypt	As-Saff	Proportion	2000	34.9	43.6	27.4
Egypt	As-Saff	Proportion	2017	29.6	44.2	19.0

Table 4: ORS by Unit *(continued)*

Country	Unit	Metric	Year	Mean	Upper	Lower
Egypt	As-Saff	Count	2000	0	0	0
Egypt	As-Saff	Count	2017	0	0	0
Egypt	Sirs al-Layyanah	Proportion	2000	34.0	42.5	26.5
Egypt	Sirs al-Layyanah	Proportion	2017	29.7	43.0	19.2
Egypt	Sirs al-Layyanah	Count	2000	0	0	0
Egypt	Sirs al-Layyanah	Count	2017	0	0	0
Egypt	Matay	Proportion	2000	37.8	47.5	28.4
Egypt	Matay	Proportion	2017	31.0	45.2	18.6
Egypt	Matay	Count	2000	0	0	0
Egypt	Matay	Count	2017	0	0	0
Egypt	Al-Marj	Proportion	2000	31.4	38.9	24.1
Egypt	Al-Marj	Proportion	2017	27.2	40.7	17.2
Egypt	Al-Marj	Count	2000	0	0	0
Egypt	Al-Marj	Count	2017	0	0	0
Egypt	Shubra al-Khaymah 1	Proportion	2000	29.9	37.5	23.5
Egypt	Shubra al-Khaymah 1	Proportion	2017	25.2	38.1	15.9
Egypt	Shubra al-Khaymah 1	Count	2000	0	0	0
Egypt	Shubra al-Khaymah 1	Count	2017	0	0	0
Egypt	Bilbays	Proportion	2000	35.7	44.0	27.9
Egypt	Bilbays	Proportion	2017	30.4	45.3	19.1
Egypt	Bilbays	Count	2000	0	0	0
Egypt	Bilbays	Count	2017	0	0	0
Egypt	Dayrut	Proportion	2000	33.4	42.6	25.5
Egypt	Dayrut	Proportion	2017	29.8	44.4	16.9
Egypt	Dayrut	Count	2000	0	0	0
Egypt	Dayrut	Count	2017	0	0	0
Egypt	Port Fuad	Proportion	2000	37.3	48.1	27.4
Egypt	Port Fuad	Proportion	2017	30.2	45.3	18.5
Egypt	Port Fuad	Count	2000	0	0	0
Egypt	Port Fuad	Count	2017	0	0	0
Egypt	Sidi Salim	Proportion	2000	34.1	43.7	25.7
Egypt	Sidi Salim	Proportion	2017	32.2	46.5	21.6
Egypt	Sidi Salim	Count	2000	0	0	0
Egypt	Sidi Salim	Count	2017	0	0	0
Egypt	Qina	Proportion	2000	29.2	38.6	21.4
Egypt	Qina	Proportion	2017	28.2	42.9	17.3
Egypt	Qina	Count	2000	0	0	0
Egypt	Qina	Count	2017	0	0	0
Egypt	Shurtah al-Qasimah	Proportion	2000	27.2	38.8	17.4
Egypt	Shurtah al-Qasimah	Proportion	2017	28.4	39.6	18.4
Egypt	Shurtah al-Qasimah	Count	2000	0	0	0
Egypt	Shurtah al-Qasimah	Count	2017	0	0	0
Egypt	New Akhmim City	Proportion	2000	37.6	49.4	27.7
Egypt	New Akhmim City	Proportion	2017	26.9	39.7	15.5
Egypt	New Akhmim City	Count	2000	0	0	0
Egypt	New Akhmim City	Count	2017	0	0	0
Egypt	Dikirnis	Proportion	2000	41.1	49.0	33.7
Egypt	Dikirnis	Proportion	2017	30.9	45.5	18.8
Egypt	Dikirnis	Count	2000	0	0	0

Table 4: ORS by Unit *(continued)*

Country	Unit	Metric	Year	Mean	Upper	Lower
Egypt	Dikirnis	Count	2017	0	0	0
Egypt	Kafr ad-Dawwar	Proportion	2000	26.0	35.2	19.3
Egypt	Kafr ad-Dawwar	Proportion	2017	31.6	47.3	19.7
Egypt	Kafr ad-Dawwar	Count	2000	0	0	0
Egypt	Kafr ad-Dawwar	Count	2017	0	0	0
Egypt	Tanta 2	Proportion	2000	35.3	42.9	27.3
Egypt	Tanta 2	Proportion	2017	31.1	44.8	20.5
Egypt	Tanta 2	Count	2000	0	0	0
Egypt	Tanta 2	Count	2017	0	0	0
Egypt	Burj al-'Arab	Proportion	2000	25.6	36.5	18.0
Egypt	Burj al-'Arab	Proportion	2017	28.6	43.2	16.9
Egypt	Burj al-'Arab	Count	2000	0	0	0
Egypt	Burj al-'Arab	Count	2017	0	0	0
Egypt	Atfih	Proportion	2000	37.5	46.3	30.2
Egypt	Atfih	Proportion	2017	29.3	43.8	17.8
Egypt	Atfih	Count	2000	0	0	0
Egypt	Atfih	Count	2017	0	0	0
Egypt	Tala	Proportion	2000	36.0	44.5	27.9
Egypt	Tala	Proportion	2017	31.6	44.7	20.8
Egypt	Tala	Count	2000	0	0	0
Egypt	Tala	Count	2017	0	0	0
Egypt	New Minya City	Proportion	2000	33.2	42.4	25.1
Egypt	New Minya City	Proportion	2017	29.1	43.1	17.6
Egypt	New Minya City	Count	2000	0	0	0
Egypt	New Minya City	Count	2017	0	0	0
Egypt	Al-Matariyah	Proportion	2000	30.0	37.4	23.3
Egypt	Al-Matariyah	Proportion	2017	25.2	37.4	15.5
Egypt	Al-Matariyah	Count	2000	0	0	0
Egypt	Al-Matariyah	Count	2017	0	0	0
Egypt	Shubra al-Khaymah 2	Proportion	2000	30.0	38.0	23.4
Egypt	Shubra al-Khaymah 2	Proportion	2017	25.4	38.1	15.9
Egypt	Shubra al-Khaymah 2	Count	2000	0	0	0
Egypt	Shubra al-Khaymah 2	Count	2017	0	0	0
Egypt	Diyarb Najm	Proportion	2000	38.3	45.7	30.6
Egypt	Diyarb Najm	Proportion	2017	30.8	44.9	18.9
Egypt	Diyarb Najm	Count	2000	0	0	0
Egypt	Diyarb Najm	Count	2017	0	0	0
Egypt	Manfalut	Proportion	2000	34.0	42.9	25.7
Egypt	Manfalut	Proportion	2017	30.3	46.1	17.0
Egypt	Manfalut	Count	2000	0	0	0
Egypt	Manfalut	Count	2017	0	0	0
Egypt	Port Fuad 2	Proportion	2000	37.2	47.9	27.2
Egypt	Port Fuad 2	Proportion	2017	30.2	45.3	18.5
Egypt	Port Fuad 2	Count	2000	0	0	0
Egypt	Port Fuad 2	Count	2017	0	0	0
Egypt	Qina City	Proportion	2000	28.0	37.8	20.0
Egypt	Qina City	Proportion	2017	28.3	43.6	16.5
Egypt	Qina City	Count	2000	0	0	0
Egypt	Qina City	Count	2017	0	0	0

Table 4: ORS by Unit *(continued)*

Country	Unit	Metric	Year	Mean	Upper	Lower
Egypt	Shurtah Rumanah	Proportion	2000	32.6	43.7	21.3
Egypt	Shurtah Rumanah	Proportion	2017	29.5	45.0	16.6
Egypt	Shurtah Rumanah	Count	2000	0	0	0
Egypt	Shurtah Rumanah	Count	2017	0	0	0
Egypt	New Sawhaj City	Proportion	2000	36.7	48.9	27.0
Egypt	New Sawhaj City	Proportion	2017	26.5	38.2	15.7
Egypt	New Sawhaj City	Count	2000	0	0	0
Egypt	New Sawhaj City	Count	2017	0	0	0
Egypt	Gamsa	Proportion	2000	40.6	50.2	33.0
Egypt	Gamsa	Proportion	2017	28.5	42.6	16.7
Egypt	Gamsa	Count	2000	0	1	0
Egypt	Gamsa	Count	2017	0	0	0
Egypt	Kawm Hamadah	Proportion	2000	34.5	43.2	26.6
Egypt	Kawm Hamadah	Proportion	2017	31.5	45.5	21.2
Egypt	Kawm Hamadah	Count	2000	0	0	0
Egypt	Kawm Hamadah	Count	2017	0	0	0
Egypt	Zifta	Proportion	2000	36.9	44.2	29.9
Egypt	Zifta	Proportion	2017	30.7	42.3	19.0
Egypt	Zifta	Count	2000	0	0	0
Egypt	Zifta	Count	2017	0	0	0
Egypt	Karmuz	Proportion	2000	22.9	31.4	15.8
Egypt	Karmuz	Proportion	2017	26.3	41.7	16.2
Egypt	Karmuz	Count	2000	0	0	0
Egypt	Karmuz	Count	2017	0	0	0
Egypt	Bulaq al-Dakrur	Proportion	2000	29.5	36.3	23.2
Egypt	Bulaq al-Dakrur	Proportion	2017	25.2	38.1	15.9
Egypt	Bulaq al-Dakrur	Count	2000	0	0	0
Egypt	Bulaq al-Dakrur	Count	2017	0	0	0
Egypt	Samalut	Proportion	2000	36.3	47.0	27.2
Egypt	Samalut	Proportion	2017	31.2	44.9	19.0
Egypt	Samalut	Count	2000	0	0	0
Egypt	Samalut	Count	2017	0	0	0
Egypt	Al-Muski	Proportion	2000	29.7	36.3	23.3
Egypt	Al-Muski	Proportion	2017	23.3	36.0	14.5
Egypt	Al-Muski	Count	2000	0	0	0
Egypt	Al-Muski	Count	2017	0	0	0
Egypt	Tukh	Proportion	2000	32.9	40.2	26.1
Egypt	Tukh	Proportion	2017	29.4	42.3	18.2
Egypt	Tukh	Count	2000	0	0	0
Egypt	Tukh	Count	2017	0	0	0
Egypt	Faqus	Proportion	2000	38.1	45.5	30.0
Egypt	Faqus	Proportion	2017	31.3	46.7	18.9
Egypt	Faqus	Count	2000	0	0	0
Egypt	Faqus	Count	2017	0	0	0
Egypt	New Asyut City	Proportion	2000	30.2	39.0	23.0
Egypt	New Asyut City	Proportion	2017	28.7	44.5	15.4
Egypt	New Asyut City	Count	2000	0	0	0
Egypt	New Asyut City	Count	2017	0	0	0

Table 4: ORS by Unit *(continued)*

Country	Unit	Metric	Year	Mean	Upper	Lower
Egypt	Port Sa'id Police Department	Proportion	2000	37.2	47.9	27.4
Egypt	Port Sa'id Police Department	Proportion	2017	28.1	42.7	17.6
Egypt	Port Sa'id Police Department	Count	2000	0	0	0
Egypt	Port Sa'id Police Department	Count	2017	0	0	0
Egypt	Qus	Proportion	2000	29.6	40.0	21.4
Egypt	Qus	Proportion	2017	29.7	44.4	18.1
Egypt	Qus	Count	2000	0	0	0
Egypt	Qus	Count	2017	0	0	0
Egypt	Saqultah	Proportion	2000	35.2	45.1	27.6
Egypt	Saqultah	Proportion	2017	25.9	39.9	15.0
Egypt	Saqultah	Count	2000	0	0	0
Egypt	Saqultah	Count	2017	0	0	0
Egypt	Mahalat Dimnah	Proportion	2000	40.3	47.4	32.7
Egypt	Mahalat Dimnah	Proportion	2017	30.3	44.4	18.8
Egypt	Mahalat Dimnah	Count	2000	0	0	0
Egypt	Mahalat Dimnah	Count	2017	0	0	0
Egypt	Rosetta	Proportion	2000	29.2	37.5	20.9
Egypt	Rosetta	Proportion	2017	32.2	47.3	19.7
Egypt	Rosetta	Count	2000	0	0	0
Egypt	Rosetta	Count	2017	0	0	0
Egypt	Muharam Bik	Proportion	2000	24.3	33.2	17.1
Egypt	Muharam Bik	Proportion	2017	30.9	47.4	19.4
Egypt	Muharam Bik	Count	2000	0	0	0
Egypt	Muharam Bik	Count	2017	0	0	0
Egypt	Giza	Proportion	2000	30.8	37.9	24.4
Egypt	Giza	Proportion	2017	27.4	41.0	17.2
Egypt	Giza	Count	2000	0	0	0
Egypt	Giza	Count	2017	0	0	0
Egypt	Unorganized in Al Minya	Proportion	2000	34.6	42.5	27.5
Egypt	Unorganized in Al Minya	Proportion	2017	29.7	43.2	19.0
Egypt	Unorganized in Al Minya	Count	2000	0	0	0
Egypt	Unorganized in Al Minya	Count	2017	0	0	0
Egypt	Al-Waili	Proportion	2000	29.7	36.4	23.3
Egypt	Al-Waili	Proportion	2017	23.2	35.8	14.3
Egypt	Al-Waili	Count	2000	0	0	0
Egypt	Al-Waili	Count	2017	0	0	0
Egypt	Unorganized in Al Qalyubiyah	Proportion	2000	31.9	38.8	24.8
Egypt	Unorganized in Al Qalyubiyah	Proportion	2017	28.0	42.2	17.6
Egypt	Unorganized in Al Qalyubiyah	Count	2000	0	0	0
Egypt	Unorganized in Al Qalyubiyah	Count	2017	0	0	0
Egypt	Hihya	Proportion	2000	38.6	46.1	30.5
Egypt	Hihya	Proportion	2017	31.6	47.4	19.3

Table 4: ORS by Unit *(continued)*

Country	Unit	Metric	Year	Mean	Upper	Lower
Egypt	Hihya	Count	2000	0	0	0
Egypt	Hihya	Count	2017	0	0	0
Egypt	Sahil Salim	Proportion	2000	32.6	41.2	25.7
Egypt	Sahil Salim	Proportion	2017	29.7	45.9	17.5
Egypt	Sahil Salim	Count	2000	0	0	0
Egypt	Sahil Salim	Count	2017	0	0	0
Egypt	Unorganized in Qina	Proportion	2000	30.6	38.2	24.4
Egypt	Unorganized in Qina	Proportion	2017	28.9	41.4	19.1
Egypt	Unorganized in Qina	Count	2000	0	0	0
Egypt	Unorganized in Qina	Count	2017	0	0	0
Egypt	Sawhaj	Proportion	2000	35.3	46.2	26.8
Egypt	Sawhaj	Proportion	2017	26.1	38.3	15.3
Egypt	Sawhaj	Count	2000	0	0	0
Egypt	Sawhaj	Count	2017	0	0	0
Egypt	Minyat an-Nasr	Proportion	2000	41.1	49.1	33.4
Egypt	Minyat an-Nasr	Proportion	2017	30.6	46.0	18.4
Egypt	Minyat an-Nasr	Count	2000	0	0	0
Egypt	Minyat an-Nasr	Count	2017	0	0	0
Egypt	Shubra Khit	Proportion	2000	33.2	42.0	25.5
Egypt	Shubra Khit	Proportion	2017	31.8	44.9	21.7
Egypt	Shubra Khit	Count	2000	0	0	0
Egypt	Shubra Khit	Count	2017	0	0	0
Egypt	New Burj al-'Arab City	Proportion	2000	27.2	38.6	19.0
Egypt	New Burj al-'Arab City	Proportion	2017	30.4	45.7	18.9
Egypt	New Burj al-'Arab City	Count	2000	0	0	0
Egypt	New Burj al-'Arab City	Count	2017	0	0	0
Egypt	Imbabah	Proportion	2000	32.1	39.8	25.7
Egypt	Imbabah	Proportion	2017	27.7	41.0	18.3
Egypt	Imbabah	Count	2000	0	0	0
Egypt	Imbabah	Count	2017	0	0	0
Egypt	An-Nuzhah	Proportion	2000	32.3	40.2	24.8
Egypt	An-Nuzhah	Proportion	2017	27.6	40.7	18.0
Egypt	An-Nuzhah	Count	2000	0	0	0
Egypt	An-Nuzhah	Count	2017	0	0	0
Egypt	Kafr Saqr	Proportion	2000	39.8	47.6	32.4
Egypt	Kafr Saqr	Proportion	2017	32.2	47.8	19.5
Egypt	Kafr Saqr	Count	2000	0	0	0
Egypt	Kafr Saqr	Count	2017	0	0	0
Egypt	Sidfa	Proportion	2000	33.9	43.0	26.2
Egypt	Sidfa	Proportion	2017	29.8	46.3	17.6
Egypt	Sidfa	Count	2000	0	0	0
Egypt	Sidfa	Count	2017	0	0	0
Egypt	Sawhaj 2	Proportion	2000	34.6	45.5	25.9
Egypt	Sawhaj 2	Proportion	2017	25.7	37.9	15.0
Egypt	Sawhaj 2	Count	2000	0	0	0
Egypt	Sawhaj 2	Count	2017	0	0	0
Egypt	Mit Ghamr	Proportion	2000	36.6	44.0	28.8
Egypt	Mit Ghamr	Proportion	2017	29.5	42.4	18.3
Egypt	Mit Ghamr	Count	2000	0	0	0

Table 4: ORS by Unit *(continued)*

Country	Unit	Metric	Year	Mean	Upper	Lower
Egypt	Mit Ghamr	Count	2017	0	0	0
Egypt	Unorganized in Al Buhayrah	Proportion	2000	31.9	42.9	22.1
Egypt	Unorganized in Al Buhayrah	Proportion	2017	28.5	42.6	16.8
Egypt	Unorganized in Al Buhayrah	Count	2000	0	0	0
Egypt	Unorganized in Al Buhayrah	Count	2017	0	0	0
Egypt	Port al-Basal	Proportion	2000	23.1	31.5	15.9
Egypt	Port al-Basal	Proportion	2017	27.3	42.8	16.9
Egypt	Port al-Basal	Count	2000	0	0	0
Egypt	Port al-Basal	Count	2017	0	0	0
Egypt	Kirdasah	Proportion	2000	31.1	37.9	24.9
Egypt	Kirdasah	Proportion	2017	27.1	38.9	17.5
Egypt	Kirdasah	Count	2000	0	0	0
Egypt	Kirdasah	Count	2017	0	0	0
Egypt	Ancient Cairo	Proportion	2000	31.0	38.1	24.3
Egypt	Ancient Cairo	Proportion	2017	27.5	40.3	17.6
Egypt	Ancient Cairo	Count	2000	0	0	0
Egypt	Ancient Cairo	Count	2017	0	0	0
Egypt	Mashtul as-Suq	Proportion	2000	35.7	43.5	28.0
Egypt	Mashtul as-Suq	Proportion	2017	31.0	45.2	19.5
Egypt	Mashtul as-Suq	Count	2000	0	0	0
Egypt	Mashtul as-Suq	Count	2017	0	0	0
Egypt	Unorganized in Asyut	Proportion	2000	32.0	39.0	25.5
Egypt	Unorganized in Asyut	Proportion	2017	29.1	42.8	16.8
Egypt	Unorganized in Asyut	Count	2000	0	0	0
Egypt	Unorganized in Asyut	Count	2017	0	0	0
Egypt	Tahta	Proportion	2000	33.8	43.1	24.9
Egypt	Tahta	Proportion	2017	26.8	41.7	15.8
Egypt	Tahta	Count	2000	0	0	0
Egypt	Tahta	Count	2017	0	0	0
Egypt	Mit Salsil	Proportion	2000	41.2	49.9	33.5
Egypt	Mit Salsil	Proportion	2017	30.7	46.4	19.0
Egypt	Mit Salsil	Count	2000	0	0	0
Egypt	Mit Salsil	Count	2017	0	0	0
Egypt	Wadi Al-Natron	Proportion	2000	31.7	42.6	21.9
Egypt	Wadi Al-Natron	Proportion	2017	28.3	42.2	16.4
Egypt	Wadi Al-Natron	Count	2000	0	0	0
Egypt	Wadi Al-Natron	Count	2017	0	0	0
Egypt	Port Alexandria Police Department	Proportion	2000	23.4	31.9	16.3
Egypt	Port Alexandria Police Department	Proportion	2017	27.6	43.3	17.0
Egypt	Port Alexandria Police Department	Count	2000	0	0	0
Egypt	Port Alexandria Police Department	Count	2017	0	0	0
Egypt	Sheikh Zayed	Proportion	2000	31.6	39.6	24.2

Table 4: ORS by Unit *(continued)*

Country	Unit	Metric	Year	Mean	Upper	Lower
Egypt	Sheikh Zayed	Proportion	2017	27.8	42.0	17.7
Egypt	Sheikh Zayed	Count	2000	0	0	0
Egypt	Sheikh Zayed	Count	2017	0	0	0
Egypt	As-Sajil	Proportion	2000	30.0	36.9	23.6
Egypt	As-Sajil	Proportion	2017	24.7	37.5	15.4
Egypt	As-Sajil	Count	2000	0	0	0
Egypt	As-Sajil	Count	2017	0	0	0
Egypt	Minya al-Qamh	Proportion	2000	35.8	43.1	28.6
Egypt	Minya al-Qamh	Proportion	2017	30.8	44.9	18.9
Egypt	Minya al-Qamh	Count	2000	0	0	0
Egypt	Minya al-Qamh	Count	2017	0	0	0
Egypt	Tahta City	Proportion	2000	33.4	43.0	24.5
Egypt	Tahta City	Proportion	2017	27.0	42.1	15.8
Egypt	Tahta City	Count	2000	0	0	0
Egypt	Tahta City	Count	2017	0	0	0
Egypt	Nabaruh	Proportion	2000	40.9	49.2	32.8
Egypt	Nabaruh	Proportion	2017	31.5	46.5	19.7
Egypt	Nabaruh	Count	2000	0	0	0
Egypt	Nabaruh	Count	2017	0	0	0
Egypt	West Nubariyah	Proportion	2000	30.1	40.2	21.7
Egypt	West Nubariyah	Proportion	2017	29.4	43.1	19.1
Egypt	West Nubariyah	Count	2000	0	0	0
Egypt	West Nubariyah	Count	2017	0	0	0
Egypt	Sidi Jabir	Proportion	2000	24.1	32.8	17.1
Egypt	Sidi Jabir	Proportion	2017	30.5	46.9	19.1
Egypt	Sidi Jabir	Count	2000	0	0	0
Egypt	Sidi Jabir	Count	2017	0	0	0
Egypt	Sixth of October 1 City	Proportion	2000	32.3	41.0	25.1
Egypt	Sixth of October 1 City	Proportion	2017	27.9	42.3	17.3
Egypt	Sixth of October 1 City	Count	2000	0	0	0
Egypt	Sixth of October 1 City	Count	2017	0	0	0
Egypt	As-Salam	Proportion	2000	32.0	39.6	24.7
Egypt	As-Salam	Proportion	2017	27.9	41.8	18.0
Egypt	As-Salam	Count	2000	0	0	0
Egypt	As-Salam	Count	2017	0	0	0
Egypt	New Salhiyah	Proportion	2000	35.0	42.8	26.8
Egypt	New Salhiyah	Proportion	2017	29.3	44.8	17.5
Egypt	New Salhiyah	Count	2000	0	0	0
Egypt	New Salhiyah	Count	2017	0	0	0
Egypt	Tima	Proportion	2000	34.4	43.4	26.0
Egypt	Tima	Proportion	2017	28.7	44.0	17.2
Egypt	Tima	Count	2000	0	0	0
Egypt	Tima	Count	2017	0	0	0
Egypt	Shirbin	Proportion	2000	38.6	46.3	31.0
Egypt	Shirbin	Proportion	2017	27.1	42.4	16.0
Egypt	Shirbin	Count	2000	0	0	0
Egypt	Shirbin	Count	2017	0	0	0
Egypt	Unorganized in Al Iskandariyah	Proportion	2000	30.6	41.1	22.3

Table 4: ORS by Unit *(continued)*

Country	Unit	Metric	Year	Mean	Upper	Lower
Egypt	Unorganized in Al Iskandariyah	Proportion	2017	31.1	46.6	19.8
Egypt	Unorganized in Al Iskandariyah	Count	2000	0	0	0
Egypt	Unorganized in Al Iskandariyah	Count	2017	0	0	0
Egypt	Sixth of October 2 City	Proportion	2000	32.2	41.7	24.5
Egypt	Sixth of October 2 City	Proportion	2017	28.1	43.6	17.4
Egypt	Sixth of October 2 City	Count	2000	0	0	0
Egypt	Sixth of October 2 City	Count	2017	0	0	0
Egypt	As-Sayidah Zaynab	Proportion	2000	31.1	38.1	24.6
Egypt	As-Sayidah Zaynab	Proportion	2017	27.6	39.9	17.6
Egypt	As-Sayidah Zaynab	Count	2000	0	0	0
Egypt	As-Sayidah Zaynab	Count	2017	0	0	0
Egypt	Unorganized in Ash Sharqiyah	Proportion	2000	33.9	42.1	25.7
Egypt	Unorganized in Ash Sharqiyah	Proportion	2017	28.6	42.7	18.6
Egypt	Unorganized in Ash Sharqiyah	Count	2000	0	0	0
Egypt	Unorganized in Ash Sharqiyah	Count	2017	0	0	0
Egypt	Unorganized in Suhaj	Proportion	2000	34.9	43.9	27.9
Egypt	Unorganized in Suhaj	Proportion	2017	26.3	37.8	16.2
Egypt	Unorganized in Suhaj	Count	2000	0	0	0
Egypt	Unorganized in Suhaj	Count	2017	0	0	0
Egypt	Talkha	Proportion	2000	39.1	45.9	32.0
Egypt	Talkha	Proportion	2017	28.6	41.8	18.2
Egypt	Talkha	Count	2000	0	0	0
Egypt	Talkha	Count	2017	0	0	0
Egypt	Unorganized in Al Jizah	Proportion	2000	33.4	39.4	27.7
Egypt	Unorganized in Al Jizah	Proportion	2017	28.6	41.2	19.4
Egypt	Unorganized in Al Jizah	Count	2000	0	0	0
Egypt	Unorganized in Al Jizah	Count	2017	0	0	0
Egypt	Ash-Sharabiyah	Proportion	2000	29.6	36.4	23.2
Egypt	Ash-Sharabiyah	Proportion	2017	23.0	35.4	14.1
Egypt	Ash-Sharabiyah	Count	2000	0	0	0
Egypt	Ash-Sharabiyah	Count	2017	0	0	0
Egypt	Zaqaziq	Proportion	2000	37.5	44.6	30.0
Egypt	Zaqaziq	Proportion	2017	31.2	45.8	19.2
Egypt	Zaqaziq	Count	2000	0	0	0
Egypt	Zaqaziq	Count	2017	0	0	0
Egypt	Timay al-Imdid	Proportion	2000	40.3	47.3	33.1
Egypt	Timay al-Imdid	Proportion	2017	31.4	46.4	19.7
Egypt	Timay al-Imdid	Count	2000	0	0	0
Egypt	Timay al-Imdid	Count	2017	0	0	0
Egypt	Ash-Shruq	Proportion	2000	34.0	42.3	26.4
Egypt	Ash-Shruq	Proportion	2017	28.3	41.3	18.0
Egypt	Ash-Shruq	Count	2000	0	0	0
Egypt	Ash-Shruq	Count	2017	0	0	0

Table 4: ORS by Unit *(continued)*

Country	Unit	Metric	Year	Mean	Upper	Lower
Egypt	Zaqaziq 1	Proportion	2000	37.3	44.6	29.7
Egypt	Zaqaziq 1	Proportion	2017	31.1	46.2	19.3
Egypt	Zaqaziq 1	Count	2000	0	0	0
Egypt	Zaqaziq 1	Count	2017	0	0	0
Egypt	At-Tebin	Proportion	2000	31.8	39.8	24.5
Egypt	At-Tebin	Proportion	2017	28.9	43.7	18.3
Egypt	At-Tebin	Count	2000	0	0	0
Egypt	At-Tebin	Count	2017	0	0	0
Egypt	Zaqaziq 2	Proportion	2000	37.1	44.4	29.6
Egypt	Zaqaziq 2	Proportion	2017	31.0	46.1	19.2
Egypt	Zaqaziq 2	Count	2000	0	0	0
Egypt	Zaqaziq 2	Count	2017	0	0	0
Egypt	Az-Zahir	Proportion	2000	29.6	36.2	23.2
Egypt	Az-Zahir	Proportion	2017	23.1	35.6	14.3
Egypt	Az-Zahir	Count	2000	0	0	0
Egypt	Az-Zahir	Count	2017	0	0	0
Egypt	Az-Zaytun	Proportion	2000	29.2	36.6	22.8
Egypt	Az-Zaytun	Proportion	2017	23.2	35.7	13.7
Egypt	Az-Zaytun	Count	2000	0	0	0
Egypt	Az-Zaytun	Count	2017	0	0	0
Egypt	Bab ash-Sha'riyah	Proportion	2000	29.6	36.2	23.2
Egypt	Bab ash-Sha'riyah	Proportion	2017	23.1	35.6	14.3
Egypt	Bab ash-Sha'riyah	Count	2000	0	0	0
Egypt	Bab ash-Sha'riyah	Count	2017	0	0	0
Egypt	Badr	Proportion	2000	35.4	45.6	26.6
Egypt	Badr	Proportion	2017	29.7	44.6	19.5
Egypt	Badr	Count	2000	0	0	0
Egypt	Badr	Count	2017	0	0	0
Egypt	Bulaq	Proportion	2000	31.0	37.8	24.9
Egypt	Bulaq	Proportion	2017	27.6	39.5	18.0
Egypt	Bulaq	Count	2000	0	0	0
Egypt	Bulaq	Count	2017	0	0	0
Egypt	Hada'iq al-Qubbah	Proportion	2000	29.6	36.7	23.2
Egypt	Hada'iq al-Qubbah	Proportion	2017	23.0	35.3	13.9
Egypt	Hada'iq al-Qubbah	Count	2000	0	0	0
Egypt	Hada'iq al-Qubbah	Count	2017	0	0	0
Egypt	Heliopolis	Proportion	2000	29.3	36.8	22.6
Egypt	Heliopolis	Proportion	2017	23.5	35.8	13.9
Egypt	Heliopolis	Count	2000	0	0	0
Egypt	Heliopolis	Count	2017	0	0	0
Egypt	Helwan	Proportion	2000	31.2	39.3	23.9
Egypt	Helwan	Proportion	2017	27.8	43.7	17.3
Egypt	Helwan	Count	2000	0	0	0
Egypt	Helwan	Count	2017	0	0	0
Egypt	Monshat Nasr	Proportion	2000	30.0	36.5	23.7
Egypt	Monshat Nasr	Proportion	2017	24.4	36.9	15.5
Egypt	Monshat Nasr	Count	2000	0	0	0
Egypt	Monshat Nasr	Count	2017	0	0	0
Egypt	Nasr City 1	Proportion	2000	32.5	39.6	25.3

Table 4: ORS by Unit *(continued)*

Country	Unit	Metric	Year	Mean	Upper	Lower
Egypt	Nasr City 1	Proportion	2017	27.9	40.4	18.6
Egypt	Nasr City 1	Count	2000	0	0	0
Egypt	Nasr City 1	Count	2017	0	0	0
Egypt	Nasr City 2	Proportion	2000	30.8	37.7	23.9
Egypt	Nasr City 2	Proportion	2017	25.4	37.6	16.0
Egypt	Nasr City 2	Count	2000	0	0	0
Egypt	Nasr City 2	Count	2017	0	0	0
Egypt	New Cairo 1	Proportion	2000	33.2	41.1	25.5
Egypt	New Cairo 1	Proportion	2017	29.4	43.1	19.0
Egypt	New Cairo 1	Count	2000	0	0	0
Egypt	New Cairo 1	Count	2017	0	0	0
Egypt	New Cairo 2	Proportion	2000	33.5	41.9	25.6
Egypt	New Cairo 2	Proportion	2017	29.8	43.4	19.1
Egypt	New Cairo 2	Count	2000	0	0	0
Egypt	New Cairo 2	Count	2017	0	0	0
Egypt	New Cairo 3	Proportion	2000	32.5	40.0	25.1
Egypt	New Cairo 3	Proportion	2017	29.3	43.3	19.1
Egypt	New Cairo 3	Count	2000	0	0	0
Egypt	New Cairo 3	Count	2017	0	0	0
Egypt	Qasr an-Nil	Proportion	2000	31.0	37.8	24.8
Egypt	Qasr an-Nil	Proportion	2017	27.6	39.5	17.9
Egypt	Qasr an-Nil	Count	2000	0	0	0
Egypt	Qasr an-Nil	Count	2017	0	0	0
Egypt	Rud al-Faraj	Proportion	2000	30.9	37.8	24.9
Egypt	Rud al-Faraj	Proportion	2017	27.5	39.5	17.9
Egypt	Rud al-Faraj	Count	2000	0	0	0
Egypt	Rud al-Faraj	Count	2017	0	0	0
Egypt	Shubra	Proportion	2000	30.1	36.6	23.9
Egypt	Shubra	Proportion	2017	24.9	37.3	15.9
Egypt	Shubra	Count	2000	0	0	0
Egypt	Shubra	Count	2017	0	0	0
Egypt	Turah	Proportion	2000	31.7	39.3	24.9
Egypt	Turah	Proportion	2017	28.6	43.2	18.4
Egypt	Turah	Count	2000	0	0	0
Egypt	Turah	Count	2017	0	0	0
Egypt	Unorganized in Al Qahirah	Proportion	2000	32.2	39.3	25.6
Egypt	Unorganized in Al Qahirah	Proportion	2017	28.1	41.7	18.4
Egypt	Unorganized in Al Qahirah	Count	2000	0	0	0
Egypt	Unorganized in Al Qahirah	Count	2017	0	0	0
Egypt	Zamalik	Proportion	2000	31.0	37.8	24.9
Egypt	Zamalik	Proportion	2017	27.6	39.5	18.0
Egypt	Zamalik	Count	2000	0	0	0
Egypt	Zamalik	Count	2017	0	0	0
Egypt	Zawiyya Al-Hamra	Proportion	2000	29.7	36.9	23.2
Egypt	Zawiyya Al-Hamra	Proportion	2017	23.0	35.2	13.8
Egypt	Zawiyya Al-Hamra	Count	2000	0	0	0
Egypt	Zawiyya Al-Hamra	Count	2017	0	0	0
El Salvador	Ahuachapán	Proportion	2000	47.7	65.8	28.9
El Salvador	Ahuachapán	Proportion	2017	60.4	77.7	46.4

Table 4: ORS by Unit *(continued)*

Country	Unit	Metric	Year	Mean	Upper	Lower
El Salvador	Ahuachapán	Count	2000	0	1	0
El Salvador	Ahuachapán	Count	2017	1	1	0
El Salvador	Cinquera	Proportion	2000	50.5	67.5	29.9
El Salvador	Cinquera	Proportion	2017	58.2	75.4	40.7
El Salvador	Cinquera	Count	2000	1	1	0
El Salvador	Cinquera	Count	2017	1	1	0
El Salvador	Agua Caliente	Proportion	2000	46.9	65.0	27.8
El Salvador	Agua Caliente	Proportion	2017	55.6	73.9	37.7
El Salvador	Agua Caliente	Count	2000	0	1	0
El Salvador	Agua Caliente	Count	2017	1	1	0
El Salvador	Candelaria	Proportion	2000	52.8	70.4	33.9
El Salvador	Candelaria	Proportion	2017	60.1	77.9	41.0
El Salvador	Candelaria	Count	2000	1	1	0
El Salvador	Candelaria	Count	2017	1	1	0
El Salvador	Antiguo Cuscatlán	Proportion	2000	51.2	69.5	31.0
El Salvador	Antiguo Cuscatlán	Proportion	2017	59.9	77.9	41.2
El Salvador	Antiguo Cuscatlán	Count	2000	1	1	0
El Salvador	Antiguo Cuscatlán	Count	2017	1	1	0
El Salvador	Cuyultitán	Proportion	2000	52.2	71.2	32.6
El Salvador	Cuyultitán	Proportion	2017	59.7	78.4	40.5
El Salvador	Cuyultitán	Count	2000	1	1	0
El Salvador	Cuyultitán	Count	2017	1	1	0
El Salvador	Anamorós	Proportion	2000	48.1	65.0	30.5
El Salvador	Anamorós	Proportion	2017	52.3	72.3	32.5
El Salvador	Anamorós	Count	2000	0	1	0
El Salvador	Anamorós	Count	2017	1	1	0
El Salvador	Arambala	Proportion	2000	50.2	68.6	30.8
El Salvador	Arambala	Proportion	2017	56.7	74.8	37.1
El Salvador	Arambala	Count	2000	1	1	0
El Salvador	Arambala	Count	2017	1	1	0
El Salvador	Carolina	Proportion	2000	49.5	67.7	32.4
El Salvador	Carolina	Proportion	2017	56.7	75.8	38.1
El Salvador	Carolina	Count	2000	0	1	0
El Salvador	Carolina	Count	2017	1	1	0
El Salvador	Aguilares	Proportion	2000	48.9	67.6	30.8
El Salvador	Aguilares	Proportion	2017	56.7	76.3	38.3
El Salvador	Aguilares	Count	2000	0	1	0
El Salvador	Aguilares	Count	2017	1	1	0
El Salvador	Apastepeque	Proportion	2000	51.5	68.8	33.5
El Salvador	Apastepeque	Proportion	2017	58.0	76.2	38.7
El Salvador	Apastepeque	Count	2000	1	1	0
El Salvador	Apastepeque	Count	2017	1	1	0
El Salvador	Candelaria de la Frontera	Proportion	2000	46.9	65.7	27.3
El Salvador	Candelaria de la Frontera	Proportion	2017	58.9	74.7	42.1
El Salvador	Candelaria de la Frontera	Count	2000	0	1	0
El Salvador	Candelaria de la Frontera	Count	2017	1	1	0
El Salvador	Acajutla	Proportion	2000	47.3	64.7	27.8
El Salvador	Acajutla	Proportion	2017	59.5	76.1	40.1
El Salvador	Acajutla	Count	2000	0	1	0

Table 4: ORS by Unit *(continued)*

Country	Unit	Metric	Year	Mean	Upper	Lower
El Salvador	Acajutla	Count	2017	1	1	0
El Salvador	Alegría	Proportion	2000	50.8	67.4	31.4
El Salvador	Alegría	Proportion	2017	57.5	76.1	39.6
El Salvador	Alegría	Count	2000	1	1	0
El Salvador	Alegría	Count	2017	1	1	0
El Salvador	Apaneca	Proportion	2000	47.2	67.9	27.7
El Salvador	Apaneca	Proportion	2017	60.8	76.7	43.3
El Salvador	Apaneca	Count	2000	0	1	0
El Salvador	Apaneca	Count	2017	1	1	0
El Salvador	Dolores	Proportion	2000	49.5	64.9	33.0
El Salvador	Dolores	Proportion	2017	57.4	75.3	38.9
El Salvador	Dolores	Count	2000	0	1	0
El Salvador	Dolores	Count	2017	1	1	0
El Salvador	Arcatao	Proportion	2000	51.5	68.8	32.0
El Salvador	Arcatao	Proportion	2017	59.6	74.7	44.6
El Salvador	Arcatao	Count	2000	1	1	0
El Salvador	Arcatao	Count	2017	1	1	0
El Salvador	Cojutepeque	Proportion	2000	52.8	71.3	34.1
El Salvador	Cojutepeque	Proportion	2017	59.5	78.4	41.9
El Salvador	Cojutepeque	Count	2000	1	1	0
El Salvador	Cojutepeque	Count	2017	1	1	0
El Salvador	Chiltiupán	Proportion	2000	50.4	67.7	31.5
El Salvador	Chiltiupán	Proportion	2017	60.6	78.4	42.6
El Salvador	Chiltiupán	Count	2000	1	1	0
El Salvador	Chiltiupán	Count	2017	1	1	0
El Salvador	El Rosario	Proportion	2000	51.1	70.9	32.5
El Salvador	El Rosario	Proportion	2017	57.8	77.0	37.6
El Salvador	El Rosario	Count	2000	1	1	0
El Salvador	El Rosario	Count	2017	1	1	0
El Salvador	Bolívar	Proportion	2000	47.1	66.0	29.3
El Salvador	Bolívar	Proportion	2017	52.6	72.7	34.9
El Salvador	Bolívar	Count	2000	0	1	0
El Salvador	Bolívar	Count	2017	1	1	0
El Salvador	Cacaopera	Proportion	2000	48.2	66.0	31.0
El Salvador	Cacaopera	Proportion	2017	54.4	74.3	36.8
El Salvador	Cacaopera	Count	2000	0	1	0
El Salvador	Cacaopera	Count	2017	1	1	0
El Salvador	Chapeltique	Proportion	2000	48.4	65.6	30.9
El Salvador	Chapeltique	Proportion	2017	54.9	73.5	37.9
El Salvador	Chapeltique	Count	2000	0	1	0
El Salvador	Chapeltique	Count	2017	1	1	0
El Salvador	Apopa	Proportion	2000	49.7	68.6	31.4
El Salvador	Apopa	Proportion	2017	58.0	76.8	40.5
El Salvador	Apopa	Count	2000	0	1	0
El Salvador	Apopa	Count	2017	1	1	0
El Salvador	Guadalupe	Proportion	2000	52.4	70.3	33.8
El Salvador	Guadalupe	Proportion	2017	59.6	78.9	40.8
El Salvador	Guadalupe	Count	2000	1	1	0
El Salvador	Guadalupe	Count	2017	1	1	0

Table 4: ORS by Unit *(continued)*

Country	Unit	Metric	Year	Mean	Upper	Lower
El Salvador	Chalchuapa	Proportion	2000	48.8	65.7	29.5
El Salvador	Chalchuapa	Proportion	2017	60.7	77.4	43.0
El Salvador	Chalchuapa	Count	2000	0	1	0
El Salvador	Chalchuapa	Count	2017	1	1	0
El Salvador	Armenia	Proportion	2000	50.1	67.5	29.7
El Salvador	Armenia	Proportion	2017	60.6	76.7	44.5
El Salvador	Armenia	Count	2000	1	1	0
El Salvador	Armenia	Count	2017	1	1	0
El Salvador	Berlín	Proportion	2000	50.2	66.3	32.3
El Salvador	Berlín	Proportion	2017	57.0	74.1	41.0
El Salvador	Berlín	Count	2000	1	1	0
El Salvador	Berlín	Count	2017	1	1	0
El Salvador	Atiquizaya	Proportion	2000	48.8	66.6	28.6
El Salvador	Atiquizaya	Proportion	2017	60.5	74.4	45.6
El Salvador	Atiquizaya	Count	2000	0	1	0
El Salvador	Atiquizaya	Count	2017	1	1	0
El Salvador	Guacotecti	Proportion	2000	51.8	68.8	31.5
El Salvador	Guacotecti	Proportion	2017	59.0	76.9	40.6
El Salvador	Guacotecti	Count	2000	1	1	0
El Salvador	Guacotecti	Count	2017	1	1	0
El Salvador	Azacualpa	Proportion	2000	50.0	68.0	29.3
El Salvador	Azacualpa	Proportion	2017	57.1	74.6	37.1
El Salvador	Azacualpa	Count	2000	0	1	0
El Salvador	Azacualpa	Count	2017	1	1	0
El Salvador	El Carmen	Proportion	2000	53.1	72.6	33.0
El Salvador	El Carmen	Proportion	2017	59.5	77.4	41.4
El Salvador	El Carmen	Count	2000	1	1	0
El Salvador	El Carmen	Count	2017	1	1	0
El Salvador	Ciudad Arce	Proportion	2000	50.1	68.7	30.9
El Salvador	Ciudad Arce	Proportion	2017	59.6	77.9	42.2
El Salvador	Ciudad Arce	Count	2000	1	1	0
El Salvador	Ciudad Arce	Count	2017	1	1	0
El Salvador	Jerusalén	Proportion	2000	53.0	71.7	34.6
El Salvador	Jerusalén	Proportion	2017	60.1	77.9	41.1
El Salvador	Jerusalén	Count	2000	1	1	0
El Salvador	Jerusalén	Count	2017	1	1	0
El Salvador	Concepción de Oriente	Proportion	2000	49.2	66.8	33.2
El Salvador	Concepción de Oriente	Proportion	2017	53.2	73.0	34.9
El Salvador	Concepción de Oriente	Count	2000	0	1	0
El Salvador	Concepción de Oriente	Count	2017	1	1	0
El Salvador	Chilanga	Proportion	2000	48.0	64.7	30.1
El Salvador	Chilanga	Proportion	2017	53.9	74.3	34.9
El Salvador	Chilanga	Count	2000	0	1	0
El Salvador	Chilanga	Count	2017	1	1	0
El Salvador	Chinameca	Proportion	2000	49.9	66.9	32.0
El Salvador	Chinameca	Proportion	2017	55.6	73.0	37.5
El Salvador	Chinameca	Count	2000	0	1	0
El Salvador	Chinameca	Count	2017	1	1	0
El Salvador	Ayutuxtepeque	Proportion	2000	50.5	70.1	31.3

Table 4: ORS by Unit *(continued)*

Country	Unit	Metric	Year	Mean	Upper	Lower
El Salvador	Ayutuxtepeque	Proportion	2017	59.7	78.7	39.3
El Salvador	Ayutuxtepeque	Count	2000	1	1	0
El Salvador	Ayutuxtepeque	Count	2017	1	1	0
El Salvador	San Cayetano Istepeque	Proportion	2000	53.8	73.8	35.1
El Salvador	San Cayetano Istepeque	Proportion	2017	59.7	78.0	39.2
El Salvador	San Cayetano Istepeque	Count	2000	1	1	0
El Salvador	San Cayetano Istepeque	Count	2017	1	1	0
El Salvador	Coatepeque	Proportion	2000	49.4	67.8	30.9
El Salvador	Coatepeque	Proportion	2017	60.0	77.0	43.3
El Salvador	Coatepeque	Count	2000	0	1	0
El Salvador	Coatepeque	Count	2017	1	1	0
El Salvador	Caluco	Proportion	2000	49.3	68.9	29.2
El Salvador	Caluco	Proportion	2017	61.4	77.5	45.6
El Salvador	Caluco	Count	2000	0	1	0
El Salvador	Caluco	Count	2017	1	1	0
El Salvador	California	Proportion	2000	51.5	68.8	32.4
El Salvador	California	Proportion	2017	57.7	76.5	40.3
El Salvador	California	Count	2000	1	1	0
El Salvador	California	Count	2017	1	1	0
El Salvador	Concepción de Ataco	Proportion	2000	46.8	65.4	28.7
El Salvador	Concepción de Ataco	Proportion	2017	60.2	77.0	43.0
El Salvador	Concepción de Ataco	Count	2000	0	1	0
El Salvador	Concepción de Ataco	Count	2017	1	1	0
El Salvador	Ilobasco	Proportion	2000	51.7	68.7	34.4
El Salvador	Ilobasco	Proportion	2017	59.0	76.3	40.5
El Salvador	Ilobasco	Count	2000	1	1	0
El Salvador	Ilobasco	Count	2017	1	1	0
El Salvador	Chalatenango	Proportion	2000	49.7	67.7	32.0
El Salvador	Chalatenango	Proportion	2017	56.0	73.7	37.3
El Salvador	Chalatenango	Count	2000	0	1	0
El Salvador	Chalatenango	Count	2017	1	1	0
El Salvador	El Rosario	Proportion	2000	53.2	72.3	32.0
El Salvador	El Rosario	Proportion	2017	59.1	78.4	41.5
El Salvador	El Rosario	Count	2000	1	1	0
El Salvador	El Rosario	Count	2017	1	1	0
El Salvador	Colón	Proportion	2000	52.0	71.1	32.8
El Salvador	Colón	Proportion	2017	61.5	79.1	45.3
El Salvador	Colón	Count	2000	1	1	0
El Salvador	Colón	Count	2017	1	1	0
El Salvador	Mercedes La Ceiba	Proportion	2000	52.9	73.8	33.7
El Salvador	Mercedes La Ceiba	Proportion	2017	60.3	78.8	39.4
El Salvador	Mercedes La Ceiba	Count	2000	1	1	0
El Salvador	Mercedes La Ceiba	Count	2017	1	1	0
El Salvador	Conchagua	Proportion	2000	48.2	66.6	30.5
El Salvador	Conchagua	Proportion	2017	53.3	72.3	35.2
El Salvador	Conchagua	Count	2000	0	1	0
El Salvador	Conchagua	Count	2017	1	1	0
El Salvador	Corinto	Proportion	2000	49.1	67.1	29.9
El Salvador	Corinto	Proportion	2017	54.4	73.9	36.8

Table 4: ORS by Unit *(continued)*

Country	Unit	Metric	Year	Mean	Upper	Lower
El Salvador	Corinto	Count	2000	0	1	0
El Salvador	Corinto	Count	2017	1	1	0
El Salvador	Chirilagua	Proportion	2000	47.1	66.1	29.4
El Salvador	Chirilagua	Proportion	2017	54.0	72.4	35.0
El Salvador	Chirilagua	Count	2000	0	1	0
El Salvador	Chirilagua	Count	2017	1	1	0
El Salvador	Cuscatancingo	Proportion	2000	50.1	70.4	31.4
El Salvador	Cuscatancingo	Proportion	2017	58.9	77.9	41.6
El Salvador	Cuscatancingo	Count	2000	1	1	0
El Salvador	Cuscatancingo	Count	2017	1	1	0
El Salvador	San Esteban Catarina	Proportion	2000	52.2	68.6	33.6
El Salvador	San Esteban Catarina	Proportion	2017	59.0	76.6	40.8
El Salvador	San Esteban Catarina	Count	2000	1	1	0
El Salvador	San Esteban Catarina	Count	2017	1	1	0
El Salvador	El Congo	Proportion	2000	49.2	67.1	30.3
El Salvador	El Congo	Proportion	2017	60.1	77.1	43.7
El Salvador	El Congo	Count	2000	0	1	0
El Salvador	El Congo	Count	2017	1	1	0
El Salvador	Cuisnahuat	Proportion	2000	49.3	68.6	30.7
El Salvador	Cuisnahuat	Proportion	2017	61.2	78.3	41.7
El Salvador	Cuisnahuat	Count	2000	0	1	0
El Salvador	Cuisnahuat	Count	2017	1	1	0
El Salvador	Concepción Batres	Proportion	2000	47.2	66.4	29.0
El Salvador	Concepción Batres	Proportion	2017	53.4	71.0	34.9
El Salvador	Concepción Batres	Count	2000	0	1	0
El Salvador	Concepción Batres	Count	2017	1	1	0
El Salvador	El Refugio	Proportion	2000	49.6	68.6	29.1
El Salvador	El Refugio	Proportion	2017	61.0	78.5	39.4
El Salvador	El Refugio	Count	2000	0	1	0
El Salvador	El Refugio	Count	2017	1	1	0
El Salvador	Jutiapa	Proportion	2000	50.4	67.0	31.2
El Salvador	Jutiapa	Proportion	2017	58.0	74.3	40.8
El Salvador	Jutiapa	Count	2000	1	1	0
El Salvador	Jutiapa	Count	2017	1	1	0
El Salvador	Citalá	Proportion	2000	46.6	64.8	28.4
El Salvador	Citalá	Proportion	2017	54.9	71.0	37.8
El Salvador	Citalá	Count	2000	0	1	0
El Salvador	Citalá	Count	2017	1	1	0
El Salvador	Monte San Juan	Proportion	2000	52.6	71.1	32.2
El Salvador	Monte San Juan	Proportion	2017	59.3	78.8	41.8
El Salvador	Monte San Juan	Count	2000	1	1	0
El Salvador	Monte San Juan	Count	2017	1	1	0
El Salvador	Comasagua	Proportion	2000	51.8	70.2	32.1
El Salvador	Comasagua	Proportion	2017	61.4	79.8	43.7
El Salvador	Comasagua	Count	2000	1	1	0
El Salvador	Comasagua	Count	2017	1	1	0
El Salvador	Olocuilta	Proportion	2000	51.9	69.0	35.6
El Salvador	Olocuilta	Proportion	2017	59.3	77.4	41.1
El Salvador	Olocuilta	Count	2000	1	1	0

Table 4: ORS by Unit *(continued)*

Country	Unit	Metric	Year	Mean	Upper	Lower
El Salvador	Olocuilta	Count	2017	1	1	0
El Salvador	El Carmen	Proportion	2000	47.0	65.6	31.2
El Salvador	El Carmen	Proportion	2017	53.0	72.8	34.9
El Salvador	El Carmen	Count	2000	0	1	0
El Salvador	El Carmen	Count	2017	1	1	0
El Salvador	Delicias de Concepción	Proportion	2000	48.0	66.3	29.1
El Salvador	Delicias de Concepción	Proportion	2017	54.8	75.4	35.0
El Salvador	Delicias de Concepción	Count	2000	0	1	0
El Salvador	Delicias de Concepción	Count	2017	1	1	0
El Salvador	Ciudad Barrios	Proportion	2000	50.2	67.8	32.9
El Salvador	Ciudad Barrios	Proportion	2017	56.8	76.0	39.1
El Salvador	Ciudad Barrios	Count	2000	1	1	0
El Salvador	Ciudad Barrios	Count	2017	1	1	0
El Salvador	Delgado	Proportion	2000	50.1	68.8	30.6
El Salvador	Delgado	Proportion	2017	58.7	76.9	41.1
El Salvador	Delgado	Count	2000	1	1	0
El Salvador	Delgado	Count	2017	1	1	0
El Salvador	San Ildefonso	Proportion	2000	49.7	65.8	33.1
El Salvador	San Ildefonso	Proportion	2017	56.6	75.3	37.9
El Salvador	San Ildefonso	Count	2000	0	1	0
El Salvador	San Ildefonso	Count	2017	1	1	0
El Salvador	El Porvenir	Proportion	2000	48.7	67.0	28.6
El Salvador	El Porvenir	Proportion	2017	60.4	76.4	44.9
El Salvador	El Porvenir	Count	2000	0	1	0
El Salvador	El Porvenir	Count	2017	1	1	0
El Salvador	Izalco	Proportion	2000	49.7	67.5	30.9
El Salvador	Izalco	Proportion	2017	61.4	76.5	46.1
El Salvador	Izalco	Count	2000	0	1	0
El Salvador	Izalco	Count	2017	1	1	0
El Salvador	El Triunfo	Proportion	2000	49.8	68.0	31.5
El Salvador	El Triunfo	Proportion	2017	56.1	74.3	38.5
El Salvador	El Triunfo	Count	2000	0	1	0
El Salvador	El Triunfo	Count	2017	1	1	0
El Salvador	Guaymango	Proportion	2000	46.2	63.1	28.0
El Salvador	Guaymango	Proportion	2017	59.7	76.7	43.6
El Salvador	Guaymango	Count	2000	0	1	0
El Salvador	Guaymango	Count	2017	1	1	0
El Salvador	San Isidro	Proportion	2000	50.9	67.6	32.3
El Salvador	San Isidro	Proportion	2017	59.0	77.6	42.6
El Salvador	San Isidro	Count	2000	1	1	0
El Salvador	San Isidro	Count	2017	1	1	0
El Salvador	Comalapa	Proportion	2000	47.9	65.2	27.1
El Salvador	Comalapa	Proportion	2017	55.5	74.7	36.5
El Salvador	Comalapa	Count	2000	0	1	0
El Salvador	Comalapa	Count	2017	1	1	0
El Salvador	Oratorio de Concepción	Proportion	2000	51.2	68.9	28.9
El Salvador	Oratorio de Concepción	Proportion	2017	58.5	78.1	42.2
El Salvador	Oratorio de Concepción	Count	2000	1	1	0
El Salvador	Oratorio de Concepción	Count	2017	1	1	0

Table 4: ORS by Unit *(continued)*

Country	Unit	Metric	Year	Mean	Upper	Lower
El Salvador	Huizúcar	Proportion	2000	52.2	69.7	34.0
El Salvador	Huizúcar	Proportion	2017	60.5	78.7	41.6
El Salvador	Huizúcar	Count	2000	1	1	0
El Salvador	Huizúcar	Count	2017	1	1	0
El Salvador	Paraíso de Osorio	Proportion	2000	53.3	70.5	34.4
El Salvador	Paraíso de Osorio	Proportion	2017	60.0	77.2	43.2
El Salvador	Paraíso de Osorio	Count	2000	1	1	0
El Salvador	Paraíso de Osorio	Count	2017	1	1	0
El Salvador	El Sauce	Proportion	2000	47.4	64.0	30.4
El Salvador	El Sauce	Proportion	2017	51.6	70.9	34.5
El Salvador	El Sauce	Count	2000	0	1	0
El Salvador	El Sauce	Count	2017	1	1	0
El Salvador	El Divisadero	Proportion	2000	46.5	64.5	29.4
El Salvador	El Divisadero	Proportion	2017	52.4	69.9	34.1
El Salvador	El Divisadero	Count	2000	0	1	0
El Salvador	El Divisadero	Count	2017	1	1	0
El Salvador	Comacarán	Proportion	2000	47.1	65.0	29.2
El Salvador	Comacarán	Proportion	2017	53.2	73.1	35.0
El Salvador	Comacarán	Count	2000	0	1	0
El Salvador	Comacarán	Count	2017	1	1	0
El Salvador	El Paisnal	Proportion	2000	47.9	65.4	30.5
El Salvador	El Paisnal	Proportion	2017	56.0	74.0	37.5
El Salvador	El Paisnal	Count	2000	0	1	0
El Salvador	El Paisnal	Count	2017	1	1	0
El Salvador	San Lorenzo	Proportion	2000	52.9	71.7	30.4
El Salvador	San Lorenzo	Proportion	2017	59.5	77.7	40.6
El Salvador	San Lorenzo	Count	2000	1	1	0
El Salvador	San Lorenzo	Count	2017	1	1	0
El Salvador	Lago de Coatepeque	Proportion	2000	49.9	66.8	31.8
El Salvador	Lago de Coatepeque	Proportion	2017	61.0	76.7	45.3
El Salvador	Lago de Coatepeque	Count	2000	0	1	0
El Salvador	Lago de Coatepeque	Count	2017	1	1	0
El Salvador	Juayúa	Proportion	2000	48.0	65.4	28.4
El Salvador	Juayúa	Proportion	2017	60.8	77.7	41.5
El Salvador	Juayúa	Count	2000	0	1	0
El Salvador	Juayúa	Count	2017	1	1	0
El Salvador	Ereguayquín	Proportion	2000	47.6	67.3	28.8
El Salvador	Ereguayquín	Proportion	2017	53.0	72.4	33.2
El Salvador	Ereguayquín	Count	2000	0	1	0
El Salvador	Ereguayquín	Count	2017	1	1	0
El Salvador	Jujutla	Proportion	2000	45.4	62.5	27.4
El Salvador	Jujutla	Proportion	2017	59.3	75.2	43.4
El Salvador	Jujutla	Count	2000	0	1	0
El Salvador	Jujutla	Count	2017	1	1	0
El Salvador	Sensuntepeque	Proportion	2000	50.5	66.4	33.7
El Salvador	Sensuntepeque	Proportion	2017	58.2	75.1	41.2
El Salvador	Sensuntepeque	Count	2000	1	1	0
El Salvador	Sensuntepeque	Count	2017	1	1	0
El Salvador	Concepción Quezaltepeque	Proportion	2000	49.4	67.6	30.1

Table 4: ORS by Unit *(continued)*

Country	Unit	Metric	Year	Mean	Upper	Lower
El Salvador	Concepción Quezaltepeque	Proportion	2017	56.1	73.7	37.4
El Salvador	Concepción Quezaltepeque	Count	2000	0	1	0
El Salvador	Concepción Quezaltepeque	Count	2017	1	1	0
El Salvador	San Bartolomé Perulapía	Proportion	2000	52.0	70.9	33.5
El Salvador	San Bartolomé Perulapía	Proportion	2017	58.9	80.2	40.3
El Salvador	San Bartolomé Perulapía	Count	2000	1	1	0
El Salvador	San Bartolomé Perulapía	Count	2017	1	1	0
El Salvador	Jayaque	Proportion	2000	51.2	69.0	32.9
El Salvador	Jayaque	Proportion	2017	61.3	78.4	44.5
El Salvador	Jayaque	Count	2000	1	1	0
El Salvador	Jayaque	Count	2017	1	1	0
El Salvador	San Antonio Masahuat	Proportion	2000	51.8	70.9	33.0
El Salvador	San Antonio Masahuat	Proportion	2017	58.8	78.5	39.2
El Salvador	San Antonio Masahuat	Count	2000	1	1	0
El Salvador	San Antonio Masahuat	Count	2017	1	1	0
El Salvador	Intipucá	Proportion	2000	47.3	65.4	30.4
El Salvador	Intipucá	Proportion	2017	54.0	72.9	36.1
El Salvador	Intipucá	Count	2000	0	1	0
El Salvador	Intipucá	Count	2017	1	1	0
El Salvador	El Rosario	Proportion	2000	49.2	65.8	31.4
El Salvador	El Rosario	Proportion	2017	56.4	75.8	35.7
El Salvador	El Rosario	Count	2000	0	1	0
El Salvador	El Rosario	Count	2017	1	1	0
El Salvador	El Tránsito	Proportion	2000	47.3	65.9	29.1
El Salvador	El Tránsito	Proportion	2017	53.1	72.3	33.2
El Salvador	El Tránsito	Count	2000	0	1	0
El Salvador	El Tránsito	Count	2017	1	1	0
El Salvador	Guazapa	Proportion	2000	48.9	67.0	29.6
El Salvador	Guazapa	Proportion	2017	56.8	74.3	39.3
El Salvador	Guazapa	Count	2000	0	1	0
El Salvador	Guazapa	Count	2017	1	1	0
El Salvador	San Sebastián	Proportion	2000	52.9	69.0	33.0
El Salvador	San Sebastián	Proportion	2017	59.3	76.5	42.9
El Salvador	San Sebastián	Count	2000	1	1	0
El Salvador	San Sebastián	Count	2017	1	1	0
El Salvador	Lago de Guija	Proportion	2000	45.8	64.7	25.9
El Salvador	Lago de Guija	Proportion	2017	56.3	75.9	38.6
El Salvador	Lago de Guija	Count	2000	0	1	0
El Salvador	Lago de Guija	Count	2017	1	1	0
El Salvador	Nahuizalco	Proportion	2000	50.0	68.7	28.3
El Salvador	Nahuizalco	Proportion	2017	61.9	79.1	44.9
El Salvador	Nahuizalco	Count	2000	0	1	0
El Salvador	Nahuizalco	Count	2017	1	1	0
El Salvador	Estanzuelas	Proportion	2000	49.2	64.9	32.2
El Salvador	Estanzuelas	Proportion	2017	55.7	75.5	35.9
El Salvador	Estanzuelas	Count	2000	0	1	0
El Salvador	Estanzuelas	Count	2017	1	1	0
El Salvador	San Francisco Menéndez	Proportion	2000	44.4	62.9	25.9
El Salvador	San Francisco Menéndez	Proportion	2017	58.8	75.4	42.6

Table 4: ORS by Unit *(continued)*

Country	Unit	Metric	Year	Mean	Upper	Lower
El Salvador	San Francisco Menéndez	Count	2000	0	1	0
El Salvador	San Francisco Menéndez	Count	2017	1	1	0
El Salvador	Tejutepeque	Proportion	2000	51.3	69.4	33.0
El Salvador	Tejutepeque	Proportion	2017	59.0	76.1	41.9
El Salvador	Tejutepeque	Count	2000	1	1	0
El Salvador	Tejutepeque	Count	2017	1	1	0
El Salvador	Dulce Nombre de María	Proportion	2000	47.7	66.7	28.1
El Salvador	Dulce Nombre de María	Proportion	2017	55.5	72.6	37.9
El Salvador	Dulce Nombre de María	Count	2000	0	1	0
El Salvador	Dulce Nombre de María	Count	2017	1	1	0
El Salvador	San Cristóbal	Proportion	2000	54.2	73.3	35.7
El Salvador	San Cristóbal	Proportion	2017	59.4	77.2	39.2
El Salvador	San Cristóbal	Count	2000	1	1	0
El Salvador	San Cristóbal	Count	2017	1	1	0
El Salvador	Jicalapa	Proportion	2000	49.8	68.5	29.5
El Salvador	Jicalapa	Proportion	2017	60.8	79.9	41.9
El Salvador	Jicalapa	Count	2000	0	1	0
El Salvador	Jicalapa	Count	2017	1	1	0
El Salvador	San Emigdio	Proportion	2000	52.8	73.3	29.6
El Salvador	San Emigdio	Proportion	2017	59.7	77.7	39.1
El Salvador	San Emigdio	Count	2000	1	1	0
El Salvador	San Emigdio	Count	2017	1	1	0
El Salvador	La Unión	Proportion	2000	48.3	67.1	30.3
El Salvador	La Unión	Proportion	2017	53.4	72.2	36.1
El Salvador	La Unión	Count	2000	0	1	0
El Salvador	La Unión	Count	2017	1	1	0
El Salvador	Gualococti	Proportion	2000	49.0	68.6	32.1
El Salvador	Gualococti	Proportion	2017	56.2	77.0	36.8
El Salvador	Gualococti	Count	2000	0	1	0
El Salvador	Gualococti	Count	2017	1	1	0
El Salvador	Lolotique	Proportion	2000	49.4	66.8	32.3
El Salvador	Lolotique	Proportion	2017	55.6	74.5	37.1
El Salvador	Lolotique	Count	2000	0	1	0
El Salvador	Lolotique	Count	2017	1	1	0
El Salvador	Ilopango	Proportion	2000	49.8	69.4	30.1
El Salvador	Ilopango	Proportion	2017	58.1	76.4	40.6
El Salvador	Ilopango	Count	2000	0	1	0
El Salvador	Ilopango	Count	2017	1	1	0
El Salvador	San Vicente	Proportion	2000	51.7	68.0	33.9
El Salvador	San Vicente	Proportion	2017	58.0	75.1	40.6
El Salvador	San Vicente	Count	2000	1	1	0
El Salvador	San Vicente	Count	2017	1	1	0
El Salvador	Masahuat	Proportion	2000	46.0	66.8	25.6
El Salvador	Masahuat	Proportion	2017	56.5	74.4	38.3
El Salvador	Masahuat	Count	2000	0	1	0
El Salvador	Masahuat	Count	2017	1	1	0
El Salvador	Nahulingo	Proportion	2000	48.8	68.0	29.0
El Salvador	Nahulingo	Proportion	2017	60.4	77.5	41.9
El Salvador	Nahulingo	Count	2000	0	1	0

Table 4: ORS by Unit *(continued)*

Country	Unit	Metric	Year	Mean	Upper	Lower
El Salvador	Nahulingo	Count	2017	1	1	0
El Salvador	Jiquilisco	Proportion	2000	50.5	67.7	34.3
El Salvador	Jiquilisco	Proportion	2017	56.8	74.9	39.8
El Salvador	Jiquilisco	Count	2000	1	1	0
El Salvador	Jiquilisco	Count	2017	1	1	0
El Salvador	San Lorenzo	Proportion	2000	47.7	66.6	29.8
El Salvador	San Lorenzo	Proportion	2017	60.3	75.0	44.0
El Salvador	San Lorenzo	Count	2000	0	1	0
El Salvador	San Lorenzo	Count	2017	1	1	0
El Salvador	Victoria	Proportion	2000	50.8	66.8	34.9
El Salvador	Victoria	Proportion	2017	58.7	76.1	42.5
El Salvador	Victoria	Count	2000	1	1	0
El Salvador	Victoria	Count	2017	1	1	0
El Salvador	El Carrizal	Proportion	2000	47.7	66.6	27.3
El Salvador	El Carrizal	Proportion	2017	55.1	72.4	37.7
El Salvador	El Carrizal	Count	2000	0	1	0
El Salvador	El Carrizal	Count	2017	1	1	0
El Salvador	San José Guayabal	Proportion	2000	50.4	67.8	32.2
El Salvador	San José Guayabal	Proportion	2017	58.2	76.2	43.6
El Salvador	San José Guayabal	Count	2000	1	1	0
El Salvador	San José Guayabal	Count	2017	1	1	0
El Salvador	La Libertad	Proportion	2000	51.2	69.0	30.5
El Salvador	La Libertad	Proportion	2017	59.0	77.4	39.8
El Salvador	La Libertad	Count	2000	1	1	0
El Salvador	La Libertad	Count	2017	1	1	0
El Salvador	San Francisco Chinameca	Proportion	2000	52.3	67.6	32.8
El Salvador	San Francisco Chinameca	Proportion	2017	59.8	78.6	39.9
El Salvador	San Francisco Chinameca	Count	2000	1	1	0
El Salvador	San Francisco Chinameca	Count	2017	1	1	0
El Salvador	Lislique	Proportion	2000	48.9	67.9	30.6
El Salvador	Lislique	Proportion	2017	54.2	74.0	35.9
El Salvador	Lislique	Count	2000	0	1	0
El Salvador	Lislique	Count	2017	1	1	0
El Salvador	Guatajiagua	Proportion	2000	48.6	65.8	31.9
El Salvador	Guatajiagua	Proportion	2017	55.6	76.0	36.6
El Salvador	Guatajiagua	Count	2000	0	1	0
El Salvador	Guatajiagua	Count	2017	1	1	0
El Salvador	Moncagua	Proportion	2000	48.8	67.3	31.9
El Salvador	Moncagua	Proportion	2017	54.7	72.9	37.5
El Salvador	Moncagua	Count	2000	0	1	0
El Salvador	Moncagua	Count	2017	1	1	0
El Salvador	Lago de Llopango	Proportion	2000	51.4	69.6	32.6
El Salvador	Lago de Llopango	Proportion	2017	59.0	76.3	41.4
El Salvador	Lago de Llopango	Count	2000	1	1	0
El Salvador	Lago de Llopango	Count	2017	1	1	0
El Salvador	Santa Clara	Proportion	2000	50.1	65.7	34.8
El Salvador	Santa Clara	Proportion	2017	57.7	75.6	40.1
El Salvador	Santa Clara	Count	2000	1	1	0
El Salvador	Santa Clara	Count	2017	1	1	0

Table 4: ORS by Unit *(continued)*

Country	Unit	Metric	Year	Mean	Upper	Lower
El Salvador	Metapán	Proportion	2000	45.8	63.7	26.9
El Salvador	Metapán	Proportion	2017	55.3	72.1	37.9
El Salvador	Metapán	Count	2000	0	1	0
El Salvador	Metapán	Count	2017	1	1	0
El Salvador	Salcoatitán	Proportion	2000	48.9	67.4	29.4
El Salvador	Salcoatitán	Proportion	2017	61.4	77.8	45.6
El Salvador	Salcoatitán	Count	2000	0	1	0
El Salvador	Salcoatitán	Count	2017	1	1	0
El Salvador	Jucuapa	Proportion	2000	50.4	67.4	32.2
El Salvador	Jucuapa	Proportion	2017	56.3	75.2	38.1
El Salvador	Jucuapa	Count	2000	1	1	0
El Salvador	Jucuapa	Count	2017	1	1	0
El Salvador	San Pedro Puxtla	Proportion	2000	47.4	65.8	28.6
El Salvador	San Pedro Puxtla	Proportion	2017	60.5	76.8	44.0
El Salvador	San Pedro Puxtla	Count	2000	0	1	0
El Salvador	San Pedro Puxtla	Count	2017	1	1	0
El Salvador	El Paraíso	Proportion	2000	48.1	66.4	29.3
El Salvador	El Paraíso	Proportion	2017	55.3	73.0	38.5
El Salvador	El Paraíso	Count	2000	0	1	0
El Salvador	El Paraíso	Count	2017	1	1	0
El Salvador	San Pedro Perulapán	Proportion	2000	52.5	68.9	35.9
El Salvador	San Pedro Perulapán	Proportion	2017	59.0	78.9	40.3
El Salvador	San Pedro Perulapán	Count	2000	1	1	0
El Salvador	San Pedro Perulapán	Count	2017	1	1	0
El Salvador	Nueva San Salvador	Proportion	2000	52.3	70.7	33.6
El Salvador	Nueva San Salvador	Proportion	2017	60.8	78.9	43.0
El Salvador	Nueva San Salvador	Count	2000	1	1	0
El Salvador	Nueva San Salvador	Count	2017	1	1	0
El Salvador	San Juan Nonualco	Proportion	2000	51.7	69.7	33.4
El Salvador	San Juan Nonualco	Proportion	2017	58.0	75.9	38.9
El Salvador	San Juan Nonualco	Count	2000	1	1	0
El Salvador	San Juan Nonualco	Count	2017	1	1	0
El Salvador	Meanguera del Golfo	Proportion	2000	47.0	67.6	28.8
El Salvador	Meanguera del Golfo	Proportion	2017	53.1	74.0	31.6
El Salvador	Meanguera del Golfo	Count	2000	0	1	0
El Salvador	Meanguera del Golfo	Count	2017	1	1	0
El Salvador	Joateca	Proportion	2000	49.4	65.1	34.3
El Salvador	Joateca	Proportion	2017	56.0	74.5	36.8
El Salvador	Joateca	Count	2000	0	1	0
El Salvador	Joateca	Count	2017	1	1	0
El Salvador	Nueva Guadalupe	Proportion	2000	50.3	69.3	30.5
El Salvador	Nueva Guadalupe	Proportion	2017	55.5	77.0	34.6
El Salvador	Nueva Guadalupe	Count	2000	1	1	0
El Salvador	Nueva Guadalupe	Count	2017	1	1	0
El Salvador	Mejicanos	Proportion	2000	50.7	70.7	31.0
El Salvador	Mejicanos	Proportion	2017	59.4	78.7	40.8
El Salvador	Mejicanos	Count	2000	1	1	0
El Salvador	Mejicanos	Count	2017	1	1	0
El Salvador	Santo Domingo	Proportion	2000	53.5	70.8	35.2

Table 4: ORS by Unit *(continued)*

Country	Unit	Metric	Year	Mean	Upper	Lower
El Salvador	Santo Domingo	Proportion	2017	59.5	77.1	41.9
El Salvador	Santo Domingo	Count	2000	1	1	0
El Salvador	Santo Domingo	Count	2017	1	1	0
El Salvador	San Antonio Pajonal	Proportion	2000	46.2	66.8	26.4
El Salvador	San Antonio Pajonal	Proportion	2017	57.9	77.4	41.4
El Salvador	San Antonio Pajonal	Count	2000	0	1	0
El Salvador	San Antonio Pajonal	Count	2017	1	1	0
El Salvador	San Antonio del Monte	Proportion	2000	49.7	69.5	30.0
El Salvador	San Antonio del Monte	Proportion	2017	61.1	78.4	40.8
El Salvador	San Antonio del Monte	Count	2000	0	1	0
El Salvador	San Antonio del Monte	Count	2017	1	1	0
El Salvador	Jucuarán	Proportion	2000	47.6	65.4	30.3
El Salvador	Jucuarán	Proportion	2017	54.6	72.9	35.5
El Salvador	Jucuarán	Count	2000	0	1	0
El Salvador	Jucuarán	Count	2017	1	1	0
El Salvador	Tacuba	Proportion	2000	46.3	65.2	28.4
El Salvador	Tacuba	Proportion	2017	60.6	77.4	45.9
El Salvador	Tacuba	Count	2000	0	1	0
El Salvador	Tacuba	Count	2017	1	1	0
El Salvador	Embalse Cerron Grande	Proportion	2000	49.5	66.2	31.9
El Salvador	Embalse Cerron Grande	Proportion	2017	56.7	72.8	40.5
El Salvador	Embalse Cerron Grande	Count	2000	0	1	0
El Salvador	Embalse Cerron Grande	Count	2017	1	1	0
El Salvador	San Rafael Cedros	Proportion	2000	53.2	71.7	33.1
El Salvador	San Rafael Cedros	Proportion	2017	59.5	76.9	42.1
El Salvador	San Rafael Cedros	Count	2000	1	1	0
El Salvador	San Rafael Cedros	Count	2017	1	1	0
El Salvador	Nuevo Cuscatlán	Proportion	2000	52.7	70.8	32.7
El Salvador	Nuevo Cuscatlán	Proportion	2017	61.1	80.2	40.3
El Salvador	Nuevo Cuscatlán	Count	2000	1	1	0
El Salvador	Nuevo Cuscatlán	Count	2017	1	1	0
El Salvador	San Juan Talpa	Proportion	2000	51.0	68.5	32.3
El Salvador	San Juan Talpa	Proportion	2017	58.0	76.4	39.0
El Salvador	San Juan Talpa	Count	2000	1	1	0
El Salvador	San Juan Talpa	Count	2017	1	1	0
El Salvador	Nueva Esparta	Proportion	2000	48.5	66.0	30.9
El Salvador	Nueva Esparta	Proportion	2017	53.6	73.4	36.7
El Salvador	Nueva Esparta	Count	2000	0	1	0
El Salvador	Nueva Esparta	Count	2017	1	1	0
El Salvador	Jocoaitique	Proportion	2000	49.6	67.2	31.9
El Salvador	Jocoaitique	Proportion	2017	55.9	74.9	37.4
El Salvador	Jocoaitique	Count	2000	0	1	0
El Salvador	Jocoaitique	Count	2017	1	1	0
El Salvador	Nuevo Edén de San Juan	Proportion	2000	48.7	65.9	32.8
El Salvador	Nuevo Edén de San Juan	Proportion	2017	56.3	75.6	39.1
El Salvador	Nuevo Edén de San Juan	Count	2000	0	1	0
El Salvador	Nuevo Edén de San Juan	Count	2017	1	1	0
El Salvador	Nejapa	Proportion	2000	50.1	66.9	31.1
El Salvador	Nejapa	Proportion	2017	58.0	74.8	39.3

Table 4: ORS by Unit *(continued)*

Country	Unit	Metric	Year	Mean	Upper	Lower
El Salvador	Nejapa	Count	2000	1	1	0
El Salvador	Nejapa	Count	2017	1	1	0
El Salvador	Tecoluca	Proportion	2000	50.9	67.1	34.8
El Salvador	Tecoluca	Proportion	2017	57.6	75.8	40.2
El Salvador	Tecoluca	Count	2000	1	1	0
El Salvador	Tecoluca	Count	2017	1	1	0
El Salvador	San Sebastián Salitrillo	Proportion	2000	49.7	67.3	29.1
El Salvador	San Sebastián Salitrillo	Proportion	2017	60.8	76.8	43.2
El Salvador	San Sebastián Salitrillo	Count	2000	0	1	0
El Salvador	San Sebastián Salitrillo	Count	2017	1	1	0
El Salvador	San Julián	Proportion	2000	50.0	68.4	31.1
El Salvador	San Julián	Proportion	2017	61.3	76.5	44.1
El Salvador	San Julián	Count	2000	0	1	0
El Salvador	San Julián	Count	2017	1	1	0
El Salvador	Mercedes Umaña	Proportion	2000	49.2	67.2	30.8
El Salvador	Mercedes Umaña	Proportion	2017	56.6	75.9	39.4
El Salvador	Mercedes Umaña	Count	2000	0	1	0
El Salvador	Mercedes Umaña	Count	2017	1	1	0
El Salvador	Turín	Proportion	2000	48.7	67.3	29.3
El Salvador	Turín	Proportion	2017	60.6	76.4	42.3
El Salvador	Turín	Count	2000	0	1	0
El Salvador	Turín	Count	2017	1	1	0
El Salvador	La Laguna	Proportion	2000	47.9	67.3	26.6
El Salvador	La Laguna	Proportion	2017	55.1	74.5	35.5
El Salvador	La Laguna	Count	2000	0	1	0
El Salvador	La Laguna	Count	2017	1	1	0
El Salvador	San Ramón	Proportion	2000	53.2	71.4	33.5
El Salvador	San Ramón	Proportion	2017	59.9	77.6	38.4
El Salvador	San Ramón	Count	2000	1	1	0
El Salvador	San Ramón	Count	2017	1	1	0
El Salvador	Opico	Proportion	2000	50.6	67.9	30.6
El Salvador	Opico	Proportion	2017	60.1	77.0	44.4
El Salvador	Opico	Count	2000	1	1	0
El Salvador	Opico	Count	2017	1	1	0
El Salvador	San Juan Tepezontes	Proportion	2000	52.4	70.9	31.8
El Salvador	San Juan Tepezontes	Proportion	2017	59.3	78.7	39.4
El Salvador	San Juan Tepezontes	Count	2000	1	1	0
El Salvador	San Juan Tepezontes	Count	2017	1	1	0
El Salvador	Pasaquina	Proportion	2000	48.3	65.3	31.8
El Salvador	Pasaquina	Proportion	2017	52.6	71.7	35.4
El Salvador	Pasaquina	Count	2000	0	1	0
El Salvador	Pasaquina	Count	2017	1	1	0
El Salvador	Jocoro	Proportion	2000	46.8	65.1	29.7
El Salvador	Jocoro	Proportion	2017	52.5	74.0	34.5
El Salvador	Jocoro	Count	2000	0	1	0
El Salvador	Jocoro	Count	2017	1	1	0
El Salvador	Quelepa	Proportion	2000	48.6	68.2	30.1
El Salvador	Quelepa	Proportion	2017	54.0	73.9	33.1
El Salvador	Quelepa	Count	2000	0	1	0

Table 4: ORS by Unit *(continued)*

Country	Unit	Metric	Year	Mean	Upper	Lower
El Salvador	Quelepa	Count	2017	1	1	0
El Salvador	Panchimalco	Proportion	2000	51.4	68.5	34.0
El Salvador	Panchimalco	Proportion	2017	59.5	78.4	42.1
El Salvador	Panchimalco	Count	2000	1	1	0
El Salvador	Panchimalco	Count	2017	1	1	0
El Salvador	Tepetitán	Proportion	2000	53.9	70.4	35.1
El Salvador	Tepetitán	Proportion	2017	59.8	77.2	42.7
El Salvador	Tepetitán	Count	2000	1	1	0
El Salvador	Tepetitán	Count	2017	1	1	0
El Salvador	Santa Ana	Proportion	2000	49.0	66.1	29.1
El Salvador	Santa Ana	Proportion	2017	59.9	77.8	43.1
El Salvador	Santa Ana	Count	2000	0	1	0
El Salvador	Santa Ana	Count	2017	1	1	0
El Salvador	Santa Catarina Masahuat	Proportion	2000	49.4	68.9	27.6
El Salvador	Santa Catarina Masahuat	Proportion	2017	61.7	78.9	43.7
El Salvador	Santa Catarina Masahuat	Count	2000	0	1	0
El Salvador	Santa Catarina Masahuat	Count	2017	1	1	0
El Salvador	Nueva Granada	Proportion	2000	48.8	67.0	31.4
El Salvador	Nueva Granada	Proportion	2017	55.9	74.7	38.6
El Salvador	Nueva Granada	Count	2000	0	1	0
El Salvador	Nueva Granada	Count	2017	1	1	0
El Salvador	La Palma	Proportion	2000	47.3	64.8	28.5
El Salvador	La Palma	Proportion	2017	54.4	72.3	37.4
El Salvador	La Palma	Count	2000	0	1	0
El Salvador	La Palma	Count	2017	1	1	0
El Salvador	Santa Cruz Analquito	Proportion	2000	53.0	71.1	33.3
El Salvador	Santa Cruz Analquito	Proportion	2017	59.6	77.9	43.0
El Salvador	Santa Cruz Analquito	Count	2000	1	1	0
El Salvador	Santa Cruz Analquito	Count	2017	1	1	0
El Salvador	Quezaltepeque	Proportion	2000	50.3	69.2	30.2
El Salvador	Quezaltepeque	Proportion	2017	58.7	77.0	40.7
El Salvador	Quezaltepeque	Count	2000	1	1	0
El Salvador	Quezaltepeque	Count	2017	1	1	0
El Salvador	San Luis La Herradura	Proportion	2000	52.7	69.3	34.3
El Salvador	San Luis La Herradura	Proportion	2017	59.0	77.9	42.1
El Salvador	San Luis La Herradura	Count	2000	1	1	0
El Salvador	San Luis La Herradura	Count	2017	1	1	0
El Salvador	Polorós	Proportion	2000	48.4	67.4	30.0
El Salvador	Polorós	Proportion	2017	53.4	73.7	35.2
El Salvador	Polorós	Count	2000	0	1	0
El Salvador	Polorós	Count	2017	1	1	0
El Salvador	Lolotiquillo	Proportion	2000	48.4	66.5	29.3
El Salvador	Lolotiquillo	Proportion	2017	53.3	75.5	34.1
El Salvador	Lolotiquillo	Count	2000	0	1	0
El Salvador	Lolotiquillo	Count	2017	1	1	0
El Salvador	San Antonio	Proportion	2000	49.8	68.5	31.5
El Salvador	San Antonio	Proportion	2017	56.8	76.7	36.2
El Salvador	San Antonio	Count	2000	0	1	0
El Salvador	San Antonio	Count	2017	1	1	0

Table 4: ORS by Unit *(continued)*

Country	Unit	Metric	Year	Mean	Upper	Lower
El Salvador	Rosario de Mora	Proportion	2000	50.6	69.5	32.9
El Salvador	Rosario de Mora	Proportion	2017	58.5	77.5	40.8
El Salvador	Rosario de Mora	Count	2000	1	1	0
El Salvador	Rosario de Mora	Count	2017	1	1	0
El Salvador	Verapaz	Proportion	2000	53.9	71.2	35.6
El Salvador	Verapaz	Proportion	2017	60.0	79.1	42.4
El Salvador	Verapaz	Count	2000	1	1	0
El Salvador	Verapaz	Count	2017	1	1	0
El Salvador	Santa Rosa Guachipilín	Proportion	2000	46.3	66.1	25.7
El Salvador	Santa Rosa Guachipilín	Proportion	2017	56.0	72.6	38.7
El Salvador	Santa Rosa Guachipilín	Count	2000	0	1	0
El Salvador	Santa Rosa Guachipilín	Count	2017	1	1	0
El Salvador	Santa Isabel Ishuatán	Proportion	2000	49.3	68.7	31.5
El Salvador	Santa Isabel Ishuatán	Proportion	2017	61.1	78.8	43.4
El Salvador	Santa Isabel Ishuatán	Count	2000	0	1	0
El Salvador	Santa Isabel Ishuatán	Count	2017	1	1	0
El Salvador	Ozatlán	Proportion	2000	49.6	67.4	31.7
El Salvador	Ozatlán	Proportion	2017	55.6	73.3	38.4
El Salvador	Ozatlán	Count	2000	0	1	0
El Salvador	Ozatlán	Count	2017	1	1	0
El Salvador	La Reina	Proportion	2000	47.4	65.1	28.6
El Salvador	La Reina	Proportion	2017	55.3	73.3	38.9
El Salvador	La Reina	Count	2000	0	1	0
El Salvador	La Reina	Count	2017	1	1	0
El Salvador	Santa Cruz Michapa	Proportion	2000	52.9	71.2	33.4
El Salvador	Santa Cruz Michapa	Proportion	2017	59.2	78.0	41.6
El Salvador	Santa Cruz Michapa	Count	2000	1	1	0
El Salvador	Santa Cruz Michapa	Count	2017	1	1	0
El Salvador	Sacacoyo	Proportion	2000	51.3	70.0	29.7
El Salvador	Sacacoyo	Proportion	2017	60.8	77.7	44.1
El Salvador	Sacacoyo	Count	2000	1	1	0
El Salvador	Sacacoyo	Count	2017	1	1	0
El Salvador	San Luis Talpa	Proportion	2000	50.8	68.2	31.4
El Salvador	San Luis Talpa	Proportion	2017	58.0	77.8	38.7
El Salvador	San Luis Talpa	Count	2000	1	1	0
El Salvador	San Luis Talpa	Count	2017	1	1	0
El Salvador	San Alejo	Proportion	2000	47.7	65.4	30.8
El Salvador	San Alejo	Proportion	2017	53.4	71.9	36.1
El Salvador	San Alejo	Count	2000	0	1	0
El Salvador	San Alejo	Count	2017	1	1	0
El Salvador	Meanguera	Proportion	2000	48.8	66.2	32.4
El Salvador	Meanguera	Proportion	2017	55.3	74.9	37.0
El Salvador	Meanguera	Count	2000	0	1	0
El Salvador	Meanguera	Count	2017	1	1	0
El Salvador	San Gerardo	Proportion	2000	49.2	65.9	34.0
El Salvador	San Gerardo	Proportion	2017	57.0	74.2	38.9
El Salvador	San Gerardo	Count	2000	0	1	0
El Salvador	San Gerardo	Count	2017	1	1	0
El Salvador	San Marcos	Proportion	2000	51.1	68.5	33.0

Table 4: ORS by Unit *(continued)*

Country	Unit	Metric	Year	Mean	Upper	Lower
El Salvador	San Marcos	Proportion	2017	59.7	77.8	41.4
El Salvador	San Marcos	Count	2000	1	1	0
El Salvador	San Marcos	Count	2017	1	1	0
El Salvador	Santiago de la Frontera	Proportion	2000	46.5	66.7	27.9
El Salvador	Santiago de la Frontera	Proportion	2017	58.9	75.7	43.8
El Salvador	Santiago de la Frontera	Count	2000	0	1	0
El Salvador	Santiago de la Frontera	Count	2017	1	1	0
El Salvador	Santo Domingo	Proportion	2000	47.4	65.8	28.8
El Salvador	Santo Domingo	Proportion	2017	60.1	76.9	41.4
El Salvador	Santo Domingo	Count	2000	0	1	0
El Salvador	Santo Domingo	Count	2017	1	1	0
El Salvador	Puerto El Triunfo	Proportion	2000	51.1	70.0	32.2
El Salvador	Puerto El Triunfo	Proportion	2017	56.6	75.9	37.0
El Salvador	Puerto El Triunfo	Count	2000	1	1	0
El Salvador	Puerto El Triunfo	Count	2017	1	1	0
El Salvador	Las Vueltas	Proportion	2000	48.5	66.5	28.8
El Salvador	Las Vueltas	Proportion	2017	56.2	73.3	39.8
El Salvador	Las Vueltas	Count	2000	0	1	0
El Salvador	Las Vueltas	Count	2017	1	1	0
El Salvador	Suchitoto	Proportion	2000	50.0	66.9	33.2
El Salvador	Suchitoto	Proportion	2017	57.6	73.7	40.3
El Salvador	Suchitoto	Count	2000	0	1	0
El Salvador	Suchitoto	Count	2017	1	1	0
El Salvador	San José Villanueva	Proportion	2000	52.2	70.7	33.5
El Salvador	San José Villanueva	Proportion	2017	60.5	79.4	43.7
El Salvador	San José Villanueva	Count	2000	1	1	0
El Salvador	San José Villanueva	Count	2017	1	1	0
El Salvador	San Miguel Tepezontes	Proportion	2000	52.6	71.3	30.8
El Salvador	San Miguel Tepezontes	Proportion	2017	59.3	78.5	39.5
El Salvador	San Miguel Tepezontes	Count	2000	1	1	0
El Salvador	San Miguel Tepezontes	Count	2017	1	1	0
El Salvador	San José	Proportion	2000	47.6	64.8	28.5
El Salvador	San José	Proportion	2017	52.2	73.5	33.4
El Salvador	San José	Count	2000	0	1	0
El Salvador	San José	Count	2017	1	1	0
El Salvador	Osicala	Proportion	2000	48.4	66.2	30.2
El Salvador	Osicala	Proportion	2017	55.3	74.2	36.0
El Salvador	Osicala	Count	2000	0	1	0
El Salvador	Osicala	Count	2017	1	1	0
El Salvador	San Jorge	Proportion	2000	48.7	66.3	30.6
El Salvador	San Jorge	Proportion	2017	54.5	73.2	37.6
El Salvador	San Jorge	Count	2000	0	1	0
El Salvador	San Jorge	Count	2017	1	1	0
El Salvador	San Martín	Proportion	2000	50.6	69.4	31.2
El Salvador	San Martín	Proportion	2017	58.9	78.1	40.5
El Salvador	San Martín	Count	2000	1	1	0
El Salvador	San Martín	Count	2017	1	1	0
El Salvador	Texistepeque	Proportion	2000	46.4	66.1	28.0
El Salvador	Texistepeque	Proportion	2017	57.6	76.3	40.5

Table 4: ORS by Unit *(continued)*

Country	Unit	Metric	Year	Mean	Upper	Lower
El Salvador	Texistepeque	Count	2000	0	1	0
El Salvador	Texistepeque	Count	2017	1	1	0
El Salvador	Sonsonate	Proportion	2000	48.5	66.7	28.9
El Salvador	Sonsonate	Proportion	2017	60.2	76.3	42.6
El Salvador	Sonsonate	Count	2000	0	1	0
El Salvador	Sonsonate	Count	2017	1	1	0
El Salvador	San Agustín	Proportion	2000	50.5	67.1	31.4
El Salvador	San Agustín	Proportion	2017	57.4	75.8	38.2
El Salvador	San Agustín	Count	2000	1	1	0
El Salvador	San Agustín	Count	2017	1	1	0
El Salvador	Nombre de Jesús	Proportion	2000	51.0	68.5	32.6
El Salvador	Nombre de Jesús	Proportion	2017	59.2	74.8	43.2
El Salvador	Nombre de Jesús	Count	2000	1	1	0
El Salvador	Nombre de Jesús	Count	2017	1	1	0
El Salvador	Tenancingo	Proportion	2000	51.0	68.5	30.8
El Salvador	Tenancingo	Proportion	2017	58.7	77.8	40.8
El Salvador	Tenancingo	Count	2000	1	1	0
El Salvador	Tenancingo	Count	2017	1	1	0
El Salvador	San Matías	Proportion	2000	48.5	65.6	28.7
El Salvador	San Matías	Proportion	2017	58.1	75.1	40.2
El Salvador	San Matías	Count	2000	0	1	0
El Salvador	San Matías	Count	2017	1	1	0
El Salvador	San Pedro Masahuat	Proportion	2000	51.3	69.4	32.9
El Salvador	San Pedro Masahuat	Proportion	2017	58.2	76.7	38.7
El Salvador	San Pedro Masahuat	Count	2000	1	1	0
El Salvador	San Pedro Masahuat	Count	2017	1	1	0
El Salvador	Santa Rosa de Lima	Proportion	2000	47.6	65.3	29.5
El Salvador	Santa Rosa de Lima	Proportion	2017	51.9	72.3	32.2
El Salvador	Santa Rosa de Lima	Count	2000	0	1	0
El Salvador	Santa Rosa de Lima	Count	2017	1	1	0
El Salvador	Perquín	Proportion	2000	51.3	68.7	33.3
El Salvador	Perquín	Proportion	2017	57.9	76.2	39.0
El Salvador	Perquín	Count	2000	1	1	0
El Salvador	Perquín	Count	2017	1	1	0
El Salvador	San Luis de la Reina	Proportion	2000	48.9	66.8	31.0
El Salvador	San Luis de la Reina	Proportion	2017	55.9	74.7	36.2
El Salvador	San Luis de la Reina	Count	2000	0	1	0
El Salvador	San Luis de la Reina	Count	2017	1	1	0
El Salvador	San Salvador	Proportion	2000	50.6	68.8	30.7
El Salvador	San Salvador	Proportion	2017	59.6	77.8	41.8
El Salvador	San Salvador	Count	2000	1	1	0
El Salvador	San Salvador	Count	2017	1	1	0
El Salvador	Sonzacate	Proportion	2000	50.2	70.0	30.3
El Salvador	Sonzacate	Proportion	2017	61.6	78.0	42.9
El Salvador	Sonzacate	Count	2000	1	1	0
El Salvador	Sonzacate	Count	2017	1	1	0
El Salvador	San Buenaventura	Proportion	2000	49.8	66.7	31.1
El Salvador	San Buenaventura	Proportion	2017	56.1	75.3	38.4
El Salvador	San Buenaventura	Count	2000	0	1	0

Table 4: ORS by Unit *(continued)*

Country	Unit	Metric	Year	Mean	Upper	Lower
El Salvador	San Buenaventura	Count	2017	1	1	0
El Salvador	Nueva Concepción	Proportion	2000	47.1	65.1	29.0
El Salvador	Nueva Concepción	Proportion	2017	56.2	74.2	38.7
El Salvador	Nueva Concepción	Count	2000	0	1	0
El Salvador	Nueva Concepción	Count	2017	1	1	0
El Salvador	San Pablo Tacachico	Proportion	2000	47.8	66.8	29.6
El Salvador	San Pablo Tacachico	Proportion	2017	57.3	75.5	37.1
El Salvador	San Pablo Tacachico	Count	2000	0	1	0
El Salvador	San Pablo Tacachico	Count	2017	1	1	0
El Salvador	San Pedro Nonualco	Proportion	2000	53.3	70.8	35.0
El Salvador	San Pedro Nonualco	Proportion	2017	59.9	76.8	41.6
El Salvador	San Pedro Nonualco	Count	2000	1	1	0
El Salvador	San Pedro Nonualco	Count	2017	1	1	0
El Salvador	Yayantique	Proportion	2000	47.1	66.3	29.3
El Salvador	Yayantique	Proportion	2017	52.8	72.9	35.4
El Salvador	Yayantique	Count	2000	0	1	0
El Salvador	Yayantique	Count	2017	1	1	0
El Salvador	San Carlos	Proportion	2000	46.9	65.0	28.8
El Salvador	San Carlos	Proportion	2017	52.5	73.0	33.3
El Salvador	San Carlos	Count	2000	0	1	0
El Salvador	San Carlos	Count	2017	1	1	0
El Salvador	San Miguel	Proportion	2000	48.0	66.0	29.4
El Salvador	San Miguel	Proportion	2017	53.4	71.5	34.8
El Salvador	San Miguel	Count	2000	0	1	0
El Salvador	San Miguel	Count	2017	1	1	0
El Salvador	Santiago Texacuangos	Proportion	2000	51.1	69.5	32.4
El Salvador	Santiago Texacuangos	Proportion	2017	58.6	77.5	39.5
El Salvador	Santiago Texacuangos	Count	2000	1	1	0
El Salvador	Santiago Texacuangos	Count	2017	1	1	0
El Salvador	San Dionisio	Proportion	2000	49.7	67.7	32.6
El Salvador	San Dionisio	Proportion	2017	55.9	73.2	37.6
El Salvador	San Dionisio	Count	2000	0	1	0
El Salvador	San Dionisio	Count	2017	1	1	0
El Salvador	Nueva Trinidad	Proportion	2000	48.6	66.7	30.2
El Salvador	Nueva Trinidad	Proportion	2017	56.4	74.4	37.1
El Salvador	Nueva Trinidad	Count	2000	0	1	0
El Salvador	Nueva Trinidad	Count	2017	1	1	0
El Salvador	Talnique	Proportion	2000	51.6	70.0	32.4
El Salvador	Talnique	Proportion	2017	61.1	77.6	43.3
El Salvador	Talnique	Count	2000	1	1	0
El Salvador	Talnique	Count	2017	1	1	0
El Salvador	San Rafael Obrajuelo	Proportion	2000	51.4	68.7	32.6
El Salvador	San Rafael Obrajuelo	Proportion	2017	57.3	75.3	39.1
El Salvador	San Rafael Obrajuelo	Count	2000	1	1	0
El Salvador	San Rafael Obrajuelo	Count	2017	1	1	0
El Salvador	Yucuaiquín	Proportion	2000	47.6	65.1	30.2
El Salvador	Yucuaiquín	Proportion	2017	53.8	73.4	34.5
El Salvador	Yucuaiquín	Count	2000	0	1	0
El Salvador	Yucuaiquín	Count	2017	1	1	0

Table 4: ORS by Unit *(continued)*

Country	Unit	Metric	Year	Mean	Upper	Lower
El Salvador	San Fernando	Proportion	2000	50.2	70.2	29.1
El Salvador	San Fernando	Proportion	2017	56.8	77.4	37.2
El Salvador	San Fernando	Count	2000	1	1	0
El Salvador	San Fernando	Count	2017	1	1	0
El Salvador	San Rafael	Proportion	2000	48.1	66.5	28.7
El Salvador	San Rafael	Proportion	2017	53.8	74.0	35.0
El Salvador	San Rafael	Count	2000	0	1	0
El Salvador	San Rafael	Count	2017	1	1	0
El Salvador	Santo Tomás	Proportion	2000	50.9	69.3	31.4
El Salvador	Santo Tomás	Proportion	2017	58.6	78.1	38.2
El Salvador	Santo Tomás	Count	2000	1	1	0
El Salvador	Santo Tomás	Count	2017	1	1	0
El Salvador	San Francisco Javier	Proportion	2000	50.2	68.6	30.4
El Salvador	San Francisco Javier	Proportion	2017	56.7	75.0	38.0
El Salvador	San Francisco Javier	Count	2000	1	1	0
El Salvador	San Francisco Javier	Count	2017	1	1	0
El Salvador	Ojos de Agua	Proportion	2000	48.7	67.9	29.5
El Salvador	Ojos de Agua	Proportion	2017	56.3	73.2	37.0
El Salvador	Ojos de Agua	Count	2000	0	1	0
El Salvador	Ojos de Agua	Count	2017	1	1	0
El Salvador	Tamanique	Proportion	2000	50.9	70.6	32.7
El Salvador	Tamanique	Proportion	2017	60.4	78.9	43.5
El Salvador	Tamanique	Count	2000	1	1	0
El Salvador	Tamanique	Count	2017	1	1	0
El Salvador	Santa María Ostuma	Proportion	2000	53.6	71.6	34.8
El Salvador	Santa María Ostuma	Proportion	2017	60.1	78.0	42.4
El Salvador	Santa María Ostuma	Count	2000	1	1	0
El Salvador	Santa María Ostuma	Count	2017	1	1	0
El Salvador	San Francisco Gotera	Proportion	2000	48.1	66.5	28.6
El Salvador	San Francisco Gotera	Proportion	2017	52.5	74.2	33.7
El Salvador	San Francisco Gotera	Count	2000	0	1	0
El Salvador	San Francisco Gotera	Count	2017	1	1	0
El Salvador	Sesori	Proportion	2000	48.5	64.4	32.1
El Salvador	Sesori	Proportion	2017	56.0	75.0	38.6
El Salvador	Sesori	Count	2000	0	1	0
El Salvador	Sesori	Count	2017	1	1	0
El Salvador	Soyapango	Proportion	2000	49.9	68.7	29.5
El Salvador	Soyapango	Proportion	2017	58.5	76.0	41.7
El Salvador	Soyapango	Count	2000	0	1	0
El Salvador	Soyapango	Count	2017	1	1	0
El Salvador	Santa Elena	Proportion	2000	48.8	65.9	30.9
El Salvador	Santa Elena	Proportion	2017	54.9	72.7	37.3
El Salvador	Santa Elena	Count	2000	0	1	0
El Salvador	Santa Elena	Count	2017	1	1	0
El Salvador	Potonico	Proportion	2000	49.2	66.7	30.7
El Salvador	Potonico	Proportion	2017	57.4	74.6	42.2
El Salvador	Potonico	Count	2000	0	1	0
El Salvador	Potonico	Count	2017	1	1	0
El Salvador	Teotepeque	Proportion	2000	49.7	68.1	32.6

Table 4: ORS by Unit *(continued)*

Country	Unit	Metric	Year	Mean	Upper	Lower
El Salvador	Teotepeque	Proportion	2017	61.1	77.4	45.5
El Salvador	Teotepeque	Count	2000	0	1	0
El Salvador	Teotepeque	Count	2017	1	1	0
El Salvador	Santiago Nonualco	Proportion	2000	51.8	69.0	32.8
El Salvador	Santiago Nonualco	Proportion	2017	58.2	75.5	38.8
El Salvador	Santiago Nonualco	Count	2000	1	1	0
El Salvador	Santiago Nonualco	Count	2017	1	1	0
El Salvador	San Isidro	Proportion	2000	49.4	67.1	30.8
El Salvador	San Isidro	Proportion	2017	56.3	76.4	36.0
El Salvador	San Isidro	Count	2000	0	1	0
El Salvador	San Isidro	Count	2017	1	1	0
El Salvador	Uluazapa	Proportion	2000	47.5	66.3	30.3
El Salvador	Uluazapa	Proportion	2017	53.4	72.8	34.9
El Salvador	Uluazapa	Count	2000	0	1	0
El Salvador	Uluazapa	Count	2017	1	1	0
El Salvador	Tonacatepeque	Proportion	2000	50.4	70.0	31.2
El Salvador	Tonacatepeque	Proportion	2017	57.9	77.2	39.9
El Salvador	Tonacatepeque	Count	2000	1	1	0
El Salvador	Tonacatepeque	Count	2017	1	1	0
El Salvador	Santa María	Proportion	2000	48.4	66.0	29.9
El Salvador	Santa María	Proportion	2017	53.8	73.2	34.4
El Salvador	Santa María	Count	2000	0	1	0
El Salvador	Santa María	Count	2017	1	1	0
El Salvador	San Antonio de la Cruz	Proportion	2000	48.3	66.0	28.9
El Salvador	San Antonio de la Cruz	Proportion	2017	56.4	74.7	38.1
El Salvador	San Antonio de la Cruz	Count	2000	0	1	0
El Salvador	San Antonio de la Cruz	Count	2017	1	1	0
El Salvador	Tepecoyo	Proportion	2000	50.9	68.7	31.5
El Salvador	Tepecoyo	Proportion	2017	61.1	78.1	46.7
El Salvador	Tepecoyo	Count	2000	1	1	0
El Salvador	Tepecoyo	Count	2017	1	1	0
El Salvador	Tapalhuaca	Proportion	2000	51.2	70.0	30.6
El Salvador	Tapalhuaca	Proportion	2017	58.4	78.0	40.8
El Salvador	Tapalhuaca	Count	2000	1	1	0
El Salvador	Tapalhuaca	Count	2017	1	1	0
El Salvador	San Simón	Proportion	2000	50.3	68.4	33.8
El Salvador	San Simón	Proportion	2017	56.5	76.4	36.2
El Salvador	San Simón	Count	2000	1	1	0
El Salvador	San Simón	Count	2017	1	1	0
El Salvador	Santiago de María	Proportion	2000	51.2	68.5	32.5
El Salvador	Santiago de María	Proportion	2017	57.0	74.8	38.5
El Salvador	Santiago de María	Count	2000	1	1	0
El Salvador	Santiago de María	Count	2017	1	1	0
El Salvador	San Antonio Los Ranchos	Proportion	2000	50.0	67.8	29.5
El Salvador	San Antonio Los Ranchos	Proportion	2017	56.5	73.7	36.9
El Salvador	San Antonio Los Ranchos	Count	2000	0	1	0
El Salvador	San Antonio Los Ranchos	Count	2017	1	1	0
El Salvador	Zaragoza	Proportion	2000	52.0	71.4	33.1
El Salvador	Zaragoza	Proportion	2017	60.7	78.2	42.2

Table 4: ORS by Unit *(continued)*

Country	Unit	Metric	Year	Mean	Upper	Lower
El Salvador	Zaragoza	Count	2000	1	1	0
El Salvador	Zaragoza	Count	2017	1	1	0
El Salvador	Zacatecoluca	Proportion	2000	51.4	67.6	33.3
El Salvador	Zacatecoluca	Proportion	2017	57.9	75.8	39.4
El Salvador	Zacatecoluca	Count	2000	1	1	0
El Salvador	Zacatecoluca	Count	2017	1	1	0
El Salvador	Sensembra	Proportion	2000	47.9	65.3	29.5
El Salvador	Sensembra	Proportion	2017	54.4	75.0	36.7
El Salvador	Sensembra	Count	2000	0	1	0
El Salvador	Sensembra	Count	2017	1	1	0
El Salvador	Tecapán	Proportion	2000	50.6	68.0	31.3
El Salvador	Tecapán	Proportion	2017	57.2	75.5	40.5
El Salvador	Tecapán	Count	2000	1	1	0
El Salvador	Tecapán	Count	2017	1	1	0
El Salvador	San Fernando	Proportion	2000	48.8	66.9	31.3
El Salvador	San Fernando	Proportion	2017	55.9	72.4	39.1
El Salvador	San Fernando	Count	2000	0	1	0
El Salvador	San Fernando	Count	2017	1	1	0
El Salvador	Sociedad	Proportion	2000	47.7	64.6	31.0
El Salvador	Sociedad	Proportion	2017	53.2	74.0	34.1
El Salvador	Sociedad	Count	2000	0	1	0
El Salvador	Sociedad	Count	2017	1	1	0
El Salvador	Usulután	Proportion	2000	49.1	67.7	31.8
El Salvador	Usulután	Proportion	2017	55.1	73.5	35.6
El Salvador	Usulután	Count	2000	0	1	0
El Salvador	Usulután	Count	2017	1	1	0
El Salvador	San Francisco Lempa	Proportion	2000	49.9	69.7	30.6
El Salvador	San Francisco Lempa	Proportion	2017	57.7	76.3	38.3
El Salvador	San Francisco Lempa	Count	2000	0	1	0
El Salvador	San Francisco Lempa	Count	2017	1	1	0
El Salvador	Torola	Proportion	2000	51.2	69.7	34.1
El Salvador	Torola	Proportion	2017	57.4	77.1	38.3
El Salvador	Torola	Count	2000	1	1	0
El Salvador	Torola	Count	2017	1	1	0
El Salvador	San Francisco Morazán	Proportion	2000	47.4	65.9	28.1
El Salvador	San Francisco Morazán	Proportion	2017	55.4	72.3	37.5
El Salvador	San Francisco Morazán	Count	2000	0	1	0
El Salvador	San Francisco Morazán	Count	2017	1	1	0
El Salvador	Yamabal	Proportion	2000	47.7	65.2	31.3
El Salvador	Yamabal	Proportion	2017	54.2	73.8	34.7
El Salvador	Yamabal	Count	2000	0	1	0
El Salvador	Yamabal	Count	2017	1	1	0
El Salvador	San Ignacio	Proportion	2000	49.7	66.4	30.6
El Salvador	San Ignacio	Proportion	2017	55.7	72.3	38.9
El Salvador	San Ignacio	Count	2000	0	1	0
El Salvador	San Ignacio	Count	2017	1	1	0
El Salvador	Yoloaiquín	Proportion	2000	47.8	65.7	28.4
El Salvador	Yoloaiquín	Proportion	2017	54.4	76.1	34.9
El Salvador	Yoloaiquín	Count	2000	0	1	0

Table 4: ORS by Unit *(continued)*

Country	Unit	Metric	Year	Mean	Upper	Lower
El Salvador	Yoloaiquín	Count	2017	1	1	0
El Salvador	San Isidro Labrador	Proportion	2000	49.1	66.1	30.5
El Salvador	San Isidro Labrador	Proportion	2017	56.1	74.5	37.5
El Salvador	San Isidro Labrador	Count	2000	0	1	0
El Salvador	San Isidro Labrador	Count	2017	1	1	0
El Salvador	San José Cancasque	Proportion	2000	49.1	68.0	31.2
El Salvador	San José Cancasque	Proportion	2017	57.4	76.2	39.0
El Salvador	San José Cancasque	Count	2000	0	1	0
El Salvador	San José Cancasque	Count	2017	1	1	0
El Salvador	San José Las Flores	Proportion	2000	48.6	67.4	29.5
El Salvador	San José Las Flores	Proportion	2017	56.1	74.7	38.3
El Salvador	San José Las Flores	Count	2000	0	1	0
El Salvador	San José Las Flores	Count	2017	1	1	0
El Salvador	San Luis del Carmen	Proportion	2000	49.5	69.9	28.1
El Salvador	San Luis del Carmen	Proportion	2017	57.5	76.1	39.5
El Salvador	San Luis del Carmen	Count	2000	0	1	0
El Salvador	San Luis del Carmen	Count	2017	1	1	0
El Salvador	San Miguel de Mercedes	Proportion	2000	49.1	67.5	31.1
El Salvador	San Miguel de Mercedes	Proportion	2017	56.0	75.6	35.5
El Salvador	San Miguel de Mercedes	Count	2000	0	1	0
El Salvador	San Miguel de Mercedes	Count	2017	1	1	0
El Salvador	San Rafael	Proportion	2000	47.6	65.4	27.5
El Salvador	San Rafael	Proportion	2017	54.9	72.1	37.5
El Salvador	San Rafael	Count	2000	0	1	0
El Salvador	San Rafael	Count	2017	1	1	0
El Salvador	Santa Rita	Proportion	2000	48.2	65.7	28.4
El Salvador	Santa Rita	Proportion	2017	55.2	73.3	36.8
El Salvador	Santa Rita	Count	2000	0	1	0
El Salvador	Santa Rita	Count	2017	1	1	0
El Salvador	Tejutla	Proportion	2000	47.7	65.5	28.0
El Salvador	Tejutla	Proportion	2017	55.3	73.4	37.1
El Salvador	Tejutla	Count	2000	0	1	0
El Salvador	Tejutla	Count	2017	1	1	0
Equatorial Guinea	Mabana	Proportion	2000	NA	NA	NA
Equatorial Guinea	Mabana	Proportion	2017	NA	NA	NA
Equatorial Guinea	Mabana	Count	2000	NA	NA	NA
Equatorial Guinea	Mabana	Count	2017	NA	NA	NA
Equatorial Guinea	Baney	Proportion	2000	59.4	69.1	50.3
Equatorial Guinea	Baney	Proportion	2017	50.0	78.4	23.6
Equatorial Guinea	Baney	Count	2000	1	1	1
Equatorial Guinea	Baney	Count	2017	0	1	0
Equatorial Guinea	Luba	Proportion	2000	53.2	63.0	43.3
Equatorial Guinea	Luba	Proportion	2017	45.8	73.4	20.2
Equatorial Guinea	Luba	Count	2000	1	1	0
Equatorial Guinea	Luba	Count	2017	0	1	0
Equatorial Guinea	Acurenam	Proportion	2000	27.0	33.0	21.2
Equatorial Guinea	Acurenam	Proportion	2017	41.8	69.4	19.7
Equatorial Guinea	Acurenam	Count	2000	0	0	0
Equatorial Guinea	Acurenam	Count	2017	0	1	0

Table 4: ORS by Unit *(continued)*

Country	Unit	Metric	Year	Mean	Upper	Lower
Equatorial Guinea	Bidjabidjan	Proportion	2000	24.1	30.5	18.3
Equatorial Guinea	Bidjabidjan	Proportion	2017	39.5	68.1	18.4
Equatorial Guinea	Bidjabidjan	Count	2000	0	0	0
Equatorial Guinea	Bidjabidjan	Count	2017	0	1	0
Equatorial Guinea	Bata	Proportion	2000	31.9	39.0	25.7
Equatorial Guinea	Bata	Proportion	2017	45.4	71.2	23.2
Equatorial Guinea	Bata	Count	2000	0	0	0
Equatorial Guinea	Bata	Count	2017	0	1	0
Equatorial Guinea	Aconibe	Proportion	2000	25.2	34.0	18.5
Equatorial Guinea	Aconibe	Proportion	2017	38.7	67.2	17.2
Equatorial Guinea	Aconibe	Count	2000	0	0	0
Equatorial Guinea	Aconibe	Count	2017	0	1	0
Equatorial Guinea	San Antonio de Palé	Proportion	2000	NA	NA	NA
Equatorial Guinea	San Antonio de Palé	Proportion	2017	NA	NA	NA
Equatorial Guinea	San Antonio de Palé	Count	2000	NA	NA	NA
Equatorial Guinea	San Antonio de Palé	Count	2017	NA	NA	NA
Equatorial Guinea	Malabo	Proportion	2000	53.5	62.3	44.8
Equatorial Guinea	Malabo	Proportion	2017	45.4	74.8	19.7
Equatorial Guinea	Malabo	Count	2000	1	1	0
Equatorial Guinea	Malabo	Count	2017	0	1	0
Equatorial Guinea	Riaba	Proportion	2000	53.1	62.6	43.5
Equatorial Guinea	Riaba	Proportion	2017	45.3	74.0	20.2
Equatorial Guinea	Riaba	Count	2000	1	1	0
Equatorial Guinea	Riaba	Count	2017	0	1	0
Equatorial Guinea	Bicurga	Proportion	2000	25.6	31.8	20.6
Equatorial Guinea	Bicurga	Proportion	2017	42.7	69.5	20.7
Equatorial Guinea	Bicurga	Count	2000	0	0	0
Equatorial Guinea	Bicurga	Count	2017	0	1	0
Equatorial Guinea	Ebebiyin	Proportion	2000	23.7	30.1	18.1
Equatorial Guinea	Ebebiyin	Proportion	2017	39.4	68.0	18.1
Equatorial Guinea	Ebebiyin	Count	2000	0	0	0
Equatorial Guinea	Ebebiyin	Count	2017	0	1	0
Equatorial Guinea	Bitica	Proportion	2000	29.4	36.7	23.2
Equatorial Guinea	Bitica	Proportion	2017	44.1	69.4	22.2
Equatorial Guinea	Bitica	Count	2000	0	0	0
Equatorial Guinea	Bitica	Count	2017	0	1	0
Equatorial Guinea	Añisok	Proportion	2000	23.2	28.4	18.1
Equatorial Guinea	Añisok	Proportion	2017	40.2	67.7	19.8
Equatorial Guinea	Añisok	Count	2000	0	0	0
Equatorial Guinea	Añisok	Count	2017	0	1	0
Equatorial Guinea	Rebola	Proportion	2000	55.8	64.7	47.3
Equatorial Guinea	Rebola	Proportion	2017	47.0	76.0	20.7
Equatorial Guinea	Rebola	Count	2000	1	1	0
Equatorial Guinea	Rebola	Count	2017	0	1	0
Equatorial Guinea	Evinayong	Proportion	2000	25.4	30.9	20.5
Equatorial Guinea	Evinayong	Proportion	2017	42.2	70.5	20.2
Equatorial Guinea	Evinayong	Count	2000	0	0	0
Equatorial Guinea	Evinayong	Count	2017	0	1	0
Equatorial Guinea	Micomeseng	Proportion	2000	24.6	31.1	19.0

Table 4: ORS by Unit *(continued)*

Country	Unit	Metric	Year	Mean	Upper	Lower
Equatorial Guinea	Micomeseng	Proportion	2017	40.4	66.6	19.1
Equatorial Guinea	Micomeseng	Count	2000	0	0	0
Equatorial Guinea	Micomeseng	Count	2017	0	1	0
Equatorial Guinea	Cogo	Proportion	2000	26.7	33.5	20.3
Equatorial Guinea	Cogo	Proportion	2017	42.3	68.9	21.0
Equatorial Guinea	Cogo	Count	2000	0	0	0
Equatorial Guinea	Cogo	Count	2017	0	1	0
Equatorial Guinea	Ayene	Proportion	2000	24.0	31.1	18.2
Equatorial Guinea	Ayene	Proportion	2017	39.3	68.0	17.4
Equatorial Guinea	Ayene	Count	2000	0	0	0
Equatorial Guinea	Ayene	Count	2017	0	1	0
Equatorial Guinea	Niefang	Proportion	2000	26.2	31.9	20.6
Equatorial Guinea	Niefang	Proportion	2017	41.6	68.0	19.4
Equatorial Guinea	Niefang	Count	2000	0	0	0
Equatorial Guinea	Niefang	Count	2017	0	1	0
Equatorial Guinea	Nasng	Proportion	2000	23.5	29.4	18.1
Equatorial Guinea	Nasng	Proportion	2017	40.1	67.5	19.1
Equatorial Guinea	Nasng	Count	2000	0	0	0
Equatorial Guinea	Nasng	Count	2017	0	1	0
Equatorial Guinea	Corisco	Proportion	2000	NA	NA	NA
Equatorial Guinea	Corisco	Proportion	2017	NA	NA	NA
Equatorial Guinea	Corisco	Count	2000	NA	NA	NA
Equatorial Guinea	Corisco	Count	2017	NA	NA	NA
Equatorial Guinea	Ciudad Nueva Oyala	Proportion	2000	23.9	28.9	19.1
Equatorial Guinea	Ciudad Nueva Oyala	Proportion	2017	40.7	68.6	18.6
Equatorial Guinea	Ciudad Nueva Oyala	Count	2000	0	0	0
Equatorial Guinea	Ciudad Nueva Oyala	Count	2017	0	1	0
Equatorial Guinea	Nkimi	Proportion	2000	27.1	33.3	20.6
Equatorial Guinea	Nkimi	Proportion	2017	41.2	68.2	18.5
Equatorial Guinea	Nkimi	Count	2000	0	0	0
Equatorial Guinea	Nkimi	Count	2017	0	1	0
Equatorial Guinea	Nkue	Proportion	2000	23.2	29.5	17.4
Equatorial Guinea	Nkue	Proportion	2017	39.2	66.2	18.7
Equatorial Guinea	Nkue	Count	2000	0	0	0
Equatorial Guinea	Nkue	Count	2017	0	1	0
Equatorial Guinea	Machinda	Proportion	2000	31.5	39.4	24.9
Equatorial Guinea	Machinda	Proportion	2017	42.6	69.6	20.4
Equatorial Guinea	Machinda	Count	2000	0	0	0
Equatorial Guinea	Machinda	Count	2017	0	1	0
Equatorial Guinea	Mongomeyen	Proportion	2000	25.7	33.2	19.5
Equatorial Guinea	Mongomeyen	Proportion	2017	40.8	69.0	18.4
Equatorial Guinea	Mongomeyen	Count	2000	0	0	0
Equatorial Guinea	Mongomeyen	Count	2017	0	1	0
Equatorial Guinea	Nsok-Nsomo	Proportion	2000	23.7	28.9	18.9
Equatorial Guinea	Nsok-Nsomo	Proportion	2017	40.3	68.8	19.3
Equatorial Guinea	Nsok-Nsomo	Count	2000	0	0	0
Equatorial Guinea	Nsok-Nsomo	Count	2017	0	1	0
Equatorial Guinea	Mbini	Proportion	2000	29.6	38.2	22.0
Equatorial Guinea	Mbini	Proportion	2017	43.9	70.5	21.1

Table 4: ORS by Unit *(continued)*

Country	Unit	Metric	Year	Mean	Upper	Lower
Equatorial Guinea	Mbini	Count	2000	0	0	0
Equatorial Guinea	Mbini	Count	2017	0	1	0
Equatorial Guinea	Mongomo	Proportion	2000	24.2	31.2	18.2
Equatorial Guinea	Mongomo	Proportion	2017	41.1	69.4	18.2
Equatorial Guinea	Mongomo	Count	2000	0	0	0
Equatorial Guinea	Mongomo	Count	2017	0	1	0
Equatorial Guinea	Rio Campo	Proportion	2000	25.9	33.4	18.9
Equatorial Guinea	Rio Campo	Proportion	2017	41.4	68.2	19.9
Equatorial Guinea	Rio Campo	Count	2000	0	0	0
Equatorial Guinea	Rio Campo	Count	2017	0	1	0
Equatorial Guinea	Nsork	Proportion	2000	25.3	34.5	18.6
Equatorial Guinea	Nsork	Proportion	2017	38.4	67.4	16.4
Equatorial Guinea	Nsork	Count	2000	0	0	0
Equatorial Guinea	Nsork	Count	2017	0	1	0
Eritrea	Asmara City	Proportion	2000	44.7	57.0	32.7
Eritrea	Asmara City	Proportion	2017	45.2	60.1	30.0
Eritrea	Asmara City	Count	2000	0	1	0
Eritrea	Asmara City	Count	2017	0	1	0
Eritrea	Afabet	Proportion	2000	43.5	57.2	30.8
Eritrea	Afabet	Proportion	2017	41.7	56.9	27.5
Eritrea	Afabet	Count	2000	0	1	0
Eritrea	Afabet	Count	2017	0	1	0
Eritrea	Areta’	Proportion	2000	41.4	53.7	30.0
Eritrea	Areta’	Proportion	2017	38.2	54.8	23.8
Eritrea	Areta’	Count	2000	0	1	0
Eritrea	Areta’	Count	2017	0	1	0
Eritrea	Akordat	Proportion	2000	39.3	51.5	28.2
Eritrea	Akordat	Proportion	2017	41.7	58.0	27.8
Eritrea	Akordat	Count	2000	0	1	0
Eritrea	Akordat	Count	2017	0	1	0
Eritrea	Adi Keyih	Proportion	2000	43.3	57.0	32.1
Eritrea	Adi Keyih	Proportion	2017	43.1	57.2	27.9
Eritrea	Adi Keyih	Count	2000	0	1	0
Eritrea	Adi Keyih	Count	2017	0	1	0
Eritrea	Adi Teklezan	Proportion	2000	46.8	60.5	34.8
Eritrea	Adi Teklezan	Proportion	2017	45.5	59.2	31.8
Eritrea	Adi Teklezan	Count	2000	0	1	0
Eritrea	Adi Teklezan	Count	2017	0	1	0
Eritrea	Berikh	Proportion	2000	44.8	57.3	33.4
Eritrea	Berikh	Proportion	2017	45.0	59.8	29.9
Eritrea	Berikh	Count	2000	0	1	0
Eritrea	Berikh	Count	2017	0	1	0
Eritrea	Foro	Proportion	2000	41.1	53.4	29.6
Eritrea	Foro	Proportion	2017	39.7	54.1	25.3
Eritrea	Foro	Count	2000	0	1	0
Eritrea	Foro	Count	2017	0	1	0
Eritrea	Central So. Red-Sea	Proportion	2000	43.1	58.0	30.3
Eritrea	Central So. Red-Sea	Proportion	2017	38.4	54.2	24.1
Eritrea	Central So. Red-Sea	Count	2000	0	1	0

Table 4: ORS by Unit *(continued)*

Country	Unit	Metric	Year	Mean	Upper	Lower
Eritrea	Central So. Red-Sea	Count	2017	0	1	0
Eritrea	Barentu	Proportion	2000	39.3	51.3	27.7
Eritrea	Barentu	Proportion	2017	42.2	57.1	28.0
Eritrea	Barentu	Count	2000	0	1	0
Eritrea	Barentu	Count	2017	0	1	0
Eritrea	Adi Kwala	Proportion	2000	39.6	54.1	29.1
Eritrea	Adi Kwala	Proportion	2017	41.2	56.4	26.8
Eritrea	Adi Kwala	Count	2000	0	1	0
Eritrea	Adi Kwala	Count	2017	0	1	0
Eritrea	Asmat	Proportion	2000	43.2	56.6	30.8
Eritrea	Asmat	Proportion	2017	45.4	61.7	30.7
Eritrea	Asmat	Count	2000	0	1	0
Eritrea	Asmat	Count	2017	0	1	0
Eritrea	Ghala Nefhi	Proportion	2000	44.7	57.7	33.1
Eritrea	Ghala Nefhi	Proportion	2017	44.0	58.9	29.4
Eritrea	Ghala Nefhi	Count	2000	0	1	0
Eritrea	Ghala Nefhi	Count	2017	0	1	0
Eritrea	Ghelaelo'	Proportion	2000	40.4	53.5	28.9
Eritrea	Ghelaelo'	Proportion	2017	39.1	53.1	24.8
Eritrea	Ghelaelo'	Count	2000	0	1	0
Eritrea	Ghelaelo'	Count	2017	0	1	0
Eritrea	Dahlak	Proportion	2000	41.3	55.2	27.4
Eritrea	Dahlak	Proportion	2017	37.9	54.2	24.0
Eritrea	Dahlak	Count	2000	0	1	0
Eritrea	Dahlak	Count	2017	0	1	0
Eritrea	Dghe	Proportion	2000	38.9	51.0	28.6
Eritrea	Dghe	Proportion	2017	42.4	58.7	28.3
Eritrea	Dghe	Count	2000	0	1	0
Eritrea	Dghe	Count	2017	0	1	0
Eritrea	Areza	Proportion	2000	41.1	54.1	29.8
Eritrea	Areza	Proportion	2017	42.5	56.0	29.0
Eritrea	Areza	Count	2000	0	1	0
Eritrea	Areza	Count	2017	0	1	0
Eritrea	Elabered	Proportion	2000	44.7	57.8	32.6
Eritrea	Elabered	Proportion	2017	44.7	58.4	29.5
Eritrea	Elabered	Count	2000	0	1	0
Eritrea	Elabered	Count	2017	0	1	0
Eritrea	Serejeka	Proportion	2000	46.5	59.0	34.8
Eritrea	Serejeka	Proportion	2017	45.7	60.6	31.1
Eritrea	Serejeka	Count	2000	0	1	0
Eritrea	Serejeka	Count	2017	0	1	0
Eritrea	Ghida'e	Proportion	2000	42.9	56.2	31.3
Eritrea	Ghida'e	Proportion	2017	41.4	55.7	27.2
Eritrea	Ghida'e	Count	2000	0	1	0
Eritrea	Ghida'e	Count	2017	0	1	0
Eritrea	So. Southern Red-Sea	Proportion	2000	44.2	60.0	30.0
Eritrea	So. Southern Red-Sea	Proportion	2017	37.0	54.3	22.5
Eritrea	So. Southern Red-Sea	Count	2000	0	1	0
Eritrea	So. Southern Red-Sea	Count	2017	0	1	0

Table 4: ORS by Unit *(continued)*

Country	Unit	Metric	Year	Mean	Upper	Lower
Eritrea	Forto	Proportion	2000	38.1	49.4	27.4
Eritrea	Forto	Proportion	2017	44.6	59.2	29.9
Eritrea	Forto	Count	2000	0	0	0
Eritrea	Forto	Count	2017	0	1	0
Eritrea	Dekemehare	Proportion	2000	42.8	56.5	31.8
Eritrea	Dekemehare	Proportion	2017	42.3	56.7	27.4
Eritrea	Dekemehare	Count	2000	0	1	0
Eritrea	Dekemehare	Count	2017	0	1	0
Eritrea	Gheleb	Proportion	2000	46.6	60.6	34.7
Eritrea	Gheleb	Proportion	2017	45.7	60.1	31.4
Eritrea	Gheleb	Count	2000	0	1	0
Eritrea	Gheleb	Count	2017	0	1	0
Eritrea	Karora	Proportion	2000	44.6	58.0	32.5
Eritrea	Karora	Proportion	2017	42.9	58.6	28.1
Eritrea	Karora	Count	2000	0	1	0
Eritrea	Karora	Count	2017	0	1	0
Eritrea	Gogne	Proportion	2000	37.4	49.2	26.7
Eritrea	Gogne	Proportion	2017	42.4	57.6	27.9
Eritrea	Gogne	Count	2000	0	0	0
Eritrea	Gogne	Count	2017	0	1	0
Eritrea	Dibarwa	Proportion	2000	42.9	56.0	31.9
Eritrea	Dibarwa	Proportion	2017	42.9	58.0	28.7
Eritrea	Dibarwa	Count	2000	0	1	0
Eritrea	Dibarwa	Count	2017	0	1	0
Eritrea	Habero	Proportion	2000	43.5	56.5	30.2
Eritrea	Habero	Proportion	2017	45.0	61.6	30.1
Eritrea	Habero	Count	2000	0	1	0
Eritrea	Habero	Count	2017	0	1	0
Eritrea	Mitswa‘e City	Proportion	2000	40.4	54.3	28.3
Eritrea	Mitswa‘e City	Proportion	2017	38.1	52.4	24.0
Eritrea	Mitswa‘e City	Count	2000	0	1	0
Eritrea	Mitswa‘e City	Count	2017	0	1	0
Eritrea	Haykota	Proportion	2000	36.7	48.9	25.4
Eritrea	Haykota	Proportion	2017	43.4	59.0	29.0
Eritrea	Haykota	Count	2000	0	0	0
Eritrea	Haykota	Count	2017	0	1	0
Eritrea	Kudo Bu‘er	Proportion	2000	40.5	54.7	29.9
Eritrea	Kudo Bu‘er	Proportion	2017	42.2	56.5	28.9
Eritrea	Kudo Bu‘er	Count	2000	0	1	0
Eritrea	Kudo Bu‘er	Count	2017	0	1	0
Eritrea	Hagaz	Proportion	2000	42.6	55.1	30.9
Eritrea	Hagaz	Proportion	2017	44.2	58.3	28.9
Eritrea	Hagaz	Count	2000	0	1	0
Eritrea	Hagaz	Count	2017	0	1	0
Eritrea	Nakfa	Proportion	2000	45.4	59.0	32.3
Eritrea	Nakfa	Proportion	2017	45.8	63.0	29.4
Eritrea	Nakfa	Count	2000	0	1	0
Eritrea	Nakfa	Count	2017	0	1	0
Eritrea	La‘Elay Gash	Proportion	2000	37.9	50.3	26.6

Table 4: ORS by Unit *(continued)*

Country	Unit	Metric	Year	Mean	Upper	Lower
Eritrea	La‘Elay Gash	Proportion	2017	41.2	55.8	26.4
Eritrea	La‘Elay Gash	Count	2000	0	1	0
Eritrea	La‘Elay Gash	Count	2017	0	1	0
Eritrea	May Mine	Proportion	2000	39.3	52.5	27.1
Eritrea	May Mine	Proportion	2017	41.9	56.9	28.0
Eritrea	May Mine	Count	2000	0	1	0
Eritrea	May Mine	Count	2017	0	1	0
Eritrea	Halhal	Proportion	2000	44.3	56.6	32.2
Eritrea	Halhal	Proportion	2017	45.6	60.6	30.9
Eritrea	Halhal	Count	2000	0	1	0
Eritrea	Halhal	Count	2017	0	1	0
Eritrea	Sheib	Proportion	2000	42.1	56.4	30.2
Eritrea	Sheib	Proportion	2017	40.6	55.6	26.5
Eritrea	Sheib	Count	2000	0	1	0
Eritrea	Sheib	Count	2017	0	1	0
Eritrea	Logo Anseba	Proportion	2000	44.8	58.0	33.6
Eritrea	Logo Anseba	Proportion	2017	44.4	59.3	29.4
Eritrea	Logo Anseba	Count	2000	0	1	0
Eritrea	Logo Anseba	Count	2017	0	1	0
Eritrea	Mendefera	Proportion	2000	41.6	55.2	31.0
Eritrea	Mendefera	Proportion	2017	42.2	56.6	28.8
Eritrea	Mendefera	Count	2000	0	1	0
Eritrea	Mendefera	Count	2017	0	1	0
Eritrea	Keren	Proportion	2000	43.7	57.5	31.0
Eritrea	Keren	Proportion	2017	43.7	57.8	28.3
Eritrea	Keren	Count	2000	0	1	0
Eritrea	Keren	Count	2017	0	1	0
Eritrea	Mansura	Proportion	2000	40.8	52.9	29.8
Eritrea	Mansura	Proportion	2017	42.6	56.7	27.9
Eritrea	Mansura	Count	2000	0	1	0
Eritrea	Mansura	Count	2017	0	1	0
Eritrea	Segeneyiti	Proportion	2000	43.0	56.7	32.1
Eritrea	Segeneyiti	Proportion	2017	42.9	57.4	27.6
Eritrea	Segeneyiti	Count	2000	0	1	0
Eritrea	Segeneyiti	Count	2017	0	1	0
Eritrea	Kerke Bet	Proportion	2000	40.5	52.7	29.2
Eritrea	Kerke Bet	Proportion	2017	43.3	59.4	29.3
Eritrea	Kerke Bet	Count	2000	0	1	0
Eritrea	Kerke Bet	Count	2017	0	1	0
Eritrea	Mogolo	Proportion	2000	38.3	50.5	27.6
Eritrea	Mogolo	Proportion	2017	42.3	56.9	28.2
Eritrea	Mogolo	Count	2000	0	1	0
Eritrea	Mogolo	Count	2017	0	1	0
Eritrea	Senafe	Proportion	2000	42.6	56.3	30.9
Eritrea	Senafe	Proportion	2017	42.7	57.4	27.6
Eritrea	Senafe	Count	2000	0	1	0
Eritrea	Senafe	Count	2017	0	1	0
Eritrea	Sel‘a	Proportion	2000	41.9	55.6	30.4
Eritrea	Sel‘a	Proportion	2017	43.3	59.9	29.0

Table 4: ORS by Unit *(continued)*

Country	Unit	Metric	Year	Mean	Upper	Lower
Eritrea	Sel'a	Count	2000	0	1	0
Eritrea	Sel'a	Count	2017	0	1	0
Eritrea	Omhajer	Proportion	2000	34.3	46.4	24.7
Eritrea	Omhajer	Proportion	2017	40.6	55.8	25.4
Eritrea	Omhajer	Count	2000	0	0	0
Eritrea	Omhajer	Count	2017	0	1	0
Eritrea	Tsorena	Proportion	2000	41.6	55.9	30.0
Eritrea	Tsorena	Proportion	2017	41.0	55.3	26.1
Eritrea	Tsorena	Count	2000	0	1	0
Eritrea	Tsorena	Count	2017	0	1	0
Eritrea	Shemboko	Proportion	2000	39.8	51.7	29.6
Eritrea	Shemboko	Proportion	2017	42.2	56.0	28.3
Eritrea	Shemboko	Count	2000	0	1	0
Eritrea	Shemboko	Count	2017	0	1	0
Eritrea	Teseneye	Proportion	2000	35.7	47.1	25.7
Eritrea	Teseneye	Proportion	2017	43.3	58.7	28.3
Eritrea	Teseneye	Count	2000	0	0	0
Eritrea	Teseneye	Count	2017	0	1	0
Ethiopia	Addis Abeba	Proportion	2000	26.4	33.1	19.9
Ethiopia	Addis Abeba	Proportion	2017	49.9	60.3	41.4
Ethiopia	Addis Abeba	Count	2000	0	0	0
Ethiopia	Addis Abeba	Count	2017	0	1	0
Ethiopia	Afar Zone 1	Proportion	2000	16.4	20.3	13.1
Ethiopia	Afar Zone 1	Proportion	2017	34.5	40.4	28.3
Ethiopia	Afar Zone 1	Count	2000	0	0	0
Ethiopia	Afar Zone 1	Count	2017	0	0	0
Ethiopia	Agew Awi	Proportion	2000	10.6	13.4	8.1
Ethiopia	Agew Awi	Proportion	2017	31.0	37.8	24.4
Ethiopia	Agew Awi	Count	2000	0	0	0
Ethiopia	Agew Awi	Count	2017	0	0	0
Ethiopia	Asosa	Proportion	2000	14.8	18.7	11.4
Ethiopia	Asosa	Proportion	2017	38.1	45.9	31.4
Ethiopia	Asosa	Count	2000	0	0	0
Ethiopia	Asosa	Count	2017	0	0	0
Ethiopia	Dire Dawa	Proportion	2000	25.7	33.8	19.3
Ethiopia	Dire Dawa	Proportion	2017	45.2	55.0	35.3
Ethiopia	Dire Dawa	Count	2000	0	0	0
Ethiopia	Dire Dawa	Count	2017	0	1	0
Ethiopia	Agnuak	Proportion	2000	24.8	31.3	19.5
Ethiopia	Agnuak	Proportion	2017	48.9	57.7	40.9
Ethiopia	Agnuak	Count	2000	0	0	0
Ethiopia	Agnuak	Count	2017	0	1	0
Ethiopia	Hareri	Proportion	2000	23.1	28.5	17.7
Ethiopia	Hareri	Proportion	2017	41.5	52.8	32.4
Ethiopia	Hareri	Count	2000	0	0	0
Ethiopia	Hareri	Count	2017	0	1	0
Ethiopia	Arsi	Proportion	2000	11.7	15.4	8.9
Ethiopia	Arsi	Proportion	2017	30.8	37.6	25.2
Ethiopia	Arsi	Count	2000	0	0	0

Table 4: ORS by Unit *(continued)*

Country	Unit	Metric	Year	Mean	Upper	Lower
Ethiopia	Arsi	Count	2017	0	0	0
Ethiopia	Afder	Proportion	2000	16.1	21.4	12.5
Ethiopia	Afder	Proportion	2017	32.6	39.4	26.6
Ethiopia	Afder	Count	2000	0	0	0
Ethiopia	Afder	Count	2017	0	0	0
Ethiopia	Alaba	Proportion	2000	13.8	18.0	10.1
Ethiopia	Alaba	Proportion	2017	38.4	48.0	30.1
Ethiopia	Alaba	Count	2000	0	0	0
Ethiopia	Alaba	Count	2017	0	0	0
Ethiopia	Debubawi	Proportion	2000	14.2	18.1	11.1
Ethiopia	Debubawi	Proportion	2017	37.6	44.2	30.9
Ethiopia	Debubawi	Count	2000	0	0	0
Ethiopia	Debubawi	Count	2017	0	0	0
Ethiopia	Afar Zone 2	Proportion	2000	14.7	18.5	11.6
Ethiopia	Afar Zone 2	Proportion	2017	34.2	40.8	27.9
Ethiopia	Afar Zone 2	Count	2000	0	0	0
Ethiopia	Afar Zone 2	Count	2017	0	0	0
Ethiopia	Argoba	Proportion	2000	12.7	16.7	8.7
Ethiopia	Argoba	Proportion	2017	34.2	43.4	25.6
Ethiopia	Argoba	Count	2000	0	0	0
Ethiopia	Argoba	Count	2017	0	0	0
Ethiopia	Kemashi	Proportion	2000	11.8	14.5	9.2
Ethiopia	Kemashi	Proportion	2017	33.7	40.1	26.4
Ethiopia	Kemashi	Count	2000	0	0	0
Ethiopia	Kemashi	Count	2017	0	0	0
Ethiopia	Majang	Proportion	2000	15.6	20.6	11.8
Ethiopia	Majang	Proportion	2017	35.3	45.2	27.5
Ethiopia	Majang	Count	2000	0	0	0
Ethiopia	Majang	Count	2017	0	0	0
Ethiopia	Bale	Proportion	2000	13.0	16.9	10.1
Ethiopia	Bale	Proportion	2017	31.5	38.3	25.5
Ethiopia	Bale	Count	2000	0	0	0
Ethiopia	Bale	Count	2017	0	0	0
Ethiopia	Doolo	Proportion	2000	22.6	28.9	17.4
Ethiopia	Doolo	Proportion	2017	40.7	47.9	33.6
Ethiopia	Doolo	Count	2000	0	0	0
Ethiopia	Doolo	Count	2017	0	0	0
Ethiopia	Alle	Proportion	2000	13.0	16.6	9.5
Ethiopia	Alle	Proportion	2017	33.0	40.6	26.0
Ethiopia	Alle	Count	2000	0	0	0
Ethiopia	Alle	Count	2017	0	0	0
Ethiopia	Mehakelegnaw	Proportion	2000	13.1	16.9	10.3
Ethiopia	Mehakelegnaw	Proportion	2017	37.0	43.4	29.3
Ethiopia	Mehakelegnaw	Count	2000	0	0	0
Ethiopia	Mehakelegnaw	Count	2017	0	0	0
Ethiopia	Afar Zone 3	Proportion	2000	14.8	18.5	11.2
Ethiopia	Afar Zone 3	Proportion	2017	36.2	43.7	30.2
Ethiopia	Afar Zone 3	Count	2000	0	0	0
Ethiopia	Afar Zone 3	Count	2017	0	0	0

Table 4: ORS by Unit *(continued)*

Country	Unit	Metric	Year	Mean	Upper	Lower
Ethiopia	Bahir Dar Special Zone	Proportion	2000	16.7	26.3	9.1
Ethiopia	Bahir Dar Special Zone	Proportion	2017	42.5	56.8	26.0
Ethiopia	Bahir Dar Special Zone	Count	2000	0	0	0
Ethiopia	Bahir Dar Special Zone	Count	2017	0	1	0
Ethiopia	Metekel	Proportion	2000	11.6	14.9	9.1
Ethiopia	Metekel	Proportion	2017	33.1	39.3	27.3
Ethiopia	Metekel	Count	2000	0	0	0
Ethiopia	Metekel	Count	2017	0	0	0
Ethiopia	Nuer	Proportion	2000	19.0	23.8	14.5
Ethiopia	Nuer	Proportion	2017	41.6	50.1	32.8
Ethiopia	Nuer	Count	2000	0	0	0
Ethiopia	Nuer	Count	2017	0	1	0
Ethiopia	Borena	Proportion	2000	12.0	14.7	9.5
Ethiopia	Borena	Proportion	2017	31.6	38.1	25.6
Ethiopia	Borena	Count	2000	0	0	0
Ethiopia	Borena	Count	2017	0	0	0
Ethiopia	Fafan	Proportion	2000	19.4	23.5	15.7
Ethiopia	Fafan	Proportion	2017	36.4	43.9	29.4
Ethiopia	Fafan	Count	2000	0	0	0
Ethiopia	Fafan	Count	2017	0	0	0
Ethiopia	Amaro	Proportion	2000	10.9	14.0	8.2
Ethiopia	Amaro	Proportion	2017	31.1	38.9	24.3
Ethiopia	Amaro	Count	2000	0	0	0
Ethiopia	Amaro	Count	2017	0	0	0
Ethiopia	Mi'irabawi	Proportion	2000	14.5	18.1	11.0
Ethiopia	Mi'irabawi	Proportion	2017	38.6	45.4	31.7
Ethiopia	Mi'irabawi	Count	2000	0	0	0
Ethiopia	Mi'irabawi	Count	2017	0	0	0
Ethiopia	Afar Zone 4	Proportion	2000	12.9	16.6	9.8
Ethiopia	Afar Zone 4	Proportion	2017	31.6	38.2	25.6
Ethiopia	Afar Zone 4	Count	2000	0	0	0
Ethiopia	Afar Zone 4	Count	2017	0	0	0
Ethiopia	Debub Gondar	Proportion	2000	9.3	12.4	7.0
Ethiopia	Debub Gondar	Proportion	2017	29.3	36.5	23.5
Ethiopia	Debub Gondar	Count	2000	0	0	0
Ethiopia	Debub Gondar	Count	2017	0	0	0
Ethiopia	Debub Mirab Shewa	Proportion	2000	11.5	14.9	8.4
Ethiopia	Debub Mirab Shewa	Proportion	2017	32.1	39.9	26.3
Ethiopia	Debub Mirab Shewa	Count	2000	0	0	0
Ethiopia	Debub Mirab Shewa	Count	2017	0	0	0
Ethiopia	Jarar	Proportion	2000	15.8	20.8	11.9
Ethiopia	Jarar	Proportion	2017	30.4	37.2	24.2
Ethiopia	Jarar	Count	2000	0	0	0
Ethiopia	Jarar	Count	2017	0	0	0
Ethiopia	Basketo	Proportion	2000	8.8	12.0	6.3
Ethiopia	Basketo	Proportion	2017	25.3	33.6	17.9
Ethiopia	Basketo	Count	2000	0	0	0
Ethiopia	Basketo	Count	2017	0	0	0
Ethiopia	Misraqawi	Proportion	2000	14.1	17.9	11.1

Table 4: ORS by Unit *(continued)*

Country	Unit	Metric	Year	Mean	Upper	Lower
Ethiopia	Misraqawi	Proportion	2017	36.0	42.5	28.8
Ethiopia	Misraqawi	Count	2000	0	0	0
Ethiopia	Misraqawi	Count	2017	0	0	0
Ethiopia	Afar Zone 5	Proportion	2000	14.5	18.4	11.0
Ethiopia	Afar Zone 5	Proportion	2017	35.3	42.7	29.1
Ethiopia	Afar Zone 5	Count	2000	0	0	0
Ethiopia	Afar Zone 5	Count	2017	0	0	0
Ethiopia	Debub Wollo	Proportion	2000	11.5	14.0	9.1
Ethiopia	Debub Wollo	Proportion	2017	31.6	38.5	25.0
Ethiopia	Debub Wollo	Count	2000	0	0	0
Ethiopia	Debub Wollo	Count	2017	0	0	0
Ethiopia	Guji	Proportion	2000	11.6	14.9	9.0
Ethiopia	Guji	Proportion	2017	29.2	35.9	23.6
Ethiopia	Guji	Count	2000	0	0	0
Ethiopia	Guji	Count	2017	0	0	0
Ethiopia	Korahe	Proportion	2000	17.7	24.0	13.7
Ethiopia	Korahe	Proportion	2017	34.6	42.0	27.8
Ethiopia	Korahe	Count	2000	0	0	0
Ethiopia	Korahe	Count	2017	0	0	0
Ethiopia	Bench Maji	Proportion	2000	13.5	17.2	10.2
Ethiopia	Bench Maji	Proportion	2017	31.1	37.8	24.4
Ethiopia	Bench Maji	Count	2000	0	0	0
Ethiopia	Bench Maji	Count	2017	0	0	0
Ethiopia	Semien Mi'irabaw	Proportion	2000	13.1	16.6	10.2
Ethiopia	Semien Mi'irabaw	Proportion	2017	37.6	44.9	30.5
Ethiopia	Semien Mi'irabaw	Count	2000	0	0	0
Ethiopia	Semien Mi'irabaw	Count	2017	0	0	0
Ethiopia	Mirab Gojjam	Proportion	2000	10.3	13.1	7.8
Ethiopia	Mirab Gojjam	Proportion	2017	31.1	37.2	24.2
Ethiopia	Mirab Gojjam	Count	2000	0	0	0
Ethiopia	Mirab Gojjam	Count	2017	0	0	0
Ethiopia	Horo Guduru	Proportion	2000	9.5	12.0	6.9
Ethiopia	Horo Guduru	Proportion	2017	27.7	34.4	21.1
Ethiopia	Horo Guduru	Count	2000	0	0	0
Ethiopia	Horo Guduru	Count	2017	0	0	0
Ethiopia	Liben	Proportion	2000	12.8	16.3	9.9
Ethiopia	Liben	Proportion	2017	28.2	34.2	23.0
Ethiopia	Liben	Count	2000	0	0	0
Ethiopia	Liben	Count	2017	0	0	0
Ethiopia	Burji	Proportion	2000	12.7	15.7	9.2
Ethiopia	Burji	Proportion	2017	33.1	40.6	26.4
Ethiopia	Burji	Count	2000	0	0	0
Ethiopia	Burji	Count	2017	0	0	0
Ethiopia	Misraq Gojjam	Proportion	2000	9.4	12.3	7.0
Ethiopia	Misraq Gojjam	Proportion	2017	28.9	35.3	22.2
Ethiopia	Misraq Gojjam	Count	2000	0	0	0
Ethiopia	Misraq Gojjam	Count	2017	0	0	0
Ethiopia	Ilubabor	Proportion	2000	11.5	14.9	8.7
Ethiopia	Ilubabor	Proportion	2017	30.1	37.4	23.9

Table 4: ORS by Unit *(continued)*

Country	Unit	Metric	Year	Mean	Upper	Lower
Ethiopia	Ilubabor	Count	2000	0	0	0
Ethiopia	Ilubabor	Count	2017	0	0	0
Ethiopia	Nogob	Proportion	2000	16.8	21.3	13.0
Ethiopia	Nogob	Proportion	2017	35.5	42.3	28.6
Ethiopia	Nogob	Count	2000	0	0	0
Ethiopia	Nogob	Count	2017	0	0	0
Ethiopia	Dawro	Proportion	2000	10.4	13.1	7.9
Ethiopia	Dawro	Proportion	2017	31.3	37.9	24.4
Ethiopia	Dawro	Count	2000	0	0	0
Ethiopia	Dawro	Count	2017	0	0	0
Ethiopia	North Shewa	Proportion	2000	11.7	14.8	9.2
Ethiopia	North Shewa	Proportion	2017	32.1	39.3	25.7
Ethiopia	North Shewa	Count	2000	0	0	0
Ethiopia	North Shewa	Count	2017	0	0	0
Ethiopia	Jimma	Proportion	2000	10.5	13.4	8.3
Ethiopia	Jimma	Proportion	2017	28.7	35.5	22.6
Ethiopia	Jimma	Count	2000	0	0	0
Ethiopia	Jimma	Count	2017	0	0	0
Ethiopia	Shabelle	Proportion	2000	16.6	21.9	12.5
Ethiopia	Shabelle	Proportion	2017	33.2	40.3	26.9
Ethiopia	Shabelle	Count	2000	0	0	0
Ethiopia	Shabelle	Count	2017	0	0	0
Ethiopia	Debub Omo	Proportion	2000	13.8	17.7	10.6
Ethiopia	Debub Omo	Proportion	2017	33.3	40.0	27.1
Ethiopia	Debub Omo	Count	2000	0	0	0
Ethiopia	Debub Omo	Count	2017	0	0	0
Ethiopia	Oromia	Proportion	2000	14.9	18.3	11.4
Ethiopia	Oromia	Proportion	2017	37.3	44.6	30.2
Ethiopia	Oromia	Count	2000	0	0	0
Ethiopia	Oromia	Count	2017	0	0	0
Ethiopia	Kelem Wellega	Proportion	2000	13.0	16.9	9.9
Ethiopia	Kelem Wellega	Proportion	2017	33.0	40.3	26.0
Ethiopia	Kelem Wellega	Count	2000	0	0	0
Ethiopia	Kelem Wellega	Count	2017	0	0	0
Ethiopia	Siti	Proportion	2000	15.4	18.8	12.4
Ethiopia	Siti	Proportion	2017	34.6	42.0	28.7
Ethiopia	Siti	Count	2000	0	0	0
Ethiopia	Siti	Count	2017	0	0	0
Ethiopia	Derashe	Proportion	2000	12.4	16.0	9.1
Ethiopia	Derashe	Proportion	2017	32.3	40.9	25.4
Ethiopia	Derashe	Count	2000	0	0	0
Ethiopia	Derashe	Count	2017	0	0	0
Ethiopia	Semen Gondar	Proportion	2000	11.4	14.3	9.0
Ethiopia	Semen Gondar	Proportion	2017	33.5	39.8	27.6
Ethiopia	Semen Gondar	Count	2000	0	0	0
Ethiopia	Semen Gondar	Count	2017	0	0	0
Ethiopia	Mirab Arsi	Proportion	2000	11.9	15.1	9.2
Ethiopia	Mirab Arsi	Proportion	2017	31.4	38.2	25.7
Ethiopia	Mirab Arsi	Count	2000	0	0	0

Table 4: ORS by Unit *(continued)*

Country	Unit	Metric	Year	Mean	Upper	Lower
Ethiopia	Mirab Arsi	Count	2017	0	0	0
Ethiopia	Gamo Gofa	Proportion	2000	11.3	14.2	8.7
Ethiopia	Gamo Gofa	Proportion	2017	31.0	37.6	24.3
Ethiopia	Gamo Gofa	Count	2000	0	0	0
Ethiopia	Gamo Gofa	Count	2017	0	0	0
Ethiopia	Semen Wello	Proportion	2000	11.1	14.1	8.8
Ethiopia	Semen Wello	Proportion	2017	31.3	37.9	25.0
Ethiopia	Semen Wello	Count	2000	0	0	0
Ethiopia	Semen Wello	Count	2017	0	0	0
Ethiopia	Mirab Hararghe	Proportion	2000	11.8	14.7	9.0
Ethiopia	Mirab Hararghe	Proportion	2017	31.7	39.8	26.1
Ethiopia	Mirab Hararghe	Count	2000	0	0	0
Ethiopia	Mirab Hararghe	Count	2017	0	0	0
Ethiopia	Gedeo	Proportion	2000	12.4	16.0	9.6
Ethiopia	Gedeo	Proportion	2017	31.8	38.9	25.4
Ethiopia	Gedeo	Count	2000	0	0	0
Ethiopia	Gedeo	Count	2017	0	0	0
Ethiopia	Wag Himra	Proportion	2000	10.0	12.9	7.6
Ethiopia	Wag Himra	Proportion	2017	31.3	38.5	25.4
Ethiopia	Wag Himra	Count	2000	0	0	0
Ethiopia	Wag Himra	Count	2017	0	0	0
Ethiopia	Mirab Shewa	Proportion	2000	10.6	13.5	8.1
Ethiopia	Mirab Shewa	Proportion	2017	30.2	37.2	23.5
Ethiopia	Mirab Shewa	Count	2000	0	0	0
Ethiopia	Mirab Shewa	Count	2017	0	0	0
Ethiopia	Gurage	Proportion	2000	11.2	14.0	8.6
Ethiopia	Gurage	Proportion	2017	33.5	40.9	26.9
Ethiopia	Gurage	Count	2000	0	0	0
Ethiopia	Gurage	Count	2017	0	0	0
Ethiopia	Mirab Welega	Proportion	2000	12.3	15.4	9.4
Ethiopia	Mirab Welega	Proportion	2017	31.9	38.5	25.3
Ethiopia	Mirab Welega	Count	2000	0	0	0
Ethiopia	Mirab Welega	Count	2017	0	0	0
Ethiopia	Hadiya	Proportion	2000	11.6	15.0	9.0
Ethiopia	Hadiya	Proportion	2017	34.1	41.6	26.8
Ethiopia	Hadiya	Count	2000	0	0	0
Ethiopia	Hadiya	Count	2017	0	0	0
Ethiopia	Misraq Harerge	Proportion	2000	13.7	16.8	10.8
Ethiopia	Misraq Harerge	Proportion	2017	32.1	39.7	26.1
Ethiopia	Misraq Harerge	Count	2000	0	0	0
Ethiopia	Misraq Harerge	Count	2017	0	0	0
Ethiopia	Keffa	Proportion	2000	11.6	14.8	8.9
Ethiopia	Keffa	Proportion	2017	29.2	36.1	22.9
Ethiopia	Keffa	Count	2000	0	0	0
Ethiopia	Keffa	Count	2017	0	0	0
Ethiopia	Misraq Shewa	Proportion	2000	15.6	19.5	12.1
Ethiopia	Misraq Shewa	Proportion	2017	38.6	46.6	32.1
Ethiopia	Misraq Shewa	Count	2000	0	0	0
Ethiopia	Misraq Shewa	Count	2017	0	0	0

Table 4: ORS by Unit *(continued)*

Country	Unit	Metric	Year	Mean	Upper	Lower
Ethiopia	Kembata Tembaro	Proportion	2000	11.6	15.0	8.7
Ethiopia	Kembata Tembaro	Proportion	2017	34.7	42.3	27.3
Ethiopia	Kembata Tembaro	Count	2000	0	0	0
Ethiopia	Kembata Tembaro	Count	2017	0	0	0
Ethiopia	Misraq Wellega	Proportion	2000	10.6	13.5	8.0
Ethiopia	Misraq Wellega	Proportion	2017	29.6	35.9	23.7
Ethiopia	Misraq Wellega	Count	2000	0	0	0
Ethiopia	Misraq Wellega	Count	2017	0	0	0
Ethiopia	Konso	Proportion	2000	14.6	19.3	11.2
Ethiopia	Konso	Proportion	2017	36.0	44.2	28.3
Ethiopia	Konso	Count	2000	0	0	0
Ethiopia	Konso	Count	2017	0	0	0
Ethiopia	North Shewa	Proportion	2000	11.0	14.0	8.2
Ethiopia	North Shewa	Proportion	2017	31.0	38.3	25.0
Ethiopia	North Shewa	Count	2000	0	0	0
Ethiopia	North Shewa	Count	2017	0	0	0
Ethiopia	Konta	Proportion	2000	11.4	14.6	8.2
Ethiopia	Konta	Proportion	2017	31.3	38.0	24.6
Ethiopia	Konta	Count	2000	0	0	0
Ethiopia	Konta	Count	2017	0	0	0
Ethiopia	Sheka	Proportion	2000	15.0	19.2	11.2
Ethiopia	Sheka	Proportion	2017	34.9	43.7	27.2
Ethiopia	Sheka	Count	2000	0	0	0
Ethiopia	Sheka	Count	2017	0	0	0
Ethiopia	Sidama	Proportion	2000	12.1	15.3	9.4
Ethiopia	Sidama	Proportion	2017	33.0	40.0	27.0
Ethiopia	Sidama	Count	2000	0	0	0
Ethiopia	Sidama	Count	2017	0	0	0
Ethiopia	Silti	Proportion	2000	12.1	15.3	9.5
Ethiopia	Silti	Proportion	2017	35.5	42.9	28.0
Ethiopia	Silti	Count	2000	0	0	0
Ethiopia	Silti	Count	2017	0	0	0
Ethiopia	Wolayita	Proportion	2000	12.3	15.4	9.2
Ethiopia	Wolayita	Proportion	2017	35.9	43.0	29.2
Ethiopia	Wolayita	Count	2000	0	0	0
Ethiopia	Wolayita	Count	2017	0	0	0
Ethiopia	Yem	Proportion	2000	10.3	13.4	7.5
Ethiopia	Yem	Proportion	2017	30.6	38.2	23.9
Ethiopia	Yem	Count	2000	0	0	0
Ethiopia	Yem	Count	2017	0	0	0
Gabon	Komo	Proportion	2000	24.5	32.1	18.3
Gabon	Komo	Proportion	2017	30.9	52.0	12.9
Gabon	Komo	Count	2000	0	0	0
Gabon	Komo	Count	2017	0	1	0
Gabon	Léboumbi-Leyou	Proportion	2000	30.7	39.1	23.8
Gabon	Léboumbi-Leyou	Proportion	2017	30.3	53.7	13.4
Gabon	Léboumbi-Leyou	Count	2000	0	0	0
Gabon	Léboumbi-Leyou	Count	2017	0	1	0
Gabon	Abanga-Bigné	Proportion	2000	31.0	39.6	23.3

Table 4: ORS by Unit *(continued)*

Country	Unit	Metric	Year	Mean	Upper	Lower
Gabon	Abanga-Bigné	Proportion	2017	30.5	53.1	13.6
Gabon	Abanga-Bigné	Count	2000	0	0	0
Gabon	Abanga-Bigné	Count	2017	0	1	0
Gabon	Boumi-lowetsi	Proportion	2000	38.0	48.1	26.2
Gabon	Boumi-lowetsi	Proportion	2017	31.5	53.7	15.9
Gabon	Boumi-lowetsi	Count	2000	0	0	0
Gabon	Boumi-lowetsi	Count	2017	0	1	0
Gabon	Basse Banio	Proportion	2000	27.2	40.0	17.0
Gabon	Basse Banio	Proportion	2017	27.1	48.8	12.4
Gabon	Basse Banio	Count	2000	0	0	0
Gabon	Basse Banio	Count	2017	0	0	0
Gabon	Ivindo	Proportion	2000	22.8	32.7	15.1
Gabon	Ivindo	Proportion	2017	29.4	51.5	12.6
Gabon	Ivindo	Count	2000	0	0	0
Gabon	Ivindo	Count	2017	0	1	0
Gabon	Lolo Bouenguidi	Proportion	2000	35.3	45.7	26.1
Gabon	Lolo Bouenguidi	Proportion	2017	31.0	51.3	15.0
Gabon	Lolo Bouenguidi	Count	2000	0	0	0
Gabon	Lolo Bouenguidi	Count	2017	0	1	0
Gabon	Bendjé	Proportion	2000	28.9	38.7	20.4
Gabon	Bendjé	Proportion	2017	29.3	50.7	12.5
Gabon	Bendjé	Count	2000	0	0	0
Gabon	Bendjé	Count	2017	0	1	0
Gabon	Haut-Como	Proportion	2000	26.9	36.3	18.5
Gabon	Haut-Como	Proportion	2017	34.5	59.0	16.1
Gabon	Haut-Como	Count	2000	0	0	0
Gabon	Haut-Como	Count	2017	0	1	0
Gabon	Komo-Mondah	Proportion	2000	20.8	26.9	16.4
Gabon	Komo-Mondah	Proportion	2017	36.3	59.2	15.5
Gabon	Komo-Mondah	Count	2000	0	0	0
Gabon	Komo-Mondah	Count	2017	0	1	0
Gabon	Léconi-Djoué	Proportion	2000	26.9	35.4	18.8
Gabon	Léconi-Djoué	Proportion	2017	30.1	54.1	12.8
Gabon	Léconi-Djoué	Count	2000	0	0	0
Gabon	Léconi-Djoué	Count	2017	0	1	0
Gabon	Ogooué et des Lacs	Proportion	2000	31.2	40.9	22.8
Gabon	Ogooué et des Lacs	Proportion	2017	29.4	49.0	13.0
Gabon	Ogooué et des Lacs	Count	2000	0	0	0
Gabon	Ogooué et des Lacs	Count	2017	0	0	0
Gabon	Dola	Proportion	2000	35.5	45.3	24.9
Gabon	Dola	Proportion	2017	30.0	50.6	14.9
Gabon	Dola	Count	2000	0	0	0
Gabon	Dola	Count	2017	0	1	0
Gabon	Douigny	Proportion	2000	32.5	43.7	22.8
Gabon	Douigny	Proportion	2017	27.6	51.1	13.3
Gabon	Douigny	Count	2000	0	0	0
Gabon	Douigny	Count	2017	0	1	0
Gabon	Lopé	Proportion	2000	27.4	37.0	19.9
Gabon	Lopé	Proportion	2017	29.2	49.6	12.7

Table 4: ORS by Unit *(continued)*

Country	Unit	Metric	Year	Mean	Upper	Lower
Gabon	Lopé	Count	2000	0	0	0
Gabon	Lopé	Count	2017	0	0	0
Gabon	Lombo-Bouenguidi	Proportion	2000	34.9	45.0	26.1
Gabon	Lombo-Bouenguidi	Proportion	2017	31.5	53.4	14.3
Gabon	Lombo-Bouenguidi	Count	2000	0	0	0
Gabon	Lombo-Bouenguidi	Count	2017	0	1	0
Gabon	Étimboué	Proportion	2000	32.3	42.6	23.1
Gabon	Étimboué	Proportion	2017	28.0	51.7	12.8
Gabon	Étimboué	Count	2000	0	0	0
Gabon	Étimboué	Count	2017	0	1	0
Gabon	Haut-Ntem	Proportion	2000	24.6	35.3	15.5
Gabon	Haut-Ntem	Proportion	2017	31.4	56.4	13.2
Gabon	Haut-Ntem	Count	2000	0	0	0
Gabon	Haut-Ntem	Count	2017	0	1	0
Gabon	Noya	Proportion	2000	23.2	29.8	18.1
Gabon	Noya	Proportion	2017	34.2	58.3	15.2
Gabon	Noya	Count	2000	0	0	0
Gabon	Noya	Count	2017	0	1	0
Gabon	Lékoko	Proportion	2000	31.3	40.8	23.6
Gabon	Lékoko	Proportion	2017	29.9	51.9	14.0
Gabon	Lékoko	Count	2000	0	0	0
Gabon	Lékoko	Count	2017	0	1	0
Gabon	Douya Onoye	Proportion	2000	38.3	48.2	30.0
Gabon	Douya Onoye	Proportion	2017	29.3	53.4	13.7
Gabon	Douya Onoye	Count	2000	0	0	0
Gabon	Douya Onoye	Count	2017	0	1	0
Gabon	Haute-Banio	Proportion	2000	24.7	39.5	12.4
Gabon	Haute-Banio	Proportion	2017	27.1	48.8	11.2
Gabon	Haute-Banio	Count	2000	0	0	0
Gabon	Haute-Banio	Count	2017	0	0	0
Gabon	Mvoung	Proportion	2000	23.9	32.9	16.6
Gabon	Mvoung	Proportion	2017	30.0	54.2	11.9
Gabon	Mvoung	Count	2000	0	0	0
Gabon	Mvoung	Count	2017	0	1	0
Gabon	Mouloudnou	Proportion	2000	28.2	36.2	21.1
Gabon	Mouloudnou	Proportion	2017	29.4	51.0	13.2
Gabon	Mouloudnou	Count	2000	0	0	0
Gabon	Mouloudnou	Count	2017	0	1	0
Gabon	Ndougou	Proportion	2000	32.1	43.5	21.7
Gabon	Ndougou	Proportion	2017	26.9	48.5	12.0
Gabon	Ndougou	Count	2000	0	0	0
Gabon	Ndougou	Count	2017	0	0	0
Gabon	Ntem	Proportion	2000	25.5	34.1	17.4
Gabon	Ntem	Proportion	2017	33.3	59.5	14.1
Gabon	Ntem	Count	2000	0	0	0
Gabon	Ntem	Count	2017	0	1	0
Gabon	Mpassa	Proportion	2000	28.8	35.7	21.7
Gabon	Mpassa	Proportion	2017	30.2	54.9	13.5
Gabon	Mpassa	Count	2000	0	0	0

Table 4: ORS by Unit *(continued)*

Country	Unit	Metric	Year	Mean	Upper	Lower
Gabon	Mpassa	Count	2017	0	1	0
Gabon	Louetsi-Wano	Proportion	2000	36.3	46.9	24.6
Gabon	Louetsi-Wano	Proportion	2017	30.0	51.7	14.3
Gabon	Louetsi-Wano	Count	2000	0	0	0
Gabon	Louetsi-Wano	Count	2017	0	1	0
Gabon	Mougoutsi	Proportion	2000	29.5	42.7	19.6
Gabon	Mougoutsi	Proportion	2017	28.5	48.3	14.0
Gabon	Mougoutsi	Count	2000	0	0	0
Gabon	Mougoutsi	Count	2017	0	0	0
Gabon	Zadié	Proportion	2000	23.4	37.0	14.6
Gabon	Zadié	Proportion	2017	28.4	49.5	11.4
Gabon	Zadié	Count	2000	0	0	0
Gabon	Zadié	Count	2017	0	0	0
Gabon	Okano	Proportion	2000	25.9	34.6	19.1
Gabon	Okano	Proportion	2017	32.2	55.3	12.8
Gabon	Okano	Count	2000	0	0	0
Gabon	Okano	Count	2017	0	1	0
Gabon	Plateaux	Proportion	2000	26.7	37.5	17.1
Gabon	Plateaux	Proportion	2017	29.6	51.5	13.4
Gabon	Plateaux	Count	2000	0	0	0
Gabon	Plateaux	Count	2017	0	1	0
Gabon	Ndolou	Proportion	2000	36.9	48.1	26.9
Gabon	Ndolou	Proportion	2017	28.8	52.8	13.2
Gabon	Ndolou	Count	2000	0	0	0
Gabon	Ndolou	Count	2017	0	1	0
Gabon	Woleu	Proportion	2000	23.9	33.0	17.3
Gabon	Woleu	Proportion	2017	32.7	57.7	13.2
Gabon	Woleu	Count	2000	0	0	0
Gabon	Woleu	Count	2017	0	1	0
Gabon	Sébé-Brikolo	Proportion	2000	25.9	36.6	17.7
Gabon	Sébé-Brikolo	Proportion	2017	30.0	51.0	13.4
Gabon	Sébé-Brikolo	Count	2000	0	0	0
Gabon	Sébé-Brikolo	Count	2017	0	1	0
Gabon	Ogoulou	Proportion	2000	38.8	48.8	29.8
Gabon	Ogoulou	Proportion	2017	30.7	52.6	14.7
Gabon	Ogoulou	Count	2000	0	0	0
Gabon	Ogoulou	Count	2017	0	1	0
Gabon	Tsamba Mangotsi	Proportion	2000	37.4	47.6	28.5
Gabon	Tsamba Mangotsi	Proportion	2017	28.7	49.4	13.4
Gabon	Tsamba Mangotsi	Count	2000	0	0	0
Gabon	Tsamba Mangotsi	Count	2017	0	0	0
Gambia	Banjul	Proportion	2000	NA	NA	NA
Gambia	Banjul	Proportion	2017	NA	NA	NA
Gambia	Banjul	Count	2000	NA	NA	NA
Gambia	Banjul	Count	2017	NA	NA	NA
Gambia	Jarra Central	Proportion	2000	41.4	57.1	27.2
Gambia	Jarra Central	Proportion	2017	40.3	55.8	26.4
Gambia	Jarra Central	Count	2000	0	1	0
Gambia	Jarra Central	Count	2017	0	1	0

Table 4: ORS by Unit *(continued)*

Country	Unit	Metric	Year	Mean	Upper	Lower
Gambia	Fulladu West	Proportion	2000	37.6	51.8	25.5
Gambia	Fulladu West	Proportion	2017	43.4	59.4	27.4
Gambia	Fulladu West	Count	2000	0	1	0
Gambia	Fulladu West	Count	2017	0	1	0
Gambia	Central Baddibu	Proportion	2000	44.8	57.6	30.4
Gambia	Central Baddibu	Proportion	2017	42.6	56.2	28.4
Gambia	Central Baddibu	Count	2000	0	1	0
Gambia	Central Baddibu	Count	2017	0	1	0
Gambia	Fulladu East	Proportion	2000	38.1	50.7	26.9
Gambia	Fulladu East	Proportion	2017	42.8	57.8	27.0
Gambia	Fulladu East	Count	2000	0	1	0
Gambia	Fulladu East	Count	2017	0	1	0
Gambia	Foni Bintang Karanai	Proportion	2000	49.0	62.9	33.3
Gambia	Foni Bintang Karanai	Proportion	2017	49.3	63.7	33.8
Gambia	Foni Bintang Karanai	Count	2000	0	1	0
Gambia	Foni Bintang Karanai	Count	2017	0	1	0
Gambia	Kanifing	Proportion	2000	45.8	59.9	31.0
Gambia	Kanifing	Proportion	2017	53.3	71.6	34.8
Gambia	Kanifing	Count	2000	0	1	0
Gambia	Kanifing	Count	2017	1	1	0
Gambia	Jarra East	Proportion	2000	39.8	54.4	26.1
Gambia	Jarra East	Proportion	2017	41.2	56.9	26.1
Gambia	Jarra East	Count	2000	0	1	0
Gambia	Jarra East	Count	2017	0	1	0
Gambia	Janjanbureh	Proportion	2000	37.5	53.3	22.4
Gambia	Janjanbureh	Proportion	2017	43.7	62.0	25.2
Gambia	Janjanbureh	Count	2000	0	1	0
Gambia	Janjanbureh	Count	2017	0	1	0
Gambia	Jokadu	Proportion	2000	44.8	59.7	30.7
Gambia	Jokadu	Proportion	2017	45.7	60.4	31.2
Gambia	Jokadu	Count	2000	0	1	0
Gambia	Jokadu	Count	2017	0	1	0
Gambia	Kantora	Proportion	2000	37.4	51.1	25.4
Gambia	Kantora	Proportion	2017	41.8	57.7	26.8
Gambia	Kantora	Count	2000	0	1	0
Gambia	Kantora	Count	2017	0	1	0
Gambia	Foni Bondali	Proportion	2000	49.7	63.7	34.3
Gambia	Foni Bondali	Proportion	2017	46.4	62.2	29.9
Gambia	Foni Bondali	Count	2000	0	1	0
Gambia	Foni Bondali	Count	2017	0	1	0
Gambia	Jarra West	Proportion	2000	43.9	58.1	30.7
Gambia	Jarra West	Proportion	2017	41.5	56.5	26.8
Gambia	Jarra West	Count	2000	0	1	0
Gambia	Jarra West	Count	2017	0	1	0
Gambia	Lower Saloum	Proportion	2000	37.5	51.5	26.0
Gambia	Lower Saloum	Proportion	2017	40.9	56.6	26.4
Gambia	Lower Saloum	Count	2000	0	1	0
Gambia	Lower Saloum	Count	2017	0	1	0
Gambia	Lower Baddibu	Proportion	2000	47.5	61.7	32.1

Table 4: ORS by Unit *(continued)*

Country	Unit	Metric	Year	Mean	Upper	Lower
Gambia	Lower Baddibu	Proportion	2017	45.5	60.8	30.2
Gambia	Lower Baddibu	Count	2000	0	1	0
Gambia	Lower Baddibu	Count	2017	0	1	0
Gambia	Sandu	Proportion	2000	37.2	50.6	25.4
Gambia	Sandu	Proportion	2017	43.9	61.1	26.5
Gambia	Sandu	Count	2000	0	1	0
Gambia	Sandu	Count	2017	0	1	0
Gambia	Foni Brefet	Proportion	2000	48.5	63.4	33.8
Gambia	Foni Brefet	Proportion	2017	51.4	67.3	36.3
Gambia	Foni Brefet	Count	2000	0	1	0
Gambia	Foni Brefet	Count	2017	1	1	0
Gambia	Kiang Central	Proportion	2000	46.1	60.2	31.6
Gambia	Kiang Central	Proportion	2017	43.0	57.4	28.1
Gambia	Kiang Central	Count	2000	0	1	0
Gambia	Kiang Central	Count	2017	0	1	0
Gambia	Niamina Dankunku	Proportion	2000	38.0	51.7	25.3
Gambia	Niamina Dankunku	Proportion	2017	41.6	58.6	27.2
Gambia	Niamina Dankunku	Count	2000	0	1	0
Gambia	Niamina Dankunku	Count	2017	0	1	0
Gambia	Lower Nuimi	Proportion	2000	43.5	57.4	28.8
Gambia	Lower Nuimi	Proportion	2017	49.8	65.0	34.8
Gambia	Lower Nuimi	Count	2000	0	1	0
Gambia	Lower Nuimi	Count	2017	0	1	0
Gambia	Wuli	Proportion	2000	36.5	49.5	26.0
Gambia	Wuli	Proportion	2017	42.8	60.8	27.1
Gambia	Wuli	Count	2000	0	0	0
Gambia	Wuli	Count	2017	0	1	0
Gambia	Foni Jarrol	Proportion	2000	47.4	61.9	32.4
Gambia	Foni Jarrol	Proportion	2017	43.7	58.7	28.5
Gambia	Foni Jarrol	Count	2000	0	1	0
Gambia	Foni Jarrol	Count	2017	0	1	0
Gambia	Kiang East	Proportion	2000	44.0	58.0	29.3
Gambia	Kiang East	Proportion	2017	40.9	54.6	26.1
Gambia	Kiang East	Count	2000	0	1	0
Gambia	Kiang East	Count	2017	0	1	0
Gambia	Niamina East	Proportion	2000	36.6	50.0	24.6
Gambia	Niamina East	Proportion	2017	42.5	57.9	27.2
Gambia	Niamina East	Count	2000	0	0	0
Gambia	Niamina East	Count	2017	0	1	0
Gambia	Upper Baddibu	Proportion	2000	41.2	53.3	28.6
Gambia	Upper Baddibu	Proportion	2017	40.7	56.0	27.0
Gambia	Upper Baddibu	Count	2000	0	1	0
Gambia	Upper Baddibu	Count	2017	0	1	0
Gambia	Foni Kansala	Proportion	2000	50.7	64.9	34.8
Gambia	Foni Kansala	Proportion	2017	48.5	64.0	31.6
Gambia	Foni Kansala	Count	2000	1	1	0
Gambia	Foni Kansala	Count	2017	0	1	0
Gambia	Kiang West	Proportion	2000	48.9	63.0	33.9
Gambia	Kiang West	Proportion	2017	46.9	61.9	31.4

Table 4: ORS by Unit *(continued)*

Country	Unit	Metric	Year	Mean	Upper	Lower
Gambia	Kiang West	Count	2000	0	1	0
Gambia	Kiang West	Count	2017	0	1	0
Gambia	Niamina West	Proportion	2000	39.0	53.2	26.7
Gambia	Niamina West	Proportion	2017	41.9	58.6	27.2
Gambia	Niamina West	Count	2000	0	1	0
Gambia	Niamina West	Count	2017	0	1	0
Gambia	Upper Nuimi	Proportion	2000	46.0	61.9	31.3
Gambia	Upper Nuimi	Proportion	2017	49.6	66.3	33.9
Gambia	Upper Nuimi	Count	2000	0	1	0
Gambia	Upper Nuimi	Count	2017	0	1	0
Gambia	Kombo Central	Proportion	2000	48.1	60.1	35.2
Gambia	Kombo Central	Proportion	2017	55.4	71.8	37.8
Gambia	Kombo Central	Count	2000	0	1	0
Gambia	Kombo Central	Count	2017	1	1	0
Gambia	Niani	Proportion	2000	36.1	49.8	25.0
Gambia	Niani	Proportion	2017	43.1	59.1	27.7
Gambia	Niani	Count	2000	0	0	0
Gambia	Niani	Count	2017	0	1	0
Gambia	Kombo East	Proportion	2000	47.6	60.0	34.0
Gambia	Kombo East	Proportion	2017	53.4	69.1	35.6
Gambia	Kombo East	Count	2000	0	1	0
Gambia	Kombo East	Count	2017	1	1	0
Gambia	Nianija	Proportion	2000	36.3	50.3	23.6
Gambia	Nianija	Proportion	2017	43.1	59.0	27.8
Gambia	Nianija	Count	2000	0	1	0
Gambia	Nianija	Count	2017	0	1	0
Gambia	Kombo Saint Mary	Proportion	2000	46.9	61.9	32.6
Gambia	Kombo Saint Mary	Proportion	2017	54.5	71.6	35.5
Gambia	Kombo Saint Mary	Count	2000	0	1	0
Gambia	Kombo Saint Mary	Count	2017	1	1	0
Gambia	Sami	Proportion	2000	36.8	49.1	24.3
Gambia	Sami	Proportion	2017	43.6	59.9	26.6
Gambia	Sami	Count	2000	0	0	0
Gambia	Sami	Count	2017	0	1	0
Gambia	Kombo South	Proportion	2000	46.3	59.0	33.0
Gambia	Kombo South	Proportion	2017	54.8	71.5	36.5
Gambia	Kombo South	Count	2000	0	1	0
Gambia	Kombo South	Count	2017	1	1	0
Gambia	Upper Saloum	Proportion	2000	35.7	49.3	23.7
Gambia	Upper Saloum	Proportion	2017	41.3	57.3	27.4
Gambia	Upper Saloum	Count	2000	0	0	0
Gambia	Upper Saloum	Count	2017	0	1	0
Ghana	Adansi North	Proportion	2000	35.3	58.2	17.8
Ghana	Adansi North	Proportion	2017	46.9	70.9	23.2
Ghana	Adansi North	Count	2000	0	1	0
Ghana	Adansi North	Count	2017	0	1	0
Ghana	Asunafo North	Proportion	2000	33.9	54.5	16.3
Ghana	Asunafo North	Proportion	2017	41.2	67.8	20.2
Ghana	Asunafo North	Count	2000	0	1	0

Table 4: ORS by Unit *(continued)*

Country	Unit	Metric	Year	Mean	Upper	Lower
Ghana	Asunafo North	Count	2017	0	1	0
Ghana	Abura-Asebu-Kwamankese	Proportion	2000	37.6	62.0	19.2
Ghana	Abura-Asebu-Kwamankese	Proportion	2017	49.9	73.6	22.8
Ghana	Abura-Asebu-Kwamankese	Count	2000	0	1	0
Ghana	Abura-Asebu-Kwamankese	Count	2017	0	1	0
Ghana	Afram Plains	Proportion	2000	28.2	50.0	13.2
Ghana	Afram Plains	Proportion	2017	39.2	61.2	20.0
Ghana	Afram Plains	Count	2000	0	0	0
Ghana	Afram Plains	Count	2017	0	1	0
Ghana	Accra	Proportion	2000	26.5	45.3	11.7
Ghana	Accra	Proportion	2017	39.8	65.6	20.3
Ghana	Accra	Count	2000	0	0	0
Ghana	Accra	Count	2017	0	1	0
Ghana	Bole	Proportion	2000	28.1	45.6	14.9
Ghana	Bole	Proportion	2017	41.6	65.0	22.6
Ghana	Bole	Count	2000	0	0	0
Ghana	Bole	Count	2017	0	1	0
Ghana	Bawku Municipal	Proportion	2000	38.9	56.1	21.5
Ghana	Bawku Municipal	Proportion	2017	33.5	53.5	15.4
Ghana	Bawku Municipal	Count	2000	0	1	0
Ghana	Bawku Municipal	Count	2017	0	1	0
Ghana	Jirapa Lambussie	Proportion	2000	30.8	52.3	13.2
Ghana	Jirapa Lambussie	Proportion	2017	37.8	64.3	18.6
Ghana	Jirapa Lambussie	Count	2000	0	1	0
Ghana	Jirapa Lambussie	Count	2017	0	1	0
Ghana	Adaklu Anyigbe	Proportion	2000	24.2	39.7	11.7
Ghana	Adaklu Anyigbe	Proportion	2017	35.3	56.1	16.8
Ghana	Adaklu Anyigbe	Count	2000	0	0	0
Ghana	Adaklu Anyigbe	Count	2017	0	1	0
Ghana	Ahanta West	Proportion	2000	42.9	63.6	22.7
Ghana	Ahanta West	Proportion	2017	51.3	75.2	24.3
Ghana	Ahanta West	Count	2000	0	1	0
Ghana	Ahanta West	Count	2017	1	1	0
Ghana	Adansi South	Proportion	2000	36.6	59.3	18.6
Ghana	Adansi South	Proportion	2017	49.5	74.7	25.6
Ghana	Adansi South	Count	2000	0	1	0
Ghana	Adansi South	Count	2017	0	1	0
Ghana	Asunafo South	Proportion	2000	35.9	58.1	18.1
Ghana	Asunafo South	Proportion	2017	42.5	65.6	22.0
Ghana	Asunafo South	Count	2000	0	1	0
Ghana	Asunafo South	Count	2017	0	1	0
Ghana	Agona	Proportion	2000	35.5	55.1	16.0
Ghana	Agona	Proportion	2017	48.9	72.2	28.3
Ghana	Agona	Count	2000	0	1	0
Ghana	Agona	Count	2017	0	1	0

Table 4: ORS by Unit *(continued)*

Country	Unit	Metric	Year	Mean	Upper	Lower
Ghana	Akwapim North	Proportion	2000	27.2	46.5	12.8
Ghana	Akwapim North	Proportion	2017	40.8	65.5	22.5
Ghana	Akwapim North	Count	2000	0	0	0
Ghana	Akwapim North	Count	2017	0	1	0
Ghana	Dangbe East	Proportion	2000	25.1	42.8	11.4
Ghana	Dangbe East	Proportion	2017	37.1	61.0	18.9
Ghana	Dangbe East	Count	2000	0	0	0
Ghana	Dangbe East	Count	2017	0	1	0
Ghana	Bunkpurugu Yunyoo	Proportion	2000	34.7	51.4	18.8
Ghana	Bunkpurugu Yunyoo	Proportion	2017	35.4	55.8	17.4
Ghana	Bunkpurugu Yunyoo	Count	2000	0	1	0
Ghana	Bunkpurugu Yunyoo	Count	2017	0	1	0
Ghana	Bawku West	Proportion	2000	37.1	56.6	19.9
Ghana	Bawku West	Proportion	2017	35.1	56.4	15.0
Ghana	Bawku West	Count	2000	0	1	0
Ghana	Bawku West	Count	2017	0	1	0
Ghana	Lawra	Proportion	2000	31.0	55.2	13.4
Ghana	Lawra	Proportion	2017	37.1	64.1	17.4
Ghana	Lawra	Count	2000	0	1	0
Ghana	Lawra	Count	2017	0	1	0
Ghana	Akatsi	Proportion	2000	23.7	40.5	12.1
Ghana	Akatsi	Proportion	2017	34.5	55.7	16.4
Ghana	Akatsi	Count	2000	0	0	0
Ghana	Akatsi	Count	2017	0	1	0
Ghana	Aowin-Suaman	Proportion	2000	43.1	64.7	27.4
Ghana	Aowin-Suaman	Proportion	2017	46.1	69.0	27.1
Ghana	Aowin-Suaman	Count	2000	0	1	0
Ghana	Aowin-Suaman	Count	2017	0	1	0
Ghana	Afigya Sekyere	Proportion	2000	32.6	56.3	15.9
Ghana	Afigya Sekyere	Proportion	2017	42.9	67.5	20.1
Ghana	Afigya Sekyere	Count	2000	0	1	0
Ghana	Afigya Sekyere	Count	2017	0	1	0
Ghana	Asutifi	Proportion	2000	32.8	52.3	15.1
Ghana	Asutifi	Proportion	2017	40.8	63.7	20.1
Ghana	Asutifi	Count	2000	0	1	0
Ghana	Asutifi	Count	2017	0	1	0
Ghana	Ajumako-Enyan-Esiam	Proportion	2000	37.9	60.5	19.7
Ghana	Ajumako-Enyan-Esiam	Proportion	2017	50.2	72.8	26.3
Ghana	Ajumako-Enyan-Esiam	Count	2000	0	1	0
Ghana	Ajumako-Enyan-Esiam	Count	2017	1	1	0
Ghana	Akwapim South	Proportion	2000	28.6	47.1	12.7
Ghana	Akwapim South	Proportion	2017	42.0	65.4	22.5
Ghana	Akwapim South	Count	2000	0	0	0
Ghana	Akwapim South	Count	2017	0	1	0
Ghana	Dangbe West	Proportion	2000	23.3	40.0	11.5
Ghana	Dangbe West	Proportion	2017	34.1	57.3	15.9
Ghana	Dangbe West	Count	2000	0	0	0
Ghana	Dangbe West	Count	2017	0	1	0
Ghana	Central Gonja	Proportion	2000	29.1	51.1	12.7

Table 4: ORS by Unit *(continued)*

Country	Unit	Metric	Year	Mean	Upper	Lower
Ghana	Central Gonja	Proportion	2017	40.0	62.9	19.4
Ghana	Central Gonja	Count	2000	0	1	0
Ghana	Central Gonja	Count	2017	0	1	0
Ghana	Bolgatanga	Proportion	2000	37.2	58.4	19.4
Ghana	Bolgatanga	Proportion	2017	36.8	61.9	14.0
Ghana	Bolgatanga	Count	2000	0	1	0
Ghana	Bolgatanga	Count	2017	0	1	0
Ghana	Nadowli	Proportion	2000	29.9	49.3	12.9
Ghana	Nadowli	Proportion	2017	39.6	64.7	18.7
Ghana	Nadowli	Count	2000	0	0	0
Ghana	Nadowli	Count	2017	0	1	0
Ghana	Ho	Proportion	2000	24.8	41.0	12.3
Ghana	Ho	Proportion	2017	35.9	56.8	16.2
Ghana	Ho	Count	2000	0	0	0
Ghana	Ho	Count	2017	0	1	0
Ghana	Bia	Proportion	2000	35.3	53.8	19.6
Ghana	Bia	Proportion	2017	41.3	67.4	21.8
Ghana	Bia	Count	2000	0	1	0
Ghana	Bia	Count	2017	0	1	0
Ghana	Ahafo Ano North	Proportion	2000	32.9	53.0	15.4
Ghana	Ahafo Ano North	Proportion	2017	40.9	63.5	19.3
Ghana	Ahafo Ano North	Count	2000	0	1	0
Ghana	Ahafo Ano North	Count	2017	0	1	0
Ghana	Atebubu-Amantin	Proportion	2000	30.0	52.0	14.2
Ghana	Atebubu-Amantin	Proportion	2017	42.3	66.9	20.0
Ghana	Atebubu-Amantin	Count	2000	0	1	0
Ghana	Atebubu-Amantin	Count	2017	0	1	0
Ghana	Asikuma Odoben Brakwa	Proportion	2000	38.0	58.0	17.8
Ghana	Asikuma Odoben Brakwa	Proportion	2017	51.8	74.6	29.8
Ghana	Asikuma Odoben Brakwa	Count	2000	0	1	0
Ghana	Asikuma Odoben Brakwa	Count	2017	1	1	0
Ghana	Asuogyaman	Proportion	2000	25.8	44.0	11.3
Ghana	Asuogyaman	Proportion	2017	38.0	60.9	19.3
Ghana	Asuogyaman	Count	2000	0	0	0
Ghana	Asuogyaman	Count	2017	0	1	0
Ghana	Ga East	Proportion	2000	27.0	46.3	11.7
Ghana	Ga East	Proportion	2017	40.1	65.6	21.0
Ghana	Ga East	Count	2000	0	0	0
Ghana	Ga East	Count	2017	0	1	0
Ghana	East Gonja	Proportion	2000	29.6	50.1	13.3
Ghana	East Gonja	Proportion	2017	37.8	60.9	18.0
Ghana	East Gonja	Count	2000	0	1	0
Ghana	East Gonja	Count	2017	0	1	0
Ghana	Bongo	Proportion	2000	37.1	58.5	18.8
Ghana	Bongo	Proportion	2017	35.9	59.9	13.8
Ghana	Bongo	Count	2000	0	1	0
Ghana	Bongo	Count	2017	0	1	0
Ghana	Sissala East	Proportion	2000	32.4	51.4	15.9
Ghana	Sissala East	Proportion	2017	38.5	62.0	17.9

Table 4: ORS by Unit *(continued)*

Country	Unit	Metric	Year	Mean	Upper	Lower
Ghana	Sissala East	Count	2000	0	1	0
Ghana	Sissala East	Count	2017	0	1	0
Ghana	Hohoe	Proportion	2000	25.6	42.6	13.1
Ghana	Hohoe	Proportion	2017	35.3	57.6	16.5
Ghana	Hohoe	Count	2000	0	0	0
Ghana	Hohoe	Count	2017	0	1	0
Ghana	Bibiani Anhwiaso Bekwai	Proportion	2000	37.0	59.2	19.9
Ghana	Bibiani Anhwiaso Bekwai	Proportion	2017	44.0	68.6	24.4
Ghana	Bibiani Anhwiaso Bekwai	Count	2000	0	1	0
Ghana	Bibiani Anhwiaso Bekwai	Count	2017	0	1	0
Ghana	Ahafo Ano South	Proportion	2000	32.9	54.5	16.3
Ghana	Ahafo Ano South	Proportion	2017	41.2	64.0	18.9
Ghana	Ahafo Ano South	Count	2000	0	1	0
Ghana	Ahafo Ano South	Count	2017	0	1	0
Ghana	Berekum	Proportion	2000	29.6	46.2	13.1
Ghana	Berekum	Proportion	2017	39.6	64.7	20.3
Ghana	Berekum	Count	2000	0	0	0
Ghana	Berekum	Count	2017	0	1	0
Ghana	Assin North	Proportion	2000	38.5	61.0	19.0
Ghana	Assin North	Proportion	2017	51.5	77.1	28.1
Ghana	Assin North	Count	2000	0	1	0
Ghana	Assin North	Count	2017	1	1	0
Ghana	Atiwa	Proportion	2000	32.3	51.3	14.6
Ghana	Atiwa	Proportion	2017	48.3	74.3	25.3
Ghana	Atiwa	Count	2000	0	1	0
Ghana	Atiwa	Count	2017	0	1	0
Ghana	Ga West	Proportion	2000	27.6	46.3	12.0
Ghana	Ga West	Proportion	2017	40.8	66.1	22.1
Ghana	Ga West	Count	2000	0	0	0
Ghana	Ga West	Count	2017	0	1	0
Ghana	East Mamprusi	Proportion	2000	34.3	52.7	18.2
Ghana	East Mamprusi	Proportion	2017	37.0	57.2	16.2
Ghana	East Mamprusi	Count	2000	0	1	0
Ghana	East Mamprusi	Count	2017	0	1	0
Ghana	Builsa	Proportion	2000	34.9	57.3	16.9
Ghana	Builsa	Proportion	2017	37.9	63.3	16.4
Ghana	Builsa	Count	2000	0	1	0
Ghana	Builsa	Count	2017	0	1	0
Ghana	Sissala West	Proportion	2000	31.3	50.7	14.0
Ghana	Sissala West	Proportion	2017	38.0	63.4	17.1
Ghana	Sissala West	Count	2000	0	1	0
Ghana	Sissala West	Count	2017	0	1	0
Ghana	Jasikan	Proportion	2000	26.3	45.9	13.1
Ghana	Jasikan	Proportion	2017	34.8	57.0	16.2
Ghana	Jasikan	Count	2000	0	0	0
Ghana	Jasikan	Count	2017	0	1	0
Ghana	Jomoro	Proportion	2000	51.9	71.8	34.0
Ghana	Jomoro	Proportion	2017	54.2	73.8	31.5
Ghana	Jomoro	Count	2000	1	1	0

Table 4: ORS by Unit *(continued)*

Country	Unit	Metric	Year	Mean	Upper	Lower
Ghana	Jomoro	Count	2017	1	1	0
Ghana	Amansie Central	Proportion	2000	35.0	57.6	18.4
Ghana	Amansie Central	Proportion	2017	45.3	70.0	22.3
Ghana	Amansie Central	Count	2000	0	1	0
Ghana	Amansie Central	Count	2017	0	1	0
Ghana	Dormaa	Proportion	2000	30.8	48.8	14.6
Ghana	Dormaa	Proportion	2017	39.8	66.6	20.3
Ghana	Dormaa	Count	2000	0	0	0
Ghana	Dormaa	Count	2017	0	1	0
Ghana	Assin South	Proportion	2000	38.6	60.7	19.6
Ghana	Assin South	Proportion	2017	51.3	75.4	25.5
Ghana	Assin South	Count	2000	0	1	0
Ghana	Assin South	Count	2017	1	1	0
Ghana	Birim North	Proportion	2000	34.1	54.0	16.1
Ghana	Birim North	Proportion	2017	49.4	72.9	25.4
Ghana	Birim North	Count	2000	0	1	0
Ghana	Birim North	Count	2017	0	1	0
Ghana	Tema	Proportion	2000	24.8	44.4	11.3
Ghana	Tema	Proportion	2017	37.2	61.1	18.9
Ghana	Tema	Count	2000	0	0	0
Ghana	Tema	Count	2017	0	1	0
Ghana	Gushiegu	Proportion	2000	31.1	51.8	15.4
Ghana	Gushiegu	Proportion	2017	38.7	60.5	19.0
Ghana	Gushiegu	Count	2000	0	1	0
Ghana	Gushiegu	Count	2017	0	1	0
Ghana	Garu Tempane	Proportion	2000	38.4	58.3	22.8
Ghana	Garu Tempane	Proportion	2017	34.2	55.1	16.2
Ghana	Garu Tempane	Count	2000	0	1	0
Ghana	Garu Tempane	Count	2017	0	1	0
Ghana	Wa	Proportion	2000	29.4	48.5	13.1
Ghana	Wa	Proportion	2017	40.9	64.2	18.5
Ghana	Wa	Count	2000	0	0	0
Ghana	Wa	Count	2017	0	1	0
Ghana	Kadjebi	Proportion	2000	25.7	41.8	13.2
Ghana	Kadjebi	Proportion	2017	32.3	53.8	14.6
Ghana	Kadjebi	Count	2000	0	0	0
Ghana	Kadjebi	Count	2017	0	1	0
Ghana	Juabeso	Proportion	2000	38.7	58.6	23.3
Ghana	Juabeso	Proportion	2017	43.0	67.2	23.2
Ghana	Juabeso	Count	2000	0	1	0
Ghana	Juabeso	Count	2017	0	1	0
Ghana	Amansie East	Proportion	2000	34.6	58.7	17.4
Ghana	Amansie East	Proportion	2017	47.2	69.8	23.4
Ghana	Amansie East	Count	2000	0	1	0
Ghana	Amansie East	Count	2017	0	1	0
Ghana	Jaman North	Proportion	2000	28.2	43.0	13.8
Ghana	Jaman North	Proportion	2017	39.5	64.3	20.6
Ghana	Jaman North	Count	2000	0	0	0
Ghana	Jaman North	Count	2017	0	1	0

Table 4: ORS by Unit *(continued)*

Country	Unit	Metric	Year	Mean	Upper	Lower
Ghana	Awutu Efutu Senya	Proportion	2000	31.0	52.2	13.8
Ghana	Awutu Efutu Senya	Proportion	2017	44.3	69.4	24.1
Ghana	Awutu Efutu Senya	Count	2000	0	1	0
Ghana	Awutu Efutu Senya	Count	2017	0	1	0
Ghana	Birim South	Proportion	2000	36.5	53.9	16.6
Ghana	Birim South	Proportion	2017	51.4	75.4	28.1
Ghana	Birim South	Count	2000	0	1	0
Ghana	Birim South	Count	2017	1	1	0
Ghana	Karaga	Proportion	2000	31.1	51.4	15.5
Ghana	Karaga	Proportion	2017	40.0	65.0	17.1
Ghana	Karaga	Count	2000	0	1	0
Ghana	Karaga	Count	2017	0	1	0
Ghana	Kassena Nankana	Proportion	2000	36.3	59.3	17.5
Ghana	Kassena Nankana	Proportion	2017	36.8	62.8	15.6
Ghana	Kassena Nankana	Count	2000	0	1	0
Ghana	Kassena Nankana	Count	2017	0	1	0
Ghana	Wa East	Proportion	2000	29.9	50.6	13.8
Ghana	Wa East	Proportion	2017	40.4	66.5	17.2
Ghana	Wa East	Count	2000	0	1	0
Ghana	Wa East	Count	2017	0	1	0
Ghana	Keta	Proportion	2000	24.1	41.8	12.5
Ghana	Keta	Proportion	2017	34.6	56.6	17.7
Ghana	Keta	Count	2000	0	0	0
Ghana	Keta	Count	2017	0	1	0
Ghana	Mpohor Wassa East	Proportion	2000	41.9	62.9	21.9
Ghana	Mpohor Wassa East	Proportion	2017	52.0	75.4	25.0
Ghana	Mpohor Wassa East	Count	2000	0	1	0
Ghana	Mpohor Wassa East	Count	2017	1	1	0
Ghana	Amansie West	Proportion	2000	34.9	56.1	17.9
Ghana	Amansie West	Proportion	2017	43.6	67.7	22.5
Ghana	Amansie West	Count	2000	0	1	0
Ghana	Amansie West	Count	2017	0	1	0
Ghana	Jaman South	Proportion	2000	28.6	45.4	14.1
Ghana	Jaman South	Proportion	2017	38.7	63.1	21.2
Ghana	Jaman South	Count	2000	0	0	0
Ghana	Jaman South	Count	2017	0	1	0
Ghana	Cape Coast	Proportion	2000	38.1	61.9	19.6
Ghana	Cape Coast	Proportion	2017	50.3	73.5	23.3
Ghana	Cape Coast	Count	2000	0	1	0
Ghana	Cape Coast	Count	2017	1	1	0
Ghana	East Akim	Proportion	2000	31.5	50.9	13.4
Ghana	East Akim	Proportion	2017	46.7	70.5	26.3
Ghana	East Akim	Count	2000	0	1	0
Ghana	East Akim	Count	2017	0	1	0
Ghana	Nanumba North	Proportion	2000	28.6	49.7	12.7
Ghana	Nanumba North	Proportion	2017	36.2	56.7	17.1
Ghana	Nanumba North	Count	2000	0	0	0
Ghana	Nanumba North	Count	2017	0	1	0
Ghana	Talensi Nabdam	Proportion	2000	36.5	57.7	18.4

Table 4: ORS by Unit *(continued)*

Country	Unit	Metric	Year	Mean	Upper	Lower
Ghana	Talensi Nabdam	Proportion	2017	36.9	61.0	13.8
Ghana	Talensi Nabdam	Count	2000	0	1	0
Ghana	Talensi Nabdam	Count	2017	0	1	0
Ghana	Wa West	Proportion	2000	29.9	49.9	13.2
Ghana	Wa West	Proportion	2017	41.5	64.5	19.2
Ghana	Wa West	Count	2000	0	0	0
Ghana	Wa West	Count	2017	0	1	0
Ghana	Ketu	Proportion	2000	21.7	35.1	11.2
Ghana	Ketu	Proportion	2017	31.5	49.6	16.3
Ghana	Ketu	Count	2000	0	0	0
Ghana	Ketu	Count	2017	0	0	0
Ghana	Nzema East	Proportion	2000	50.3	70.6	31.7
Ghana	Nzema East	Proportion	2017	55.8	76.9	32.6
Ghana	Nzema East	Count	2000	1	1	0
Ghana	Nzema East	Count	2017	1	1	0
Ghana	Asante Akim North	Proportion	2000	31.6	52.3	15.7
Ghana	Asante Akim North	Proportion	2017	44.7	71.1	21.9
Ghana	Asante Akim North	Count	2000	0	1	0
Ghana	Asante Akim North	Count	2017	0	1	0
Ghana	Kintampo North	Proportion	2000	29.3	50.0	13.7
Ghana	Kintampo North	Proportion	2017	42.4	67.9	21.1
Ghana	Kintampo North	Count	2000	0	0	0
Ghana	Kintampo North	Count	2017	0	1	0
Ghana	Gomoa	Proportion	2000	34.8	56.0	16.1
Ghana	Gomoa	Proportion	2017	47.4	70.2	25.3
Ghana	Gomoa	Count	2000	0	1	0
Ghana	Gomoa	Count	2017	0	1	0
Ghana	Fanteakwa	Proportion	2000	29.3	48.0	12.8
Ghana	Fanteakwa	Proportion	2017	43.8	70.1	24.1
Ghana	Fanteakwa	Count	2000	0	0	0
Ghana	Fanteakwa	Count	2017	0	1	0
Ghana	Nanumba South	Proportion	2000	28.0	46.6	13.1
Ghana	Nanumba South	Proportion	2017	34.6	57.7	15.4
Ghana	Nanumba South	Count	2000	0	0	0
Ghana	Nanumba South	Count	2017	0	1	0
Ghana	Kpandu	Proportion	2000	26.0	44.7	12.9
Ghana	Kpandu	Proportion	2017	36.0	59.3	16.1
Ghana	Kpandu	Count	2000	0	0	0
Ghana	Kpandu	Count	2017	0	1	0
Ghana	Sefwi Wiawso	Proportion	2000	38.9	61.6	22.7
Ghana	Sefwi Wiawso	Proportion	2017	44.2	67.6	23.5
Ghana	Sefwi Wiawso	Count	2000	0	1	0
Ghana	Sefwi Wiawso	Count	2017	0	1	0
Ghana	Asante Akim South	Proportion	2000	32.4	55.0	14.8
Ghana	Asante Akim South	Proportion	2017	47.0	72.3	23.1
Ghana	Asante Akim South	Count	2000	0	1	0
Ghana	Asante Akim South	Count	2017	0	1	0
Ghana	Kintampo South	Proportion	2000	30.4	52.1	14.9
Ghana	Kintampo South	Proportion	2017	43.0	67.6	22.9

Table 4: ORS by Unit *(continued)*

Country	Unit	Metric	Year	Mean	Upper	Lower
Ghana	Kintampo South	Count	2000	0	1	0
Ghana	Kintampo South	Count	2017	0	1	0
Ghana	Komenda-Edina-Eguafo-Abirem	Proportion	2000	38.8	62.0	20.0
Ghana	Komenda-Edina-Eguafo-Abirem	Proportion	2017	50.1	73.4	22.5
Ghana	Komenda-Edina-Eguafo-Abirem	Count	2000	0	1	0
Ghana	Komenda-Edina-Eguafo-Abirem	Count	2017	1	1	0
Ghana	Kwabibirem	Proportion	2000	34.6	55.0	16.1
Ghana	Kwabibirem	Proportion	2017	50.2	73.8	26.6
Ghana	Kwabibirem	Count	2000	0	1	0
Ghana	Kwabibirem	Count	2017	1	1	0
Ghana	Saboba Chereponi	Proportion	2000	27.2	42.0	15.0
Ghana	Saboba Chereponi	Proportion	2017	35.6	57.0	17.6
Ghana	Saboba Chereponi	Count	2000	0	0	0
Ghana	Saboba Chereponi	Count	2017	0	1	0
Ghana	Krachi	Proportion	2000	28.7	50.0	14.1
Ghana	Krachi	Proportion	2017	35.1	58.7	15.0
Ghana	Krachi	Count	2000	0	0	0
Ghana	Krachi	Count	2017	0	1	0
Ghana	Shama Ahanta East	Proportion	2000	41.6	65.2	20.8
Ghana	Shama Ahanta East	Proportion	2017	52.0	77.0	23.9
Ghana	Shama Ahanta East	Count	2000	0	1	0
Ghana	Shama Ahanta East	Count	2017	1	1	0
Ghana	Atwima	Proportion	2000	34.7	57.2	17.9
Ghana	Atwima	Proportion	2017	44.4	67.0	22.1
Ghana	Atwima	Count	2000	0	1	0
Ghana	Atwima	Count	2017	0	1	0
Ghana	Nkoranza	Proportion	2000	31.3	54.0	15.8
Ghana	Nkoranza	Proportion	2017	42.7	67.7	21.3
Ghana	Nkoranza	Count	2000	0	1	0
Ghana	Nkoranza	Count	2017	0	1	0
Ghana	Lower Denkyira	Proportion	2000	39.9	61.0	19.7
Ghana	Lower Denkyira	Proportion	2017	51.7	74.9	27.2
Ghana	Lower Denkyira	Count	2000	0	1	0
Ghana	Lower Denkyira	Count	2017	1	1	0
Ghana	Kwahu South	Proportion	2000	29.6	50.4	13.6
Ghana	Kwahu South	Proportion	2017	43.7	72.3	21.7
Ghana	Kwahu South	Count	2000	0	1	0
Ghana	Kwahu South	Count	2017	0	1	0
Ghana	Savelugu Nanton	Proportion	2000	30.9	51.2	15.1
Ghana	Savelugu Nanton	Proportion	2017	39.9	66.0	16.8
Ghana	Savelugu Nanton	Count	2000	0	1	0
Ghana	Savelugu Nanton	Count	2017	0	1	0
Ghana	Krachi East	Proportion	2000	27.2	45.3	13.1
Ghana	Krachi East	Proportion	2017	33.5	56.2	15.0
Ghana	Krachi East	Count	2000	0	0	0
Ghana	Krachi East	Count	2017	0	1	0

Table 4: ORS by Unit *(continued)*

Country	Unit	Metric	Year	Mean	Upper	Lower
Ghana	Wasa Amenfi East	Proportion	2000	38.8	63.7	18.8
Ghana	Wasa Amenfi East	Proportion	2017	47.6	71.2	24.1
Ghana	Wasa Amenfi East	Count	2000	0	1	0
Ghana	Wasa Amenfi East	Count	2017	0	1	0
Ghana	Atwima Mponua	Proportion	2000	34.8	55.6	17.7
Ghana	Atwima Mponua	Proportion	2017	42.5	65.6	22.3
Ghana	Atwima Mponua	Count	2000	0	1	0
Ghana	Atwima Mponua	Count	2017	0	1	0
Ghana	Pru	Proportion	2000	29.8	50.5	13.7
Ghana	Pru	Proportion	2017	41.8	64.1	20.6
Ghana	Pru	Count	2000	0	1	0
Ghana	Pru	Count	2017	0	1	0
Ghana	Mfantsiman	Proportion	2000	36.9	60.6	18.6
Ghana	Mfantsiman	Proportion	2017	49.2	71.9	23.5
Ghana	Mfantsiman	Count	2000	0	1	0
Ghana	Mfantsiman	Count	2017	0	1	0
Ghana	Kwahu West	Proportion	2000	31.4	52.5	13.9
Ghana	Kwahu West	Proportion	2017	47.0	76.1	22.9
Ghana	Kwahu West	Count	2000	0	1	0
Ghana	Kwahu West	Count	2017	0	1	0
Ghana	Sawa-Tuna-Kalba	Proportion	2000	28.4	47.8	14.2
Ghana	Sawa-Tuna-Kalba	Proportion	2017	41.3	66.3	18.5
Ghana	Sawa-Tuna-Kalba	Count	2000	0	0	0
Ghana	Sawa-Tuna-Kalba	Count	2017	0	1	0
Ghana	Nkwanta	Proportion	2000	26.0	43.2	12.8
Ghana	Nkwanta	Proportion	2017	32.0	52.1	15.0
Ghana	Nkwanta	Count	2000	0	0	0
Ghana	Nkwanta	Count	2017	0	1	0
Ghana	Wasa Amenfi West	Proportion	2000	40.7	64.3	22.3
Ghana	Wasa Amenfi West	Proportion	2017	46.9	70.0	24.6
Ghana	Wasa Amenfi West	Count	2000	0	1	0
Ghana	Wasa Amenfi West	Count	2017	0	1	0
Ghana	Bosomtwe-Kwanwoma	Proportion	2000	35.5	57.4	19.0
Ghana	Bosomtwe-Kwanwoma	Proportion	2017	46.0	71.3	24.5
Ghana	Bosomtwe-Kwanwoma	Count	2000	0	1	0
Ghana	Bosomtwe-Kwanwoma	Count	2017	0	1	0
Ghana	Sene	Proportion	2000	28.9	50.7	14.7
Ghana	Sene	Proportion	2017	38.9	62.7	18.1
Ghana	Sene	Count	2000	0	1	0
Ghana	Sene	Count	2017	0	1	0
Ghana	Upper Denkyira	Proportion	2000	38.2	61.9	18.5
Ghana	Upper Denkyira	Proportion	2017	48.4	71.5	24.2
Ghana	Upper Denkyira	Count	2000	0	1	0
Ghana	Upper Denkyira	Count	2017	0	1	0
Ghana	Manya Krobo	Proportion	2000	27.2	48.1	12.1
Ghana	Manya Krobo	Proportion	2017	40.5	66.4	20.1
Ghana	Manya Krobo	Count	2000	0	0	0
Ghana	Manya Krobo	Count	2017	0	1	0
Ghana	Tamale	Proportion	2000	31.9	54.3	14.9

Table 4: ORS by Unit *(continued)*

Country	Unit	Metric	Year	Mean	Upper	Lower
Ghana	Tamale	Proportion	2017	39.5	65.5	16.1
Ghana	Tamale	Count	2000	0	1	0
Ghana	Tamale	Count	2017	0	1	0
Ghana	North Tongu	Proportion	2000	24.6	40.2	12.1
Ghana	North Tongu	Proportion	2017	36.0	59.0	17.5
Ghana	North Tongu	Count	2000	0	0	0
Ghana	North Tongu	Count	2017	0	1	0
Ghana	Wassa West	Proportion	2000	43.5	65.0	23.3
Ghana	Wassa West	Proportion	2017	52.2	73.7	25.8
Ghana	Wassa West	Count	2000	0	1	0
Ghana	Wassa West	Count	2017	1	1	0
Ghana	Ejisu-Juabeng	Proportion	2000	33.9	57.1	17.8
Ghana	Ejisu-Juabeng	Proportion	2017	45.9	72.0	22.2
Ghana	Ejisu-Juabeng	Count	2000	0	1	0
Ghana	Ejisu-Juabeng	Count	2017	0	1	0
Ghana	Sunyani	Proportion	2000	31.0	48.6	14.1
Ghana	Sunyani	Proportion	2017	40.2	64.7	19.8
Ghana	Sunyani	Count	2000	0	0	0
Ghana	Sunyani	Count	2017	0	1	0
Ghana	New Juaben	Proportion	2000	30.4	50.3	12.8
Ghana	New Juaben	Proportion	2017	45.0	70.0	25.1
Ghana	New Juaben	Count	2000	0	1	0
Ghana	New Juaben	Count	2017	0	1	0
Ghana	Tolon-Kumbungu	Proportion	2000	30.2	51.8	13.0
Ghana	Tolon-Kumbungu	Proportion	2017	39.1	66.6	15.9
Ghana	Tolon-Kumbungu	Count	2000	0	1	0
Ghana	Tolon-Kumbungu	Count	2017	0	1	0
Ghana	South Dayi	Proportion	2000	25.4	42.7	11.6
Ghana	South Dayi	Proportion	2017	36.6	59.4	16.2
Ghana	South Dayi	Count	2000	0	0	0
Ghana	South Dayi	Count	2017	0	1	0
Ghana	Ejura Sekyedumase	Proportion	2000	30.9	53.3	14.6
Ghana	Ejura Sekyedumase	Proportion	2017	42.4	67.2	20.7
Ghana	Ejura Sekyedumase	Count	2000	0	1	0
Ghana	Ejura Sekyedumase	Count	2017	0	1	0
Ghana	Tain	Proportion	2000	30.2	49.1	13.8
Ghana	Tain	Proportion	2017	41.8	65.5	23.7
Ghana	Tain	Count	2000	0	0	0
Ghana	Tain	Count	2017	0	1	0
Ghana	Suhum Kraboa Coaltar	Proportion	2000	31.6	49.9	14.0
Ghana	Suhum Kraboa Coaltar	Proportion	2017	46.2	69.4	24.8
Ghana	Suhum Kraboa Coaltar	Count	2000	0	0	0
Ghana	Suhum Kraboa Coaltar	Count	2017	0	1	0
Ghana	West Gonja	Proportion	2000	28.7	49.1	13.7
Ghana	West Gonja	Proportion	2017	39.9	62.4	17.6
Ghana	West Gonja	Count	2000	0	0	0
Ghana	West Gonja	Count	2017	0	1	0
Ghana	South Tongu	Proportion	2000	24.3	40.7	12.6
Ghana	South Tongu	Proportion	2017	35.1	57.7	17.7

Table 4: ORS by Unit *(continued)*

Country	Unit	Metric	Year	Mean	Upper	Lower
Ghana	South Tongu	Count	2000	0	0	0
Ghana	South Tongu	Count	2017	0	1	0
Ghana	Kumasi	Proportion	2000	36.1	59.3	19.7
Ghana	Kumasi	Proportion	2017	46.9	70.9	24.4
Ghana	Kumasi	Count	2000	0	1	0
Ghana	Kumasi	Count	2017	0	1	0
Ghana	Tano North	Proportion	2000	31.6	52.4	15.2
Ghana	Tano North	Proportion	2017	40.5	63.6	18.5
Ghana	Tano North	Count	2000	0	1	0
Ghana	Tano North	Count	2017	0	1	0
Ghana	West Akim	Proportion	2000	34.0	52.4	15.0
Ghana	West Akim	Proportion	2017	48.6	70.7	27.5
Ghana	West Akim	Count	2000	0	1	0
Ghana	West Akim	Count	2017	0	1	0
Ghana	West Mamprusi	Proportion	2000	33.8	55.7	17.3
Ghana	West Mamprusi	Proportion	2017	38.2	63.5	15.5
Ghana	West Mamprusi	Count	2000	0	1	0
Ghana	West Mamprusi	Count	2017	0	1	0
Ghana	Kwabre	Proportion	2000	34.4	57.0	16.7
Ghana	Kwabre	Proportion	2017	44.8	69.1	20.5
Ghana	Kwabre	Count	2000	0	1	0
Ghana	Kwabre	Count	2017	0	1	0
Ghana	Tano South	Proportion	2000	32.1	51.7	15.7
Ghana	Tano South	Proportion	2017	41.1	63.4	18.3
Ghana	Tano South	Count	2000	0	1	0
Ghana	Tano South	Count	2017	0	1	0
Ghana	Yilo Krobo	Proportion	2000	27.8	46.8	12.4
Ghana	Yilo Krobo	Proportion	2017	41.8	67.2	21.9
Ghana	Yilo Krobo	Count	2000	0	0	0
Ghana	Yilo Krobo	Count	2017	0	1	0
Ghana	Yendi	Proportion	2000	28.9	47.5	14.6
Ghana	Yendi	Proportion	2017	38.2	59.8	17.4
Ghana	Yendi	Count	2000	0	0	0
Ghana	Yendi	Count	2017	0	1	0
Ghana	Obuasi Municipal	Proportion	2000	37.2	61.8	18.0
Ghana	Obuasi Municipal	Proportion	2017	48.6	73.7	24.0
Ghana	Obuasi Municipal	Count	2000	0	1	0
Ghana	Obuasi Municipal	Count	2017	0	1	0
Ghana	Techiman	Proportion	2000	31.3	53.3	15.4
Ghana	Techiman	Proportion	2017	42.3	65.5	22.2
Ghana	Techiman	Count	2000	0	1	0
Ghana	Techiman	Count	2017	0	1	0
Ghana	Zabzugu Tatale	Proportion	2000	26.2	40.9	12.3
Ghana	Zabzugu Tatale	Proportion	2017	34.8	54.9	16.4
Ghana	Zabzugu Tatale	Count	2000	0	0	0
Ghana	Zabzugu Tatale	Count	2017	0	1	0
Ghana	Offinso	Proportion	2000	32.4	53.7	16.2
Ghana	Offinso	Proportion	2017	42.2	66.5	19.7
Ghana	Offinso	Count	2000	0	1	0

Table 4: ORS by Unit *(continued)*

Country	Unit	Metric	Year	Mean	Upper	Lower
Ghana	Offinso	Count	2017	0	1	0
Ghana	Sekyere East	Proportion	2000	30.7	51.1	15.7
Ghana	Sekyere East	Proportion	2017	42.9	67.9	22.0
Ghana	Sekyere East	Count	2000	0	1	0
Ghana	Sekyere East	Count	2017	0	1	0
Ghana	Sekyere West	Proportion	2000	30.7	52.4	14.5
Ghana	Sekyere West	Proportion	2017	42.3	65.2	20.7
Ghana	Sekyere West	Count	2000	0	1	0
Ghana	Sekyere West	Count	2017	0	1	0
Guatemala	Chahal	Proportion	2000	37.8	58.9	21.0
Guatemala	Chahal	Proportion	2017	38.6	56.9	22.4
Guatemala	Chahal	Count	2000	0	1	0
Guatemala	Chahal	Count	2017	0	1	0
Guatemala	Cubulco	Proportion	2000	41.5	60.8	22.0
Guatemala	Cubulco	Proportion	2017	48.0	65.0	31.5
Guatemala	Cubulco	Count	2000	0	1	0
Guatemala	Cubulco	Count	2017	0	1	0
Guatemala	Acatenango	Proportion	2000	40.7	61.0	20.1
Guatemala	Acatenango	Proportion	2017	46.2	64.6	29.2
Guatemala	Acatenango	Count	2000	0	1	0
Guatemala	Acatenango	Count	2017	0	1	0
Guatemala	Camotán	Proportion	2000	41.0	65.1	21.3
Guatemala	Camotán	Proportion	2017	45.6	62.9	30.0
Guatemala	Camotán	Count	2000	0	1	0
Guatemala	Camotán	Count	2017	0	1	0
Guatemala	El Jícaro	Proportion	2000	44.6	66.2	22.7
Guatemala	El Jícaro	Proportion	2017	51.9	69.8	35.5
Guatemala	El Jícaro	Count	2000	0	1	0
Guatemala	El Jícaro	Count	2017	1	1	0
Guatemala	Escuintla	Proportion	2000	39.5	60.6	20.5
Guatemala	Escuintla	Proportion	2017	46.0	62.7	29.9
Guatemala	Escuintla	Count	2000	0	1	0
Guatemala	Escuintla	Count	2017	0	1	0
Guatemala	Amatitlán	Proportion	2000	43.6	65.3	22.7
Guatemala	Amatitlán	Proportion	2017	49.7	65.4	32.9
Guatemala	Amatitlán	Count	2000	0	1	0
Guatemala	Amatitlán	Count	2017	0	1	0
Guatemala	Aguacatán	Proportion	2000	40.4	62.3	19.5
Guatemala	Aguacatán	Proportion	2017	48.2	65.7	32.4
Guatemala	Aguacatán	Count	2000	0	1	0
Guatemala	Aguacatán	Count	2017	0	1	0
Guatemala	El Estor	Proportion	2000	38.2	60.6	20.6
Guatemala	El Estor	Proportion	2017	39.9	56.1	24.7
Guatemala	El Estor	Count	2000	0	1	0
Guatemala	El Estor	Count	2017	0	1	0
Guatemala	Jalapa	Proportion	2000	42.3	63.8	22.6
Guatemala	Jalapa	Proportion	2017	52.6	69.3	37.6
Guatemala	Jalapa	Count	2000	0	1	0
Guatemala	Jalapa	Count	2017	1	1	0

Table 4: ORS by Unit *(continued)*

Country	Unit	Metric	Year	Mean	Upper	Lower
Guatemala	Agua Blanca	Proportion	2000	43.7	63.3	23.1
Guatemala	Agua Blanca	Proportion	2017	53.2	69.7	36.3
Guatemala	Agua Blanca	Count	2000	0	1	0
Guatemala	Agua Blanca	Count	2017	1	1	0
Guatemala	Dolores	Proportion	2000	40.2	59.9	22.6
Guatemala	Dolores	Proportion	2017	40.4	57.0	24.4
Guatemala	Dolores	Count	2000	0	1	0
Guatemala	Dolores	Count	2017	0	1	0
Guatemala	Almolonga	Proportion	2000	40.8	64.1	18.8
Guatemala	Almolonga	Proportion	2017	43.4	63.5	24.4
Guatemala	Almolonga	Count	2000	0	1	0
Guatemala	Almolonga	Count	2017	0	1	0
Guatemala	Canillá	Proportion	2000	40.4	61.3	20.2
Guatemala	Canillá	Proportion	2017	47.3	65.1	30.4
Guatemala	Canillá	Count	2000	0	1	0
Guatemala	Canillá	Count	2017	0	1	0
Guatemala	Champerico	Proportion	2000	38.9	62.0	19.3
Guatemala	Champerico	Proportion	2017	40.6	57.7	24.2
Guatemala	Champerico	Count	2000	0	1	0
Guatemala	Champerico	Count	2017	0	1	0
Guatemala	Alotenango	Proportion	2000	40.9	62.7	20.2
Guatemala	Alotenango	Proportion	2017	47.2	64.9	29.8
Guatemala	Alotenango	Count	2000	0	1	0
Guatemala	Alotenango	Count	2017	0	1	0
Guatemala	Ayutla	Proportion	2000	38.5	60.8	19.3
Guatemala	Ayutla	Proportion	2017	40.7	57.5	25.0
Guatemala	Ayutla	Count	2000	0	1	0
Guatemala	Ayutla	Count	2017	0	1	0
Guatemala	Barberena	Proportion	2000	42.8	63.6	21.6
Guatemala	Barberena	Proportion	2017	53.9	70.1	38.4
Guatemala	Barberena	Count	2000	0	1	0
Guatemala	Barberena	Count	2017	1	1	0
Guatemala	Concepción	Proportion	2000	40.3	62.2	19.0
Guatemala	Concepción	Proportion	2017	44.6	63.2	27.8
Guatemala	Concepción	Count	2000	0	1	0
Guatemala	Concepción	Count	2017	0	1	0
Guatemala	Chicacao	Proportion	2000	37.0	57.8	17.7
Guatemala	Chicacao	Proportion	2017	39.4	57.6	25.6
Guatemala	Chicacao	Count	2000	0	1	0
Guatemala	Chicacao	Count	2017	0	1	0
Guatemala	Momostenango	Proportion	2000	39.6	61.6	19.5
Guatemala	Momostenango	Proportion	2017	45.5	60.9	30.4
Guatemala	Momostenango	Count	2000	0	1	0
Guatemala	Momostenango	Count	2017	0	1	0
Guatemala	Cabañas	Proportion	2000	43.4	63.9	22.5
Guatemala	Cabañas	Proportion	2017	49.9	66.4	33.5
Guatemala	Cabañas	Count	2000	0	1	0
Guatemala	Cabañas	Count	2017	0	1	0
Guatemala	Chisec	Proportion	2000	35.5	56.3	18.9

Table 4: ORS by Unit *(continued)*

Country	Unit	Metric	Year	Mean	Upper	Lower
Guatemala	Chisec	Proportion	2017	39.1	56.8	23.7
Guatemala	Chisec	Count	2000	0	1	0
Guatemala	Chisec	Count	2017	0	1	0
Guatemala	Granados	Proportion	2000	42.3	61.8	22.3
Guatemala	Granados	Proportion	2017	48.7	65.0	31.5
Guatemala	Granados	Count	2000	0	1	0
Guatemala	Granados	Count	2017	0	1	0
Guatemala	Chimaltenango	Proportion	2000	42.1	64.0	20.7
Guatemala	Chimaltenango	Proportion	2017	45.8	64.5	28.3
Guatemala	Chimaltenango	Count	2000	0	1	0
Guatemala	Chimaltenango	Count	2017	0	1	0
Guatemala	Chiquimula	Proportion	2000	42.0	65.6	22.3
Guatemala	Chiquimula	Proportion	2017	47.8	64.5	29.8
Guatemala	Chiquimula	Count	2000	0	1	0
Guatemala	Chiquimula	Count	2017	0	1	0
Guatemala	Guastatoya	Proportion	2000	45.6	65.4	23.4
Guatemala	Guastatoya	Proportion	2017	52.4	67.8	34.8
Guatemala	Guastatoya	Count	2000	0	1	0
Guatemala	Guastatoya	Count	2017	1	1	0
Guatemala	Guanagazapa	Proportion	2000	39.7	59.4	20.9
Guatemala	Guanagazapa	Proportion	2017	48.8	65.5	32.4
Guatemala	Guanagazapa	Count	2000	0	1	0
Guatemala	Guanagazapa	Count	2017	0	1	0
Guatemala	Chinautla	Proportion	2000	44.1	65.5	21.2
Guatemala	Chinautla	Proportion	2017	49.6	66.9	33.3
Guatemala	Chinautla	Count	2000	0	1	0
Guatemala	Chinautla	Count	2017	0	1	0
Guatemala	Chiantla	Proportion	2000	39.6	61.3	20.5
Guatemala	Chiantla	Proportion	2017	48.6	65.9	33.1
Guatemala	Chiantla	Count	2000	0	1	0
Guatemala	Chiantla	Count	2017	0	1	0
Guatemala	Livingston	Proportion	2000	38.3	60.8	21.2
Guatemala	Livingston	Proportion	2017	37.9	53.9	22.7
Guatemala	Livingston	Count	2000	0	1	0
Guatemala	Livingston	Count	2017	0	1	0
Guatemala	Mataquescuintla	Proportion	2000	40.9	63.3	20.0
Guatemala	Mataquescuintla	Proportion	2017	51.7	68.4	34.5
Guatemala	Mataquescuintla	Count	2000	0	1	0
Guatemala	Mataquescuintla	Count	2017	1	1	0
Guatemala	Asunción Mita	Proportion	2000	44.3	66.2	24.3
Guatemala	Asunción Mita	Proportion	2017	55.7	72.1	38.9
Guatemala	Asunción Mita	Count	2000	0	1	0
Guatemala	Asunción Mita	Count	2017	1	1	0
Guatemala	Flores	Proportion	2000	40.9	60.5	24.4
Guatemala	Flores	Proportion	2017	41.7	59.6	26.4
Guatemala	Flores	Count	2000	0	1	0
Guatemala	Flores	Count	2017	0	1	0
Guatemala	Cabricán	Proportion	2000	39.1	59.3	19.3
Guatemala	Cabricán	Proportion	2017	45.1	62.9	28.8

Table 4: ORS by Unit *(continued)*

Country	Unit	Metric	Year	Mean	Upper	Lower
Guatemala	Cabricán	Count	2000	0	1	0
Guatemala	Cabricán	Count	2017	0	1	0
Guatemala	Chajul	Proportion	2000	35.9	58.2	17.2
Guatemala	Chajul	Proportion	2017	43.4	60.9	29.0
Guatemala	Chajul	Count	2000	0	1	0
Guatemala	Chajul	Count	2017	0	1	0
Guatemala	El Asintal	Proportion	2000	36.5	59.3	18.4
Guatemala	El Asintal	Proportion	2017	38.2	56.9	23.1
Guatemala	El Asintal	Count	2000	0	1	0
Guatemala	El Asintal	Count	2017	0	1	0
Guatemala	Antigua Guatemala	Proportion	2000	42.7	63.3	21.1
Guatemala	Antigua Guatemala	Proportion	2017	47.8	65.8	31.4
Guatemala	Antigua Guatemala	Count	2000	0	1	0
Guatemala	Antigua Guatemala	Count	2017	0	1	0
Guatemala	Catarina	Proportion	2000	36.4	59.2	17.7
Guatemala	Catarina	Proportion	2017	38.2	55.0	24.3
Guatemala	Catarina	Count	2000	0	1	0
Guatemala	Catarina	Count	2017	0	1	0
Guatemala	Casillas	Proportion	2000	41.7	63.7	21.6
Guatemala	Casillas	Proportion	2017	54.0	69.5	39.1
Guatemala	Casillas	Count	2000	0	1	0
Guatemala	Casillas	Count	2017	1	1	0
Guatemala	NA	Proportion	2000	40.7	61.8	19.5
Guatemala	NA	Proportion	2017	44.7	61.9	28.2
Guatemala	NA	Count	2000	0	1	0
Guatemala	NA	Count	2017	0	1	0
Guatemala	Cuyotenango	Proportion	2000	36.8	57.1	18.2
Guatemala	Cuyotenango	Proportion	2017	38.3	56.9	23.4
Guatemala	Cuyotenango	Count	2000	0	1	0
Guatemala	Cuyotenango	Count	2017	0	1	0
Guatemala	San Andrés Xecul	Proportion	2000	39.8	63.5	19.2
Guatemala	San Andrés Xecul	Proportion	2017	42.4	61.3	27.1
Guatemala	San Andrés Xecul	Count	2000	0	1	0
Guatemala	San Andrés Xecul	Count	2017	0	1	0
Guatemala	Estanzuela	Proportion	2000	42.8	66.0	22.6
Guatemala	Estanzuela	Proportion	2017	46.7	64.5	30.3
Guatemala	Estanzuela	Count	2000	0	1	0
Guatemala	Estanzuela	Count	2017	0	1	0
Guatemala	Cobán	Proportion	2000	40.1	59.6	21.1
Guatemala	Cobán	Proportion	2017	45.6	62.2	29.1
Guatemala	Cobán	Count	2000	0	1	0
Guatemala	Cobán	Count	2017	0	1	0
Guatemala	Purulhá	Proportion	2000	39.6	59.4	20.2
Guatemala	Purulhá	Proportion	2017	45.8	62.1	30.7
Guatemala	Purulhá	Count	2000	0	1	0
Guatemala	Purulhá	Count	2017	0	1	0
Guatemala	Comalapa	Proportion	2000	40.2	61.2	20.2
Guatemala	Comalapa	Proportion	2017	46.2	63.6	29.2
Guatemala	Comalapa	Count	2000	0	1	0

Table 4: ORS by Unit *(continued)*

Country	Unit	Metric	Year	Mean	Upper	Lower
Guatemala	Comalapa	Count	2017	0	1	0
Guatemala	Concepción Las Minas	Proportion	2000	42.9	62.7	24.0
Guatemala	Concepción Las Minas	Proportion	2017	51.5	68.6	35.2
Guatemala	Concepción Las Minas	Count	2000	0	1	0
Guatemala	Concepción Las Minas	Count	2017	1	1	0
Guatemala	Morazán	Proportion	2000	44.8	64.4	23.3
Guatemala	Morazán	Proportion	2017	51.9	69.2	35.4
Guatemala	Morazán	Count	2000	0	1	0
Guatemala	Morazán	Count	2017	1	1	0
Guatemala	Iztapa	Proportion	2000	42.4	64.0	21.7
Guatemala	Iztapa	Proportion	2017	50.7	70.2	34.0
Guatemala	Iztapa	Count	2000	0	1	0
Guatemala	Iztapa	Count	2017	1	1	0
Guatemala	Chuarrancho	Proportion	2000	43.7	62.8	23.6
Guatemala	Chuarrancho	Proportion	2017	50.3	66.8	33.9
Guatemala	Chuarrancho	Count	2000	0	1	0
Guatemala	Chuarrancho	Count	2017	1	1	0
Guatemala	Colotenango	Proportion	2000	40.3	64.2	18.9
Guatemala	Colotenango	Proportion	2017	49.4	66.8	32.8
Guatemala	Colotenango	Count	2000	0	1	0
Guatemala	Colotenango	Count	2017	0	1	0
Guatemala	Los Amates	Proportion	2000	40.1	62.3	22.8
Guatemala	Los Amates	Proportion	2017	42.1	60.4	26.0
Guatemala	Los Amates	Count	2000	0	1	0
Guatemala	Los Amates	Count	2017	0	1	0
Guatemala	Monjas	Proportion	2000	43.4	66.0	23.3
Guatemala	Monjas	Proportion	2017	53.9	70.2	38.8
Guatemala	Monjas	Count	2000	0	1	0
Guatemala	Monjas	Count	2017	1	1	0
Guatemala	Atescatempa	Proportion	2000	45.4	67.1	25.1
Guatemala	Atescatempa	Proportion	2017	58.0	74.4	39.6
Guatemala	Atescatempa	Count	2000	0	1	0
Guatemala	Atescatempa	Count	2017	1	1	0
Guatemala	La Libertad	Proportion	2000	38.6	58.3	19.5
Guatemala	La Libertad	Proportion	2017	42.3	60.6	26.5
Guatemala	La Libertad	Count	2000	0	1	0
Guatemala	La Libertad	Count	2017	0	1	0
Guatemala	Cajolá	Proportion	2000	38.2	59.3	18.1
Guatemala	Cajolá	Proportion	2017	42.2	60.9	27.5
Guatemala	Cajolá	Count	2000	0	1	0
Guatemala	Cajolá	Count	2017	0	1	0
Guatemala	Chicaman	Proportion	2000	39.8	60.5	20.8
Guatemala	Chicaman	Proportion	2017	46.9	62.7	31.6
Guatemala	Chicaman	Count	2000	0	1	0
Guatemala	Chicaman	Count	2017	0	1	0
Guatemala	Nuevo San Carlos	Proportion	2000	36.3	57.8	17.0
Guatemala	Nuevo San Carlos	Proportion	2017	38.2	57.3	23.9
Guatemala	Nuevo San Carlos	Count	2000	0	1	0
Guatemala	Nuevo San Carlos	Count	2017	0	1	0

Table 4: ORS by Unit *(continued)*

Country	Unit	Metric	Year	Mean	Upper	Lower
Guatemala	Ciudad Vieja	Proportion	2000	43.0	67.5	22.1
Guatemala	Ciudad Vieja	Proportion	2017	47.7	69.0	28.4
Guatemala	Ciudad Vieja	Count	2000	0	1	0
Guatemala	Ciudad Vieja	Count	2017	0	1	0
Guatemala	Comitancillo	Proportion	2000	36.1	57.7	15.9
Guatemala	Comitancillo	Proportion	2017	40.4	58.9	25.8
Guatemala	Comitancillo	Count	2000	0	1	0
Guatemala	Comitancillo	Count	2017	0	1	0
Guatemala	Chiquimulilla	Proportion	2000	40.9	60.8	21.8
Guatemala	Chiquimulilla	Proportion	2017	53.3	69.3	39.2
Guatemala	Chiquimulilla	Count	2000	0	1	0
Guatemala	Chiquimulilla	Count	2017	1	1	0
Guatemala	Nahualá	Proportion	2000	38.3	59.8	18.9
Guatemala	Nahualá	Proportion	2017	41.9	58.9	26.9
Guatemala	Nahualá	Count	2000	0	1	0
Guatemala	Nahualá	Count	2017	0	1	0
Guatemala	Mazatenango	Proportion	2000	37.0	59.9	18.0
Guatemala	Mazatenango	Proportion	2017	37.8	56.5	23.2
Guatemala	Mazatenango	Count	2000	0	1	0
Guatemala	Mazatenango	Count	2017	0	1	0
Guatemala	San Bartolo	Proportion	2000	40.1	63.2	18.9
Guatemala	San Bartolo	Proportion	2017	46.2	64.0	29.8
Guatemala	San Bartolo	Count	2000	0	1	0
Guatemala	San Bartolo	Count	2017	0	1	0
Guatemala	Gualán	Proportion	2000	40.9	64.1	21.8
Guatemala	Gualán	Proportion	2017	43.8	60.2	28.7
Guatemala	Gualán	Count	2000	0	1	0
Guatemala	Gualán	Count	2017	0	1	0
Guatemala	Fray Bartolomé de las Casas	Proportion	2000	37.9	58.1	20.0
Guatemala	Fray Bartolomé de las Casas	Proportion	2017	39.4	55.9	23.8
Guatemala	Fray Bartolomé de las Casas	Count	2000	0	1	0
Guatemala	Fray Bartolomé de las Casas	Count	2017	0	1	0
Guatemala	Rabinal	Proportion	2000	43.2	62.0	22.7
Guatemala	Rabinal	Proportion	2017	49.5	66.6	33.7
Guatemala	Rabinal	Count	2000	0	1	0
Guatemala	Rabinal	Count	2017	0	1	0
Guatemala	El Tejar	Proportion	2000	41.7	63.6	20.8
Guatemala	El Tejar	Proportion	2017	45.5	64.2	27.3
Guatemala	El Tejar	Count	2000	0	1	0
Guatemala	El Tejar	Count	2017	0	1	0
Guatemala	Esquipulas	Proportion	2000	43.6	64.0	24.9
Guatemala	Esquipulas	Proportion	2017	49.5	65.6	33.3
Guatemala	Esquipulas	Count	2000	0	1	0
Guatemala	Esquipulas	Count	2017	0	1	0
Guatemala	San Agustín Acasaguastlán	Proportion	2000	44.4	65.0	22.9

Table 4: ORS by Unit *(continued)*

Country	Unit	Metric	Year	Mean	Upper	Lower
Guatemala	San Agustín Acasaguastlán	Proportion	2017	51.5	67.7	36.2
Guatemala	San Agustín Acasaguastlán	Count	2000	0	1	0
Guatemala	San Agustín Acasaguastlán	Count	2017	1	1	0
Guatemala	La Democracia	Proportion	2000	39.7	61.4	21.1
Guatemala	La Democracia	Proportion	2017	44.5	61.4	28.9
Guatemala	La Democracia	Count	2000	0	1	0
Guatemala	La Democracia	Count	2017	0	1	0
Guatemala	Fraijanes	Proportion	2000	42.8	65.6	21.0
Guatemala	Fraijanes	Proportion	2017	51.1	68.2	33.2
Guatemala	Fraijanes	Count	2000	0	1	0
Guatemala	Fraijanes	Count	2017	1	1	0
Guatemala	Concepción Huista	Proportion	2000	40.5	64.0	21.2
Guatemala	Concepción Huista	Proportion	2017	50.6	67.9	32.8
Guatemala	Concepción Huista	Count	2000	0	1	0
Guatemala	Concepción Huista	Count	2017	1	1	0
Guatemala	Morales	Proportion	2000	40.1	62.6	23.4
Guatemala	Morales	Proportion	2017	41.3	58.9	26.1
Guatemala	Morales	Count	2000	0	1	0
Guatemala	Morales	Count	2017	0	1	0
Guatemala	San Carlos Alzatate	Proportion	2000	38.8	61.7	19.5
Guatemala	San Carlos Alzatate	Proportion	2017	50.6	67.5	36.1
Guatemala	San Carlos Alzatate	Count	2000	0	1	0
Guatemala	San Carlos Alzatate	Count	2017	1	1	0
Guatemala	Comapa	Proportion	2000	42.9	66.1	22.7
Guatemala	Comapa	Proportion	2017	57.2	72.1	40.9
Guatemala	Comapa	Count	2000	0	1	0
Guatemala	Comapa	Count	2017	1	1	0
Guatemala	Melchor de Mencos	Proportion	2000	42.7	61.4	24.8
Guatemala	Melchor de Mencos	Proportion	2017	43.2	60.7	28.0
Guatemala	Melchor de Mencos	Count	2000	0	1	0
Guatemala	Melchor de Mencos	Count	2017	0	1	0
Guatemala	Cantel	Proportion	2000	39.9	62.7	18.9
Guatemala	Cantel	Proportion	2017	42.6	61.5	27.6
Guatemala	Cantel	Count	2000	0	1	0
Guatemala	Cantel	Count	2017	0	1	0
Guatemala	Chiché	Proportion	2000	39.9	62.7	19.9
Guatemala	Chiché	Proportion	2017	45.7	60.8	28.2
Guatemala	Chiché	Count	2000	0	1	0
Guatemala	Chiché	Count	2017	0	1	0
Guatemala	Retalhuleu	Proportion	2000	37.5	59.5	19.3
Guatemala	Retalhuleu	Proportion	2017	39.1	56.1	24.7
Guatemala	Retalhuleu	Count	2000	0	1	0
Guatemala	Retalhuleu	Count	2017	0	1	0
Guatemala	Jocotenango	Proportion	2000	42.1	65.6	19.7
Guatemala	Jocotenango	Proportion	2017	47.1	64.5	31.0
Guatemala	Jocotenango	Count	2000	0	1	0
Guatemala	Jocotenango	Count	2017	0	1	0
Guatemala	Concepción Tutuapa	Proportion	2000	38.5	59.1	19.5
Guatemala	Concepción Tutuapa	Proportion	2017	45.0	62.1	30.0

Table 4: ORS by Unit *(continued)*

Country	Unit	Metric	Year	Mean	Upper	Lower
Guatemala	Concepción Tutuapa	Count	2000	0	1	0
Guatemala	Concepción Tutuapa	Count	2017	0	1	0
Guatemala	Cuilapa	Proportion	2000	43.1	64.1	21.6
Guatemala	Cuilapa	Proportion	2017	54.4	70.7	38.7
Guatemala	Cuilapa	Count	2000	0	1	0
Guatemala	Cuilapa	Count	2017	1	1	0
Guatemala	Panajachel	Proportion	2000	42.1	63.9	19.4
Guatemala	Panajachel	Proportion	2017	45.6	62.9	25.7
Guatemala	Panajachel	Count	2000	0	1	0
Guatemala	Panajachel	Count	2017	0	1	0
Guatemala	Patulul	Proportion	2000	37.7	55.9	19.6
Guatemala	Patulul	Proportion	2017	41.0	58.9	26.1
Guatemala	Patulul	Count	2000	0	1	0
Guatemala	Patulul	Count	2017	0	1	0
Guatemala	San Cristóbal Totonicapán	Proportion	2000	40.2	61.0	17.4
Guatemala	San Cristóbal Totonicapán	Proportion	2017	43.0	60.6	27.7
Guatemala	San Cristóbal Totonicapán	Count	2000	0	1	0
Guatemala	San Cristóbal Totonicapán	Count	2017	0	1	0
Guatemala	Huité	Proportion	2000	42.4	64.1	21.7
Guatemala	Huité	Proportion	2017	48.9	64.6	32.0
Guatemala	Huité	Count	2000	0	1	0
Guatemala	Huité	Count	2017	0	1	0
Guatemala	Lanquín	Proportion	2000	37.8	56.8	19.5
Guatemala	Lanquín	Proportion	2017	42.2	59.6	25.9
Guatemala	Lanquín	Count	2000	0	1	0
Guatemala	Lanquín	Count	2017	0	1	0
Guatemala	Salamá	Proportion	2000	42.7	61.8	21.8
Guatemala	Salamá	Proportion	2017	49.5	65.9	34.1
Guatemala	Salamá	Count	2000	0	1	0
Guatemala	Salamá	Count	2017	0	1	0
Guatemala	Parramos	Proportion	2000	40.8	65.0	20.5
Guatemala	Parramos	Proportion	2017	45.9	63.5	27.1
Guatemala	Parramos	Count	2000	0	1	0
Guatemala	Parramos	Count	2017	0	1	0
Guatemala	Ipala	Proportion	2000	43.8	65.0	22.2
Guatemala	Ipala	Proportion	2017	51.4	68.3	34.9
Guatemala	Ipala	Count	2000	0	1	0
Guatemala	Ipala	Count	2017	1	1	0
Guatemala	San Antonio La Paz	Proportion	2000	44.5	63.6	22.2
Guatemala	San Antonio La Paz	Proportion	2017	52.1	68.7	35.0
Guatemala	San Antonio La Paz	Count	2000	0	1	0
Guatemala	San Antonio La Paz	Count	2017	1	1	0
Guatemala	La Gomera	Proportion	2000	40.1	59.8	21.4
Guatemala	La Gomera	Proportion	2017	43.3	61.8	28.3
Guatemala	La Gomera	Count	2000	0	1	0
Guatemala	La Gomera	Count	2017	0	1	0
Guatemala	Mixco	Proportion	2000	44.5	65.4	22.1
Guatemala	Mixco	Proportion	2017	49.5	67.3	32.3
Guatemala	Mixco	Count	2000	0	1	0

Table 4: ORS by Unit *(continued)*

Country	Unit	Metric	Year	Mean	Upper	Lower
Guatemala	Mixco	Count	2017	0	1	0
Guatemala	Cuilco	Proportion	2000	39.2	60.1	19.3
Guatemala	Cuilco	Proportion	2017	47.5	65.2	30.1
Guatemala	Cuilco	Count	2000	0	1	0
Guatemala	Cuilco	Count	2017	0	1	0
Guatemala	NA	Proportion	2000	38.8	61.0	21.8
Guatemala	NA	Proportion	2017	39.3	55.9	24.2
Guatemala	NA	Count	2000	0	1	0
Guatemala	NA	Count	2017	0	1	0
Guatemala	San Luis Jilotepeque	Proportion	2000	43.1	64.5	23.0
Guatemala	San Luis Jilotepeque	Proportion	2017	51.3	67.8	35.7
Guatemala	San Luis Jilotepeque	Count	2000	0	1	0
Guatemala	San Luis Jilotepeque	Count	2017	1	1	0
Guatemala	Conguaco	Proportion	2000	42.1	65.1	21.8
Guatemala	Conguaco	Proportion	2017	56.7	71.6	40.9
Guatemala	Conguaco	Count	2000	0	1	0
Guatemala	Conguaco	Count	2017	1	1	0
Guatemala	Poptún	Proportion	2000	39.8	60.4	21.2
Guatemala	Poptún	Proportion	2017	39.3	56.7	23.1
Guatemala	Poptún	Count	2000	0	1	0
Guatemala	Poptún	Count	2017	0	1	0
Guatemala	Coatepeque	Proportion	2000	37.5	59.9	18.9
Guatemala	Coatepeque	Proportion	2017	39.4	56.1	25.4
Guatemala	Coatepeque	Count	2000	0	1	0
Guatemala	Coatepeque	Count	2017	0	1	0
Guatemala	Chichicastenango	Proportion	2000	40.1	62.6	18.8
Guatemala	Chichicastenango	Proportion	2017	45.2	61.7	28.8
Guatemala	Chichicastenango	Count	2000	0	1	0
Guatemala	Chichicastenango	Count	2017	0	1	0
Guatemala	San Andrés Villa Seca	Proportion	2000	37.1	58.7	19.2
Guatemala	San Andrés Villa Seca	Proportion	2017	38.9	58.4	23.6
Guatemala	San Andrés Villa Seca	Count	2000	0	1	0
Guatemala	San Andrés Villa Seca	Count	2017	0	1	0
Guatemala	Magdalena Milpas Altas	Proportion	2000	43.2	62.5	22.8
Guatemala	Magdalena Milpas Altas	Proportion	2017	48.5	66.1	32.3
Guatemala	Magdalena Milpas Altas	Count	2000	0	1	0
Guatemala	Magdalena Milpas Altas	Count	2017	0	1	0
Guatemala	El Quetzal	Proportion	2000	36.7	59.6	17.1
Guatemala	El Quetzal	Proportion	2017	39.5	55.8	25.6
Guatemala	El Quetzal	Count	2000	0	1	0
Guatemala	El Quetzal	Count	2017	0	1	0
Guatemala	Guazacapán	Proportion	2000	41.2	61.7	20.1
Guatemala	Guazacapán	Proportion	2017	52.5	69.6	36.2
Guatemala	Guazacapán	Count	2000	0	1	0
Guatemala	Guazacapán	Count	2017	1	1	0
Guatemala	San Andrés Semetabaj	Proportion	2000	39.7	60.8	19.4
Guatemala	San Andrés Semetabaj	Proportion	2017	44.9	61.7	28.1
Guatemala	San Andrés Semetabaj	Count	2000	0	1	0
Guatemala	San Andrés Semetabaj	Count	2017	0	1	0

Table 4: ORS by Unit *(continued)*

Country	Unit	Metric	Year	Mean	Upper	Lower
Guatemala	Pueblo Nuevo	Proportion	2000	36.8	60.4	16.9
Guatemala	Pueblo Nuevo	Proportion	2017	38.7	58.8	23.5
Guatemala	Pueblo Nuevo	Count	2000	0	1	0
Guatemala	Pueblo Nuevo	Count	2017	0	1	0
Guatemala	San Francisco El Alto	Proportion	2000	39.9	62.3	20.0
Guatemala	San Francisco El Alto	Proportion	2017	43.5	60.5	27.1
Guatemala	San Francisco El Alto	Count	2000	0	1	0
Guatemala	San Francisco El Alto	Count	2017	0	1	0
Guatemala	La Unión	Proportion	2000	40.9	64.0	21.7
Guatemala	La Unión	Proportion	2017	45.3	63.3	29.3
Guatemala	La Unión	Count	2000	0	1	0
Guatemala	La Unión	Count	2017	0	1	0
Guatemala	Panzós	Proportion	2000	38.3	58.4	19.2
Guatemala	Panzós	Proportion	2017	41.9	59.3	26.4
Guatemala	Panzós	Count	2000	0	1	0
Guatemala	Panzós	Count	2017	0	1	0
Guatemala	San Jerónimo	Proportion	2000	43.4	62.8	22.2
Guatemala	San Jerónimo	Proportion	2017	50.5	67.1	35.6
Guatemala	San Jerónimo	Count	2000	0	1	0
Guatemala	San Jerónimo	Count	2017	1	1	0
Guatemala	Patzicía	Proportion	2000	40.6	63.2	21.2
Guatemala	Patzicía	Proportion	2017	45.8	62.5	27.5
Guatemala	Patzicía	Count	2000	0	1	0
Guatemala	Patzicía	Count	2017	0	1	0
Guatemala	Jocotán	Proportion	2000	40.8	62.9	21.6
Guatemala	Jocotán	Proportion	2017	46.2	63.1	30.1
Guatemala	Jocotán	Count	2000	0	1	0
Guatemala	Jocotán	Count	2017	0	1	0
Guatemala	San Cristóbal Acasaguastlán	Proportion	2000	42.5	64.9	23.2
Guatemala	San Cristóbal Acasaguastlán	Proportion	2017	49.5	65.6	33.4
Guatemala	San Cristóbal Acasaguastlán	Count	2000	0	1	0
Guatemala	San Cristóbal Acasaguastlán	Count	2017	0	1	0
Guatemala	Masagua	Proportion	2000	40.4	62.3	21.9
Guatemala	Masagua	Proportion	2017	46.8	63.6	30.9
Guatemala	Masagua	Count	2000	0	1	0
Guatemala	Masagua	Count	2017	0	1	0
Guatemala	Palencia	Proportion	2000	43.9	64.0	22.5
Guatemala	Palencia	Proportion	2017	51.4	67.4	35.6
Guatemala	Palencia	Count	2000	0	1	0
Guatemala	Palencia	Count	2017	1	1	0
Guatemala	Huehuetenango	Proportion	2000	41.8	63.8	20.2
Guatemala	Huehuetenango	Proportion	2017	49.3	66.8	34.8
Guatemala	Huehuetenango	Count	2000	0	1	0
Guatemala	Huehuetenango	Count	2017	0	1	0
Guatemala	Puerto Barrios	Proportion	2000	40.1	61.5	23.5

Table 4: ORS by Unit *(continued)*

Country	Unit	Metric	Year	Mean	Upper	Lower
Guatemala	Puerto Barrios	Proportion	2017	40.6	56.9	22.1
Guatemala	Puerto Barrios	Count	2000	0	1	0
Guatemala	Puerto Barrios	Count	2017	0	1	0
Guatemala	San Manuel Chaparrón	Proportion	2000	43.6	66.6	23.2
Guatemala	San Manuel Chaparrón	Proportion	2017	53.4	70.0	37.7
Guatemala	San Manuel Chaparrón	Count	2000	0	1	0
Guatemala	San Manuel Chaparrón	Count	2017	1	1	0
Guatemala	El Adelanto	Proportion	2000	43.3	66.4	22.8
Guatemala	El Adelanto	Proportion	2017	56.7	75.0	38.0
Guatemala	El Adelanto	Count	2000	0	1	0
Guatemala	El Adelanto	Count	2017	1	1	0
Guatemala	San Andrés	Proportion	2000	41.3	61.4	22.0
Guatemala	San Andrés	Proportion	2017	44.3	63.4	27.9
Guatemala	San Andrés	Count	2000	0	1	0
Guatemala	San Andrés	Count	2017	0	1	0
Guatemala	Colomba	Proportion	2000	36.6	57.6	18.1
Guatemala	Colomba	Proportion	2017	39.1	56.2	24.8
Guatemala	Colomba	Count	2000	0	1	0
Guatemala	Colomba	Count	2017	0	1	0
Guatemala	Chinique	Proportion	2000	39.8	59.7	19.9
Guatemala	Chinique	Proportion	2017	45.3	63.4	29.2
Guatemala	Chinique	Count	2000	0	1	0
Guatemala	Chinique	Count	2017	0	1	0
Guatemala	San Felipe	Proportion	2000	36.7	58.4	18.8
Guatemala	San Felipe	Proportion	2017	38.7	59.4	22.8
Guatemala	San Felipe	Count	2000	0	1	0
Guatemala	San Felipe	Count	2017	0	1	0
Guatemala	Pastores	Proportion	2000	41.8	63.1	20.5
Guatemala	Pastores	Proportion	2017	46.5	65.8	28.7
Guatemala	Pastores	Count	2000	0	1	0
Guatemala	Pastores	Count	2017	0	1	0
Guatemala	El Rodeo	Proportion	2000	36.9	60.7	17.5
Guatemala	El Rodeo	Proportion	2017	39.3	55.4	24.4
Guatemala	El Rodeo	Count	2000	0	1	0
Guatemala	El Rodeo	Count	2017	0	1	0
Guatemala	Nueva Santa Rosa	Proportion	2000	42.9	64.7	21.2
Guatemala	Nueva Santa Rosa	Proportion	2017	54.2	70.1	37.9
Guatemala	Nueva Santa Rosa	Count	2000	0	1	0
Guatemala	Nueva Santa Rosa	Count	2017	1	1	0
Guatemala	San Antonio Palopó	Proportion	2000	40.3	62.9	19.7
Guatemala	San Antonio Palopó	Proportion	2017	45.3	64.7	26.5
Guatemala	San Antonio Palopó	Count	2000	0	1	0
Guatemala	San Antonio Palopó	Count	2017	0	1	0
Guatemala	Río Bravo	Proportion	2000	35.7	54.1	17.4
Guatemala	Río Bravo	Proportion	2017	38.4	55.5	22.9
Guatemala	Río Bravo	Count	2000	0	1	0
Guatemala	Río Bravo	Count	2017	0	1	0
Guatemala	Santa Lucía La Reforma	Proportion	2000	39.7	60.5	18.7
Guatemala	Santa Lucía La Reforma	Proportion	2017	46.0	63.4	29.5

Table 4: ORS by Unit *(continued)*

Country	Unit	Metric	Year	Mean	Upper	Lower
Guatemala	Santa Lucía La Reforma	Count	2000	0	1	0
Guatemala	Santa Lucía La Reforma	Count	2017	0	1	0
Guatemala	Río Hondo	Proportion	2000	39.2	62.9	20.9
Guatemala	Río Hondo	Proportion	2017	43.7	60.7	28.1
Guatemala	Río Hondo	Count	2000	0	1	0
Guatemala	Río Hondo	Count	2017	0	1	0
Guatemala	San Cristóbal Verapaz	Proportion	2000	40.8	62.4	21.4
Guatemala	San Cristóbal Verapaz	Proportion	2017	47.3	65.1	31.0
Guatemala	San Cristóbal Verapaz	Count	2000	0	1	0
Guatemala	San Cristóbal Verapaz	Count	2017	0	1	0
Guatemala	San Miguel Chicaj	Proportion	2000	43.5	63.4	22.5
Guatemala	San Miguel Chicaj	Proportion	2017	50.3	66.7	34.8
Guatemala	San Miguel Chicaj	Count	2000	0	1	0
Guatemala	San Miguel Chicaj	Count	2017	1	1	0
Guatemala	Patzún	Proportion	2000	40.2	61.6	19.1
Guatemala	Patzún	Proportion	2017	45.7	62.2	29.3
Guatemala	Patzún	Count	2000	0	1	0
Guatemala	Patzún	Count	2017	0	1	0
Guatemala	Olopa	Proportion	2000	41.3	63.1	22.5
Guatemala	Olopa	Proportion	2017	46.9	65.4	29.3
Guatemala	Olopa	Count	2000	0	1	0
Guatemala	Olopa	Count	2017	0	1	0
Guatemala	Sanarate	Proportion	2000	45.8	65.7	24.5
Guatemala	Sanarate	Proportion	2017	52.5	68.6	36.6
Guatemala	Sanarate	Count	2000	0	1	0
Guatemala	Sanarate	Count	2017	1	1	0
Guatemala	Nueva Concepción	Proportion	2000	38.9	59.8	21.5
Guatemala	Nueva Concepción	Proportion	2017	40.8	58.3	25.1
Guatemala	Nueva Concepción	Count	2000	0	1	0
Guatemala	Nueva Concepción	Count	2017	0	1	0
Guatemala	Petapa	Proportion	2000	43.9	67.1	22.4
Guatemala	Petapa	Proportion	2017	49.8	69.6	33.0
Guatemala	Petapa	Count	2000	0	1	0
Guatemala	Petapa	Count	2017	0	1	0
Guatemala	Jacaltenango	Proportion	2000	40.4	64.1	22.0
Guatemala	Jacaltenango	Proportion	2017	50.7	67.2	34.0
Guatemala	Jacaltenango	Count	2000	0	1	0
Guatemala	Jacaltenango	Count	2017	1	1	0
Guatemala	San Luis	Proportion	2000	39.4	63.8	22.3
Guatemala	San Luis	Proportion	2017	40.0	57.6	23.6
Guatemala	San Luis	Count	2000	0	1	0
Guatemala	San Luis	Count	2017	0	1	0
Guatemala	San Pedro Pinula	Proportion	2000	42.1	63.1	21.9
Guatemala	San Pedro Pinula	Proportion	2017	52.0	68.4	36.5
Guatemala	San Pedro Pinula	Count	2000	0	1	0
Guatemala	San Pedro Pinula	Count	2017	1	1	0
Guatemala	El Progreso	Proportion	2000	43.3	66.1	22.3
Guatemala	El Progreso	Proportion	2017	54.8	71.0	38.2
Guatemala	El Progreso	Count	2000	0	1	0

Table 4: ORS by Unit *(continued)*

Country	Unit	Metric	Year	Mean	Upper	Lower
Guatemala	El Progreso	Count	2017	1	1	0
Guatemala	San Benito	Proportion	2000	41.1	60.9	24.1
Guatemala	San Benito	Proportion	2017	42.0	60.6	25.2
Guatemala	San Benito	Count	2000	0	1	0
Guatemala	San Benito	Count	2017	0	1	0
Guatemala	Concepción Chiquirichapa	Proportion	2000	39.0	63.5	18.3
Guatemala	Concepción Chiquirichapa	Proportion	2017	42.2	62.4	26.5
Guatemala	Concepción Chiquirichapa	Count	2000	0	1	0
Guatemala	Concepción Chiquirichapa	Count	2017	0	1	0
Guatemala	Cunén	Proportion	2000	37.5	59.3	18.4
Guatemala	Cunén	Proportion	2017	45.0	61.5	29.6
Guatemala	Cunén	Count	2000	0	1	0
Guatemala	Cunén	Count	2017	0	1	0
Guatemala	San Martín Zapotitlán	Proportion	2000	36.8	60.0	17.4
Guatemala	San Martín Zapotitlán	Proportion	2017	38.5	60.1	19.9
Guatemala	San Martín Zapotitlán	Count	2000	0	1	0
Guatemala	San Martín Zapotitlán	Count	2017	0	1	0
Guatemala	San Antonio Aguas Calientes	Proportion	2000	42.0	64.4	19.6
Guatemala	San Antonio Aguas Calientes	Proportion	2017	46.3	66.7	28.4
Guatemala	San Antonio Aguas Calientes	Count	2000	0	1	0
Guatemala	San Antonio Aguas Calientes	Count	2017	0	1	0
Guatemala	El Tumbador	Proportion	2000	36.9	61.0	17.0
Guatemala	El Tumbador	Proportion	2017	39.9	56.9	25.2
Guatemala	El Tumbador	Count	2000	0	1	0
Guatemala	El Tumbador	Count	2017	0	1	0
Guatemala	Oratorio	Proportion	2000	42.5	63.2	21.9
Guatemala	Oratorio	Proportion	2017	55.9	70.7	42.2
Guatemala	Oratorio	Count	2000	0	1	0
Guatemala	Oratorio	Count	2017	1	1	0
Guatemala	San José Chacayá	Proportion	2000	39.6	63.2	18.7
Guatemala	San José Chacayá	Proportion	2017	43.0	62.2	24.2
Guatemala	San José Chacayá	Count	2000	0	1	0
Guatemala	San José Chacayá	Count	2017	0	1	0
Guatemala	Samayac	Proportion	2000	37.2	58.6	17.9
Guatemala	Samayac	Proportion	2017	39.3	58.4	24.1
Guatemala	Samayac	Count	2000	0	1	0
Guatemala	Samayac	Count	2017	0	1	0
Guatemala	Santa María Chiquimula	Proportion	2000	39.0	61.1	17.4
Guatemala	Santa María Chiquimula	Proportion	2017	44.4	61.1	29.4
Guatemala	Santa María Chiquimula	Count	2000	0	1	0
Guatemala	Santa María Chiquimula	Count	2017	0	1	0
Guatemala	San Diego	Proportion	2000	42.6	64.3	21.7
Guatemala	San Diego	Proportion	2017	50.9	68.1	34.5
Guatemala	San Diego	Count	2000	0	1	0
Guatemala	San Diego	Count	2017	1	1	0

Table 4: ORS by Unit *(continued)*

Country	Unit	Metric	Year	Mean	Upper	Lower
Guatemala	San Juan Chamelco	Proportion	2000	39.7	60.1	20.9
Guatemala	San Juan Chamelco	Proportion	2017	45.7	63.6	28.2
Guatemala	San Juan Chamelco	Count	2000	0	1	0
Guatemala	San Juan Chamelco	Count	2017	0	1	0
Guatemala	Santa Cruz El Chol	Proportion	2000	43.1	63.4	22.6
Guatemala	Santa Cruz El Chol	Proportion	2017	49.7	66.7	33.3
Guatemala	Santa Cruz El Chol	Count	2000	0	1	0
Guatemala	Santa Cruz El Chol	Count	2017	0	1	0
Guatemala	Pochuta	Proportion	2000	38.7	60.5	18.6
Guatemala	Pochuta	Proportion	2017	43.0	60.9	28.1
Guatemala	Pochuta	Count	2000	0	1	0
Guatemala	Pochuta	Count	2017	0	1	0
Guatemala	Quezaltepeque	Proportion	2000	42.3	63.6	23.7
Guatemala	Quezaltepeque	Proportion	2017	49.5	68.9	31.6
Guatemala	Quezaltepeque	Count	2000	0	1	0
Guatemala	Quezaltepeque	Count	2017	0	1	0
Guatemala	Sansare	Proportion	2000	44.9	65.7	22.0
Guatemala	Sansare	Proportion	2017	53.5	69.6	36.9
Guatemala	Sansare	Count	2000	0	1	0
Guatemala	Sansare	Count	2017	1	1	0
Guatemala	Palín	Proportion	2000	41.8	63.5	20.2
Guatemala	Palín	Proportion	2017	48.0	67.5	30.8
Guatemala	Palín	Count	2000	0	1	0
Guatemala	Palín	Count	2017	0	1	0
Guatemala	San José del Golfo	Proportion	2000	44.5	64.4	24.5
Guatemala	San José del Golfo	Proportion	2017	50.6	67.5	34.2
Guatemala	San José del Golfo	Count	2000	0	1	0
Guatemala	San José del Golfo	Count	2017	1	1	0
Guatemala	La Democracia	Proportion	2000	40.2	63.1	21.1
Guatemala	La Democracia	Proportion	2017	50.2	68.2	32.7
Guatemala	La Democracia	Count	2000	0	1	0
Guatemala	La Democracia	Count	2017	1	1	0
Guatemala	Jalpatagua	Proportion	2000	42.5	65.1	22.2
Guatemala	Jalpatagua	Proportion	2017	56.6	72.3	41.8
Guatemala	Jalpatagua	Count	2000	0	1	0
Guatemala	Jalpatagua	Count	2017	1	1	0
Guatemala	San Francisco	Proportion	2000	41.0	61.3	22.0
Guatemala	San Francisco	Proportion	2017	42.6	61.2	25.7
Guatemala	San Francisco	Count	2000	0	1	0
Guatemala	San Francisco	Count	2017	0	1	0
Guatemala	El Palmar	Proportion	2000	36.4	59.1	18.1
Guatemala	El Palmar	Proportion	2017	39.4	57.6	24.9
Guatemala	El Palmar	Count	2000	0	1	0
Guatemala	El Palmar	Count	2017	0	1	0
Guatemala	Ixcán	Proportion	2000	36.3	56.6	17.9
Guatemala	Ixcán	Proportion	2017	42.9	59.3	27.2
Guatemala	Ixcán	Count	2000	0	1	0
Guatemala	Ixcán	Count	2017	0	1	0
Guatemala	San Sebastián	Proportion	2000	36.3	60.9	14.5

Table 4: ORS by Unit *(continued)*

Country	Unit	Metric	Year	Mean	Upper	Lower
Guatemala	San Sebastián	Proportion	2017	38.2	57.4	22.2
Guatemala	San Sebastián	Count	2000	0	1	0
Guatemala	San Sebastián	Count	2017	0	1	0
Guatemala	San Bartolomé Milpas Altas	Proportion	2000	41.5	63.4	20.0
Guatemala	San Bartolomé Milpas Altas	Proportion	2017	47.5	68.5	28.7
Guatemala	San Bartolomé Milpas Altas	Count	2000	0	1	0
Guatemala	San Bartolomé Milpas Altas	Count	2017	0	1	0
Guatemala	Esquipulas Palo Gordo	Proportion	2000	37.4	59.4	17.3
Guatemala	Esquipulas Palo Gordo	Proportion	2017	40.6	59.9	24.3
Guatemala	Esquipulas Palo Gordo	Count	2000	0	1	0
Guatemala	Esquipulas Palo Gordo	Count	2017	0	1	0
Guatemala	Pueblo Nuevo Viñas	Proportion	2000	41.6	61.0	21.8
Guatemala	Pueblo Nuevo Viñas	Proportion	2017	52.4	68.1	36.7
Guatemala	Pueblo Nuevo Viñas	Count	2000	0	1	0
Guatemala	Pueblo Nuevo Viñas	Count	2017	1	1	0
Guatemala	San Juan La Laguna	Proportion	2000	39.4	62.7	19.6
Guatemala	San Juan La Laguna	Proportion	2017	42.9	61.7	26.7
Guatemala	San Juan La Laguna	Count	2000	0	1	0
Guatemala	San Juan La Laguna	Count	2017	0	1	0
Guatemala	San Antonio Suchitepéquez	Proportion	2000	36.5	60.3	17.9
Guatemala	San Antonio Suchitepéquez	Proportion	2017	38.2	57.4	23.9
Guatemala	San Antonio Suchitepéquez	Count	2000	0	1	0
Guatemala	San Antonio Suchitepéquez	Count	2017	0	1	0
Guatemala	Totonicapán	Proportion	2000	40.1	63.4	19.7
Guatemala	Totonicapán	Proportion	2017	43.3	59.6	29.0
Guatemala	Totonicapán	Count	2000	0	1	0
Guatemala	Totonicapán	Count	2017	0	1	0
Guatemala	Teculután	Proportion	2000	41.0	65.9	20.4
Guatemala	Teculután	Proportion	2017	46.2	64.5	29.5
Guatemala	Teculután	Count	2000	0	1	0
Guatemala	Teculután	Count	2017	0	1	0
Guatemala	San Pedro Carchá	Proportion	2000	39.3	59.5	20.3
Guatemala	San Pedro Carchá	Proportion	2017	44.0	61.4	27.4
Guatemala	San Pedro Carchá	Count	2000	0	1	0
Guatemala	San Pedro Carchá	Count	2017	0	1	0
Guatemala	San Andrés Itzapa	Proportion	2000	41.5	66.4	20.2
Guatemala	San Andrés Itzapa	Proportion	2017	45.8	63.6	29.8
Guatemala	San Andrés Itzapa	Count	2000	0	1	0
Guatemala	San Andrés Itzapa	Count	2017	0	1	0
Guatemala	San Jacinto	Proportion	2000	42.2	64.8	23.1
Guatemala	San Jacinto	Proportion	2017	48.7	67.4	30.9
Guatemala	San Jacinto	Count	2000	0	1	0
Guatemala	San Jacinto	Count	2017	0	1	0
Guatemala	San José	Proportion	2000	42.1	62.7	22.1
Guatemala	San José	Proportion	2017	48.2	66.4	30.9
Guatemala	San José	Count	2000	0	1	0

Table 4: ORS by Unit *(continued)*

Country	Unit	Metric	Year	Mean	Upper	Lower
Guatemala	San José	Count	2017	0	1	0
Guatemala	San José Pinula	Proportion	2000	42.2	63.2	21.3
Guatemala	San José Pinula	Proportion	2017	51.3	67.3	34.0
Guatemala	San José Pinula	Count	2000	0	1	0
Guatemala	San José Pinula	Count	2017	1	1	0
Guatemala	La Libertad	Proportion	2000	40.0	62.1	19.9
Guatemala	La Libertad	Proportion	2017	49.4	67.6	31.4
Guatemala	La Libertad	Count	2000	0	1	0
Guatemala	La Libertad	Count	2017	0	1	0
Guatemala	Jerez	Proportion	2000	45.7	65.4	26.7
Guatemala	Jerez	Proportion	2017	59.4	74.9	42.4
Guatemala	Jerez	Count	2000	0	1	0
Guatemala	Jerez	Count	2017	1	1	0
Guatemala	San José	Proportion	2000	41.1	61.7	24.6
Guatemala	San José	Proportion	2017	42.3	61.1	27.2
Guatemala	San José	Count	2000	0	1	0
Guatemala	San José	Count	2017	0	1	0
Guatemala	Flores Costa Cuca	Proportion	2000	37.0	62.9	18.5
Guatemala	Flores Costa Cuca	Proportion	2017	38.6	56.1	23.6
Guatemala	Flores Costa Cuca	Count	2000	0	1	0
Guatemala	Flores Costa Cuca	Count	2017	0	1	0
Guatemala	Joyabaj	Proportion	2000	40.7	62.1	21.8
Guatemala	Joyabaj	Proportion	2017	46.7	63.0	29.7
Guatemala	Joyabaj	Count	2000	0	1	0
Guatemala	Joyabaj	Count	2017	0	1	0
Guatemala	Santa Cruz Muluá	Proportion	2000	36.4	58.3	18.2
Guatemala	Santa Cruz Muluá	Proportion	2017	38.0	56.1	22.6
Guatemala	Santa Cruz Muluá	Count	2000	0	1	0
Guatemala	Santa Cruz Muluá	Count	2017	0	1	0
Guatemala	San Lucas Sacatepéquez	Proportion	2000	43.6	65.9	19.8
Guatemala	San Lucas Sacatepéquez	Proportion	2017	48.3	66.2	30.2
Guatemala	San Lucas Sacatepéquez	Count	2000	0	1	0
Guatemala	San Lucas Sacatepéquez	Count	2017	0	1	0
Guatemala	Ixchiguan	Proportion	2000	38.5	59.0	19.3
Guatemala	Ixchiguan	Proportion	2017	43.0	60.8	28.0
Guatemala	Ixchiguan	Count	2000	0	1	0
Guatemala	Ixchiguan	Count	2017	0	1	0
Guatemala	San Juan Tecuaco	Proportion	2000	40.3	62.3	20.9
Guatemala	San Juan Tecuaco	Proportion	2017	53.7	70.2	37.7
Guatemala	San Juan Tecuaco	Count	2000	0	1	0
Guatemala	San Juan Tecuaco	Count	2017	1	1	0
Guatemala	San Lucas Tolimán	Proportion	2000	39.8	61.0	20.0
Guatemala	San Lucas Tolimán	Proportion	2017	44.3	62.8	28.4
Guatemala	San Lucas Tolimán	Count	2000	0	1	0
Guatemala	San Lucas Tolimán	Count	2017	0	1	0
Guatemala	San Bernardino	Proportion	2000	37.0	58.9	17.7
Guatemala	San Bernardino	Proportion	2017	38.4	55.8	23.4
Guatemala	San Bernardino	Count	2000	0	1	0
Guatemala	San Bernardino	Count	2017	0	1	0

Table 4: ORS by Unit *(continued)*

Country	Unit	Metric	Year	Mean	Upper	Lower
Guatemala	Usumatlán	Proportion	2000	42.9	66.3	22.0
Guatemala	Usumatlán	Proportion	2017	48.7	67.2	32.3
Guatemala	Usumatlán	Count	2000	0	1	0
Guatemala	Usumatlán	Count	2017	0	1	0
Guatemala	Santa Cruz Verapaz	Proportion	2000	41.3	61.8	21.8
Guatemala	Santa Cruz Verapaz	Proportion	2017	47.3	65.2	30.3
Guatemala	Santa Cruz Verapaz	Count	2000	0	1	0
Guatemala	Santa Cruz Verapaz	Count	2017	0	1	0
Guatemala	San José Poaquil	Proportion	2000	40.4	62.4	20.7
Guatemala	San José Poaquil	Proportion	2017	46.1	62.0	29.3
Guatemala	San José Poaquil	Count	2000	0	1	0
Guatemala	San José Poaquil	Count	2017	0	1	0
Guatemala	San José La Arada	Proportion	2000	41.8	65.2	22.6
Guatemala	San José La Arada	Proportion	2017	48.3	67.1	30.1
Guatemala	San José La Arada	Count	2000	0	1	0
Guatemala	San José La Arada	Count	2017	0	1	0
Guatemala	San Vicente Pacaya	Proportion	2000	40.7	62.6	20.7
Guatemala	San Vicente Pacaya	Proportion	2017	48.8	65.1	32.3
Guatemala	San Vicente Pacaya	Count	2000	0	1	0
Guatemala	San Vicente Pacaya	Count	2017	0	1	0
Guatemala	San Juan Sacatepéquez	Proportion	2000	43.2	64.1	21.2
Guatemala	San Juan Sacatepéquez	Proportion	2017	48.1	65.0	31.0
Guatemala	San Juan Sacatepéquez	Count	2000	0	1	0
Guatemala	San Juan Sacatepéquez	Count	2017	0	1	0
Guatemala	Malacatancito	Proportion	2000	41.5	64.5	20.2
Guatemala	Malacatancito	Proportion	2017	48.6	63.9	33.2
Guatemala	Malacatancito	Count	2000	0	1	0
Guatemala	Malacatancito	Count	2017	0	1	0
Guatemala	Jutiapa	Proportion	2000	43.1	65.3	23.9
Guatemala	Jutiapa	Proportion	2017	55.3	71.8	39.1
Guatemala	Jutiapa	Count	2000	0	1	0
Guatemala	Jutiapa	Count	2017	1	1	0
Guatemala	San Luis	Proportion	2000	38.3	59.2	20.8
Guatemala	San Luis	Proportion	2017	38.2	54.6	22.4
Guatemala	San Luis	Count	2000	0	1	0
Guatemala	San Luis	Count	2017	0	1	0
Guatemala	Génova	Proportion	2000	36.5	59.8	18.6
Guatemala	Génova	Proportion	2017	38.6	56.2	24.6
Guatemala	Génova	Count	2000	0	1	0
Guatemala	Génova	Count	2017	0	1	0
Guatemala	Nebaj	Proportion	2000	38.0	60.3	18.6
Guatemala	Nebaj	Proportion	2017	46.8	64.7	31.7
Guatemala	Nebaj	Count	2000	0	1	0
Guatemala	Nebaj	Count	2017	0	1	0
Guatemala	San Miguel Dueñas	Proportion	2000	41.7	62.6	22.6
Guatemala	San Miguel Dueñas	Proportion	2017	47.0	63.8	30.5
Guatemala	San Miguel Dueñas	Count	2000	0	1	0
Guatemala	San Miguel Dueñas	Count	2017	0	1	0
Guatemala	La Reforma	Proportion	2000	36.2	60.4	16.9

Table 4: ORS by Unit *(continued)*

Country	Unit	Metric	Year	Mean	Upper	Lower
Guatemala	La Reforma	Proportion	2017	39.5	55.6	26.2
Guatemala	La Reforma	Count	2000	0	1	0
Guatemala	La Reforma	Count	2017	0	1	0
Guatemala	San Rafaél Las Flores	Proportion	2000	40.8	62.8	21.0
Guatemala	San Rafaél Las Flores	Proportion	2017	52.6	69.1	37.0
Guatemala	San Rafaél Las Flores	Count	2000	0	1	0
Guatemala	San Rafaél Las Flores	Count	2017	1	1	0
Guatemala	San Marcos La Laguna	Proportion	2000	38.9	62.8	17.0
Guatemala	San Marcos La Laguna	Proportion	2017	43.3	63.3	25.3
Guatemala	San Marcos La Laguna	Count	2000	0	1	0
Guatemala	San Marcos La Laguna	Count	2017	0	1	0
Guatemala	San Francisco Zapotitlán	Proportion	2000	37.1	57.7	16.8
Guatemala	San Francisco Zapotitlán	Proportion	2017	38.7	58.2	22.7
Guatemala	San Francisco Zapotitlán	Count	2000	0	1	0
Guatemala	San Francisco Zapotitlán	Count	2017	0	1	0
Guatemala	Zacapa	Proportion	2000	42.3	66.1	22.3
Guatemala	Zacapa	Proportion	2017	46.8	63.1	28.8
Guatemala	Zacapa	Count	2000	0	1	0
Guatemala	Zacapa	Count	2017	0	1	0
Guatemala	Santa María Cahabón	Proportion	2000	37.2	56.5	19.3
Guatemala	Santa María Cahabón	Proportion	2017	40.0	57.2	24.8
Guatemala	Santa María Cahabón	Count	2000	0	1	0
Guatemala	Santa María Cahabón	Count	2017	0	1	0
Guatemala	San Martín Jilotepeque	Proportion	2000	41.2	61.3	20.8
Guatemala	San Martín Jilotepeque	Proportion	2017	46.8	63.2	29.1
Guatemala	San Martín Jilotepeque	Count	2000	0	1	0
Guatemala	San Martín Jilotepeque	Count	2017	0	1	0
Guatemala	San Juan Ermita	Proportion	2000	41.2	63.4	21.3
Guatemala	San Juan Ermita	Proportion	2017	47.3	65.6	29.7
Guatemala	San Juan Ermita	Count	2000	0	1	0
Guatemala	San Juan Ermita	Count	2017	0	1	0
Guatemala	Santa Lucía Cotzumalguapa	Proportion	2000	38.2	59.7	18.7
Guatemala	Santa Lucía Cotzumalguapa	Proportion	2017	41.8	60.6	25.8
Guatemala	Santa Lucía Cotzumalguapa	Count	2000	0	1	0
Guatemala	Santa Lucía Cotzumalguapa	Count	2017	0	1	0
Guatemala	San Pedro Ayampuc	Proportion	2000	44.3	64.0	21.9
Guatemala	San Pedro Ayampuc	Proportion	2017	49.7	66.6	32.0
Guatemala	San Pedro Ayampuc	Count	2000	0	1	0
Guatemala	San Pedro Ayampuc	Count	2017	0	1	0
Guatemala	Nentón	Proportion	2000	38.7	62.0	20.7
Guatemala	Nentón	Proportion	2017	49.5	67.8	33.0
Guatemala	Nentón	Count	2000	0	1	0
Guatemala	Nentón	Count	2017	0	1	0
Guatemala	Moyuta	Proportion	2000	42.2	64.3	23.1
Guatemala	Moyuta	Proportion	2017	56.1	70.7	40.8
Guatemala	Moyuta	Count	2000	0	1	0

Table 4: ORS by Unit *(continued)*

Country	Unit	Metric	Year	Mean	Upper	Lower
Guatemala	Moyuta	Count	2017	1	1	0
Guatemala	Santa Ana	Proportion	2000	40.7	61.7	22.8
Guatemala	Santa Ana	Proportion	2017	41.2	59.6	25.8
Guatemala	Santa Ana	Count	2000	0	1	0
Guatemala	Santa Ana	Count	2017	0	1	0
Guatemala	Huitán	Proportion	2000	38.6	61.1	18.0
Guatemala	Huitán	Proportion	2017	43.9	62.7	27.8
Guatemala	Huitán	Count	2000	0	1	0
Guatemala	Huitán	Count	2017	0	1	0
Guatemala	Pachalúm	Proportion	2000	42.2	61.7	22.3
Guatemala	Pachalúm	Proportion	2017	47.5	65.0	29.1
Guatemala	Pachalúm	Count	2000	0	1	0
Guatemala	Pachalúm	Count	2017	0	1	0
Guatemala	Santa Catarina Barahona	Proportion	2000	40.3	66.2	19.4
Guatemala	Santa Catarina Barahona	Proportion	2017	45.7	64.5	27.6
Guatemala	Santa Catarina Barahona	Count	2000	0	1	0
Guatemala	Santa Catarina Barahona	Count	2017	0	1	0
Guatemala	Malacatán	Proportion	2000	37.1	58.7	17.8
Guatemala	Malacatán	Proportion	2017	39.2	55.0	24.5
Guatemala	Malacatán	Count	2000	0	1	0
Guatemala	Malacatán	Count	2017	0	1	0
Guatemala	Santa Cruz Naranjo	Proportion	2000	42.5	64.8	19.7
Guatemala	Santa Cruz Naranjo	Proportion	2017	53.3	70.8	37.3
Guatemala	Santa Cruz Naranjo	Count	2000	0	1	0
Guatemala	Santa Cruz Naranjo	Count	2017	1	1	0
Guatemala	San Pablo La Laguna	Proportion	2000	39.0	64.2	16.9
Guatemala	San Pablo La Laguna	Proportion	2017	43.3	64.0	23.7
Guatemala	San Pablo La Laguna	Count	2000	0	1	0
Guatemala	San Pablo La Laguna	Count	2017	0	1	0
Guatemala	San Gabriel	Proportion	2000	37.1	62.8	17.3
Guatemala	San Gabriel	Proportion	2017	37.4	56.8	21.9
Guatemala	San Gabriel	Count	2000	0	1	0
Guatemala	San Gabriel	Count	2017	0	1	0
Guatemala	Senahú	Proportion	2000	37.7	57.1	18.7
Guatemala	Senahú	Proportion	2017	41.4	57.9	25.6
Guatemala	Senahú	Count	2000	0	1	0
Guatemala	Senahú	Count	2017	0	1	0
Guatemala	Santa Apolonia	Proportion	2000	39.9	61.1	18.5
Guatemala	Santa Apolonia	Proportion	2017	45.3	62.8	28.9
Guatemala	Santa Apolonia	Count	2000	0	1	0
Guatemala	Santa Apolonia	Count	2017	0	1	0
Guatemala	Siquinalá	Proportion	2000	38.0	57.6	18.8
Guatemala	Siquinalá	Proportion	2017	42.4	60.8	25.9
Guatemala	Siquinalá	Count	2000	0	1	0
Guatemala	Siquinalá	Count	2017	0	1	0
Guatemala	San Pedro Sacatepéquez	Proportion	2000	43.4	64.2	21.3
Guatemala	San Pedro Sacatepéquez	Proportion	2017	48.3	65.9	30.0
Guatemala	San Pedro Sacatepéquez	Count	2000	0	1	0
Guatemala	San Pedro Sacatepéquez	Count	2017	0	1	0

Table 4: ORS by Unit *(continued)*

Country	Unit	Metric	Year	Mean	Upper	Lower
Guatemala	San Antonio Huista	Proportion	2000	40.3	63.2	20.1
Guatemala	San Antonio Huista	Proportion	2017	50.3	68.2	32.6
Guatemala	San Antonio Huista	Count	2000	0	1	0
Guatemala	San Antonio Huista	Count	2017	1	1	0
Guatemala	Pasaco	Proportion	2000	41.1	61.9	20.8
Guatemala	Pasaco	Proportion	2017	54.4	70.6	39.4
Guatemala	Pasaco	Count	2000	0	1	0
Guatemala	Pasaco	Count	2017	1	1	0
Guatemala	Sayaxché	Proportion	2000	39.6	59.6	21.6
Guatemala	Sayaxché	Proportion	2017	42.8	60.3	27.6
Guatemala	Sayaxché	Count	2000	0	1	0
Guatemala	Sayaxché	Count	2017	0	1	0
Guatemala	La Esperanza	Proportion	2000	39.5	63.4	18.2
Guatemala	La Esperanza	Proportion	2017	41.9	63.2	25.2
Guatemala	La Esperanza	Count	2000	0	1	0
Guatemala	La Esperanza	Count	2017	0	1	0
Guatemala	Patzité	Proportion	2000	39.5	62.5	18.6
Guatemala	Patzité	Proportion	2017	45.1	62.7	28.1
Guatemala	Patzité	Count	2000	0	1	0
Guatemala	Patzité	Count	2017	0	1	0
Guatemala	Santa Lucía Milpas Altas	Proportion	2000	43.6	64.8	23.4
Guatemala	Santa Lucía Milpas Altas	Proportion	2017	48.6	64.9	29.8
Guatemala	Santa Lucía Milpas Altas	Count	2000	0	1	0
Guatemala	Santa Lucía Milpas Altas	Count	2017	0	1	0
Guatemala	Nuevo Progreso	Proportion	2000	36.6	59.2	16.8
Guatemala	Nuevo Progreso	Proportion	2017	39.5	55.1	25.7
Guatemala	Nuevo Progreso	Count	2000	0	1	0
Guatemala	Nuevo Progreso	Count	2017	0	1	0
Guatemala	Santa María Ixhuatán	Proportion	2000	41.8	64.2	21.4
Guatemala	Santa María Ixhuatán	Proportion	2017	54.5	69.1	38.2
Guatemala	Santa María Ixhuatán	Count	2000	0	1	0
Guatemala	Santa María Ixhuatán	Count	2017	1	1	0
Guatemala	San Pedro La Laguna	Proportion	2000	39.1	61.4	18.8
Guatemala	San Pedro La Laguna	Proportion	2017	43.0	61.6	28.7
Guatemala	San Pedro La Laguna	Count	2000	0	1	0
Guatemala	San Pedro La Laguna	Count	2017	0	1	0
Guatemala	San José El Idolo	Proportion	2000	35.9	59.2	16.8
Guatemala	San José El Idolo	Proportion	2017	38.0	58.3	23.3
Guatemala	San José El Idolo	Count	2000	0	1	0
Guatemala	San José El Idolo	Count	2017	0	1	0
Guatemala	Tactic	Proportion	2000	40.8	59.9	21.4
Guatemala	Tactic	Proportion	2017	47.4	65.0	30.9
Guatemala	Tactic	Count	2000	0	1	0
Guatemala	Tactic	Count	2017	0	1	0
Guatemala	Santa Cruz Balanyá	Proportion	2000	39.5	62.3	20.1
Guatemala	Santa Cruz Balanyá	Proportion	2017	46.1	63.8	28.7
Guatemala	Santa Cruz Balanyá	Count	2000	0	1	0
Guatemala	Santa Cruz Balanyá	Count	2017	0	1	0
Guatemala	Tiquisate	Proportion	2000	38.2	58.3	20.1

Table 4: ORS by Unit *(continued)*

Country	Unit	Metric	Year	Mean	Upper	Lower
Guatemala	Tiquisate	Proportion	2017	39.8	58.2	24.3
Guatemala	Tiquisate	Count	2000	0	1	0
Guatemala	Tiquisate	Count	2017	0	1	0
Guatemala	San Raymundo	Proportion	2000	43.1	63.7	21.6
Guatemala	San Raymundo	Proportion	2017	48.8	66.9	32.2
Guatemala	San Raymundo	Count	2000	0	1	0
Guatemala	San Raymundo	Count	2017	0	1	0
Guatemala	San Gaspar Ixchil	Proportion	2000	40.5	63.6	19.6
Guatemala	San Gaspar Ixchil	Proportion	2017	49.0	67.2	31.7
Guatemala	San Gaspar Ixchil	Count	2000	0	1	0
Guatemala	San Gaspar Ixchil	Count	2017	0	1	0
Guatemala	Quezada	Proportion	2000	42.8	64.6	22.2
Guatemala	Quezada	Proportion	2017	55.6	72.2	40.3
Guatemala	Quezada	Count	2000	0	1	0
Guatemala	Quezada	Count	2017	1	1	0
Guatemala	Olintepeque	Proportion	2000	39.5	62.6	17.7
Guatemala	Olintepeque	Proportion	2017	42.0	58.8	27.5
Guatemala	Olintepeque	Count	2000	0	1	0
Guatemala	Olintepeque	Count	2017	0	1	0
Guatemala	Sacapulas	Proportion	2000	39.8	61.3	20.9
Guatemala	Sacapulas	Proportion	2017	47.6	63.5	31.3
Guatemala	Sacapulas	Count	2000	0	1	0
Guatemala	Sacapulas	Count	2017	0	1	0
Guatemala	Santa María de Jesús	Proportion	2000	41.7	61.8	21.7
Guatemala	Santa María de Jesús	Proportion	2017	48.3	65.9	32.3
Guatemala	Santa María de Jesús	Count	2000	0	1	0
Guatemala	Santa María de Jesús	Count	2017	0	1	0
Guatemala	Ocos	Proportion	2000	39.3	61.8	19.9
Guatemala	Ocos	Proportion	2017	41.4	58.8	25.5
Guatemala	Ocos	Count	2000	0	1	0
Guatemala	Ocos	Count	2017	0	1	0
Guatemala	Santa Rosa de Lima	Proportion	2000	41.6	62.4	19.7
Guatemala	Santa Rosa de Lima	Proportion	2017	52.4	69.0	36.1
Guatemala	Santa Rosa de Lima	Count	2000	0	1	0
Guatemala	Santa Rosa de Lima	Count	2017	1	1	0
Guatemala	Santa Catarina Ixtahuacan	Proportion	2000	38.4	60.6	19.6
Guatemala	Santa Catarina Ixtahuacan	Proportion	2017	41.8	58.2	27.0
Guatemala	Santa Catarina Ixtahuacan	Count	2000	0	1	0
Guatemala	Santa Catarina Ixtahuacan	Count	2017	0	1	0
Guatemala	San Juan Bautista	Proportion	2000	37.0	58.7	19.5
Guatemala	San Juan Bautista	Proportion	2017	40.3	58.5	24.4
Guatemala	San Juan Bautista	Count	2000	0	1	0
Guatemala	San Juan Bautista	Count	2017	0	1	0
Guatemala	Tamahú	Proportion	2000	39.2	59.1	20.2
Guatemala	Tamahú	Proportion	2017	46.0	63.7	28.4
Guatemala	Tamahú	Count	2000	0	1	0
Guatemala	Tamahú	Count	2017	0	1	0
Guatemala	Tecpán Guatemala	Proportion	2000	39.9	62.0	20.4
Guatemala	Tecpán Guatemala	Proportion	2017	45.1	60.9	28.5

Table 4: ORS by Unit *(continued)*

Country	Unit	Metric	Year	Mean	Upper	Lower
Guatemala	Tecpán Guatemala	Count	2000	0	1	0
Guatemala	Tecpán Guatemala	Count	2017	0	1	0
Guatemala	Santa Catarina Pinula	Proportion	2000	43.5	66.9	21.8
Guatemala	Santa Catarina Pinula	Proportion	2017	50.0	66.8	32.2
Guatemala	Santa Catarina Pinula	Count	2000	0	1	0
Guatemala	Santa Catarina Pinula	Count	2017	0	1	0
Guatemala	San Ildefonso Ixtahuacán	Proportion	2000	39.8	61.4	19.5
Guatemala	San Ildefonso Ixtahuacán	Proportion	2017	48.3	65.4	31.4
Guatemala	San Ildefonso Ixtahuacán	Count	2000	0	1	0
Guatemala	San Ildefonso Ixtahuacán	Count	2017	0	1	0
Guatemala	San José Acatempa	Proportion	2000	43.0	64.8	22.2
Guatemala	San José Acatempa	Proportion	2017	56.1	71.6	41.5
Guatemala	San José Acatempa	Count	2000	0	1	0
Guatemala	San José Acatempa	Count	2017	1	1	0
Guatemala	Ostuncalco	Proportion	2000	38.1	59.8	18.7
Guatemala	Ostuncalco	Proportion	2017	41.5	59.5	27.1
Guatemala	Ostuncalco	Count	2000	0	1	0
Guatemala	Ostuncalco	Count	2017	0	1	0
Guatemala	San Andrés Sajcabajá	Proportion	2000	39.7	60.9	20.5
Guatemala	San Andrés Sajcabajá	Proportion	2017	47.1	64.1	30.6
Guatemala	San Andrés Sajcabajá	Count	2000	0	1	0
Guatemala	San Andrés Sajcabajá	Count	2017	0	1	0
Guatemala	Santiago Sacatepéquez	Proportion	2000	42.2	63.9	19.9
Guatemala	Santiago Sacatepéquez	Proportion	2017	47.3	66.5	28.9
Guatemala	Santiago Sacatepéquez	Count	2000	0	1	0
Guatemala	Santiago Sacatepéquez	Count	2017	0	1	0
Guatemala	Pajapita	Proportion	2000	37.6	60.1	18.2
Guatemala	Pajapita	Proportion	2017	39.9	56.7	25.3
Guatemala	Pajapita	Count	2000	0	1	0
Guatemala	Pajapita	Count	2017	0	1	0
Guatemala	Taxisco	Proportion	2000	41.5	61.6	22.3
Guatemala	Taxisco	Proportion	2017	52.2	69.3	36.9
Guatemala	Taxisco	Count	2000	0	1	0
Guatemala	Taxisco	Count	2017	1	1	0
Guatemala	Santa Catarina Palopó	Proportion	2000	41.7	63.4	18.9
Guatemala	Santa Catarina Palopó	Proportion	2017	45.7	63.0	26.1
Guatemala	Santa Catarina Palopó	Count	2000	0	1	0
Guatemala	Santa Catarina Palopó	Count	2017	0	1	0
Guatemala	San Lorenzo	Proportion	2000	37.3	59.9	18.8
Guatemala	San Lorenzo	Proportion	2017	39.0	58.0	23.4
Guatemala	San Lorenzo	Count	2000	0	1	0
Guatemala	San Lorenzo	Count	2017	0	1	0
Guatemala	Tucurú	Proportion	2000	38.6	59.2	18.8
Guatemala	Tucurú	Proportion	2017	44.2	61.4	27.7
Guatemala	Tucurú	Count	2000	0	1	0
Guatemala	Tucurú	Count	2017	0	1	0
Guatemala	Yepocapa	Proportion	2000	39.0	58.5	19.0
Guatemala	Yepocapa	Proportion	2017	44.2	61.2	27.4
Guatemala	Yepocapa	Count	2000	0	1	0

Table 4: ORS by Unit *(continued)*

Country	Unit	Metric	Year	Mean	Upper	Lower
Guatemala	Yepocapa	Count	2017	0	1	0
Guatemala	Villa Canales	Proportion	2000	43.5	63.7	20.9
Guatemala	Villa Canales	Proportion	2017	50.7	66.8	36.1
Guatemala	Villa Canales	Count	2000	0	1	0
Guatemala	Villa Canales	Count	2017	1	1	0
Guatemala	San Juan Atitán	Proportion	2000	39.3	61.2	19.9
Guatemala	San Juan Atitán	Proportion	2017	47.6	66.8	30.9
Guatemala	San Juan Atitán	Count	2000	0	1	0
Guatemala	San Juan Atitán	Count	2017	0	1	0
Guatemala	Santa Catarina Mita	Proportion	2000	43.3	65.7	22.7
Guatemala	Santa Catarina Mita	Proportion	2017	53.8	70.3	37.9
Guatemala	Santa Catarina Mita	Count	2000	0	1	0
Guatemala	Santa Catarina Mita	Count	2017	1	1	0
Guatemala	Palestina de Los Altos	Proportion	2000	37.6	58.0	18.2
Guatemala	Palestina de Los Altos	Proportion	2017	41.3	57.7	25.2
Guatemala	Palestina de Los Altos	Count	2000	0	1	0
Guatemala	Palestina de Los Altos	Count	2017	0	1	0
Guatemala	San Antonio Ilotenango	Proportion	2000	40.8	63.6	19.1
Guatemala	San Antonio Ilotenango	Proportion	2017	45.8	64.0	29.1
Guatemala	San Antonio Ilotenango	Count	2000	0	1	0
Guatemala	San Antonio Ilotenango	Count	2017	0	1	0
Guatemala	Santo Domingo Xenacoj	Proportion	2000	41.7	65.1	19.0
Guatemala	Santo Domingo Xenacoj	Proportion	2017	46.6	63.2	27.4
Guatemala	Santo Domingo Xenacoj	Count	2000	0	1	0
Guatemala	Santo Domingo Xenacoj	Count	2017	0	1	0
Guatemala	Río Blanco	Proportion	2000	38.5	61.8	18.5
Guatemala	Río Blanco	Proportion	2017	43.7	62.3	26.9
Guatemala	Río Blanco	Count	2000	0	1	0
Guatemala	Río Blanco	Count	2017	0	1	0
Guatemala	Santa Clara La Laguna	Proportion	2000	39.0	63.0	18.0
Guatemala	Santa Clara La Laguna	Proportion	2017	43.2	62.5	27.0
Guatemala	Santa Clara La Laguna	Count	2000	0	1	0
Guatemala	Santa Clara La Laguna	Count	2017	0	1	0
Guatemala	San Miguel Panán	Proportion	2000	36.6	59.1	16.5
Guatemala	San Miguel Panán	Proportion	2017	38.4	57.9	22.6
Guatemala	San Miguel Panán	Count	2000	0	1	0
Guatemala	San Miguel Panán	Count	2017	0	1	0
Guatemala	Zaragoza	Proportion	2000	41.5	63.5	20.3
Guatemala	Zaragoza	Proportion	2017	46.0	62.4	29.9
Guatemala	Zaragoza	Count	2000	0	1	0
Guatemala	Zaragoza	Count	2017	0	1	0
Guatemala	Villa Nueva	Proportion	2000	43.9	65.0	23.8
Guatemala	Villa Nueva	Proportion	2017	49.6	67.2	33.1
Guatemala	Villa Nueva	Count	2000	0	1	0
Guatemala	Villa Nueva	Count	2017	0	1	0
Guatemala	San Juan Ixcoy	Proportion	2000	39.7	61.6	20.3
Guatemala	San Juan Ixcoy	Proportion	2017	50.3	68.8	31.5
Guatemala	San Juan Ixcoy	Count	2000	0	1	0
Guatemala	San Juan Ixcoy	Count	2017	1	1	0

Table 4: ORS by Unit *(continued)*

Country	Unit	Metric	Year	Mean	Upper	Lower
Guatemala	Yupiltepeque	Proportion	2000	43.9	67.0	22.7
Guatemala	Yupiltepeque	Proportion	2017	57.0	74.9	39.4
Guatemala	Yupiltepeque	Count	2000	0	1	0
Guatemala	Yupiltepeque	Count	2017	1	1	0
Guatemala	Quetzaltenango	Proportion	2000	39.8	60.2	18.8
Guatemala	Quetzaltenango	Proportion	2017	42.4	60.1	27.8
Guatemala	Quetzaltenango	Count	2000	0	1	0
Guatemala	Quetzaltenango	Count	2017	0	1	0
Guatemala	San Bartolomé Jocotenango	Proportion	2000	39.4	61.7	20.5
Guatemala	San Bartolomé Jocotenango	Proportion	2017	46.9	63.2	30.3
Guatemala	San Bartolomé Jocotenango	Count	2000	0	1	0
Guatemala	San Bartolomé Jocotenango	Count	2017	0	1	0
Guatemala	Sumpango	Proportion	2000	41.6	63.1	19.3
Guatemala	Sumpango	Proportion	2017	46.0	64.1	27.7
Guatemala	Sumpango	Count	2000	0	1	0
Guatemala	Sumpango	Count	2017	0	1	0
Guatemala	San Antonio Sacatepéquez	Proportion	2000	38.0	60.6	18.1
Guatemala	San Antonio Sacatepéquez	Proportion	2017	41.9	60.7	25.8
Guatemala	San Antonio Sacatepéquez	Count	2000	0	1	0
Guatemala	San Antonio Sacatepéquez	Count	2017	0	1	0
Guatemala	Santa Cruz La Laguna	Proportion	2000	39.8	63.5	18.6
Guatemala	Santa Cruz La Laguna	Proportion	2017	43.6	62.7	26.2
Guatemala	Santa Cruz La Laguna	Count	2000	0	1	0
Guatemala	Santa Cruz La Laguna	Count	2017	0	1	0
Guatemala	San Pablo Jocopilas	Proportion	2000	37.9	61.0	17.5
Guatemala	San Pablo Jocopilas	Proportion	2017	39.6	59.8	24.5
Guatemala	San Pablo Jocopilas	Count	2000	0	1	0
Guatemala	San Pablo Jocopilas	Count	2017	0	1	0
Guatemala	ZONA 1	Proportion	2000	43.4	66.8	18.4
Guatemala	ZONA 1	Proportion	2017	50.0	69.7	30.9
Guatemala	ZONA 1	Count	2000	0	1	0
Guatemala	ZONA 1	Count	2017	0	1	0
Guatemala	San Mateo Ixtatán	Proportion	2000	38.0	59.1	20.9
Guatemala	San Mateo Ixtatán	Proportion	2017	48.1	65.8	32.5
Guatemala	San Mateo Ixtatán	Count	2000	0	1	0
Guatemala	San Mateo Ixtatán	Count	2017	0	1	0
Guatemala	Zapotitlán	Proportion	2000	43.9	66.8	22.7
Guatemala	Zapotitlán	Proportion	2017	57.6	74.1	40.1
Guatemala	Zapotitlán	Count	2000	0	1	0
Guatemala	Zapotitlán	Count	2017	1	1	0
Guatemala	Salcajá	Proportion	2000	40.1	61.0	19.9
Guatemala	Salcajá	Proportion	2017	42.6	59.3	28.4
Guatemala	Salcajá	Count	2000	0	1	0
Guatemala	Salcajá	Count	2017	0	1	0
Guatemala	San Juan Cotzal	Proportion	2000	39.6	60.4	18.0

Table 4: ORS by Unit *(continued)*

Country	Unit	Metric	Year	Mean	Upper	Lower
Guatemala	San Juan Cotzal	Proportion	2017	48.2	64.5	33.1
Guatemala	San Juan Cotzal	Count	2000	0	1	0
Guatemala	San Juan Cotzal	Count	2017	0	1	0
Guatemala	San Cristobal Cucho	Proportion	2000	36.2	61.1	16.2
Guatemala	San Cristobal Cucho	Proportion	2017	40.0	58.4	24.3
Guatemala	San Cristobal Cucho	Count	2000	0	1	0
Guatemala	San Cristobal Cucho	Count	2017	0	1	0
Guatemala	Santa Lucía Utatlán	Proportion	2000	39.5	61.3	19.4
Guatemala	Santa Lucía Utatlán	Proportion	2017	43.4	62.7	28.2
Guatemala	Santa Lucía Utatlán	Count	2000	0	1	0
Guatemala	Santa Lucía Utatlán	Count	2017	0	1	0
Guatemala	Santa Bárbara	Proportion	2000	37.2	57.2	18.5
Guatemala	Santa Bárbara	Proportion	2017	40.6	59.1	25.6
Guatemala	Santa Bárbara	Count	2000	0	1	0
Guatemala	Santa Bárbara	Count	2017	0	1	0
Guatemala	ZONA 10	Proportion	2000	43.3	68.3	19.2
Guatemala	ZONA 10	Proportion	2017	51.1	71.8	31.8
Guatemala	ZONA 10	Count	2000	0	1	0
Guatemala	ZONA 10	Count	2017	1	1	0
Guatemala	San Miguel Acatán	Proportion	2000	39.3	62.6	21.3
Guatemala	San Miguel Acatán	Proportion	2017	50.3	67.4	33.6
Guatemala	San Miguel Acatán	Count	2000	0	1	0
Guatemala	San Miguel Acatán	Count	2017	1	1	0
Guatemala	San Carlos Sija	Proportion	2000	39.0	59.3	18.4
Guatemala	San Carlos Sija	Proportion	2017	44.6	61.5	29.2
Guatemala	San Carlos Sija	Count	2000	0	1	0
Guatemala	San Carlos Sija	Count	2017	0	1	0
Guatemala	San Pedro Jocopilas	Proportion	2000	39.6	61.7	20.4
Guatemala	San Pedro Jocopilas	Proportion	2017	46.2	62.5	30.8
Guatemala	San Pedro Jocopilas	Count	2000	0	1	0
Guatemala	San Pedro Jocopilas	Count	2017	0	1	0
Guatemala	San José Ojetenam	Proportion	2000	38.8	59.7	18.8
Guatemala	San José Ojetenam	Proportion	2017	43.7	61.9	27.3
Guatemala	San José Ojetenam	Count	2000	0	1	0
Guatemala	San José Ojetenam	Count	2017	0	1	0
Guatemala	Santa María Visitación	Proportion	2000	38.9	63.5	18.8
Guatemala	Santa María Visitación	Proportion	2017	42.8	62.7	25.9
Guatemala	Santa María Visitación	Count	2000	0	1	0
Guatemala	Santa María Visitación	Count	2017	0	1	0
Guatemala	Santo Domingo Suchitepequez	Proportion	2000	37.0	59.0	18.7
Guatemala	Santo Domingo Suchitepequez	Proportion	2017	38.3	56.7	24.2
Guatemala	Santo Domingo Suchitepequez	Count	2000	0	1	0
Guatemala	Santo Domingo Suchitepequez	Count	2017	0	1	0
Guatemala	ZONA 11	Proportion	2000	44.3	67.9	22.9
Guatemala	ZONA 11	Proportion	2017	50.1	69.5	31.5
Guatemala	ZONA 11	Count	2000	0	1	0

Table 4: ORS by Unit *(continued)*

Country	Unit	Metric	Year	Mean	Upper	Lower
Guatemala	ZONA 11	Count	2017	1	1	0
Guatemala	San Pedro Necta	Proportion	2000	39.6	64.2	18.8
Guatemala	San Pedro Necta	Proportion	2017	49.5	67.5	32.3
Guatemala	San Pedro Necta	Count	2000	0	1	0
Guatemala	San Pedro Necta	Count	2017	0	1	0
Guatemala	San Francisco La Unión	Proportion	2000	39.0	61.9	17.0
Guatemala	San Francisco La Unión	Proportion	2017	42.4	59.9	27.8
Guatemala	San Francisco La Unión	Count	2000	0	1	0
Guatemala	San Francisco La Unión	Count	2017	0	1	0
Guatemala	Santa Cruz del Quiché	Proportion	2000	41.0	64.7	19.3
Guatemala	Santa Cruz del Quiché	Proportion	2017	46.2	63.7	29.4
Guatemala	Santa Cruz del Quiché	Count	2000	0	1	0
Guatemala	Santa Cruz del Quiché	Count	2017	0	1	0
Guatemala	San Lorenzo	Proportion	2000	37.4	60.5	17.1
Guatemala	San Lorenzo	Proportion	2017	41.5	60.8	25.1
Guatemala	San Lorenzo	Count	2000	0	1	0
Guatemala	San Lorenzo	Count	2017	0	1	0
Guatemala	Santiago Atitlán	Proportion	2000	40.1	61.2	20.8
Guatemala	Santiago Atitlán	Proportion	2017	43.7	60.9	28.6
Guatemala	Santiago Atitlán	Count	2000	0	1	0
Guatemala	Santiago Atitlán	Count	2017	0	1	0
Guatemala	Santo Tomás La Unión	Proportion	2000	38.3	59.8	18.1
Guatemala	Santo Tomás La Unión	Proportion	2017	40.1	60.9	23.4
Guatemala	Santo Tomás La Unión	Count	2000	0	1	0
Guatemala	Santo Tomás La Unión	Count	2017	0	1	0
Guatemala	ZONA 12	Proportion	2000	44.2	64.7	22.3
Guatemala	ZONA 12	Proportion	2017	50.2	69.0	33.0
Guatemala	ZONA 12	Count	2000	0	1	0
Guatemala	ZONA 12	Count	2017	1	1	0
Guatemala	San Rafael La Independencia	Proportion	2000	39.3	63.8	19.9
Guatemala	San Rafael La Independencia	Proportion	2017	49.8	67.2	31.2
Guatemala	San Rafael La Independencia	Count	2000	0	1	0
Guatemala	San Rafael La Independencia	Count	2017	0	1	0
Guatemala	San Martín Sacatepéquez	Proportion	2000	37.2	61.0	18.3
Guatemala	San Martín Sacatepéquez	Proportion	2017	40.6	57.8	26.2
Guatemala	San Martín Sacatepéquez	Count	2000	0	1	0
Guatemala	San Martín Sacatepéquez	Count	2017	0	1	0
Guatemala	Uspantán	Proportion	2000	39.3	59.5	19.1
Guatemala	Uspantán	Proportion	2017	47.1	62.9	31.6
Guatemala	Uspantán	Count	2000	0	1	0
Guatemala	Uspantán	Count	2017	0	1	0
Guatemala	San Marcos	Proportion	2000	38.1	59.2	17.9
Guatemala	San Marcos	Proportion	2017	41.4	57.9	27.0
Guatemala	San Marcos	Count	2000	0	1	0
Guatemala	San Marcos	Count	2017	0	1	0

Table 4: ORS by Unit *(continued)*

Country	Unit	Metric	Year	Mean	Upper	Lower
Guatemala	Sololá	Proportion	2000	40.3	61.7	18.1
Guatemala	Sololá	Proportion	2017	43.9	61.1	26.3
Guatemala	Sololá	Count	2000	0	1	0
Guatemala	Sololá	Count	2017	0	1	0
Guatemala	Zunilito	Proportion	2000	37.6	59.0	16.4
Guatemala	Zunilito	Proportion	2017	39.2	58.5	22.6
Guatemala	Zunilito	Count	2000	0	1	0
Guatemala	Zunilito	Count	2017	0	1	0
Guatemala	ZONA 13	Proportion	2000	43.6	68.5	20.6
Guatemala	ZONA 13	Proportion	2017	50.8	70.1	32.2
Guatemala	ZONA 13	Count	2000	0	1	0
Guatemala	ZONA 13	Count	2017	1	1	0
Guatemala	San Rafael Petzal	Proportion	2000	40.3	64.0	19.8
Guatemala	San Rafael Petzal	Proportion	2017	49.5	67.5	31.7
Guatemala	San Rafael Petzal	Count	2000	0	1	0
Guatemala	San Rafael Petzal	Count	2017	0	1	0
Guatemala	San Mateo	Proportion	2000	39.5	62.0	19.3
Guatemala	San Mateo	Proportion	2017	42.3	62.0	26.7
Guatemala	San Mateo	Count	2000	0	1	0
Guatemala	San Mateo	Count	2017	0	1	0
Guatemala	Zacualpa	Proportion	2000	39.6	61.5	21.4
Guatemala	Zacualpa	Proportion	2017	46.3	62.7	29.5
Guatemala	Zacualpa	Count	2000	0	1	0
Guatemala	Zacualpa	Count	2017	0	1	0
Guatemala	San Miguel Ixtahuacán	Proportion	2000	36.1	55.2	17.7
Guatemala	San Miguel Ixtahuacán	Proportion	2017	42.8	60.6	26.3
Guatemala	San Miguel Ixtahuacán	Count	2000	0	1	0
Guatemala	San Miguel Ixtahuacán	Count	2017	0	1	0
Guatemala	ZONA 14	Proportion	2000	43.8	68.1	21.5
Guatemala	ZONA 14	Proportion	2017	50.6	69.7	32.8
Guatemala	ZONA 14	Count	2000	0	1	0
Guatemala	ZONA 14	Count	2017	1	1	0
Guatemala	San Sebastián Coatán	Proportion	2000	38.5	61.3	20.2
Guatemala	San Sebastián Coatán	Proportion	2017	49.7	67.6	32.8
Guatemala	San Sebastián Coatán	Count	2000	0	1	0
Guatemala	San Sebastián Coatán	Count	2017	0	1	0
Guatemala	San Miguel Sigüilá	Proportion	2000	39.0	62.8	18.6
Guatemala	San Miguel Sigüilá	Proportion	2017	42.6	61.9	23.4
Guatemala	San Miguel Sigüilá	Count	2000	0	1	0
Guatemala	San Miguel Sigüilá	Count	2017	0	1	0
Guatemala	San Pablo	Proportion	2000	37.5	59.7	17.2
Guatemala	San Pablo	Proportion	2017	40.3	57.3	25.4
Guatemala	San Pablo	Count	2000	0	1	0
Guatemala	San Pablo	Count	2017	0	1	0
Guatemala	ZONA 15	Proportion	2000	43.7	67.2	21.6
Guatemala	ZONA 15	Proportion	2017	50.4	67.3	32.0
Guatemala	ZONA 15	Count	2000	0	1	0
Guatemala	ZONA 15	Count	2017	1	1	0

Table 4: ORS by Unit *(continued)*

Country	Unit	Metric	Year	Mean	Upper	Lower
Guatemala	San Sebastián Huehuetenango	Proportion	2000	39.5	60.3	20.4
Guatemala	San Sebastián Huehuetenango	Proportion	2017	48.6	68.0	32.7
Guatemala	San Sebastián Huehuetenango	Count	2000	0	1	0
Guatemala	San Sebastián Huehuetenango	Count	2017	0	1	0
Guatemala	Sibilia	Proportion	2000	37.8	59.0	16.6
Guatemala	Sibilia	Proportion	2017	42.9	59.4	27.7
Guatemala	Sibilia	Count	2000	0	1	0
Guatemala	Sibilia	Count	2017	0	1	0
Guatemala	San Pedro Sacatepéquez	Proportion	2000	38.3	62.0	18.8
Guatemala	San Pedro Sacatepéquez	Proportion	2017	41.6	60.5	26.8
Guatemala	San Pedro Sacatepéquez	Count	2000	0	1	0
Guatemala	San Pedro Sacatepéquez	Count	2017	0	1	0
Guatemala	ZONA 16	Proportion	2000	44.3	68.5	23.2
Guatemala	ZONA 16	Proportion	2017	49.9	67.3	31.2
Guatemala	ZONA 16	Count	2000	0	1	0
Guatemala	ZONA 16	Count	2017	0	1	0
Guatemala	Santa Ana Huista	Proportion	2000	41.1	63.3	20.7
Guatemala	Santa Ana Huista	Proportion	2017	51.4	69.5	33.7
Guatemala	Santa Ana Huista	Count	2000	0	1	0
Guatemala	Santa Ana Huista	Count	2017	1	1	0
Guatemala	Zunil	Proportion	2000	38.6	58.9	17.9
Guatemala	Zunil	Proportion	2017	42.5	61.5	26.7
Guatemala	Zunil	Count	2000	0	1	0
Guatemala	Zunil	Count	2017	0	1	0
Guatemala	San Rafaél Pie de la Cuesta	Proportion	2000	37.1	59.6	17.2
Guatemala	San Rafaél Pie de la Cuesta	Proportion	2017	40.3	57.3	25.5
Guatemala	San Rafaél Pie de la Cuesta	Count	2000	0	1	0
Guatemala	San Rafaél Pie de la Cuesta	Count	2017	0	1	0
Guatemala	ZONA 17	Proportion	2000	44.4	67.1	21.4
Guatemala	ZONA 17	Proportion	2017	50.0	68.8	32.9
Guatemala	ZONA 17	Count	2000	0	1	0
Guatemala	ZONA 17	Count	2017	0	1	0
Guatemala	Santa Bárbara	Proportion	2000	40.0	61.2	20.4
Guatemala	Santa Bárbara	Proportion	2017	48.3	66.3	32.6
Guatemala	Santa Bárbara	Count	2000	0	1	0
Guatemala	Santa Bárbara	Count	2017	0	1	0
Guatemala	San Sibinal	Proportion	2000	37.6	60.1	17.6
Guatemala	San Sibinal	Proportion	2017	41.8	60.0	26.2
Guatemala	San Sibinal	Count	2000	0	1	0
Guatemala	San Sibinal	Count	2017	0	1	0
Guatemala	ZONA 18	Proportion	2000	44.3	64.9	21.2
Guatemala	ZONA 18	Proportion	2017	50.1	67.7	33.9

Table 4: ORS by Unit *(continued)*

Country	Unit	Metric	Year	Mean	Upper	Lower
Guatemala	ZONA 18	Count	2000	0	1	0
Guatemala	ZONA 18	Count	2017	1	1	0
Guatemala	Santa Cruz Barillas	Proportion	2000	37.9	59.3	19.1
Guatemala	Santa Cruz Barillas	Proportion	2017	47.8	65.4	32.2
Guatemala	Santa Cruz Barillas	Count	2000	0	1	0
Guatemala	Santa Cruz Barillas	Count	2017	0	1	0
Guatemala	Sipacapa	Proportion	2000	36.8	58.0	16.4
Guatemala	Sipacapa	Proportion	2017	42.8	59.9	28.2
Guatemala	Sipacapa	Count	2000	0	1	0
Guatemala	Sipacapa	Count	2017	0	1	0
Guatemala	ZONA 19	Proportion	2000	45.4	69.0	22.9
Guatemala	ZONA 19	Proportion	2017	50.3	69.0	31.9
Guatemala	ZONA 19	Count	2000	0	1	0
Guatemala	ZONA 19	Count	2017	1	1	0
Guatemala	Santa Eulalia	Proportion	2000	38.1	60.7	18.8
Guatemala	Santa Eulalia	Proportion	2017	48.6	66.0	32.4
Guatemala	Santa Eulalia	Count	2000	0	1	0
Guatemala	Santa Eulalia	Count	2017	0	1	0
Guatemala	Tacaná	Proportion	2000	38.5	58.8	19.2
Guatemala	Tacaná	Proportion	2017	43.5	59.8	26.2
Guatemala	Tacaná	Count	2000	0	1	0
Guatemala	Tacaná	Count	2017	0	1	0
Guatemala	ZONA 2	Proportion	2000	43.7	66.3	20.3
Guatemala	ZONA 2	Proportion	2017	49.9	67.4	34.2
Guatemala	ZONA 2	Count	2000	0	1	0
Guatemala	ZONA 2	Count	2017	0	1	0
Guatemala	Santiago Chimaltenango	Proportion	2000	40.1	63.3	19.6
Guatemala	Santiago Chimaltenango	Proportion	2017	49.7	69.3	32.4
Guatemala	Santiago Chimaltenango	Count	2000	0	1	0
Guatemala	Santiago Chimaltenango	Count	2017	0	1	0
Guatemala	Tajumulco	Proportion	2000	37.5	57.8	17.7
Guatemala	Tajumulco	Proportion	2017	41.5	58.3	26.8
Guatemala	Tajumulco	Count	2000	0	1	0
Guatemala	Tajumulco	Count	2017	0	1	0
Guatemala	ZONA 22	Proportion	2000	44.5	65.9	21.7
Guatemala	ZONA 22	Proportion	2017	50.1	69.5	31.8
Guatemala	ZONA 22	Count	2000	0	1	0
Guatemala	ZONA 22	Count	2017	1	1	0
Guatemala	Soloma	Proportion	2000	40.0	63.0	20.4
Guatemala	Soloma	Proportion	2017	50.2	68.6	31.9
Guatemala	Soloma	Count	2000	0	1	0
Guatemala	Soloma	Count	2017	1	1	0
Guatemala	Tejutla	Proportion	2000	37.4	58.6	17.5
Guatemala	Tejutla	Proportion	2017	42.8	60.2	28.6
Guatemala	Tejutla	Count	2000	0	1	0
Guatemala	Tejutla	Count	2017	0	1	0
Guatemala	ZONA 24	Proportion	2000	44.2	67.0	20.7
Guatemala	ZONA 24	Proportion	2017	50.1	68.0	31.9
Guatemala	ZONA 24	Count	2000	0	1	0

Table 4: ORS by Unit *(continued)*

Country	Unit	Metric	Year	Mean	Upper	Lower
Guatemala	ZONA 24	Count	2017	1	1	0
Guatemala	Tectitán	Proportion	2000	38.1	59.3	18.5
Guatemala	Tectitán	Proportion	2017	44.7	60.9	28.9
Guatemala	Tectitán	Count	2000	0	1	0
Guatemala	Tectitán	Count	2017	0	1	0
Guatemala	ZONA 25	Proportion	2000	44.4	68.1	19.6
Guatemala	ZONA 25	Proportion	2017	50.7	68.6	33.0
Guatemala	ZONA 25	Count	2000	0	1	0
Guatemala	ZONA 25	Count	2017	1	1	0
Guatemala	Todos Santos Cuchumatán	Proportion	2000	38.8	60.8	20.8
Guatemala	Todos Santos Cuchumatán	Proportion	2017	48.1	67.1	32.1
Guatemala	Todos Santos Cuchumatán	Count	2000	0	1	0
Guatemala	Todos Santos Cuchumatán	Count	2017	0	1	0
Guatemala	ZONA 3	Proportion	2000	43.4	66.9	19.7
Guatemala	ZONA 3	Proportion	2017	50.2	70.1	32.2
Guatemala	ZONA 3	Count	2000	0	1	0
Guatemala	ZONA 3	Count	2017	1	1	0
Guatemala	ZONA 4	Proportion	2000	43.3	68.3	19.3
Guatemala	ZONA 4	Proportion	2017	51.1	71.6	32.2
Guatemala	ZONA 4	Count	2000	0	1	0
Guatemala	ZONA 4	Count	2017	1	1	0
Guatemala	ZONA 5	Proportion	2000	43.9	65.7	20.7
Guatemala	ZONA 5	Proportion	2017	50.4	69.6	34.4
Guatemala	ZONA 5	Count	2000	0	1	0
Guatemala	ZONA 5	Count	2017	1	1	0
Guatemala	ZONA 6	Proportion	2000	44.3	65.7	20.8
Guatemala	ZONA 6	Proportion	2017	49.9	67.5	34.3
Guatemala	ZONA 6	Count	2000	0	1	0
Guatemala	ZONA 6	Count	2017	0	1	0
Guatemala	ZONA 7	Proportion	2000	44.6	67.7	21.8
Guatemala	ZONA 7	Proportion	2017	50.2	67.7	33.2
Guatemala	ZONA 7	Count	2000	0	1	0
Guatemala	ZONA 7	Count	2017	1	1	0
Guatemala	ZONA 8	Proportion	2000	43.3	68.3	19.3
Guatemala	ZONA 8	Proportion	2017	51.1	71.6	32.2
Guatemala	ZONA 8	Count	2000	0	1	0
Guatemala	ZONA 8	Count	2017	1	1	0
Guatemala	ZONA 9	Proportion	2000	43.3	68.4	18.9
Guatemala	ZONA 9	Proportion	2017	51.2	72.4	31.7
Guatemala	ZONA 9	Count	2000	0	1	0
Guatemala	ZONA 9	Count	2017	1	1	0
Guinea	Boffa	Proportion	2000	32.7	60.6	15.5
Guinea	Boffa	Proportion	2017	37.9	57.9	18.9
Guinea	Boffa	Count	2000	0	1	0
Guinea	Boffa	Count	2017	0	1	0
Guinea	Conakry	Proportion	2000	43.2	69.5	23.0
Guinea	Conakry	Proportion	2017	51.3	73.4	31.7
Guinea	Conakry	Count	2000	0	1	0
Guinea	Conakry	Count	2017	1	1	0

Table 4: ORS by Unit *(continued)*

Country	Unit	Metric	Year	Mean	Upper	Lower
Guinea	Dabola	Proportion	2000	14.3	28.4	5.0
Guinea	Dabola	Proportion	2017	28.3	47.8	13.0
Guinea	Dabola	Count	2000	0	0	0
Guinea	Dabola	Count	2017	0	0	0
Guinea	Kankan	Proportion	2000	18.2	36.9	7.3
Guinea	Kankan	Proportion	2017	29.8	52.3	13.2
Guinea	Kankan	Count	2000	0	0	0
Guinea	Kankan	Count	2017	0	1	0
Guinea	Coyah	Proportion	2000	37.9	61.8	19.8
Guinea	Coyah	Proportion	2017	49.3	71.3	30.7
Guinea	Coyah	Count	2000	0	1	0
Guinea	Coyah	Count	2017	0	1	0
Guinea	Koubia	Proportion	2000	15.8	33.6	6.4
Guinea	Koubia	Proportion	2017	23.6	42.1	10.3
Guinea	Koubia	Count	2000	0	0	0
Guinea	Koubia	Count	2017	0	0	0
Guinea	Dalaba	Proportion	2000	16.3	35.2	7.0
Guinea	Dalaba	Proportion	2017	26.9	47.7	13.0
Guinea	Dalaba	Count	2000	0	0	0
Guinea	Dalaba	Count	2017	0	0	0
Guinea	Beyla	Proportion	2000	22.3	44.4	9.8
Guinea	Beyla	Proportion	2017	28.0	49.6	12.7
Guinea	Beyla	Count	2000	0	0	0
Guinea	Beyla	Count	2017	0	0	0
Guinea	Boké	Proportion	2000	29.9	55.1	14.6
Guinea	Boké	Proportion	2017	31.9	51.7	13.6
Guinea	Boké	Count	2000	0	1	0
Guinea	Boké	Count	2017	0	1	0
Guinea	Dinguiraye	Proportion	2000	12.0	24.8	4.6
Guinea	Dinguiraye	Proportion	2017	23.3	41.3	10.1
Guinea	Dinguiraye	Count	2000	0	0	0
Guinea	Dinguiraye	Count	2017	0	0	0
Guinea	Kérouané	Proportion	2000	25.7	47.7	10.8
Guinea	Kérouané	Proportion	2017	33.8	56.1	15.0
Guinea	Kérouané	Count	2000	0	0	0
Guinea	Kérouané	Count	2017	0	1	0
Guinea	Dubréka	Proportion	2000	31.7	57.8	15.5
Guinea	Dubréka	Proportion	2017	39.8	59.4	21.4
Guinea	Dubréka	Count	2000	0	1	0
Guinea	Dubréka	Count	2017	0	1	0
Guinea	Labé	Proportion	2000	18.4	39.2	7.9
Guinea	Labé	Proportion	2017	25.7	46.0	11.8
Guinea	Labé	Count	2000	0	0	0
Guinea	Labé	Count	2017	0	0	0
Guinea	Mamou	Proportion	2000	15.8	30.8	6.2
Guinea	Mamou	Proportion	2017	30.9	51.7	16.6
Guinea	Mamou	Count	2000	0	0	0
Guinea	Mamou	Count	2017	0	1	0
Guinea	Guéckédou	Proportion	2000	37.4	59.0	18.3

Table 4: ORS by Unit *(continued)*

Country	Unit	Metric	Year	Mean	Upper	Lower
Guinea	Guéckédou	Proportion	2017	44.4	63.6	29.4
Guinea	Guéckédou	Count	2000	0	1	0
Guinea	Guéckédou	Count	2017	0	1	0
Guinea	Fria	Proportion	2000	30.2	54.8	13.1
Guinea	Fria	Proportion	2017	34.6	55.9	15.5
Guinea	Fria	Count	2000	0	1	0
Guinea	Fria	Count	2017	0	1	0
Guinea	Faranah	Proportion	2000	17.6	32.3	8.3
Guinea	Faranah	Proportion	2017	32.4	50.8	16.1
Guinea	Faranah	Count	2000	0	0	0
Guinea	Faranah	Count	2017	0	1	0
Guinea	Kouroussa	Proportion	2000	15.9	30.4	6.5
Guinea	Kouroussa	Proportion	2017	28.3	51.3	12.0
Guinea	Kouroussa	Count	2000	0	0	0
Guinea	Kouroussa	Count	2017	0	1	0
Guinea	Forécariah	Proportion	2000	28.2	49.8	14.6
Guinea	Forécariah	Proportion	2017	44.0	65.0	28.3
Guinea	Forécariah	Count	2000	0	0	0
Guinea	Forécariah	Count	2017	0	1	0
Guinea	Lélouma	Proportion	2000	22.1	44.2	9.4
Guinea	Lélouma	Proportion	2017	25.5	46.4	11.9
Guinea	Lélouma	Count	2000	0	0	0
Guinea	Lélouma	Count	2017	0	0	0
Guinea	Pita	Proportion	2000	19.9	42.4	8.4
Guinea	Pita	Proportion	2017	27.4	47.1	11.8
Guinea	Pita	Count	2000	0	0	0
Guinea	Pita	Count	2017	0	0	0
Guinea	Lola	Proportion	2000	23.7	44.0	9.3
Guinea	Lola	Proportion	2017	30.5	48.7	14.5
Guinea	Lola	Count	2000	0	0	0
Guinea	Lola	Count	2017	0	0	0
Guinea	Gaoual	Proportion	2000	28.8	49.1	14.0
Guinea	Gaoual	Proportion	2017	25.4	41.9	11.3
Guinea	Gaoual	Count	2000	0	0	0
Guinea	Gaoual	Count	2017	0	0	0
Guinea	Kissidougou	Proportion	2000	26.9	46.7	11.5
Guinea	Kissidougou	Proportion	2017	35.4	55.6	17.8
Guinea	Kissidougou	Count	2000	0	0	0
Guinea	Kissidougou	Count	2017	0	1	0
Guinea	Mandiana	Proportion	2000	13.5	27.9	5.0
Guinea	Mandiana	Proportion	2017	25.3	47.3	11.4
Guinea	Mandiana	Count	2000	0	0	0
Guinea	Mandiana	Count	2017	0	0	0
Guinea	Kindia	Proportion	2000	21.4	43.3	8.4
Guinea	Kindia	Proportion	2017	34.1	53.8	17.9
Guinea	Kindia	Count	2000	0	0	0
Guinea	Kindia	Count	2017	0	1	0
Guinea	Mali	Proportion	2000	19.8	36.8	8.7
Guinea	Mali	Proportion	2017	24.7	41.1	10.5

Table 4: ORS by Unit *(continued)*

Country	Unit	Metric	Year	Mean	Upper	Lower
Guinea	Mali	Count	2000	0	0	0
Guinea	Mali	Count	2017	0	0	0
Guinea	Macenta	Proportion	2000	33.0	54.3	16.3
Guinea	Macenta	Proportion	2017	40.0	62.4	20.5
Guinea	Macenta	Count	2000	0	1	0
Guinea	Macenta	Count	2017	0	1	0
Guinea	Koundara	Proportion	2000	29.3	46.0	16.2
Guinea	Koundara	Proportion	2017	24.2	39.9	10.1
Guinea	Koundara	Count	2000	0	0	0
Guinea	Koundara	Count	2017	0	0	0
Guinea	Siguiri	Proportion	2000	12.2	23.7	4.3
Guinea	Siguiri	Proportion	2017	25.2	50.3	11.0
Guinea	Siguiri	Count	2000	0	0	0
Guinea	Siguiri	Count	2017	0	1	0
Guinea	Télimélé	Proportion	2000	25.5	51.6	11.9
Guinea	Télimélé	Proportion	2017	28.6	48.5	12.2
Guinea	Télimélé	Count	2000	0	1	0
Guinea	Télimélé	Count	2017	0	0	0
Guinea	Tougué	Proportion	2000	14.3	30.4	5.7
Guinea	Tougué	Proportion	2017	24.1	43.6	10.6
Guinea	Tougué	Count	2000	0	0	0
Guinea	Tougué	Count	2017	0	0	0
Guinea	Nzérékoré	Proportion	2000	26.3	45.8	10.4
Guinea	Nzérékoré	Proportion	2017	34.1	55.0	16.2
Guinea	Nzérékoré	Count	2000	0	0	0
Guinea	Nzérékoré	Count	2017	0	1	0
Guinea	Yamou	Proportion	2000	28.9	46.7	12.8
Guinea	Yamou	Proportion	2017	38.1	60.0	18.2
Guinea	Yamou	Count	2000	0	0	0
Guinea	Yamou	Count	2017	0	1	0
Guinea-Bissau	Bafata	Proportion	2000	42.0	53.8	28.7
Guinea-Bissau	Bafata	Proportion	2017	31.0	49.4	16.0
Guinea-Bissau	Bafata	Count	2000	0	1	0
Guinea-Bissau	Bafata	Count	2017	0	0	0
Guinea-Bissau	Prabis	Proportion	2000	36.6	48.9	25.1
Guinea-Bissau	Prabis	Proportion	2017	37.7	58.9	21.4
Guinea-Bissau	Prabis	Count	2000	0	0	0
Guinea-Bissau	Prabis	Count	2017	0	1	0
Guinea-Bissau	Bissau	Proportion	2000	38.2	50.8	25.3
Guinea-Bissau	Bissau	Proportion	2017	36.0	57.8	19.0
Guinea-Bissau	Bissau	Count	2000	0	1	0
Guinea-Bissau	Bissau	Count	2017	0	1	0
Guinea-Bissau	Bolama	Proportion	2000	36.3	50.3	22.8
Guinea-Bissau	Bolama	Proportion	2017	42.5	64.7	23.9
Guinea-Bissau	Bolama	Count	2000	0	1	0
Guinea-Bissau	Bolama	Count	2017	0	1	0
Guinea-Bissau	Bigene	Proportion	2000	31.0	42.7	20.9
Guinea-Bissau	Bigene	Proportion	2017	28.9	43.0	16.2
Guinea-Bissau	Bigene	Count	2000	0	0	0

Table 4: ORS by Unit *(continued)*

Country	Unit	Metric	Year	Mean	Upper	Lower
Guinea-Bissau	Bigene	Count	2017	0	0	0
Guinea-Bissau	Boe	Proportion	2000	42.7	59.9	28.2
Guinea-Bissau	Boe	Proportion	2017	28.6	48.0	13.4
Guinea-Bissau	Boe	Count	2000	0	1	0
Guinea-Bissau	Boe	Count	2017	0	0	0
Guinea-Bissau	Bissora	Proportion	2000	33.5	45.8	22.4
Guinea-Bissau	Bissora	Proportion	2017	29.2	46.0	15.9
Guinea-Bissau	Bissora	Count	2000	0	0	0
Guinea-Bissau	Bissora	Count	2017	0	0	0
Guinea-Bissau	Buba	Proportion	2000	37.8	52.1	23.6
Guinea-Bissau	Buba	Proportion	2017	40.5	65.3	22.3
Guinea-Bissau	Buba	Count	2000	0	1	0
Guinea-Bissau	Buba	Count	2017	0	1	0
Guinea-Bissau	Bedanda	Proportion	2000	36.0	53.1	21.1
Guinea-Bissau	Bedanda	Proportion	2017	42.6	71.0	23.3
Guinea-Bissau	Bedanda	Count	2000	0	1	0
Guinea-Bissau	Bedanda	Count	2017	0	1	0
Guinea-Bissau	Bambadinca	Proportion	2000	39.1	52.3	26.0
Guinea-Bissau	Bambadinca	Proportion	2017	33.8	54.8	16.6
Guinea-Bissau	Bambadinca	Count	2000	0	1	0
Guinea-Bissau	Bambadinca	Count	2017	0	1	0
Guinea-Bissau	Quinhamel	Proportion	2000	33.9	46.0	22.8
Guinea-Bissau	Quinhamel	Proportion	2017	36.7	57.7	21.0
Guinea-Bissau	Quinhamel	Count	2000	0	0	0
Guinea-Bissau	Quinhamel	Count	2017	0	1	0
Guinea-Bissau	Bubaque	Proportion	2000	31.5	44.8	19.2
Guinea-Bissau	Bubaque	Proportion	2017	43.0	68.3	21.9
Guinea-Bissau	Bubaque	Count	2000	0	0	0
Guinea-Bissau	Bubaque	Count	2017	0	1	0
Guinea-Bissau	Bula	Proportion	2000	31.6	43.1	21.0
Guinea-Bissau	Bula	Proportion	2017	31.1	48.6	17.5
Guinea-Bissau	Bula	Count	2000	0	0	0
Guinea-Bissau	Bula	Count	2017	0	0	0
Guinea-Bissau	Gabu	Proportion	2000	46.1	59.1	32.8
Guinea-Bissau	Gabu	Proportion	2017	29.0	48.5	14.5
Guinea-Bissau	Gabu	Count	2000	0	1	0
Guinea-Bissau	Gabu	Count	2017	0	0	0
Guinea-Bissau	Farim	Proportion	2000	33.9	45.5	24.5
Guinea-Bissau	Farim	Proportion	2017	28.6	44.4	16.1
Guinea-Bissau	Farim	Count	2000	0	0	0
Guinea-Bissau	Farim	Count	2017	0	0	0
Guinea-Bissau	Empada	Proportion	2000	37.5	52.0	24.2
Guinea-Bissau	Empada	Proportion	2017	43.5	68.6	24.5
Guinea-Bissau	Empada	Count	2000	0	1	0
Guinea-Bissau	Empada	Count	2017	0	1	0
Guinea-Bissau	Cacine	Proportion	2000	35.0	54.6	20.1
Guinea-Bissau	Cacine	Proportion	2017	41.9	71.3	20.8
Guinea-Bissau	Cacine	Count	2000	0	1	0
Guinea-Bissau	Cacine	Count	2017	0	1	0

Table 4: ORS by Unit *(continued)*

Country	Unit	Metric	Year	Mean	Upper	Lower
Guinea-Bissau	Contuboel	Proportion	2000	37.6	50.0	26.4
Guinea-Bissau	Contuboel	Proportion	2017	29.4	47.6	15.3
Guinea-Bissau	Contuboel	Count	2000	0	0	0
Guinea-Bissau	Contuboel	Count	2017	0	0	0
Guinea-Bissau	Safim	Proportion	2000	36.5	48.3	23.9
Guinea-Bissau	Safim	Proportion	2017	36.1	56.4	20.6
Guinea-Bissau	Safim	Count	2000	0	0	0
Guinea-Bissau	Safim	Count	2017	0	1	0
Guinea-Bissau	Caravela	Proportion	2000	31.8	47.1	19.5
Guinea-Bissau	Caravela	Proportion	2017	42.8	68.2	22.1
Guinea-Bissau	Caravela	Count	2000	0	0	0
Guinea-Bissau	Caravela	Count	2017	0	1	0
Guinea-Bissau	Cacheu	Proportion	2000	30.6	42.8	20.6
Guinea-Bissau	Cacheu	Proportion	2017	32.2	49.4	18.8
Guinea-Bissau	Cacheu	Count	2000	0	0	0
Guinea-Bissau	Cacheu	Count	2017	0	0	0
Guinea-Bissau	Piche	Proportion	2000	45.6	58.3	32.5
Guinea-Bissau	Piche	Proportion	2017	27.4	44.7	14.4
Guinea-Bissau	Piche	Count	2000	0	1	0
Guinea-Bissau	Piche	Count	2017	0	0	0
Guinea-Bissau	Mansaba	Proportion	2000	36.7	48.2	25.1
Guinea-Bissau	Mansaba	Proportion	2017	30.4	48.2	16.4
Guinea-Bissau	Mansaba	Count	2000	0	0	0
Guinea-Bissau	Mansaba	Count	2017	0	0	0
Guinea-Bissau	Fulacunda	Proportion	2000	38.3	52.8	24.7
Guinea-Bissau	Fulacunda	Proportion	2017	38.4	61.3	20.5
Guinea-Bissau	Fulacunda	Count	2000	0	1	0
Guinea-Bissau	Fulacunda	Count	2017	0	1	0
Guinea-Bissau	Catio	Proportion	2000	36.5	53.6	22.0
Guinea-Bissau	Catio	Proportion	2017	44.1	71.1	24.7
Guinea-Bissau	Catio	Count	2000	0	1	0
Guinea-Bissau	Catio	Count	2017	0	1	0
Guinea-Bissau	Galomaro	Proportion	2000	40.6	54.6	27.9
Guinea-Bissau	Galomaro	Proportion	2017	31.8	52.9	15.5
Guinea-Bissau	Galomaro	Count	2000	0	1	0
Guinea-Bissau	Galomaro	Count	2017	0	1	0
Guinea-Bissau	Caio	Proportion	2000	31.8	44.0	21.7
Guinea-Bissau	Caio	Proportion	2017	37.4	56.8	21.1
Guinea-Bissau	Caio	Count	2000	0	0	0
Guinea-Bissau	Caio	Count	2017	0	1	0
Guinea-Bissau	Pirada	Proportion	2000	43.3	56.1	31.0
Guinea-Bissau	Pirada	Proportion	2017	27.4	45.2	15.0
Guinea-Bissau	Pirada	Count	2000	0	1	0
Guinea-Bissau	Pirada	Count	2017	0	0	0
Guinea-Bissau	Mansoa	Proportion	2000	38.0	50.9	25.1
Guinea-Bissau	Mansoa	Proportion	2017	33.2	52.6	17.9
Guinea-Bissau	Mansoa	Count	2000	0	1	0
Guinea-Bissau	Mansoa	Count	2017	0	1	0
Guinea-Bissau	Tite	Proportion	2000	37.0	49.3	24.6

Table 4: ORS by Unit *(continued)*

Country	Unit	Metric	Year	Mean	Upper	Lower
Guinea-Bissau	Tite	Proportion	2017	39.1	61.0	22.4
Guinea-Bissau	Tite	Count	2000	0	0	0
Guinea-Bissau	Tite	Count	2017	0	1	0
Guinea-Bissau	Quebo	Proportion	2000	37.1	53.3	21.2
Guinea-Bissau	Quebo	Proportion	2017	37.4	62.8	18.5
Guinea-Bissau	Quebo	Count	2000	0	1	0
Guinea-Bissau	Quebo	Count	2017	0	1	0
Guinea-Bissau	Gamamundo	Proportion	2000	38.9	50.9	26.7
Guinea-Bissau	Gamamundo	Proportion	2017	31.4	50.4	15.2
Guinea-Bissau	Gamamundo	Count	2000	0	1	0
Guinea-Bissau	Gamamundo	Count	2017	0	1	0
Guinea-Bissau	Canghungo	Proportion	2000	31.6	44.6	20.5
Guinea-Bissau	Canghungo	Proportion	2017	34.4	54.6	18.6
Guinea-Bissau	Canghungo	Count	2000	0	0	0
Guinea-Bissau	Canghungo	Count	2017	0	1	0
Guinea-Bissau	Sonaco	Proportion	2000	44.4	58.1	31.3
Guinea-Bissau	Sonaco	Proportion	2017	29.2	46.5	15.6
Guinea-Bissau	Sonaco	Count	2000	0	1	0
Guinea-Bissau	Sonaco	Count	2017	0	0	0
Guinea-Bissau	Nhacra	Proportion	2000	35.7	47.9	24.3
Guinea-Bissau	Nhacra	Proportion	2017	32.9	52.6	17.7
Guinea-Bissau	Nhacra	Count	2000	0	0	0
Guinea-Bissau	Nhacra	Count	2017	0	1	0
Guinea-Bissau	Xitole	Proportion	2000	38.4	55.0	23.8
Guinea-Bissau	Xitole	Proportion	2017	34.4	57.1	17.1
Guinea-Bissau	Xitole	Count	2000	0	1	0
Guinea-Bissau	Xitole	Count	2017	0	1	0
Guinea-Bissau	Sao Domingos	Proportion	2000	31.0	43.6	20.7
Guinea-Bissau	Sao Domingos	Proportion	2017	31.5	45.5	19.2
Guinea-Bissau	Sao Domingos	Count	2000	0	0	0
Guinea-Bissau	Sao Domingos	Count	2017	0	0	0
Guyana	Barima / Amakura	Proportion	2000	33.0	39.9	24.8
Guyana	Barima / Amakura	Proportion	2017	50.0	60.9	38.7
Guyana	Barima / Amakura	Count	2000	0	0	0
Guyana	Barima / Amakura	Count	2017	0	1	0
Guyana	Agatash	Proportion	2000	39.3	47.6	31.3
Guyana	Agatash	Proportion	2017	52.6	63.8	41.7
Guyana	Agatash	Count	2000	0	0	0
Guyana	Agatash	Count	2017	1	1	0
Guyana	Cane Grove Land Development Scheme	Proportion	2000	33.8	40.8	26.7
Guyana	Cane Grove Land Development Scheme	Proportion	2017	44.0	54.5	31.7
Guyana	Cane Grove Land Development Scheme	Count	2000	0	0	0
Guyana	Cane Grove Land Development Scheme	Count	2017	0	1	0
Guyana	Black Bush Polder land Development Scheme	Proportion	2000	42.6	52.1	34.6

Table 4: ORS by Unit *(continued)*

Country	Unit	Metric	Year	Mean	Upper	Lower
Guyana	Black Bush Polder land Development Scheme	Proportion	2017	52.5	62.9	40.4
Guyana	Black Bush Polder land Development Scheme	Count	2000	0	1	0
Guyana	Black Bush Polder land Development Scheme	Count	2017	1	1	0
Guyana	Amsterdam (Demerara River) / Vriesland	Proportion	2000	36.2	43.2	28.4
Guyana	Amsterdam (Demerara River) / Vriesland	Proportion	2017	42.1	51.8	31.3
Guyana	Amsterdam (Demerara River) / Vriesland	Count	2000	0	0	0
Guyana	Amsterdam (Demerara River) / Vriesland	Count	2017	0	1	0
Guyana	Abary / Mahaicony	Proportion	2000	35.2	42.8	28.3
Guyana	Abary / Mahaicony	Proportion	2017	47.8	59.4	35.4
Guyana	Abary / Mahaicony	Count	2000	0	0	0
Guyana	Abary / Mahaicony	Count	2017	0	1	0
Guyana	Anna Regina	Proportion	2000	42.4	50.9	34.2
Guyana	Anna Regina	Proportion	2017	47.8	59.1	36.1
Guyana	Anna Regina	Count	2000	0	1	0
Guyana	Anna Regina	Count	2017	0	1	0
Guyana	Chenapau River	Proportion	2000	26.4	34.1	19.2
Guyana	Chenapau River	Proportion	2017	40.0	50.1	29.9
Guyana	Chenapau River	Count	2000	0	0	0
Guyana	Chenapau River	Count	2017	0	1	0
Guyana	Berbice River Settlements	Proportion	2000	34.6	42.3	26.9
Guyana	Berbice River Settlements	Proportion	2017	44.7	56.6	34.7
Guyana	Berbice River Settlements	Count	2000	0	0	0
Guyana	Berbice River Settlements	Count	2017	0	1	0
Guyana	Aishalton - Karaudanawa, Achiwib	Proportion	2000	33.7	42.9	25.0
Guyana	Aishalton - Karaudanawa, Achiwib	Proportion	2017	39.1	49.6	29.2
Guyana	Aishalton - Karaudanawa, Achiwib	Count	2000	0	0	0
Guyana	Aishalton - Karaudanawa, Achiwib	Count	2017	0	0	0
Guyana	Mabaruma / Kumaka / Hosororo	Proportion	2000	30.7	38.8	22.1
Guyana	Mabaruma / Kumaka / Hosororo	Proportion	2017	46.4	57.5	34.7
Guyana	Mabaruma / Kumaka / Hosororo	Count	2000	0	0	0
Guyana	Mabaruma / Kumaka / Hosororo	Count	2017	0	1	0
Guyana	Arau	Proportion	2000	32.4	41.0	24.4
Guyana	Arau	Proportion	2017	49.3	60.2	37.6
Guyana	Arau	Count	2000	0	0	0
Guyana	Arau	Count	2017	0	1	0

Table 4: ORS by Unit *(continued)*

Country	Unit	Metric	Year	Mean	Upper	Lower
Guyana	City of Georgetown	Proportion	2000	35.1	42.2	27.3
Guyana	City of Georgetown	Proportion	2017	39.7	49.5	28.9
Guyana	City of Georgetown	Count	2000	0	0	0
Guyana	City of Georgetown	Count	2017	0	0	0
Guyana	Borlam (No.37) / Kintyre	Proportion	2000	40.9	49.6	33.8
Guyana	Borlam (No.37) / Kintyre	Proportion	2017	51.1	62.0	39.1
Guyana	Borlam (No.37) / Kintyre	Count	2000	0	0	0
Guyana	Borlam (No.37) / Kintyre	Count	2017	1	1	0
Guyana	Blankenburg / Hague	Proportion	2000	37.7	45.8	29.8
Guyana	Blankenburg / Hague	Proportion	2017	45.1	55.9	33.4
Guyana	Blankenburg / Hague	Count	2000	0	0	0
Guyana	Blankenburg / Hague	Count	2017	0	1	0
Guyana	Bel Air / Woodlands	Proportion	2000	37.3	45.1	30.2
Guyana	Bel Air / Woodlands	Proportion	2017	46.9	58.3	34.7
Guyana	Bel Air / Woodlands	Count	2000	0	0	0
Guyana	Bel Air / Woodlands	Count	2017	0	1	0
Guyana	Charity / Urasara	Proportion	2000	37.6	45.8	30.2
Guyana	Charity / Urasara	Proportion	2017	44.7	56.1	34.7
Guyana	Charity / Urasara	Count	2000	0	0	0
Guyana	Charity / Urasara	Count	2017	0	1	0
Guyana	Kaibarupai	Proportion	2000	31.7	40.2	23.5
Guyana	Kaibarupai	Proportion	2017	45.4	55.8	34.9
Guyana	Kaibarupai	Count	2000	0	0	0
Guyana	Kaibarupai	Count	2017	0	1	0
Guyana	Coomaka Lands	Proportion	2000	34.4	41.8	26.2
Guyana	Coomaka Lands	Proportion	2017	43.5	54.5	33.5
Guyana	Coomaka Lands	Count	2000	0	0	0
Guyana	Coomaka Lands	Count	2017	0	1	0
Guyana	Ireng / Sawariwau (Including St. Ignatius)	Proportion	2000	35.6	43.9	27.4
Guyana	Ireng / Sawariwau (Including St. Ignatius)	Proportion	2017	40.4	51.5	30.7
Guyana	Ireng / Sawariwau (Including St. Ignatius)	Count	2000	0	0	0
Guyana	Ireng / Sawariwau (Including St. Ignatius)	Count	2017	0	1	0
Guyana	Matthews Ridge / Arakaka (Matakai) / Port Kaituma	Proportion	2000	30.5	37.8	23.4
Guyana	Matthews Ridge / Arakaka (Matakai) / Port Kaituma	Proportion	2017	50.1	62.1	39.4
Guyana	Matthews Ridge / Arakaka (Matakai) / Port Kaituma	Count	2000	0	0	0
Guyana	Matthews Ridge / Arakaka (Matakai) / Port Kaituma	Count	2017	1	1	0
Guyana	Bartica	Proportion	2000	39.4	47.6	31.3
Guyana	Bartica	Proportion	2017	52.7	63.8	41.8
Guyana	Bartica	Count	2000	0	0	0
Guyana	Bartica	Count	2017	1	1	0
Guyana	Demerara Conservancy	Proportion	2000	34.3	41.0	27.0
Guyana	Demerara Conservancy	Proportion	2017	43.6	53.8	32.1

Table 4: ORS by Unit *(continued)*

Country	Unit	Metric	Year	Mean	Upper	Lower
Guyana	Demerara Conservancy	Count	2000	0	0	0
Guyana	Demerara Conservancy	Count	2017	0	1	0
Guyana	Bush Lot / Adventure	Proportion	2000	40.1	49.4	32.0
Guyana	Bush Lot / Adventure	Proportion	2017	54.5	64.7	42.7
Guyana	Bush Lot / Adventure	Count	2000	0	0	0
Guyana	Bush Lot / Adventure	Count	2017	1	1	0
Guyana	Bonasika / Boerasirie	Proportion	2000	37.7	45.7	30.2
Guyana	Bonasika / Boerasirie	Proportion	2017	45.3	55.9	34.1
Guyana	Bonasika / Boerasirie	Count	2000	0	0	0
Guyana	Bonasika / Boerasirie	Count	2017	0	1	0
Guyana	Chance / Hamlet	Proportion	2000	33.5	41.2	26.5
Guyana	Chance / Hamlet	Proportion	2017	44.5	55.1	32.3
Guyana	Chance / Hamlet	Count	2000	0	0	0
Guyana	Chance / Hamlet	Count	2017	0	1	0
Guyana	Good Hope / Pomona	Proportion	2000	36.6	44.9	29.0
Guyana	Good Hope / Pomona	Proportion	2017	43.1	53.3	31.2
Guyana	Good Hope / Pomona	Count	2000	0	0	0
Guyana	Good Hope / Pomona	Count	2017	0	1	0
Guyana	Kopanang, Waipa, Kenepai	Proportion	2000	30.4	38.3	22.3
Guyana	Kopanang, Waipa, Kenepai	Proportion	2017	44.0	54.4	33.7
Guyana	Kopanang, Waipa, Kenepai	Count	2000	0	0	0
Guyana	Kopanang, Waipa, Kenepai	Count	2017	0	1	0
Guyana	Ituni	Proportion	2000	33.8	41.6	25.9
Guyana	Ituni	Proportion	2017	44.1	55.5	34.0
Guyana	Ituni	Count	2000	0	0	0
Guyana	Ituni	Count	2017	0	1	0
Guyana	Marudi	Proportion	2000	32.7	41.5	23.6
Guyana	Marudi	Proportion	2017	39.1	50.2	29.8
Guyana	Marudi	Count	2000	0	0	0
Guyana	Marudi	Count	2017	0	1	0
Guyana	Rest of Region 1	Proportion	2000	31.7	37.7	25.5
Guyana	Rest of Region 1	Proportion	2017	51.1	61.8	40.5
Guyana	Rest of Region 1	Count	2000	0	0	0
Guyana	Rest of Region 1	Count	2017	1	1	0
Guyana	Jawalla, Kubenang River	Proportion	2000	35.8	44.6	27.2
Guyana	Jawalla, Kubenang River	Proportion	2017	58.0	67.1	44.6
Guyana	Jawalla, Kubenang River	Count	2000	0	0	0
Guyana	Jawalla, Kubenang River	Count	2017	1	1	0
Guyana	Diamond / Golden Grove	Proportion	2000	35.3	42.4	27.8
Guyana	Diamond / Golden Grove	Proportion	2017	43.1	53.3	32.0
Guyana	Diamond / Golden Grove	Count	2000	0	0	0
Guyana	Diamond / Golden Grove	Count	2017	0	1	0
Guyana	Cane Field / Enterprise	Proportion	2000	45.1	53.5	37.8
Guyana	Cane Field / Enterprise	Proportion	2017	56.3	66.9	44.3
Guyana	Cane Field / Enterprise	Count	2000	0	1	0
Guyana	Cane Field / Enterprise	Count	2017	1	1	0
Guyana	Canal No. 2 (part) + The Belle + Little Alliance	Proportion	2000	34.9	42.3	27.6

Table 4: ORS by Unit *(continued)*

Country	Unit	Metric	Year	Mean	Upper	Lower
Guyana	Canal No. 2 (part) + The Belle + Little Alliance	Proportion	2017	41.7	51.6	30.9
Guyana	Canal No. 2 (part) + The Belle + Little Alliance	Count	2000	0	0	0
Guyana	Canal No. 2 (part) + The Belle + Little Alliance	Count	2017	0	1	0
Guyana	Farm / Woodlands	Proportion	2000	33.5	40.8	26.4
Guyana	Farm / Woodlands	Proportion	2017	43.8	54.4	31.4
Guyana	Farm / Woodlands	Count	2000	0	0	0
Guyana	Farm / Woodlands	Count	2017	0	1	0
Guyana	Paradise / Evergreen (including Somerset and Berks)	Proportion	2000	43.0	52.5	34.8
Guyana	Paradise / Evergreen (including Somerset and Berks)	Proportion	2017	44.9	56.4	34.1
Guyana	Paradise / Evergreen (including Somerset and Berks)	Count	2000	0	1	0
Guyana	Paradise / Evergreen (including Somerset and Berks)	Count	2017	0	1	0
Guyana	Madhia + Kurubrong River + Mona Falls	Proportion	2000	27.8	36.2	20.4
Guyana	Madhia + Kurubrong River + Mona Falls	Proportion	2017	41.3	52.3	31.0
Guyana	Madhia + Kurubrong River + Mona Falls	Count	2000	0	0	0
Guyana	Madhia + Kurubrong River + Mona Falls	Count	2017	0	1	0
Guyana	Kwakwani	Proportion	2000	32.8	40.9	24.9
Guyana	Kwakwani	Proportion	2017	41.8	53.7	31.0
Guyana	Kwakwani	Count	2000	0	0	0
Guyana	Kwakwani	Count	2017	0	1	0
Guyana	Rest of Region 9	Proportion	2000	35.3	42.7	27.5
Guyana	Rest of Region 9	Proportion	2017	47.1	56.0	38.0
Guyana	Rest of Region 9	Count	2000	0	0	0
Guyana	Rest of Region 9	Count	2017	0	1	0
Guyana	Waini	Proportion	2000	32.7	39.9	25.7
Guyana	Waini	Proportion	2017	46.2	58.0	35.1
Guyana	Waini	Count	2000	0	0	0
Guyana	Waini	Count	2017	0	1	0
Guyana	Kamarang	Proportion	2000	35.0	43.3	26.9
Guyana	Kamarang	Proportion	2017	55.1	64.7	42.8
Guyana	Kamarang	Count	2000	0	0	0
Guyana	Kamarang	Count	2017	1	1	0
Guyana	Eccles / Ramsburg	Proportion	2000	35.3	42.6	27.6
Guyana	Eccles / Ramsburg	Proportion	2017	42.9	53.4	31.5
Guyana	Eccles / Ramsburg	Count	2000	0	0	0
Guyana	Eccles / Ramsburg	Count	2017	0	1	0

Table 4: ORS by Unit *(continued)*

Country	Unit	Metric	Year	Mean	Upper	Lower
Guyana	Corentyne River	Proportion	2000	42.3	51.3	35.1
Guyana	Corentyne River	Proportion	2017	53.6	64.1	41.7
Guyana	Corentyne River	Count	2000	0	1	0
Guyana	Corentyne River	Count	2017	1	1	0
Guyana	Canals Polder	Proportion	2000	34.3	41.7	27.1
Guyana	Canals Polder	Proportion	2017	41.4	51.7	30.3
Guyana	Canals Polder	Count	2000	0	0	0
Guyana	Canals Polder	Count	2017	0	1	0
Guyana	Gelderland / No. 3	Proportion	2000	38.6	46.6	31.1
Guyana	Gelderland / No. 3	Proportion	2017	48.5	59.5	36.5
Guyana	Gelderland / No. 3	Count	2000	0	0	0
Guyana	Gelderland / No. 3	Count	2017	0	1	0
Guyana	Riverstown / Annandale	Proportion	2000	37.7	45.9	29.8
Guyana	Riverstown / Annandale	Proportion	2017	43.3	54.0	32.1
Guyana	Riverstown / Annandale	Count	2000	0	0	0
Guyana	Riverstown / Annandale	Count	2017	0	1	0
Guyana	Maripari River + Kurukabaru	Proportion	2000	33.0	40.9	24.2
Guyana	Maripari River + Kurukabaru	Proportion	2017	46.7	57.2	35.6
Guyana	Maripari River + Kurukabaru	Count	2000	0	0	0
Guyana	Maripari River + Kurukabaru	Count	2017	0	1	0
Guyana	Linden	Proportion	2000	32.8	39.9	24.6
Guyana	Linden	Proportion	2017	40.9	51.8	30.8
Guyana	Linden	Count	2000	0	0	0
Guyana	Linden	Count	2017	0	1	0
Guyana	Sand Creek - Dadanawa, Catunarib, Sawariwau	Proportion	2000	33.9	42.4	26.3
Guyana	Sand Creek - Dadanawa, Catunarib, Sawariwau	Proportion	2017	41.2	52.2	31.0
Guyana	Sand Creek - Dadanawa, Catunarib, Sawariwau	Count	2000	0	0	0
Guyana	Sand Creek - Dadanawa, Catunarib, Sawariwau	Count	2017	0	1	0
Guyana	Karambaru to Kukui River + Phillipi	Proportion	2000	35.8	44.7	27.0
Guyana	Karambaru to Kukui River + Phillipi	Proportion	2017	55.3	64.5	42.2
Guyana	Karambaru to Kukui River + Phillipi	Count	2000	0	0	0
Guyana	Karambaru to Kukui River + Phillipi	Count	2017	1	1	0
Guyana	Enmore / Hope	Proportion	2000	36.0	43.3	28.2
Guyana	Enmore / Hope	Proportion	2017	46.2	57.1	33.7
Guyana	Enmore / Hope	Count	2000	0	0	0
Guyana	Enmore / Hope	Count	2017	0	1	0
Guyana	Corriverton	Proportion	2000	44.2	53.9	35.9
Guyana	Corriverton	Proportion	2017	55.8	67.2	43.5

Table 4: ORS by Unit *(continued)*

Country	Unit	Metric	Year	Mean	Upper	Lower
Guyana	Corriverton	Count	2000	0	1	0
Guyana	Corriverton	Count	2017	1	1	0
Guyana	Cornelia Ida / Stewartville	Proportion	2000	38.0	46.2	30.0
Guyana	Cornelia Ida / Stewartville	Proportion	2017	45.3	56.1	33.5
Guyana	Cornelia Ida / Stewartville	Count	2000	0	0	0
Guyana	Cornelia Ida / Stewartville	Count	2017	0	1	0
Guyana	Naarstigheid / Union	Proportion	2000	36.7	43.8	29.8
Guyana	Naarstigheid / Union	Proportion	2017	47.5	58.7	35.2
Guyana	Naarstigheid / Union	Count	2000	0	0	0
Guyana	Naarstigheid / Union	Count	2017	0	1	0
Guyana	Supernaam River, Bethany and Mashabo Villages	Proportion	2000	34.2	41.4	27.1
Guyana	Supernaam River, Bethany and Mashabo Villages	Proportion	2017	42.5	53.2	31.7
Guyana	Supernaam River, Bethany and Mashabo Villages	Count	2000	0	0	0
Guyana	Supernaam River, Bethany and Mashabo Villages	Count	2017	0	1	0
Guyana	Monkey Mountain	Proportion	2000	32.0	40.5	22.8
Guyana	Monkey Mountain	Proportion	2017	45.9	56.4	34.1
Guyana	Monkey Mountain	Count	2000	0	0	0
Guyana	Monkey Mountain	Count	2017	0	1	0
Guyana	Mabura Hills	Proportion	2000	32.2	40.5	23.9
Guyana	Mabura Hills	Proportion	2017	44.1	55.2	33.6
Guyana	Mabura Hills	Count	2000	0	0	0
Guyana	Mabura Hills	Count	2017	0	1	0
Guyana	Toka - Jakaretinga	Proportion	2000	35.1	42.7	26.4
Guyana	Toka - Jakaretinga	Proportion	2017	44.7	55.6	34.6
Guyana	Toka - Jakaretinga	Count	2000	0	0	0
Guyana	Toka - Jakaretinga	Count	2017	0	1	0
Guyana	Paruima	Proportion	2000	36.1	44.5	27.4
Guyana	Paruima	Proportion	2017	55.1	64.5	43.4
Guyana	Paruima	Count	2000	0	0	0
Guyana	Paruima	Count	2017	1	1	0
Guyana	Foulis / Buxton	Proportion	2000	35.0	42.2	27.8
Guyana	Foulis / Buxton	Proportion	2017	43.9	54.7	31.9
Guyana	Foulis / Buxton	Count	2000	0	0	0
Guyana	Foulis / Buxton	Count	2017	0	1	0
Guyana	East Bank Berbice	Proportion	2000	39.0	49.6	31.6
Guyana	East Bank Berbice	Proportion	2017	57.5	65.8	47.9
Guyana	East Bank Berbice	Count	2000	0	0	0
Guyana	East Bank Berbice	Count	2017	1	1	0
Guyana	Good Hope / Hydronie	Proportion	2000	38.7	46.8	31.4
Guyana	Good Hope / Hydronie	Proportion	2017	46.7	57.5	35.3
Guyana	Good Hope / Hydronie	Count	2000	0	0	0
Guyana	Good Hope / Hydronie	Count	2017	0	1	0
Guyana	Rising Sun / Profit	Proportion	2000	34.5	41.5	27.3
Guyana	Rising Sun / Profit	Proportion	2017	45.8	57.1	33.8
Guyana	Rising Sun / Profit	Count	2000	0	0	0
Guyana	Rising Sun / Profit	Count	2017	0	1	0

Table 4: ORS by Unit *(continued)*

Country	Unit	Metric	Year	Mean	Upper	Lower
Guyana	Zorg-en-Vlygt / Aberdeen	Proportion	2000	36.5	44.8	29.2
Guyana	Zorg-en-Vlygt / Aberdeen	Proportion	2017	43.3	54.0	32.2
Guyana	Zorg-en-Vlygt / Aberdeen	Count	2000	0	0	0
Guyana	Zorg-en-Vlygt / Aberdeen	Count	2017	0	1	0
Guyana	Paramakatoi	Proportion	2000	30.7	39.1	22.6
Guyana	Paramakatoi	Proportion	2017	45.9	56.5	34.8
Guyana	Paramakatoi	Count	2000	0	0	0
Guyana	Paramakatoi	Count	2017	0	1	0
Guyana	Makouria River	Proportion	2000	39.3	47.5	31.2
Guyana	Makouria River	Proportion	2017	52.5	63.7	41.7
Guyana	Makouria River	Count	2000	0	0	0
Guyana	Makouria River	Count	2017	1	1	0
Guyana	Yakarinta - Wowetta, Surama	Proportion	2000	32.2	40.0	23.8
Guyana	Yakarinta - Wowetta, Surama	Proportion	2017	45.6	56.0	35.6
Guyana	Yakarinta - Wowetta, Surama	Count	2000	0	0	0
Guyana	Yakarinta - Wowetta, Surama	Count	2017	0	1	0
Guyana	Rest of Region 7	Proportion	2000	34.3	41.0	27.5
Guyana	Rest of Region 7	Proportion	2017	53.4	62.4	43.0
Guyana	Rest of Region 7	Count	2000	0	0	0
Guyana	Rest of Region 7	Count	2017	1	1	0
Guyana	Good Success / Caledonia	Proportion	2000	34.5	41.2	26.9
Guyana	Good Success / Caledonia	Proportion	2017	40.8	50.6	30.5
Guyana	Good Success / Caledonia	Count	2000	0	0	0
Guyana	Good Success / Caledonia	Count	2017	0	1	0
Guyana	Enfield / New Doe Park	Proportion	2000	39.8	48.2	32.2
Guyana	Enfield / New Doe Park	Proportion	2017	49.2	60.2	37.6
Guyana	Enfield / New Doe Park	Count	2000	0	0	0
Guyana	Enfield / New Doe Park	Count	2017	0	1	0
Guyana	Klein Pouderoyen / Best	Proportion	2000	33.6	41.0	26.3
Guyana	Klein Pouderoyen / Best	Proportion	2017	39.4	49.7	28.5
Guyana	Klein Pouderoyen / Best	Count	2000	0	0	0
Guyana	Klein Pouderoyen / Best	Count	2017	0	0	0
Guyana	Rosignol / Zeelust	Proportion	2000	37.7	45.7	30.4
Guyana	Rosignol / Zeelust	Proportion	2017	47.3	58.6	35.2
Guyana	Rosignol / Zeelust	Count	2000	0	0	0
Guyana	Rosignol / Zeelust	Count	2017	0	1	0
Guyana	Rest of Region 8	Proportion	2000	30.0	37.5	21.3
Guyana	Rest of Region 8	Proportion	2017	44.2	54.1	34.6
Guyana	Rest of Region 8	Count	2000	0	0	0
Guyana	Rest of Region 8	Count	2017	0	1	0
Guyana	Rest of Region 10	Proportion	2000	33.2	40.6	25.7
Guyana	Rest of Region 10	Proportion	2017	44.5	55.4	34.2
Guyana	Rest of Region 10	Count	2000	0	0	0
Guyana	Rest of Region 10	Count	2017	0	1	0
Guyana	Yarong Paru - Good Hope	Proportion	2000	33.2	41.2	24.1

Table 4: ORS by Unit *(continued)*

Country	Unit	Metric	Year	Mean	Upper	Lower
Guyana	Yarong Paru - Good Hope	Proportion	2017	45.5	55.1	35.2
Guyana	Yarong Paru - Good Hope	Count	2000	0	0	0
Guyana	Yarong Paru - Good Hope	Count	2017	0	1	0
Guyana	Waramadan	Proportion	2000	34.1	42.0	25.9
Guyana	Waramadan	Proportion	2017	53.7	63.9	41.4
Guyana	Waramadan	Count	2000	0	0	0
Guyana	Waramadan	Count	2017	1	1	0
Guyana	Grove / Haslington	Proportion	2000	35.4	42.8	27.6
Guyana	Grove / Haslington	Proportion	2017	46.1	57.2	33.6
Guyana	Grove / Haslington	Count	2000	0	0	0
Guyana	Grove / Haslington	Count	2017	0	1	0
Guyana	Fyrish / Gibraltar	Proportion	2000	41.7	50.8	34.5
Guyana	Fyrish / Gibraltar	Proportion	2017	54.6	65.4	42.6
Guyana	Fyrish / Gibraltar	Count	2000	0	1	0
Guyana	Fyrish / Gibraltar	Count	2017	1	1	0
Guyana	Leguan (Essequibo Islands)	Proportion	2000	37.0	45.2	29.6
Guyana	Leguan (Essequibo Islands)	Proportion	2017	45.3	56.0	34.1
Guyana	Leguan (Essequibo Islands)	Count	2000	0	0	0
Guyana	Leguan (Essequibo Islands)	Count	2017	0	1	0
Guyana	St. Francis Mission	Proportion	2000	36.2	43.1	28.8
Guyana	St. Francis Mission	Proportion	2017	45.8	57.5	34.5
Guyana	St. Francis Mission	Count	2000	0	0	0
Guyana	St. Francis Mission	Count	2017	0	1	0
Guyana	Herstelling / Little Diamond	Proportion	2000	36.2	43.4	28.6
Guyana	Herstelling / Little Diamond	Proportion	2017	44.4	54.7	33.3
Guyana	Herstelling / Little Diamond	Count	2000	0	0	0
Guyana	Herstelling / Little Diamond	Count	2017	0	1	0
Guyana	Hampshire / Kilcoy	Proportion	2000	42.6	52.2	34.9
Guyana	Hampshire / Kilcoy	Proportion	2017	54.6	65.2	42.4
Guyana	Hampshire / Kilcoy	Count	2000	0	1	0
Guyana	Hampshire / Kilcoy	Count	2017	1	1	0
Guyana	Lower West Demerara	Proportion	2000	35.4	43.0	28.0
Guyana	Lower West Demerara	Proportion	2017	43.3	54.7	32.4
Guyana	Lower West Demerara	Count	2000	0	0	0
Guyana	Lower West Demerara	Count	2017	0	1	0
Guyana	Tempe / Seafield	Proportion	2000	35.6	42.9	28.5
Guyana	Tempe / Seafield	Proportion	2017	49.0	60.4	36.5
Guyana	Tempe / Seafield	Count	2000	0	0	0
Guyana	Tempe / Seafield	Count	2017	0	1	0
Guyana	La Bonne Intention / Better Hope	Proportion	2000	33.0	39.6	25.8

Table 4: ORS by Unit *(continued)*

Country	Unit	Metric	Year	Mean	Upper	Lower
Guyana	La Bonne Intention / Better Hope	Proportion	2017	41.3	51.6	30.2
Guyana	La Bonne Intention / Better Hope	Count	2000	0	0	0
Guyana	La Bonne Intention / Better Hope	Count	2017	0	1	0
Guyana	Hogstye / Lancaster	Proportion	2000	40.5	49.8	32.8
Guyana	Hogstye / Lancaster	Proportion	2017	52.5	62.9	40.7
Guyana	Hogstye / Lancaster	Count	2000	0	0	0
Guyana	Hogstye / Lancaster	Count	2017	1	1	0
Guyana	Meer Zorgen / Malgre Tout	Proportion	2000	34.6	42.0	27.1
Guyana	Meer Zorgen / Malgre Tout	Proportion	2017	41.5	52.2	30.8
Guyana	Meer Zorgen / Malgre Tout	Count	2000	0	0	0
Guyana	Meer Zorgen / Malgre Tout	Count	2017	0	1	0
Guyana	West bank Berbice	Proportion	2000	38.9	47.3	31.4
Guyana	West bank Berbice	Proportion	2017	48.9	60.0	37.1
Guyana	West bank Berbice	Count	2000	0	0	0
Guyana	West bank Berbice	Count	2017	0	1	0
Guyana	La Reconnaissance / Mon Repos	Proportion	2000	34.0	41.1	26.7
Guyana	La Reconnaissance / Mon Repos	Proportion	2017	42.4	52.9	30.9
Guyana	La Reconnaissance / Mon Repos	Count	2000	0	0	0
Guyana	La Reconnaissance / Mon Repos	Count	2017	0	1	0
Guyana	Jackson Creek / Crabwood Creek	Proportion	2000	40.1	49.4	32.1
Guyana	Jackson Creek / Crabwood Creek	Proportion	2017	51.5	63.9	39.4
Guyana	Jackson Creek / Crabwood Creek	Count	2000	0	0	0
Guyana	Jackson Creek / Crabwood Creek	Count	2017	1	1	0
Guyana	Nismes / La Grange	Proportion	2000	35.9	43.5	28.3
Guyana	Nismes / La Grange	Proportion	2017	43.8	54.3	32.9
Guyana	Nismes / La Grange	Count	2000	0	0	0
Guyana	Nismes / La Grange	Count	2017	0	1	0
Guyana	Woodley Park / Bath	Proportion	2000	37.3	45.1	30.4
Guyana	Woodley Park / Bath	Proportion	2017	46.9	58.3	34.8
Guyana	Woodley Park / Bath	Count	2000	0	0	0
Guyana	Woodley Park / Bath	Count	2017	0	1	0
Guyana	Mocha / Arcadia	Proportion	2000	32.7	39.7	25.1
Guyana	Mocha / Arcadia	Proportion	2017	39.9	50.2	29.1
Guyana	Mocha / Arcadia	Count	2000	0	0	0
Guyana	Mocha / Arcadia	Count	2017	0	1	0

Table 4: ORS by Unit *(continued)*

Country	Unit	Metric	Year	Mean	Upper	Lower
Guyana	John / Port Mourant	Proportion	2000	43.0	52.6	35.1
Guyana	John / Port Mourant	Proportion	2017	55.8	66.2	43.7
Guyana	John / Port Mourant	Count	2000	0	1	0
Guyana	John / Port Mourant	Count	2017	1	1	0
Guyana	Nouvelle Flanders / La Jalousie	Proportion	2000	35.2	42.7	27.6
Guyana	Nouvelle Flanders / La Jalousie	Proportion	2017	43.4	54.1	32.7
Guyana	Nouvelle Flanders / La Jalousie	Count	2000	0	0	0
Guyana	Nouvelle Flanders / La Jalousie	Count	2017	0	1	0
Guyana	Plaisance / Industry	Proportion	2000	33.0	39.6	25.9
Guyana	Plaisance / Industry	Proportion	2017	41.1	51.3	30.1
Guyana	Plaisance / Industry	Count	2000	0	0	0
Guyana	Plaisance / Industry	Count	2017	0	1	0
Guyana	Joppa / Macedonia	Proportion	2000	42.0	51.4	34.2
Guyana	Joppa / Macedonia	Proportion	2017	51.7	62.1	39.9
Guyana	Joppa / Macedonia	Count	2000	0	1	0
Guyana	Joppa / Macedonia	Count	2017	1	1	0
Guyana	Parika / Mora	Proportion	2000	36.5	44.4	28.9
Guyana	Parika / Mora	Proportion	2017	41.4	51.7	30.0
Guyana	Parika / Mora	Count	2000	0	0	0
Guyana	Parika / Mora	Count	2017	0	1	0
Guyana	Soesdyke-Linden highway (including Timehri)	Proportion	2000	38.8	45.8	31.2
Guyana	Soesdyke-Linden highway (including Timehri)	Proportion	2017	47.6	58.2	37.1
Guyana	Soesdyke-Linden highway (including Timehri)	Count	2000	0	0	0
Guyana	Soesdyke-Linden highway (including Timehri)	Count	2017	0	1	0
Guyana	New Amsterdam	Proportion	2000	40.9	49.6	33.1
Guyana	New Amsterdam	Proportion	2017	50.3	61.4	38.2
Guyana	New Amsterdam	Count	2000	0	0	0
Guyana	New Amsterdam	Count	2017	1	1	0
Guyana	Patentia / Toevlugt	Proportion	2000	35.7	43.0	28.1
Guyana	Patentia / Toevlugt	Proportion	2017	43.0	53.1	32.1
Guyana	Patentia / Toevlugt	Count	2000	0	0	0
Guyana	Patentia / Toevlugt	Count	2017	0	1	0
Guyana	St. Cuthberts / Orange Nassau (Mahaica River)	Proportion	2000	35.6	42.5	28.3
Guyana	St. Cuthberts / Orange Nassau (Mahaica River)	Proportion	2017	44.9	56.1	33.7
Guyana	St. Cuthberts / Orange Nassau (Mahaica River)	Count	2000	0	0	0
Guyana	St. Cuthberts / Orange Nassau (Mahaica River)	Count	2017	0	1	0
Guyana	No. 38 / Ordnance Fortlands	Proportion	2000	41.7	50.1	34.3

Table 4: ORS by Unit *(continued)*

Country	Unit	Metric	Year	Mean	Upper	Lower
Guyana	No. 38 / Ordnance Fortlands	Proportion	2017	51.1	61.9	39.0
Guyana	No. 38 / Ordnance Fortlands	Count	2000	0	1	0
Guyana	No. 38 / Ordnance Fortlands	Count	2017	1	1	0
Guyana	Sparta / Bonasika and Rest of Essequibo Islands	Proportion	2000	35.9	43.9	28.7
Guyana	Sparta / Bonasika and Rest of Essequibo Islands	Proportion	2017	42.9	53.8	32.1
Guyana	Sparta / Bonasika and Rest of Essequibo Islands	Count	2000	0	0	0
Guyana	Sparta / Bonasika and Rest of Essequibo Islands	Count	2017	0	1	0
Guyana	Te Huist Coverden / Soesdyke	Proportion	2000	41.6	48.8	34.0
Guyana	Te Huist Coverden / Soesdyke	Proportion	2017	48.7	58.8	37.2
Guyana	Te Huist Coverden / Soesdyke	Count	2000	0	0	0
Guyana	Te Huist Coverden / Soesdyke	Count	2017	0	1	0
Guyana	No.51 Village / Good Hope	Proportion	2000	40.5	50.0	32.9
Guyana	No.51 Village / Good Hope	Proportion	2017	50.6	61.1	38.8
Guyana	No.51 Village / Good Hope	Count	2000	0	0	0
Guyana	No.51 Village / Good Hope	Count	2017	1	1	0
Guyana	Uitvlugt / Tuschen	Proportion	2000	37.8	46.1	29.9
Guyana	Uitvlugt / Tuschen	Proportion	2017	45.9	56.7	34.3
Guyana	Uitvlugt / Tuschen	Count	2000	0	0	0
Guyana	Uitvlugt / Tuschen	Count	2017	0	1	0
Guyana	Triumph / Beterverwagting	Proportion	2000	32.9	39.6	25.4
Guyana	Triumph / Beterverwagting	Proportion	2017	41.0	51.2	29.8
Guyana	Triumph / Beterverwagting	Count	2000	0	0	0
Guyana	Triumph / Beterverwagting	Count	2017	0	1	0
Guyana	No.74 Village / No.52 Village	Proportion	2000	41.4	51.5	33.4
Guyana	No.74 Village / No.52 Village	Proportion	2017	52.6	63.5	40.5
Guyana	No.74 Village / No.52 Village	Count	2000	0	1	0
Guyana	No.74 Village / No.52 Village	Count	2017	1	1	0
Guyana	Vergenoegen / Greenwich Park	Proportion	2000	38.5	46.9	31.1
Guyana	Vergenoegen / Greenwich Park	Proportion	2017	48.3	59.3	37.0
Guyana	Vergenoegen / Greenwich Park	Count	2000	0	0	0

Table 4: ORS by Unit *(continued)*

Country	Unit	Metric	Year	Mean	Upper	Lower
Guyana	Vergenoegen / Greenwich Park	Count	2017	0	1	0
Guyana	Vereeniging / Unity	Proportion	2000	34.3	41.1	27.3
Guyana	Vereeniging / Unity	Proportion	2017	45.5	56.1	33.0
Guyana	Vereeniging / Unity	Count	2000	0	0	0
Guyana	Vereeniging / Unity	Count	2017	0	1	0
Guyana	Rose Hall	Proportion	2000	42.9	52.6	35.0
Guyana	Rose Hall	Proportion	2017	55.3	65.7	43.1
Guyana	Rose Hall	Count	2000	0	1	0
Guyana	Rose Hall	Count	2017	1	1	0
Guyana	Wakenaam (Essequibo Islands)	Proportion	2000	37.1	45.1	29.9
Guyana	Wakenaam (Essequibo Islands)	Proportion	2017	43.9	54.3	32.5
Guyana	Wakenaam (Essequibo Islands)	Count	2000	0	0	0
Guyana	Wakenaam (Essequibo Islands)	Count	2017	0	1	0
Guyana	Tarlogie / Maida	Proportion	2000	41.1	50.4	33.0
Guyana	Tarlogie / Maida	Proportion	2017	52.9	63.0	41.2
Guyana	Tarlogie / Maida	Count	2000	0	1	0
Guyana	Tarlogie / Maida	Count	2017	1	1	0
Guyana	Whim / Bloomfield	Proportion	2000	40.4	49.7	32.7
Guyana	Whim / Bloomfield	Proportion	2017	52.8	63.3	41.0
Guyana	Whim / Bloomfield	Count	2000	0	0	0
Guyana	Whim / Bloomfield	Count	2017	1	1	0
Haiti	Cerca La Source	Proportion	2000	34.9	45.3	26.1
Haiti	Cerca La Source	Proportion	2017	44.3	55.8	31.7
Haiti	Cerca La Source	Count	2000	0	0	0
Haiti	Cerca La Source	Count	2017	0	1	0
Haiti	Anse d'Hainault	Proportion	2000	33.4	43.6	22.1
Haiti	Anse d'Hainault	Proportion	2017	43.5	57.1	31.4
Haiti	Anse d'Hainault	Count	2000	0	0	0
Haiti	Anse d'Hainault	Count	2017	0	1	0
Haiti	Dessalines	Proportion	2000	40.7	51.8	31.4
Haiti	Dessalines	Proportion	2017	37.6	48.4	26.7
Haiti	Dessalines	Count	2000	0	1	0
Haiti	Dessalines	Count	2017	0	0	0
Haiti	l'Anse-à-Veau	Proportion	2000	31.3	40.5	22.1
Haiti	l'Anse-à-Veau	Proportion	2017	36.5	48.4	26.3
Haiti	l'Anse-à-Veau	Count	2000	0	0	0
Haiti	l'Anse-à-Veau	Count	2017	0	0	0
Haiti	Fort-Liberté	Proportion	2000	35.8	45.7	27.3
Haiti	Fort-Liberté	Proportion	2017	42.8	55.6	30.0
Haiti	Fort-Liberté	Count	2000	0	0	0
Haiti	Fort-Liberté	Count	2017	0	1	0
Haiti	Môle Saint-Nicolas	Proportion	2000	36.0	46.7	25.2
Haiti	Môle Saint-Nicolas	Proportion	2017	34.0	47.0	23.3
Haiti	Môle Saint-Nicolas	Count	2000	0	0	0
Haiti	Môle Saint-Nicolas	Count	2017	0	0	0

Table 4: ORS by Unit *(continued)*

Country	Unit	Metric	Year	Mean	Upper	Lower
Haiti	Borgne	Proportion	2000	35.6	44.2	27.7
Haiti	Borgne	Proportion	2017	36.3	46.7	25.4
Haiti	Borgne	Count	2000	0	0	0
Haiti	Borgne	Count	2017	0	0	0
Haiti	Croix-des-Bouquets	Proportion	2000	38.0	48.1	28.7
Haiti	Croix-des-Bouquets	Proportion	2017	42.1	53.2	31.7
Haiti	Croix-des-Bouquets	Count	2000	0	0	0
Haiti	Croix-des-Bouquets	Count	2017	0	1	0
Haiti	Bainet	Proportion	2000	33.9	44.5	23.7
Haiti	Bainet	Proportion	2017	35.6	48.1	24.5
Haiti	Bainet	Count	2000	0	0	0
Haiti	Bainet	Count	2017	0	0	0
Haiti	Aquin	Proportion	2000	31.0	40.6	22.0
Haiti	Aquin	Proportion	2017	36.3	48.2	26.1
Haiti	Aquin	Count	2000	0	0	0
Haiti	Aquin	Count	2017	0	0	0
Haiti	Hinche	Proportion	2000	37.4	46.7	28.3
Haiti	Hinche	Proportion	2017	42.1	53.4	30.2
Haiti	Hinche	Count	2000	0	0	0
Haiti	Hinche	Count	2017	0	1	0
Haiti	Corail	Proportion	2000	30.2	39.4	20.8
Haiti	Corail	Proportion	2017	38.7	49.8	27.8
Haiti	Corail	Count	2000	0	0	0
Haiti	Corail	Count	2017	0	0	0
Haiti	Gros-Morne	Proportion	2000	35.4	45.7	26.9
Haiti	Gros-Morne	Proportion	2017	33.2	44.2	22.2
Haiti	Gros-Morne	Count	2000	0	0	0
Haiti	Gros-Morne	Count	2017	0	0	0
Haiti	Miragoâne	Proportion	2000	35.2	45.9	25.5
Haiti	Miragoâne	Proportion	2017	36.5	50.6	25.8
Haiti	Miragoâne	Count	2000	0	0	0
Haiti	Miragoâne	Count	2017	0	1	0
Haiti	le Trou-du-Nord	Proportion	2000	36.4	46.0	28.0
Haiti	le Trou-du-Nord	Proportion	2017	40.8	53.8	29.1
Haiti	le Trou-du-Nord	Count	2000	0	0	0
Haiti	le Trou-du-Nord	Count	2017	0	1	0
Haiti	Port-de-Paix	Proportion	2000	34.6	45.2	26.2
Haiti	Port-de-Paix	Proportion	2017	32.3	43.7	21.1
Haiti	Port-de-Paix	Count	2000	0	0	0
Haiti	Port-de-Paix	Count	2017	0	0	0
Haiti	Grande-Rivière du Nord	Proportion	2000	37.0	46.0	28.4
Haiti	Grande-Rivière du Nord	Proportion	2017	39.1	51.5	27.4
Haiti	Grande-Rivière du Nord	Count	2000	0	0	0
Haiti	Grande-Rivière du Nord	Count	2017	0	1	0
Haiti	l'Arcahaie	Proportion	2000	42.3	54.1	30.9
Haiti	l'Arcahaie	Proportion	2017	40.2	53.5	28.3
Haiti	l'Arcahaie	Count	2000	0	1	0
Haiti	l'Arcahaie	Count	2017	0	1	0
Haiti	Belle-Anse	Proportion	2000	32.4	43.2	23.0

Table 4: ORS by Unit *(continued)*

Country	Unit	Metric	Year	Mean	Upper	Lower
Haiti	Belle-Anse	Proportion	2017	38.5	49.9	28.3
Haiti	Belle-Anse	Count	2000	0	0	0
Haiti	Belle-Anse	Count	2017	0	0	0
Haiti	les Cayes	Proportion	2000	29.0	38.3	20.4
Haiti	les Cayes	Proportion	2017	37.1	48.5	27.1
Haiti	les Cayes	Count	2000	0	0	0
Haiti	les Cayes	Count	2017	0	0	0
Haiti	Lascahobas	Proportion	2000	35.1	45.2	26.0
Haiti	Lascahobas	Proportion	2017	43.3	54.3	31.5
Haiti	Lascahobas	Count	2000	0	0	0
Haiti	Lascahobas	Count	2017	0	1	0
Haiti	Jérémie	Proportion	2000	33.2	44.1	22.7
Haiti	Jérémie	Proportion	2017	41.8	54.3	29.8
Haiti	Jérémie	Count	2000	0	0	0
Haiti	Jérémie	Count	2017	0	1	0
Haiti	les Gonaïves	Proportion	2000	40.0	49.9	30.1
Haiti	les Gonaïves	Proportion	2017	35.9	48.4	23.3
Haiti	les Gonaïves	Count	2000	0	0	0
Haiti	les Gonaïves	Count	2017	0	0	0
Haiti	Ouanaminthe	Proportion	2000	35.8	45.2	26.4
Haiti	Ouanaminthe	Proportion	2017	44.6	56.7	31.5
Haiti	Ouanaminthe	Count	2000	0	0	0
Haiti	Ouanaminthe	Count	2017	0	1	0
Haiti	Saint-Louis du Nord	Proportion	2000	33.3	43.9	25.0
Haiti	Saint-Louis du Nord	Proportion	2017	31.9	43.7	21.8
Haiti	Saint-Louis du Nord	Count	2000	0	0	0
Haiti	Saint-Louis du Nord	Count	2017	0	0	0
Haiti	l'Acul-du-Nord	Proportion	2000	36.3	45.5	27.1
Haiti	l'Acul-du-Nord	Proportion	2017	36.6	49.4	24.7
Haiti	l'Acul-du-Nord	Count	2000	0	0	0
Haiti	l'Acul-du-Nord	Count	2017	0	0	0
Haiti	La Gonâve	Proportion	2000	41.0	53.5	29.1
Haiti	La Gonâve	Proportion	2017	36.8	51.3	24.0
Haiti	La Gonâve	Count	2000	0	1	0
Haiti	La Gonâve	Count	2017	0	1	0
Haiti	Jacmel	Proportion	2000	34.9	46.4	24.3
Haiti	Jacmel	Proportion	2017	37.0	47.2	25.5
Haiti	Jacmel	Count	2000	0	0	0
Haiti	Jacmel	Count	2017	0	0	0
Haiti	les Chardonnières	Proportion	2000	30.6	40.5	20.7
Haiti	les Chardonnières	Proportion	2017	41.3	54.5	29.5
Haiti	les Chardonnières	Count	2000	0	0	0
Haiti	les Chardonnières	Count	2017	0	1	0
Haiti	Mirebalais	Proportion	2000	38.1	48.6	28.5
Haiti	Mirebalais	Proportion	2017	41.8	53.0	30.5
Haiti	Mirebalais	Count	2000	0	0	0
Haiti	Mirebalais	Count	2017	0	1	0
Haiti	Marmelade	Proportion	2000	38.2	47.5	28.9
Haiti	Marmelade	Proportion	2017	37.5	49.0	25.8

Table 4: ORS by Unit *(continued)*

Country	Unit	Metric	Year	Mean	Upper	Lower
Haiti	Marmelade	Count	2000	0	0	0
Haiti	Marmelade	Count	2017	0	0	0
Haiti	Vallières	Proportion	2000	35.5	45.2	26.5
Haiti	Vallières	Proportion	2017	43.0	56.2	30.0
Haiti	Vallières	Count	2000	0	0	0
Haiti	Vallières	Count	2017	0	1	0
Haiti	le Cap-Haïtien	Proportion	2000	37.3	48.6	27.5
Haiti	le Cap-Haïtien	Proportion	2017	37.6	51.2	25.1
Haiti	le Cap-Haïtien	Count	2000	0	0	0
Haiti	le Cap-Haïtien	Count	2017	0	1	0
Haiti	Léogâne	Proportion	2000	37.4	47.0	27.7
Haiti	Léogâne	Proportion	2017	36.6	48.5	25.5
Haiti	Léogâne	Count	2000	0	0	0
Haiti	Léogâne	Count	2017	0	0	0
Haiti	les Côteaux	Proportion	2000	29.8	40.4	20.3
Haiti	les Côteaux	Proportion	2017	39.6	51.2	27.5
Haiti	les Côteaux	Count	2000	0	0	0
Haiti	les Côteaux	Count	2017	0	1	0
Haiti	Saint-Marc	Proportion	2000	42.2	52.6	31.9
Haiti	Saint-Marc	Proportion	2017	38.2	50.4	26.2
Haiti	Saint-Marc	Count	2000	0	1	0
Haiti	Saint-Marc	Count	2017	0	1	0
Haiti	le Limbé	Proportion	2000	35.5	46.2	26.0
Haiti	le Limbé	Proportion	2017	34.2	46.2	22.2
Haiti	le Limbé	Count	2000	0	0	0
Haiti	le Limbé	Count	2017	0	0	0
Haiti	Port-au-Prince	Proportion	2000	42.7	54.9	32.0
Haiti	Port-au-Prince	Proportion	2017	42.9	54.3	30.3
Haiti	Port-au-Prince	Count	2000	0	1	0
Haiti	Port-au-Prince	Count	2017	0	1	0
Haiti	Port-Salut	Proportion	2000	28.8	39.1	19.6
Haiti	Port-Salut	Proportion	2017	38.2	49.9	25.7
Haiti	Port-Salut	Count	2000	0	0	0
Haiti	Port-Salut	Count	2017	0	0	0
Haiti	Plaisance	Proportion	2000	35.4	45.3	26.7
Haiti	Plaisance	Proportion	2017	33.9	45.6	22.1
Haiti	Plaisance	Count	2000	0	0	0
Haiti	Plaisance	Count	2017	0	0	0
Haiti	Saint-Raphaël	Proportion	2000	37.8	47.5	29.2
Haiti	Saint-Raphaël	Proportion	2017	39.3	51.3	27.1
Haiti	Saint-Raphaël	Count	2000	0	0	0
Haiti	Saint-Raphaël	Count	2017	0	1	0
Honduras	Arizona	Proportion	2000	52.6	71.0	34.9
Honduras	Arizona	Proportion	2017	51.3	70.6	31.4
Honduras	Arizona	Count	2000	1	1	0
Honduras	Arizona	Count	2017	1	1	0
Honduras	Apacilagua	Proportion	2000	60.4	75.8	41.2
Honduras	Apacilagua	Proportion	2017	60.2	76.0	42.4
Honduras	Apacilagua	Count	2000	1	1	0

Table 4: ORS by Unit *(continued)*

Country	Unit	Metric	Year	Mean	Upper	Lower
Honduras	Apacilagua	Count	2017	1	1	0
Honduras	Balfate	Proportion	2000	55.0	73.0	34.4
Honduras	Balfate	Proportion	2017	52.0	70.7	32.0
Honduras	Balfate	Count	2000	1	1	0
Honduras	Balfate	Count	2017	1	1	0
Honduras	Ajuterique	Proportion	2000	58.4	76.8	39.7
Honduras	Ajuterique	Proportion	2017	60.0	77.6	39.5
Honduras	Ajuterique	Count	2000	1	1	0
Honduras	Ajuterique	Count	2017	1	1	0
Honduras	Cabañas	Proportion	2000	53.8	72.9	34.6
Honduras	Cabañas	Proportion	2017	58.2	75.8	38.5
Honduras	Cabañas	Count	2000	1	1	0
Honduras	Cabañas	Count	2017	1	1	0
Honduras	Choloma	Proportion	2000	52.5	70.3	34.1
Honduras	Choloma	Proportion	2017	53.1	72.0	31.9
Honduras	Choloma	Count	2000	1	1	0
Honduras	Choloma	Count	2017	1	1	0
Honduras	Alauca	Proportion	2000	62.9	77.3	44.4
Honduras	Alauca	Proportion	2017	59.9	76.7	42.9
Honduras	Alauca	Count	2000	1	1	0
Honduras	Alauca	Count	2017	1	1	0
Honduras	Alubarén	Proportion	2000	58.9	75.5	38.0
Honduras	Alubarén	Proportion	2017	60.4	77.2	40.3
Honduras	Alubarén	Count	2000	1	1	0
Honduras	Alubarén	Count	2017	1	1	0
Honduras	Ahuas	Proportion	2000	52.8	69.7	33.5
Honduras	Ahuas	Proportion	2017	52.4	70.3	33.4
Honduras	Ahuas	Count	2000	1	1	0
Honduras	Ahuas	Count	2017	1	1	0
Honduras	Camasca	Proportion	2000	58.7	76.8	41.3
Honduras	Camasca	Proportion	2017	65.8	81.5	45.1
Honduras	Camasca	Count	2000	1	1	0
Honduras	Camasca	Count	2017	1	1	0
Honduras	Guanaja	Proportion	2000	52.6	70.3	28.3
Honduras	Guanaja	Proportion	2017	51.2	70.4	28.7
Honduras	Guanaja	Count	2000	1	1	0
Honduras	Guanaja	Count	2017	1	1	0
Honduras	Aguaqueterique	Proportion	2000	60.0	75.6	42.4
Honduras	Aguaqueterique	Proportion	2017	62.0	76.8	43.6
Honduras	Aguaqueterique	Count	2000	1	1	0
Honduras	Aguaqueterique	Count	2017	1	1	0
Honduras	Belen	Proportion	2000	55.8	75.0	38.6
Honduras	Belen	Proportion	2017	61.6	77.8	42.2
Honduras	Belen	Count	2000	1	1	0
Honduras	Belen	Count	2017	1	1	0
Honduras	Belén Gualcho	Proportion	2000	54.3	75.5	34.0
Honduras	Belén Gualcho	Proportion	2017	60.6	76.9	42.4
Honduras	Belén Gualcho	Count	2000	1	1	0
Honduras	Belén Gualcho	Count	2017	1	1	0

Table 4: ORS by Unit *(continued)*

Country	Unit	Metric	Year	Mean	Upper	Lower
Honduras	Campamento	Proportion	2000	59.5	76.2	39.1
Honduras	Campamento	Proportion	2017	55.1	74.4	37.5
Honduras	Campamento	Count	2000	1	1	0
Honduras	Campamento	Count	2017	1	1	0
Honduras	Arada	Proportion	2000	54.0	74.1	36.3
Honduras	Arada	Proportion	2017	58.9	76.1	39.7
Honduras	Arada	Count	2000	1	1	0
Honduras	Arada	Count	2017	1	1	0
Honduras	Alianza	Proportion	2000	57.7	73.8	39.3
Honduras	Alianza	Proportion	2017	61.2	77.7	42.5
Honduras	Alianza	Count	2000	1	1	0
Honduras	Alianza	Count	2017	1	1	0
Honduras	Arenal	Proportion	2000	55.3	73.1	34.5
Honduras	Arenal	Proportion	2017	53.9	74.8	33.8
Honduras	Arenal	Count	2000	1	1	0
Honduras	Arenal	Count	2017	1	1	0
Honduras	El Porvenir	Proportion	2000	52.1	71.8	32.6
Honduras	El Porvenir	Proportion	2017	50.0	69.0	31.3
Honduras	El Porvenir	Count	2000	1	1	0
Honduras	El Porvenir	Count	2017	0	1	0
Honduras	Choluteca	Proportion	2000	57.7	74.1	39.3
Honduras	Choluteca	Proportion	2017	59.8	77.1	42.4
Honduras	Choluteca	Count	2000	1	1	0
Honduras	Choluteca	Count	2017	1	1	0
Honduras	Bonito Oriental	Proportion	2000	56.4	74.2	35.1
Honduras	Bonito Oriental	Proportion	2017	54.7	73.6	34.3
Honduras	Bonito Oriental	Count	2000	1	1	0
Honduras	Bonito Oriental	Count	2017	1	1	0
Honduras	Comayagua	Proportion	2000	58.9	76.4	40.3
Honduras	Comayagua	Proportion	2017	59.9	78.1	41.4
Honduras	Comayagua	Count	2000	1	1	0
Honduras	Comayagua	Count	2017	1	1	0
Honduras	Concepción	Proportion	2000	53.4	71.9	34.8
Honduras	Concepción	Proportion	2017	57.7	74.1	38.1
Honduras	Concepción	Count	2000	1	1	0
Honduras	Concepción	Count	2017	1	1	0
Honduras	La Lima	Proportion	2000	53.6	72.0	34.8
Honduras	La Lima	Proportion	2017	53.8	74.0	33.5
Honduras	La Lima	Count	2000	1	1	0
Honduras	La Lima	Count	2017	1	1	0
Honduras	Danlí	Proportion	2000	61.6	76.8	44.1
Honduras	Danlí	Proportion	2017	56.4	75.5	38.3
Honduras	Danlí	Count	2000	1	1	0
Honduras	Danlí	Count	2017	1	1	0
Honduras	Cedros	Proportion	2000	58.3	75.6	39.8
Honduras	Cedros	Proportion	2017	57.1	75.1	37.2
Honduras	Cedros	Count	2000	1	1	0
Honduras	Cedros	Count	2017	1	1	0
Honduras	Brus Laguna	Proportion	2000	53.2	70.2	34.2

Table 4: ORS by Unit *(continued)*

Country	Unit	Metric	Year	Mean	Upper	Lower
Honduras	Brus Laguna	Proportion	2017	53.0	69.6	32.8
Honduras	Brus Laguna	Count	2000	1	1	0
Honduras	Brus Laguna	Count	2017	1	1	0
Honduras	Colomoncagua	Proportion	2000	59.2	76.7	40.7
Honduras	Colomoncagua	Proportion	2017	65.7	81.4	44.9
Honduras	Colomoncagua	Count	2000	1	1	0
Honduras	Colomoncagua	Count	2017	1	1	0
Honduras	José Santos Guardiola	Proportion	2000	51.8	74.3	27.4
Honduras	José Santos Guardiola	Proportion	2017	49.3	71.7	28.2
Honduras	José Santos Guardiola	Count	2000	1	1	0
Honduras	José Santos Guardiola	Count	2017	0	1	0
Honduras	Cabañas	Proportion	2000	58.8	74.8	40.1
Honduras	Cabañas	Proportion	2017	64.2	80.2	44.8
Honduras	Cabañas	Count	2000	1	1	0
Honduras	Cabañas	Count	2017	1	1	0
Honduras	Candelaria	Proportion	2000	58.4	77.0	37.2
Honduras	Candelaria	Proportion	2017	65.3	80.9	48.9
Honduras	Candelaria	Count	2000	1	1	0
Honduras	Candelaria	Count	2017	1	1	0
Honduras	Concepción	Proportion	2000	56.2	74.4	37.7
Honduras	Concepción	Proportion	2017	61.7	78.0	43.5
Honduras	Concepción	Count	2000	1	1	0
Honduras	Concepción	Count	2017	1	1	0
Honduras	Catacamas	Proportion	2000	56.9	72.5	38.7
Honduras	Catacamas	Proportion	2017	52.5	69.1	33.7
Honduras	Catacamas	Count	2000	1	1	0
Honduras	Catacamas	Count	2017	1	1	0
Honduras	Atima	Proportion	2000	54.5	73.6	37.1
Honduras	Atima	Proportion	2017	59.1	77.3	39.7
Honduras	Atima	Count	2000	1	1	0
Honduras	Atima	Count	2017	1	1	0
Honduras	Amapala	Proportion	2000	57.3	73.0	36.4
Honduras	Amapala	Proportion	2017	61.0	79.8	42.1
Honduras	Amapala	Count	2000	1	1	0
Honduras	Amapala	Count	2017	1	1	0
Honduras	El Negrito	Proportion	2000	53.3	71.5	33.7
Honduras	El Negrito	Proportion	2017	53.6	72.3	34.3
Honduras	El Negrito	Count	2000	1	1	0
Honduras	El Negrito	Count	2017	1	1	0
Honduras	Esparta	Proportion	2000	52.1	69.9	35.1
Honduras	Esparta	Proportion	2017	50.6	69.3	30.9
Honduras	Esparta	Count	2000	1	1	0
Honduras	Esparta	Count	2017	1	1	0
Honduras	Concepción de Maria	Proportion	2000	58.4	75.4	39.0
Honduras	Concepción de Maria	Proportion	2017	59.9	77.0	42.4
Honduras	Concepción de Maria	Count	2000	1	1	0
Honduras	Concepción de Maria	Count	2017	1	1	0
Honduras	Iriona	Proportion	2000	54.6	71.5	35.2
Honduras	Iriona	Proportion	2017	54.0	71.6	34.3

Table 4: ORS by Unit *(continued)*

Country	Unit	Metric	Year	Mean	Upper	Lower
Honduras	Iriona	Count	2000	1	1	0
Honduras	Iriona	Count	2017	1	1	0
Honduras	El Rosario	Proportion	2000	57.3	76.5	37.5
Honduras	El Rosario	Proportion	2017	59.8	77.7	38.5
Honduras	El Rosario	Count	2000	1	1	0
Honduras	El Rosario	Count	2017	1	1	0
Honduras	Copán Ruinas	Proportion	2000	52.2	71.6	34.7
Honduras	Copán Ruinas	Proportion	2017	56.4	73.7	36.9
Honduras	Copán Ruinas	Count	2000	1	1	0
Honduras	Copán Ruinas	Count	2017	1	1	0
Honduras	Omoa	Proportion	2000	52.5	71.5	34.2
Honduras	Omoa	Proportion	2017	53.3	73.2	31.7
Honduras	Omoa	Count	2000	1	1	0
Honduras	Omoa	Count	2017	1	1	0
Honduras	El Paraíso	Proportion	2000	63.1	79.1	45.9
Honduras	El Paraíso	Proportion	2017	58.9	78.6	41.0
Honduras	El Paraíso	Count	2000	1	1	0
Honduras	El Paraíso	Count	2017	1	1	0
Honduras	Curarén	Proportion	2000	59.1	75.3	41.6
Honduras	Curarén	Proportion	2017	61.5	77.0	43.1
Honduras	Curarén	Count	2000	1	1	0
Honduras	Curarén	Count	2017	1	1	0
Honduras	Juan Francisco Bulnes	Proportion	2000	54.3	72.4	34.5
Honduras	Juan Francisco Bulnes	Proportion	2017	53.9	72.3	34.6
Honduras	Juan Francisco Bulnes	Count	2000	1	1	0
Honduras	Juan Francisco Bulnes	Count	2017	1	1	0
Honduras	Concepción	Proportion	2000	59.0	75.3	40.1
Honduras	Concepción	Proportion	2017	65.0	80.1	45.8
Honduras	Concepción	Count	2000	1	1	0
Honduras	Concepción	Count	2017	1	1	0
Honduras	Roatán	Proportion	2000	51.4	72.8	27.3
Honduras	Roatán	Proportion	2017	49.4	70.9	29.1
Honduras	Roatán	Count	2000	1	1	0
Honduras	Roatán	Count	2017	0	1	0
Honduras	Cane	Proportion	2000	60.7	77.7	41.7
Honduras	Cane	Proportion	2017	61.5	79.1	41.5
Honduras	Cane	Count	2000	1	1	0
Honduras	Cane	Count	2017	1	1	0
Honduras	Cololaca	Proportion	2000	56.0	74.4	36.3
Honduras	Cololaca	Proportion	2017	62.8	78.8	46.0
Honduras	Cololaca	Count	2000	1	1	0
Honduras	Cololaca	Count	2017	1	1	0
Honduras	Dolores Merendon	Proportion	2000	55.1	74.5	34.5
Honduras	Dolores Merendon	Proportion	2017	61.4	77.9	40.9
Honduras	Dolores Merendon	Count	2000	1	1	0
Honduras	Dolores Merendon	Count	2017	1	1	0
Honduras	Concordia	Proportion	2000	58.7	75.0	39.8
Honduras	Concordia	Proportion	2017	54.7	74.1	35.6
Honduras	Concordia	Count	2000	1	1	0

Table 4: ORS by Unit *(continued)*

Country	Unit	Metric	Year	Mean	Upper	Lower
Honduras	Concordia	Count	2017	1	1	0
Honduras	Azacualpa	Proportion	2000	53.2	73.9	32.9
Honduras	Azacualpa	Proportion	2017	55.7	74.8	37.2
Honduras	Azacualpa	Count	2000	1	1	0
Honduras	Azacualpa	Count	2017	1	1	0
Honduras	Aramecina	Proportion	2000	57.4	73.3	39.6
Honduras	Aramecina	Proportion	2017	60.0	77.4	42.8
Honduras	Aramecina	Count	2000	1	1	0
Honduras	Aramecina	Count	2017	1	1	0
Honduras	El Progreso	Proportion	2000	53.3	71.6	34.0
Honduras	El Progreso	Proportion	2017	54.1	74.1	34.4
Honduras	El Progreso	Count	2000	1	1	0
Honduras	El Progreso	Count	2017	1	1	0
Honduras	Jutiapa	Proportion	2000	54.6	72.5	35.0
Honduras	Jutiapa	Proportion	2017	52.5	71.7	32.7
Honduras	Jutiapa	Count	2000	1	1	0
Honduras	Jutiapa	Count	2017	1	1	0
Honduras	Duyure	Proportion	2000	63.0	77.9	45.2
Honduras	Duyure	Proportion	2017	61.2	78.5	43.6
Honduras	Duyure	Count	2000	1	1	0
Honduras	Duyure	Count	2017	1	1	0
Honduras	Limón	Proportion	2000	56.0	73.7	34.8
Honduras	Limón	Proportion	2017	55.2	73.9	34.7
Honduras	Limón	Count	2000	1	1	0
Honduras	Limón	Count	2017	1	1	0
Honduras	Esquías	Proportion	2000	57.5	74.1	37.9
Honduras	Esquías	Proportion	2017	57.9	76.6	38.6
Honduras	Esquías	Count	2000	1	1	0
Honduras	Esquías	Count	2017	1	1	0
Honduras	Corquín	Proportion	2000	55.0	74.4	34.0
Honduras	Corquín	Proportion	2017	60.4	77.3	41.0
Honduras	Corquín	Count	2000	1	1	0
Honduras	Corquín	Count	2017	1	1	0
Honduras	Pimienta	Proportion	2000	53.1	72.8	33.2
Honduras	Pimienta	Proportion	2017	54.5	75.4	36.2
Honduras	Pimienta	Count	2000	1	1	0
Honduras	Pimienta	Count	2017	1	1	0
Honduras	Guinope	Proportion	2000	61.7	77.4	42.0
Honduras	Guinope	Proportion	2017	59.6	76.5	42.3
Honduras	Guinope	Count	2000	1	1	0
Honduras	Guinope	Count	2017	1	1	0
Honduras	Distrito Central	Proportion	2000	59.6	75.7	40.7
Honduras	Distrito Central	Proportion	2017	58.3	74.6	36.8
Honduras	Distrito Central	Count	2000	1	1	0
Honduras	Distrito Central	Count	2017	1	1	0
Honduras	Puerto Lempira	Proportion	2000	51.9	67.8	34.2
Honduras	Puerto Lempira	Proportion	2017	50.8	69.5	30.4
Honduras	Puerto Lempira	Count	2000	1	1	0
Honduras	Puerto Lempira	Count	2017	1	1	0

Table 4: ORS by Unit *(continued)*

Country	Unit	Metric	Year	Mean	Upper	Lower
Honduras	Dolores	Proportion	2000	58.1	76.2	39.5
Honduras	Dolores	Proportion	2017	64.7	80.4	46.7
Honduras	Dolores	Count	2000	1	1	0
Honduras	Dolores	Count	2017	1	1	0
Honduras	Utila	Proportion	2000	51.1	72.4	27.9
Honduras	Utila	Proportion	2017	49.6	71.5	27.9
Honduras	Utila	Count	2000	1	1	0
Honduras	Utila	Count	2017	0	1	0
Honduras	Chinacla	Proportion	2000	60.5	77.4	43.8
Honduras	Chinacla	Proportion	2017	65.0	81.0	46.2
Honduras	Chinacla	Count	2000	1	1	0
Honduras	Chinacla	Count	2017	1	1	0
Honduras	Erandique	Proportion	2000	57.0	77.0	39.7
Honduras	Erandique	Proportion	2017	64.1	80.3	45.4
Honduras	Erandique	Count	2000	1	1	0
Honduras	Erandique	Count	2017	1	1	0
Honduras	Fraternidad	Proportion	2000	55.0	74.0	35.6
Honduras	Fraternidad	Proportion	2017	61.3	77.7	41.0
Honduras	Fraternidad	Count	2000	1	1	0
Honduras	Fraternidad	Count	2017	1	1	0
Honduras	Dulce Nombre de Culmí	Proportion	2000	55.6	71.7	35.4
Honduras	Dulce Nombre de Culmí	Proportion	2017	53.3	70.5	35.3
Honduras	Dulce Nombre de Culmí	Count	2000	1	1	0
Honduras	Dulce Nombre de Culmí	Count	2017	1	1	0
Honduras	Ceguaca	Proportion	2000	55.1	75.6	36.8
Honduras	Ceguaca	Proportion	2017	58.6	78.0	38.9
Honduras	Ceguaca	Count	2000	1	1	0
Honduras	Ceguaca	Count	2017	1	1	0
Honduras	Caridad	Proportion	2000	57.3	74.7	39.2
Honduras	Caridad	Proportion	2017	59.9	77.2	39.1
Honduras	Caridad	Count	2000	1	1	0
Honduras	Caridad	Count	2017	1	1	0
Honduras	Jocón	Proportion	2000	55.6	73.3	33.5
Honduras	Jocón	Proportion	2017	54.9	74.7	35.2
Honduras	Jocón	Count	2000	1	1	0
Honduras	Jocón	Count	2017	1	1	0
Honduras	La Ceiba	Proportion	2000	52.4	72.7	29.5
Honduras	La Ceiba	Proportion	2017	50.6	70.6	30.4
Honduras	La Ceiba	Count	2000	1	1	0
Honduras	La Ceiba	Count	2017	1	1	0
Honduras	El Corpus	Proportion	2000	59.7	75.9	41.0
Honduras	El Corpus	Proportion	2017	60.7	77.0	42.6
Honduras	El Corpus	Count	2000	1	1	0
Honduras	El Corpus	Count	2017	1	1	0
Honduras	Sabá	Proportion	2000	55.8	73.7	32.1
Honduras	Sabá	Proportion	2017	52.7	73.4	32.9
Honduras	Sabá	Count	2000	1	1	0
Honduras	Sabá	Count	2017	1	1	0
Honduras	Humuya	Proportion	2000	60.9	79.1	40.9

Table 4: ORS by Unit *(continued)*

Country	Unit	Metric	Year	Mean	Upper	Lower
Honduras	Humuya	Proportion	2017	62.4	79.7	43.7
Honduras	Humuya	Count	2000	1	1	0
Honduras	Humuya	Count	2017	1	1	0
Honduras	Cucuyagua	Proportion	2000	54.3	75.0	34.4
Honduras	Cucuyagua	Proportion	2017	58.8	75.4	37.9
Honduras	Cucuyagua	Count	2000	1	1	0
Honduras	Cucuyagua	Count	2017	1	1	0
Honduras	Potrerillos	Proportion	2000	53.0	74.3	33.4
Honduras	Potrerillos	Proportion	2017	55.0	74.8	33.7
Honduras	Potrerillos	Count	2000	1	1	0
Honduras	Potrerillos	Count	2017	1	1	0
Honduras	Jacaleapa	Proportion	2000	62.2	77.4	43.4
Honduras	Jacaleapa	Proportion	2017	58.2	74.9	38.6
Honduras	Jacaleapa	Count	2000	1	1	0
Honduras	Jacaleapa	Count	2017	1	1	0
Honduras	El Porvenir	Proportion	2000	57.6	76.3	36.7
Honduras	El Porvenir	Proportion	2017	56.8	75.9	38.9
Honduras	El Porvenir	Count	2000	1	1	0
Honduras	El Porvenir	Count	2017	1	1	0
Honduras	Ramón Villeda Morales	Proportion	2000	49.9	67.0	32.7
Honduras	Ramón Villeda Morales	Proportion	2017	49.4	69.0	27.6
Honduras	Ramón Villeda Morales	Count	2000	0	1	0
Honduras	Ramón Villeda Morales	Count	2017	0	1	0
Honduras	Intibucá	Proportion	2000	58.2	76.9	39.4
Honduras	Intibucá	Proportion	2017	63.4	79.7	43.5
Honduras	Intibucá	Count	2000	1	1	0
Honduras	Intibucá	Count	2017	1	1	0
Honduras	Guajiquiro	Proportion	2000	60.0	76.6	41.9
Honduras	Guajiquiro	Proportion	2017	63.3	78.5	44.7
Honduras	Guajiquiro	Count	2000	1	1	0
Honduras	Guajiquiro	Count	2017	1	1	0
Honduras	Gracias	Proportion	2000	55.1	73.8	36.6
Honduras	Gracias	Proportion	2017	60.1	76.7	40.2
Honduras	Gracias	Count	2000	1	1	0
Honduras	Gracias	Count	2017	1	1	0
Honduras	La Encarnación	Proportion	2000	54.2	74.4	32.6
Honduras	La Encarnación	Proportion	2017	59.8	78.1	39.7
Honduras	La Encarnación	Count	2000	1	1	0
Honduras	La Encarnación	Count	2017	1	1	0
Honduras	El Rosario	Proportion	2000	57.0	74.6	34.8
Honduras	El Rosario	Proportion	2017	54.3	74.9	36.1
Honduras	El Rosario	Count	2000	1	1	0
Honduras	El Rosario	Count	2017	1	1	0
Honduras	Chinda	Proportion	2000	53.2	73.4	37.0
Honduras	Chinda	Proportion	2017	56.9	77.3	37.1
Honduras	Chinda	Count	2000	1	1	0
Honduras	Chinda	Count	2017	1	1	0
Honduras	Goascorán	Proportion	2000	57.6	73.5	39.3
Honduras	Goascorán	Proportion	2017	60.0	76.5	41.6

Table 4: ORS by Unit *(continued)*

Country	Unit	Metric	Year	Mean	Upper	Lower
Honduras	Goascorán	Count	2000	1	1	0
Honduras	Goascorán	Count	2017	1	1	0
Honduras	Morazán	Proportion	2000	53.6	72.5	33.1
Honduras	Morazán	Proportion	2017	54.0	72.8	36.0
Honduras	Morazán	Count	2000	1	1	0
Honduras	Morazán	Count	2017	1	1	0
Honduras	La Masica	Proportion	2000	52.2	70.9	33.1
Honduras	La Masica	Proportion	2017	50.6	69.8	29.3
Honduras	La Masica	Count	2000	1	1	0
Honduras	La Masica	Count	2017	1	1	0
Honduras	El Triunfo	Proportion	2000	56.9	73.0	38.7
Honduras	El Triunfo	Proportion	2017	59.0	75.6	40.7
Honduras	El Triunfo	Count	2000	1	1	0
Honduras	El Triunfo	Count	2017	1	1	0
Honduras	Santa Fé	Proportion	2000	55.8	74.1	33.9
Honduras	Santa Fé	Proportion	2017	53.3	72.9	33.3
Honduras	Santa Fé	Count	2000	1	1	0
Honduras	Santa Fé	Count	2017	1	1	0
Honduras	La Libertad	Proportion	2000	56.5	74.6	37.0
Honduras	La Libertad	Proportion	2017	57.6	77.3	40.5
Honduras	La Libertad	Count	2000	1	1	0
Honduras	La Libertad	Count	2017	1	1	0
Honduras	Dolores	Proportion	2000	53.2	73.1	32.9
Honduras	Dolores	Proportion	2017	57.3	74.7	35.8
Honduras	Dolores	Count	2000	1	1	0
Honduras	Dolores	Count	2017	1	1	0
Honduras	Puerto Cortés	Proportion	2000	52.1	74.2	32.3
Honduras	Puerto Cortés	Proportion	2017	51.7	69.4	30.7
Honduras	Puerto Cortés	Count	2000	1	1	0
Honduras	Puerto Cortés	Count	2017	1	1	0
Honduras	Liure	Proportion	2000	61.2	77.0	42.3
Honduras	Liure	Proportion	2017	61.0	77.4	42.9
Honduras	Liure	Count	2000	1	1	0
Honduras	Liure	Count	2017	1	1	0
Honduras	Guaimaca	Proportion	2000	59.0	77.4	39.0
Honduras	Guaimaca	Proportion	2017	55.7	74.9	36.9
Honduras	Guaimaca	Count	2000	1	1	0
Honduras	Guaimaca	Count	2017	1	1	0
Honduras	Wampusirpi	Proportion	2000	50.8	67.3	31.3
Honduras	Wampusirpi	Proportion	2017	49.3	67.5	31.5
Honduras	Wampusirpi	Count	2000	1	1	0
Honduras	Wampusirpi	Count	2017	0	1	0
Honduras	Jesús de Otoro	Proportion	2000	58.0	76.1	40.8
Honduras	Jesús de Otoro	Proportion	2017	61.9	78.5	43.3
Honduras	Jesús de Otoro	Count	2000	1	1	0
Honduras	Jesús de Otoro	Count	2017	1	1	0
Honduras	La Paz	Proportion	2000	60.0	77.7	41.2
Honduras	La Paz	Proportion	2017	61.4	79.2	42.0
Honduras	La Paz	Count	2000	1	1	0

Table 4: ORS by Unit *(continued)*

Country	Unit	Metric	Year	Mean	Upper	Lower
Honduras	La Paz	Count	2017	1	1	0
Honduras	Gualcince	Proportion	2000	57.9	75.4	38.9
Honduras	Gualcince	Proportion	2017	65.2	80.3	47.2
Honduras	Gualcince	Count	2000	1	1	0
Honduras	Gualcince	Count	2017	1	1	0
Honduras	La Labor	Proportion	2000	55.1	74.0	36.2
Honduras	La Labor	Proportion	2017	61.1	77.7	41.3
Honduras	La Labor	Count	2000	1	1	0
Honduras	La Labor	Count	2017	1	1	0
Honduras	Esquipulas del Norte	Proportion	2000	55.9	72.2	35.4
Honduras	Esquipulas del Norte	Proportion	2017	54.4	73.9	34.9
Honduras	Esquipulas del Norte	Count	2000	1	1	0
Honduras	Esquipulas del Norte	Count	2017	1	1	0
Honduras	Concepción del Norte	Proportion	2000	53.4	71.5	37.3
Honduras	Concepción del Norte	Proportion	2017	56.4	74.3	37.2
Honduras	Concepción del Norte	Count	2000	1	1	0
Honduras	Concepción del Norte	Count	2017	1	1	0
Honduras	Langue	Proportion	2000	58.3	76.0	39.1
Honduras	Langue	Proportion	2017	60.2	78.1	41.2
Honduras	Langue	Count	2000	1	1	0
Honduras	Langue	Count	2017	1	1	0
Honduras	Olanchito	Proportion	2000	55.4	72.8	35.8
Honduras	Olanchito	Proportion	2017	53.2	73.2	34.9
Honduras	Olanchito	Count	2000	1	1	0
Honduras	Olanchito	Count	2017	1	1	0
Honduras	San Francisco	Proportion	2000	52.3	71.9	32.6
Honduras	San Francisco	Proportion	2017	51.1	70.8	30.5
Honduras	San Francisco	Count	2000	1	1	0
Honduras	San Francisco	Count	2017	1	1	0
Honduras	Marcovia	Proportion	2000	57.4	74.2	38.7
Honduras	Marcovia	Proportion	2017	60.4	76.9	42.2
Honduras	Marcovia	Count	2000	1	1	0
Honduras	Marcovia	Count	2017	1	1	0
Honduras	Santa Rosa de Aguán	Proportion	2000	56.4	73.8	33.9
Honduras	Santa Rosa de Aguán	Proportion	2017	54.8	73.7	32.9
Honduras	Santa Rosa de Aguán	Count	2000	1	1	0
Honduras	Santa Rosa de Aguán	Count	2017	1	1	0
Honduras	La Trinidad	Proportion	2000	56.3	75.3	37.4
Honduras	La Trinidad	Proportion	2017	58.1	76.6	40.4
Honduras	La Trinidad	Count	2000	1	1	0
Honduras	La Trinidad	Count	2017	1	1	0
Honduras	Dulce Nombre	Proportion	2000	53.3	73.5	33.3
Honduras	Dulce Nombre	Proportion	2017	57.8	74.6	38.6
Honduras	Dulce Nombre	Count	2000	1	1	0
Honduras	Dulce Nombre	Count	2017	1	1	0
Honduras	San Antonio de Cortés	Proportion	2000	53.0	72.2	36.3
Honduras	San Antonio de Cortés	Proportion	2017	55.5	75.0	36.1
Honduras	San Antonio de Cortés	Count	2000	1	1	0
Honduras	San Antonio de Cortés	Count	2017	1	1	0

Table 4: ORS by Unit *(continued)*

Country	Unit	Metric	Year	Mean	Upper	Lower
Honduras	Morocelí	Proportion	2000	60.9	76.4	41.5
Honduras	Morocelí	Proportion	2017	57.6	75.8	38.7
Honduras	Morocelí	Count	2000	1	1	0
Honduras	Morocelí	Count	2017	1	1	0
Honduras	La Libertad	Proportion	2000	58.3	73.5	39.5
Honduras	La Libertad	Proportion	2017	60.8	79.1	42.0
Honduras	La Libertad	Count	2000	1	1	0
Honduras	La Libertad	Count	2017	1	1	0
Honduras	La Esperanza	Proportion	2000	59.3	78.5	39.9
Honduras	La Esperanza	Proportion	2017	64.5	81.4	44.9
Honduras	La Esperanza	Count	2000	1	1	0
Honduras	La Esperanza	Count	2017	1	1	0
Honduras	Lauterique	Proportion	2000	59.1	76.8	40.2
Honduras	Lauterique	Proportion	2017	61.8	79.8	40.5
Honduras	Lauterique	Count	2000	1	1	0
Honduras	Lauterique	Count	2017	1	1	0
Honduras	Guarita	Proportion	2000	56.1	73.6	37.5
Honduras	Guarita	Proportion	2017	63.3	79.3	47.7
Honduras	Guarita	Count	2000	1	1	0
Honduras	Guarita	Count	2017	1	1	0
Honduras	Lucerna	Proportion	2000	55.2	74.3	34.9
Honduras	Lucerna	Proportion	2017	60.4	76.4	40.7
Honduras	Lucerna	Count	2000	1	1	0
Honduras	Lucerna	Count	2017	1	1	0
Honduras	Gualaco	Proportion	2000	57.1	73.0	37.1
Honduras	Gualaco	Proportion	2017	53.6	72.2	33.4
Honduras	Gualaco	Count	2000	1	1	0
Honduras	Gualaco	Count	2017	1	1	0
Honduras	Concepción del Sur	Proportion	2000	55.1	76.2	38.3
Honduras	Concepción del Sur	Proportion	2017	59.1	77.9	40.1
Honduras	Concepción del Sur	Count	2000	1	1	0
Honduras	Concepción del Sur	Count	2017	1	1	0
Honduras	Nacaome	Proportion	2000	58.5	74.8	40.0
Honduras	Nacaome	Proportion	2017	60.6	77.4	42.7
Honduras	Nacaome	Count	2000	1	1	0
Honduras	Nacaome	Count	2017	1	1	0
Honduras	Santa Rita	Proportion	2000	53.9	74.2	35.1
Honduras	Santa Rita	Proportion	2017	54.9	74.9	32.9
Honduras	Santa Rita	Count	2000	1	1	0
Honduras	Santa Rita	Count	2017	1	1	0
Honduras	Tela	Proportion	2000	52.1	70.1	33.6
Honduras	Tela	Proportion	2017	51.1	70.1	32.1
Honduras	Tela	Count	2000	1	1	0
Honduras	Tela	Count	2017	1	1	0
Honduras	Morolica	Proportion	2000	62.0	77.6	45.1
Honduras	Morolica	Proportion	2017	60.9	77.1	42.4
Honduras	Morolica	Count	2000	1	1	0
Honduras	Morolica	Count	2017	1	1	0
Honduras	Sonaguera	Proportion	2000	55.6	73.4	32.9

Table 4: ORS by Unit *(continued)*

Country	Unit	Metric	Year	Mean	Upper	Lower
Honduras	Sonaguera	Proportion	2017	53.0	74.1	33.8
Honduras	Sonaguera	Count	2000	1	1	0
Honduras	Sonaguera	Count	2017	1	1	0
Honduras	Lamaní	Proportion	2000	60.7	77.4	41.0
Honduras	Lamaní	Proportion	2017	62.1	77.9	42.4
Honduras	Lamaní	Count	2000	1	1	0
Honduras	Lamaní	Count	2017	1	1	0
Honduras	El Paraíso	Proportion	2000	52.7	72.6	33.7
Honduras	El Paraíso	Proportion	2017	56.0	73.2	35.4
Honduras	El Paraíso	Count	2000	1	1	0
Honduras	El Paraíso	Count	2017	1	1	0
Honduras	San Francisco de Yojoa	Proportion	2000	53.1	74.2	35.6
Honduras	San Francisco de Yojoa	Proportion	2017	55.3	75.8	35.2
Honduras	San Francisco de Yojoa	Count	2000	1	1	0
Honduras	San Francisco de Yojoa	Count	2017	1	1	0
Honduras	Oropolí	Proportion	2000	62.2	79.2	43.4
Honduras	Oropolí	Proportion	2017	59.8	76.0	41.7
Honduras	Oropolí	Count	2000	1	1	0
Honduras	Oropolí	Count	2017	1	1	0
Honduras	La Venta	Proportion	2000	58.6	74.3	38.0
Honduras	La Venta	Proportion	2017	59.7	76.4	42.3
Honduras	La Venta	Count	2000	1	1	0
Honduras	La Venta	Count	2017	1	1	0
Honduras	Magdalena	Proportion	2000	58.8	77.0	38.6
Honduras	Magdalena	Proportion	2017	65.9	81.5	47.3
Honduras	Magdalena	Count	2000	1	1	0
Honduras	Magdalena	Count	2017	1	1	0
Honduras	Marcala	Proportion	2000	60.2	76.2	42.4
Honduras	Marcala	Proportion	2017	64.8	79.7	44.1
Honduras	Marcala	Count	2000	1	1	0
Honduras	Marcala	Count	2017	1	1	0
Honduras	La Campa	Proportion	2000	55.4	75.0	36.3
Honduras	La Campa	Proportion	2017	61.8	78.1	44.9
Honduras	La Campa	Count	2000	1	1	0
Honduras	La Campa	Count	2017	1	1	0
Honduras	Mercedes	Proportion	2000	55.9	74.7	35.6
Honduras	Mercedes	Proportion	2017	62.7	77.7	46.5
Honduras	Mercedes	Count	2000	1	1	0
Honduras	Mercedes	Count	2017	1	1	0
Honduras	Guarizama	Proportion	2000	57.0	73.8	36.8
Honduras	Guarizama	Proportion	2017	53.0	71.0	33.7
Honduras	Guarizama	Count	2000	1	1	0
Honduras	Guarizama	Count	2017	1	1	0
Honduras	El Nispero	Proportion	2000	54.8	75.1	35.3
Honduras	El Nispero	Proportion	2017	59.5	77.6	41.2
Honduras	El Nispero	Count	2000	1	1	0
Honduras	El Nispero	Count	2017	1	1	0
Honduras	San Francisco de Coray	Proportion	2000	58.5	75.5	41.1
Honduras	San Francisco de Coray	Proportion	2017	60.5	78.2	40.7

Table 4: ORS by Unit *(continued)*

Country	Unit	Metric	Year	Mean	Upper	Lower
Honduras	San Francisco de Coray	Count	2000	1	1	0
Honduras	San Francisco de Coray	Count	2017	1	1	0
Honduras	Sulaco	Proportion	2000	55.9	75.0	36.1
Honduras	Sulaco	Proportion	2017	55.3	74.2	35.8
Honduras	Sulaco	Count	2000	1	1	0
Honduras	Sulaco	Count	2017	1	1	0
Honduras	Namasigue	Proportion	2000	56.5	72.7	38.9
Honduras	Namasigue	Proportion	2017	59.0	77.2	40.5
Honduras	Namasigue	Count	2000	1	1	0
Honduras	Namasigue	Count	2017	1	1	0
Honduras	Tocoa	Proportion	2000	56.1	74.4	34.4
Honduras	Tocoa	Proportion	2017	53.8	72.9	33.0
Honduras	Tocoa	Count	2000	1	1	0
Honduras	Tocoa	Count	2017	1	1	0
Honduras	Las Lajas	Proportion	2000	56.0	74.4	38.1
Honduras	Las Lajas	Proportion	2017	57.6	76.4	39.1
Honduras	Las Lajas	Count	2000	1	1	0
Honduras	Las Lajas	Count	2017	1	1	0
Honduras	Florida	Proportion	2000	52.4	71.1	35.7
Honduras	Florida	Proportion	2017	55.7	73.0	35.6
Honduras	Florida	Count	2000	1	1	0
Honduras	Florida	Count	2017	1	1	0
Honduras	San Manuel	Proportion	2000	52.8	73.2	34.5
Honduras	San Manuel	Proportion	2017	54.0	74.5	33.8
Honduras	San Manuel	Count	2000	1	1	0
Honduras	San Manuel	Count	2017	1	1	0
Honduras	Potrerillos	Proportion	2000	62.0	78.4	41.8
Honduras	Potrerillos	Proportion	2017	58.0	74.9	37.7
Honduras	Potrerillos	Count	2000	1	1	0
Honduras	Potrerillos	Count	2017	1	1	0
Honduras	Lepaterique	Proportion	2000	59.7	74.5	42.0
Honduras	Lepaterique	Proportion	2017	60.9	75.6	40.4
Honduras	Lepaterique	Count	2000	1	1	0
Honduras	Lepaterique	Count	2017	1	1	0
Honduras	Masaguara	Proportion	2000	58.9	76.0	40.7
Honduras	Masaguara	Proportion	2017	63.6	79.1	45.0
Honduras	Masaguara	Count	2000	1	1	0
Honduras	Masaguara	Count	2017	1	1	0
Honduras	Mercedes de Oriente	Proportion	2000	58.5	74.6	39.4
Honduras	Mercedes de Oriente	Proportion	2017	61.8	77.9	44.3
Honduras	Mercedes de Oriente	Count	2000	1	1	0
Honduras	Mercedes de Oriente	Count	2017	1	1	0
Honduras	La Iguala	Proportion	2000	54.5	73.5	36.9
Honduras	La Iguala	Proportion	2017	60.1	76.6	41.4
Honduras	La Iguala	Count	2000	1	1	0
Honduras	La Iguala	Count	2017	1	1	0
Honduras	Ocotepeque	Proportion	2000	55.7	74.9	35.5
Honduras	Ocotepeque	Proportion	2017	62.0	80.5	42.9
Honduras	Ocotepeque	Count	2000	1	1	0

Table 4: ORS by Unit *(continued)*

Country	Unit	Metric	Year	Mean	Upper	Lower
Honduras	Ocotepeque	Count	2017	1	1	0
Honduras	Guata	Proportion	2000	55.9	72.5	35.3
Honduras	Guata	Proportion	2017	53.6	72.3	34.1
Honduras	Guata	Count	2000	1	1	0
Honduras	Guata	Count	2017	1	1	0
Honduras	Gualala	Proportion	2000	53.8	73.0	36.8
Honduras	Gualala	Proportion	2017	57.7	77.1	37.9
Honduras	Gualala	Count	2000	1	1	0
Honduras	Gualala	Count	2017	1	1	0
Honduras	San Lorenzo	Proportion	2000	59.1	74.6	38.5
Honduras	San Lorenzo	Proportion	2017	61.3	79.6	42.8
Honduras	San Lorenzo	Count	2000	1	1	0
Honduras	San Lorenzo	Count	2017	1	1	0
Honduras	Victoria	Proportion	2000	54.6	73.2	34.4
Honduras	Victoria	Proportion	2017	55.6	74.6	37.8
Honduras	Victoria	Count	2000	1	1	0
Honduras	Victoria	Count	2017	1	1	0
Honduras	Orocuina	Proportion	2000	60.0	74.8	40.5
Honduras	Orocuina	Proportion	2017	60.4	76.6	41.9
Honduras	Orocuina	Count	2000	1	1	0
Honduras	Orocuina	Count	2017	1	1	0
Honduras	Trujillo	Proportion	2000	55.9	74.2	33.8
Honduras	Trujillo	Proportion	2017	53.8	72.7	34.3
Honduras	Trujillo	Count	2000	1	1	0
Honduras	Trujillo	Count	2017	1	1	0
Honduras	Lejamaní	Proportion	2000	59.1	77.7	39.2
Honduras	Lejamaní	Proportion	2017	60.4	77.6	39.6
Honduras	Lejamaní	Count	2000	1	1	0
Honduras	Lejamaní	Count	2017	1	1	0
Honduras	La Jigua	Proportion	2000	52.2	72.1	36.0
Honduras	La Jigua	Proportion	2017	55.0	73.9	36.5
Honduras	La Jigua	Count	2000	1	1	0
Honduras	La Jigua	Count	2017	1	1	0
Honduras	San Pedro Sula	Proportion	2000	53.2	70.2	35.0
Honduras	San Pedro Sula	Proportion	2017	54.0	72.0	32.6
Honduras	San Pedro Sula	Count	2000	1	1	0
Honduras	San Pedro Sula	Count	2017	1	1	0
Honduras	San Antonio de Flores	Proportion	2000	62.2	78.5	43.0
Honduras	San Antonio de Flores	Proportion	2017	60.3	75.9	41.8
Honduras	San Antonio de Flores	Count	2000	1	1	0
Honduras	San Antonio de Flores	Count	2017	1	1	0
Honduras	Maraita	Proportion	2000	60.6	76.6	40.6
Honduras	Maraita	Proportion	2017	59.2	77.2	39.8
Honduras	Maraita	Count	2000	1	1	0
Honduras	Maraita	Count	2017	1	1	0
Honduras	San Antonio	Proportion	2000	58.5	75.0	39.9
Honduras	San Antonio	Proportion	2017	65.5	81.4	48.9
Honduras	San Antonio	Count	2000	1	1	0
Honduras	San Antonio	Count	2017	1	1	0

Table 4: ORS by Unit *(continued)*

Country	Unit	Metric	Year	Mean	Upper	Lower
Honduras	Opatoro	Proportion	2000	60.1	75.7	41.1
Honduras	Opatoro	Proportion	2017	64.4	79.3	45.7
Honduras	Opatoro	Count	2000	1	1	0
Honduras	Opatoro	Count	2017	1	1	0
Honduras	La Unión	Proportion	2000	54.5	75.3	34.1
Honduras	La Unión	Proportion	2017	59.4	78.8	38.2
Honduras	La Unión	Count	2000	1	1	0
Honduras	La Unión	Count	2017	1	1	0
Honduras	San Fernando	Proportion	2000	54.0	73.1	35.6
Honduras	San Fernando	Proportion	2017	59.5	75.9	41.2
Honduras	San Fernando	Count	2000	1	1	0
Honduras	San Fernando	Count	2017	1	1	0
Honduras	Guayape	Proportion	2000	57.7	75.6	38.1
Honduras	Guayape	Proportion	2017	54.9	75.5	35.6
Honduras	Guayape	Count	2000	1	1	0
Honduras	Guayape	Count	2017	1	1	0
Honduras	Ilama	Proportion	2000	53.8	72.5	37.1
Honduras	Ilama	Proportion	2017	57.0	75.5	38.4
Honduras	Ilama	Count	2000	1	1	0
Honduras	Ilama	Count	2017	1	1	0
Honduras	Yorito	Proportion	2000	55.2	74.5	35.1
Honduras	Yorito	Proportion	2017	55.1	75.1	38.2
Honduras	Yorito	Count	2000	1	1	0
Honduras	Yorito	Count	2017	1	1	0
Honduras	Pespire	Proportion	2000	58.4	73.7	40.3
Honduras	Pespire	Proportion	2017	59.7	76.0	41.9
Honduras	Pespire	Count	2000	1	1	0
Honduras	Pespire	Count	2017	1	1	0
Honduras	Meámbar	Proportion	2000	55.3	74.6	36.5
Honduras	Meámbar	Proportion	2017	57.7	76.0	40.1
Honduras	Meámbar	Count	2000	1	1	0
Honduras	Meámbar	Count	2017	1	1	0
Honduras	La Unión	Proportion	2000	54.3	73.9	35.3
Honduras	La Unión	Proportion	2017	58.7	75.9	39.7
Honduras	La Unión	Count	2000	1	1	0
Honduras	La Unión	Count	2017	1	1	0
Honduras	Santa Cruz de Yojoa	Proportion	2000	53.2	73.1	34.1
Honduras	Santa Cruz de Yojoa	Proportion	2017	55.7	74.3	37.4
Honduras	Santa Cruz de Yojoa	Count	2000	1	1	0
Honduras	Santa Cruz de Yojoa	Count	2017	1	1	0
Honduras	San Lucas	Proportion	2000	61.5	77.5	41.5
Honduras	San Lucas	Proportion	2017	60.2	76.6	38.9
Honduras	San Lucas	Count	2000	1	1	0
Honduras	San Lucas	Count	2017	1	1	0
Honduras	Marale	Proportion	2000	56.3	74.6	34.4
Honduras	Marale	Proportion	2017	55.4	74.4	35.8
Honduras	Marale	Count	2000	1	1	0
Honduras	Marale	Count	2017	1	1	0
Honduras	San Francisco de Opalaca	Proportion	2000	56.5	74.2	38.1

Table 4: ORS by Unit *(continued)*

Country	Unit	Metric	Year	Mean	Upper	Lower
Honduras	San Francisco de Opalaca	Proportion	2017	62.2	78.8	44.6
Honduras	San Francisco de Opalaca	Count	2000	1	1	0
Honduras	San Francisco de Opalaca	Count	2017	1	1	0
Honduras	San Antonio del Norte	Proportion	2000	59.1	76.6	38.9
Honduras	San Antonio del Norte	Proportion	2017	61.9	78.0	43.7
Honduras	San Antonio del Norte	Count	2000	1	1	0
Honduras	San Antonio del Norte	Count	2017	1	1	0
Honduras	La Virtud	Proportion	2000	58.3	75.5	38.0
Honduras	La Virtud	Proportion	2017	65.9	81.0	48.7
Honduras	La Virtud	Count	2000	1	1	0
Honduras	La Virtud	Count	2017	1	1	0
Honduras	San Francisco del Valle	Proportion	2000	55.6	75.0	37.2
Honduras	San Francisco del Valle	Proportion	2017	62.0	78.0	45.3
Honduras	San Francisco del Valle	Count	2000	1	1	0
Honduras	San Francisco del Valle	Count	2017	1	1	0
Honduras	Jano	Proportion	2000	56.2	72.6	36.3
Honduras	Jano	Proportion	2017	54.2	73.3	34.9
Honduras	Jano	Count	2000	1	1	0
Honduras	Jano	Count	2017	1	1	0
Honduras	Las Vegas	Proportion	2000	55.3	75.7	36.3
Honduras	Las Vegas	Proportion	2017	58.7	77.9	39.1
Honduras	Las Vegas	Count	2000	1	1	0
Honduras	Las Vegas	Count	2017	1	1	0
Honduras	Yoro	Proportion	2000	55.3	73.0	34.6
Honduras	Yoro	Proportion	2017	54.7	74.2	35.4
Honduras	Yoro	Count	2000	1	1	0
Honduras	Yoro	Count	2017	1	1	0
Honduras	San Antonio de Flores	Proportion	2000	57.9	73.4	39.8
Honduras	San Antonio de Flores	Proportion	2017	59.1	77.7	38.7
Honduras	San Antonio de Flores	Count	2000	1	1	0
Honduras	San Antonio de Flores	Count	2017	1	1	0
Honduras	Minas de Oro	Proportion	2000	56.5	75.2	36.9
Honduras	Minas de Oro	Proportion	2017	57.0	76.7	39.0
Honduras	Minas de Oro	Count	2000	1	1	0
Honduras	Minas de Oro	Count	2017	1	1	0
Honduras	Nueva Arcadia	Proportion	2000	53.0	73.2	34.2
Honduras	Nueva Arcadia	Proportion	2017	55.4	73.8	34.6
Honduras	Nueva Arcadia	Count	2000	1	1	0
Honduras	Nueva Arcadia	Count	2017	1	1	0
Honduras	Villanueva	Proportion	2000	52.8	71.6	35.2
Honduras	Villanueva	Proportion	2017	54.4	74.7	34.7
Honduras	Villanueva	Count	2000	1	1	0
Honduras	Villanueva	Count	2017	1	1	0
Honduras	San Matías	Proportion	2000	62.4	78.0	43.7
Honduras	San Matías	Proportion	2017	58.4	76.6	38.0
Honduras	San Matías	Count	2000	1	1	0
Honduras	San Matías	Count	2017	1	1	0
Honduras	Nueva Armenia	Proportion	2000	60.9	75.8	40.8
Honduras	Nueva Armenia	Proportion	2017	60.2	77.2	41.8

Table 4: ORS by Unit *(continued)*

Country	Unit	Metric	Year	Mean	Upper	Lower
Honduras	Nueva Armenia	Count	2000	1	1	0
Honduras	Nueva Armenia	Count	2017	1	1	0
Honduras	San Isidro	Proportion	2000	57.8	76.1	40.8
Honduras	San Isidro	Proportion	2017	62.1	79.6	41.6
Honduras	San Isidro	Count	2000	1	1	0
Honduras	San Isidro	Count	2017	1	1	0
Honduras	San José	Proportion	2000	60.5	76.2	40.6
Honduras	San José	Proportion	2017	65.0	81.7	45.7
Honduras	San José	Count	2000	1	1	0
Honduras	San José	Count	2017	1	1	0
Honduras	Las Flores	Proportion	2000	54.4	74.4	35.9
Honduras	Las Flores	Proportion	2017	59.2	78.5	39.8
Honduras	Las Flores	Count	2000	1	1	0
Honduras	Las Flores	Count	2017	1	1	0
Honduras	San Jorge	Proportion	2000	54.9	75.2	35.6
Honduras	San Jorge	Proportion	2017	61.1	78.4	42.4
Honduras	San Jorge	Count	2000	1	1	0
Honduras	San Jorge	Count	2017	1	1	0
Honduras	Juticalpa	Proportion	2000	58.4	75.1	39.4
Honduras	Juticalpa	Proportion	2017	53.1	71.9	33.5
Honduras	Juticalpa	Count	2000	1	1	0
Honduras	Juticalpa	Count	2017	1	1	0
Honduras	Macuelizo	Proportion	2000	53.1	73.4	36.1
Honduras	Macuelizo	Proportion	2017	56.0	74.6	37.4
Honduras	Macuelizo	Count	2000	1	1	0
Honduras	Macuelizo	Count	2017	1	1	0
Honduras	San Isidro	Proportion	2000	59.3	77.0	40.2
Honduras	San Isidro	Proportion	2017	59.9	78.3	40.4
Honduras	San Isidro	Count	2000	1	1	0
Honduras	San Isidro	Count	2017	1	1	0
Honduras	Ojo de Agua	Proportion	2000	56.5	75.0	36.9
Honduras	Ojo de Agua	Proportion	2017	57.3	76.5	39.8
Honduras	Ojo de Agua	Count	2000	1	1	0
Honduras	Ojo de Agua	Count	2017	1	1	0
Honduras	San Agustín	Proportion	2000	53.8	73.2	34.5
Honduras	San Agustín	Proportion	2017	57.9	75.3	36.4
Honduras	San Agustín	Count	2000	1	1	0
Honduras	San Agustín	Count	2017	1	1	0
Honduras	Soledad	Proportion	2000	60.8	77.4	39.5
Honduras	Soledad	Proportion	2017	61.3	77.4	42.0
Honduras	Soledad	Count	2000	1	1	0
Honduras	Soledad	Count	2017	1	1	0
Honduras	Ojojona	Proportion	2000	60.0	75.6	41.9
Honduras	Ojojona	Proportion	2017	59.6	75.7	40.0
Honduras	Ojojona	Count	2000	1	1	0
Honduras	Ojojona	Count	2017	1	1	0
Honduras	San Juan	Proportion	2000	56.7	76.2	37.2
Honduras	San Juan	Proportion	2017	63.0	79.5	45.2
Honduras	San Juan	Count	2000	1	1	0

Table 4: ORS by Unit *(continued)*

Country	Unit	Metric	Year	Mean	Upper	Lower
Honduras	San Juan	Count	2017	1	1	0
Honduras	San Juan	Proportion	2000	60.0	77.1	39.3
Honduras	San Juan	Proportion	2017	62.7	78.3	44.1
Honduras	San Juan	Count	2000	1	1	0
Honduras	San Juan	Count	2017	1	1	0
Honduras	Lepaera	Proportion	2000	54.2	74.1	35.2
Honduras	Lepaera	Proportion	2017	58.7	75.4	39.5
Honduras	Lepaera	Count	2000	1	1	0
Honduras	Lepaera	Count	2017	1	1	0
Honduras	San Marcos	Proportion	2000	55.7	75.1	37.9
Honduras	San Marcos	Proportion	2017	62.2	79.4	44.4
Honduras	San Marcos	Count	2000	1	1	0
Honduras	San Marcos	Count	2017	1	1	0
Honduras	La Unión	Proportion	2000	56.4	73.4	35.9
Honduras	La Unión	Proportion	2017	54.5	73.9	37.5
Honduras	La Unión	Count	2000	1	1	0
Honduras	La Unión	Count	2017	1	1	0
Honduras	Naranjito	Proportion	2000	53.2	72.4	35.4
Honduras	Naranjito	Proportion	2017	57.3	75.3	36.6
Honduras	Naranjito	Count	2000	1	1	0
Honduras	Naranjito	Count	2017	1	1	0
Honduras	San José	Proportion	2000	59.0	74.7	39.4
Honduras	San José	Proportion	2017	60.6	76.4	42.3
Honduras	San José	Count	2000	1	1	0
Honduras	San José	Count	2017	1	1	0
Honduras	San Jerónimo	Proportion	2000	57.1	75.6	39.0
Honduras	San Jerónimo	Proportion	2017	58.2	77.7	39.5
Honduras	San Jerónimo	Count	2000	1	1	0
Honduras	San Jerónimo	Count	2017	1	1	0
Honduras	San Antonio	Proportion	2000	51.9	70.6	32.5
Honduras	San Antonio	Proportion	2017	55.2	72.7	34.6
Honduras	San Antonio	Count	2000	1	1	0
Honduras	San Antonio	Count	2017	1	1	0
Honduras	Teupasenti	Proportion	2000	60.3	76.8	41.3
Honduras	Teupasenti	Proportion	2017	56.2	75.3	37.8
Honduras	Teupasenti	Count	2000	1	1	0
Honduras	Teupasenti	Count	2017	1	1	0
Honduras	Orica	Proportion	2000	57.5	75.7	37.5
Honduras	Orica	Proportion	2017	55.3	74.2	36.2
Honduras	Orica	Count	2000	1	1	0
Honduras	Orica	Count	2017	1	1	0
Honduras	San Marcos de la Sierra	Proportion	2000	59.1	75.0	41.1
Honduras	San Marcos de la Sierra	Proportion	2017	65.1	81.3	44.0
Honduras	San Marcos de la Sierra	Count	2000	1	1	0
Honduras	San Marcos de la Sierra	Count	2017	1	1	0
Honduras	San Pedro de Tutule	Proportion	2000	60.6	78.1	42.7
Honduras	San Pedro de Tutule	Proportion	2017	64.8	80.8	45.5
Honduras	San Pedro de Tutule	Count	2000	1	1	0
Honduras	San Pedro de Tutule	Count	2017	1	1	0

Table 4: ORS by Unit *(continued)*

Country	Unit	Metric	Year	Mean	Upper	Lower
Honduras	Mapulaca	Proportion	2000	58.8	77.5	38.0
Honduras	Mapulaca	Proportion	2017	65.2	80.7	45.9
Honduras	Mapulaca	Count	2000	1	1	0
Honduras	Mapulaca	Count	2017	1	1	0
Honduras	Santa Fé	Proportion	2000	54.2	73.1	35.6
Honduras	Santa Fé	Proportion	2017	60.7	76.6	42.2
Honduras	Santa Fé	Count	2000	1	1	0
Honduras	Santa Fé	Count	2017	1	1	0
Honduras	Mangulile	Proportion	2000	56.3	74.7	35.7
Honduras	Mangulile	Proportion	2017	54.9	75.0	37.1
Honduras	Mangulile	Count	2000	1	1	0
Honduras	Mangulile	Count	2017	1	1	0
Honduras	Nueva Frontera	Proportion	2000	52.9	72.7	36.0
Honduras	Nueva Frontera	Proportion	2017	56.0	74.7	36.7
Honduras	Nueva Frontera	Count	2000	1	1	0
Honduras	Nueva Frontera	Count	2017	1	1	0
Honduras	San Marcos de Colón	Proportion	2000	62.1	77.5	44.9
Honduras	San Marcos de Colón	Proportion	2017	61.4	77.1	43.4
Honduras	San Marcos de Colón	Count	2000	1	1	0
Honduras	San Marcos de Colón	Count	2017	1	1	0
Honduras	San José de Comayagua	Proportion	2000	56.0	76.5	37.5
Honduras	San José de Comayagua	Proportion	2017	59.8	77.9	40.9
Honduras	San José de Comayagua	Count	2000	1	1	0
Honduras	San José de Comayagua	Count	2017	1	1	0
Honduras	San Jerónimo	Proportion	2000	52.6	72.2	34.2
Honduras	San Jerónimo	Proportion	2017	56.3	73.5	36.6
Honduras	San Jerónimo	Count	2000	1	1	0
Honduras	San Jerónimo	Count	2017	1	1	0
Honduras	Texiguat	Proportion	2000	61.8	76.6	42.4
Honduras	Texiguat	Proportion	2017	60.7	76.2	42.8
Honduras	Texiguat	Count	2000	1	1	0
Honduras	Texiguat	Count	2017	1	1	0
Honduras	Reitoca	Proportion	2000	58.9	75.0	38.7
Honduras	Reitoca	Proportion	2017	60.2	75.8	41.7
Honduras	Reitoca	Count	2000	1	1	0
Honduras	Reitoca	Count	2017	1	1	0
Honduras	San Miguelito	Proportion	2000	57.6	75.4	39.9
Honduras	San Miguelito	Proportion	2017	63.8	79.1	46.7
Honduras	San Miguelito	Count	2000	1	1	0
Honduras	San Miguelito	Count	2017	1	1	0
Honduras	Santa Ana	Proportion	2000	58.9	74.8	41.0
Honduras	Santa Ana	Proportion	2017	63.9	79.7	45.6
Honduras	Santa Ana	Count	2000	1	1	0
Honduras	Santa Ana	Count	2017	1	1	0
Honduras	Piraera	Proportion	2000	57.9	76.4	39.1
Honduras	Piraera	Proportion	2017	65.2	80.8	45.8
Honduras	Piraera	Count	2000	1	1	0
Honduras	Piraera	Count	2017	1	1	0
Honduras	Sensenti	Proportion	2000	55.1	75.3	35.4

Table 4: ORS by Unit *(continued)*

Country	Unit	Metric	Year	Mean	Upper	Lower
Honduras	Sensenti	Proportion	2017	60.9	78.1	42.2
Honduras	Sensenti	Count	2000	1	1	0
Honduras	Sensenti	Count	2017	1	1	0
Honduras	Manto	Proportion	2000	57.5	74.6	38.1
Honduras	Manto	Proportion	2017	53.3	72.1	34.1
Honduras	Manto	Count	2000	1	1	0
Honduras	Manto	Count	2017	1	1	0
Honduras	Nuevo Celilac	Proportion	2000	54.3	73.5	37.7
Honduras	Nuevo Celilac	Proportion	2017	58.6	75.5	39.2
Honduras	Nuevo Celilac	Count	2000	1	1	0
Honduras	Nuevo Celilac	Count	2017	1	1	0
Honduras	Santa Ana de Yusguare	Proportion	2000	58.6	75.7	37.9
Honduras	Santa Ana de Yusguare	Proportion	2017	59.9	77.4	42.4
Honduras	Santa Ana de Yusguare	Count	2000	1	1	0
Honduras	Santa Ana de Yusguare	Count	2017	1	1	0
Honduras	San José del Potrero	Proportion	2000	56.7	74.8	36.7
Honduras	San José del Potrero	Proportion	2017	56.4	75.9	38.2
Honduras	San José del Potrero	Count	2000	1	1	0
Honduras	San José del Potrero	Count	2017	1	1	0
Honduras	San José	Proportion	2000	53.1	72.7	33.9
Honduras	San José	Proportion	2017	57.3	75.7	36.4
Honduras	San José	Count	2000	1	1	0
Honduras	San José	Count	2017	1	1	0
Honduras	Trojes	Proportion	2000	58.9	73.7	42.5
Honduras	Trojes	Proportion	2017	53.7	73.3	34.0
Honduras	Trojes	Count	2000	1	1	0
Honduras	Trojes	Count	2017	1	1	0
Honduras	Sabanagrande	Proportion	2000	59.7	75.0	41.2
Honduras	Sabanagrande	Proportion	2017	59.8	75.8	42.3
Honduras	Sabanagrande	Count	2000	1	1	0
Honduras	Sabanagrande	Count	2017	1	1	0
Honduras	Santa Lucía	Proportion	2000	58.2	75.2	37.6
Honduras	Santa Lucía	Proportion	2017	65.8	82.1	47.7
Honduras	Santa Lucía	Count	2000	1	1	0
Honduras	Santa Lucía	Count	2017	1	1	0
Honduras	Santa Elena	Proportion	2000	59.0	75.4	40.9
Honduras	Santa Elena	Proportion	2017	65.0	79.8	44.9
Honduras	Santa Elena	Count	2000	1	1	0
Honduras	Santa Elena	Count	2017	1	1	0
Honduras	San Andrés	Proportion	2000	56.6	76.0	37.4
Honduras	San Andrés	Proportion	2017	64.1	80.1	45.7
Honduras	San Andrés	Count	2000	1	1	0
Honduras	San Andrés	Count	2017	1	1	0
Honduras	Sinuapa	Proportion	2000	56.0	75.4	36.5
Honduras	Sinuapa	Proportion	2017	61.9	79.4	42.9
Honduras	Sinuapa	Count	2000	1	1	0
Honduras	Sinuapa	Count	2017	1	1	0
Honduras	Patuca	Proportion	2000	58.8	73.6	41.2
Honduras	Patuca	Proportion	2017	52.9	71.5	33.6

Table 4: ORS by Unit *(continued)*

Country	Unit	Metric	Year	Mean	Upper	Lower
Honduras	Patuca	Count	2000	1	1	0
Honduras	Patuca	Count	2017	1	1	0
Honduras	Petoa	Proportion	2000	53.4	71.8	35.3
Honduras	Petoa	Proportion	2017	56.0	74.4	37.8
Honduras	Petoa	Count	2000	1	1	0
Honduras	Petoa	Count	2017	1	1	0
Honduras	San Luis	Proportion	2000	57.1	75.4	37.3
Honduras	San Luis	Proportion	2017	57.5	77.3	39.5
Honduras	San Luis	Count	2000	1	1	0
Honduras	San Luis	Count	2017	1	1	0
Honduras	San Juan de Opoa	Proportion	2000	54.0	76.0	34.8
Honduras	San Juan de Opoa	Proportion	2017	57.9	75.8	36.3
Honduras	San Juan de Opoa	Count	2000	1	1	0
Honduras	San Juan de Opoa	Count	2017	1	1	0
Honduras	Vado Ancho	Proportion	2000	61.6	77.3	42.7
Honduras	Vado Ancho	Proportion	2017	60.8	75.8	43.5
Honduras	Vado Ancho	Count	2000	1	1	0
Honduras	Vado Ancho	Count	2017	1	1	0
Honduras	San Antonio de Oriente	Proportion	2000	60.0	76.5	39.6
Honduras	San Antonio de Oriente	Proportion	2017	58.0	76.1	37.2
Honduras	San Antonio de Oriente	Count	2000	1	1	0
Honduras	San Antonio de Oriente	Count	2017	1	1	0
Honduras	Yamaranguila	Proportion	2000	58.4	75.0	41.2
Honduras	Yamaranguila	Proportion	2017	64.6	79.9	46.9
Honduras	Yamaranguila	Count	2000	1	1	0
Honduras	Yamaranguila	Count	2017	1	1	0
Honduras	Santa María	Proportion	2000	60.8	77.7	40.5
Honduras	Santa María	Proportion	2017	64.7	80.4	45.7
Honduras	Santa María	Count	2000	1	1	0
Honduras	Santa María	Count	2017	1	1	0
Honduras	San Francisco	Proportion	2000	57.9	75.7	38.7
Honduras	San Francisco	Proportion	2017	64.7	81.3	45.7
Honduras	San Francisco	Count	2000	1	1	0
Honduras	San Francisco	Count	2017	1	1	0
Honduras	Salamá	Proportion	2000	57.7	74.7	36.4
Honduras	Salamá	Proportion	2017	54.2	73.7	35.3
Honduras	Salamá	Count	2000	1	1	0
Honduras	Salamá	Count	2017	1	1	0
Honduras	Protección	Proportion	2000	53.1	73.3	35.4
Honduras	Protección	Proportion	2017	57.1	76.5	37.3
Honduras	Protección	Count	2000	1	1	0
Honduras	Protección	Count	2017	1	1	0
Honduras	San Sebastián	Proportion	2000	60.8	77.4	40.6
Honduras	San Sebastián	Proportion	2017	62.5	78.8	43.0
Honduras	San Sebastián	Count	2000	1	1	0
Honduras	San Sebastián	Count	2017	1	1	0
Honduras	San Nicolás	Proportion	2000	52.1	71.5	35.0
Honduras	San Nicolás	Proportion	2017	55.9	74.4	35.4
Honduras	San Nicolás	Count	2000	1	1	0

Table 4: ORS by Unit *(continued)*

Country	Unit	Metric	Year	Mean	Upper	Lower
Honduras	San Nicolás	Count	2017	1	1	0
Honduras	Yauyupe	Proportion	2000	61.6	76.9	40.6
Honduras	Yauyupe	Proportion	2017	60.4	78.3	41.6
Honduras	Yauyupe	Count	2000	1	1	0
Honduras	Yauyupe	Count	2017	1	1	0
Honduras	San Buenaventura	Proportion	2000	60.1	76.2	39.6
Honduras	San Buenaventura	Proportion	2017	59.0	75.4	38.6
Honduras	San Buenaventura	Count	2000	1	1	0
Honduras	San Buenaventura	Count	2017	1	1	0
Honduras	Santiago de Puringla	Proportion	2000	59.8	78.4	40.9
Honduras	Santiago de Puringla	Proportion	2017	63.7	80.7	46.1
Honduras	Santiago de Puringla	Count	2000	1	1	0
Honduras	Santiago de Puringla	Count	2017	1	1	0
Honduras	San Juan Guarita	Proportion	2000	57.6	75.9	37.0
Honduras	San Juan Guarita	Proportion	2017	65.3	81.4	47.6
Honduras	San Juan Guarita	Count	2000	1	1	0
Honduras	San Juan Guarita	Count	2017	1	1	0
Honduras	San Esteban	Proportion	2000	56.3	73.9	38.2
Honduras	San Esteban	Proportion	2017	53.9	70.7	34.4
Honduras	San Esteban	Count	2000	1	1	0
Honduras	San Esteban	Count	2017	1	1	0
Honduras	Quimistán	Proportion	2000	52.7	71.4	35.4
Honduras	Quimistán	Proportion	2017	55.1	74.8	36.1
Honduras	Quimistán	Count	2000	1	1	0
Honduras	Quimistán	Count	2017	1	1	0
Honduras	Siguatepeque	Proportion	2000	57.8	77.1	38.2
Honduras	Siguatepeque	Proportion	2017	60.4	78.5	41.9
Honduras	Siguatepeque	Count	2000	1	1	0
Honduras	Siguatepeque	Count	2017	1	1	0
Honduras	San Pedro	Proportion	2000	54.9	75.4	34.5
Honduras	San Pedro	Proportion	2017	60.1	77.4	40.3
Honduras	San Pedro	Count	2000	1	1	0
Honduras	San Pedro	Count	2017	1	1	0
Honduras	Yuscarán	Proportion	2000	61.8	77.6	42.8
Honduras	Yuscarán	Proportion	2017	59.1	75.1	39.9
Honduras	Yuscarán	Count	2000	1	1	0
Honduras	Yuscarán	Count	2017	1	1	0
Honduras	San Ignacio	Proportion	2000	57.6	74.8	37.4
Honduras	San Ignacio	Proportion	2017	55.7	74.9	35.2
Honduras	San Ignacio	Count	2000	1	1	0
Honduras	San Ignacio	Count	2017	1	1	0
Honduras	Yarula	Proportion	2000	59.1	74.6	39.2
Honduras	Yarula	Proportion	2017	65.0	80.7	44.4
Honduras	Yarula	Count	2000	1	1	0
Honduras	Yarula	Count	2017	1	1	0
Honduras	San Manuel Colohete	Proportion	2000	54.7	73.8	35.7
Honduras	San Manuel Colohete	Proportion	2017	61.2	78.4	42.4
Honduras	San Manuel Colohete	Count	2000	1	1	0
Honduras	San Manuel Colohete	Count	2017	1	1	0

Table 4: ORS by Unit *(continued)*

Country	Unit	Metric	Year	Mean	Upper	Lower
Honduras	San Francisco de Becerra	Proportion	2000	57.7	74.5	39.7
Honduras	San Francisco de Becerra	Proportion	2017	52.3	71.2	32.5
Honduras	San Francisco de Becerra	Count	2000	1	1	0
Honduras	San Francisco de Becerra	Count	2017	1	1	0
Honduras	San Francisco de Ojuera	Proportion	2000	55.9	75.0	38.8
Honduras	San Francisco de Ojuera	Proportion	2017	60.6	77.6	41.3
Honduras	San Francisco de Ojuera	Count	2000	1	1	0
Honduras	San Francisco de Ojuera	Count	2017	1	1	0
Honduras	Taulabe	Proportion	2000	55.9	75.8	38.8
Honduras	Taulabe	Proportion	2017	58.7	76.5	41.2
Honduras	Taulabe	Count	2000	1	1	0
Honduras	Taulabe	Count	2017	1	1	0
Honduras	Santa Rita	Proportion	2000	53.5	72.2	35.1
Honduras	Santa Rita	Proportion	2017	57.5	74.4	38.1
Honduras	Santa Rita	Count	2000	1	1	0
Honduras	Santa Rita	Count	2017	1	1	0
Honduras	San Juan de Flores	Proportion	2000	59.8	76.7	40.0
Honduras	San Juan de Flores	Proportion	2017	57.1	75.9	36.0
Honduras	San Juan de Flores	Count	2000	1	1	0
Honduras	San Juan de Flores	Count	2017	1	1	0
Honduras	San Marcos de Caiquín	Proportion	2000	55.6	75.7	36.4
Honduras	San Marcos de Caiquín	Proportion	2017	62.1	78.0	43.2
Honduras	San Marcos de Caiquín	Count	2000	1	1	0
Honduras	San Marcos de Caiquín	Count	2017	1	1	0
Honduras	San Francisco de la Paz	Proportion	2000	57.4	73.9	36.2
Honduras	San Francisco de la Paz	Proportion	2017	52.8	70.9	33.3
Honduras	San Francisco de la Paz	Count	2000	1	1	0
Honduras	San Francisco de la Paz	Count	2017	1	1	0
Honduras	San José de Colinas	Proportion	2000	53.7	73.5	36.7
Honduras	San José de Colinas	Proportion	2017	57.6	76.3	37.5
Honduras	San José de Colinas	Count	2000	1	1	0
Honduras	San José de Colinas	Count	2017	1	1	0
Honduras	Villa de San Antonio	Proportion	2000	60.1	77.4	41.3
Honduras	Villa de San Antonio	Proportion	2017	60.8	78.4	41.3
Honduras	Villa de San Antonio	Count	2000	1	1	0
Honduras	Villa de San Antonio	Count	2017	1	1	0
Honduras	Santa Rosa de Copán	Proportion	2000	54.4	74.3	35.6
Honduras	Santa Rosa de Copán	Proportion	2017	58.1	76.0	36.8
Honduras	Santa Rosa de Copán	Count	2000	1	1	0
Honduras	Santa Rosa de Copán	Count	2017	1	1	0
Honduras	San Miguelito	Proportion	2000	58.7	76.2	40.7
Honduras	San Miguelito	Proportion	2017	60.8	78.0	41.6
Honduras	San Miguelito	Count	2000	1	1	0
Honduras	San Miguelito	Count	2017	1	1	0
Honduras	San Rafael	Proportion	2000	54.7	74.6	36.1
Honduras	San Rafael	Proportion	2017	60.3	78.0	41.9
Honduras	San Rafael	Count	2000	1	1	0
Honduras	San Rafael	Count	2017	1	1	0
Honduras	Santa Maria del Real	Proportion	2000	57.4	73.9	39.6

Table 4: ORS by Unit *(continued)*

Country	Unit	Metric	Year	Mean	Upper	Lower
Honduras	Santa Maria del Real	Proportion	2017	52.7	72.2	32.3
Honduras	Santa Maria del Real	Count	2000	1	1	0
Honduras	Santa Maria del Real	Count	2017	1	1	0
Honduras	San Luis	Proportion	2000	53.5	72.5	36.8
Honduras	San Luis	Proportion	2017	57.8	76.9	38.5
Honduras	San Luis	Count	2000	1	1	0
Honduras	San Luis	Count	2017	1	1	0
Honduras	Trinidad	Proportion	2000	53.3	73.6	35.7
Honduras	Trinidad	Proportion	2017	56.5	76.4	34.8
Honduras	Trinidad	Count	2000	1	1	0
Honduras	Trinidad	Count	2017	1	1	0
Honduras	Santa Ana	Proportion	2000	60.1	75.8	39.5
Honduras	Santa Ana	Proportion	2017	58.9	76.1	39.5
Honduras	Santa Ana	Count	2000	1	1	0
Honduras	Santa Ana	Count	2017	1	1	0
Honduras	San Sebastian	Proportion	2000	55.1	73.6	36.1
Honduras	San Sebastian	Proportion	2017	62.1	78.2	45.4
Honduras	San Sebastian	Count	2000	1	1	0
Honduras	San Sebastian	Count	2017	1	1	0
Honduras	Silca	Proportion	2000	57.9	76.0	38.6
Honduras	Silca	Proportion	2017	53.6	72.6	33.9
Honduras	Silca	Count	2000	1	1	0
Honduras	Silca	Count	2017	1	1	0
Honduras	San Marcos	Proportion	2000	52.9	72.0	36.7
Honduras	San Marcos	Proportion	2017	56.0	75.0	35.4
Honduras	San Marcos	Count	2000	1	1	0
Honduras	San Marcos	Count	2017	1	1	0
Honduras	Veracruz	Proportion	2000	53.4	73.2	34.2
Honduras	Veracruz	Proportion	2017	57.1	75.2	35.7
Honduras	Veracruz	Count	2000	1	1	0
Honduras	Veracruz	Count	2017	1	1	0
Honduras	Santa Lucía	Proportion	2000	60.0	78.4	39.1
Honduras	Santa Lucía	Proportion	2017	58.0	75.7	35.4
Honduras	Santa Lucía	Count	2000	1	1	0
Honduras	Santa Lucía	Count	2017	1	1	0
Honduras	Santa Cruz	Proportion	2000	56.1	75.9	37.9
Honduras	Santa Cruz	Proportion	2017	62.9	78.7	44.6
Honduras	Santa Cruz	Count	2000	1	1	0
Honduras	Santa Cruz	Count	2017	1	1	0
Honduras	Yocón	Proportion	2000	56.6	74.0	36.2
Honduras	Yocón	Proportion	2017	54.5	75.2	35.8
Honduras	Yocón	Count	2000	1	1	0
Honduras	Yocón	Count	2017	1	1	0
Honduras	San Nicolás	Proportion	2000	54.3	73.6	35.8
Honduras	San Nicolás	Proportion	2017	59.2	77.6	40.6
Honduras	San Nicolás	Count	2000	1	1	0
Honduras	San Nicolás	Count	2017	1	1	0
Honduras	Talanga	Proportion	2000	58.7	76.3	40.5
Honduras	Talanga	Proportion	2017	56.3	75.2	34.6

Table 4: ORS by Unit *(continued)*

Country	Unit	Metric	Year	Mean	Upper	Lower
Honduras	Talanga	Count	2000	1	1	0
Honduras	Talanga	Count	2017	1	1	0
Honduras	Talgua	Proportion	2000	54.4	74.1	34.7
Honduras	Talgua	Proportion	2017	59.1	76.5	37.6
Honduras	Talgua	Count	2000	1	1	0
Honduras	Talgua	Count	2017	1	1	0
Honduras	San Pedro Zacapa	Proportion	2000	55.9	75.5	38.0
Honduras	San Pedro Zacapa	Proportion	2017	59.3	77.2	40.2
Honduras	San Pedro Zacapa	Count	2000	1	1	0
Honduras	San Pedro Zacapa	Count	2017	1	1	0
Honduras	Tatumbla	Proportion	2000	59.5	75.9	39.3
Honduras	Tatumbla	Proportion	2017	58.1	75.0	36.3
Honduras	Tatumbla	Count	2000	1	1	0
Honduras	Tatumbla	Count	2017	1	1	0
Honduras	Tambla	Proportion	2000	56.5	76.7	37.3
Honduras	Tambla	Proportion	2017	64.2	80.2	47.1
Honduras	Tambla	Count	2000	1	1	0
Honduras	Tambla	Count	2017	1	1	0
Honduras	San Vicente Centenario	Proportion	2000	53.4	73.0	35.7
Honduras	San Vicente Centenario	Proportion	2017	58.0	77.0	40.3
Honduras	San Vicente Centenario	Count	2000	1	1	0
Honduras	San Vicente Centenario	Count	2017	1	1	0
Honduras	Valle de Ángeles	Proportion	2000	60.4	76.0	40.5
Honduras	Valle de Ángeles	Proportion	2017	57.7	75.6	36.4
Honduras	Valle de Ángeles	Count	2000	1	1	0
Honduras	Valle de Ángeles	Count	2017	1	1	0
Honduras	Tomalá	Proportion	2000	56.3	77.2	35.8
Honduras	Tomalá	Proportion	2017	64.0	79.8	45.3
Honduras	Tomalá	Count	2000	1	1	0
Honduras	Tomalá	Count	2017	1	1	0
Honduras	Santa Bárbara	Proportion	2000	54.3	73.8	36.9
Honduras	Santa Bárbara	Proportion	2017	57.9	77.6	38.3
Honduras	Santa Bárbara	Count	2000	1	1	0
Honduras	Santa Bárbara	Count	2017	1	1	0
Honduras	Vallecillo	Proportion	2000	58.0	75.7	38.6
Honduras	Vallecillo	Proportion	2017	58.5	76.5	38.9
Honduras	Vallecillo	Count	2000	1	1	0
Honduras	Vallecillo	Count	2017	1	1	0
Honduras	Valladolid	Proportion	2000	57.7	76.9	36.6
Honduras	Valladolid	Proportion	2017	65.3	80.6	47.4
Honduras	Valladolid	Count	2000	1	1	0
Honduras	Valladolid	Count	2017	1	1	0
Honduras	Santa Rita	Proportion	2000	54.8	74.1	36.3
Honduras	Santa Rita	Proportion	2017	59.2	77.1	39.2
Honduras	Santa Rita	Count	2000	1	1	0
Honduras	Santa Rita	Count	2017	1	1	0
Honduras	Villa de San Francisco	Proportion	2000	60.3	77.3	41.4
Honduras	Villa de San Francisco	Proportion	2017	57.1	74.1	36.3
Honduras	Villa de San Francisco	Count	2000	1	1	0

Table 4: ORS by Unit *(continued)*

Country	Unit	Metric	Year	Mean	Upper	Lower
Honduras	Villa de San Francisco	Count	2017	1	1	0
Honduras	Virginia	Proportion	2000	57.1	73.8	38.4
Honduras	Virginia	Proportion	2017	64.0	78.9	46.7
Honduras	Virginia	Count	2000	1	1	0
Honduras	Virginia	Count	2017	1	1	0
Honduras	Trinidad de Copán	Proportion	2000	53.5	71.3	37.0
Honduras	Trinidad de Copán	Proportion	2017	56.8	76.8	38.5
Honduras	Trinidad de Copán	Count	2000	1	1	0
Honduras	Trinidad de Copán	Count	2017	1	1	0
India	Nicobars	Proportion	2000	48.3	65.3	35.0
India	Nicobars	Proportion	2017	68.9	79.8	55.6
India	Nicobars	Count	2000	0	1	0
India	Nicobars	Count	2017	1	1	1
India	Anantapur	Proportion	2000	42.1	59.1	27.2
India	Anantapur	Proportion	2017	64.6	76.3	52.4
India	Anantapur	Count	2000	0	1	0
India	Anantapur	Count	2017	1	1	1
India	Anjaw	Proportion	2000	43.7	62.2	28.8
India	Anjaw	Proportion	2017	66.7	81.1	50.0
India	Anjaw	Count	2000	0	1	0
India	Anjaw	Count	2017	1	1	0
India	Baksa	Proportion	2000	41.0	57.7	25.4
India	Baksa	Proportion	2017	60.9	77.1	44.0
India	Baksa	Count	2000	0	1	0
India	Baksa	Count	2017	1	1	0
India	Araria	Proportion	2000	25.9	43.7	13.8
India	Araria	Proportion	2017	50.8	68.3	32.3
India	Araria	Count	2000	0	0	0
India	Araria	Count	2017	1	1	0
India	Baloda Bazar	Proportion	2000	36.7	56.5	21.9
India	Baloda Bazar	Proportion	2017	59.0	73.8	40.8
India	Baloda Bazar	Count	2000	0	1	0
India	Baloda Bazar	Count	2017	1	1	0
India	North Goa	Proportion	2000	48.9	65.9	27.9
India	North Goa	Proportion	2017	70.7	84.6	51.7
India	North Goa	Count	2000	0	1	0
India	North Goa	Count	2017	1	1	1
India	Ahmedabad	Proportion	2000	39.5	59.7	24.9
India	Ahmedabad	Proportion	2017	62.3	76.8	44.8
India	Ahmedabad	Count	2000	0	1	0
India	Ahmedabad	Count	2017	1	1	0
India	Ambala	Proportion	2000	37.5	55.2	21.6
India	Ambala	Proportion	2017	61.2	76.1	41.8
India	Ambala	Count	2000	0	1	0
India	Ambala	Count	2017	1	1	0
India	Bilaspur	Proportion	2000	46.1	64.1	28.2
India	Bilaspur	Proportion	2017	68.3	82.1	50.2
India	Bilaspur	Count	2000	0	1	0
India	Bilaspur	Count	2017	1	1	1

Table 4: ORS by Unit *(continued)*

Country	Unit	Metric	Year	Mean	Upper	Lower
India	Anantnag	Proportion	2000	40.9	59.3	25.3
India	Anantnag	Proportion	2017	62.5	78.1	46.3
India	Anantnag	Count	2000	0	1	0
India	Anantnag	Count	2017	1	1	0
India	Bokaro	Proportion	2000	40.2	57.4	25.4
India	Bokaro	Proportion	2017	60.8	75.8	43.0
India	Bokaro	Count	2000	0	1	0
India	Bokaro	Count	2017	1	1	0
India	Bagalkot	Proportion	2000	40.9	57.3	26.4
India	Bagalkot	Proportion	2017	64.4	77.3	48.7
India	Bagalkot	Count	2000	0	1	0
India	Bagalkot	Count	2017	1	1	0
India	Alappuzha	Proportion	2000	48.6	68.4	28.4
India	Alappuzha	Proportion	2017	70.9	84.1	54.2
India	Alappuzha	Count	2000	0	1	0
India	Alappuzha	Count	2017	1	1	1
India	Agar Malwa	Proportion	2000	36.7	55.4	22.5
India	Agar Malwa	Proportion	2017	56.9	73.8	38.9
India	Agar Malwa	Count	2000	0	1	0
India	Agar Malwa	Count	2017	1	1	0
India	Ahmadnagar	Proportion	2000	44.9	60.8	29.1
India	Ahmadnagar	Proportion	2017	64.6	78.5	49.4
India	Ahmadnagar	Count	2000	0	1	0
India	Ahmadnagar	Count	2017	1	1	0
India	Bishnupur	Proportion	2000	44.7	62.0	29.0
India	Bishnupur	Proportion	2017	64.2	79.4	46.9
India	Bishnupur	Count	2000	0	1	0
India	Bishnupur	Count	2017	1	1	0
India	East Garo Hills	Proportion	2000	39.6	54.7	25.3
India	East Garo Hills	Proportion	2017	64.6	78.3	45.9
India	East Garo Hills	Count	2000	0	1	0
India	East Garo Hills	Count	2017	1	1	0
India	Aizawl	Proportion	2000	42.1	57.5	27.2
India	Aizawl	Proportion	2017	63.6	77.2	49.7
India	Aizawl	Count	2000	0	1	0
India	Aizawl	Count	2017	1	1	0
India	Dimapur	Proportion	2000	42.7	63.0	23.0
India	Dimapur	Proportion	2017	63.7	78.9	43.5
India	Dimapur	Count	2000	0	1	0
India	Dimapur	Count	2017	1	1	0
India	West	Proportion	2000	44.3	63.8	27.6
India	West	Proportion	2017	63.7	79.4	47.0
India	West	Count	2000	0	1	0
India	West	Count	2017	1	1	0
India	Angul	Proportion	2000	43.3	58.5	29.2
India	Angul	Proportion	2017	64.6	79.6	46.9
India	Angul	Count	2000	0	1	0
India	Angul	Count	2017	1	1	0
India	Amritsar	Proportion	2000	36.8	54.9	21.7

Table 4: ORS by Unit *(continued)*

Country	Unit	Metric	Year	Mean	Upper	Lower
India	Amritsar	Proportion	2017	59.2	76.6	40.5
India	Amritsar	Count	2000	0	1	0
India	Amritsar	Count	2017	1	1	0
India	Ajmer	Proportion	2000	34.8	52.2	19.8
India	Ajmer	Proportion	2017	57.5	73.7	42.3
India	Ajmer	Count	2000	0	1	0
India	Ajmer	Count	2017	1	1	0
India	East Sikkim	Proportion	2000	47.6	66.8	28.8
India	East Sikkim	Proportion	2017	68.9	82.6	49.2
India	East Sikkim	Count	2000	0	1	0
India	East Sikkim	Count	2017	1	1	0
India	Ariyalur	Proportion	2000	43.0	60.7	26.3
India	Ariyalur	Proportion	2017	64.1	79.0	47.3
India	Ariyalur	Count	2000	0	1	0
India	Ariyalur	Count	2017	1	1	0
India	Adilabad	Proportion	2000	47.1	65.4	31.1
India	Adilabad	Proportion	2017	65.4	80.7	47.7
India	Adilabad	Count	2000	0	1	0
India	Adilabad	Count	2017	1	1	0
India	Dhalai	Proportion	2000	43.8	62.4	26.5
India	Dhalai	Proportion	2017	64.8	79.6	48.7
India	Dhalai	Count	2000	0	1	0
India	Dhalai	Count	2017	1	1	0
India	Agra	Proportion	2000	26.8	42.1	15.5
India	Agra	Proportion	2017	47.8	64.9	29.5
India	Agra	Count	2000	0	0	0
India	Agra	Count	2017	0	1	0
India	Almora	Proportion	2000	44.1	64.1	27.9
India	Almora	Proportion	2017	64.5	77.2	48.9
India	Almora	Count	2000	0	1	0
India	Almora	Count	2017	1	1	0
India	Alipurduar	Proportion	2000	50.5	66.3	29.6
India	Alipurduar	Proportion	2017	70.2	84.0	56.4
India	Alipurduar	Count	2000	1	1	0
India	Alipurduar	Count	2017	1	1	1
India	North & Middle Andaman	Proportion	2000	44.9	59.9	28.7
India	North & Middle Andaman	Proportion	2017	68.3	81.1	53.9
India	North & Middle Andaman	Count	2000	0	1	0
India	North & Middle Andaman	Count	2017	1	1	1
India	Chittoor	Proportion	2000	42.8	58.1	28.8
India	Chittoor	Proportion	2017	64.8	77.0	49.5
India	Chittoor	Count	2000	0	1	0
India	Chittoor	Count	2017	1	1	0
India	Changlang	Proportion	2000	41.6	59.5	27.3
India	Changlang	Proportion	2017	64.4	78.2	43.4
India	Changlang	Count	2000	0	1	0
India	Changlang	Count	2017	1	1	0
India	Barpeta	Proportion	2000	39.8	57.7	22.8
India	Barpeta	Proportion	2017	60.5	75.3	42.7

Table 4: ORS by Unit *(continued)*

Country	Unit	Metric	Year	Mean	Upper	Lower
India	Barpeta	Count	2000	0	1	0
India	Barpeta	Count	2017	1	1	0
India	Arwal	Proportion	2000	24.0	41.0	12.6
India	Arwal	Proportion	2017	46.2	65.7	27.8
India	Arwal	Count	2000	0	0	0
India	Arwal	Count	2017	0	1	0
India	Balod	Proportion	2000	40.1	58.2	22.1
India	Balod	Proportion	2017	59.7	74.0	43.4
India	Balod	Count	2000	0	1	0
India	Balod	Count	2017	1	1	0
India	South Goa	Proportion	2000	47.3	64.9	29.4
India	South Goa	Proportion	2017	70.5	84.1	55.1
India	South Goa	Count	2000	0	1	0
India	South Goa	Count	2017	1	1	1
India	Amreli	Proportion	2000	38.8	56.7	22.5
India	Amreli	Proportion	2017	59.4	74.5	42.9
India	Amreli	Count	2000	0	1	0
India	Amreli	Count	2017	1	1	0
India	Bhiwani	Proportion	2000	31.7	51.4	17.6
India	Bhiwani	Proportion	2017	54.1	71.0	37.8
India	Bhiwani	Count	2000	0	1	0
India	Bhiwani	Count	2017	1	1	0
India	Chamba	Proportion	2000	46.9	64.3	28.2
India	Chamba	Proportion	2017	68.0	81.7	50.8
India	Chamba	Count	2000	0	1	0
India	Chamba	Count	2017	1	1	1
India	Badgam	Proportion	2000	41.8	59.2	25.3
India	Badgam	Proportion	2017	63.0	77.0	44.9
India	Badgam	Count	2000	0	1	0
India	Badgam	Count	2017	1	1	0
India	Chatra	Proportion	2000	38.2	54.8	22.9
India	Chatra	Proportion	2017	57.8	75.3	39.6
India	Chatra	Count	2000	0	1	0
India	Chatra	Count	2017	1	1	0
India	Bangalore Rural	Proportion	2000	45.8	64.3	28.5
India	Bangalore Rural	Proportion	2017	67.6	80.8	50.4
India	Bangalore Rural	Count	2000	0	1	0
India	Bangalore Rural	Count	2017	1	1	1
India	Ernakulam	Proportion	2000	49.2	70.2	30.7
India	Ernakulam	Proportion	2017	71.1	83.6	53.3
India	Ernakulam	Count	2000	0	1	0
India	Ernakulam	Count	2017	1	1	1
India	Alirajpur	Proportion	2000	38.7	58.3	22.0
India	Alirajpur	Proportion	2017	56.4	75.6	38.1
India	Alirajpur	Count	2000	0	1	0
India	Alirajpur	Count	2017	1	1	0
India	Akola	Proportion	2000	43.8	59.6	26.8
India	Akola	Proportion	2017	63.7	77.0	45.8
India	Akola	Count	2000	0	1	0

Table 4: ORS by Unit *(continued)*

Country	Unit	Metric	Year	Mean	Upper	Lower
India	Akola	Count	2017	1	1	0
India	Chandel	Proportion	2000	44.3	60.8	28.0
India	Chandel	Proportion	2017	63.8	79.5	46.5
India	Chandel	Count	2000	0	1	0
India	Chandel	Count	2017	1	1	0
India	East Khasi Hills	Proportion	2000	45.3	63.9	29.4
India	East Khasi Hills	Proportion	2017	69.4	82.5	52.9
India	East Khasi Hills	Count	2000	0	1	0
India	East Khasi Hills	Count	2017	1	1	1
India	Champhai	Proportion	2000	43.3	58.8	28.0
India	Champhai	Proportion	2017	64.3	78.7	49.0
India	Champhai	Count	2000	0	1	0
India	Champhai	Count	2017	1	1	0
India	Kiphire	Proportion	2000	47.0	68.6	28.8
India	Kiphire	Proportion	2017	65.9	83.2	47.0
India	Kiphire	Count	2000	0	1	0
India	Kiphire	Count	2017	1	1	0
India	Central	Proportion	2000	46.5	67.3	27.9
India	Central	Proportion	2017	65.0	80.6	46.7
India	Central	Count	2000	0	1	0
India	Central	Count	2017	1	1	0
India	Balangir	Proportion	2000	41.4	57.2	24.3
India	Balangir	Proportion	2017	65.7	79.1	49.9
India	Balangir	Count	2000	0	1	0
India	Balangir	Count	2017	1	1	0
India	Barnala	Proportion	2000	34.7	53.3	19.4
India	Barnala	Proportion	2017	56.8	73.7	42.1
India	Barnala	Count	2000	0	1	0
India	Barnala	Count	2017	1	1	0
India	Alwar	Proportion	2000	31.0	47.2	17.4
India	Alwar	Proportion	2017	51.5	68.9	36.0
India	Alwar	Count	2000	0	0	0
India	Alwar	Count	2017	1	1	0
India	North Sikkim	Proportion	2000	46.1	64.4	28.8
India	North Sikkim	Proportion	2017	67.5	80.8	49.7
India	North Sikkim	Count	2000	0	1	0
India	North Sikkim	Count	2017	1	1	0
India	Chennai	Proportion	2000	49.2	67.5	29.5
India	Chennai	Proportion	2017	68.4	82.6	50.6
India	Chennai	Count	2000	0	1	0
India	Chennai	Count	2017	1	1	1
India	Hyderabad	Proportion	2000	49.2	68.4	31.1
India	Hyderabad	Proportion	2017	69.1	82.0	51.2
India	Hyderabad	Count	2000	0	1	0
India	Hyderabad	Count	2017	1	1	1
India	Gomati	Proportion	2000	44.0	64.9	25.7
India	Gomati	Proportion	2017	65.4	80.7	48.2
India	Gomati	Count	2000	0	1	0
India	Gomati	Count	2017	1	1	0

Table 4: ORS by Unit *(continued)*

Country	Unit	Metric	Year	Mean	Upper	Lower
India	Aligarh	Proportion	2000	24.7	38.2	13.0
India	Aligarh	Proportion	2017	44.5	62.4	27.6
India	Aligarh	Count	2000	0	0	0
India	Aligarh	Count	2017	0	1	0
India	Bageshwar	Proportion	2000	43.8	63.0	28.7
India	Bageshwar	Proportion	2017	64.0	78.2	47.9
India	Bageshwar	Count	2000	0	1	0
India	Bageshwar	Count	2017	1	1	0
India	Bankura	Proportion	2000	43.1	58.8	29.5
India	Bankura	Proportion	2017	63.2	80.8	46.7
India	Bankura	Count	2000	0	1	0
India	Bankura	Count	2017	1	1	0
India	South Andaman	Proportion	2000	45.9	61.9	28.7
India	South Andaman	Proportion	2017	69.0	82.7	53.0
India	South Andaman	Count	2000	0	1	0
India	South Andaman	Count	2017	1	1	1
India	East Godavari	Proportion	2000	39.9	59.6	26.3
India	East Godavari	Proportion	2017	61.4	74.4	45.1
India	East Godavari	Count	2000	0	1	0
India	East Godavari	Count	2017	1	1	0
India	Dibang Valley	Proportion	2000	42.1	56.9	28.4
India	Dibang Valley	Proportion	2017	64.8	79.5	49.8
India	Dibang Valley	Count	2000	0	1	0
India	Dibang Valley	Count	2017	1	1	0
India	Bongaigaon	Proportion	2000	42.5	59.5	25.0
India	Bongaigaon	Proportion	2017	63.6	78.9	47.2
India	Bongaigaon	Count	2000	0	1	0
India	Bongaigaon	Count	2017	1	1	0
India	Aurangabad	Proportion	2000	25.2	40.8	12.8
India	Aurangabad	Proportion	2017	48.3	67.2	32.1
India	Aurangabad	Count	2000	0	0	0
India	Aurangabad	Count	2017	0	1	0
India	Balrampur	Proportion	2000	38.6	55.5	24.8
India	Balrampur	Proportion	2017	60.2	74.8	43.8
India	Balrampur	Count	2000	0	1	0
India	Balrampur	Count	2017	1	1	0
India	Anand	Proportion	2000	35.6	53.5	21.0
India	Anand	Proportion	2017	56.6	72.1	38.8
India	Anand	Count	2000	0	1	0
India	Anand	Count	2017	1	1	0
India	Faridabad	Proportion	2000	33.7	49.9	17.4
India	Faridabad	Proportion	2017	55.7	74.6	38.4
India	Faridabad	Count	2000	0	0	0
India	Faridabad	Count	2017	1	1	0
India	Hamirpur	Proportion	2000	44.5	63.4	26.5
India	Hamirpur	Proportion	2017	66.6	81.5	47.3
India	Hamirpur	Count	2000	0	1	0
India	Hamirpur	Count	2017	1	1	0
India	Bandipore	Proportion	2000	41.4	59.9	24.6

Table 4: ORS by Unit *(continued)*

Country	Unit	Metric	Year	Mean	Upper	Lower
India	Bandipore	Proportion	2017	62.6	78.5	46.1
India	Bandipore	Count	2000	0	1	0
India	Bandipore	Count	2017	1	1	0
India	Deoghar	Proportion	2000	39.1	57.5	22.8
India	Deoghar	Proportion	2017	59.6	76.0	42.5
India	Deoghar	Count	2000	0	1	0
India	Deoghar	Count	2017	1	1	0
India	Bangalore	Proportion	2000	49.5	68.2	29.7
India	Bangalore	Proportion	2017	70.3	84.4	50.1
India	Bangalore	Count	2000	0	1	0
India	Bangalore	Count	2017	1	1	1
India	Idukki	Proportion	2000	49.1	65.9	32.1
India	Idukki	Proportion	2017	69.9	83.7	53.0
India	Idukki	Count	2000	0	1	0
India	Idukki	Count	2017	1	1	1
India	Anuppur	Proportion	2000	39.0	57.2	23.2
India	Anuppur	Proportion	2017	61.2	75.5	44.1
India	Anuppur	Count	2000	0	1	0
India	Anuppur	Count	2017	1	1	0
India	Amravati	Proportion	2000	43.9	58.9	28.0
India	Amravati	Proportion	2017	64.0	77.5	48.3
India	Amravati	Count	2000	0	1	0
India	Amravati	Count	2017	1	1	0
India	Churachandpur	Proportion	2000	45.2	62.6	29.6
India	Churachandpur	Proportion	2017	64.8	78.5	48.4
India	Churachandpur	Count	2000	0	1	0
India	Churachandpur	Count	2017	1	1	0
India	East Jaintia Hills	Proportion	2000	42.9	58.1	26.2
India	East Jaintia Hills	Proportion	2017	67.0	81.3	49.7
India	East Jaintia Hills	Count	2000	0	1	0
India	East Jaintia Hills	Count	2017	1	1	0
India	Kolasib	Proportion	2000	44.3	60.0	28.2
India	Kolasib	Proportion	2017	66.1	80.6	52.6
India	Kolasib	Count	2000	0	1	0
India	Kolasib	Count	2017	1	1	1
India	Kohima	Proportion	2000	47.2	65.2	29.3
India	Kohima	Proportion	2017	66.5	81.3	46.2
India	Kohima	Count	2000	0	1	0
India	Kohima	Count	2017	1	1	0
India	East	Proportion	2000	43.9	63.8	25.5
India	East	Proportion	2017	62.7	78.8	44.5
India	East	Count	2000	0	1	0
India	East	Count	2017	1	1	0
India	Baleshwar	Proportion	2000	45.3	62.8	28.2
India	Baleshwar	Proportion	2017	64.5	80.5	47.8
India	Baleshwar	Count	2000	0	1	0
India	Baleshwar	Count	2017	1	1	0
India	Bathinda	Proportion	2000	34.2	53.2	18.2
India	Bathinda	Proportion	2017	56.6	71.4	41.5

Table 4: ORS by Unit *(continued)*

Country	Unit	Metric	Year	Mean	Upper	Lower
India	Bathinda	Count	2000	0	1	0
India	Bathinda	Count	2017	1	1	0
India	Banswara	Proportion	2000	34.3	51.2	21.6
India	Banswara	Proportion	2017	56.7	73.5	39.6
India	Banswara	Count	2000	0	1	0
India	Banswara	Count	2017	1	1	0
India	South Sikkim	Proportion	2000	46.1	63.8	27.6
India	South Sikkim	Proportion	2017	68.1	81.5	51.8
India	South Sikkim	Count	2000	0	1	0
India	South Sikkim	Count	2017	1	1	1
India	Coimbatore	Proportion	2000	45.1	64.6	27.4
India	Coimbatore	Proportion	2017	67.6	82.1	51.8
India	Coimbatore	Count	2000	0	1	0
India	Coimbatore	Count	2017	1	1	1
India	Karimnagar	Proportion	2000	44.5	62.4	26.7
India	Karimnagar	Proportion	2017	64.6	81.8	45.1
India	Karimnagar	Count	2000	0	1	0
India	Karimnagar	Count	2017	1	1	0
India	Khowai	Proportion	2000	43.7	64.7	25.4
India	Khowai	Proportion	2017	65.3	80.5	47.8
India	Khowai	Count	2000	0	1	0
India	Khowai	Count	2017	1	1	0
India	Allahabad	Proportion	2000	26.2	39.9	14.8
India	Allahabad	Proportion	2017	49.5	65.1	31.1
India	Allahabad	Count	2000	0	0	0
India	Allahabad	Count	2017	0	1	0
India	Chamoli	Proportion	2000	45.4	60.9	30.6
India	Chamoli	Proportion	2017	65.4	81.0	46.8
India	Chamoli	Count	2000	0	1	0
India	Chamoli	Count	2017	1	1	0
India	Purba Barddhaman	Proportion	2000	42.1	59.2	27.0
India	Purba Barddhaman	Proportion	2017	60.8	76.4	45.7
India	Purba Barddhaman	Count	2000	0	1	0
India	Purba Barddhaman	Count	2017	1	1	0
India	Chandigarh	Proportion	2000	46.3	64.8	28.4
India	Chandigarh	Proportion	2017	68.8	81.3	50.4
India	Chandigarh	Count	2000	0	1	0
India	Chandigarh	Count	2017	1	1	1
India	Guntur	Proportion	2000	39.7	58.7	25.4
India	Guntur	Proportion	2017	61.6	75.5	47.6
India	Guntur	Count	2000	0	1	0
India	Guntur	Count	2017	1	1	0
India	East Kameng	Proportion	2000	41.9	60.7	27.4
India	East Kameng	Proportion	2017	65.0	80.4	48.2
India	East Kameng	Count	2000	0	1	0
India	East Kameng	Count	2017	1	1	0
India	Cachar	Proportion	2000	42.9	61.1	24.5
India	Cachar	Proportion	2017	62.8	77.3	46.2
India	Cachar	Count	2000	0	1	0

Table 4: ORS by Unit *(continued)*

Country	Unit	Metric	Year	Mean	Upper	Lower
India	Cachar	Count	2017	1	1	0
India	Banka	Proportion	2000	25.9	41.6	12.8
India	Banka	Proportion	2017	50.0	66.7	31.6
India	Banka	Count	2000	0	0	0
India	Banka	Count	2017	0	1	0
India	Bastar	Proportion	2000	40.9	61.4	24.4
India	Bastar	Proportion	2017	62.1	76.3	44.8
India	Bastar	Count	2000	0	1	0
India	Bastar	Count	2017	1	1	0
India	Arvalli	Proportion	2000	37.1	56.2	22.5
India	Arvalli	Proportion	2017	61.6	77.1	43.5
India	Arvalli	Count	2000	0	1	0
India	Arvalli	Count	2017	1	1	0
India	Fatehabad	Proportion	2000	31.8	47.7	19.3
India	Fatehabad	Proportion	2017	55.0	72.0	39.4
India	Fatehabad	Count	2000	0	0	0
India	Fatehabad	Count	2017	1	1	0
India	Kangra	Proportion	2000	44.9	62.2	27.6
India	Kangra	Proportion	2017	67.2	81.1	50.8
India	Kangra	Count	2000	0	1	0
India	Kangra	Count	2017	1	1	1
India	Baramulla	Proportion	2000	40.7	58.7	25.4
India	Baramulla	Proportion	2017	62.3	77.0	45.0
India	Baramulla	Count	2000	0	1	0
India	Baramulla	Count	2017	1	1	0
India	Dhanbad	Proportion	2000	40.3	61.3	25.4
India	Dhanbad	Proportion	2017	61.0	76.5	44.9
India	Dhanbad	Count	2000	0	1	0
India	Dhanbad	Count	2017	1	1	0
India	Belagavi	Proportion	2000	44.8	60.4	30.3
India	Belagavi	Proportion	2017	65.4	78.2	48.7
India	Belagavi	Count	2000	0	1	0
India	Belagavi	Count	2017	1	1	0
India	Kannur	Proportion	2000	47.2	65.8	31.4
India	Kannur	Proportion	2017	69.8	83.2	53.1
India	Kannur	Count	2000	0	1	0
India	Kannur	Count	2017	1	1	1
India	Ashoknagar	Proportion	2000	36.5	52.8	22.3
India	Ashoknagar	Proportion	2017	57.9	72.0	41.2
India	Ashoknagar	Count	2000	0	1	0
India	Ashoknagar	Count	2017	1	1	0
India	Aurangabad	Proportion	2000	45.7	63.6	30.9
India	Aurangabad	Proportion	2017	65.4	77.9	49.6
India	Aurangabad	Count	2000	0	1	0
India	Aurangabad	Count	2017	1	1	0
India	Imphal East	Proportion	2000	45.7	63.3	29.4
India	Imphal East	Proportion	2017	65.0	81.0	48.6
India	Imphal East	Count	2000	0	1	0
India	Imphal East	Count	2017	1	1	0

Table 4: ORS by Unit *(continued)*

Country	Unit	Metric	Year	Mean	Upper	Lower
India	North Garo Hills	Proportion	2000	39.1	54.8	24.0
India	North Garo Hills	Proportion	2017	62.0	76.9	43.5
India	North Garo Hills	Count	2000	0	1	0
India	North Garo Hills	Count	2017	1	1	0
India	Lawangtlai	Proportion	2000	41.3	57.2	26.4
India	Lawangtlai	Proportion	2017	65.4	78.8	49.2
India	Lawangtlai	Count	2000	0	1	0
India	Lawangtlai	Count	2017	1	1	0
India	Longleng	Proportion	2000	47.2	66.5	29.8
India	Longleng	Proportion	2017	65.2	81.2	47.1
India	Longleng	Count	2000	0	1	0
India	Longleng	Count	2017	1	1	0
India	New Delhi	Proportion	2000	46.4	66.4	28.1
India	New Delhi	Proportion	2017	65.0	80.5	47.0
India	New Delhi	Count	2000	0	1	0
India	New Delhi	Count	2017	1	1	0
India	Bargarh	Proportion	2000	40.4	56.3	23.4
India	Bargarh	Proportion	2017	64.8	80.5	49.3
India	Bargarh	Count	2000	0	1	0
India	Bargarh	Count	2017	1	1	0
India	Faridkot	Proportion	2000	34.7	51.7	19.8
India	Faridkot	Proportion	2017	57.6	73.9	41.2
India	Faridkot	Count	2000	0	1	0
India	Faridkot	Count	2017	1	1	0
India	Baran	Proportion	2000	32.3	46.2	18.6
India	Baran	Proportion	2017	55.1	71.1	38.8
India	Baran	Count	2000	0	0	0
India	Baran	Count	2017	1	1	0
India	West Sikkim	Proportion	2000	46.0	63.2	27.5
India	West Sikkim	Proportion	2017	67.8	82.7	51.3
India	West Sikkim	Count	2000	0	1	0
India	West Sikkim	Count	2017	1	1	1
India	Cuddalore	Proportion	2000	42.8	58.7	26.7
India	Cuddalore	Proportion	2017	63.3	77.9	45.4
India	Cuddalore	Count	2000	0	1	0
India	Cuddalore	Count	2017	1	1	0
India	Khammam	Proportion	2000	43.0	59.7	26.6
India	Khammam	Proportion	2017	63.2	78.1	47.9
India	Khammam	Count	2000	0	1	0
India	Khammam	Count	2017	1	1	0
India	North Tripura	Proportion	2000	45.9	64.4	29.6
India	North Tripura	Proportion	2017	66.2	80.9	50.4
India	North Tripura	Count	2000	0	1	0
India	North Tripura	Count	2017	1	1	1
India	Ambedkar Nagar	Proportion	2000	24.8	41.0	12.9
India	Ambedkar Nagar	Proportion	2017	45.8	63.4	30.1
India	Ambedkar Nagar	Count	2000	0	0	0
India	Ambedkar Nagar	Count	2017	0	1	0
India	Champawat	Proportion	2000	42.2	60.5	26.0

Table 4: ORS by Unit *(continued)*

Country	Unit	Metric	Year	Mean	Upper	Lower
India	Champawat	Proportion	2017	62.9	77.0	45.0
India	Champawat	Count	2000	0	1	0
India	Champawat	Count	2017	1	1	0
India	Birbhum	Proportion	2000	42.1	58.5	26.6
India	Birbhum	Proportion	2017	62.8	79.1	45.3
India	Birbhum	Count	2000	0	1	0
India	Birbhum	Count	2017	1	1	0
India	Dadra and Nagar Haveli	Proportion	2000	43.0	60.9	23.9
India	Dadra and Nagar Haveli	Proportion	2017	62.9	79.2	44.9
India	Dadra and Nagar Haveli	Count	2000	0	1	0
India	Dadra and Nagar Haveli	Count	2017	1	1	0
India	Krishna	Proportion	2000	40.6	58.0	26.1
India	Krishna	Proportion	2017	62.2	75.7	47.8
India	Krishna	Count	2000	0	1	0
India	Krishna	Count	2017	1	1	0
India	East Siang	Proportion	2000	42.5	61.1	25.9
India	East Siang	Proportion	2017	65.4	79.2	49.4
India	East Siang	Count	2000	0	1	0
India	East Siang	Count	2017	1	1	0
India	Chirang	Proportion	2000	42.9	60.5	25.3
India	Chirang	Proportion	2017	63.7	77.9	48.0
India	Chirang	Count	2000	0	1	0
India	Chirang	Count	2017	1	1	0
India	Begusarai	Proportion	2000	25.4	41.2	13.6
India	Begusarai	Proportion	2017	48.4	66.0	30.3
India	Begusarai	Count	2000	0	0	0
India	Begusarai	Count	2017	0	1	0
India	Bemetara	Proportion	2000	37.3	58.9	20.7
India	Bemetara	Proportion	2017	59.4	73.6	43.7
India	Bemetara	Count	2000	0	1	0
India	Bemetara	Count	2017	1	1	0
India	Banaskantha	Proportion	2000	36.3	52.6	23.2
India	Banaskantha	Proportion	2017	59.1	73.7	44.3
India	Banaskantha	Count	2000	0	1	0
India	Banaskantha	Count	2017	1	1	0
India	Gurugram	Proportion	2000	34.9	53.1	20.3
India	Gurugram	Proportion	2017	58.0	76.2	41.3
India	Gurugram	Count	2000	0	1	0
India	Gurugram	Count	2017	1	1	0
India	Kinnaur	Proportion	2000	47.3	63.1	31.3
India	Kinnaur	Proportion	2017	70.1	82.6	54.4
India	Kinnaur	Count	2000	0	1	0
India	Kinnaur	Count	2017	1	1	1
India	Doda	Proportion	2000	43.8	61.2	28.7
India	Doda	Proportion	2017	64.8	78.6	48.0
India	Doda	Count	2000	0	1	0
India	Doda	Count	2017	1	1	0
India	Dumka	Proportion	2000	38.4	55.6	23.7
India	Dumka	Proportion	2017	59.1	75.6	40.1

Table 4: ORS by Unit *(continued)*

Country	Unit	Metric	Year	Mean	Upper	Lower
India	Dumka	Count	2000	0	1	0
India	Dumka	Count	2017	1	1	0
India	Ballary	Proportion	2000	40.2	58.6	23.5
India	Ballary	Proportion	2017	63.1	77.0	49.9
India	Ballary	Count	2000	0	1	0
India	Ballary	Count	2017	1	1	0
India	Kasaragod	Proportion	2000	47.2	67.9	30.4
India	Kasaragod	Proportion	2017	70.3	83.6	53.9
India	Kasaragod	Count	2000	0	1	0
India	Kasaragod	Count	2017	1	1	1
India	Balaghat	Proportion	2000	38.9	53.7	22.8
India	Balaghat	Proportion	2017	60.0	72.3	43.7
India	Balaghat	Count	2000	0	1	0
India	Balaghat	Count	2017	1	1	0
India	Bhandara	Proportion	2000	42.2	57.6	25.3
India	Bhandara	Proportion	2017	64.1	77.1	49.1
India	Bhandara	Count	2000	0	1	0
India	Bhandara	Count	2017	1	1	0
India	Imphal West	Proportion	2000	45.0	63.1	28.7
India	Imphal West	Proportion	2017	64.3	80.5	46.9
India	Imphal West	Count	2000	0	1	0
India	Imphal West	Count	2017	1	1	0
India	Ri Bhoi	Proportion	2000	40.2	59.3	25.9
India	Ri Bhoi	Proportion	2017	63.4	77.1	46.8
India	Ri Bhoi	Count	2000	0	1	0
India	Ri Bhoi	Count	2017	1	1	0
India	Lunglei	Proportion	2000	42.2	57.9	26.8
India	Lunglei	Proportion	2017	66.3	80.9	49.4
India	Lunglei	Count	2000	0	1	0
India	Lunglei	Count	2017	1	1	0
India	Mokokchung	Proportion	2000	46.5	65.1	29.5
India	Mokokchung	Proportion	2017	65.2	81.1	47.1
India	Mokokchung	Count	2000	0	1	0
India	Mokokchung	Count	2017	1	1	0
India	North	Proportion	2000	45.4	65.6	26.9
India	North	Proportion	2017	64.0	80.0	46.0
India	North	Count	2000	0	1	0
India	North	Count	2017	1	1	0
India	Boudh	Proportion	2000	40.7	56.3	27.0
India	Boudh	Proportion	2017	63.7	78.3	46.3
India	Boudh	Count	2000	0	1	0
India	Boudh	Count	2017	1	1	0
India	Fatehgarh Sahib	Proportion	2000	38.8	55.3	22.6
India	Fatehgarh Sahib	Proportion	2017	61.3	75.3	45.0
India	Fatehgarh Sahib	Count	2000	0	1	0
India	Fatehgarh Sahib	Count	2017	1	1	0
India	Barmer	Proportion	2000	32.3	46.6	19.4
India	Barmer	Proportion	2017	53.1	67.5	37.9
India	Barmer	Count	2000	0	0	0

Table 4: ORS by Unit *(continued)*

Country	Unit	Metric	Year	Mean	Upper	Lower
India	Barmer	Count	2017	1	1	0
India	Dharmapuri	Proportion	2000	44.1	62.5	23.1
India	Dharmapuri	Proportion	2017	65.7	79.9	49.1
India	Dharmapuri	Count	2000	0	1	0
India	Dharmapuri	Count	2017	1	1	0
India	Mahbubnagar	Proportion	2000	41.4	57.3	25.3
India	Mahbubnagar	Proportion	2017	65.8	78.2	48.0
India	Mahbubnagar	Count	2000	0	1	0
India	Mahbubnagar	Count	2017	1	1	0
India	Sipahijala	Proportion	2000	42.7	60.6	24.4
India	Sipahijala	Proportion	2017	64.4	79.6	46.2
India	Sipahijala	Count	2000	0	1	0
India	Sipahijala	Count	2017	1	1	0
India	Amethi	Proportion	2000	26.5	41.9	14.5
India	Amethi	Proportion	2017	48.3	65.3	31.5
India	Amethi	Count	2000	0	0	0
India	Amethi	Count	2017	0	1	0
India	Dehradun	Proportion	2000	44.5	62.2	27.6
India	Dehradun	Proportion	2017	65.4	78.9	47.6
India	Dehradun	Count	2000	0	1	0
India	Dehradun	Count	2017	1	1	0
India	Dakshin Dinajpur	Proportion	2000	40.5	58.2	23.9
India	Dakshin Dinajpur	Proportion	2017	63.6	76.5	47.3
India	Dakshin Dinajpur	Count	2000	0	1	0
India	Dakshin Dinajpur	Count	2017	1	1	0
India	Daman	Proportion	2000	48.0	67.0	26.4
India	Daman	Proportion	2017	68.0	81.5	50.2
India	Daman	Count	2000	0	1	0
India	Daman	Count	2017	1	1	1
India	Kurnool	Proportion	2000	40.7	56.2	25.3
India	Kurnool	Proportion	2017	63.1	76.8	47.2
India	Kurnool	Count	2000	0	1	0
India	Kurnool	Count	2017	1	1	0
India	Kurung Kumey	Proportion	2000	43.7	60.4	29.4
India	Kurung Kumey	Proportion	2017	67.0	81.5	49.9
India	Kurung Kumey	Count	2000	0	1	0
India	Kurung Kumey	Count	2017	1	1	0
India	Darrang	Proportion	2000	38.7	53.7	22.7
India	Darrang	Proportion	2017	59.5	74.4	43.3
India	Darrang	Count	2000	0	1	0
India	Darrang	Count	2017	1	1	0
India	Bhagalpur	Proportion	2000	26.1	44.3	12.7
India	Bhagalpur	Proportion	2017	50.0	66.4	32.7
India	Bhagalpur	Count	2000	0	0	0
India	Bhagalpur	Count	2017	0	1	0
India	Bijapur	Proportion	2000	40.5	58.2	25.9
India	Bijapur	Proportion	2017	60.4	73.4	44.4
India	Bijapur	Count	2000	0	1	0
India	Bijapur	Count	2017	1	1	0

Table 4: ORS by Unit *(continued)*

Country	Unit	Metric	Year	Mean	Upper	Lower
India	Bharuch	Proportion	2000	40.2	57.0	24.7
India	Bharuch	Proportion	2017	60.9	75.4	42.5
India	Bharuch	Count	2000	0	1	0
India	Bharuch	Count	2017	1	1	0
India	Hisar	Proportion	2000	31.3	49.6	18.6
India	Hisar	Proportion	2017	54.1	69.9	37.8
India	Hisar	Count	2000	0	0	0
India	Hisar	Count	2017	1	1	0
India	Kullu	Proportion	2000	47.1	64.4	30.5
India	Kullu	Proportion	2017	69.0	81.9	50.3
India	Kullu	Count	2000	0	1	0
India	Kullu	Count	2017	1	1	1
India	Ganderbal	Proportion	2000	43.3	60.3	26.9
India	Ganderbal	Proportion	2017	64.6	78.4	47.4
India	Ganderbal	Count	2000	0	1	0
India	Ganderbal	Count	2017	1	1	0
India	Garhwa	Proportion	2000	36.8	54.0	23.7
India	Garhwa	Proportion	2017	57.5	74.2	41.1
India	Garhwa	Count	2000	0	1	0
India	Garhwa	Count	2017	1	1	0
India	Bidar	Proportion	2000	45.6	63.0	30.0
India	Bidar	Proportion	2017	65.2	77.6	50.8
India	Bidar	Count	2000	0	1	0
India	Bidar	Count	2017	1	1	1
India	Kollam	Proportion	2000	48.2	68.9	30.9
India	Kollam	Proportion	2017	70.8	83.6	51.9
India	Kollam	Count	2000	0	1	0
India	Kollam	Count	2017	1	1	1
India	Barwani	Proportion	2000	39.5	58.1	23.6
India	Barwani	Proportion	2017	56.8	72.9	39.6
India	Barwani	Count	2000	0	1	0
India	Barwani	Count	2017	1	1	0
India	Bid	Proportion	2000	43.9	59.7	28.7
India	Bid	Proportion	2017	63.8	77.5	49.5
India	Bid	Count	2000	0	1	0
India	Bid	Count	2017	1	1	0
India	Senapati	Proportion	2000	45.8	63.8	29.4
India	Senapati	Proportion	2017	65.6	81.0	46.9
India	Senapati	Count	2000	0	1	0
India	Senapati	Count	2017	1	1	0
India	South Garo Hills	Proportion	2000	38.0	53.3	22.9
India	South Garo Hills	Proportion	2017	63.8	79.2	45.9
India	South Garo Hills	Count	2000	0	1	0
India	South Garo Hills	Count	2017	1	1	0
India	Mamit	Proportion	2000	40.5	57.1	25.6
India	Mamit	Proportion	2017	63.6	77.9	49.7
India	Mamit	Count	2000	0	1	0
India	Mamit	Count	2017	1	1	0
India	Mon	Proportion	2000	47.3	65.7	28.2

Table 4: ORS by Unit *(continued)*

Country	Unit	Metric	Year	Mean	Upper	Lower
India	Mon	Proportion	2017	65.9	82.1	47.5
India	Mon	Count	2000	0	1	0
India	Mon	Count	2017	1	1	0
India	North East	Proportion	2000	43.7	64.4	25.1
India	North East	Proportion	2017	62.0	79.0	43.5
India	North East	Count	2000	0	1	0
India	North East	Count	2017	1	1	0
India	Bhadrak	Proportion	2000	44.8	63.9	27.4
India	Bhadrak	Proportion	2017	63.1	77.7	46.0
India	Bhadrak	Count	2000	0	1	0
India	Bhadrak	Count	2017	1	1	0
India	Fazilka	Proportion	2000	32.2	48.8	16.2
India	Fazilka	Proportion	2017	55.8	70.7	39.9
India	Fazilka	Count	2000	0	0	0
India	Fazilka	Count	2017	1	1	0
India	Bharatpur	Proportion	2000	28.6	44.2	16.1
India	Bharatpur	Proportion	2017	48.8	65.5	32.5
India	Bharatpur	Count	2000	0	0	0
India	Bharatpur	Count	2017	0	1	0
India	Dindigul	Proportion	2000	44.9	66.0	28.7
India	Dindigul	Proportion	2017	65.3	79.2	49.8
India	Dindigul	Count	2000	0	1	0
India	Dindigul	Count	2017	1	1	0
India	Medak	Proportion	2000	42.7	59.6	25.6
India	Medak	Proportion	2017	65.1	79.3	50.2
India	Medak	Count	2000	0	1	0
India	Medak	Count	2017	1	1	1
India	South Tripura	Proportion	2000	41.7	60.2	23.3
India	South Tripura	Proportion	2017	64.0	79.9	47.3
India	South Tripura	Count	2000	0	1	0
India	South Tripura	Count	2017	1	1	0
India	Amroha	Proportion	2000	25.0	43.7	13.3
India	Amroha	Proportion	2017	45.8	63.3	29.0
India	Amroha	Count	2000	0	0	0
India	Amroha	Count	2017	0	1	0
India	Pauri Garhwal	Proportion	2000	43.9	60.9	27.7
India	Pauri Garhwal	Proportion	2017	64.7	78.5	47.8
India	Pauri Garhwal	Count	2000	0	1	0
India	Pauri Garhwal	Count	2017	1	1	0
India	Darjiling	Proportion	2000	47.6	64.3	30.2
India	Darjiling	Proportion	2017	69.5	83.9	53.9
India	Darjiling	Count	2000	0	1	0
India	Darjiling	Count	2017	1	1	1
India	Diu	Proportion	2000	42.2	61.0	24.8
India	Diu	Proportion	2017	62.8	79.0	44.7
India	Diu	Count	2000	0	1	0
India	Diu	Count	2017	1	1	0
India	Nellore	Proportion	2000	40.5	58.4	22.0
India	Nellore	Proportion	2017	62.7	78.2	46.8

Table 4: ORS by Unit *(continued)*

Country	Unit	Metric	Year	Mean	Upper	Lower
India	Nellore	Count	2000	0	1	0
India	Nellore	Count	2017	1	1	0
India	Lohit	Proportion	2000	41.9	60.0	26.5
India	Lohit	Proportion	2017	65.4	80.8	48.7
India	Lohit	Count	2000	0	1	0
India	Lohit	Count	2017	1	1	0
India	Dhemaji	Proportion	2000	42.8	59.1	26.9
India	Dhemaji	Proportion	2017	63.0	77.1	45.1
India	Dhemaji	Count	2000	0	1	0
India	Dhemaji	Count	2017	1	1	0
India	Bhojpur	Proportion	2000	24.6	41.6	12.1
India	Bhojpur	Proportion	2017	46.9	63.4	29.7
India	Bhojpur	Count	2000	0	0	0
India	Bhojpur	Count	2017	0	1	0
India	Bilaspur	Proportion	2000	37.9	58.9	23.3
India	Bilaspur	Proportion	2017	61.7	75.3	45.6
India	Bilaspur	Count	2000	0	1	0
India	Bilaspur	Count	2017	1	1	0
India	Bhavnagar	Proportion	2000	39.4	57.0	21.6
India	Bhavnagar	Proportion	2017	58.7	73.4	44.6
India	Bhavnagar	Count	2000	0	1	0
India	Bhavnagar	Count	2017	1	1	0
India	Jhajjar	Proportion	2000	31.5	50.4	17.2
India	Jhajjar	Proportion	2017	54.3	71.3	38.8
India	Jhajjar	Count	2000	0	1	0
India	Jhajjar	Count	2017	1	1	0
India	Lahul & Spiti	Proportion	2000	47.0	61.7	32.5
India	Lahul & Spiti	Proportion	2017	68.4	79.7	53.6
India	Lahul & Spiti	Count	2000	0	1	0
India	Lahul & Spiti	Count	2017	1	1	1
India	Jammu	Proportion	2000	41.2	59.1	25.3
India	Jammu	Proportion	2017	63.1	78.2	47.0
India	Jammu	Count	2000	0	1	0
India	Jammu	Count	2017	1	1	0
India	Giridih	Proportion	2000	38.9	58.3	23.5
India	Giridih	Proportion	2017	58.5	73.3	42.2
India	Giridih	Count	2000	0	1	0
India	Giridih	Count	2017	1	1	0
India	Vijaypura	Proportion	2000	42.2	59.4	26.4
India	Vijaypura	Proportion	2017	65.0	78.4	50.7
India	Vijaypura	Count	2000	0	1	0
India	Vijaypura	Count	2017	1	1	1
India	Kottayam	Proportion	2000	48.4	69.6	29.4
India	Kottayam	Proportion	2017	70.5	84.1	55.3
India	Kottayam	Count	2000	0	1	0
India	Kottayam	Count	2017	1	1	1
India	Betul	Proportion	2000	40.7	58.1	24.2
India	Betul	Proportion	2017	60.2	76.1	43.7
India	Betul	Count	2000	0	1	0

Table 4: ORS by Unit *(continued)*

Country	Unit	Metric	Year	Mean	Upper	Lower
India	Betul	Count	2017	1	1	0
India	Buldana	Proportion	2000	43.4	60.0	28.3
India	Buldana	Proportion	2017	63.1	77.5	47.1
India	Buldana	Count	2000	0	1	0
India	Buldana	Count	2017	1	1	0
India	Tamenglong	Proportion	2000	45.0	63.4	27.5
India	Tamenglong	Proportion	2017	65.4	79.8	45.8
India	Tamenglong	Count	2000	0	1	0
India	Tamenglong	Count	2017	1	1	0
India	South West Garo Hills	Proportion	2000	36.5	53.0	22.5
India	South West Garo Hills	Proportion	2017	59.6	75.5	42.1
India	South West Garo Hills	Count	2000	0	1	0
India	South West Garo Hills	Count	2017	1	1	0
India	Saiha	Proportion	2000	43.6	61.4	27.9
India	Saiha	Proportion	2017	66.5	81.8	49.5
India	Saiha	Count	2000	0	1	0
India	Saiha	Count	2017	1	1	0
India	Peren	Proportion	2000	43.9	64.3	25.9
India	Peren	Proportion	2017	64.4	79.1	45.9
India	Peren	Count	2000	0	1	0
India	Peren	Count	2017	1	1	0
India	North West	Proportion	2000	43.7	62.5	26.3
India	North West	Proportion	2017	63.6	79.2	46.7
India	North West	Count	2000	0	1	0
India	North West	Count	2017	1	1	0
India	Cuttack	Proportion	2000	44.3	60.7	27.3
India	Cuttack	Proportion	2017	63.2	78.0	45.9
India	Cuttack	Count	2000	0	1	0
India	Cuttack	Count	2017	1	1	0
India	Firozpur	Proportion	2000	32.9	48.7	18.4
India	Firozpur	Proportion	2017	56.0	74.1	40.3
India	Firozpur	Count	2000	0	0	0
India	Firozpur	Count	2017	1	1	0
India	Bhilwara	Proportion	2000	34.3	50.5	20.2
India	Bhilwara	Proportion	2017	56.9	71.7	39.7
India	Bhilwara	Count	2000	0	1	0
India	Bhilwara	Count	2017	1	1	0
India	Erode	Proportion	2000	43.4	62.7	27.3
India	Erode	Proportion	2017	65.3	78.9	47.4
India	Erode	Count	2000	0	1	0
India	Erode	Count	2017	1	1	0
India	Nalgonda	Proportion	2000	42.2	59.7	26.4
India	Nalgonda	Proportion	2017	63.6	78.8	47.3
India	Nalgonda	Count	2000	0	1	0
India	Nalgonda	Count	2017	1	1	0
India	Unokoti	Proportion	2000	44.6	64.9	27.5
India	Unokoti	Proportion	2017	65.7	82.5	47.0
India	Unokoti	Count	2000	0	1	0
India	Unokoti	Count	2017	1	1	0

Table 4: ORS by Unit *(continued)*

Country	Unit	Metric	Year	Mean	Upper	Lower
India	Auraiya	Proportion	2000	26.3	41.6	14.4
India	Auraiya	Proportion	2017	47.1	67.2	30.7
India	Auraiya	Count	2000	0	0	0
India	Auraiya	Count	2017	0	1	0
India	Hardwar	Proportion	2000	36.9	55.2	21.3
India	Hardwar	Proportion	2017	57.9	74.0	39.2
India	Hardwar	Count	2000	0	1	0
India	Hardwar	Count	2017	1	1	0
India	Haora	Proportion	2000	47.0	66.2	29.7
India	Haora	Proportion	2017	65.2	80.4	48.1
India	Haora	Count	2000	0	1	0
India	Haora	Count	2017	1	1	0
India	Lakshadweep	Proportion	2000	38.8	53.0	25.8
India	Lakshadweep	Proportion	2017	NA	NA	NA
India	Lakshadweep	Count	2000	0	1	0
India	Lakshadweep	Count	2017	NA	NA	NA
India	Prakasam	Proportion	2000	39.7	58.0	24.4
India	Prakasam	Proportion	2017	61.5	75.8	47.8
India	Prakasam	Count	2000	0	1	0
India	Prakasam	Count	2017	1	1	0
India	Longding	Proportion	2000	43.6	60.3	27.0
India	Longding	Proportion	2017	63.0	78.6	46.1
India	Longding	Count	2000	0	1	0
India	Longding	Count	2017	1	1	0
India	Dhubri	Proportion	2000	43.0	61.3	25.4
India	Dhubri	Proportion	2017	62.8	77.9	47.3
India	Dhubri	Count	2000	0	1	0
India	Dhubri	Count	2017	1	1	0
India	Buxar	Proportion	2000	23.5	40.0	10.8
India	Buxar	Proportion	2017	46.3	64.5	28.7
India	Buxar	Count	2000	0	0	0
India	Buxar	Count	2017	0	1	0
India	Dantewada	Proportion	2000	41.5	60.7	25.7
India	Dantewada	Proportion	2017	62.0	77.4	42.0
India	Dantewada	Count	2000	0	1	0
India	Dantewada	Count	2017	1	1	0
India	Botad	Proportion	2000	39.3	57.5	21.1
India	Botad	Proportion	2017	59.0	73.7	42.3
India	Botad	Count	2000	0	1	0
India	Botad	Count	2017	1	1	0
India	Jind	Proportion	2000	31.6	48.5	19.3
India	Jind	Proportion	2017	54.5	69.3	37.9
India	Jind	Count	2000	0	0	0
India	Jind	Count	2017	1	1	0
India	Mandi	Proportion	2000	45.2	63.5	26.9
India	Mandi	Proportion	2017	67.6	81.0	49.6
India	Mandi	Count	2000	0	1	0
India	Mandi	Count	2017	1	1	0
India	Kargil	Proportion	2000	43.6	60.0	27.7

Table 4: ORS by Unit *(continued)*

Country	Unit	Metric	Year	Mean	Upper	Lower
India	Kargil	Proportion	2017	63.5	76.6	48.3
India	Kargil	Count	2000	0	1	0
India	Kargil	Count	2017	1	1	0
India	Godda	Proportion	2000	35.8	55.5	18.7
India	Godda	Proportion	2017	56.7	72.1	40.0
India	Godda	Count	2000	0	1	0
India	Godda	Count	2017	1	1	0
India	Chamrajnagar	Proportion	2000	42.9	60.2	25.5
India	Chamrajnagar	Proportion	2017	64.7	78.7	46.2
India	Chamrajnagar	Count	2000	0	1	0
India	Chamrajnagar	Count	2017	1	1	0
India	Kozhikode	Proportion	2000	48.0	67.1	29.3
India	Kozhikode	Proportion	2017	71.0	82.8	55.7
India	Kozhikode	Count	2000	0	1	0
India	Kozhikode	Count	2017	1	1	1
India	Bhind	Proportion	2000	31.4	48.0	18.9
India	Bhind	Proportion	2017	54.0	71.2	34.8
India	Bhind	Count	2000	0	0	0
India	Bhind	Count	2017	1	1	0
India	Chandrapur	Proportion	2000	43.4	59.7	28.1
India	Chandrapur	Proportion	2017	64.7	78.3	50.1
India	Chandrapur	Count	2000	0	1	0
India	Chandrapur	Count	2017	1	1	1
India	Thoubal	Proportion	2000	43.3	60.9	26.9
India	Thoubal	Proportion	2017	63.3	80.2	46.9
India	Thoubal	Count	2000	0	1	0
India	Thoubal	Count	2017	1	1	0
India	South West Khasi Hills	Proportion	2000	49.9	66.8	34.9
India	South West Khasi Hills	Proportion	2017	73.4	84.4	58.8
India	South West Khasi Hills	Count	2000	0	1	0
India	South West Khasi Hills	Count	2017	1	1	1
India	Serchhip	Proportion	2000	43.3	60.1	27.2
India	Serchhip	Proportion	2017	65.7	80.1	50.7
India	Serchhip	Count	2000	0	1	0
India	Serchhip	Count	2017	1	1	1
India	Phek	Proportion	2000	46.4	63.9	28.8
India	Phek	Proportion	2017	65.3	81.1	47.2
India	Phek	Count	2000	0	1	0
India	Phek	Count	2017	1	1	0
India	South	Proportion	2000	45.2	63.3	26.8
India	South	Proportion	2017	65.0	81.1	48.7
India	South	Count	2000	0	1	0
India	South	Count	2017	1	1	0
India	Deogarh	Proportion	2000	42.0	58.6	24.8
India	Deogarh	Proportion	2017	63.9	79.6	46.5
India	Deogarh	Count	2000	0	1	0
India	Deogarh	Count	2017	1	1	0
India	Gurdaspur	Proportion	2000	36.9	54.6	21.4
India	Gurdaspur	Proportion	2017	58.3	76.9	39.0

Table 4: ORS by Unit *(continued)*

Country	Unit	Metric	Year	Mean	Upper	Lower
India	Gurdaspur	Count	2000	0	1	0
India	Gurdaspur	Count	2017	1	1	0
India	Bikaner	Proportion	2000	33.6	51.8	19.4
India	Bikaner	Proportion	2017	57.3	72.2	43.6
India	Bikaner	Count	2000	0	1	0
India	Bikaner	Count	2017	1	1	0
India	Kancheepuram	Proportion	2000	45.6	62.7	29.7
India	Kancheepuram	Proportion	2017	66.3	80.8	47.8
India	Kancheepuram	Count	2000	0	1	0
India	Kancheepuram	Count	2017	1	1	0
India	Nizamabad	Proportion	2000	44.3	61.9	26.6
India	Nizamabad	Proportion	2017	64.3	78.9	48.5
India	Nizamabad	Count	2000	0	1	0
India	Nizamabad	Count	2017	1	1	0
India	West Tripura	Proportion	2000	43.7	63.0	24.2
India	West Tripura	Proportion	2017	65.9	82.1	46.8
India	West Tripura	Count	2000	0	1	0
India	West Tripura	Count	2017	1	1	0
India	Azamgarh	Proportion	2000	24.6	39.9	13.9
India	Azamgarh	Proportion	2017	45.6	62.3	29.9
India	Azamgarh	Count	2000	0	0	0
India	Azamgarh	Count	2017	0	1	0
India	Nainital	Proportion	2000	41.8	61.4	25.6
India	Nainital	Proportion	2017	62.1	75.0	47.8
India	Nainital	Count	2000	0	1	0
India	Nainital	Count	2017	1	1	0
India	Hugli	Proportion	2000	43.7	61.8	28.4
India	Hugli	Proportion	2017	62.1	78.0	46.4
India	Hugli	Count	2000	0	1	0
India	Hugli	Count	2017	1	1	0
India	Karaikal	Proportion	2000	42.1	60.6	26.2
India	Karaikal	Proportion	2017	63.0	78.5	45.2
India	Karaikal	Count	2000	0	1	0
India	Karaikal	Count	2017	1	1	0
India	Srikakulam	Proportion	2000	39.2	55.9	24.0
India	Srikakulam	Proportion	2017	61.3	75.5	43.5
India	Srikakulam	Count	2000	0	1	0
India	Srikakulam	Count	2017	1	1	0
India	Lower Dibang Valley	Proportion	2000	41.8	59.1	26.5
India	Lower Dibang Valley	Proportion	2017	65.3	80.2	49.3
India	Lower Dibang Valley	Count	2000	0	1	0
India	Lower Dibang Valley	Count	2017	1	1	0
India	Dibrugarh	Proportion	2000	43.7	59.5	27.5
India	Dibrugarh	Proportion	2017	63.6	77.9	47.2
India	Dibrugarh	Count	2000	0	1	0
India	Dibrugarh	Count	2017	1	1	0
India	Darbhanga	Proportion	2000	25.6	42.2	13.3
India	Darbhanga	Proportion	2017	48.0	64.8	28.3
India	Darbhanga	Count	2000	0	0	0

Table 4: ORS by Unit *(continued)*

Country	Unit	Metric	Year	Mean	Upper	Lower
India	Darbhanga	Count	2017	0	1	0
India	Dhamtari	Proportion	2000	38.2	58.0	20.9
India	Dhamtari	Proportion	2017	58.6	74.1	41.4
India	Dhamtari	Count	2000	0	1	0
India	Dhamtari	Count	2017	1	1	0
India	Chhotaudepur	Proportion	2000	39.3	58.8	21.9
India	Chhotaudepur	Proportion	2017	59.6	76.0	42.2
India	Chhotaudepur	Count	2000	0	1	0
India	Chhotaudepur	Count	2017	1	1	0
India	Kaithal	Proportion	2000	33.9	50.8	20.1
India	Kaithal	Proportion	2017	57.4	73.8	39.7
India	Kaithal	Count	2000	0	1	0
India	Kaithal	Count	2017	1	1	0
India	Shimla	Proportion	2000	46.6	62.8	29.5
India	Shimla	Proportion	2017	69.8	80.7	52.4
India	Shimla	Count	2000	0	1	0
India	Shimla	Count	2017	1	1	1
India	Kathua	Proportion	2000	41.6	59.2	24.9
India	Kathua	Proportion	2017	63.3	78.8	45.3
India	Kathua	Count	2000	0	1	0
India	Kathua	Count	2017	1	1	0
India	Gumla	Proportion	2000	40.7	57.7	26.5
India	Gumla	Proportion	2017	62.0	75.8	46.1
India	Gumla	Count	2000	0	1	0
India	Gumla	Count	2017	1	1	0
India	Chikballapura	Proportion	2000	45.4	64.1	28.5
India	Chikballapura	Proportion	2017	66.1	80.2	50.3
India	Chikballapura	Count	2000	0	1	0
India	Chikballapura	Count	2017	1	1	1
India	Malappuram	Proportion	2000	45.8	66.5	26.9
India	Malappuram	Proportion	2017	69.7	81.7	54.3
India	Malappuram	Count	2000	0	1	0
India	Malappuram	Count	2017	1	1	1
India	Bhopal	Proportion	2000	41.0	60.9	22.7
India	Bhopal	Proportion	2017	62.3	79.3	43.4
India	Bhopal	Count	2000	0	1	0
India	Bhopal	Count	2017	1	1	0
India	Dhule	Proportion	2000	43.2	60.2	29.2
India	Dhule	Proportion	2017	63.8	77.8	46.4
India	Dhule	Count	2000	0	1	0
India	Dhule	Count	2017	1	1	0
India	Ukhrul	Proportion	2000	45.5	63.2	29.5
India	Ukhrul	Proportion	2017	65.3	80.4	49.1
India	Ukhrul	Count	2000	0	1	0
India	Ukhrul	Count	2017	1	1	0
India	West Garo Hills	Proportion	2000	39.5	54.8	24.7
India	West Garo Hills	Proportion	2017	62.6	77.9	45.2
India	West Garo Hills	Count	2000	0	1	0
India	West Garo Hills	Count	2017	1	1	0

Table 4: ORS by Unit *(continued)*

Country	Unit	Metric	Year	Mean	Upper	Lower
India	Tuensang	Proportion	2000	48.5	69.2	30.9
India	Tuensang	Proportion	2017	66.8	82.1	49.2
India	Tuensang	Count	2000	0	1	0
India	Tuensang	Count	2017	1	1	0
India	South West	Proportion	2000	43.9	62.9	27.4
India	South West	Proportion	2017	64.0	79.6	47.0
India	South West	Count	2000	0	1	0
India	South West	Count	2017	1	1	0
India	Dhenkanal	Proportion	2000	44.1	61.2	28.1
India	Dhenkanal	Proportion	2017	63.8	78.3	44.1
India	Dhenkanal	Count	2000	0	1	0
India	Dhenkanal	Count	2017	1	1	0
India	Hoshiarpur	Proportion	2000	38.3	56.8	21.7
India	Hoshiarpur	Proportion	2017	60.3	77.1	42.6
India	Hoshiarpur	Count	2000	0	1	0
India	Hoshiarpur	Count	2017	1	1	0
India	Bundi	Proportion	2000	33.3	52.4	19.5
India	Bundi	Proportion	2017	54.5	71.9	36.6
India	Bundi	Count	2000	0	1	0
India	Bundi	Count	2017	1	1	0
India	Kanniyakumari	Proportion	2000	43.8	62.1	26.2
India	Kanniyakumari	Proportion	2017	64.5	80.5	46.3
India	Kanniyakumari	Count	2000	0	1	0
India	Kanniyakumari	Count	2017	1	1	0
India	Ranga Reddy	Proportion	2000	45.2	62.6	27.9
India	Ranga Reddy	Proportion	2017	67.7	80.2	49.3
India	Ranga Reddy	Count	2000	0	1	0
India	Ranga Reddy	Count	2017	1	1	0
India	Baghpat	Proportion	2000	26.4	41.7	14.7
India	Baghpat	Proportion	2017	47.2	65.0	29.1
India	Baghpat	Count	2000	0	0	0
India	Baghpat	Count	2017	0	1	0
India	Pithoragarh	Proportion	2000	43.2	58.7	26.7
India	Pithoragarh	Proportion	2017	63.9	77.4	50.0
India	Pithoragarh	Count	2000	0	1	0
India	Pithoragarh	Count	2017	1	1	0
India	Jalpaiguri	Proportion	2000	47.1	64.2	27.8
India	Jalpaiguri	Proportion	2017	68.7	82.8	51.6
India	Jalpaiguri	Count	2000	0	1	0
India	Jalpaiguri	Count	2017	1	1	1
India	Mahe	Proportion	2000	46.7	65.6	30.0
India	Mahe	Proportion	2017	68.9	83.6	51.0
India	Mahe	Count	2000	0	1	0
India	Mahe	Count	2017	1	1	1
India	Visakhapatnam	Proportion	2000	41.0	59.1	27.4
India	Visakhapatnam	Proportion	2017	64.6	79.2	47.9
India	Visakhapatnam	Count	2000	0	1	0
India	Visakhapatnam	Count	2017	1	1	0
India	Lower Subansiri	Proportion	2000	41.7	57.5	26.8

Table 4: ORS by Unit *(continued)*

Country	Unit	Metric	Year	Mean	Upper	Lower
India	Lower Subansiri	Proportion	2017	64.3	80.1	44.7
India	Lower Subansiri	Count	2000	0	1	0
India	Lower Subansiri	Count	2017	1	1	0
India	Dima Hasao	Proportion	2000	41.4	59.1	24.5
India	Dima Hasao	Proportion	2017	62.5	76.1	45.2
India	Dima Hasao	Count	2000	0	1	0
India	Dima Hasao	Count	2017	1	1	0
India	Gaya	Proportion	2000	26.6	40.8	12.7
India	Gaya	Proportion	2017	49.7	67.2	32.3
India	Gaya	Count	2000	0	0	0
India	Gaya	Count	2017	0	1	0
India	Durg	Proportion	2000	40.7	60.7	22.2
India	Durg	Proportion	2017	61.4	76.3	44.9
India	Durg	Count	2000	0	1	0
India	Durg	Count	2017	1	1	0
India	Dohad	Proportion	2000	39.3	58.2	23.8
India	Dohad	Proportion	2017	61.1	79.3	43.6
India	Dohad	Count	2000	0	1	0
India	Dohad	Count	2017	1	1	0
India	Karnal	Proportion	2000	33.9	51.4	20.0
India	Karnal	Proportion	2017	56.9	73.7	37.7
India	Karnal	Count	2000	0	1	0
India	Karnal	Count	2017	1	1	0
India	Sirmaur	Proportion	2000	44.9	63.1	27.7
India	Sirmaur	Proportion	2017	68.0	79.4	49.9
India	Sirmaur	Count	2000	0	1	0
India	Sirmaur	Count	2017	1	1	0
India	Kishtwar	Proportion	2000	44.7	60.5	30.2
India	Kishtwar	Proportion	2017	64.9	77.9	51.3
India	Kishtwar	Count	2000	0	1	0
India	Kishtwar	Count	2017	1	1	1
India	Hazaribagh	Proportion	2000	39.7	56.1	22.2
India	Hazaribagh	Proportion	2017	60.2	75.2	42.3
India	Hazaribagh	Count	2000	0	1	0
India	Hazaribagh	Count	2017	1	1	0
India	Chikmagalur	Proportion	2000	43.6	62.0	27.9
India	Chikmagalur	Proportion	2017	66.8	82.0	51.0
India	Chikmagalur	Count	2000	0	1	0
India	Chikmagalur	Count	2017	1	1	1
India	Palakkad	Proportion	2000	44.1	62.9	24.9
India	Palakkad	Proportion	2017	67.5	81.5	50.1
India	Palakkad	Count	2000	0	1	0
India	Palakkad	Count	2017	1	1	1
India	Burhanpur	Proportion	2000	40.8	58.6	25.0
India	Burhanpur	Proportion	2017	57.2	73.9	38.6
India	Burhanpur	Count	2000	0	1	0
India	Burhanpur	Count	2017	1	1	0
India	Gadchiroli	Proportion	2000	42.9	57.3	28.2
India	Gadchiroli	Proportion	2017	64.3	76.4	50.6

Table 4: ORS by Unit *(continued)*

Country	Unit	Metric	Year	Mean	Upper	Lower
India	Gadchiroli	Count	2000	0	1	0
India	Gadchiroli	Count	2017	1	1	1
India	Jiribam	Proportion	2000	43.5	61.5	25.6
India	Jiribam	Proportion	2017	63.4	78.1	44.6
India	Jiribam	Count	2000	0	1	0
India	Jiribam	Count	2017	1	1	0
India	West Khasi Hills	Proportion	2000	49.3	65.5	35.1
India	West Khasi Hills	Proportion	2017	72.9	83.8	58.3
India	West Khasi Hills	Count	2000	0	1	0
India	West Khasi Hills	Count	2017	1	1	1
India	Wokha	Proportion	2000	44.7	64.2	27.8
India	Wokha	Proportion	2017	63.9	78.9	44.6
India	Wokha	Count	2000	0	1	0
India	Wokha	Count	2017	1	1	0
India	Shahdara	Proportion	2000	43.5	64.1	25.0
India	Shahdara	Proportion	2017	62.1	78.8	43.4
India	Shahdara	Count	2000	0	1	0
India	Shahdara	Count	2017	1	1	0
India	Gajapati	Proportion	2000	42.4	59.7	27.2
India	Gajapati	Proportion	2017	63.3	79.1	47.0
India	Gajapati	Count	2000	0	1	0
India	Gajapati	Count	2017	1	1	0
India	Jalandhar	Proportion	2000	38.2	53.4	21.3
India	Jalandhar	Proportion	2017	60.1	76.9	43.0
India	Jalandhar	Count	2000	0	1	0
India	Jalandhar	Count	2017	1	1	0
India	Chittaurgarh	Proportion	2000	35.4	51.9	21.5
India	Chittaurgarh	Proportion	2017	58.3	71.6	44.0
India	Chittaurgarh	Count	2000	0	1	0
India	Chittaurgarh	Count	2017	1	1	0
India	Karur	Proportion	2000	43.6	63.3	26.3
India	Karur	Proportion	2017	64.2	78.7	47.1
India	Karur	Count	2000	0	1	0
India	Karur	Count	2017	1	1	0
India	Warangal Urban	Proportion	2000	44.5	62.5	28.6
India	Warangal Urban	Proportion	2017	65.5	80.2	47.4
India	Warangal Urban	Count	2000	0	1	0
India	Warangal Urban	Count	2017	1	1	0
India	Bahraich	Proportion	2000	23.7	38.9	12.8
India	Bahraich	Proportion	2017	45.0	62.2	28.7
India	Bahraich	Count	2000	0	0	0
India	Bahraich	Count	2017	0	1	0
India	Rudraprayag	Proportion	2000	44.5	61.9	28.6
India	Rudraprayag	Proportion	2017	65.4	80.3	46.5
India	Rudraprayag	Count	2000	0	1	0
India	Rudraprayag	Count	2017	1	1	0
India	Koch Bihar	Proportion	2000	45.6	61.2	28.4
India	Koch Bihar	Proportion	2017	66.6	79.8	51.0
India	Koch Bihar	Count	2000	0	1	0

Table 4: ORS by Unit *(continued)*

Country	Unit	Metric	Year	Mean	Upper	Lower
India	Koch Bihar	Count	2017	1	1	1
India	Puducherry	Proportion	2000	44.2	61.3	28.7
India	Puducherry	Proportion	2017	64.0	79.5	45.4
India	Puducherry	Count	2000	0	1	0
India	Puducherry	Count	2017	1	1	0
India	Vizianagaram	Proportion	2000	40.1	61.4	26.7
India	Vizianagaram	Proportion	2017	63.1	78.2	47.1
India	Vizianagaram	Count	2000	0	1	0
India	Vizianagaram	Count	2017	1	1	0
India	Namsai	Proportion	2000	40.7	59.1	25.2
India	Namsai	Proportion	2017	64.0	79.8	45.3
India	Namsai	Count	2000	0	1	0
India	Namsai	Count	2017	1	1	0
India	Goalpara	Proportion	2000	40.9	57.2	26.1
India	Goalpara	Proportion	2017	62.4	77.2	45.7
India	Goalpara	Count	2000	0	1	0
India	Goalpara	Count	2017	1	1	0
India	Gopalganj	Proportion	2000	23.0	35.9	12.6
India	Gopalganj	Proportion	2017	44.9	62.3	28.2
India	Gopalganj	Count	2000	0	0	0
India	Gopalganj	Count	2017	0	1	0
India	Gariaband	Proportion	2000	37.7	55.7	22.3
India	Gariaband	Proportion	2017	58.7	72.9	42.6
India	Gariaband	Count	2000	0	1	0
India	Gariaband	Count	2017	1	1	0
India	Devbhumi Dwarka	Proportion	2000	39.9	55.3	23.3
India	Devbhumi Dwarka	Proportion	2017	61.4	75.4	45.5
India	Devbhumi Dwarka	Count	2000	0	1	0
India	Devbhumi Dwarka	Count	2017	1	1	0
India	Kurukshetra	Proportion	2000	36.5	53.8	22.5
India	Kurukshetra	Proportion	2017	59.6	75.8	40.1
India	Kurukshetra	Count	2000	0	1	0
India	Kurukshetra	Count	2017	1	1	0
India	Solan	Proportion	2000	44.4	61.0	27.8
India	Solan	Proportion	2017	67.3	80.9	48.8
India	Solan	Count	2000	0	1	0
India	Solan	Count	2017	1	1	0
India	Kulgam	Proportion	2000	41.6	61.3	24.9
India	Kulgam	Proportion	2017	62.7	78.1	43.8
India	Kulgam	Count	2000	0	1	0
India	Kulgam	Count	2017	1	1	0
India	Jamtara	Proportion	2000	38.4	58.5	23.2
India	Jamtara	Proportion	2017	58.6	75.3	40.0
India	Jamtara	Count	2000	0	1	0
India	Jamtara	Count	2017	1	1	0
India	Chitradurga	Proportion	2000	41.6	60.0	25.6
India	Chitradurga	Proportion	2017	65.0	77.0	50.7
India	Chitradurga	Count	2000	0	1	0
India	Chitradurga	Count	2017	1	1	1

Table 4: ORS by Unit *(continued)*

Country	Unit	Metric	Year	Mean	Upper	Lower
India	Pathanamthitta	Proportion	2000	47.3	67.3	30.3
India	Pathanamthitta	Proportion	2017	69.5	82.6	52.5
India	Pathanamthitta	Count	2000	0	1	0
India	Pathanamthitta	Count	2017	1	1	1
India	Chhatarpur	Proportion	2000	33.5	50.7	20.2
India	Chhatarpur	Proportion	2017	55.4	69.9	39.9
India	Chhatarpur	Count	2000	0	1	0
India	Chhatarpur	Count	2017	1	1	0
India	Gondiya	Proportion	2000	42.2	58.2	24.1
India	Gondiya	Proportion	2017	64.4	77.4	50.7
India	Gondiya	Count	2000	0	1	0
India	Gondiya	Count	2017	1	1	1
India	Pherzawl	Proportion	2000	43.1	61.2	25.7
India	Pherzawl	Proportion	2017	62.6	76.1	46.5
India	Pherzawl	Count	2000	0	1	0
India	Pherzawl	Count	2017	1	1	0
India	West Jaintia Hills	Proportion	2000	43.4	59.6	27.5
India	West Jaintia Hills	Proportion	2017	67.5	81.8	51.0
India	West Jaintia Hills	Count	2000	0	1	0
India	West Jaintia Hills	Count	2017	1	1	1
India	Zunheboto	Proportion	2000	47.0	65.7	28.7
India	Zunheboto	Proportion	2017	65.8	81.3	46.1
India	Zunheboto	Count	2000	0	1	0
India	Zunheboto	Count	2017	1	1	0
India	South East	Proportion	2000	44.8	63.4	26.0
India	South East	Proportion	2017	63.8	79.8	47.1
India	South East	Count	2000	0	1	0
India	South East	Count	2017	1	1	0
India	Ganjam	Proportion	2000	40.7	57.5	26.4
India	Ganjam	Proportion	2017	62.2	78.0	43.2
India	Ganjam	Count	2000	0	1	0
India	Ganjam	Count	2017	1	1	0
India	Kapurthala	Proportion	2000	37.1	50.9	21.5
India	Kapurthala	Proportion	2017	59.6	77.3	41.9
India	Kapurthala	Count	2000	0	1	0
India	Kapurthala	Count	2017	1	1	0
India	Churu	Proportion	2000	33.4	48.5	21.6
India	Churu	Proportion	2017	56.2	70.7	40.7
India	Churu	Count	2000	0	0	0
India	Churu	Count	2017	1	1	0
India	Krishnagiri	Proportion	2000	45.0	62.2	26.9
India	Krishnagiri	Proportion	2017	67.2	79.2	49.8
India	Krishnagiri	Count	2000	0	1	0
India	Krishnagiri	Count	2017	1	1	0
India	Bhadradri Kothagudem	Proportion	2000	43.1	60.0	26.4
India	Bhadradri Kothagudem	Proportion	2017	63.2	78.1	46.3
India	Bhadradri Kothagudem	Count	2000	0	1	0
India	Bhadradri Kothagudem	Count	2017	1	1	0
India	Ballia	Proportion	2000	26.2	41.8	13.9

Table 4: ORS by Unit *(continued)*

Country	Unit	Metric	Year	Mean	Upper	Lower
India	Ballia	Proportion	2017	45.8	63.1	28.8
India	Ballia	Count	2000	0	0	0
India	Ballia	Count	2017	0	1	0
India	Tehri Garhwal	Proportion	2000	45.7	60.6	29.7
India	Tehri Garhwal	Proportion	2017	66.7	81.3	51.3
India	Tehri Garhwal	Count	2000	0	1	0
India	Tehri Garhwal	Count	2017	1	1	1
India	Kolkata	Proportion	2000	50.1	67.8	32.6
India	Kolkata	Proportion	2017	67.3	82.2	49.0
India	Kolkata	Count	2000	1	1	0
India	Kolkata	Count	2017	1	1	0
India	Yanam	Proportion	2000	40.6	60.9	23.2
India	Yanam	Proportion	2017	62.5	78.2	44.4
India	Yanam	Count	2000	0	1	0
India	Yanam	Count	2017	1	1	0
India	West Godavari	Proportion	2000	38.6	55.6	23.2
India	West Godavari	Proportion	2017	59.9	76.3	40.7
India	West Godavari	Count	2000	0	1	0
India	West Godavari	Count	2017	1	1	0
India	Papum Pare	Proportion	2000	41.8	59.0	27.4
India	Papum Pare	Proportion	2017	64.3	80.5	44.8
India	Papum Pare	Count	2000	0	1	0
India	Papum Pare	Count	2017	1	1	0
India	Golaghat	Proportion	2000	39.7	56.9	25.0
India	Golaghat	Proportion	2017	58.5	73.8	41.9
India	Golaghat	Count	2000	0	1	0
India	Golaghat	Count	2017	1	1	0
India	Jamui	Proportion	2000	27.4	43.5	14.1
India	Jamui	Proportion	2017	51.0	67.7	34.0
India	Jamui	Count	2000	0	0	0
India	Jamui	Count	2017	1	1	0
India	Janjgir-Champa	Proportion	2000	33.9	50.4	17.7
India	Janjgir-Champa	Proportion	2017	58.6	73.2	39.6
India	Janjgir-Champa	Count	2000	0	1	0
India	Janjgir-Champa	Count	2017	1	1	0
India	Gandhinagar	Proportion	2000	36.5	55.1	21.6
India	Gandhinagar	Proportion	2017	60.1	75.6	43.2
India	Gandhinagar	Count	2000	0	1	0
India	Gandhinagar	Count	2017	1	1	0
India	Mahendragarh	Proportion	2000	32.1	51.3	17.9
India	Mahendragarh	Proportion	2017	54.7	71.8	38.8
India	Mahendragarh	Count	2000	0	1	0
India	Mahendragarh	Count	2017	1	1	0
India	Una	Proportion	2000	43.1	64.0	25.6
India	Una	Proportion	2017	65.7	81.7	48.6
India	Una	Count	2000	0	1	0
India	Una	Count	2017	1	1	0
India	Kupwara	Proportion	2000	41.2	58.5	27.4
India	Kupwara	Proportion	2017	62.8	77.9	44.5

Table 4: ORS by Unit *(continued)*

Country	Unit	Metric	Year	Mean	Upper	Lower
India	Kupwara	Count	2000	0	1	0
India	Kupwara	Count	2017	1	1	0
India	Khunti	Proportion	2000	39.8	58.6	24.9
India	Khunti	Proportion	2017	60.8	76.1	43.9
India	Khunti	Count	2000	0	1	0
India	Khunti	Count	2017	1	1	0
India	Dakshina Kannada	Proportion	2000	46.3	65.0	28.5
India	Dakshina Kannada	Proportion	2017	69.5	83.7	53.5
India	Dakshina Kannada	Count	2000	0	1	0
India	Dakshina Kannada	Count	2017	1	1	1
India	Thiruvananthapuram	Proportion	2000	47.2	65.0	30.1
India	Thiruvananthapuram	Proportion	2017	69.3	82.3	51.0
India	Thiruvananthapuram	Count	2000	0	1	0
India	Thiruvananthapuram	Count	2017	1	1	1
India	Chhindwara	Proportion	2000	40.6	57.7	25.8
India	Chhindwara	Proportion	2017	61.7	78.2	46.2
India	Chhindwara	Count	2000	0	1	0
India	Chhindwara	Count	2017	1	1	0
India	Hingoli	Proportion	2000	43.6	60.3	26.8
India	Hingoli	Proportion	2017	63.9	79.3	46.9
India	Hingoli	Count	2000	0	1	0
India	Hingoli	Count	2017	1	1	0
India	Tengnoupal	Proportion	2000	44.0	61.5	28.3
India	Tengnoupal	Proportion	2017	63.5	79.2	46.4
India	Tengnoupal	Count	2000	0	1	0
India	Tengnoupal	Count	2017	1	1	0
India	Jagatsinghapur	Proportion	2000	43.8	61.0	24.4
India	Jagatsinghapur	Proportion	2017	62.2	78.4	44.3
India	Jagatsinghapur	Count	2000	0	1	0
India	Jagatsinghapur	Count	2017	1	1	0
India	Ludhiana	Proportion	2000	41.2	57.9	25.3
India	Ludhiana	Proportion	2017	63.0	75.7	48.5
India	Ludhiana	Count	2000	0	1	0
India	Ludhiana	Count	2017	1	1	0
India	Dausa	Proportion	2000	31.8	50.1	18.9
India	Dausa	Proportion	2017	51.3	67.4	33.1
India	Dausa	Count	2000	0	1	0
India	Dausa	Count	2017	1	1	0
India	Madurai	Proportion	2000	43.6	65.0	25.3
India	Madurai	Proportion	2017	64.0	79.9	45.9
India	Madurai	Count	2000	0	1	0
India	Madurai	Count	2017	1	1	0
India	Jagitial	Proportion	2000	43.8	61.9	25.9
India	Jagitial	Proportion	2017	63.2	78.8	45.1
India	Jagitial	Count	2000	0	1	0
India	Jagitial	Count	2017	1	1	0
India	Balrampur	Proportion	2000	25.8	41.8	14.5
India	Balrampur	Proportion	2017	46.5	61.8	30.6
India	Balrampur	Count	2000	0	0	0

Table 4: ORS by Unit *(continued)*

Country	Unit	Metric	Year	Mean	Upper	Lower
India	Balrampur	Count	2017	0	1	0
India	Udham Singh Nagar	Proportion	2000	36.0	53.6	21.8
India	Udham Singh Nagar	Proportion	2017	56.6	70.1	43.1
India	Udham Singh Nagar	Count	2000	0	1	0
India	Udham Singh Nagar	Count	2017	1	1	0
India	Maldah	Proportion	2000	40.5	58.2	25.9
India	Maldah	Proportion	2017	62.5	76.2	45.4
India	Maldah	Count	2000	0	1	0
India	Maldah	Count	2017	1	1	0
India	Y.S.R.	Proportion	2000	41.9	59.3	26.5
India	Y.S.R.	Proportion	2017	63.1	76.7	48.6
India	Y.S.R.	Count	2000	0	1	0
India	Y.S.R.	Count	2017	1	1	0
India	Tawang	Proportion	2000	41.7	60.1	23.7
India	Tawang	Proportion	2017	63.6	79.0	44.5
India	Tawang	Count	2000	0	1	0
India	Tawang	Count	2017	1	1	0
India	Hailakandi	Proportion	2000	42.5	61.5	25.0
India	Hailakandi	Proportion	2017	62.9	77.0	48.3
India	Hailakandi	Count	2000	0	1	0
India	Hailakandi	Count	2017	1	1	0
India	Jehanabad	Proportion	2000	25.8	43.3	11.8
India	Jehanabad	Proportion	2017	48.6	65.9	29.5
India	Jehanabad	Count	2000	0	0	0
India	Jehanabad	Count	2017	0	1	0
India	Jashpur	Proportion	2000	39.4	58.2	23.8
India	Jashpur	Proportion	2017	61.6	75.0	44.3
India	Jashpur	Count	2000	0	1	0
India	Jashpur	Count	2017	1	1	0
India	Gir Somnath	Proportion	2000	39.5	56.9	23.1
India	Gir Somnath	Proportion	2017	61.0	75.7	43.7
India	Gir Somnath	Count	2000	0	1	0
India	Gir Somnath	Count	2017	1	1	0
India	Mewat	Proportion	2000	29.2	45.2	15.7
India	Mewat	Proportion	2017	50.0	69.0	32.9
India	Mewat	Count	2000	0	0	0
India	Mewat	Count	2017	0	1	0
India	Leh (Ladakh)	Proportion	2000	45.1	56.8	32.3
India	Leh (Ladakh)	Proportion	2017	65.1	77.0	53.2
India	Leh (Ladakh)	Count	2000	0	1	0
India	Leh (Ladakh)	Count	2017	1	1	1
India	Koderma	Proportion	2000	38.6	58.3	21.1
India	Koderma	Proportion	2017	58.3	73.3	42.3
India	Koderma	Count	2000	0	1	0
India	Koderma	Count	2017	1	1	0
India	Davanagere	Proportion	2000	39.6	57.4	22.7
India	Davanagere	Proportion	2017	63.9	76.6	49.0
India	Davanagere	Count	2000	0	1	0
India	Davanagere	Count	2017	1	1	0

Table 4: ORS by Unit *(continued)*

Country	Unit	Metric	Year	Mean	Upper	Lower
India	Thrissur	Proportion	2000	45.6	66.3	25.9
India	Thrissur	Proportion	2017	69.0	82.9	52.3
India	Thrissur	Count	2000	0	1	0
India	Thrissur	Count	2017	1	1	1
India	Damoh	Proportion	2000	36.9	54.2	21.9
India	Damoh	Proportion	2017	59.2	74.6	42.5
India	Damoh	Count	2000	0	1	0
India	Damoh	Count	2017	1	1	0
India	Jalgaon	Proportion	2000	42.4	61.1	27.3
India	Jalgaon	Proportion	2017	61.8	77.0	43.7
India	Jalgaon	Count	2000	0	1	0
India	Jalgaon	Count	2017	1	1	0
India	Kakching	Proportion	2000	43.7	60.5	27.5
India	Kakching	Proportion	2017	63.3	80.5	46.0
India	Kakching	Count	2000	0	1	0
India	Kakching	Count	2017	1	1	0
India	Jajapur	Proportion	2000	45.7	64.5	27.4
India	Jajapur	Proportion	2017	64.3	77.8	46.6
India	Jajapur	Count	2000	0	1	0
India	Jajapur	Count	2017	1	1	0
India	Mansa	Proportion	2000	33.6	50.8	19.8
India	Mansa	Proportion	2017	56.1	72.4	41.8
India	Mansa	Count	2000	0	1	0
India	Mansa	Count	2017	1	1	0
India	Dhaulpur	Proportion	2000	29.4	44.5	15.9
India	Dhaulpur	Proportion	2017	50.7	68.9	31.3
India	Dhaulpur	Count	2000	0	0	0
India	Dhaulpur	Count	2017	1	1	0
India	Nagappattinam	Proportion	2000	42.1	57.4	26.2
India	Nagappattinam	Proportion	2017	62.9	78.6	45.5
India	Nagappattinam	Count	2000	0	1	0
India	Nagappattinam	Count	2017	1	1	0
India	Jangoan	Proportion	2000	44.1	63.1	25.2
India	Jangoan	Proportion	2017	65.3	80.4	48.3
India	Jangoan	Count	2000	0	1	0
India	Jangoan	Count	2017	1	1	0
India	Banda	Proportion	2000	26.5	39.5	14.9
India	Banda	Proportion	2017	47.3	63.0	28.2
India	Banda	Count	2000	0	0	0
India	Banda	Count	2017	0	1	0
India	Uttarkashi	Proportion	2000	46.0	60.3	29.9
India	Uttarkashi	Proportion	2017	66.6	79.6	49.0
India	Uttarkashi	Count	2000	0	1	0
India	Uttarkashi	Count	2017	1	1	0
India	Murshidabad	Proportion	2000	41.5	59.4	26.1
India	Murshidabad	Proportion	2017	63.2	79.7	48.4
India	Murshidabad	Count	2000	0	1	0
India	Murshidabad	Count	2017	1	1	0
India	Tirap	Proportion	2000	43.1	57.2	27.5

Table 4: ORS by Unit *(continued)*

Country	Unit	Metric	Year	Mean	Upper	Lower
India	Tirap	Proportion	2017	63.5	78.7	47.5
India	Tirap	Count	2000	0	1	0
India	Tirap	Count	2017	1	1	0
India	Jorhat	Proportion	2000	42.5	60.2	26.7
India	Jorhat	Proportion	2017	60.9	76.2	41.4
India	Jorhat	Count	2000	0	1	0
India	Jorhat	Count	2017	1	1	0
India	Kaimur	Proportion	2000	23.8	39.0	12.0
India	Kaimur	Proportion	2017	47.9	65.3	29.6
India	Kaimur	Count	2000	0	0	0
India	Kaimur	Count	2017	0	1	0
India	Kabeerdham	Proportion	2000	37.7	56.7	22.3
India	Kabeerdham	Proportion	2017	59.9	74.0	41.8
India	Kabeerdham	Count	2000	0	1	0
India	Kabeerdham	Count	2017	1	1	0
India	Jamnagar	Proportion	2000	40.6	56.2	23.7
India	Jamnagar	Proportion	2017	62.5	77.2	46.5
India	Jamnagar	Count	2000	0	1	0
India	Jamnagar	Count	2017	1	1	0
India	Palwal	Proportion	2000	28.4	44.4	15.7
India	Palwal	Proportion	2017	49.7	69.2	33.3
India	Palwal	Count	2000	0	0	0
India	Palwal	Count	2017	0	1	0
India	Poonch	Proportion	2000	41.1	57.3	25.7
India	Poonch	Proportion	2017	62.8	77.5	45.1
India	Poonch	Count	2000	0	1	0
India	Poonch	Count	2017	1	1	0
India	Latehar	Proportion	2000	39.4	57.3	23.9
India	Latehar	Proportion	2017	59.8	76.4	43.1
India	Latehar	Count	2000	0	1	0
India	Latehar	Count	2017	1	1	0
India	Dharwad	Proportion	2000	43.2	62.9	25.6
India	Dharwad	Proportion	2017	66.7	80.2	50.9
India	Dharwad	Count	2000	0	1	0
India	Dharwad	Count	2017	1	1	1
India	Wayanad	Proportion	2000	48.7	67.8	31.3
India	Wayanad	Proportion	2017	71.8	84.4	57.8
India	Wayanad	Count	2000	0	1	0
India	Wayanad	Count	2017	1	1	1
India	Datia	Proportion	2000	32.9	51.4	19.2
India	Datia	Proportion	2017	55.2	72.8	37.8
India	Datia	Count	2000	0	1	0
India	Datia	Count	2017	1	1	0
India	Jalna	Proportion	2000	44.1	58.8	28.4
India	Jalna	Proportion	2017	63.6	76.7	47.7
India	Jalna	Count	2000	0	1	0
India	Jalna	Count	2017	1	1	0
India	Kamjong	Proportion	2000	44.1	61.5	28.5
India	Kamjong	Proportion	2017	63.8	77.8	47.3

Table 4: ORS by Unit *(continued)*

Country	Unit	Metric	Year	Mean	Upper	Lower
India	Kamjong	Count	2000	0	1	0
India	Kamjong	Count	2017	1	1	0
India	Jharsuguda	Proportion	2000	43.3	61.6	26.2
India	Jharsuguda	Proportion	2017	67.9	82.8	51.3
India	Jharsuguda	Count	2000	0	1	0
India	Jharsuguda	Count	2017	1	1	1
India	Moga	Proportion	2000	34.4	49.7	18.6
India	Moga	Proportion	2017	56.5	73.0	41.1
India	Moga	Count	2000	0	0	0
India	Moga	Count	2017	1	1	0
India	Dungarpur	Proportion	2000	32.7	49.2	20.1
India	Dungarpur	Proportion	2017	56.3	72.8	39.0
India	Dungarpur	Count	2000	0	0	0
India	Dungarpur	Count	2017	1	1	0
India	Namakkal	Proportion	2000	46.5	64.3	28.1
India	Namakkal	Proportion	2017	67.6	80.8	49.4
India	Namakkal	Count	2000	0	1	0
India	Namakkal	Count	2017	1	1	0
India	Jayashankar Bhupalapal	Proportion	2000	42.8	59.5	27.2
India	Jayashankar Bhupalapal	Proportion	2017	63.4	76.4	44.9
India	Jayashankar Bhupalapal	Count	2000	0	1	0
India	Jayashankar Bhupalapal	Count	2017	1	1	0
India	Barabanki	Proportion	2000	25.2	42.2	12.8
India	Barabanki	Proportion	2017	47.4	64.9	28.7
India	Barabanki	Count	2000	0	0	0
India	Barabanki	Count	2017	0	1	0
India	Nadia	Proportion	2000	40.2	57.0	24.4
India	Nadia	Proportion	2017	60.7	77.5	44.7
India	Nadia	Count	2000	0	1	0
India	Nadia	Count	2017	1	1	0
India	Upper Siang	Proportion	2000	42.6	59.7	26.3
India	Upper Siang	Proportion	2017	66.6	80.3	50.8
India	Upper Siang	Count	2000	0	1	0
India	Upper Siang	Count	2017	1	1	1
India	Kamrup Metropolitan	Proportion	2000	40.1	57.7	24.6
India	Kamrup Metropolitan	Proportion	2017	61.5	75.8	43.7
India	Kamrup Metropolitan	Count	2000	0	1	0
India	Kamrup Metropolitan	Count	2017	1	1	0
India	Katihar	Proportion	2000	26.5	44.1	14.2
India	Katihar	Proportion	2017	50.0	66.0	32.0
India	Katihar	Count	2000	0	0	0
India	Katihar	Count	2017	0	1	0
India	Kondagaon	Proportion	2000	41.2	58.0	26.3
India	Kondagaon	Proportion	2017	61.3	75.4	43.6
India	Kondagaon	Count	2000	0	1	0
India	Kondagaon	Count	2017	1	1	0
India	Junagadh	Proportion	2000	39.4	56.2	22.4
India	Junagadh	Proportion	2017	60.6	76.7	41.6
India	Junagadh	Count	2000	0	1	0

Table 4: ORS by Unit *(continued)*

Country	Unit	Metric	Year	Mean	Upper	Lower
India	Junagadh	Count	2017	1	1	0
India	Panchkula	Proportion	2000	41.7	60.0	25.1
India	Panchkula	Proportion	2017	64.9	78.2	46.5
India	Panchkula	Count	2000	0	1	0
India	Panchkula	Count	2017	1	1	0
India	Pulwama	Proportion	2000	42.6	60.9	26.9
India	Pulwama	Proportion	2017	64.3	78.3	47.2
India	Pulwama	Count	2000	0	1	0
India	Pulwama	Count	2017	1	1	0
India	Lohardaga	Proportion	2000	40.6	60.7	24.6
India	Lohardaga	Proportion	2017	61.7	78.2	43.3
India	Lohardaga	Count	2000	0	1	0
India	Lohardaga	Count	2017	1	1	0
India	Gadag	Proportion	2000	40.6	58.6	25.0
India	Gadag	Proportion	2017	65.6	79.6	49.8
India	Gadag	Count	2000	0	1	0
India	Gadag	Count	2017	1	1	0
India	Dewas	Proportion	2000	38.1	55.8	22.4
India	Dewas	Proportion	2017	58.0	74.6	41.1
India	Dewas	Count	2000	0	1	0
India	Dewas	Count	2017	1	1	0
India	Kolhapur	Proportion	2000	44.2	62.1	28.3
India	Kolhapur	Proportion	2017	65.5	78.2	47.5
India	Kolhapur	Count	2000	0	1	0
India	Kolhapur	Count	2017	1	1	0
India	Kangpokpi	Proportion	2000	44.4	62.4	28.3
India	Kangpokpi	Proportion	2017	64.3	80.0	47.6
India	Kangpokpi	Count	2000	0	1	0
India	Kangpokpi	Count	2017	1	1	0
India	Kalahandi	Proportion	2000	40.9	57.9	25.6
India	Kalahandi	Proportion	2017	64.4	76.6	47.1
India	Kalahandi	Count	2000	0	1	0
India	Kalahandi	Count	2017	1	1	0
India	Muktsar	Proportion	2000	33.0	49.4	16.8
India	Muktsar	Proportion	2017	55.6	70.6	38.7
India	Muktsar	Count	2000	0	0	0
India	Muktsar	Count	2017	1	1	0
India	Ganganagar	Proportion	2000	27.8	40.8	17.3
India	Ganganagar	Proportion	2017	50.5	65.1	36.6
India	Ganganagar	Count	2000	0	0	0
India	Ganganagar	Count	2017	1	1	0
India	Perambalur	Proportion	2000	43.1	58.7	26.8
India	Perambalur	Proportion	2017	64.7	79.3	48.3
India	Perambalur	Count	2000	0	1	0
India	Perambalur	Count	2017	1	1	0
India	Jogulamba Gadwa	Proportion	2000	39.6	57.1	22.8
India	Jogulamba Gadwa	Proportion	2017	63.3	78.5	44.6
India	Jogulamba Gadwa	Count	2000	0	1	0
India	Jogulamba Gadwa	Count	2017	1	1	0

Table 4: ORS by Unit *(continued)*

Country	Unit	Metric	Year	Mean	Upper	Lower
India	Bareilly	Proportion	2000	24.6	40.4	14.1
India	Bareilly	Proportion	2017	47.0	62.6	30.8
India	Bareilly	Count	2000	0	0	0
India	Bareilly	Count	2017	0	1	0
India	North 24 Parganas	Proportion	2000	45.7	63.5	30.3
India	North 24 Parganas	Proportion	2017	64.6	78.0	47.5
India	North 24 Parganas	Count	2000	0	1	0
India	North 24 Parganas	Count	2017	1	1	0
India	Upper Subansiri	Proportion	2000	42.7	59.1	27.8
India	Upper Subansiri	Proportion	2017	65.4	78.5	45.9
India	Upper Subansiri	Count	2000	0	1	0
India	Upper Subansiri	Count	2017	1	1	0
India	Kamrup	Proportion	2000	39.2	56.8	23.5
India	Kamrup	Proportion	2017	59.8	75.2	44.0
India	Kamrup	Count	2000	0	1	0
India	Kamrup	Count	2017	1	1	0
India	Khagaria	Proportion	2000	25.0	40.2	12.6
India	Khagaria	Proportion	2017	48.1	66.5	29.2
India	Khagaria	Count	2000	0	0	0
India	Khagaria	Count	2017	0	1	0
India	Korba	Proportion	2000	37.5	54.7	21.7
India	Korba	Proportion	2017	62.3	75.0	46.2
India	Korba	Count	2000	0	1	0
India	Korba	Count	2017	1	1	0
India	Kachchh	Proportion	2000	38.2	51.3	22.7
India	Kachchh	Proportion	2017	60.9	73.2	46.1
India	Kachchh	Count	2000	0	1	0
India	Kachchh	Count	2017	1	1	0
India	Panipat	Proportion	2000	32.0	49.5	18.3
India	Panipat	Proportion	2017	55.2	71.5	38.1
India	Panipat	Count	2000	0	0	0
India	Panipat	Count	2017	1	1	0
India	Rajouri	Proportion	2000	40.9	58.4	25.7
India	Rajouri	Proportion	2017	62.4	77.9	44.2
India	Rajouri	Count	2000	0	1	0
India	Rajouri	Count	2017	1	1	0
India	Pakur	Proportion	2000	37.8	58.4	20.7
India	Pakur	Proportion	2017	58.1	75.8	40.1
India	Pakur	Count	2000	0	1	0
India	Pakur	Count	2017	1	1	0
India	Kalaburgi	Proportion	2000	43.7	59.2	28.7
India	Kalaburgi	Proportion	2017	66.0	78.8	52.1
India	Kalaburgi	Count	2000	0	1	0
India	Kalaburgi	Count	2017	1	1	1
India	Dhar	Proportion	2000	37.8	55.3	23.8
India	Dhar	Proportion	2017	56.3	71.5	38.4
India	Dhar	Count	2000	0	1	0
India	Dhar	Count	2017	1	1	0
India	Latur	Proportion	2000	43.4	60.2	27.8

Table 4: ORS by Unit *(continued)*

Country	Unit	Metric	Year	Mean	Upper	Lower
India	Latur	Proportion	2017	64.4	76.1	49.3
India	Latur	Count	2000	0	1	0
India	Latur	Count	2017	1	1	0
India	Noney	Proportion	2000	44.3	61.2	27.8
India	Noney	Proportion	2017	64.3	78.1	45.5
India	Noney	Count	2000	0	1	0
India	Noney	Count	2017	1	1	0
India	Kandhamal	Proportion	2000	43.1	57.2	28.1
India	Kandhamal	Proportion	2017	65.3	78.8	47.5
India	Kandhamal	Count	2000	0	1	0
India	Kandhamal	Count	2017	1	1	0
India	Pathankot	Proportion	2000	40.7	57.9	22.7
India	Pathankot	Proportion	2017	62.8	77.9	43.1
India	Pathankot	Count	2000	0	1	0
India	Pathankot	Count	2017	1	1	0
India	Hanumangarh	Proportion	2000	29.4	42.7	16.2
India	Hanumangarh	Proportion	2017	52.4	67.9	35.8
India	Hanumangarh	Count	2000	0	0	0
India	Hanumangarh	Count	2017	1	1	0
India	Pudukkottai	Proportion	2000	43.3	61.6	26.6
India	Pudukkottai	Proportion	2017	63.8	79.3	48.0
India	Pudukkottai	Count	2000	0	1	0
India	Pudukkottai	Count	2017	1	1	0
India	Kamareddy	Proportion	2000	43.8	61.2	26.9
India	Kamareddy	Proportion	2017	64.9	79.1	49.7
India	Kamareddy	Count	2000	0	1	0
India	Kamareddy	Count	2017	1	1	0
India	Basti	Proportion	2000	23.9	41.6	12.3
India	Basti	Proportion	2017	44.5	60.1	28.9
India	Basti	Count	2000	0	0	0
India	Basti	Count	2017	0	1	0
India	Pashchim Medinipur	Proportion	2000	42.7	60.4	29.3
India	Pashchim Medinipur	Proportion	2017	62.8	78.2	47.5
India	Pashchim Medinipur	Count	2000	0	1	0
India	Pashchim Medinipur	Count	2017	1	1	0
India	West Kameng	Proportion	2000	41.0	57.8	24.6
India	West Kameng	Proportion	2017	62.9	79.1	44.4
India	West Kameng	Count	2000	0	1	0
India	West Kameng	Count	2017	1	1	0
India	Karbi Anglong	Proportion	2000	38.2	55.8	23.5
India	Karbi Anglong	Proportion	2017	58.6	72.3	40.8
India	Karbi Anglong	Count	2000	0	1	0
India	Karbi Anglong	Count	2017	1	1	0
India	Kishanganj	Proportion	2000	28.4	45.0	14.2
India	Kishanganj	Proportion	2017	52.6	70.5	35.7
India	Kishanganj	Count	2000	0	0	0
India	Kishanganj	Count	2017	1	1	0
India	Koriya	Proportion	2000	39.4	57.9	24.8
India	Koriya	Proportion	2017	61.4	75.9	44.2

Table 4: ORS by Unit *(continued)*

Country	Unit	Metric	Year	Mean	Upper	Lower
India	Koriya	Count	2000	0	1	0
India	Koriya	Count	2017	1	1	0
India	Kheda	Proportion	2000	36.7	56.6	23.0
India	Kheda	Proportion	2017	59.1	74.0	42.1
India	Kheda	Count	2000	0	1	0
India	Kheda	Count	2017	1	1	0
India	Rewari	Proportion	2000	32.1	51.0	16.9
India	Rewari	Proportion	2017	54.7	72.4	39.8
India	Rewari	Count	2000	0	1	0
India	Rewari	Count	2017	1	1	0
India	Ramban	Proportion	2000	43.2	61.5	27.3
India	Ramban	Proportion	2017	64.6	79.2	46.8
India	Ramban	Count	2000	0	1	0
India	Ramban	Count	2017	1	1	0
India	Palamu	Proportion	2000	37.3	53.4	21.5
India	Palamu	Proportion	2017	57.3	72.6	41.8
India	Palamu	Count	2000	0	1	0
India	Palamu	Count	2017	1	1	0
India	Hassan	Proportion	2000	42.8	62.4	25.7
India	Hassan	Proportion	2017	66.3	80.6	50.1
India	Hassan	Count	2000	0	1	0
India	Hassan	Count	2017	1	1	1
India	Dindori	Proportion	2000	39.2	56.0	23.0
India	Dindori	Proportion	2017	61.0	74.9	45.5
India	Dindori	Count	2000	0	1	0
India	Dindori	Count	2017	1	1	0
India	Mumbai City	Proportion	2000	51.2	71.0	31.6
India	Mumbai City	Proportion	2017	70.6	83.9	54.8
India	Mumbai City	Count	2000	1	1	0
India	Mumbai City	Count	2017	1	1	1
India	Kendrapara	Proportion	2000	45.0	63.9	27.5
India	Kendrapara	Proportion	2017	62.9	77.7	43.3
India	Kendrapara	Count	2000	0	1	0
India	Kendrapara	Count	2017	1	1	0
India	Patiala	Proportion	2000	38.6	53.7	24.5
India	Patiala	Proportion	2017	61.6	75.5	44.9
India	Patiala	Count	2000	0	1	0
India	Patiala	Count	2017	1	1	0
India	Jaipur	Proportion	2000	34.7	52.0	20.5
India	Jaipur	Proportion	2017	55.4	71.7	39.5
India	Jaipur	Count	2000	0	1	0
India	Jaipur	Count	2017	1	1	0
India	Ramanathapuram	Proportion	2000	43.0	60.9	27.6
India	Ramanathapuram	Proportion	2017	62.1	76.7	46.3
India	Ramanathapuram	Count	2000	0	1	0
India	Ramanathapuram	Count	2017	1	1	0
India	Kumuram Bheem Asifabad	Proportion	2000	45.4	63.2	28.5
India	Kumuram Bheem Asifabad	Proportion	2017	64.7	79.0	49.2
India	Kumuram Bheem Asifabad	Count	2000	0	1	0

Table 4: ORS by Unit *(continued)*

Country	Unit	Metric	Year	Mean	Upper	Lower
India	Kumuram Bheem Asifabad	Count	2017	1	1	0
India	Bijnor	Proportion	2000	27.0	40.2	14.6
India	Bijnor	Proportion	2017	48.1	64.2	32.9
India	Bijnor	Count	2000	0	0	0
India	Bijnor	Count	2017	0	1	0
India	Purba Medinipur	Proportion	2000	46.7	62.7	31.5
India	Purba Medinipur	Proportion	2017	65.8	81.0	49.0
India	Purba Medinipur	Count	2000	0	1	0
India	Purba Medinipur	Count	2017	1	1	0
India	West Siang	Proportion	2000	40.1	57.5	24.7
India	West Siang	Proportion	2017	63.5	78.7	44.4
India	West Siang	Count	2000	0	1	0
India	West Siang	Count	2017	1	1	0
India	Karimganj	Proportion	2000	38.4	55.5	23.1
India	Karimganj	Proportion	2017	61.6	76.5	44.4
India	Karimganj	Count	2000	0	1	0
India	Karimganj	Count	2017	1	1	0
India	Lakhisarai	Proportion	2000	26.4	43.5	13.6
India	Lakhisarai	Proportion	2017	49.5	67.2	32.1
India	Lakhisarai	Count	2000	0	0	0
India	Lakhisarai	Count	2017	0	1	0
India	Mahasamund	Proportion	2000	37.5	55.0	20.6
India	Mahasamund	Proportion	2017	59.7	74.4	43.0
India	Mahasamund	Count	2000	0	1	0
India	Mahasamund	Count	2017	1	1	0
India	Mahesana	Proportion	2000	37.1	55.1	22.2
India	Mahesana	Proportion	2017	60.2	76.0	42.2
India	Mahesana	Count	2000	0	1	0
India	Mahesana	Count	2017	1	1	0
India	Rohtak	Proportion	2000	31.4	50.8	15.8
India	Rohtak	Proportion	2017	53.8	71.0	37.9
India	Rohtak	Count	2000	0	1	0
India	Rohtak	Count	2017	1	1	0
India	Reasi	Proportion	2000	42.0	60.0	26.1
India	Reasi	Proportion	2017	62.8	78.5	45.1
India	Reasi	Count	2000	0	1	0
India	Reasi	Count	2017	1	1	0
India	Pashchimi Singhbhum	Proportion	2000	38.5	54.3	23.8
India	Pashchimi Singhbhum	Proportion	2017	59.3	73.4	43.5
India	Pashchimi Singhbhum	Count	2000	0	1	0
India	Pashchimi Singhbhum	Count	2017	1	1	0
India	Haveri	Proportion	2000	41.6	60.5	24.4
India	Haveri	Proportion	2017	65.6	78.6	52.0
India	Haveri	Count	2000	0	1	0
India	Haveri	Count	2017	1	1	1
India	East Nimar	Proportion	2000	38.7	56.4	23.3
India	East Nimar	Proportion	2017	55.9	70.6	36.4
India	East Nimar	Count	2000	0	1	0
India	East Nimar	Count	2017	1	1	0

Table 4: ORS by Unit *(continued)*

Country	Unit	Metric	Year	Mean	Upper	Lower
India	Mumbai Suburban	Proportion	2000	52.3	71.4	32.4
India	Mumbai Suburban	Proportion	2017	71.6	85.1	55.7
India	Mumbai Suburban	Count	2000	1	1	0
India	Mumbai Suburban	Count	2017	1	1	1
India	Kendujhar	Proportion	2000	43.7	59.7	28.3
India	Kendujhar	Proportion	2017	64.5	77.9	49.2
India	Kendujhar	Count	2000	0	1	0
India	Kendujhar	Count	2017	1	1	0
India	Rupnagar	Proportion	2000	41.4	60.6	24.9
India	Rupnagar	Proportion	2017	63.8	78.0	48.5
India	Rupnagar	Count	2000	0	1	0
India	Rupnagar	Count	2017	1	1	0
India	Jaisalmer	Proportion	2000	32.0	46.5	21.7
India	Jaisalmer	Proportion	2017	52.5	64.7	38.1
India	Jaisalmer	Count	2000	0	0	0
India	Jaisalmer	Count	2017	1	1	0
India	Salem	Proportion	2000	44.5	62.2	25.2
India	Salem	Proportion	2017	66.0	79.8	49.7
India	Salem	Count	2000	0	1	0
India	Salem	Count	2017	1	1	0
India	Mahuababad	Proportion	2000	42.4	58.9	27.0
India	Mahuababad	Proportion	2017	62.9	79.0	44.5
India	Mahuababad	Count	2000	0	1	0
India	Mahuababad	Count	2017	1	1	0
India	Budaun	Proportion	2000	23.2	38.0	12.2
India	Budaun	Proportion	2017	44.3	62.7	29.0
India	Budaun	Count	2000	0	0	0
India	Budaun	Count	2017	0	1	0
India	Puruliya	Proportion	2000	45.0	61.2	29.9
India	Puruliya	Proportion	2017	65.9	81.1	48.9
India	Puruliya	Count	2000	0	1	0
India	Puruliya	Count	2017	1	1	0
India	Pakke Kessang	Proportion	2000	40.1	56.2	24.5
India	Pakke Kessang	Proportion	2017	62.7	78.0	44.3
India	Pakke Kessang	Count	2000	0	1	0
India	Pakke Kessang	Count	2017	1	1	0
India	Kokrajhar	Proportion	2000	44.1	60.7	26.6
India	Kokrajhar	Proportion	2017	63.6	78.2	49.2
India	Kokrajhar	Count	2000	0	1	0
India	Kokrajhar	Count	2017	1	1	0
India	Madhepura	Proportion	2000	24.3	40.4	11.7
India	Madhepura	Proportion	2017	47.1	65.4	29.3
India	Madhepura	Count	2000	0	0	0
India	Madhepura	Count	2017	0	1	0
India	Mungeli	Proportion	2000	36.5	56.4	20.3
India	Mungeli	Proportion	2017	59.3	75.3	44.6
India	Mungeli	Count	2000	0	1	0
India	Mungeli	Count	2017	1	1	0
India	Mahisagar	Proportion	2000	37.6	56.5	22.3

Table 4: ORS by Unit *(continued)*

Country	Unit	Metric	Year	Mean	Upper	Lower
India	Mahisagar	Proportion	2017	61.4	77.9	42.3
India	Mahisagar	Count	2000	0	1	0
India	Mahisagar	Count	2017	1	1	0
India	Sirsa	Proportion	2000	29.5	44.2	16.6
India	Sirsa	Proportion	2017	52.4	70.4	36.9
India	Sirsa	Count	2000	0	0	0
India	Sirsa	Count	2017	1	1	0
India	Samba	Proportion	2000	40.8	59.9	24.4
India	Samba	Proportion	2017	63.0	78.3	45.9
India	Samba	Count	2000	0	1	0
India	Samba	Count	2017	1	1	0
India	Purbi Singhbhum	Proportion	2000	40.4	56.0	24.7
India	Purbi Singhbhum	Proportion	2017	60.8	78.5	44.8
India	Purbi Singhbhum	Count	2000	0	1	0
India	Purbi Singhbhum	Count	2017	1	1	0
India	Kodagu	Proportion	2000	46.7	65.9	29.0
India	Kodagu	Proportion	2017	69.2	82.1	55.2
India	Kodagu	Count	2000	0	1	0
India	Kodagu	Count	2017	1	1	1
India	Guna	Proportion	2000	37.1	53.6	20.5
India	Guna	Proportion	2017	58.5	73.7	41.4
India	Guna	Count	2000	0	1	0
India	Guna	Count	2017	1	1	0
India	Nagpur	Proportion	2000	45.5	62.5	29.0
India	Nagpur	Proportion	2017	67.2	81.4	50.6
India	Nagpur	Count	2000	0	1	0
India	Nagpur	Count	2017	1	1	1
India	Khordha	Proportion	2000	44.0	60.8	27.6
India	Khordha	Proportion	2017	63.7	77.3	44.9
India	Khordha	Count	2000	0	1	0
India	Khordha	Count	2017	1	1	0
India	Sahibzada Ajit Singh Nagar	Proportion	2000	41.6	58.1	25.0
India	Sahibzada Ajit Singh Nagar	Proportion	2017	65.1	78.4	46.4
India	Sahibzada Ajit Singh Nagar	Count	2000	0	1	0
India	Sahibzada Ajit Singh Nagar	Count	2017	1	1	0
India	Jalor	Proportion	2000	31.3	46.6	18.6
India	Jalor	Proportion	2017	52.4	67.9	37.6
India	Jalor	Count	2000	0	0	0
India	Jalor	Count	2017	1	1	0
India	Sivaganga	Proportion	2000	43.0	61.5	26.4
India	Sivaganga	Proportion	2017	63.5	78.0	47.1
India	Sivaganga	Count	2000	0	1	0
India	Sivaganga	Count	2017	1	1	0
India	Mancherial	Proportion	2000	45.2	61.1	30.0
India	Mancherial	Proportion	2017	64.6	80.0	48.7
India	Mancherial	Count	2000	0	1	0

Table 4: ORS by Unit *(continued)*

Country	Unit	Metric	Year	Mean	Upper	Lower
India	Mancherial	Count	2017	1	1	0
India	Bulandshahr	Proportion	2000	24.4	41.5	13.4
India	Bulandshahr	Proportion	2017	44.7	61.2	28.9
India	Bulandshahr	Count	2000	0	0	0
India	Bulandshahr	Count	2017	0	1	0
India	South 24 Parganas	Proportion	2000	46.4	64.6	30.6
India	South 24 Parganas	Proportion	2017	64.9	79.8	46.4
India	South 24 Parganas	Count	2000	0	1	0
India	South 24 Parganas	Count	2017	1	1	0
India	Kra Daddi	Proportion	2000	45.7	62.9	29.7
India	Kra Daddi	Proportion	2017	68.8	83.1	51.7
India	Kra Daddi	Count	2000	0	1	0
India	Kra Daddi	Count	2017	1	1	1
India	Lakhimpur	Proportion	2000	41.8	58.4	27.3
India	Lakhimpur	Proportion	2017	61.8	76.7	43.6
India	Lakhimpur	Count	2000	0	1	0
India	Lakhimpur	Count	2017	1	1	0
India	Madhubani	Proportion	2000	25.1	40.3	13.6
India	Madhubani	Proportion	2017	47.7	64.8	30.5
India	Madhubani	Count	2000	0	0	0
India	Madhubani	Count	2017	0	1	0
India	Narayanpur	Proportion	2000	41.5	58.2	24.7
India	Narayanpur	Proportion	2017	61.5	76.4	46.1
India	Narayanpur	Count	2000	0	1	0
India	Narayanpur	Count	2017	1	1	0
India	Morbi	Proportion	2000	39.0	58.6	21.2
India	Morbi	Proportion	2017	60.4	75.2	43.9
India	Morbi	Count	2000	0	1	0
India	Morbi	Count	2017	1	1	0
India	Sonipat	Proportion	2000	31.3	48.3	17.1
India	Sonipat	Proportion	2017	54.2	70.4	38.8
India	Sonipat	Count	2000	0	0	0
India	Sonipat	Count	2017	1	1	0
India	Shupiyan	Proportion	2000	41.5	60.0	25.0
India	Shupiyan	Proportion	2017	63.2	78.2	44.4
India	Shupiyan	Count	2000	0	1	0
India	Shupiyan	Count	2017	1	1	0
India	Ramgarh	Proportion	2000	41.5	60.8	25.5
India	Ramgarh	Proportion	2017	62.7	77.6	44.6
India	Ramgarh	Count	2000	0	1	0
India	Ramgarh	Count	2017	1	1	0
India	Kolar	Proportion	2000	45.8	63.3	27.0
India	Kolar	Proportion	2017	65.7	80.2	46.6
India	Kolar	Count	2000	0	1	0
India	Kolar	Count	2017	1	1	0
India	Gwalior	Proportion	2000	36.1	54.6	21.2
India	Gwalior	Proportion	2017	58.4	76.1	39.2
India	Gwalior	Count	2000	0	1	0
India	Gwalior	Count	2017	1	1	0

Table 4: ORS by Unit *(continued)*

Country	Unit	Metric	Year	Mean	Upper	Lower
India	Nanded	Proportion	2000	43.5	62.6	29.3
India	Nanded	Proportion	2017	64.3	78.5	49.9
India	Nanded	Count	2000	0	1	0
India	Nanded	Count	2017	1	1	0
India	Koraput	Proportion	2000	44.8	60.4	29.9
India	Koraput	Proportion	2017	67.5	80.6	52.2
India	Koraput	Count	2000	0	1	0
India	Koraput	Count	2017	1	1	1
India	Sangrur	Proportion	2000	38.1	55.0	23.5
India	Sangrur	Proportion	2017	60.7	75.0	44.4
India	Sangrur	Count	2000	0	1	0
India	Sangrur	Count	2017	1	1	0
India	Jhalawar	Proportion	2000	33.5	48.9	20.1
India	Jhalawar	Proportion	2017	55.6	72.5	39.3
India	Jhalawar	Count	2000	0	0	0
India	Jhalawar	Count	2017	1	1	0
India	Thanjavur	Proportion	2000	41.7	58.1	26.0
India	Thanjavur	Proportion	2017	62.7	77.7	46.0
India	Thanjavur	Count	2000	0	1	0
India	Thanjavur	Count	2017	1	1	0
India	Medchal Malkajgiri	Proportion	2000	46.8	63.9	28.8
India	Medchal Malkajgiri	Proportion	2017	68.7	81.7	52.3
India	Medchal Malkajgiri	Count	2000	0	1	0
India	Medchal Malkajgiri	Count	2017	1	1	1
India	Chandauli	Proportion	2000	26.5	40.9	14.0
India	Chandauli	Proportion	2017	47.5	65.0	31.2
India	Chandauli	Count	2000	0	0	0
India	Chandauli	Count	2017	0	1	0
India	Uttar Dinajpur	Proportion	2000	39.7	56.5	23.7
India	Uttar Dinajpur	Proportion	2017	62.4	74.9	44.4
India	Uttar Dinajpur	Count	2000	0	1	0
India	Uttar Dinajpur	Count	2017	1	1	0
India	Kamle	Proportion	2000	43.2	61.3	27.7
India	Kamle	Proportion	2017	64.8	80.0	46.8
India	Kamle	Count	2000	0	1	0
India	Kamle	Count	2017	1	1	0
India	Morigaon	Proportion	2000	37.8	55.0	21.8
India	Morigaon	Proportion	2017	58.5	73.8	41.7
India	Morigaon	Count	2000	0	1	0
India	Morigaon	Count	2017	1	1	0
India	Munger	Proportion	2000	26.4	41.1	14.0
India	Munger	Proportion	2017	49.9	67.5	31.3
India	Munger	Count	2000	0	0	0
India	Munger	Count	2017	0	1	0
India	Raigarh	Proportion	2000	36.8	53.1	20.1
India	Raigarh	Proportion	2017	61.4	76.1	45.0
India	Raigarh	Count	2000	0	1	0
India	Raigarh	Count	2017	1	1	0
India	Narmada	Proportion	2000	41.4	60.6	23.9

Table 4: ORS by Unit *(continued)*

Country	Unit	Metric	Year	Mean	Upper	Lower
India	Narmada	Proportion	2017	61.7	77.5	43.2
India	Narmada	Count	2000	0	1	0
India	Narmada	Count	2017	1	1	0
India	Yamunanagar	Proportion	2000	36.9	57.5	21.1
India	Yamunanagar	Proportion	2017	60.5	75.8	43.6
India	Yamunanagar	Count	2000	0	1	0
India	Yamunanagar	Count	2017	1	1	0
India	Srinagar	Proportion	2000	43.0	61.0	26.4
India	Srinagar	Proportion	2017	64.6	78.9	46.4
India	Srinagar	Count	2000	0	1	0
India	Srinagar	Count	2017	1	1	0
India	Ranchi	Proportion	2000	42.4	60.9	27.6
India	Ranchi	Proportion	2017	64.0	78.8	46.4
India	Ranchi	Count	2000	0	1	0
India	Ranchi	Count	2017	1	1	0
India	Koppal	Proportion	2000	40.8	56.9	22.8
India	Koppal	Proportion	2017	64.5	77.8	52.0
India	Koppal	Count	2000	0	1	0
India	Koppal	Count	2017	1	1	1
India	Harda	Proportion	2000	35.9	54.3	19.0
India	Harda	Proportion	2017	55.5	72.2	36.3
India	Harda	Count	2000	0	1	0
India	Harda	Count	2017	1	1	0
India	Nandurbar	Proportion	2000	41.9	59.9	24.6
India	Nandurbar	Proportion	2017	63.4	77.5	45.7
India	Nandurbar	Count	2000	0	1	0
India	Nandurbar	Count	2017	1	1	0
India	Malkangiri	Proportion	2000	42.9	59.6	27.5
India	Malkangiri	Proportion	2017	65.2	78.4	49.1
India	Malkangiri	Count	2000	0	1	0
India	Malkangiri	Count	2017	1	1	0
India	Shahid Bhagat Singh Nagar	Proportion	2000	40.2	58.7	24.1
India	Shahid Bhagat Singh Nagar	Proportion	2017	62.0	76.1	45.8
India	Shahid Bhagat Singh Nagar	Count	2000	0	1	0
India	Shahid Bhagat Singh Nagar	Count	2017	1	1	0
India	Jhunjhunun	Proportion	2000	31.9	47.4	19.5
India	Jhunjhunun	Proportion	2017	55.0	72.4	37.4
India	Jhunjhunun	Count	2000	0	0	0
India	Jhunjhunun	Count	2017	1	1	0
India	The Nilgiris	Proportion	2000	48.7	67.5	32.4
India	The Nilgiris	Proportion	2017	71.3	84.4	55.5
India	The Nilgiris	Count	2000	0	1	0
India	The Nilgiris	Count	2017	1	1	1
India	Nagarkurnool	Proportion	2000	42.0	60.1	25.6
India	Nagarkurnool	Proportion	2017	64.5	77.1	47.0
India	Nagarkurnool	Count	2000	0	1	0

Table 4: ORS by Unit *(continued)*

Country	Unit	Metric	Year	Mean	Upper	Lower
India	Nagarkurnool	Count	2017	1	1	0
India	Chitrakoot	Proportion	2000	27.4	42.1	13.9
India	Chitrakoot	Proportion	2017	49.6	67.7	30.3
India	Chitrakoot	Count	2000	0	0	0
India	Chitrakoot	Count	2017	0	1	0
India	Jhargram	Proportion	2000	40.5	57.4	25.2
India	Jhargram	Proportion	2017	61.4	76.9	42.6
India	Jhargram	Count	2000	0	1	0
India	Jhargram	Count	2017	1	1	0
India	Lower Siang	Proportion	2000	43.8	60.4	27.5
India	Lower Siang	Proportion	2017	64.5	79.2	46.0
India	Lower Siang	Count	2000	0	1	0
India	Lower Siang	Count	2017	1	1	0
India	Nagaon	Proportion	2000	38.9	55.7	23.6
India	Nagaon	Proportion	2017	60.1	73.9	43.5
India	Nagaon	Count	2000	0	1	0
India	Nagaon	Count	2017	1	1	0
India	Muzaffarpur	Proportion	2000	26.2	44.0	14.5
India	Muzaffarpur	Proportion	2017	47.5	65.4	29.5
India	Muzaffarpur	Count	2000	0	0	0
India	Muzaffarpur	Count	2017	0	1	0
India	Raipur	Proportion	2000	39.5	59.2	21.1
India	Raipur	Proportion	2017	61.1	77.4	44.5
India	Raipur	Count	2000	0	1	0
India	Raipur	Count	2017	1	1	0
India	Navsari	Proportion	2000	42.8	61.6	24.4
India	Navsari	Proportion	2017	62.0	76.8	42.3
India	Navsari	Count	2000	0	1	0
India	Navsari	Count	2017	1	1	0
India	Charkhi Dadri	Proportion	2000	32.9	52.4	18.0
India	Charkhi Dadri	Proportion	2017	56.5	74.5	38.5
India	Charkhi Dadri	Count	2000	0	1	0
India	Charkhi Dadri	Count	2017	1	1	0
India	Udhampur	Proportion	2000	43.2	63.6	27.4
India	Udhampur	Proportion	2017	64.5	79.5	47.4
India	Udhampur	Count	2000	0	1	0
India	Udhampur	Count	2017	1	1	0
India	Sahibganj	Proportion	2000	35.9	54.2	20.6
India	Sahibganj	Proportion	2017	57.1	71.9	39.4
India	Sahibganj	Count	2000	0	1	0
India	Sahibganj	Count	2017	1	1	0
India	Mandya	Proportion	2000	42.6	60.5	25.6
India	Mandya	Proportion	2017	65.1	77.2	50.7
India	Mandya	Count	2000	0	1	0
India	Mandya	Count	2017	1	1	1
India	Hoshangabad	Proportion	2000	36.0	53.8	22.3
India	Hoshangabad	Proportion	2017	57.4	74.8	40.7
India	Hoshangabad	Count	2000	0	1	0
India	Hoshangabad	Count	2017	1	1	0

Table 4: ORS by Unit *(continued)*

Country	Unit	Metric	Year	Mean	Upper	Lower
India	Nashik	Proportion	2000	45.9	63.2	30.4
India	Nashik	Proportion	2017	66.7	80.7	50.9
India	Nashik	Count	2000	0	1	0
India	Nashik	Count	2017	1	1	1
India	Mayurbhanj	Proportion	2000	43.2	57.8	27.8
India	Mayurbhanj	Proportion	2017	63.5	76.8	49.0
India	Mayurbhanj	Count	2000	0	1	0
India	Mayurbhanj	Count	2017	1	1	0
India	Tarn Taran	Proportion	2000	33.4	51.0	20.7
India	Tarn Taran	Proportion	2017	56.6	73.8	40.4
India	Tarn Taran	Count	2000	0	1	0
India	Tarn Taran	Count	2017	1	1	0
India	Jodhpur	Proportion	2000	34.1	49.6	21.7
India	Jodhpur	Proportion	2017	55.9	70.3	40.3
India	Jodhpur	Count	2000	0	0	0
India	Jodhpur	Count	2017	1	1	0
India	Theni	Proportion	2000	44.3	65.2	27.3
India	Theni	Proportion	2017	64.8	79.3	46.6
India	Theni	Count	2000	0	1	0
India	Theni	Count	2017	1	1	0
India	Nirmal	Proportion	2000	45.7	64.0	29.7
India	Nirmal	Proportion	2017	64.7	79.2	48.7
India	Nirmal	Count	2000	0	1	0
India	Nirmal	Count	2017	1	1	0
India	Deoria	Proportion	2000	26.0	41.3	14.6
India	Deoria	Proportion	2017	46.4	64.2	29.0
India	Deoria	Count	2000	0	0	0
India	Deoria	Count	2017	0	1	0
India	Kalimpong	Proportion	2000	50.1	68.1	30.5
India	Kalimpong	Proportion	2017	71.0	83.7	54.4
India	Kalimpong	Count	2000	1	1	0
India	Kalimpong	Count	2017	1	1	1
India	Shi Yomi	Proportion	2000	44.5	62.3	27.5
India	Shi Yomi	Proportion	2017	66.3	81.3	48.3
India	Shi Yomi	Count	2000	0	1	0
India	Shi Yomi	Count	2017	1	1	0
India	Nalbari	Proportion	2000	40.4	56.7	24.2
India	Nalbari	Proportion	2017	60.0	77.2	41.5
India	Nalbari	Count	2000	0	1	0
India	Nalbari	Count	2017	1	1	0
India	Nalanda	Proportion	2000	25.8	42.7	12.8
India	Nalanda	Proportion	2017	48.3	64.6	31.8
India	Nalanda	Count	2000	0	0	0
India	Nalanda	Count	2017	0	1	0
India	Rajnandgaon	Proportion	2000	40.4	56.7	23.4
India	Rajnandgaon	Proportion	2017	60.5	73.8	45.5
India	Rajnandgaon	Count	2000	0	1	0
India	Rajnandgaon	Count	2017	1	1	0
India	Panch Mahals	Proportion	2000	39.4	60.1	23.4

Table 4: ORS by Unit *(continued)*

Country	Unit	Metric	Year	Mean	Upper	Lower
India	Panch Mahals	Proportion	2017	61.8	78.2	44.7
India	Panch Mahals	Count	2000	0	1	0
India	Panch Mahals	Count	2017	1	1	0
India	Saraikela Kharsawan	Proportion	2000	38.9	55.1	24.8
India	Saraikela Kharsawan	Proportion	2017	60.4	77.5	43.0
India	Saraikela Kharsawan	Count	2000	0	1	0
India	Saraikela Kharsawan	Count	2017	1	1	0
India	Mysuru	Proportion	2000	42.9	59.1	25.1
India	Mysuru	Proportion	2017	65.8	79.4	51.8
India	Mysuru	Count	2000	0	1	0
India	Mysuru	Count	2017	1	1	1
India	Indore	Proportion	2000	41.2	58.8	24.7
India	Indore	Proportion	2017	60.6	77.9	42.9
India	Indore	Count	2000	0	1	0
India	Indore	Count	2017	1	1	0
India	Osmanabad	Proportion	2000	42.8	58.9	28.1
India	Osmanabad	Proportion	2017	63.9	77.8	47.9
India	Osmanabad	Count	2000	0	1	0
India	Osmanabad	Count	2017	1	1	0
India	Nabarangapur	Proportion	2000	44.1	61.3	28.3
India	Nabarangapur	Proportion	2017	66.7	78.2	48.6
India	Nabarangapur	Count	2000	0	1	0
India	Nabarangapur	Count	2017	1	1	0
India	Karauli	Proportion	2000	31.8	47.4	17.6
India	Karauli	Proportion	2017	51.9	68.5	33.5
India	Karauli	Count	2000	0	0	0
India	Karauli	Count	2017	1	1	0
India	Thiruvallur	Proportion	2000	46.1	63.9	29.0
India	Thiruvallur	Proportion	2017	66.7	79.3	50.2
India	Thiruvallur	Count	2000	0	1	0
India	Thiruvallur	Count	2017	1	1	1
India	Peddapalli	Proportion	2000	43.3	61.0	27.2
India	Peddapalli	Proportion	2017	63.2	79.8	43.6
India	Peddapalli	Count	2000	0	1	0
India	Peddapalli	Count	2017	1	1	0
India	Etah	Proportion	2000	24.0	40.6	12.2
India	Etah	Proportion	2017	44.3	63.6	28.5
India	Etah	Count	2000	0	0	0
India	Etah	Count	2017	0	1	0
India	Paschimi Barddhama	Proportion	2000	45.6	64.1	28.8
India	Paschimi Barddhama	Proportion	2017	66.2	82.7	48.7
India	Paschimi Barddhama	Count	2000	0	1	0
India	Paschimi Barddhama	Count	2017	1	1	0
India	Siang	Proportion	2000	46.2	65.3	29.1
India	Siang	Proportion	2017	69.1	82.1	51.0
India	Siang	Count	2000	0	1	0
India	Siang	Count	2017	1	1	1
India	Sivasagar	Proportion	2000	43.8	61.7	28.8
India	Sivasagar	Proportion	2017	62.6	78.4	45.9

Table 4: ORS by Unit *(continued)*

Country	Unit	Metric	Year	Mean	Upper	Lower
India	Sivasagar	Count	2000	0	1	0
India	Sivasagar	Count	2017	1	1	0
India	Nawada	Proportion	2000	25.4	42.2	12.2
India	Nawada	Proportion	2017	48.0	65.1	32.4
India	Nawada	Count	2000	0	0	0
India	Nawada	Count	2017	0	1	0
India	Sukma	Proportion	2000	38.6	55.3	25.0
India	Sukma	Proportion	2017	59.2	73.6	40.4
India	Sukma	Count	2000	0	1	0
India	Sukma	Count	2017	1	1	0
India	Patan	Proportion	2000	35.5	51.2	21.8
India	Patan	Proportion	2017	57.5	73.2	39.4
India	Patan	Count	2000	0	1	0
India	Patan	Count	2017	1	1	0
India	Simdega	Proportion	2000	38.8	55.7	22.2
India	Simdega	Proportion	2017	60.7	76.8	42.9
India	Simdega	Count	2000	0	1	0
India	Simdega	Count	2017	1	1	0
India	Raichur	Proportion	2000	40.5	58.7	23.2
India	Raichur	Proportion	2017	63.3	76.3	48.0
India	Raichur	Count	2000	0	1	0
India	Raichur	Count	2017	1	1	0
India	Jabalpur	Proportion	2000	39.0	56.1	21.6
India	Jabalpur	Proportion	2017	61.2	75.7	44.3
India	Jabalpur	Count	2000	0	1	0
India	Jabalpur	Count	2017	1	1	0
India	Palghar	Proportion	2000	47.7	66.8	28.2
India	Palghar	Proportion	2017	68.5	82.8	50.1
India	Palghar	Count	2000	0	1	0
India	Palghar	Count	2017	1	1	1
India	Nayagarh	Proportion	2000	42.7	58.8	28.1
India	Nayagarh	Proportion	2017	63.3	78.3	46.0
India	Nayagarh	Count	2000	0	1	0
India	Nayagarh	Count	2017	1	1	0
India	Kota	Proportion	2000	35.2	51.0	21.9
India	Kota	Proportion	2017	57.7	73.9	38.7
India	Kota	Count	2000	0	1	0
India	Kota	Count	2017	1	1	0
India	Thiruvarur	Proportion	2000	41.4	57.9	26.0
India	Thiruvarur	Proportion	2017	62.4	78.7	44.2
India	Thiruvarur	Count	2000	0	1	0
India	Thiruvarur	Count	2017	1	1	0
India	Rajanna Sircilla	Proportion	2000	45.0	62.1	28.1
India	Rajanna Sircilla	Proportion	2017	65.3	81.1	45.6
India	Rajanna Sircilla	Count	2000	0	1	0
India	Rajanna Sircilla	Count	2017	1	1	0
India	Etawah	Proportion	2000	25.8	41.1	14.1
India	Etawah	Proportion	2017	47.5	66.3	30.0
India	Etawah	Count	2000	0	0	0

Table 4: ORS by Unit *(continued)*

Country	Unit	Metric	Year	Mean	Upper	Lower
India	Etawah	Count	2017	0	1	0
India	Sonitpur	Proportion	2000	39.2	55.5	23.7
India	Sonitpur	Proportion	2017	60.7	75.7	42.7
India	Sonitpur	Count	2000	0	1	0
India	Sonitpur	Count	2017	1	1	0
India	Pashchim Champaran	Proportion	2000	24.8	38.2	13.9
India	Pashchim Champaran	Proportion	2017	47.1	65.9	29.6
India	Pashchim Champaran	Count	2000	0	0	0
India	Pashchim Champaran	Count	2017	0	1	0
India	Surajpur	Proportion	2000	38.5	55.9	24.3
India	Surajpur	Proportion	2017	61.4	75.8	45.6
India	Surajpur	Count	2000	0	1	0
India	Surajpur	Count	2017	1	1	0
India	Porbandar	Proportion	2000	40.8	58.0	23.3
India	Porbandar	Proportion	2017	62.8	77.7	45.2
India	Porbandar	Count	2000	0	1	0
India	Porbandar	Count	2017	1	1	0
India	Ramanagara	Proportion	2000	44.2	61.3	26.9
India	Ramanagara	Proportion	2017	66.9	79.6	48.7
India	Ramanagara	Count	2000	0	1	0
India	Ramanagara	Count	2017	1	1	0
India	Jhabua	Proportion	2000	38.0	57.1	22.4
India	Jhabua	Proportion	2017	56.3	74.4	36.1
India	Jhabua	Count	2000	0	1	0
India	Jhabua	Count	2017	1	1	0
India	Parbhani	Proportion	2000	44.1	60.6	29.0
India	Parbhani	Proportion	2017	63.6	77.8	45.6
India	Parbhani	Count	2000	0	1	0
India	Parbhani	Count	2017	1	1	0
India	Nuapada	Proportion	2000	41.2	59.6	22.5
India	Nuapada	Proportion	2017	64.1	78.7	47.2
India	Nuapada	Count	2000	0	1	0
India	Nuapada	Count	2017	1	1	0
India	Nagaur	Proportion	2000	32.8	46.4	21.7
India	Nagaur	Proportion	2017	54.9	70.5	40.2
India	Nagaur	Count	2000	0	0	0
India	Nagaur	Count	2017	1	1	0
India	Tuticorin	Proportion	2000	44.0	61.4	25.6
India	Tuticorin	Proportion	2017	62.6	78.9	45.6
India	Tuticorin	Count	2000	0	1	0
India	Tuticorin	Count	2017	1	1	0
India	Sangareddy	Proportion	2000	43.5	60.3	26.6
India	Sangareddy	Proportion	2017	66.3	78.6	53.0
India	Sangareddy	Count	2000	0	1	0
India	Sangareddy	Count	2017	1	1	1
India	Faizabad	Proportion	2000	26.3	43.0	13.6
India	Faizabad	Proportion	2017	47.6	66.1	31.8
India	Faizabad	Count	2000	0	0	0
India	Faizabad	Count	2017	0	1	0

Table 4: ORS by Unit *(continued)*

Country	Unit	Metric	Year	Mean	Upper	Lower
India	Tinsukia	Proportion	2000	42.4	58.3	26.1
India	Tinsukia	Proportion	2017	64.4	79.2	48.0
India	Tinsukia	Count	2000	0	1	0
India	Tinsukia	Count	2017	1	1	0
India	Patna	Proportion	2000	27.7	45.3	15.1
India	Patna	Proportion	2017	50.2	64.4	33.4
India	Patna	Count	2000	0	0	0
India	Patna	Count	2017	1	1	0
India	Surguja	Proportion	2000	39.3	56.7	24.6
India	Surguja	Proportion	2017	62.0	77.1	44.2
India	Surguja	Count	2000	0	1	0
India	Surguja	Count	2017	1	1	0
India	Rajkot	Proportion	2000	40.9	57.8	24.0
India	Rajkot	Proportion	2017	62.1	76.7	46.9
India	Rajkot	Count	2000	0	1	0
India	Rajkot	Count	2017	1	1	0
India	Shivamogga	Proportion	2000	42.2	57.1	25.3
India	Shivamogga	Proportion	2017	65.9	78.4	48.8
India	Shivamogga	Count	2000	0	1	0
India	Shivamogga	Count	2017	1	1	0
India	Katni	Proportion	2000	38.0	56.8	20.9
India	Katni	Proportion	2017	59.3	73.8	41.8
India	Katni	Count	2000	0	1	0
India	Katni	Count	2017	1	1	0
India	Pune	Proportion	2000	47.8	64.5	30.0
India	Pune	Proportion	2017	67.6	81.1	52.4
India	Pune	Count	2000	0	1	0
India	Pune	Count	2017	1	1	1
India	Puri	Proportion	2000	43.7	61.9	26.9
India	Puri	Proportion	2017	63.3	77.8	46.3
India	Puri	Count	2000	0	1	0
India	Puri	Count	2017	1	1	0
India	Pali	Proportion	2000	33.2	49.0	19.6
India	Pali	Proportion	2017	55.7	70.8	41.5
India	Pali	Count	2000	0	0	0
India	Pali	Count	2017	1	1	0
India	Tiruchirappalli	Proportion	2000	43.4	59.9	25.4
India	Tiruchirappalli	Proportion	2017	64.4	78.9	48.4
India	Tiruchirappalli	Count	2000	0	1	0
India	Tiruchirappalli	Count	2017	1	1	0
India	Siddipet	Proportion	2000	44.5	62.0	27.4
India	Siddipet	Proportion	2017	65.9	80.7	48.4
India	Siddipet	Count	2000	0	1	0
India	Siddipet	Count	2017	1	1	0
India	Farrukhabad	Proportion	2000	25.1	44.3	11.9
India	Farrukhabad	Proportion	2017	44.8	62.3	27.4
India	Farrukhabad	Count	2000	0	0	0
India	Farrukhabad	Count	2017	0	1	0
India	Udalguri	Proportion	2000	40.4	56.1	23.7

Table 4: ORS by Unit *(continued)*

Country	Unit	Metric	Year	Mean	Upper	Lower
India	Udalguri	Proportion	2017	61.0	75.9	44.8
India	Udalguri	Count	2000	0	1	0
India	Udalguri	Count	2017	1	1	0
India	Purba Champaran	Proportion	2000	24.8	38.4	14.3
India	Purba Champaran	Proportion	2017	45.7	65.0	30.3
India	Purba Champaran	Count	2000	0	0	0
India	Purba Champaran	Count	2017	0	1	0
India	Uttar Bastar Kanker	Proportion	2000	40.7	59.4	24.2
India	Uttar Bastar Kanker	Proportion	2017	60.3	73.5	44.1
India	Uttar Bastar Kanker	Count	2000	0	1	0
India	Uttar Bastar Kanker	Count	2017	1	1	0
India	Sabar Kantha	Proportion	2000	37.2	55.0	22.6
India	Sabar Kantha	Proportion	2017	61.4	78.1	44.5
India	Sabar Kantha	Count	2000	0	1	0
India	Sabar Kantha	Count	2017	1	1	0
India	Tumakuru	Proportion	2000	43.8	63.3	27.3
India	Tumakuru	Proportion	2017	65.8	78.1	51.7
India	Tumakuru	Count	2000	0	1	0
India	Tumakuru	Count	2017	1	1	1
India	Mandla	Proportion	2000	39.0	55.4	24.2
India	Mandla	Proportion	2017	61.9	76.4	45.9
India	Mandla	Count	2000	0	1	0
India	Mandla	Count	2017	1	1	0
India	Raigad	Proportion	2000	50.8	67.9	33.8
India	Raigad	Proportion	2017	71.2	82.6	57.1
India	Raigad	Count	2000	1	1	0
India	Raigad	Count	2017	1	1	1
India	Rayagada	Proportion	2000	42.7	63.0	26.9
India	Rayagada	Proportion	2017	64.3	78.2	48.0
India	Rayagada	Count	2000	0	1	0
India	Rayagada	Count	2017	1	1	0
India	Pratapgarh	Proportion	2000	34.6	51.3	21.4
India	Pratapgarh	Proportion	2017	56.8	72.8	40.0
India	Pratapgarh	Count	2000	0	1	0
India	Pratapgarh	Count	2017	1	1	0
India	Tirunelveli	Proportion	2000	43.2	62.8	25.8
India	Tirunelveli	Proportion	2017	63.4	79.3	45.1
India	Tirunelveli	Count	2000	0	1	0
India	Tirunelveli	Count	2017	1	1	0
India	Suryapet	Proportion	2000	42.4	61.4	23.7
India	Suryapet	Proportion	2017	63.0	78.0	48.2
India	Suryapet	Count	2000	0	1	0
India	Suryapet	Count	2017	1	1	0
India	Fatehpur	Proportion	2000	26.2	40.2	13.7
India	Fatehpur	Proportion	2017	47.8	63.7	29.5
India	Fatehpur	Count	2000	0	0	0
India	Fatehpur	Count	2017	0	1	0
India	South Salmara Mancachar	Proportion	2000	41.0	58.2	23.8
India	South Salmara Mancachar	Proportion	2017	61.5	76.5	44.6

Table 4: ORS by Unit *(continued)*

Country	Unit	Metric	Year	Mean	Upper	Lower
India	South Salmara Mancachar	Count	2000	0	1	0
India	South Salmara Mancachar	Count	2017	1	1	0
India	Purnia	Proportion	2000	25.9	42.9	13.0
India	Purnia	Proportion	2017	49.6	66.6	33.2
India	Purnia	Count	2000	0	0	0
India	Purnia	Count	2017	0	1	0
India	Surat	Proportion	2000	42.7	59.4	25.8
India	Surat	Proportion	2017	63.7	78.2	47.1
India	Surat	Count	2000	0	1	0
India	Surat	Count	2017	1	1	0
India	Udupi	Proportion	2000	47.6	66.0	29.9
India	Udupi	Proportion	2017	70.8	82.6	54.6
India	Udupi	Count	2000	0	1	0
India	Udupi	Count	2017	1	1	1
India	Mandsaur	Proportion	2000	37.2	56.0	22.2
India	Mandsaur	Proportion	2017	57.9	73.4	39.3
India	Mandsaur	Count	2000	0	1	0
India	Mandsaur	Count	2017	1	1	0
India	Ratnagiri	Proportion	2000	49.8	67.6	35.0
India	Ratnagiri	Proportion	2017	71.0	81.9	57.6
India	Ratnagiri	Count	2000	0	1	0
India	Ratnagiri	Count	2017	1	1	1
India	Sambalpur	Proportion	2000	42.4	60.0	26.5
India	Sambalpur	Proportion	2017	65.9	80.7	48.3
India	Sambalpur	Count	2000	0	1	0
India	Sambalpur	Count	2017	1	1	0
India	Rajsamand	Proportion	2000	36.1	53.0	21.0
India	Rajsamand	Proportion	2017	59.4	74.2	44.4
India	Rajsamand	Count	2000	0	1	0
India	Rajsamand	Count	2017	1	1	0
India	Tiruppur	Proportion	2000	43.8	62.6	27.4
India	Tiruppur	Proportion	2017	65.6	79.5	49.4
India	Tiruppur	Count	2000	0	1	0
India	Tiruppur	Count	2017	1	1	0
India	Vikarabad	Proportion	2000	42.5	60.6	26.1
India	Vikarabad	Proportion	2017	66.4	79.5	47.9
India	Vikarabad	Count	2000	0	1	0
India	Vikarabad	Count	2017	1	1	0
India	Firozabad	Proportion	2000	24.4	39.9	13.4
India	Firozabad	Proportion	2017	45.6	65.6	28.6
India	Firozabad	Count	2000	0	0	0
India	Firozabad	Count	2017	0	1	0
India	West Karbi Anglong	Proportion	2000	39.2	56.0	23.0
India	West Karbi Anglong	Proportion	2017	60.1	74.1	44.7
India	West Karbi Anglong	Count	2000	0	1	0
India	West Karbi Anglong	Count	2017	1	1	0
India	Rohtas	Proportion	2000	24.3	38.1	12.5
India	Rohtas	Proportion	2017	47.8	65.8	30.1
India	Rohtas	Count	2000	0	0	0

Table 4: ORS by Unit *(continued)*

Country	Unit	Metric	Year	Mean	Upper	Lower
India	Rohtas	Count	2017	0	1	0
India	Surendranagar	Proportion	2000	39.2	55.1	22.8
India	Surendranagar	Proportion	2017	59.4	73.2	42.8
India	Surendranagar	Count	2000	0	1	0
India	Surendranagar	Count	2017	1	1	0
India	Uttara Kannada	Proportion	2000	46.8	62.4	31.7
India	Uttara Kannada	Proportion	2017	68.9	80.5	54.5
India	Uttara Kannada	Count	2000	0	1	0
India	Uttara Kannada	Count	2017	1	1	1
India	Morena	Proportion	2000	31.6	47.4	16.2
India	Morena	Proportion	2017	53.0	70.4	36.0
India	Morena	Count	2000	0	0	0
India	Morena	Count	2017	1	1	0
India	Sangli	Proportion	2000	43.5	61.3	28.6
India	Sangli	Proportion	2017	65.2	77.2	48.2
India	Sangli	Count	2000	0	1	0
India	Sangli	Count	2017	1	1	0
India	Sonepur	Proportion	2000	39.7	57.4	24.2
India	Sonepur	Proportion	2017	64.0	78.8	46.7
India	Sonepur	Count	2000	0	1	0
India	Sonepur	Count	2017	1	1	0
India	Sawai Madhopur	Proportion	2000	33.4	52.3	19.7
India	Sawai Madhopur	Proportion	2017	53.7	70.7	38.6
India	Sawai Madhopur	Count	2000	0	1	0
India	Sawai Madhopur	Count	2017	1	1	0
India	Tiruvannamalai	Proportion	2000	44.4	61.3	29.0
India	Tiruvannamalai	Proportion	2017	65.1	78.6	49.7
India	Tiruvannamalai	Count	2000	0	1	0
India	Tiruvannamalai	Count	2017	1	1	0
India	Wanaparthy	Proportion	2000	41.3	59.1	24.6
India	Wanaparthy	Proportion	2017	64.5	78.1	46.1
India	Wanaparthy	Count	2000	0	1	0
India	Wanaparthy	Count	2017	1	1	0
India	Gautam Buddha Nagar	Proportion	2000	31.9	49.8	16.9
India	Gautam Buddha Nagar	Proportion	2017	52.8	70.5	34.5
India	Gautam Buddha Nagar	Count	2000	0	0	0
India	Gautam Buddha Nagar	Count	2017	1	1	0
India	Majuli	Proportion	2000	40.9	57.6	26.9
India	Majuli	Proportion	2017	59.4	75.2	41.7
India	Majuli	Count	2000	0	1	0
India	Majuli	Count	2017	1	1	0
India	Saharsa	Proportion	2000	24.7	40.3	12.4
India	Saharsa	Proportion	2017	47.3	66.3	28.8
India	Saharsa	Count	2000	0	0	0
India	Saharsa	Count	2017	0	1	0
India	Tapi	Proportion	2000	41.9	59.7	25.3
India	Tapi	Proportion	2017	62.1	77.5	44.6
India	Tapi	Count	2000	0	1	0
India	Tapi	Count	2017	1	1	0

Table 4: ORS by Unit *(continued)*

Country	Unit	Metric	Year	Mean	Upper	Lower
India	Yadgir	Proportion	2000	40.7	56.7	24.1
India	Yadgir	Proportion	2017	64.6	77.4	49.4
India	Yadgir	Count	2000	0	1	0
India	Yadgir	Count	2017	1	1	0
India	Narsimhapur	Proportion	2000	36.3	53.5	22.1
India	Narsimhapur	Proportion	2017	57.8	73.9	39.7
India	Narsimhapur	Count	2000	0	1	0
India	Narsimhapur	Count	2017	1	1	0
India	Satara	Proportion	2000	45.8	63.1	31.1
India	Satara	Proportion	2017	66.7	79.7	50.4
India	Satara	Count	2000	0	1	0
India	Satara	Count	2017	1	1	1
India	Sundargarh	Proportion	2000	43.3	59.9	29.1
India	Sundargarh	Proportion	2017	66.5	78.5	50.7
India	Sundargarh	Count	2000	0	1	0
India	Sundargarh	Count	2017	1	1	1
India	Sikar	Proportion	2000	32.8	48.5	19.5
India	Sikar	Proportion	2017	55.4	70.6	37.9
India	Sikar	Count	2000	0	0	0
India	Sikar	Count	2017	1	1	0
India	Vellore	Proportion	2000	45.2	61.5	29.5
India	Vellore	Proportion	2017	65.9	78.5	50.9
India	Vellore	Count	2000	0	1	0
India	Vellore	Count	2017	1	1	1
India	Warangal Rural	Proportion	2000	42.6	58.9	26.5
India	Warangal Rural	Proportion	2017	63.3	77.7	46.7
India	Warangal Rural	Count	2000	0	1	0
India	Warangal Rural	Count	2017	1	1	0
India	Ghaziabad	Proportion	2000	31.0	49.4	17.3
India	Ghaziabad	Proportion	2017	51.6	70.1	33.7
India	Ghaziabad	Count	2000	0	0	0
India	Ghaziabad	Count	2017	1	1	0
India	Charaideo	Proportion	2000	42.4	57.9	26.7
India	Charaideo	Proportion	2017	61.5	78.9	45.4
India	Charaideo	Count	2000	0	1	0
India	Charaideo	Count	2017	1	1	0
India	Samastipur	Proportion	2000	25.5	41.7	13.4
India	Samastipur	Proportion	2017	47.5	63.2	28.5
India	Samastipur	Count	2000	0	0	0
India	Samastipur	Count	2017	0	1	0
India	Dang	Proportion	2000	45.0	61.5	29.1
India	Dang	Proportion	2017	64.5	78.6	46.3
India	Dang	Count	2000	0	1	0
India	Dang	Count	2017	1	1	0
India	Neemuch	Proportion	2000	37.4	55.0	22.7
India	Neemuch	Proportion	2017	58.9	73.7	39.3
India	Neemuch	Count	2000	0	1	0
India	Neemuch	Count	2017	1	1	0
India	Sindhudurg	Proportion	2000	47.7	65.5	29.6

Table 4: ORS by Unit *(continued)*

Country	Unit	Metric	Year	Mean	Upper	Lower
India	Sindhudurg	Proportion	2017	68.6	81.8	53.4
India	Sindhudurg	Count	2000	0	1	0
India	Sindhudurg	Count	2017	1	1	1
India	Sirohi	Proportion	2000	34.6	52.0	20.8
India	Sirohi	Proportion	2017	57.7	73.3	40.3
India	Sirohi	Count	2000	0	1	0
India	Sirohi	Count	2017	1	1	0
India	Viluppuram	Proportion	2000	43.6	59.3	29.4
India	Viluppuram	Proportion	2017	64.0	77.6	50.0
India	Viluppuram	Count	2000	0	1	0
India	Viluppuram	Count	2017	1	1	0
India	Yadadri Bhuvanagiri	Proportion	2000	43.6	60.6	25.7
India	Yadadri Bhuvanagiri	Proportion	2017	65.6	80.0	49.4
India	Yadadri Bhuvanagiri	Count	2000	0	1	0
India	Yadadri Bhuvanagiri	Count	2017	1	1	0
India	Ghazipur	Proportion	2000	26.0	41.4	12.7
India	Ghazipur	Proportion	2017	46.8	64.4	29.6
India	Ghazipur	Count	2000	0	0	0
India	Ghazipur	Count	2017	0	1	0
India	Biswanath	Proportion	2000	39.8	58.7	24.9
India	Biswanath	Proportion	2017	60.6	75.9	42.8
India	Biswanath	Count	2000	0	1	0
India	Biswanath	Count	2017	1	1	0
India	Saran	Proportion	2000	24.3	41.3	13.9
India	Saran	Proportion	2017	46.3	63.2	29.1
India	Saran	Count	2000	0	0	0
India	Saran	Count	2017	0	1	0
India	Vadodara	Proportion	2000	41.1	60.0	25.9
India	Vadodara	Proportion	2017	62.8	77.4	44.5
India	Vadodara	Count	2000	0	1	0
India	Vadodara	Count	2017	1	1	0
India	Panna	Proportion	2000	35.0	50.7	19.3
India	Panna	Proportion	2017	56.0	71.3	39.6
India	Panna	Count	2000	0	1	0
India	Panna	Count	2017	1	1	0
India	Solapur	Proportion	2000	41.8	59.7	24.8
India	Solapur	Proportion	2017	64.4	78.2	49.9
India	Solapur	Count	2000	0	1	0
India	Solapur	Count	2017	1	1	0
India	Tonk	Proportion	2000	33.1	50.6	18.4
India	Tonk	Proportion	2017	53.4	71.3	36.1
India	Tonk	Count	2000	0	1	0
India	Tonk	Count	2017	1	1	0
India	Virudunagar	Proportion	2000	43.3	62.5	26.0
India	Virudunagar	Proportion	2017	63.2	78.8	46.4
India	Virudunagar	Count	2000	0	1	0
India	Virudunagar	Count	2017	1	1	0
India	Gonda	Proportion	2000	24.0	41.5	12.6
India	Gonda	Proportion	2017	44.8	60.5	29.8

Table 4: ORS by Unit *(continued)*

Country	Unit	Metric	Year	Mean	Upper	Lower
India	Gonda	Count	2000	0	0	0
India	Gonda	Count	2017	0	1	0
India	Hojai	Proportion	2000	38.7	55.9	22.2
India	Hojai	Proportion	2017	60.3	75.0	42.9
India	Hojai	Count	2000	0	1	0
India	Hojai	Count	2017	1	1	0
India	Sheikhpura	Proportion	2000	25.8	42.5	12.6
India	Sheikhpura	Proportion	2017	48.1	65.6	31.7
India	Sheikhpura	Count	2000	0	0	0
India	Sheikhpura	Count	2017	0	1	0
India	Valsad	Proportion	2000	45.2	63.8	24.6
India	Valsad	Proportion	2017	64.6	79.3	48.6
India	Valsad	Count	2000	0	1	0
India	Valsad	Count	2017	1	1	0
India	Raisen	Proportion	2000	37.7	55.0	24.3
India	Raisen	Proportion	2017	58.8	75.7	41.9
India	Raisen	Count	2000	0	1	0
India	Raisen	Count	2017	1	1	0
India	Thane	Proportion	2000	50.7	68.6	32.3
India	Thane	Proportion	2017	70.7	83.6	55.6
India	Thane	Count	2000	1	1	0
India	Thane	Count	2017	1	1	1
India	Udaipur	Proportion	2000	36.1	52.0	22.4
India	Udaipur	Proportion	2017	59.1	71.6	43.0
India	Udaipur	Count	2000	0	1	0
India	Udaipur	Count	2017	1	1	0
India	Gorakhpur	Proportion	2000	25.0	39.3	13.6
India	Gorakhpur	Proportion	2017	45.2	63.9	28.8
India	Gorakhpur	Count	2000	0	0	0
India	Gorakhpur	Count	2017	0	1	0
India	Sheohar	Proportion	2000	26.0	42.4	15.0
India	Sheohar	Proportion	2017	47.1	66.7	30.7
India	Sheohar	Count	2000	0	0	0
India	Sheohar	Count	2017	0	1	0
India	Rajgarh	Proportion	2000	36.9	54.6	20.2
India	Rajgarh	Proportion	2017	57.9	73.6	39.7
India	Rajgarh	Count	2000	0	1	0
India	Rajgarh	Count	2017	1	1	0
India	Wardha	Proportion	2000	42.7	59.9	25.4
India	Wardha	Proportion	2017	64.3	77.0	48.5
India	Wardha	Count	2000	0	1	0
India	Wardha	Count	2017	1	1	0
India	Hamirpur	Proportion	2000	25.8	40.3	14.9
India	Hamirpur	Proportion	2017	48.2	64.7	31.6
India	Hamirpur	Count	2000	0	0	0
India	Hamirpur	Count	2017	0	1	0
India	Sitamarhi	Proportion	2000	25.4	39.8	13.4
India	Sitamarhi	Proportion	2017	46.6	63.4	29.4
India	Sitamarhi	Count	2000	0	0	0

Table 4: ORS by Unit *(continued)*

Country	Unit	Metric	Year	Mean	Upper	Lower
India	Sitamarhi	Count	2017	0	1	0
India	Ratlam	Proportion	2000	37.9	54.3	23.6
India	Ratlam	Proportion	2017	58.1	74.9	40.9
India	Ratlam	Count	2000	0	1	0
India	Ratlam	Count	2017	1	1	0
India	Washim	Proportion	2000	44.8	60.7	26.6
India	Washim	Proportion	2017	64.9	79.4	50.5
India	Washim	Count	2000	0	1	0
India	Washim	Count	2017	1	1	1
India	Hapur	Proportion	2000	25.6	43.1	13.3
India	Hapur	Proportion	2017	46.2	65.8	29.6
India	Hapur	Count	2000	0	0	0
India	Hapur	Count	2017	0	1	0
India	Siwan	Proportion	2000	22.7	37.5	11.9
India	Siwan	Proportion	2017	44.5	61.0	27.9
India	Siwan	Count	2000	0	0	0
India	Siwan	Count	2017	0	1	0
India	Rewa	Proportion	2000	35.8	52.5	21.6
India	Rewa	Proportion	2017	57.3	72.1	39.6
India	Rewa	Count	2000	0	1	0
India	Rewa	Count	2017	1	1	0
India	Yavatmal	Proportion	2000	43.8	61.0	29.4
India	Yavatmal	Proportion	2017	64.5	76.4	51.1
India	Yavatmal	Count	2000	0	1	0
India	Yavatmal	Count	2017	1	1	1
India	Hardoi	Proportion	2000	24.5	38.9	12.2
India	Hardoi	Proportion	2017	44.1	64.3	28.2
India	Hardoi	Count	2000	0	0	0
India	Hardoi	Count	2017	0	1	0
India	Supaul	Proportion	2000	24.6	40.4	12.9
India	Supaul	Proportion	2017	48.8	67.6	29.3
India	Supaul	Count	2000	0	0	0
India	Supaul	Count	2017	0	1	0
India	Sagar	Proportion	2000	37.5	54.2	23.4
India	Sagar	Proportion	2017	59.1	74.3	42.6
India	Sagar	Count	2000	0	1	0
India	Sagar	Count	2017	1	1	0
India	Hathras	Proportion	2000	25.5	40.1	12.6
India	Hathras	Proportion	2017	46.6	65.9	29.2
India	Hathras	Count	2000	0	0	0
India	Hathras	Count	2017	0	1	0
India	Vaishali	Proportion	2000	25.6	42.4	14.2
India	Vaishali	Proportion	2017	47.0	63.0	30.1
India	Vaishali	Count	2000	0	0	0
India	Vaishali	Count	2017	0	1	0
India	Satna	Proportion	2000	36.9	53.7	21.4
India	Satna	Proportion	2017	58.0	73.9	39.6
India	Satna	Count	2000	0	1	0
India	Satna	Count	2017	1	1	0

Table 4: ORS by Unit *(continued)*

Country	Unit	Metric	Year	Mean	Upper	Lower
India	Jalaun	Proportion	2000	26.7	42.9	15.1
India	Jalaun	Proportion	2017	48.7	66.3	32.7
India	Jalaun	Count	2000	0	0	0
India	Jalaun	Count	2017	0	1	0
India	Sehore	Proportion	2000	37.8	54.4	21.7
India	Sehore	Proportion	2017	59.2	74.1	43.7
India	Sehore	Count	2000	0	1	0
India	Sehore	Count	2017	1	1	0
India	Jaunpur	Proportion	2000	25.0	38.3	12.8
India	Jaunpur	Proportion	2017	46.7	62.7	31.6
India	Jaunpur	Count	2000	0	0	0
India	Jaunpur	Count	2017	0	1	0
India	Seoni	Proportion	2000	39.3	53.9	23.7
India	Seoni	Proportion	2017	61.1	76.3	46.9
India	Seoni	Count	2000	0	1	0
India	Seoni	Count	2017	1	1	0
India	Jhansi	Proportion	2000	29.8	46.6	17.5
India	Jhansi	Proportion	2017	52.2	67.1	37.0
India	Jhansi	Count	2000	0	0	0
India	Jhansi	Count	2017	1	1	0
India	Shahdol	Proportion	2000	38.2	54.7	23.0
India	Shahdol	Proportion	2017	59.0	72.0	40.6
India	Shahdol	Count	2000	0	1	0
India	Shahdol	Count	2017	1	1	0
India	Kannauj	Proportion	2000	24.4	41.3	11.9
India	Kannauj	Proportion	2017	44.4	63.6	28.2
India	Kannauj	Count	2000	0	0	0
India	Kannauj	Count	2017	0	1	0
India	Shajapur	Proportion	2000	37.0	55.2	19.6
India	Shajapur	Proportion	2017	57.9	74.8	41.1
India	Shajapur	Count	2000	0	1	0
India	Shajapur	Count	2017	1	1	0
India	Kanpur Dehat	Proportion	2000	26.4	42.3	13.4
India	Kanpur Dehat	Proportion	2017	47.6	66.4	29.5
India	Kanpur Dehat	Count	2000	0	0	0
India	Kanpur Dehat	Count	2017	0	1	0
India	Sheopur	Proportion	2000	33.8	49.5	19.2
India	Sheopur	Proportion	2017	55.5	69.9	39.4
India	Sheopur	Count	2000	0	0	0
India	Sheopur	Count	2017	1	1	0
India	Kanpur Nagar	Proportion	2000	27.5	43.8	14.3
India	Kanpur Nagar	Proportion	2017	49.3	67.3	29.5
India	Kanpur Nagar	Count	2000	0	0	0
India	Kanpur Nagar	Count	2017	0	1	0
India	Shivpuri	Proportion	2000	36.4	52.3	21.9
India	Shivpuri	Proportion	2017	57.6	73.7	41.5
India	Shivpuri	Count	2000	0	1	0
India	Shivpuri	Count	2017	1	1	0
India	Kasganj	Proportion	2000	23.7	38.4	12.3

Table 4: ORS by Unit *(continued)*

Country	Unit	Metric	Year	Mean	Upper	Lower
India	Kasganj	Proportion	2017	43.7	63.7	28.6
India	Kasganj	Count	2000	0	0	0
India	Kasganj	Count	2017	0	1	0
India	Sidhi	Proportion	2000	37.0	54.1	22.2
India	Sidhi	Proportion	2017	57.8	72.4	37.9
India	Sidhi	Count	2000	0	1	0
India	Sidhi	Count	2017	1	1	0
India	Kaushambi	Proportion	2000	28.4	43.0	15.6
India	Kaushambi	Proportion	2017	51.6	68.0	33.8
India	Kaushambi	Count	2000	0	0	0
India	Kaushambi	Count	2017	1	1	0
India	Singrauli	Proportion	2000	38.7	53.9	23.0
India	Singrauli	Proportion	2017	60.5	74.2	45.5
India	Singrauli	Count	2000	0	1	0
India	Singrauli	Count	2017	1	1	0
India	Kushinagar	Proportion	2000	25.4	40.4	14.1
India	Kushinagar	Proportion	2017	44.3	63.3	28.9
India	Kushinagar	Count	2000	0	0	0
India	Kushinagar	Count	2017	0	1	0
India	Tikamgarh	Proportion	2000	33.8	51.0	20.0
India	Tikamgarh	Proportion	2017	56.5	71.8	40.5
India	Tikamgarh	Count	2000	0	1	0
India	Tikamgarh	Count	2017	1	1	0
India	Lakhimpur Kheri	Proportion	2000	24.1	38.2	12.8
India	Lakhimpur Kheri	Proportion	2017	46.2	64.1	31.3
India	Lakhimpur Kheri	Count	2000	0	0	0
India	Lakhimpur Kheri	Count	2017	0	1	0
India	Ujjain	Proportion	2000	38.5	56.7	23.5
India	Ujjain	Proportion	2017	57.9	73.9	39.7
India	Ujjain	Count	2000	0	1	0
India	Ujjain	Count	2017	1	1	0
India	Lalitpur	Proportion	2000	29.5	44.0	18.2
India	Lalitpur	Proportion	2017	51.4	67.0	35.0
India	Lalitpur	Count	2000	0	0	0
India	Lalitpur	Count	2017	1	1	0
India	Umaria	Proportion	2000	38.2	56.8	22.8
India	Umaria	Proportion	2017	58.8	73.6	42.3
India	Umaria	Count	2000	0	1	0
India	Umaria	Count	2017	1	1	0
India	Lucknow	Proportion	2000	28.8	45.6	14.5
India	Lucknow	Proportion	2017	53.0	71.9	31.4
India	Lucknow	Count	2000	0	0	0
India	Lucknow	Count	2017	1	1	0
India	Vidisha	Proportion	2000	36.0	56.1	21.4
India	Vidisha	Proportion	2017	56.7	72.9	38.1
India	Vidisha	Count	2000	0	1	0
India	Vidisha	Count	2017	1	1	0
India	Maharajganj	Proportion	2000	25.0	39.4	13.3
India	Maharajganj	Proportion	2017	44.3	59.8	30.6

Table 4: ORS by Unit *(continued)*

Country	Unit	Metric	Year	Mean	Upper	Lower
India	Maharajganj	Count	2000	0	0	0
India	Maharajganj	Count	2017	0	1	0
India	Khargone	Proportion	2000	38.1	55.2	23.4
India	Khargone	Proportion	2017	54.6	70.8	36.3
India	Khargone	Count	2000	0	1	0
India	Khargone	Count	2017	1	1	0
India	Mahoba	Proportion	2000	26.9	42.2	15.3
India	Mahoba	Proportion	2017	49.4	66.7	34.4
India	Mahoba	Count	2000	0	0	0
India	Mahoba	Count	2017	0	1	0
India	Niwari	Proportion	2000	35.0	53.1	20.1
India	Niwari	Proportion	2017	57.0	72.0	41.6
India	Niwari	Count	2000	0	1	0
India	Niwari	Count	2017	1	1	0
India	Mainpuri	Proportion	2000	24.5	42.3	12.9
India	Mainpuri	Proportion	2017	45.3	63.0	28.7
India	Mainpuri	Count	2000	0	0	0
India	Mainpuri	Count	2017	0	1	0
India	Mathura	Proportion	2000	25.1	38.6	14.0
India	Mathura	Proportion	2017	45.2	65.5	27.0
India	Mathura	Count	2000	0	0	0
India	Mathura	Count	2017	0	1	0
India	Mau	Proportion	2000	26.4	42.0	13.9
India	Mau	Proportion	2017	47.3	65.8	30.5
India	Mau	Count	2000	0	0	0
India	Mau	Count	2017	0	1	0
India	Meerut	Proportion	2000	28.3	44.8	14.9
India	Meerut	Proportion	2017	49.4	67.2	31.7
India	Meerut	Count	2000	0	0	0
India	Meerut	Count	2017	0	1	0
India	Mirzapur	Proportion	2000	28.4	42.9	16.1
India	Mirzapur	Proportion	2017	50.7	65.9	36.4
India	Mirzapur	Count	2000	0	0	0
India	Mirzapur	Count	2017	1	1	0
India	Moradabad	Proportion	2000	25.2	42.3	13.4
India	Moradabad	Proportion	2017	45.7	62.8	29.0
India	Moradabad	Count	2000	0	0	0
India	Moradabad	Count	2017	0	1	0
India	Muzaffarnagar	Proportion	2000	27.3	44.4	14.8
India	Muzaffarnagar	Proportion	2017	48.6	65.6	29.9
India	Muzaffarnagar	Count	2000	0	0	0
India	Muzaffarnagar	Count	2017	0	1	0
India	Pilibhit	Proportion	2000	24.9	39.8	14.2
India	Pilibhit	Proportion	2017	47.2	62.6	31.6
India	Pilibhit	Count	2000	0	0	0
India	Pilibhit	Count	2017	0	1	0
India	Pratapgarh	Proportion	2000	25.3	39.7	13.4
India	Pratapgarh	Proportion	2017	47.9	63.9	31.5
India	Pratapgarh	Count	2000	0	0	0

Table 4: ORS by Unit *(continued)*

Country	Unit	Metric	Year	Mean	Upper	Lower
India	Pratapgarh	Count	2017	0	1	0
India	Rae Bareli	Proportion	2000	26.3	40.3	13.7
India	Rae Bareli	Proportion	2017	48.9	65.8	31.0
India	Rae Bareli	Count	2000	0	0	0
India	Rae Bareli	Count	2017	0	1	0
India	Rampur	Proportion	2000	25.6	41.9	13.5
India	Rampur	Proportion	2017	46.9	63.1	31.1
India	Rampur	Count	2000	0	0	0
India	Rampur	Count	2017	0	1	0
India	Saharanpur	Proportion	2000	30.4	49.1	17.2
India	Saharanpur	Proportion	2017	52.7	67.8	34.7
India	Saharanpur	Count	2000	0	0	0
India	Saharanpur	Count	2017	1	1	0
India	Sambhal	Proportion	2000	23.8	41.9	13.3
India	Sambhal	Proportion	2017	44.5	60.1	28.6
India	Sambhal	Count	2000	0	0	0
India	Sambhal	Count	2017	0	1	0
India	Sant Kabir Nagar	Proportion	2000	23.7	40.2	11.9
India	Sant Kabir Nagar	Proportion	2017	44.0	62.9	28.1
India	Sant Kabir Nagar	Count	2000	0	0	0
India	Sant Kabir Nagar	Count	2017	0	1	0
India	Sant Ravi Das Nagar	Proportion	2000	26.0	40.7	12.3
India	Sant Ravi Das Nagar	Proportion	2017	48.3	64.6	29.7
India	Sant Ravi Das Nagar	Count	2000	0	0	0
India	Sant Ravi Das Nagar	Count	2017	0	1	0
India	Shahjahanpur	Proportion	2000	23.9	39.0	13.2
India	Shahjahanpur	Proportion	2017	45.0	61.1	30.7
India	Shahjahanpur	Count	2000	0	0	0
India	Shahjahanpur	Count	2017	0	1	0
India	Shamli	Proportion	2000	26.8	44.1	15.5
India	Shamli	Proportion	2017	48.6	67.1	28.8
India	Shamli	Count	2000	0	0	0
India	Shamli	Count	2017	0	1	0
India	Shravasti	Proportion	2000	25.0	42.3	13.4
India	Shravasti	Proportion	2017	45.5	61.9	27.5
India	Shravasti	Count	2000	0	0	0
India	Shravasti	Count	2017	0	1	0
India	Siddharth Nagar	Proportion	2000	24.4	40.2	13.5
India	Siddharth Nagar	Proportion	2017	44.4	59.0	28.8
India	Siddharth Nagar	Count	2000	0	0	0
India	Siddharth Nagar	Count	2017	0	1	0
India	Sitapur	Proportion	2000	23.8	39.0	12.5
India	Sitapur	Proportion	2017	44.7	65.0	26.3
India	Sitapur	Count	2000	0	0	0
India	Sitapur	Count	2017	0	1	0
India	Sonbhadra	Proportion	2000	32.1	46.6	19.6
India	Sonbhadra	Proportion	2017	53.9	71.2	38.1
India	Sonbhadra	Count	2000	0	0	0
India	Sonbhadra	Count	2017	1	1	0

Table 4: ORS by Unit *(continued)*

Country	Unit	Metric	Year	Mean	Upper	Lower
India	Sultanpur	Proportion	2000	25.4	40.2	13.1
India	Sultanpur	Proportion	2017	47.6	64.3	32.5
India	Sultanpur	Count	2000	0	0	0
India	Sultanpur	Count	2017	0	1	0
India	Unnao	Proportion	2000	25.6	40.9	13.1
India	Unnao	Proportion	2017	47.6	65.7	29.0
India	Unnao	Count	2000	0	0	0
India	Unnao	Count	2017	0	1	0
India	Varanasi	Proportion	2000	29.4	44.7	15.8
India	Varanasi	Proportion	2017	51.8	69.9	36.0
India	Varanasi	Count	2000	0	0	0
India	Varanasi	Count	2017	1	1	0
Indonesia	Aceh Barat Daya	Proportion	2000	33.1	42.6	23.9
Indonesia	Aceh Barat Daya	Proportion	2017	30.5	38.1	24.2
Indonesia	Aceh Barat Daya	Count	2000	0	0	0
Indonesia	Aceh Barat Daya	Count	2017	0	0	0
Indonesia	Badung	Proportion	2000	44.8	54.3	36.2
Indonesia	Badung	Proportion	2017	44.7	52.8	37.6
Indonesia	Badung	Count	2000	0	1	0
Indonesia	Badung	Count	2017	0	1	0
Indonesia	Bangka Barat	Proportion	2000	43.9	53.0	34.7
Indonesia	Bangka Barat	Proportion	2017	42.7	50.5	35.2
Indonesia	Bangka Barat	Count	2000	0	1	0
Indonesia	Bangka Barat	Count	2017	0	1	0
Indonesia	Cilegon	Proportion	2000	36.7	43.3	29.9
Indonesia	Cilegon	Proportion	2017	41.4	47.0	35.3
Indonesia	Cilegon	Count	2000	0	0	0
Indonesia	Cilegon	Count	2017	0	0	0
Indonesia	Bengkulu Selatan	Proportion	2000	39.2	48.2	30.7
Indonesia	Bengkulu Selatan	Proportion	2017	38.4	44.9	31.0
Indonesia	Bengkulu Selatan	Count	2000	0	0	0
Indonesia	Bengkulu Selatan	Count	2017	0	0	0
Indonesia	Boalemo	Proportion	2000	44.1	53.4	35.6
Indonesia	Boalemo	Proportion	2017	39.4	47.2	31.9
Indonesia	Boalemo	Count	2000	0	1	0
Indonesia	Boalemo	Count	2017	0	0	0
Indonesia	Jakarta Barat	Proportion	2000	38.6	45.0	32.2
Indonesia	Jakarta Barat	Proportion	2017	41.2	46.9	35.5
Indonesia	Jakarta Barat	Count	2000	0	0	0
Indonesia	Jakarta Barat	Count	2017	0	0	0
Indonesia	Batang Hari	Proportion	2000	46.9	55.9	37.6
Indonesia	Batang Hari	Proportion	2017	39.7	47.5	32.7
Indonesia	Batang Hari	Count	2000	0	1	0
Indonesia	Batang Hari	Count	2017	0	0	0
Indonesia	Bandung Barat	Proportion	2000	35.9	42.7	30.2
Indonesia	Bandung Barat	Proportion	2017	40.9	46.9	35.8
Indonesia	Bandung Barat	Count	2000	0	0	0
Indonesia	Bandung Barat	Count	2017	0	0	0
Indonesia	Banjarnegara	Proportion	2000	35.4	43.2	28.7

Table 4: ORS by Unit *(continued)*

Country	Unit	Metric	Year	Mean	Upper	Lower
Indonesia	Banjarnegara	Proportion	2017	31.7	37.3	26.5
Indonesia	Banjarnegara	Count	2000	0	0	0
Indonesia	Banjarnegara	Count	2017	0	0	0
Indonesia	Bangkalan	Proportion	2000	39.8	49.1	30.8
Indonesia	Bangkalan	Proportion	2017	35.3	41.4	29.2
Indonesia	Bangkalan	Count	2000	0	0	0
Indonesia	Bangkalan	Count	2017	0	0	0
Indonesia	Bengkayang	Proportion	2000	41.9	51.2	32.7
Indonesia	Bengkayang	Proportion	2017	38.7	47.6	31.1
Indonesia	Bengkayang	Count	2000	0	1	0
Indonesia	Bengkayang	Count	2017	0	0	0
Indonesia	Balangan	Proportion	2000	36.9	45.5	28.7
Indonesia	Balangan	Proportion	2017	30.8	37.9	24.3
Indonesia	Balangan	Count	2000	0	0	0
Indonesia	Balangan	Count	2017	0	0	0
Indonesia	Barito Selatan	Proportion	2000	40.3	49.2	31.6
Indonesia	Barito Selatan	Proportion	2017	31.2	38.2	25.3
Indonesia	Barito Selatan	Count	2000	0	0	0
Indonesia	Barito Selatan	Count	2017	0	0	0
Indonesia	Balikpapan	Proportion	2000	42.7	51.6	33.1
Indonesia	Balikpapan	Proportion	2017	34.0	41.0	26.4
Indonesia	Balikpapan	Count	2000	0	1	0
Indonesia	Balikpapan	Count	2017	0	0	0
Indonesia	Batam	Proportion	2000	43.8	54.5	34.2
Indonesia	Batam	Proportion	2017	43.8	52.0	35.4
Indonesia	Batam	Count	2000	0	1	0
Indonesia	Batam	Count	2017	0	1	0
Indonesia	Bandar Lampung	Proportion	2000	37.9	45.2	30.6
Indonesia	Bandar Lampung	Proportion	2017	40.6	46.7	33.7
Indonesia	Bandar Lampung	Count	2000	0	0	0
Indonesia	Bandar Lampung	Count	2017	0	0	0
Indonesia	Halmahera Barat	Proportion	2000	48.3	61.1	37.1
Indonesia	Halmahera Barat	Proportion	2017	46.4	54.3	38.1
Indonesia	Halmahera Barat	Count	2000	0	1	0
Indonesia	Halmahera Barat	Count	2017	0	1	0
Indonesia	Ambon	Proportion	2000	37.9	49.3	26.9
Indonesia	Ambon	Proportion	2017	39.7	47.7	32.3
Indonesia	Ambon	Count	2000	0	0	0
Indonesia	Ambon	Count	2017	0	0	0
Indonesia	Bima	Proportion	2000	47.7	56.0	38.1
Indonesia	Bima	Proportion	2017	46.4	53.8	39.2
Indonesia	Bima	Count	2000	0	1	0
Indonesia	Bima	Count	2017	0	1	0
Indonesia	Alor	Proportion	2000	53.1	63.0	43.8
Indonesia	Alor	Proportion	2017	47.2	54.8	39.1
Indonesia	Alor	Count	2000	1	1	0
Indonesia	Alor	Count	2017	0	1	0
Indonesia	Fakfak	Proportion	2000	48.3	61.3	35.9
Indonesia	Fakfak	Proportion	2017	48.7	58.4	38.9

Table 4: ORS by Unit *(continued)*

Country	Unit	Metric	Year	Mean	Upper	Lower
Indonesia	Fakfak	Count	2000	0	1	0
Indonesia	Fakfak	Count	2017	0	1	0
Indonesia	Asmat	Proportion	2000	49.7	59.1	40.0
Indonesia	Asmat	Proportion	2017	46.7	56.5	36.4
Indonesia	Asmat	Count	2000	0	1	0
Indonesia	Asmat	Count	2017	0	1	0
Indonesia	Bengkalis	Proportion	2000	37.6	46.9	30.2
Indonesia	Bengkalis	Proportion	2017	36.2	43.3	29.4
Indonesia	Bengkalis	Count	2000	0	0	0
Indonesia	Bengkalis	Count	2017	0	0	0
Indonesia	Majene	Proportion	2000	43.1	51.8	35.7
Indonesia	Majene	Proportion	2017	30.2	36.2	24.0
Indonesia	Majene	Count	2000	0	1	0
Indonesia	Majene	Count	2017	0	0	0
Indonesia	Bantaeng	Proportion	2000	38.8	47.3	31.1
Indonesia	Bantaeng	Proportion	2017	28.7	35.0	23.0
Indonesia	Bantaeng	Count	2000	0	0	0
Indonesia	Bantaeng	Count	2017	0	0	0
Indonesia	Banggai Kepulauan	Proportion	2000	40.4	48.5	31.9
Indonesia	Banggai Kepulauan	Proportion	2017	35.1	43.6	27.6
Indonesia	Banggai Kepulauan	Count	2000	0	0	0
Indonesia	Banggai Kepulauan	Count	2017	0	0	0
Indonesia	Bau-Bau	Proportion	2000	40.9	49.7	32.8
Indonesia	Bau-Bau	Proportion	2017	31.2	37.9	24.6
Indonesia	Bau-Bau	Count	2000	0	0	0
Indonesia	Bau-Bau	Count	2017	0	0	0
Indonesia	Bitung	Proportion	2000	35.7	47.3	26.8
Indonesia	Bitung	Proportion	2017	40.1	48.3	31.3
Indonesia	Bitung	Count	2000	0	0	0
Indonesia	Bitung	Count	2017	0	0	0
Indonesia	Agam	Proportion	2000	33.9	41.3	27.7
Indonesia	Agam	Proportion	2017	30.6	36.6	24.8
Indonesia	Agam	Count	2000	0	0	0
Indonesia	Agam	Count	2017	0	0	0
Indonesia	Banyu Asin	Proportion	2000	43.8	53.2	35.4
Indonesia	Banyu Asin	Proportion	2017	42.0	49.4	35.7
Indonesia	Banyu Asin	Count	2000	0	1	0
Indonesia	Banyu Asin	Count	2017	0	0	0
Indonesia	Asahan	Proportion	2000	31.1	39.4	25.2
Indonesia	Asahan	Proportion	2017	28.7	34.5	22.8
Indonesia	Asahan	Count	2000	0	0	0
Indonesia	Asahan	Count	2017	0	0	0
Indonesia	Bantul	Proportion	2000	37.4	45.1	30.4
Indonesia	Bantul	Proportion	2017	31.3	37.4	25.1
Indonesia	Bantul	Count	2000	0	0	0
Indonesia	Bantul	Count	2017	0	0	0
Indonesia	Bulungan	Proportion	2000	45.6	56.3	35.7
Indonesia	Bulungan	Proportion	2017	38.5	47.9	29.4
Indonesia	Bulungan	Count	2000	0	1	0

Table 4: ORS by Unit *(continued)*

Country	Unit	Metric	Year	Mean	Upper	Lower
Indonesia	Bulungan	Count	2017	0	0	0
Indonesia	Aceh Barat	Proportion	2000	32.9	43.3	24.4
Indonesia	Aceh Barat	Proportion	2017	29.8	37.3	23.4
Indonesia	Aceh Barat	Count	2000	0	0	0
Indonesia	Aceh Barat	Count	2017	0	0	0
Indonesia	Bangli	Proportion	2000	43.8	53.2	35.7
Indonesia	Bangli	Proportion	2017	43.2	51.7	36.9
Indonesia	Bangli	Count	2000	0	1	0
Indonesia	Bangli	Count	2017	0	1	0
Indonesia	Bangka Selatan	Proportion	2000	44.1	52.1	36.8
Indonesia	Bangka Selatan	Proportion	2017	43.7	52.6	36.0
Indonesia	Bangka Selatan	Count	2000	0	1	0
Indonesia	Bangka Selatan	Count	2017	0	1	0
Indonesia	Kota Serang	Proportion	2000	37.6	44.5	31.0
Indonesia	Kota Serang	Proportion	2017	42.7	48.1	36.5
Indonesia	Kota Serang	Count	2000	0	0	0
Indonesia	Kota Serang	Count	2017	0	0	0
Indonesia	Bengkulu Tengah	Proportion	2000	39.1	47.8	31.2
Indonesia	Bengkulu Tengah	Proportion	2017	36.9	44.7	30.0
Indonesia	Bengkulu Tengah	Count	2000	0	0	0
Indonesia	Bengkulu Tengah	Count	2017	0	0	0
Indonesia	Bone Bolango	Proportion	2000	42.8	52.2	33.3
Indonesia	Bone Bolango	Proportion	2017	39.6	48.3	32.2
Indonesia	Bone Bolango	Count	2000	0	1	0
Indonesia	Bone Bolango	Count	2017	0	0	0
Indonesia	Jakarta Pusat	Proportion	2000	40.0	46.6	33.2
Indonesia	Jakarta Pusat	Proportion	2017	41.5	47.3	35.7
Indonesia	Jakarta Pusat	Count	2000	0	0	0
Indonesia	Jakarta Pusat	Count	2017	0	0	0
Indonesia	Bungo	Proportion	2000	42.3	51.3	33.5
Indonesia	Bungo	Proportion	2017	36.3	43.3	30.1
Indonesia	Bungo	Count	2000	0	1	0
Indonesia	Bungo	Count	2017	0	0	0
Indonesia	Bandung	Proportion	2000	36.1	42.7	29.8
Indonesia	Bandung	Proportion	2017	40.7	46.3	35.5
Indonesia	Bandung	Count	2000	0	0	0
Indonesia	Bandung	Count	2017	0	0	0
Indonesia	Banyumas	Proportion	2000	35.7	43.6	28.9
Indonesia	Banyumas	Proportion	2017	32.1	37.6	26.9
Indonesia	Banyumas	Count	2000	0	0	0
Indonesia	Banyumas	Count	2017	0	0	0
Indonesia	Banyuwangi	Proportion	2000	38.8	47.5	30.5
Indonesia	Banyuwangi	Proportion	2017	36.7	44.1	30.6
Indonesia	Banyuwangi	Count	2000	0	0	0
Indonesia	Banyuwangi	Count	2017	0	0	0
Indonesia	Kapuas Hulu	Proportion	2000	42.4	51.5	33.0
Indonesia	Kapuas Hulu	Proportion	2017	36.2	45.0	27.6
Indonesia	Kapuas Hulu	Count	2000	0	1	0
Indonesia	Kapuas Hulu	Count	2017	0	0	0

Table 4: ORS by Unit *(continued)*

Country	Unit	Metric	Year	Mean	Upper	Lower
Indonesia	Banjar Baru	Proportion	2000	34.7	43.3	26.5
Indonesia	Banjar Baru	Proportion	2017	29.5	36.3	23.4
Indonesia	Banjar Baru	Count	2000	0	0	0
Indonesia	Banjar Baru	Count	2017	0	0	0
Indonesia	Barito Timur	Proportion	2000	38.2	47.7	29.4
Indonesia	Barito Timur	Proportion	2017	30.2	37.0	24.3
Indonesia	Barito Timur	Count	2000	0	0	0
Indonesia	Barito Timur	Count	2017	0	0	0
Indonesia	Berau	Proportion	2000	45.8	55.8	36.6
Indonesia	Berau	Proportion	2017	37.8	46.9	29.2
Indonesia	Berau	Count	2000	0	1	0
Indonesia	Berau	Count	2017	0	0	0
Indonesia	Bintan	Proportion	2000	44.3	54.7	35.2
Indonesia	Bintan	Proportion	2017	44.6	52.6	36.1
Indonesia	Bintan	Count	2000	0	1	0
Indonesia	Bintan	Count	2017	0	1	0
Indonesia	Lampung Barat	Proportion	2000	36.5	44.8	28.2
Indonesia	Lampung Barat	Proportion	2017	38.9	46.3	32.5
Indonesia	Lampung Barat	Count	2000	0	0	0
Indonesia	Lampung Barat	Count	2017	0	0	0
Indonesia	Halmahera Selatan	Proportion	2000	40.8	52.6	30.5
Indonesia	Halmahera Selatan	Proportion	2017	41.8	50.4	33.9
Indonesia	Halmahera Selatan	Count	2000	0	1	0
Indonesia	Halmahera Selatan	Count	2017	0	1	0
Indonesia	Buru Selatan	Proportion	2000	40.8	52.1	29.9
Indonesia	Buru Selatan	Proportion	2017	39.3	49.0	30.8
Indonesia	Buru Selatan	Count	2000	0	1	0
Indonesia	Buru Selatan	Count	2017	0	0	0
Indonesia	Dompu	Proportion	2000	49.2	58.3	39.1
Indonesia	Dompu	Proportion	2017	47.3	54.3	40.1
Indonesia	Dompu	Count	2000	0	1	0
Indonesia	Dompu	Count	2017	0	1	0
Indonesia	Belu	Proportion	2000	55.4	64.8	47.5
Indonesia	Belu	Proportion	2017	52.2	59.2	44.6
Indonesia	Belu	Count	2000	1	1	0
Indonesia	Belu	Count	2017	1	1	0
Indonesia	Kaimana	Proportion	2000	46.4	59.0	35.4
Indonesia	Kaimana	Proportion	2017	46.0	55.5	37.7
Indonesia	Kaimana	Count	2000	0	1	0
Indonesia	Kaimana	Count	2017	0	1	0
Indonesia	Biak Numfor	Proportion	2000	47.2	56.7	36.0
Indonesia	Biak Numfor	Proportion	2017	44.8	53.9	35.2
Indonesia	Biak Numfor	Count	2000	0	1	0
Indonesia	Biak Numfor	Count	2017	0	1	0
Indonesia	Dumai	Proportion	2000	36.6	46.1	28.5
Indonesia	Dumai	Proportion	2017	35.0	41.7	28.6
Indonesia	Dumai	Count	2000	0	0	0
Indonesia	Dumai	Count	2017	0	0	0
Indonesia	Mamasa	Proportion	2000	43.7	53.6	35.4

Table 4: ORS by Unit *(continued)*

Country	Unit	Metric	Year	Mean	Upper	Lower
Indonesia	Mamasa	Proportion	2017	30.7	36.6	25.4
Indonesia	Mamasa	Count	2000	0	1	0
Indonesia	Mamasa	Count	2017	0	0	0
Indonesia	Barru	Proportion	2000	37.4	46.2	29.7
Indonesia	Barru	Proportion	2017	26.1	31.3	21.2
Indonesia	Barru	Count	2000	0	0	0
Indonesia	Barru	Count	2017	0	0	0
Indonesia	Banggai	Proportion	2000	40.6	48.7	32.3
Indonesia	Banggai	Proportion	2017	33.8	41.8	27.4
Indonesia	Banggai	Count	2000	0	0	0
Indonesia	Banggai	Count	2017	0	0	0
Indonesia	Bombana	Proportion	2000	40.7	49.0	33.4
Indonesia	Bombana	Proportion	2017	29.1	35.5	22.5
Indonesia	Bombana	Count	2000	0	0	0
Indonesia	Bombana	Count	2017	0	0	0
Indonesia	Bolaang Mongondow Selatan	Proportion	2000	40.8	51.1	31.2
Indonesia	Bolaang Mongondow Selatan	Proportion	2017	39.5	47.1	32.0
Indonesia	Bolaang Mongondow Selatan	Count	2000	0	1	0
Indonesia	Bolaang Mongondow Selatan	Count	2017	0	0	0
Indonesia	Bukittinggi	Proportion	2000	34.2	41.7	28.1
Indonesia	Bukittinggi	Proportion	2017	29.9	35.6	23.7
Indonesia	Bukittinggi	Count	2000	0	0	0
Indonesia	Bukittinggi	Count	2017	0	0	0
Indonesia	Empat Lawang	Proportion	2000	40.6	49.3	32.3
Indonesia	Empat Lawang	Proportion	2017	37.9	44.6	31.5
Indonesia	Empat Lawang	Count	2000	0	0	0
Indonesia	Empat Lawang	Count	2017	0	0	0
Indonesia	Batu Bara	Proportion	2000	29.4	36.8	23.3
Indonesia	Batu Bara	Proportion	2017	27.9	33.8	22.6
Indonesia	Batu Bara	Count	2000	0	0	0
Indonesia	Batu Bara	Count	2017	0	0	0
Indonesia	Gunung Kidul	Proportion	2000	36.8	44.8	29.9
Indonesia	Gunung Kidul	Proportion	2017	30.9	37.4	25.2
Indonesia	Gunung Kidul	Count	2000	0	0	0
Indonesia	Gunung Kidul	Count	2017	0	0	0
Indonesia	Malinau	Proportion	2000	44.9	54.0	35.9
Indonesia	Malinau	Proportion	2017	39.3	49.7	30.3
Indonesia	Malinau	Count	2000	0	1	0
Indonesia	Malinau	Count	2017	0	0	0
Indonesia	Aceh Besar	Proportion	2000	36.1	47.9	27.6
Indonesia	Aceh Besar	Proportion	2017	30.9	38.1	23.7
Indonesia	Aceh Besar	Count	2000	0	0	0
Indonesia	Aceh Besar	Count	2017	0	0	0
Indonesia	Buleleng	Proportion	2000	40.2	49.6	32.0
Indonesia	Buleleng	Proportion	2017	39.5	47.3	33.2
Indonesia	Buleleng	Count	2000	0	0	0

Table 4: ORS by Unit *(continued)*

Country	Unit	Metric	Year	Mean	Upper	Lower
Indonesia	Buleleng	Count	2017	0	0	0
Indonesia	Bangka Tengah	Proportion	2000	43.7	52.1	36.2
Indonesia	Bangka Tengah	Proportion	2017	43.0	51.3	35.8
Indonesia	Bangka Tengah	Count	2000	0	1	0
Indonesia	Bangka Tengah	Count	2017	0	1	0
Indonesia	Kota Tangerang	Proportion	2000	37.3	43.7	31.1
Indonesia	Kota Tangerang	Proportion	2017	38.9	44.2	33.3
Indonesia	Kota Tangerang	Count	2000	0	0	0
Indonesia	Kota Tangerang	Count	2017	0	0	0
Indonesia	Bengkulu Utara	Proportion	2000	39.3	48.3	31.2
Indonesia	Bengkulu Utara	Proportion	2017	36.4	44.0	30.2
Indonesia	Bengkulu Utara	Count	2000	0	0	0
Indonesia	Bengkulu Utara	Count	2017	0	0	0
Indonesia	Danau Limboto	Proportion	2000	43.7	53.4	34.1
Indonesia	Danau Limboto	Proportion	2017	39.6	47.5	32.3
Indonesia	Danau Limboto	Count	2000	0	1	0
Indonesia	Danau Limboto	Count	2017	0	0	0
Indonesia	Jakarta Selatan	Proportion	2000	38.2	44.5	32.0
Indonesia	Jakarta Selatan	Proportion	2017	40.6	46.1	34.8
Indonesia	Jakarta Selatan	Count	2000	0	0	0
Indonesia	Jakarta Selatan	Count	2017	0	0	0
Indonesia	Jambi	Proportion	2000	45.2	53.8	36.3
Indonesia	Jambi	Proportion	2017	40.7	48.0	33.5
Indonesia	Jambi	Count	2000	0	1	0
Indonesia	Jambi	Count	2017	0	0	0
Indonesia	Banjar	Proportion	2000	37.1	44.8	30.1
Indonesia	Banjar	Proportion	2017	36.8	41.9	30.6
Indonesia	Banjar	Count	2000	0	0	0
Indonesia	Banjar	Count	2017	0	0	0
Indonesia	Batang	Proportion	2000	35.2	42.4	28.6
Indonesia	Batang	Proportion	2017	30.8	36.7	25.4
Indonesia	Batang	Count	2000	0	0	0
Indonesia	Batang	Count	2017	0	0	0
Indonesia	Batu	Proportion	2000	38.6	47.4	31.2
Indonesia	Batu	Proportion	2017	33.8	40.2	27.9
Indonesia	Batu	Count	2000	0	0	0
Indonesia	Batu	Count	2017	0	0	0
Indonesia	Kayong Utara	Proportion	2000	42.8	52.4	33.3
Indonesia	Kayong Utara	Proportion	2017	38.6	47.7	30.6
Indonesia	Kayong Utara	Count	2000	0	1	0
Indonesia	Kayong Utara	Count	2017	0	0	0
Indonesia	Banjar	Proportion	2000	35.1	43.3	27.2
Indonesia	Banjar	Proportion	2017	29.3	36.3	23.7
Indonesia	Banjar	Count	2000	0	0	0
Indonesia	Banjar	Count	2017	0	0	0
Indonesia	Barito Utara	Proportion	2000	43.4	52.2	34.2
Indonesia	Barito Utara	Proportion	2017	34.1	41.8	27.5
Indonesia	Barito Utara	Count	2000	0	1	0
Indonesia	Barito Utara	Count	2017	0	0	0

Table 4: ORS by Unit *(continued)*

Country	Unit	Metric	Year	Mean	Upper	Lower
Indonesia	Bontang	Proportion	2000	42.9	52.7	34.1
Indonesia	Bontang	Proportion	2017	35.8	42.6	28.5
Indonesia	Bontang	Count	2000	0	1	0
Indonesia	Bontang	Count	2017	0	0	0
Indonesia	Karimun	Proportion	2000	42.1	52.3	33.5
Indonesia	Karimun	Proportion	2017	40.7	48.2	32.4
Indonesia	Karimun	Count	2000	0	1	0
Indonesia	Karimun	Count	2017	0	0	0
Indonesia	Lampung Selatan	Proportion	2000	37.2	44.4	30.3
Indonesia	Lampung Selatan	Proportion	2017	39.6	45.1	33.3
Indonesia	Lampung Selatan	Count	2000	0	0	0
Indonesia	Lampung Selatan	Count	2017	0	0	0
Indonesia	Halmahera Tengah	Proportion	2000	45.0	56.3	34.5
Indonesia	Halmahera Tengah	Proportion	2017	45.8	53.5	37.6
Indonesia	Halmahera Tengah	Count	2000	0	1	0
Indonesia	Halmahera Tengah	Count	2017	0	1	0
Indonesia	Buru	Proportion	2000	41.8	53.3	31.2
Indonesia	Buru	Proportion	2017	39.5	49.0	31.7
Indonesia	Buru	Count	2000	0	1	0
Indonesia	Buru	Count	2017	0	0	0
Indonesia	Kota Bima	Proportion	2000	45.7	54.6	36.5
Indonesia	Kota Bima	Proportion	2017	45.3	52.9	38.2
Indonesia	Kota Bima	Count	2000	0	1	0
Indonesia	Kota Bima	Count	2017	0	1	0
Indonesia	Ende	Proportion	2000	52.0	61.3	42.0
Indonesia	Ende	Proportion	2017	45.7	54.2	37.7
Indonesia	Ende	Count	2000	1	1	0
Indonesia	Ende	Count	2017	0	1	0
Indonesia	Kota Sorong	Proportion	2000	42.7	52.8	31.3
Indonesia	Kota Sorong	Proportion	2017	47.7	57.1	35.7
Indonesia	Kota Sorong	Count	2000	0	1	0
Indonesia	Kota Sorong	Count	2017	0	1	0
Indonesia	Boven Digoel	Proportion	2000	47.1	57.5	36.3
Indonesia	Boven Digoel	Proportion	2017	44.2	53.3	33.0
Indonesia	Boven Digoel	Count	2000	0	1	0
Indonesia	Boven Digoel	Count	2017	0	1	0
Indonesia	Indragiri Hilir	Proportion	2000	40.6	49.9	31.7
Indonesia	Indragiri Hilir	Proportion	2017	37.0	44.4	29.1
Indonesia	Indragiri Hilir	Count	2000	0	0	0
Indonesia	Indragiri Hilir	Count	2017	0	0	0
Indonesia	Mamuju Utara	Proportion	2000	44.4	53.2	35.5
Indonesia	Mamuju Utara	Proportion	2017	32.7	39.5	27.2
Indonesia	Mamuju Utara	Count	2000	0	1	0
Indonesia	Mamuju Utara	Count	2017	0	0	0
Indonesia	Bone	Proportion	2000	37.7	45.6	30.3
Indonesia	Bone	Proportion	2017	26.4	31.6	21.2
Indonesia	Bone	Count	2000	0	0	0
Indonesia	Bone	Count	2017	0	0	0
Indonesia	Buol	Proportion	2000	43.7	52.2	35.5

Table 4: ORS by Unit *(continued)*

Country	Unit	Metric	Year	Mean	Upper	Lower
Indonesia	Buol	Proportion	2017	38.3	45.7	29.7
Indonesia	Buol	Count	2000	0	1	0
Indonesia	Buol	Count	2017	0	0	0
Indonesia	Buton Utara	Proportion	2000	41.1	49.5	32.4
Indonesia	Buton Utara	Proportion	2017	31.3	38.8	24.7
Indonesia	Buton Utara	Count	2000	0	0	0
Indonesia	Buton Utara	Count	2017	0	0	0
Indonesia	Bolaang Mongondow Timur	Proportion	2000	37.6	48.1	28.8
Indonesia	Bolaang Mongondow Timur	Proportion	2017	39.5	47.6	32.1
Indonesia	Bolaang Mongondow Timur	Count	2000	0	0	0
Indonesia	Bolaang Mongondow Timur	Count	2017	0	0	0
Indonesia	Danau	Proportion	2000	36.9	45.6	29.8
Indonesia	Danau	Proportion	2017	32.2	39.3	25.4
Indonesia	Danau	Count	2000	0	0	0
Indonesia	Danau	Count	2017	0	0	0
Indonesia	Lahat	Proportion	2000	40.2	48.4	31.2
Indonesia	Lahat	Proportion	2017	38.7	44.9	32.5
Indonesia	Lahat	Count	2000	0	0	0
Indonesia	Lahat	Count	2017	0	0	0
Indonesia	Dairi	Proportion	2000	27.6	35.2	20.8
Indonesia	Dairi	Proportion	2017	26.6	31.5	21.8
Indonesia	Dairi	Count	2000	0	0	0
Indonesia	Dairi	Count	2017	0	0	0
Indonesia	Kota Yogyakarta	Proportion	2000	37.2	44.9	30.5
Indonesia	Kota Yogyakarta	Proportion	2017	30.1	36.0	23.9
Indonesia	Kota Yogyakarta	Count	2000	0	0	0
Indonesia	Kota Yogyakarta	Count	2017	0	0	0
Indonesia	Nunukan	Proportion	2000	45.0	55.2	34.2
Indonesia	Nunukan	Proportion	2017	40.9	51.2	31.5
Indonesia	Nunukan	Count	2000	0	1	0
Indonesia	Nunukan	Count	2017	0	1	0
Indonesia	Aceh Jaya	Proportion	2000	35.0	46.6	26.9
Indonesia	Aceh Jaya	Proportion	2017	31.1	39.1	24.6
Indonesia	Aceh Jaya	Count	2000	0	0	0
Indonesia	Aceh Jaya	Count	2017	0	0	0
Indonesia	Denpasar	Proportion	2000	46.9	56.5	37.9
Indonesia	Denpasar	Proportion	2017	47.0	55.0	39.3
Indonesia	Denpasar	Count	2000	0	1	0
Indonesia	Denpasar	Count	2017	0	1	0
Indonesia	Bangka	Proportion	2000	44.7	53.8	35.9
Indonesia	Bangka	Proportion	2017	42.5	51.4	35.8
Indonesia	Bangka	Count	2000	0	1	0
Indonesia	Bangka	Count	2017	0	1	0
Indonesia	Lebak	Proportion	2000	34.3	40.6	28.1
Indonesia	Lebak	Proportion	2017	39.2	44.6	34.1
Indonesia	Lebak	Count	2000	0	0	0

Table 4: ORS by Unit *(continued)*

Country	Unit	Metric	Year	Mean	Upper	Lower
Indonesia	Lebak	Count	2017	0	0	0
Indonesia	Bengkulu	Proportion	2000	39.1	47.8	30.7
Indonesia	Bengkulu	Proportion	2017	36.7	44.8	29.7
Indonesia	Bengkulu	Count	2000	0	0	0
Indonesia	Bengkulu	Count	2017	0	0	0
Indonesia	Gorontalo Utara	Proportion	2000	43.9	53.4	35.3
Indonesia	Gorontalo Utara	Proportion	2017	39.5	47.3	32.1
Indonesia	Gorontalo Utara	Count	2000	0	1	0
Indonesia	Gorontalo Utara	Count	2017	0	0	0
Indonesia	Jakarta Timur	Proportion	2000	38.2	44.4	31.4
Indonesia	Jakarta Timur	Proportion	2017	40.9	46.5	35.3
Indonesia	Jakarta Timur	Count	2000	0	0	0
Indonesia	Jakarta Timur	Count	2017	0	0	0
Indonesia	Kerinci	Proportion	2000	40.5	50.7	32.8
Indonesia	Kerinci	Proportion	2017	34.8	42.8	28.9
Indonesia	Kerinci	Count	2000	0	1	0
Indonesia	Kerinci	Count	2017	0	0	0
Indonesia	Bekasi	Proportion	2000	36.8	42.8	30.2
Indonesia	Bekasi	Proportion	2017	40.8	45.9	35.8
Indonesia	Bekasi	Count	2000	0	0	0
Indonesia	Bekasi	Count	2017	0	0	0
Indonesia	Blora	Proportion	2000	37.0	44.7	29.4
Indonesia	Blora	Proportion	2017	31.2	37.4	25.9
Indonesia	Blora	Count	2000	0	0	0
Indonesia	Blora	Count	2017	0	0	0
Indonesia	Blitar	Proportion	2000	38.7	47.2	30.9
Indonesia	Blitar	Proportion	2017	32.5	38.7	26.5
Indonesia	Blitar	Count	2000	0	0	0
Indonesia	Blitar	Count	2017	0	0	0
Indonesia	Ketapang	Proportion	2000	42.9	51.9	33.8
Indonesia	Ketapang	Proportion	2017	38.4	46.6	30.0
Indonesia	Ketapang	Count	2000	0	1	0
Indonesia	Ketapang	Count	2017	0	0	0
Indonesia	Banjarmasin	Proportion	2000	35.9	44.8	27.6
Indonesia	Banjarmasin	Proportion	2017	29.3	36.5	23.4
Indonesia	Banjarmasin	Count	2000	0	0	0
Indonesia	Banjarmasin	Count	2017	0	0	0
Indonesia	Gunung Mas	Proportion	2000	41.8	50.9	31.8
Indonesia	Gunung Mas	Proportion	2017	34.2	41.5	25.9
Indonesia	Gunung Mas	Count	2000	0	1	0
Indonesia	Gunung Mas	Count	2017	0	0	0
Indonesia	Kutai Barat	Proportion	2000	46.5	55.8	38.1
Indonesia	Kutai Barat	Proportion	2017	35.5	43.5	29.0
Indonesia	Kutai Barat	Count	2000	0	1	0
Indonesia	Kutai Barat	Count	2017	0	0	0
Indonesia	Kepulauan Anambas	Proportion	2000	45.1	55.5	35.1
Indonesia	Kepulauan Anambas	Proportion	2017	43.8	55.2	34.7
Indonesia	Kepulauan Anambas	Count	2000	0	1	0
Indonesia	Kepulauan Anambas	Count	2017	0	1	0

Table 4: ORS by Unit *(continued)*

Country	Unit	Metric	Year	Mean	Upper	Lower
Indonesia	Lampung Tengah	Proportion	2000	37.6	44.7	30.7
Indonesia	Lampung Tengah	Proportion	2017	39.6	46.6	33.4
Indonesia	Lampung Tengah	Count	2000	0	0	0
Indonesia	Lampung Tengah	Count	2017	0	0	0
Indonesia	Halmahera Timur	Proportion	2000	50.8	63.0	39.6
Indonesia	Halmahera Timur	Proportion	2017	47.6	55.7	38.4
Indonesia	Halmahera Timur	Count	2000	1	1	0
Indonesia	Halmahera Timur	Count	2017	0	1	0
Indonesia	Kepulauan Aru	Proportion	2000	44.5	58.3	34.4
Indonesia	Kepulauan Aru	Proportion	2017	44.5	56.0	34.6
Indonesia	Kepulauan Aru	Count	2000	0	1	0
Indonesia	Kepulauan Aru	Count	2017	0	1	0
Indonesia	Lombok Barat	Proportion	2000	53.5	61.3	44.8
Indonesia	Lombok Barat	Proportion	2017	51.1	58.8	44.0
Indonesia	Lombok Barat	Count	2000	1	1	0
Indonesia	Lombok Barat	Count	2017	1	1	0
Indonesia	Flores Timur	Proportion	2000	49.8	59.9	40.8
Indonesia	Flores Timur	Proportion	2017	44.4	52.8	36.4
Indonesia	Flores Timur	Count	2000	0	1	0
Indonesia	Flores Timur	Count	2017	0	1	0
Indonesia	Manokwari	Proportion	2000	45.5	57.0	34.5
Indonesia	Manokwari	Proportion	2017	46.2	54.7	37.6
Indonesia	Manokwari	Count	2000	0	1	0
Indonesia	Manokwari	Count	2017	0	1	0
Indonesia	Deiyai	Proportion	2000	46.0	56.0	35.2
Indonesia	Deiyai	Proportion	2017	44.7	55.1	35.5
Indonesia	Deiyai	Count	2000	0	1	0
Indonesia	Deiyai	Count	2017	0	1	0
Indonesia	Indragiri Hulu	Proportion	2000	41.5	50.0	32.9
Indonesia	Indragiri Hulu	Proportion	2017	35.4	42.4	27.6
Indonesia	Indragiri Hulu	Count	2000	0	0	0
Indonesia	Indragiri Hulu	Count	2017	0	0	0
Indonesia	Mamuju	Proportion	2000	44.2	53.0	36.1
Indonesia	Mamuju	Proportion	2017	31.5	37.5	26.1
Indonesia	Mamuju	Count	2000	0	1	0
Indonesia	Mamuju	Count	2017	0	0	0
Indonesia	Bulukumba	Proportion	2000	38.4	46.7	30.8
Indonesia	Bulukumba	Proportion	2017	28.4	34.7	22.6
Indonesia	Bulukumba	Count	2000	0	0	0
Indonesia	Bulukumba	Count	2017	0	0	0
Indonesia	Donggala	Proportion	2000	42.1	51.3	32.9
Indonesia	Donggala	Proportion	2017	34.3	41.7	28.3
Indonesia	Donggala	Count	2000	0	1	0
Indonesia	Donggala	Count	2017	0	0	0
Indonesia	Buton	Proportion	2000	41.1	49.7	33.4
Indonesia	Buton	Proportion	2017	31.4	38.1	25.3
Indonesia	Buton	Count	2000	0	0	0
Indonesia	Buton	Count	2017	0	0	0
Indonesia	Bolaang Mongondow Utara	Proportion	2000	41.3	50.8	32.6

Table 4: ORS by Unit *(continued)*

Country	Unit	Metric	Year	Mean	Upper	Lower
Indonesia	Bolaang Mongondow Utara	Proportion	2017	39.5	47.8	32.0
Indonesia	Bolaang Mongondow Utara	Count	2000	0	1	0
Indonesia	Bolaang Mongondow Utara	Count	2017	0	0	0
Indonesia	Dharmasraya	Proportion	2000	38.9	47.9	30.9
Indonesia	Dharmasraya	Proportion	2017	32.9	39.5	26.5
Indonesia	Dharmasraya	Count	2000	0	0	0
Indonesia	Dharmasraya	Count	2017	0	0	0
Indonesia	Lubuklinggau	Proportion	2000	39.4	48.2	31.8
Indonesia	Lubuklinggau	Proportion	2017	37.4	45.6	31.1
Indonesia	Lubuklinggau	Count	2000	0	0	0
Indonesia	Lubuklinggau	Count	2017	0	0	0
Indonesia	Deli Serdang	Proportion	2000	30.5	38.0	23.3
Indonesia	Deli Serdang	Proportion	2017	28.0	33.0	23.7
Indonesia	Deli Serdang	Count	2000	0	0	0
Indonesia	Deli Serdang	Count	2017	0	0	0
Indonesia	Kulon Progo	Proportion	2000	36.7	44.7	29.3
Indonesia	Kulon Progo	Proportion	2017	31.0	37.2	25.3
Indonesia	Kulon Progo	Count	2000	0	0	0
Indonesia	Kulon Progo	Count	2017	0	0	0
Indonesia	Tana Tidung	Proportion	2000	45.8	57.0	35.0
Indonesia	Tana Tidung	Proportion	2017	40.0	50.5	30.3
Indonesia	Tana Tidung	Count	2000	0	1	0
Indonesia	Tana Tidung	Count	2017	0	1	0
Indonesia	Aceh Selatan	Proportion	2000	31.4	39.9	23.4
Indonesia	Aceh Selatan	Proportion	2017	29.8	36.1	23.8
Indonesia	Aceh Selatan	Count	2000	0	0	0
Indonesia	Aceh Selatan	Count	2017	0	0	0
Indonesia	Gianyar	Proportion	2000	45.5	55.3	37.3
Indonesia	Gianyar	Proportion	2017	45.8	53.9	38.4
Indonesia	Gianyar	Count	2000	0	1	0
Indonesia	Gianyar	Count	2017	0	1	0
Indonesia	Belitung Timur	Proportion	2000	46.5	55.2	37.5
Indonesia	Belitung Timur	Proportion	2017	42.2	52.0	33.1
Indonesia	Belitung Timur	Count	2000	0	1	0
Indonesia	Belitung Timur	Count	2017	0	1	0
Indonesia	Pandeglang	Proportion	2000	34.5	41.3	27.5
Indonesia	Pandeglang	Proportion	2017	39.7	45.3	34.1
Indonesia	Pandeglang	Count	2000	0	0	0
Indonesia	Pandeglang	Count	2017	0	0	0
Indonesia	Kaur	Proportion	2000	38.4	47.6	29.5
Indonesia	Kaur	Proportion	2017	38.4	44.9	31.5
Indonesia	Kaur	Count	2000	0	0	0
Indonesia	Kaur	Count	2017	0	0	0
Indonesia	Gorontalo	Proportion	2000	43.6	53.4	34.7
Indonesia	Gorontalo	Proportion	2017	39.3	47.0	32.4
Indonesia	Gorontalo	Count	2000	0	1	0
Indonesia	Gorontalo	Count	2017	0	0	0
Indonesia	Jakarta Utara	Proportion	2000	38.2	44.6	31.4
Indonesia	Jakarta Utara	Proportion	2017	40.6	46.3	35.1

Table 4: ORS by Unit *(continued)*

Country	Unit	Metric	Year	Mean	Upper	Lower
Indonesia	Jakarta Utara	Count	2000	0	0	0
Indonesia	Jakarta Utara	Count	2017	0	0	0
Indonesia	Merangin	Proportion	2000	43.2	52.9	35.1
Indonesia	Merangin	Proportion	2017	37.7	46.0	31.3
Indonesia	Merangin	Count	2000	0	1	0
Indonesia	Merangin	Count	2017	0	0	0
Indonesia	Bogor	Proportion	2000	34.8	40.7	29.0
Indonesia	Bogor	Proportion	2017	40.8	45.5	35.3
Indonesia	Bogor	Count	2000	0	0	0
Indonesia	Bogor	Count	2017	0	0	0
Indonesia	Boyolali	Proportion	2000	36.0	43.4	29.5
Indonesia	Boyolali	Proportion	2017	29.7	35.8	25.0
Indonesia	Boyolali	Count	2000	0	0	0
Indonesia	Boyolali	Count	2017	0	0	0
Indonesia	Bojonegoro	Proportion	2000	37.7	46.6	30.0
Indonesia	Bojonegoro	Proportion	2017	31.7	38.0	26.3
Indonesia	Bojonegoro	Count	2000	0	0	0
Indonesia	Bojonegoro	Count	2017	0	0	0
Indonesia	Kota Pontianak	Proportion	2000	39.4	49.3	30.8
Indonesia	Kota Pontianak	Proportion	2017	36.6	45.2	28.8
Indonesia	Kota Pontianak	Count	2000	0	0	0
Indonesia	Kota Pontianak	Count	2017	0	0	0
Indonesia	Barito Kuala	Proportion	2000	36.1	44.5	27.6
Indonesia	Barito Kuala	Proportion	2017	29.8	36.5	24.2
Indonesia	Barito Kuala	Count	2000	0	0	0
Indonesia	Barito Kuala	Count	2017	0	0	0
Indonesia	Kapuas	Proportion	2000	39.3	47.2	31.4
Indonesia	Kapuas	Proportion	2017	32.2	38.5	26.1
Indonesia	Kapuas	Count	2000	0	0	0
Indonesia	Kapuas	Count	2017	0	0	0
Indonesia	Kutai Kartanegara	Proportion	2000	44.7	53.8	35.4
Indonesia	Kutai Kartanegara	Proportion	2017	34.7	41.0	27.9
Indonesia	Kutai Kartanegara	Count	2000	0	1	0
Indonesia	Kutai Kartanegara	Count	2017	0	0	0
Indonesia	Lingga	Proportion	2000	44.4	54.4	35.1
Indonesia	Lingga	Proportion	2017	43.4	50.8	35.3
Indonesia	Lingga	Count	2000	0	1	0
Indonesia	Lingga	Count	2017	0	1	0
Indonesia	Lampung Timur	Proportion	2000	38.1	45.7	31.2
Indonesia	Lampung Timur	Proportion	2017	40.1	46.6	33.2
Indonesia	Lampung Timur	Count	2000	0	0	0
Indonesia	Lampung Timur	Count	2017	0	0	0
Indonesia	Halmahera Utara	Proportion	2000	51.8	63.8	40.3
Indonesia	Halmahera Utara	Proportion	2017	47.8	55.7	39.0
Indonesia	Halmahera Utara	Count	2000	1	1	0
Indonesia	Halmahera Utara	Count	2017	0	1	0
Indonesia	Maluku Barat Daya	Proportion	2000	49.1	59.1	39.4
Indonesia	Maluku Barat Daya	Proportion	2017	48.1	57.1	40.1
Indonesia	Maluku Barat Daya	Count	2000	0	1	0

Table 4: ORS by Unit *(continued)*

Country	Unit	Metric	Year	Mean	Upper	Lower
Indonesia	Maluku Barat Daya	Count	2017	0	1	0
Indonesia	Lombok Tengah	Proportion	2000	55.1	63.1	46.5
Indonesia	Lombok Tengah	Proportion	2017	52.2	60.7	45.3
Indonesia	Lombok Tengah	Count	2000	1	1	0
Indonesia	Lombok Tengah	Count	2017	1	1	0
Indonesia	Kota Kupang	Proportion	2000	52.9	62.6	42.8
Indonesia	Kota Kupang	Proportion	2017	47.5	55.7	40.1
Indonesia	Kota Kupang	Count	2000	1	1	0
Indonesia	Kota Kupang	Count	2017	0	1	0
Indonesia	Maybrat	Proportion	2000	46.1	57.0	35.0
Indonesia	Maybrat	Proportion	2017	48.1	57.3	37.8
Indonesia	Maybrat	Count	2000	0	1	0
Indonesia	Maybrat	Count	2017	0	1	0
Indonesia	Dogiyai	Proportion	2000	45.2	55.9	34.0
Indonesia	Dogiyai	Proportion	2017	43.8	54.4	34.8
Indonesia	Dogiyai	Count	2000	0	1	0
Indonesia	Dogiyai	Count	2017	0	1	0
Indonesia	Kampar	Proportion	2000	37.9	46.8	31.3
Indonesia	Kampar	Proportion	2017	34.2	40.9	28.0
Indonesia	Kampar	Count	2000	0	0	0
Indonesia	Kampar	Count	2017	0	0	0
Indonesia	Polewali Mandar	Proportion	2000	42.8	52.2	35.3
Indonesia	Polewali Mandar	Proportion	2017	29.6	35.5	24.3
Indonesia	Polewali Mandar	Count	2000	0	1	0
Indonesia	Polewali Mandar	Count	2017	0	0	0
Indonesia	Enrekang	Proportion	2000	38.9	47.2	31.2
Indonesia	Enrekang	Proportion	2017	27.2	32.8	22.6
Indonesia	Enrekang	Count	2000	0	0	0
Indonesia	Enrekang	Count	2017	0	0	0
Indonesia	Morowali	Proportion	2000	40.2	47.6	32.9
Indonesia	Morowali	Proportion	2017	30.4	37.3	24.1
Indonesia	Morowali	Count	2000	0	0	0
Indonesia	Morowali	Count	2017	0	0	0
Indonesia	Kendari	Proportion	2000	40.9	48.9	32.4
Indonesia	Kendari	Proportion	2017	30.4	37.5	24.4
Indonesia	Kendari	Count	2000	0	0	0
Indonesia	Kendari	Count	2017	0	0	0
Indonesia	Bolaang Mongondow	Proportion	2000	37.8	48.2	28.4
Indonesia	Bolaang Mongondow	Proportion	2017	38.0	45.7	30.8
Indonesia	Bolaang Mongondow	Count	2000	0	0	0
Indonesia	Bolaang Mongondow	Count	2017	0	0	0
Indonesia	Kepulauan Mentawai	Proportion	2000	41.6	52.1	32.6
Indonesia	Kepulauan Mentawai	Proportion	2017	37.0	44.8	29.5
Indonesia	Kepulauan Mentawai	Count	2000	0	1	0
Indonesia	Kepulauan Mentawai	Count	2017	0	0	0
Indonesia	Muara Enim	Proportion	2000	42.5	50.9	34.8
Indonesia	Muara Enim	Proportion	2017	40.7	46.5	35.3
Indonesia	Muara Enim	Count	2000	0	1	0
Indonesia	Muara Enim	Count	2017	0	0	0

Table 4: ORS by Unit *(continued)*

Country	Unit	Metric	Year	Mean	Upper	Lower
Indonesia	Gunungsitoli	Proportion	2000	29.7	37.7	22.5
Indonesia	Gunungsitoli	Proportion	2017	29.4	36.4	22.8
Indonesia	Gunungsitoli	Count	2000	0	0	0
Indonesia	Gunungsitoli	Count	2017	0	0	0
Indonesia	Sleman	Proportion	2000	36.7	43.9	29.8
Indonesia	Sleman	Proportion	2017	31.1	37.4	25.8
Indonesia	Sleman	Count	2000	0	0	0
Indonesia	Sleman	Count	2017	0	0	0
Indonesia	Aceh Singkil	Proportion	2000	28.7	37.0	21.5
Indonesia	Aceh Singkil	Proportion	2017	28.2	33.4	22.9
Indonesia	Aceh Singkil	Count	2000	0	0	0
Indonesia	Aceh Singkil	Count	2017	0	0	0
Indonesia	Jembrana	Proportion	2000	40.3	49.5	31.8
Indonesia	Jembrana	Proportion	2017	40.2	47.6	33.3
Indonesia	Jembrana	Count	2000	0	0	0
Indonesia	Jembrana	Count	2017	0	0	0
Indonesia	Belitung	Proportion	2000	45.9	54.6	37.4
Indonesia	Belitung	Proportion	2017	42.4	51.1	33.6
Indonesia	Belitung	Count	2000	0	1	0
Indonesia	Belitung	Count	2017	0	1	0
Indonesia	Serang	Proportion	2000	36.9	43.5	30.4
Indonesia	Serang	Proportion	2017	42.0	47.2	36.0
Indonesia	Serang	Count	2000	0	0	0
Indonesia	Serang	Count	2017	0	0	0
Indonesia	Kepahiang	Proportion	2000	40.3	49.0	32.1
Indonesia	Kepahiang	Proportion	2017	38.1	46.0	31.4
Indonesia	Kepahiang	Count	2000	0	0	0
Indonesia	Kepahiang	Count	2017	0	0	0
Indonesia	Kota Gorontalo	Proportion	2000	44.0	53.5	34.2
Indonesia	Kota Gorontalo	Proportion	2017	39.4	47.5	32.1
Indonesia	Kota Gorontalo	Count	2000	0	1	0
Indonesia	Kota Gorontalo	Count	2017	0	0	0
Indonesia	Kepulauan Seribu	Proportion	2000	35.6	41.7	29.4
Indonesia	Kepulauan Seribu	Proportion	2017	38.6	43.9	33.1
Indonesia	Kepulauan Seribu	Count	2000	0	0	0
Indonesia	Kepulauan Seribu	Count	2017	0	0	0
Indonesia	Muaro Jambi	Proportion	2000	46.2	54.9	37.7
Indonesia	Muaro Jambi	Proportion	2017	39.9	47.1	32.8
Indonesia	Muaro Jambi	Count	2000	0	1	0
Indonesia	Muaro Jambi	Count	2017	0	0	0
Indonesia	Ciamis	Proportion	2000	36.4	43.7	29.8
Indonesia	Ciamis	Proportion	2017	36.3	41.5	30.6
Indonesia	Ciamis	Count	2000	0	0	0
Indonesia	Ciamis	Count	2017	0	0	0
Indonesia	Brebes	Proportion	2000	35.6	42.2	29.2
Indonesia	Brebes	Proportion	2017	34.6	40.0	28.5
Indonesia	Brebes	Count	2000	0	0	0
Indonesia	Brebes	Count	2017	0	0	0
Indonesia	Bondowoso	Proportion	2000	36.7	45.3	28.5

Table 4: ORS by Unit *(continued)*

Country	Unit	Metric	Year	Mean	Upper	Lower
Indonesia	Bondowoso	Proportion	2017	35.3	42.2	28.9
Indonesia	Bondowoso	Count	2000	0	0	0
Indonesia	Bondowoso	Count	2017	0	0	0
Indonesia	Kubu Raya	Proportion	2000	40.0	49.2	31.5
Indonesia	Kubu Raya	Proportion	2017	37.3	46.2	29.2
Indonesia	Kubu Raya	Count	2000	0	0	0
Indonesia	Kubu Raya	Count	2017	0	0	0
Indonesia	Hulu Sungai Selatan	Proportion	2000	36.0	44.7	27.9
Indonesia	Hulu Sungai Selatan	Proportion	2017	29.6	35.9	23.6
Indonesia	Hulu Sungai Selatan	Count	2000	0	0	0
Indonesia	Hulu Sungai Selatan	Count	2017	0	0	0
Indonesia	Katingan	Proportion	2000	42.2	51.4	33.2
Indonesia	Katingan	Proportion	2017	34.5	41.9	27.5
Indonesia	Katingan	Count	2000	0	1	0
Indonesia	Katingan	Count	2017	0	0	0
Indonesia	Kutai Timur	Proportion	2000	46.7	56.8	38.0
Indonesia	Kutai Timur	Proportion	2017	37.5	44.1	30.2
Indonesia	Kutai Timur	Count	2000	0	1	0
Indonesia	Kutai Timur	Count	2017	0	0	0
Indonesia	Natuna	Proportion	2000	45.5	57.7	35.0
Indonesia	Natuna	Proportion	2017	43.5	54.6	33.6
Indonesia	Natuna	Count	2000	0	1	0
Indonesia	Natuna	Count	2017	0	1	0
Indonesia	Lampung Utara	Proportion	2000	37.3	44.7	29.6
Indonesia	Lampung Utara	Proportion	2017	39.1	46.9	33.2
Indonesia	Lampung Utara	Count	2000	0	0	0
Indonesia	Lampung Utara	Count	2017	0	0	0
Indonesia	Kepulauan Sula	Proportion	2000	40.4	50.4	30.8
Indonesia	Kepulauan Sula	Proportion	2017	38.5	47.4	30.5
Indonesia	Kepulauan Sula	Count	2000	0	1	0
Indonesia	Kepulauan Sula	Count	2017	0	0	0
Indonesia	Maluku Tengah	Proportion	2000	38.4	50.2	27.5
Indonesia	Maluku Tengah	Proportion	2017	41.4	48.8	33.5
Indonesia	Maluku Tengah	Count	2000	0	1	0
Indonesia	Maluku Tengah	Count	2017	0	0	0
Indonesia	Lombok Timur	Proportion	2000	53.0	61.5	44.7
Indonesia	Lombok Timur	Proportion	2017	51.0	58.8	44.8
Indonesia	Lombok Timur	Count	2000	1	1	0
Indonesia	Lombok Timur	Count	2017	1	1	0
Indonesia	Kupang	Proportion	2000	54.3	64.2	45.0
Indonesia	Kupang	Proportion	2017	49.4	56.3	42.5
Indonesia	Kupang	Count	2000	1	1	0
Indonesia	Kupang	Count	2017	0	1	0
Indonesia	Raja Ampat	Proportion	2000	41.4	51.9	30.3
Indonesia	Raja Ampat	Proportion	2017	43.8	52.3	33.7
Indonesia	Raja Ampat	Count	2000	0	1	0
Indonesia	Raja Ampat	Count	2017	0	1	0
Indonesia	Intan Jaya	Proportion	2000	47.7	57.7	37.6
Indonesia	Intan Jaya	Proportion	2017	45.6	56.2	36.7

Table 4: ORS by Unit *(continued)*

Country	Unit	Metric	Year	Mean	Upper	Lower
Indonesia	Intan Jaya	Count	2000	0	1	0
Indonesia	Intan Jaya	Count	2017	0	1	0
Indonesia	Kepulauan Meranti	Proportion	2000	40.8	51.0	33.0
Indonesia	Kepulauan Meranti	Proportion	2017	38.2	45.9	30.6
Indonesia	Kepulauan Meranti	Count	2000	0	1	0
Indonesia	Kepulauan Meranti	Count	2017	0	0	0
Indonesia	Gowa	Proportion	2000	39.3	47.8	31.9
Indonesia	Gowa	Proportion	2017	28.9	34.9	23.2
Indonesia	Gowa	Count	2000	0	0	0
Indonesia	Gowa	Count	2017	0	0	0
Indonesia	Palu	Proportion	2000	42.9	52.7	33.2
Indonesia	Palu	Proportion	2017	34.1	41.1	27.6
Indonesia	Palu	Count	2000	0	1	0
Indonesia	Palu	Count	2017	0	0	0
Indonesia	Kolaka Utara	Proportion	2000	40.4	48.7	32.4
Indonesia	Kolaka Utara	Proportion	2017	27.9	33.7	22.7
Indonesia	Kolaka Utara	Count	2000	0	0	0
Indonesia	Kolaka Utara	Count	2017	0	0	0
Indonesia	Kepulauan Sangihe	Proportion	2000	39.3	49.3	29.5
Indonesia	Kepulauan Sangihe	Proportion	2017	41.5	52.0	32.5
Indonesia	Kepulauan Sangihe	Count	2000	0	0	0
Indonesia	Kepulauan Sangihe	Count	2017	0	1	0
Indonesia	Kota Solok	Proportion	2000	34.6	43.5	27.7
Indonesia	Kota Solok	Proportion	2017	30.7	38.0	23.6
Indonesia	Kota Solok	Count	2000	0	0	0
Indonesia	Kota Solok	Count	2017	0	0	0
Indonesia	Musi Banyuasin	Proportion	2000	44.0	53.5	35.3
Indonesia	Musi Banyuasin	Proportion	2017	40.5	48.2	34.4
Indonesia	Musi Banyuasin	Count	2000	0	1	0
Indonesia	Musi Banyuasin	Count	2017	0	0	0
Indonesia	Humbang Hasundutan	Proportion	2000	27.7	34.9	21.0
Indonesia	Humbang Hasundutan	Proportion	2017	26.9	32.5	21.9
Indonesia	Humbang Hasundutan	Count	2000	0	0	0
Indonesia	Humbang Hasundutan	Count	2017	0	0	0
Indonesia	Aceh Tamiang	Proportion	2000	32.0	40.0	23.9
Indonesia	Aceh Tamiang	Proportion	2017	29.6	36.2	24.5
Indonesia	Aceh Tamiang	Count	2000	0	0	0
Indonesia	Aceh Tamiang	Count	2017	0	0	0
Indonesia	Karangasem	Proportion	2000	47.3	56.8	38.4
Indonesia	Karangasem	Proportion	2017	46.6	53.9	39.2
Indonesia	Karangasem	Count	2000	0	1	0
Indonesia	Karangasem	Count	2017	0	1	0
Indonesia	Pangkalpinang	Proportion	2000	44.8	53.9	36.2
Indonesia	Pangkalpinang	Proportion	2017	43.9	53.2	36.8
Indonesia	Pangkalpinang	Count	2000	0	1	0
Indonesia	Pangkalpinang	Count	2017	0	1	0
Indonesia	Tangerang Selatan	Proportion	2000	35.9	41.7	30.0
Indonesia	Tangerang Selatan	Proportion	2017	38.1	43.1	32.6
Indonesia	Tangerang Selatan	Count	2000	0	0	0

Table 4: ORS by Unit *(continued)*

Country	Unit	Metric	Year	Mean	Upper	Lower
Indonesia	Tangerang Selatan	Count	2017	0	0	0
Indonesia	Lebong	Proportion	2000	39.3	48.4	31.5
Indonesia	Lebong	Proportion	2017	36.6	45.0	30.2
Indonesia	Lebong	Count	2000	0	0	0
Indonesia	Lebong	Count	2017	0	0	0
Indonesia	Pohuwato	Proportion	2000	43.5	52.3	35.5
Indonesia	Pohuwato	Proportion	2017	37.8	45.4	30.5
Indonesia	Pohuwato	Count	2000	0	1	0
Indonesia	Pohuwato	Count	2017	0	0	0
Indonesia	Sarolangun	Proportion	2000	44.1	53.7	36.0
Indonesia	Sarolangun	Proportion	2017	38.1	45.9	31.7
Indonesia	Sarolangun	Count	2000	0	1	0
Indonesia	Sarolangun	Count	2017	0	0	0
Indonesia	Cianjur	Proportion	2000	35.4	41.3	29.3
Indonesia	Cianjur	Proportion	2017	40.7	46.4	35.7
Indonesia	Cianjur	Count	2000	0	0	0
Indonesia	Cianjur	Count	2017	0	0	0
Indonesia	Cilacap	Proportion	2000	35.9	43.5	29.0
Indonesia	Cilacap	Proportion	2017	33.3	38.3	27.5
Indonesia	Cilacap	Count	2000	0	0	0
Indonesia	Cilacap	Count	2017	0	0	0
Indonesia	Gresik	Proportion	2000	39.4	48.6	31.0
Indonesia	Gresik	Proportion	2017	34.0	39.7	27.8
Indonesia	Gresik	Count	2000	0	0	0
Indonesia	Gresik	Count	2017	0	0	0
Indonesia	Landak	Proportion	2000	40.2	49.1	31.6
Indonesia	Landak	Proportion	2017	37.7	45.5	29.5
Indonesia	Landak	Count	2000	0	0	0
Indonesia	Landak	Count	2017	0	0	0
Indonesia	Hulu Sungai Tengah	Proportion	2000	35.5	43.9	27.4
Indonesia	Hulu Sungai Tengah	Proportion	2017	29.5	36.4	23.5
Indonesia	Hulu Sungai Tengah	Count	2000	0	0	0
Indonesia	Hulu Sungai Tengah	Count	2017	0	0	0
Indonesia	Kotawaringin Barat	Proportion	2000	40.4	50.0	30.7
Indonesia	Kotawaringin Barat	Proportion	2017	33.8	41.2	26.3
Indonesia	Kotawaringin Barat	Count	2000	0	0	0
Indonesia	Kotawaringin Barat	Count	2017	0	0	0
Indonesia	Paser	Proportion	2000	42.1	51.1	32.4
Indonesia	Paser	Proportion	2017	33.2	40.0	26.7
Indonesia	Paser	Count	2000	0	1	0
Indonesia	Paser	Count	2017	0	0	0
Indonesia	Tanjungpinang	Proportion	2000	43.7	54.5	34.9
Indonesia	Tanjungpinang	Proportion	2017	44.1	52.1	35.7
Indonesia	Tanjungpinang	Count	2000	0	1	0
Indonesia	Tanjungpinang	Count	2017	0	1	0
Indonesia	Mesuji	Proportion	2000	41.5	50.2	33.8
Indonesia	Mesuji	Proportion	2017	43.1	49.6	37.5
Indonesia	Mesuji	Count	2000	0	1	0
Indonesia	Mesuji	Count	2017	0	0	0

Table 4: ORS by Unit *(continued)*

Country	Unit	Metric	Year	Mean	Upper	Lower
Indonesia	Pulau Morotai	Proportion	2000	49.7	61.9	38.0
Indonesia	Pulau Morotai	Proportion	2017	46.1	55.9	36.0
Indonesia	Pulau Morotai	Count	2000	0	1	0
Indonesia	Pulau Morotai	Count	2017	0	1	0
Indonesia	Maluku Tenggara Barat	Proportion	2000	41.8	53.8	30.8
Indonesia	Maluku Tenggara Barat	Proportion	2017	44.2	55.9	34.5
Indonesia	Maluku Tenggara Barat	Count	2000	0	1	0
Indonesia	Maluku Tenggara Barat	Count	2017	0	1	0
Indonesia	Lombok Utara	Proportion	2000	51.5	59.4	42.4
Indonesia	Lombok Utara	Proportion	2017	49.7	57.4	42.9
Indonesia	Lombok Utara	Count	2000	1	1	0
Indonesia	Lombok Utara	Count	2017	0	1	0
Indonesia	Lembata	Proportion	2000	51.5	61.0	42.2
Indonesia	Lembata	Proportion	2017	46.0	53.6	38.1
Indonesia	Lembata	Count	2000	1	1	0
Indonesia	Lembata	Count	2017	0	1	0
Indonesia	Sorong Selatan	Proportion	2000	46.7	57.7	34.6
Indonesia	Sorong Selatan	Proportion	2017	49.6	58.4	39.4
Indonesia	Sorong Selatan	Count	2000	0	1	0
Indonesia	Sorong Selatan	Count	2017	0	1	0
Indonesia	Jayapura	Proportion	2000	44.9	55.2	33.5
Indonesia	Jayapura	Proportion	2017	43.4	52.9	33.1
Indonesia	Jayapura	Count	2000	0	1	0
Indonesia	Jayapura	Count	2017	0	1	0
Indonesia	Kuantan Singingi	Proportion	2000	39.0	47.1	31.5
Indonesia	Kuantan Singingi	Proportion	2017	33.4	40.4	27.0
Indonesia	Kuantan Singingi	Count	2000	0	0	0
Indonesia	Kuantan Singingi	Count	2017	0	0	0
Indonesia	Jeneponto	Proportion	2000	39.1	47.8	31.8
Indonesia	Jeneponto	Proportion	2017	29.2	36.3	23.2
Indonesia	Jeneponto	Count	2000	0	0	0
Indonesia	Jeneponto	Count	2017	0	0	0
Indonesia	Parigi Moutong	Proportion	2000	41.5	50.1	33.4
Indonesia	Parigi Moutong	Proportion	2017	34.1	40.2	28.3
Indonesia	Parigi Moutong	Count	2000	0	1	0
Indonesia	Parigi Moutong	Count	2017	0	0	0
Indonesia	Kolaka	Proportion	2000	41.0	49.7	33.5
Indonesia	Kolaka	Proportion	2017	28.6	34.1	22.4
Indonesia	Kolaka	Count	2000	0	0	0
Indonesia	Kolaka	Count	2017	0	0	0
Indonesia	Kepulauan Talaud	Proportion	2000	40.2	50.7	29.6
Indonesia	Kepulauan Talaud	Proportion	2017	41.2	51.9	32.2
Indonesia	Kepulauan Talaud	Count	2000	0	1	0
Indonesia	Kepulauan Talaud	Count	2017	0	1	0
Indonesia	Lima Puluh Kota	Proportion	2000	34.0	41.4	27.8
Indonesia	Lima Puluh Kota	Proportion	2017	30.7	37.1	24.3
Indonesia	Lima Puluh Kota	Count	2000	0	0	0
Indonesia	Lima Puluh Kota	Count	2017	0	0	0
Indonesia	Musi Rawas	Proportion	2000	42.6	51.6	34.5

Table 4: ORS by Unit *(continued)*

Country	Unit	Metric	Year	Mean	Upper	Lower
Indonesia	Musi Rawas	Proportion	2017	38.4	46.4	32.4
Indonesia	Musi Rawas	Count	2000	0	1	0
Indonesia	Musi Rawas	Count	2017	0	0	0
Indonesia	Karo	Proportion	2000	27.9	35.0	21.2
Indonesia	Karo	Proportion	2017	26.9	31.8	21.9
Indonesia	Karo	Count	2000	0	0	0
Indonesia	Karo	Count	2017	0	0	0
Indonesia	Aceh Tengah	Proportion	2000	32.5	41.5	24.0
Indonesia	Aceh Tengah	Proportion	2017	28.5	34.7	23.0
Indonesia	Aceh Tengah	Count	2000	0	0	0
Indonesia	Aceh Tengah	Count	2017	0	0	0
Indonesia	Klungkung	Proportion	2000	48.8	58.4	39.8
Indonesia	Klungkung	Proportion	2017	49.2	57.3	41.5
Indonesia	Klungkung	Count	2000	0	1	0
Indonesia	Klungkung	Count	2017	0	1	0
Indonesia	Tangerang	Proportion	2000	36.0	42.3	30.0
Indonesia	Tangerang	Proportion	2017	39.3	44.3	34.1
Indonesia	Tangerang	Count	2000	0	0	0
Indonesia	Tangerang	Count	2017	0	0	0
Indonesia	Mukomuko	Proportion	2000	41.5	51.7	33.2
Indonesia	Mukomuko	Proportion	2017	36.0	44.2	29.5
Indonesia	Mukomuko	Count	2000	0	1	0
Indonesia	Mukomuko	Count	2017	0	0	0
Indonesia	Sungai Penuh	Proportion	2000	41.0	51.4	33.0
Indonesia	Sungai Penuh	Proportion	2017	35.7	43.9	29.8
Indonesia	Sungai Penuh	Count	2000	0	1	0
Indonesia	Sungai Penuh	Count	2017	0	0	0
Indonesia	Cimahi	Proportion	2000	36.7	43.5	30.7
Indonesia	Cimahi	Proportion	2017	42.2	48.1	37.0
Indonesia	Cimahi	Count	2000	0	0	0
Indonesia	Cimahi	Count	2017	0	0	0
Indonesia	Demak	Proportion	2000	36.6	43.9	29.1
Indonesia	Demak	Proportion	2017	30.5	36.2	25.2
Indonesia	Demak	Count	2000	0	0	0
Indonesia	Demak	Count	2017	0	0	0
Indonesia	Jember	Proportion	2000	37.6	46.0	29.7
Indonesia	Jember	Proportion	2017	35.7	43.5	29.3
Indonesia	Jember	Count	2000	0	0	0
Indonesia	Jember	Count	2017	0	0	0
Indonesia	Melawi	Proportion	2000	41.6	51.7	33.0
Indonesia	Melawi	Proportion	2017	35.9	44.7	28.1
Indonesia	Melawi	Count	2000	0	1	0
Indonesia	Melawi	Count	2017	0	0	0
Indonesia	Hulu Sungai Utara	Proportion	2000	36.0	45.0	27.7
Indonesia	Hulu Sungai Utara	Proportion	2017	29.4	36.4	23.3
Indonesia	Hulu Sungai Utara	Count	2000	0	0	0
Indonesia	Hulu Sungai Utara	Count	2017	0	0	0
Indonesia	Kotawaringin Timur	Proportion	2000	40.7	50.0	31.5
Indonesia	Kotawaringin Timur	Proportion	2017	33.6	41.7	26.3

Table 4: ORS by Unit *(continued)*

Country	Unit	Metric	Year	Mean	Upper	Lower
Indonesia	Kotawaringin Timur	Count	2000	0	0	0
Indonesia	Kotawaringin Timur	Count	2017	0	0	0
Indonesia	Penajam Paser Utara	Proportion	2000	43.2	52.6	33.9
Indonesia	Penajam Paser Utara	Proportion	2017	34.3	41.6	27.1
Indonesia	Penajam Paser Utara	Count	2000	0	1	0
Indonesia	Penajam Paser Utara	Count	2017	0	0	0
Indonesia	Metro	Proportion	2000	38.6	46.5	31.3
Indonesia	Metro	Proportion	2017	41.5	48.2	34.8
Indonesia	Metro	Count	2000	0	0	0
Indonesia	Metro	Count	2017	0	0	0
Indonesia	Ternate	Proportion	2000	40.4	53.1	29.2
Indonesia	Ternate	Proportion	2017	42.2	50.6	34.2
Indonesia	Ternate	Count	2000	0	1	0
Indonesia	Ternate	Count	2017	0	1	0
Indonesia	Maluku Tenggara	Proportion	2000	43.5	56.2	32.6
Indonesia	Maluku Tenggara	Proportion	2017	43.8	55.8	35.0
Indonesia	Maluku Tenggara	Count	2000	0	1	0
Indonesia	Maluku Tenggara	Count	2017	0	1	0
Indonesia	Mataram	Proportion	2000	54.4	62.3	45.5
Indonesia	Mataram	Proportion	2017	51.8	59.6	44.6
Indonesia	Mataram	Count	2000	1	1	0
Indonesia	Mataram	Count	2017	1	1	0
Indonesia	Manggarai Barat	Proportion	2000	49.6	58.0	40.3
Indonesia	Manggarai Barat	Proportion	2017	46.9	54.9	39.2
Indonesia	Manggarai Barat	Count	2000	0	1	0
Indonesia	Manggarai Barat	Count	2017	0	1	0
Indonesia	Sorong	Proportion	2000	44.3	54.2	32.9
Indonesia	Sorong	Proportion	2017	48.9	58.4	37.7
Indonesia	Sorong	Count	2000	0	1	0
Indonesia	Sorong	Count	2017	0	1	0
Indonesia	Jayawijaya	Proportion	2000	46.9	56.2	37.7
Indonesia	Jayawijaya	Proportion	2017	43.9	53.7	34.1
Indonesia	Jayawijaya	Count	2000	0	1	0
Indonesia	Jayawijaya	Count	2017	0	1	0
Indonesia	Pekanbaru	Proportion	2000	39.3	48.1	32.4
Indonesia	Pekanbaru	Proportion	2017	35.1	42.2	28.6
Indonesia	Pekanbaru	Count	2000	0	0	0
Indonesia	Pekanbaru	Count	2017	0	0	0
Indonesia	Kepulauan Selayar	Proportion	2000	40.8	49.4	32.7
Indonesia	Kepulauan Selayar	Proportion	2017	33.1	40.8	26.3
Indonesia	Kepulauan Selayar	Count	2000	0	0	0
Indonesia	Kepulauan Selayar	Count	2017	0	0	0
Indonesia	Poso	Proportion	2000	40.3	48.9	31.9
Indonesia	Poso	Proportion	2017	30.5	36.8	24.6
Indonesia	Poso	Count	2000	0	0	0
Indonesia	Poso	Count	2017	0	0	0
Indonesia	Konawe Selatan	Proportion	2000	40.9	48.9	32.7
Indonesia	Konawe Selatan	Proportion	2017	29.7	35.8	23.9
Indonesia	Konawe Selatan	Count	2000	0	0	0

Table 4: ORS by Unit *(continued)*

Country	Unit	Metric	Year	Mean	Upper	Lower
Indonesia	Konawe Selatan	Count	2017	0	0	0
Indonesia	Kotamobagu	Proportion	2000	36.2	47.1	27.1
Indonesia	Kotamobagu	Proportion	2017	37.3	44.8	30.0
Indonesia	Kotamobagu	Count	2000	0	0	0
Indonesia	Kotamobagu	Count	2017	0	0	0
Indonesia	Padang Panjang	Proportion	2000	34.6	42.7	28.0
Indonesia	Padang Panjang	Proportion	2017	30.5	37.3	23.4
Indonesia	Padang Panjang	Count	2000	0	0	0
Indonesia	Padang Panjang	Count	2017	0	0	0
Indonesia	Ogan Ilir	Proportion	2000	42.8	52.2	35.0
Indonesia	Ogan Ilir	Proportion	2017	41.6	48.1	35.5
Indonesia	Ogan Ilir	Count	2000	0	1	0
Indonesia	Ogan Ilir	Count	2017	0	0	0
Indonesia	Kota Binjai	Proportion	2000	31.2	38.7	23.9
Indonesia	Kota Binjai	Proportion	2017	28.5	33.3	23.7
Indonesia	Kota Binjai	Count	2000	0	0	0
Indonesia	Kota Binjai	Count	2017	0	0	0
Indonesia	Aceh Tenggara	Proportion	2000	29.7	36.8	22.5
Indonesia	Aceh Tenggara	Proportion	2017	28.8	34.3	23.8
Indonesia	Aceh Tenggara	Count	2000	0	0	0
Indonesia	Aceh Tenggara	Count	2017	0	0	0
Indonesia	Tabanan	Proportion	2000	40.7	49.7	32.4
Indonesia	Tabanan	Proportion	2017	40.2	48.1	33.1
Indonesia	Tabanan	Count	2000	0	0	0
Indonesia	Tabanan	Count	2017	0	0	0
Indonesia	Rejang Lebong	Proportion	2000	40.3	48.9	32.0
Indonesia	Rejang Lebong	Proportion	2017	38.2	46.4	32.0
Indonesia	Rejang Lebong	Count	2000	0	0	0
Indonesia	Rejang Lebong	Count	2017	0	0	0
Indonesia	Tanjung Jabung B	Proportion	2000	42.4	51.5	33.3
Indonesia	Tanjung Jabung B	Proportion	2017	36.7	44.1	29.4
Indonesia	Tanjung Jabung B	Count	2000	0	1	0
Indonesia	Tanjung Jabung B	Count	2017	0	0	0
Indonesia	Cirebon	Proportion	2000	37.6	44.2	30.8
Indonesia	Cirebon	Proportion	2017	38.9	44.1	33.8
Indonesia	Cirebon	Count	2000	0	0	0
Indonesia	Cirebon	Count	2017	0	0	0
Indonesia	Grobogan	Proportion	2000	36.5	43.8	29.5
Indonesia	Grobogan	Proportion	2017	30.3	36.6	25.3
Indonesia	Grobogan	Count	2000	0	0	0
Indonesia	Grobogan	Count	2017	0	0	0
Indonesia	Jombang	Proportion	2000	39.1	47.3	31.3
Indonesia	Jombang	Proportion	2017	33.3	39.5	27.2
Indonesia	Jombang	Count	2000	0	0	0
Indonesia	Jombang	Count	2017	0	0	0
Indonesia	Pontianak	Proportion	2000	40.1	49.6	31.7
Indonesia	Pontianak	Proportion	2017	37.9	47.2	30.3
Indonesia	Pontianak	Count	2000	0	0	0
Indonesia	Pontianak	Count	2017	0	0	0

Table 4: ORS by Unit *(continued)*

Country	Unit	Metric	Year	Mean	Upper	Lower
Indonesia	Kota Baru	Proportion	2000	37.5	46.1	29.6
Indonesia	Kota Baru	Proportion	2017	31.1	38.2	24.6
Indonesia	Kota Baru	Count	2000	0	0	0
Indonesia	Kota Baru	Count	2017	0	0	0
Indonesia	Lamandau	Proportion	2000	40.9	51.0	31.8
Indonesia	Lamandau	Proportion	2017	35.1	42.9	27.5
Indonesia	Lamandau	Count	2000	0	1	0
Indonesia	Lamandau	Count	2017	0	0	0
Indonesia	Samarinda	Proportion	2000	44.0	53.6	34.7
Indonesia	Samarinda	Proportion	2017	34.2	41.0	27.2
Indonesia	Samarinda	Count	2000	0	1	0
Indonesia	Samarinda	Count	2017	0	0	0
Indonesia	Pesawaran	Proportion	2000	36.3	43.0	29.6
Indonesia	Pesawaran	Proportion	2017	38.8	45.0	32.7
Indonesia	Pesawaran	Count	2000	0	0	0
Indonesia	Pesawaran	Count	2017	0	0	0
Indonesia	Tidore Kepulauan	Proportion	2000	40.5	52.9	29.6
Indonesia	Tidore Kepulauan	Proportion	2017	42.3	50.3	33.7
Indonesia	Tidore Kepulauan	Count	2000	0	1	0
Indonesia	Tidore Kepulauan	Count	2017	0	1	0
Indonesia	Seram Bagian Barat	Proportion	2000	39.6	51.8	28.4
Indonesia	Seram Bagian Barat	Proportion	2017	41.3	49.3	34.5
Indonesia	Seram Bagian Barat	Count	2000	0	1	0
Indonesia	Seram Bagian Barat	Count	2017	0	0	0
Indonesia	Sumbawa Barat	Proportion	2000	58.7	66.9	48.9
Indonesia	Sumbawa Barat	Proportion	2017	52.1	59.9	45.8
Indonesia	Sumbawa Barat	Count	2000	1	1	0
Indonesia	Sumbawa Barat	Count	2017	1	1	0
Indonesia	Manggarai Timur	Proportion	2000	47.9	57.2	38.6
Indonesia	Manggarai Timur	Proportion	2017	44.5	52.5	36.7
Indonesia	Manggarai Timur	Count	2000	0	1	0
Indonesia	Manggarai Timur	Count	2017	0	1	0
Indonesia	Tambrauw	Proportion	2000	47.3	58.3	35.6
Indonesia	Tambrauw	Proportion	2017	48.3	57.0	37.6
Indonesia	Tambrauw	Count	2000	0	1	0
Indonesia	Tambrauw	Count	2017	0	1	0
Indonesia	Keerom	Proportion	2000	44.3	54.9	33.5
Indonesia	Keerom	Proportion	2017	42.8	52.9	32.4
Indonesia	Keerom	Count	2000	0	1	0
Indonesia	Keerom	Count	2017	0	1	0
Indonesia	Pelalawan	Proportion	2000	39.9	48.1	31.7
Indonesia	Pelalawan	Proportion	2017	35.7	43.3	28.7
Indonesia	Pelalawan	Count	2000	0	0	0
Indonesia	Pelalawan	Count	2017	0	0	0
Indonesia	Luwu Timur	Proportion	2000	40.2	48.3	31.9
Indonesia	Luwu Timur	Proportion	2017	29.2	36.4	22.9
Indonesia	Luwu Timur	Count	2000	0	0	0
Indonesia	Luwu Timur	Count	2017	0	0	0
Indonesia	Sigi	Proportion	2000	41.8	51.0	33.3

Table 4: ORS by Unit *(continued)*

Country	Unit	Metric	Year	Mean	Upper	Lower
Indonesia	Sigi	Proportion	2017	32.0	38.2	25.9
Indonesia	Sigi	Count	2000	0	1	0
Indonesia	Sigi	Count	2017	0	0	0
Indonesia	Konawe Utara	Proportion	2000	41.7	49.9	33.9
Indonesia	Konawe Utara	Proportion	2017	29.7	36.1	23.8
Indonesia	Konawe Utara	Count	2000	0	0	0
Indonesia	Konawe Utara	Count	2017	0	0	0
Indonesia	Manado	Proportion	2000	37.5	47.9	28.8
Indonesia	Manado	Proportion	2017	39.5	47.8	31.1
Indonesia	Manado	Count	2000	0	0	0
Indonesia	Manado	Count	2017	0	0	0
Indonesia	Padang Pariaman	Proportion	2000	34.6	42.8	28.1
Indonesia	Padang Pariaman	Proportion	2017	31.1	37.8	24.1
Indonesia	Padang Pariaman	Count	2000	0	0	0
Indonesia	Padang Pariaman	Count	2017	0	0	0
Indonesia	Ogan Komering Ilir	Proportion	2000	42.8	51.8	35.4
Indonesia	Ogan Komering Ilir	Proportion	2017	43.1	48.8	37.5
Indonesia	Ogan Komering Ilir	Count	2000	0	1	0
Indonesia	Ogan Komering Ilir	Count	2017	0	0	0
Indonesia	Kota Medan	Proportion	2000	31.7	39.3	24.2
Indonesia	Kota Medan	Proportion	2017	28.3	33.1	23.6
Indonesia	Kota Medan	Count	2000	0	0	0
Indonesia	Kota Medan	Count	2017	0	0	0
Indonesia	Aceh Timur	Proportion	2000	33.1	41.8	25.4
Indonesia	Aceh Timur	Proportion	2017	29.6	35.8	24.6
Indonesia	Aceh Timur	Count	2000	0	0	0
Indonesia	Aceh Timur	Count	2017	0	0	0
Indonesia	Seluma	Proportion	2000	39.0	48.0	30.9
Indonesia	Seluma	Proportion	2017	37.7	45.4	30.5
Indonesia	Seluma	Count	2000	0	0	0
Indonesia	Seluma	Count	2017	0	0	0
Indonesia	Tanjung Jabung T	Proportion	2000	44.8	53.8	35.8
Indonesia	Tanjung Jabung T	Proportion	2017	39.8	47.1	32.3
Indonesia	Tanjung Jabung T	Count	2000	0	1	0
Indonesia	Tanjung Jabung T	Count	2017	0	0	0
Indonesia	Depok	Proportion	2000	35.9	42.1	29.9
Indonesia	Depok	Proportion	2017	41.1	46.0	35.9
Indonesia	Depok	Count	2000	0	0	0
Indonesia	Depok	Count	2017	0	0	0
Indonesia	Jepara	Proportion	2000	35.9	44.0	28.1
Indonesia	Jepara	Proportion	2017	30.0	36.0	24.1
Indonesia	Jepara	Count	2000	0	0	0
Indonesia	Jepara	Count	2017	0	0	0
Indonesia	Kediri	Proportion	2000	38.6	47.2	30.7
Indonesia	Kediri	Proportion	2017	32.4	38.4	26.5
Indonesia	Kediri	Count	2000	0	0	0
Indonesia	Kediri	Count	2017	0	0	0
Indonesia	Sambas	Proportion	2000	41.4	50.2	31.3
Indonesia	Sambas	Proportion	2017	38.7	48.7	31.0

Table 4: ORS by Unit *(continued)*

Country	Unit	Metric	Year	Mean	Upper	Lower
Indonesia	Sambas	Count	2000	0	1	0
Indonesia	Sambas	Count	2017	0	0	0
Indonesia	Tabalong	Proportion	2000	37.1	45.6	28.6
Indonesia	Tabalong	Proportion	2017	30.2	37.3	24.3
Indonesia	Tabalong	Count	2000	0	0	0
Indonesia	Tabalong	Count	2017	0	0	0
Indonesia	Murung Raya	Proportion	2000	42.8	51.5	33.7
Indonesia	Murung Raya	Proportion	2017	35.4	42.6	28.8
Indonesia	Murung Raya	Count	2000	0	1	0
Indonesia	Murung Raya	Count	2017	0	0	0
Indonesia	Tarakan	Proportion	2000	41.2	51.6	30.5
Indonesia	Tarakan	Proportion	2017	37.7	48.1	28.0
Indonesia	Tarakan	Count	2000	0	1	0
Indonesia	Tarakan	Count	2017	0	0	0
Indonesia	Pringsewu	Proportion	2000	35.7	42.5	28.8
Indonesia	Pringsewu	Proportion	2017	38.5	45.3	32.7
Indonesia	Pringsewu	Count	2000	0	0	0
Indonesia	Pringsewu	Count	2017	0	0	0
Indonesia	Seram Bagian Timur	Proportion	2000	40.4	53.1	28.4
Indonesia	Seram Bagian Timur	Proportion	2017	42.0	49.6	33.1
Indonesia	Seram Bagian Timur	Count	2000	0	1	0
Indonesia	Seram Bagian Timur	Count	2017	0	0	0
Indonesia	Sumbawa	Proportion	2000	54.9	63.7	44.9
Indonesia	Sumbawa	Proportion	2017	50.7	57.7	43.8
Indonesia	Sumbawa	Count	2000	1	1	0
Indonesia	Sumbawa	Count	2017	1	1	0
Indonesia	Manggarai	Proportion	2000	47.8	56.8	38.6
Indonesia	Manggarai	Proportion	2017	45.3	52.8	37.8
Indonesia	Manggarai	Count	2000	0	1	0
Indonesia	Manggarai	Count	2017	0	1	0
Indonesia	Teluk Bintuni	Proportion	2000	47.6	59.4	36.4
Indonesia	Teluk Bintuni	Proportion	2017	49.6	57.9	40.5
Indonesia	Teluk Bintuni	Count	2000	0	1	0
Indonesia	Teluk Bintuni	Count	2017	0	1	0
Indonesia	Kepulauan Yapen	Proportion	2000	46.1	55.8	36.1
Indonesia	Kepulauan Yapen	Proportion	2017	44.4	53.9	35.1
Indonesia	Kepulauan Yapen	Count	2000	0	1	0
Indonesia	Kepulauan Yapen	Count	2017	0	1	0
Indonesia	Rokan Hilir	Proportion	2000	34.8	42.8	28.0
Indonesia	Rokan Hilir	Proportion	2017	33.9	40.2	27.9
Indonesia	Rokan Hilir	Count	2000	0	0	0
Indonesia	Rokan Hilir	Count	2017	0	0	0
Indonesia	Luwu Utara	Proportion	2000	40.3	49.5	31.9
Indonesia	Luwu Utara	Proportion	2017	28.8	34.5	23.4
Indonesia	Luwu Utara	Count	2000	0	0	0
Indonesia	Luwu Utara	Count	2017	0	0	0
Indonesia	Tojo Una-Una	Proportion	2000	40.5	48.6	31.4
Indonesia	Tojo Una-Una	Proportion	2017	33.0	40.9	26.7
Indonesia	Tojo Una-Una	Count	2000	0	0	0

Table 4: ORS by Unit *(continued)*

Country	Unit	Metric	Year	Mean	Upper	Lower
Indonesia	Tojo Una-Una	Count	2017	0	0	0
Indonesia	Konawe	Proportion	2000	41.3	49.6	34.1
Indonesia	Konawe	Proportion	2017	30.0	36.0	23.9
Indonesia	Konawe	Count	2000	0	0	0
Indonesia	Konawe	Count	2017	0	0	0
Indonesia	Minahasa Selatan	Proportion	2000	36.3	45.9	27.4
Indonesia	Minahasa Selatan	Proportion	2017	38.8	46.9	31.6
Indonesia	Minahasa Selatan	Count	2000	0	0	0
Indonesia	Minahasa Selatan	Count	2017	0	0	0
Indonesia	Padang	Proportion	2000	36.5	46.4	29.1
Indonesia	Padang	Proportion	2017	32.0	39.0	24.6
Indonesia	Padang	Count	2000	0	0	0
Indonesia	Padang	Count	2017	0	0	0
Indonesia	Ogan Komering Ulu Selatan	Proportion	2000	37.9	46.5	30.0
Indonesia	Ogan Komering Ulu Selatan	Proportion	2017	38.9	46.0	32.7
Indonesia	Ogan Komering Ulu Selatan	Count	2000	0	0	0
Indonesia	Ogan Komering Ulu Selatan	Count	2017	0	0	0
Indonesia	Kota Tanjungbalai	Proportion	2000	34.4	42.5	27.8
Indonesia	Kota Tanjungbalai	Proportion	2017	31.5	38.3	25.4
Indonesia	Kota Tanjungbalai	Count	2000	0	0	0
Indonesia	Kota Tanjungbalai	Count	2017	0	0	0
Indonesia	Aceh Utara	Proportion	2000	33.9	43.0	25.8
Indonesia	Aceh Utara	Proportion	2017	29.4	35.4	24.1
Indonesia	Aceh Utara	Count	2000	0	0	0
Indonesia	Aceh Utara	Count	2017	0	0	0
Indonesia	Tebo	Proportion	2000	43.0	51.6	34.2
Indonesia	Tebo	Proportion	2017	36.9	43.8	30.5
Indonesia	Tebo	Count	2000	0	1	0
Indonesia	Tebo	Count	2017	0	0	0
Indonesia	Garut	Proportion	2000	37.2	44.0	30.5
Indonesia	Garut	Proportion	2017	40.8	46.0	35.7
Indonesia	Garut	Count	2000	0	0	0
Indonesia	Garut	Count	2017	0	0	0
Indonesia	Karanganyar	Proportion	2000	36.5	44.0	29.3
Indonesia	Karanganyar	Proportion	2017	30.3	36.2	25.1
Indonesia	Karanganyar	Count	2000	0	0	0
Indonesia	Karanganyar	Count	2017	0	0	0
Indonesia	Kota Blitar	Proportion	2000	39.1	47.8	31.2
Indonesia	Kota Blitar	Proportion	2017	32.5	38.9	26.4
Indonesia	Kota Blitar	Count	2000	0	0	0
Indonesia	Kota Blitar	Count	2017	0	0	0
Indonesia	Sanggau	Proportion	2000	40.8	49.4	32.1
Indonesia	Sanggau	Proportion	2017	37.4	46.0	28.7
Indonesia	Sanggau	Count	2000	0	0	0
Indonesia	Sanggau	Count	2017	0	0	0

Table 4: ORS by Unit *(continued)*

Country	Unit	Metric	Year	Mean	Upper	Lower
Indonesia	Tanah Bumbu	Proportion	2000	35.8	44.6	28.1
Indonesia	Tanah Bumbu	Proportion	2017	29.8	36.7	23.2
Indonesia	Tanah Bumbu	Count	2000	0	0	0
Indonesia	Tanah Bumbu	Count	2017	0	0	0
Indonesia	Palangka Raya	Proportion	2000	41.0	50.0	32.1
Indonesia	Palangka Raya	Proportion	2017	33.6	41.5	26.4
Indonesia	Palangka Raya	Count	2000	0	0	0
Indonesia	Palangka Raya	Count	2017	0	0	0
Indonesia	Tanggamus	Proportion	2000	35.9	43.3	28.4
Indonesia	Tanggamus	Proportion	2017	38.9	45.7	32.8
Indonesia	Tanggamus	Count	2000	0	0	0
Indonesia	Tanggamus	Count	2017	0	0	0
Indonesia	Tual	Proportion	2000	42.9	55.4	31.7
Indonesia	Tual	Proportion	2017	43.4	55.0	34.1
Indonesia	Tual	Count	2000	0	1	0
Indonesia	Tual	Count	2017	0	1	0
Indonesia	Nagekeo	Proportion	2000	52.3	61.6	43.0
Indonesia	Nagekeo	Proportion	2017	46.5	54.1	37.8
Indonesia	Nagekeo	Count	2000	1	1	0
Indonesia	Nagekeo	Count	2017	0	1	0
Indonesia	Teluk Wondama	Proportion	2000	45.2	57.3	34.0
Indonesia	Teluk Wondama	Proportion	2017	46.3	54.6	37.6
Indonesia	Teluk Wondama	Count	2000	0	1	0
Indonesia	Teluk Wondama	Count	2017	0	1	0
Indonesia	Kota Jayapura	Proportion	2000	42.3	52.7	31.1
Indonesia	Kota Jayapura	Proportion	2017	41.8	51.3	30.9
Indonesia	Kota Jayapura	Count	2000	0	1	0
Indonesia	Kota Jayapura	Count	2017	0	1	0
Indonesia	Rokan Hulu	Proportion	2000	36.2	45.9	30.0
Indonesia	Rokan Hulu	Proportion	2017	33.7	40.1	26.5
Indonesia	Rokan Hulu	Count	2000	0	0	0
Indonesia	Rokan Hulu	Count	2017	0	0	0
Indonesia	Luwu	Proportion	2000	39.5	47.5	32.2
Indonesia	Luwu	Proportion	2017	27.6	33.1	23.0
Indonesia	Luwu	Count	2000	0	0	0
Indonesia	Luwu	Count	2017	0	0	0
Indonesia	Toli-Toli	Proportion	2000	42.4	50.7	34.1
Indonesia	Toli-Toli	Proportion	2017	36.4	42.8	28.7
Indonesia	Toli-Toli	Count	2000	0	1	0
Indonesia	Toli-Toli	Count	2017	0	0	0
Indonesia	Muna	Proportion	2000	41.4	49.9	33.1
Indonesia	Muna	Proportion	2017	30.1	36.3	23.7
Indonesia	Muna	Count	2000	0	0	0
Indonesia	Muna	Count	2017	0	0	0
Indonesia	Minahasa Tenggara	Proportion	2000	35.8	45.7	27.3
Indonesia	Minahasa Tenggara	Proportion	2017	38.8	46.8	31.4
Indonesia	Minahasa Tenggara	Count	2000	0	0	0
Indonesia	Minahasa Tenggara	Count	2017	0	0	0
Indonesia	Pariaman	Proportion	2000	36.4	45.4	29.5

Table 4: ORS by Unit *(continued)*

Country	Unit	Metric	Year	Mean	Upper	Lower
Indonesia	Pariaman	Proportion	2017	32.8	39.4	25.1
Indonesia	Pariaman	Count	2000	0	0	0
Indonesia	Pariaman	Count	2017	0	0	0
Indonesia	Ogan Komering Ulu Timur	Proportion	2000	40.8	49.8	32.6
Indonesia	Ogan Komering Ulu Timur	Proportion	2017	41.4	48.1	34.8
Indonesia	Ogan Komering Ulu Timur	Count	2000	0	0	0
Indonesia	Ogan Komering Ulu Timur	Count	2017	0	0	0
Indonesia	Labuhanbatu Selatan	Proportion	2000	31.8	39.1	25.4
Indonesia	Labuhanbatu Selatan	Proportion	2017	30.4	36.5	24.3
Indonesia	Labuhanbatu Selatan	Count	2000	0	0	0
Indonesia	Labuhanbatu Selatan	Count	2017	0	0	0
Indonesia	Banda Aceh	Proportion	2000	37.5	49.3	28.8
Indonesia	Banda Aceh	Proportion	2017	31.6	39.3	24.1
Indonesia	Banda Aceh	Count	2000	0	0	0
Indonesia	Banda Aceh	Count	2017	0	0	0
Indonesia	Indramayu	Proportion	2000	37.5	43.9	30.7
Indonesia	Indramayu	Proportion	2017	40.5	45.3	34.6
Indonesia	Indramayu	Count	2000	0	0	0
Indonesia	Indramayu	Count	2017	0	0	0
Indonesia	Kebumen	Proportion	2000	36.1	44.0	28.4
Indonesia	Kebumen	Proportion	2017	31.5	37.6	25.6
Indonesia	Kebumen	Count	2000	0	0	0
Indonesia	Kebumen	Count	2017	0	0	0
Indonesia	Kota Kediri	Proportion	2000	39.2	48.1	31.2
Indonesia	Kota Kediri	Proportion	2017	32.9	38.8	27.0
Indonesia	Kota Kediri	Count	2000	0	0	0
Indonesia	Kota Kediri	Count	2017	0	0	0
Indonesia	Sekadau	Proportion	2000	40.8	49.2	32.1
Indonesia	Sekadau	Proportion	2017	36.7	44.8	28.0
Indonesia	Sekadau	Count	2000	0	0	0
Indonesia	Sekadau	Count	2017	0	0	0
Indonesia	Tanah Laut	Proportion	2000	35.0	43.3	26.8
Indonesia	Tanah Laut	Proportion	2017	29.6	36.0	23.3
Indonesia	Tanah Laut	Count	2000	0	0	0
Indonesia	Tanah Laut	Count	2017	0	0	0
Indonesia	Pulang Pisau	Proportion	2000	41.0	49.1	32.6
Indonesia	Pulang Pisau	Proportion	2017	33.2	40.5	26.5
Indonesia	Pulang Pisau	Count	2000	0	0	0
Indonesia	Pulang Pisau	Count	2017	0	0	0
Indonesia	Tulang Bawang Barat	Proportion	2000	38.5	46.6	31.6
Indonesia	Tulang Bawang Barat	Proportion	2017	40.5	47.8	34.5
[truncated: 594,347 more chars]
