# Supplementary material for: Mapping geographical inequalities in oral rehydration therapy coverage in low-income and middle-income countries, 2000–17
Source: Lancet Glob Health. 2020 Jul 22;8(8):e1038–60. doi: 10.1016/S2214-109X(20)30230-8 (PMC7388204; doi:10.1016/S2214-109X(20)30230-8)
Supplement: Supplementary appendix 1 [file mmc1.pdf]

THE LANCET
Global Health

Supplementary appendix 1

This appendix formed part of the original submission and has been peer reviewed. We post it as supplied by the authors.

Supplement to: Local Burden of Disease Diarrhoea Collaborators. Mapping geographical inequalities in oral rehydration therapy coverage in low-income and middle-income countries, 2000–17. *Lancet Glob Health* 2020; **8:** e1038–60.

Mapping geographic inequalities in oral rehydration therapy coverage in low- and middle-income countries, 2000–2017

Appendix

Contents

0.0 GATHER compliance **3**

1.0 Case definition of modelled outputs **3**

1.1 Oral rehydration solution (ORS) 3

1.2 Recommended home fluids (RHF) 3

1.3 Oral rehydration therapy (ORT) 3

2.0 Data **4**

2.1 Summary of included data sources 4

2.2 Aggregation to finest possible geography 4

2.3 Creation of pseudo-points within areal units 4

2.4 Assigning covariates to points 5

2.5 Administrative boundaries 5

3.0 Geostatistical model **5**

3.1 Model geographies 5

3.2 Ensemble covariate modelling via stacked generalisation 5

3.3 Geostatistical model 6

3.4 Priors 6

3.5 Mesh creation 7

3.6 Fitted parameters and estimate generation 7

4.0 Model vetting and validation **8**

4.1 Vetting stacker models and time trends 8

4.2 In-sample validation 8

4.3 Out-of-sample validation 8

5.0 Supplemental results **8**

5.1 Oral rehydration therapy coverage and number of children untreated 8

5.2 Annualised rate of change (AROC) in ORS, RHF, and ORT coverage 8

5.3 Geographic inequality 9

5.4 Correlation between ORS, RHF, and diarrhoeal mortality rate 9
5.5 Counterfactual analysis of deaths averted 9
6.0 Appendix figures **11**
7.0 Appendix tables **84**
8.0 Appendix references **156**
9.0 Author Contributions **158**

0.0 GATHER compliance

Please see appendix table 1 for discussion on how this study meets the conditions for GATHER guidelines.

1.0 Case definition of modelled outputs

1.1 Oral rehydration solution (ORS)

ORS was defined as a pre-packaged electrolyte solution containing glucose or another form of sugar or starch, as well as sodium, chloride, potassium, and bicarbonate or citrate. This included reduced osmolarity ORS (total osmolarity ≤250 mmol/l) and higher osmolarity ORS (up to 370 mmol/l).[1] Diarrhoea was defined as a child having had three or more abnormally loose or watery stools within a 24-hour period.

The modelled output was the *proportion of children under 5 years-old (under-5) with diarrhoea that received ORS*, and corresponded to the following survey questions that were asked about children who had diarrhoea in the previous 2–4.29 weeks (14 days–30 days):

- Did child receive a special packet called [local name for solution]?
- Did child receive a pre-packaged ORS liquid?
- This included any pre-made ORS solution, with designations such as *"oral rehydration solution", "ors", "sro", "serum from pharmacy", "soro", "suero", "pre-packaged", "special packet", "pedialyte"*.

1.2 Recommended home fluids (RHF)

RHF were defined as all possible home fluid alternatives, including but not limited to sugar-salt solution, cereal-salt solution, rice-water solution, and additional fluids, such as plain water, juice, tea, or rice water. The only solution that is consistently not included in the "recommended fluid" definition is soda.[1]

The modelled output was the *proportion of children under-5 with diarrhoea that received RHF*, and corresponded to the following survey questions that were asked about children who had had diarrhoea in the previous 2-4.29 weeks (14 days–30 days):

- Did child receive government-recommended home fluids?
- Did child receive any other fluid-based treatment [written in or listed]?
- This includes any home fluid that may increase hydration, with designations such as *"recommended home solution", "suero casero", "sugar and salt solution", "cereal-water", "sweetened", "salted", "rice water", "soup", "fruit juice", "yoghurt-based drink", "tea"*.

Given the broad range in RHF definitions between countries, in addition to the fact that survey questions change over time and between survey series, we performed an analysis to adjust all "non-standard" RHF survey questions to a "standard" question. RHF questions were classified as including phrasing that fell into categories of (1) recommended or acceptable home fluids, (2) sugar and salt solutions, (3) other home fluids, and/or (4) other liquid foods, for a total of 16 different possible definition combinations. The "standard" questionnaires were ones that asked only about category 1—recommended or acceptable home fluids—which were found most frequently across all surveys. We fit a logistic regression model to surveys across all LMICs, regressing coverage on definition, country-level fixed effects, and a natural cubic spline on survey year. Coverage reported by non-standard surveys was adjusted by the coefficient of the fixed effect for the non-standard definition in logit space. See appendix table 6 for the resulting RHF coverage adjustments.

1.3 Oral rehydration therapy (ORT)

ORT was defined as any form of oral rehydration treatment, including treatment with either ORS, RHF, or both. This also included rare cases when survey data could not be separated into ORS and RHF individual categories.[1] The modelled output was the *proportion of children under-5 with diarrhoea that received either ORS or RHF, or both*. It was modelled using the ORS and RHF survey data described above. It was also adjusted for differences in RHF survey questions using the methods described in appendix section 1.2. See appendix table 7 for the resulting ORT coverage adjustments.

2.0 Data

2.1 Summary of included data sources

This analysis selected 94 countries based on their Socio-demographic Index (SDI) published in the GBD[2] (see appendix table 2). The SDI is a measure of development that combines education, fertility, and income. We primarily aimed to include all countries in the middle, lower-middle, or low SDI quintiles, with several exceptions. Albania and Moldova were excluded despite middle SDI status due to geographic discontinuity with other included countries and lack of available survey data. Despite the requisite SDI quintiles for inclusion, we excluded the countries of Brazil, China, Cape Verde, Cuba, Western Sahara, French Guiana, Iran, Libya, Trinidad and Tobago, and Venezuela as they had no relevant data available. We also excluded island nations with fewer than one million inhabitants because they typically lacked survey data and did not have sufficient geographic continuity for a geospatial analytic approach to be advantageous over a national one; these nations were Fiji, Solomon Islands, Maldives, Vanuatu, Samoa, Saint Lucia, Kiribati, St. Vincent and the Grenadines, Grenada, Micronesia, Tonga, Seychelles, Dominica, Marshall Islands, and American Samoa.

The household surveys used to model ORS coverage can be found at http://ghdx.healthdata.org/record/ihme-data/lmic-oral-rehydration-therapy-coverage-geospatial-estimates-2000-2017 and visualised in appendix figures 3a–e. For a survey to be considered for this analysis, we required that it fit our country inclusion criteria outlined above, provided geography information more granular than the national level, had data collected during the time frame of 2000 to 2017, provided survey weights if the survey was not self-weighted, provided data on whether a child had diarrhoea in the weeks prior to the survey and data on whether children who had diarrhoea had received either ORS or RHF as treatment. After screening 1,611 sources that were tagged for diarrhoea relevance in the Global Health Data Exchange (GHDx)[3], 385 sources met all of the inclusion criteria.

Select data sources were excluded from the analysis due to: missing survey weights for areal data, non-geographically representative sampling, or untrustworthy data. Untrustworthy data were determined by the survey administrator or by systematic review, as seen in appendix figure 2 and further explained in section 4.1. After systematic review of all data sources, 378 sources were included in the final model dataset.

Of the 378 surveys included in the final ORS model, 99 were of non-standard type. A standard survey was defined as a survey that asked for any diarrhoea treatment type in the questionnaire, as this was the most common questionnaire structure. More specifically, these surveys allowed for an "other" treatment to be recorded if the child received a treatment not already listed. Non-standard surveys ranged from having two to 11 diarrhoea treatment options with no "other" write-in choice. Of the 99 non-standard surveys, four only had one diarrhoea treatment option; three of which only asked if the child had received, ORS while one only asked if the child had received zinc.

2.2 Aggregation to finest possible geography

We aggregated/summarised the individual-level microdata to the finest possible spatial resolution available—preferably, a latitude and longitude pair representing the location of the survey cluster/primary sampling unit. Where point-level referencing was not available, we matched survey microdata to the smallest polygon/areal unit possible. We calculated the effective sample size for each spatial aggregation (point and polygon) via the Kish approximation considering the underlying complex survey design.[6] After aggregation, the adjustments described below (sections 2.3–2.4) were applied.

2.3 Creation of pseudo-points within areal units

We created pseudo-points for areal data via a population-weighted resampling process as our desired model requires data of a single geometric type (e.g., latitude/longitude point). Specifically, we randomly generated 10,000 candidate points from within each areal unit using the WorldPop total population raster as a spatial distribution weight.[7] K-means clustering was performed to aggregate candidate points into the pseudo-points used for modelling. These pseudo-points were assigned analytical weights proportional to the number of candidate points that entered into the k-means cluster. Each pseudo-point generated by this process was assigned the ORS coverage observed from the survey for that polygon.

2.4 Assigning covariates to points

We compiled fifteen covariates that were indexed at the subnational level and had conceivable relationships with ORT: Access to cities, ratio of children dependents (age 0 to 14) to working adults (age 15 to 64), distance from rivers or lakes, night-time lights^{TV}, elevation, number of children under 5 per woman of childbearing age, population^{TV}, aridity^{TV}, urban or rural^{TV}, urban proportion of the location^{TV}, irrigation, number of people whose daily vitamin A needs could be met, prevalence of under-5 stunting^{TV}, prevalence of under-5 wasting^{TV}, and maternal education^{TV} (TV= time-varying covariates). We also included the Healthcare Access and Quality Index[8] and the proportion of pregnant women who received four or more antenatal care visits as national-level time-varying covariates. We filtered these covariates for multi-collinearity within each modelling region (see appendix figure 5) using variance inflation factor (VIF)[9] analysis using a threshold of VIF <3. Appendix figure 4 displays the spatial patterns of the covariates and appendix table 3 lists the source information. Appendix table 4 shows the final covariates selected for each region following VIF analysis. Once assembled, we conducted a spatial query to match covariate values spatially and temporally to our collection of points and pseudo points. For numerical stability, all covariates were centred and scaled to mean 0, with a standard deviation of 1.

2.5 Administrative boundaries

For this analysis we used shapefiles from the Database of Global Administrative Areas (GADM)[10] to define relevant country and subnational/administrative boundaries. We made slight adjustments to ensure proper nesting of administrative units, and made larger adjustments in the Democratic Republic of the Congo and India where collaborators in these countries had indicated mistakes in administrative boundaries in the shapefiles.

3.0 Geostatistical model

3.1 Model geographies

We stratified our data and analyses into 23 contiguous regions selected in order to improve computational tractability and to take advantage of the a-priori grouping based on country-level epidemiological profiles from the Global Burden of Disease study.[11] Appendix figure 5 shows the configuration of the regions. We ran individual models on eight countries, which allowed us to account for the unique spatial and temporal trends in ORS and RHF compared to their neighbouring countries; these were for Nigeria, the Democratic Republic of the Congo, Kenya, Ethiopia, Yemen, Zimbabwe, India, and Pakistan. For the same reason we split the South America region into a northern region (including Columbia, Guyana, and Suriname) and southern region (including Ecuador, Peru, Bolivia, and Paraguay). We did not include buffer areas around these regions in order to allow for border effects that could reasonably be driven by differences in national-level policies between neighbouring countries.

3.2 Ensemble covariate modelling via stacked generalisation

We used a stacked generalisation ensemble model framework to capture non-linear effects and complex interactions among our covariates.[4] For each region (see section 3.1), we fit three child models to our dataset: a generalised additive model (GAM), a penalised regression with the elastic net penalty, and a boosted regression tree (BRT). As described below in section 3.3, we use a spatio-temporal Gaussian process regression as the parent ensembler.

Parameters for the GAM (spline type and number of knots) were selected by expert prior with a maximum of 4 knots, and the lambda parameter for the elastic net regression was selected by cross validation. Initial hyperparameters for the BRT (namely tree complexity, learning rate, and number of trees) were selected using non-parametric Bayesian optimisation over a finite space, where the objective function was the negative mean absolute error of the BRT fit.[5] See appendix table 8 for all initial hyperparameters that were selected by modelling region.

Each child model was fit using five-fold cross validation to reduce overfitting, and the out-of-sample predictions across the child model hold-outs were compiled into a single set of model predictions. Additionally, each child model was fit on 100% of the data and a full set of in-sample predictions were created. The out-of-sample predictions per child model were fed to the parent geostatistical model (see section 3.3 below) as covariates for fitting while the in-sample predictions from the child models are used during the parent model's prediction step.

3.3 Geostatistical model

Binomial count data are modelled within a Bayesian hierarchical modelling framework using a logit link function and a spatially- and temporally-explicit hierarchical generalised linear regression model to estimate the point prevalence (or coverage) of ORS, RHF, or ORT in each modelling region (see section 3.1). Our model was constructed as follows:

$$C_i | p_i,\ N_i \sim \text{Binomial}(p_i,\ N_i)$$

$$\text{logit}(p_i) = \beta_0 + \boldsymbol{X_i \beta} + \epsilon_{GP_i} + \epsilon_{ctry_i} + \epsilon_{study_i} + \epsilon_i$$

$$\sum \boldsymbol{\beta} = 1$$

$$\epsilon_{ctry_i} \sim \text{N}(0, \sigma^2_{ctry})$$

$$\epsilon_i \sim \text{N}(0, \sigma^2_{nug})$$

$$\boldsymbol{\epsilon_{GP}} | \boldsymbol{\Sigma}_{\text{space}},\ \boldsymbol{\Sigma}_{time} \sim \text{GP}(0,\ \boldsymbol{\Sigma}_{\text{space}} \otimes \boldsymbol{\Sigma}_{time})$$

$$\boldsymbol{\Sigma}_{\text{space}} = \sigma^2 \frac{2^{1-\nu}}{\Gamma(\nu)} \times \left(\frac{\sqrt{8}}{\rho_s} \boldsymbol{D}\right)^{\nu} \times \text{K}_\nu \left(\frac{\sqrt{8}}{\rho_s} \boldsymbol{D}\right)$$

$$\Sigma_{time_{j,k}} = \rho^{|t_k - t_j|}.$$

For each region, we modelled the number of under-5 children at location-time, i, among a sample size, N_i, with diarrhoea who received ORS, RHF, and ORT, respectively, as binomial count data, C_i. The counts, C_i, probabilities, p_i, predictions from the three child models, $\boldsymbol{X_i}$, and residual terms, ϵ_* , are all indexed at a space-time coordinate. The term p_i represents both the annual proportion and the annual probability that an individual child will receive ORS, RHF, or ORT, respectively, given the child resides at that particular location. The logit of annual coverage, logit(p_i), was modelled as a linear combination of the three child models, $\boldsymbol{X_i}$; a correlated spatio-temporal error term, ϵ_{GP_i}; and an independent error term, ϵ_i. Coefficients, $\boldsymbol{\beta}$, on the child models represent their respective predictive weighting in the mean logit link and are constrained to sum to one. ϵ_{ctry_i} is a country random effect. The country random effect was not used in individual country models, with the exception of India where we set this term to be state-level random effects. $\boldsymbol{\epsilon_{GP}}$, is modelled as a three-dimensional Gaussian process in space-time centred at zero and with a covariance matrix constructed from a Kroenecker product of spatial and temporal covariance kernels. The spatial covariance, $\boldsymbol{\Sigma}_{\text{space}}$, is modelled using a Matérn covariance function,[12] and temporal covariance, $\boldsymbol{\Sigma}_{\text{time}}$, as an autoregressive order 1 (AR1) function represented in the model with 18 annual knots.

This approach leveraged the data's residual correlation structure to more accurately predict coverage estimates for locations with no data, while also propagating the dependence in the data through to uncertainty estimates.[13] The posterior distributions were fit using computationally efficient and accurate approximations in R-INLA[14,15] (integrated nested Laplace approximation) with the stochastic partial differential equations (SPDE)[16] approximation to the Gaussian process residuals.

3.4 Priors

The following priors were used:

- $\beta_0 \sim N(\mu = 0, \sigma^2 = 1000)$,
- $\boldsymbol{\beta} \sim N(\boldsymbol{\mu}, \Sigma)$,
    - $\boldsymbol{\mu} = (\frac{1}{3}, \frac{1}{3}, \frac{1}{3})'$
    - $\Sigma = 1{,}000 * I_{3\,x\,3}$
- $log\left(\frac{1+\rho}{1-\rho}\right) \sim N(\mu = 2, \sigma^2 = 1/(1.2^2))$,

- $\left(\frac{1}{\sigma^2_{country}}\right) \sim gamma(\alpha = 4, \gamma = 4)$,
- $\left(\frac{1}{\sigma^2_{study}}\right) \sim gamma(\alpha = 1, \gamma = 0.00005)$,
- $\left(\frac{1}{\sigma^2_{nugget}}\right) \sim gamma(\alpha = 1, \gamma = 0.00005)$,

We used a "penalized complexity" prior[17] for the marginal standard deviation, σ, and spatial range, ρ_s, of the spatio-temporal Gaussian process, $\mathbf{\Sigma}_{\text{space}}$. The priors for these parameters were specified such that Pr(σ > 3) = 0.05 and $\Pr(\rho_s < X) = 0.05$, where X was 5% of the max extent of the mesh for each modelling region as indicated below:

Central sub-Saharan Africa - **2.04**
Eastern sub-Saharan Africa - **2.33**
Horn of Africa - **2.07**
Southern sub-Saharan Africa - **1.65**
Western sub-Saharan Africa - **3.06**
Mexico, Central America, and the Caribbean - **3.47**
Northern South America - **1.98**
Southern South America - **2.48**
Central Asia – **1.98**
Middle East - **2.63**
South Asia - **1.81**
Southeast Asia - **1.98**
Malay Archipelago - **3.68**
North Africa Middle East - **2.10**
Ethiopia - **1.35**
India - **2.06**
Kenya - **1.08**
Mongolia - **2.20**
Nigeria - **1.19**
Democratic Republic of Congo - **1.55**
Pakistan - **1.42**
Yemen - **1.23**
Zimbabwe - **0.97**

The mean (μ) and variance (σ^2) parameters for the hyperpriors selected by INLA for the meshes in each region can be found in appendix table 5. In our parameterisation we represent α and γ in the *gamma* distribution as rate and shape, respectively. The starting set of hyperparameters were selected using INLA defaults, as well as a previously performed sensitivity analysis.[18] From this initial set, we modified the prior on the variance of the country random effects to allow high levels of variation between countries due to the strong effect that national-level policies may have on ORS and RHF coverage.

3.5 Mesh creation

We constructed the finite elements mesh for the stochastic partial differential equation approximation to the Gaussian process regression using a simplified polygon boundary (in which coastlines and complex boundaries were smoothed) for each of the regions within our model. This paper uses an improved mesh that is constructed on the S2 domain. This allows distance to be calculated along the sphere instead of using Euclidean distance between latitude and longitude coordinates. This mesh also generates denser vertices in data rich areas. We set the minimum triangle edge length to 25 kilometres, the maximum triangle length to 1,000 kilometres, with the mesh extending 500 kilometres past the region's boundary. An example of finite elements mesh-constructed for Southern sub-Saharan Africa mesh can be found in appendix figure 6.

3.6 Fitted parameters and estimate generation

Fitted parameters and hyperparameters, as well as their 95% credible intervals are shown by indicator and region in appendix table 5. All estimates were generated by taking 250 draws from the posterior distribution. For estimates at the 5 × 5-km grid-cell level, these draws were used directly to generate estimates and uncertainty. We generated 95% credible intervals around the mean of our estimates (appendix figures 7–15) by taking the 2.5% and 97.5% quantiles of each of the draws, at the grid-cell or administrative level.

To aggregate our results to second administrative-level units (e.g., districts, counties) for each draw, we fractionally assigned each grid cell to any intersecting unit by examining the starting area of the grid cell and the relative areas of the resulting geometric intersections, and took population-weighted averages of grid cells assigned to each unit. To aggregate to first administrative-level units (e.g., states, provinces), we computed population-weighted averages of nested second administrative units. To aggregate to country levels, we computed population-weighted averages of nested first administrative units.

4.0 Model vetting and validation

4.1 Vetting stacker models and time trends

For each intermediate model and for final models, we created line plots of our estimates for each of the stacking models and the final INLA model including uncertainty overlaid on the input data. We created and reviewed these plots for each country and for each first administrative unit. These plots allowed us to (1) identify unreasonable time trends caused by covariates in the absence of data, allowing us to remove those covariates; (2) identify outlier data caused by non-representative surveys or mistakes in data extraction; (3) identify countries with unique patterns in ORS coverage, which deserved individual country analyses; and (4) understand how the individual stacking models and final geostatistical model each contributed to the spatial and temporal estimates.

4.2 In-sample validation

To explore the in-sample validity of our models, we plotted our predictions vs. the observed data by modelling regions and by year at the country-level, first administrative-level, and second administrative-level aggregations (appendix figures 16-33). We also calculated mean error (ME, or bias), root-mean-squared-error (RMSE, which summarises total variance), and 95% coverage of our predictive intervals (the proportion of observed in-sample data that fall within our predicted 95% credible intervals). The in-sample fit statistics are shown in appendix tables 9a-f, 11a-f, and 13a-f for ORS, RHF, and ORT models, respectively.

4.3 Out-of-sample validation

We examined the predictive validity of our modelling strategy using five-fold out-of-sample cross-validation. Folds were created by randomly assigning entire second administrative units, stratified by region, to one of five folds. For each modelling region, we ran the entire modelling process once per fold, in addition to the full in-sample runs described above, generating a complete set of out-of-sample predictions. Using these out-of-sample predictions, we then calculated mean error (ME, or bias), root-mean-squared-error (RMSE, which summarises total variance), and 95% coverage of our predictive intervals (the proportion of observed out-of-sample data that fall within our predicted 95% credible intervals) aggregated to the spatial holdout level. Appendix figures 34–51 show out-of-sample prediction vs. observed data. Appendix tables 10a-f, 12a-f, and 14a-f summarise out-of-sample statistics for ORS, RHF, and ORT models, respectively.

5.0 Supplemental results

5.1 Oral rehydration therapy coverage and number of children untreated

Appendix figures 7–15 provide additional visualisations of estimates of the proportion of under-5 children with diarrhoea that received treatment and mean number of under-5 children untreated with ORS, RHF, and ORT, respectively, including mean estimates, and upper and lower uncertainty interval estimates. Extended data tables 1-6 include country-level and second administrative level (unit) estimates of diarrhoeal mortality rate and ORS, RHF, and ORT coverage in 2017, including mean estimates, and upper and lower uncertainty interval estimates.

5.2 Annualised rate of change (AROC) in ORS, RHF, and ORT coverage

We computed the AROC in ORS, RHF, and ORT coverage from 2000 to 2017. Appendix figures 52–54 show estimates of the AROC in the proportion of under-5 children with diarrhoea that received ORS, RHF, and ORT, respectively, from 2000 to 2017 including mean estimates, and upper and lower uncertainty interval estimates.
For each grid cell, we calculated AROC by log-transforming our posterior mean coverage estimates from each year from 2000 to 2017, $coverage_{i,yr}^{l}$, and determining the rate of change between each pair of adjacent years (beginning with yr=2001):

$$AROC_{i,yr}^{l} = coverage_{i,yr}^{l} - coverage_{i,yr-1}^{l}.$$

Then, we took a weighted average AROC across the study period, placing more weight on more recent AROCs, and to calculate grid-cell-level AROCs. We defined weights as:

$$w_{yr} = \frac{(yr-2000)^{\gamma}}{\sum_{2001}^{2017}(yr-2000)^{\gamma}},$$

whereby different weights can be given to years across the study period by selecting the appropriate γ. For this analysis, we weighted years by relative amount of data. We then calculated grid-cell-level weighted-AROC:

$$AROC_i = \sum_{2001}^{2017} w_{yr} AROC_{i,yr}^{l}.$$

5.3 Geographic inequality

We created national-level geographic inequality metrics using the Gini coefficient. The Gini coefficient assesses the magnitude of disparity between the richest and poorest individuals.[19] Equality corresponds to wealth uniformly distributed across the population and inequality corresponds to a small number of individuals possessing the majority of the wealth. The Gini coefficient can be calculated directly from the Lorenz curve, which sorts individuals by their income and plots cumulative percentages of individuals against their corresponding fraction of wealth. For the purposes of this study, "wealth" is defined as ORS, RHF, and ORT coverage for each second administrative unit. The Gini coefficient is then calculated as one minus twice the area under the Lorenz curve. An alternative formulation of the Gini coefficient, which gives the same result, calculates the relative mean absolute difference in wealth, and then observes that the Gini coefficient is half the resulting quantity. If x_i is the wealth of the ith individual (out of n individuals), the Gini coefficient, G, is given as:

$$G = \frac{\sum_{i=1}^{n} \sum_{j=1}^{n} |x_i - x_j|}{2n \sum_{i=1}^{n} x_i}$$

We also quantified geographic inequalities within countries over time as both the relative and absolute difference between ORS, RHF, and ORT coverage, respectively, in each second administrative unit and its country mean using the following formulas:

$$Absolute\ inequality = coverage_{unit} - coverage_{country}$$

$$Relative\ inequality = \frac{coverage_{unit} - coverage_{country}}{coverage_{country}}$$

Appendix figure 55 shows a map of relative geographic inequality in the proportion of under-5 children with diarrhoea that received ORS, RHF, and ORT for 2000 and 2017. Appendix figures 56 and 57 show relative and absolute deviation from the mean for RHF and ORT, respectively, corresponding to Figure 3 in the main text.

5.4 Correlation between ORS, RHF, and diarrhoeal mortality rate

We computed the correlation over time from 2000 to 2017 between ORS and diarrhoeal mortality rates, RHF and diarrhoeal mortality rates, and ORS and RHF coverage at each second administrative unit using Spearman's rho statistic. Appendix figure 58a shows estimates of the correlation between mean ORS and RHF from 2000 to 2017. Appendix figure 58b shows estimates of the correlation between mean ORS and diarrhoeal mortality rates from 2000 to 2017. Appendix figure 58c shows estimates of the correlation between mean RHF and diarrhoeal mortality rates from 2000 to 2017.

5.5 Counterfactual analysis of deaths averted

We performed a counterfactual analysis of the estimated number of diarrhoeal deaths averted that were associated with ORS scale-up from 2000 to 2017. For this analysis, we used our estimates of the number of diarrhoeal deaths in 2000 and 2017 from Reiner *et al.*[20] and our estimated ORS coverage in 2000 and 2017. We also used previous estimates from a systematic review that demonstrated that in locations with 75% ORS coverage, 69% (95% uncertainty interval, 51–80) of diarrhoeal deaths could be averted.[1] Using these estimates, we assumed that the relative risk (RR) of death with ORS treatment was 1 – 0.69 (0.51–0.8) = 0.31 (0.20–0.49). Additionally, we performed a sensitivity analysis of these RR estimates by halving and doubling the estimated RR. We then made the calculations for this analysis using the following formulas:

$$RR_{death\ if\ no\ ORS\ given} = \frac{1}{0.31} = 3.2$$

$$PAF_{2000} = \frac{(1 - coverage_{2000}) * (RR_{death\ if\ no\ ORS\ given} - 1)}{((1 - coverage_{2000}) * (RR_{death\ if\ no\ ORS\ given} - 1) + 1)}$$

$$PAF_{2017} = \frac{(1 - coverage_{2017}) * (RR_{death\ if\ no\ ORS\ given} - 1)}{((1 - coverage_{2017}) * (RR_{death\ if\ no\ ORS\ given} - 1) + 1)}$$

$$Number\ averted = Deaths_{2017} * (\frac{1 - PAF_{2017}}{1 - PAF_{2000}} * PAF_{2000} - PAF_{2017})$$

$$Rate\ averted = \frac{Number\ averted}{Total\ number\ of\ children\ under\ 5} * 1000$$

Appendix figures 59 and 60 show the results of the sensitivity analysis. Appendix figure 59 shows estimated deaths averted if the RR were halved (RR = 1.6), and appendix figure 60 shows estimated deaths averted if the RR were doubled (RR = 6.4).

6.0 Appendix figures

Appendix Figure 1. Geospatial modelling flowchart 14

Appendix Figure 2. Data inclusion flowchart 16

Appendix Figure 3a–e. ORS use data availability by type and country 18

Appendix Figure 4. Covariates used in mapping 24

Appendix Figure 5. Map of modelling regions 26

Appendix Figure 6. Finite elements mesh 27

Appendix Figure 7. Posterior means and 95% uncertainty intervals for ORS coverage at the 5 × 5-km grid-cell level for 2017 28

Appendix Figure 8. Posterior means and 95% uncertainty intervals for RHF coverage at the 5 × 5-km grid-cell level for 2017 29

Appendix Figure 9. Posterior means and 95% uncertainty intervals for ORT coverage at the 5 × 5-km grid-cell level for 2017 30

Appendix Figure 10. Posterior means and 95% uncertainty intervals for ORS coverage at the second-administrative-unit level for 2017 31

Appendix Figure 11. Posterior means and 95% uncertainty intervals for RHF coverage at the second-administrative-unit level for 2017 32

Appendix Figure 12. Posterior means and 95% uncertainty intervals for ORT coverage at the second-administrative-unit level for 2017 33

Appendix Figure 13. Posterior means and 95% uncertainty intervals for ORS coverage at the first-administrative-unit level for 2017 34

Appendix Figure 14. Posterior means and 95% uncertainty intervals for RHF coverage at the first-administrative-unit level for 2017 35

Appendix Figure 15. Posterior means and 95% uncertainty intervals for ORT coverage at the first-administrative-unit level for 2017 36

Appendix Figure 16. In-sample validation plot for ORS by country and region 38

Appendix Figure 17. In-sample validation plot for ORS by first administrative unit and region 39

Appendix Figure 18. In-sample validation plot for ORS by second administrative unit and region 40

Appendix Figure 19. In-sample validation plot for ORS by country and year 41

Appendix Figure 20. In-sample validation plot for ORS by first administrative unit and year 42

Appendix Figure 21. In-sample validation plot for ORS by second administrative unit and year 43

Appendix Figure 22. In-sample validation plot for RHF by country and region 44

Appendix Figure 23. In-sample validation plot for RHF by first administrative unit and region 45

Appendix Figure 24. In-sample validation plot for RHF by second administrative unit and region 46

Appendix Figure 25. In-sample validation plot for RHF by country and year 47
Appendix Figure 26. In-sample validation plot for RHF by first administrative unit and year 48
Appendix Figure 27. In-sample validation plot for RHF by second administrative unit and year 49
Appendix Figure 28. In-sample validation plot for ORT by country and region 50
Appendix Figure 29. In-sample validation plot for ORT by first administrative unit and region 51
Appendix Figure 30. In-sample validation plot for ORT by second administrative unit and region 52
Appendix Figure 31. In-sample validation plot for ORT by country and year 53
Appendix Figure 32. In-sample validation plot for ORT by first administrative unit and year 54
Appendix Figure 33. In-sample validation plot for ORT by second administrative unit and year 55
Appendix Figure 34. Out-of-sample validation plot for ORS by country and region 57
Appendix Figure 35. Out-of-sample validation plot for ORS by first administrative unit and region 58
Appendix Figure 36. Out-of-sample validation plot for ORS by second administrative unit and region 59
Appendix Figure 37. Out-of-sample validation plot for ORS by country and year 60
Appendix Figure 38. Out-of-sample validation plot for ORS by first administrative unit and year 61
Appendix Figure 39. Out-of-sample validation plot for ORS by second administrative unit and year 62
Appendix Figure 40. Out-of-sample validation plot for RHF by country and region 63
Appendix Figure 41. Out-of-sample validation plot for RHF by first administrative unit and region 64
Appendix Figure 42. Out-of-sample validation plot for RHF by second administrative unit and region 65
Appendix Figure 43. Out-of-sample validation plot for RHF by country and year 66
Appendix Figure 44. Out-of-sample validation plot for RHF by first administrative unit and year 67
Appendix Figure 45. Out-of-sample validation plot for RHF by second administrative unit and year 68
Appendix Figure 46. Out-of-sample validation plot for ORT by country and region 69
Appendix Figure 47. Out-of-sample validation plot for ORT by first administrative unit and region 70
Appendix Figure 48. Out-of-sample validation plot for ORT by second administrative unit and region 71
Appendix Figure 49. Out-of-sample validation plot for ORT by country and year 72
Appendix Figure 50. Out-of-sample validation plot for ORT by first administrative unit and year 73
Appendix Figure 51. Out-of-sample validation plot for ORT by second administrative unit and year 74
Appendix Figure 52. ORS annualised rate of change, 2000–2017 75
Appendix Figure 53. RHF annualised rate of change, 2000–2017 76
Appendix Figure 54. ORT annualised rate of change, 2000–2017 77

Appendix Figure 55. Relative geographic inequality maps of ORS, RHF, and ORT coverage by second administrative unit, 2017 78

Appendix Figure 56. Geographic inequalities within countries in the proportion of under-5 children with diarrhoea who received recommended home fluids (RHF), 2000 and 2017. 79

Appendix Figure 57. Geographic inequalities within countries in the proportion of under-5 children with diarrhoea who received oral rehydration therapy (ORT), 2000 and 2017. 80

Appendix Figure 58. Correlation between ORS, RHF, and diarrhoeal mortality rates by second administrative unit, 2017 81

Appendix Figure 59. Sensitivity analysis of deaths averted by ORS scale-up from 2000 to 2017: halving the estimated relative risk of death without ORS treatment (RR = 1.6) 82

Appendix Figure 60. Sensitivity analysis of deaths averted by ORS scale-up from 2000 to 2017: doubling the estimated relative risk of death without ORS treatment (RR = 6.4) 83

Appendix Figure 1. Geospatial modelling flowchart

The geospatial modelling process consists of four sections. First (in blue), we compile all available survey data that can be referenced to a coordinate/point (e.g., survey cluster) or small polygon unit and calculate ORS, RHF, and ORT coverage at the respective level (sections 1.0 and 2.0). Data matched to polygons are resampled into pseudo-points using a k-means clustering algorithm (section 2.3). Covariates are subsequently merged to the points and pseudo-points via a spatial join (section 2.4). Second (green), we use the point data and their associated covariates and a stacked generalisation ensemble model (section 3.2). The child models—boosted regression trees, generalised additive models, and elastic net regression—are fit using a five-fold cross validation process (section 3.3). The cross-validated predictions from each model then serve as the covariate values for the main/parent model (Spatio-temporal GPR model) (section 3.3). The predictions from when the child models are fit on all the data (rather than 4/5^{ths} implied by the cross-validation) are then used to create posterior predictions of ORS, RHF, and ORT coverage in a 5 × 5-km grid for the years 2000–2017 (section 3.3). Finally (orange), we aggregate our estimates to first and second administrative units (section 3.6).

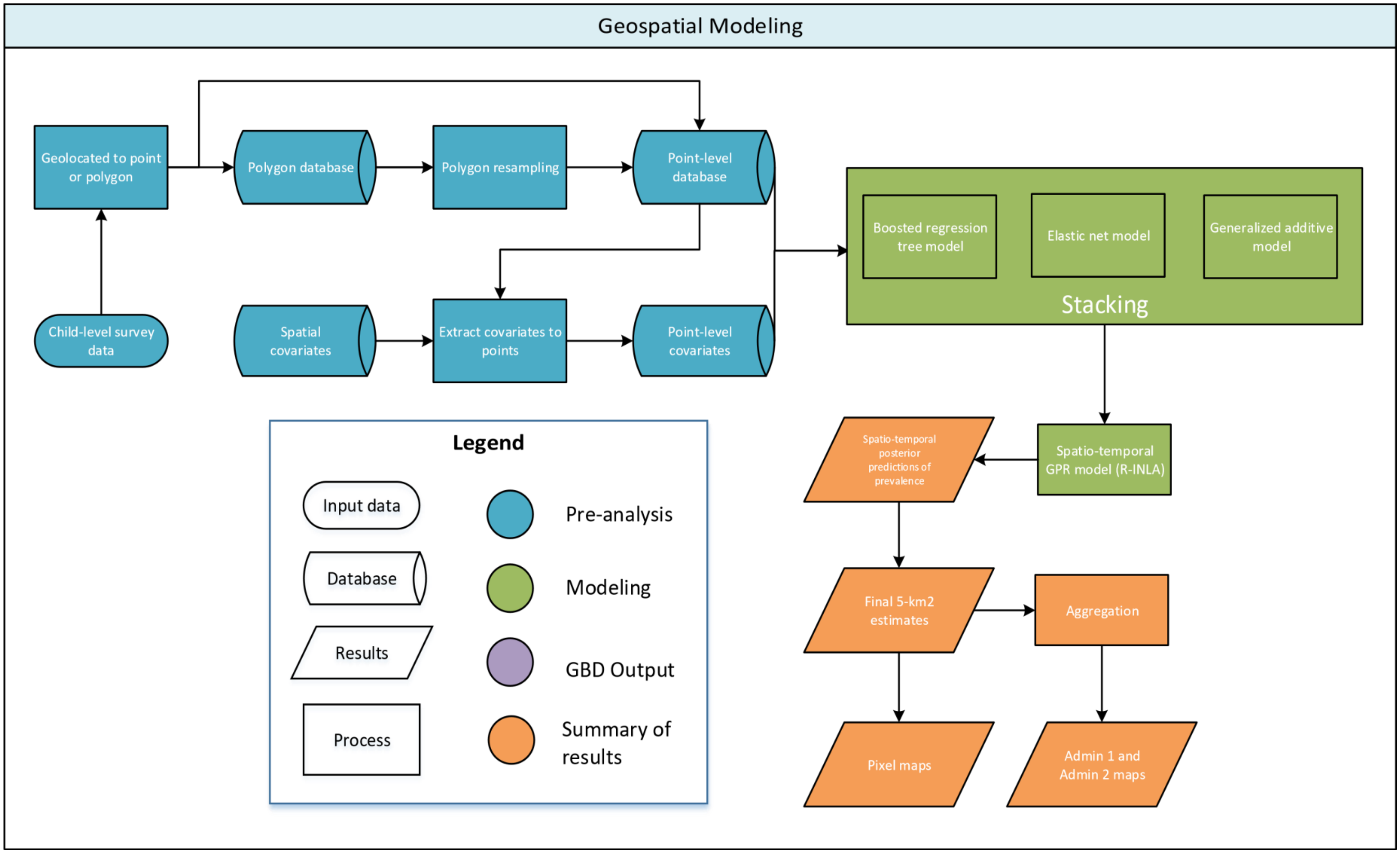

Geospatial Modeling
Geolocated to point or polygon
Polygon database
Polygon resampling
Point-level database
Child-level survey data
Spatial covariates
Extract covariates to points
Point-level covariates
Boosted regression tree model
Elastic net model
Generalized additive model
Stacking
Spatio-temporal GPR model (R-INLA)
Spatio-temporal posterior predictions of prevalence
Final 5-km2 estimates
Aggregation
Pixel maps
Admin 1 and Admin 2 maps
Legend
Input data
Database
Results
Process
Pre-analysis
Modeling
GBD Output
Summary of results

Appendix Figure 2. Data inclusion flowchart

The data vetting process used in this analysis includes multiple steps. First (in green), we compiled all sources tagged as relevant to diarrhoea from the GHDx and extracted data sources that met all of our inclusion criteria (section 2.1). If a data source did not meet all of our inclusion criteria, that source was excluded (orange). Second (light blue), the data was processed according to the geospatial modelling framework (sections 2.1– 4.3, appendix figure 1). Third (dark blue), line plots were created for each country and independently scrutinised for data quality over time (section 4.1). Next, each survey that was flagged as off-trend from the line plots was reviewed. If a survey was found to have a data processing mistake, the mistake was fixed and the process started over. Additionally, if there seemed to be a reasonable explanation for why a survey was off trend (e.g., a natural disaster that could potentially explain an uptick in diarrhoea prevalence) then that survey was included in the final dataset. If a survey did not have any processing errors and there was no reasonable explanation for why it was off-trend, that survey was then excluded from the final dataset.

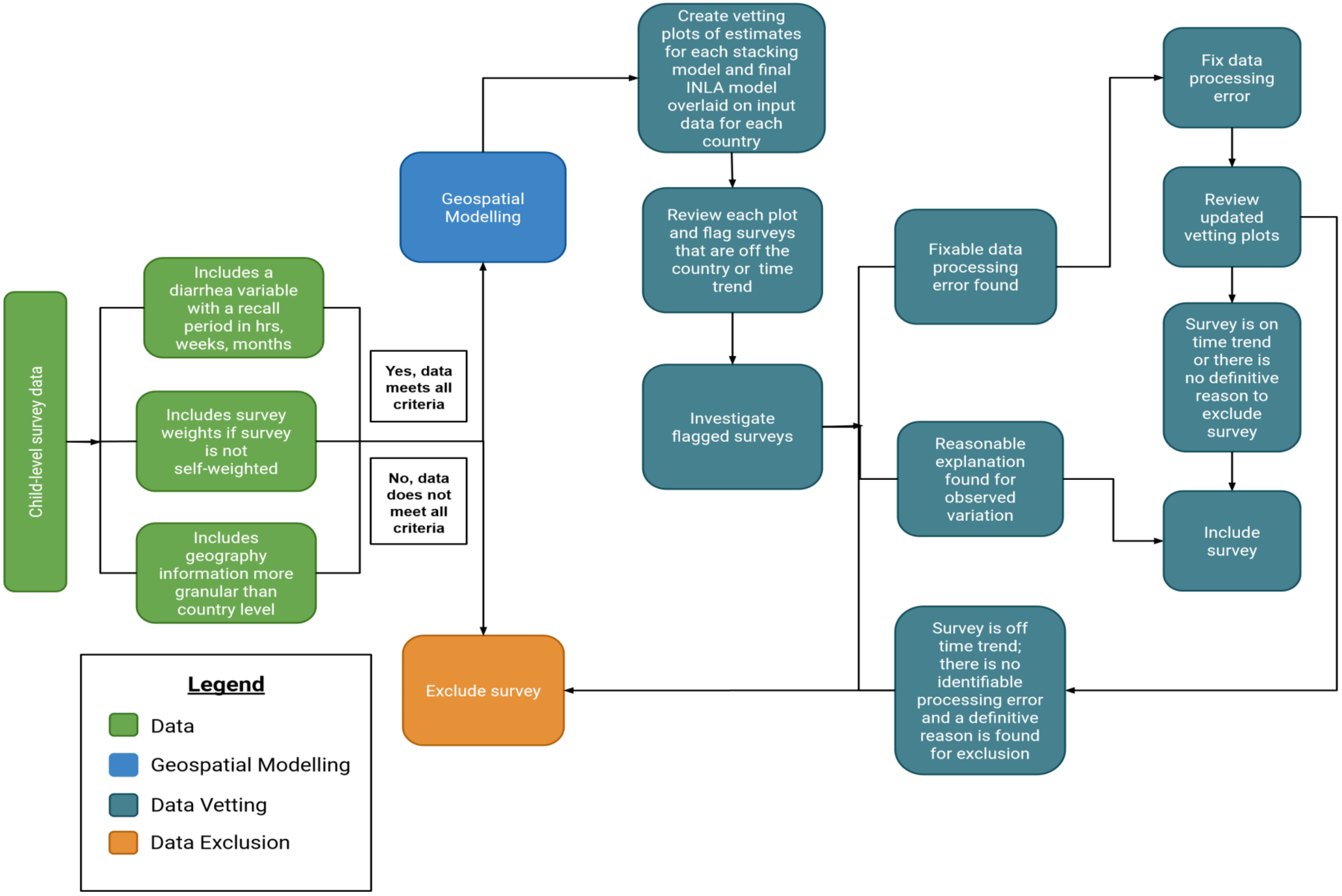
Child-level survey data
Includes a diarrhea variable with a recall period in hrs, weeks, months
Includes survey weights if survey is not self-weighted
Includes geography information more granular than country level
Yes, data meets all criteria
No, data does not meet all criteria
Geospatial Modelling
Exclude survey
Create vetting plots of estimates for each stacking model and final INLA model overlaid on input data for each country
Review each plot and flag surveys that are off the country or time trend
Investigate flagged surveys
Fixable data processing error found
Reasonable explanation found for observed variation
Survey is off time trend; there is no identifiable processing error and a definitive reason is found for exclusion
Fix data processing error
Review updated vetting plots
Survey is on time trend or there is no definitive reason to exclude survey
Include survey
Legend
Data
Geospatial Modelling
Data Vetting
Data Exclusion

Appendix Figure 3a–e. ORS use data availability by type and country

All data are shown by country and year of survey and mapped at their corresponding geopositioned coordinate or area. In the left panel, the total number of points and polygons (areal) for each country are plotted by data source, type, and sample size. Sample size represents the number of individual microdata records for each survey. In the right panel, mean ORS coverage for the input coordinate or area are mapped. Figure **a)** shows ORS data availability in Africa by type and country from 2000–2017. Figure **b)** shows ORS data availability in Latin America and the Caribbean by type and country from 2000–2017. Figure **c)** shows ORS data availability in Southeast Asia by type and country from 2000–2017. Figure **d)** shows ORS data availability in south Asia by type and country from 2000–2017. Figure **e)** shows ORS data availability in the Middle East and central Asia by type and country from 2000–2017. Since the ORS, RHF, and ORT data sources and survey questionnaires were very similar, we only present ORS data coverage.

a)

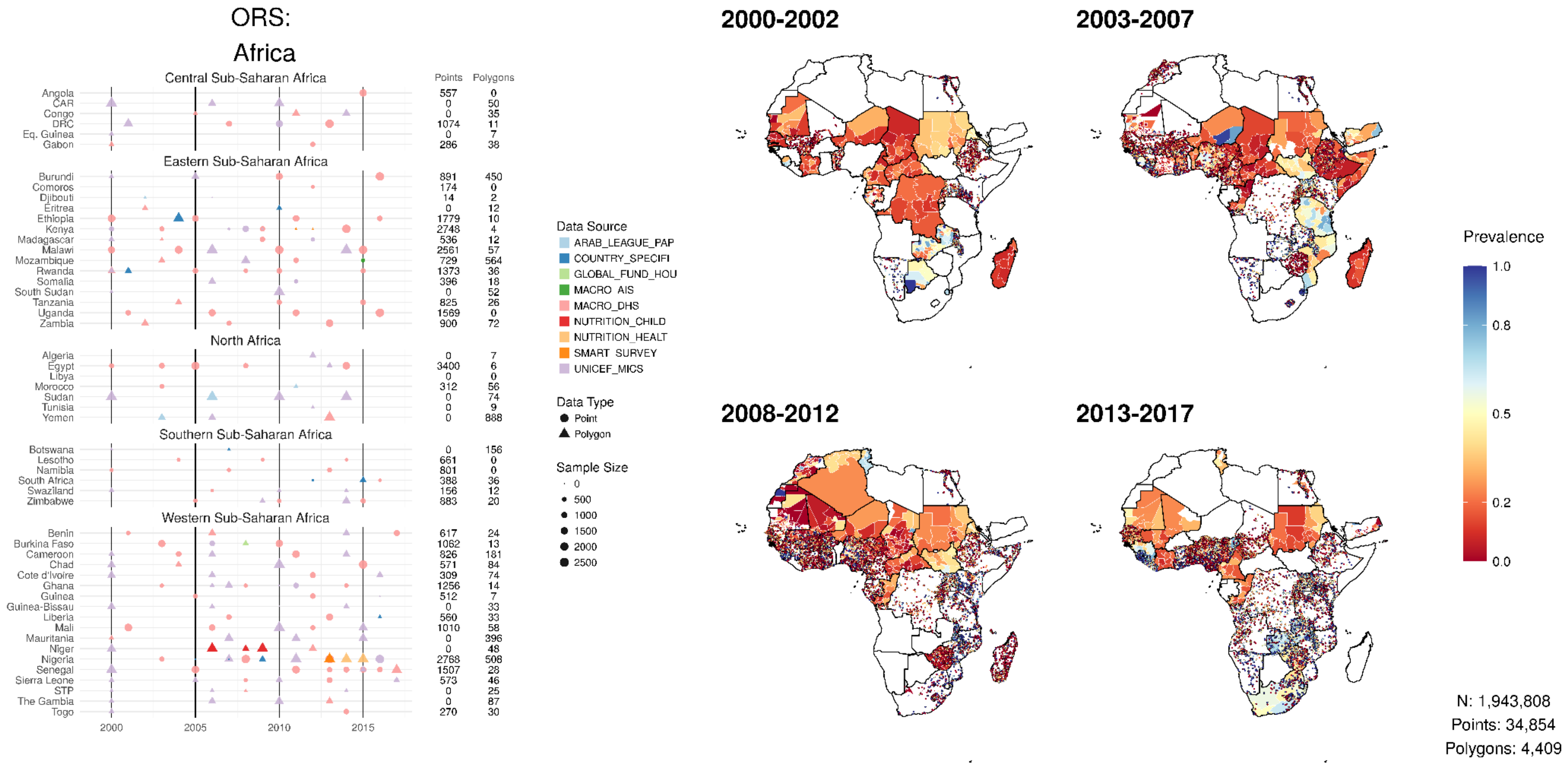

ORS:
Africa
Central Sub-Saharan Africa
Points
Polygons
Angola 557 0
CAR 0 50
Congo 0 35
DRC 1074 11
Eq. Guinea 0 7
Gabon 286 38
Eastern Sub-Saharan Africa
Burundi 891 450
Comoros 174 0
Djibouti 14 2
Eritrea 0 12
Ethiopia 1779 10
Kenya 2748 4
Madagascar 536 12
Malawi 2561 57
Mozambique 729 564
Rwanda 1373 36
Somalia 396 18
South Sudan 0 52
Tanzania 825 26
Uganda 1569 0
Zambia 900 72
North Africa
Algeria 0 7
Egypt 3400 6
Libya 0 0
Morocco 312 56
Sudan 0 74
Tunisia 0 9
Yemen 0 888
Southern Sub-Saharan Africa
Botswana 0 156
Lesotho 661 0
Namibia 801 0
South Africa 388 36
Swaziland 156 12
Zimbabwe 883 20
Western Sub-Saharan Africa
Benin 617 24
Burkina Faso 1062 13
Cameroon 826 181
Chad 571 84
Cote d'Ivoire 309 74
Ghana 1256 14
Guinea 512 7
Guinea-Bissau 0 33
Liberia 560 33
Mali 1010 58
Mauritania 0 396
Niger 0 48
Nigeria 2768 508
Senegal 1507 28
Sierra Leone 573 46
STP 0 25
The Gambia 0 87
Togo 270 30
2000
2005
2010
2015
Data Source
ARAB_LEAGUE_PAP
COUNTRY_SPECIFI
GLOBAL_FUND_HOU
MACRO_AIS
MACRO_DHS
NUTRITION_CHILD
NUTRITION_HEALT
SMART_SURVEY
UNICEF_MICS
Data Type
Point
Polygon
Sample Size
0
500
1000
1500
2000
2500
2000-2002
2003-2007
2008-2012
2013-2017
Prevalence
1.0
0.8
0.5
0.2
0.0
N: 1,943,808
Points: 34,854
Polygons: 4,409

b)

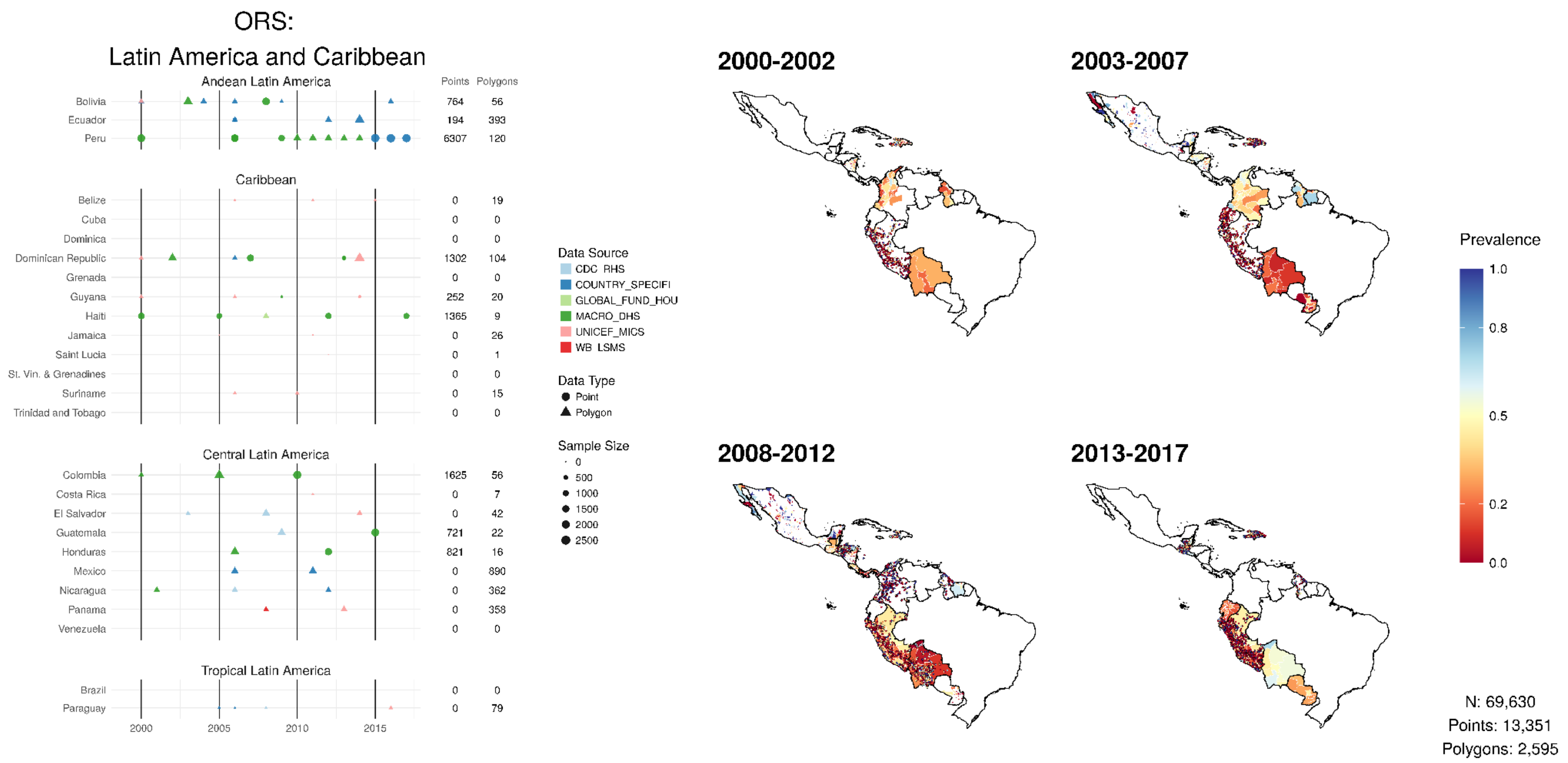
ORS:
Latin America and Caribbean
Andean Latin America
Points
Polygons
Bolivia 764 56
Ecuador 194 393
Peru 6307 120
Caribbean
Belize 0 19
Cuba 0 0
Dominica 0 0
Dominican Republic 1302 104
Grenada 0 0
Guyana 252 20
Haiti 1365 9
Jamaica 0 26
Saint Lucia 0 1
St. Vin. & Grenadines 0 0
Suriname 0 15
Trinidad and Tobago 0 0
Central Latin America
Colombia 1625 56
Costa Rica 0 7
El Salvador 0 42
Guatemala 721 22
Honduras 821 16
Mexico 0 890
Nicaragua 0 362
Panama 0 358
Venezuela 0 0
Tropical Latin America
Brazil 0 0
Paraguay 0 79
2000
2005
2010
2015
Data Source
CDC_RHS
COUNTRY_SPECIFI
GLOBAL_FUND_HOU
MACRO_DHS
UNICEF_MICS
WB_LSMS
Data Type
Point
Polygon
Sample Size
0
500
1000
1500
2000
2500
2000-2002
2003-2007
2008-2012
2013-2017
Prevalence
1.0
0.8
0.5
0.2
0.0
N: 69,630
Points: 13,351
Polygons: 2,595

c)

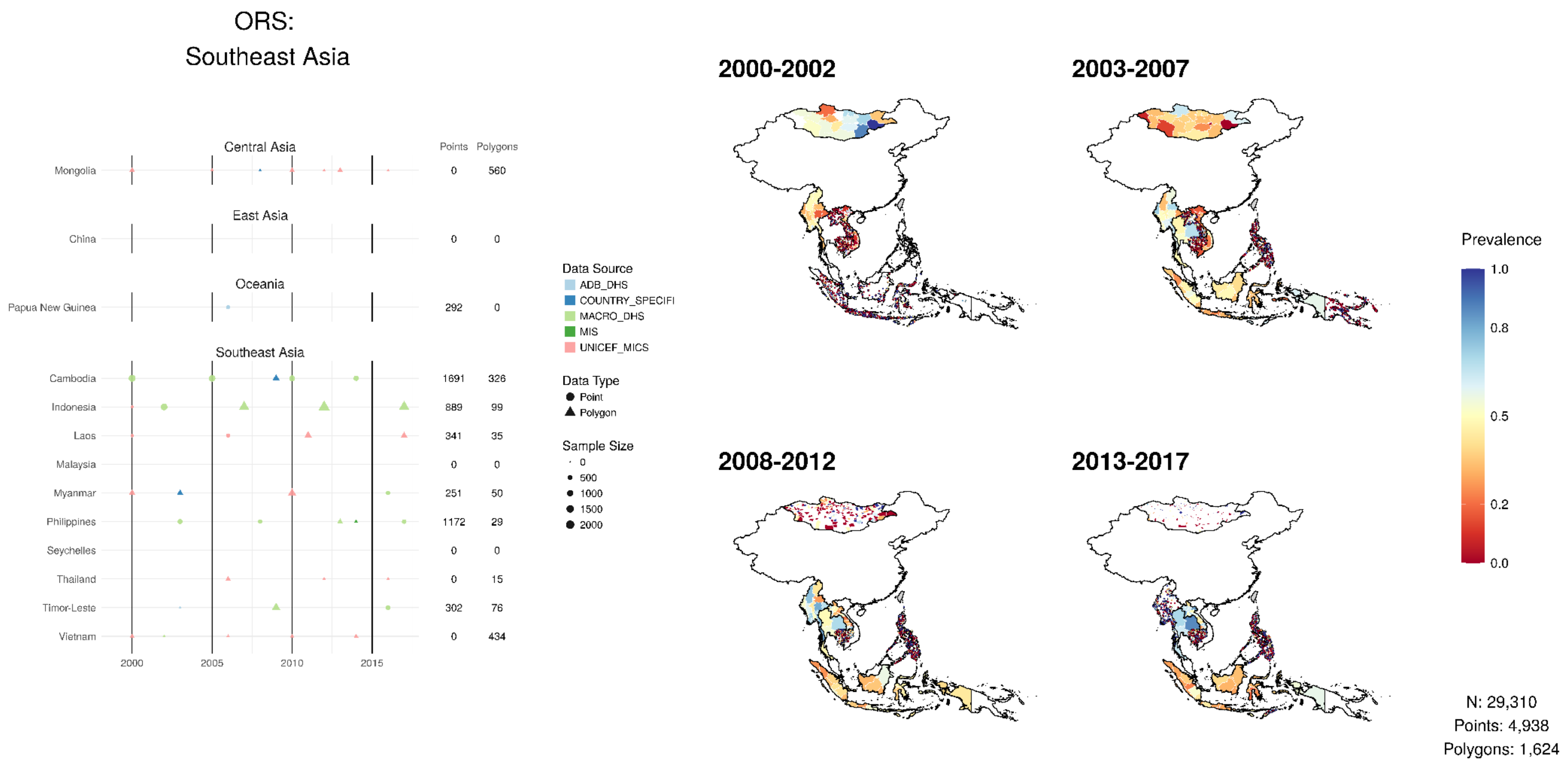
ORS:
Southeast Asia
Central Asia
Mongolia
East Asia
China
Oceania
Papua New Guinea
Southeast Asia
Cambodia
Indonesia
Laos
Malaysia
Myanmar
Philippines
Seychelles
Thailand
Timor-Leste
Vietnam
2000
2005
2010
2015
Points Polygons
Mongolia 0 560
China 0 0
Papua New Guinea 292 0
Cambodia 1691 326
Indonesia 889 99
Laos 341 35
Malaysia 0 0
Myanmar 251 50
Philippines 1172 29
Seychelles 0 0
Thailand 0 15
Timor-Leste 302 76
Vietnam 0 434
Data Source
ADB_DHS
COUNTRY_SPECIFI
MACRO_DHS
MIS
UNICEF_MICS
Data Type
Point
Polygon
Sample Size
0
500
1000
1500
2000
2000-2002
2003-2007
2008-2012
2013-2017
Prevalence
1.0
0.8
0.5
0.2
0.0
N: 29,310
Points: 4,938
Polygons: 1,624

d)

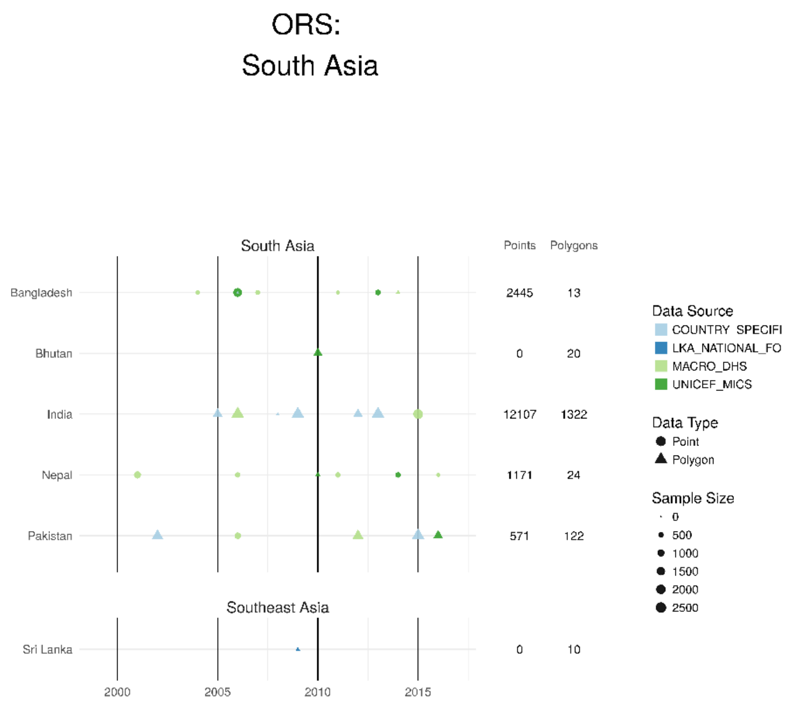
ORS:
South Asia
South Asia
Points
Polygons
Bangladesh
2445
13
Bhutan
0
20
India
12107
1322
Nepal
1171
24
Pakistan
571
122
Southeast Asia
Sri Lanka
0
10
2000
2005
2010
2015
Data Source
COUNTRY_SPECIFI
LKA_NATIONAL_FO
MACRO_DHS
UNICEF_MICS
Data Type
Point
Polygon
Sample Size
0
500
1000
1500
2000
2500

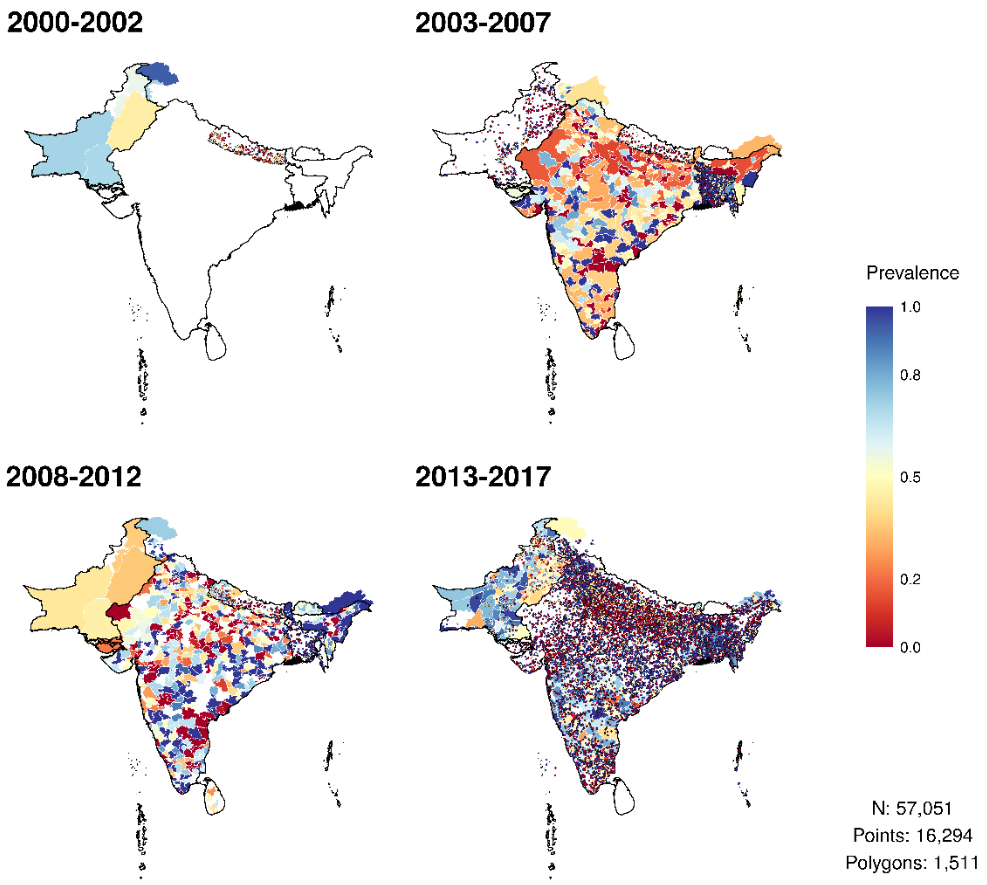
2000-2002
2003-2007
2008-2012
2013-2017
Prevalence
1.0
0.8
0.5
0.2
0.0
N: 57,051
Points: 16,294
Polygons: 1,511

e)

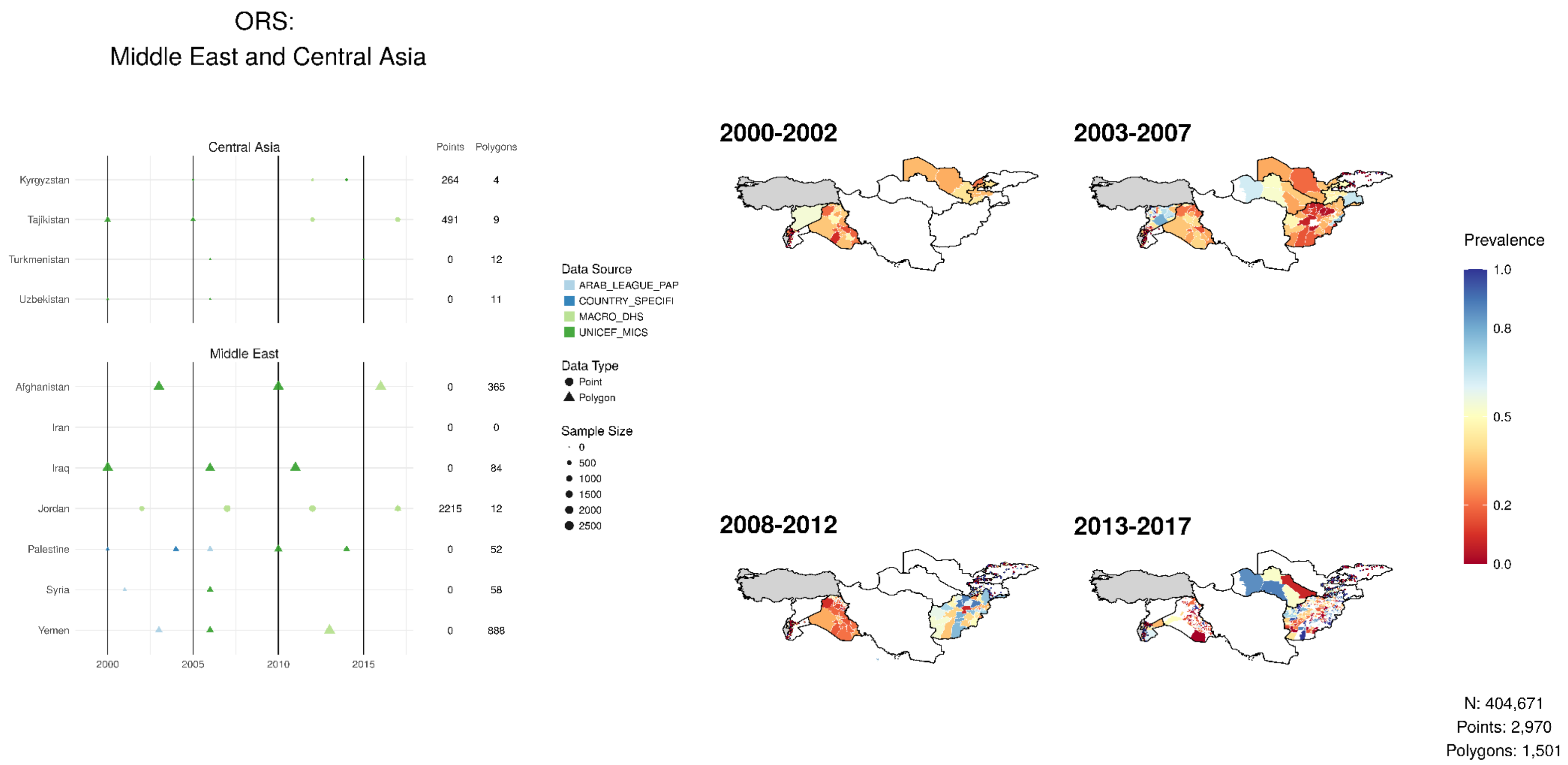
ORS:
Middle East and Central Asia
Central Asia
Points
Polygons
Kyrgyzstan 264 4
Tajikistan 491 9
Turkmenistan 0 12
Uzbekistan 0 11
Middle East
Afghanistan 0 365
Iran 0 0
Iraq 0 84
Jordan 2215 12
Palestine 0 52
Syria 0 58
Yemen 0 888
2000
2005
2010
2015
Data Source
ARAB_LEAGUE_PAP
COUNTRY_SPECIFI
MACRO_DHS
UNICEF_MICS
Data Type
Point
Polygon
Sample Size
0
500
1000
1500
2000
2500
2000-2002
2003-2007
2008-2012
2013-2017
Prevalence
1.0
0.8
0.5
0.2
0.0
N: 404,671
Points: 2,970
Polygons: 1,501

Appendix Figure 4. Covariates used in mapping

We used fifteen covariate raster layers of possible socio-economic and environmental correlates of ORS, RHF, and/or ORT coverage as inputs for the stacking modelling process. Time-varying covariates (^{TV}) are presented for the year 2017. For the year of production of non-time-varying covariates, please refer to the individual covariate citation appendix table 3 for additional detail. Covariates are labelled as follows: Access to cities [*access2*], ratio of children dependents (age 0 to 14) to working adults (age 15 to 64) [*depratio*], distance from rivers or lakes [*distriverslakes*], night-time lights^{TV} [*dmspntl*], elevation [*elevation*], number of children under 5 per woman of childbearing age [*fertility*], urban or rural^{TV} [*ghlsurbanicity*], number of people whose daily vitamin A needs could be met [*herreronyield*], irrigation [*irrigation*], urban proportion of the location^{TV} [*landcover*], aridity^{TV} [*aridity*], population^{TV} [*worldpop_raked*], prevalence of under-5 stunting^{TV} [*stunting_mod_b*], prevalence of under-5 wasting^{TV} [*wasting_mod_b*], and maternal education [*education*]. Maps reflect administrative boundaries, land cover, lakes, and population; dark grey-coloured grid cells were classified as "barren or sparsely vegetated" and had fewer than ten people per 1 × 1-km grid cell.[7,21–25]

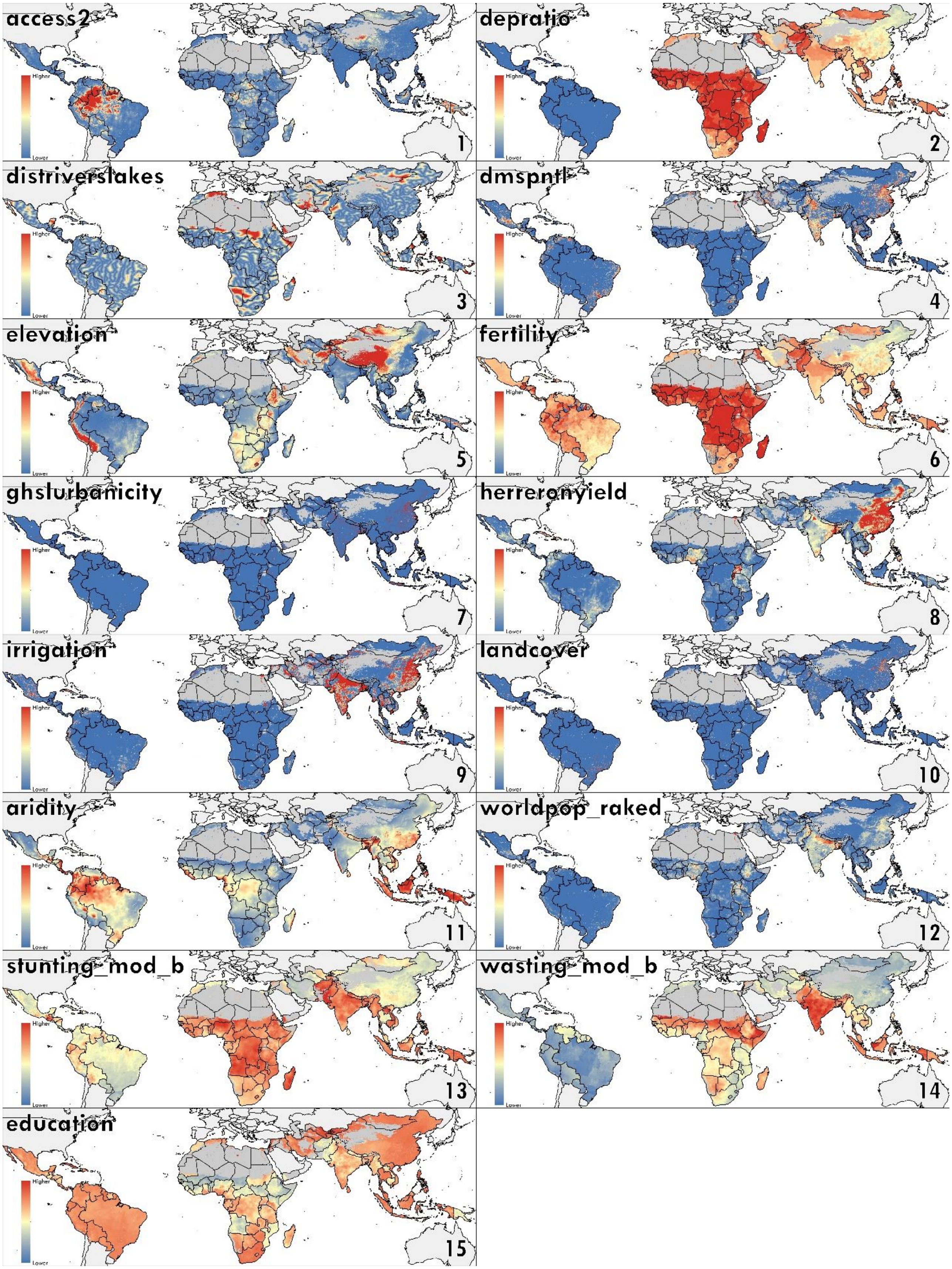
access2
1
depratio
2
distriverslakes
3
dmspntl
4
elevation
5
fertility
6
ghslurbanicity
7
herreronyield
8
irrigation
9
landcover
10
aridity
11
worldpop_raked
12
stunting_mod_b
13
wasting_mod_b
14
education
15
Higher
Lower

Appendix Figure 5. Map of modelling regions

We stratified our data and analyses into 23 regions selected to align with the Global Burden of Disease study[11] and to allow for country-specific models in countries with distinct ORS patterns. Each colour represents a different modelling region; grey indicating countries that we did not include in this stage of our analysis. In order of appearance in the legend, the regions are: the horn of Africa (burgundy), Ethiopia (dark green), Yemen (dark purple), central Asia (brown), Mongolia (lavender), central sub-Saharan Africa (light purple), the Democratic Republic of the Congo (olive green), eastern sub-Saharan Africa (light pink), Kenya (navy), Zimbabwe (charcoal grey), Malay Archipelago (fuchsia pink), Mexico, the Caribbean and Central America (apple red), the Middle East (mint green), north Africa and the Middle East (cobalt blue), northern South America (green), southern South America (grey), southeast Asia (light blue), south Asia (peach), India (lime green), Pakistan (yellow), southern sub-Saharan Africa (teal), western sub-Saharan Africa (orange), and Nigeria (pale yellow).

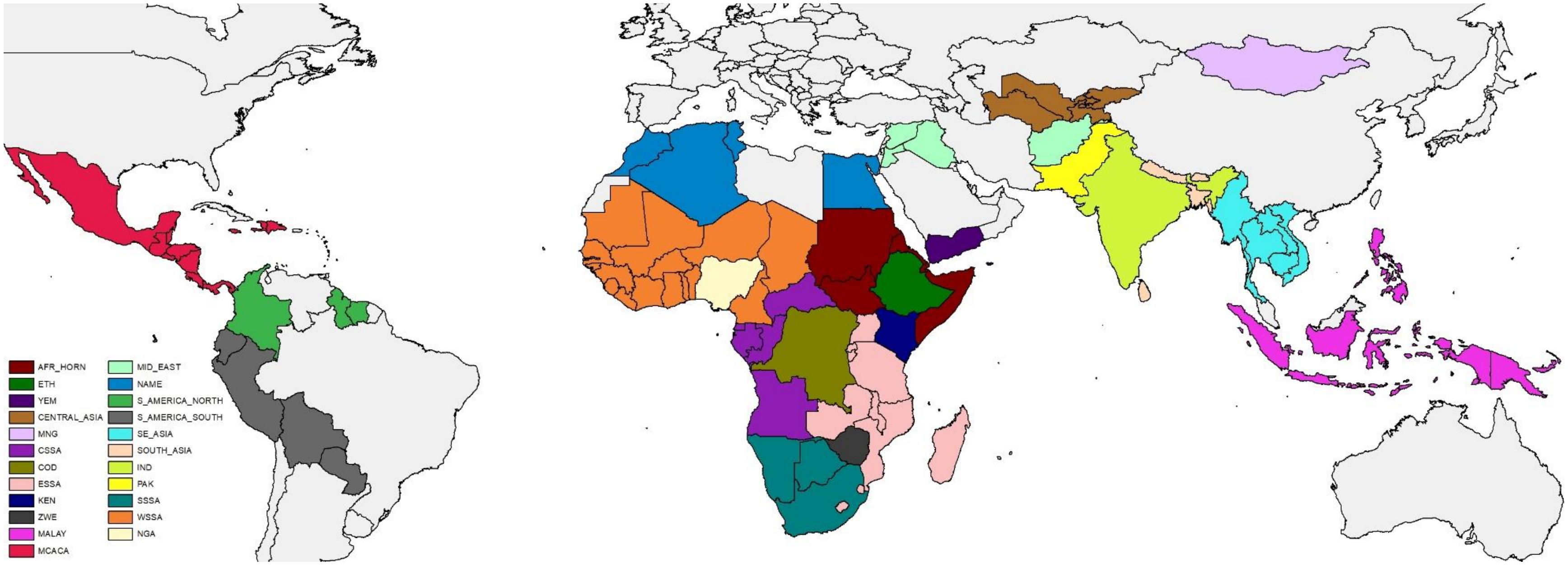

Appendix Figure 6. Finite elements mesh

The finite elements mesh used to fit the space-time correlated error for the Southern sub-Saharan Africa region. Both the fine-scale mesh over land in the modelling region and the coarser buffer region mesh are shown.

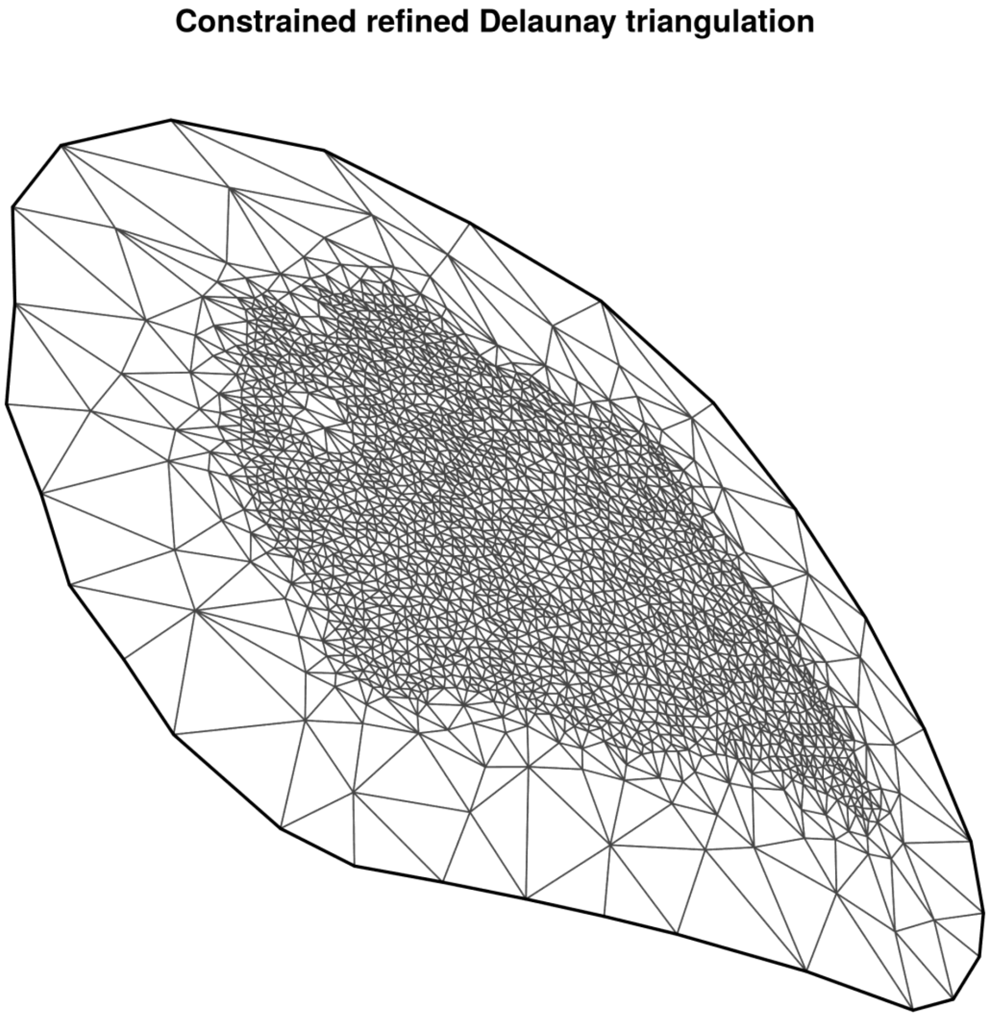

Appendix Figure 7. Posterior means and 95% uncertainty intervals for ORS coverage at the 5 × 5-km grid-cell level for 2017

Maps reflect administrative boundaries, land cover, lakes, and population; dark grey-coloured grid cells were classified as "barren or sparsely vegetated" and had fewer than ten people per 1 × 1-km grid cell; light grey countries were not included in these analyses.[7,21–25]

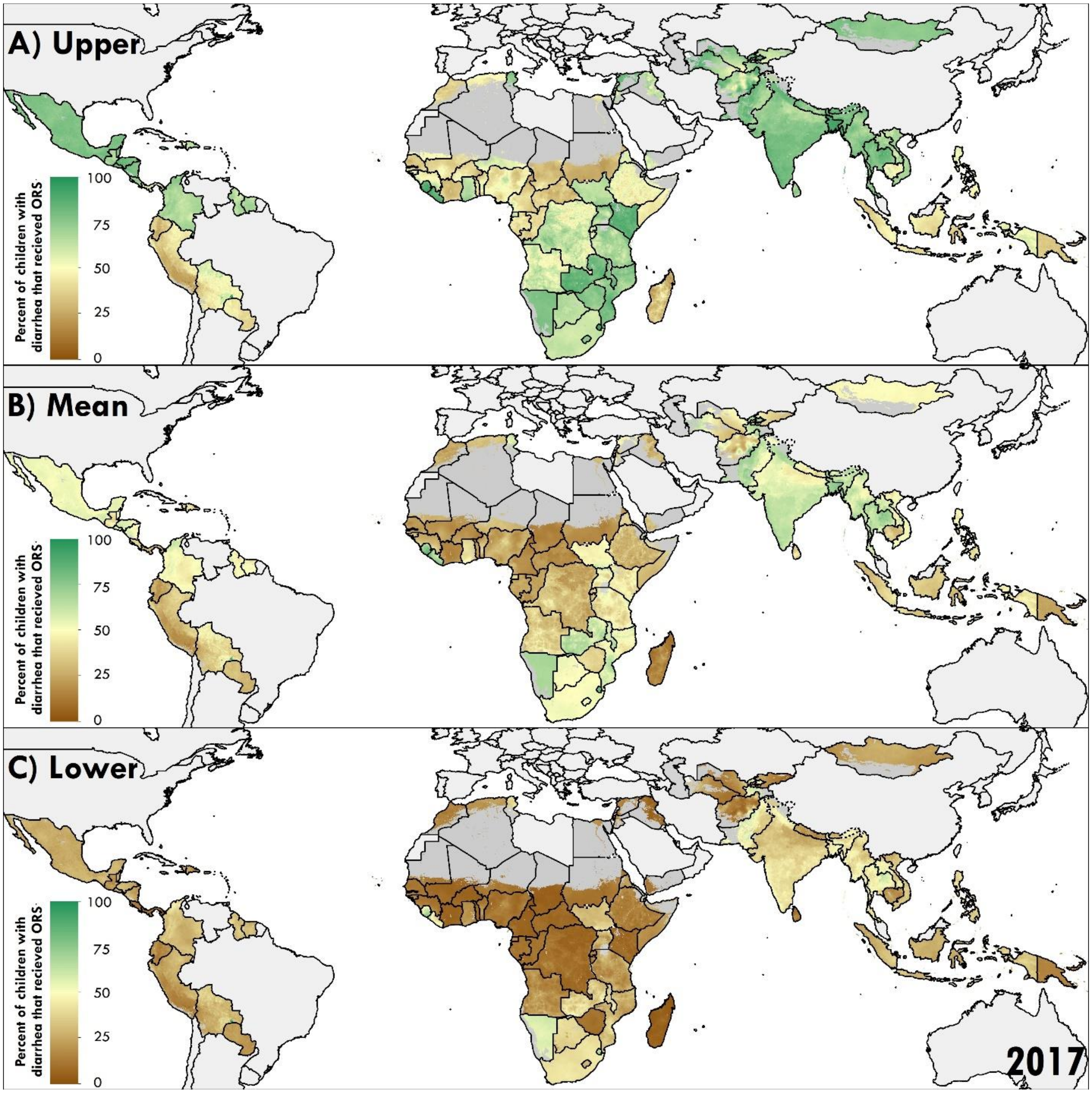

Appendix Figure 8. Posterior means and 95% uncertainty intervals for RHF coverage at the 5 × 5-km grid-cell level for 2017

Maps reflect administrative boundaries, land cover, lakes, and population; dark grey-coloured grid cells were classified as “barren or sparsely vegetated” and had fewer than ten people per 1 × 1-km grid cell; light grey countries were not included in these analyses.[7,21–25]

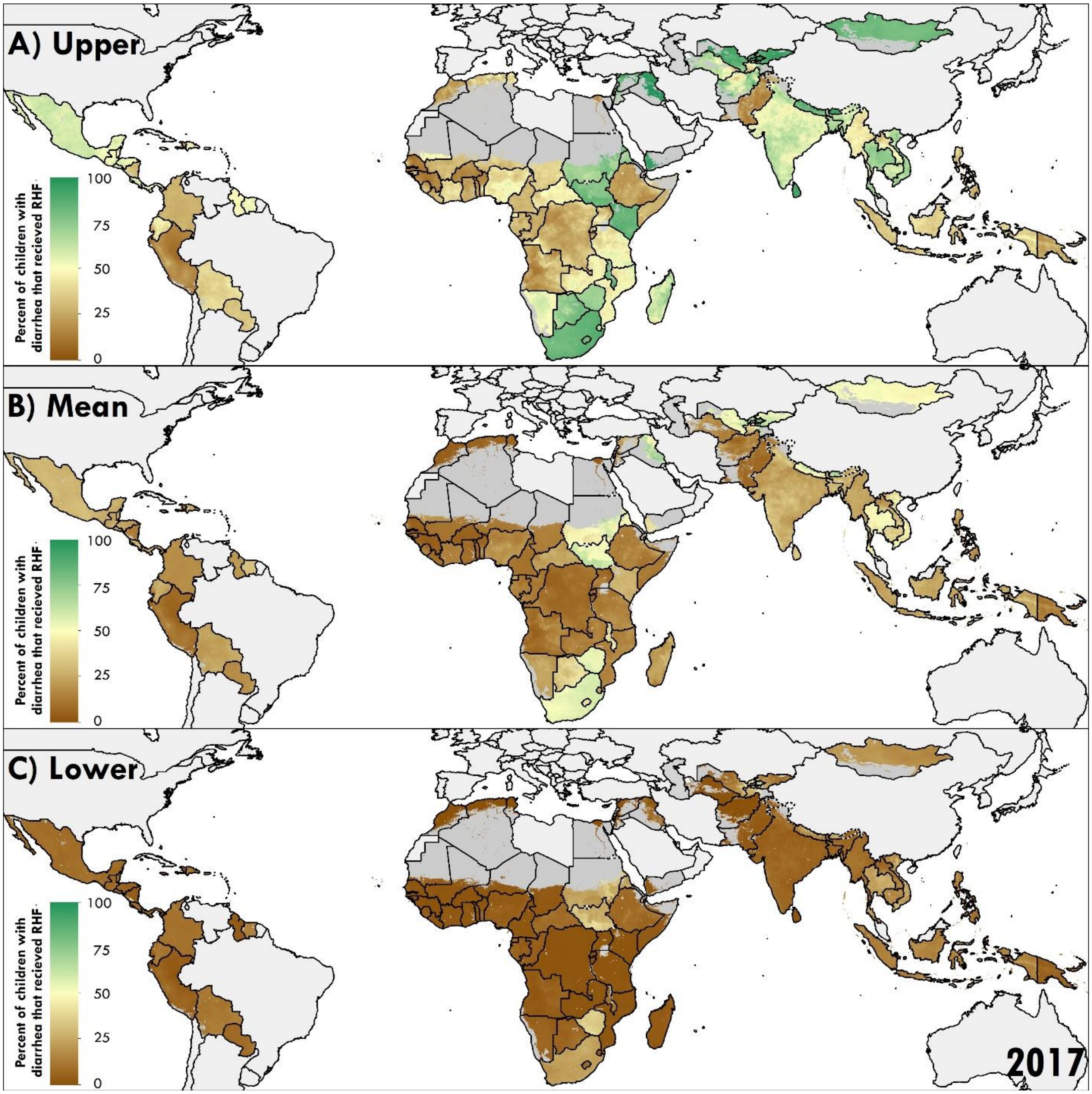

Appendix Figure 9. Posterior means and 95% uncertainty intervals for ORT coverage at the 5 × 5-km grid-cell level for 2017

Maps reflect administrative boundaries, land cover, lakes, and population; dark grey-coloured grid cells were classified as "barren or sparsely vegetated" and had fewer than ten people per 1 × 1-km grid cell; light grey countries were not included in these analyses.[7,21–25]

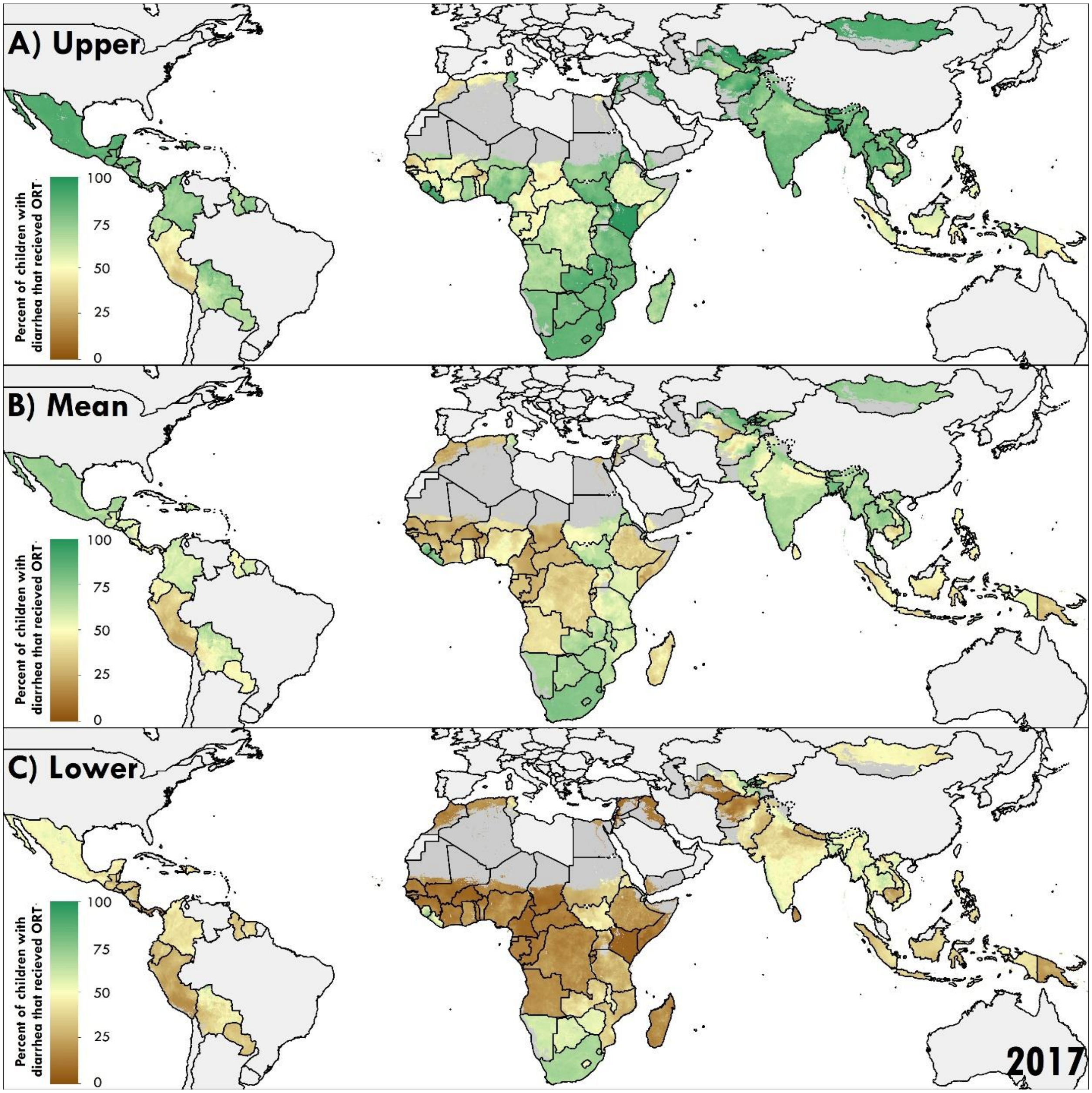

Appendix Figure 10. Posterior means and 95% uncertainty intervals for ORS coverage at the second-administrative-unit level for 2017

Maps reflect administrative boundaries, land cover, lakes, and population; dark grey-coloured grid cells were classified as "barren or sparsely vegetated" and had fewer than ten people per 1 × 1-km grid cell; light grey countries were not included in these analyses.[7,21–25]

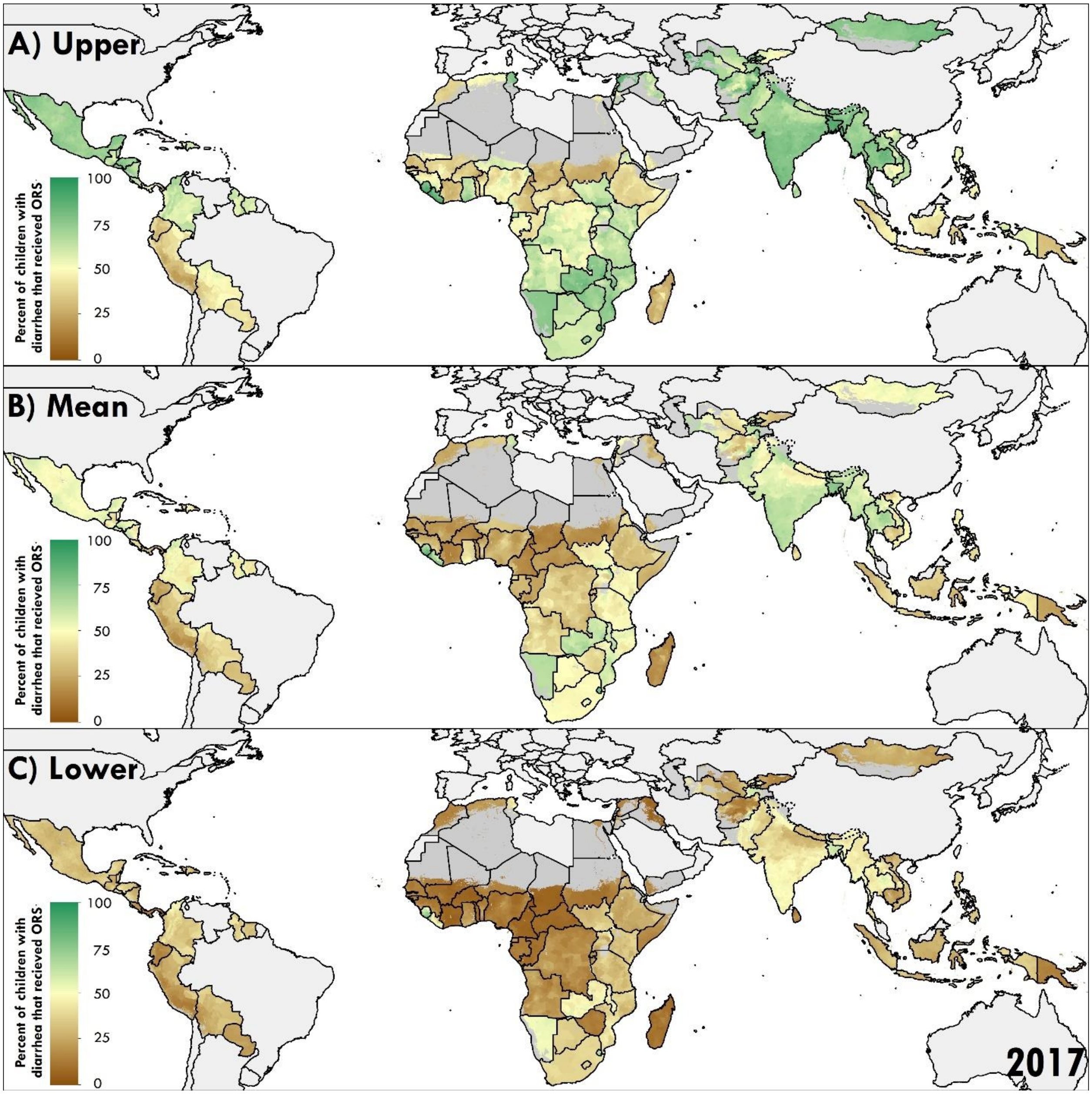

Appendix Figure 11. Posterior means and 95% uncertainty intervals for RHF coverage at the second-administrative-unit level for 2017

Maps reflect administrative boundaries, land cover, lakes, and population; dark grey-coloured grid cells were classified as "barren or sparsely vegetated" and had fewer than ten people per 1 × 1-km grid cell; light grey countries were not included in these analyses.[7,21–25]

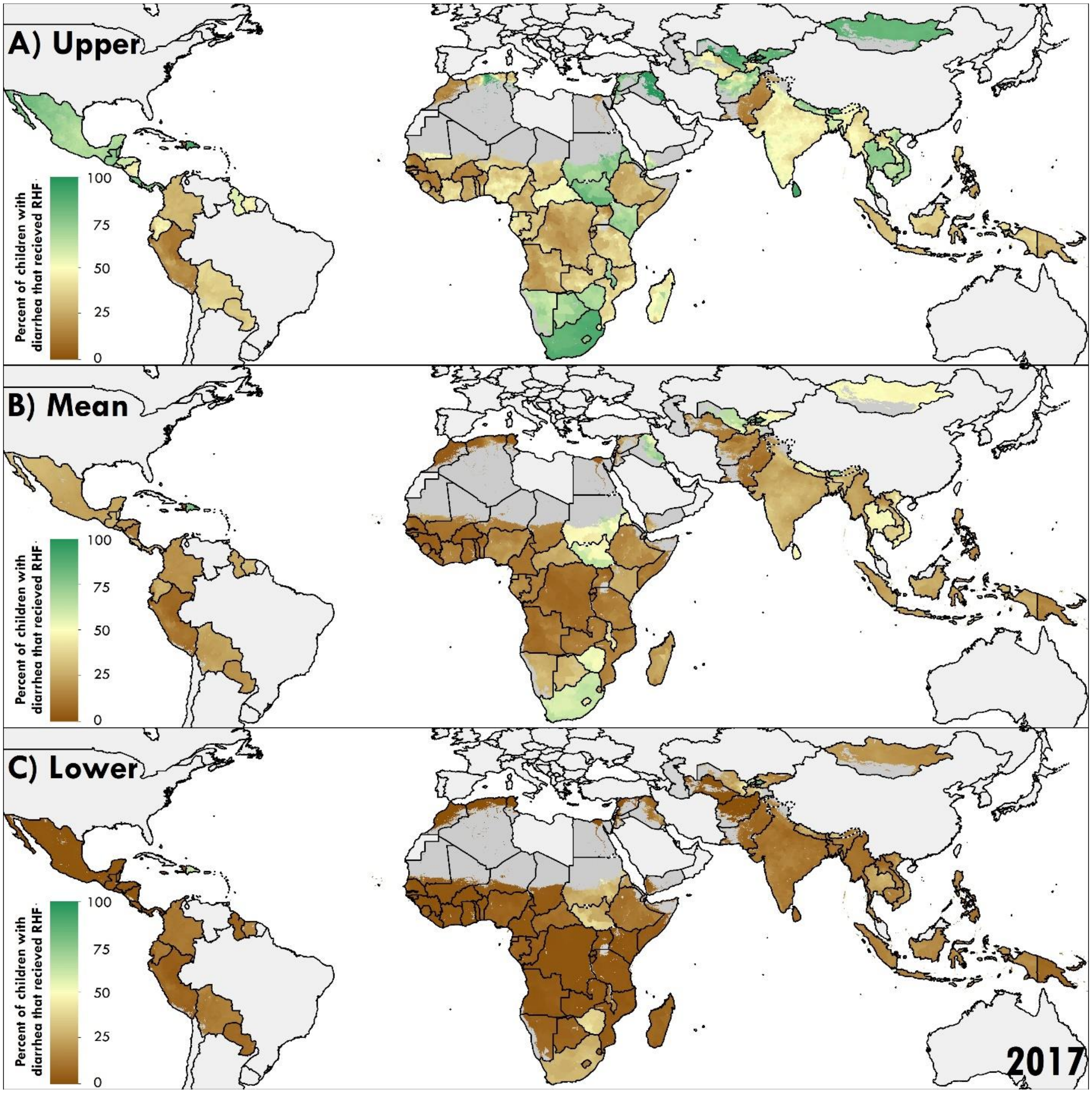

Appendix Figure 12. Posterior means and 95% uncertainty intervals for ORT coverage at the second-administrative-unit level for 2017

Maps reflect administrative boundaries, land cover, lakes, and population; dark grey-coloured grid cells were classified as "barren or sparsely vegetated" and had fewer than ten people per 1 × 1-km grid cell; light grey countries were not included in these analyses.[7,21–25]

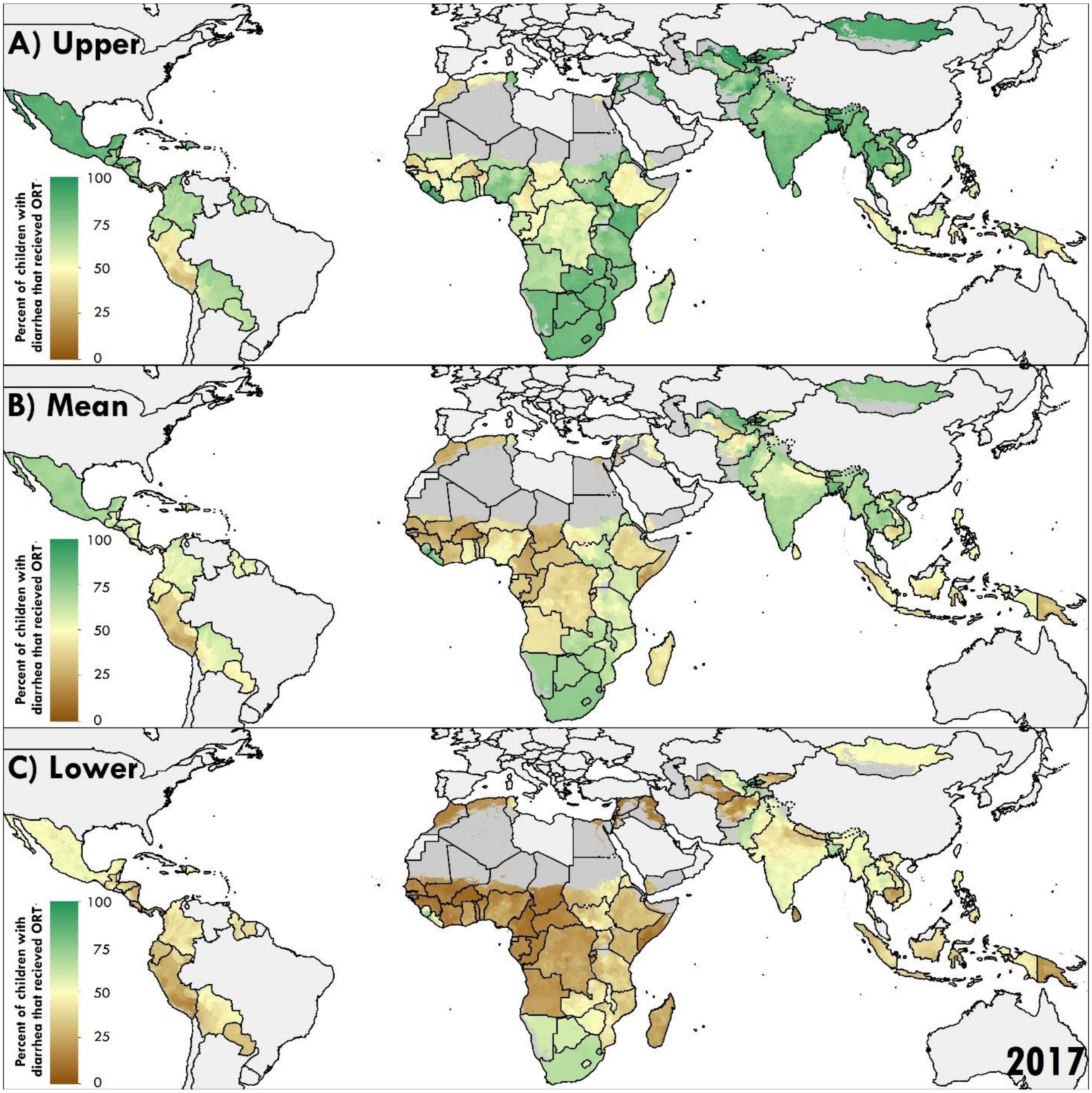

Appendix Figure 13. Posterior means and 95% uncertainty intervals for ORS coverage at the first-administrative-unit level for 2017

Maps reflect administrative boundaries, land cover, lakes, and population; dark grey-coloured grid cells were classified as “barren or sparsely vegetated” and had fewer than ten people per 1 × 1-km grid cell; light grey countries were not included in these analyses.[7,21–25]

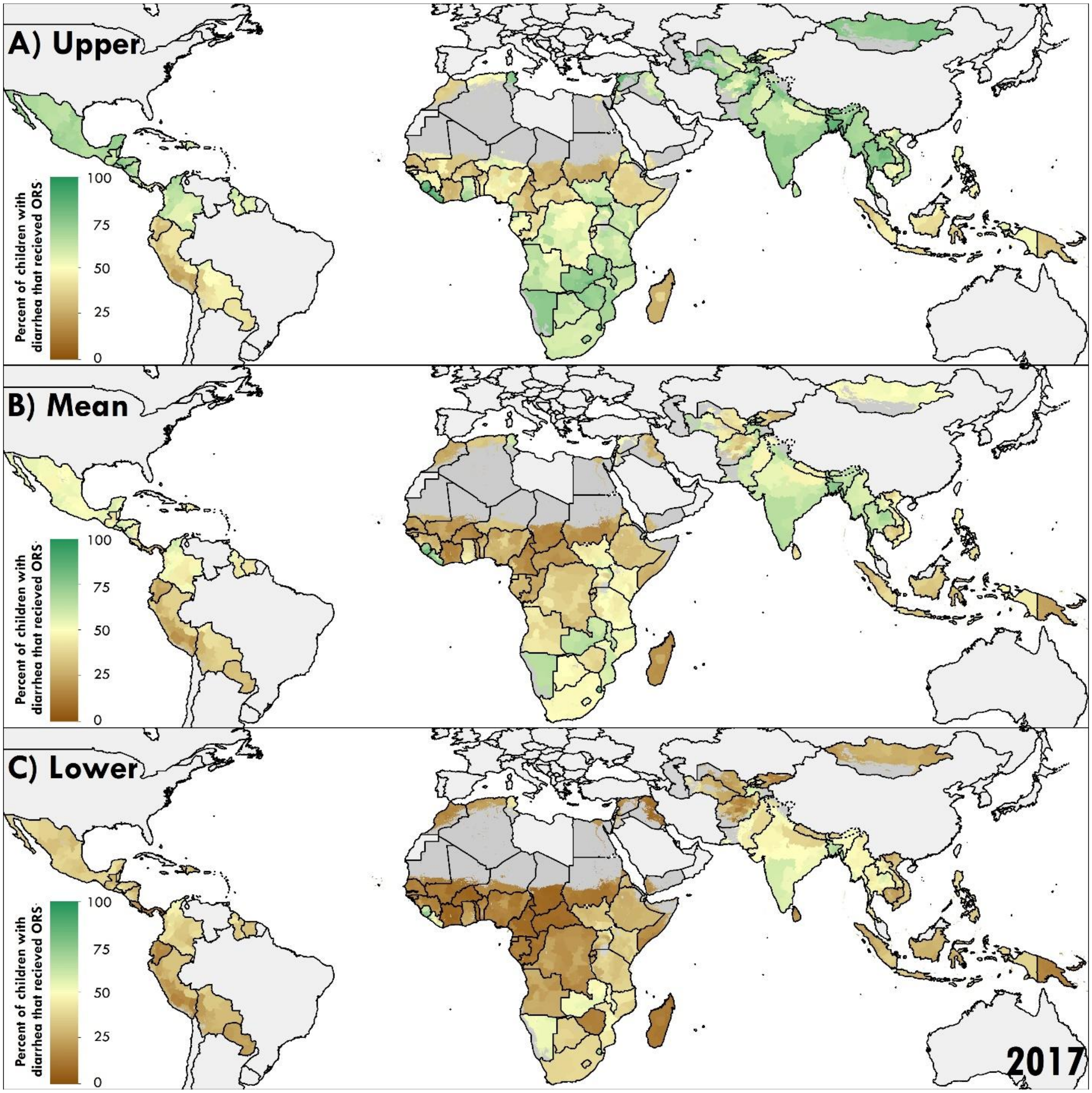

Appendix Figure 14. Posterior means and 95% uncertainty intervals for RHF coverage at the first-administrative-unit level for 2017

Maps reflect administrative boundaries, land cover, lakes, and population; dark grey-coloured grid cells were classified as "barren or sparsely vegetated" and had fewer than ten people per 1 × 1-km grid cell; light grey countries were not included in these analyses.[7,21–25]

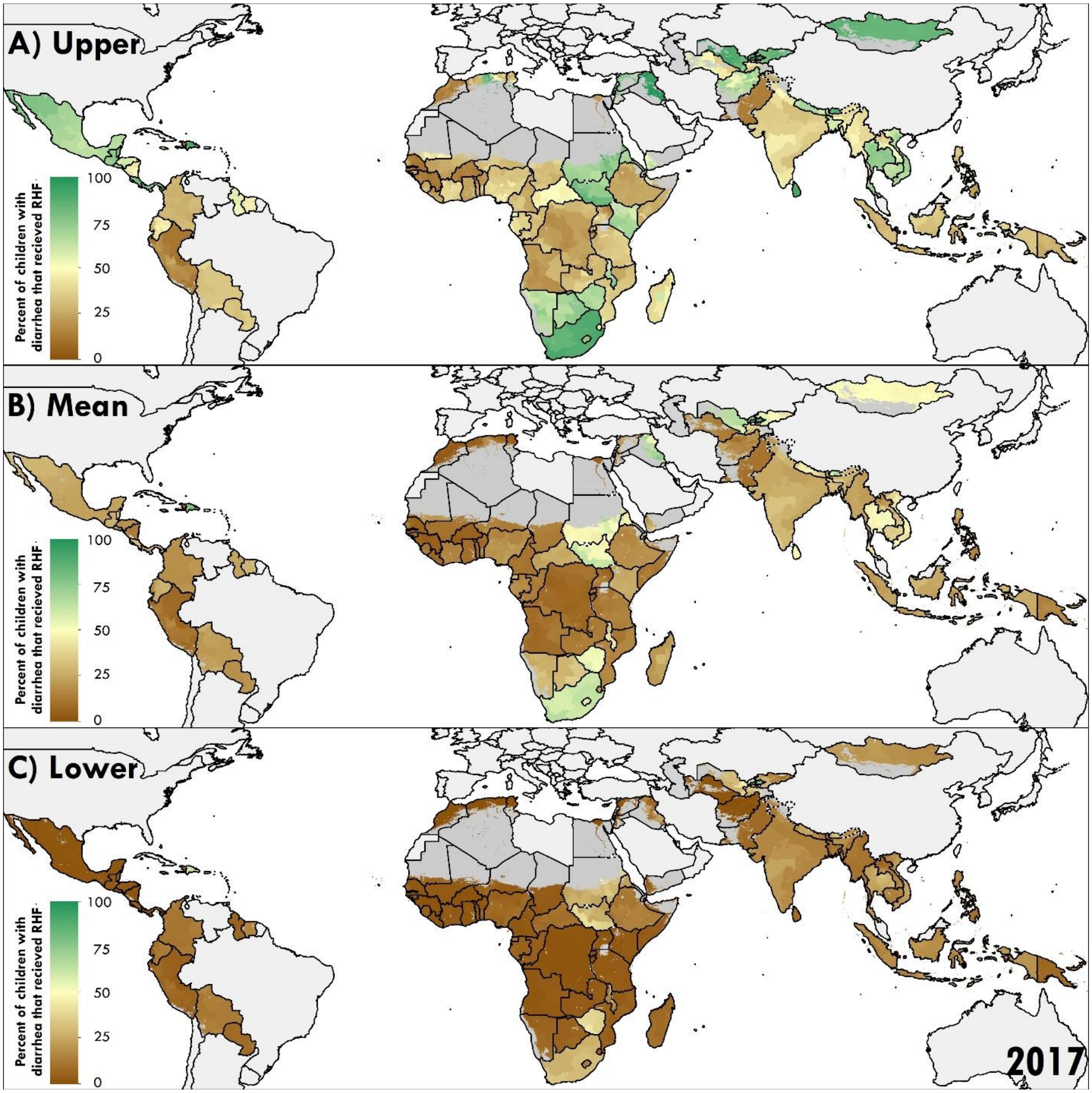

Appendix Figure 15. Posterior means and 95% uncertainty intervals for ORT coverage at the first-administrative-unit level for 2017

Maps reflect administrative boundaries, land cover, lakes, and population; dark grey-coloured grid cells were classified as "barren or sparsely vegetated" and had fewer than ten people per 1 × 1-km grid cell; light grey countries were not included in these analyses.[7,21–25]

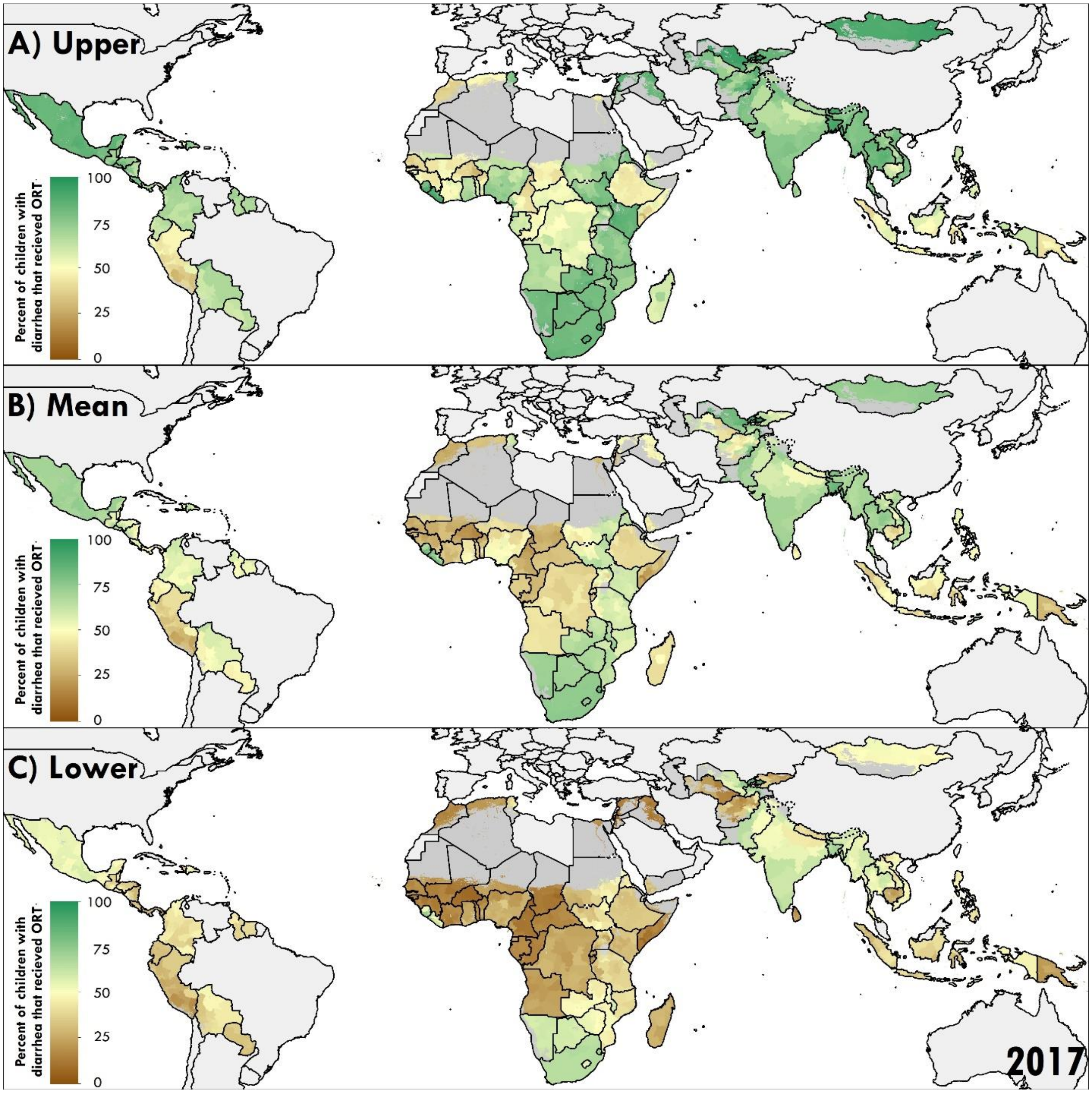

Appendix Figure 16-33. In-sample validation plots for ORS, RHF, and ORT

Each plot shows the survey data on the x-axis and mean posterior predictions of coverage y-axis. The size of each dot is proportional to sample size in the underlying data. Estimates are shown aggregated to country, first administrative, and second administrative levels. Estimates are also shown stratified by region and by year. For corresponding in-sample fit statistics see appendix tables 9, 11, and 13.

Regions are labelled in the following manner: the Democratic Republic of the Congo [*COD*], the Horn of Africa [*dia_afr_horn-eth-yem*], Central Asia [*dia_central_asia*], Central sub-Saharan Africa [*dia_cssa-cod*], Eastern sub-Saharan Africa [*dia_essa-zwe-ken*], Malay Archipelago [*dia_malay*], Mexico, the Caribbean, and Central America [*dia_mcaca*], the Middle East [*dia_mid_east*], North Africa and the Middle East [*dia_name*], northern South America [*dia_s_america_n*], southern South America [*dia_s_america_s*], Southeast Asia [*dia_se_asia*], South Asia [*dia_south_asia-ind-pak*], Southern sub-Saharan Africa [*dia_sssa*], Western sub-Saharan Africa [*dia_wssa-nga*], Ethiopia [*ETH*], India [*IND*], Kenya [*KEN*], Mongolia [*MNG*], Nigeria [*NGA*], Pakistan [*PAK*], Yemen [*YEM*], and Zimbabwe [*ZWE*].

Appendix Figure 16. In-sample validation plot for ORS by country and region

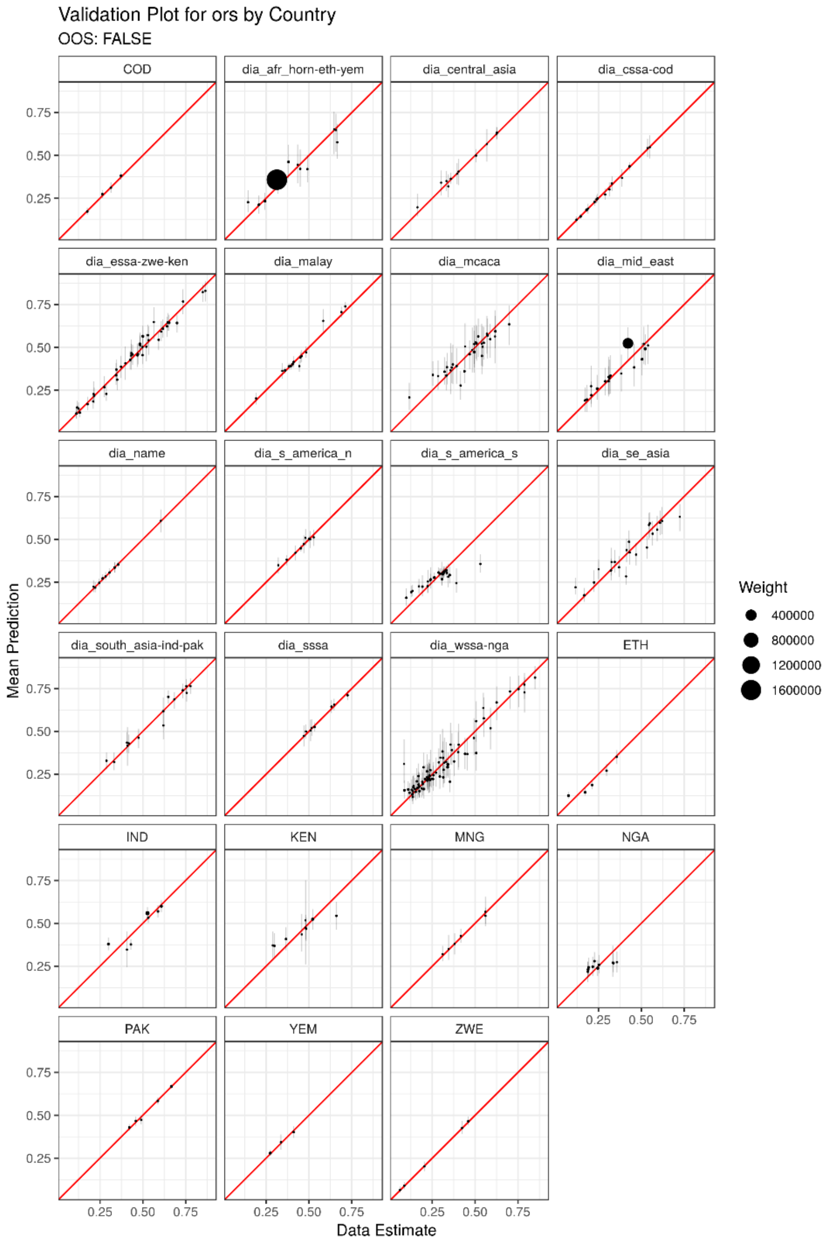

Appendix Figure 17. In-sample validation plot for ORS by first administrative unit and region

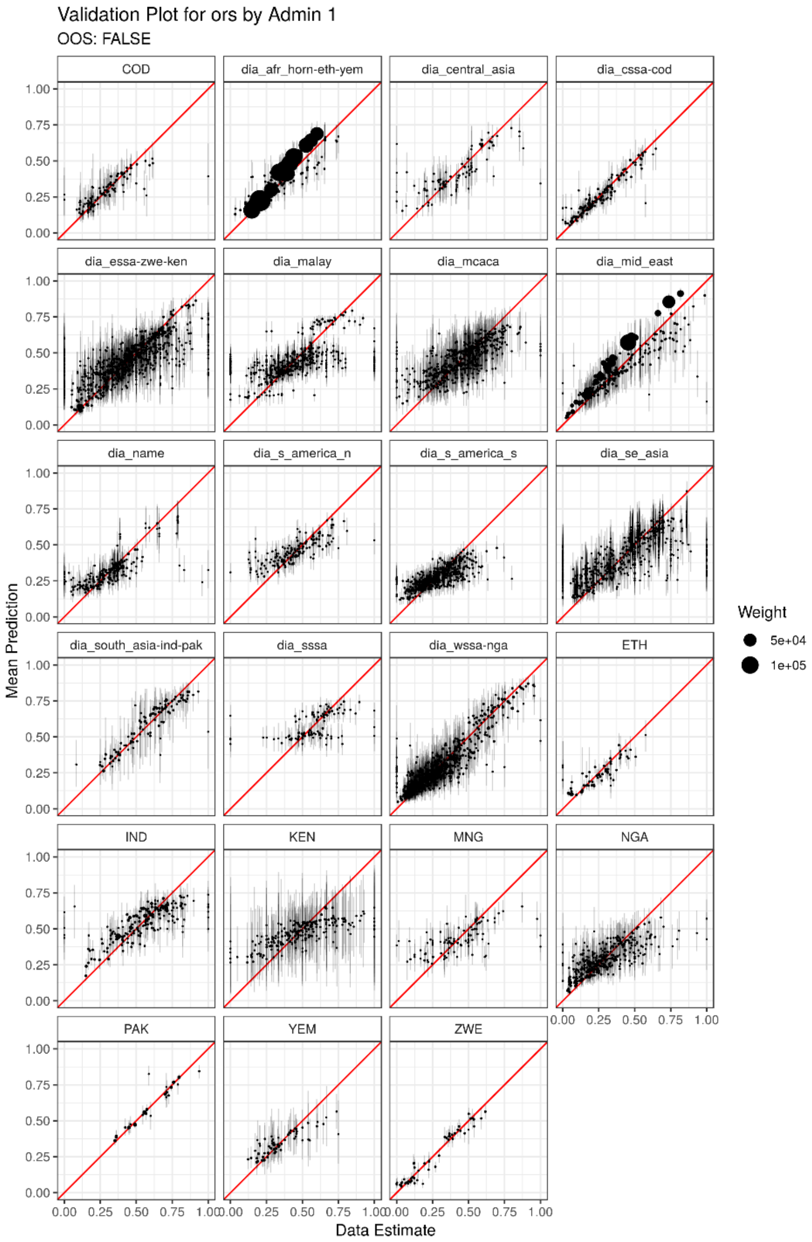

Appendix Figure 18. In-sample validation plot for ORS by second administrative unit and region

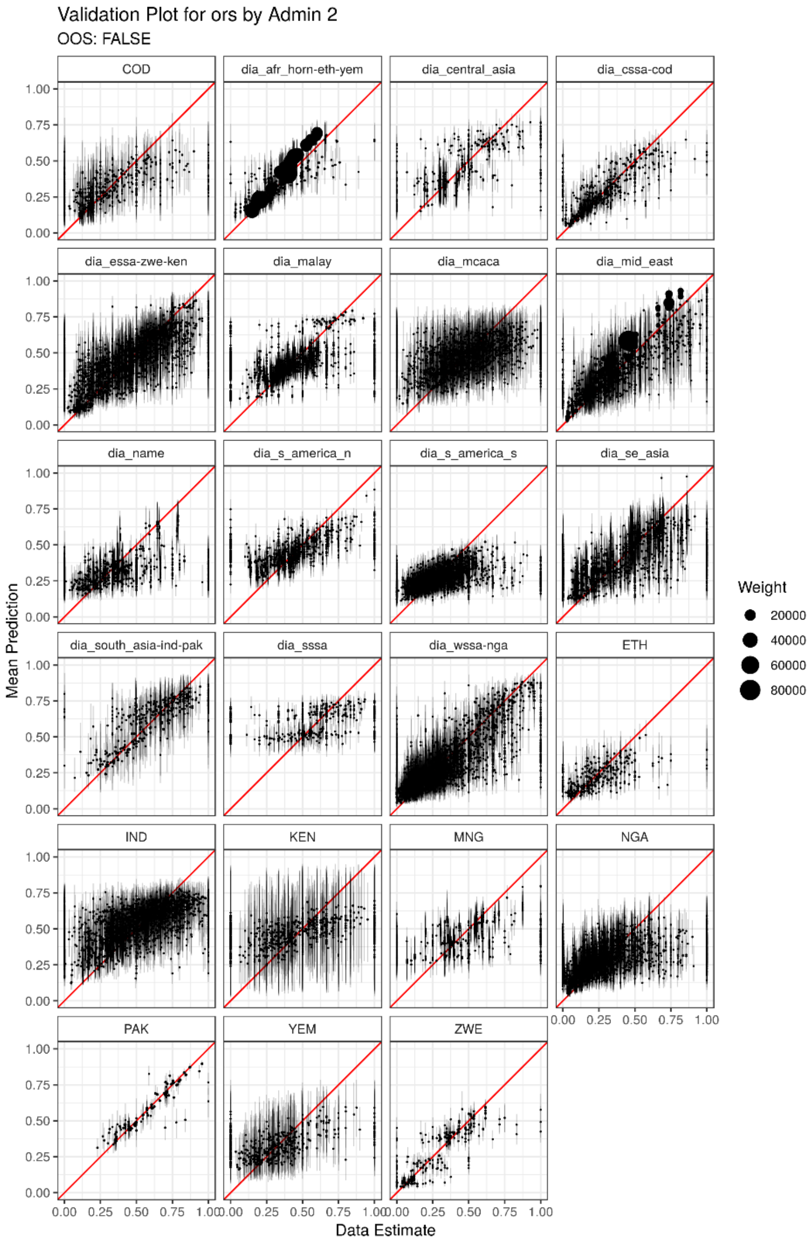

Appendix Figure 19. In-sample validation plot for ORS by country and year

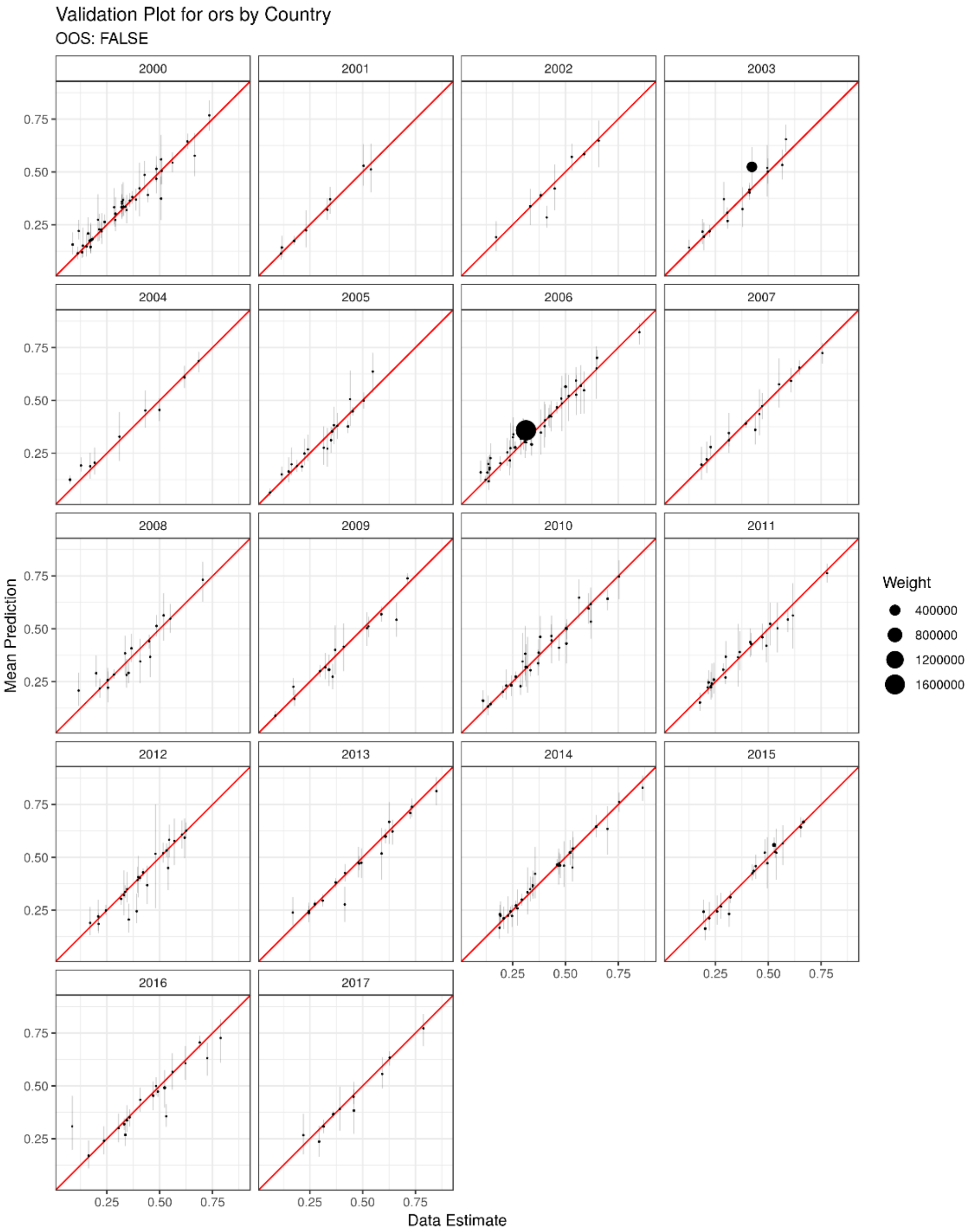

Appendix Figure 20. In-sample validation plot for ORS by first administrative unit and year

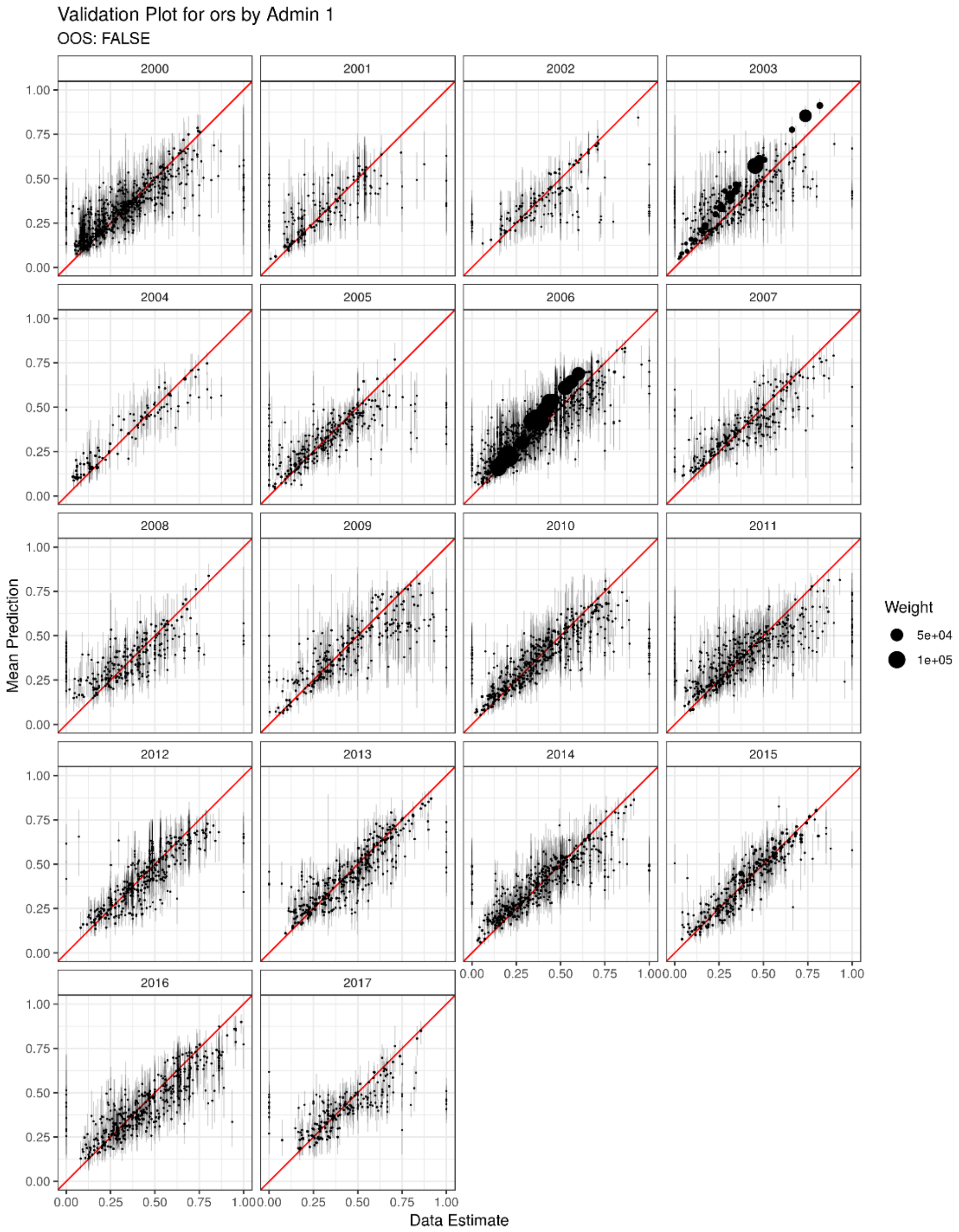

Appendix Figure 21. In-sample validation plot for ORS by second administrative unit and year

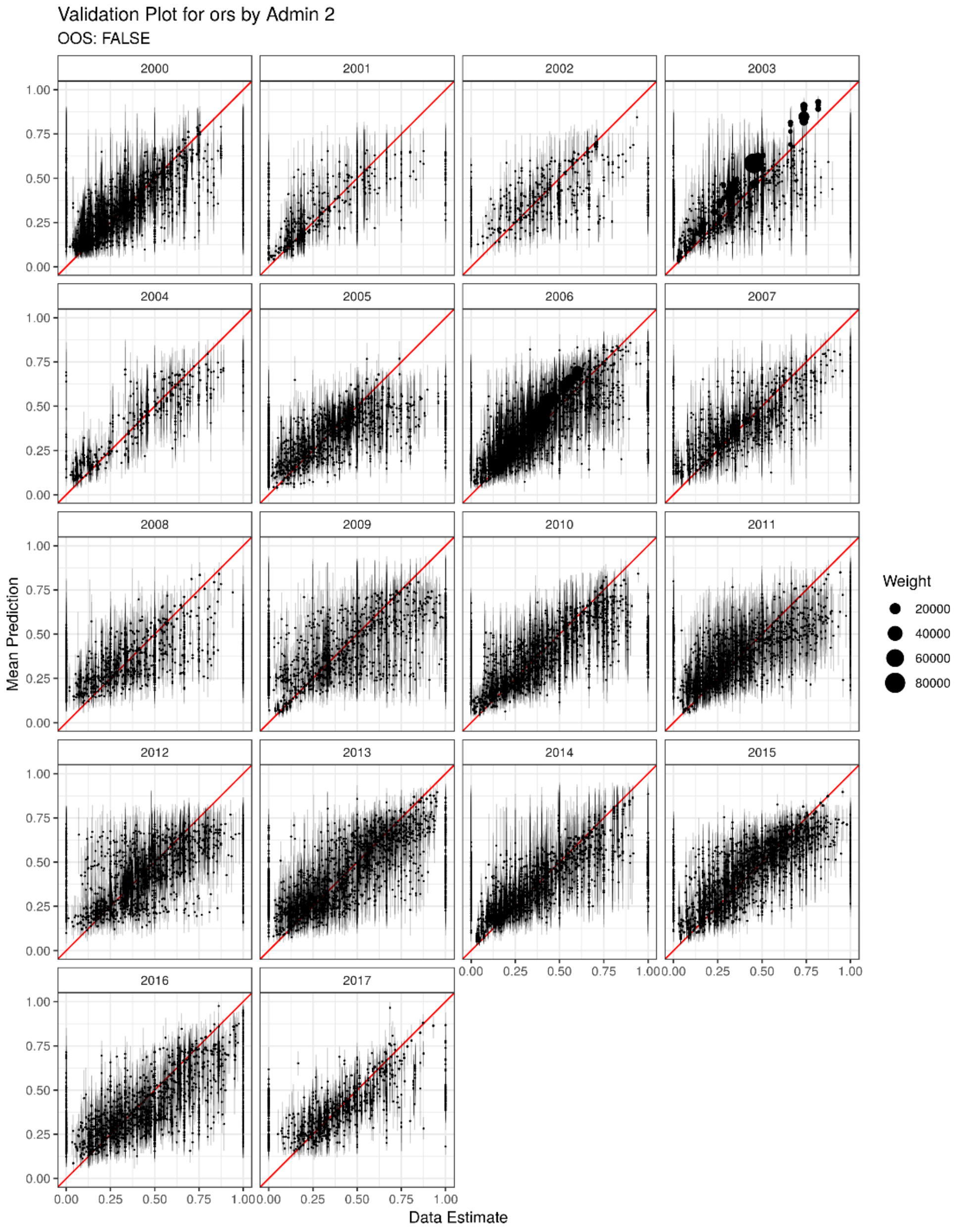

Appendix Figure 22. In-sample validation plot for RHF by country and region

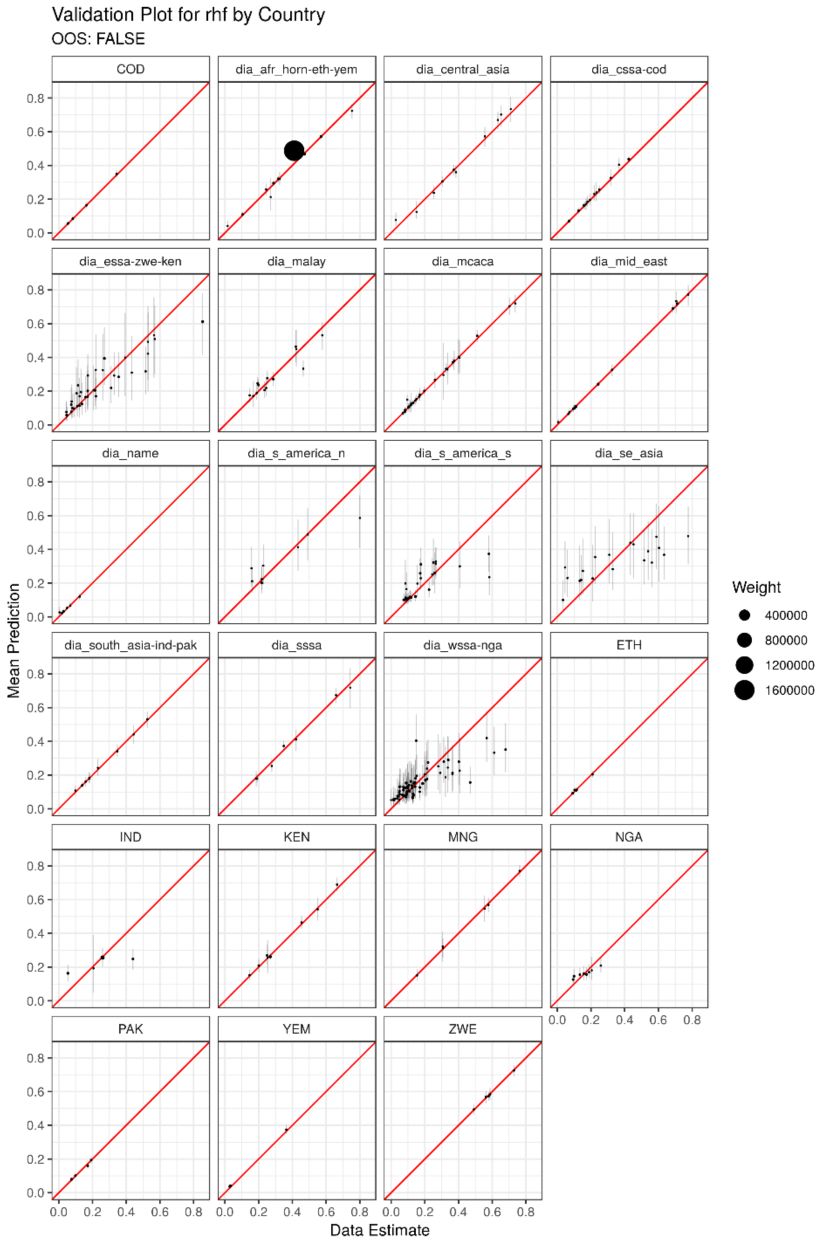

Appendix Figure 23. In-sample validation plot for RHF by first administrative unit and region

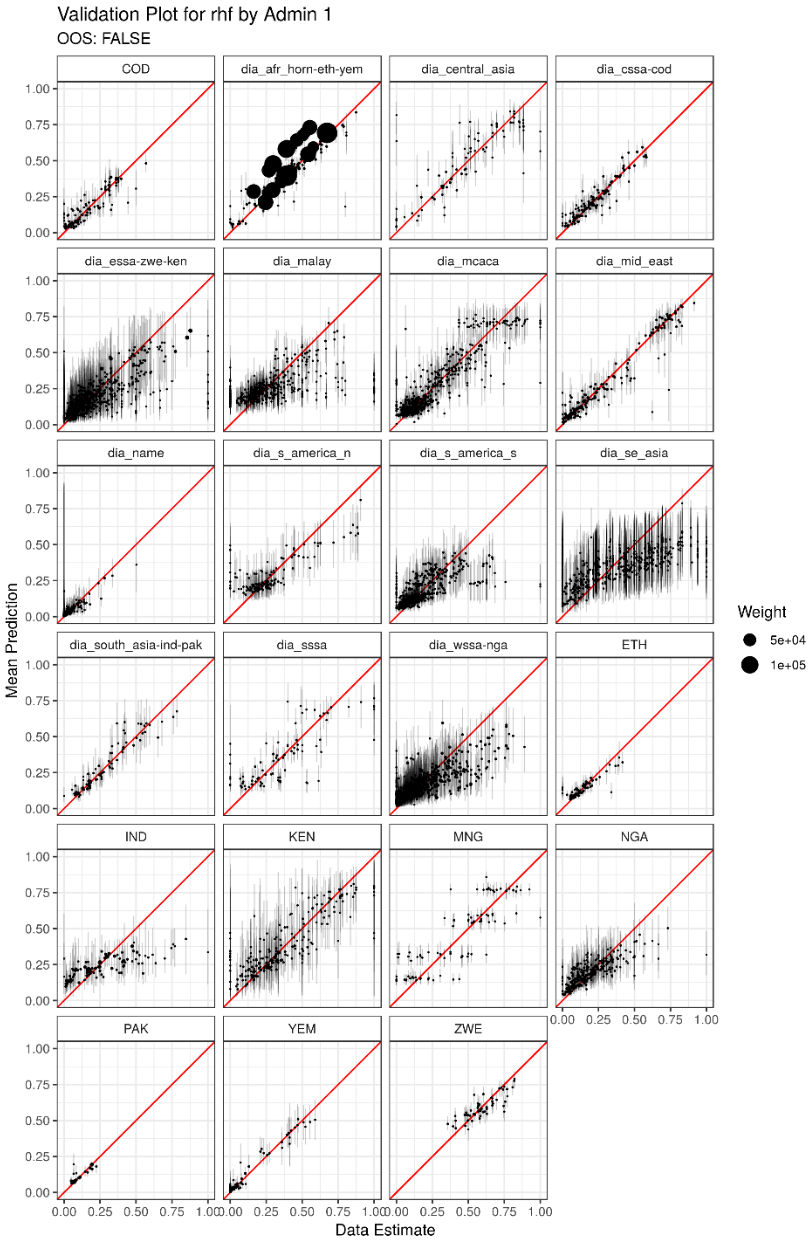

Appendix Figure 24. In-sample validation plot for RHF by second administrative unit and region

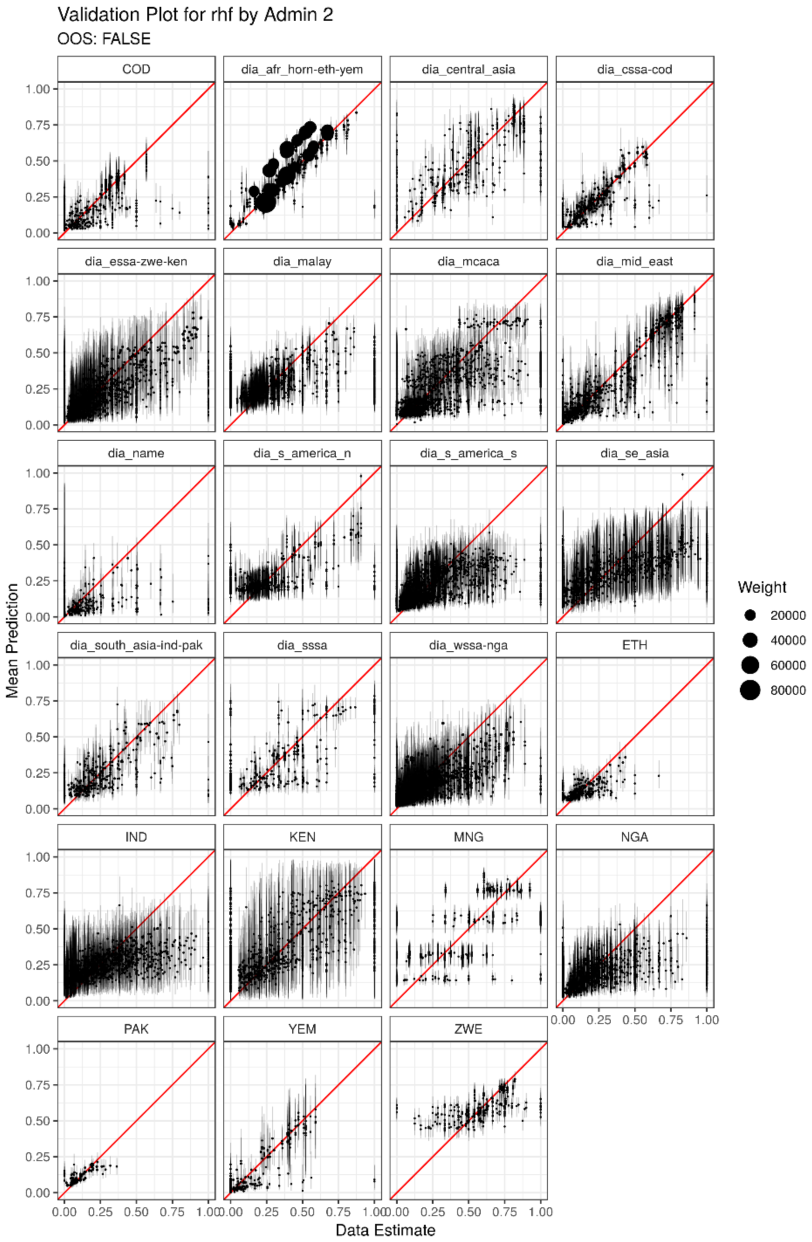

Appendix Figure 25. In-sample validation plot for RHF by country and year

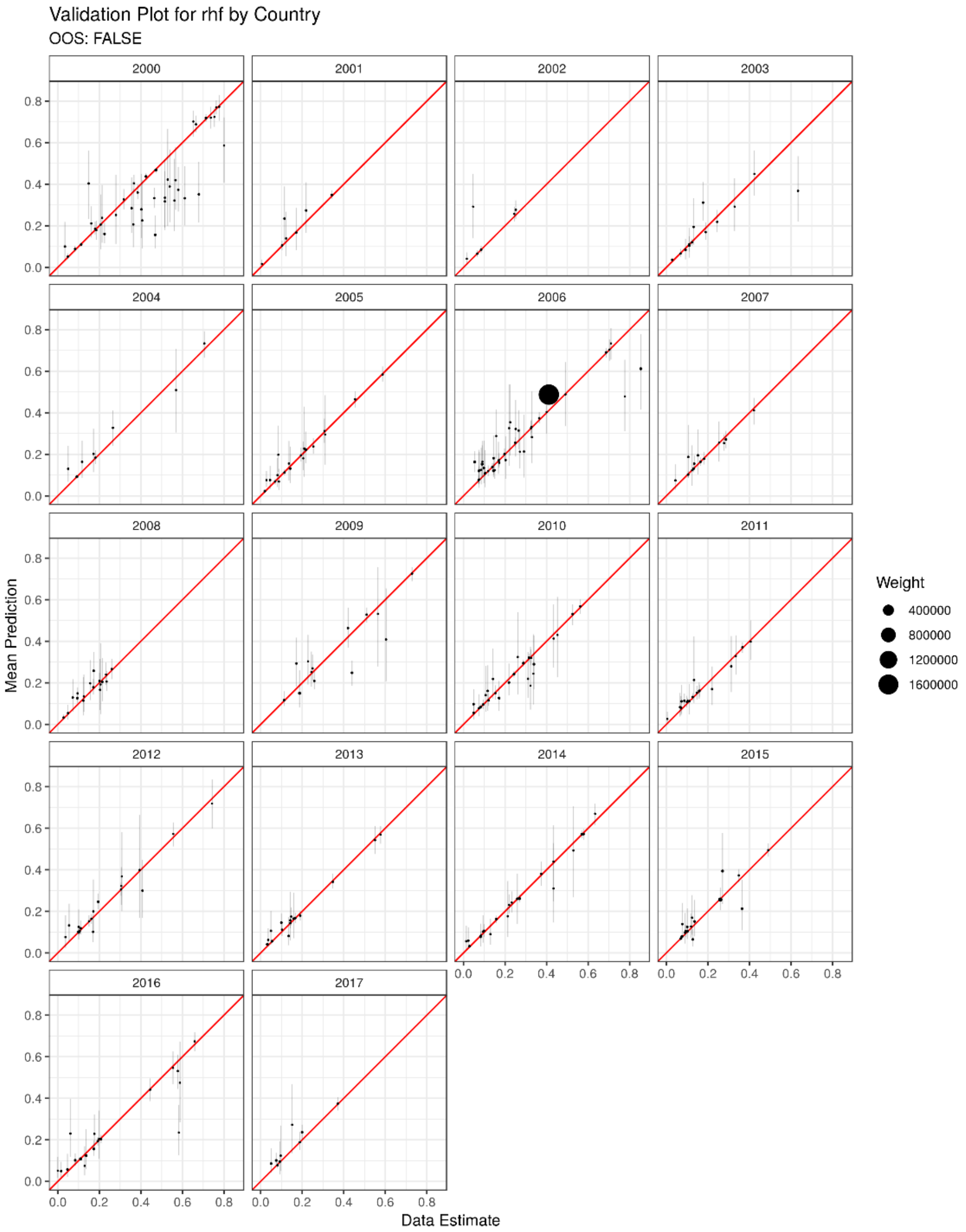

Appendix Figure 26. In-sample validation plot for RHF by first administrative unit and year

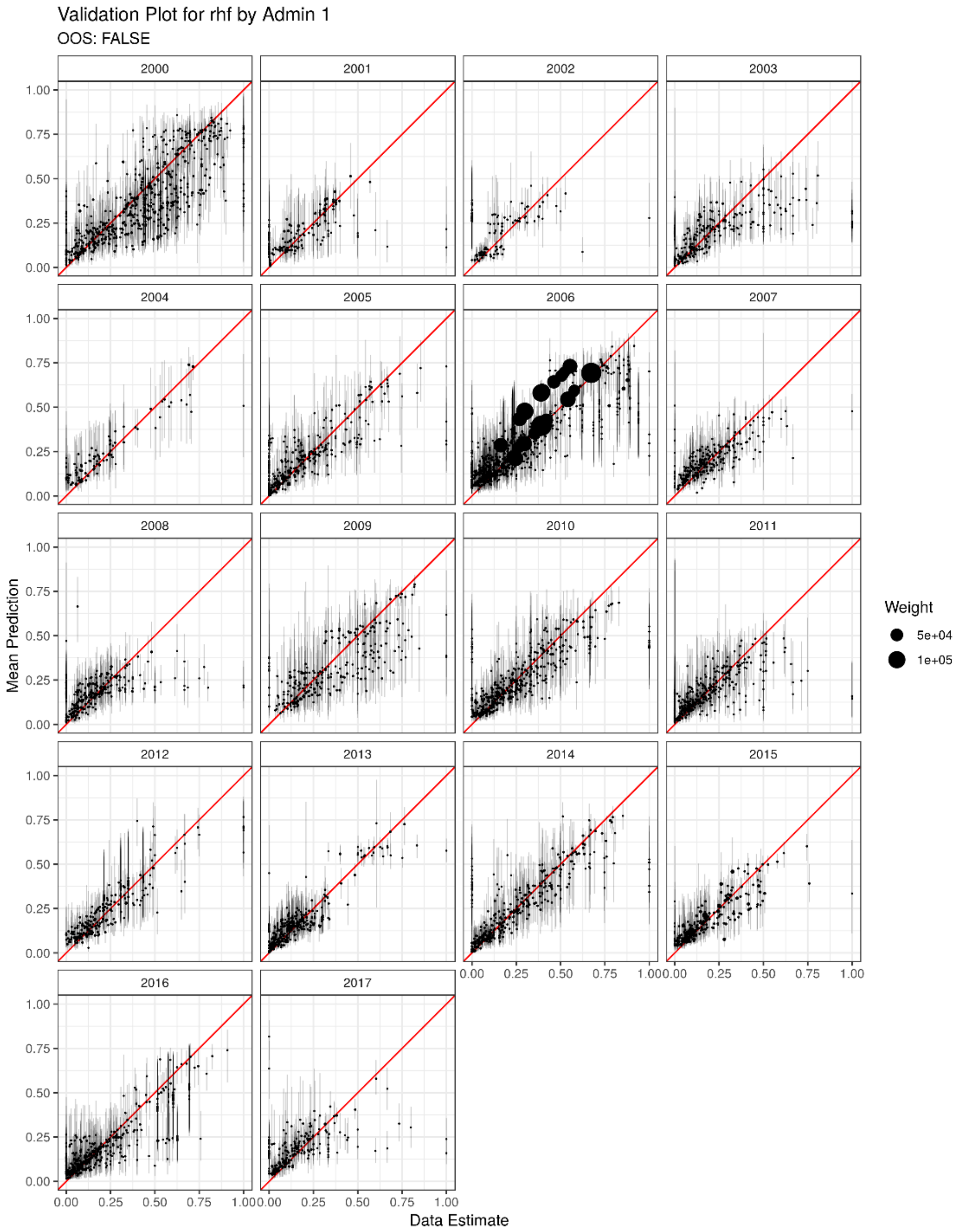

Appendix Figure 27. In-sample validation plot for RHF by second administrative unit and year

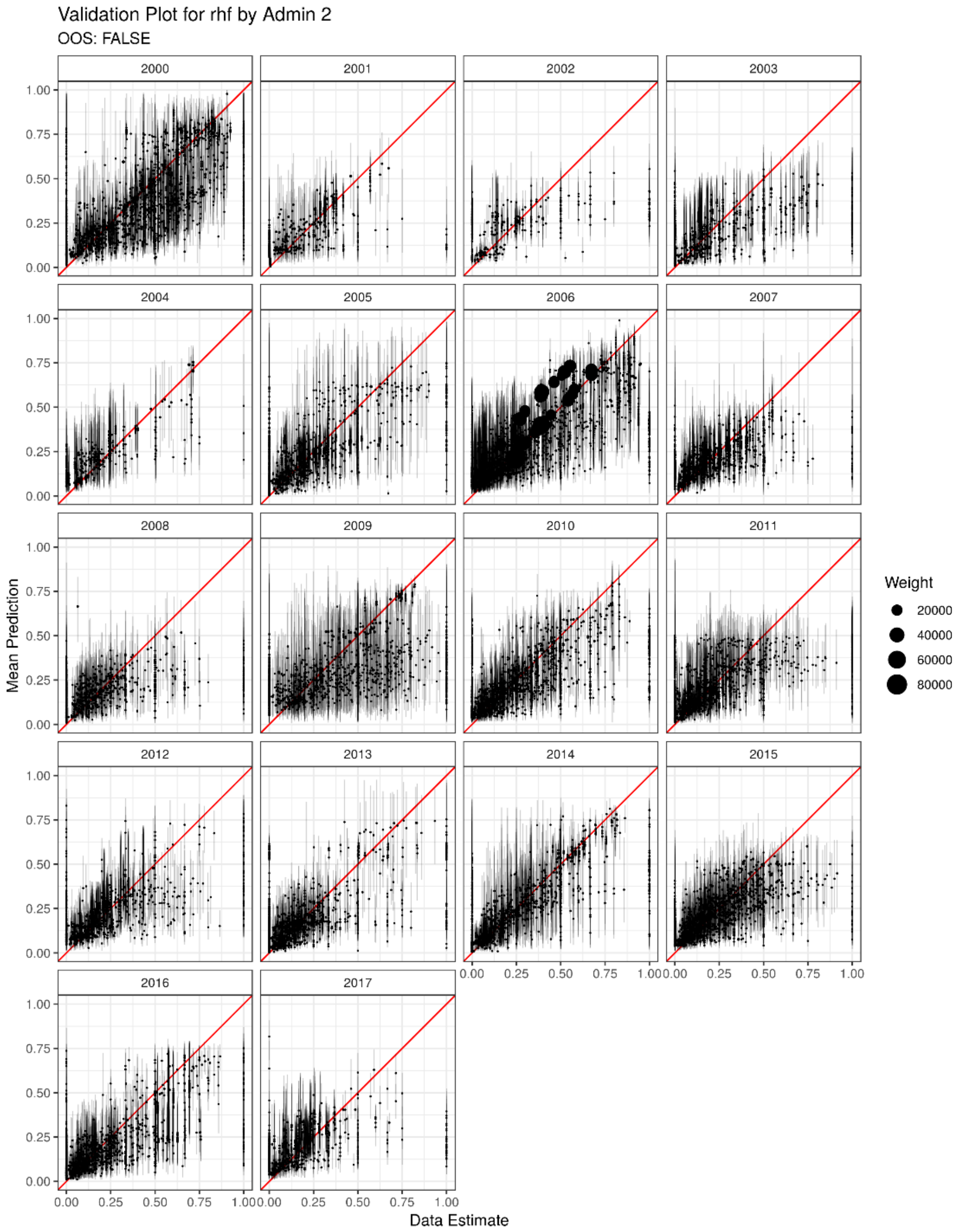

Appendix Figure 28. In-sample validation plot for ORT by country and region

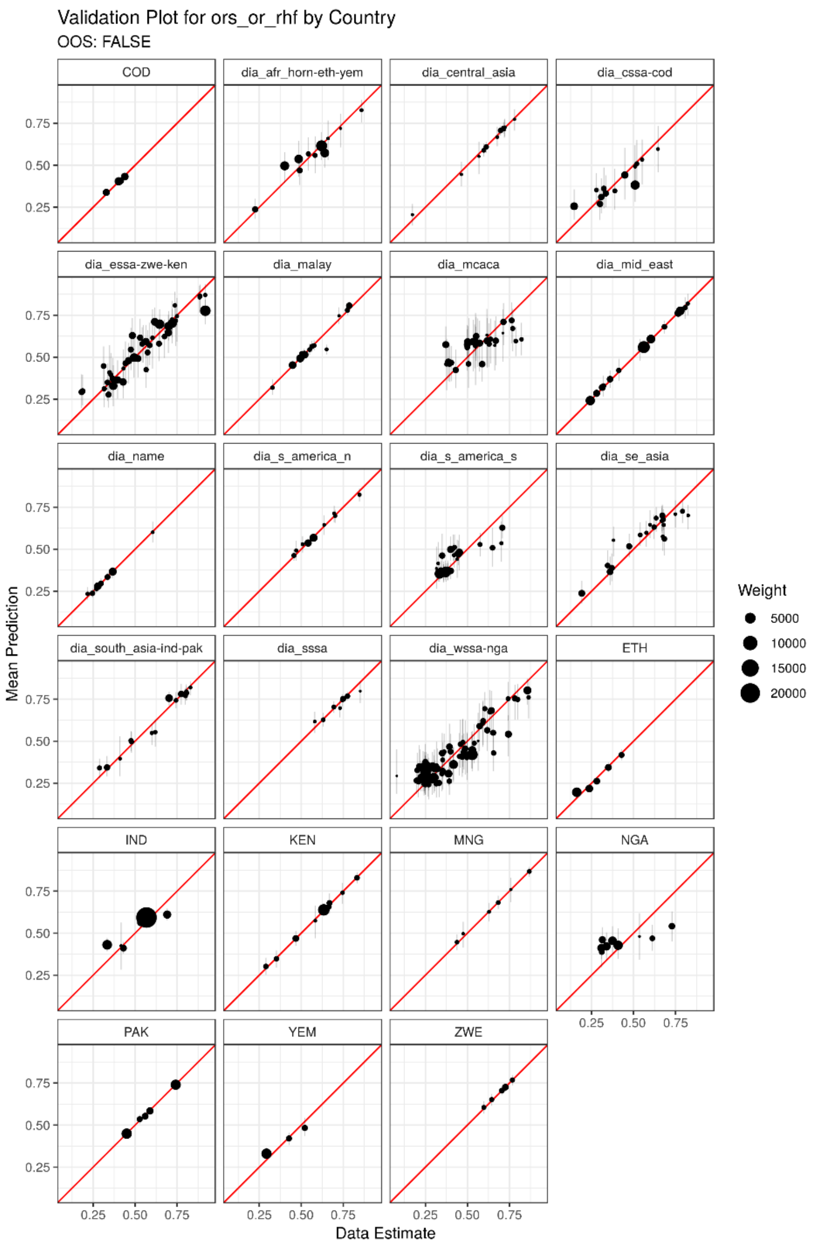

Appendix Figure 29. In-sample validation plot for ORT by first administrative unit and region

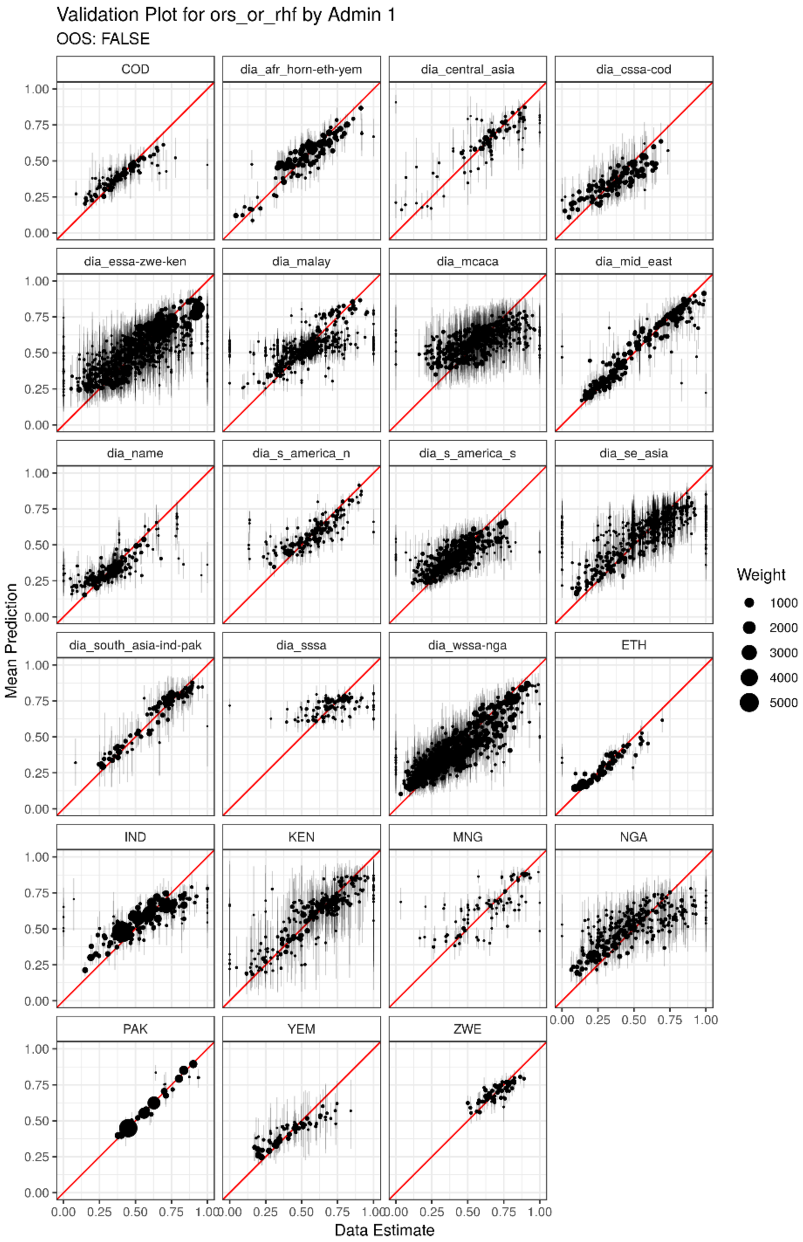

Appendix Figure 30. In-sample validation plot for ORT by second administrative unit and region

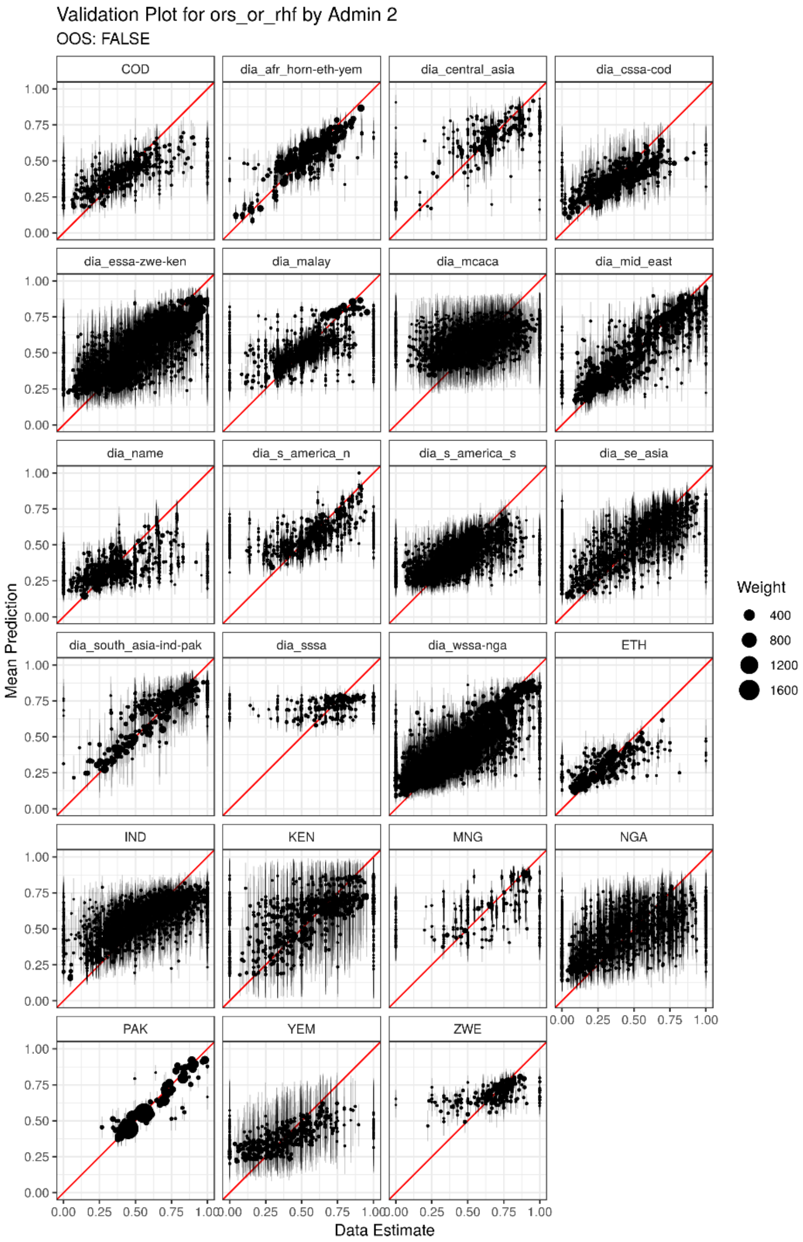

Appendix Figure 31. In-sample validation plot for ORT by country and year

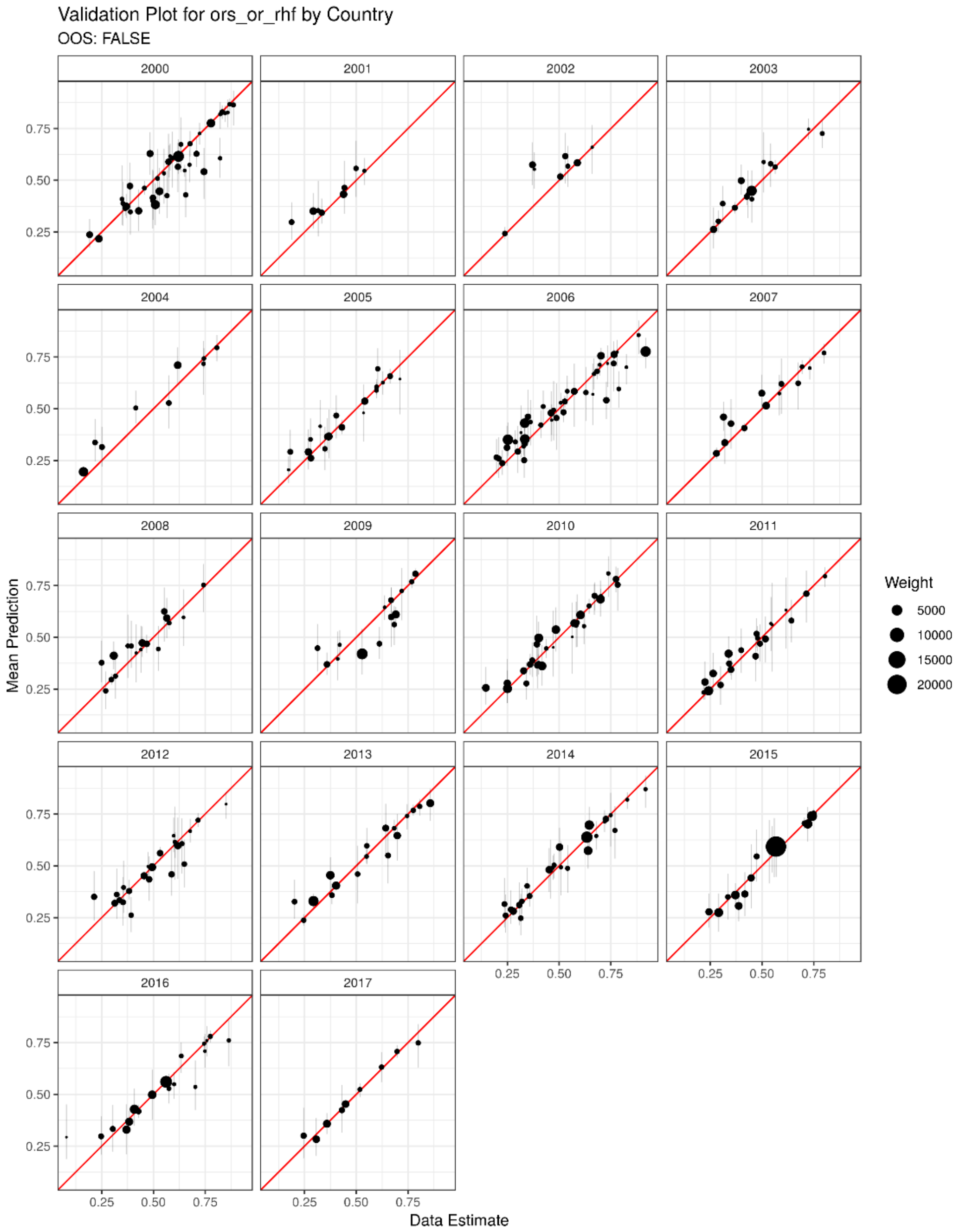

Appendix Figure 32. In-sample validation plot for ORT by first administrative unit and year

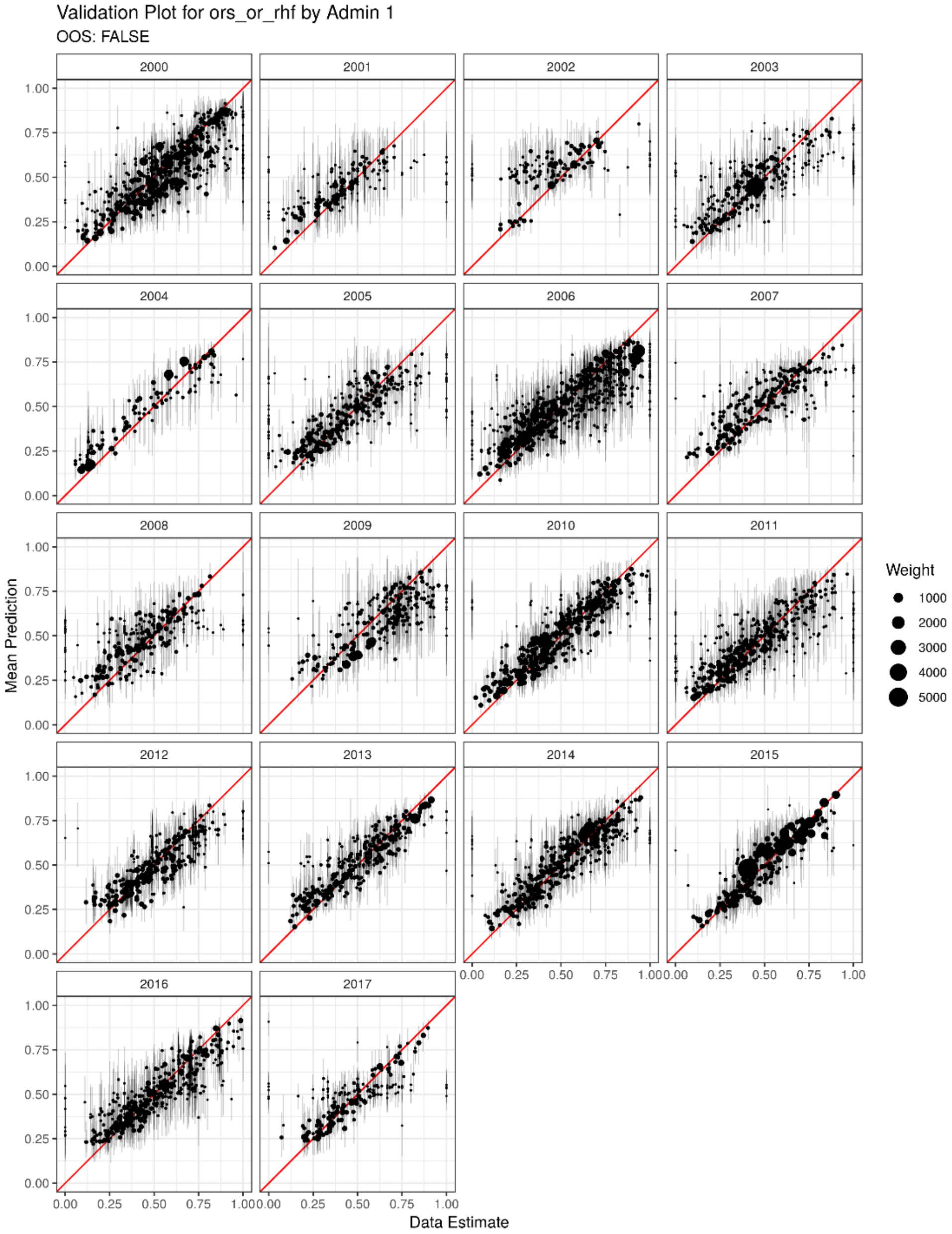

Appendix Figure 33. In-sample validation plot for ORT by second administrative unit and year

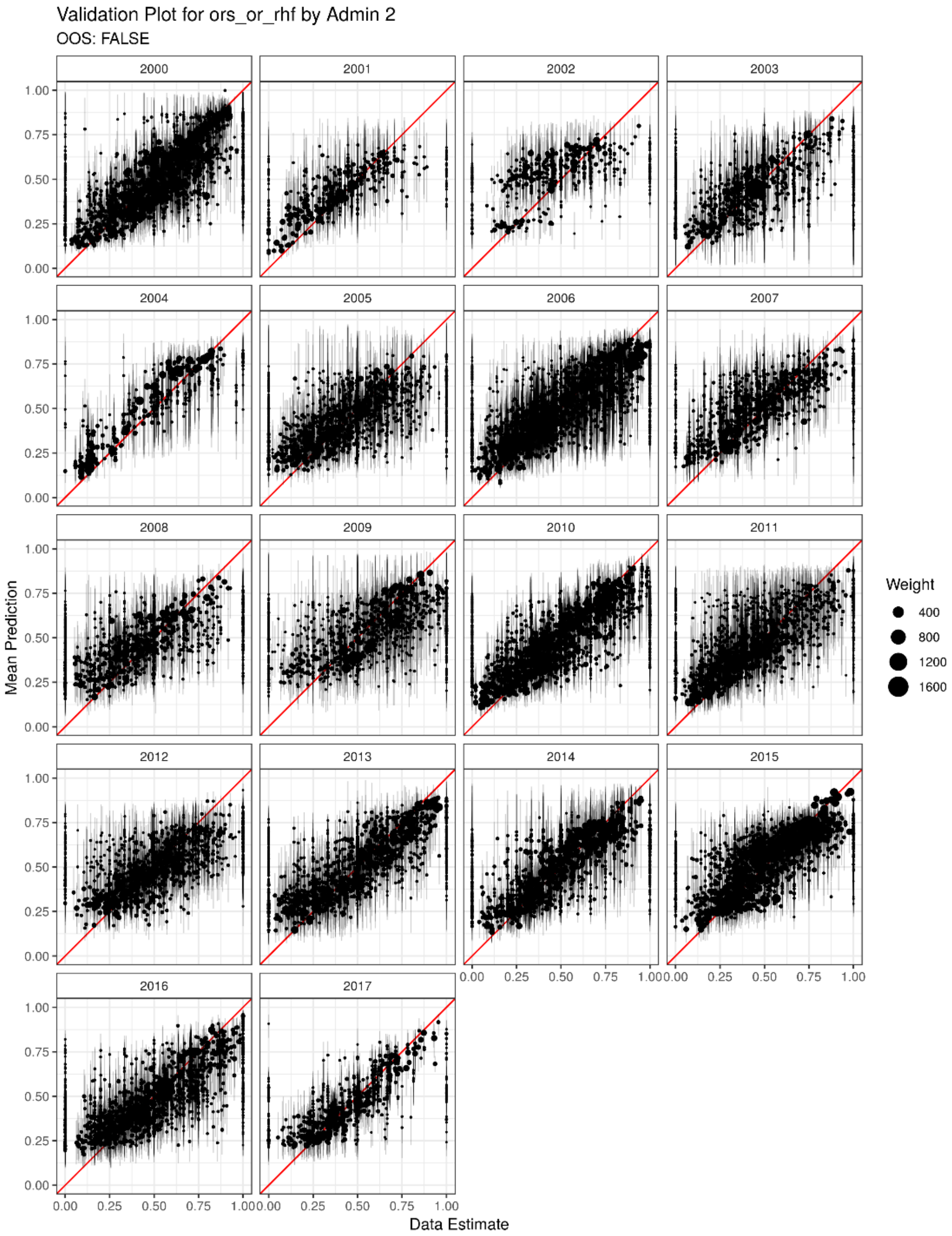

Appendix Figures 34-51. Out-of-sample validation plots for ORS, RHF, and ORT

Each plot shows coverage estimates from the survey data on the x-axis and mean posterior predictions of coverage y-axis. The size of each dot is proportional to sample size in the underlying data. Estimates are shown aggregated to country, first administrative, and second administrative levels. Estimates are also shown stratified by region and by year. For corresponding out-of-sample fit statistics see appendix tables 10, 12, and 14.

Regions are labelled in the following manner: the Democratic Republic of the Congo [*COD*], the Horn of Africa [*dia_afr_horn-eth-yem*], Central Asia [*dia_central_asia*], Central sub-Saharan Africa [*dia_cssa-cod*], Eastern sub-Saharan Africa [*dia_essa-zwe-ken*], Malay Archipelago [*dia_malay*], Mexico, the Caribbean, and Central America [*dia_mcaca*], the Middle East [*dia_mid_east*], North Africa and the Middle East [*dia_name*], northern South America [*dia_s_america_n*], southern South America [*dia_s_america_s*], Southeast Asia [*dia_se_asia*], South Asia [*dia_south_asia-ind-pak*], Southern sub-Saharan Africa [*dia_sssa*], Western sub-Saharan Africa [*dia_wssa-nga*], Ethiopia [*ETH*], India [*IND*], Kenya [*KEN*], Mongolia [*MNG*], Nigeria [*NGA*], Pakistan [*PAK*], Yemen [*YEM*], and Zimbabwe [*ZWE*].

Appendix Figure 34. Out-of-sample validation plot for ORS by country and region

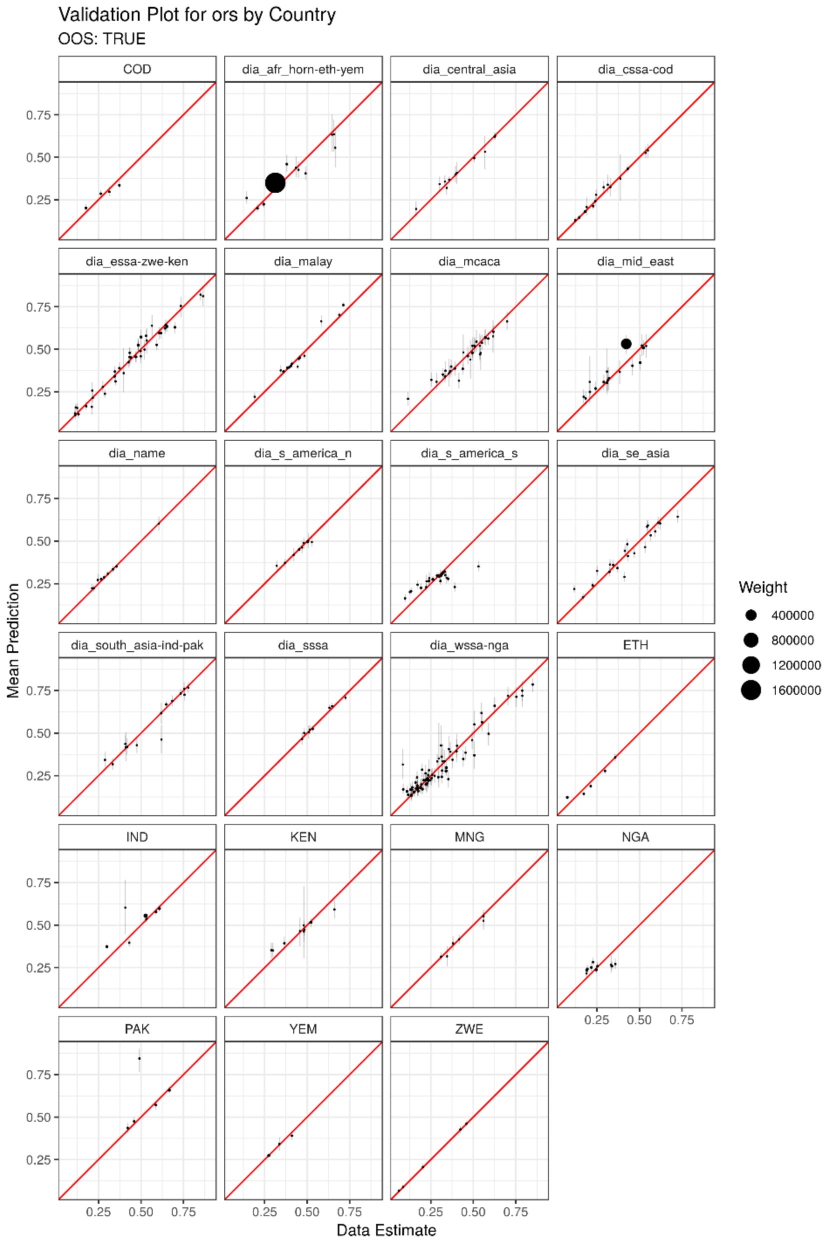

Appendix Figure 35. Out-of-sample validation plot for ORS by first administrative unit and region

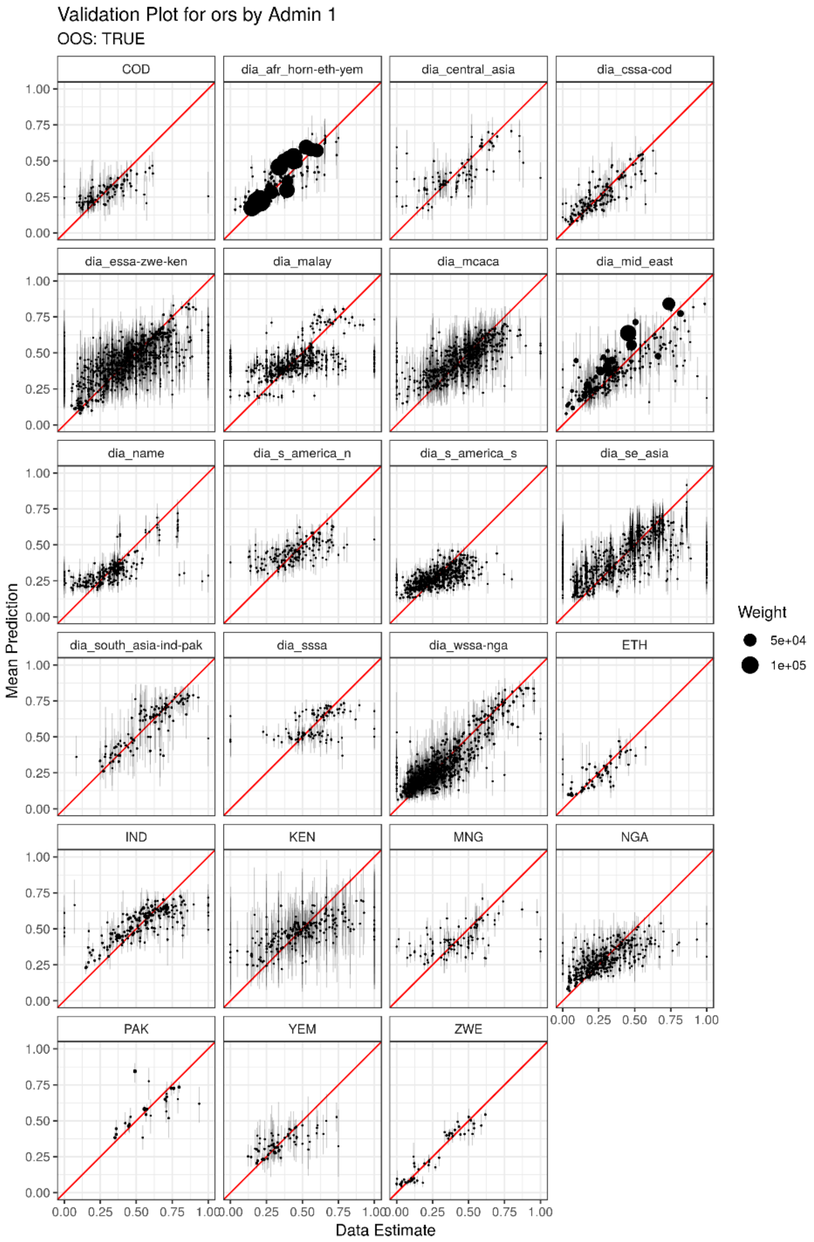

Appendix Figure 36. Out-of-sample validation plot for ORS by second administrative unit and region

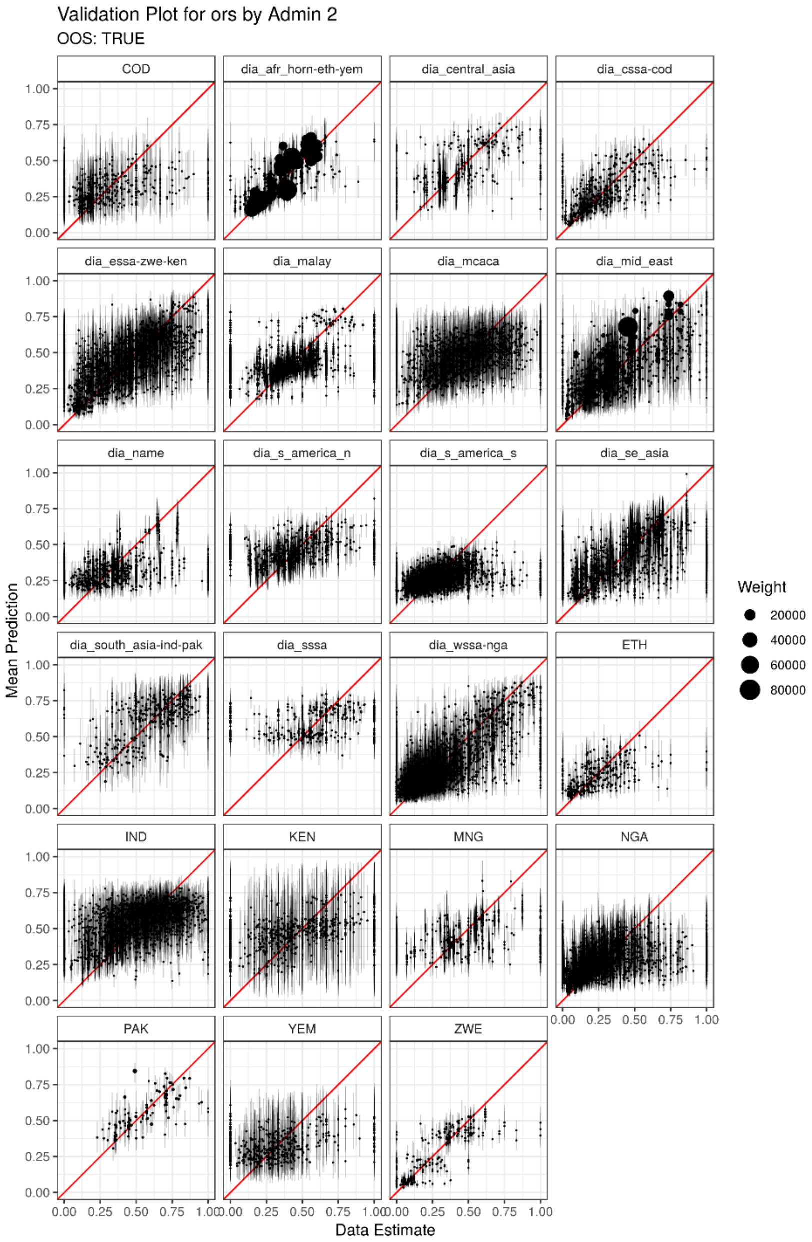

Appendix Figure 37. Out-of-sample validation plot for ORS by country and year

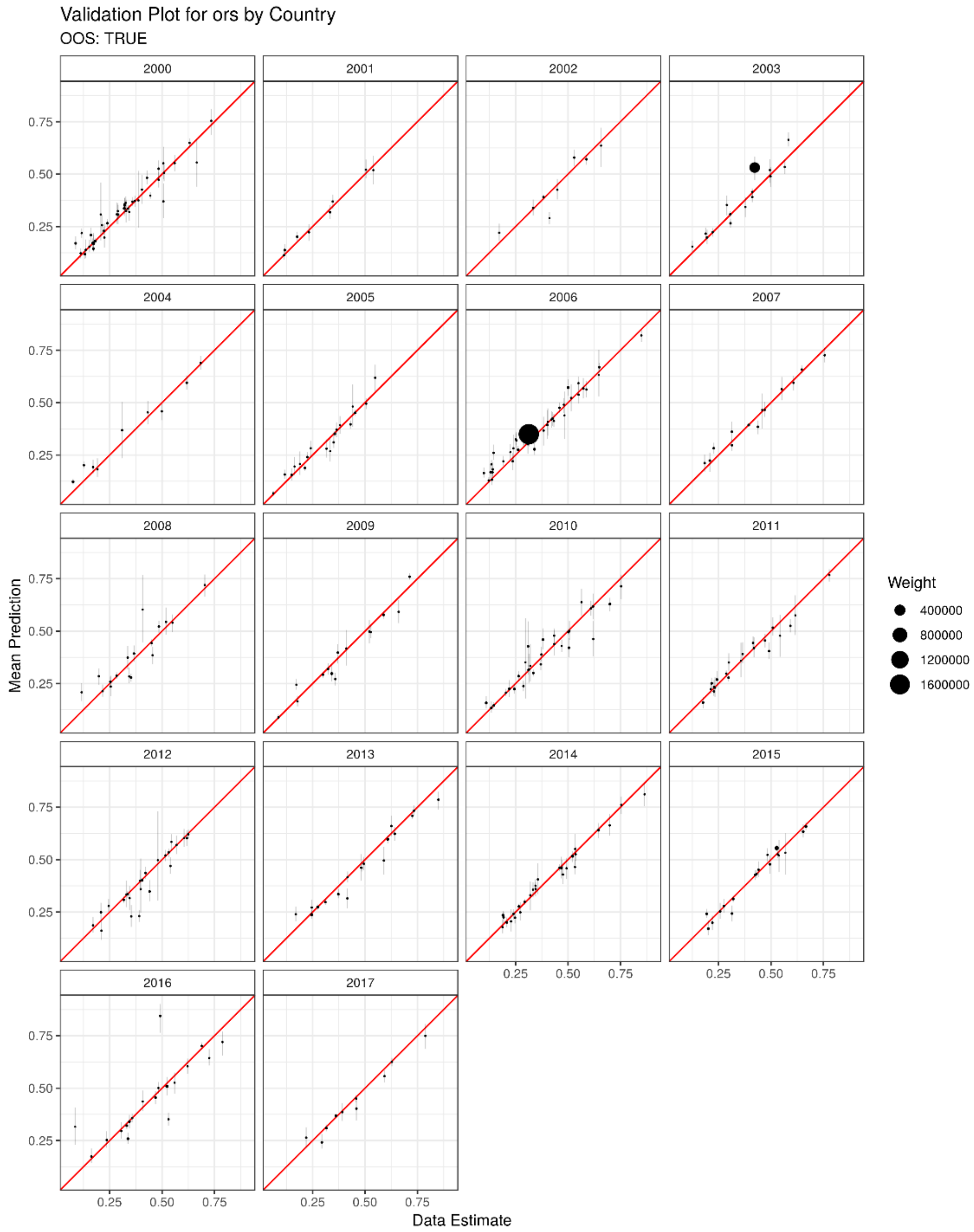

Appendix Figure 38. Out-of-sample validation plot for ORS by first administrative unit and year

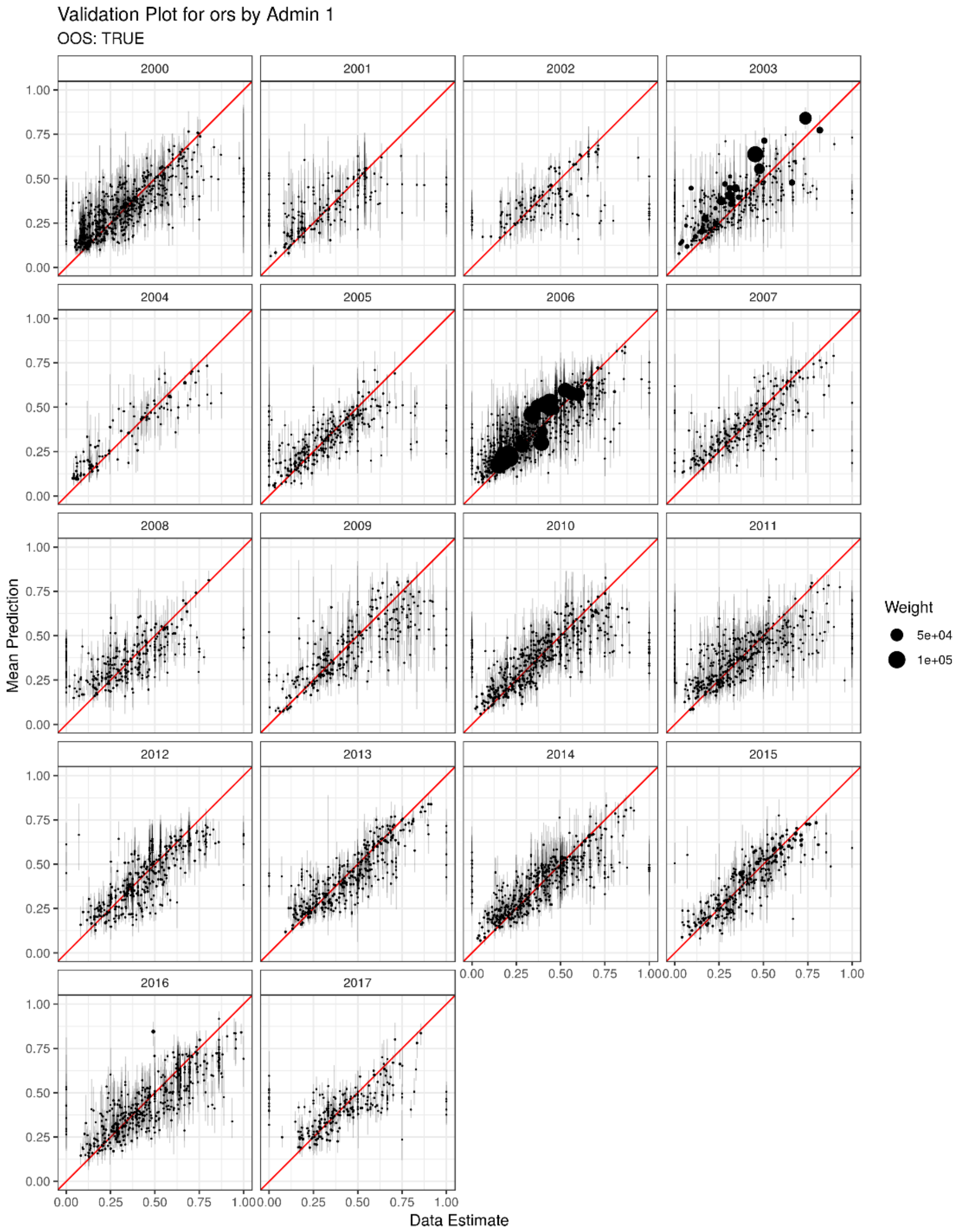

Appendix Figure 39. Out-of-sample validation plot for ORS by second administrative unit and year

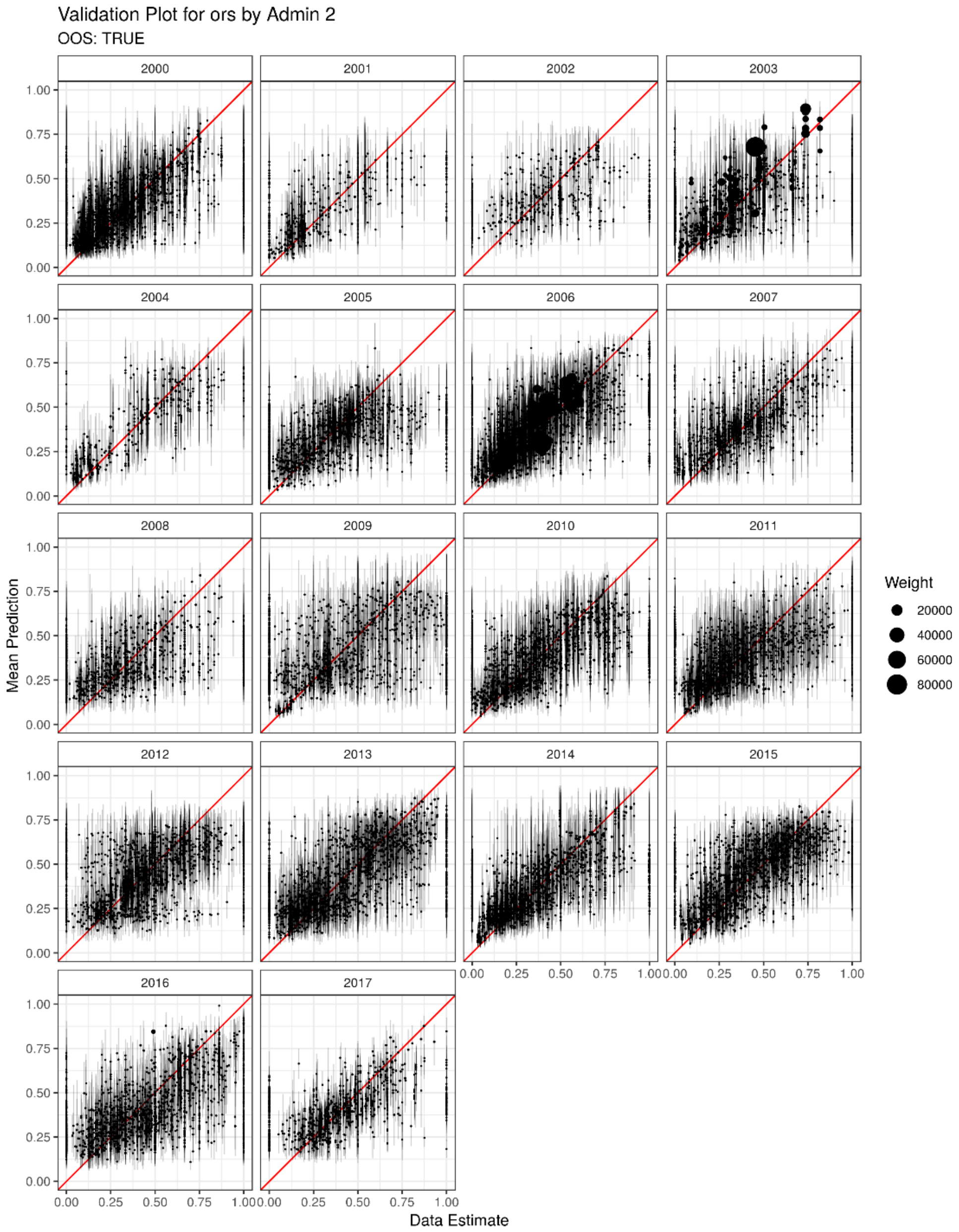

Appendix Figure 40. Out-of-sample validation plot for RHF by country and region

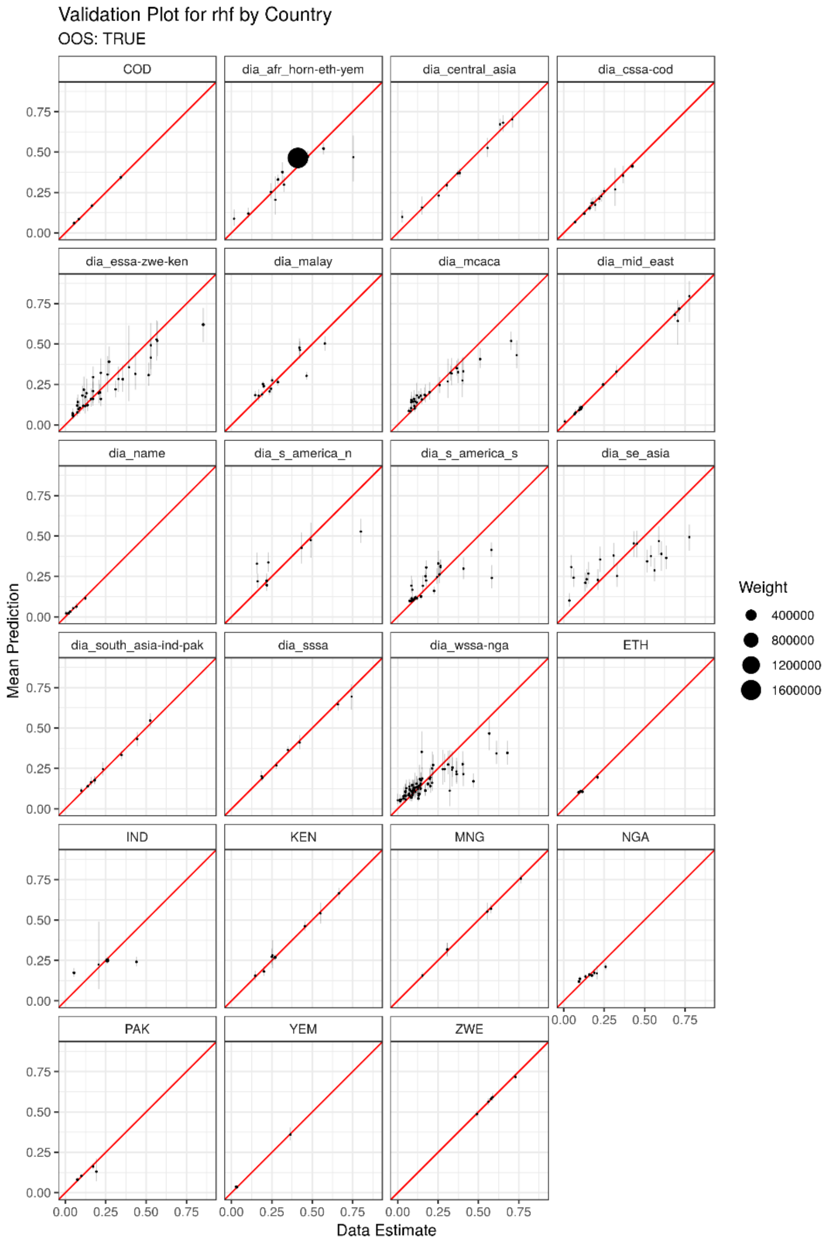

Appendix Figure 41. Out-of-sample validation plot for RHF by first administrative unit and region

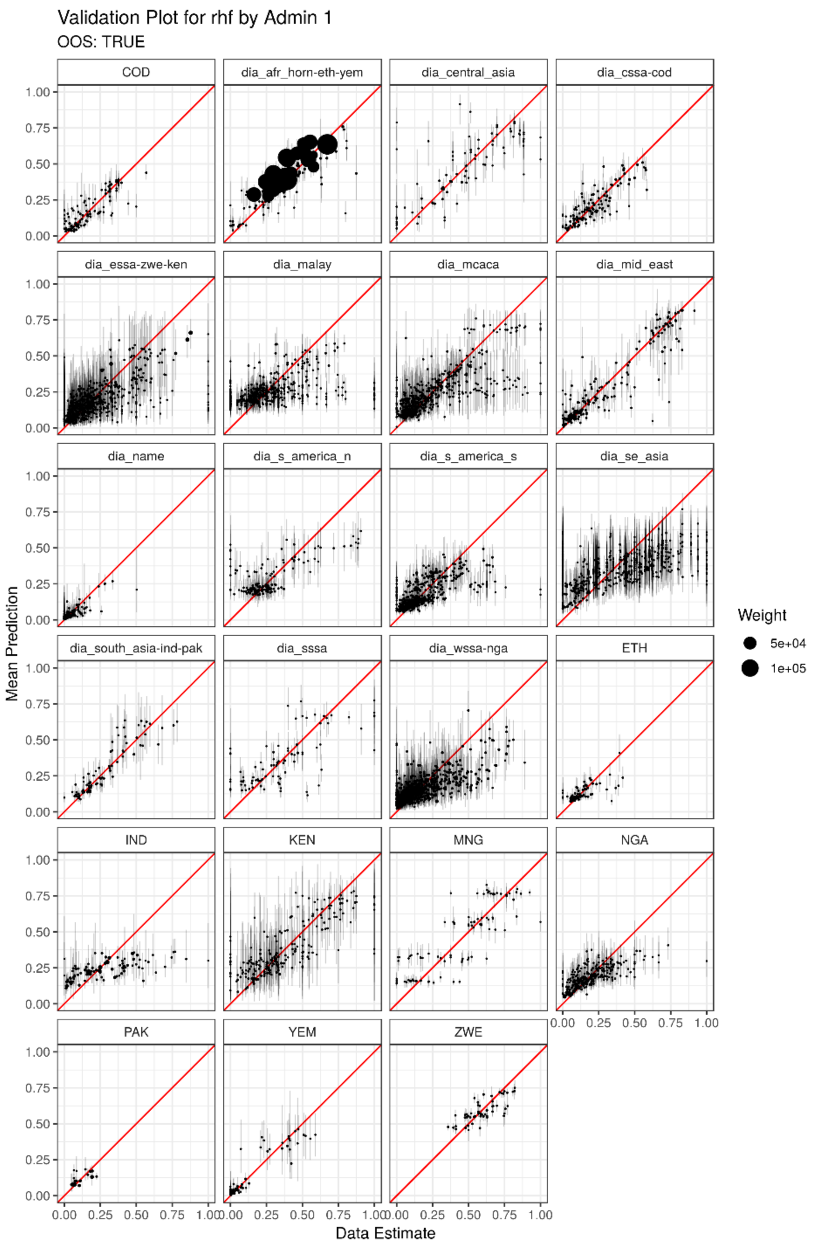

Appendix Figure 42. Out-of-sample validation plot for RHF by second administrative unit and region

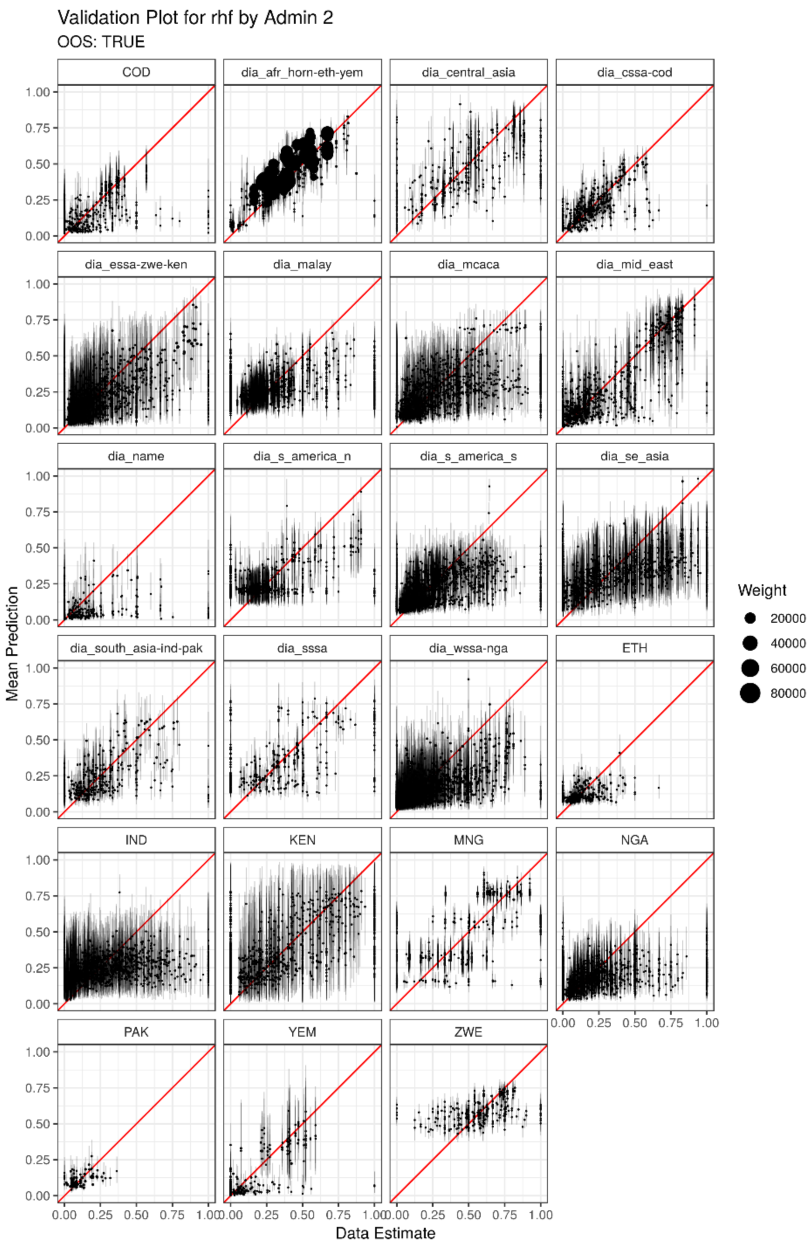

Appendix Figure 43. Out-of-sample validation plot for RHF by country and year

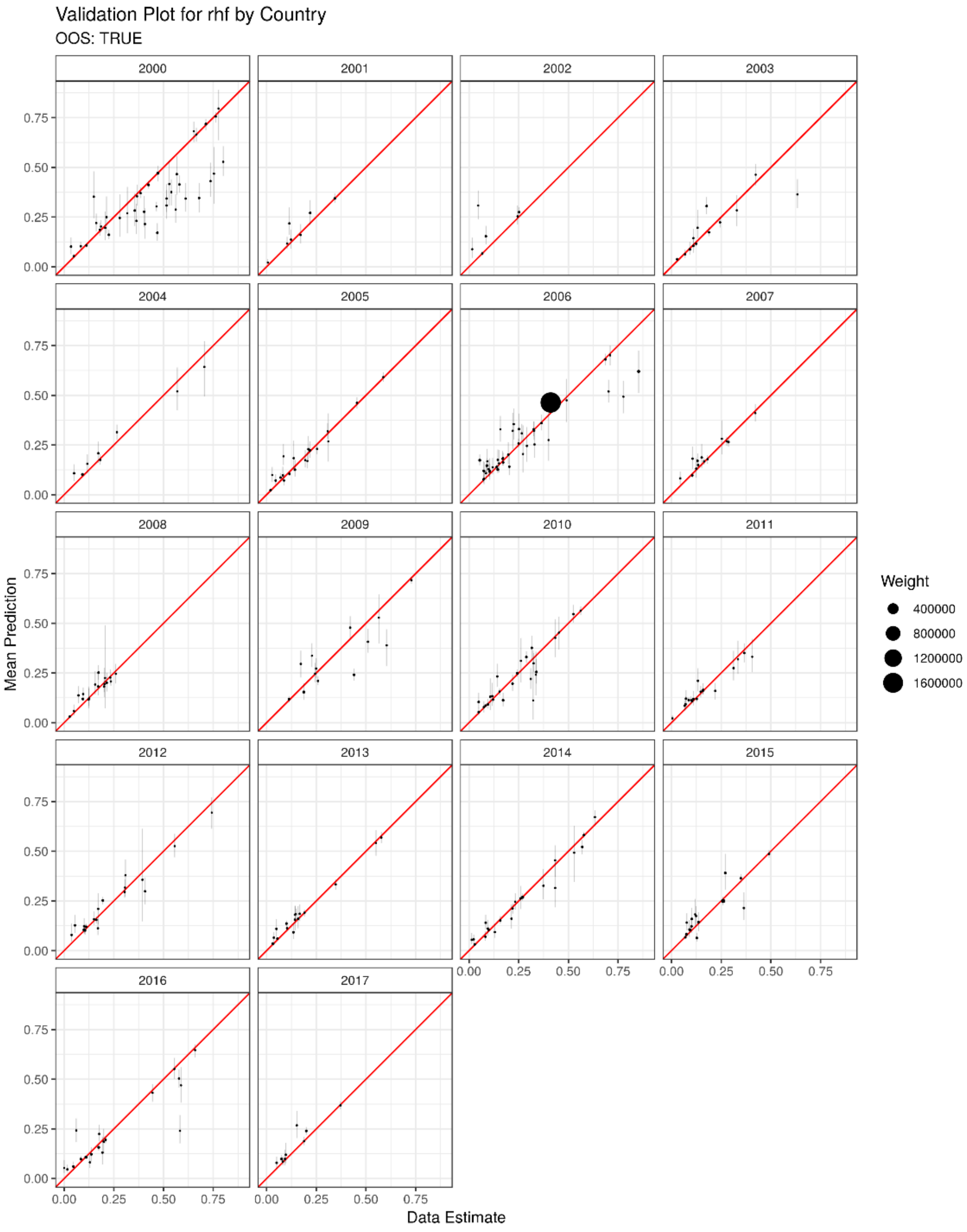

Appendix Figure 44. Out-of-sample validation plot for RHF by first administrative unit and year

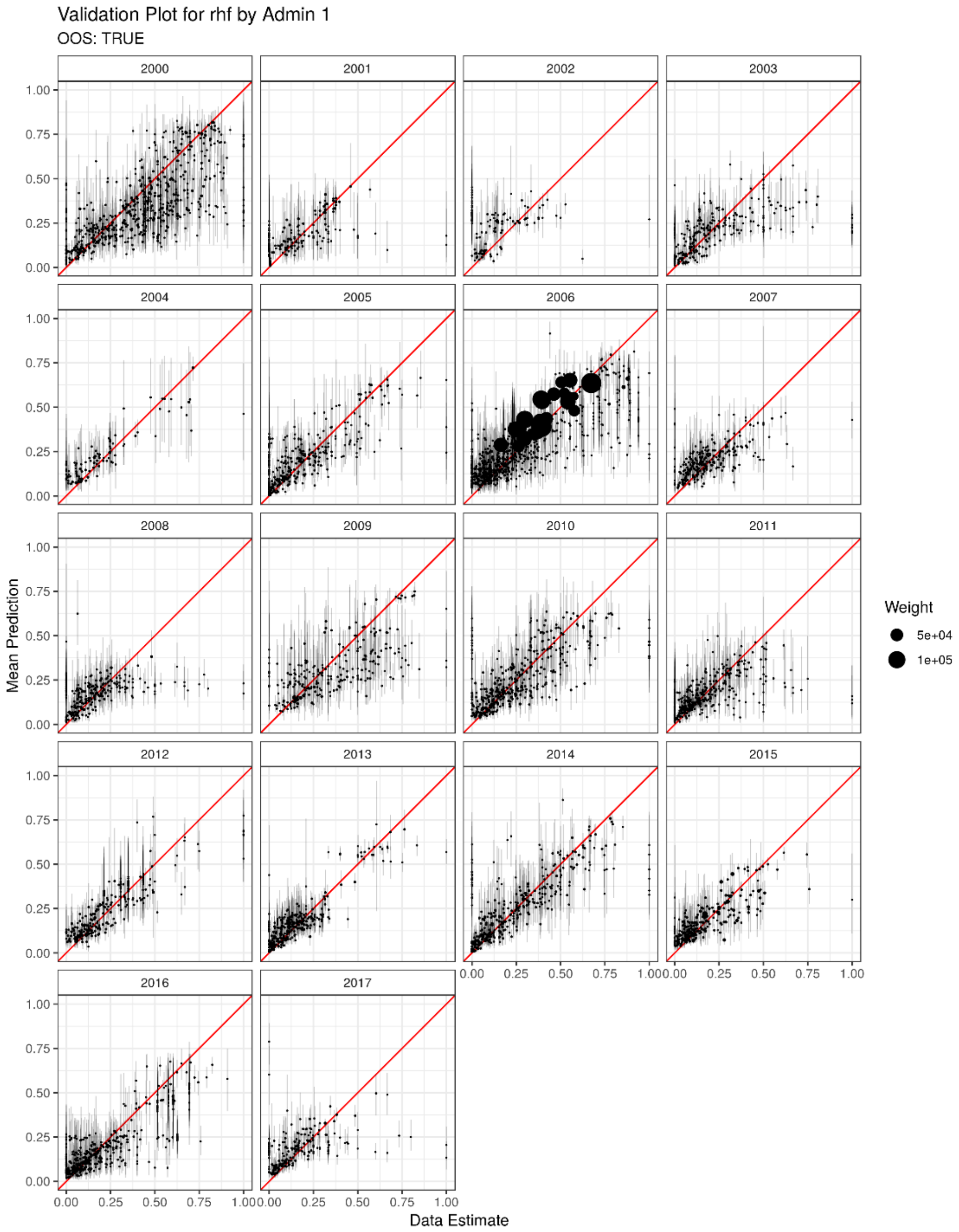

Appendix Figure 45. Out-of-sample validation plot for RHF by second administrative unit and year

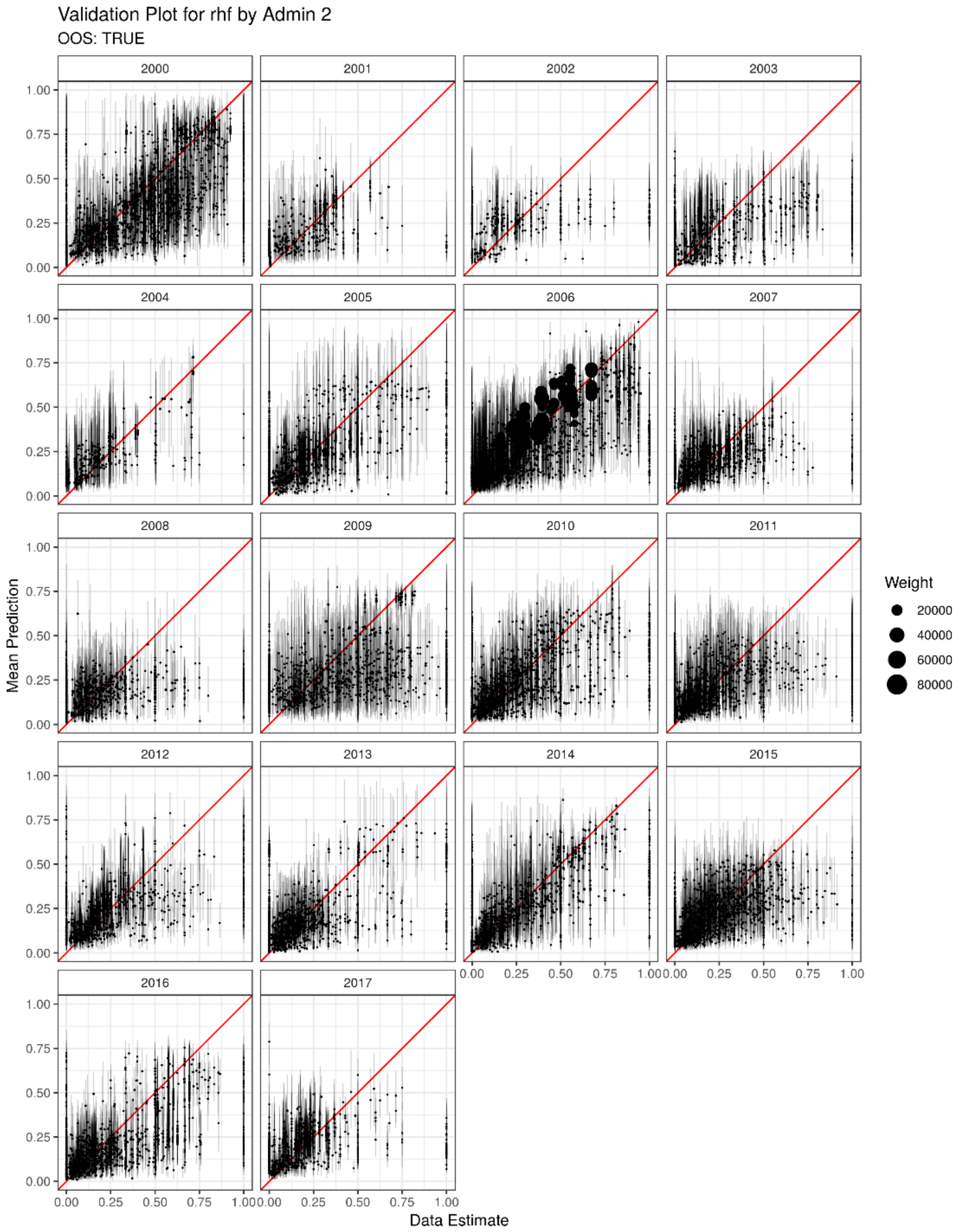

Appendix Figure 46. Out-of-sample validation plot for ORT by country and region

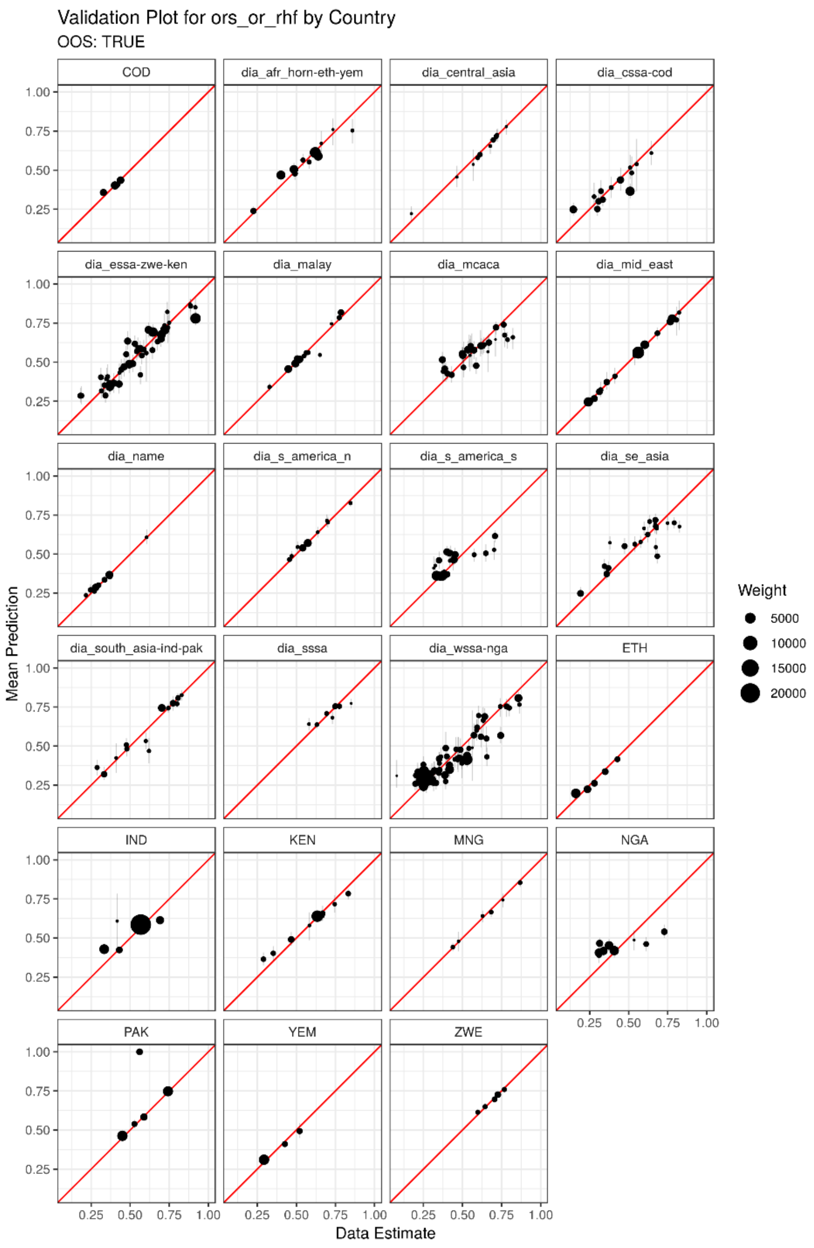

Appendix Figure 47. Out-of-sample validation plot for ORT by first administrative unit and region

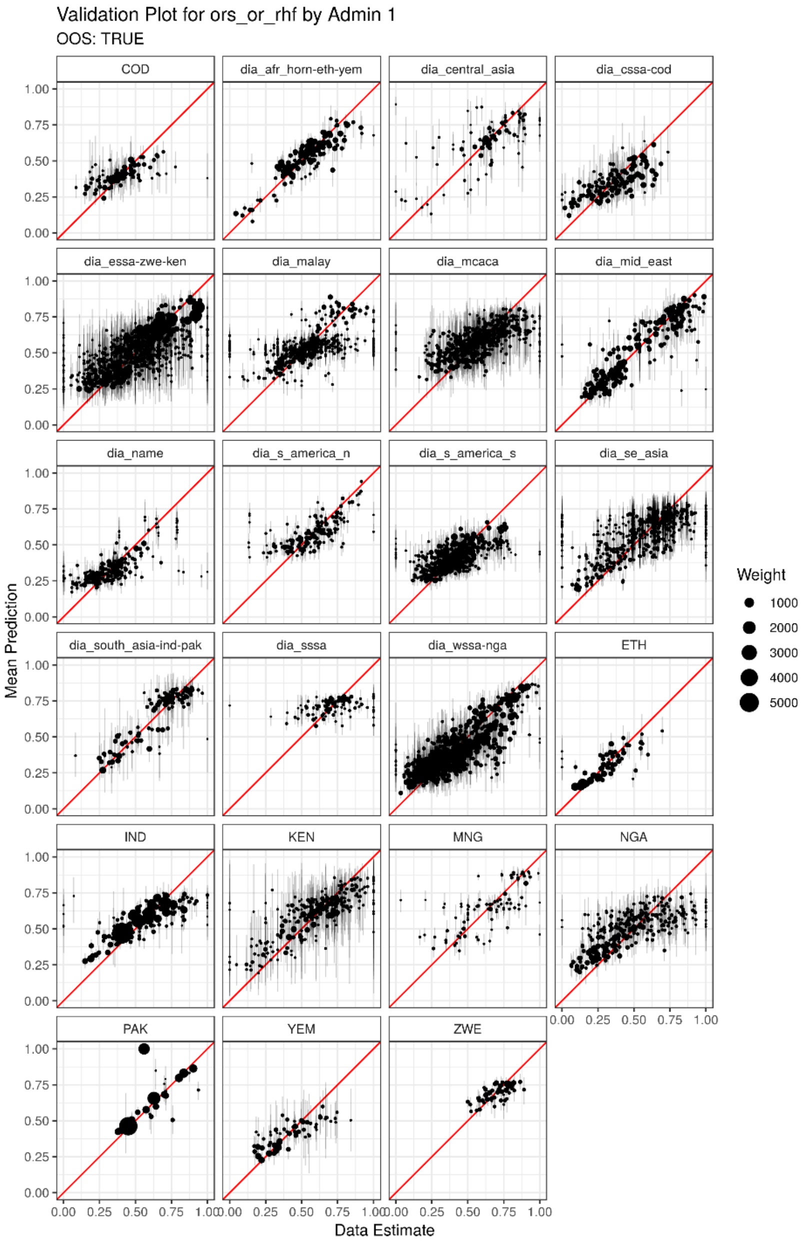

.

Appendix Figure 48. Out-of-sample validation plot for ORT by second administrative unit and region

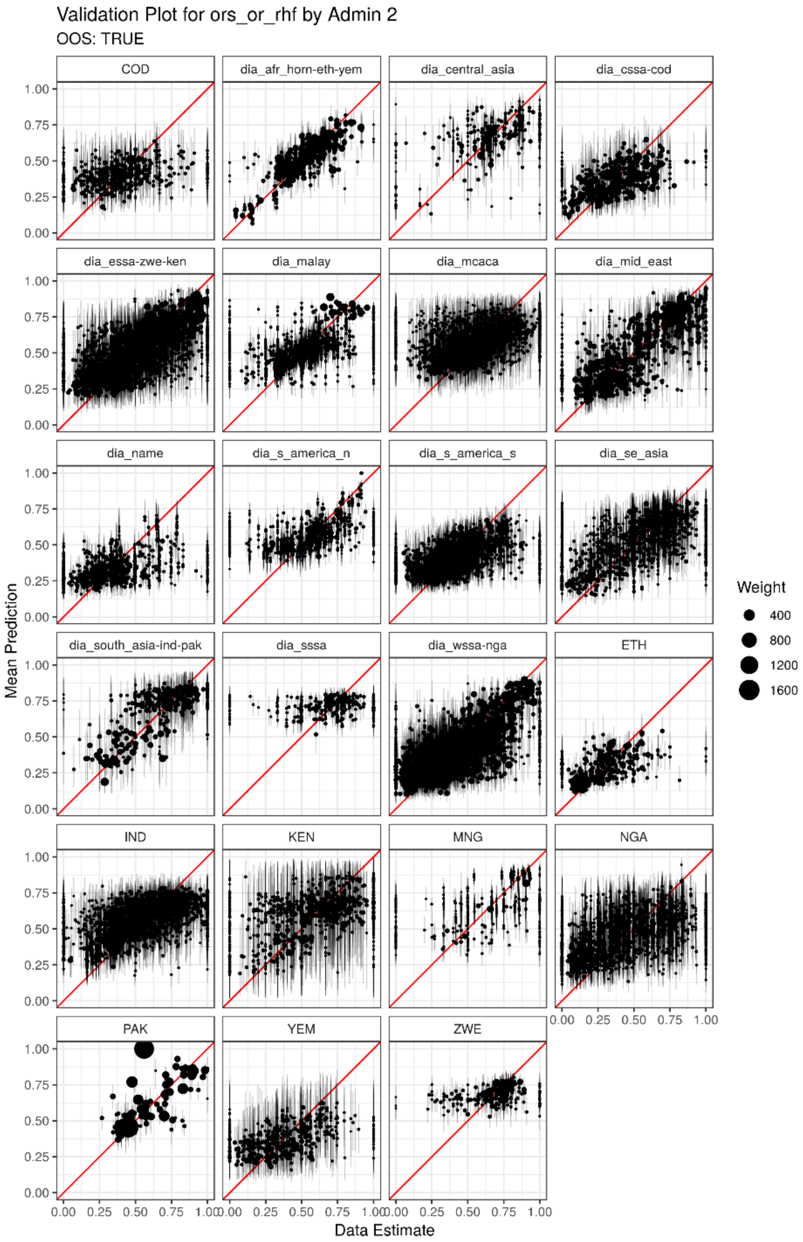

Appendix Figure 49. Out-of-sample validation plot for ORT by country and year

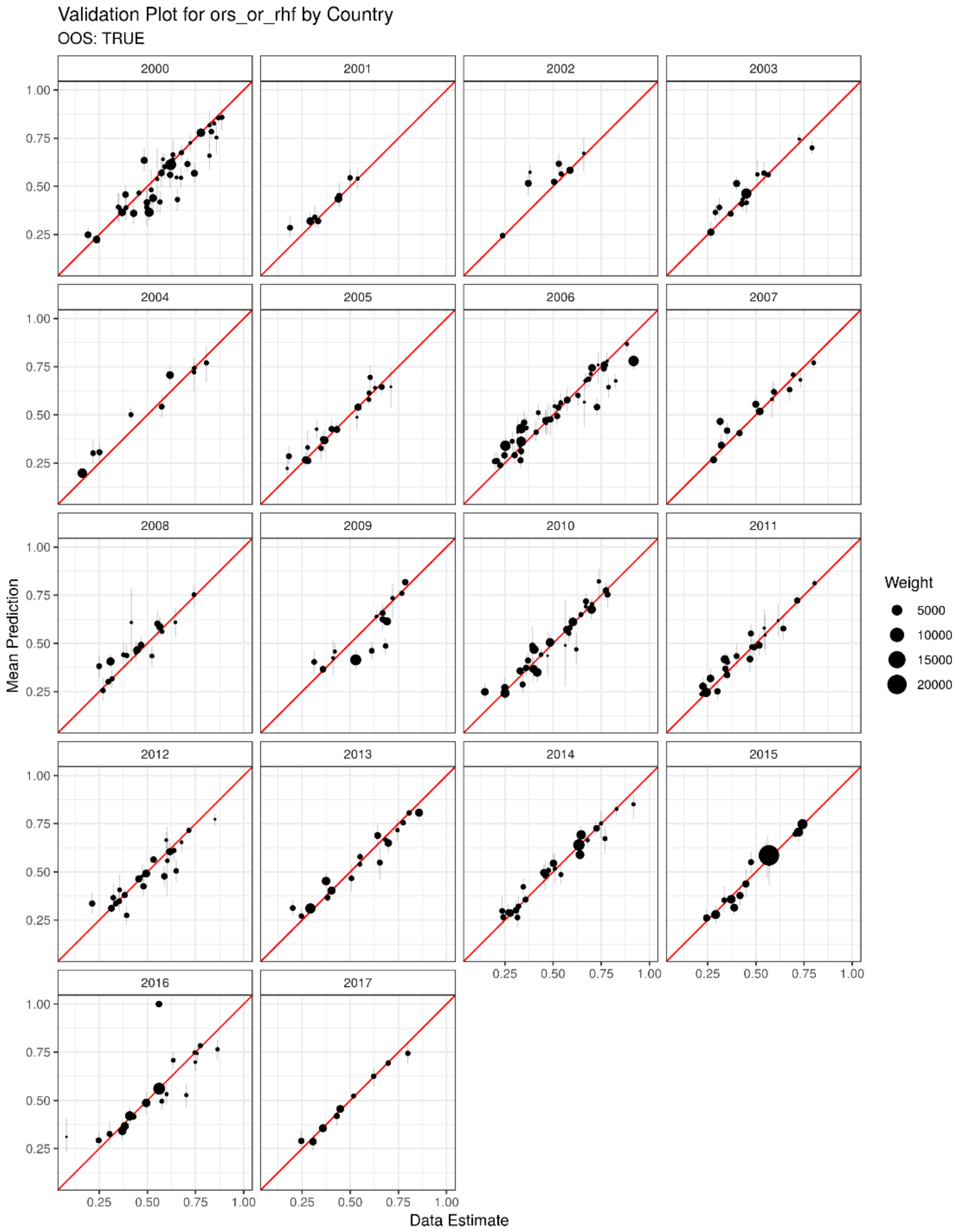

Appendix Figure 50. Out-of-sample validation plot for ORT by first administrative unit and year

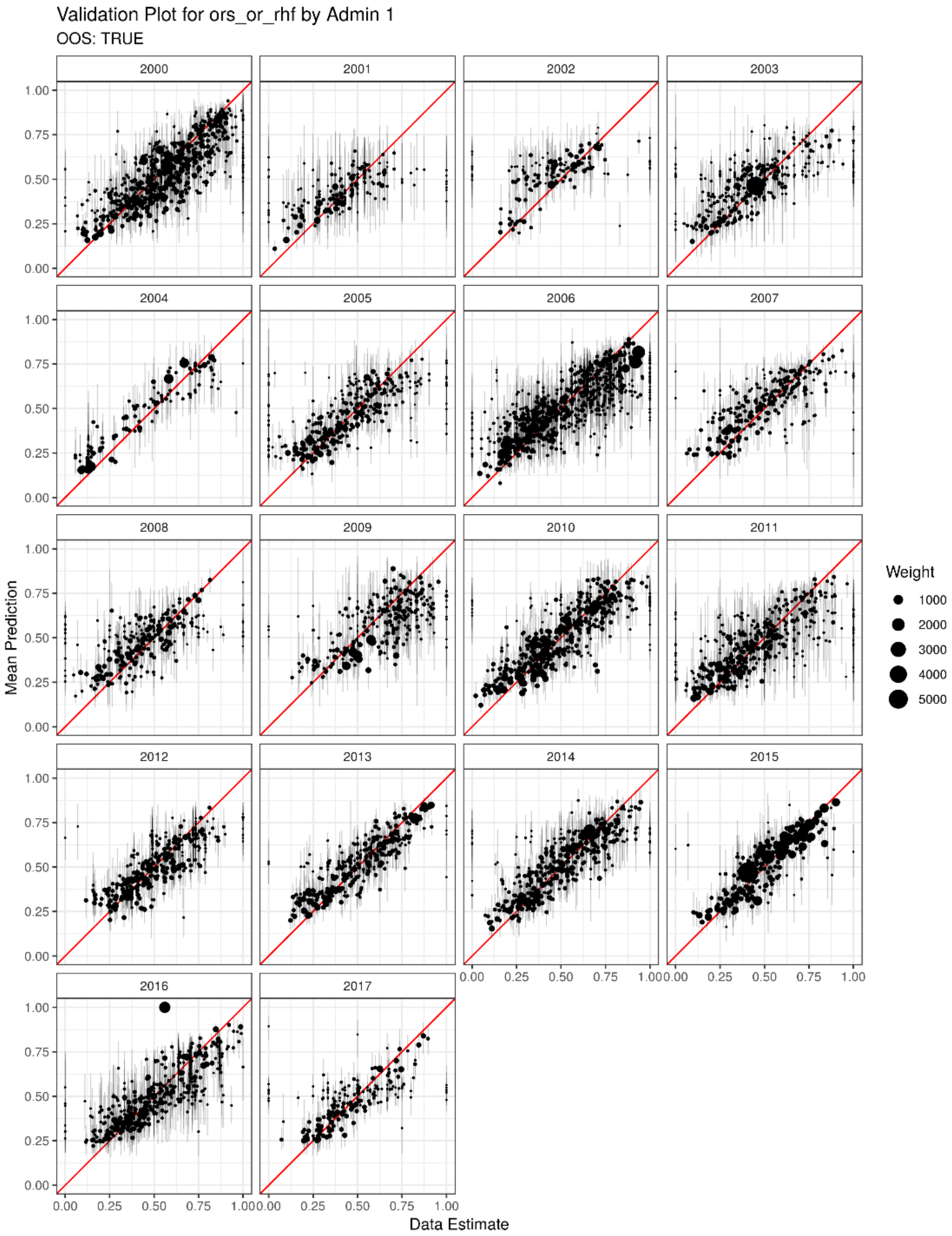

Appendix Figure 51. Out-of-sample validation plot for ORT by second administrative unit and year

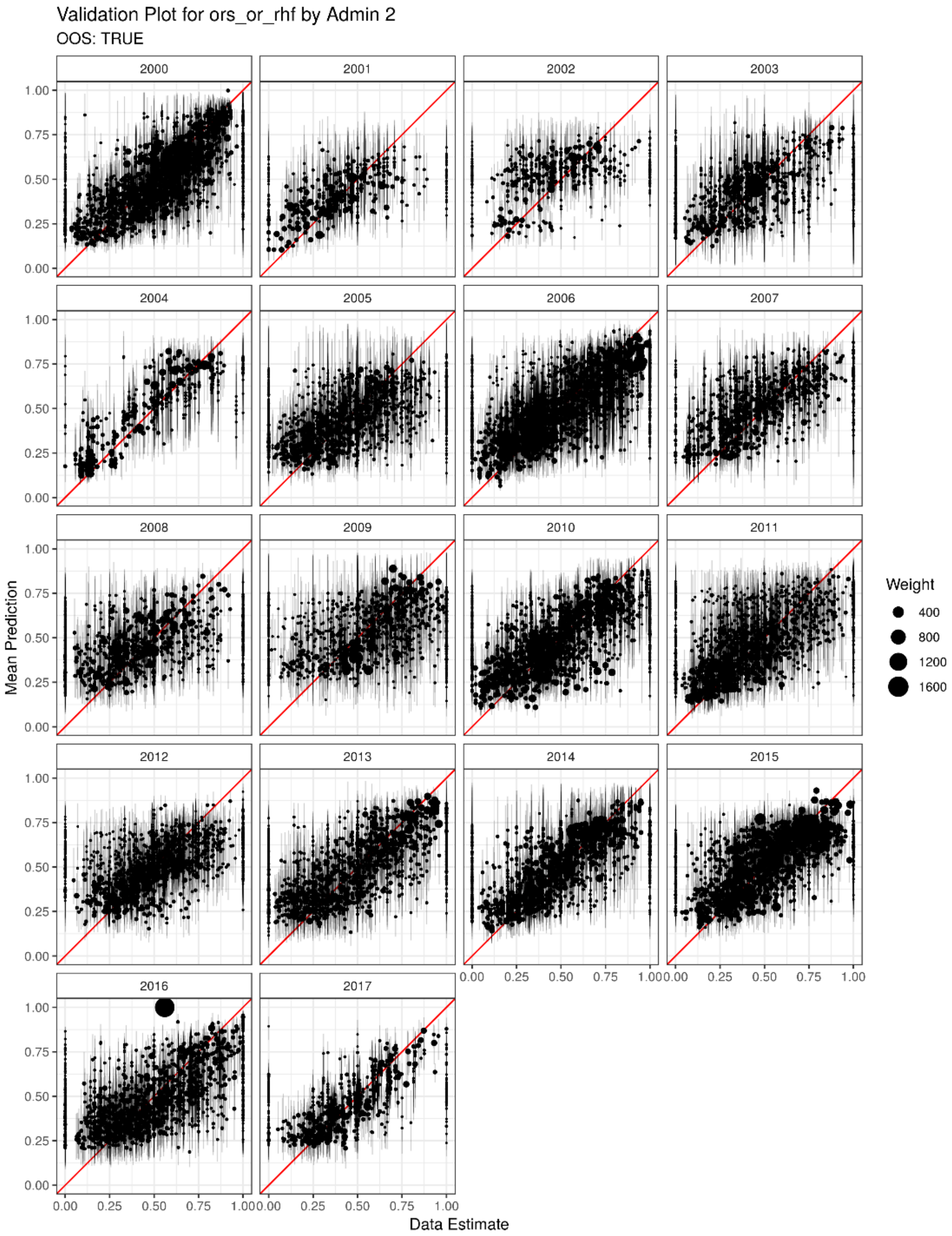

Appendix Figure 52. ORS annualised rate of change, 2000–2017

Maps reflect administrative boundaries, land cover, lakes, and population; dark grey-coloured grid cells were classified as "barren or sparsely vegetated" and had fewer than ten people per 1 × 1-km grid cell; light grey countries were not included in these analyses.[7,21–25]

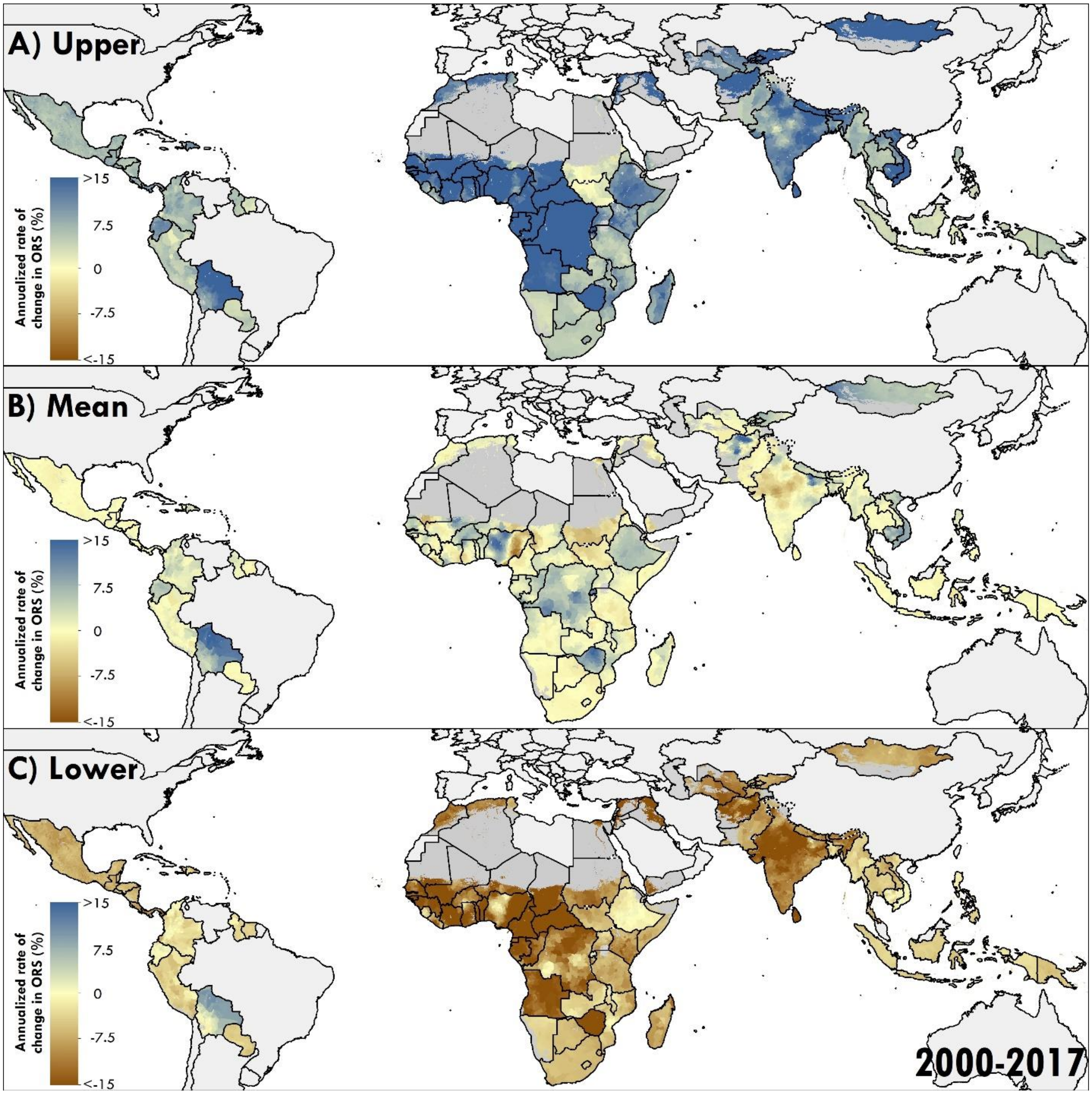

Appendix Figure 53. RHF annualised rate of change, 2000–2017

Maps reflect administrative boundaries, land cover, lakes, and population; dark grey-coloured grid cells were classified as "barren or sparsely vegetated" and had fewer than ten people per 1 × 1-km grid cell; light grey countries were not included in these analyses.[7,21–25]

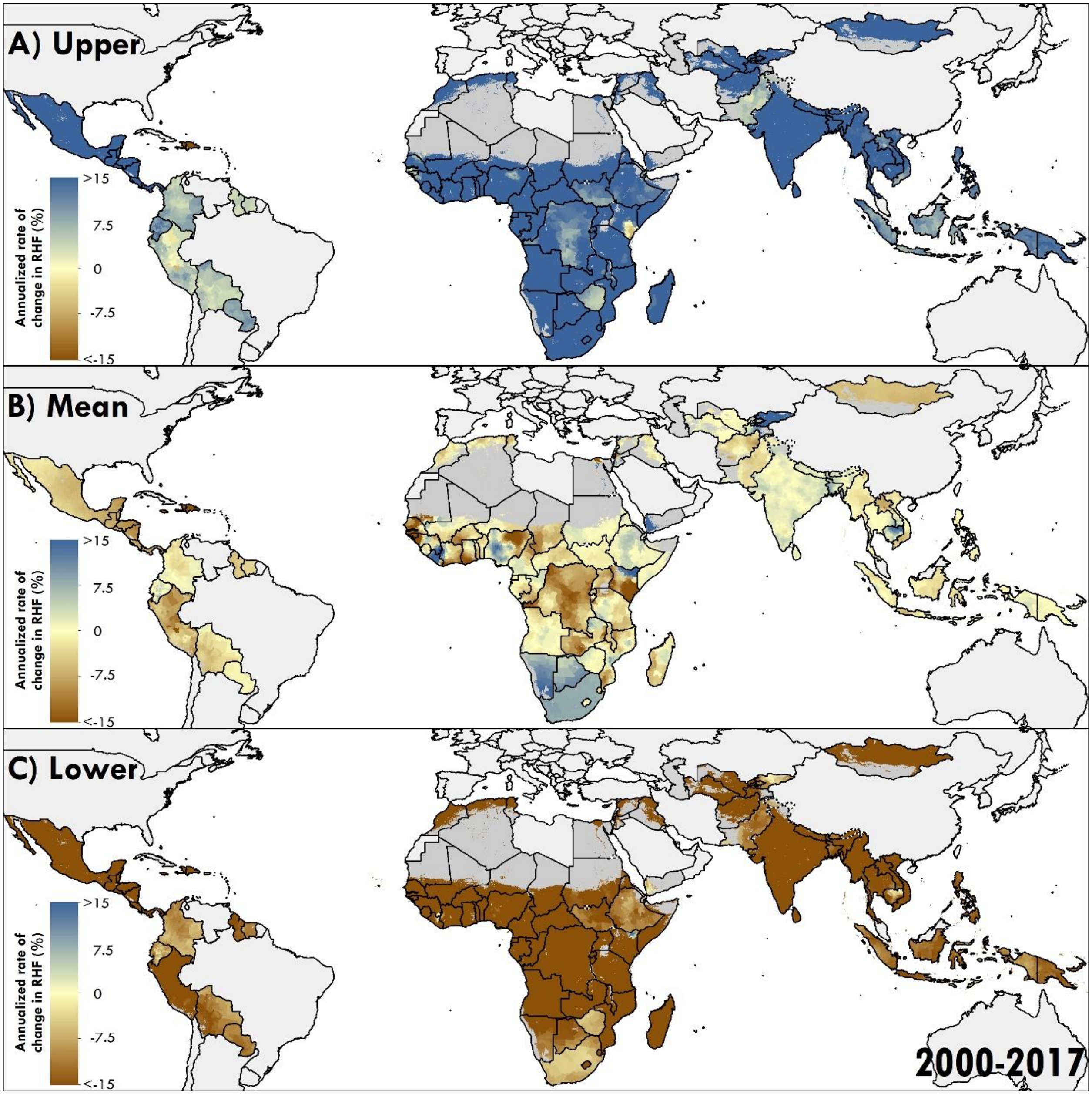

Appendix Figure 54. ORT annualised rate of change, 2000–2017

Maps reflect administrative boundaries, land cover, lakes, and population; dark grey-coloured grid cells were classified as “barren or sparsely vegetated” and had fewer than ten people per 1 × 1-km grid cell; light grey countries were not included in these analyses.[7,21–25]

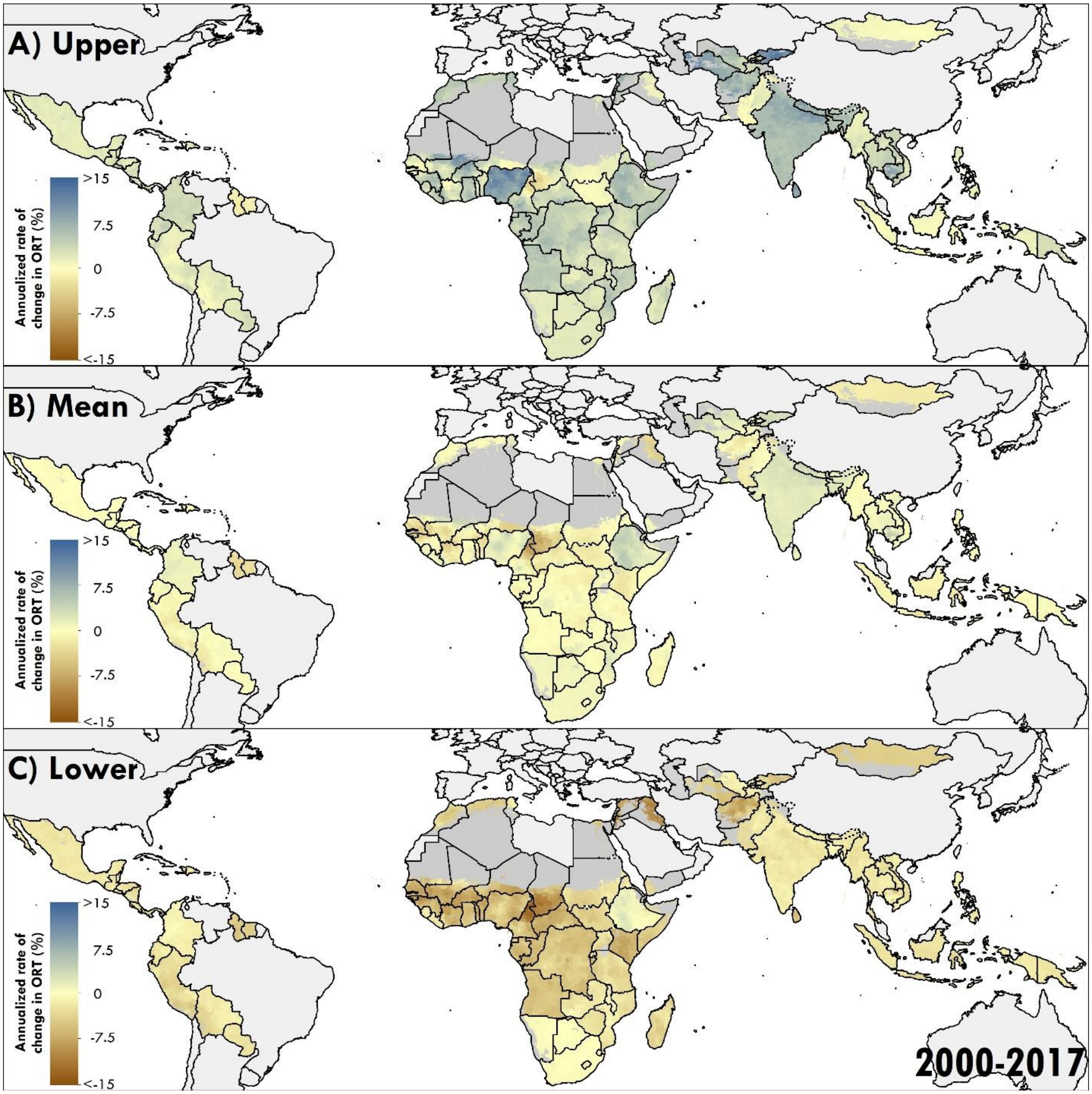

Appendix Figure 55. Relative geographic inequality maps of ORS, RHF, and ORT coverage by second administrative unit, 2017

Maps reflect administrative boundaries, land cover, lakes, and population; dark grey-coloured grid cells were classified as "barren or sparsely vegetated" and had fewer than ten people per 1 × 1-km grid cell; light grey countries were not included in these analyses.[7,21–25]

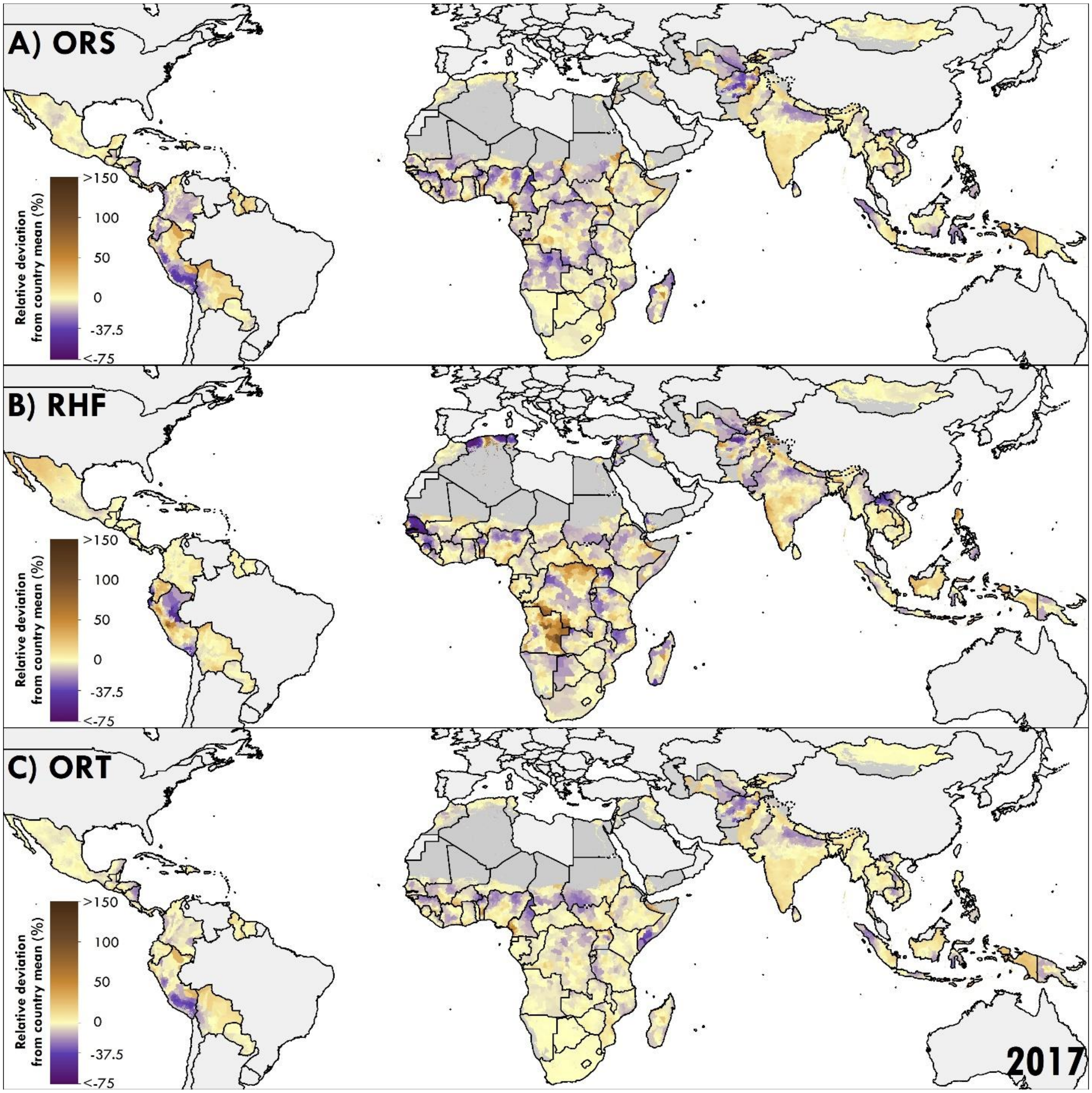

Appendix Figure 56. Geographic inequalities within countries in the proportion of under-5 children with diarrhoea who received recommended home fluids (RHF), 2000 and 2017.

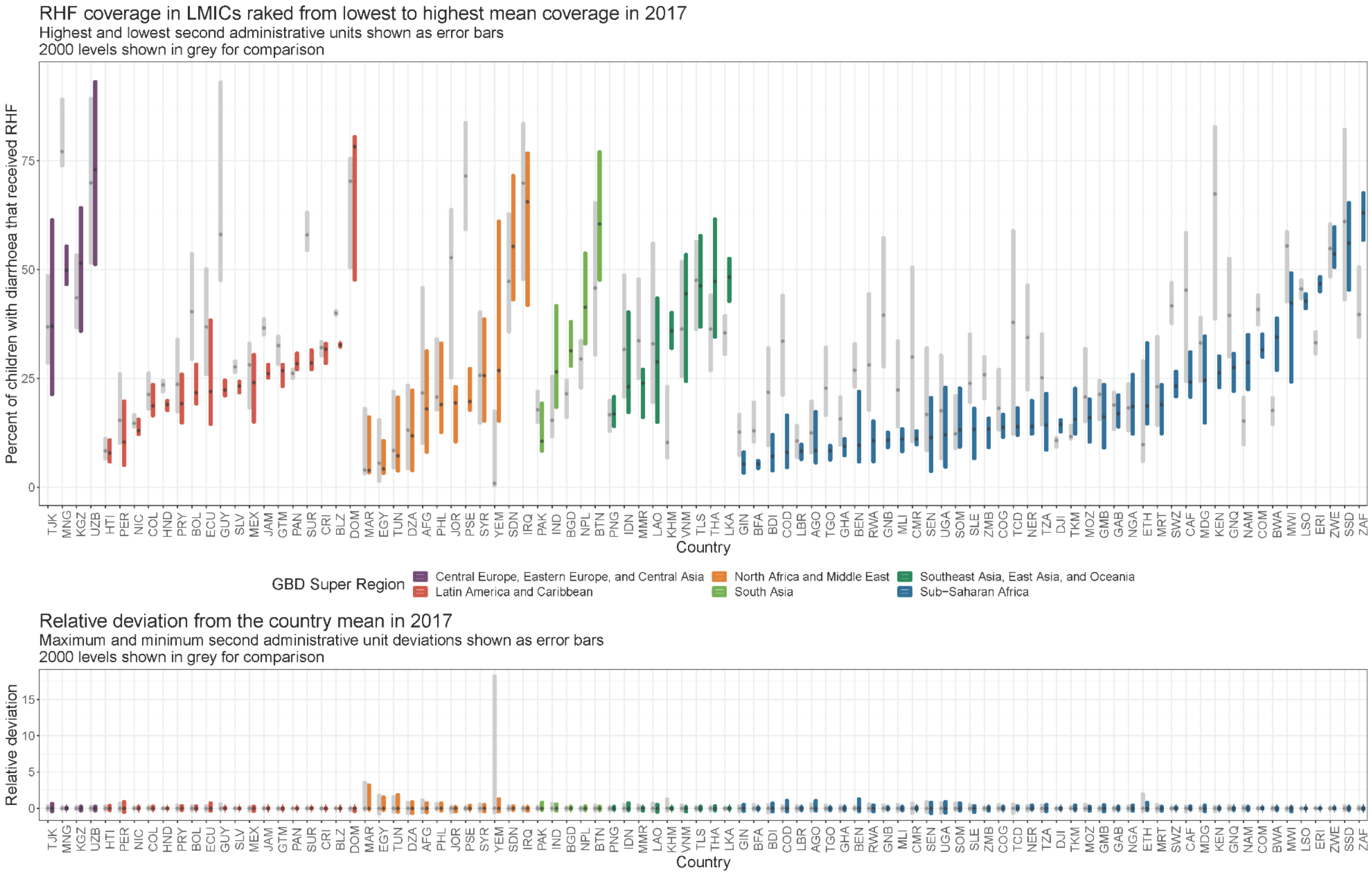

Appendix Figure 57. Geographic inequalities within countries in the proportion of under-5 children with diarrhoea who received oral rehydration therapy (ORT), 2000 and 2017.

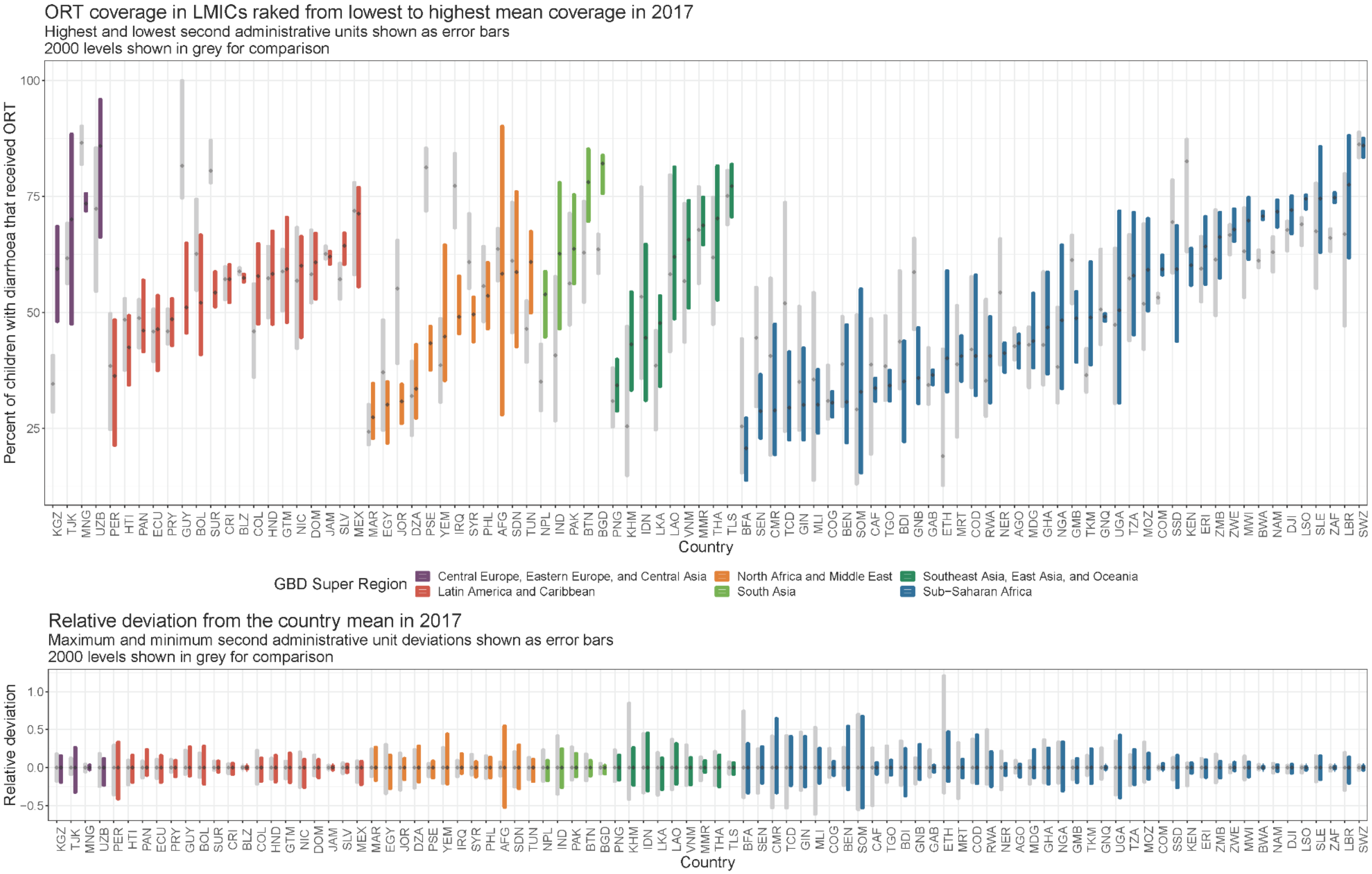

Appendix Figure 58. Correlation between ORS, RHF, and diarrhoeal mortality rates by second administrative unit, 2017

Panel A shows the correlation between ORS and RHF coverage. Panel B shows the correlation between ORS and diarrhoeal mortality rates. Panel C shows the correlation between RHF and diarrhoeal mortality rates. Maps reflect administrative boundaries, land cover, lakes, and population; dark grey-coloured grid cells were classified as "barren or sparsely vegetated" and had fewer than ten people per 1 × 1-km grid cell; light grey countries were not included in these analyses.[7,21–25]

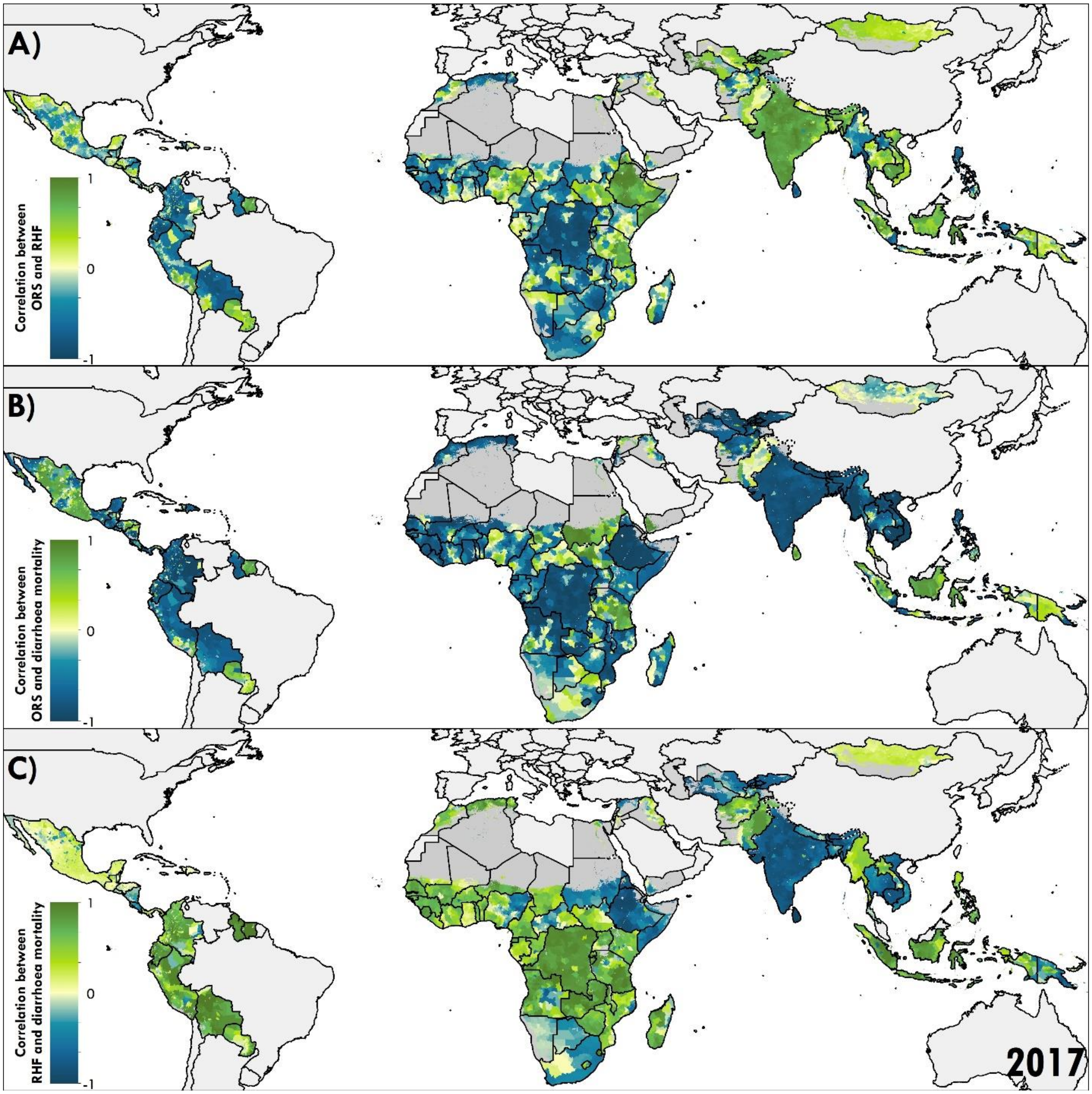

Appendix Figure 59. Sensitivity analysis of deaths averted by ORS scale-up from 2000 to 2017: halving the estimated relative risk of death without ORS treatment (RR = 1.6)

Maps reflect administrative boundaries, land cover, lakes, and population; dark grey-coloured grid cells were classified as "barren or sparsely vegetated" and had fewer than ten people per 1 × 1-km grid cell; light grey countries were not included in these analyses.[7,21–25]

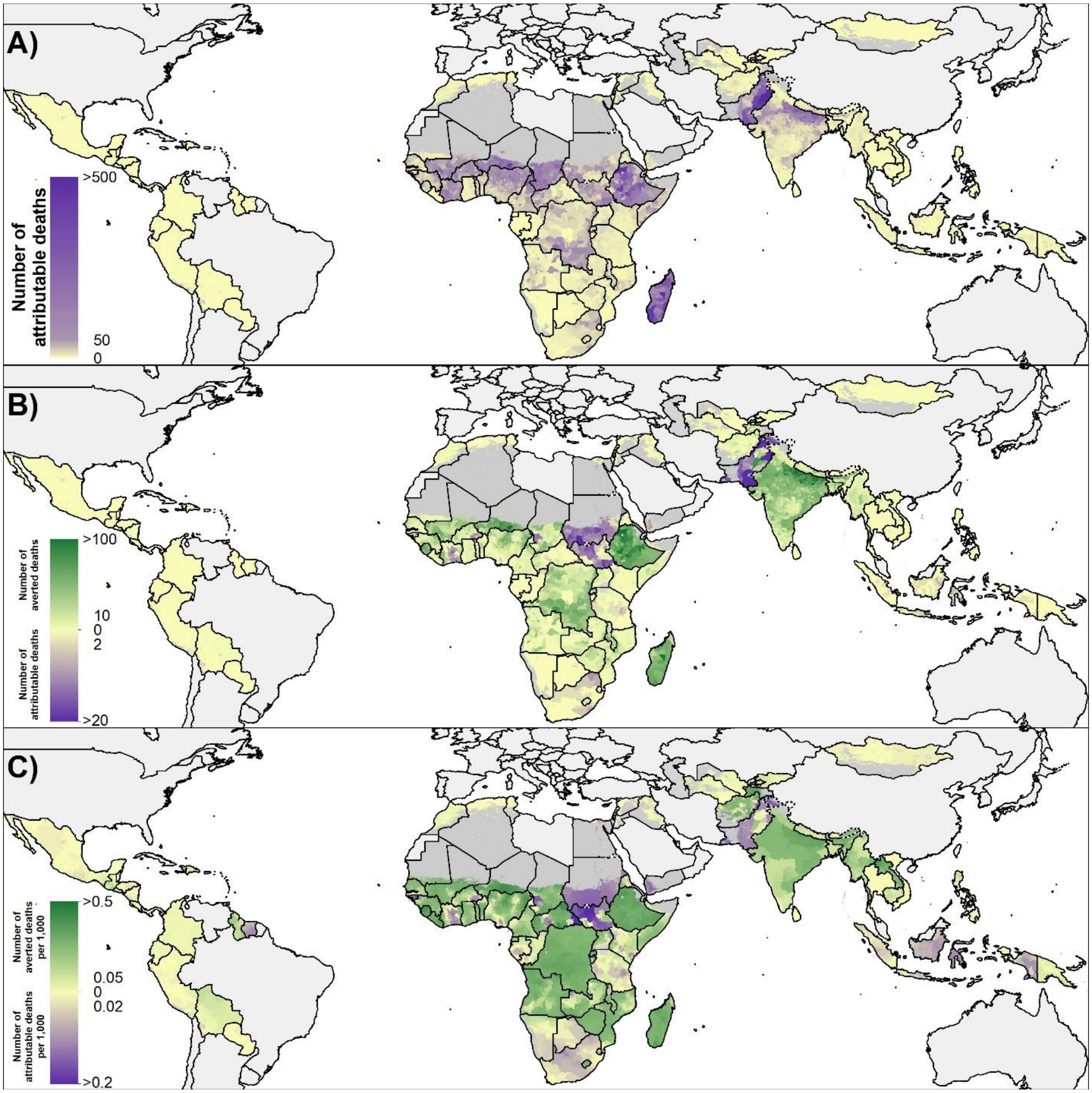

Appendix Figure 60. Sensitivity analysis of deaths averted by ORS scale-up from 2000 to 2017: doubling the estimated relative risk of death without ORS treatment (RR = 6.4)

Maps reflect administrative boundaries, land cover, lakes, and population; dark grey-coloured grid cells were classified as "barren or sparsely vegetated" and had fewer than ten people per 1 × 1-km grid cell; light grey countries were not included in these analyses.[7,21–25]

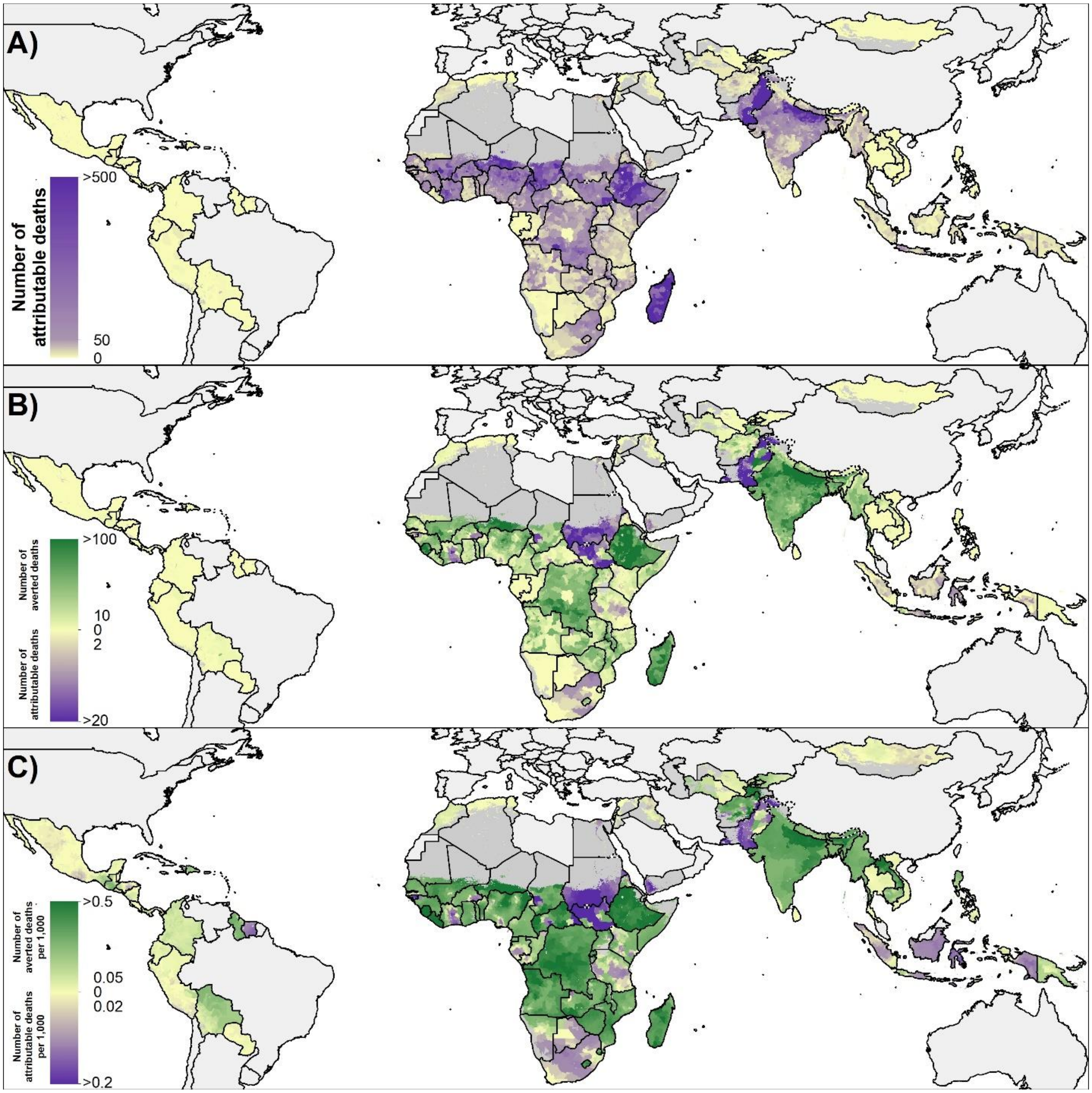

7.0 Appendix tables

Appendix Table 1. Compliance for GATHER (Guidelines for Accurate and transparent Health Estimates Reporting) 85

Appendix Table 2. List of countries included in analysis, stratified by Socio-demographic Index[26] 87

Appendix Table 3. Covariates used in mapping 88

Appendix Table 4a–c. Covariates used in ensemble covariate modelling via stacked generalisation, stratified by modelling region 91

Appendix Table 5a–c. Fitted parameters 99

Appendix Table 6. Recommended home fluid (RHF) definition adjustments 110

Appendix Table 7. Oral rehydration therapy (ORT) definition adjustments 113

Appendix Table 8a-c. Parameters used for boosted regression trees 117

Appendix Table 9a–f. In-sample fit statistics for ORS coverage 120

Appendix Table 10a-f. Out-of-sample fit statistics for ORS coverage 126

Appendix Table 11a–f. In-sample fit statistics for RHF coverage 132

Appendix Table 12a–f. Out-of-sample fit statistics for RHF coverage 138

Appendix Table 13a–f. In-sample fit statistics for ORT coverage 144

Appendix Table 14a–f. Out-of-sample fit statistics for ORT coverage 150

Appendix Table 1. Compliance for GATHER (Guidelines for Accurate and transparent Health Estimates Reporting)

Item #	Checklist item	Reported on page #
**Objectives and funding**		
**1**	Define the indicator(s), populations (including age, sex, and geographic entities), and time period(s) for which estimates were made.	Manuscript: Methods<br>Appendix: Sections 1.0, 2.0
**2**	List the funding sources for the work.	Manuscript: Methods
**Data Inputs**		
For all data inputs from multiple sources that are synthesised as part of the study:		
**3**	Describe how the data were identified and how the data were accessed.	Manuscript: Methods<br>Appendix: Section 2.1
**4**	Specify the inclusion and exclusion criteria. Identify all ad-hoc exclusions.	Manuscript: Methods<br>Appendix: Sections 1.0 and 2.1
**5**	Provide information on all included data sources and their main characteristics. For each data source used, report reference information or contact name/institution, population represented, data collection method, year(s) of data collection, sex and age range, diagnostic criteria or measurement method, and sample size, as relevant.	Manuscript: Methods Appendix: Sections 1.0 and 2.1, http://ghdx.healthdata.org/record/ihme-data/lmic-oral-rehydration-therapy-coverage-geospatial-estimates-2000-2017
**6**	Identify and describe any categories of input data that have potentially important biases (e.g., based on characteristics listed in item 5).	Appendix: Section 2.0
For data inputs that contribute to the analysis but were not synthesised as part of the study:		
**7**	Describe and give sources for any other data inputs.	Manuscript: Methods<br>Appendix: Sections 2.0, 3.0
**8**	Provide all data inputs in a file format from which data can be efficiently extracted (e.g., a spreadsheet rather than a PDF), including all relevant meta-data listed in item 5. For any data inputs that cannot be shared because of ethical or legal reasons, such as third-party ownership, provide a contact name or the name of the institution that retains the right to the data.	Available at http://ghdx.healthdata.org/record/ihme-data/lmic-oral-rehydration-therapy-coverage-geospatial-estimates-2000-2017
**9**	Provide a conceptual overview of the data analysis method. A diagram may be helpful.	Appendix: Section 3.0, Appendix Figures 1, 2
**10**	Provide a detailed description of all steps of the analysis, including mathematical formulae. This description should cover, as relevant, data	Manuscript: Methods<br>Appendix: Sections 2.0, 3.0

	cleaning, data pre-processing, data adjustments and weighting of data sources, and mathematical or statistical model(s).	
**11**	Describe how candidate models were evaluated and how the final model(s) were selected.	Manuscript: Methods<br>Appendix: Sections 3.0, 4.0
**12**	Provide the results of an evaluation of model performance, if done, as well as the results of any relevant sensitivity analysis.	Manuscript: Methods<br>Appendix: Sections 4.0, 5.5
**13**	Describe methods for calculating uncertainty of the estimates. State which sources of uncertainty were, and were not, accounted for in the uncertainty analysis.	Manuscript: Methods<br>Appendix: Sections 3.0
**14**	State how analytic or statistical source code used to generate estimates can be accessed.	Available at http://ghdx.healthdata.org/record/ihme-data/lmic-oral-rehydration-therapy-coverage-geospatial-estimates-2000-2017
**Results and Discussion**		
**15**	Provide published estimates in a file format from which data can be efficiently extracted.	Raster files for spatial data and CSVs of admin 1 and admin 2 estimates to be made available at<br>http://ghdx.healthdata.org/record/ihme-data/lmic-oral-rehydration-therapy-coverage-geospatial-estimates-2000-2017
**16**	Report a quantitative measure of the uncertainty of the estimates (e.g. credible intervals).	Manuscript: Results<br>Appendix: Section 3.0
**17**	Interpret results in light of existing evidence. If updating a previous set of estimates, describe the reasons for changes in estimates.	Manuscript: Discussion
**18**	Discuss limitations of the estimates. Include a discussion of any modelling assumptions or data limitations that affect interpretation of the estimates.	Manuscript: Discussion

Appendix Table 2. List of countries included in analysis, stratified by Socio-demographic Index[26]

Low SDI	Low Middle SDI	Middle SDI
Afghanistan	Angola	Algeria
Bangladesh	Belize	Botswana
Benin	Bhutan	Colombia
Burkina Faso	Bolivia	Costa Rica
Burundi	Cambodia	Ecuador
Central African Republic	Cameroon	Equatorial Guinea
Chad	Djibouti	Gabon
Comoros	Dominican Republic	Indonesia
Côte d'Ivoire	Egypt	Jamaica
Democratic Republic of the Congo	El Salvador	Jordan
Eritrea	Ghana	Mexico
Ethiopia	Guatemala	Mongolia
Guinea	Guyana	Namibia
Guinea-Bissau	Honduras	Panama
Haiti	India	Paraguay
Liberia	Iraq	Peru
Madagascar	Kenya	Philippines
Malawi	Kyrgyzstan	South Africa
Mali	Laos	Sri Lanka
Mozambique	Lesotho	Suriname
Nepal	Mauritania	Syria
Niger	Morocco	Thailand
Papua New Guinea	Myanmar	Tunisia
Rwanda	Nicaragua	Turkmenistan
Senegal	Nigeria	Uzbekistan
Sierra Leone	Pakistan	Vietnam
Somalia	Palestine	
South Sudan	Republic of the Congo	
Tanzania	São Tomé and Príncipe	
The Gambia	Sudan	
Togo	Swaziland (eSwatini)	
Uganda	Tajikistan	
Yemen	Timor-Leste	
	Zambia	
	Zimbabwe	

Appendix Table 3. Covariates used in mapping

A variety of socio-economic and environmental variables were used to predict ORS, RHF, and ORT coverage. Where available, the finest spatio-temporal resolution of gridded datasets was used.

Covariate	Temporal Resolution	Source	Reference
**Access to cities**	Static	Oxford	Weiss, D., Nelson, A., Gibson, H., *et al.* A global map of travel time to cities to assess inequalities in accessibility in 2015. *Nature* **533**, 333–336 (2018).
**Ratio of children dependents (age 0 to 14) to working adults (age 15 to 64)**	Static	WorldPop (derived)	Lloyd, C.T., Sorichetta, A., & Tatem, A.J. High resolution global gridded data for use in population studies. *Sci Data* **4**, 170001 (2017). https://doi.org/10.1038/sdata.2017.1<br><br>WorldPop. Get data. Available at: http://www.worldpop.org.uk/data/get_data/. (Accessed: 25th July 2017)
**Distance from rivers or lakes**	Static	Natural Earth Data (derived)	Natural Earth. Rivers and lake centerlines dataset. Available at: http://www.naturalearthdata.com/downloads/10m-physical-vectors/10m-rivers-lake-centerlines/. (Accessed: 24th July 2017)
**Nighttime lights[TV]**	Annual	NOAA DMSP satellite program (derived)	Savory, D.J., Andrade-Pacheco, R., Gething, P.W., Midekisa, A., Bennett, A., & Sturrock, H.J.W. Intercalibration and Gaussian Process Modeling of Nighttime Lights Imagery for Measuring Urbanization Trends in Africa 2000–2013. *Remote Sens.* **9**, 713 (2017). Available at: http://www.mdpi.com/2072-4292/9/7/713
**Elevantion**	Static	NOAA GLOBE	Hastings, David A. and Paula K. Dunbar. Global Land One-kilometer Base Elevation (GLOBE) Digital Elevation Model, Documentation, Volume 1.0. Key to Geophysical Records Documentation (KGRD) 34. National Oceanic and Atmospheric Administration, National Geophysical Data Center, 325 Broadway, Boulder, Colorado 80303, U.S.A (1999).<br><br>GLOBE Task Team and others (Hastings, David A., Paula K. Dunbar, Gerald M. Elphingstone, Mark Bootz, Hiroshi Murakami, Hiroshi Maruyama, Hiroshi Masaharu, Peter Holland, John Payne, Nevin A. Bryant, Thomas L. Logan, J.-P. Muller, Gunter Schreier, and John S. MacDonald), eds., 1999. The Global Land One-kilometer Base Elevation (GLOBE) Digital Elevation Model, Version 1.0. National Oceanic and Atmospheric Administration, National Geophysical Data Center, 325

Covariate	Temporal Resolution	Source	Reference
			Broadway, Boulder, Colorado 80305-3328, U.S.A. Available at: https://www.ngdc.noaa.gov/mgg/topo/globe.html. (Accessed: 16th February 2017)
**Fertility**	Annual	WorldPop (derived)	Lloyd, C.T., Sorichetta, A., & Tatem, A.J. High resolution global gridded data for use in population studies. *Sci. Data* **4**, 170001 (2017). https://doi.org/10.1038/sdata2017.1<br><br>WorldPop. Get data. Available at: http://www.worldpop.org.uk/data/get_data/. (Accessed: 25th July 2017)
**Population[TV]**	Annual	WorldPop	Lloyd, C.T., Sorichetta, A., & Tatem, A.J. High resolution global gridded data for use in population studies. *Sci. Data* **4**, 170001 (2017). https://doi.org/10.1038/sdata2017.1<br><br>WorldPop. Get data. Available at: http://www.worldpop.org.uk/data/get_data/. (Accessed: 25th July 2017)
**Aridity[TV]**	Annual	WorldClim (derived)	Zomer, R.J., Trabucco, A., Bossio, D.A., & Verchot, L.V. Climate change mitigation: A spatial analysis of global land suitability for clean development mechanism afforestation and reforestation. *Agriculture, Ecosystems & Environment* **126**, 67–80 (2008). https:/doi.org/10.1016/j.agee.2008.01.014<br><br>Global Aridity Index (Global-Aridity) and Global Potential Evapo-Transpiration (Global-PET) Methodology and Geospatial Dataset Description (2009). Available at: http://www.cgiar-csi.org/data/global-aridity-and-pet-database
**Urban or rural[TV]**	Annual	European Commission/GHS	Pesaresi, M., *et al*. Operating procedure for the production of the Global Human Settlement Layer from Landsat data of the epochs 1975, 1990, 2000, and 2014. (Publications Office of the European Union, 2016). Available at: http://ghsl.jrc.ec.europa.eu/data.php
**Urban proportion of the location[TV]**	Annual	MODIS	Friedl, M. & Sulla-Menashe, D. MCD12Q1v006.MODIS/Terra+Aqua Land Cover Type Yearly L3 Global 500m SIN Grid https://doi.org/10.5067/MODIS/MCD12Q1.006 (NASA EOSDIS Land Processes DAAC, 2019)
**Irrigation**	Static	University of Frankfurt and FAO	Siebert, S., Doll, P., Hoogeveen, J., Faures, J.-M., Frenken, K., & Feick, S. Development and validation of the global map of irrigation areas. *Hydrology and Earth System Sciences* **9**, (5) 535–547 (2005).

Covariate	Temporal Resolution	Source	Reference
			Goethe-Universität. Generation of a digital global map of irrigation areas. Available at: https://www.unifrankfurt.de/45218039/Global_Irrigation_Map. (Accessed: 25th July 2017). Also from: http://www.fao.org/nr/water/aquastat/irrigationmap/index10.stm
**Number of people whose daily vitamin A needs could be met (nutrient yield)**	Static	Herrero et al. (modelled)	Herrero, M., *et al*. Farming and the geography of nutrient production for human use: a transdisciplinary analysis. *Lancet Planet. Health* **1**, e33–e42 (2017).
**Prevalence of under-5 stunting** [TV]	Annual	Internally modelled	Kinyoki, D.K., Osgood-Zimmerman, A.E., Pickering, B.V., et al. Mapping child growth failure across low- and middle-income countries. Nature 577, 231–234 (2020). http://doi.org/10.1038/s41586-019-1878-8
**Prevalence of under-5 wasting**[TV]	Annual	Internally modelled	Kinyoki, D.K., Osgood-Zimmerman, A.E., Pickering, B.V., et al. Mapping child growth failure across low- and middle-income countries. Nature 577, 231–234 (2020). http://doi.org/10.1038/s41586-019-1878-8
**Maternal education**	Annual	Internally modelled	Graetz, N., Woyczynski, L., Wilson, K.F. *et al*. Mapping disparities in education across low- and middle-income countries. *Nature* **577**, 235–238 (2020). https://doi.org/10.1038/s41586-019-1872-1

Appendix Table 4a–c. Covariates used in ensemble covariate modelling via stacked generalisation, stratified by modelling region

Table **a)** presents the covariates used in each modelling region for the ORS generalised additive model (GAM), penalised regression with the elastic net penalty, and boosted regression tree (BRT) models. Table **b)** presents the covariates used for RHF, and table **c)** presents covariates used for ORT.

a) Covariates used to model ORS

Region	Access to cities	Ratio of children deps. to working adults	Dist. to rivers or lakes	Night-time lights^{TV}	Elevation	Fertility	Urban-icity^{TV}	Nutrient yield	Irrigation	Land cover	Aridity^{TV}	Pop.^{TV}	Stunting ^{TV}	Wasting ^{TV}	Haqi*	ANC4 Coverage Prop*	Maternal education
**Central sub-Saharan Africa**	**TRUE**	**TRUE**	**TRUE**	**FASLE**	**TRUE**	**TRUE**	**TRUE**	**TRUE**	**TRUE**	**TRUE**	**TRUE**	**TRUE**	**FALSE**	**TRUE**	**FALSE**	**FALSE**	**TRUE**
**Eastern sub-Saharan Africa**	**TRUE**	**TRUE**	**TRUE**	**FALSE**	**TRUE**	**FALSE**	**TRUE**	**TRUE**	**TRUE**	**TRUE**	**TRUE**	**FALSE**	**TRUE**	**TRUE**	**TRUE**	**TRUE**	**TRUE**
**Horn of Africa**	**TRUE**	**FALSE**	**TRUE**	**FALSE**	**TRUE**	**TRUE**	**TRUE**	**TRUE**	**TRUE**	**FALSE**	**TRUE**	**TRUE**	**TRUE**	**TRUE**	**FALSE**	**TRUE**	**TRUE**
**Southern sub-Saharan Africa**	**TRUE**	**FALSE**	**TRUE**	**FALSE**	**TRUE**	**TRUE**	**TRUE**	**TRUE**	**TRUE**	**TRUE**	**TRUE**	**TRUE**	**FALSE**	**FALSE**	**FALSE**	**TRUE**	**TRUE**
**Western sub-Saharan Africa**	**TRUE**	**TRUE**	**TRUE**	**FALSE**	**TRUE**	**FALSE**	**TRUE**	**TRUE**	**TRUE**	**FALSE**	**TRUE**	**TRUE**	**FALSE**	**TRUE**	**TRUE**	**FALSE**	**TRUE**
**Mexico, Central America, and the Caribbean**	**TRUE**	**FALSE**	**TRUE**	**TRUE**	**TRUE**	**TRUE**	**TRUE**	**TRUE**	**TRUE**	**FALSE**	**TRUE**	**TRUE**	**FALSE**	**FALSE**	**FALSE**	**FALSE**	**TRUE**
**Northern South America**	**TRUE**	**FALSE**	**TRUE**	**FALSE**	**TRUE**	**TRUE**	**TRUE**	**TRUE**	**TRUE**	**FALSE**	**TRUE**	**TRUE**	**TRUE**	**FALSE**	**TRUE**	**TRUE**	**TRUE**

Region	Access to cities	Ratio of children deps. to working adults	Dist. to rivers or lakes	Night-time lights^{TV}	Elevation	Fertility	Urban-icity^{TV}	Nutrient yield	Irrigation	Land cover	Aridity^{TV}	Pop.^{TV}	Stunting ^{TV}	Wasting ^{TV}	Haqi*	ANC4 Coverage Prop*	Maternal education
Southern South America	TRUE	FALSE	TRUE	FALSE	TRUE	TRUE	TRUE	TRUE	TRUE	TRUE	TRUE	TRUE	FALSE	TRUE	TRUE	FALSE	TRUE
Central Asia	TRUE	TRUE	TRUE	FALSE	FALSE	FALSE	TRUE	TRUE	TRUE	FALSE	TRUE	TRUE	FALSE	TRUE	TRUE	FALSE	TRUE
Middle East	TRUE	FALSE	TRUE	FALSE	TRUE	TRUE	TRUE	TRUE	TRUE	TRUE	TRUE	TRUE	FALSE	FALSE	FALSE	FLASE	TRUE
South Asia	TRUE	FALSE	TRUE	FALSE	FALSE	TRUE	TRUE	TRUE	TRUE	FALSE	TRUE	TRUE	FALSE	TRUE	TRUE	FALSE	TRUE
Southeast Asia	TRUE	FALSE	TRUE	TRUE	TRUE	TRUE	TRUE	TRUE	TRUE	FALSE	TRUE	TRUE	FALSE	TRUE	FALSE	TRUE	TRUE
Malay Archipelago	TRUE	FALSE	TRUE	FALSE	TRUE	TRUE	TRUE	TRUE	TRUE	FALSE	TRUE	TRUE	FALSE	TRUE	FALSE	TRUE	TRUE
North Africa & Middle East	TRUE	TRUE	TRUE	FALSE	TRUE	TRUE	TRUE	TRUE	FALSE	FALSE	TRUE	TRUE	TRUE	TRUE	FALSE	TRUE	TRUE
Ethiopia	TRUE	FALSE	TRUE	FALSE	TRUE	TRUE	TRUE	TRUE	TRUE	FALSE	TRUE	TRUE	FALSE	FALSE	FALSE	TRUE	TRUE
India	TRUE	FALSE	TRUE	TRUE	TRUE	TRUE	TRUE	TRUE	TRUE	FALSE	TRUE	TRUE	FALSE	TRUE	FALSE	TRUE	TRUE
Kenya	TRUE	FALSE	TRUE	FALSE	TRUE	TRUE	TRUE	TRUE	TRUE	FALSE	TRUE	TRUE	TRUE	FALSE	TRUE	TRUE	TRUE
Mongolia	TRUE	FALSE	TRUE	FALSE	TRUE	TRUE	TRUE	TRUE	TRUE	TRUE	TRUE	FALSE	FALSE	TRUE	FALSE	FALSE	TRUE
Nigeria	TRUE	FALSE	TRUE	FALSE	TRUE	FALSE	TRUE	TRUE	TRUE	FALSE	FALSE	TRUE	FALSE	TRUE	TRUE	FALSE	TRUE

Region	Access to cities	Ratio of children deps. to working adults	Dist. to rivers or lakes	Night-time lights^{TV}	Elevation	Fertility	Urban-icity^{TV}	Nutrient yield	Irrigation	Land cover	Aridity^{TV}	Pop.^{TV}	Stunting ^{TV}	Wasting ^{TV}	Haqi*	ANC4 Coverage Prop*	Maternal education
**Democratic Republic of Congo**	**TRUE**	**FALSE**	**TRUE**	**FALSE**	**TRUE**	**TRUE**	**TRUE**	**TRUE**	**TRUE**	**FALSE**	**TRUE**	**TRUE**	**FALSE**	**TRUE**	**FALSE**	**FALSE**	**TRUE**
**Pakistan**	**TRUE**	**TRUE**	**TRUE**	**FALSE**	**TRUE**	**FALSE**	**TRUE**	**TRUE**	**TRUE**	**FALSE**	**TRUE**	**TRUE**	**FALSE**	**TRUE**	**FALSE**	**TRUE**	**TRUE**
**Yemen**	**TRUE**	**FALSE**	**TRUE**	**FALSE**	**FALSE**	**TRUE**	**TRUE**	**TRUE**	**TRUE**	**TRUE**	**TRUE**	**TRUE**	**FALSE**	**TRUE**	**FALSE**	**TRUE**	**TRUE**
**Zimbabwe**	**TRUE**	**TRUE**	**TRUE**	**FALSE**	**TRUE**	**FALSE**	**FALSE**	**TRUE**	**TRUE**	**TRUE**	**TRUE**	**TRUE**	**FALSE**	**TRUE**	**FALSE**	**TRUE**	**TRUE**

*GBD covariate included in the VIF selection
^{TV} Time-varying

b) Covariates used to model RHF

Region	Access to cities	Ratio of children deps. to working adults	Dist. to rivers or lakes	Night-time lights^{TV}	Elevation	Fertility	Urban-icity^{TV}	Nutrient yield	Irrigation	Land cover	Aridity^{TV}	Pop.^{TV}	Stunting ^{TV}	Wasting ^{TV}	Haqi*	ANC4 Coverage Prop*	Maternal education
**Central sub-Saharan Africa**	**TRUE**	**TRUE**	**TRUE**	**FALSE**	**TRUE**	**TRUE**	**TRUE**	**TRUE**	**TRUE**	**TRUE**	**TRUE**	**TRUE**	**FALSE**	**TRUE**	**FALSE**	**FALSE**	**TRUE**
**Eastern sub-Saharan Africa**	**TRUE**	**TRUE**	**TRUE**	**FALSE**	**TRUE**	**FALSE**	**TRUE**	**TRUE**	**TRUE**	**TRUE**	**TRUE**	**FALSE**	**TRUE**	**TRUE**	**TRUE**	**FALSE**	**TRUE**
**Horn of Africa**	**TRUE**	**FALSE**	**TRUE**	**TRUE**	**TRUE**	**TRUE**	**TRUE**	**TRUE**	**TRUE**	**FALSE**	**TRUE**	**TRUE**	**TRUE**	**TRUE**	**TRUE**	**FALSE**	**TRUE**

Region	Access to cities	Ratio of children deps. to working adults	Dist. to rivers or lakes	Night-time lights[TV]	Elevation	Fertility	Urban-icity[TV]	Nutrient yield	Irrigation	Land cover	Aridity[TV]	Pop.[TV]	Stunting [TV]	Wasting [TV]	Haqi*	ANC4 Coverage Prop*	Maternal education
Southern sub-Saharan Africa	TRUE	FALSE	TRUE	FALSE	TRUE	TRUE	TRUE	TRUE	TRUE	TRUE	TRUE	TRUE	FALSE	FALSE	FALSE	TRUE	TRUE
Western sub-Saharan Africa	TRUE	TRUE	TRUE	FALSE	TRUE	FALSE	TRUE	TRUE	TRUE	FALSE	TRUE	TRUE	FALSE	TRUE	TRUE	FALSE	TRUE
Mexico, Central America, and the Caribbean	TRUE	FALSE	TRUE	FALSE	TRUE	TRUE	TRUE	TRUE	TRUE	TRUE	TRUE	TRUE	FALSE	FALSE	FALSE	FALSE	TRUE
Northern South America	TRUE	FALSE	TRUE	FALSE	TRUE	TRUE	TRUE	TRUE	TRUE	FALSE	TRUE	TRUE	TRUE	FALSE	TRUE	TRUE	TRUE
Southern South America	TRUE	FALSE	TRUE	FALSE	TRUE	TRUE	TRUE	TRUE	TRUE	TRUE	TRUE	TRUE	FALSE	TRUE	TRUE	FALSE	TRUE
Central Asia	TRUE	TRUE	TRUE	FALSE	FALSE	FALSE	TRUE	TRUE	TRUE	FALSE	TRUE	TRUE	TRUE	TRUE	FALSE	FALSE	TRUE
Middle East	TRUE	FALSE	TRUE	FALSE	TRUE	TRUE	TRUE	TRUE	TRUE	TRUE	TRUE	TRUE	FALSE	TRUE	FALSE	FALSE	TRUE
South Asia	TRUE	FALSE	TRUE	FALSE	FALSE	TRUE	TRUE	TRUE	TRUE	FALSE	TRUE	TRUE	FALSE	TRUE	TRUE	FALSE	TRUE
Southeast Asia	TRUE	FALSE	TRUE	TRUE	TRUE	TRUE	TRUE	TRUE	TRUE	FALSE	TRUE	TRUE	FALSE	TRUE	FALSE	TRUE	TRUE
Malay Archipelago	TRUE	FALSE	TRUE	FALSE	TRUE	TRUE	TRUE	TRUE	TRUE	FALSE	TRUE	TRUE	FALSE	TRUE	FALSE	FALSE	TRUE

Region	Access to cities	Ratio of children deps. to working adults	Dist. to rivers or lakes	Night-time lights^{TV}	Elevation	Fertility	Urban-icity^{TV}	Nutrient yield	Irrigation	Land cover	Aridity^{TV}	Pop.^{TV}	Stunting ^{TV}	Wasting ^{TV}	Haqi*	ANC4 Coverage Prop*	Maternal education
**North Africa & Middle East**	**TRUE**	**TRUE**	**TRUE**	**FALSE**	**TRUE**	**TRUE**	**TRUE**	**TRUE**	**TRUE**	**FALSE**	**TRUE**	**TRUE**	**TRUE**	**TRUE**	**TRUE**	**FALSE**	**TRUE**
**Ethiopia**	**TRUE**	**FALSE**	**TRUE**	**FALSE**	**TRUE**	**TRUE**	**TRUE**	**TRUE**	**TRUE**	**FALSE**	**TRUE**	**TRUE**	**FALSE**	**FALSE**	**FALSE**	**TRUE**	**TRUE**
**India**	**TRUE**	**FALSE**	**TRUE**	**TRUE**	**TRUE**	**TRUE**	**TRUE**	**TRUE**	**TRUE**	**FALSE**	**TRUE**	**TRUE**	**FALSE**	**TRUE**	**FALSE**	**TRUE**	**TRUE**
**Kenya**	**TRUE**	**FALSE**	**TRUE**	**FALSE**	**TRUE**	**TRUE**	**TRUE**	**TRUE**	**TRUE**	**FALSE**	**TRUE**	**TRUE**	**TRUE**	**FALSE**	**TRUE**	**TRUE**	**TRUE**
**Mongolia**	**TRUE**	**FALSE**	**TRUE**	**FALSE**	**TRUE**	**TRUE**	**TRUE**	**TRUE**	**TRUE**	**TRUE**	**TRUE**	**FALSE**	**FALSE**	**TRUE**	**FALSE**	**FALSE**	**TRUE**
**Nigeria**	**TRUE**	**FALSE**	**TRUE**	**FALSE**	**TRUE**	**FALSE**	**TRUE**	**TRUE**	**TRUE**	**FALSE**	**FALSE**	**TRUE**	**FALSE**	**FALSE**	**TRUE**	**FALSE**	**TRUE**
**Democratic Republic of Congo**	**TRUE**	**TRUE**	**TRUE**	**FALSE**	**TRUE**	**FALSE**	**TRUE**	**TRUE**	**TRUE**	**FALSE**	**TRUE**	**TRUE**	**FALSE**	**TRUE**	**FALSE**	**FALSE**	**TRUE**
**Pakistan**	**TRUE**	**TRUE**	**TRUE**	**FALSE**	**TRUE**	**FALSE**	**TRUE**	**TRUE**	**TRUE**	**FALSE**	**TRUE**	**TRUE**	**FALSE**	**TRUE**	**FALSE**	**TRUE**	**TRUE**
**Yemen**	**TRUE**	**FALSE**	**TRUE**	**FALSE**	**FALSE**	**TRUE**	**TRUE**	**TRUE**	**TRUE**	**TRUE**	**TRUE**	**TRUE**	**FALSE**	**TRUE**	**FALSE**	**TRUE**	**TRUE**
**Zimbabwe**	**TRUE**	**TRUE**	**TRUE**	**FALSE**	**TRUE**	**FALSE**	**FALSE**	**TRUE**	**TRUE**	**TRUE**	**TRUE**	**TRUE**	**FALSE**	**TRUE**	**FALSE**	**TRUE**	**TRUE**

*GBD covariate included in the VIF selection
^{TV} Time-varying

c) Covariates used to model ORT

Region	Access to cities	Ratio of children deps. to working adults	Dist. to rivers or lakes	Night-time lights^{TV}	Elevation	Fertility	Urban-icity^{TV}	Nutrient yield	Irrigation	Land cover	Aridity^{TV}	Pop.^{TV}	Stunting ^{TV}	Wasting ^{TV}	Haqi*	ANC4 Coverage Prop*	Maternal education
Central sub-Saharan Africa	TRUE	TRUE	TRUE	FALSE	TRUE	TRUE	TRUE	TRUE	TRUE	TRUE	TRUE	TRUE	FALSE	TRUE	FALSE	FALSE	TRUE
Eastern sub-Saharan Africa	TRUE	TRUE	TRUE	FALSE	TRUE	FALSE	TRUE	TRUE	TRUE	TRUE	TRUE	FALSE	TRUE	TRUE	TRUE	TRUE	TRUE
Horn of Africa	TRUE	FALSE	TRUE	FALSE	TRUE	TRUE	TRUE	TRUE	TRUE	FALSE	TRUE	TRUE	TRUE	TRUE	FALSE	FALSE	TRUE
Southern sub-Saharan Africa	TRUE	FALSE	TRUE	FALSE	TRUE	TRUE	TRUE	TRUE	TRUE	TRUE	TRUE	TRUE	FALSE	FALSE	FALSE	TRUE	TRUE
Western sub-Saharan Africa	TRUE	TRUE	TRUE	FALSE	TRUE	FALSE	TRUE	TRUE	TRUE	FALSE	TRUE	TRUE	FALSE	TRUE	TRUE	FALSE	TRUE
Mexico, Central America, and the Caribbean	TRUE	FALSE	TRUE	FALSE	TRUE	TRUE	TRUE	TRUE	TRUE	TRUE	TRUE	TRUE	FALSE	FALSE	FALSE	FALSE	TRUE
Northern South America	TRUE	FALSE	TRUE	FALSE	TRUE	TRUE	TRUE	TRUE	TRUE	FALSE	TRUE	TRUE	TRUE	FALSE	TRUE	TRUE	TRUE
Southern South America	TRUE	FALSE	TRUE	FALSE	TRUE	TRUE	TRUE	TRUE	TRUE	TRUE	TRUE	TRUE	FALSE	TRUE	TRUE	FALSE	TRUE

Region	Access to cities	Ratio of children deps. to working adults	Dist. to rivers or lakes	Night-time lights^{TV}	Elevation	Fertility	Urban-icity^{TV}	Nutrient yield	Irrigation	Land cover	Aridity^{TV}	Pop.^{TV}	Stunting ^{TV}	Wasting ^{TV}	Haqi*	ANC4 Coverage Prop*	Maternal education
Central Asia	TRUE	TRUE	TRUE	FALSE	FALSE	FALSE	TRUE	TRUE	TRUE	FALSE	TRUE	TRUE	FALSE	TRUE	TRUE	FALSE	TRUE
Middle East	TRUE	FLASE	TRUE	FALSE	TRUE	TRUE	TRUE	TRUE	TRUE	TRUE	TRUE	TRUE	FALSE	FALSE	FALSE	FALSE	TRUE
South Asia	TRUE	FALSE	TRUE	FALSE	FALSE	TRUE	TRUE	TRUE	TRUE	FALSE	TRUE	TRUE	FALSE	TRUE	TRUE	FALSE	TRUE
Southeast Asia	TRUE	FALSE	TRUE	TRUE	TRUE	TRUE	TRUE	TRUE	TRUE	FALSE	TRUE	TRUE	FALSE	TRUE	FALSE	TRUE	TRUE
Malay Archipelago	TRUE	FALSE	TRUE	FALSE	TRUE	TRUE	TRUE	TRUE	TRUE	FALSE	TRUE	TRUE	FALSE	TRUE	FALSE	TRUE	TRUE
North Africa & Middle East	TRUE	TRUE	TRUE	FALSE	TRUE	TRUE	TRUE	TRUE	FALSE	FALSE	TRUE	TRUE	TRUE	TRUE	FALSE	TRUE	TRUE
Ethiopia	TRUE	FALSE	TRUE	FALSE	TRUE	TRUE	TRUE	TRUE	TRUE	FALSE	TRUE	TRUE	FALSE	FALSE	FALSE	TRUE	TRUE
India	TRUE	FALSE	TRUE	TRUE	TRUE	TRUE	TRUE	TRUE	TRUE	FALSE	TRUE	TRUE	FALSE	TRUE	FALSE	TRUE	TRUE
Kenya	TRUE	FALSE	TRUE	FALSE	TRUE	TRUE	TRUE	TRUE	TRUE	FALSE	TRUE	TRUE	TRUE	FALSE	TRUE	TRUE	TRUE
Mongolia	TRUE	FALSE	TRUE	FALSE	TRUE	TRUE	TRUE	TRUE	TRUE	TRUE	TRUE	FALSE	FALSE	TRUE	FALSE	FALSE	TRUE
Nigeria	TRUE	FALSE	TRUE	FALSE	TRUE	FALSE	TRUE	TRUE	TRUE	FALSE	FALSE	TRUE	FALSE	FALSE	TRUE	FALSE	TRUE
Democratic Republic of Congo	TRUE	TRUE	TRUE	FALSE	TRUE	FALSE	TRUE	TRUE	TRUE	FALSE	TRUE	TRUE	FALSE	TRUE	FALSE	FALSE	TRUE

Region	Access to cities	Ratio of children deps. to working adults	Dist. to rivers or lakes	Night-time lights^{TV}	Elevation	Fertility	Urban-icity^{TV}	Nutrient yield	Irrigation	Land cover	Aridity^{TV}	Pop.^{TV}	Stunting ^{TV}	Wasting ^{TV}	Haqi*	ANC4 Coverage Prop*	Maternal education
**Pakistan**	**TRUE**	**TRUE**	**TRUE**	**FALSE**	**TRUE**	**FALSE**	**TRUE**	**TRUE**	**TRUE**	**FALSE**	**TRUE**	**TRUE**	**FALSE**	**TRUE**	**TRUE**	**FALSE**	**TRUE**
**Yemen**	**TRUE**	**FALSE**	**TRUE**	**FALSE**	**FALSE**	**TRUE**	**TRUE**	**TRUE**	**TRUE**	**TRUE**	**TRUE**	**TRUE**	**FALSE**	**TRUE**	**FALSE**	**TRUE**	**TRUE**
**Zimbabwe**	**TRUE**	**TRUE**	**TRUE**	**FALSE**	**TRUE**	**FALSE**	**FALSE**	**TRUE**	**TRUE**	**TRUE**	**TRUE**	**TRUE**	**FALSE**	**TRUE**	**FALSE**	**TRUE**	**TRUE**

*GBD covariate included in the VIF selection
^{TV} Time-varying

Appendix Table 5a–c. Fitted parameters

Posterior lower, median, and upper quantiles (0.025%, 0.50%, 0.975%) for the main parameters by region. The first four rows provide information on the fixed effects: the intercept (int) and the covariates (gam, gbm, and enet) correspond to the predicted ensemble rasters. Fitted values for the spatio-temporal field hyperparameters and the precision parameters (inverse variance) for random effects are shown in the columns to the right. Table **a)** presents fitted parameters for ORS, table **b)** presents fitted parameters for RHF, and table **c)** presents fitted parameters for ORT.

a) Fitted Parameters for ORS

	Quantiles	int	gam	gbm	enet	Nominal Range	Nominal Variance	Ar1 ρ	precis	Country Random Effect Precision
**Central sub-Saharan Africa quantiles**	**0.025**	-0.152	0.070	0.140	0.168	3.242	0.392	-0.556	1444.354	1202.774
	**0.500**	0.072	0.314	0.279	0.407	4.814	0.506	0.443	13720.362	12243.837
	**0.975**	0.295	0.558	0.418	0.645	7.507	0.649	0.834	69741.942	64993.108
**Eastern sub-Saharan Africa quantiles**	**0.025**	-0.211	-0.152	0.289	0.532	1.797	0.372	0.738	7.069	12.096
	**0.500**	-0.094	-0.032	0.374	0.658	2.316	0.425	0.838	9.915	28.728
	**0.975**	0.022	0.088	0.460	0.783	3.290	0.501	0.903	13.619	69.658
**Horn of Africa quantiles**	**0.025**	0.184	-0.026	0.555	0.323	3.059	0.218	0.772	110.456	2.965
	**0.500**	0.371	0.000	0.615	0.385	4.925	0.306	0.892	142.221	11.655
	**0.975**	0.557	0.026	0.675	0.447	7.370	0.414	0.945	187.867	45.421
**Southern sub-Saharan Africa quantiles**	**0.025**	-0.282	-0.158	-0.172	0.554	2.801	0.105	-0.490	1494.745	1330.822
	**0.500**	-0.130	0.133	0.035	0.831	8.871	0.210	0.528	14018.591	13509.739
	**0.975**	0.021	0.429	0.242	1.104	32.077	0.373	0.929	72328.543	70544.522
**Western sub-Saharan Africa quantiles**	**0.025**	-0.279	0.034	0.031	0.698	2.528	0.473	0.321	30.242	4.706
	**0.500**	-0.145	0.132	0.071	0.796	3.310	0.554	0.666	54.219	8.101
	**0.975**	-0.011	0.230	0.114	0.893	3.993	0.618	0.792	124.482	13.225
**Mexico, Central America, and**	**0.025**	-0.247	-0.079	-0.028	0.567	1.919	0.302	0.724	8.651	4.287
	**0.500**	-0.049	0.121	0.148	0.732	3.456	0.411	0.888	14.845	11.178

	Quantiles	int	gam	gbm	enet	Nominal Range	Nominal Variance	Ar1 ρ	precis	Country Random Effect Precision
**the Caribbean quantiles**	**0.975**	0.149	0.320	0.323	0.896	6.483	0.559	0.955	24.561	36.490
**Northern South America quantiles**	**0.025**	-0.14024	0.078289	0.218121	0.19496	1.9681	0.1312	0.2475	1477.332	1347.9
	**0.500**	0.022845	0.293961	0.310078	0.395307	4.4043	0.2273	0.8039	14002.69	13554.43
	**0.975**	0.185837	0.509941	0.403561	0.594659	10.8962	0.3555	0.9573	70695.19	68972.11
**Southern South America quantiles**	**0.025**	-0.22602	0.01558	0.306018	0.303108	2.3752	0.1595	0.7187	1607.34	5.2784
	**0.500**	-0.1051	0.171786	0.373393	0.45478	3.8194	0.2106	0.884	14697.01	10.3338
	**0.975**	0.015481	0.327964	0.440824	0.606132	7.3371	0.278	0.9562	70265.85	20.3719
**Central Asia quantiles**	**0.025**	-0.633	-0.421	-0.018	0.852	5.748	0.417	0.359	1286.838	1218.612
	**0.500**	-0.037	-0.116	0.045	1.072	12.594	0.636	0.850	13341.863	12469.408
	**0.975**	0.559	0.101	0.115	1.376	24.900	1.020	0.960	67529.599	65529.552
**Middle East quantiles**	**0.025**	-0.176	0.044	0.297	0.399	1.476	0.681	0.497	13.760	2.489
	**0.500**	0.098	0.118	0.385	0.496	2.014	0.834	0.701	19.739	15.855
	**0.975**	0.372	0.194	0.474	0.593	2.487	0.955	0.803	29.318	155.974
**South Asia quantiles**	**0.025**	-0.327	-0.068	0.211	0.199	0.681	0.260	-0.431	2.510	14.994
	**0.500**	-0.112	0.145	0.421	0.434	1.107	0.423	0.138	5.685	1553.945
	**0.975**	0.104	0.359	0.631	0.668	2.016	0.548	0.673	10.356	21369065.688
**Southeast Asia quantiles**	**0.025**	-0.136	0.038	0.090	0.349	1.496	0.307	0.627	1429.542	5.525
	**0.500**	-0.034	0.244	0.214	0.539	2.061	0.366	0.802	14397.853	14.269
	**0.975**	0.067	0.450	0.347	0.728	3.199	0.451	0.887	71431.252	40.910
**Malay Archipelago quantiles**	**0.025**	-0.340	-0.339	0.318	0.418	3.742	0.166	0.676	1489.502	1434.824
	**0.500**	-0.188	-0.113	0.481	0.632	7.417	0.242	0.909	14353.437	13977.461
	**0.975**	-0.036	0.113	0.644	0.845	14.769	0.337	0.975	74909.322	74767.999

	Quantiles	int	gam	gbm	enet	Nominal Range	Nominal Variance	Ar1 ρ	precis	Country Random Effect Precision
**North Africa & Middle East quantiles**	**0.025**	-0.034	0.101	0.039	0.236	1.193	0.231	-0.245	1347.328	1373.874
	**0.500**	0.119	0.341	0.188	0.471	2.092	0.321	0.463	13354.937	13764.183
	**0.975**	0.272	0.585	0.337	0.702	3.715	0.419	0.812	70968.144	71390.215
**Ethiopia quantiles**	**0.025**	-0.093	0.103	0.141	0.104	1.508	0.103	-0.101	3.536	--
	**0.500**	0.020	0.352	0.337	0.310	5.545	0.180	0.576	6.686	--
	**0.975**	0.133	0.602	0.533	0.515	22.632	0.323	0.875	12.858	--
**India quantiles**	**0.025**	-0.004	0.082	0.078	0.187	1.685	0.371	-0.388	1233.449	--
	**0.500**	0.086	0.260	0.302	0.437	2.162	0.427	-0.015	12501.551	--
	**0.975**	0.176	0.438	0.527	0.687	2.906	0.549	0.266	66595.255	--
**Kenya quantiles**	**0.025**	-0.223	-0.073	0.308	0.072	1.010	0.228	-0.261	0.719	--
	**0.500**	-0.031	0.037	0.604	0.361	2.632	0.348	0.488	0.915	--
	**0.975**	0.161	0.144	0.900	0.649	7.387	0.507	0.868	1.078	--
**Mongolia quantiles**	**0.025**	-0.417	-0.988	0.031	0.803	12.984	0.251	-0.339	1312.575	--
	**0.500**	0.072	-0.444	0.100	1.338	29.080	0.432	0.513	13267.176	--
	**0.975**	0.560	0.097	0.181	1.874	72.650	0.724	0.910	67222.250	--
**Nigeria quantiles**	**0.025**	-0.211	0.063	0.087	0.410	2.550	0.485	0.309	1322.474	--
	**0.500**	-0.006	0.233	0.204	0.563	3.314	0.564	0.537	13665.344	--
	**0.975**	0.198	0.405	0.320	0.715	4.448	0.662	0.679	71031.139	--
**Democratic Republic of Congo quantiles**	**0.025**	-0.266	-0.043	0.235	0.146	2.245	0.374	-0.152	4.126	--
	**0.500**	-0.060	0.206	0.445	0.350	4.939	0.494	0.509	6.725	--
	**0.975**	0.145	0.453	0.656	0.553	9.059	0.643	0.810	12.971	--

	Quantiles	int	gam	gbm	enet	Nominal Range	Nominal Variance	Ar1 ρ	precis	Country Random Effect Precision
**Pakistan quantiles**	**0.025**	-0.141	-0.144	0.563	0.097	1.703	0.221	-0.542	1350.220	--
	**0.500**	-0.020	0.027	0.695	0.277	3.458	0.312	0.304	14501.074	--
	**0.975**	0.101	0.198	0.830	0.457	8.150	0.453	0.790	70251.106	--
**Yemen quantiles**	**0.025**	-0.140	-0.308	0.192	0.227	0.603	0.270	-0.183	3.157	--
	**0.500**	0.041	-0.010	0.489	0.521	1.275	0.401	0.687	5.566	--
	**0.975**	0.221	0.289	0.786	0.813	3.241	0.596	0.942	9.930	--
**Zimbabwe quantiles**	**0.025**	-1.133	-0.240	-0.017	0.468	9.151	0.366	-0.205	1392.057	--
	**0.500**	-0.288	-0.026	0.252	0.766	16.179	0.618	0.603	13668.394	--
	**0.975**	0.555	0.214	0.521	1.055	29.516	0.990	0.897	69191.754	--

b) Fitted Parameters for RHF

	Quantiles	int	gam	gbm	enet	Nominal Range	Nominal Variance	Ar1 ρ	precis	Country Random Effect Precision
**Central sub-Saharan Africa quantiles**	**0.025**	-0.314	-0.193	0.194	0.491	3.944	0.376	-0.685	1255.561	1168.322
	**0.500**	-0.020	-0.010	0.324	0.686	6.987	0.559	0.287	14002.006	14368.159
	**0.975**	0.274	0.173	0.454	0.880	11.347	0.782	0.829	69462.532	70822.273
**Eastern sub-Saharan Africa quantiles**	**0.025**	-0.637	-0.044	0.013	0.831	2.037	0.692	0.090	4.953	0.988
	**0.500**	-0.434	0.039	0.046	0.915	2.531	0.776	0.510	8.331	1.828
	**0.975**	-0.230	0.121	0.080	1.000	3.219	0.876	0.706	13.354	3.185

	Quantiles	int	gam	gbm	enet	Nominal Range	Nominal Variance	Ar1 ρ	precis	Country Random Effect Precision
**Horn of Africa quantiles**	**0.025**	-0.717	0.004	0.716	0.121	8.501	0.401	-0.336	108.128	1249.976
	**0.500**	-0.409	0.050	0.768	0.182	11.667	0.514	0.257	130.478	13315.180
	**0.975**	-0.103	0.095	0.821	0.243	18.044	0.720	0.581	157.797	67617.826
**Southern sub-Saharan Africa quantiles**	**0.025**	-0.455	-0.554	-0.177	0.596	10.476	0.340	-0.467	1311.238	1261.496
	**0.500**	0.126	-0.121	0.109	1.014	23.498	0.562	0.366	13180.350	13021.044
	**0.975**	0.707	0.291	0.396	1.447	55.534	0.925	0.846	67018.581	66757.898
**Western sub-Saharan Africa quantiles**	**0.025**	-0.550	0.019	0.034	0.657	2.865	0.672	-0.479	1280.280	0.769
	**0.500**	-0.419	0.136	0.086	0.777	3.422	0.741	-0.163	14309.942	1.572
	**0.975**	-0.288	0.252	0.142	0.897	4.049	0.824	0.404	74953.852	2.448
**Mexico, Central America, and the Caribbean quantiles**	**0.025**	-0.593	-0.223	-0.038	0.832	10.996	0.769	-0.273	8.424	1233.988
	**0.500**	0.002	-0.066	0.079	0.987	21.031	1.037	0.224	15.361	12445.182
	**0.975**	0.597	0.090	0.196	1.142	33.914	1.361	0.579	30.154	68345.756
**Northern South America quantiles**	**0.025**	-0.104	-0.171	0.119	0.256	1.851	0.070	-0.245	1450.632	0.971
	**0.500**	0.071	0.146	0.287	0.559	6.575	0.174	0.594	14101.728	3.968
	**0.975**	0.244	0.465	0.474	0.857	29.661	0.362	0.924	72000.943	19.616
**Southern South America quantiles**	**0.025**	-0.056	-0.279	0.063	0.787	2.188	0.297	0.653	1756.166	1.120
	**0.500**	0.147	-0.087	0.107	0.979	3.362	0.371	0.823	15303.277	2.112
	**0.975**	0.350	0.105	0.153	1.171	5.230	0.452	0.908	76916.029	3.816
**Central Asia quantiles**	**0.025**	-1.042	-0.291	-0.019	0.768	6.271	0.676	-0.485	1223.786	1249.928
	**0.500**	-0.293	-0.038	0.022	1.015	12.397	1.031	0.432	12799.934	13735.963
	**0.975**	0.456	0.214	0.065	1.263	22.371	1.508	0.842	66395.758	68634.355
	**0.025**	-0.869	0.049	-0.020	0.783	1.975	0.904	-0.357	13.867	1321.237

	Quantiles	int	gam	gbm	enet	Nominal Range	Nominal Variance	Ar1 ρ	precis	Country Random Effect Precision
**Middle East quantiles**	**0.500**	-0.295	0.124	0.011	0.865	3.729	1.415	0.256	41.163	14511.181
	**0.975**	0.278	0.202	0.042	0.944	5.409	1.850	0.766	294.014	71616.310
**South Asia quantiles**	**0.025**	-0.360	0.064	-0.044	0.295	2.860	0.456	-0.034	4.187	1253.200
	**0.500**	0.095	0.342	0.115	0.543	5.901	0.658	0.543	10.604	13258.802
	**0.975**	0.549	0.620	0.275	0.789	13.303	0.970	0.873	31.970	68767.570
**Southeast Asia quantiles**	**0.025**	-0.208	-0.193	0.192	0.586	1.886	0.297	-0.402	1360.514	0.482
	**0.500**	-0.061	-0.058	0.318	0.739	3.562	0.394	0.287	14126.027	1.036
	**0.975**	0.084	0.077	0.446	0.891	6.296	0.514	0.735	69199.496	2.175
**Malay Archipelago quantiles**	**0.025**	-0.249	-0.124	0.273	0.331	2.365	0.231	-0.367	20.353	4.708
	**0.500**	-0.086	0.077	0.398	0.524	4.251	0.303	0.450	42.835	16.033
	**0.975**	0.076	0.282	0.523	0.714	7.559	0.398	0.833	116.696	58.905
**North Africa & Middle East quantiles**	**0.025**	-1.206	-0.154	-0.037	0.832	1.046	1.070	-0.093	1157.704	881.047
	**0.500**	-0.787	-0.032	0.012	1.015	1.571	1.347	0.343	11157.622	9156.474
	**0.975**	-0.376	0.149	0.064	1.141	2.738	1.761	0.630	66916.962	61765.325
**Ethiopia quantiles**	**0.025**	-0.143	-0.258	0.402	0.132	1.569	0.218	-0.149	1313.781	--
	**0.500**	0.019	0.015	0.642	0.339	3.765	0.335	0.589	13320.682	--
	**0.975**	0.180	0.301	0.878	0.546	10.324	0.480	0.881	67096.113	--
**India quantiles**	**0.025**	-0.541	-0.246	0.033	0.617	1.091	0.468	-0.176	2.520	--
	**0.500**	-0.252	-0.035	0.204	0.827	1.764	0.574	0.400	2.960	--
	**0.975**	0.036	0.180	0.382	1.034	2.507	0.659	0.705	3.627	--
**Kenya quantiles**	**0.025**	-1.047	-0.190	-0.365	0.917	2.908	0.920	-0.509	0.573	--
	**0.500**	-0.439	-0.058	-0.105	1.183	5.162	1.330	0.169	0.683	--

	Quantiles	int	gam	gbm	enet	Nominal Range	Nominal Variance	Ar1 ρ	precis	Country Random Effect Precision
	**0.975**	0.167	0.039	0.155	1.451	8.032	1.738	0.695	0.825	--
**Mongolia quantiles**	**0.025**	-1.052	-0.433	-0.031	0.945	40.120	0.078	-0.005	1302.203	--
	**0.500**	-0.015	-0.202	0.032	1.166	106.600	0.238	0.713	13390.430	--
	**0.975**	1.022	0.028	0.104	1.388	356.212	0.601	0.941	67907.604	--
**Nigeria quantiles**	**0.025**	-0.182	-0.178	0.055	0.497	1.771	0.598	-0.142	1395.160	--
	**0.500**	0.004	0.076	0.219	0.705	2.438	0.697	0.251	13377.094	--
	**0.975**	0.190	0.329	0.383	0.914	3.359	0.826	0.584	72016.962	--
**Democratic Republic of Congo quantiles**	**0.025**	-0.747	-0.416	-0.033	0.747	9.400	0.549	0.524	1632.915	--
	**0.500**	-0.014	-0.177	0.178	0.999	15.543	0.787	0.819	14671.059	--
	**0.975**	0.718	0.062	0.387	1.253	26.555	1.116	0.936	75632.840	--
**Pakistan quantiles**	**0.025**	-0.125	-0.277	0.546	-0.278	0.802	0.148	-0.203	1351.844	--
	**0.500**	-0.007	0.104	0.832	0.065	1.568	0.276	0.605	13593.773	--
	**0.975**	0.111	0.485	1.116	0.408	3.339	0.432	0.902	68160.696	--
**Yemen quantiles**	**0.025**	-0.471	-0.006	0.205	0.017	0.694	0.652	-0.454	1023.754	--
	**0.500**	-0.044	0.283	0.428	0.286	1.384	0.893	0.658	14392.332	--
	**0.975**	0.379	0.573	0.661	0.548	2.857	1.246	0.918	72119.185	--
**Zimbabwe quantiles**	**0.025**	-0.241	-0.163	0.033	0.155	3.787	0.138	-0.404	1308.475	--
	**0.500**	0.010	0.213	0.310	0.477	9.601	0.281	0.551	13187.205	--
	**0.975**	0.260	0.590	0.588	0.797	26.935	0.567	0.965	67022.739	--

	Quantiles	int	gam	gbm	enet	Nominal Range	Nominal Variance	Ar1 ρ	precis	Country Random Effect Precision

c) Fitted Parameters for ORT

	Quantiles	int	gam	gbm	enet	Nominal Range	Nominal Variance	Ar1 ρ	precis	Country Random Effect Precision
**Central sub-Saharan Africa quantiles**	**0.025**	-0.098	-0.206	0.095	0.632	2.775	0.333	-0.701	1514.270	1.752
	**0.500**	0.112	-0.042	0.223	0.819	4.451	0.440	0.157	14771.952	7.090
	**0.975**	0.322	0.122	0.351	1.005	6.940	0.583	0.877	76851.041	29.365
**Eastern sub-Saharan Africa quantiles**	**0.025**	-0.151	-0.020	0.046	0.668	1.706	0.453	0.730	7.803	2.017
	**0.500**	-0.010	0.097	0.113	0.788	2.153	0.508	0.824	10.544	3.564
	**0.975**	0.131	0.213	0.186	0.907	2.714	0.597	0.884	15.870	5.804
**Horn of Africa quantiles**	**0.025**	-0.105	-0.077	0.081	0.553	6.049	0.267	0.378	1961.615	4.247
	**0.500**	0.193	0.108	0.151	0.737	11.024	0.416	0.828	15826.407	30.038
	**0.975**	0.491	0.292	0.231	0.920	22.243	0.685	0.950	78213.600	871.464
**Southern sub-Saharan Africa quantiles**	**0.025**	-0.389	-0.135	-0.150	0.367	11.172	0.045	-0.089	1339.751	1370.305
	**0.500**	-0.056	0.246	0.048	0.704	38.880	0.149	0.770	13418.046	13508.765
	**0.975**	0.277	0.628	0.248	1.041	157.653	0.419	0.969	68081.254	68770.004
**Western sub-Saharan Africa quantiles**	**0.025**	-0.276	0.082	0.049	0.585	2.708	0.499	0.443	46.021	3.153
	**0.500**	-0.148	0.207	0.085	0.708	3.459	0.561	0.642	97.105	5.024
	**0.975**	-0.019	0.331	0.122	0.831	4.172	0.623	0.754	318.231	8.380
	**0.025**	-0.111	-0.100	-0.042	0.632	1.878	0.279	0.788	8.627	2.318

	Quantiles	int	gam	gbm	enet	Nominal Range	Nominal Variance	Ar1 ρ	precis	Country Random Effect Precision
**Mexico, Central America, and the Caribbean quantiles**	**0.500**	0.088	0.104	0.085	0.811	3.236	0.378	0.922	13.216	4.380
	**0.975**	0.287	0.307	0.213	0.990	5.700	0.502	0.970	20.341	8.173
**Northern South America quantiles**	**0.025**	-0.078	-0.018	0.264	0.067	2.839	0.159	-0.200	1373.716	1261.219
	**0.500**	0.106	0.243	0.459	0.297	6.087	0.266	0.603	13723.667	13002.781
	**0.975**	0.289	0.504	0.657	0.526	15.551	0.429	0.896	68162.012	66776.191
**Southern South America quantiles**	**0.025**	-0.014	-0.162	0.319	0.402	2.483	0.231	0.461	1704.881	2.898
	**0.500**	0.121	0.017	0.414	0.569	3.509	0.279	0.689	14919.754	5.743
	**0.975**	0.256	0.195	0.509	0.736	5.182	0.334	0.829	71672.296	10.840
**Central Asia quantiles**	**0.025**	-0.548	0.049	-0.016	0.413	6.234	0.509	-0.079	1272.225	1255.533
	**0.500**	-0.034	0.309	0.014	0.676	11.077	0.759	0.654	13455.235	12937.975
	**0.975**	0.480	0.576	0.046	0.932	20.943	1.124	0.913	67811.401	66565.049
**Middle East quantiles**	**0.025**	-0.274	-0.029	-0.017	0.758	2.988	0.851	0.441	8.905	1311.527
	**0.500**	0.128	0.099	0.013	0.887	3.798	1.006	0.712	13.177	13708.623
	**0.975**	0.530	0.226	0.044	1.017	5.231	1.236	0.843	20.337	71057.570
**South Asia quantiles**	**0.025**	-0.386	-0.266	0.287	0.277	0.556	0.469	-0.335	2.953	6.534
	**0.500**	-0.126	-0.045	0.502	0.544	1.095	0.559	0.392	6.534	41.989
	**0.975**	0.133	0.175	0.717	0.810	2.045	0.637	0.732	12.866	482.416
**Southeast Asia quantiles**	**0.025**	-0.015	-0.176	0.368	0.302	1.270	0.297	-0.489	1392.028	3.303
	**0.500**	0.073	-0.003	0.514	0.488	1.974	0.372	0.429	14200.222	9.204
	**0.975**	0.162	0.172	0.660	0.674	2.885	0.449	0.955	69433.551	23.859
	**0.025**	-0.293	-0.183	0.401	0.075	3.318	0.178	0.809	1263.451	1210.816

	Quantiles	int	gam	gbm	enet	Nominal Range	Nominal Variance	Ar1 ρ	precis	Country Random Effect Precision
**Malay Archipelago quantiles**	**0.500**	-0.144	0.063	0.638	0.299	6.271	0.252	0.935	12897.427	13294.162
	**0.975**	0.005	0.308	0.875	0.523	11.981	0.343	0.978	66435.009	67647.577
**North Africa Middle East quantiles**	**0.025**	-0.090	0.001	0.190	0.243	1.563	0.243	-0.241	1228.287	1228.146
	**0.500**	0.074	0.208	0.339	0.452	2.873	0.329	0.351	12379.764	12119.808
	**0.975**	0.239	0.419	0.489	0.658	5.516	0.445	0.738	66586.799	66276.726
**Ethiopia quantiles**	**0.025**	-0.066	0.027	0.222	-0.006	0.926	0.157	0.053	1239.205	--
	**0.500**	0.046	0.304	0.449	0.247	2.341	0.252	0.773	13785.822	--
	**0.975**	0.157	0.582	0.675	0.499	6.530	0.373	0.952	68800.493	--
**India quantiles**	**0.025**	-0.028	-0.056	0.083	0.340	1.082	0.338	-0.589	13.271	--
	**0.500**	0.083	0.126	0.307	0.566	1.683	0.399	-0.056	33.247	--
	**0.975**	0.193	0.311	0.530	0.791	2.426	0.451	0.417	161.099	--
**Kenya quantiles**	**0.025**	-0.345	-0.274	0.075	0.412	4.081	0.619	-0.169	0.566	--
	**0.500**	0.074	-0.035	0.346	0.705	7.106	0.864	0.279	0.733	--
	**0.975**	0.494	0.164	0.619	1.003	15.329	1.172	0.602	0.861	--
**Mongolia quantiles**	**0.025**	-0.616	-0.414	-0.033	0.658	20.270	0.218	0.306	1294.920	--
	**0.500**	0.246	-0.039	0.007	1.031	43.037	0.427	0.803	13487.727	--
	**0.975**	1.108	0.338	0.049	1.402	100.114	0.825	0.959	67855.615	--
**Nigeria quantiles**	**0.025**	-0.016	-0.040	0.026	0.580	1.996	0.574	0.004	17.172	--
	**0.500**	0.179	0.096	0.150	0.743	2.691	0.662	0.297	114.114	--
	**0.975**	0.373	0.252	0.287	0.896	3.466	0.756	0.518	7348.429	--
	**0.025**	-0.126	-0.252	0.493	0.161	1.970	0.381	-0.354	1217.790	--

	Quantiles	int	gam	gbm	enet	Nominal Range	Nominal Variance	Ar1 ρ	precis	Country Random Effect Precision
**Democratic Republic of Congo quantiles**	**0.500**	-0.001	-0.077	0.712	0.375	2.968	0.467	0.369	12508.192	--
	**0.975**	0.123	0.073	0.935	0.589	4.545	0.598	0.683	66406.321	--
**Pakistan quantiles**	**0.025**	-0.081	-0.045	0.786	-0.169	1.825	0.161	-0.339	1627.285	--
	**0.500**	0.026	0.094	0.918	-0.013	3.491	0.248	0.491	14439.591	--
	**0.975**	0.133	0.233	1.052	0.142	6.823	0.372	0.865	69774.332	--
**Yemen quantiles**	**0.025**	-0.065	-0.504	0.051	0.392	0.453	0.305	0.451	3.036	--
	**0.500**	0.118	-0.151	0.417	0.733	0.865	0.422	0.869	4.835	--
	**0.975**	0.302	0.203	0.784	1.072	1.819	0.577	0.971	7.971	--
**Zimbabwe quantiles**	**0.025**	-0.374	-0.543	-0.014	0.402	3.445	0.192	-0.290	1318.668	--
	**0.500**	-0.063	-0.077	0.276	0.802	8.264	0.370	0.550	13514.276	--
	**0.975**	0.249	0.387	0.567	1.202	20.646	0.718	0.936	67864.360	--

Appendix Table 6. Recommended home fluid (RHF) definition adjustments

Estimates of RHF coverage for each survey that was adjusted by methods described in appendix section 1.2. Coverage before adjustment is shown as "unadjusted" and coverage after adjustment is shown as "adjusted".

NID	Country	Source	Start Year	Unadjusted	Adjusted
56830	AFG	UNICEF MICS	2010	0.241	0.243
157018	AFG	MACRO_DHS	2016	0.109	0.110
1994	BDI	UNICEF MICS	2000	0.267	0.206
18902	BGD	MACRO_DHS	2004	0.181	0.183
951	BGD	UNICEF MICS	2006	0.137	0.138
95474	BGD	Urban Health Survey	2006	0.154	0.157
18913	BGD	MACRO DHS	2007	0.181	0.182
55956	BGD	MACRO_DHS	2011	0.097	0.098
151086	BGD	UNICEF MICS	2013	0.349	0.346
157021	BGD	MACRO DHS	2014	0.228	0.232
264910	BLZ	UNICEF MICS	2015	0.121	0.119
1245	BOL	Household Survey	2000	0.335	0.322
1289	BOL	UNICEF MICS	2000	0.887	0.857
32374	BOL	Household Survey	2004	0.273	0.262
148343	BOL	Household Survey	2006	0.276	0.264
148346	BOL	Household Survey	2009	0.261	0.244
40028	BTN	UNICEF MICS	2010	0.603	0.523
1404	BWA	UNICEF MICS	2000	0.226	0.186
2209	CAF	UNICEF MICS	2000	0.487	0.424
2223	CAF	UNICEF MICS	2006	0.249	0.249
26444	CIV	UNICEF MICS	2000	0.462	0.402
218611	CIV	UNICEF MICS	2016	0.254	0.199
2053	CMR	UNICEF MICS	2000	0.665	0.611
244455	CMR	UNICEF MICS	2014	0.093	0.094
3161	COD	UNICEF MICS	2001	0.404	0.343
234733	COG	UNICEF MICS	2014	0.180	0.158
125596	CRI	UNICEF MICS	2011	0.339	0.335
3392	DJI	Arab League PAPFAM	2002	0.016	0.016
27069	DOM	UNICEF MICS	2000	0.780	0.737
3455	DOM	National Multipurpose Household Survey	2006	0.762	0.702
200697	DOM	UNICEF MICS	2014	0.084	0.084
153674	ECU	National Health and Nutrition Survey	2012	0.407	0.392
19511	EGY	MACRO_DHS	2000	0.048	0.048
19529	EGY	MACRO DHS	2003	0.122	0.124
19521	EGY	MACRO DHS	2005	0.020	0.020
26842	EGY	MACRO DHS	2008	0.027	0.028
154897	EGY	MACRO DHS	2014	0.028	0.029
19539	ERI	MACRO DHS	2002	0.262	0.244
19571	ETH	MACRO DHS	2000	0.111	0.113
34085	ETH	Welfare Monitoring Survey	2004	0.090	0.092
19557	ETH	MACRO DHS	2005	0.117	0.115
76706	GAB	MACRO DHS	2012	0.168	0.169
160576	GHA	UNICEF MICS	2007	0.044	0.044
56241	GHA	UNICEF MICS	2010	0.363	0.344

63993	GHA	UNICEF MICS	2011	0.069	0.066
3922	GMB	UNICEF MICS	2000	0.427	0.365
91506	GMB	UNICEF MICS	2010	0.096	0.098
27215	GNB	UNICEF MICS	2010	0.444	0.386
3655	GNQ	UNICEF MICS	2000	0.430	0.368
4779	GTM	CDC Reproductive Health Survey	2009	0.588	0.510
4916	GUY	UNICEF MICS	2000	0.855	0.800
27009	IDN	UNICEF MICS	2000	0.486	0.466
20021	IDN	MACRO DHS	2007	0.282	0.287
19963	IND	MACRO DHS	2006	0.055	0.054
23258	IND	District Level Household Survey (DLHS-3)	2008	0.295	0.206
65181	IND	Coverage Evaluation Survey	2009	0.516	0.439
157050	IND	MACRO DHS	2015	0.280	0.260
7054	IRQ	UNICEF MICS	2000	0.763	0.713
7028	IRQ	UNICEF MICS	2006	0.754	0.686
385708	IRQ	UNICEF MICS	2018	0.025	0.025
7149	JAM	UNICEF MICS	2005	0.313	0.311
20073	JOR	MACRO DHS	2002	0.063	0.068
20083	JOR	MACRO DHS	2007	0.100	0.107
77517	JOR	MACRO DHS	2012	0.097	0.104
356955	JOR	MACRO DHS	2017	0.178	0.180
7387	KEN	UNICEF MICS	2000	0.688	0.666
7375	KEN	Integrated Household Budget Survey	2005	0.478	0.456
56420	KEN	UNICEF MICS	2009	0.206	0.210
398748	KEN	SMART Survey	2011	0.388	0.313
203654	KEN	UNICEF MICS	2013	0.709	0.666
203663	KEN	UNICEF MICS	2013	0.796	0.767
203664	KEN	UNICEF MICS	2013	0.362	0.315
135416	KEN	UNICEF MICS	2011	0.125	0.127
162283	KGZ	UNICEF MICS	2014	0.669	0.634
7618	LAO	UNICEF MICS	2000	0.587	0.561
103973	LAO	UNICEF MICS	2011	0.133	0.133
375362	LAO	UNICEF MICS	2017	0.155	0.152
149906	LCA	UNICEF MICS	2012	0.186	0.190
20167	LSO	MACRO DHS	2004	0.566	0.569
21382	LSO	MACRO DHS	2009	0.562	0.565
27020	MDG	UNICEF MICS	2000	0.579	0.515
8618	MEX	National Survey of Health and Nutrition (ENSANUT)	2006	0.372	0.327
81748	MEX	National Survey of Health and Nutrition (ENSANUT)	2011	0.411	0.366
270627	MLI	UNICEF MICS	2010	0.177	0.172
248224	MLI	UNICEF MICS	2015	0.098	0.101
8932	MMR	UNICEF MICS	2000	0.578	0.515
141910	MMR	Multiple Indicator Cluster Survey	2003	0.688	0.633
8788	MNG	UNICEF MICS	2000	0.805	0.763
189045	MNG	UNICEF MICS	2012	0.306	0.306
150866	MNG	UNICEF MICS	2013	0.576	0.578

335994	MNG	UNICEF MICS	2016	0.588	0.589
336042	MNG	UNICEF MICS	2016	0.392	0.394
20394	MOZ	MACRO DHS	2003	0.172	0.131
27031	MOZ	UNICEF MICS	2008	0.249	0.218
267343	MRT	UNICEF MICS	2015	0.386	0.364
7919	MWI	UNICEF MICS	2006	0.889	0.853
218581	MWI	MACRO DHS	2015	0.269	0.271
20428	NAM	MACRO DHS	2007	0.276	0.278
9439	NER	UNICEF MICS	2000	0.744	0.679
50393	NGA	Reproductive Health, Child Health, and Education Household, School, and Health Facility Baseline Survey	2005	0.208	0.205
50426	NGA	Reproductive Health, Child Health, and Education Household, School, and Health Facility Midline Survey	2007	0.208	0.208
151719	NGA	Living Standards Survey	2009	0.256	0.259
76703	NGA	UNICEF MICS	2011	0.165	0.157
218613	NGA	UNICEF MICS	2016	0.172	0.174
20487	NIC	MACRO DHS	2001	0.131	0.103
286782	NPL	MACRO DHS	2016	0.473	0.445
308316	PAK	UNICEF MICS	2016	0.199	0.193
422512	PAK	UNICEF MICS	2017	0.154	0.147
413666	PER	MACRO DHS	2006	0.164	0.145
413667	PER	MACRO DHS	2006	0.164	0.145
358824	PER	Demographic and Family Health Survey (ENDES)	2017	0.078	0.075
41830	PRY	Permanent Household Survey (EPH)	2005	0.085	0.085
324470	PRY	UNICEF MICS	2016	0.579	0.583
10001	PSE	Multiple Indicator Cluster Survey	2000	0.824	0.777
20596	PSE	Demographic and Health Survey	2004	0.765	0.706
9999	PSE	Arab League PAPFAM	2006	0.073	0.073
26930	RWA	UNICEF MICS	2000	0.759	0.709
11319	RWA	Integrated Household Living Conditions Survey	2001	0.117	0.115
12243	SDN	UNICEF MICS	2000	0.536	0.472
200617	SDN	UNICEF MICS	2014	0.642	0.570
27044	SEN	UNICEF MICS	2000	0.569	0.469
287639	SEN	UNICEF MICS	2015	0.328	0.293
11639	SLE	UNICEF MICS	2000	0.335	0.280
11649	SLE	UNICEF MICS	2005	0.136	0.136
76700	SLE	UNICEF MICS	2010	0.117	0.117
218619	SLE	UNICEF MICS	2017	0.097	0.097
200636	SLV	UNICEF MICS	2014	0.458	0.374
11774	SOM	UNICEF MICS	2006	0.104	0.104
12232	SSD	UNICEF MICS	2000	0.784	0.754
27055	STP	UNICEF MICS	2000	0.377	0.317
214640	STP	UNICEF MICS	2014	0.307	0.220

12289	SUR	UNICEF MICS	2006	0.574	0.491
81203	SUR	UNICEF MICS	2010	0.520	0.433
12320	SWZ	UNICEF MICS	2000	0.594	0.529
20829	SWZ	MACRO DHS	2006	0.217	0.220
30325	SWZ	UNICEF MICS	2010	0.256	0.261
200707	SWZ	UNICEF MICS	2014	0.427	0.433
12379	SYR	Arab League PAPFAM	2001	0.006	0.006
2244	TCD	UNICEF MICS	2000	0.621	0.567
76701	TCD	UNICEF MICS	2010	0.401	0.339
12886	TGO	UNICEF MICS	2000	0.465	0.406
12732	THA	UNICEF MICS	2006	0.225	0.225
12595	TJK	UNICEF MICS	2000	0.447	0.385
13436	UZB	UNICEF MICS	2000	0.705	0.652
13445	UZB	UNICEF MICS	2006	0.718	0.710
13708	VNM	UNICEF MICS	2000	0.601	0.539
21058	VNM	MACRO DHS	2002	0.061	0.046
13719	VNM	UNICEF MICS	2006	0.792	0.777
57999	VNM	UNICEF MICS	2010	0.454	0.453
152735	VNM	UNICEF MICS	2014	0.435	0.434
13795	YEM	Arab League PAPFAM	2003	0.027	0.028
13816	YEM	UNICEF MICS	2006	0.444	0.364
35493	ZWE	UNICEF MICS	2009	0.746	0.729
152720	ZWE	UNICEF MICS	2014	0.572	0.578

Appendix Table 7. Oral rehydration therapy (ORT) definition adjustments

Estimates of ORT (treatment with either ORS or RHF) coverage for each survey that was adjusted by methods described in appendix section 1.2. Coverage before adjustment is shown as "unadjusted" and coverage after adjustment is shown as "adjusted".

NID	Country	Source	Start Year	Unadjusted	Adjusted
56830	AFG	UNICEF MICS	2010	0.604	0.603
157018	AFG	MACRO_DHS	2016	0.562	0.561
1994	BDI	UNICEF MICS	2000	0.408	0.349
18902	BGD	MACRO DHS	2004	0.747	0.745
951	BGD	UNICEF MICS	2006	0.698	0.696
95474	BGD	Urban Health Survey	2006	0.887	0.885
18913	BGD	MACRO DHS	2007	0.796	0.794
55956	BGD	MACRO_DHS	2011	0.808	0.806
151086	BGD	UNICEF MICS	2013	0.803	0.805
157021	BGD	MACRO DHS	2014	0.833	0.829
264910	BLZ	UNICEF MICS	2015	0.567	0.559
1245	BOL	Household Survey	2000	0.537	0.549
1289	BOL	UNICEF MICS	2000	0.902	0.880
32374	BOL	Household Survey	2004	0.411	0.422
148343	BOL	Household Survey	2006	0.411	0.423
148346	BOL	Household Survey	2009	0.433	0.418
40028	BTN	UNICEF MICS	2010	0.818	0.776
1404	BWA	UNICEF MICS	2000	0.626	0.581
2209	CAF	UNICEF MICS	2000	0.563	0.510
26444	CIV	UNICEF MICS	2000	0.550	0.498
218611	CIV	UNICEF MICS	2016	0.349	0.304

2053	CMR	UNICEF MICS	2000	0.701	0.656
244455	CMR	UNICEF MICS	2014	0.243	0.242
3161	COD	UNICEF MICS	2001	0.494	0.439
234733	COG	UNICEF MICS	2014	0.328	0.308
125596	CRI	UNICEF MICS	2011	0.555	0.548
3392	DJI	Arab League PAPFAM	2002	0.665	0.659
27069	DOM	UNICEF MICS	2000	0.850	0.822
3455	DOM	National Multipurpose Household Survey	2006	0.820	0.788
153674	ECU	National Health and Nutrition Survey	2012	0.649	0.640
19511	EGY	MACRO DHS	2000	0.365	0.362
19529	EGY	MACRO DHS	2003	0.372	0.369
19521	EGY	MACRO_DHS	2005	0.369	0.366
26842	EGY	MACRO DHS	2008	0.301	0.299
154897	EGY	MACRO DHS	2014	0.280	0.278
19539	ERI	MACRO DHS	2002	0.555	0.540
19571	ETH	MACRO DHS	2000	0.239	0.236
34085	ETH	Welfare Monitoring Survey	2004	0.165	0.162
19557	ETH	MACRO DHS	2005	0.287	0.281
76706	GAB	MACRO DHS	2012	0.329	0.328
56241	GHA	UNICEF MICS	2010	0.577	0.563
63993	GHA	UNICEF MICS	2011	0.406	0.398
303458	GIN	UNICEF MICS	2016	0.086	0.080
3922	GMB	UNICEF MICS	2000	0.649	0.597
3935	GMB	UNICEF MICS	2006	0.487	0.487
91506	GMB	UNICEF MICS	2010	0.398	0.392
27215	GNB	UNICEF MICS	2010	0.556	0.511
3655	GNQ	UNICEF MICS	2000	0.574	0.520
4779	GTM	CDC Reproductive Health Survey	2009	0.713	0.669
4916	GUY	UNICEF MICS	2000	0.882	0.848
27009	IDN	UNICEF MICS	2000	0.675	0.656
20021	IDN	MACRO DHS	2007	0.526	0.520
19963	IND	MACRO DHS	2006	0.340	0.333
23258	IND	District Level Household Survey (DLHS-3)	2008	0.509	0.416
65181	IND	Coverage Evaluation Survey	2009	0.741	0.693
157050	IND	MACRO DHS	2015	0.587	0.568
7054	IRQ	UNICEF MICS	2000	0.814	0.778
7028	IRQ	UNICEF MICS	2006	0.810	0.766
7149	JAM	UNICEF MICS	2005	0.714	0.711
20073	JOR	MACRO DHS	2002	0.210	0.235
20083	JOR	MACRO DHS	2007	0.250	0.277
77517	JOR	MACRO DHS	2012	0.281	0.310
356955	JOR	MACRO DHS	2017	0.536	0.533
7387	KEN	UNICEF MICS	2000	0.845	0.833
7375	KEN	Integrated Household Budget Survey	2005	0.680	0.664
56420	KEN	UNICEF MICS	2009	0.429	0.422
203654	KEN	UNICEF MICS	2013	0.775	0.752
203663	KEN	UNICEF MICS	2013	0.883	0.865

203664	KEN	UNICEF MICS	2013	0.686	0.648
135416	KEN	UNICEF MICS	2011	0.356	0.352
162283	KGZ	UNICEF MICS	2014	0.739	0.719
7618	LAO	UNICEF MICS	2000	0.698	0.674
103973	LAO	UNICEF MICS	2011	0.474	0.474
375362	LAO	UNICEF MICS	2017	0.629	0.622
149906	LCA	UNICEF MICS	2012	0.186	0.182
20167	LSO	MACRO DHS	2004	0.744	0.742
21382	LSO	MACRO DHS	2009	0.722	0.720
27020	MDG	UNICEF MICS	2000	0.619	0.566
20223	MDG	MACRO DHS	2003	0.428	0.428
8618	MEX	National Survey of Health and Nutrition	2006	0.787	0.763
81748	MEX	National Survey of Health and Nutrition (ENSANUT)	2011	0.739	0.714
270627	MLI	UNICEF MICS	2010	0.258	0.250
248224	MLI	UNICEF MICS	2015	0.249	0.244
8932	MMR	UNICEF MICS	2000	0.723	0.677
141910	MMR	Multiple Indicator Cluster Survey	2003	0.824	0.791
8788	MNG	UNICEF MICS	2000	0.892	0.869
8777	MNG	UNICEF MICS	2005	0.628	0.628
150866	MNG	UNICEF MICS	2013	0.684	0.683
335994	MNG	UNICEF MICS	2016	0.760	0.759
336042	MNG	UNICEF MICS	2016	0.755	0.754
20394	MOZ	MACRO DHS	2003	0.591	0.542
27031	MOZ	UNICEF MICS	2008	0.596	0.566
267343	MRT	UNICEF MICS	2015	0.431	0.416
7919	MWI	UNICEF MICS	2006	0.933	0.918
218581	MWI	MACRO DHS	2015	0.722	0.721
20428	NAM	MACRO DHS	2007	0.696	0.694
9439	NER	UNICEF MICS	2000	0.782	0.744
50393	NGA	Reproductive Health, Child Health, and Education Household, School, and Health Facility Baseline Survey	2005	0.542	0.535
9522	NGA	World Bank Core Welfare Indicator	2006	0.733	0.728
50426	NGA	Reproductive Health, Child Health, and Education Household, School, and Health Facility Midline Survey	2007	0.750	0.748
151719	NGA	Living Standards Survey	2009	0.614	0.612
76703	NGA	UNICEF MICS	2011	0.329	0.339
218613	NGA	UNICEF MICS	2016	0.409	0.408
20487	NIC	MACRO DHS	2001	0.543	0.498
286782	NPL	MACRO DHS	2016	0.622	0.600
387266	PAK	UNICEF MICS	2003	0.450	0.450
308316	PAK	UNICEF MICS	2016	0.571	0.559
422512	PAK	UNICEF MICS	2017	0.380	0.392
413666	PER	MACRO DHS	2006	0.361	0.334

413667	PER	MACRO DHS	2006	0.361	0.334
146860	PER	MACRO DHS	2013	0.382	0.382
358824	PER	Demographic and Family Health Survey	2017	0.365	0.356
324470	PRY	UNICEF MICS	2016	0.703	0.702
10001	PSE	Multiple Indicator Cluster Survey	2000	0.852	0.823
20596	PSE	Demographic and Health Survey	2004	0.838	0.806
20722	RWA	MACRO DHS	2000	0.214	0.214
26930	RWA	UNICEF MICS	2000	0.779	0.738
11319	RWA	Integrated Household Living Conditions Survey	2001	0.192	0.187
12243	SDN	UNICEF MICS	2000	0.672	0.621
200617	SDN	UNICEF MICS	2014	0.684	0.640
27044	SEN	UNICEF MICS	2000	0.600	0.530
287639	SEN	UNICEF MICS	2015	0.489	0.467
11639	SLE	UNICEF MICS	2000	0.681	0.633
200636	SLV	UNICEF MICS	2014	0.814	0.770
12232	SSD	UNICEF MICS	2000	0.877	0.859
27055	STP	UNICEF MICS	2000	0.605	0.551
214640	STP	UNICEF MICS	2014	0.594	0.509
12289	SUR	UNICEF MICS	2006	0.746	0.697
81203	SUR	UNICEF MICS	2010	0.755	0.702
12320	SWZ	UNICEF MICS	2000	0.907	0.887
20829	SWZ	MACRO DHS	2006	0.885	0.884
30325	SWZ	UNICEF MICS	2010	0.742	0.738
200707	SWZ	UNICEF MICS	2014	0.919	0.917
12379	SYR	Arab League PAPFAM	2001	0.545	0.539
2244	TCD	UNICEF MICS	2000	0.663	0.618
19315	TCD	MACRO DHS	2004	0.218	0.218
76701	TCD	UNICEF MICS	2010	0.461	0.417
12886	TGO	UNICEF MICS	2000	0.549	0.497
296646	THA	UNICEF MICS	2016	0.751	0.751
12595	TJK	UNICEF MICS	2000	0.664	0.613
13436	UZB	UNICEF MICS	2000	0.763	0.723
13445	UZB	UNICEF MICS	2006	0.788	0.780
13708	VNM	UNICEF MICS	2000	0.629	0.576
21058	VNM	MACRO DHS	2002	0.429	0.381
13719	VNM	UNICEF MICS	2006	0.832	0.824
57999	VNM	UNICEF MICS	2010	0.672	0.670
152735	VNM	UNICEF MICS	2014	0.684	0.681
13465	VUT	UNICEF MICS	2007	0.518	0.518
13795	YEM	Arab League PAPFAM	2003	0.429	0.425
13816	YEM	UNICEF MICS	2006	0.585	0.522
317239	ZAF	Living Conditions Survey	2015	0.750	0.750
35493	ZWE	UNICEF MICS	2009	0.778	0.767
152720	ZWE	UNICEF MICS	2014	0.731	0.726

Appendix Table 8a-c. Parameters used for boosted regression trees

Table **a)** presents initial fitted parameters for ORS, table **b)** presents initial fitted parameters for RHF, and table **c)** presents initial fitted parameters for ORT.

a) Initial fitted Parameters for ORS BRT

Region	Tree complexity	Min. observations per node	Number of trees	Learning rate	Bagging fraction	CV folds
Central sub-Saharan Africa	4	11	448	0.068	0.5	3
Eastern sub-Saharan Africa	4	8	4113	0.012	0.5	3
Horn of Africa	4	11	3536	0.028	0.5	3
Southern sub-Saharan Africa	4	12	482	0.018	0.5	3
Western sub-Saharan Africa	4	10	4254	0.035	0.5	3
Mexico, Central America, and the Caribbean	4	11	964	0.008	0.5	3
Northern South America	4	12	989	0.076	0.5	3
Southern South America	4	11	1506	0.073	0.5	3
Central Asia	4	7	3255	0.015	0.5	3
Middle East	4	11	879	0.036	0.5	3
South Asia	4	11	348	0.017	0.5	3
Southeast Asia	4	13	571	0.041	0.5	3
Malay Archipelago	4	12	753	0.026	0.5	3
North Africa Middle East	4	13	540	0.031	0.5	3
Ethiopia	4	10	2352	0.003	0.5	3
India	4	12	121	0.044	0.5	3
Kenya	4	13	336	0.010	0.5	3
Mongolia	4	11	1542	0.044	0.5	3
Nigeria	4	9	627	0.037	0.5	3
Democratic Republic of Congo	4	10	699	0.015	0.5	3
Pakistan	4	12	379	0.049	0.5	3

Yemen	3	12	653	0.010	0.5	3
Zimbabwe	4	11	191	0.044	0.5	3

b) Initial fitted Parameters for RHF BRT

Region	Tree complexity	Min. observations per node	Number of trees	Learning rate	Bagging fraction	CV folds
Central sub-Saharan Africa	4	10	1499	0.022	0.5	3
Eastern sub-Saharan Africa	4	13	897	0.043	0.5	3
Horn of Africa	4	14	2938	0.045	0.5	3
Southern sub-Saharan Africa	4	12	323	0.022	0.5	3
Western sub-Saharan Africa	4	12	1971	0.058	0.5	3
Mexico, Central America, and the Caribbean	4	11	604	0.025	0.5	3
Northern South America	4	13	440	0.034	0.5	3
Southern South America	4	10	3964	0.046	0.5	3
Central Asia	4	11	2435	0.025	0.5	3
Middle East	4	12	391	0.030	0.5	3
South Asia	3	11	217	0.066	0.5	3
Southeast Asia	4	13	1258	0.018	0.5	3
Malay Archipelago	4	11	2882	0.011	0.5	3
North Africa Middle East	4	10	203	0.076	0.5	3
Ethiopia	4	11	1168	0.005	0.5	3
India	4	13	1617	0.008	0.5	3
Kenya	4	13	405	0.017	0.5	3
Mongolia	4	10	11608	0.003	0.5	3
Nigeria	4	9	913	0.018	0.5	3
Democratic Republic of Congo	4	11	1675	0.005	0.5	3
Pakistan	4	12	331	0.024	0.5	3
Yemen	4	12	293	0.011	0.5	3

Zimbabwe	4	12	237	0.026	0.5	3

c) Initial fitted Parameters for ORT BRT

Region	Tree complexity	Min. observations per node	Number of trees	Learning rate	Bagging fraction	CV folds
Central sub-Saharan Africa	4	13	619	0.038	0.5	3
Eastern sub-Saharan Africa	4	12	3842	0.011	0.5	3
Horn of Africa	4	9	3133	0.044	0.5	3
Southern sub-Saharan Africa	4	12	405	0.023	0.5	3
Western sub-Saharan Africa	4	12	1605	0.047	0.5	3
Mexico, Central America, and the Caribbean	4	11	773	0.023	0.5	3
Northern South America	4	12	252	0.054	0.5	3
Southern South America	4	13	733	0.083	0.5	3
Central Asia	4	11	2140	0.035	0.5	3
Middle East	4	11	398	0.073	0.5	3
South Asia	4	10	206	0.022	0.5	3
Southeast Asia	4	13	1335	0.015	0.5	3
Malay Archipelago	4	11	4982	0.002	0.5	3
North Africa Middle East	4	13	1269	0.012	0.5	3
Ethiopia	4	10	307	0.018	0.5	3
India	4	13	2568	0.003	0.5	3
Kenya	4	13	5748	0.001	0.5	3
Mongolia	4	11	1097	0.035	0.5	3
Nigeria	4	9	376	0.040	0.5	3
Democratic Republic of Congo	4	11	961	0.007	0.5	3

Pakistan	4	12	497	0.025	0.5	3
Yemen	3	12	337	0.019	0.5	3
Zimbabwe	4	12	308	0.032	0.5	3

Appendix Table 9a–f. In-sample fit statistics for ORS coverage

In-sample fit statistics are shown for country-level **(a,b)**, first-administrative-level **(c,d)**, and second-administrative-level **(e,f)** aggregations. Metrics are shown by year **(a,c,e)** and by modelling region **(b,d,f)**.

a) Predictive in-sample metrics by year aggregated to the country level for ORS coverage

Year	Mean Err.	RMSE	Median SS	Corr.	95% Cov.
**2000**	-0.009	1470.623	720.660	0.966	0.957
**2001**	-0.007	1316.150	1018.743	0.992	0.988
**2002**	-0.005	1168.647	1058.500	0.983	0.988
**2003**	-0.098	94360.051	846.000	0.923	0.839
**2004**	-0.020	1609.596	920.273	0.996	0.892
**2005**	0.002	1159.478	781.000	0.970	0.977
**2006**	-0.045	258928.634	944.500	0.954	0.944
**2007**	-0.002	1311.484	1227.000	0.976	0.967
**2008**	-0.010	1164.565	691.828	0.939	0.945
**2009**	0.020	1781.464	933.476	0.974	0.946
**2010**	0.000	1786.160	1328.774	0.974	0.954
**2011**	-0.003	1487.037	1110.363	0.977	0.967
**2012**	0.021	1102.006	904.042	0.940	0.969
**2013**	0.007	2303.752	967.871	0.985	0.933
**2014**	-0.005	1876.514	867.366	0.992	0.970
**2015**	-0.008	5695.699	1880.925	0.983	0.966
**2016**	0.026	2074.895	692.000	0.963	0.952
**2017**	0.018	1533.792	1239.000	0.969	0.983

b) Predictive in-sample metrics by region aggregated to the country level for ORS coverage

Region	Mean Err.	RMSE	Median SS	Corr.	95% Cov.
**Democratic Republic of the Congo**	-0.004	1968.695	1919.123	0.999	0.992
**Ethiopia**	-0.004	2201.108	1537.000	0.949	0.914
**India**	-0.023	8810.080	2480.539	0.937	0.952
**Kenya**	-0.006	2094.307	860.408	0.950	0.994
**Mongolia**	-0.001	357.741	338.038	0.997	0.993
**Nigeria**	-0.007	3019.836	2554.349	0.696	0.882
**Pakistan**	0.001	2409.329	1790.423	0.997	0.927
**Yemen**	-0.003	2775.498	1160.062	0.998	0.983
**Zimbabwe**	-0.002	894.329	652.000	1.000	0.981
**Horn of Africa**	-0.045	484406.576	1136.059	0.881	0.940
**Central Asia**	-0.003	428.442	210.000	0.997	0.963
**Central sub-Saharan Africa**	0.000	1392.228	757.944	0.999	0.973
**Eastern sub-Saharan Africa**	-0.001	1684.072	1018.743	0.983	0.991
**Malay Archipelago**	-0.003	1208.808	645.500	0.995	0.949
**Mexico, Central America, and the Caribbean**	0.008	1120.158	862.000	0.908	0.969
**The Middle East**	-0.093	88660.487	1539.000	0.802	0.845
**Northern Africa**	-0.001	1198.529	876.500	0.998	0.991
**Northern South America**	-0.002	1051.571	308.405	0.986	0.967
**Southern South America**	0.006	1640.939	1167.385	0.852	0.923
**Southeast Asia**	-0.002	768.145	547.000	0.971	0.972
**South Asia**	-0.008	895.997	602.000	0.979	0.994
**Southern sub-Saharan Africa**	0.001	552.546	410.500	0.993	0.991
**Western sub-Saharan Africa**	0.004	1617.444	1083.085	0.961	0.939

c) Predictive in-sample metrics by year aggregated to the first administrative level for ORS coverage

Year	Mean Err.	RMSE	Median SS	Corr.	95% Cov.
**2000**	-0.009	124.749	31.000	0.929	0.957
**2001**	-0.007	94.166	19.000	0.931	0.988
**2002**	-0.005	99.002	38.000	0.922	0.988
**2003**	-0.098	6180.200	23.000	0.990	0.839
**2004**	-0.020	222.682	45.000	0.982	0.892
**2005**	0.002	91.824	46.689	0.915	0.977
**2006**	-0.045	13554.391	25.851	0.992	0.944
**2007**	-0.002	91.417	50.000	0.928	0.967
**2008**	-0.010	96.257	32.000	0.869	0.945
**2009**	0.020	141.357	36.158	0.910	0.946
**2010**	0.000	160.496	60.630	0.951	0.954
**2011**	-0.003	96.499	31.000	0.914	0.967
**2012**	0.021	90.518	32.272	0.902	0.969
**2013**	0.007	142.621	47.260	0.959	0.933
**2014**	-0.005	152.348	59.752	0.958	0.970
**2015**	-0.008	487.509	95.000	0.948	0.966
**2016**	0.026	113.804	27.000	0.924	0.952
**2017**	0.018	96.041	47.805	0.915	0.983

d) Predictive in-sample metrics by region aggregated to the first administrative level for ORS coverage

Region	Mean Err.	RMSE	Median SS	Corr.	95% Cov.
**Democratic Republic of the Congo**	-0.004	89.312	60.637	0.935	0.992
**Ethiopia**	-0.004	259.951	115.000	0.922	0.914
**India**	-0.023	563.341	73.054	0.897	0.952
**Kenya**	-0.006	83.984	22.500	0.830	0.994
**Mongolia**	-0.001	31.145	15.153	0.777	0.993
**Nigeria**	-0.007	117.333	47.000	0.855	0.882
**Pakistan**	0.001	627.073	318.656	0.994	0.927
**Yemen**	-0.003	145.913	59.986	0.908	0.983
**Zimbabwe**	-0.002	93.166	76.150	0.978	0.981
**Horn of Africa**	-0.045	31423.142	167.978	0.995	0.940
**Central Asia**	-0.003	78.383	13.489	0.909	0.963
**Central sub-Saharan Africa**	0.000	133.795	105.676	0.974	0.973
**Eastern sub-Saharan Africa**	-0.001	192.927	23.000	0.938	0.991
**Malay Archipelago**	-0.003	41.574	9.000	0.835	0.949
**Mexico, Central America, and the Caribbean**	0.008	70.858	39.381	0.827	0.969
**The Middle East**	-0.093	7365.448	131.000	0.982	0.845
**Northern Africa**	-0.001	64.951	29.000	0.858	0.991
**Northern South America**	-0.002	54.937	32.503	0.886	0.967
**Southern South America**	0.006	93.734	54.017	0.841	0.923
**Southeast Asia**	-0.002	32.542	4.503	0.881	0.972
**South Asia**	-0.008	128.953	71.762	0.957	0.994
**Southern sub-Saharan Africa**	0.001	52.888	26.258	0.761	0.991
**Western sub-Saharan Africa**	0.004	177.374	91.769	0.950	0.939

e) Predictive in-sample metrics by year aggregated to the second administrative level for ORS coverage

Year	Mean Err.	RMSE	Median SS	Corr.	95% Cov.
**2000**	-0.009	33.873	4.664	0.854	0.957
**2001**	-0.007	28.786	7.000	0.855	0.988
**2002**	-0.005	23.869	4.876	0.800	0.988
**2003**	-0.098	1968.138	4.000	0.971	0.839
**2004**	-0.020	41.587	8.227	0.962	0.892
**2005**	0.002	16.767	3.938	0.766	0.977
**2006**	-0.045	3388.140	4.786	0.986	0.944
**2007**	-0.002	19.408	5.000	0.834	0.967
**2008**	-0.010	22.180	4.000	0.697	0.945
**2009**	0.020	29.576	3.823	0.765	0.946
**2010**	0.000	38.311	7.000	0.887	0.954
**2011**	-0.003	23.865	5.198	0.797	0.967
**2012**	0.021	20.187	3.400	0.754	0.969
**2013**	0.007	21.383	7.000	0.845	0.933
**2014**	-0.005	30.904	7.838	0.866	0.970
**2015**	-0.008	42.502	12.000	0.864	0.966
**2016**	0.026	37.845	5.049	0.803	0.952
**2017**	0.018	23.215	4.786	0.805	0.983

f) Predictive in-sample metrics by region aggregated to the second administrative level for ORS coverage

Region	Mean Err.	RMSE	Median SS	Corr.	95% Cov.
**Democratic Republic of the Congo**	-0.004	21.747	7.998	0.760	0.992
**Ethiopia**	-0.004	41.328	20.000	0.804	0.914
**India**	-0.023	23.931	5.556	0.766	0.952
**Kenya**	-0.006	18.520	6.000	0.605	0.994
**Mongolia**	-0.001	7.586	0.902	0.562	0.993
**Nigeria**	-0.007	11.323	4.213	0.623	0.882
**Pakistan**	0.001	177.134	60.924	0.967	0.927
**Yemen**	-0.003	18.182	7.058	0.661	0.983
**Zimbabwe**	-0.002	21.154	10.000	0.906	0.981
**Horn of Africa**	-0.045	8469.299	36.997	0.994	0.940
**Central Asia**	-0.003	14.077	3.000	0.755	0.963
**Central sub-Saharan Africa**	0.000	47.199	15.289	0.902	0.973
**Eastern sub-Saharan Africa**	-0.001	39.871	10.000	0.869	0.991
**Malay Archipelago**	-0.003	10.692	3.000	0.648	0.949
**Mexico, Central America, and the Caribbean**	0.008	22.214	4.000	0.622	0.969
**The Middle East**	-0.093	2321.434	19.000	0.968	0.845
**Northern Africa**	-0.001	12.505	3.000	0.571	0.991
**Northern South America**	-0.002	11.995	2.969	0.720	0.967
**Southern South America**	0.006	21.787	4.412	0.710	0.923
**Southeast Asia**	-0.002	8.490	2.000	0.735	0.972
**South Asia**	-0.008	34.133	11.000	0.885	0.994
**Southern sub-Saharan Africa**	0.001	11.334	5.000	0.511	0.991
**Western sub-Saharan Africa**	0.004	51.238	18.000	0.899	0.939

Appendix Table 10a-f. Out-of-sample fit statistics for ORS coverage

Out-of-sample fit statistics are shown for country-level **(a,b)**, first-administrative-level **(c,d)**, and second-administrative-level **(e,f)** aggregations. Metrics are shown by year **(a,c,e)** and by modelling region **(b,d,f)**.

a) Predictive out-of-sample metrics by year aggregated to the country level for ORS coverage.

Year	Mean Err.	RMSE	Median SS	Corr.	95% Cov.
**2000**	-0.011	1470.624	720.660	0.960	0.896
**2001**	-0.011	1316.154	1018.743	0.993	0.975
**2002**	-0.007	1168.649	1058.500	0.974	0.970
**2003**	-0.106	94360.053	846.000	0.915	0.604
**2004**	-0.018	1609.595	920.273	0.994	0.896
**2005**	0.001	1159.478	781.000	0.979	0.953
**2006**	-0.036	258928.632	944.500	0.957	0.893
**2007**	-0.008	1311.489	1227.000	0.981	0.948
**2008**	-0.008	1164.563	691.828	0.935	0.937
**2009**	0.019	1781.465	933.476	0.978	0.929
**2010**	0.002	1786.158	1328.774	0.965	0.898
**2011**	-0.001	1487.036	1110.363	0.973	0.928
**2012**	0.018	1102.008	904.042	0.931	0.934
**2013**	0.013	2303.748	967.871	0.986	0.903
**2014**	-0.002	1876.512	867.366	0.992	0.940
**2015**	-0.006	5695.698	1880.925	0.985	0.924
**2016**	0.001	2074.912	692.000	0.811	0.867
**2017**	0.016	1533.794	1239.000	0.977	0.957

b) Predictive out-of-sample metrics by region aggregated to the country level for ORS coverage.

Region	Mean Err.	RMSE	Median SS	Corr.	95% Cov.
**Democratic Republic of the Congo**	0.004	1968.688	1919.123	0.982	0.947
**Ethiopia**	-0.005	2201.109	1537.000	0.952	0.917
**India**	-0.022	8810.080	2480.539	0.954	0.928
**Kenya**	-0.002	2094.305	860.408	0.993	0.992
**Mongolia**	0.004	357.737	338.038	0.993	0.948
**Nigeria**	-0.006	3019.835	2554.349	0.534	0.852
**Pakistan**	-0.047	2409.370	1790.423	0.409	0.644
**Yemen**	0.003	2775.493	1160.062	0.994	0.974
**Zimbabwe**	-0.002	894.329	652.000	1.000	0.951
**Horn of Africa**	-0.036	484406.573	1136.059	0.864	0.891
**Central Asia**	-0.001	428.440	210.000	0.996	0.955
**Central sub-Saharan Africa**	-0.003	1392.230	757.944	0.993	0.823
**Eastern sub-Saharan Africa**	0.000	1684.070	1018.743	0.977	0.976
**Malay Archipelago**	-0.007	1208.810	645.500	0.992	0.914
**Mexico, Central America, and the Caribbean**	0.006	1120.160	862.000	0.951	0.964
**The Middle East**	-0.101	88660.489	1539.000	0.798	0.611
**Northern Africa**	-0.004	1198.532	876.500	0.993	0.966
**Northern South America**	0.002	1051.568	308.405	0.989	0.951
**Southern South America**	0.007	1640.938	1167.385	0.805	0.884
**Southeast Asia**	-0.002	768.145	547.000	0.974	0.939
**South Asia**	0.004	895.987	602.000	0.970	0.968
**Southern sub-Saharan Africa**	0.000	552.546	410.500	0.989	0.982
**Western sub-Saharan Africa**	0.005	1617.443	1083.085	0.957	0.909

c) Predictive out-of-sample metrics by year aggregated to the first administrative level for ORS coverage.

Year	Mean Err.	RMSE	Median SS	Corr.	95% Cov.
**2000**	-0.011	124.750	31.000	0.872	0.896
**2001**	-0.011	94.168	19.000	0.874	0.975
**2002**	-0.007	99.004	38.000	0.872	0.970
**2003**	-0.106	6180.202	23.000	0.914	0.604
**2004**	-0.018	222.681	45.000	0.962	0.896
**2005**	0.001	91.824	46.689	0.892	0.953
**2006**	-0.036	13554.389	25.851	0.944	0.893
**2007**	-0.008	91.421	50.000	0.904	0.948
**2008**	-0.008	96.256	32.000	0.827	0.937
**2009**	0.019	141.358	36.158	0.878	0.929
**2010**	0.002	160.495	60.630	0.907	0.898
**2011**	-0.001	96.499	31.000	0.853	0.928
**2012**	0.018	90.520	32.272	0.845	0.934
**2013**	0.013	142.617	47.260	0.947	0.903
**2014**	-0.002	152.346	59.752	0.929	0.940
**2015**	-0.006	487.508	95.000	0.930	0.924
**2016**	0.001	113.817	27.000	0.771	0.867
**2017**	0.016	96.042	47.805	0.886	0.957

d) Predictive out-of-sample metrics by region aggregated to the first administrative level for ORS coverage.

Region	Mean Err.	RMSE	Median SS	Corr.	95% Cov.
**Democratic Republic of the Congo**	0.004	89.306	60.637	0.840	0.947
**Ethiopia**	-0.005	259.952	115.000	0.886	0.917
**India**	-0.022	563.341	73.054	0.883	0.928
**Kenya**	-0.002	83.982	22.500	0.764	0.992
**Mongolia**	0.004	31.142	15.153	0.706	0.948
**Nigeria**	-0.006	117.333	47.000	0.802	0.852
**Pakistan**	-0.047	627.104	318.656	0.550	0.644
**Yemen**	0.003	145.908	59.986	0.799	0.974
**Zimbabwe**	-0.002	93.166	76.150	0.962	0.951
**Horn of Africa**	-0.036	31423.139	167.978	0.946	0.891
**Central Asia**	-0.001	78.382	13.489	0.858	0.955
**Central sub-Saharan Africa**	-0.003	133.797	105.676	0.862	0.823
**Eastern sub-Saharan Africa**	0.000	192.926	23.000	0.905	0.976
**Malay Archipelago**	-0.007	41.576	9.000	0.783	0.914
**Mexico, Central America, and the Caribbean**	0.006	70.860	39.381	0.795	0.964
**The Middle East**	-0.101	7365.450	131.000	0.906	0.611
**Northern Africa**	-0.004	64.953	29.000	0.777	0.966
**Northern South America**	0.002	54.934	32.503	0.761	0.951
**Southern South America**	0.007	93.733	54.017	0.745	0.884
**Southeast Asia**	-0.002	32.542	4.503	0.831	0.939
**South Asia**	0.004	128.944	71.762	0.882	0.968
**Southern sub-Saharan Africa**	0.000	52.888	26.258	0.697	0.982
**Western sub-Saharan Africa**	0.005	177.374	91.769	0.913	0.909

e) Predictive out-of-sample metrics by year aggregated to the second administrative level for ORS coverage.

Year	Mean Err.	RMSE	Median SS	Corr.	95% Cov.
**2000**	-0.011	33.874	4.664	0.782	0.896
**2001**	-0.011	28.788	7.000	0.763	0.975
**2002**	-0.007	23.870	4.876	0.701	0.970
**2003**	-0.106	1968.139	4.000	0.834	0.604
**2004**	-0.018	41.586	8.227	0.925	0.896
**2005**	0.001	16.767	3.938	0.695	0.953
**2006**	-0.036	3388.139	4.786	0.923	0.893
**2007**	-0.008	19.411	5.000	0.769	0.948
**2008**	-0.008	22.179	4.000	0.602	0.937
**2009**	0.019	29.576	3.823	0.698	0.929
**2010**	0.002	38.310	7.000	0.812	0.898
**2011**	-0.001	23.865	5.198	0.686	0.928
**2012**	0.018	20.188	3.400	0.659	0.934
**2013**	0.013	21.379	7.000	0.777	0.903
**2014**	-0.002	30.902	7.838	0.816	0.940
**2015**	-0.006	42.501	12.000	0.785	0.924
**2016**	0.001	37.853	5.049	0.609	0.867
**2017**	0.016	23.216	4.786	0.747	0.957

f) Predictive out-of-sample metrics by region aggregated to the second administrative level for ORS coverage.

Region	Mean Err.	RMSE	Median SS	Corr.	95% Cov.
**Democratic Republic of the Congo**	0.004	21.743	7.998	0.473	0.947
**Ethiopia**	-0.005	41.329	20.000	0.730	0.917
**India**	-0.022	23.931	5.556	0.626	0.928
**Kenya**	-0.002	18.518	6.000	0.453	0.992
**Mongolia**	0.004	7.585	0.902	0.403	0.948
**Nigeria**	-0.006	11.322	4.213	0.500	0.852
**Pakistan**	-0.047	177.158	60.924	0.476	0.644
**Yemen**	0.003	18.179	7.058	0.402	0.974
**Zimbabwe**	-0.002	21.154	10.000	0.873	0.951
**Horn of Africa**	-0.036	8469.296	36.997	0.929	0.891
**Central Asia**	-0.001	14.076	3.000	0.670	0.955
**Central sub-Saharan Africa**	-0.003	47.201	15.289	0.735	0.823
**Eastern sub-Saharan Africa**	0.000	39.871	10.000	0.798	0.976
**Malay Archipelago**	-0.007	10.694	3.000	0.583	0.914
**Mexico, Central America, and the Caribbean**	0.006	22.215	4.000	0.551	0.964
**The Middle East**	-0.101	2321.435	19.000	0.830	0.611
**Northern Africa**	-0.004	12.506	3.000	0.426	0.966
**Northern South America**	0.002	11.994	2.969	0.517	0.951
**Southern South America**	0.007	21.787	4.412	0.573	0.884
**Southeast Asia**	-0.002	8.490	2.000	0.652	0.939
**South Asia**	0.004	34.126	11.000	0.718	0.968
**Southern sub-Saharan Africa**	0.000	11.335	5.000	0.397	0.982
**Western sub-Saharan Africa**	0.005	51.238	18.000	0.832	0.909

Appendix Table 11a–f. In-sample fit statistics for RHF coverage

In-sample fit statistics are shown for country-level **(a,b)**, first-administrative-level **(c,d)**, and second-administrative-level **(e,f)** aggregations. Metrics are shown by year **(a,c,e)** and by modelling region **(b,d,f)**.

a) Predictive in-sample metrics by year aggregated to the country level for RHF coverage.

Year	Mean Err.	RMSE	Median SS	Corr.	95% Cov.
**2000**	0.062	1482.905	724.100	0.805	0.776
**2001**	-0.033	1310.228	1003.000	0.913	0.937
**2002**	-0.017	960.613	771.880	0.909	0.982
**2003**	-0.006	961.419	702.000	0.777	0.844
**2004**	-0.021	1514.386	792.615	0.984	0.941
**2005**	-0.006	1115.409	679.496	0.992	0.978
**2006**	-0.074	262067.219	1025.000	0.915	0.534
**2007**	-0.012	1264.424	1168.000	0.943	0.972
**2008**	-0.022	1142.811	689.775	0.870	0.965
**2009**	0.039	1846.747	941.000	0.866	0.950
**2010**	0.003	1683.697	1331.656	0.964	0.948
**2011**	-0.009	1343.189	1173.704	0.959	0.968
**2012**	-0.010	1076.493	887.084	0.923	0.964
**2013**	-0.009	1752.845	967.423	0.971	0.979
**2014**	0.001	1776.169	1032.000	0.993	0.971
**2015**	-0.004	5976.174	1878.534	0.891	0.956
**2016**	0.002	2051.820	687.000	0.926	0.953
**2017**	-0.028	1488.609	1215.000	0.945	0.965

b) Predictive in-sample metrics by region aggregated to the country level for RHF coverage.

Region	Mean Err.	RMSE	Median SS	Corr.	95% Cov.
**Democratic Republic of the Congo**	-0.002	2219.555	942.868	0.997	0.993
**Ethiopia**	0.000	352.013	330.878	1.000	0.975
**India**	0.004	2147.630	2068.000	0.917	0.971
**Kenya**	-0.002	2519.506	1625.743	0.996	0.964
**Mongolia**	0.000	2734.831	1165.156	1.000	0.986
**Nigeria**	-0.008	909.974	665.308	0.998	0.984
**Pakistan**	-0.001	505945.584	1198.224	0.892	0.536
**Yemen**	-0.009	439.912	212.891	0.992	0.926
**Zimbabwe**	0.002	1309.888	585.892	0.999	0.957
**Horn of Africa**	-0.076	1519.276	928.000	0.941	0.899
**Central Asia**	0.000	1219.221	687.000	0.941	0.961
**Central sub-Saharan Africa**	-0.008	1096.429	862.000	0.998	0.957
**Eastern sub-Saharan Africa**	0.014	2302.265	1388.784	1.000	0.945
**Malay Archipelago**	-0.014	1339.721	942.000	0.994	0.965
**Mexico, Central America, and the Caribbean**	-0.004	1109.404	323.591	0.943	0.949
**The Middle East**	-0.001	1639.347	1167.385	0.777	0.915
**Northern Africa**	-0.003	733.086	513.000	0.871	0.909
**Northern South America**	0.006	956.557	540.000	1.000	0.960
**Southern South America**	-0.004	535.459	349.000	0.994	0.950
**Southeast Asia**	0.010	1612.029	1071.876	0.773	0.880
**South Asia**	-0.001	2219.555	942.868	0.997	0.993
**Southern sub-Saharan Africa**	-0.002	352.013	330.878	1.000	0.975
**Western sub-Saharan Africa**	0.014	2147.630	2068.000	0.917	0.971

c) Predictive in-sample metrics by year aggregated to the first administrative level for RHF coverage.

Year	Mean Err.	RMSE	Median SS	Corr.	95% Cov.
**2000**	0.062	118.151	31.000	0.807	0.776
**2001**	-0.033	83.460	18.000	0.884	0.937
**2002**	-0.017	52.905	35.000	0.824	0.982
**2003**	-0.006	66.954	22.000	0.778	0.844
**2004**	-0.021	179.188	44.670	0.971	0.941
**2005**	-0.006	84.415	35.695	0.927	0.978
**2006**	-0.074	13574.501	24.999	0.825	0.534
**2007**	-0.012	88.704	48.105	0.864	0.972
**2008**	-0.022	94.383	31.000	0.742	0.965
**2009**	0.039	143.801	39.000	0.833	0.950
**2010**	0.003	132.284	60.311	0.930	0.948
**2011**	-0.009	86.703	29.866	0.893	0.968
**2012**	-0.010	98.892	39.952	0.900	0.964
**2013**	-0.009	116.687	37.500	0.933	0.979
**2014**	0.001	101.621	62.000	0.959	0.971
**2015**	-0.004	527.095	101.916	0.831	0.956
**2016**	0.002	113.395	27.000	0.908	0.953
**2017**	-0.028	86.758	33.339	0.855	0.965

d) Predictive in-sample metrics by region aggregated to the first administrative level for RHF coverage.

Region	Mean Err.	RMSE	Median SS	Corr.	95% Cov.
**Democratic Republic of the Congo**	-0.002	88.017	60.637	0.948	0.948
**Ethiopia**	0.000	260.083	115.000	0.953	0.975
**India**	0.004	741.329	113.647	0.786	0.971
**Kenya**	-0.002	84.590	23.000	0.911	0.993
**Mongolia**	0.000	30.495	15.000	0.910	0.975
**Nigeria**	-0.008	88.039	33.000	0.880	0.971
**Pakistan**	-0.001	699.314	349.000	0.970	0.964
**Yemen**	-0.009	144.257	58.592	0.987	0.986
**Zimbabwe**	0.002	94.911	77.427	0.866	0.984
**Horn of Africa**	-0.076	32660.520	168.824	0.816	0.536
**Central Asia**	0.000	79.093	15.198	0.931	0.926
**Central sub-Saharan Africa**	-0.008	132.560	95.707	0.970	0.957
**Eastern sub-Saharan Africa**	0.014	150.346	22.000	0.927	0.899
**Malay Archipelago**	-0.014	40.530	9.000	0.820	0.961
**Mexico, Central America, and the Caribbean**	-0.004	69.528	39.000	0.914	0.957
**The Middle East**	-0.001	167.360	97.894	0.990	0.945
**Northern Africa**	-0.003	71.634	38.500	0.932	0.965
**Northern South America**	0.006	56.075	35.629	0.896	0.949
**Southern South America**	-0.004	93.386	54.259	0.777	0.915
**Southeast Asia**	0.010	31.196	4.000	0.838	0.909
**South Asia**	-0.001	118.772	67.277	0.966	0.960
**Southern sub-Saharan Africa**	-0.002	52.539	19.000	0.887	0.950
**Western sub-Saharan Africa**	0.014	179.297	89.000	0.803	0.880

e) Predictive in-sample metrics by year aggregated to the second administrative level for RHF coverage.

Year	Mean Err.	RMSE	Median SS	Corr.	95% Cov.
**2000**	-0.061	1168.990	5.000	0.848	0.601
**2001**	0.062	33.867	4.425	0.780	0.776
**2002**	-0.033	24.628	7.000	0.811	0.937
**2003**	-0.017	17.252	3.000	0.625	0.982
**2004**	-0.006	15.909	3.342	0.684	0.844
**2005**	-0.021	35.086	7.827	0.947	0.941
**2006**	-0.006	16.771	4.000	0.817	0.978
**2007**	-0.074	3351.538	4.727	0.821	0.534
**2008**	-0.012	18.795	4.791	0.702	0.972
**2009**	-0.022	21.905	4.000	0.605	0.965
**2010**	0.039	29.982	3.736	0.722	0.950
**2011**	0.003	35.826	7.000	0.847	0.948
**2012**	-0.009	20.677	5.000	0.730	0.968
**2013**	-0.010	25.287	4.023	0.771	0.964
**2014**	-0.009	22.277	6.000	0.782	0.979
**2015**	0.001	29.270	8.000	0.901	0.971
**2016**	-0.004	45.596	13.000	0.769	0.956
**2017**	0.002	37.726	5.000	0.809	0.953

f) Predictive in-sample metrics by region aggregated to the second administrative level for RHF coverage.

Region	Mean Err.	RMSE	Median SS	Corr.	95% Cov.
**Democratic Republic of the Congo**	-0.002	21.569	7.414	0.800	0.948
**Ethiopia**	0.000	41.250	20.000	0.778	0.975
**India**	0.004	28.588	6.827	0.735	0.971
**Kenya**	-0.002	18.132	6.000	0.732	0.993
**Mongolia**	0.000	7.279	0.898	0.746	0.975
**Nigeria**	-0.008	8.542	3.000	0.621	0.971
**Pakistan**	-0.001	197.902	61.000	0.893	0.964
**Yemen**	-0.009	18.093	7.000	0.937	0.986
**Zimbabwe**	0.002	21.528	10.000	0.670	0.984
**Horn of Africa**	-0.076	8622.075	35.805	0.815	0.536
**Central Asia**	0.000	14.823	3.000	0.772	0.926
**Central sub-Saharan Africa**	-0.008	45.952	14.000	0.934	0.957
**Eastern sub-Saharan Africa**	0.014	33.713	9.000	0.901	0.899
**Malay Archipelago**	-0.014	10.478	3.000	0.662	0.961
**Mexico, Central America, and the Caribbean**	-0.004	22.664	4.000	0.771	0.957
**The Middle East**	-0.001	38.236	13.000	0.970	0.945
**Northern Africa**	-0.003	11.483	4.000	0.685	0.965
**Northern South America**	0.006	12.209	3.000	0.778	0.949
**Southern South America**	-0.004	21.707	4.357	0.677	0.915
**Southeast Asia**	0.010	8.485	1.813	0.758	0.909
**South Asia**	-0.001	26.078	9.000	0.870	0.960
**Southern sub-Saharan Africa**	-0.002	11.930	5.000	0.733	0.950
**Western sub-Saharan Africa**	0.014	51.605	17.827	0.777	0.880

Appendix Table 12a–f. Out-of-sample fit statistics for RHF coverage

Out-of-sample fit statistics are shown for country-level **(a,b)**, first-administrative-level **(c,d)**, and second-administrative-level **(e,f)** aggregations. Metrics are shown by year **(a,c,e)** and by modelling region **(b,d,f)**.

a) Predictive out-of-sample metrics by year aggregated to the country level for RHF coverage.

Year	Mean Err.	RMSE	Median SS	Corr.	95% Cov.
**2000**	0.068	1482.901	724.100	0.811	0.761
**2001**	-0.029	1310.224	1003.000	0.928	0.925
**2002**	-0.041	960.633	771.880	0.847	0.914
**2003**	-0.006	961.419	702.000	0.780	0.866
**2004**	-0.015	1514.382	792.615	0.991	0.923
**2005**	-0.006	1115.409	679.496	0.988	0.965
**2006**	-0.052	262067.215	1025.000	0.928	0.703
**2007**	-0.016	1264.427	1168.000	0.946	0.956
**2008**	-0.017	1142.807	689.775	0.861	0.958
**2009**	0.048	1846.740	941.000	0.842	0.866
**2010**	0.001	1683.698	1331.656	0.935	0.873
**2011**	-0.008	1343.188	1173.704	0.951	0.943
**2012**	-0.010	1076.493	887.084	0.921	0.946
**2013**	-0.010	1753.005	967.423	0.976	0.960
**2014**	0.003	1776.168	1032.000	0.988	0.940
**2015**	-0.003	5976.173	1878.534	0.878	0.922
**2016**	0.008	2051.816	687.000	0.920	0.915
**2017**	-0.027	1488.608	1215.000	0.951	0.950

b) Predictive out-of-sample metrics by region aggregated to the country level for RHF coverage.

Region	Mean Err.	RMSE	Median SS	Corr.	95% Cov.
**Democratic Republic of the Congo**	-0.003	1941.536	1911.687	1.000	0.944
**Ethiopia**	-0.001	2200.143	1513.000	0.977	0.946
**India**	0.009	11388.811	3188.615	0.779	0.937
**Kenya**	0.000	2219.554	942.868	0.997	0.984
**Mongolia**	0.002	352.012	330.878	1.000	0.977
**Nigeria**	-0.005	2147.627	2068.000	0.960	0.951
**Pakistan**	0.007	2519.498	1625.743	0.906	0.846
**Yemen**	-0.003	2735.369	1165.156	1.000	0.945
**Zimbabwe**	0.001	909.975	665.308	0.998	0.953
**Horn of Africa**	-0.053	505945.577	1198.224	0.870	0.696
**Central Asia**	0.004	439.908	212.891	0.990	0.909
**Central sub-Saharan Africa**	0.007	1309.877	585.892	0.997	0.893
**Eastern sub-Saharan Africa**	0.015	1519.276	928.000	0.945	0.907
**Malay Archipelago**	-0.015	1219.222	687.000	0.909	0.901
**Mexico, Central America, and the Caribbean**	-0.006	1096.431	862.000	0.964	0.917
**The Middle East**	0.001	2302.263	1388.784	0.999	0.882
**Northern Africa**	-0.001	1339.719	942.000	0.995	0.951
**Northern South America**	0.008	1109.403	323.591	0.883	0.934
**Southern South America**	-0.005	1639.348	1167.385	0.814	0.911
**Southeast Asia**	0.011	733.085	513.000	0.839	0.881
**South Asia**	-0.003	956.558	540.000	0.998	0.948
**Southern sub-Saharan Africa**	-0.002	535.459	349.000	0.997	0.893
**Western sub-Saharan Africa**	0.016	1612.027	1071.876	0.807	0.855

c) Predictive out-of-sample metrics by year aggregated to the first administrative level for RHF coverage.

Year	Mean Err.	RMSE	Median SS	Corr.	95% Cov.
**2000**	0.068	118.147	31.000	0.774	0.761
**2001**	-0.029	83.458	18.000	0.839	0.925
**2002**	-0.041	52.923	35.000	0.704	0.914
**2003**	-0.006	66.954	22.000	0.737	0.866
**2004**	-0.015	179.185	44.670	0.952	0.923
**2005**	-0.006	84.415	35.695	0.895	0.965
**2006**	-0.052	13574.497	24.999	0.852	0.703
**2007**	-0.016	88.706	48.105	0.771	0.956
**2008**	-0.017	94.380	31.000	0.669	0.958
**2009**	0.048	143.796	39.000	0.734	0.866
**2010**	0.001	132.285	60.311	0.828	0.873
**2011**	-0.008	86.703	29.866	0.818	0.943
**2012**	-0.010	98.892	39.952	0.848	0.946
**2013**	-0.010	116.693	37.500	0.920	0.960
**2014**	0.003	101.620	62.000	0.930	0.940
**2015**	-0.003	527.095	101.916	0.780	0.922
**2016**	0.008	113.392	27.000	0.875	0.915
**2017**	-0.027	86.758	33.339	0.820	0.950

d) Predictive out-of-sample metrics by region aggregated to the first administrative level for RHF coverage.

Region	Mean Err.	RMSE	Median SS	Corr.	95% Cov.
**Democratic Republic of the Congo**	-0.003	88.018	60.637	0.925	0.944
**Ethiopia**	-0.001	260.083	115.000	0.772	0.946
**India**	0.009	741.328	113.647	0.686	0.937
**Kenya**	0.000	84.589	23.000	0.876	0.984
**Mongolia**	0.002	30.494	15.000	0.901	0.977
**Nigeria**	-0.005	88.037	33.000	0.833	0.951
**Pakistan**	0.007	699.309	349.000	0.830	0.846
**Yemen**	-0.003	144.275	58.592	0.943	0.945
**Zimbabwe**	0.001	94.912	77.427	0.768	0.953
**Horn of Africa**	-0.053	32660.511	168.824	0.843	0.696
**Central Asia**	0.004	79.091	15.198	0.869	0.909
**Central sub-Saharan Africa**	0.007	132.548	95.707	0.897	0.893
**Eastern sub-Saharan Africa**	0.015	150.346	22.000	0.913	0.907
**Malay Archipelago**	-0.015	40.530	9.000	0.674	0.901
**Mexico, Central America, and the Caribbean**	-0.006	69.530	39.000	0.790	0.917
**The Middle East**	0.001	167.358	97.894	0.970	0.882
**Northern Africa**	-0.001	71.633	38.500	0.874	0.951
**Northern South America**	0.008	56.074	35.629	0.802	0.934
**Southern South America**	-0.005	93.387	54.259	0.773	0.911
**Southeast Asia**	0.011	31.196	4.000	0.775	0.881
**South Asia**	-0.003	118.774	67.277	0.934	0.948
**Southern sub-Saharan Africa**	-0.002	52.539	19.000	0.839	0.893
**Western sub-Saharan Africa**	0.016	179.295	89.000	0.765	0.855

e) Predictive out-of-sample metrics by year aggregated to the second administrative level for RHF coverage.

Year	Mean Err.	RMSE	Median SS	Corr.	95% Cov.
**2000**	0.068	33.864	4.425	0.733	0.761
**2001**	-0.029	24.626	7.000	0.643	0.925
**2002**	-0.041	17.265	3.000	0.478	0.914
**2003**	-0.006	15.910	3.342	0.600	0.866
**2004**	-0.015	35.083	7.827	0.917	0.923
**2005**	-0.006	16.771	4.000	0.754	0.965
**2006**	-0.052	3351.535	4.727	0.809	0.703
**2007**	-0.016	18.797	4.791	0.544	0.956
**2008**	-0.017	21.902	4.000	0.423	0.958
**2009**	0.048	29.979	3.736	0.570	0.866
**2010**	0.001	35.827	7.000	0.679	0.873
**2011**	-0.008	20.677	5.000	0.603	0.943
**2012**	-0.010	25.287	4.023	0.671	0.946
**2013**	-0.010	22.278	6.000	0.698	0.960
**2014**	0.003	29.269	8.000	0.852	0.940
**2015**	-0.003	45.595	13.000	0.614	0.922
**2016**	0.008	37.724	5.000	0.711	0.915
**2017**	-0.027	20.027	4.248	0.640	0.950

f) Predictive out-of-sample metrics by region aggregated to the second administrative level for RHF coverage.

Region	Mean Err.	RMSE	Median SS	Corr.	95% Cov.
**Democratic Republic of the Congo**	-0.003	21.569	7.414	0.737	0.944
**Ethiopia**	-0.001	41.250	20.000	0.528	0.946
**India**	0.009	28.586	6.827	0.428	0.937
**Kenya**	0.000	18.131	6.000	0.632	0.984
**Mongolia**	0.002	7.279	0.898	0.728	0.977
**Nigeria**	-0.005	8.540	3.000	0.433	0.951
**Pakistan**	0.007	197.897	61.000	0.542	0.846
**Yemen**	-0.003	18.089	7.000	0.854	0.945
**Zimbabwe**	0.001	21.528	10.000	0.522	0.953
**Horn of Africa**	-0.053	8622.067	35.805	0.799	0.696
**Central Asia**	0.004	14.821	3.000	0.675	0.909
**Central sub-Saharan Africa**	0.007	45.944	14.000	0.841	0.893
**Eastern sub-Saharan Africa**	0.015	33.713	9.000	0.853	0.907
**Malay Archipelago**	-0.015	10.478	3.000	0.506	0.901
**Mexico, Central America, and the Caribbean**	-0.006	22.666	4.000	0.631	0.917
**The Middle East**	0.001	38.235	13.000	0.931	0.882
**Northern Africa**	-0.001	11.482	4.000	0.521	0.951
**Northern South America**	0.008	12.208	3.000	0.663	0.934
**Southern South America**	-0.005	21.708	4.357	0.636	0.911
**Southeast Asia**	0.011	8.485	1.813	0.651	0.881
**South Asia**	-0.003	26.080	9.000	0.787	0.948
**Southern sub-Saharan Africa**	-0.002	11.931	5.000	0.654	0.893
**Western sub-Saharan Africa**	0.016	51.604	17.827	0.656	0.855

Appendix Table 13a–f. In-sample fit statistics for ORT coverage

In-sample fit statistics are shown for country-level **(a,b)**, first-administrative-level **(c,d)**, and second-administrative-level **(e,f)** aggregations. Metrics are shown by year **(a,c,e)** and by modelling region **(b,d,f)**.

a) Predictive in-sample metrics by year aggregated to the country level for ORT coverage.

Year	Mean Err.	RMSE	Median SS	Corr.	95% Cov.
**2000**	0.032	1481.607	724.600	0.893	0.879
**2001**	-0.036	1320.610	1020.743	0.941	0.964
**2002**	-0.063	1171.350	1058.500	0.743	0.899
**2003**	-0.014	1504.177	846.000	0.939	0.962
**2004**	-0.045	1610.539	922.242	0.979	0.909
**2005**	-0.014	1157.876	916.000	0.956	0.969
**2006**	-0.006	1669.469	1027.000	0.951	0.870
**2007**	-0.031	1325.827	1246.000	0.928	0.936
**2008**	-0.038	1172.165	691.828	0.916	0.956
**2009**	0.052	1782.503	938.976	0.860	0.950
**2010**	-0.012	1793.782	1331.656	0.962	0.922
**2011**	-0.013	1486.056	1112.210	0.958	0.970
**2012**	0.013	1126.820	933.000	0.883	0.974
**2013**	-0.008	1802.264	962.000	0.973	0.980
**2014**	-0.008	1786.090	868.347	0.966	0.973
**2015**	-0.008	5816.660	1869.000	0.978	0.949
**2016**	0.004	2086.229	693.000	0.967	0.963
**2017**	0.000	1489.828	1215.000	0.988	0.984

b) Predictive in-sample metrics by region aggregated to the country level for ORT coverage.

Region	Mean Err.	RMSE	Median SS	Corr.	95% Cov.
**Democratic Republic of the Congo**	0.000	1973.724	1920.623	0.997	0.986
**Ethiopia**	-0.004	2209.427	1543.000	0.973	0.909
**India**	-0.024	9363.632	1997.666	0.921	0.951
**Kenya**	-0.003	2121.621	864.408	0.999	0.990
**Mongolia**	-0.003	358.181	338.538	1.000	0.967
**Nigeria**	-0.043	2139.806	1769.000	0.832	0.916
**Pakistan**	0.002	2883.932	1838.331	1.000	0.950
**Yemen**	-0.017	2777.311	1169.057	1.000	0.983
**Zimbabwe**	-0.002	911.898	669.585	1.000	0.991
**Horn of Africa**	-0.011	2331.054	1069.969	0.917	0.926
**Central Asia**	0.000	430.586	214.000	0.996	0.971
**Central sub-Saharan Africa**	0.015	1445.771	933.000	0.849	0.863
**Eastern sub-Saharan Africa**	0.003	1686.255	1020.743	0.923	0.902
**Malay Archipelago**	-0.001	1258.274	693.000	0.990	0.956
**Mexico, Central America, and the Caribbean**	-0.020	1121.292	862.000	0.689	0.935
**The Middle East**	-0.001	2382.334	1389.783	1.000	0.962
**Northern Africa**	0.000	1200.787	878.000	0.997	0.991
**Northern South America**	0.001	1052.763	311.344	0.995	0.985
**Southern South America**	-0.008	1643.096	1167.385	0.819	0.936
**Southeast Asia**	-0.009	770.511	547.000	0.961	0.978
**South Asia**	-0.012	896.296	602.000	0.984	0.983
**Southern sub-Saharan Africa**	0.001	550.427	413.000	0.973	0.980
**Western sub-Saharan Africa**	0.004	1642.347	1085.170	0.907	0.925

c) Predictive in-sample metrics by year aggregated to the first administrative level for ORT coverage.

Year	Mean Err.	RMSE	Median SS	Corr.	95% Cov.
**2000**	0.032	125.330	31.000	0.879	0.879
**2001**	-0.036	94.015	18.000	0.899	0.964
**2002**	-0.063	99.127	38.500	0.760	0.899
**2003**	-0.014	265.782	20.000	0.874	0.962
**2004**	-0.045	222.751	45.097	0.970	0.909
**2005**	-0.014	86.522	34.561	0.891	0.969
**2006**	-0.006	148.659	24.606	0.932	0.870
**2007**	-0.031	91.908	51.631	0.885	0.936
**2008**	-0.038	96.687	32.000	0.859	0.956
**2009**	0.052	141.163	36.000	0.830	0.950
**2010**	-0.012	161.115	62.000	0.937	0.922
**2011**	-0.013	96.521	31.100	0.915	0.970
**2012**	0.013	90.571	31.793	0.866	0.974
**2013**	-0.008	127.119	40.214	0.957	0.980
**2014**	-0.008	155.359	58.788	0.933	0.973
**2015**	-0.008	518.452	101.000	0.935	0.949
**2016**	0.004	114.229	27.000	0.931	0.963
**2017**	0.000	86.910	33.971	0.932	0.984

d) Predictive in-sample metrics by region aggregated to the first administrative level for ORT coverage.

Region	Mean Err.	RMSE	Median SS	Corr.	95% Cov.
**Democratic Republic of the Congo**	0.000	89.087	60.319	0.945	0.986
**Ethiopia**	-0.004	260.803	115.000	0.952	0.909
**India**	-0.024	593.583	71.610	0.900	0.951
**Kenya**	-0.003	77.816	21.000	0.893	0.990
**Mongolia**	-0.003	31.158	15.153	0.862	0.967
**Nigeria**	-0.043	85.662	33.000	0.841	0.916
**Pakistan**	0.002	1034.970	283.830	0.995	0.950
**Yemen**	-0.017	146.051	59.986	0.936	0.983
**Zimbabwe**	-0.002	95.076	76.129	0.849	0.991
**Horn of Africa**	-0.011	197.514	141.973	0.913	0.926
**Central Asia**	0.000	77.169	13.696	0.889	0.971
**Central sub-Saharan Africa**	0.015	130.829	104.065	0.875	0.863
**Eastern sub-Saharan Africa**	0.003	192.998	23.000	0.893	0.902
**Malay Archipelago**	-0.001	41.604	9.000	0.819	0.956
**Mexico, Central America, and the Caribbean**	-0.020	70.942	39.965	0.682	0.935
**The Middle East**	-0.001	178.443	109.945	0.976	0.962
**Northern Africa**	0.000	65.062	29.000	0.858	0.991
**Northern South America**	0.001	55.102	32.870	0.881	0.985
**Southern South America**	-0.008	93.765	54.637	0.827	0.936
**Southeast Asia**	-0.009	32.473	4.579	0.899	0.978
**South Asia**	-0.012	128.954	71.762	0.972	0.983
**Southern sub-Saharan Africa**	0.001	52.699	24.000	0.588	0.980
**Western sub-Saharan Africa**	0.004	183.106	91.939	0.904	0.925

e) Predictive in-sample metrics by year aggregated to the second administrative level for ORT coverage.

Year	Mean Err.	RMSE	Median SS	Corr.	95% Cov.
**2000**	0.032	34.366	4.501	0.825	0.879
**2001**	-0.036	28.808	7.000	0.815	0.964
**2002**	-0.063	23.996	4.010	0.651	0.899
**2003**	-0.014	54.985	3.610	0.700	0.962
**2004**	-0.045	41.589	8.159	0.950	0.909
**2005**	-0.014	16.020	3.661	0.734	0.969
**2006**	-0.006	28.886	4.103	0.890	0.870
**2007**	-0.031	19.541	5.000	0.790	0.936
**2008**	-0.038	22.238	4.000	0.702	0.956
**2009**	0.052	29.831	3.823	0.687	0.950
**2010**	-0.012	38.339	7.000	0.858	0.922
**2011**	-0.013	23.895	5.000	0.817	0.970
**2012**	0.013	20.316	3.280	0.731	0.974
**2013**	-0.008	25.238	6.000	0.855	0.980
**2014**	-0.008	33.135	7.999	0.834	0.973
**2015**	-0.008	45.696	13.000	0.845	0.949
**2016**	0.004	37.920	5.202	0.805	0.963
**2017**	0.000	19.534	4.263	0.823	0.984

f) Predictive in-sample metrics by region aggregated to the second administrative level for ORT coverage.

Region	Mean Err.	RMSE	Median SS	Corr.	95% Cov.
Democratic Republic of the Congo	0.000	21.702	7.000	0.783	0.986
Ethiopia	-0.004	41.447	20.000	0.840	0.909
India	-0.024	24.377	5.000	0.783	0.951
Kenya	-0.003	17.014	5.000	0.672	0.990
Mongolia	-0.003	7.588	0.907	0.622	0.967
Nigeria	-0.043	8.424	3.161	0.680	0.916
Pakistan	0.002	256.224	60.529	0.971	0.950
Yemen	-0.017	18.231	7.058	0.713	0.983
Zimbabwe	-0.002	21.823	10.000	0.621	0.991
Horn of Africa	-0.011	54.944	27.378	0.892	0.926
Central Asia	0.000	15.102	3.000	0.741	0.971
Central sub-Saharan Africa	0.015	47.247	15.000	0.814	0.863
Eastern sub-Saharan Africa	0.003	40.027	10.000	0.834	0.902
Malay Archipelago	-0.001	10.618	3.000	0.630	0.956
Mexico, Central America, and the Caribbean	-0.020	22.219	4.000	0.540	0.935
The Middle East	-0.001	40.480	14.000	0.935	0.962
Northern Africa	0.000	12.509	3.025	0.575	0.991
Northern South America	0.001	12.059	2.940	0.679	0.985
Southern South America	-0.008	21.730	4.356	0.689	0.936
Southeast Asia	-0.009	8.492	2.000	0.776	0.978
South Asia	-0.012	34.145	11.000	0.916	0.983
Southern sub-Saharan Africa	0.001	11.602	5.000	0.384	0.980
Western sub-Saharan Africa	0.004	52.647	18.000	0.855	0.925

Appendix Table 14a–f. Out-of-sample fit statistics for ORT coverage

Out-of-sample fit statistics are shown for country-level **(a,b)**, first-administrative-level **(c,d)**, and second-administrative-level **(e,f)** aggregations. Metrics are shown by year **(a,c,e)** and by modelling region **(b,d,f)**.

a) Predictive out-of-sample metrics by year aggregated to the country level for ORT coverage.

Year	Mean Err.	RMSE	Median SS	Corr.	95% Cov.
**2000**	0.035	1481.605	724.600	0.894	0.854
**2001**	-0.021	1320.597	1020.743	0.949	0.948
**2002**	-0.052	1171.340	1058.500	0.856	0.920
**2003**	-0.019	1504.181	846.000	0.915	0.931
**2004**	-0.041	1610.535	922.242	0.984	0.892
**2005**	-0.010	1157.873	916.000	0.966	0.937
**2006**	-0.006	1669.469	1027.000	0.958	0.855
**2007**	-0.028	1325.825	1246.000	0.929	0.919
**2008**	-0.037	1172.164	691.828	0.922	0.945
**2009**	0.058	1782.499	938.976	0.863	0.875
**2010**	-0.007	1793.778	1331.656	0.962	0.846
**2011**	-0.014	1486.057	1112.210	0.956	0.940
**2012**	0.011	1126.821	933.000	0.898	0.944
**2013**	-0.004	1802.262	962.000	0.977	0.956
**2014**	-0.009	1786.090	868.347	0.976	0.945
**2015**	-0.006	5816.659	1869.000	0.984	0.920
**2016**	-0.019	2086.245	693.000	0.771	0.871
**2017**	0.003	1489.826	1215.000	0.991	0.962

b) Predictive out-of-sample metrics by region aggregated to the country level for ORT coverage.

Region	Mean Err.	RMSE	Median SS	Corr.	95% Cov.
**Democratic Republic of the Congo**	-0.004	1973.728	1920.623	0.984	0.949
**Ethiopia**	-0.005	2209.427	1543.000	0.977	0.899
**India**	-0.020	9363.630	1997.666	0.936	0.932
**Kenya**	-0.007	2121.624	864.408	0.998	0.986
**Mongolia**	0.006	358.173	338.538	0.999	0.965
**Nigeria**	-0.039	2139.803	1769.000	0.787	0.888
**Pakistan**	-0.050	2883.976	1838.331	0.668	0.757
**Yemen**	-0.004	2777.301	1169.057	0.999	0.966
**Zimbabwe**	0.000	911.896	669.585	0.996	0.986
**Horn of Africa**	-0.003	2331.048	1069.969	0.964	0.833
**Central Asia**	0.006	430.581	214.000	0.992	0.903
**Central sub-Saharan Africa**	0.023	1445.764	933.000	0.813	0.823
**Eastern sub-Saharan Africa**	0.004	1686.254	1020.743	0.930	0.903
**Malay Archipelago**	-0.004	1258.276	693.000	0.988	0.907
**Mexico, Central America, and the Caribbean**	-0.011	1121.285	862.000	0.856	0.941
**The Middle East**	0.000	2382.333	1389.783	0.999	0.911
**Northern Africa**	-0.003	1200.791	878.000	0.991	0.978
**Northern South America**	-0.002	1052.765	311.344	0.994	0.956
**Southern South America**	-0.008	1643.096	1167.385	0.784	0.892
**Southeast Asia**	-0.011	770.513	547.000	0.912	0.930
**South Asia**	-0.005	896.290	602.000	0.972	0.961
**Southern sub-Saharan Africa**	0.000	550.427	413.000	0.927	0.968
**Western sub-Saharan Africa**	0.007	1642.344	1085.170	0.919	0.891

c) Predictive out-of-sample metrics by year aggregated to the first administrative level for ORT coverage.

Year	Mean Err.	RMSE	Median SS	Corr.	95% Cov.
**2000**	0.035	125.329	31.000	0.843	0.854
**2001**	-0.021	94.007	18.000	0.833	0.948
**2002**	-0.052	99.120	38.500	0.782	0.920
**2003**	-0.019	265.783	20.000	0.822	0.931
**2004**	-0.041	222.749	45.097	0.960	0.892
**2005**	-0.010	86.520	34.561	0.846	0.937
**2006**	-0.006	148.659	24.606	0.913	0.855
**2007**	-0.028	91.905	51.631	0.852	0.919
**2008**	-0.037	96.686	32.000	0.813	0.945
**2009**	0.058	141.160	36.000	0.752	0.875
**2010**	-0.007	161.112	62.000	0.883	0.846
**2011**	-0.014	96.522	31.100	0.860	0.940
**2012**	0.011	90.572	31.793	0.820	0.944
**2013**	-0.004	127.118	40.214	0.942	0.956
**2014**	-0.009	155.359	58.788	0.905	0.945
**2015**	-0.006	518.451	101.000	0.920	0.920
**2016**	-0.019	114.241	27.000	0.757	0.871
**2017**	0.003	86.908	33.971	0.907	0.962

d) Predictive out-of-sample metrics by region aggregated to the first administrative level for ORT coverage.

Region	Mean Err.	RMSE	Median SS	Corr.	95% Cov.
**Democratic Republic of the Congo**	-0.004	89.090	60.319	0.791	0.949
**Ethiopia**	-0.005	260.804	115.000	0.875	0.899
**India**	-0.020	593.582	71.610	0.883	0.932
**Kenya**	-0.007	77.819	21.000	0.834	0.986
**Mongolia**	0.006	31.152	15.153	0.839	0.965
**Nigeria**	-0.039	85.660	33.000	0.811	0.888
**Pakistan**	-0.050	1034.996	283.830	0.696	0.757
**Yemen**	-0.004	146.041	59.986	0.850	0.966
**Zimbabwe**	0.000	95.074	76.129	0.726	0.986
**Horn of Africa**	-0.003	197.507	141.973	0.888	0.833
**Central Asia**	0.006	77.165	13.696	0.730	0.903
**Central sub-Saharan Africa**	0.023	130.821	104.065	0.758	0.823
**Eastern sub-Saharan Africa**	0.004	192.998	23.000	0.868	0.903
**Malay Archipelago**	-0.004	41.605	9.000	0.764	0.907
**Mexico, Central America, and the Caribbean**	-0.011	70.936	39.965	0.703	0.941
**The Middle East**	0.000	178.441	109.945	0.942	0.911
**Northern Africa**	-0.003	65.064	29.000	0.783	0.978
**Northern South America**	-0.002	55.104	32.870	0.782	0.956
**Southern South America**	-0.008	93.765	54.637	0.732	0.892
**Southeast Asia**	-0.011	32.474	4.579	0.808	0.930
**South Asia**	-0.005	128.949	71.762	0.927	0.961
**Southern sub-Saharan Africa**	0.000	52.700	24.000	0.524	0.968
**Western sub-Saharan Africa**	0.007	183.103	91.939	0.868	0.891

e) Predictive out-of-sample metrics by year aggregated to the second administrative level for ORT coverage.

Year	Mean Err.	RMSE	Median SS	Corr.	95% Cov.
**2000**	0.035	34.365	4.501	0.771	0.854
**2001**	-0.021	28.801	7.000	0.662	0.948
**2002**	-0.052	23.990	4.010	0.608	0.920
**2003**	-0.019	54.986	3.610	0.608	0.931
**2004**	-0.041	41.587	8.159	0.926	0.892
**2005**	-0.010	16.018	3.661	0.636	0.937
**2006**	-0.006	28.886	4.103	0.844	0.855
**2007**	-0.028	19.540	5.000	0.707	0.919
**2008**	-0.037	22.237	4.000	0.565	0.945
**2009**	0.058	29.829	3.823	0.560	0.875
**2010**	-0.007	38.337	7.000	0.749	0.846
**2011**	-0.014	23.896	5.000	0.714	0.940
**2012**	0.011	20.316	3.280	0.631	0.944
**2013**	-0.004	25.236	6.000	0.778	0.956
**2014**	-0.009	33.135	7.999	0.781	0.945
**2015**	-0.006	45.695	13.000	0.753	0.920
**2016**	-0.019	37.928	5.202	0.606	0.871
**2017**	0.003	19.533	4.263	0.772	0.962

f) Predictive out-of-sample metrics by region aggregated to the second administrative level for ORT coverage.

Region	Mean Err.	RMSE	Median SS	Corr.	95% Cov.
**Democratic Republic of the Congo**	-0.004	21.705	7.000	0.376	0.949
**Ethiopia**	-0.005	41.448	20.000	0.703	0.899
**India**	-0.020	24.375	5.000	0.641	0.932
**Kenya**	-0.007	17.017	5.000	0.507	0.986
**Mongolia**	0.006	7.586	0.907	0.582	0.965
**Nigeria**	-0.039	8.422	3.161	0.551	0.888
**Pakistan**	-0.050	256.249	60.529	0.615	0.757
**Yemen**	-0.004	18.222	7.058	0.486	0.966
**Zimbabwe**	0.000	21.822	10.000	0.422	0.986
**Horn of Africa**	-0.003	54.937	27.378	0.840	0.833
**Central Asia**	0.006	15.099	3.000	0.546	0.903
**Central sub-Saharan Africa**	0.023	47.242	15.000	0.645	0.823
**Eastern sub-Saharan Africa**	0.004	40.026	10.000	0.768	0.903
**Malay Archipelago**	-0.004	10.620	3.000	0.565	0.907
**Mexico, Central America, and the Caribbean**	-0.011	22.215	4.000	0.513	0.941
**The Middle East**	0.000	40.479	14.000	0.865	0.911
**Northern Africa**	-0.003	12.511	3.025	0.436	0.978
**Northern South America**	-0.002	12.061	2.940	0.550	0.956
**Southern South America**	-0.008	21.730	4.356	0.550	0.892
**Southeast Asia**	-0.011	8.494	2.000	0.654	0.930
**South Asia**	-0.005	34.141	11.000	0.796	0.961
**Southern sub-Saharan Africa**	0.000	11.603	5.000	0.243	0.968
**Western sub-Saharan Africa**	0.007	52.645	18.000	0.767	0.891

8.0 Appendix references

1 Munos MK, Walker CLF, Black RE. The effect of oral rehydration solution and recommended home fluids on diarrhoea mortality. *Int J Epidemiol* 2010; **39**: i75–87.

2 Dicker D, Nguyen G, Abate D, *et al.* Global, regional, and national age-sex-specific mortality and life expectancy, 1950–2017: a systematic analysis for the Global Burden of Disease Study 2017. *Lancet* 2018; **392**: 1684–735.

3 Institute for Health Metrics and Evaluation. Global Health Data Exchange (GHDx). http://ghdx.healthdata.org/ (accessed June 18, 2019).

4 Bhatt S, Cameron E, Flaxman SR, Weiss DJ, Smith DL, Gething PW. Improved prediction accuracy for disease risk mapping using Gaussian process stacked generalization. *J R Soc Interface* 2017; **14**: 20170520.

5 Head T, MechCoder, Louppe G, *et al.* scikit-optimize/scikit-optimize: v0.5.2. Zenodo, 2018 DOI:10.5281/zenodo.1207017.

6 Kish L. Survey sampling. New York: John Wiley & Sons, Inc., 1965.

7 WorldPop. Get data. pop_release 2019_08_29. http://www.worldpop.org.uk/data/get_data/ (accessed July 25, 2017).

8 Fullman N, Yearwood J, Abay SM, *et al.* Measuring performance on the Healthcare Access and Quality Index for 195 countries and territories and selected subnational locations: a systematic analysis from the Global Burden of Disease Study 2016. *Lancet* 2018; **391**: 2236–71.

9 Faraway JJ. Linear Models with R, Chapter 4, Problems with the Predictors. CRC Press, 2004.

10 Database of Global Administrative Areas. GADM data version 3.6. Shapefile version 2019_09_10. https://gadm.org/data.html (accessed Sept 10, 2019).

11 Murray CJ, Ezzati M, Flaxman AD, *et al.* GBD 2010: design, definitions, and metrics. *Lancet* 2012; **380**: 2063–6.

12 Stein ML. Interpolation of Spatial Data - Some Theory for Kriging. Springer, 1999 https://www.springer.com/us/book/9780387986296.

13 Gelfand AE, Diggle PJ, Guttorp P, Fuentes M. Handbook of Spatial Statistics. CRC Press, 2010 https://www.crcpress.com/Handbook-of-Spatial-Statistics/Gelfand-Diggle-Guttorp-Fuentes/p/book/9781420072877.

14 Rue H, Martino S, Chopin N. Approximate Bayesian inference for latent Gaussian models by using integrated nested Laplace approximations. *J R Stat Soc Ser B Stat Methodol* 2009; **71**: 319–92.

15 Martins TG, Simpson D, Lindgren F, Rue H. Bayesian computing with INLA: new features. *CSDA* 2013; **67**: 68–83.

16 Lindgren F, Rue H, Lindström J. An explicit link between Gaussian fields and Gaussian Markov random fields: the stochastic partial differential equation approach. *J R Stat Soc Ser B Stat Methodol* 2011; **73**: 423–98.

17 Fuglstad G-A, Simpson D, Lindgren F, Rue H. Constructing Priors that Penalize the Complexity of Gaussian Random Fields. *J Am Stat Assoc* 2019; **114**: 445–52.

18 Dwyer-Lindgren L, Cork MA, Sligar A, *et al.* Mapping HIV prevalence in sub-Saharan Africa between 2000 and 2017. *Nature* 2019; **570**: 189–93.

19 De Maio FG. Income inequality measures. *J Epidemiol Community Health* 2007; **61**: 849–52.

20 Reiner, Jr. RC, Local Burden of Disease Diarrhoea Collaborators, Hay SI. Mapping geographic inequalities in childhood diarrhoeal morbidity and mortality in low-income and middle-income countries, 2000–2017. Under Review.

21 GeoNetwork. The Global Administrative Unit Layers (GAUL). 2015. http://www.fao.org/geonetwork/srv/en/main.home (accessed June 1, 2017).

22 Friedl M, Sulla-Menashe D. MCD12Q1v006.MODIS/Terra+Aqua Land Cover Type Yearly L3 Global 500m SIN Grid. NASA EOSDIS Land Process. DAAC. 2019. https://doi.org/10.5067/MODIS/MCD12Q1.006 (accessed June 1, 2017).

23 World Wildlife Fund. Global Lakes and Wetlands Database, Level 3. 2004. https://www.worldwildlife.org/pages/global-lakes-and-wetlands-database (accessed June 1, 2017).

24 Tatem AJ. WorldPop, open data for spatial demography. *Sci Data* 2017; **4**: 170004.

25 Lehner B, Döll P. Development and validation of a global database of lakes, reservoirs and wetlands. *J Hydrol* 2004; **296**: 1–22.

26 Institute for Health Metrics and Evaluation. Global Burden of Disease Study 2017 (GBD 2017) Socio-Demographic Index (SDI) 1950–2017 Seattle, United States: Institute for Health Metrics and Evaluation (IHME), 2018. 2018. http://ghdx.healthdata.org/record/ihme-data/gbd-2017-socio-demographic-index-sdi-1950%E2%80%932017 (accessed June 1, 2019).

9.0 Author Contributions

Managing the estimation or publications process
Bobby Reiner, Brigette Blacker, Lauren Schaeffer, Paulina Lindstedt, Natalie Cormier.

Writing the first draft of the manuscript
Kirsten Wiens, Lauren Schaeffer, Bobby Reiner, Simon Hay.

Primary responsibility for this manuscript focused on: applying analytical methods to produce estimates
Kirsten Wiens.

Primary responsibility for this manuscript focused on: seeking, cataloguing, extracting, or cleaning data; designing or coding figures and tables
Paulina Lindstedt, Matthew Baumann, Kimberly Johnson.

Providing data or critical feedback on data sources
Hedayat Abbastabar, Michael RM Abrigo, Manfred Accrombessi, Maryam Adabi, Daniel Adane, Oladimeji Adebayo, Rufus A Adedoyin, Victor Adekanmbi, Olatunji Adetokunboh, Mohsen Afarideh, Sohail Ahmad, Muktar Ahmed, Fares Alahdab, Jacqueline E Alcalde-Rabanal, Beriwan Ali, Syed Aljunid, Amir Almasi-Hashiani, Hesham Al-Mekhlafi, Nelson Alvis-Guzman, Nelson J Alvis-Zakzuk, Fereshteh Ansari, Jalal Arabloo, Hany Aref, Olatunde Aremu, Bahram Armoon, Amit Arora, Krishna K Aryal, Seyyed shamsadin Athari, Nefsu Awoke, Beatriz Paulina Ayala Quintanilla, Yared Aynalem, Samad Azari, Senthilkumar Balakrishnan, Maciej Banach, Joseph Banoub, Till Bärnighausen, Sanjay Basu, Estifanos Baye, Masoud Behzadifar, Meysam Behzadifar, Robert Bernstein, Ali Bijani, Henok Biresaw, Soufiane Boufous, Andrey Briko, Gabrielle Britton, Gesssessew Bugssa, Sharath Burugina Nagaraja, Reinhard Busse, Jorge Cano, Carlos Castañeda-Orjuela, Franz Castro, Vijay Kumar Chattu, Devasahayam Christopher, Dinh Toi Chu, Vera M Costa, Carlos Culquichicon, Giovanni Damiani, Lalit Dandona, Rakhi Dandona, Amira Hamed Darwish, Ahmad Daryani, Rajat Das Gupta, Bárbara Niegia de Goulart, Fernando de la Hoz, Asmamaw Demis, Dereje Demissie, Kebede Deribe, Samath Dharmaratne, Preeti Dhillon, Meghnath Dhimal, Govinda Dhungana, Kerrie Doyle, Bereket Duko, Andem Effiong, Nevine El Nahas, Maysaa El Sayed Zaki, Hajer Elkout, Iqbal Elyazar, Aklilu Endalamaw , Sadaf Esteghamati, Alireza Esteghamati, Arash Etemadi, Mohammad Fareed, Farshad Farzadfar, Valery Feigin, Morenike Oluwatoyin Folayan, Takeshi Fukumoto, Mohamed Gad, Teshome Gebre, Feleke Gebremeskel, Gebrekiros Gebremichael, Ayele Geleto, Keyghobad Ghadiri, Ahmad Ghashghaee, Pramesh Raj Ghimire, Nelson Gomes, Ayman Grada, Harish Gugnani, Yuming Guo, Nima Hafezi-Nejad, Arvin Haj-Mirzaian, Demelash Woldeyohannes Handiso, Hamidreza Haririan, Edris Hasanpoor, Amir Hasanzadeh, Hadi Hassankhani, Hamid Y Hassen, Mohamed Hegazy, Behnam Heidari, Delia Hendrie, Claudiu Herteliu, Thomas Hird, Enayatollah Homaie Rad, Praveen Hoogar, Mohammad Ali Hosseini, Mehdi Hosseinzadeh, Mowafa Househ , Mohamed HSAIRI, Ehimario Igumbor, Usman Iqbal, Sheikh Mohammed Shariful Islam, Nader Jahanmehr, Mihajlo Jakovljevic, Ahamarshan Jayaraman Nagarajan, John Ji, Kimberly Johnson, Jost B Jonas, Jacek Jozwiak, Zubair Kabir, André Karch, Surendra Karki, Amir Kasaeian, Gebremicheal Kasahun, Getachew Kassa, Gbenga Kayode, Abraham Getachew Kelbore, Yousef Khader, Morteza Abdullatif Khafaie, Nauman Khalid, Rovshan Khalilov, Md Nuruzzaman Khan, Khaled Khatab, Amir Khater, Salman Khazaei, Mohammad Khazaei, Mohammad Hossein Khosravi, Jagdish Khubchandani, Anh Kim Dang, Adnan Kisa, Sezer Kisa, Soewarta Kosen, Parvaiz Koul, Kewal Krishan, Manasi Kumar, Pushpendra Kumar, Dian Kusuma, Sheetal Lad, Dharmesh Lal, Felix Lam, Van Lansingh, Savita Lasrado, Mostafa Leili, Cheru Tesema Leshargie, Simin Liu, Shiwei Liu, Tsegaye Lolaso, Joshua Longbottom, Jaifred Christian Lopez, Mohammed Magdy Abd El Razek, D R Mahadeshwara Prasad, Narayan Mahotra, Navid Manafi, Mohammad Ali Mansournia, Joemer Maravilla, Francisco Rogerlândio Martins-Melo, Manu Mathur, Benjamin Mayala, Mohsen Mazidi, Colm McAlinden, Ziad A Memish, Walter Mendoza, Ritesh G Menezes, Bartosz Miazgowski, Biniam Minuye, Erkin Mirrakhimov, Babak Moazen, Bahram Mohajer, Amjad Mohamadi Bolbanabad, Karzan Mohammad, Dara Mohammad, Naser Mohammad Gholi Mezerji, Shafiu Mohammed, Jemal Abdu Mohammed , Farnam Mohebi, Ali Mokdad, Mariam Molokhia, Yoshan Moodley, Masoud Moradi, Mohammad Moradi-Joo, Ilais Moreno Velásquez, Seyyed Meysam Mousavi, Miliva Mozaffor, Kamarul Imran Musa, Ghulam Mustafa, Saravanan Muthupandian, Seyed Sina Naghibi Irvani, Bruno Nascimento, Javad Nazari, Ionut Negoi, Josephine Ngunjiri, Dabere Nigatu, Marzieh Nojomi, Jean Jacques Noubiap, Okechukwu Ogah, Andrew T Olagunju, Tinuke O Olagunju, Bolajoko Olusanya, Obinna Onwujekwe, Mahesh P A, Jagadish Rao Padubidri, Sangram Kishor Patel, Angel Paternina-Caicedo, Kebreab Paulos, Alexandre Pereira, Bakhtiar

Piroozi, Maarten Postma, Hadi Pourjafar, Hossein Poustchi , Sergio Prada, Narayan Prasad, Hedley Quintana, Amir Radfar, Alireza Rafiei, Fakher Rahim, Vafa Rahimi-Movaghar, Vafa Rahimi-Movaghar, Afarin Rahimi-Movaghar, Mohammad Hifz Ur Rahman, Fatemeh Rajati, Saleem Rana, Chhabi Lal Ranabhat, Salman Rawaf, Lal Rawal, Andre Renzaho, Seyed Mohammad Riahi, Jennifer Rickard, Leonardo Roever, Luca Ronfani, Enrico Rubagotti, Siamak Sabour, Ehsan Sadeghi, Rajesh Sagar, S Mohammad Sajadi, Mohammad Reza Salahshoor, Nasir Salam, Hosni Salem, Marwa Salem, Yahya Salimi, Hamideh Salimzadeh, Abdallah M Samy, Milena Santric-Milicevic, Bruno Sao Jose, Abdur Razzaque Sarker, Nizal Sarrafzadegan, Brijesh Sathian, Sonia Saxena, David C Schwebel, Sadaf Sepanlou, Edson Serván-Mori, Masood Ali Shaikh, Ali Shalash, Seifadin Ahmed Shallo , Morteza Shamsizadeh, Mohd Shannawaz, B Suresh Shetty, Jae Il Shin, Soraya Siabani, Jasvinder Singh, Virendra Singh, Eirini Skiadaresi, Mohammadreza Sobhiyeh, Chandrashekhar T Sreeramareddy, Agus Sudaryanto, Hafiz Ansar Rasul Suleria, Bryan Sykes, Rafael Tabarés-Seisdedos, Getasew Taddesse, Degena Bahrey Tadesse, Nihal Thomas, Alemayehu Toma, Marcos Roberto Tovani-Palone, Bach Tran, Khanh Bao Tran, Reta Tsegaye, Irfan Ullah, Bhaskaran Unnikrishnan, Muhammad Shariq Usman, Pascual Valdez, Yasir Waheed, Yafeng Wang, Girmay Weldesamuel, Ronny Westerman, Taweewat Wiangkham, Kirsten Wiens, Charles Shey Wiysonge, Haileab Fekadu Wolde, Gelin Xu, Ali Yadollahpour, Alex Yeshaneh, Christopher Yilgwan, Tesfaye Yitna, Naohiro Yonemoto, Seok-Jun Yoon, Mustafa Younis, Siddhesh Zadey, Zoubida Zaidi, Mohammad Zamani, Nejimu Zepro, Yunquan Zhang, Xiu-Ju Zhao, Arash Ziapour, Yves Miel Zuniga.

Development of methods or computational machinery
Olatunji Adetokunboh, Saeed Amini, Samad Azari, Meysam Behzadifar, Sharath Burugina Nagaraja, Ahmad Daryani, Rajat Das Gupta, Dereje Demissie, Aniruddha Deshpande, Maysaa El Sayed Zaki, Ahmad Ghashghaee, Nathaniel Henry, Mohammad Ali Hosseini, Mowafa Househ , Mihiretu Kebede, Rovshan Khalilov, Adnan Kisa, Sezer Kisa, Pushpendra Kumar, Paulina Lindstedt, Hassan Magdy Abd El Razek, Navid Manafi, Ali Mokdad, Javad Nazari, Okechukwu Ogah, Ashish Pathak, Chhabi Lal Ranabhat, Seyed Mohammad Riahi, Enrico Rubagotti, Mohammad Reza Salahshoor, Abdallah M Samy, Abdur Razzaque Sarker, Ronny Westerman, Kirsten Wiens, Ali Yadollahpour, Seok-Jun Yoon, Zoubida Zaidi, Arash Ziapour

Providing critical feedback on methods or results
Hedayat Abbastabar, Foad Abd-Allah, Ahmed Abdelalim, Ibrahim Abdollahpour, Hailemariam Abiy, Lucas Guimarães Abreu, Michael RM Abrigo, Ahmed Abualhasan, Dilaram Acharya, Abdu A Adamu, Daniel Adane, Oladimeji Adebayo, Victor Adekanmbi, Olatunji Adetokunboh, Beyene Adhena, Mohsen Afarideh, Sohail Ahmad, Keivan Ahmadi, Muktar Ahmed, Anwar Ahmed, Rushdia Ahmed, Dessalegn Ajema, Temesgen Yihunie Akalu, Fares Alahdab, Ziyad Al-Aly, Samiah Alam, Noore Alam, Genet Melak Alamene, Turki Alanzi, Jacqueline E Alcalde-Rabanal, Beriwan Ali, Mehran Alijanzadeh, Vahid Alipour, Syed Aljunid, Amir Almasi-Hashiani, Hesham Al-Mekhlafi, Khalid Altirkawi, Nelson Alvis-Guzman, Nelson J Alvis-Zakzuk, Saeed Amini, Arianna Maever Amit, Fereshteh Ansari, Carl Abelardo Antonio, Ernoiz Antriyandarti, Amir Anushiravani, Jalal Arabloo, Olatunde Aremu, Amit Arora, Krishna K Aryal, Afsaneh Arzani, Hagos Tasew Atalay, Seyyed shamsadin Athari, Sachin Atre, Nefsu Awoke, Beatriz Paulina Ayala Quintanilla, Getinet Ayano, Martin Ayanore, Yared Aynalem, Samad Azari, peter azzopardi, Ebrahim Babaee, Tesleem Babalola, Alaa Badawi, Mohan Bairwa, Shankar Bakkannavar, Senthilkumar Balakrishnan, Maciej Banach, Joseph Banoub, Aleksandra Barac, Till Bärnighausen, Sanjay Basu, Mohsen Bayati, Estifanos Baye, Neeraj Bedi, Mahya Beheshti, Masoud Behzadifar, Meysam Behzadifar, Bayu Begashaw Bekele, Yaschilal Muche Belayneh, Michelle Bell, Derrick Bennett, Robert Bernstein, Anusha Ganapati Bhat, Suraj Bhattari, Soumyadeep Bhaumik, Zulfiqar Bhutta, Ali Bijani, Boris Bikbov, Henok Biresaw, Hunduma Bojia, Soufiane Boufous, Oliver Brady, Nicola Luigi Bragazzi, Andrey Briko, Gabrielle Britton, Gesssessew Bugssa, Sharath Burugina Nagaraja, Reinhard Busse, Zahid Butt, Alessandra C Goulart, Luis Alberto Cámera, Ismael Campos-Nonato, Jorge Cano, Rosario Cárdenas, Deborah Carvalho Malta, Carlos Castañeda-Orjuela, Franz Castro, Wagaye Chanie, Pranab Chatterjee, Vijay Kumar Chattu, Ken Chin, Dinh Toi Chu, Vera M Costa, Giovanni Damiani, Lalit Dandona, Rakhi Dandona, Amira Hamed Darwish, Ahmad Daryani, Jai Das, Rajat Das Gupta, Adrian Davis, Bárbara Niegia de Goulart, Fernando de la Hoz, Asmamaw Demis, Dereje Demissie, Getu Demissie, Edgar Denova-Gutiérrez, Kebede Deribe, Assefa Desalew, Aniruddha Deshpande, Preeti Dhillon, Meghnath Dhimal, Daniel Diaz, Shirin Djalalinia, Kerrie Doyle, Eleonora Dubljanin, Bereket Duko, Mohammad Ebrahimi Kalan, Hisham Atan Edinur, Andem Effiong, Aziz Eftekhari, Nevine El Nahas, Iman El Sayed, Maysaa El Sayed Zaki, Teshome Elema, Aisha Elsharkawy, Aklilu Endalamaw , Tewodros Eshete, Arash Etemadi, Oluchi Ezekannagha, Mohammad Fareed, Farshad Farzadfar, Mehdi Fazlzadeh, Getahun Fentaw, Seyed-Mohammad Fereshtehnejad, Florian Fischer, Nataliya Foigt, Morenike Oluwatoyin Folayan, Masoud Foroutan, Richard

Franklin, Takeshi Fukumoto, Mohamed Gad, Teshome Gebre, Ketema Bizuwork Gebremedhin, Merhawi Gebremedhin, Gebrekiros Gebremichael, Ayele Geleto, Hailay Gesesew, Kebede Embaye Gezae, Ahmad Ghashghaee, Pramesh Raj Ghimire, Paramjit Gill, Tiffany K Gill, Themba G Ginindza, Bedilu Girma, Nelson Gomes, Sameer Gopalani, Ayman Grada, Harish Gugnani, Davide Guido, Rafael Guimarães, Yuming Guo, Nima Hafezi-Nejad, Dessalegn Haile, Samer Hamidi, Demelash Woldeyohannes Handiso, Md Mehedi Hasan, Edris Hasanpoor, Amir Hasanzadeh, Hadi Hassankhani, Hamid Y Hassen, Simon Hay, Mohamed Hegazy, Behzad Heibati, Behnam Heidari, Delia Hendrie, Nathaniel Henry, Claudiu Herteliu, Fatemeh Heydarpour, Fatemeh Heydarpour, Hagos Hidru, Thomas Hird, Enayatollah Homaie Rad, Praveen Hoogar, Naznin Hossain, Mohammad Ali Hosseini, Mehdi Hosseinzadeh, Mowafa Househ , Guoqing Hu, Mohammedaman Mama Hussen, Mohammed IM Gubari, Segun Emmanuel Ibitoye, Ehimario Igumbor, Olayinka Ilesanmi, Milena Ilic, Usman Iqbal, Sheikh Mohammed Shariful Islam, Chinwe Juliana Iwu, Neda Izadi, Anelisa Jaca, Nader Jahanmehr, Mihajlo Jakovljevic, Amir Jalali, Ahamarshan Jayaraman Nagarajan, Achala Jayatilleke, Vivekanand Jha, Ravi Prakash Jha, John Ji, Jost B Jonas, Jacek Jozwiak, Ali Kabir, Zubair Kabir, Amaha Kahsay, Tanuj Kanchan, Behzad Karami Matin, André Karch, Mohd Anisul Karim, Hamidreza Karimi-Sari, Surendra Karki, Amir Kasaeian, Yawukal Kasahun, Habtamu Kasaye , Getachew Kassa, Gebrehiwot G Kassa, Gbenga Kayode, Mihiretu Kebede, Peter Keiyoro, Abraham Getachew Kelbore, Daniel Ketema, Yousef Khader, Morteza Abdullatif Khafaie, Nauman Khalid, Rovshan Khalilov, Md Nuruzzaman Khan, Junaid Khan, Ejaz Khan, Khaled Khatab, Mona Khater, Maryam Khayamzadeh, Mohammad Hossein Khosravi, Jagdish Khubchandani, Ali Kiadaliri, Yun Jin Kim, Anh Kim Dang, Ruth Kimokoti, Adnan Kisa, Sezer Kisa, Niranjan Kissoon, Shivakumar KM KM Shivakumar, Tufa Kolola, Ai Koyanagi, Moritz Kraemer, Kewal Krishan, Nuworza Kugbey, Manasi Kumar, Pushpendra Kumar, Vivek Kumar, Dian Kusuma, Sheetal Lad, Dharmesh Lal, Felix Lam, Savita Lasrado, Avula Laxmaiah, Kate LeGrand, Shanshan Li, Shai Linn, Simin Liu, Shiwei Liu, Rakesh Lodha, Tsegaye Lolaso, Mohammed Magdy Abd El Razek, Hassan Magdy Abd El Razek, D R Mahadeshwara Prasad, Phetole Mahasha, Azeem Majeed, Abdullah Mamun, Navid Manafi, Narendar Manohar, Mohammad Ali Mansournia, Chabila C Mapoma, Joemer Maravilla, Santi Martini, Francisco Rogerlândio Martins-Melo, Anthony Masaka, Benjamin Massenburg, Manu Mathur, Benjamin Mayala, Mohsen Mazidi, Colm McAlinden, Birhanu Geta Meharie, Kala M Mehta, Tefera Chane Mekonnen, Peter Memiah, Ziad A Memish, Walter Mendoza, Ritesh G Menezes, Elias Merdassa, Seid Tiku Mereta, Ted R Miller, GK Mini, Biniam Minuye, Erkin Mirrakhimov, Babak Moazen, Amjad Mohamadi Bolbanabad, Yousef Mohammad, Dara Mohammad, Naser Mohammad Gholi Mezerji, Roghayeh Mohammadibakhsh, Noushin Mohammadifard, Shafiu Mohammed, Jemal Abdu Mohammed , Farnam Mohebi, Ali Mokdad, Mariam Molokhia, Lorenzo Monasta, Ghobad Moradi, Mohammad Moradi-Joo, Maziar Moradi-Lakeh, Linda Morales, Abbas Mosapour, Simin Mouodi, Miliva Mozaffor, Kindie Fentahun Muchie, kebadnew Mulatu, Moses Muriithi, GVS Murthy, Kamarul Imran Musa, Saravanan Muthupandian, Ashraf Nabhan, Mehdi Naderi, Seyed Sina Naghibi Irvani, Kovin Naidoo, Gurudatta Naik, Farid Najafi, Vinay Nangia, Bruno Nascimento, Javad Nazari, Duduzile Ndwandwe, Ayenew Negesse, Ionut Negoi, Josephine Ngunjiri, Dabere Nigatu, Solomon Nigatu, Dina Nur Anggraini Ningrum, Chukwudi Nnaji, Ole F Norheim, Jean Jacques Noubiap, soraya Nouraei Motlagh, Bogdan Oancea, Okechukwu Ogah, Felix Ogbo, In-Hwan Oh, Andrew T Olagunju, Tinuke O Olagunju, Bolajoko Olusanya, Obinna Onwujekwe, Doris V Ortega-Altamirano, Frank Osei, Mayowa Owolabi, Mahesh P A, Jagadish Rao Padubidri, Smita Pakhale, Sangram Kishor Patel, Angel Paternina-Caicedo, Ashish Pathak, Deepak Paudel, Kebreab Paulos, Veincent Christian Pepito, Alexandre Pereira, Julia Pescarini, Meghdad Pirsaheb , Maarten Postma, Hadi Pourjafar, Farshad Pourmalek, Akram Pourshams, Sergio Prada, Navid Rabiee, Amir Radfar, Alireza Rafiei, Fakher Rahim, Vafa Rahimi-Movaghar, Vafa Rahimi-Movaghar, Muhammad Aziz Rahman, Mohammad Hifz Ur Rahman, Md SHafiur Rahman, Chhabi Lal Ranabhat, Salman Rawaf, Lal Rawal, Vishnu Renjith, Andre Renzaho, Serge Resnikoff, Melese Reta, Negar Rezaei, Mohammad sadegh Rezai, Seyed Mohammad Riahi, Ana Isabel Ribeiro, Jennifer Rickard, Leonardo Roever, Enrico Rubagotti, Yogesh Sabde, Siamak Sabour, Ehsan Sadeghi, Roya Safari-Faramani, Rajesh Sagar, Amirhossein Sahebkar, Mohammad Ali Sahraian, Mohammad Ali Sahraian, S Mohammad Sajadi, Mohammad Reza Salahshoor, Nasir Salam, Hosni Salem, Marwa Salem, Yahya Salimi, Abdallah M Samy, Juan Sanabria, Milena Santric-Milicevic, Sivan Saraswathy, Kaushik Sarkar, Abdur Razzaque Sarker, Benn Sartorius, Brijesh Sathian, Thirunavukkarasu Sathish, Monika Sawhney, Lauren Schaeffer, David C Schwebel, Anbissa Muleta Senbeta, Subramanian Senthilkumaran, Sadaf Sepanlou, Edson Serván-Mori, Masood Ali Shaikh, Ali Shalash, Seifadin Ahmed Shallo , Mehran Shams-Beyranvand, Mohammadbagher Shamsi, Morteza Shamsizadeh, Mohd Shannawaz, Kiomars Sharafi, Aziz Sheikh, wondimeneh shiferaw, Desalegn Markos Shifti, Mika Shigematsu, Jae Il Shin, Rahman Shiri, Reza Shirkoohi, Soraya Siabani, Diego Augusto Santos Silva, Jasvinder Singh, Ambrish Singh, Narinder Pal Singh, Malede Sisay, Eirini Skiadaresi, Matiwos Soboka, Ranjani Somayaji, Moslem Soofi, Muluken Sorrie, Ireneous Soyiri, Chandrashekhar T Sreeramareddy, Agus Sudaryanto, Mu'awiyyah Babale Sufiyan, Hafiz Ansar Rasul

Suleria, Marufa Sultana, Bruno Sunguya, Bryan Sykes, Rafael Tabarés-Seisdedos, Degena Bahrey Tadesse, Ingan Tarigan, Aberash Tassew, Mohamad-Hani Temsah, Berhe Etsay Tesfay, Belay Tessema, Kavumpurathu Thankappan, Nihal Thomas, Alemayehu Toma, Marcos Roberto Tovani-Palone, Eugenio Traini, Bach Tran, Khanh Bao Tran, Reta Tsegaye, Irfan Ullah, Bhaskaran Unnikrishnan, Muhammad Shariq Usman, Pascual Valdez, Santosh Varughese, Francesco S Violante, Sebastian Vollmer, Yasir Waheed, Mitchell Wallin, Yuan-Pang Wang, Yafeng Wang, Girmay Weldesamuel, Catherine Welgan, Ronny Westerman, Taweewat Wiangkham, Kirsten Wiens, Charles Shey Wiysonge, Haileab Fekadu Wolde, Dawit Zewdu Wondafrash , Ai-Min Wu, Gelin Xu, Ali Yadollahpour, Tomohide Yamada, Alex Yeshaneh, Christopher Yilgwan, Mekdes Yilma, Paul Yip, Engida Yisma, Tesfaye Yitna, Naohiro Yonemoto, Seok-Jun Yoon, Mustafa Younis, Mahmoud Yousefifard, Chuanhua Yu, Hasan Yusefzadeh, Siddhesh Zadey, Zoubida Zaidi, Sojib Bin Zaman, Hamed Zandian, Nejimu Zepro, Taddese Zerfu, Yunquan Zhang, Xiu-Ju Zhao, Arash Ziapour, Sanjay Zodpey,

Drafting the manuscript or revising it critically for important intellectual content
Hedayat Abbastabar, Foad Abd-Allah, Ahmed Abdelalim, Kedir Abegaz, Lucas Guimarães Abreu, Ahmed Abualhasan, Oladimeji Adebayo, Rufus A Adedoyin, Victor Adekanmbi, Olatunji Adetokunboh, Muktar Ahmed, Rushdia Ahmed, Temesgen Yihunie Akalu, Fares Alahdab, Samiah Alam, Genet Melak Alamene, Jacqueline E Alcalde-Rabanal, Ali Almasi, Amir Almasi-Hashiani, Nelson Alvis-Guzman, Saeed Amini, Carl Abelardo Antonio, Jalal Arabloo, Olatunde Aremu, Mehran Asadi-Aliabadi, Hagos Tasew Atalay, Seyyed shamsadin Athari, Nefsu Awoke, Beatriz Paulina Ayala Quintanilla, Martin Ayanore, peter azzopardi, Alaa Badawi, Shankar Bakkannavar, Senthilkumar Balakrishnan, Maciej Banach, Aleksandra Barac, Till Bärnighausen, Huda Basaleem, Sanjay Basu, Neeraj Bedi, Masoud Behzadifar, Meysam Behzadifar, Michelle Bell, Anusha Ganapati Bhat, Boris Bikbov, Raaj Kishore Biswas, Hunduma Bojia, Oliver Brady, Nikolay Briko, Gabrielle Britton, Gesssessew Bugssa, Sharath Burugina Nagaraja, Josip Car, Felix Carvalho, Carlos Castañeda-Orjuela, Franz Castro, Pranab Chatterjee, Vijay Kumar Chattu, Ken Chin, Devasahayam Christopher, Dinh Toi Chu, Vera M Costa, Giovanni Damiani, Amira Hamed Darwish, Ahmad Daryani, Gail Davey, Claudio Davila-Cervantes, Bárbara Niegia de Goulart, Asmamaw Demis, Dereje Demissie, Gebre Teklemariam Demoz, Edgar Denova-Gutiérrez, Kebede Deribe, Assefa Desalew, Samath Dharmaratne, Meghnath Dhimal, Govinda Dhungana, Daniel Diaz, Isaac Dipeolu, Kerrie Doyle, Andre Duraes, Andem Effiong, Aziz Eftekhari, Iman El Sayed, Maysaa El Sayed Zaki, Maha El Tantawi, Hala Elhabashy, Shaimaa El-Jaafary, Aisha Elsharkawy, Aklilu Endalamaw , Tewodros Eshete, Arash Etemadi, Oluchi Ezekannagha, Valery Feigin, Getahun Fentaw, Seyed-Mohammad Fereshtehnejad, Florian Fischer, Nataliya Foigt, Morenike Oluwatoyin Folayan, Masoud Foroutan, Takeshi Fukumoto, Mohamed Gad, Gebreamlak Gebremeskel, Gebrekiros Gebremichael, Hailay Gesesew, Paramjit Gill, Tiffany K Gill, Themba G Ginindza, Nelson Gomes, Sameer Gopalani, Ayman Grada, Harish Gugnani, Rafael Guimarães, Rajeev Gupta, Nima Hafezi-Nejad, Randah Hamadeh, Demelash Woldeyohannes Handiso, Ninuk Hariyani, Ahmed Hasaballah, Edris Hasanpoor, Amir Hasanzadeh, Hamid Y Hassen, Simon Hay, Mohamed Hegazy, Claudiu Herteliu, Thomas Hird, Praveen Hoogar, Naznin Hossain, Mohammad Ali Hosseini, Mehdi Hosseinzadeh, Mowafa Househ , Segun Emmanuel Ibitoye, Ehimario Igumbor, Olayinka Ilesanmi, Milena Ilic, Mohammad-Hasan Imani-Nasab, Usman Iqbal, Sheikh Mohammed Shariful Islam, Chinwe Juliana Iwu, Mihajlo Jakovljevic, Ahamarshan Jayaraman Nagarajan, Vivekanand Jha, Ravi Prakash Jha, Jost B Jonas, Jacek Jozwiak, Ali Kabir, Hamed Kalani, André Karch, Hamidreza Karimi-Sari, Gebremicheal Kasahun, Yawukal kasahun, Habtamu Kasaye , Gbenga Kayode, Mihiretu Kebede, Andre Pascal Kengne, Morteza Abdullatif Khafaie, Rovshan Khalilov, Junaid Khan, Ejaz Khan, Khaled Khatab, Mona Khater, Mohammad Khazaei, Mohammad Hossein Khosravi, Jagdish Khubchandani, Ali Kiadaliri, Yun Jin Kim, Anh Kim Dang, Adnan Kisa, Sezer Kisa, Niranjan Kissoon, Shivakumar KM KM Shivakumar, Dr Sonali Kochhar, Parvaiz Koul, Ai Koyanagi, Kewal Krishan, Nuworza Kugbey, Pushpendra Kumar, Vivek Kumar, Dian Kusuma, Carlo La Vecchia, Ben Lacey, Faris Lami, Prabhat Lamichhane, Savita Lasrado, Paul Lee, Kate LeGrand, Shanshan Li, Simin Liu, Joshua Longbottom, Jaifred Christian Lopez, Mohammed Magdy Abd El Razek, Hassan Magdy Abd El Razek, Azeem Majeed, Abdullah Mamun, Navid Manafi, Ana Laura Manda, Mohammad Ali Mansournia, Gabriel Martinez, Francisco Rogerlândio Martins-Melo, Benjamin Massenburg, Mohsen Mazidi, Colm McAlinden, Walter Mendoza, Ritesh G Menezes, Tuomo Meretoja, Tomislav Mestrovic, Ted R Miller, GK Mini, Babak Moazen, Bahram Mohajer, Karzan Mohammad, Yousef Mohammad, Dara Mohammad, Shafiu Mohammed, Farnam Mohebi, Ali Mokdad, Mariam Molokhia, Lorenzo Monasta, Catrin Moore, Ghobad Moradi, Maziar Moradi-Lakeh, Paula Moraga, Abbas Mosapour, Seyyed Meysam Mousavi, Miliva Mozaffor, Sandra Munro, Kamarul Imran Musa, Seyed Sina Naghibi Irvani, Vinay Nangia, Jobert Richie Nansseu, Bruno Nascimento, Javad Nazari, Ayenew Negesse, Ionut Negoi, Josephine Ngunjiri, Dabere Nigatu, Ole F Norheim, Jean Jacques Noubiap, Okechukwu Ogah, Felix Ogbo, In-Hwan Oh, Andrew T Olagunju, Tinuke O Olagunju, Bolajoko Olusanya, Obinna

Onwujekwe, Eyal Oren, Doris V Ortega-Altamirano, Osayomwanbo Osarenotor, Mayowa Owolabi, Mahesh P A, Jagadish Rao Padubidri, Smita Pakhale, George Patton, Veincent Christian Pepito, Alexandre Pereira, Norberto Perico, Aslam Pervaiz, Julia Pescarini, Maarten Postma, Farshad Pourmalek, Sergio Prada, Navid Rabiee, Amir Radfar, Fakher Rahim, Vafa Rahimi-Movaghar, Vafa Rahimi-Movaghar, Afarin Rahimi-Movaghar, Muhammad Aziz Rahman, Mohammad Hifz Ur Rahman, Chhabi Lal Ranabhat, Davide Rasella, Salman Rawaf, Lal Rawal, Wasiq Rawasia, Vishnu Renjith, Andre Renzaho, Seyed Mohammad Riahi, Ana Isabel Ribeiro, Jennifer Rickard, Maria Jesus Rios Blancas , Leonardo Roever, Luca Ronfani, Jennifer Ross, Anas Saad, Rajesh Sagar, Mohammad Ali Sahraian, Mohammad Ali Sahraian, Mohammad Reza Salahshoor, Nasir Salam, Hosni Salem, Marwa Salem, Hamideh Salimzadeh, Abdallah M Samy, Juan Sanabria, Milena Santric-Milicevic, Kaushik Sarkar, Abdur Razzaque Sarker, Lauren Schaeffer, David C Schwebel, Sadaf Sepanlou, Azadeh Shafieesabet, Ali Shalash, Mehran Shams-Beyranvand, Hatem Shehata, Aziz Sheikh, Desalegn Markos Shifti, Mika Shigematsu, Reza Shirkoohi, Kenji Shiubya, Diego Augusto Santos Silva, Jasvinder Singh, Eirini Skiadaresi, Anton Sokhan, Ranjani Somayaji, Muluken Sorrie, Ireneous Soyiri, Chandrashekhar T Sreeramareddy, Mu'awiyyah Babale Sufiyan, Bryan Sykes, Rafael Tabarés-Seisdedos, Takahiro Tabuchi, Zemenu Tadesse, Yonatal Mesfin Tefera, Mohamad-Hani Temsah, Fisaha Tesfay, Nihal Thomas, Alemayehu Toma, Roman Topor-Madry, Marcos Roberto Tovani-Palone, Bach Tran, Reta Tsegaye, Irfan Ullah, Bhaskaran Unnikrishnan, Benjamin Uzochukwu, Francesco S Violante, Yuan-Pang Wang, Catherine Welgan, Ronny Westerman, Kirsten Wiens, Charles Shey Wiysonge, Haileab Fekadu Wolde, Dawit Zewdu Wondafrash , Ai-Min Wu, Hossein Yahyazadeh, Hiroshi Yatsuya, Christopher Yilgwan, Mekdes Yilma, Tesfaye Yitna, Naohiro Yonemoto, Seok-Jun Yoon, Mahmoud Yousefifard, Hebat-Allah Yousof, Zoubida Zaidi, Sojib Bin Zaman, Mohammad Zamani, Xiu-Ju Zhao, Arash Ziapour

Management of the overall research enterprise (for example, through membership in the Scientific Council)
Rufus A Adedoyin, Muktar Ahmed, Meysam Behzadifar, Brigette Blacker, Deborah Carvalho Malta, Devasahayam Christopher, Lalit Dandona, Adrian Davis, Aziz Eftekhari, Simon Hay, Mohammad Ali Hosseini, Mowafa Househ , Hamidreza Karimi-Sari, Pushpendra Kumar, Aubrey Levine, Paulina Lindstedt, Mohammed Magdy Abd El Razek, Hassan Magdy Abd El Razek, Navid Manafi, Mohsen Mazidi, Ali Mokdad, Mahesh P A, George Patton, Navid Rabiee, Lal Rawal, Vishnu Renjith, Rajesh Sagar, Mohammad Reza Salahshoor, Abdur Razzaque Sarker, Benn Sartorius, Morteza Shamsizadeh, Kenji Shiubya, Roman Topor-Madry, Andrea Werdecker
